# The transcriptional profile of keloidal Schwann cells

**DOI:** 10.1038/s12276-022-00874-1

**Published:** 2022-11-04

**Authors:** Martin Direder, Matthias Wielscher, Tamara Weiss, Maria Laggner, Dragan Copic, Katharina Klas, Daniel Bormann, Vera Vorstandlechner, Erwin Tschachler, Hendrik Jan Ankersmit, Michael Mildner

**Affiliations:** 1 Laboratory for Cardiac and Thoracic Diagnosis, Regeneration and Applied Immunology, Department of Thoracic Surgery, Medical University of Vienna, Vienna, Austria; 2 Aposcience AG (FN 308089y), Dresdner Straße 87/A21, Vienna, Austria; 3 Department of Dermatology, Medical University of Vienna, Vienna, Austria; 4 Department of Plastic, Reconstructive, and Aesthetic Surgery, Medical University of Vienna, Vienna, Austria

**Keywords:** Mechanisms of disease, RNA sequencing

## Abstract

Recently, a specific Schwann cell type with profibrotic and tissue regenerative properties that contributes to keloid formation has been identified. In the present study, we reanalyzed published single-cell RNA sequencing (scRNA-seq) studies of keloids, healthy skin, and normal scars to reliably determine the specific gene expression profile of keloid-specific Schwann cell types in more detail. We were able to confirm the presence of the repair-like, profibrotic Schwann cell type in the datasets of all three studies and identified a specific gene-set for these Schwann cells. In contrast to keloids, in normal scars, the number of Schwann cells was not increased, nor was their gene expression profile distinctly different from that of Schwann cells of normal skin. In addition, our bioinformatics analysis provided evidence for a role of transcription factors of the AP1, STAT, and KLF families, and members of the IER genes in the dedifferentiation process of keloidal Schwann cells. Together, our analysis strengthens the role of the profibrotic Schwann cell type in the formation of keloids. Knowledge of the exact gene expression profile of these Schwann cells will facilitate their identification in other organs and diseases.

## Skin injury: How Schwann cells mediate abnormal scar formation

Reprogrammed genetic activity in specialized versions of Schwann cells, which support the development and functioning of the peripheral nervous system, promotes enlarged abnormal scars known as keloid scars during repair of nerves in skin injuries. Nerve injury causes neighboring Schwann cells to revert to an immature state, and then develop specific repair functions that help nerve healing. Michael Mildner and colleagues at the Medical University of Vienna, Austria, analyzed previously published data on changes in gene activity in Schwann cells active in keloid scars. They identified a role for a set of transcription factors, proteins that control the activity of specific genes, and for a family of genes involved in reprogramming the Schwann cells. These findings may assist understanding of Schwann cell activity in other organs and conditions.

## Introduction

Schwann cells are glial cells of the peripheral nervous system and ensure proper nerve development and integrity^1^. After peripheral nerve injury, mature Schwann cells undergo transcriptional reprogramming that involves dedifferentiation into an immature cell state and the acquisition of repair-specific functions^2,3^. These repair Schwann cells are essential to orchestrate nerve regeneration by attracting immune cells to the site of injury, phagocytosis of myelin debris, secretion of neurotrophic and neuritogenic factors and formation of regeneration tracks (Bungner bands) to stimulate and guide regrowing axons^4^. Dedifferentiation into repair Schwann cells has been shown to involve the expression and activation of a variety of specific factors^5^, including *JUN, STAT3, BDNF, ARTN, IGFBP2,* and *GDNF*^4^. In particular, *OLIG1* and *SHH* have been suggested as specific markers for Schwann cell repair^4^.

In healthy skin, Schwann cells persist in myelinating and nonmyelinating states^1^. Recently, Parfejevs et al. provided evidence that Schwann cells contribute not only to the regeneration of the nerve but also to dermal wound healing by proliferating and emanating from the disrupted nerve. These skin-derived repair Schwann cells populate the damaged area, where they support the differentiation of fibroblasts into myofibroblasts and promote wound contraction and closure as well as re-epithelization^6^. After the completion of neuronal regeneration and wound healing, skin repair Schwann cells redifferentiate into their adult state and ensheath the restored axons^7^. Ideally, the entire healing process results in an asymptomatic, fine-lined, flat scar.

Abnormal scars, such as hypertrophic scars or keloids, can result in serious health problems, including movement restrictions, persistent itch, and pain^8–11^. Keloids represent a special type of scar characterized by tumor-like continuous growth beyond the margins of the original wound^8^. They exclusively develop in humans, and the lack of adequate model systems complicates basic research into their pathogenesis^12,13^. Therefore, despite several decades of research, the exact mechanistic events driving keloid formation remain largely unclear^14^.

The recent development of single-cell RNA sequencing (scRNA-seq) enables a completely new approach to decode disease pathomechanisms. To date, three research groups have applied scRNA-seq to study keloid tissue at the cellular and transcriptional levels. Liu et al. compared the center of keloids with adjacent skin. These researchers identified various dysregulated genes and pathways in keloidal fibroblasts and endothelial cells and revealed TWIST1 as an important factor in keloidal fibrogenesis^15^. Deng et al. applied scRNA-seq to compare keloids with normal scars^16^. This group subdivided keloidal fibroblasts into four major groups and identified an increase in extracellular matrix (ECM)-producing mesenchymal fibroblasts in keloid tissue. Whereas these studies focused on fibroblasts and endothelial cells, a recent study by our group demonstrated that keloids contain an increased number of phenotypically distinct Schwann cells^17^. The vast majority of these keloidal Schwann cells displayed a cellular state comparable to that described for Schwann cells in regenerating nerves^17^. Our additional finding that these Schwann cells expressed multiple genes associated with matrix formation makes them highly plausible candidates for playing a crucial role in the development of keloids^17^.

Owing to donor variabilities, differences in sample preparation and data processing, scRNA-seq studies are not always straightforwardly comparable^18–21^. For more consistent data, individual datasets from different laboratories should be re-evaluated in a combined analysis, as previously shown for skin fibroblast populations^22^. This study clearly demonstrated that, despite differences in the experimental procedures, major similarities among all fibroblast populations are conserved across the scRNA-seq datasets published by the different laboratories. Studies on the comparability of smaller cell clusters, such as Schwann cells, in scRNA-seq datasets are thus far not available.

Here, we performed a comparative analysis of Schwann cells in different scRNA-seq datasets of healthy skin, normal scars, keloids, and keloid-adjacent skin from four independent research groups to elucidate the gene-set most reliably identifying the keloidal Schwann cell population. In addition, we compared two distinct bioinformatics approaches for the analysis of numerous different scRNA-seq datasets, revealing significant variations in the two calculation methods. Our study shows that the presence of the previously described repair-like, profibrotic Schwann cells in keloids is indeed conserved across different scRNA-seq datasets and provides a specific expression pattern for keloidal Schwann cells. Thus far, unrecognized cells might represent a novel, potential target for keloid treatment.

## Materials and methods

### Data acquisition

**Fig. 1:**
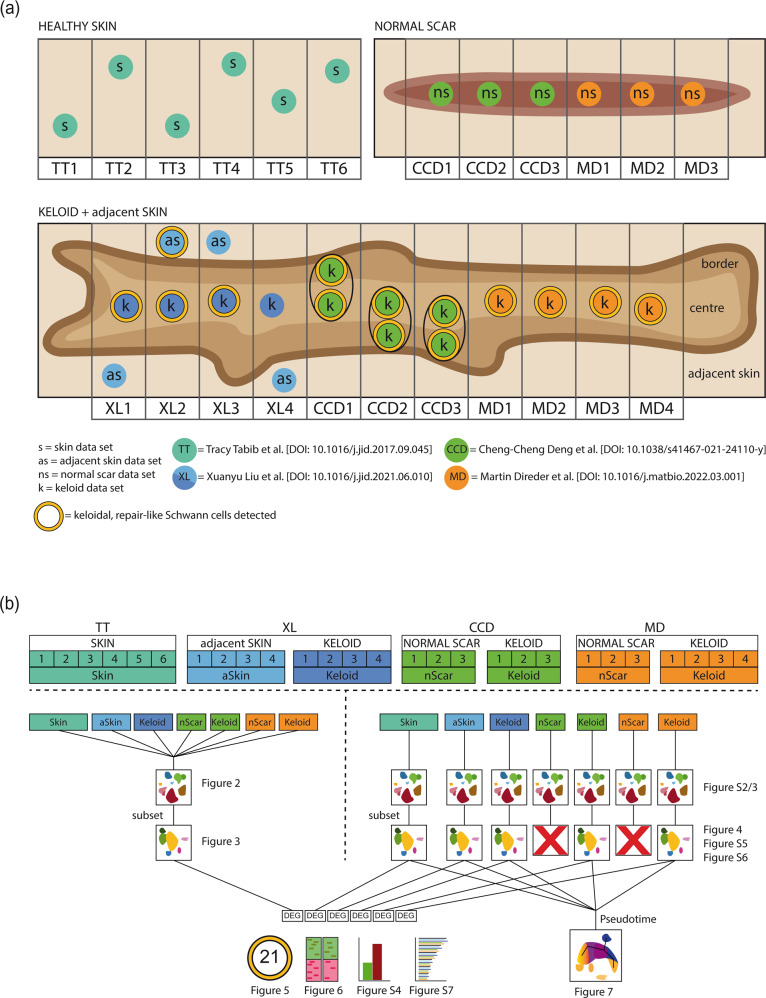
Graphical scheme of the included datasets and bioinformatics method overview. **a** Schematic illustration of the regional sampling point of every dataset. **b** Synoptical chart of all dataset combinations included in this study with links for each respective figure. Skin (s), adjacent skin (as/aSkin), normal scar (ns/nScar), keloid (k); data source: Tracy Tabib et al., 2018 (TT)^18^; Xuanyu Liu et al., 2021 (XL)^15^, Cheng-Cheng Deng et al., 2021 (CCD)^16^, Martin Direder et al., 2022 (MD)^17^; DEG differentially expressed gene.

In this study, scRNA-seq datasets of 6 healthy skin, 6 normal scar, 11 keloid and 4 keloid-adjacent skin samples were analyzed (Fig. 1a). ScRNA-seq data of normal scars and keloids have already been generated by Direder et al. and were deposited in NCBI´s Gene Expression Omnibus (GSE181316)^17^. Skin data published by Tabib et al. (2018) were downloaded from https://dom.pitt.edu/wp-content/uploads/2018/10/Skin_6Control_rawUMI.zip and https://dom.pitt.edu/wp-content/uploads/2018/10/Skin_6Control_Metadata.zip^18^. ScRNA-seq data of keloids and normal scars generated by Deng et al. were downloaded from the Gene Expression Omnibus database (dataset GSE163973)^16^. Transcriptomic data from Liu et al. were downloaded from the Genome Sequencing Archive (BioProject PRJCA003143)^15^. Count tables were obtained from the original fastq read files applying the CellRanger pipeline (10X Genomics CellRanger 3.0.2, Pleasonton, CA, USA)^23^.

### Data processing

Computational analyses were performed utilizing R and R Studio (version 4.0.3, The R Foundation, Vienna, Austria). All features of the included datasets were screened, and doublets were removed. The feature designation was harmonized, and only features detected in all datasets were included in subsequent analyses. Datasets were transformed into a Seurat object using Seurat (Seurat v4.0.1, Satija Lab)^24^. Sctransform normalization in combination with the glmGamPoi package and removal of the mitochondrial mapping percentage were applied to preprocess the data^25,26^. Erythrocytes were excluded by removing cells with a hemoglobin subunit beta (HBB) expression >5. For this study, the included datasets were combined all together and individually by source and tissue type (Fig. 1b). All dataset combinations were processed according to the Seurat Vignette. Principal component analysis (PCA), uniform manifold approximation (UMAP) and projection dimensionality reduction were applied according to the Seurat protocol. Clusters were identified as cell types according to their expression of well-established marker genes^17^. Schwann cell clusters were subsetted, and data were preprocessed and calculated in the same way as described above. The commands “RunUMAP” and “FindNeighbors” were performed on 30 dimensions, and “FindClusters” was applied with a resolution of “0.3”. Subset clusters expressing marker genes typical for melanocytes were removed. Schwann cells were characterized by the expression of established marker genes^17,27^. Differentially expressed genes were defined as genes displaying an average fold change >2. The SC-Keloid-specific gene expression pattern was identified by examination of SC-Keloid against all myelinating and nonmyelinating Schwann cells of all dataset combinations (Supplementary Tables 1–5). From these lists, the top 100 upregulated genes were compared, and only genes included in all gene lists were selected as members of the SC-Keloid gene expression pattern. Genes without a clear demarcation from the myelinating and nonmyelinating Schwann cells were excluded. Gene Ontology (GO) enrichment analysis was performed using Metascape with 0.05 as the *p*-value cutoff and 2 as the minimum enrichment score^28^. Only corresponding GO terms with “Summary” Group ID are shown. For pseudotime analysis, the Schwann cell clusters from all single calculated datasets were combined and preprocessed in the same way. As normal scar datasets from both sources revealed no Schwann cell cluster, these datasets were excluded from the pseudotime analysis. The commands “RunUMAP” and “FindNeighbors” were performed on 18 dimensions, and “FindClusters” was applied with a resolution of 0.5. Monocle3 (Monocle3, v.0.2.3.0, Trapnell Lab, Seattle, Washington, USA) was used to create the pseudotime trajectory^29–33^. The principal graph was not pruned, and a total of 7 centers were determined. The generated Metascape lists were matched, and only –log10(*p*) values of terms identified in all lists were pictured together with their arithmetic mean. For identification of upregulated genes specific for the branching point in the combined Schwann cell object, the command “Findmarkers” was executed, comparing the newly identified Seurat cluster located at the branching point against all remaining Schwann cells. Potential interactions of Schwann cells at the branching point or of Schwann cell subtypes in keloids and healthy skin were analyzed using CellChat^34^.

### Sequencing data juxtaposition to the gene expression profile of peripheral nerve regeneration

The human homologs of the gene list published by Bosse et al.^5^ were identified using the GenBank accession number and the website UniProt (https://www.uniprot.org/, accessed on 2021-10-18). Relative expression levels were identified using the command “FindMarkers” with SC-Keloid as ident.1 and the myelinating and nonmyelinating cluster as the opposite ident. A min.pct of 0.01 and a logfc.threshold of 0.01 was set. Expression changes <0.01 were set as 0. Significantly regulated genes published by Bosse et al. were identified in our list of differentially expressed genes, and expression levels were colored corresponding to the color code by Bosse et al.

### Collagen alignment examination

Hematoxylin and eosin staining of healthy skin, normal scar, and keloid samples was imaged. Alignment of collagen bundles was determined by applying Curvealign V4.0 Beta (MATLAB software, Cleve Moler, MathWorks, Natick, Massachusetts, USA). Collagen color, contrast and brightness were edited by Adobe Photoshop CS6 (Adobe, Inc., San Jose, CA, USA). The region of interest (ROI) was defined by size (256 height, 256 width), and four ROIs per condition were analyzed. The coefficiency of alignment was statistically evaluated.

### Immunofluorescence

Tissue samples were fixed in neutral buffered 4.5% formaldehyde (SAV Liquid Production GmbH, Flintsbach am Inn, Germany) at 4 °C overnight, followed by a washing step with phosphate-buffered saline (PBS) overnight. Dehydration was performed by sequential incubations with 10%, 25%, and 42% sucrose for 24 h each. Tissues were embedded in optimal cutting temperature compound (OCT compound, TissueTek, Sakura, Alphen aan den Rijn, The Netherlands) and stored at −80 °C. Samples were cut into 10 µm-thick sections and dried for 30 min at room temperature. Slides were blocked and permeabilized for 15 min with 1% BSA, 5% goat serum (DAKO, Glostrup, Denmark), and 0.3% Triton-X (Sigma Aldrich, St. Louis, MO, USA) in 1x PBS. Sections were incubated with primary antibody solution overnight at 4 °C followed by three washing steps with 1x PBS for 5 min each (Supplementary Table 6). Sections were incubated with secondary antibodies and 50 µg/ml 4,6-diamidino-2-phenylindole (DAPI, Thermo Fisher Scientific, Waltham, MA, USA) in 1x PBS for 50 min at room temperature. After three washing steps, sections were covered using mounting medium and stored at 4 °C. Images were acquired using a confocal laser scanning microscope (TCS SP8X, Leica, Wetzlar, Germany) equipped with a 10x (0.3 HCPL FluoTar), a 20x (0.75 HC-Plan-Apochromat, Multimmersion), a 20x (0.75 HC- Plan-Apochromat) and a 63x (1.3 HC-Plan-Apochromat, Glycerol) objective using the Leica application suite X version 1.8.1.13759 or LAS AF Lite software (both Leica). Final images constitute a maximum projection of total z-stacks.

### Statistics

Statistical evaluation was performed using GraphPad Prism 8 software (GraphPad Software Inc., La Jolla, CA, USA). The Shapiro‒Wilk test was used to test for normal distribution. One-way ANOVA with Tukey’s *post-hoc* test was used to compare three and more groups. *p*-values are marked with asterisks: *p < 0.05, **p < 0.01, ***p < 0.001 ****p < 0.0001.

## Results

### Combined integration of all scRNA-seq datasets identifies significant differences in the cellular composition of normal skin, normal scars, and keloids

**Fig. 2:**
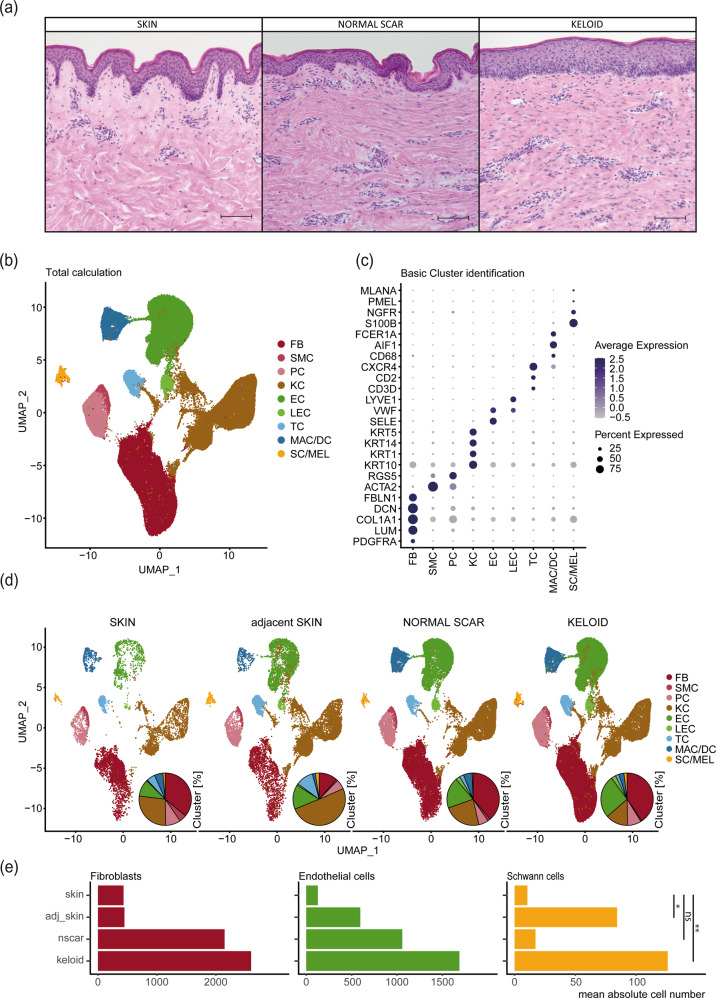
Cellular composition of skin, normal scars, keloids, and keloid-adjacent skin. **a** Hematoxylin-eosin staining of skin, normal scar, and keloid. Scale bars: 250 µm. **b** UMAP plot after integration of all datasets. Cluster identifications of fibroblasts (FB), smooth muscle cells (SMC), pericytes (PC), keratinocytes (KC), endothelial cells (EC), lymphatic endothelial cells (LEC), T cells (TC), macrophages and dendritic cells (MAC/DC), Schwann cells and melanocytes (SC/MEL). **c** Dot plots showing well-known marker genes to characterize clusters: *PDGFRA*, *LUM*, *COL1A1*, *DCN*, *FBLN1* for FB, *ACTA2*, *RGS5* for SMC and PC, *KRT10*, *KRT1*, *KRT14*, *KRT5* for KC, *SELE*, *VWF* for EC, *LYVE1* for LEC, *CD3D*, *CD2*, *CXCR4* for TC, *CD68*, *AIF1* for MAC, *FCER1A* for DC, *S100B*, *NGFR* for SC, *PMEL*, *MLANA* for MEL; Dot size symbolizes percentage of cells expressing the gene, color gradient represents average gene expression. **d** Split UMAP plots show the cellular composition within each tissue. Pie plots depict the relative amounts of each cell type within a tissue. **e** Bar plots depict arithmetic means of the absolute numbers of FB, EC, and SC within each condition. Asterisks represent *p*-values: *p < 0.05, **p < 0.01, ***p < 0.001, ns: not significant.

Histological examination of normal skin samples, normal scars and keloids showed increased dermal cell numbers and abnormalities of the ECM in both types of scars, including increased linearity of collagen bundles (Fig. 2a and Supplementary Fig. 1)^35^. To determine how cell populations differ among healthy skin, skin adjacent to keloids, normal scars and keloids, we performed a combined integration of individual scRNA-seq datasets^15–18^. In total, datasets of six normal skin samples, four skin samples adjacent to keloids, six normal scars and eleven keloids were included. Demographic data on the samples and methodological differences in sample preparation are shown in Table 1 and Supplementary Table 7, respectively. Combined bioinformatics analysis of the datasets revealed several main cell clusters (Fig. 2b), which were further characterized by marker gene expression (Fig. 2c). The clusters were identified as fibroblasts (FB), smooth muscle cells (SMC), pericytes (PC), keratinocytes (KC), endothelial cells (EC), lymphatic endothelial cells (LEC), T cells (TC), macrophages (MAC), dendritic cells (DC), Schwann cells (SC) and melanocytes (MEL) (Fig. 2b, c). Interestingly, macrophages and dendritic cells as well as Schwann cells and melanocytes clustered together (Fig. 2b). Split analysis revealed similar cell clusters but different cell numbers in each condition (Fig. 2d). While the cell numbers of fibroblasts and endothelial cells were increased in normal scars and keloids, increased numbers of Schwann cells were only detectable in keloids, indicating a specific role of Schwann cells in the pathogenesis of keloids (Fig. 2e).

**Table 1:** Donor information.

Tissue	Source	Paper ID	Original ID	Site	Age	Sex	Race
Normal skin	TT^18^	TT1_s	SC18control	Forearm	63	Male	Caucasian
Normal skin	TT^18^	TT2_s	SC1control	Forearm	54	Male	Caucasian
Normal skin	TT^18^	TT3_s	SC32control	Forearm	66	Female	Caucasian
Normal skin	TT^18^	TT4_s	SC33control	Forearm	23	Female	Asian
Normal skin	TT^18^	TT5_s	SC34control	Forearm	62	Female	Caucasian
Normal skin	TT^18^	TT6_s	SC4control	Forearm	24	Male	Caucasian
Adjacent skin	XL^15^	XL1_s	K007CTRL	Chest	28	Female	Chinese
Adjacent skin	XL^15^	XL2_s	K009CTRL	Chest	32	Female	Chinese
Adjacent skin	XL^15^	XL3_s	K013CTRL	Chest	26	Female	Chinese
Adjacent skin	XL^15^	XL4_s	K012CTRL	Chest	26	Female	Chinese
Normal scar	CCD^16^	CCD1_ns	human_normal_scar_sample1	Back	39	Male	Han nationality
Normal scar	CCD^16^	CCD2_ns	human_normal_scar_sample2	Chest	28	Male	Han nationality
Normal scar	CCD^16^	CCD3_ns	human_normal_scar_sample3	Chest	26	Female	Han nationality
Normal scar	MD^17^	MD1_ns	scar1	Abdomen	26	Female	Caucasian
Normal scar	MD^17^	MD2_ns	scar2	Abdomen	43	Female	Caucasian
Normal scar	MD^17^	MD3_ns	scar3	Abdomen	60	Male	Caucasian
Keloid	XL^15^	XL1_k	K007CASE	Chest	28	Female	Chinese
Keloid	XL^15^	XL2_k	K009CASE	Chest	32	Female	Chinese
Keloid	XL^15^	XL3_k	K013CASE	Chest	26	Female	Chinese
Keloid	XL^15^	XL4_k	K012CASE	Chest	26	Female	Chinese
Keloid	CCD^16^	CCD1_k	human_keloid_sample1	Back	20	Male	Han nationality
Keloid	CCD^16^	CCD2_k	human_keloid_sample2	Chest	23	Male	Han nationality
Keloid	CCD^16^	CCD3_k	human_keloid_sample3	Chest	34	Female	Han nationality
Keloid	MD^17^	MD1_k	keloid_1	Chest	36	Female	Caucasian
Keloid	MD^17^	MD2_k	keloid_2	Earlobe	60	Female	African
Keloid	MD^17^	MD3_k	keloid_3 L	Earlobe—left	34	Male	Caucasian
Keloid	MD^17^	MD4_k	keloid_3R	Earlobe—right	34	Male	Caucasian

### Keloidal Schwann cells are conserved in different scRNA-seq datasets

**Fig. 3:**
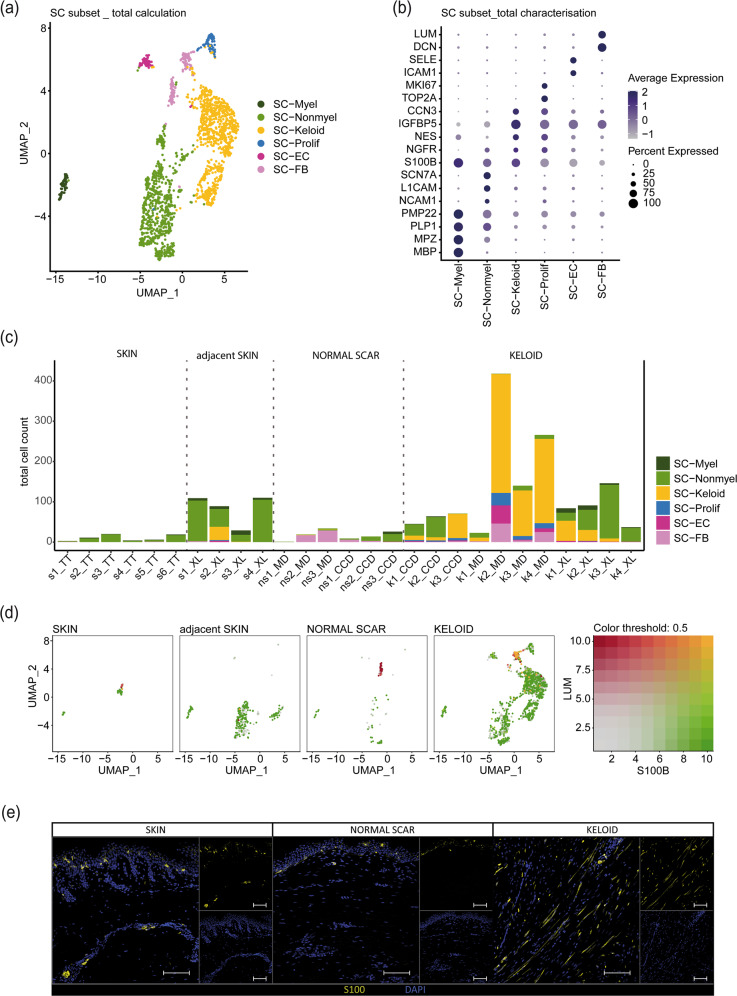
Schwann cell subset detects profibrotic Schwann cells in datasets from all sources. **a** UMAP plot of the Schwann cell subset (SC subset) after melanocyte removal. **b** Dot plot identifying the Schwann cell subcluster by expression of marker genes: *MBP*, *MPZ*, *PLP1*, *PMP22* for myelinating Schwann cells (SC-Myel); *NCAM1*, *L1CAM*, *SCN7A* for nonmyelinating Schwann cells (SC-Nonmyel); *S100B*, *NGFR* as general Schwann cell marker; *NES*, *IGFBP5*, *CCN3* for keloidal Schwann cells (SC-Keloid); *TOP2A*, *MKI67* for proliferating Schwann cells (SC-Prolif), *DCN*, *LUM* for cells expressing Schwann cell-specific and fibroblast-specific genes (SC-FB), *ICAM1*, *SELE* for cells expressing Schwann cell-specific and endothelial cell-specific genes (SC-EC). **c** Bar plots depicting the absolute number of distinct Schwann cell subtypes within each dataset. skin (s), normal scar (ns), keloid (k); data source: Tracy Tabib et al., 2018 (TT)^18^; Xuanyu Liu et al., 2021 (XL)^15^, Cheng-Cheng Deng et al., 2021 (CCD)^16^, Martin Direder et al., 2021 (MD)^17^; **d** Feature blends show the expression of *S100B* (green), *LUM* (red) and double expression of both genes (yellow) in the SC subset split by tissue. **e** Immunofluorescence staining of S100-positive SCs in the dermal layer of a keloid; Scale bar: 100 µm.

As the main goals of our study were the in-depth analysis of Schwann cells in the different scar types and their comparability across different single-cell datasets, we next recalculated the combined integrated Schwann cell cluster, removed all melanocytes and performed subclustering (Fig. 3). The different Schwann cell subclusters (Fig. 3a) were identified using well-established Schwann cell markers (Fig. 3b)^17,27^. This analysis identified myelinating Schwann cells (SC-Myel), nonmyelinating Schwann cells (SC-Nonmyel), proliferating Schwann cells (SC-Prolif), Schwann cells additionally expressing genes typical for endothelial cells (SC-EC) or fibroblasts (SC-FB) and the previously described repair-like, profibrotic keloidal Schwann cells (SC-Keloid) (Fig. 3a, b). We next separated each dataset and detected only a few Schwann cells in normal skin (Fig. 3c). In contrast, the number of Schwann cells was markedly increased in keloids (Fig. 3c). Interestingly, we also found an increased number of Schwann cells in skin adjacent to keloids but not in normal scars (Fig. 3c). Schwann cells of normal skin were identified as either myelinating or nonmyelinating Schwann cells. In contrast, we found a high plasticity of Schwann cells in keloids, with a high number of cells displaying a profibrotic phenotype. These keloidal Schwann cells were also detected in one skin sample adjacent to keloids (Fig. 3c). Of note, a substantial number of cells in the Schwann cell cluster present in normal scars did not express the Schwann cell marker gene *S100B* but expressed genes typical for fibroblasts, such as *LUM*, suggesting incorrect cluster assignment of some fibroblasts in the combined integration (Fig. 3d, single red dots in normal scar). In contrast, some keloids contained Schwann cells coexpressing fibroblast markers and *S100B* (Fig. 3d, double positive, yellow dots in keloids). To confirm our transcriptomic data, we performed S100 immunostaining of normal skin, normal scars and keloids (Fig. 3e). As suggested by our scRNA-seq data, we found a markedly increased number of Schwann cells in keloids. These cells were spindle-shaped with long extensions on both ends (Fig. 3e), a morphology previously described for repair Schwann cells^7,17,36^.

**Fig. 4:**
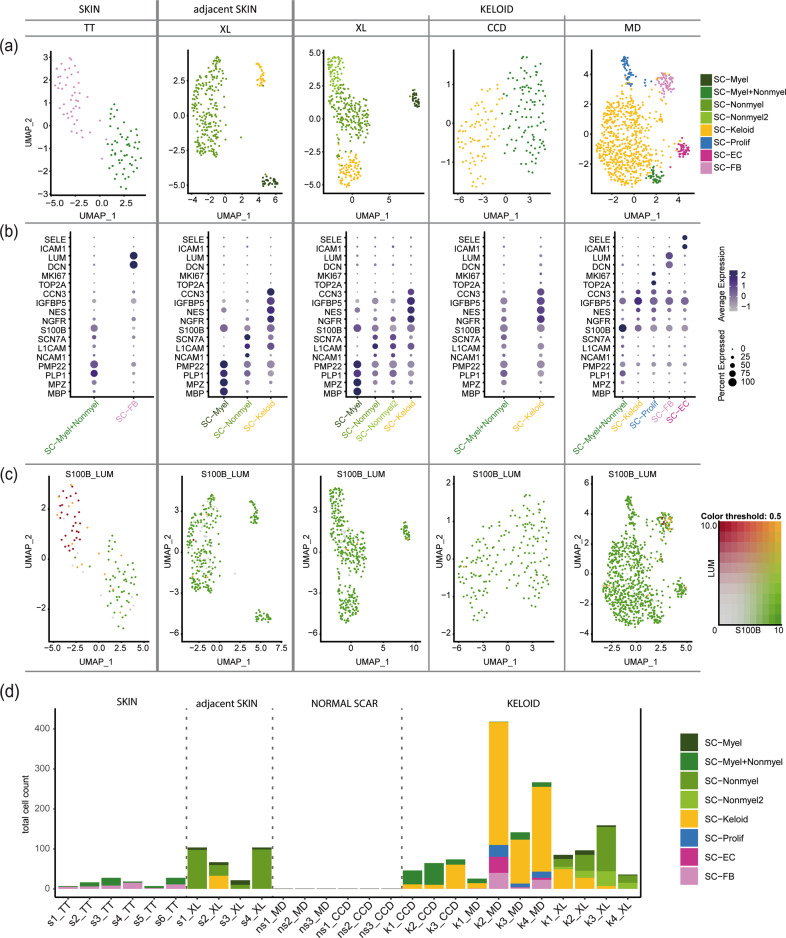
Tissue- and source-specific dataset evaluation confirms keloidal profibrotic Schwann cells. **a** UMAP plots depict subsets of identified Schwann cell clusters. Data sources: Tracy Tabib et al., 2018 (TT)^18^; Xuanyu Liu et al., 2021 (XL)^15^, Cheng-Cheng Deng et al., 2021 (CCD)^16^, Martin Direder et al., 2021 (MD)^17^; **b** Dot plots of well-known marker genes to characterize Schwann cell subtypes. *MBP*, *MPZ*, *PLP1*, and *PMP22* for myelinating Schwann cells (SC-Myel); *NCAM1*, *L1CAM*, and *SCN7A* for nonmyelinating Schwann cells (SC-Nonmyel, SC-Nonmyel2); *S100B* and *NGFR* as general Schwann cell markers; *NES*, *IGFBP5*, *CCN3* for keloidal Schwann cells (SC-Keloid); *TOP2A* and *MKI67* for proliferating Schwann cells (SC-Prolif), *DCN* and LUM for cells expressing Schwann cell and fibroblast-specific genes (SC-FB), *ICAM1* and *SELE* for cells expressing Schwann cell and endothelial cell-specific genes (SC-EC); mixed myelinating and nonmyelinating Schwann cell cluster (SC-Myel+Nonmyel); Color codes indicate average gene expression levels; Dot sizes visualize relative amounts of positive cells. **c** Feature blends reveal cells positive for *S100B* (green) and *LUM* (red) and double-positive cells (yellow). **d** Bar plots show the absolute cell count of distinct Schwann cell clusters in each included dataset. skin (s), normal scar (ns), keloid (k); data source: Tracy Tabib et al., 2018 (TT)^18^; Xuanyu Liu et al., 2021 (XL)^15^, Cheng-Cheng Deng et al., 2021 (CCD)^16^, Martin Direder et al., 2021 (MD)^17^.

To overcome possible calculation errors of combined integration, we also integrated the datasets of each study individually. In line with our previous calculation approach, we found increased numbers of Schwann cells in keloids (Supplementary Figs. 2 and 3). Interestingly, we were not able to identify a Schwann cell cluster in normal scars by separate integration of the datasets (Supplementary Fig. 2). Therefore, only healthy skin, adjacent skin and the three keloid datasets were included for further analysis. All main clusters and subclusters were calculated using the same R-protocol with corresponding identical parameters. Subset analysis uncovered up to five clusters of Schwann cell subtypes in the individual conditions (Fig. 4a). Similar to the combined calculation, individual integration of each dataset also identified the presence of profibrotic Schwann cells in almost all keloids and in one skin sample adjacent to a keloid (Fig. 4a, b). In contrast to the combined integration, we detected a high number of *S100B*-negative cells that were lumican-positive in the single integrated skin samples (Fig. 4c, single-positive red dots). Together, our data indicate that profibrotic Schwann cells are robustly detectable in keloids regardless of the calculation method. Problems with incorrectly assigned fibroblasts were found in both calculation methods and mainly affected very small Schwann cell clusters present in normal skin or normal scars (Fig. 4d).

### Characterization of a gene-set specific for keloidal, profibrotic Schwann cells

**Fig. 5:**
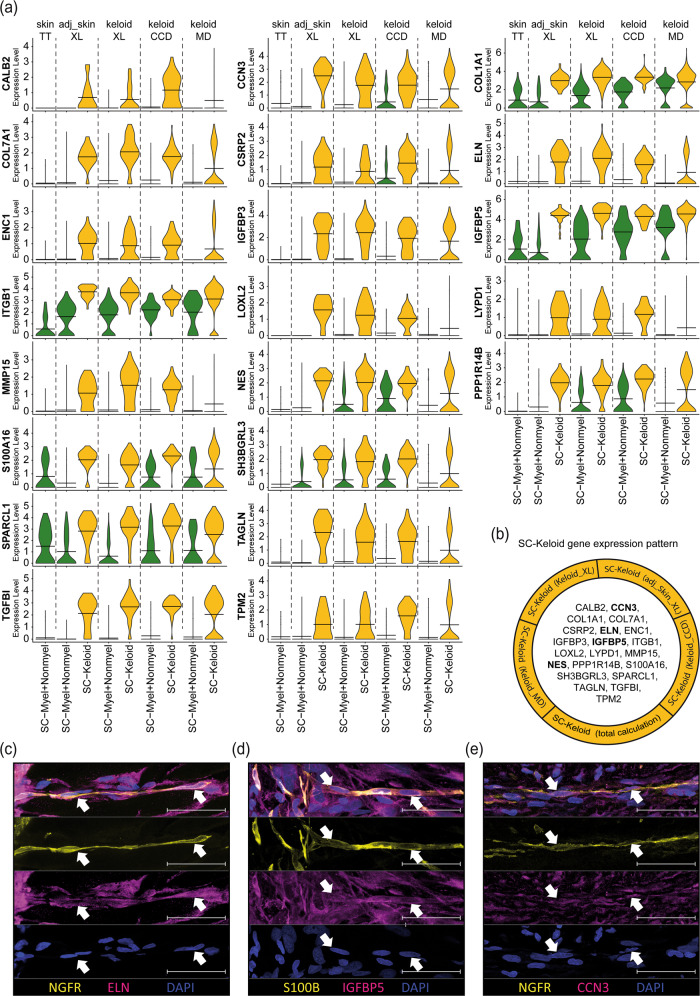
Individual dataset combinations reveal the gene expression pattern of keloidal profibrotic Schwann cells. **a** Violin plots of corresponding, meaningful genes detected in all individual top 100 gene lists comparing keloidal Schwann cells (SC-Keloid) with mature Schwann cells (myelinating and nonmyelinating Schwann cells). Data sources: Tracy Tabib et al., 2018 (TT)^18^; Xuanyu Liu et al., 2021 (XL)^15^, Cheng-Cheng Deng et al., 2021 (CCD)^16^, Martin Direder et al., 2021 (MD)^17^; adjacent skin (adj_skin); Crossbeams show mean expression values; maximum expression is depicted by vertical lines; frequency of cells with the respective expression level is shown by violin width; myelinating Schwann cells (SC-Myel); nonmyelinating Schwann cells (SC-Nonmyel, SC-Nonmyel2), myelination and nonmyelinating Schwann cell mixed cluster (SC-Myel+Nonmyel); keloidal Schwann cells (SC-Keloid); proliferating Schwann cells (SC-Prolif), cells expressing Schwann cell and endothelial cell-specific genes (SC-EC); cells expressing Schwann cell and fibroblast-specific genes (SC-FB). **b** List of all members from the SC-Keloid gene expression pattern. mRNAs in bold have been verified at the protein level. **c**–**e** Immunofluorescence staining of keloidal Schwann cells visualized by S100B or NGFR (yellow) in combination with ELN, IGFBP5 or CCN3. Arrowheads indicate double-positive Schwann cells. Nuclei were stained with DAPI (blue). Scale bars: 50 µm.

We next analyzed all differentially expressed genes by comparing one Schwann cell cluster with all other Schwann cells within each dataset. More genes were downregulated in the SC-Keloid cluster in keloids than adjacent skin (Supplementary Fig. 4 and Supplementary Tables 1–5). Evaluation of the top differentially expressed genes between profibrotic Schwann cells and myelinating/nonmyelinating Schwann cells revealed a set of 21 genes that were characteristic of profibrotic Schwann cells in keloids (Fig. 5a, b). Comparison of the mean expression values of these genes in the individual datasets confirmed the highly specific expression pattern of keloidal Schwann cells (Fig. 5a). Interestingly, the expression of most of these genes was comparable between some of the keloid-specific Schwann cell clusters (SC-Prolif, SC-EC, and SC-FB) and the profibrotic Schwann cells (SC-Keloid), indicating that although SC-Prolif, SC-EC and SC-FB cluster separately and show some additional characteristics, they also share most of the features of keloidal profibrotic Schwann cells (Supplementary Fig. 5). In our previous work, we discovered a contribution of Schwann cells to ECM formation in keloids^17^. We therefore analyzed the expression of matrix proteins in all Schwann cells of the different datasets and found that most collagen genes were significantly increased in keloidal Schwann cells in all datasets (Supplementary Fig. 6). Furthermore, GO-term analysis of keloidal Schwann cells in all datasets confirmed a strong association of these cells with ECM organization and wound healing (Supplementary Fig. 7). Interestingly, some collagens and other factors of the ECM were also found among the SC-Keloid-specific genes (Fig. 5b). Together with the previously shown NES protein expression^17^, we further confirmed ELN, IGFBP5 and CCN3 protein expression in keloidal Schwann cells (Fig. 5c–e).

### Keloidal Schwann cells show similarities with repair Schwann cells in damaged peripheral nerves

**Fig. 6:**
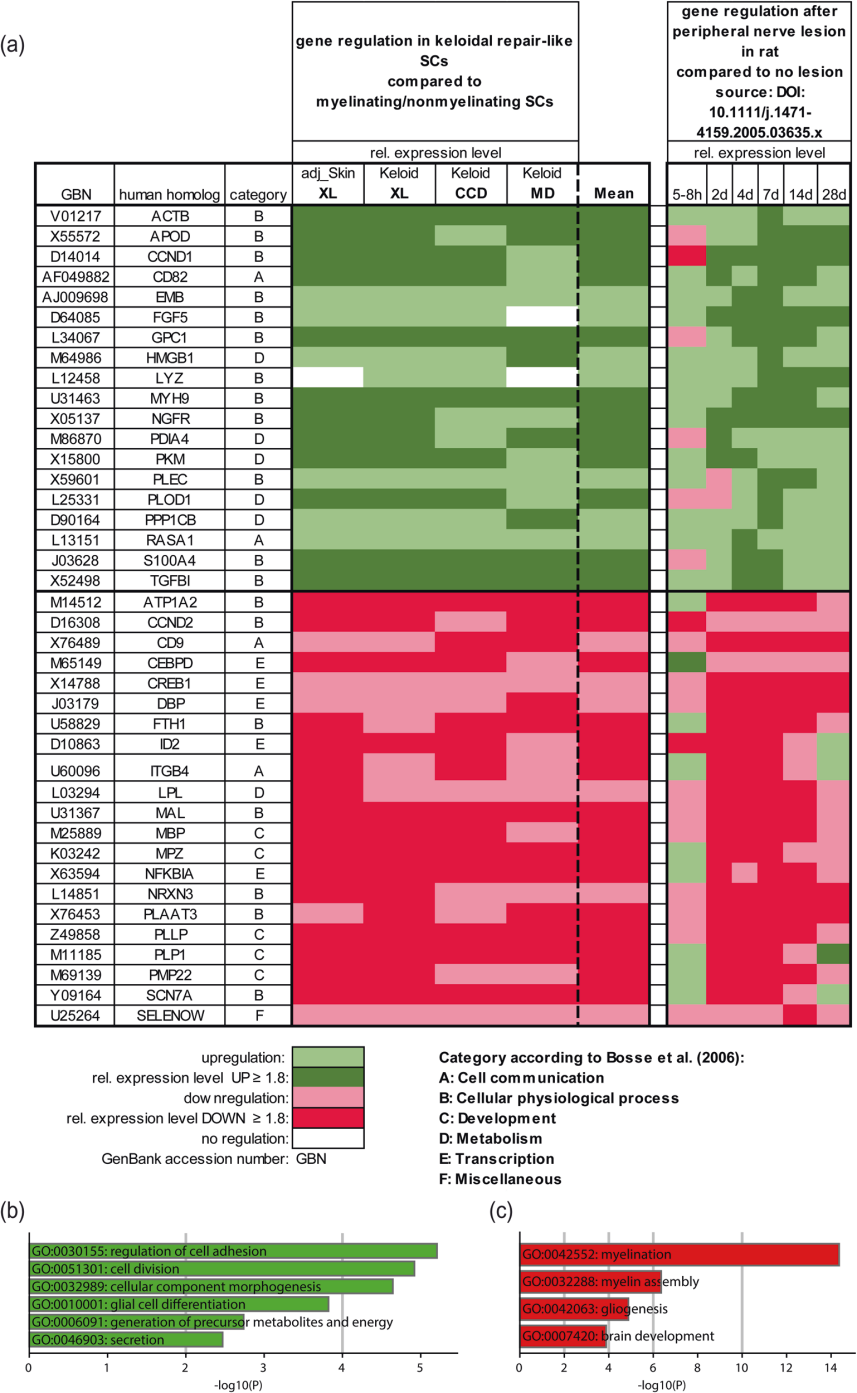
Gene regulation of keloidal Schwann cells partially matches gene regulation upon neural damage. **a** Heatmap of genetic conformities between keloidal Schwann cells and activated nerve-associated cells upon neuronal damage. Genes were sorted alphabetically and categorized according to the source list of Bosse et al. (2006)^5^. GO-term enrichment of the corresponding **b** up- and **c** downregulated genes. The bar length depicts the statistical significance of the annotated term.

Bosse et al. were the first to describe a specific panel of genes regulated in nerve-associated cells upon damage to peripheral nerves^5^. This set of genes was associated with the formation of repair Schwann cells and their function to promote regeneration of the peripheral nerve^36^. To investigate similarities of repair Schwann cells, present in the injured nerve, and keloidal Schwann cells, we compared the gene list of Bosse et al. with our dataset and detected 40 genes that were similarly regulated in both datasets (Fig. 6). For example, *ACTB*, *GPC1*, *MYH9*, *S100A4*, *TGFBI*, *ATP1A2*, *MAL*, *MPZ*, *NFKBIA*, *PLLP*, *PLP1*, and *SCN7A* showed comparable regulation (Fig. 6a and Supplementary Table 8). GO-term analysis of the corresponding regulated genes revealed a typical regulation of cellular processes associated with repair Schwann cells, including regulation of cell adhesion and division, generation of precursor metabolites and energy and downregulation of myelination (Fig. 6b, c). These data suggest that a part of the repair Schwann cell gene-set identified by Bosse et al. is conserved in keloidal Schwann cells.

### Pseudotime trajectory analysis identifies candidate factors decisive for keloidal, profibrotic Schwann cell development

**Fig. 7:**
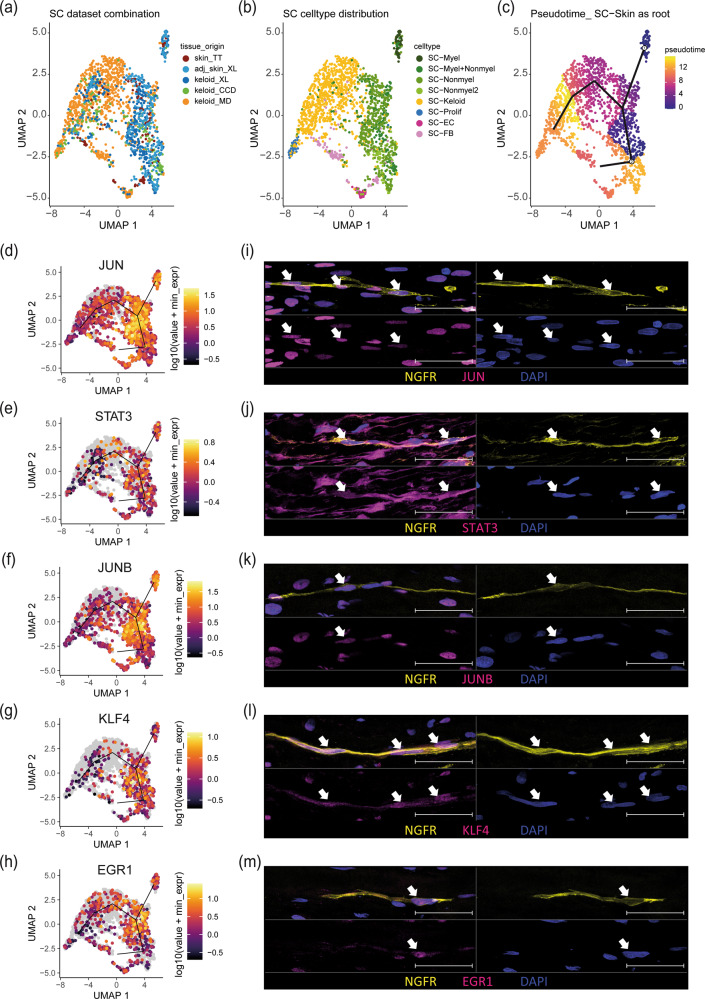
Pseudotime analysis uncovers pivotal genes in the dedifferentiation track of Schwann cells. UMAP plots combining Schwann cells detected in all individual computations colored by tissue source (**a**) and cell type (**b**) and pseudotime trajectory with principal graph (**c**, myelinating and nonmyelinating Schwann cell as root). **d**–**h** Feature plots of *JUN*, *STAT3*, *JUNB*, *KLF4*, and *EGR1*. **i**–**m** Immunofluorescence staining of keloidal Schwann cells visualized by NGFR in combination with JUN, STAT3, JUNB, KLF4, or EGR1. Nuclei were stained with DAPI (blue). Arrowheads indicate double-positive Schwann cells. Scale bars: 50 µm.

To further identify novel signaling molecules involved in the development of keloidal profibrotic Schwann cells, we performed pseudotime trajectory analysis. Therefore, we reintegrated all individually identified Schwann cell clusters from each study (Fig. 7a). A UMAP, colored by the previously defined Schwann cell subtypes, showed a clear demarcation of the different Schwann cell populations (Fig. 7b). Pseudotime trajectory calculation suggested that myelinating and nonmyelinating Schwann cells dedifferentiate toward one common branching point and then further into profibrotic Schwann cells (Fig. 7c). Since *JUN*, *STAT3*, *ARTN*, *BDNF*, *GDNF*, *SHH*, and *OLIG1* are known decisive factors for the development of repair Schwann cells upon neural damage^4^, we next plotted their expression on the pseudotime trajectory. The transcription factors *JUN* and *STAT3* were highly expressed, specifically in cells at the branching point, confirming their role in the dedifferentiation process of repair Schwann cells (Fig. 7d, e). Of note, other described factors involved in the repair of Schwann cell development in peripheral nerves (*ARTN*, *BDNF*, *GDNF*, *SHH*, *OLIG1* and *IGFBP2*) were not or only weakly expressed in keloidal Schwann cells (Supplementary Fig. 8a). In addition, our analysis identified several specific groups of transcription factors significantly enriched at the branching point, such as members of the AP-1 family (Fig. 7f and Supplementary Fig. 8b), Kruppel-like factors (KLF) (Fig. 7g and Supplementary Fig. 8c) and immediate early response genes (IER) (Supplementary Fig. 8d), as well as some other transcription factors (*EGR1*, *ATF3*, *HES1*, *ZFP36*) (Fig. 7h and Supplementary Fig. 8e), suggesting an involvement in keloidal repair-like, profibrotic Schwann cell development. Immunofluorescence staining confirmed the presence of nuclear JUN, STAT3, JUNB, KLF4 and EGR1 proteins in NGFR^+^ keloidal Schwann cells in situ (Fig. 7i–m).

To investigate cell‒cell communication processes possibly involved in the dedifferentiation process of keloidal Schwann cells, we next performed CellChat analysis. Therefore, we calculated interactions between Schwann cells located at the branching point and all other cell types present in the tissue (Supplementary Fig. 9a). Our analysis revealed a strong impact of fibroblasts on keloidal Schwann cells, suggesting the involvement of fibroblasts in the dedifferentiation process (Supplementary Fig. 9b). Furthermore, keratinocytes and smooth muscle cells/pericytes seemed to be influenced by keloidal Schwann cells (Supplementary Fig. 9b). Interestingly, such strong cell‒cell interactions were not observed in healthy skin (Supplementary Fig. 10a–d). More detailed analysis of these cell‒cell interactions in keloids mainly identified interactions with components of the ECM, including collagens and laminin subunits (Supplementary Fig. 9c). Similar cell‒cell interactions were observed between the different subsets of keloidal Schwann cells and all other cell types present in keloids (Supplementary Fig. 11a–f). Together, these data suggest that the keloidal microenvironment contributes to the formation of keloidal repair Schwann cells.

## Discussion

Recently, we identified an important role of Schwann cells in the pathogenesis of keloids^17^. Using scRNA-seq, we were able to demonstrate that a high number of Schwann cells are present in keloids and that these cells significantly contribute to the overproduction of the extracellular matrix^17^. Interestingly, these Schwann cells were not associated with axons and showed a nonclassical repair-like phenotype. However, their exact transcriptional profile and marker genes unambiguously determining this keloidal Schwann cell type have not yet been described. Although scRNA-seq represents a powerful tool for studying gene expression in organs and tissues at a single-cell resolution, the comparability of different scRNA-seq datasets and generation of reproducible data are still challenging. Donor variabilities, differences in the tissue dissociation procedure and differences in bioinformatics processing of the data are the main reasons for heterogeneous results^37–39^. Such methodological differences could significantly affect absolute cell numbers and gene expression patterns. As Schwann cell numbers are already strongly dependent on the body site^40^ and certain diseases further influence the number and expression profile of Schwann cells in the skin^17,41^, a combined evaluation of several datasets was essential to decipher the transcriptional profile of the newly identified keloidal Schwann cell type.

To date, several studies have underlined the impact of the tissue dissociation procedure on gene expression^37–39^. The commercially available whole-skin dissociation kit is the most commonly used skin dissociation method for scRNA-seq experiments^22,42^ and was used in 3 of the 4 studies analyzed (Supplementary Table 7). In only one study, keloids were digested with dispase II and collagenase IV for two hours. Interestingly, this dataset contained significantly fewer Schwann cells, suggesting that the use of the whole-skin dissociation kit yields more reliable data representing all cell types present in the tissue of interest. In the other three studies, the tissue was digested for different time periods. Of note, keloid samples generated by an overnight dissociation step showed significantly more myelinating Schwann cells compared to the studies using a shorter dissociation time (up to 2.5 h), indicating that the dissociation of Schwann cells attached to axons is difficult and can be improved by a longer digestion period. This finding is in line with established isolation methods of Schwann cells from human peripheral nerves that include an overnight dissociation step^3,43^. Furthermore, in a recent publication describing a method for the isolation of Schwann cells from healthy skin, the authors discussed preculturing steps to disintegrate the nerve for more efficient Schwann cell isolation^44^. Nevertheless, given the high plasticity of Schwann cells^2^ and unwanted effects of long-lasting isolation protocols on the transcriptome, short-term isolation protocols are preferable. In contrast to myelinating and nonmyelinating Schwann cells, we found considerable numbers of keloidal profibrotic Schwann cells in all datasets, indicating that Schwann cells that are not associated with an axon can be easily isolated from the tissue.

To compare the phenotype of keloidal Schwann cells with those of normal scars, we also included scRNA-seq data from normal scars in our analyses. Interestingly, only after a comprehensive computation of all datasets together we were able to identify a few Schwann cells in normal scars. The lack of Schwann cells in normal scars is in line with a recent publication reporting an important role of Schwann cells during wound healing in mice^6^. Parfejevs et al. showed that murine Schwann cells dedifferentiate and re-enter the cell cycle after wounding and promote wound healing by releasing paracrine factors, thereby inducing myofibroblast differentiation^6^. Importantly, these Schwann cells completely disappeared from the wounded area after successful completion of wound healing^45^. Whether these Schwann cells convert into other cells, such as fibroblasts, or migrate back to an axon remains to be elucidated. The low numbers of Schwann cells detected in normal human scars suggest that such a mechanism might also be apparent in human wound healing and that the development of keloids might be associated with defects in removing these Schwann cells after wound healing. However, further studies are necessary to fully elucidate the impact of Schwann cells on human wound healing.

The adjacent skin around keloids has been reported to exhibit features typical of keloids^14^. Strikingly, our bioinformatics analysis also uncovered keloidal profibrotic Schwann cells in one dataset of keloid-adjacent skin. This finding supports our assumption that the persistence of keloidal Schwann cells in the dermis contributes to disease progression. Two scenarios by which these Schwann cells might affect keloid progression appear reasonable. First, keloidal Schwann cells spread into the surrounding healthy skin, generating a milieu that favors the growth of keloids. Second, the continuously growing keloids might affect nerves in the surrounding healthy skin and trigger dedifferentiation of Schwann cells, which in turn further contributes to ECM overproduction, as reported previously^17^. Our transcriptomic data and bioinformatics analyses have built a good basis for further studies addressing these open questions in more sophisticated in vivo studies.

Injury of peripheral nerves causes dedifferentiation of both types of mature Schwann cells (myelinating and nonmyelinating) into repair Schwann cells^46–48^. Our pseudotime trajectory calculations suggest that myelinating and nonmyelinating Schwann cells of the skin dedifferentiate into profibrotic, repair-like Schwann cells in keloids. Interestingly, many of the marker genes specifically expressed by bona fide repair Schwann cells (*STAT3, ARTN, BDNF, GDNF, SHH, OLIG1*, and *IGFBP2*) were not or only weakly detected in keloidal Schwann cells. However, c-Jun, a key factor for guiding dedifferentiation and enabling proper function of repair Schwann cells^4,47,49–52^, was also strongly expressed in keloidal Schwann cells, especially at the branching point of the pseudotime trajectory. However, in fully dedifferentiated keloidal Schwann cells, c-Jun expression levels were again decreased. Interestingly, our analyses revealed that several other transcription factors, including other AP-1 members (*JUNB, JUND, FOS, FOSB*), members of the Kruppel-like factor family (*KLF2, KLF4, KLF6, KLF10*) and members of the immediate early response gene family (*IRE2, IRE3, IER5, IER5L*), were similarly regulated, suggesting that these transcription factors might also play an important role in the dedifferentiation process of skin Schwann cells. Whereas the function of most of these factors in Schwann cells needs further in-depth investigation, several members of the KLF family have already been reported to be crucial for Schwann cell differentiation^53–57^. In particular, the high expression of KLF4 suggests that keloidal Schwann cells indeed underwent a dedifferentiation process. The reason for the lack of bona fide repair Schwann cell markers in keloidal Schwann cells is currently not known. As c-Jun is the major regulator of most of these marker genes^4^, its downregulation might be responsible for this observation. Although c-Jun activation is important for the induction of repair Schwann cell development after neural damage, chronic denervation was shown to strongly reduce c-Jun levels in Schwann cells^4^. We therefore hypothesize that due to the prolonged axon-free occurrence of Schwann cells in keloids, their expression pattern changes significantly. Whether these keloidal Schwann cells, despite the loss of expression of several repair marker genes, still represent functional repair Schwann cells has to be determined in future experiments. However, the comparison of our dataset with a dataset of an acute nerve lesion^5^ revealed that a high number of genes characteristic of repair Schwann cells were similarly regulated in both Schwann cell types, suggesting that at least some parts of the repair Schwann cell function are still conserved in keloidal Schwann cells.

Our cell‒cell interaction analysis suggests the involvement of fibroblasts in the conversion of myelinated and nonmyelinated Schwann cells into keloid-specific profibrotic Schwann cells, as a high communication probability has been detected for collagen-integrin and midkine-integrin interactions. Interestingly, these receptor‒ligand interactions have already been reported to be involved in cellular development, reprogramming, survival and proliferation, which is consistent with our analysis^58,59^. Furthermore, our analyses suggest that keloidal Schwann cells affect keratinocytes and smooth muscle cells/pericytes, especially by PTN–NCL interactions. Such interactions have been previously shown to be involved in tumorigenesis and cell migration, suggesting their involvement in the tumor-like growth of keloids^60^. Intriguingly, only minor interactions of Schwann cells with macrophages were detected in our analysis. This result is in stark contrast to our previous findings, where we described an important crosstalk of keloidal Schwann cells and macrophages, thereby contributing to the infinite growth of keloids^17^. As bioinformatics analyses always entail a certain risk of overlooking or overinterpreting biologic processes, further studies are needed to fully understand the whole communication network of Schwann cells in keloids and their contribution to keloid formation or progression.

As we have previously shown that keloidal Schwann cells significantly contribute to ECM formation^17^, it is conceivable that keloidal Schwann cells acquire profibrotic properties. This hypothesis is supported by the identified gene-set that contains several profibrotic genes (*COL1A1, COL7A1, ELN, IGFBP3, IGFBP5, ITGB1, LOXL2, MMP15, S100A16, SPARCL1, TAGLN, TGFBI, TPM2*). The identification of this specific gene-set enables further investigations of the contribution of Schwann cells to fibrosis in other fibrotic disorders, such as liver cirrhosis, idiopathic pulmonary fibrosis or renal fibrosis. Considering the current literature and our new data, we hypothesize that there are at least two types of repair(-like) Schwann cells. One type occurs temporarily after acute injury to functionally regenerate a disrupted nerve, and another type persists after complete tissue regeneration in keloids for a long time period, thereby acquiring profibrotic properties^4,6^. Why this Schwann cell type is not removed after wound healing is currently not known and matter of ongoing experiments.

In conclusion, we confirmed the presence of a repair-like, profibrotic Schwann cell type in keloids from three independent scRNA-seq datasets and identified a conserved expression pattern of twenty-one genes that specifically characterizes this Schwann cell type. This special repair-like, profibrotic Schwann cell type present in keloids exhibits distinct genetic differences compared to classical repair Schwann cells but presumably arises from a common initial injury event. Our study has built the foundation for further studies to investigate the frequency of these cells and the contribution to fibrotic processes in other organs. With respect to the described bioinformatics analyses, our study provides a practical strategy to reliably analyze even small cell clusters and obviate incorrect cluster assignment.

## Supplementary information

Supplementary Information

## Data Availability

All scRNA-seq data are publicly accessible in NCBI´s Gene Expression Omnibus (GSE181316, GSE163973), at the Genome Sequencing Archive (BioProject PRJCA003143) and under the following links: https://dom.pitt.edu/wp-content/uploads/2018/10/Skin_6Control_rawUMI.zip. https://dom.pitt.edu/wp-content/uploads/2018/10/Skin_6Control_Metadata.zip. The source codes for generating the data are accessible under the following link: https://github.com/Mwielscher/scRNAseq/tree/main/keloidal_Schwann.
