# Supplementary material for: The transcriptional profile of keloidal Schwann cells
Source: Exp Mol Med. 2022 Nov 4;54(11):1886–900. doi: 10.1038/s12276-022-00874-1 (PMC9722693; doi:10.1038/s12276-022-00874-1)
Supplement: Supplementary file 1 — Supplementary Information [file 12276_2022_874_MOESM1_ESM.pdf]

Supplementary Fig. 1

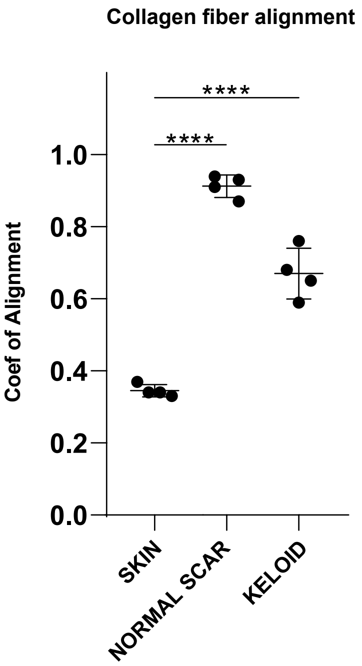

Supplementary Fig. 1: Collagen alignment in skin, normal scar and keloid
Evaluation of the alignment coefficient of healthy skin, normal scar and keloid tissue. Maximum and minimum value with <1.5 interquartile range is shown by whiskers. The middle line represents the mean. Normally distributed data were compared by one-way ANOVA with Tukey post hoc test. ****p <0.0001.

Supplementary Fig. 2

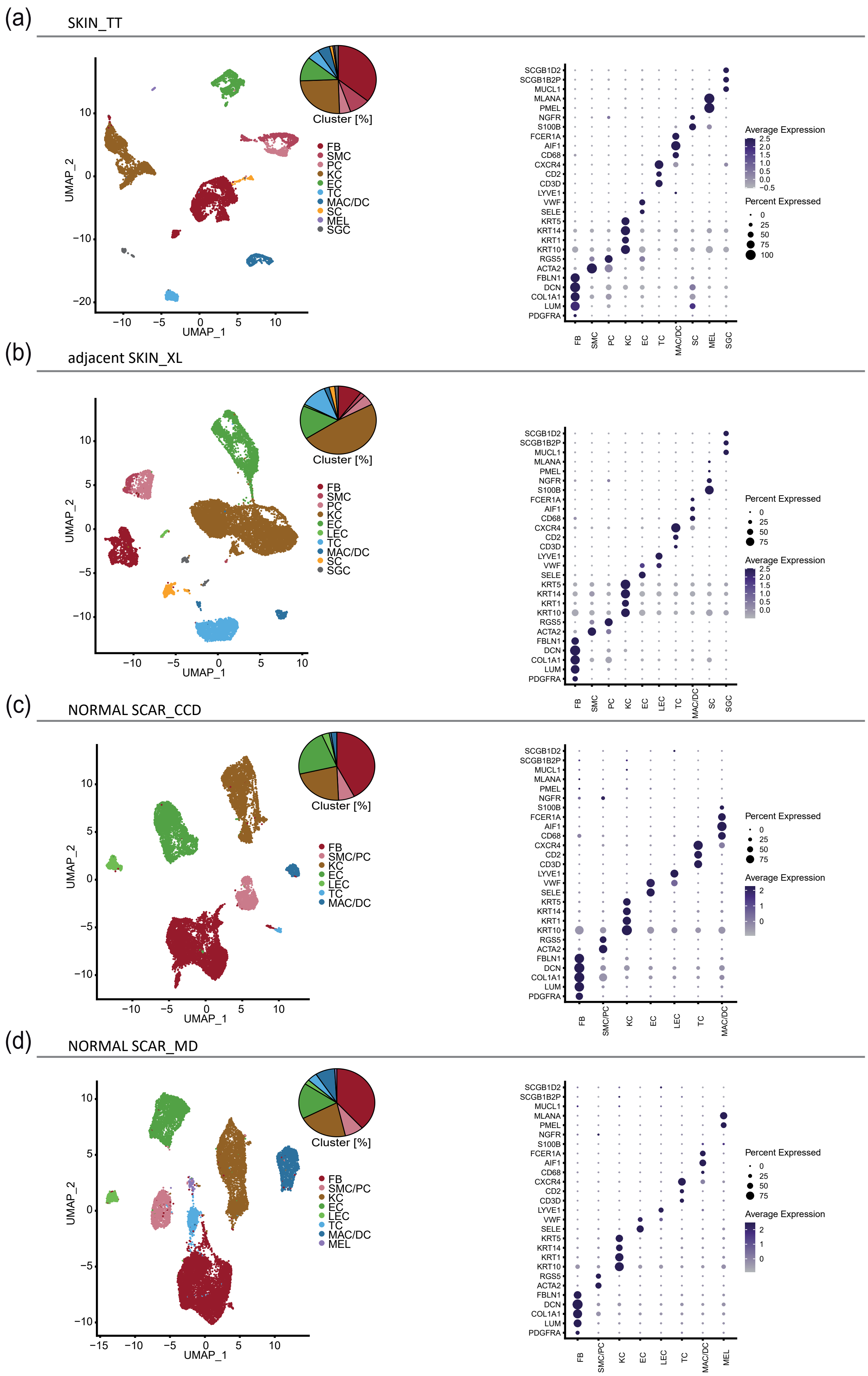

Supplementary Fig. 2: Individual dataset integration unravels cluster consistency

UMAP-Plots and Dot plots individual integrations of datasets from distinct conditions and sources: SKIN_TT, n=6 (a); adjacent SKIN_XL, n=4 (b); NORMAL SCAR_CCD, n=3 (c); NORMAL SCAR_MD, n=3 (d); cluster classification as fibroblasts (FB), smooth muscle cells (SMC), pericytes (PC), smooth muscle cells and pericytes (SMC/PC), keratinocytes (KC), endothelial cells (EC), lymphatic endothelial cells (LEC), T-cells (TC), macrophages (MAC), dendritic cells (DC), macrophages and dendritic cells (MAC/DC), Schwann cells (SC), melanocytes (MEL), erythrocytes (ERY) and sweat gland cells (SGC); Pie plots show cluster composition within each dataset combination. Dot plots showing well-known marker genes to characterise clusters: *PDGFRA*, *LUM*, *COL1A1*, *DCN*, *FBLN1* for FB; *ACTA2*, *RGS5* for SMC and PC; *KRT10*, *KRT1*, *KRT14*, *KRT5* for KC; *SELE*, *VWF* for EC; *LYVE1* for LEC; *CD3D*, *CD2*, *CXCR4* for TC; *CD68*, *AIF1* for MAC; *FCER1A* for DC; *S100B*, *NGFR* for SC; *PMEL*, *MLANA* for MEL; Dot size symbolizes percentage of cells expressing the gene, colour gradient shows level of average gene expression.

Supplementary Fig. 3

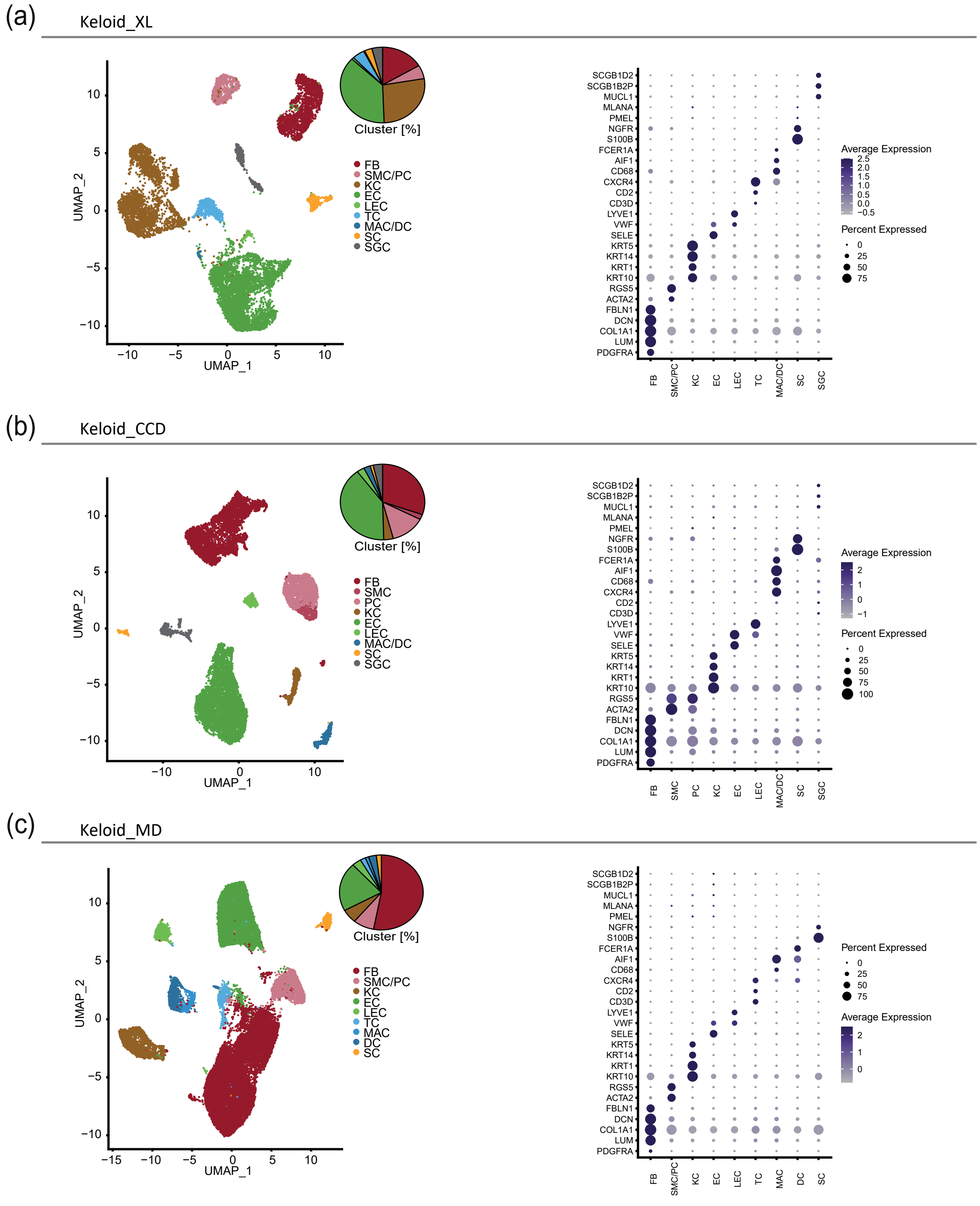

Supplementary Fig. 3: Individual dataset integration unravels cluster consistency
UMAP-plots and DotPlots individual integrations of datasets from distinct conditions and sources: KELOID_XL, n=4 (a); KELOID_CCD, n=3 (b); KELOID_MD, n=4 (c); cluster classification as fibroblasts (FB), smooth muscle cells (SMC), pericytes (PC), smooth muscle cells and pericytes (SMC/PC), keratinocytes (KC), endothelial cells (EC), lymphatic endothelial cells (LEC), T-cells (TC), macrophages (MAC), dendritic cells (DC), macrophages and dendritic cells (MAC/DC), Schwann cells (SC), melanocytes (MEL), erythrocytes (ERY) and sweat gland cells (SGC); Pie plots show cluster composition within each dataset combination. Dot plots showing well-known marker genes to characterise clusters: *PDGFRA*, *LUM*, *COL1A1*, *DCN*, *FBLN1* for FB; *ACTA2*, *RGS5* for SMC and PC; *KRT10*, *KRT1*, *KRT14*, *KRT5* for KC; *SELE*, *VWF* for EC; *LYVE1* for LEC; *CD3D*, *CD2*, *CXCR4* for TC; *CD68*, *AIF1* for MAC; *FCER1A* for DC; *S100B*, *NGFR* for SC; *PMEL*, *MLANA* for MEL; Dot size symbolizes percentage of cells expressing the gene, colour gradient shows level of average gene expression.

Supplementary Fig. 4

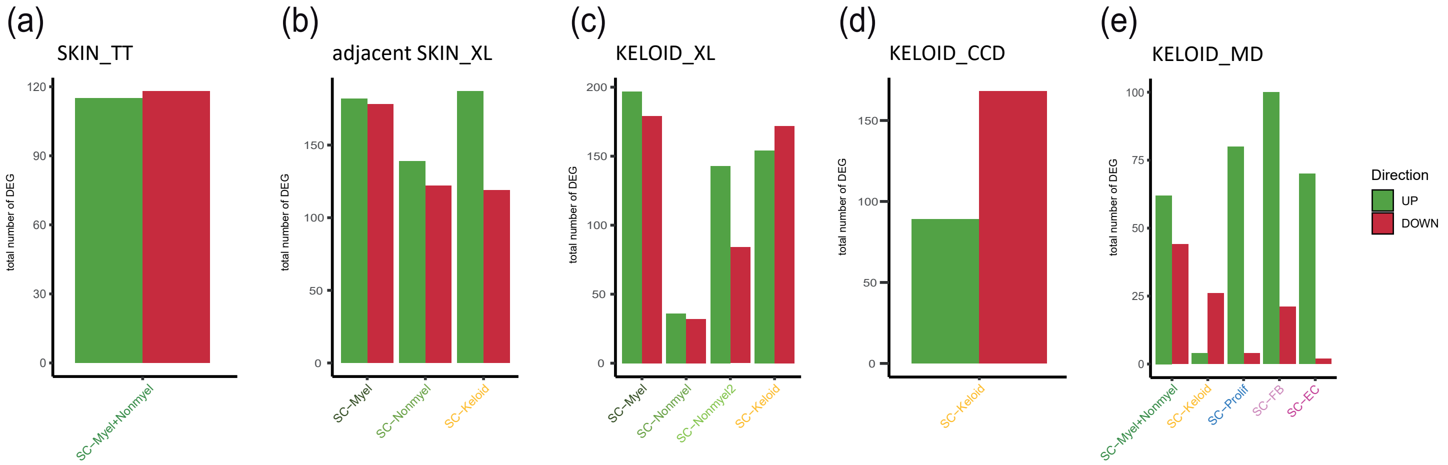

Supplementary Fig. 4: Total number of differentially expressed genes in each individual computation
Bar plots show the total number of differentially expressed genes (fold change ≥2) between all Schwann cell cluster of SKIN_TT (a); adjacent SKIN_XL (b); KELOID_XL (c); KELOID_CCD (d); KELOID_MD (e); green= UP regulation, red= DOWN regulation

Supplementary Fig. 5

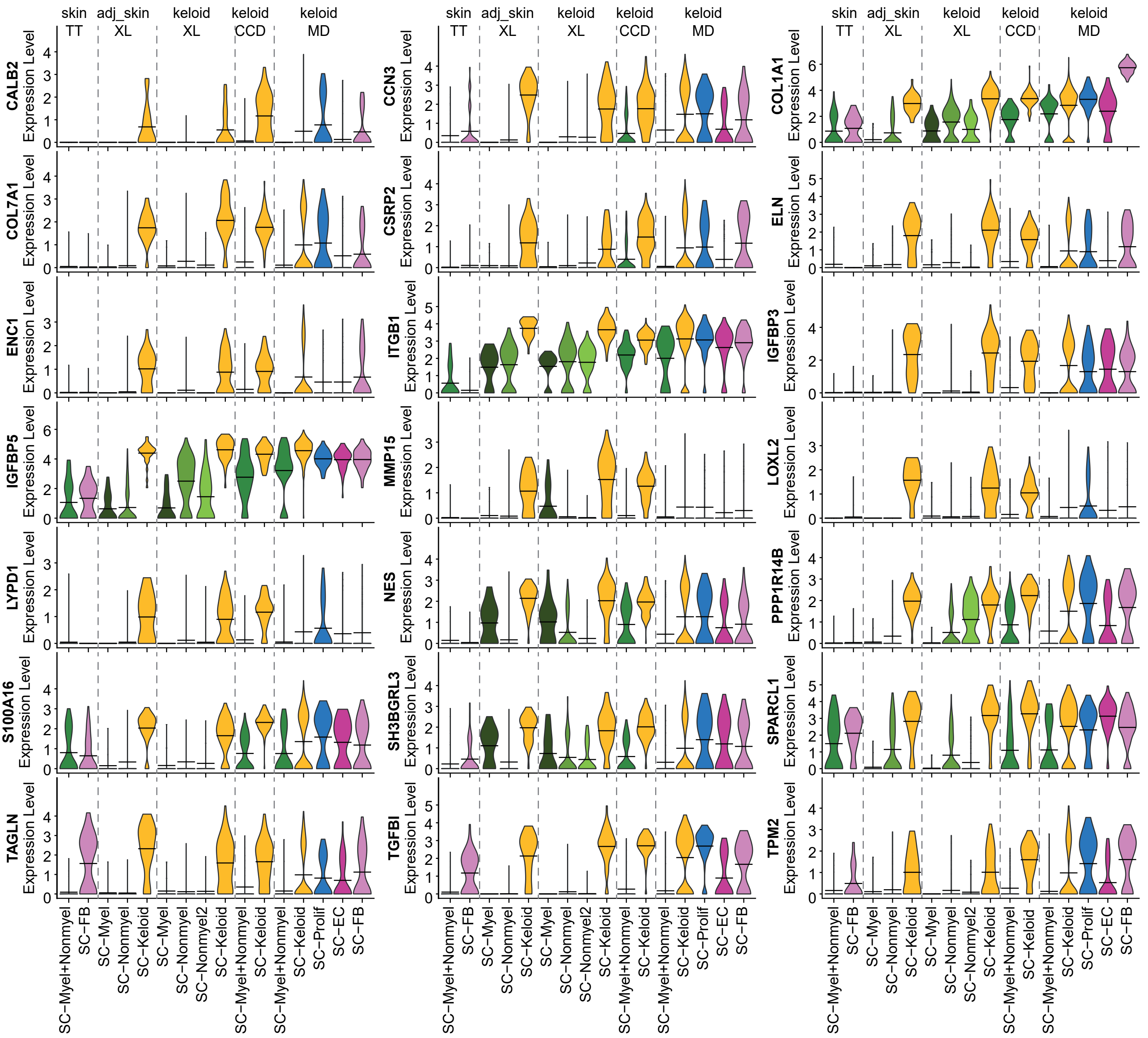

Supplementary Fig. 5: SC-Keloid pattern gene expression in all Schwann cell subtypes
Violin plots show the expression of the identified SC-Keloid expression pattern in all Schwann cell subtypes. Crossbeams mark the mean expression, vertical lines show maximum expression, violin width symbolizes frequency of cells on a distinct expression level.

Supplementary Fig. 6

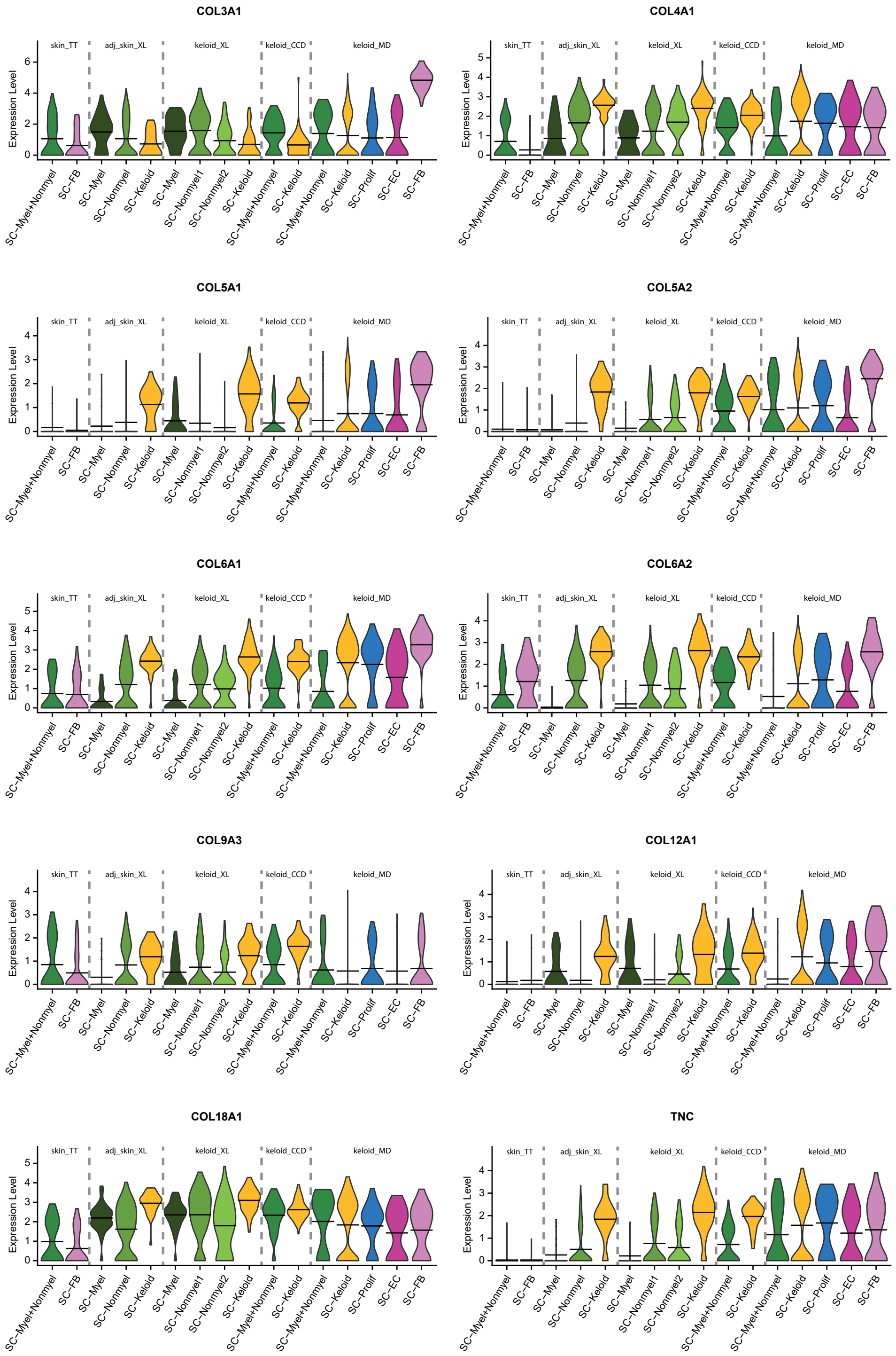

Supplementary Fig. 6: Extracellular matrix associated gene expression in SC-Keloid of distinct sources

Violin plots show the expression of several genes associated with matrix formation in all Schwann cell subtypes. Crossbeams mark the mean expression, vertical lines show maximum expression, violin width symbolizes frequency of cells on a distinct expression level.

Supplementary Fig. 7

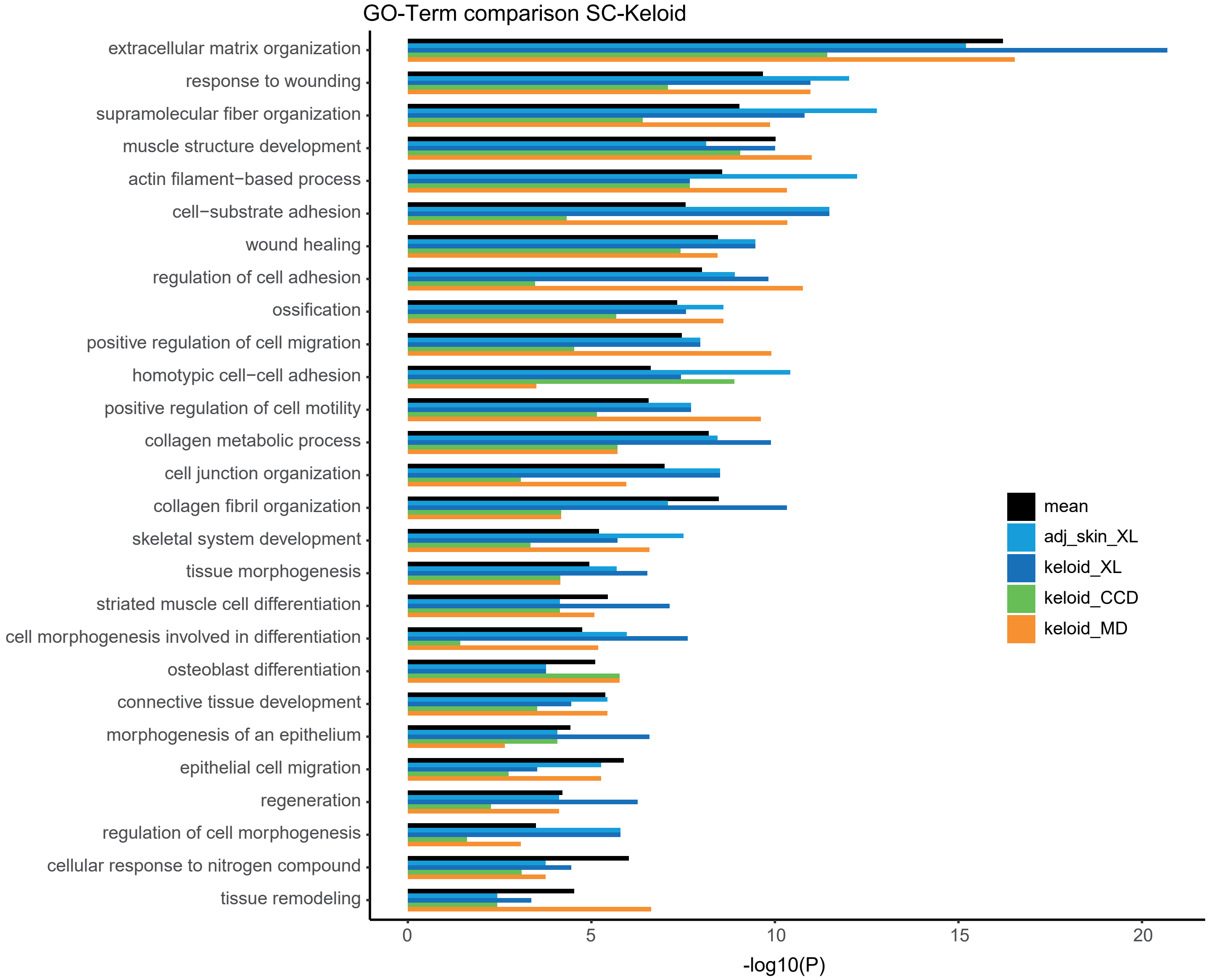

Supplementary Fig. 7: Corresponding GO-Terms encourage reported functions of keloidal Schwann cells
Enrichment analysis of top 100 upregulated genes resulted by data confrontation of SC-Keloid with myelination and nonmyelinating Schwann cells. Bar length shows statistical significance; mean value is depicted in black.

Supplementary Fig. 8

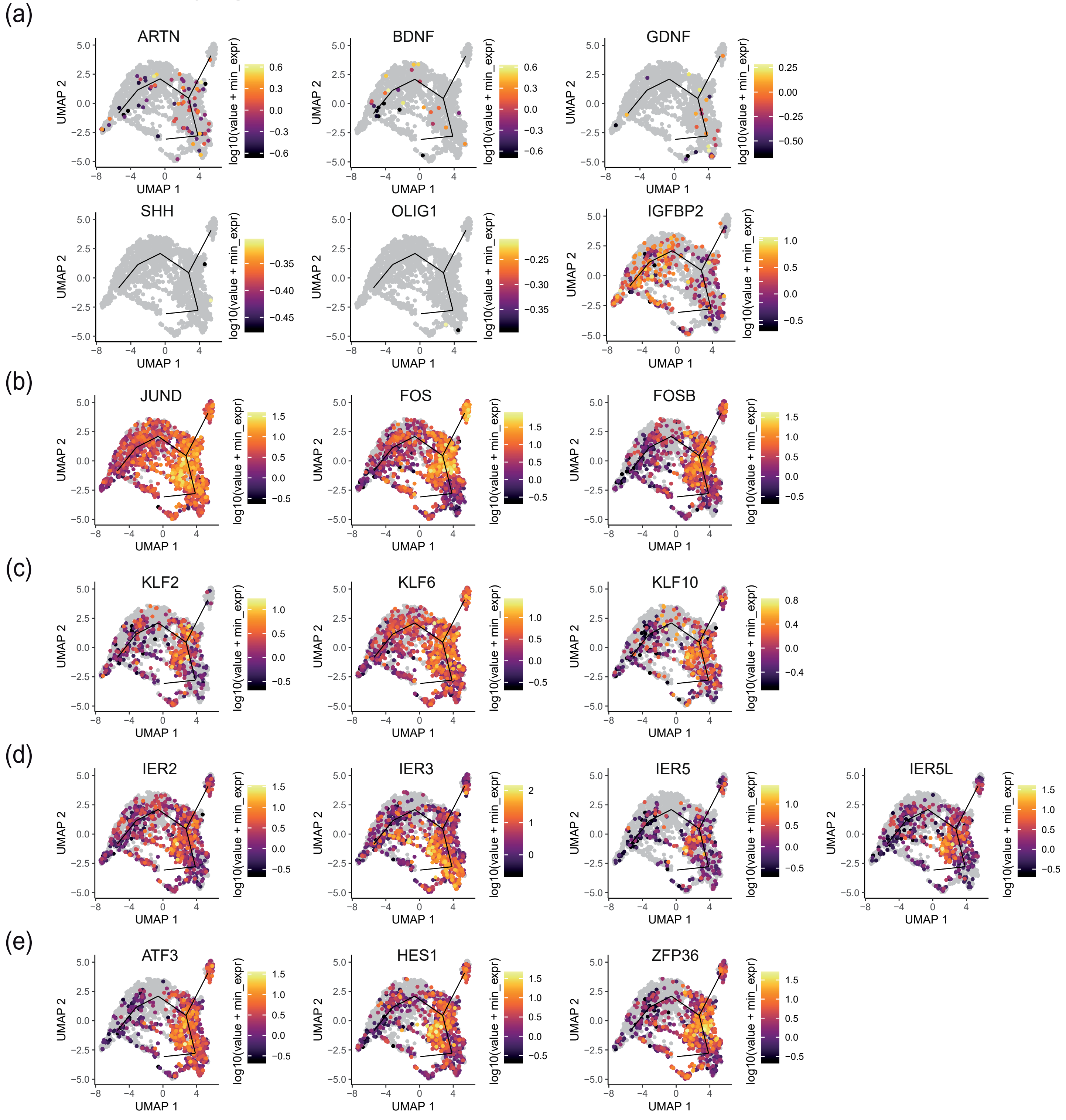

Supplementary Fig. 8: Pivotal gene groups potentially involved in the Schwann cell dedifferentiation process
(a) Feature Plots with pseudotime track of BDNF, GDNF, *ARTN*, *SHH*, *OLIG1* and *IGFBP2*. (b) Feature Plots with UMAP Plot of combined Schwann cell clusters of all individual computations as basis showing activator protein 1 (AP-1) members *JUND*, *FOS* and *FOSB*. (c) Feature Plots of the kruppel-like factors (KLF) members *KLF2*, *KLF6* and *KLF10*. (d) Feature Plots of the immediate early response (IER) genes *IER2*, *IER3*, *IER5L* and *IER5*. (e) Feature Plots of *ATF3*, *HES1* and *ZFP36*.

Supplementary Fig. 9

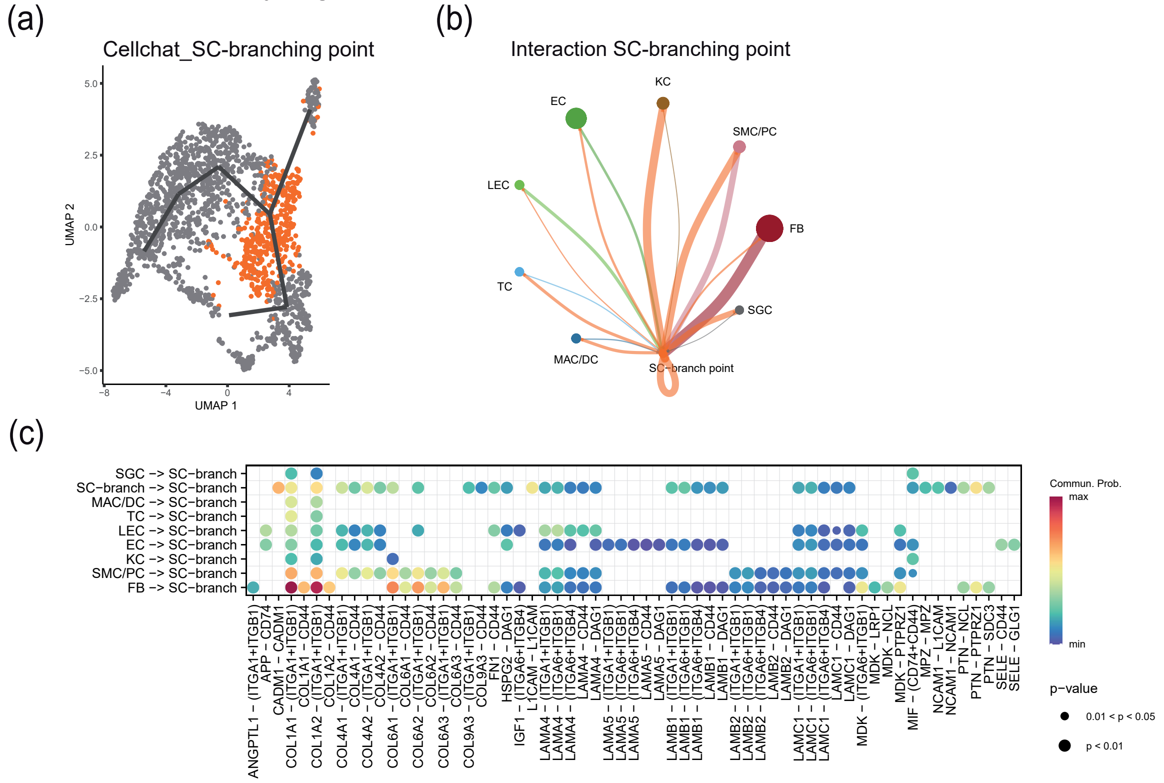

Supplementary Fig. 9: Potential impact of different cell types in the Schwann cell dedifferentiation process

(a) UMAP-Plot of combined Schwann cell clusters with determining cells at the branching point highlighted in orange. (b) Circle plot of detected interactions between the distinct keloidal cell types and the determining Schwann cells at the branching point; fibroblasts (FB), smooth muscle cells and pericytes (SMC/PC), keratinocytes (KC), endothelial cells (EC), lymphatic endothelial cells (LEC), T-cells (TC), macrophages and dendritic cells (MAC/DC), sweat gland cells (SGC); bows are coloured according to the singal-sending cell type; bow thickness symbolizes extent of detected interactions. (c) Dotplot shows all identified interactions of the distinct keloidal cell types with the pivotal Schwann cells at the branching point; dots symbolize a detected interaction, dotsize shows p-value, dot colour depicts extent of the communication probability.

Supplementary Fig. 10

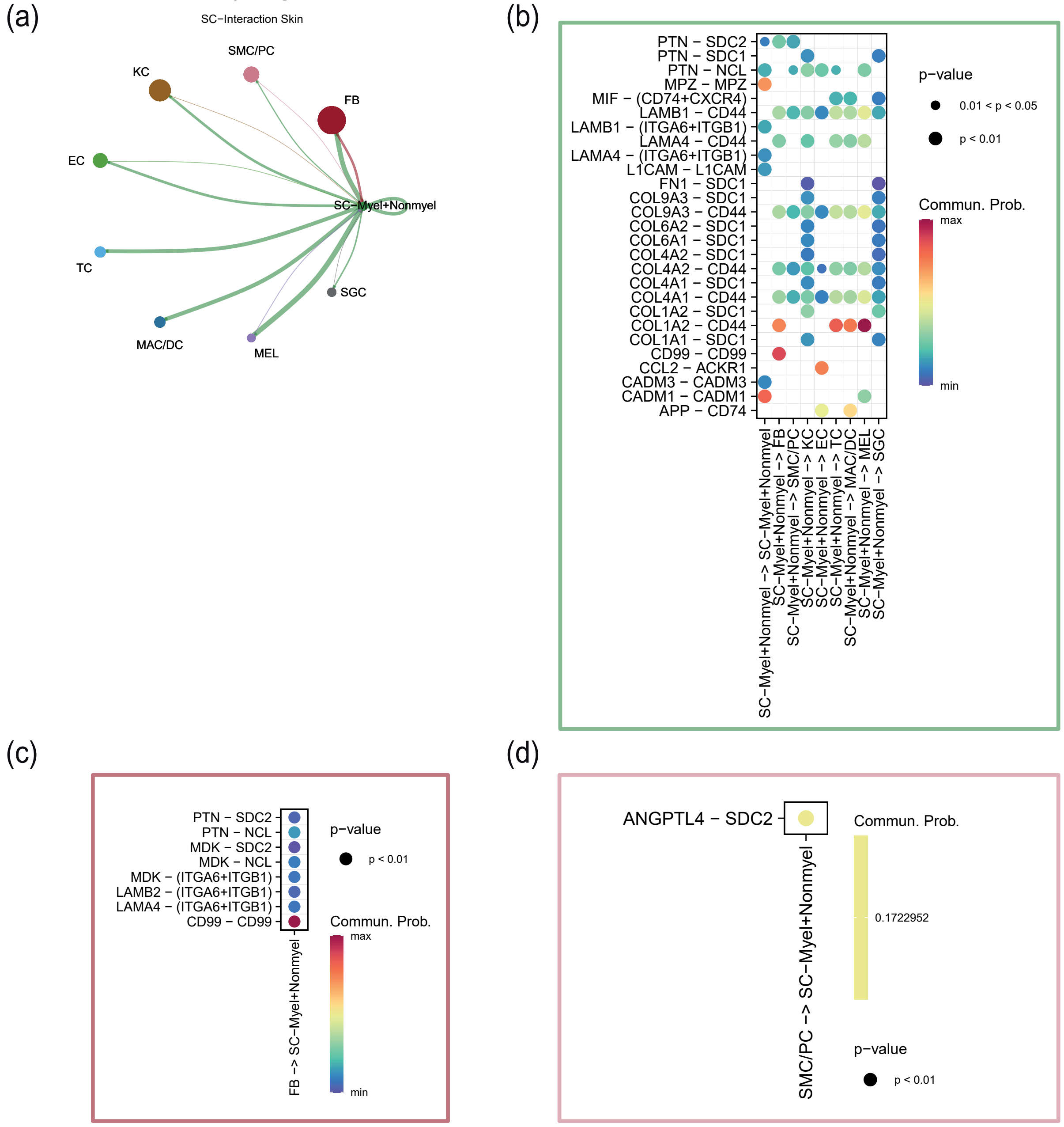

Supplementary Fig. 10: Potential Schwann cell interaction in healthy skin.
(a) Circle plot of the detected interactions in healthy skin between myelinating + nonmyelinating Schwann cells (SC-Myel+Nonmyel) and the remaining cell types [fibroblasts (FB), smooth muscle cells and pericytes (SMC/PC), keratinocytes (KC), endothelial cells (EC), T-cells (TC), macrophages and dendritic cells (MAC/DC), melanocytes (MEL), sweat gland cells (SGC)]; bows are coloured according to the singal-sending celltype; bow thickness symbolizes extent of detected interactions. Dotplots show all identified interactions with cells present in keloids sent by (b) SC-Myel+Nonmyel, (c) FB and (d) SMC/PC; dots symbolize a detected interaction, dotsize shows p-value, dot colour depicts extent of the communication probability.

Supplementary Fig. 11

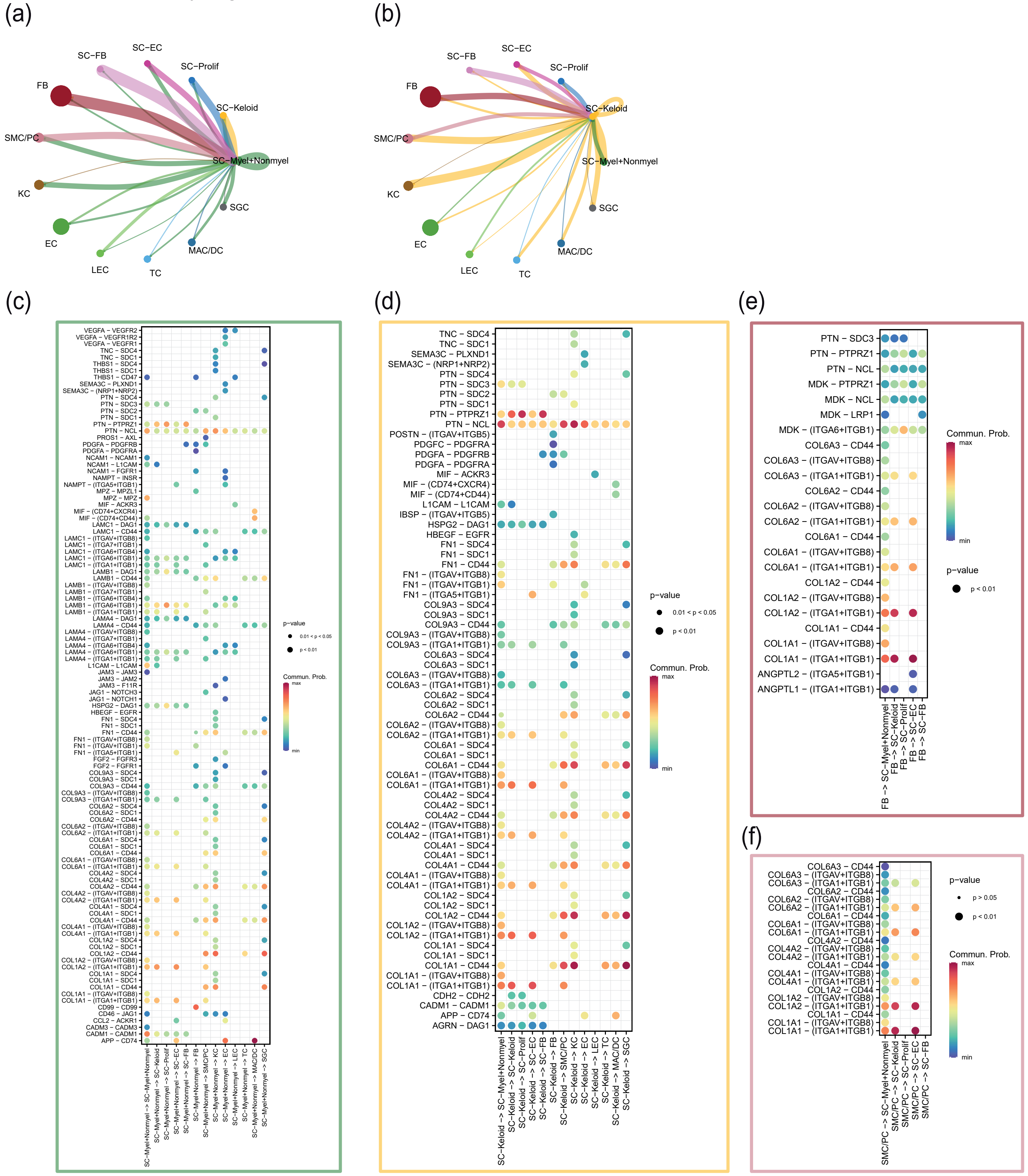

Supplementary Fig. 11: Potential Schwann cell interaction in keloids .
Circle plots of detected interactions in keloids between (a) myelinating and nonmyelinating Schwann cells (SC-Myel+Nonmyel), (b) keloidal Schwann cells (SC-Keloid) and the remaining cell types; fibroblasts (FB), smooth muscle cells and pericytes (SMC/PC), keratinocytes (KC), endothelial cells (EC), lymphatic endothelial cells (LEC), T-cells (TC), macrophages and dendritic cells (MAC/DC), sweat gland cells (SGC), proliferating Schwann cells (SC-Prolif), Schwann cells expressing genes typical for endothelial cells (SC-EC) or fibroblasts (SC-FB); bows are coloured according to the singal-sending cell type; bow thickness symbolizes extent of detected interactions. Dotplots show all identified interactions with cells present in keloids sent by (c) SC-Myel+Nonmyel, (d) SC-Keloid, (e) FB and (f) SMC/PC; dots symbolize a detected interaction, dotsize shows p-value, dot colour depicts extent of the communication probability.

Supplementary Table 1

Differentially expressed genes_adjacent-skin-XL_ comparing SC-Keloid vs. (SC-Myel, SC-Nonmyel)								
**gene**	**p_val**	**avg_log2FC**	**pct.1**	**pct.2**	**p_val_adj**	**Foldchange_UP**	**Foldchange_DOWN**	**Ratio_pct1_pct2**
IGFBP5	3,75985E-24	4,172214618	1	0,32	7,93367E-20	18,02858943	0,055467457	3,125
TAGLN	8,41937E-42	4,034051171	0,844	0,031	1,77657E-37	16,38213146	0,061042118	27,22580645
IGFBP3	5,44115E-46	3,979903772	0,906	0,027	1,14814E-41	15,7786708	0,063376695	33,55555556
TGFBI	7,82192E-48	3,809347934	0,812	0,008	1,6505E-43	14,01935367	0,071329965	101,5
CCN3	2,74623E-40	3,553600279	0,938	0,059	5,79481E-36	11,74195135	0,08516472	15,89830508
PMEPA1	6,37599E-25	2,991178137	1	0,289	1,3454E-20	7,951230464	0,125766698	3,460207612
ELN	9,39877E-25	2,744572089	0,812	0,105	1,98323E-20	6,701908962	0,149211218	7,733333333
COL1A1	2,80222E-20	2,687854737	1	0,328	5,91296E-16	6,443545503	0,155194062	3,048780488
LOXL2	1,24566E-57	2,566847202	0,906	0	2,62846E-53	5,925131621	0,168772622	#DIV/0!
APOD	1,28159E-24	2,497995728	0,75	0,074	2,70428E-20	5,649000888	0,177022454	10,13513514
ITGB1	1,17015E-18	2,47289199	1	0,715	2,46913E-14	5,551555232	0,180129704	1,398601399
NES	1,65975E-27	2,445557305	0,969	0,16	3,50224E-23	5,447360344	0,183575151	6,05625
COL7A1	3,6255E-41	2,436012037	0,938	0,051	7,65017E-37	5,411438076	0,184793762	18,39215686
S100A16	2,91126E-22	2,328345725	0,906	0,207	6,14305E-18	5,022291356	0,199112303	4,376811594
COL8A1	5,14801E-33	2,326828989	0,875	0,07	1,08628E-28	5,017014086	0,199321745	12,5
PTN	7,39689E-16	2,298231868	0,969	0,422	1,56082E-11	4,918545907	0,203312121	2,296208531
FN1	1,301E-16	2,268482516	0,969	0,348	2,74525E-12	4,818160713	0,207548079	2,784482759
PDLIM7	4,71905E-37	2,256920578	0,875	0,055	9,95767E-33	4,779701683	0,209218078	15,90909091
S100A13	1,8595E-21	2,221078711	0,938	0,207	3,92374E-17	4,662419158	0,214480931	4,531400966
PCDH20	1,15617E-21	2,200924696	0,875	0,141	2,43964E-17	4,597739399	0,217498191	6,205673759
TIMP4	2,68071E-35	2,195462089	0,75	0,027	5,65656E-31	4,580363477	0,218323285	27,77777778
PPP1R14B	1,23462E-23	2,120857034	0,969	0,191	2,60518E-19	4,349522521	0,229910294	5,073298429
MYL6	1,03389E-17	2,011367588	1	0,715	2,18161E-13	4,031642142	0,248037887	1,398601399
SH3BGRL3	1,28347E-19	1,995802282	0,969	0,258	2,70824E-15	3,98837837	0,250728468	3,755813953
PFN1	3,5014E-17	1,978006745	0,969	0,469	7,3883E-13	3,939484189	0,253840339	2,066098081
CSRP2	2,098E-25	1,97308897	0,719	0,059	4,42699E-21	3,926078368	0,254707091	12,18644068
COL5A2	2,67889E-19	1,898472142	0,938	0,203	5,65273E-15	3,728181616	0,268227276	4,620689655
TMSB10	1,17749E-15	1,88373001	1	0,91	2,48462E-11	3,690279297	0,270982199	1,098901099
COL1A2	5,80342E-15	1,875042622	1	0,578	1,22458E-10	3,66812454	0,272618879	1,730103806
SPARCL1	3,94377E-10	1,841613943	0,875	0,406	8,32175E-06	3,584107584	0,279009482	2,155172414

Supplementary Table 1

Differentially expressed genes_adjacent-skin-XL_ comparing SC-Keloid vs. (SC-Myel, SC-Nonmyel)								
gene	p_val	avg_log2FC	pct.1	pct.2	p_val_adj	Foldchange_UP	Foldchange_DOWN	Ratio_pct1_pct2
CYTL1	4,2357E-43	1,809547978	0,688	0	8,93774E-39	3,505324434	0,285280298	#DIV/0!
ACTB	1,38321E-13	1,783939308	1	0,871	2,91871E-09	3,443651882	0,290389399	1,148105626
MYL9	1,156E-11	1,781799974	0,938	0,449	2,43928E-07	3,438549165	0,290820329	2,08908686
LYPD1	1,45653E-28	1,775847192	0,656	0,031	3,07342E-24	3,424390413	0,292022778	21,16129032
IGFBP2	2,52383E-19	1,764717606	0,5	0,031	5,32554E-15	3,398074789	0,294284282	16,12903226
RAB13	1,23228E-26	1,762061509	0,875	0,105	2,60024E-22	3,391824465	0,294826578	8,333333333
CALB2	3,56695E-31	1,755884321	0,5	0	7,52662E-27	3,377332736	0,296091643	#DIV/0!
TNFAIP6	2,41712E-12	1,74427061	0,875	0,316	5,10036E-08	3,350254308	0,298484804	2,768987342
GAPDH	1,56837E-12	1,741285951	1	0,633	3,30941E-08	3,343330438	0,299102951	1,579778831
EMP3	9,37977E-13	1,728605057	0,938	0,391	1,97923E-08	3,314072255	0,301743572	2,398976982
S100A6	1,59049E-17	1,723852974	1	0,973	3,35609E-13	3,303174013	0,302739122	1,027749229
HMCN2	4,51596E-39	1,723330261	0,625	0	9,52913E-35	3,301977434	0,302848829	#DIV/0!
CD151	1,63957E-15	1,718128058	0,938	0,344	3,45966E-11	3,290092301	0,303942841	2,726744186
TNC	1,43898E-14	1,713868253	0,906	0,27	3,03638E-10	3,280392066	0,30484161	3,355555556
ENC1	6,16782E-35	1,677679442	0,75	0,027	1,30147E-30	3,199129608	0,312585022	27,77777778
PLAC9	1,92455E-18	1,664335135	0,719	0,098	4,061E-14	3,169675468	0,315489712	7,336734694
ANGPTL7	2,52819E-06	1,66379677	0,344	0,07	0,053347327	3,168492872	0,315607464	4,914285714
TUBB3	1,48872E-18	1,656575137	0,562	0,051	3,14135E-14	3,15267212	0,317191247	11,01960784
TPM4	9,21357E-15	1,64592728	0,969	0,414	1,94416E-10	3,12948938	0,319540947	2,34057971
ACTG1	8,79291E-11	1,638655876	0,938	0,855	1,85539E-06	3,113755956	0,321155548	1,097076023
ACTN1	7,19892E-23	1,614400712	0,844	0,113	1,51904E-18	3,061843856	0,326600587	7,469026549
ANGPTL2	2,27734E-28	1,601632401	0,781	0,059	4,80542E-24	3,034865123	0,329503935	13,23728814
SCX	1,03577E-15	1,598780221	0,375	0,02	2,18557E-11	3,028871181	0,330156002	18,75
MMP15	6,43568E-28	1,598644479	0,75	0,051	1,35799E-23	3,028586209	0,330187068	14,70588235
CCN2	1,65147E-21	1,59651362	0,719	0,074	3,48477E-17	3,024116293	0,330675114	9,716216216
S100A11	2,99964E-14	1,589368915	1	0,656	6,32953E-10	3,009176888	0,332316789	1,524390244
ATP5F1E	3,51941E-13	1,573708754	0,938	0,59	7,42632E-09	2,976689527	0,335943669	1,589830508
COL6A2	2,07111E-12	1,535338998	0,969	0,559	4,37025E-08	2,89856532	0,344998263	1,733452594
SLC15A3	5,08786E-23	1,516904404	0,812	0,094	1,07359E-18	2,861763407	0,349434897	8,638297872
CLIC1	3,45125E-13	1,514949366	0,906	0,359	7,28247E-09	2,857887975	0,349908747	2,52367688

Supplementary Table 1

Differentially expressed genes_adjacent-skin-XL_ comparing SC-Keloid vs. (SC-Myel, SC-Nonmyel)								
**gene**	**p_val**	**avg_log2FC**	**pct.1**	**pct.2**	**p_val_adj**	**Foldchange_UP**	**Foldchange_DOWN**	**Ratio_pct1_pct2**
TPM2	9,93652E-13	1,508973263	0,625	0,113	2,0967E-08	2,846074177	0,351361187	5,530973451
TNFRSF12A	8,08358E-11	1,508331261	0,844	0,332	1,70572E-06	2,844807949	0,351517578	2,542168675
CAMK2N1	4,02831E-22	1,507344125	0,781	0,09	8,50013E-18	2,842862112	0,351758179	8,677777778
MYL12A	2,2593E-12	1,507243911	0,938	0,461	4,76735E-08	2,842664644	0,351782614	2,034707158
NRXN2	4,06234E-26	1,498981596	0,531	0,016	8,57194E-22	2,826431231	0,353803053	33,1875
MMP14	8,92376E-17	1,487219812	0,812	0,16	1,883E-12	2,803482009	0,356699275	5,075
COL12A1	8,67368E-19	1,486346494	0,844	0,133	1,83023E-14	2,801785469	0,356915264	6,345864662
SLN	4,09886E-14	1,476862577	0,219	0	8,649E-10	2,783427638	0,359269264	#DIV/0!
ANXA1	2,01285E-13	1,474887149	0,969	0,516	4,24732E-09	2,779619003	0,359761535	1,877906977
LSM7	2,87271E-15	1,460109946	0,875	0,23	6,06171E-11	2,751293301	0,363465429	3,804347826
PLS3	9,75771E-20	1,449626818	0,875	0,141	2,05897E-15	2,731373897	0,366116115	6,205673759
RCN2	4,40851E-18	1,447215095	0,906	0,184	9,3024E-14	2,726811731	0,366728656	4,923913043
MIA	4,02272E-12	1,441904453	1	0,406	8,48835E-08	2,716792634	0,368081092	2,463054187
P4HB	7,4364E-14	1,435190889	0,938	0,305	1,56915E-09	2,704179444	0,369797944	3,075409836
CCND1	2,17438E-12	1,422847949	0,812	0,246	4,58815E-08	2,681142592	0,372975314	3,300813008
KCNMA1	3,45974E-32	1,421534097	0,688	0,023	7,3004E-28	2,678702007	0,373315134	29,91304348
ADAMTS16	1,74045E-31	1,41888453	0,594	0,012	3,67253E-27	2,673786978	0,374001373	49,5
CDK2AP1	1,07941E-15	1,414723433	0,906	0,242	2,27765E-11	2,666086212	0,375081644	3,743801653
MMP2	8,21849E-24	1,414030664	0,656	0,047	1,73418E-19	2,664806289	0,375261798	13,95744681
SERPINE2	6,08488E-17	1,411572204	0,625	0,078	1,28397E-12	2,660269127	0,375901818	8,012820513
TMSB4X	5,68687E-12	1,394562976	1	0,816	1,19999E-07	2,629088992	0,38035989	1,225490196
CALM1	6,7871E-12	1,39026314	1	0,633	1,43215E-07	2,621264869	0,381495213	1,579778831
DYNLT1	6,99478E-21	1,385087959	0,75	0,094	1,47597E-16	2,611878812	0,382866156	7,978723404
COL6A3	1,13298E-15	1,382320339	0,375	0,02	2,39071E-11	2,606873071	0,383601339	18,75
S100A10	6,13335E-15	1,380588303	1	0,922	1,2942E-10	2,603745251	0,38406215	1,084598698
CDH6	7,54585E-24	1,375899404	0,75	0,066	1,59225E-19	2,595296564	0,38531242	11,36363636
IL1RAP	8,28871E-31	1,368108473	0,719	0,031	1,749E-26	2,581319057	0,387398837	23,19354839
TAGLN2	2,08858E-11	1,343286772	0,969	0,566	4,40711E-07	2,537287098	0,394121738	1,712014134
CFL1	4,08292E-14	1,343259148	0,969	0,77	8,61537E-10	2,537238516	0,394129284	1,258441558
DSEL	5,15818E-17	1,340063808	0,656	0,086	1,08843E-12	2,531625156	0,395003185	7,627906977

Supplementary Table 1

Differentially expressed genes_adjacent-skin-XL_ comparing SC-Keloid vs. (SC-Myel, SC-Nonmyel)								
**gene**	**p_val**	**avg_log2FC**	**pct.1**	**pct.2**	**p_val_adj**	**Foldchange_UP**	**Foldchange_DOWN**	**Ratio_pct1_pct2**
CALR	8,34849E-10	1,323024364	0,938	0,473	1,76162E-05	2,501900412	0,399696165	1,983086681
HSBP1	2,42266E-14	1,296607018	1	0,344	5,11206E-10	2,456504731	0,407082464	2,906976744
ACTA2	4,01004E-16	1,295386193	0,312	0,008	8,46158E-12	2,454426888	0,407427088	39
S100A4	1,28526E-06	1,292866627	1	0,625	0,027120371	2,450144145	0,408139253	1,6
NGFR	3,33899E-09	1,292185651	0,875	0,355	7,04561E-05	2,448987908	0,408331947	2,464788732
PKM	1,2732E-10	1,289333729	0,844	0,262	2,68659E-06	2,444151527	0,409139936	3,221374046
LGALS1	1,52054E-11	1,288943421	1	0,906	3,20849E-07	2,443490374	0,40925064	1,103752759
PDIA4	1,01488E-17	1,287868345	0,75	0,109	2,1415E-13	2,441670198	0,409555722	6,880733945
ARPC2	2,82933E-12	1,286865545	0,906	0,324	5,97017E-08	2,439973612	0,409840498	2,796296296
PPIB	3,38502E-11	1,276469243	0,969	0,473	7,14272E-07	2,422453953	0,412804544	2,048625793
OST4	2,25501E-11	1,266662562	0,906	0,426	4,7583E-07	2,406043226	0,415620131	2,126760563
NME2	4,73546E-11	1,263020178	0,969	0,488	9,9923E-07	2,399976331	0,416670776	1,985655738
PRSS23	4,91333E-08	1,260981224	0,781	0,309	0,001036761	2,396586852	0,417260071	2,527508091
CDH2	1,73906E-20	1,255844191	0,688	0,07	3,66958E-16	2,388068453	0,418748465	9,828571429
ATOX1	2,77625E-12	1,253386907	0,688	0,156	5,85817E-08	2,384004414	0,419462311	4,41025641
ARL4C	2,86381E-21	1,249733683	0,562	0,035	6,04293E-17	2,377975222	0,420525828	16,05714286
PRRX1	2,43387E-27	1,246116457	0,719	0,043	5,13571E-23	2,372020465	0,421581523	16,72093023
RHOA	1,51547E-10	1,237334437	1	0,562	3,1978E-06	2,357625284	0,424155614	1,779359431
TPPP3	6,51683E-08	1,236848674	0,5	0,121	0,001375115	2,356831592	0,424298454	4,132231405
NDUFA4	5,84138E-11	1,227422752	0,969	0,477	1,23259E-06	2,341483305	0,427079705	2,031446541
VDAC1	7,79493E-17	1,22592124	0,781	0,133	1,64481E-12	2,339047631	0,427524428	5,872180451
MAFB	1,41998E-14	1,210899788	0,625	0,094	2,99629E-10	2,314819637	0,4319991	6,64893617
SERF2	2,43532E-11	1,202355557	1	0,785	5,13877E-07	2,301150843	0,434565167	1,27388535
FABP5	2,17913E-10	1,200083872	0,656	0,16	4,59819E-06	2,297530275	0,435249977	4,1
RGS3	7,4854E-13	1,19639886	0,75	0,156	1,57949E-08	2,291669282	0,436363138	4,807692308
PLOD1	1,66175E-26	1,191718688	0,656	0,035	3,50646E-22	2,284247043	0,43778102	18,74285714
COL6A1	6,21281E-10	1,189532608	0,969	0,547	1,31097E-05	2,280788401	0,438444881	1,771480804
MAGED2	4,93379E-11	1,187662294	0,938	0,355	1,04108E-06	2,277833497	0,439013651	2,642253521
MYL12B	6,5859E-11	1,181781435	1	0,531	1,38969E-06	2,268567262	0,440806855	1,883239171
ARPC3	1,71857E-11	1,179544151	0,906	0,301	3,62636E-07	2,265051968	0,441490974	3,009966777

Supplementary Table 1

Differentially expressed genes_adjacent-skin-XL_ comparing SC-Keloid vs. (SC-Myel, SC-Nonmyel)								
**gene**	**p_val**	**avg_log2FC**	**pct.1**	**pct.2**	**p_val_adj**	**Foldchange_UP**	**Foldchange_DOWN**	**Ratio_pct1_pct2**
GNG5	1,42897E-11	1,1726542	0,938	0,391	3,01528E-07	2,254260435	0,443604468	2,398976982
TIMM13	8,62823E-17	1,166721002	0,75	0,121	1,82064E-12	2,245008648	0,445432583	6,198347107
ECM1	1,10576E-13	1,163218923	0,75	0,137	2,33327E-09	2,239565596	0,446515164	5,474452555
COL18A1	6,02772E-10	1,153507631	1	0,727	1,27191E-05	2,224540911	0,449530955	1,375515818
NDUFS6	5,58667E-13	1,151401883	0,906	0,25	1,17884E-08	2,221296354	0,450187566	3,624
COTL1	5,80568E-13	1,148772448	0,844	0,211	1,22506E-08	2,217251539	0,45100882	4
CIAO2B	2,76951E-09	1,148531201	0,906	0,328	5,84394E-05	2,216880802	0,451084244	2,762195122
CTSZ	4,30946E-13	1,148349801	0,781	0,18	9,09338E-09	2,216602076	0,451140965	4,338888889
CYTOR	6,03429E-17	1,141277513	0,594	0,062	1,27329E-12	2,205762579	0,45335795	9,580645161
RTN1	2,20066E-25	1,141166372	0,406	0	4,64361E-21	2,205592661	0,453392876	#DIV/0!
FLNA	2,24119E-09	1,136814709	1	0,461	4,72914E-05	2,198949861	0,454762529	2,169197397
SEM1	2,71423E-09	1,134998682	0,812	0,305	5,72729E-05	2,196183621	0,455335333	2,662295082
BEX3	2,1903E-10	1,1348396	0,906	0,305	4,62174E-06	2,195941467	0,455385544	2,970491803
FKBP1A	2,85998E-09	1,129027162	0,844	0,293	6,03483E-05	2,187112092	0,457223936	2,880546075
ANXA2	3,2262E-08	1,12635282	1	0,863	0,000680761	2,18306157	0,458072284	1,158748552
PHLDA2	2,62142E-21	1,122011436	0,688	0,066	5,53147E-17	2,176502136	0,4594528	10,42424242
STMP1	5,16133E-16	1,120662538	0,781	0,133	1,08909E-11	2,17446809	0,459882582	5,872180451
APRT	6,69526E-15	1,120215911	0,844	0,172	1,41277E-10	2,173795027	0,460024974	4,906976744
DNAJC15	1,76927E-21	1,119429836	0,781	0,09	3,73333E-17	2,172610923	0,460275694	8,677777778
PSMA7	3,89791E-09	1,117511514	0,812	0,34	8,22497E-05	2,169723967	0,46088812	2,388235294
PTMS	3,25483E-10	1,115459343	0,969	0,562	6,86801E-06	2,166639823	0,46154418	1,724199288
MARCKSL1	1,2713E-13	1,113996863	0,844	0,188	2,68258E-09	2,164444582	0,462012291	4,489361702
TXNDC17	2,38135E-19	1,111774424	0,719	0,086	5,02489E-15	2,161112872	0,462724559	8,360465116
CALD1	4,97638E-09	1,111545361	0,906	0,418	0,000105007	2,160769769	0,462798033	2,167464115
CCT5	5,11095E-14	1,110149134	0,781	0,16	1,07846E-09	2,158679609	0,463246142	4,88125
MYLK	6,55424E-26	1,108927717	0,531	0,016	1,38301E-21	2,156852796	0,463638502	33,1875
RPL36A	6,68557E-08	1,10032706	0,969	0,664	0,001410722	2,144032924	0,466410748	1,459337349
MYDGF	9,06985E-09	1,09966004	0,75	0,266	0,000191383	2,143041875	0,46662644	2,819548872
TMED9	1,2614E-11	1,096682082	0,938	0,309	2,66167E-07	2,138622849	0,467590628	3,035598706
DNPH1	1,52952E-18	1,095102973	0,719	0,086	3,22744E-14	2,136283288	0,468102712	8,360465116

Supplementary Table 1

Differentially expressed genes_adjacent-skin-XL_ comparing SC-Keloid vs. (SC-Myel, SC-Nonmyel)								
**gene**	**p_val**	**avg_log2FC**	**pct.1**	**pct.2**	**p_val_adj**	**Foldchange_UP**	**Foldchange_DOWN**	**Ratio_pct1_pct2**
THBS2	1,0872E-15	1,086742506	0,625	0,082	2,29411E-11	2,123939255	0,470823258	7,62195122
PLP2	4,38818E-09	1,084524803	0,625	0,168	9,2595E-05	2,120676856	0,471547561	3,720238095
PPFIBP1	8,31379E-12	1,083163458	0,938	0,277	1,75429E-07	2,118676703	0,471992729	3,386281588
PGK1	1,04017E-12	1,082018327	0,75	0,16	2,19486E-08	2,116995682	0,47236752	4,6875
PHACTR1	2,68058E-16	1,077736212	0,469	0,035	5,6563E-12	2,110721467	0,473771654	13,4
P4HA2	1,39414E-11	1,077715147	0,781	0,195	2,94178E-07	2,110690649	0,473778571	4,005128205
PDLIM1	1,96597E-19	1,070230436	0,625	0,059	4,1484E-15	2,099768728	0,476242925	10,59322034
GSTP1	1,38982E-09	1,068126844	0,938	0,496	2,93266E-05	2,096709289	0,47693784	1,891129032
SSR4	7,11486E-10	1,068049488	0,906	0,352	1,50131E-05	2,096596868	0,476963414	2,573863636
RPS2	2,90465E-10	1,067111949	1	0,848	6,12911E-06	2,095234832	0,477273471	1,179245283
RPS6	1,13808E-09	1,065610537	1	0,781	2,40147E-05	2,093055456	0,477770428	1,280409731
HLA-A	1,06476E-07	1,062949738	0,969	0,68	0,002246741	2,089198739	0,478652405	1,425
FXYD5	2,26298E-15	1,060426231	0,719	0,113	4,77512E-11	2,085547586	0,479490378	6,362831858
TIMP1	3,31691E-09	1,059463825	0,969	0,59	6,999E-05	2,084156805	0,479810347	1,642372881
ELOB	1,77312E-10	1,059235007	1	0,445	3,74146E-06	2,083826274	0,479886453	2,247191011
ANAPC11	5,91328E-11	1,052860937	0,719	0,184	1,24776E-06	2,074639887	0,482011363	3,907608696
ANXA5	1,27555E-08	1,050376544	0,938	0,68	0,000269154	2,071070327	0,482842126	1,379411765
PDIA6	4,716E-11	1,045884708	0,906	0,277	9,95124E-07	2,06463207	0,484347799	3,270758123
BRK1	9,45657E-12	1,045821993	0,906	0,281	1,99543E-07	2,064542322	0,484368855	3,224199288
SSR2	2,63756E-09	1,042961473	0,906	0,367	5,56551E-05	2,060452882	0,485330196	2,46866485
FKBP3	1,18823E-19	1,042576585	0,719	0,082	2,50729E-15	2,05990326	0,485459691	8,768292683
NAA10	6,93317E-13	1,041868847	0,656	0,125	1,46297E-08	2,058892987	0,4856979	5,248
COL20A1	1,43347E-08	1,041313141	0,156	0,004	0,000302477	2,058100084	0,48588502	39
LBH	1,94499E-16	1,034403457	0,5	0,043	4,10411E-12	2,048266528	0,488217713	11,62790698
TMEM219	8,02601E-14	1,033168232	0,812	0,16	1,69357E-09	2,046513568	0,4886359	5,075
C1orf122	9,92262E-15	1,03228717	0,812	0,152	2,09377E-10	2,045264132	0,488934404	5,342105263
C1QL1	6,6483E-12	1,032275662	0,375	0,035	1,40286E-07	2,045247818	0,488938304	10,71428571
NDUFS5	1,42179E-09	1,025800982	0,938	0,426	3,00013E-05	2,036089504	0,491137545	2,201877934
UQCRH	5,68675E-11	1,025685597	0,906	0,336	1,19996E-06	2,035926666	0,491176827	2,696428571
CALU	1,16403E-07	1,020522435	0,844	0,387	0,002456214	2,028653451	0,492937815	2,180878553

Supplementary Table 1

Differentially expressed genes_adjacent-skin-XL_ comparing SC-Keloid vs. (SC-Myel, SC-Nonmyel)								
**gene**	**p_val**	**avg_log2FC**	**pct.1**	**pct.2**	**p_val_adj**	**Foldchange_UP**	**Foldchange_DOWN**	**Ratio_pct1_pct2**
SPCS1	6,74952E-10	1,019528464	0,812	0,262	1,42422E-05	2,027256255	0,493277551	3,099236641
PDIA3	1,33703E-07	1,018886679	0,844	0,383	0,002821263	2,026354628	0,493497035	2,203655352
PGAM1	1,29144E-11	1,014986062	0,719	0,16	2,72507E-07	2,020883369	0,494833109	4,49375
PSMB6	2,99465E-12	1,011743811	0,844	0,215	6,31902E-08	2,016346822	0,495946426	3,925581395
RPL26	3,75956E-10	1,011076536	1	0,82	7,93305E-06	2,015414438	0,496175864	1,219512195
NME1	2,19773E-12	1,009084832	0,625	0,113	4,63742E-08	2,012633989	0,49686133	5,530973451
NDUFB2	1,76887E-10	1,008143434	0,875	0,301	3,7325E-06	2,011321119	0,497185651	2,906976744
PDGFC	4,35563E-22	1,007112679	0,562	0,031	9,19081E-18	2,009884614	0,497541	18,12903226
ATP5PD	2,79358E-11	1,005349274	0,844	0,23	5,89473E-07	2,007429434	0,498149516	3,669565217
RNF7	3,26236E-08	0,999590193	0,562	0,141	0,00068839	1,999431968	0,500142048	3,985815603
ATP5MG	2,36403E-07	0,997404038	0,938	0,434	0,004988329	1,996404468	0,500900502	2,161290323
NIBAN2	1,96161E-09	0,991822847	0,875	0,273	4,1392E-05	1,988696125	0,502842032	3,205128205
ATP5F1B	1,58937E-09	0,991664141	0,938	0,367	3,35373E-05	1,988477366	0,502897351	2,555858311
ATP5MF	4,96727E-10	0,990883296	0,875	0,309	1,04814E-05	1,987401413	0,503169613	2,83171521
SDCCAG8	1,95389E-17	0,988476883	0,656	0,074	4,1229E-13	1,984089194	0,504009599	8,864864865
MYH9	6,07653E-10	0,987282781	0,656	0,16	1,28221E-05	1,982447666	0,504426935	4,1
CD82	5,04864E-12	0,987109621	0,656	0,117	1,06531E-07	1,982209737	0,504487483	5,606837607
KIRREL3	5,16264E-17	0,984803762	0,562	0,055	1,08937E-12	1,979044102	0,50529445	10,21818182
ABHD2	1,07359E-21	0,981873627	0,875	0,105	2,26537E-17	1,975028712	0,506321753	8,333333333
MZT2B	6,72828E-09	0,980410762	0,906	0,359	0,000141973	1,973027087	0,506835414	2,52367688
GIPC1	1,68353E-21	0,978565136	0,656	0,055	3,55243E-17	1,970504625	0,507484219	11,92727273
LOXL3	9,06058E-26	0,976121211	0,781	0,062	1,91187E-21	1,967169417	0,508344625	12,59677419
RNASEH2C	4,10837E-13	0,973581184	0,625	0,098	8,66906E-09	1,963709041	0,509240411	6,37755102
SLC9A3R2	6,91091E-14	0,97194427	0,75	0,145	1,45827E-09	1,961482236	0,509818535	5,172413793
SERPINH1	2,07858E-09	0,970359674	0,875	0,293	4,38601E-05	1,959329009	0,510378806	2,986348123
PLOD3	6,28472E-18	0,969548147	0,844	0,133	1,32614E-13	1,958227181	0,510665979	6,345864662
CAP1	1,0635E-08	0,969390486	0,875	0,352	0,000224409	1,958013193	0,510721788	2,485795455
HLA-C	2,30484E-07	0,96732598	0,938	0,742	0,004863438	1,955213268	0,511453158	1,264150943
SHISA4	4,41456E-11	0,962072589	0,656	0,137	9,31517E-07	1,948106553	0,513318945	4,788321168
H3-3A	3,93923E-10	0,960904952	1	0,812	8,31218E-06	1,946530502	0,513734565	1,231527094

Supplementary Table 1

Differentially expressed genes_adjacent-skin-XL_ comparing SC-Keloid vs. (SC-Myel, SC-Nonmyel)								
**gene**	**p_val**	**avg_log2FC**	**pct.1**	**pct.2**	**p_val_adj**	**Foldchange_UP**	**Foldchange_DOWN**	**Ratio_pct1_pct2**
TPST2	9,76652E-26	0,960626813	0,688	0,043	2,06083E-21	1,946155264	0,513833618	16
RPL17	1,49025E-07	0,959032349	1	0,742	0,003144586	1,944005565	0,51440182	1,347708895
TENM3	7,23932E-15	0,957547701	0,812	0,152	1,52757E-10	1,942006057	0,514931453	5,342105263
ADAMTSL1	6,15244E-33	0,957147971	0,594	0,008	1,29823E-28	1,941468057	0,515074145	74,25
COPS9	1,16903E-09	0,956487686	0,812	0,254	2,46676E-05	1,9405797	0,515309935	3,196850394
ATP5MJ	4,39108E-11	0,95420782	0,844	0,211	9,26562E-07	1,937515458	0,516124915	4
RPS18	1,76239E-09	0,952826188	1	0,91	3,71881E-05	1,935660837	0,516619431	1,098901099
REEP3	4,73057E-09	0,947504727	0,75	0,211	9,98197E-05	1,928534195	0,518528529	3,55450237
TXNL4A	4,19939E-13	0,946193014	0,688	0,129	8,86113E-09	1,926781549	0,519000195	5,333333333
EEF1G	2,03788E-08	0,944234447	0,969	0,551	0,000430012	1,924167573	0,519705255	1,75862069
AURKAIP1	9,89489E-13	0,944064378	0,812	0,191	2,08792E-08	1,923940761	0,519766523	4,251308901
ATP5F1C	3,44078E-10	0,942522015	0,844	0,242	7,26039E-06	1,921885004	0,520322495	3,487603306
EMC10	1,02213E-10	0,93961837	0,75	0,18	2,1568E-06	1,918020806	0,521370778	4,166666667
MCRIP1	7,42098E-11	0,939456263	0,781	0,211	1,5659E-06	1,917805301	0,521429365	3,701421801
AP2S1	2,54903E-11	0,938475567	0,594	0,117	5,37871E-07	1,916502084	0,521783936	5,076923077
SCARA3	5,1155E-14	0,937709312	0,75	0,133	1,07942E-09	1,915484447	0,522061143	5,639097744
MEF2C	2,27637E-13	0,933092425	0,719	0,133	4,80336E-09	1,909364346	0,52373451	5,406015038
PERP	1,73599E-15	0,932278512	0,656	0,09	3,66312E-11	1,90828746	0,524030064	7,288888889
NAA38	2,39382E-12	0,932158492	0,75	0,156	5,05121E-08	1,908128713	0,524073661	4,807692308
RPL28	5,24899E-10	0,932149671	1	0,863	1,10759E-05	1,908117046	0,524076865	1,158748552
QPCT	7,57111E-23	0,929673276	0,531	0,023	1,59758E-18	1,904844561	0,524977219	23,08695652
IFI27L2	3,69607E-11	0,926031853	0,875	0,238	7,79907E-07	1,900042715	0,526303957	3,676470588
GPC1	7,20353E-07	0,92573403	0,812	0,379	0,01520016	1,899650521	0,526412616	2,142480211
ALDH1A3	7,07559E-22	0,924543314	0,562	0,031	1,49302E-17	1,898083308	0,526847265	18,12903226
UQCR11	5,91166E-09	0,923640946	0,906	0,34	0,000124742	1,896896477	0,527176898	2,664705882
NOP10	3,03941E-13	0,923518911	0,719	0,133	6,41345E-09	1,896736028	0,527221493	5,406015038
RPL10A	2,73649E-09	0,923279384	1	0,754	5,77427E-05	1,896421144	0,527309033	1,326259947
CHN1	2,81721E-22	0,920740308	0,5	0,02	5,94459E-18	1,893086467	0,52823789	25
ATP5F1D	9,5967E-08	0,918658133	0,781	0,293	0,002025	1,890356234	0,529000821	2,66552901
NDUFA13	3,93899E-08	0,914713132	0,938	0,43	0,000831167	1,885194179	0,530449336	2,181395349

Supplementary Table 1

Differentially expressed genes_adjacent-skin-XL_ comparing SC-Keloid vs. (SC-Myel, SC-Nonmyel)								
**gene**	**p_val**	**avg_log2FC**	**pct.1**	**pct.2**	**p_val_adj**	**Foldchange_UP**	**Foldchange_DOWN**	**Ratio_pct1_pct2**
RABAC1	2,21101E-07	0,912935449	0,906	0,367	0,004665443	1,882872682	0,531103356	2,46866485
FAM32A	4,40666E-15	0,912216782	0,438	0,035	9,2985E-11	1,881934977	0,531367987	12,51428571
CYBA	2,63593E-10	0,912128823	0,938	0,301	5,56207E-06	1,881820242	0,531400385	3,11627907
PRDX5	4,48497E-08	0,908187168	0,844	0,293	0,000946373	1,876685849	0,532854234	2,880546075
HMGN1	1,7154E-08	0,907340828	0,875	0,309	0,000361967	1,875585236	0,533166918	2,83171521
CXCL12	8,12453E-22	0,906715814	0,656	0,051	1,71436E-17	1,874772859	0,53339795	12,8627451
AP2M1	1,00251E-08	0,906687846	0,844	0,277	0,00021154	1,874736515	0,533408291	3,046931408
TIMM8B	9,57393E-17	0,906584935	0,812	0,125	2,0202E-12	1,87460279	0,533446341	6,496
HOXB2	3,90621E-19	0,9051739	0,562	0,043	8,2425E-15	1,872770222	0,533968336	13,06976744
ZNHIT1	7,5306E-10	0,905017987	0,75	0,203	1,58903E-05	1,872567841	0,534026046	3,694581281
VAMP5	1,23611E-10	0,902965636	0,812	0,211	2,60831E-06	1,869905856	0,534786282	3,848341232
NDUFAF8	6,85646E-13	0,901792367	0,625	0,102	1,44678E-08	1,868385776	0,535221373	6,12745098
COX7A2	1,54088E-06	0,901581971	0,875	0,422	0,032514155	1,86811332	0,535299433	2,073459716
ITPA	1,33843E-21	0,900021706	0,656	0,055	2,82423E-17	1,866094059	0,535878669	11,92727273
MAD2L2	5,2274E-17	0,894617446	0,688	0,086	1,10303E-12	1,859116845	0,537889807	8
STX8	9,01486E-18	0,890436671	0,594	0,059	1,90223E-13	1,853737123	0,539450814	10,06779661
PSMB1	1,70009E-07	0,888787483	0,75	0,301	0,003587349	1,851619271	0,540067829	2,491694352
NT5E	2,65901E-27	0,888560329	0,438	0	5,61077E-23	1,851327754	0,54015287	#DIV/0!
PARVB	4,97512E-21	0,887809025	0,562	0,035	1,0498E-16	1,850363901	0,540434236	16,05714286
RPLP0	1,14478E-06	0,885849177	0,938	0,633	0,024155921	1,847851956	0,541168894	1,481832543
COX6B1	2,71446E-08	0,883505608	0,906	0,387	0,000572778	1,844852671	0,542048704	2,341085271
BBLN	2,29481E-11	0,883246192	0,688	0,152	4,84228E-07	1,844520972	0,542146181	4,526315789
SLIRP	1,88539E-09	0,882373412	0,719	0,188	3,97837E-05	1,843405438	0,54247426	3,824468085
LIMS1	1,47941E-08	0,882010639	0,781	0,254	0,00031217	1,842941963	0,542610684	3,07480315
RPL12	1,53466E-09	0,881782011	1	0,809	3,23828E-05	1,84264993	0,54269668	1,236093943
RTRAF	4,26359E-09	0,881450226	0,844	0,258	8,9966E-05	1,842226214	0,542821502	3,271317829
POMP	6,09064E-09	0,872884177	0,812	0,277	0,000128519	1,831320345	0,546054109	2,931407942
LTBP1	2,65356E-23	0,870635447	0,438	0,008	5,59927E-19	1,828468087	0,546905908	54,75
ARF3	5,31765E-12	0,868512674	0,531	0,074	1,12208E-07	1,825779668	0,547711215	7,175675676
ARF5	2,6113E-08	0,867905204	0,812	0,254	0,00055101	1,825011056	0,547941886	3,196850394

Supplementary Table 1

Differentially expressed genes_adjacent-skin-XL_ comparing SC-Keloid vs. (SC-Myel, SC-Nonmyel)								
**gene**	**p_val**	**avg_log2FC**	**pct.1**	**pct.2**	**p_val_adj**	**Foldchange_UP**	**Foldchange_DOWN**	**Ratio_pct1_pct2**
ACTG2	2,86276E-12	0,866005285	0,188	0	6,04072E-08	1,822609238	0,548663959	#DIV/0!
TGFB1I1	5,85437E-11	0,865722459	0,75	0,18	1,23533E-06	1,822251968	0,54877153	4,166666667
TAX1BP3	4,71243E-08	0,86475768	0,875	0,395	0,000994371	1,821033775	0,549138634	2,215189873
BSG	1,69711E-07	0,863840237	0,875	0,395	0,003581063	1,819876105	0,549487956	2,215189873
LIMA1	9,00804E-14	0,862454862	0,812	0,16	1,90079E-09	1,818129373	0,550015865	5,075
LSM4	4,73331E-10	0,862388329	0,531	0,098	9,98776E-06	1,818045529	0,550041231	5,418367347
KCNN4	8,55163E-26	0,862033371	0,562	0,02	1,80448E-21	1,817598275	0,550176578	28,1
COL4A2	6,93438E-07	0,861684464	1	0,715	0,014632227	1,817158753	0,550309651	1,398601399
LIF	1,29589E-15	0,861510607	0,375	0,02	2,73446E-11	1,816939784	0,550375972	18,75
PDCD5	9,21258E-10	0,861023766	0,75	0,199	1,94395E-05	1,816326756	0,550561729	3,768844221
PSMB3	4,0075E-09	0,860117422	0,719	0,199	8,45623E-05	1,815186044	0,550907717	3,613065327
DPM2	3,05311E-18	0,85983266	0,594	0,055	6,44236E-14	1,814827794	0,551016467	10,8
ANP32B	1,37162E-09	0,859019776	0,844	0,254	2,89426E-05	1,813805521	0,551327024	3,322834646
COPE	1,09697E-07	0,858684263	0,812	0,32	0,002314707	1,813383751	0,551455256	2,5375
MXRA5	5,0668E-20	0,855983818	0,438	0,016	1,06914E-15	1,809992624	0,552488439	27,375
CD63	1,36232E-09	0,854460859	1	0,824	2,87463E-05	1,808082941	0,553071973	1,213592233
COX6A1	1,54365E-07	0,852210274	0,906	0,387	0,003257246	1,805264555	0,553935432	2,341085271
COL4A1	1,07018E-06	0,852093078	0,969	0,703	0,022581915	1,805117912	0,553980432	1,378378378
RCN3	4,45799E-16	0,851880322	0,625	0,074	9,40679E-12	1,804851728	0,554062134	8,445945946
SH3GLB1	4,39761E-09	0,851805772	0,719	0,207	9,27939E-05	1,804758466	0,554090765	3,473429952
EMILIN1	1,37952E-16	0,848592995	0,625	0,07	2,91092E-12	1,800743872	0,55532606	8,928571429
ACTR2	5,17991E-12	0,840570454	0,844	0,195	1,09301E-07	1,790758084	0,55842272	4,328205128
CD276	2,40751E-20	0,839801362	0,719	0,074	5,08009E-16	1,789803695	0,558720492	9,716216216
TUBB	0,000120884	0,838684476	0,781	0,418	1	1,788418626	0,559153201	1,868421053
SELENOH	2,45519E-11	0,838527925	0,75	0,16	5,1807E-07	1,788224569	0,55921388	4,6875
COL5A1	6,76766E-10	0,83801236	0,781	0,207	1,42804E-05	1,787585639	0,559413758	3,77294686
PSMD8	1,03692E-07	0,837138502	0,688	0,238	0,002188015	1,786503205	0,559752704	2,890756303
LOXL1	4,29805E-27	0,836488049	0,531	0,012	9,06931E-23	1,785697924	0,560005131	44,25
ARPC5	6,3904E-05	0,836140219	0,781	0,422	1	1,785267449	0,560140163	1,8507109
MIR4435-2HG	9,46903E-25	0,834567242	0,594	0,027	1,99806E-20	1,783322024	0,56075122	22

Supplementary Table 1

Differentially expressed genes_adjacent-skin-XL_ comparing SC-Keloid vs. (SC-Myel, SC-Nonmyel)								
**gene**	**p_val**	**avg_log2FC**	**pct.1**	**pct.2**	**p_val_adj**	**Foldchange_UP**	**Foldchange_DOWN**	**Ratio_pct1_pct2**
DDOST	5,33167E-11	0,834093386	0,625	0,125	1,12504E-06	1,782736385	0,56093543	5
CLTA	7,37279E-09	0,832180286	0,938	0,328	0,000155573	1,780373936	0,561679757	2,859756098
SNRPD2	1,10324E-10	0,831799453	0,844	0,215	2,32794E-06	1,779904028	0,561828045	3,925581395
CLEC11A	2,58463E-12	0,830211734	0,594	0,098	5,45382E-08	1,777946279	0,56244669	6,06122449
RACK1	7,2383E-05	0,828908401	0,906	0,676	1	1,776340806	0,562955035	1,340236686
MGST3	7,88954E-09	0,828259104	0,938	0,344	0,000166477	1,775541528	0,563208455	2,726744186
PLPP4	3,124E-29	0,827941651	0,469	0	6,59194E-25	1,775150878	0,563332398	#DIV/0!
DCTN2	3,91423E-12	0,827383355	0,781	0,168	8,25941E-08	1,774464061	0,563550439	4,648809524
CBX3	6,73901E-11	0,826157904	0,844	0,211	1,422E-06	1,772957439	0,564029332	4
ISG15	9,05705E-07	0,825750218	0,656	0,184	0,019111275	1,772456497	0,564188741	3,565217391
RPL3	4,1738E-07	0,823730066	0,969	0,883	0,008807141	1,769976329	0,564979307	1,097395243
RPS24	2,53779E-08	0,822636362	1	0,852	0,000535499	1,768635022	0,565407779	1,17370892
SUMO2	1,75872E-06	0,822564439	0,906	0,531	0,037110766	1,768546852	0,565435967	1,706214689
SLC25A3	9,84327E-06	0,822497303	0,844	0,438	0,207702845	1,768464554	0,565462281	1,926940639
MRPL51	2,3518E-09	0,821345969	0,781	0,215	4,96253E-05	1,767053805	0,565913725	3,63255814
DYNLT2B	1,4711E-22	0,820927624	0,531	0,023	3,10416E-18	1,766541478	0,566077849	23,08695652
LAMB2	4,71332E-09	0,82057001	0,75	0,199	9,94557E-05	1,766103644	0,566218185	3,768844221
CSTB	1,50861E-08	0,818715535	0,812	0,242	0,000318332	1,76383491	0,566946484	3,355371901
RPLP2	5,73248E-09	0,818572413	1	0,789	0,000120961	1,763659938	0,56700273	1,267427123
SEC11A	1,11207E-08	0,818386115	0,906	0,32	0,000234657	1,763432208	0,567075953	2,83125
RPS5	4,08024E-07	0,81795507	1	0,656	0,008609712	1,762905412	0,567245408	1,524390244
CBR1	9,50275E-11	0,817624946	0,812	0,207	2,00517E-06	1,762502062	0,567375223	3,922705314
PSMB2	1,00758E-12	0,817313315	0,75	0,152	2,1261E-08	1,762121393	0,567497792	4,934210526
RPL29	1,93016E-06	0,816433554	0,969	0,828	0,040728337	1,761047172	0,56784396	1,170289855
DYNC2I2	4,3842E-11	0,816117392	0,406	0,047	9,25109E-07	1,760661287	0,567968415	8,638297872
TMEM160	2,24956E-14	0,815827144	0,562	0,07	4,7468E-10	1,760307104	0,568082693	8,028571429
SNU13	6,28775E-09	0,815380734	0,844	0,262	0,000132678	1,7597625	0,568258501	3,221374046
FIBIN	4,17431E-10	0,814438171	0,656	0,141	8,80821E-06	1,758613161	0,568629885	4,65248227
PPIA	5,11582E-08	0,813533032	1	0,762	0,001079488	1,757510163	0,568986752	1,312335958
RPLP1	4,86137E-08	0,812024875	1	0,961	0,001025797	1,755673866	0,569581868	1,040582726

Supplementary Table 1

Differentially expressed genes_adjacent-skin-XL_ comparing SC-Keloid vs. (SC-Myel, SC-Nonmyel)								
**gene**	**p_val**	**avg_log2FC**	**pct.1**	**pct.2**	**p_val_adj**	**Foldchange_UP**	**Foldchange_DOWN**	**Ratio_pct1_pct2**
CXXC5	5,59181E-09	0,811283925	0,875	0,305	0,000117993	1,754772406	0,569874473	2,868852459
MRPL57	4,91302E-13	0,809323949	0,656	0,109	1,0367E-08	1,752390075	0,570649203	6,018348624
CIB1	1,70442E-10	0,806862061	0,75	0,18	3,59649E-06	1,749402258	0,571623819	4,166666667
BCL7C	2,50129E-08	0,805703095	0,594	0,152	0,000527798	1,747997468	0,572083208	3,907894737
SRPRB	4,7158E-17	0,805233224	0,469	0,031	9,9508E-13	1,747428255	0,572269561	15,12903226
MICOS13	2,32785E-10	0,80475637	0,75	0,18	4,91199E-06	1,746850774	0,572458744	4,166666667
SSBP4	2,82891E-14	0,803042321	0,625	0,09	5,96929E-10	1,744776593	0,57313928	6,944444444
PFDN5	7,78595E-06	0,802399214	0,938	0,523	0,164291329	1,743999001	0,573394824	1,793499044
ANXA6	1,12492E-15	0,800113146	0,719	0,105	2,37369E-11	1,74123768	0,574304135	6,847619048
GNAI2	2,08509E-07	0,799637095	0,906	0,375	0,004399757	1,740663214	0,574493671	2,416
UAP1	1,37531E-15	0,799310036	0,656	0,09	2,90203E-11	1,74026865	0,574623924	7,288888889
ACTN4	9,16559E-09	0,796310101	0,875	0,277	0,000193403	1,736653701	0,575820038	3,158844765
NEDD8	6,87122E-07	0,794757142	0,844	0,305	0,014498967	1,734785323	0,576440201	2,767213115
RPS19	1,20793E-07	0,793962304	1	0,785	0,002548848	1,733829823	0,576757872	1,27388535
TRUB2	5,03869E-18	0,793912367	0,469	0,027	1,06321E-13	1,733769811	0,576777836	17,37037037
GABARAP	9,28202E-05	0,790572046	0,906	0,539	1	1,729760198	0,578114817	1,680890538
MRPL52	1,51607E-11	0,78980929	0,719	0,145	3,19906E-07	1,728845911	0,578420548	4,95862069
NME4	1,78452E-12	0,789693474	0,562	0,082	3,76552E-08	1,72870713	0,578466984	6,853658537
EIF4E2	3,40648E-14	0,789599309	0,531	0,062	7,18801E-10	1,7285943	0,578504742	8,564516129
RSU1	3,46348E-12	0,788781348	0,625	0,105	7,30829E-08	1,727614521	0,578832829	5,952380952
BCAP31	9,23764E-11	0,787490117	0,719	0,156	1,94923E-06	1,726068975	0,579351124	4,608974359
ATP5MC2	1,41333E-07	0,787297111	0,938	0,43	0,002982267	1,725838074	0,579428635	2,181395349
FAM162A	1,42121E-15	0,782941317	0,75	0,113	2,99889E-11	1,720635271	0,581180693	6,637168142
MRPL47	2,92626E-13	0,782223209	0,594	0,086	6,1747E-09	1,71977903	0,581470051	6,906976744
MTLN	4,88998E-20	0,781071686	0,688	0,066	1,03184E-15	1,718406893	0,581934351	10,42424242
NDUFA1	2,80126E-07	0,779671036	0,875	0,359	0,005910941	1,716739376	0,5824996	2,437325905
YBX1	4,6484E-06	0,77850003	0,938	0,531	0,098085891	1,7153465	0,582972595	1,766478343
RPL13	8,88476E-09	0,778131842	1	0,902	0,000187477	1,714908785	0,583121393	1,10864745
RPL19	5,39805E-08	0,777891399	1	0,852	0,001139043	1,714622998	0,583218586	1,17370892
PSMC5	1,89234E-09	0,776492334	0,656	0,16	3,99303E-05	1,712961034	0,583784441	4,1

Supplementary Table 1

Differentially expressed genes_adjacent-skin-XL_ comparing SC-Keloid vs. (SC-Myel, SC-Nonmyel)								
**gene**	**p_val**	**avg_log2FC**	**pct.1**	**pct.2**	**p_val_adj**	**Foldchange_UP**	**Foldchange_DOWN**	**Ratio_pct1_pct2**
CMSS1	2,28279E-13	0,776315614	0,594	0,086	4,81691E-09	1,712751221	0,583855955	6,906976744
TPI1	5,31689E-08	0,774858014	0,906	0,371	0,001121917	1,711021649	0,584446141	2,442048518
RPL6	2,82298E-06	0,774507197	0,969	0,707	0,059567639	1,710605634	0,584588277	1,370579915
COX7A2L	3,61635E-13	0,773681852	0,812	0,16	7,63085E-09	1,709627301	0,584922807	5,075
FADS1	2,94022E-06	0,773620072	0,406	0,09	0,062041601	1,709554093	0,584947855	4,511111111
NDUFAB1	1,98304E-09	0,772481416	0,781	0,211	4,18441E-05	1,708205348	0,585409711	3,701421801
A1BG	1,27969E-13	0,77199641	0,812	0,156	2,70027E-09	1,707631179	0,585606548	5,205128205
PLAT	1,26672E-06	0,771860936	0,969	0,441	0,026728981	1,707470834	0,585661541	2,197278912
ERP29	4,2377E-13	0,771477353	0,719	0,129	8,94197E-09	1,707016914	0,585817277	5,573643411
HOXC9	5,38738E-27	0,771422595	0,469	0,004	1,13679E-22	1,706952124	0,585839512	117,25
TMED3	1,32268E-16	0,770023435	0,562	0,055	2,79098E-12	1,705297484	0,586407949	10,21818182
SERPINE1	5,24077E-18	0,768869511	0,562	0,047	1,10585E-13	1,703934065	0,586877169	11,95744681
POLR2L	3,07893E-08	0,766947133	0,969	0,43	0,000649686	1,701665102	0,587659698	2,253488372
APMAP	5,49842E-12	0,765883017	0,469	0,059	1,16022E-07	1,700410434	0,58809331	7,949152542
PHPT1	4,46774E-07	0,764806003	0,781	0,301	0,009427372	1,699141502	0,588532502	2,594684385
CHMP3	5,03293E-13	0,763219555	0,781	0,152	1,062E-08	1,697274082	0,589180033	5,138157895
GMFG	2,2608E-10	0,762612976	0,188	0,004	4,77051E-06	1,696560615	0,589427805	47
HINT1	3,7134E-07	0,761344787	0,906	0,434	0,007835648	1,695069923	0,589946165	2,087557604
TMCO1	1,45783E-08	0,761229005	0,781	0,227	0,000307617	1,694933892	0,589993512	3,440528634
FHDC1	3,89044E-19	0,760047228	0,531	0,035	8,20922E-15	1,693546064	0,590477	15,17142857
ST3GAL4	2,17734E-12	0,759934762	0,656	0,117	4,59441E-08	1,693414047	0,590523033	5,606837607
EDF1	2,61283E-06	0,758469285	0,906	0,445	0,055133285	1,691694766	0,591123186	2,035955056
EVA1B	4,79626E-16	0,758146728	0,656	0,082	1,01206E-11	1,69131658	0,591255364	8
C4orf48	7,49712E-13	0,757854306	0,688	0,125	1,58197E-08	1,690973799	0,591375219	5,504
GNAS	2,36606E-05	0,757373495	0,969	0,727	0,499262054	1,690410338	0,591572341	1,332874828
NCS1	3,74251E-14	0,757112795	0,531	0,062	7,89707E-10	1,690104903	0,591679249	8,564516129
PYGB	1,44507E-19	0,755780635	0,688	0,07	3,04923E-15	1,688545009	0,592225848	9,828571429
CD99	5,36756E-06	0,754261912	0,906	0,5	0,113260808	1,686768414	0,592849612	1,812
RPL4	9,49063E-06	0,753202094	0,875	0,465	0,200261764	1,685529753	0,593285285	1,88172043
ATP6V0B	8,27349E-07	0,752044622	0,812	0,277	0,017457891	1,684177998	0,593761468	2,931407942

Supplementary Table 1

Differentially expressed genes_adjacent-skin-XL_ comparing SC-Keloid vs. (SC-Myel, SC-Nonmyel)								
**gene**	**p_val**	**avg_log2FC**	**pct.1**	**pct.2**	**p_val_adj**	**Foldchange_UP**	**Foldchange_DOWN**	**Ratio_pct1_pct2**
PPDPF	3,06116E-06	0,751298041	1	0,668	0,064593486	1,683306677	0,594068813	1,497005988
SSB	6,28653E-11	0,749249878	0,875	0,223	1,32652E-06	1,680918618	0,594912799	3,923766816
CMTM3	1,3891E-11	0,749144129	0,719	0,145	2,93114E-07	1,680795411	0,594956408	4,95862069
HLA-B	0,000143643	0,748809308	0,938	0,734	1	1,680405378	0,595094501	1,277929155
ABLIM3	1,22444E-14	0,748162822	0,438	0,035	2,5837E-10	1,67965254	0,595361229	12,51428571
OSTC	3,85126E-10	0,746809471	0,719	0,168	8,12654E-06	1,678077645	0,595919982	4,279761905
CTSA	1,70757E-14	0,746121782	0,625	0,086	3,60314E-10	1,677277947	0,596204107	7,26744186
UBA52	1,12313E-06	0,744137687	1	0,656	0,023699228	1,674972822	0,597024613	1,524390244
SMAP2	2,89798E-16	0,744007147	0,469	0,035	6,11503E-12	1,674821272	0,597078636	13,4
EEF1B2	1,40984E-06	0,743930347	0,938	0,465	0,02974893	1,674732118	0,597110421	2,017204301
PSMB10	7,49278E-16	0,743325172	0,562	0,059	1,58105E-11	1,674029755	0,597360947	9,525423729
RPL7A	7,20574E-06	0,743045105	1	0,777	0,152048307	1,673704812	0,597476922	1,287001287
OCIAD2	2,00705E-19	0,742701091	0,344	0,004	4,23507E-15	1,67330576	0,597619409	86
AEBP1	4,55379E-08	0,742163539	0,906	0,301	0,000960896	1,672682399	0,597842125	3,009966777
TAF10	8,40653E-11	0,741976638	0,531	0,09	1,77386E-06	1,672465717	0,597919581	5,9
LINC01116	3,76123E-10	0,740828389	0,469	0,074	7,93658E-06	1,671135121	0,598395658	6,337837838
MPG	2,15641E-13	0,738380174	0,844	0,168	4,55025E-09	1,668301654	0,599411981	5,023809524
TMEM50A	3,50301E-08	0,736873614	0,844	0,281	0,00073917	1,66656041	0,600038255	3,003558719
NRBF2	2,78342E-17	0,735473492	0,656	0,074	5,8733E-13	1,664943813	0,600620869	8,864864865
COPS6	6,09801E-08	0,734146909	0,656	0,176	0,001286741	1,663413573	0,601173404	3,727272727
MTPN	6,21629E-12	0,734129086	0,688	0,133	1,3117E-07	1,663393023	0,601180831	5,172932331
HEXB	1,63045E-15	0,732449034	0,625	0,078	3,44041E-11	1,661457091	0,601881328	8,012820513
UQCRQ	2,75692E-06	0,731653874	0,812	0,316	0,058173825	1,66054161	0,602213154	2,569620253
TAF12	1,52868E-07	0,728486413	0,375	0,066	0,003225666	1,656899862	0,603536775	5,681818182
MEAF6	2,37955E-09	0,728434613	0,719	0,176	5,02108E-05	1,656840372	0,603558446	4,085227273
NARS1	1,17417E-11	0,727915549	0,688	0,133	2,47762E-07	1,656244368	0,603775638	5,172932331
RPL10	9,12855E-08	0,727839023	1	0,926	0,001926215	1,656156517	0,603807665	1,079913607
COPZ2	3,60438E-12	0,727771517	0,594	0,098	7,60561E-08	1,656079025	0,603835919	6,06122449
POSTN	2,24722E-08	0,727348302	0,281	0,031	0,000474185	1,655593285	0,604013081	9,064516129
MICOS10	4,38759E-10	0,727344241	0,812	0,215	9,25825E-06	1,655588624	0,604014781	3,776744186

Supplementary Table 1

Differentially expressed genes_adjacent-skin-XL_ comparing SC-Keloid vs. (SC-Myel, SC-Nonmyel)								
**gene**	**p_val**	**avg_log2FC**	**pct.1**	**pct.2**	**p_val_adj**	**Foldchange_UP**	**Foldchange_DOWN**	**Ratio_pct1_pct2**
SEPTIN7	6,39873E-06	0,726691614	0,906	0,449	0,135019581	1,654839861	0,604288078	2,017817372
PFDN1	1,28589E-10	0,726124043	0,812	0,191	2,71337E-06	1,654188958	0,604525859	4,251308901
LLPH	1,13763E-17	0,724104461	0,5	0,035	2,4005E-13	1,651874933	0,605372707	14,28571429
SMARCB1	7,26066E-13	0,723923144	0,531	0,07	1,53207E-08	1,651667338	0,605448795	7,585714286
FABP7	6,94858E-18	0,722365341	0,375	0,012	1,46622E-13	1,649884853	0,606102903	31,25
TRAPPC5	7,32636E-14	0,716943046	0,75	0,137	1,54593E-09	1,643695485	0,608385196	5,474452555
RPL22	4,16124E-06	0,716391578	1	0,73	0,087806429	1,643067304	0,608617795	1,369863014
APLNR	2,86276E-12	0,716318085	0,188	0	6,04072E-08	1,642983607	0,6086488	#DIV/0!
IFT22	4,7158E-17	0,715536997	0,469	0,031	9,9508E-13	1,642094321	0,608978417	15,12903226
KIFAP3	3,61288E-19	0,715332634	0,562	0,043	7,62355E-15	1,641861728	0,609064687	13,06976744
CTHRC1	1,43028E-17	0,714597322	0,312	0,004	3,01804E-13	1,641025119	0,609375194	78
RPS26	3,19387E-06	0,714011212	0,938	0,48	0,067393938	1,640358571	0,609622809	1,954166667
CRIP2	1,00108E-05	0,711555449	0,969	0,496	0,211237807	1,637568719	0,610661396	1,953629032
PTPRZ1	1,58349E-06	0,70952698	0,781	0,281	0,033413213	1,63526787	0,611520607	2,779359431
CCT3	1,40088E-07	0,708976383	0,719	0,215	0,002955992	1,634643898	0,611754035	3,344186047
NDUFA12	1,00963E-08	0,708554547	0,688	0,18	0,000213041	1,634166007	0,611932934	3,822222222
CRIP1	5,36606E-05	0,708443514	0,875	0,43	1	1,634040243	0,611980032	2,034883721
MANF	1,20184E-12	0,708364127	0,719	0,141	2,536E-08	1,633950329	0,612013708	5,09929078
RPS3	5,01589E-05	0,708279943	1	0,762	1	1,633854987	0,612049422	1,312335958
HSD17B10	2,02022E-13	0,707119856	0,688	0,113	4,26286E-09	1,632541715	0,612541775	6,088495575
NAT14	3,14613E-11	0,706524176	0,625	0,113	6,63865E-07	1,631867787	0,612794742	5,530973451
RPS15A	3,83325E-07	0,705922296	1	0,82	0,008088547	1,631187128	0,613050448	1,219512195
TRNP1	6,27223E-13	0,705010854	0,438	0,043	1,3235E-08	1,630156929	0,613437873	10,18604651
SNRPD1	2,54322E-08	0,70318932	0,625	0,16	0,000536646	1,628100006	0,614212884	3,90625
RPS23	6,50825E-06	0,702946893	1	0,816	0,137330688	1,627826448	0,614316103	1,225490196
TMEM106C	9,51785E-11	0,702897589	0,531	0,09	2,00836E-06	1,627770817	0,614337098	5,9
TASP1	3,24098E-12	0,702867832	0,312	0,02	6,83879E-08	1,627737243	0,614349769	15,6
RPS4X	2,92524E-06	0,700746502	1	0,832	0,061725461	1,625345587	0,61525377	1,201923077
LRRFIP1	2,22064E-12	0,700744593	0,625	0,102	4,68578E-08	1,625343437	0,615254584	6,12745098
BMP1	2,42673E-14	0,699413688	0,531	0,059	5,12064E-10	1,623844727	0,615822426	9

Supplementary Table 1

Differentially expressed genes_adjacent-skin-XL_ comparing SC-Keloid vs. (SC-Myel, SC-Nonmyel)								
**gene**	**p_val**	**avg_log2FC**	**pct.1**	**pct.2**	**p_val_adj**	**Foldchange_UP**	**Foldchange_DOWN**	**Ratio_pct1_pct2**
ARHGAP21	1,2573E-20	0,698824214	0,562	0,035	2,65304E-16	1,623181373	0,616074098	16,05714286
EFHD2	1,54355E-11	0,698763472	0,5	0,07	3,25705E-07	1,623113033	0,616100037	7,142857143
KCNS3	9,05393E-12	0,697505884	0,562	0,086	1,91047E-07	1,621698792	0,616637322	6,534883721
PSMD2	1,19973E-09	0,697257496	0,656	0,152	2,53154E-05	1,621419609	0,616743497	4,315789474
ANAPC15	1,41047E-17	0,697013938	0,438	0,023	2,97624E-13	1,621145902	0,616847625	19,04347826
UQCRB	7,81416E-05	0,695454411	0,844	0,438	1	1,61939442	0,617514787	1,926940639
MRPL23	2,52444E-10	0,694870808	0,656	0,137	5,32681E-06	1,61873947	0,617764636	4,788321168
AKR7A2	3,7775E-09	0,691381454	0,469	0,082	7,9709E-05	1,61482906	0,619260592	5,719512195
IKBIP	2,57971E-19	0,690461963	0,531	0,035	5,44345E-15	1,613800188	0,619655399	15,17142857
C17orf49	1,35016E-10	0,690113882	0,562	0,102	2,84897E-06	1,613410872	0,619804922	5,509803922
EPB41L3	1,13615E-10	0,689996517	0,438	0,059	2,39739E-06	1,613279623	0,619855347	7,423728814
PIN1	8,26451E-11	0,689859084	0,781	0,168	1,74389E-06	1,613125948	0,619914398	4,648809524
CHCHD2	4,07424E-06	0,689375922	0,969	0,512	0,085970452	1,612585798	0,620122043	1,892578125
IER3IP1	6,89442E-11	0,689190873	0,688	0,141	1,45479E-06	1,612378972	0,620201589	4,879432624
DBNDD1	3,124E-29	0,688879842	0,469	0	6,59194E-25	1,612031396	0,620335313	#DIV/0!
RPS10	1,85163E-05	0,688512905	0,969	0,699	0,390711572	1,611621441	0,62049311	1,386266094
PRELID1	9,29321E-10	0,687772526	0,688	0,156	1,96096E-05	1,610794583	0,620811623	4,41025641
RASA1	2,80156E-10	0,687274798	0,344	0,035	5,91156E-06	1,610238957	0,62102584	9,828571429
ACBD3	1,51923E-10	0,686039462	0,625	0,121	3,20573E-06	1,608860749	0,621557833	5,165289256
PSMD14	1,1087E-23	0,685971637	0,656	0,043	2,33947E-19	1,608785113	0,621587055	15,25581395
ARPC1B	1,29677E-05	0,684645976	0,938	0,496	0,273630442	1,607307515	0,62215848	1,891129032
LMAN2	7,78316E-09	0,684121612	0,688	0,176	0,000164232	1,606723427	0,622384652	3,909090909
CYB5R3	9,59284E-08	0,683597922	0,75	0,242	0,002024186	1,606140301	0,622610615	3,099173554
RPL13A	4,70843E-06	0,681871095	1	0,801	0,099352563	1,604218989	0,623356292	1,248439451
TCEAL8	1,86582E-12	0,681530855	0,688	0,121	3,93707E-08	1,603840701	0,623503319	5,685950413
FAAP20	3,07283E-08	0,681366059	0,562	0,129	0,000648397	1,603657508	0,623574545	4,356589147
PSMA1	1,08334E-09	0,680918028	0,781	0,207	2,28595E-05	1,603159567	0,623768227	3,77294686
AP1S1	7,44737E-20	0,678119124	0,562	0,039	1,57147E-15	1,600052369	0,624979544	14,41025641
AP1S2	2,07585E-05	0,676580913	0,938	0,496	0,438024852	1,598347292	0,625646257	1,891129032
ZYX	1,47001E-10	0,67650903	0,531	0,094	3,10187E-06	1,598267655	0,625677431	5,64893617

Supplementary Table 1

Differentially expressed genes_adjacent-skin-XL_ comparing SC-Keloid vs. (SC-Myel, SC-Nonmyel)								
**gene**	**p_val**	**avg_log2FC**	**pct.1**	**pct.2**	**p_val_adj**	**Foldchange_UP**	**Foldchange_DOWN**	**Ratio_pct1_pct2**
PDLIM4	7,5994E-05	0,67572664	0,906	0,484	1	1,597401131	0,626016835	1,871900826
EIF4G2	1,37887E-05	0,673844587	0,875	0,449	0,290955445	1,595318617	0,626834032	1,948775056
DAD1	3,8634E-06	0,671850083	0,969	0,547	0,081521628	1,593114636	0,627701219	1,771480804
SELENON	1,22552E-14	0,671743423	0,594	0,074	2,58596E-10	1,59299686	0,627747628	8,027027027
ATP5F1A	2,64299E-07	0,671254969	0,656	0,195	0,005576977	1,59245761	0,627960201	3,364102564
MRPL3	1,23758E-13	0,670688724	0,531	0,066	2,61141E-09	1,591832707	0,628206718	8,045454545
MRPS21	4,082E-10	0,670380128	0,781	0,195	8,61342E-06	1,591492246	0,628341107	4,005128205
UBE2M	8,69443E-09	0,667607467	0,719	0,188	0,000183461	1,588436555	0,629549853	3,824468085
MAP1B	1,76407E-05	0,667386992	0,844	0,406	0,37223556	1,588193826	0,629646069	2,078817734
NUCB2	4,9271E-06	0,667279611	0,5	0,148	0,103966735	1,58807562	0,629692936	3,378378378
COX5B	4,67339E-06	0,667043412	0,875	0,375	0,098613155	1,58781564	0,629796039	2,333333333
RPL34	1,56648E-05	0,66688889	1	0,871	0,330543975	1,587645584	0,629863497	1,148105626
BZW2	7,01189E-11	0,664709927	0,531	0,086	1,47958E-06	1,585249506	0,630815525	6,174418605
NDUFS8	5,71526E-09	0,661838769	0,594	0,133	0,000120598	1,582097782	0,632072184	4,466165414
GRN	1,24078E-08	0,66060333	0,688	0,176	0,000261818	1,580743547	0,632613685	3,909090909
OLA1	1,71598E-09	0,65930738	0,531	0,102	3,62089E-05	1,579324227	0,633182207	5,205882353
PSMA6	2,30461E-09	0,657633206	0,688	0,168	4,86295E-05	1,577492566	0,633917409	4,095238095
EIF5A	0,000111594	0,656870948	0,812	0,367	1	1,576659306	0,634252432	2,21253406
GDI2	1,82373E-09	0,656561429	0,812	0,223	3,84825E-05	1,576321082	0,634388521	3,641255605
PRDX4	5,08872E-10	0,655740668	0,688	0,152	1,07377E-05	1,575424556	0,634749532	4,526315789
BOP1	1,16002E-11	0,655479362	0,375	0,035	2,44776E-07	1,575139235	0,634864511	10,71428571
RPL36	4,84438E-06	0,648676197	0,969	0,812	0,102221232	1,567729003	0,637865344	1,193349754
PARK7	1,59848E-05	0,648559014	0,781	0,348	0,337295323	1,567601669	0,637917157	2,244252874
POLR2K	1,13129E-08	0,648331722	0,719	0,188	0,000238713	1,567354717	0,638017667	3,824468085
TXN2	7,05346E-18	0,647512346	0,562	0,047	1,48835E-13	1,566464794	0,638380131	11,95744681
PHB	8,2542E-06	0,646832893	0,531	0,164	0,174171792	1,565727224	0,638680854	3,237804878
PSMC3	1,34576E-09	0,646492647	0,688	0,16	2,83969E-05	1,565358006	0,638831498	4,3
RPL8	2,64252E-05	0,646037795	0,938	0,777	0,557598502	1,564864558	0,639032941	1,207207207
SDF4	1,26449E-07	0,645909343	0,688	0,199	0,002668199	1,564725236	0,63908984	3,457286432
NDUFC2	1,54815E-06	0,645613808	0,875	0,312	0,032667575	1,564404735	0,63922077	2,804487179

Supplementary Table 1

Differentially expressed genes_adjacent-skin-XL_ comparing SC-Keloid vs. (SC-Myel, SC-Nonmyel)								
**gene**	**p_val**	**avg_log2FC**	**pct.1**	**pct.2**	**p_val_adj**	**Foldchange_UP**	**Foldchange_DOWN**	**Ratio_pct1_pct2**
UQCRC1	9,15428E-09	0,645241776	0,562	0,117	0,000193164	1,56400137	0,639385629	4,803418803
RNH1	9,571E-06	0,644993829	0,812	0,328	0,201957769	1,563732597	0,639495526	2,475609756
ARL6IP4	7,76178E-06	0,644800011	0,812	0,352	0,163781369	1,563522533	0,639581444	2,306818182
NDUFB10	8,53823E-08	0,644089635	0,781	0,234	0,001801653	1,562752852	0,639896449	3,337606838
RPS3A	5,47126E-05	0,643919328	0,969	0,766	1	1,562568383	0,639971992	1,265013055
CHMP2A	3,9707E-10	0,643687222	0,812	0,211	8,37858E-06	1,562317011	0,640074961	3,848341232
CAVIN3	1,68397E-05	0,642070581	0,969	0,555	0,355335292	1,560567307	0,640792612	1,745945946
SKP1	3,83058E-05	0,641664545	1	0,605	0,808290904	1,560128157	0,640972984	1,652892562
TRAPPC1	4,02001E-15	0,641405111	0,625	0,078	8,48261E-11	1,559847631	0,641088258	8,012820513
NACA	1,07808E-05	0,639864653	1	0,797	0,227486016	1,558182971	0,641773154	1,254705144
DYNLL1	7,72669E-08	0,639541248	1	0,516	0,001630409	1,557833716	0,641917035	1,937984496
TSPO	1,97021E-05	0,638173175	0,844	0,406	0,415734598	1,55635716	0,642526038	2,078817734
CTSB	2,40663E-11	0,63785483	0,562	0,09	5,07822E-07	1,556013773	0,642667833	6,244444444
MZT2A	1,63335E-09	0,637367619	0,594	0,121	3,44654E-05	1,555488382	0,642884905	4,909090909
MIF	7,38514E-05	0,636933547	0,906	0,57	1	1,555020443	0,643078363	1,589473684
C19orf53	2,92947E-06	0,63597615	0,75	0,289	0,061814759	1,553988847	0,643505262	2,595155709
BANF1	4,9481E-06	0,635124641	0,688	0,227	0,104409771	1,553071921	0,643885184	3,030837004
RPL32	3,49516E-06	0,634822963	1	0,938	0,073751443	1,552747197	0,644019839	1,066098081
DNAJC10	1,25246E-15	0,63480141	0,656	0,082	2,64283E-11	1,552724	0,64402946	8
NUDT1	4,64033E-13	0,631855665	0,469	0,051	9,79156E-09	1,549556828	0,645345806	9,196078431
SLC35B1	7,88975E-08	0,630793216	0,469	0,094	0,001664816	1,548416102	0,645821235	4,989361702
CKLF	1,41352E-09	0,630190764	0,5	0,086	2,98268E-05	1,547769638	0,646090978	5,813953488
TUBA1B	0,036503545	0,629680844	0,938	0,691	1	1,547222675	0,64631938	1,357452967
SRA1	2,2339E-10	0,629202339	0,469	0,07	4,71375E-06	1,546709587	0,646533783	6,7
DRAP1	7,7254E-05	0,628779246	0,781	0,336	1	1,546256057	0,646723417	2,324404762
MAPRE3	1,16171E-10	0,628604675	0,375	0,043	2,45131E-06	1,546068966	0,646801677	8,720930233
ANXA4	9,60629E-11	0,628286707	0,688	0,145	2,02702E-06	1,545728251	0,646944247	4,744827586
NDUFB7	1,77149E-05	0,627954952	0,844	0,328	0,373802353	1,545372844	0,647093032	2,573170732
TBCA	4,49368E-05	0,627942902	0,875	0,5	0,948210751	1,545359936	0,647098437	1,75
MRPS34	2,34145E-10	0,627727577	0,594	0,113	4,9407E-06	1,545129306	0,647195025	5,256637168

Supplementary Table 1

Differentially expressed genes_adjacent-skin-XL_ comparing SC-Keloid vs. (SC-Myel, SC-Nonmyel)								
**gene**	**p_val**	**avg_log2FC**	**pct.1**	**pct.2**	**p_val_adj**	**Foldchange_UP**	**Foldchange_DOWN**	**Ratio_pct1_pct2**
LAP3	1,93605E-14	0,62757391	0,5	0,051	4,08525E-10	1,544964737	0,647263964	9,803921569
NRBP1	4,00619E-10	0,627487558	0,656	0,137	8,45345E-06	1,544872267	0,647302707	4,788321168
PSMA4	2,96861E-08	0,626727563	0,719	0,207	0,000626406	1,54405866	0,647643788	3,473429952
ZNF706	2,40485E-07	0,626266612	0,969	0,371	0,005074471	1,543565402	0,647850748	2,611859838
PLPP5	3,61686E-15	0,625431657	0,469	0,039	7,63193E-11	1,542672327	0,648225798	12,02564103
BRI3	1,31501E-06	0,62540539	0,781	0,273	0,027747991	1,54264424	0,6482376	2,860805861
SYNCRIP	8,67577E-09	0,623478143	0,625	0,148	0,000183067	1,54058485	0,649104137	4,222972973
SEC61G	3,73667E-05	0,622434915	0,75	0,363	0,788473882	1,53947124	0,649573681	2,066115702
LAMTOR5	2,16223E-06	0,622374706	0,875	0,352	0,04562527	1,539406993	0,649600791	2,485795455
TMEM147	1,43922E-10	0,622312309	0,594	0,109	3,03691E-06	1,539340415	0,649628887	5,449541284
RPL11	4,00939E-06	0,621048452	1	0,871	0,084602221	1,537992483	0,650198236	1,148105626
MYL3	0,004841742	0,62049564	0,031	0	1	1,537403267	0,650447427	#DIV/0!
P3H2	9,69112E-18	0,619282021	0,344	0,008	2,04492E-13	1,536110522	0,650994825	43
GAP43	6,89484E-07	0,619050431	0,312	0,051	0,0145488	1,535863956	0,651099335	6,117647059
SPTSSA	1,55593E-06	0,618921069	0,906	0,359	0,032831648	1,535726246	0,651157719	2,52367688
HIGD1A	1,05639E-10	0,618836377	0,688	0,141	2,22909E-06	1,535636096	0,651195946	4,879432624
RIC8A	2,63322E-17	0,617602552	0,531	0,043	5,55636E-13	1,534323347	0,651753102	12,34883721
RPL35	9,1366E-06	0,617490788	1	0,793	0,192791309	1,534204489	0,651803594	1,261034048
TIMM10	6,64612E-16	0,617465309	0,531	0,051	1,4024E-11	1,534177394	0,651815106	10,41176471
SPPL2A	6,06116E-05	0,6172588	0,781	0,352	1	1,533957807	0,651908413	2,21875
HMGB1	3,95027E-06	0,6172048	1	0,824	0,083354737	1,533900391	0,651932815	1,213592233
ACSBG1	9,90862E-17	0,617178771	0,375	0,016	2,09082E-12	1,533872717	0,651944577	23,4375
URM1	2,27157E-10	0,613163036	0,531	0,094	4,79323E-06	1,529609126	0,65376179	5,64893617
SSNA1	8,58711E-08	0,613089431	0,594	0,145	0,001811965	1,529531089	0,653795145	4,096551724
FIBP	4,8988E-10	0,61246499	0,562	0,105	1,03369E-05	1,528869206	0,654078188	5,352380952
LSM1	4,80798E-15	0,611077589	0,625	0,078	1,01453E-10	1,52739964	0,6547075	8,012820513
TUSC3	1,38925E-21	0,610499127	0,344	0	2,93145E-17	1,526787338	0,654970064	#DIV/0!
CREB3	7,21863E-08	0,610401323	0,5	0,109	0,001523202	1,526683837	0,655014467	4,587155963
RPL37A	2,00353E-06	0,609741938	1	0,902	0,042276416	1,525986224	0,655313911	1,10864745
IL11	1,83852E-05	0,608702389	0,156	0,016	0,387946114	1,524887054	0,655786274	9,75

Supplementary Table 1

Differentially expressed genes_adjacent-skin-XL_ comparing SC-Keloid vs. (SC-Myel, SC-Nonmyel)								
**gene**	**p_val**	**avg_log2FC**	**pct.1**	**pct.2**	**p_val_adj**	**Foldchange_UP**	**Foldchange_DOWN**	**Ratio_pct1_pct2**
ZCRB1	5,64784E-13	0,608026882	0,688	0,113	1,19175E-08	1,52417323	0,656093402	6,088495575
SEPTIN9	5,46209E-08	0,607929082	0,781	0,227	0,001152556	1,524069911	0,65613788	3,440528634
ACOT7	2,9033E-18	0,606907637	0,531	0,039	6,12626E-14	1,522991233	0,656602598	13,61538462
NDUFA7	3,55047E-12	0,606088296	0,625	0,105	7,49185E-08	1,522126535	0,656975604	5,952380952
B4GALT1	3,48157E-09	0,605912269	0,5	0,094	7,34646E-05	1,521940828	0,657055768	5,319148936
PCBD1	8,94097E-10	0,605575297	0,375	0,047	1,88663E-05	1,521585388	0,657209256	7,978723404
ATP5PF	5,91898E-05	0,605560854	0,781	0,375	1	1,521570156	0,657215835	2,082666667
PARP1	6,44279E-07	0,605423709	0,469	0,113	0,013594923	1,521425519	0,657278314	4,150442478
AKT1	1,12093E-09	0,605284664	0,781	0,188	2,36528E-05	1,521278893	0,657341665	4,154255319
PDHB	2,42673E-14	0,604988443	0,531	0,059	5,12064E-10	1,520966569	0,657476647	9
SUGT1	7,90171E-08	0,604519183	0,531	0,121	0,001667341	1,520471931	0,657690537	4,388429752
HSPB2	0,000301659	0,603975146	0,438	0,148	1	1,519898673	0,657938597	2,959459459
DECR1	3,98814E-07	0,60338366	0,562	0,145	0,00841538	1,519275661	0,658208399	3,875862069
RPL35A	1,34564E-05	0,603295968	1	0,84	0,283943411	1,519183318	0,658248408	1,19047619
MLEC	5,81527E-09	0,602947275	0,625	0,141	0,000122708	1,518816181	0,658407523	4,432624113
PSMD1	5,08269E-10	0,602309281	0,594	0,117	1,0725E-05	1,518144673	0,658698751	5,076923077
UBL5	7,11952E-05	0,602140718	0,906	0,527	1	1,517967305	0,658775717	1,719165085
CD2BP2	5,75559E-13	0,601452865	0,562	0,078	1,21449E-08	1,517243737	0,659089885	7,205128205
HADHB	2,87858E-05	0,600659669	0,625	0,219	0,60740838	1,516409783	0,659452353	2,853881279
SDC3	2,06975E-06	0,600005121	0,938	0,398	0,043673803	1,515721947	0,659751613	2,35678392
SWI5	7,08319E-12	0,599690613	0,531	0,078	1,49462E-07	1,515391554	0,659895455	6,807692308
ATP5MC3	1,50772E-06	0,599106176	0,844	0,332	0,031814504	1,514777792	0,660162834	2,542168675
DUSP10	2,01087E-19	0,598201932	0,469	0,023	4,24314E-15	1,513828666	0,660576737	20,39130435
YWHAB	0,000144927	0,596912817	0,812	0,445	1	1,512476595	0,661167256	1,824719101
CTSD	1,92675E-05	0,59581634	0,844	0,367	0,406562689	1,511327519	0,661669948	2,29972752
RPL37	1,06417E-06	0,594811822	1	0,898	0,022454962	1,51027558	0,662130815	1,113585746
NABP2	3,76964E-13	0,594784687	0,438	0,043	7,95431E-09	1,510247174	0,662143268	10,18604651
MTX2	2,44254E-17	0,593988568	0,531	0,043	5,15401E-13	1,509414008	0,662508758	12,34883721
TMEM47	1,0548E-08	0,593682836	0,719	0,188	0,000222573	1,50909417	0,66264917	3,824468085
PLPP1	4,73794E-05	0,593371895	0,969	0,57	0,999752774	1,508768954	0,662792005	1,7

Supplementary Table 1

Differentially expressed genes_adjacent-skin-XL_ comparing SC-Keloid vs. (SC-Myel, SC-Nonmyel)								
**gene**	**p_val**	**avg_log2FC**	**pct.1**	**pct.2**	**p_val_adj**	**Foldchange_UP**	**Foldchange_DOWN**	**Ratio_pct1_pct2**
MPDU1	3,071E-13	0,592646143	0,438	0,043	6,48011E-09	1,508010154	0,663125508	10,18604651
CDKN2A	2,00705E-19	0,592250411	0,344	0,004	4,23507E-15	1,507596563	0,663307429	86
CCDC167	1,34816E-15	0,592174219	0,438	0,031	2,84476E-11	1,507516945	0,663342461	14,12903226
EDNRB	3,64754E-07	0,591194188	0,656	0,184	0,007696675	1,506493228	0,663793226	3,565217391
EXOSC10	1,29997E-08	0,590178976	0,406	0,066	0,000274307	1,505433495	0,664260496	6,151515152
LSM10	1,22053E-12	0,590116576	0,531	0,07	2,57544E-08	1,505368383	0,664289227	7,585714286
EFEMP2	1,62891E-11	0,590072492	0,531	0,078	3,43716E-07	1,505322385	0,664309526	6,807692308
STMN1	0,000460134	0,588454757	0,875	0,438	1	1,503635369	0,665054853	1,997716895
MELTF	1,86558E-08	0,588238575	0,219	0,016	0,000393656	1,503410072	0,665154517	13,6875
RPL26L1	1,2471E-11	0,588077518	0,531	0,078	2,6315E-07	1,503242247	0,665228776	6,807692308
ANXA11	1,0508E-12	0,58767225	0,625	0,098	2,21729E-08	1,50282003	0,665415672	6,37755102
NCBP2	1,1201E-11	0,587646911	0,469	0,059	2,36352E-07	1,502793635	0,665427359	7,949152542
KRTCAP2	4,00777E-06	0,586964979	0,906	0,352	0,084568053	1,502083464	0,665741967	2,573863636
SNX17	2,28791E-11	0,586954415	0,562	0,09	4,82772E-07	1,502072465	0,665746842	6,244444444
SMIM26	5,11562E-07	0,586477999	0,594	0,164	0,010794478	1,501576523	0,665966725	3,62195122
C1QBP	4,06621E-08	0,585250508	0,594	0,145	0,000858012	1,500299477	0,666533592	4,096551724
RTN3	1,24016E-05	0,584445713	0,531	0,156	0,261685793	1,499462781	0,666905516	3,403846154
MYO10	2,3932E-12	0,583637966	0,406	0,039	5,04989E-08	1,498623486	0,667279013	10,41025641
C11orf58	1,04775E-05	0,583289224	0,875	0,359	0,221085833	1,498261268	0,667440333	2,437325905
HYI	1,21188E-08	0,583287814	0,562	0,125	0,00025572	1,498259804	0,667440986	4,496
CRLF1	1,41588E-11	0,583229217	0,281	0,016	2,98764E-07	1,498198951	0,667468095	17,5625
REEP5	1,42268E-06	0,582833385	0,812	0,277	0,030019997	1,497787947	0,667651253	2,931407942
MACIR	5,47843E-13	0,582077758	0,438	0,043	1,156E-08	1,49700367	0,668001035	10,18604651
IFI27	0,005375672	0,580844946	0,5	0,242	1	1,495724997	0,668572099	2,066115702
FHL3	2,15942E-12	0,580697896	0,406	0,039	4,5566E-08	1,495572549	0,668640248	10,41025641
CAPZB	7,12848E-05	0,580363177	0,719	0,305	1	1,495225602	0,668795397	2,357377049
SNRPD3	7,28727E-09	0,580276666	0,688	0,164	0,000153769	1,495135943	0,668835502	4,195121951
SNX3	0,000205302	0,579743122	0,812	0,371	1	1,494583108	0,6690829	2,188679245
COL5A3	6,60168E-06	0,579415517	0,781	0,281	0,139302038	1,494243759	0,669234852	2,779359431
PSMC1	3,66446E-07	0,578857175	0,5	0,121	0,007732382	1,493665579	0,669493904	4,132231405

Supplementary Table 1

Differentially expressed genes_adjacent-skin-XL_ comparing SC-Keloid vs. (SC-Myel, SC-Nonmyel)								
**gene**	**p_val**	**avg_log2FC**	**pct.1**	**pct.2**	**p_val_adj**	**Foldchange_UP**	**Foldchange_DOWN**	**Ratio_pct1_pct2**
SEC61B	1,56385E-05	0,578297358	0,906	0,438	0,329987962	1,493086095	0,669753742	2,068493151
GLT8D1	2,52672E-11	0,576574408	0,625	0,109	5,33162E-07	1,49130403	0,670554079	5,733944954
EIF6	1,02563E-09	0,576277872	0,625	0,133	2,16418E-05	1,490997534	0,670691921	4,69924812
FSCN1	6,56269E-11	0,576070705	0,625	0,117	1,38479E-06	1,490783447	0,670788237	5,341880342
ISLR	2,73095E-21	0,575855689	0,375	0,004	5,76258E-17	1,49056128	0,670888217	93,75
SERBP1	1,91503E-06	0,575438748	0,875	0,336	0,040408948	1,490130568	0,671082133	2,604166667
RPS7	4,13701E-05	0,575327785	1	0,824	0,872951003	1,490015962	0,67113375	1,213592233
C5orf15	6,9939E-09	0,575211457	0,625	0,145	0,000147578	1,489895823	0,671187867	4,310344828
CAPG	3,19317E-23	0,574808762	0,406	0,004	6,73791E-19	1,489480011	0,67137524	101,5
TPGS1	2,91529E-10	0,574360458	0,594	0,109	6,15156E-06	1,48901724	0,671583896	5,449541284
PRLR	0,000247725	0,574319583	0,156	0,023	1	1,488975053	0,671602924	6,782608696
DYRK2	1,26059E-11	0,574271275	0,406	0,043	2,65998E-07	1,488925197	0,671625413	9,441860465
MTCH2	5,9331E-06	0,573268435	0,375	0,082	0,125194427	1,487890581	0,672092432	4,573170732
MORF4L2	4,99404E-06	0,573140246	0,938	0,41	0,105379137	1,487758382	0,672152153	2,287804878
DCTN3	1,99675E-07	0,572667149	0,625	0,168	0,004213349	1,487270588	0,672372605	3,720238095
SRPX	1,49624E-12	0,572247601	0,5	0,062	3,15721E-08	1,486838139	0,672568166	8,064516129
SLC25A5	1,21953E-06	0,572237275	0,875	0,34	0,025733231	1,486827498	0,672572979	2,573529412
DUT	2,10918E-05	0,5705945	0,656	0,242	0,445058645	1,485135434	0,673339264	2,710743802
PPM1G	8,76082E-10	0,568427029	0,5	0,086	1,84862E-05	1,482905876	0,674351634	5,813953488
SLC1A5	8,64449E-12	0,56815764	0,438	0,051	1,82407E-07	1,482629005	0,674477564	8,588235294
RPS20	3,72948E-05	0,566872899	0,938	0,547	0,786957122	1,481309289	0,675078464	1,714808044
GLO1	3,1714E-08	0,566775307	0,594	0,137	0,000669196	1,481209089	0,675124132	4,335766423
UQCC2	4,38893E-09	0,566762504	0,594	0,125	9,26109E-05	1,481195944	0,675130123	4,752
EMC7	4,45928E-07	0,56552525	0,688	0,199	0,009409531	1,479926216	0,675709362	3,457286432
CHID1	2,75274E-14	0,565366656	0,688	0,105	5,80856E-10	1,479763538	0,675783647	6,552380952
TPM3	1,14805E-06	0,56521995	0,781	0,273	0,024224989	1,47961307	0,67585237	2,860805861
LY6E	5,87694E-11	0,565152629	0,75	0,16	1,24009E-06	1,479544029	0,675883908	4,6875
SRI	1,07423E-05	0,564778214	0,812	0,312	0,226673373	1,4791601	0,676059339	2,602564103
FAM89B	1,62891E-11	0,564681199	0,531	0,078	3,43716E-07	1,479060637	0,676104803	6,807692308
TRIR	0,000291214	0,564201901	0,875	0,41	1	1,478569338	0,676329459	2,134146341

Supplementary Table 1

Differentially expressed genes_adjacent-skin-XL_ comparing SC-Keloid vs. (SC-Myel, SC-Nonmyel)								
**gene**	**p_val**	**avg_log2FC**	**pct.1**	**pct.2**	**p_val_adj**	**Foldchange_UP**	**Foldchange_DOWN**	**Ratio_pct1_pct2**
GUK1	1,90277E-05	0,56397763	0,844	0,379	0,401502502	1,478339508	0,676434604	2,226912929
CLDN4	1,59511E-16	0,563697503	0,375	0,016	3,36584E-12	1,478052489	0,676565959	23,4375
NPM1	0,000278114	0,561952259	0,906	0,539	1	1,476265553	0,677384904	1,680890538
TNFRSF19	4,27264E-13	0,561639693	0,688	0,109	9,01569E-09	1,475945749	0,677531678	6,311926606
POLR2G	6,42263E-11	0,56126385	0,656	0,129	1,35524E-06	1,475561294	0,677708208	5,085271318
PTPRE	2,84766E-08	0,561176236	0,469	0,09	0,000600884	1,475471686	0,677749366	5,211111111
TOMM6	1,40007E-07	0,560783145	0,812	0,25	0,002954284	1,475069719	0,677934058	3,248
CMPK1	2,29989E-06	0,560710118	0,625	0,188	0,048529927	1,474995055	0,677968374	3,324468085
RPS9	7,39803E-05	0,560557262	1	0,789	1	1,474838785	0,67804021	1,267427123
RRAGA	6,58558E-09	0,560498381	0,656	0,152	0,000138962	1,474778594	0,678067884	4,315789474
APOC2	1,24307E-11	0,559850657	0,375	0,035	2,623E-07	1,474116614	0,678372383	10,71428571
VDAC2	5,87317E-06	0,559642338	0,75	0,262	0,123929827	1,473903773	0,678470344	2,86259542
UFC1	1,62058E-11	0,558748314	0,719	0,137	3,41959E-07	1,472990692	0,678890916	5,248175182
CHST3	2,09208E-12	0,558475102	0,656	0,109	4,4145E-08	1,47271177	0,679019494	6,018348624
EIF3M	2,28222E-07	0,558163646	0,656	0,188	0,004815715	1,472393867	0,6791661	3,489361702
RPS27L	0,000211355	0,558075906	0,844	0,406	1	1,472304324	0,679207406	2,078817734
ALKBH7	1,16085E-09	0,556246491	0,656	0,141	2,44951E-05	1,470438546	0,680069224	4,65248227
COX4I1	8,0391E-05	0,555801695	0,906	0,504	1	1,469985267	0,680278927	1,797619048
HOXD-AS2	1,33663E-19	0,555661205	0,406	0,012	2,82043E-15	1,469842125	0,680345176	33,83333333
PRAF2	6,60292E-09	0,555658643	0,406	0,066	0,000139328	1,469839516	0,680346384	6,151515152
SRP72	4,29191E-09	0,555064918	0,719	0,18	9,05635E-05	1,469234744	0,680626431	3,994444444
DOK1	2,89553E-10	0,554938606	0,344	0,035	6,10986E-06	1,469106114	0,680686024	9,828571429
TMEM230	7,35497E-08	0,554379104	0,688	0,184	0,001551972	1,46853648	0,680950057	3,739130435
DCBLD2	6,51352E-06	0,554211699	0,5	0,145	0,1374417	1,468366086	0,681029077	3,448275862
RHOC	0,000312485	0,553881136	0,938	0,527	1	1,468029679	0,681185138	1,779886148
AFAP1L1	5,69635E-19	0,553574655	0,469	0,023	1,20199E-14	1,46771785	0,681329862	20,39130435
ARF4	1,16459E-05	0,552866932	0,781	0,324	0,245739348	1,466998028	0,681664174	2,410493827
UNC5B	1,82586E-19	0,552738878	0,375	0,008	3,85275E-15	1,466867823	0,681724682	46,875
AP2A1	1,06718E-07	0,552127556	0,406	0,074	0,00225185	1,46624639	0,682013614	5,486486486
BTF3L4	1,95567E-09	0,551910323	0,625	0,129	4,12666E-05	1,466025627	0,682116316	4,84496124

Supplementary Table 1

Differentially expressed genes_adjacent-skin-XL_ comparing SC-Keloid vs. (SC-Myel, SC-Nonmyel)								
**gene**	**p_val**	**avg_log2FC**	**pct.1**	**pct.2**	**p_val_adj**	**Foldchange_UP**	**Foldchange_DOWN**	**Ratio_pct1_pct2**
NDUFB3	8,91972E-10	0,551418708	0,656	0,141	1,88215E-05	1,465526147	0,682348795	4,65248227
PRKAR1B	1,12056E-11	0,550879423	0,375	0,035	2,3645E-07	1,46497843	0,682603907	10,71428571
DESI1	6,7021E-10	0,55086944	0,406	0,055	1,41421E-05	1,464968293	0,68260863	7,381818182
JTB	9,97371E-07	0,550652304	0,75	0,23	0,021045521	1,464747821	0,682711376	3,260869565
CUTA	1,32821E-05	0,550180191	0,719	0,273	0,280266545	1,46426857	0,682934825	2,633699634
NDUFS4	9,26729E-06	0,549931644	0,719	0,262	0,195549151	1,464016328	0,683052491	2,744274809
APP	3,0478E-05	0,549816008	0,844	0,406	0,643116158	1,463898988	0,683107242	2,078817734
HS2ST1	1,39928E-07	0,549747207	0,25	0,027	0,002952628	1,463829178	0,683139819	9,259259259
FBN1	5,17299E-12	0,549550849	0,531	0,074	1,09155E-07	1,463629956	0,683232805	7,175675676
TRAF1	2,19124E-17	0,549548652	0,438	0,023	4,62374E-13	1,463627727	0,683233845	19,04347826
CMC1	2,15119E-17	0,549257873	0,5	0,035	4,53922E-13	1,463332759	0,683371567	14,28571429
PRDX6	2,39676E-06	0,548914362	0,812	0,312	0,050573985	1,462984375	0,683534299	2,602564103
SQLE	2,32831E-09	0,54759265	0,438	0,07	4,91296E-05	1,461644689	0,6841608	6,257142857
C2orf27A	6,96316E-22	0,546867177	0,469	0,016	1,4693E-17	1,460909873	0,684504923	29,3125
EID1	4,1613E-05	0,54552204	0,969	0,57	0,878075411	1,459548387	0,685143438	1,7
NDUFB4	1,07526E-05	0,545054853	0,812	0,336	0,22689104	1,459075819	0,685365344	2,416666667
METTL9	1,48175E-13	0,544316662	0,656	0,098	3,12664E-09	1,458329437	0,685716118	6,693877551
ACTR10	4,41915E-06	0,544246025	0,594	0,18	0,09324855	1,458258036	0,685749693	3,3
TMBIM4	1,1109E-05	0,544071775	0,812	0,324	0,234411009	1,458081918	0,685832523	2,50617284
PCNA	9,46056E-08	0,543886623	0,375	0,062	0,001996274	1,457894803	0,685920546	6,048387097
CDK4	6,04836E-09	0,542904385	0,531	0,105	0,000127626	1,456902554	0,686387705	5,057142857
ITIH5	0,000216124	0,542055553	0,469	0,168	1	1,456045615	0,686791671	2,791666667
CNIH4	6,12691E-08	0,540390867	0,469	0,094	0,00129284	1,454366493	0,687584598	4,989361702
TMEM115	5,87232E-07	0,54027445	0,344	0,059	0,012391183	1,454249139	0,687640084	5,830508475
SPCS2	0,000379257	0,539528905	0,875	0,418	1	1,453497817	0,687995529	2,093301435
ECHDC1	1,86116E-09	0,539465902	0,625	0,133	3,92724E-05	1,453434344	0,688025575	4,69924812
ADH5	2,84037E-06	0,539335793	0,594	0,168	0,05993463	1,453303272	0,688087627	3,535714286
COX8A	7,42334E-05	0,539024516	0,812	0,375	1	1,45298974	0,688236105	2,165333333
MRPS12	4,11174E-16	0,538687993	0,469	0,035	8,67619E-12	1,452650855	0,688396662	13,4
VKORC1	8,46223E-07	0,538666269	0,656	0,191	0,017856142	1,452628982	0,688407028	3,434554974

Supplementary Table 1

Differentially expressed genes_adjacent-skin-XL_ comparing SC-Keloid vs. (SC-Myel, SC-Nonmyel)								
**gene**	**p_val**	**avg_log2FC**	**pct.1**	**pct.2**	**p_val_adj**	**Foldchange_UP**	**Foldchange_DOWN**	**Ratio_pct1_pct2**
HAGH	3,73533E-11	0,538536267	0,594	0,102	7,88192E-07	1,45249809	0,688469064	5,823529412
ACP1	7,15714E-16	0,538275764	0,688	0,09	1,51023E-11	1,452235841	0,688593389	7,644444444
RBIS	4,43793E-06	0,53822531	0,781	0,273	0,093644721	1,452185054	0,688617471	2,860805861
ILK	3,37245E-07	0,538222784	0,688	0,207	0,007116214	1,452182512	0,688618677	3,323671498
RAB34	2,20931E-07	0,538165346	0,688	0,199	0,004661874	1,452124697	0,688646094	3,457286432
S100B	0,001632504	0,537796454	1	0,805	1	1,451753441	0,6888222	1,242236025
TMA7	0,000302107	0,537631377	0,938	0,652	1	1,451587338	0,688901022	1,438650307
EIF3L	1,33815E-06	0,537586731	0,688	0,215	0,02823627	1,451542417	0,688922341	3,2
SEMA3C	9,39893E-06	0,536338446	0,938	0,395	0,198326859	1,45028702	0,689518686	2,374683544
DNM1	9,87864E-16	0,536222317	0,281	0,004	2,08449E-11	1,450170284	0,689574191	70,25
EPDR1	3,95687E-08	0,535836573	0,406	0,07	0,00083494	1,449782594	0,689758592	5,8
SNRPB2	4,75861E-05	0,535761295	0,656	0,242	1	1,449706948	0,689794583	2,710743802
EEF1E1	2,16004E-10	0,535582069	0,406	0,051	4,55789E-06	1,449526862	0,689880282	7,960784314
DNAJB11	4,0548E-07	0,53467486	0,625	0,172	0,008556038	1,448615643	0,690314235	3,63372093
AP3S1	3,09333E-07	0,534208285	0,531	0,133	0,006527244	1,448147229	0,690537523	3,992481203
SPARC	1,67658E-05	0,534176048	1	0,926	0,353774692	1,448114871	0,690552952	1,079913607
COX5A	1,79196E-08	0,533581309	0,656	0,16	0,000378122	1,447518021	0,690837686	4,1
MORN2	1,08008E-15	0,533011693	0,531	0,051	2,27908E-11	1,446946613	0,691110502	10,41176471
ITGB1BP1	6,37682E-07	0,532574588	0,781	0,227	0,013455723	1,446508286	0,691319925	3,440528634
UBE2S	7,91444E-05	0,532160623	0,594	0,207	1	1,446093287	0,69151832	2,869565217
TTYH3	6,98239E-08	0,531674092	0,312	0,043	0,001473355	1,445605692	0,691751565	7,255813953
GPAA1	2,42399E-09	0,531381308	0,625	0,133	5,11486E-05	1,445312347	0,691891965	4,69924812
ACTL6A	7,6787E-13	0,529620949	0,438	0,043	1,62028E-08	1,44354987	0,692736718	10,18604651
SNRNP200	1,33027E-07	0,529543229	0,5	0,113	0,002807008	1,443472107	0,692774038	4,424778761
CSNK2B	2,88556E-05	0,529214228	0,625	0,215	0,608882415	1,443142965	0,692932041	2,906976744
PYCR1	1,27193E-17	0,529129952	0,312	0,004	2,68389E-13	1,443058666	0,69297252	78
NDUFS7	1,2802E-08	0,528660765	0,688	0,168	0,000270134	1,442589437	0,693197922	4,095238095
PSMB7	9,99137E-07	0,528500323	0,688	0,215	0,021082797	1,442429015	0,693275017	3,2
USF2	1,89393E-06	0,527987987	0,625	0,188	0,039963853	1,441916865	0,693521259	3,324468085
LRRCC1	7,25359E-08	0,526961962	0,469	0,094	0,001530579	1,440891758	0,694014658	4,989361702

Supplementary Table 1

Differentially expressed genes_adjacent-skin-XL_ comparing SC-Keloid vs. (SC-Myel, SC-Nonmyel)								
**gene**	**p_val**	**avg_log2FC**	**pct.1**	**pct.2**	**p_val_adj**	**Foldchange_UP**	**Foldchange_DOWN**	**Ratio_pct1_pct2**
GSTZ1	1,29948E-13	0,526550231	0,469	0,047	2,74203E-09	1,4404806	0,694212751	9,978723404
MRPL15	3,19317E-23	0,526462621	0,406	0,004	6,73791E-19	1,440393127	0,694254909	101,5
NNT	1,2714E-17	0,526303124	0,344	0,008	2,68278E-13	1,440233893	0,694331667	43
PRKDC	1,39386E-07	0,526235155	0,656	0,176	0,002941179	1,440166042	0,694364379	3,727272727
YWHAH	2,06445E-05	0,525948476	0,719	0,273	0,435618556	1,439879894	0,694502371	2,633699634
PDAP1	2,38541E-08	0,525317599	0,719	0,191	0,000503346	1,439250386	0,694806136	3,764397906
HOXB7	2,20066E-25	0,525129395	0,406	0	4,64361E-21	1,439062643	0,694896782	#DIV/0!
MDH2	1,18006E-06	0,525112311	0,656	0,199	0,024900411	1,439045603	0,694905011	3,296482412
COX17	6,05974E-07	0,525010083	0,688	0,199	0,012786665	1,438943637	0,694954253	3,457286432
OPRD1	1,0613E-19	0,524924345	0,312	0	2,23945E-15	1,438858125	0,694995554	#DIV/0!
COX6C	4,41202E-06	0,524648854	0,938	0,461	0,093097957	1,438583393	0,69512828	2,034707158
CCPG1	8,84602E-09	0,524545969	0,656	0,148	0,00018666	1,438480804	0,695177855	4,432432432
ZNF428	3,92765E-06	0,524379657	0,812	0,305	0,082877431	1,438314987	0,695257999	2,662295082
TCEA1	1,54645E-07	0,523970164	0,844	0,262	0,00326316	1,437906795	0,695455368	3,221374046
ZNF385A	9,69112E-18	0,523362778	0,344	0,008	2,04492E-13	1,437301553	0,695748222	43
NDUFA11	0,000111281	0,523021766	0,812	0,344	1	1,436961856	0,695912697	2,360465116
SCAND1	7,46874E-09	0,522742353	0,719	0,184	0,000157598	1,43668358	0,69604749	3,907608696
ROMO1	2,57838E-06	0,522017014	0,719	0,242	0,054406368	1,435961445	0,696397527	2,97107438
HMGN3	3,96906E-05	0,521239839	0,812	0,34	0,83751069	1,435188105	0,696772776	2,388235294
AGTRAP	2,54026E-10	0,521136641	0,375	0,043	5,36021E-06	1,435085449	0,696822618	8,720930233
GNB1	2,30978E-05	0,521013195	0,812	0,32	0,487387371	1,434962658	0,696882246	2,5375
VPS25	1,9508E-14	0,520986855	0,469	0,043	4,11638E-10	1,434936461	0,696894969	10,90697674
DR1	7,64549E-09	0,519796621	0,469	0,082	0,000161327	1,433753115	0,69747015	5,719512195
GNG10	1,59374E-07	0,519661549	0,719	0,203	0,003362951	1,433618887	0,697535453	3,541871921
AKAP12	2,18881E-05	0,519586161	0,469	0,141	0,461860409	1,433543975	0,697571904	3,326241135
BCL2L13	8,2354E-13	0,518575324	0,344	0,023	1,73775E-08	1,432539902	0,698060835	14,95652174
SLC35B2	2,24393E-09	0,518131969	0,5	0,09	4,73491E-05	1,432099735	0,698275389	5,555555556
TMED2	7,58628E-08	0,517528699	0,719	0,199	0,001600781	1,431501021	0,698567438	3,613065327
MED11	1,20084E-11	0,517443829	0,375	0,035	2,5339E-07	1,431416812	0,698608534	10,71428571
COA3	1,19307E-13	0,516859849	0,594	0,078	2,5175E-09	1,430837514	0,698891377	7,615384615

Supplementary Table 1

Differentially expressed genes_adjacent-skin-XL_ comparing SC-Keloid vs. (SC-Myel, SC-Nonmyel)								
**gene**	**p_val**	**avg_log2FC**	**pct.1**	**pct.2**	**p_val_adj**	**Foldchange_UP**	**Foldchange_DOWN**	**Ratio_pct1_pct2**
KDELR1	5,75219E-08	0,516111715	0,75	0,215	0,001213769	1,430095722	0,699253892	3,488372093
C1orf43	1,07259E-05	0,514333697	0,656	0,223	0,226327729	1,428334317	0,700116204	2,941704036
ITGA1	2,84154E-07	0,514315704	0,688	0,199	0,005995939	1,428316503	0,700124936	3,457286432
VIM	0,001640874	0,514232538	1	0,988	1	1,428234169	0,700165296	1,012145749
PHB2	1,06586E-06	0,514065161	0,625	0,184	0,022490618	1,428068479	0,700246532	3,39673913
RPL31	0,000225636	0,513761272	0,875	0,441	1	1,427767702	0,700394048	1,984126984
NUDT21	6,41542E-07	0,51352622	0,438	0,094	0,01353718	1,427535101	0,700508169	4,659574468
HSP90B1	0,000731824	0,51344568	0,844	0,543	1	1,427455409	0,700547277	1,554327808
SHTN1	6,79406E-16	0,513091237	0,469	0,035	1,43362E-11	1,427104754	0,700719409	13,4
RGS16	1,80366E-08	0,512754098	0,75	0,23	0,00038059	1,426771297	0,700883177	3,260869565
TMEM141	4,53448E-10	0,512013657	0,469	0,07	9,56821E-06	1,426039217	0,701242987	6,7
FAM114A1	6,6626E-07	0,511489934	0,625	0,18	0,014058751	1,425521634	0,701497596	3,472222222
MAZ	2,8838E-06	0,51100833	0,531	0,148	0,060851059	1,425045842	0,701731811	3,587837838
MVP	3,84429E-12	0,510869231	0,5	0,062	8,11184E-08	1,424908451	0,701799473	8,064516129
APEX1	9,94847E-09	0,510718034	0,594	0,133	0,000209923	1,424759126	0,701873027	4,466165414
COPZ1	2,0275E-07	0,51061014	0,469	0,102	0,004278236	1,424652578	0,701925519	4,598039216
SMC4	5,21449E-08	0,509751282	0,344	0,051	0,00110031	1,423804713	0,70234351	6,745098039
PEF1	2,11029E-10	0,509438463	0,562	0,098	4,45293E-06	1,423496024	0,702495816	5,734693878
ARHGAP12	6,21281E-08	0,508608728	0,5	0,105	0,001310964	1,422677566	0,702899957	4,761904762
CNN2	1,2714E-17	0,508384364	0,344	0,008	2,68278E-13	1,422456332	0,703009279	43
BAX	4,3271E-06	0,50755742	0,531	0,152	0,091306147	1,421641222	0,703412355	3,493421053
CSPG4	7,00097E-10	0,507391638	0,562	0,102	1,47728E-05	1,42147787	0,703493189	5,509803922
MRPL20	2,44208E-07	0,507220431	0,656	0,184	0,005153024	1,42130919	0,703576679	3,565217391
ERH	7,8485E-06	0,506905914	0,844	0,32	0,165611118	1,420999369	0,70373008	2,6375
GALR1	1,83586E-09	0,506427208	0,438	0,066	3,87385E-05	1,42052794	0,703963626	6,636363636
ADARB1	2,4996E-17	0,505198971	0,438	0,023	5,2744E-13	1,41931909	0,7045632	19,04347826
MCAM	0,000179067	0,505051899	0,812	0,41	1	1,419174408	0,704635029	1,980487805
MRPL40	3,72671E-08	0,504686057	0,5	0,102	0,000786374	1,418814576	0,704813735	4,901960784
RRAS	5,11302E-05	0,504370645	0,656	0,234	1	1,418504419	0,704967843	2,803418803
NAPRT	3,22509E-12	0,504148523	0,5	0,062	6,80527E-08	1,418286038	0,70507639	8,064516129

Supplementary Table 1

Differentially expressed genes_adjacent-skin-XL_ comparing SC-Keloid vs. (SC-Myel, SC-Nonmyel)								
**gene**	**p_val**	**avg_log2FC**	**pct.1**	**pct.2**	**p_val_adj**	**Foldchange_UP**	**Foldchange_DOWN**	**Ratio_pct1_pct2**
MFSD12	4,92965E-07	0,50413729	0,656	0,184	0,010402047	1,418274995	0,70508188	3,565217391
CCT7	2,67114E-07	0,503759178	0,562	0,137	0,005636372	1,417903332	0,705266697	4,102189781
SMARCE1	4,37306E-08	0,503567712	0,688	0,176	0,000922759	1,417715169	0,705360302	3,909090909
GPR17	1,46005E-13	0,503431436	0,438	0,039	3,08084E-09	1,417581259	0,705426933	11,23076923
PXDN	9,22226E-13	0,503402029	0,5	0,059	1,94599E-08	1,417552364	0,705441313	8,474576271
GRSF1	2,35926E-10	0,503378026	0,406	0,051	4,97828E-06	1,41752878	0,705453049	7,960784314
COA1	5,86215E-13	0,503324937	0,438	0,043	1,23697E-08	1,417476617	0,705479009	10,18604651
PPP1R7	4,34639E-11	0,503128389	0,531	0,082	9,17131E-07	1,417283518	0,705575128	6,475609756
TGFB1	2,38844E-08	0,502837	0,625	0,148	0,000503985	1,416997291	0,705717651	4,222972973
M6PR	7,74708E-11	0,502640209	0,625	0,113	1,63471E-06	1,416804019	0,705813921	5,530973451
LDHB	7,17001E-06	0,502214907	0,688	0,25	0,151294484	1,416386411	0,706022024	2,752
PRKCSH	2,71895E-07	0,501891951	0,562	0,137	0,00573726	1,416069379	0,706180089	4,102189781
MTHFD1L	5,75596E-16	0,501873523	0,25	0	1,21456E-11	1,416051292	0,706189109	#DIV/0!
ARHGDIA	6,41032E-05	0,501674673	0,812	0,367	1	1,415856127	0,706286452	2,21253406
TRMT112	0,001315498	0,501041581	0,75	0,375	1	1,415234949	0,706596456	2
PRKRA	7,25359E-08	0,500588188	0,469	0,094	0,001530579	1,414790256	0,706818552	4,989361702
GUSB	2,59159E-09	0,500463692	0,594	0,121	5,46852E-05	1,414668174	0,706879549	4,909090909
ERCC1	1,40401E-12	0,499560342	0,562	0,078	2,9626E-08	1,41378265	0,707322303	7,205128205
COX16	6,22092E-07	0,499299349	0,656	0,191	0,013126757	1,41352691	0,707450274	3,434554974
MESD	7,32547E-14	0,498946006	0,594	0,078	1,54575E-09	1,413180754	0,707623563	7,615384615
PGLS	5,46318E-09	0,498745128	0,562	0,117	0,000115279	1,412983999	0,707722098	4,803418803
NDUFA9	5,57435E-10	0,498305088	0,438	0,062	1,17624E-05	1,412553087	0,707937995	7,064516129
TUBA1A	0,003477473	0,498032743	1	0,664	1	1,412286457	0,708071649	1,506024096
SERPINB8	2,00549E-07	0,497254275	0,312	0,047	0,00423179	1,411524602	0,708453822	6,638297872
HIKESHI	7,82443E-10	0,496010639	0,625	0,125	1,65103E-05	1,41030836	0,709064789	5
RSPO3	3,29263E-08	0,49583053	0,25	0,023	0,000694779	1,410132306	0,709153315	10,86956522
SNRPG	4,91975E-05	0,495028288	0,719	0,289	1	1,409348389	0,709547766	2,487889273
NHP2	2,14619E-09	0,494916447	0,625	0,133	4,52867E-05	1,409239138	0,709602773	4,69924812
MCUR1	2,87606E-09	0,493578727	0,594	0,121	6,06878E-05	1,407933045	0,710261048	4,909090909
GADD45GIP1	2,72486E-06	0,493140544	0,625	0,184	0,057497254	1,407505485	0,710476805	3,39673913

Supplementary Table 1

Differentially expressed genes_adjacent-skin-XL_ comparing SC-Keloid vs. (SC-Myel, SC-Nonmyel)								
**gene**	**p_val**	**avg_log2FC**	**pct.1**	**pct.2**	**p_val_adj**	**Foldchange_UP**	**Foldchange_DOWN**	**Ratio_pct1_pct2**
SIGIRR	3,77936E-08	0,492784454	0,625	0,148	0,000797483	1,407158123	0,710652189	4,222972973
TXNL1	6,01004E-06	0,492295022	0,594	0,18	0,12681786	1,406680828	0,710893317	3,3
TBCB	0,000488155	0,491763147	0,812	0,344	1	1,406162326	0,711155449	2,360465116
DGUOK	3,03045E-09	0,491266197	0,656	0,145	6,39455E-05	1,405678043	0,711400456	4,524137931
EIF2AK4	3,71174E-12	0,490619184	0,469	0,055	7,83214E-08	1,405047772	0,711719573	8,527272727
GNG11	1,6649E-09	0,490503387	0,719	0,168	3,51312E-05	1,404935002	0,711776701	4,279761905
EXTL2	1,35367E-09	0,490081903	0,344	0,039	2,85638E-05	1,404524609	0,711984677	8,820512821
UFD1	6,11375E-06	0,48943738	0,438	0,105	0,129006141	1,403897279	0,712302826	4,171428571
RPL14	0,001239654	0,489408254	1	0,785	1	1,403868937	0,712317207	1,27388535
NDUFA6	1,90002E-07	0,489070857	0,656	0,172	0,004009222	1,403540658	0,712483813	3,813953488
CAPN1	5,0917E-09	0,488339982	0,625	0,137	0,00010744	1,402829799	0,712844852	4,562043796
IFNAR1	7,41583E-07	0,488265762	0,594	0,156	0,015648152	1,402757632	0,712881525	3,807692308
STUB1	8,44234E-06	0,48767491	0,656	0,211	0,178141776	1,402183254	0,713173544	3,109004739
TCEAL7	1,15834E-10	0,487199723	0,531	0,086	2,44421E-06	1,401721486	0,713408484	6,174418605
FKBP8	7,28475E-05	0,486476092	0,812	0,301	1	1,401018584	0,713766407	2,697674419
EIF3I	8,83479E-08	0,486259258	0,688	0,184	0,001864229	1,400808029	0,713873692	3,739130435
ATP5PO	1,60226E-06	0,485935687	0,781	0,254	0,033809221	1,400493888	0,71403382	3,07480315
HTATSF1	8,39107E-10	0,485709484	0,406	0,055	1,7706E-05	1,400274319	0,714145783	7,381818182
HRAS	1,48171E-06	0,485633017	0,438	0,102	0,031265647	1,400200102	0,714183636	4,294117647
VPS29	2,40518E-05	0,484734549	0,625	0,219	0,50751691	1,399328371	0,714628547	2,853881279
PXDC1	6,48449E-06	0,484590148	0,688	0,227	0,136829135	1,399188316	0,714700079	3,030837004
SURF4	1,14285E-07	0,484515465	0,625	0,16	0,002411519	1,399115888	0,714737077	3,90625
CFAP20	5,39203E-11	0,484477129	0,656	0,121	1,13777E-06	1,39907871	0,71475607	5,421487603
MRPL38	8,10967E-08	0,483420503	0,562	0,129	0,001711221	1,398054404	0,715279747	4,356589147
SLC16A3	7,24756E-09	0,48326956	0,188	0,008	0,000152931	1,397908139	0,715354587	23,5
TXNDC15	1,18104E-06	0,483082423	0,531	0,141	0,024921226	1,397726824	0,715447384	3,765957447
MFAP2	2,57203E-21	0,48291444	0,375	0,004	5,42725E-17	1,397564086	0,715530694	93,75
MYG1	6,90102E-08	0,482795977	0,375	0,062	0,001456185	1,397449333	0,71558945	6,048387097
TCEAL4	3,55263E-06	0,482648966	0,844	0,305	0,07496412	1,39730694	0,715662373	2,767213115
RPL39	0,001287941	0,482458801	0,969	0,855	1	1,39712277	0,715756712	1,133333333

Supplementary Table 1

Differentially expressed genes_adjacent-skin-XL_ comparing SC-Keloid vs. (SC-Myel, SC-Nonmyel)								
**gene**	**p_val**	**avg_log2FC**	**pct.1**	**pct.2**	**p_val_adj**	**Foldchange_UP**	**Foldchange_DOWN**	**Ratio_pct1_pct2**
SSRP1	3,17659E-08	0,482457768	0,469	0,09	0,000670293	1,39712177	0,715757224	5,211111111
PDZD11	7,99979E-15	0,4822885	0,5	0,047	1,68803E-10	1,396957858	0,715841207	10,63829787
PRKACB	6,43083E-11	0,481950464	0,375	0,039	1,35697E-06	1,396630577	0,716008955	9,615384615
MRPL13	5,26874E-11	0,481938675	0,438	0,055	1,11176E-06	1,396619164	0,716014806	7,963636364
ATP6V1G1	2,7465E-05	0,481894	0,844	0,324	0,579538626	1,396575917	0,716036979	2,604938272
MRPL17	4,08383E-09	0,479932749	0,344	0,043	8,6173E-05	1,394678653	0,717011046	8
MINPP1	1,02526E-12	0,479750905	0,281	0,012	2,1634E-08	1,394502871	0,717101428	23,41666667
VBP1	2,0123E-06	0,479613944	0,531	0,141	0,042461523	1,394370491	0,717169508	3,765957447
SSR1	1,71068E-08	0,479275943	0,625	0,145	0,000360971	1,394043851	0,71733755	4,310344828
YIPF3	1,28902E-08	0,478970817	0,625	0,145	0,000271995	1,393749045	0,717489281	4,310344828
BAD	1,91275E-08	0,47861594	0,594	0,129	0,00040361	1,393406251	0,717665792	4,604651163
TLE5	4,40757E-05	0,478580681	0,906	0,41	0,930041277	1,393372197	0,717683331	2,209756098
SF3B5	1,20569E-07	0,478454806	0,656	0,18	0,002544125	1,39325063	0,717745952	3,644444444
TLN1	0,000177088	0,478407757	0,812	0,352	1	1,393205194	0,71776936	2,306818182
NIPSNAP3A	9,78982E-12	0,477668556	0,344	0,027	2,06575E-07	1,392491533	0,718137221	12,74074074
CES1	2,48095E-09	0,477362602	0,219	0,012	5,23505E-05	1,392196258	0,718289533	18,25
TALDO1	0,000195558	0,477334207	0,75	0,32	1	1,392168857	0,71830367	2,34375
DNAJC1	1,08432E-07	0,477285233	0,531	0,125	0,002288015	1,392121599	0,718328055	4,248
POP7	3,85602E-12	0,477111257	0,406	0,039	8,13659E-08	1,391953732	0,718414684	10,41025641
EIF3K	9,73766E-05	0,477041305	0,781	0,312	1	1,391886241	0,718449519	2,503205128
B3GAT3	5,10815E-10	0,476237719	0,594	0,109	1,07787E-05	1,391111172	0,718849809	5,449541284
DPH3	8,26184E-07	0,475797428	0,406	0,086	0,0174333	1,390686688	0,719069226	4,720930233
FKBP10	4,45123E-09	0,475697289	0,531	0,102	9,39255E-05	1,390590163	0,719119138	5,205882353
PSMA2	3,24863E-05	0,475507574	0,688	0,254	0,685493179	1,390407312	0,719213709	2,708661417
LAMTOR4	0,00019156	0,475269673	0,75	0,32	1	1,390178052	0,719332318	2,34375
PPP4C	3,39249E-09	0,474914039	0,562	0,109	7,15849E-05	1,389835406	0,71950966	5,155963303
ZNF787	0,000691403	0,473776507	0,312	0,094	1	1,388739985	0,7200772	3,319148936
ANP32A	1,18792E-09	0,47376427	0,688	0,148	2,50662E-05	1,388728205	0,720083308	4,648648649
SNRPB	0,001554573	0,473723568	0,719	0,375	1	1,388689027	0,720103623	1,917333333
CDK6	1,2292E-07	0,473697824	0,5	0,105	0,002593744	1,388664247	0,720116473	4,761904762

Supplementary Table 1

Differentially expressed genes_adjacent-skin-XL_ comparing SC-Keloid vs. (SC-Myel, SC-Nonmyel)								
**gene**	**p_val**	**avg_log2FC**	**pct.1**	**pct.2**	**p_val_adj**	**Foldchange_UP**	**Foldchange_DOWN**	**Ratio_pct1_pct2**
RUNX1	1,99622E-09	0,472971778	0,625	0,129	4,21223E-05	1,387965567	0,720478969	4,84496124
TATDN1	3,78582E-13	0,47173454	0,5	0,059	7,98845E-09	1,386775775	0,721097108	8,474576271
GNB5	2,37538E-09	0,471606333	0,219	0,012	5,0123E-05	1,386652543	0,721161192	18,25
AKR1A1	2,49974E-06	0,471535769	0,469	0,117	0,052746924	1,386584722	0,721196465	4,008547009
SLITRK6	2,14689E-09	0,470590671	0,688	0,152	4,53016E-05	1,385676678	0,72166907	4,526315789
AGAP2-AS1	5,01789E-11	0,469911662	0,312	0,023	1,05883E-06	1,385024658	0,722008806	13,56521739
KLF2	5,21873E-06	0,469629364	0,781	0,297	0,110120397	1,384753671	0,722150098	2,62962963
SNRPE	7,51796E-06	0,469227093	0,625	0,203	0,158636542	1,384367611	0,722351485	3,078817734
COMMD1	1,41896E-08	0,468890675	0,5	0,098	0,000299415	1,384044831	0,722519948	5,102040816
PKN1	1,49638E-09	0,468807251	0,625	0,129	3,1575E-05	1,383964801	0,722561729	4,84496124
PDGFA	0,015885062	0,468779111	0,781	0,402	1	1,383937807	0,722575823	1,94278607
ABHD12	2,50863E-07	0,468636576	0,5	0,113	0,005293468	1,383801084	0,722647216	4,424778761
HBEGF	2,45176E-05	0,468624412	0,812	0,34	0,517346134	1,383789416	0,722653308	2,388235294
LINC00623	1,14679E-10	0,468124119	0,594	0,105	2,41985E-06	1,383309633	0,722903951	5,657142857
TMEM134	1,16828E-07	0,467921637	0,406	0,074	0,002465178	1,3831155	0,723005418	5,486486486
STK17A	1,80278E-09	0,467331806	0,688	0,152	3,80406E-05	1,382550143	0,723301072	4,526315789
RAB32	8,93296E-09	0,466904033	0,5	0,094	0,000188494	1,382140264	0,723515569	5,319148936
ST6GAL1	1,46247E-13	0,466515628	0,531	0,062	3,08595E-09	1,381768212	0,723710382	8,564516129
MRPL41	3,1655E-07	0,465889346	0,75	0,227	0,006679516	1,381168509	0,724024617	3,303964758
RPS17	0,002655389	0,465317426	0,812	0,48	1	1,380621088	0,724311695	1,691666667
THOC7	3,25369E-06	0,465280105	0,5	0,137	0,068656094	1,380585374	0,724330432	3,649635036
VCAN	1,22029E-11	0,464905574	0,406	0,043	2,57494E-07	1,380227013	0,724518496	9,441860465
GNB2	0,000151709	0,464664077	0,844	0,383	1	1,379995992	0,724639786	2,203655352
SLC66A2	3,09295E-09	0,464477553	0,375	0,051	6,52643E-05	1,379817585	0,72473348	7,352941176
NDUFB8	0,000102612	0,464372135	0,781	0,324	1	1,379716766	0,724786438	2,410493827
EIF4EBP1	2,89649E-10	0,463970973	0,406	0,051	6,11189E-06	1,37933317	0,724988003	7,960784314
PIH1D1	1,114E-08	0,463632168	0,5	0,094	0,000235065	1,379009282	0,725158281	5,319148936
STMN3	1,58874E-13	0,463495236	0,469	0,047	3,3524E-09	1,378878402	0,725227111	9,978723404
SND1	8,10967E-08	0,463136505	0,562	0,129	0,001711221	1,378535581	0,725407464	4,356589147
MXRA7	1,18982E-07	0,462993243	0,688	0,184	0,002510634	1,378398697	0,725479502	3,739130435

Supplementary Table 1

Differentially expressed genes_adjacent-skin-XL_ comparing SC-Keloid vs. (SC-Myel, SC-Nonmyel)								
**gene**	**p_val**	**avg_log2FC**	**pct.1**	**pct.2**	**p_val_adj**	**Foldchange_UP**	**Foldchange_DOWN**	**Ratio_pct1_pct2**
YWHAQ	0,000284942	0,462630785	0,875	0,477	1	1,378052436	0,725661792	1,834381551
WDR83OS	8,29222E-05	0,462551801	0,906	0,41	1	1,377976993	0,725701521	2,209756098
HDLBP	1,73491E-07	0,462549558	0,656	0,168	0,003660827	1,377974851	0,72570265	3,904761905
LRPAP1	0,000375834	0,462432688	0,562	0,23	1	1,377863228	0,72576144	2,443478261
UTP14A	1,53958E-07	0,461974113	0,219	0,02	0,003248661	1,37742533	0,725992167	10,95
RPS14	0,001715062	0,461931192	1	0,863	1	1,377384352	0,726013766	1,158748552
WDR1	2,01079E-06	0,461495249	0,688	0,227	0,04242964	1,376968206	0,726233181	3,030837004
BCHE	2,54624E-06	0,461456987	0,25	0,035	0,053728244	1,376931689	0,726252441	7,142857143
SIGMAR1	1,69856E-13	0,461255777	0,469	0,047	3,58412E-09	1,376739664	0,726353737	9,978723404
EBP	2,65494E-12	0,461123304	0,438	0,047	5,60218E-08	1,376613253	0,726420436	9,319148936
CHCHD1	1,69015E-15	0,460760746	0,531	0,051	3,56638E-11	1,376267344	0,726603014	10,41176471
NMD3	1,80167E-08	0,46057335	0,344	0,047	0,00038017	1,376088589	0,7266974	7,319148936
IFI27L1	3,11952E-12	0,460253334	0,438	0,047	6,58251E-08	1,375783381	0,726858613	9,319148936
TRAPPC2L	3,32103E-12	0,460118851	0,5	0,062	7,00771E-08	1,375655141	0,726926371	8,064516129
ECHS1	1,61119E-07	0,459932112	0,625	0,16	0,003399778	1,375477091	0,727020469	3,90625
STK32B	1,0613E-19	0,459536661	0,312	0	2,23945E-15	1,375100117	0,727219777	#DIV/0!
SELENOF	8,38965E-05	0,459172949	0,75	0,32	1	1,374753489	0,727403136	2,34375
AGPAT2	1,97842E-08	0,458728461	0,406	0,066	0,000417466	1,374329999	0,727627281	6,151515152
TTR	0,082317662	0,458632455	0,031	0,004	1	1,374238546	0,727675703	7,75
RGMB	1,93978E-10	0,457330352	0,656	0,133	4,09314E-06	1,372998788	0,728332762	4,932330827
NID1	4,16806E-09	0,456860804	0,531	0,102	8,79503E-05	1,372551997	0,728569848	5,205882353
CHST14	8,61967E-11	0,45677379	0,375	0,039	1,81884E-06	1,372469215	0,728613792	9,615384615
BCS1L	1,82463E-09	0,456772445	0,312	0,031	3,85015E-05	1,372467936	0,728614472	10,06451613
SUMO1	6,29765E-05	0,456558075	0,625	0,23	1	1,372264016	0,728722745	2,717391304
SNAPC2	2,47651E-12	0,455795642	0,406	0,039	5,22567E-08	1,371538996	0,72910796	10,41025641
UBE2A	1,35507E-08	0,45543938	0,688	0,164	0,000285933	1,371200348	0,72928803	4,195121951
JAG1	2,72578E-08	0,455349493	0,562	0,125	0,000575167	1,371114918	0,72933347	4,496
RPL27	0,000642574	0,455286932	0,969	0,688	1	1,371055462	0,729365097	1,408430233
PGP	3,5898E-06	0,455236329	0,5	0,133	0,075748431	1,371007373	0,72939068	3,759398496
PAIP1	1,18032E-07	0,455016527	0,531	0,121	0,002490602	1,370798508	0,729501815	4,388429752

Supplementary Table 1

Differentially expressed genes_adjacent-skin-XL_ comparing SC-Keloid vs. (SC-Myel, SC-Nonmyel)								
**gene**	**p_val**	**avg_log2FC**	**pct.1**	**pct.2**	**p_val_adj**	**Foldchange_UP**	**Foldchange_DOWN**	**Ratio_pct1_pct2**
ORAI1	2,35856E-09	0,454917907	0,531	0,098	4,97681E-05	1,370704806	0,729551684	5,418367347
ABRACL	2,41715E-14	0,454714724	0,312	0,012	5,10043E-10	1,370511776	0,729654438	26
RPS13	0,003625039	0,454661577	1	0,82	1	1,370461289	0,729681319	1,219512195
CERCAM	1,26742E-13	0,454615188	0,438	0,039	2,67437E-09	1,370417223	0,729704781	11,23076923
IFI44L	1,51026E-15	0,454324042	0,5	0,043	3,1868E-11	1,370140691	0,729852056	11,62790698
GPX7	2,48769E-14	0,454169652	0,375	0,023	5,24927E-10	1,369994073	0,729930165	16,30434783
CHMP5	2,23283E-06	0,453836836	0,781	0,262	0,047114951	1,369678066	0,730098572	2,980916031
ITGA3	3,49126E-10	0,453412392	0,594	0,109	7,3669E-06	1,369275163	0,7303134	5,449541284
SLC31A2	4,03999E-12	0,451900956	0,219	0,004	8,52479E-08	1,367841395	0,731078913	54,75
SEC13	3,06776E-10	0,449900231	0,5	0,078	6,47329E-06	1,365945792	0,732093474	6,41025641
RAB23	4,26111E-13	0,449707206	0,312	0,016	8,99136E-09	1,365763048	0,732191431	19,5
MSC	5,75596E-16	0,44781706	0,25	0	1,21456E-11	1,363974866	0,733151339	#DIV/0!
PAFAH1B3	8,75235E-12	0,447342393	0,344	0,027	1,84683E-07	1,363526173	0,733392596	12,74074074
SUPT4H1	1,24274E-08	0,447176123	0,562	0,121	0,00026223	1,363369036	0,733477124	4,644628099
PTPMT1	1,21509E-13	0,446942058	0,469	0,047	2,56395E-09	1,363147859	0,733596135	9,978723404
CRELD1	4,26642E-06	0,446871484	0,375	0,082	0,090025755	1,363081178	0,733632022	4,573170732
GARS1	2,44211E-08	0,446624187	0,469	0,09	0,000515309	1,362847548	0,733757786	5,211111111
PLN	2,86276E-12	0,446182098	0,188	0	6,04072E-08	1,362429991	0,733982668	#DIV/0!
ATP6V1F	5,32424E-05	0,446162342	0,625	0,227	1	1,362411334	0,733992719	2,753303965
RPN2	9,28061E-05	0,445601308	0,656	0,258	1	1,361881623	0,734278209	2,542635659
WDR61	4,65033E-09	0,445163437	0,531	0,102	9,81265E-05	1,361468343	0,734501103	5,205882353
RPL41	5,18822E-05	0,44505044	1	0,953	1	1,361361712	0,734558634	1,049317943
HNRNPA1L2	0,001203934	0,444706392	0,125	0,02	1	1,361037099	0,73473383	6,25
NOPCHAP1	7,60701E-08	0,444535525	0,375	0,062	0,001605155	1,360875913	0,734820854	6,048387097
C20orf27	5,8259E-11	0,443954802	0,344	0,031	1,22932E-06	1,360328234	0,735116698	11,09677419
UBE2L3	2,74095E-05	0,443821874	0,625	0,215	0,578368189	1,360202901	0,735184434	2,906976744
XRCC6	2,08791E-05	0,443659073	0,688	0,242	0,440570765	1,360049418	0,7352674	2,842975207
SMUG1	6,60693E-14	0,443339103	0,25	0,004	1,39413E-09	1,359747811	0,735430491	62,5
KIF9	1,68712E-11	0,443334422	0,406	0,043	3,55998E-07	1,359743399	0,735432877	9,441860465
NDUFA3	5,66591E-08	0,443137335	0,625	0,152	0,001195564	1,359557656	0,735533352	4,111842105

Supplementary Table 1

Differentially expressed genes_adjacent-skin-XL_ comparing SC-Keloid vs. (SC-Myel, SC-Nonmyel)								
**gene**	**p_val**	**avg_log2FC**	**pct.1**	**pct.2**	**p_val_adj**	**Foldchange_UP**	**Foldchange_DOWN**	**Ratio_pct1_pct2**
RCN1	6,22734E-06	0,442814709	0,688	0,219	0,131403066	1,359253656	0,735697855	3,141552511
HOXC8	4,55359E-09	0,442478821	0,312	0,035	9,60852E-05	1,358937232	0,73586916	8,914285714
BPNT2	5,70593E-10	0,442424837	0,594	0,113	1,20401E-05	1,358886384	0,735896696	5,256637168
NENF	5,77844E-06	0,44236689	0,781	0,266	0,121930956	1,358831804	0,735926255	2,936090226
PGGT1B	2,67442E-07	0,44232118	0,312	0,047	0,005643297	1,358788751	0,735949572	6,638297872
NDUFA8	1,51075E-10	0,442158539	0,469	0,066	3,18783E-06	1,358635578	0,736032544	7,106060606
PEPD	1,35762E-07	0,442077318	0,625	0,16	0,002864715	1,358559092	0,736073982	3,90625
CMYA5	1,4652E-10	0,44203044	0,5	0,078	3,09171E-06	1,358514948	0,7360979	6,41025641
PSENEN	4,52463E-10	0,441901578	0,531	0,09	9,54743E-06	1,35839361	0,736163651	5,9
KCNMB1	7,90851E-18	0,441653989	0,281	0	1,66877E-13	1,358160509	0,736289999	#DIV/0!
VARS1	1,64952E-07	0,440938836	0,344	0,055	0,003480651	1,357487427	0,736655073	6,254545455
PCBP4	2,82247E-06	0,440890586	0,75	0,25	0,059557011	1,357442028	0,736679711	3
TMEM208	5,52128E-08	0,440782027	0,5	0,102	0,001165045	1,357339887	0,736735146	4,901960784
SMIM20	3,35513E-10	0,440584032	0,375	0,043	7,07967E-06	1,357153619	0,736836262	8,720930233
SLC25A11	2,63556E-10	0,440360955	0,5	0,078	5,5613E-06	1,356943786	0,736950204	6,41025641
POLR2E	2,87422E-08	0,440223925	0,688	0,172	0,000606489	1,356814906	0,737020205	4
KPNA3	9,35701E-06	0,439598319	0,312	0,062	0,19744232	1,356226668	0,737339873	5,032258065
WDR81	8,91574E-05	0,439503087	0,156	0,02	1	1,356137148	0,737388546	7,8
ATRAID	2,58943E-05	0,438935333	0,75	0,281	0,546394719	1,355603562	0,737678793	2,669039146
SLBP	2,28444E-07	0,438690932	0,469	0,102	0,004820392	1,355373934	0,737803771	4,598039216
SLC25A6	0,001434869	0,437809253	0,719	0,355	1	1,354545873	0,738254806	2,025352113
FAM207A	4,87019E-13	0,437319768	0,312	0,016	1,02766E-08	1,354086374	0,738505327	19,5
RANBP1	1,62618E-05	0,437119523	0,656	0,219	0,343140682	1,353898441	0,738607838	2,99543379
BOLA3	1,41687E-09	0,436844632	0,438	0,066	2,98974E-05	1,353640494	0,738748586	6,636363636
CCDC124	1,17076E-08	0,436824293	0,531	0,105	0,000247043	1,35362141	0,738759001	5,057142857
STIM1	4,42467E-07	0,436710955	0,344	0,059	0,00933649	1,353515074	0,73881704	5,830508475
NDUFV1	3,02366E-07	0,436603423	0,562	0,137	0,006380227	1,353414193	0,73887211	4,102189781
ADAMTS2	1,40767E-13	0,43616801	0,344	0,02	2,97033E-09	1,353005787	0,739095139	17,2
IBSP	9,12354E-07	0,436058109	0,094	0	0,019251588	1,352902722	0,739151443	#DIV/0!
ANKRD10	0,002136908	0,435776686	0,844	0,465	1	1,352638841	0,739295642	1,815053763

Supplementary Table 1

Differentially expressed genes_adjacent-skin-XL_ comparing SC-Keloid vs. (SC-Myel, SC-Nonmyel)								
**gene**	**p_val**	**avg_log2FC**	**pct.1**	**pct.2**	**p_val_adj**	**Foldchange_UP**	**Foldchange_DOWN**	**Ratio_pct1_pct2**
PLGRKT	2,11379E-11	0,435492436	0,406	0,043	4,46031E-07	1,35237236	0,739441318	9,441860465
CHPF	1,23735E-12	0,435420461	0,625	0,094	2,61094E-08	1,352304894	0,739478208	6,64893617
RPL23A	9,16318E-05	0,43523722	1	0,852	1	1,352133144	0,739572138	1,17370892
TOMM22	2,96249E-09	0,434757287	0,5	0,09	6,25116E-05	1,351683412	0,739818208	5,555555556
MACF1	5,01042E-08	0,43468207	0,719	0,184	0,001057249	1,351612942	0,739856781	3,907608696
TPT1	5,59574E-05	0,434136829	1	0,934	1	1,35110222	0,740136449	1,070663812
DHX34	8,61561E-07	0,433867563	0,125	0,004	0,018179807	1,350850073	0,740274602	31,25
HYAL2	3,14623E-10	0,432495478	0,5	0,078	6,63886E-06	1,349565948	0,74097898	6,41025641
UBE2F	1,18068E-09	0,431978038	0,438	0,066	2,49135E-05	1,349081997	0,741244789	6,636363636
SET	0,003039772	0,431802841	0,906	0,555	1	1,348918178	0,741334809	1,632432432
GTF3C6	1,12991E-10	0,431524694	0,5	0,074	2,38422E-06	1,348658136	0,74147775	6,756756757
RBMX2	5,11613E-11	0,431446717	0,438	0,055	1,07955E-06	1,348585244	0,741517827	7,963636364
ANO6	2,56914E-12	0,431196869	0,469	0,055	5,42114E-08	1,348351714	0,741646255	8,527272727
MAML2	5,57293E-08	0,431169845	0,562	0,125	0,001175944	1,348326458	0,741660148	4,496
CAMLG	8,12833E-07	0,430692986	0,656	0,184	0,017151593	1,347880865	0,741905332	3,565217391
COPS8	1,51032E-13	0,430614309	0,75	0,125	3,18692E-09	1,34780736	0,741945793	6
HM13	3,64583E-06	0,430588803	0,594	0,18	0,076930657	1,347783532	0,74195891	3,3
RPL9	0,001883592	0,43058443	1	0,84	1	1,347779447	0,741961159	1,19047619
EMC6	6,97568E-07	0,430340628	0,375	0,07	0,014719382	1,347551704	0,742086554	5,357142857
PYM1	3,02825E-10	0,430153931	0,312	0,027	6,38991E-06	1,34737733	0,742182592	11,55555556
MBTPS1	3,80935E-07	0,429875053	0,5	0,117	0,008038106	1,347116902	0,742326073	4,273504274
UBE2D2	1,12489E-05	0,429799824	0,719	0,266	0,237362104	1,347046659	0,742364782	2,703007519
ADAM9	1,75885E-09	0,429689649	0,531	0,098	3,71135E-05	1,346943793	0,742421477	5,418367347
MICU1	1,81491E-09	0,429635376	0,594	0,121	3,82964E-05	1,346893123	0,742449407	4,909090909
HDHD5	1,7341E-09	0,429511549	0,344	0,039	3,65911E-05	1,346777524	0,742513134	8,820512821
MAP2K2	2,16335E-06	0,429499462	0,719	0,227	0,04564883	1,34676624	0,742519355	3,167400881
CSRP1	0,006832456	0,429397544	0,812	0,523	1	1,346671102	0,742571812	1,552581262
UBE2G1	0,001524635	0,429324422	0,344	0,117	1	1,346602848	0,74260945	2,94017094
RALA	2,09869E-06	0,428542913	0,469	0,113	0,044284464	1,34587359	0,74301183	4,150442478
HDAC9	1,57978E-10	0,428502273	0,219	0,008	3,33348E-06	1,345835678	0,743032761	27,375

Supplementary Table 1

Differentially expressed genes_adjacent-skin-XL_ comparing SC-Keloid vs. (SC-Myel, SC-Nonmyel)								
**gene**	**p_val**	**avg_log2FC**	**pct.1**	**pct.2**	**p_val_adj**	**Foldchange_UP**	**Foldchange_DOWN**	**Ratio_pct1_pct2**
SNRPC	4,00195E-08	0,428178561	0,469	0,094	0,000844451	1,345533734	0,743199501	4,989361702
PTOV1	3,56047E-07	0,427895211	0,594	0,152	0,00751295	1,345269492	0,743345483	3,907894737
DSTN	0,004882178	0,427882392	0,781	0,402	1	1,345257539	0,743352087	1,94278607
SCPEP1	1,81114E-10	0,427666773	0,531	0,086	3,82169E-06	1,345056498	0,743463194	6,174418605
DNAJC4	6,72404E-05	0,427272485	0,312	0,074	1	1,344688944	0,74366641	4,216216216
VAT1	6,74059E-06	0,427097298	0,594	0,176	0,14223328	1,344525669	0,743756719	3,375
CYB5B	3,97904E-08	0,426848547	0,375	0,059	0,000839617	1,344293864	0,74388497	6,355932203
FRG1	2,62903E-07	0,426071598	0,438	0,09	0,005547524	1,343570103	0,744285689	4,866666667
ELP5	1,97839E-08	0,426068179	0,406	0,066	0,00041746	1,343566918	0,744287453	6,151515152
SRPX2	6,60272E-18	0,425584239	0,375	0,012	1,39324E-13	1,343116306	0,74453716	31,25
DEF8	5,11383E-11	0,425284379	0,375	0,039	1,07907E-06	1,342837172	0,744691926	9,615384615
ARPC4	6,06871E-07	0,424270164	0,625	0,172	0,012805582	1,341893489	0,745215629	3,63372093
TSR3	4,0351E-08	0,423886737	0,469	0,09	0,000851447	1,341536899	0,745413712	5,211111111
GRHPR	1,43763E-07	0,423809851	0,688	0,18	0,003033542	1,341465406	0,745453439	3,822222222
TIMP2	0,000284519	0,423789767	0,906	0,504	1	1,341446732	0,745463816	1,797619048
AMACR	1,5116E-08	0,423668848	0,156	0,004	0,000318962	1,341334303	0,7455263	39
NDUFB11	1,41859E-05	0,423641453	0,719	0,25	0,299336733	1,341308833	0,745540457	2,876
EIF2A	2,65579E-07	0,423325821	0,5	0,113	0,005603984	1,341015415	0,745703583	4,424778761
CYBC1	1,05106E-06	0,423192063	0,344	0,062	0,022178476	1,34089109	0,745772724	5,548387097
CCT6A	2,0813E-07	0,423173179	0,562	0,137	0,004391741	1,340873539	0,745782485	4,102189781
TMEM179B	5,90187E-07	0,422871447	0,562	0,141	0,012453539	1,340593131	0,745938478	3,985815603
C14orf119	3,25367E-12	0,422651579	0,406	0,039	6,86557E-08	1,340388839	0,746052168	10,41025641
SYAP1	7,03788E-10	0,422574602	0,625	0,125	1,48506E-05	1,340317323	0,746091976	5
SOCS4	7,55883E-05	0,422570231	0,281	0,062	1	1,340313262	0,746094237	4,532258065
EEF1D	0,001414457	0,42239561	0,906	0,566	1	1,340151043	0,746184548	1,600706714
AARSD1	7,90453E-06	0,421835175	0,312	0,062	0,166793541	1,339630543	0,74647447	5,032258065
CENPB	1,89251E-05	0,421703396	0,375	0,094	0,39933877	1,339508184	0,746542658	3,989361702
GLRX5	1,95411E-12	0,421533307	0,562	0,078	4,12337E-08	1,33935027	0,746630678	7,205128205
GLDC	9,87864E-16	0,420729492	0,281	0,004	2,08449E-11	1,338604243	0,747046788	70,25
GLIPR1	6,60693E-14	0,41983747	0,25	0,004	1,39413E-09	1,337776836	0,747508832	62,5

Supplementary Table 1

Differentially expressed genes_adjacent-skin-XL_ comparing SC-Keloid vs. (SC-Myel, SC-Nonmyel)								
**gene**	**p_val**	**avg_log2FC**	**pct.1**	**pct.2**	**p_val_adj**	**Foldchange_UP**	**Foldchange_DOWN**	**Ratio_pct1_pct2**
EIF3D	6,20904E-05	0,419573236	0,5	0,16	1	1,337531841	0,747645753	3,125
COL22A1	1,0613E-19	0,419514382	0,312	0	2,23945E-15	1,337477278	0,747676253	#DIV/0!
ACTR6	1,69889E-09	0,419294071	0,438	0,066	3,58483E-05	1,33727305	0,747790438	6,636363636
JPT1	5,91617E-12	0,419144366	0,438	0,047	1,24837E-07	1,337134292	0,747868038	9,319148936
DNM3	2,22625E-14	0,419001675	0,438	0,035	4,69761E-10	1,337002048	0,747942011	12,51428571
NDUFB1	4,84283E-07	0,418454138	0,781	0,238	0,010218851	1,33649472	0,748225926	3,281512605
CETN2	2,62558E-12	0,418403654	0,5	0,062	5,54024E-08	1,336447953	0,748252109	8,064516129
MFGE8	0,000290483	0,418334273	0,812	0,359	1	1,336383683	0,748288095	2,26183844
TMED1	2,39522E-09	0,417935383	0,469	0,078	5,05415E-05	1,336014238	0,748495017	6,012820513
NAV1	1,80143E-06	0,417400473	0,312	0,055	0,038011942	1,335518974	0,748772589	5,672727273
RPS19BP1	1,2443E-07	0,417353342	0,719	0,195	0,002625599	1,335475345	0,748797051	3,687179487
TMEM54	7,90851E-18	0,417351475	0,281	0	1,66877E-13	1,335473617	0,74879802	#DIV/0!
C3orf70	8,89803E-11	0,417246264	0,531	0,082	1,87757E-06	1,335376229	0,748852629	6,475609756
ZCCHC17	9,26114E-05	0,417203423	0,406	0,117	1	1,335336575	0,748874867	3,47008547
EDEM2	3,25118E-10	0,417173437	0,312	0,027	6,86031E-06	1,335308821	0,748890432	11,55555556
MVB12A	1,76693E-10	0,41701713	0,531	0,086	3,72841E-06	1,335164157	0,748971574	6,174418605
ORAI2	9,77947E-13	0,416831262	0,281	0,012	2,06357E-08	1,334992153	0,749068073	23,41666667
TMEM45A	3,92384E-14	0,416792104	0,375	0,023	8,2797E-10	1,334955919	0,749088405	16,30434783
KDELR3	1,49741E-19	0,416679888	0,5	0,027	3,15968E-15	1,334852087	0,749146673	18,51851852
CCDC28B	9,16227E-15	0,416580579	0,344	0,016	1,93333E-10	1,334760205	0,749198243	21,5
TUFM	3,52486E-06	0,415996848	0,75	0,238	0,074378091	1,334220255	0,749501438	3,151260504
CNPY2	0,000106433	0,41563788	0,781	0,309	1	1,333888319	0,749687951	2,527508091
PIGF	3,52702E-09	0,415205322	0,469	0,078	7,44236E-05	1,333488443	0,749912761	6,012820513
SRM	1,79257E-06	0,414325268	0,531	0,141	0,037825035	1,332675254	0,750370352	3,765957447
LAPTM4B	2,97506E-08	0,412919735	0,469	0,09	0,000627768	1,331377539	0,75110175	5,211111111
POP5	1,7026E-11	0,412852626	0,438	0,051	3,59265E-07	1,33131561	0,75113669	8,588235294
TMEM167A	1,22694E-07	0,412625813	0,594	0,141	0,002588969	1,331106323	0,751254789	4,212765957
RPL24	0,001762604	0,41255992	1	0,773	1	1,331045529	0,751289102	1,293661061
POLR2J	1,30385E-07	0,412370114	0,656	0,168	0,002751264	1,330870423	0,751387951	3,904761905
SLC25A39	3,17659E-08	0,411851011	0,469	0,09	0,000670293	1,330391643	0,75165836	5,211111111

Supplementary Table 1

Differentially expressed genes_adjacent-skin-XL_ comparing SC-Keloid vs. (SC-Myel, SC-Nonmyel)								
**gene**	**p_val**	**avg_log2FC**	**pct.1**	**pct.2**	**p_val_adj**	**Foldchange_UP**	**Foldchange_DOWN**	**Ratio_pct1_pct2**
DNAL4	1,00455E-14	0,411743747	0,344	0,016	2,1197E-10	1,330292732	0,751714248	21,5
LSM3	4,30116E-06	0,411510952	0,625	0,184	0,090758863	1,330078092	0,751835555	3,39673913
HAX1	8,8752E-07	0,411466998	0,469	0,109	0,018727562	1,330037569	0,751858461	4,302752294
TCTN1	1,81501E-11	0,411415094	0,375	0,035	3,82984E-07	1,329989719	0,751885511	10,71428571
ADSL	2,82236E-12	0,41098867	0,375	0,031	5,95546E-08	1,329596666	0,752107782	12,09677419
ARHGAP18	2,38781E-12	0,410972342	0,25	0,008	5,03852E-08	1,329581619	0,752116294	31,25
DIMT1	1,85005E-15	0,410306404	0,438	0,031	3,90379E-11	1,328968034	0,752463546	14,12903226
DLGAP4	1,10486E-05	0,409097269	0,625	0,203	0,233135864	1,327854682	0,753094457	3,078817734
TRPT1	1,39899E-07	0,409035808	0,406	0,074	0,002951999	1,327798114	0,75312654	5,486486486
PTPRK	0,000125068	0,409010009	0,281	0,066	1	1,32777437	0,753140008	4,257575758
PLAUR	3,66209E-06	0,408778346	0,531	0,148	0,077273796	1,327561178	0,753260954	3,587837838
DDT	4,77234E-05	0,408596857	0,656	0,23	1	1,327394183	0,75335572	2,852173913
POLR3G	2,19342E-14	0,408545393	0,312	0,012	4,62834E-10	1,327346832	0,753382594	26
ABHD17A	4,78672E-07	0,408409641	0,625	0,168	0,010100448	1,32722194	0,753453488	3,720238095
DBN1	4,81619E-09	0,408335156	0,438	0,07	0,000101626	1,327153419	0,753492389	6,257142857
DOCK6	1,90127E-05	0,408193174	0,156	0,016	0,401186735	1,327022815	0,753566547	9,75
TRIAP1	4,90197E-12	0,408091699	0,312	0,02	1,03436E-07	1,326929478	0,753619553	15,6
CCNY	3,11297E-06	0,407800725	0,438	0,105	0,06568686	1,32666188	0,753771564	4,171428571
RALY	4,0704E-08	0,407749371	0,688	0,172	0,000858895	1,326614657	0,753798395	4
TMEM126A	6,39052E-07	0,407734462	0,375	0,07	0,013484639	1,326600948	0,753806185	5,357142857
ZSWIM7	4,70877E-12	0,40751394	0,562	0,082	9,93598E-08	1,326398187	0,753921416	6,853658537
DDX52	3,25329E-10	0,407382016	0,375	0,043	6,86477E-06	1,326276903	0,75399036	8,720930233
TIPRL	3,16401E-08	0,407276498	0,562	0,121	0,000667639	1,326179903	0,754045509	4,644628099
RFLNB	6,91427E-09	0,406901202	0,188	0,008	0,000145898	1,325834962	0,754241688	23,5
RNASEK	0,005300078	0,406113137	0,906	0,527	1	1,325110929	0,754653802	1,719165085
EFHC1	2,65218E-05	0,405109709	0,406	0,105	0,559636828	1,324189604	0,755178863	3,866666667
CNIH1	3,62015E-08	0,403552212	0,469	0,09	0,000763887	1,322760814	0,755994576	5,211111111
VEGFB	1,29401E-08	0,40322031	0,594	0,129	0,000273049	1,322456538	0,756168517	4,604651163
COA5	0,017863454	0,403216761	0,188	0,062	1	1,322453285	0,756170378	3,032258065
ZDHHC9	2,03294E-05	0,403136035	0,156	0,016	0,428970333	1,32237929	0,75621269	9,75

Supplementary Table 1

Differentially expressed genes_adjacent-skin-XL_ comparing SC-Keloid vs. (SC-Myel, SC-Nonmyel)								
**gene**	**p_val**	**avg_log2FC**	**pct.1**	**pct.2**	**p_val_adj**	**Foldchange_UP**	**Foldchange_DOWN**	**Ratio_pct1_pct2**
UXS1	4,17914E-06	0,402951855	0,406	0,094	0,08818403	1,32221048	0,756309237	4,319148936
PGA5	6,3574E-05	0,402856952	0,062	0	1	1,322123505	0,756358991	#DIV/0!
CD320	1,97701E-06	0,402598351	0,375	0,078	0,041716958	1,321886538	0,756494579	4,807692308
RGS2	7,11541E-06	0,402446205	0,406	0,098	0,150142189	1,32174714	0,756574362	4,142857143
RRS1	0,050929149	0,402204072	0,094	0,027	1	1,321525325	0,756701352	3,481481481
USB1	7,06114E-09	0,40134729	0,312	0,035	0,000148997	1,320740736	0,757150872	8,914285714
RAB5IF	2,29229E-08	0,400846539	0,594	0,133	0,000483695	1,320282394	0,757413721	4,466165414
BABAM1	1,84586E-06	0,400804894	0,344	0,066	0,038949426	1,320244283	0,757435584	5,212121212
ATP6AP1	3,96151E-05	0,400665506	0,594	0,191	0,835917608	1,320116731	0,757508769	3,109947644
NAALADL1	2,65168E-08	0,400339311	0,219	0,016	0,00055953	1,319818286	0,757680061	13,6875
MBD3	7,00659E-13	0,400049499	0,5	0,059	1,47846E-08	1,319553184	0,757832281	8,474576271
SLC52A2	8,70774E-15	0,39978063	0,531	0,055	1,83742E-10	1,319307288	0,757973529	9,654545455
PSMD4	2,17881E-06	0,399731396	0,562	0,152	0,045975141	1,319262265	0,757999396	3,697368421
FAM118A	3,13778E-10	0,39968457	0,312	0,027	6,62103E-06	1,319219446	0,758023999	11,55555556
MRPL16	5,983E-07	0,399607956	0,375	0,07	0,01262473	1,319149391	0,758064255	5,357142857
DBI	0,000525529	0,399297592	0,844	0,453	1	1,318865636	0,758227353	1,863134658
CBX5	0,000969397	0,399295314	0,562	0,23	1	1,318863553	0,75822855	2,443478261
TMEM59L	9,87864E-16	0,399271081	0,281	0,004	2,08449E-11	1,318841401	0,758241286	70,25
P3H1	6,28233E-11	0,399264157	0,438	0,055	1,32563E-06	1,318835071	0,758244925	7,963636364
EMC4	7,93947E-07	0,398971462	0,656	0,18	0,016753065	1,318567531	0,758398775	3,644444444
PRPS2	1,13285E-13	0,398952633	0,406	0,031	2,39044E-09	1,318550323	0,758408672	13,09677419
R3HCC1	6,96119E-08	0,398912915	0,344	0,051	0,001468881	1,318514023	0,758429552	6,745098039
OS9	5,15103E-07	0,398813009	0,719	0,207	0,010869184	1,318422719	0,758482075	3,473429952
SRP14	0,001146824	0,398143763	1	0,723	1	1,317811264	0,758834005	1,383125864
SLC20A1	6,67402E-07	0,398039846	0,438	0,094	0,014082858	1,317716345	0,758888667	4,659574468
TMEM222	2,0714E-06	0,398013898	0,312	0,055	0,043708599	1,317692646	0,758902316	5,672727273
YIPF5	2,27455E-07	0,397927747	0,438	0,09	0,004799529	1,317613962	0,758947635	4,866666667
GART	1,85851E-11	0,397869006	0,406	0,043	3,92165E-07	1,317560314	0,758978537	9,441860465
KDM1B	2,55053E-08	0,397832013	0,219	0,016	0,000538188	1,31752653	0,758997999	13,6875
MTMR2	1,02526E-12	0,3974307	0,281	0,012	2,1634E-08	1,317160086	0,759209158	23,41666667

Supplementary Table 1

Differentially expressed genes_adjacent-skin-XL_ comparing SC-Keloid vs. (SC-Myel, SC-Nonmyel)								
gene	p_val	avg_log2FC	pct.1	pct.2	p_val_adj	Foldchange_UP	Foldchange_DOWN	Ratio_pct1_pct2
SNX5	5,18672E-09	0,397288419	0,531	0,102	0,000109445	1,317030192	0,759284036	5,205882353
NDUFB9	3,51202E-06	0,397034606	0,656	0,195	0,074107031	1,316798508	0,759417628	3,364102564
DGKB	1,88354E-08	0,396944884	0,406	0,066	0,000397447	1,316716618	0,759464858	6,151515152
RUVBL2	2,54051E-11	0,396889268	0,562	0,086	5,36072E-07	1,316665859	0,759494137	6,534883721
ENO1	0,001692114	0,396775672	0,875	0,445	1	1,316562191	0,75955394	1,966292135
RASSF8	9,41179E-07	0,396298943	0,594	0,16	0,019859823	1,316127213	0,759804972	3,7125
METTL23	3,08926E-08	0,396116415	0,375	0,059	0,000651864	1,315960709	0,759901107	6,355932203
ZMPSTE24	1,98356E-11	0,396071534	0,438	0,051	4,18551E-07	1,315919772	0,759924747	8,588235294
ZFAND6	1,39132E-05	0,394930504	0,625	0,203	0,293581694	1,31487942	0,760526011	3,078817734
RPS25	0,001280136	0,394603632	1	0,82	1	1,314581541	0,760698343	1,219512195
TM9SF1	4,86095E-10	0,394525785	0,531	0,09	1,02571E-05	1,314510608	0,760739391	5,9
SMARCA5	1,06497E-06	0,393484925	0,719	0,223	0,022471923	1,313562572	0,761288439	3,224215247
YBEY	1,17927E-11	0,393372905	0,344	0,027	2,48837E-07	1,313460583	0,761347553	12,74074074
RTCB	5,45918E-06	0,393311273	0,344	0,07	0,115194137	1,313404472	0,761380078	4,914285714
UBXN6	6,72671E-07	0,393211437	0,312	0,051	0,014194039	1,313313586	0,761432769	6,117647059
PCYT1A	5,7268E-10	0,393012372	0,438	0,062	1,20841E-05	1,313132386	0,761537839	7,064516129
LEF1	2,41715E-14	0,392540779	0,312	0,012	5,10043E-10	1,312703215	0,761786814	26
RAMP1	1,46005E-13	0,392466065	0,438	0,039	3,08084E-09	1,312635235	0,761826266	11,23076923
KMT5A	7,1866E-11	0,392302473	0,406	0,047	1,51644E-06	1,3124864	0,761912657	8,638297872
DMAC1	4,67745E-11	0,391547661	0,469	0,062	9,8699E-07	1,311799891	0,762311391	7,564516129
SMC2	1,14696E-07	0,391437357	0,375	0,062	0,002420196	1,311699599	0,762369677	6,048387097
RPS16	0,004340366	0,391386841	1	0,777	1	1,311653671	0,762396372	1,287001287
RECQL	4,26297E-12	0,391294617	0,531	0,074	8,9953E-08	1,311569826	0,76244511	7,175675676
RABL6	3,46601E-08	0,39123122	0,469	0,09	0,000731362	1,311512192	0,762478615	5,211111111
PLOD2	4,36762E-14	0,390207712	0,281	0,008	9,21611E-10	1,310582081	0,763019741	35,125
MTHFD2	1,53242E-09	0,389712032	0,344	0,039	3,23356E-05	1,31013187	0,763281943	8,820512821
TADA3	2,29309E-10	0,389523915	0,594	0,105	4,83865E-06	1,309961049	0,763381477	5,657142857
CBWD2	1,59791E-09	0,389483912	0,312	0,031	3,37176E-05	1,309924727	0,763402644	10,06451613
BCAT2	5,0197E-07	0,389441094	0,281	0,039	0,010592072	1,30988585	0,763425301	7,205128205
IMPDH2	4,90138E-07	0,389070634	0,562	0,145	0,010342395	1,309549536	0,763621362	3,875862069

Supplementary Table 1

Differentially expressed genes_adjacent-skin-XL_ comparing SC-Keloid vs. (SC-Myel, SC-Nonmyel)								
**gene**	**p_val**	**avg_log2FC**	**pct.1**	**pct.2**	**p_val_adj**	**Foldchange_UP**	**Foldchange_DOWN**	**Ratio_pct1_pct2**
SDHC	9,34443E-10	0,388233711	0,5	0,082	1,97177E-05	1,308790073	0,764064475	6,097560976
SLC39A3	1,21646E-14	0,388051518	0,406	0,027	2,56684E-10	1,308624801	0,764160972	15,03703704
IDNK	0,001371418	0,387792156	0,125	0,02	1	1,308389563	0,764298362	6,25
CHMP1A	1,37632E-06	0,387604377	0,344	0,062	0,029041819	1,308219276	0,764397849	5,548387097
CBWD1	1,64926E-08	0,387145507	0,375	0,055	0,000348011	1,307803244	0,764641015	6,818181818
UBA2	3,132E-06	0,387132929	0,5	0,129	0,066088396	1,307791842	0,764647681	3,875968992
MXRA8	0,006665234	0,387049138	0,844	0,426	1	1,307715889	0,764692093	1,981220657
NUDT15	7,46552E-05	0,386714314	0,156	0,02	1	1,307412426	0,764869585	7,8
LAMTOR2	1,04095E-07	0,386285764	0,562	0,125	0,002196502	1,307024119	0,765096822	4,496
PLEKHJ1	1,86611E-11	0,386032242	0,438	0,051	3,93768E-07	1,306794458	0,765231283	8,588235294
NUDCD2	1,14172E-06	0,385787586	0,594	0,16	0,024091397	1,306572867	0,765361064	3,7125
CIB2	4,03999E-12	0,385529192	0,219	0,004	8,52479E-08	1,306338875	0,765498156	54,75
MRPL19	2,76678E-11	0,385326448	0,531	0,078	5,83819E-07	1,306155306	0,76560574	6,807692308
RBCK1	8,28595E-07	0,384980902	0,438	0,094	0,017484174	1,3058425	0,765789136	4,659574468
CNPY3	4,16485E-12	0,384771947	0,438	0,047	8,78825E-08	1,30565338	0,765900058	9,319148936
COX20	3,74796E-06	0,3829195	0,75	0,242	0,079085727	1,303977974	0,766884119	3,099173554
SMS	1,84608E-07	0,382894046	0,594	0,145	0,003895408	1,303954967	0,76689765	4,096551724
HIGD2A	4,40576E-09	0,381907261	0,625	0,129	9,29659E-05	1,303063383	0,767422378	4,84496124
GTF3A	4,39769E-11	0,381715972	0,562	0,09	9,27957E-07	1,30289062	0,767524138	6,244444444
RPS15	0,002594662	0,381362682	1	0,906	1	1,302571605	0,767712114	1,103752759
CHCHD5	2,87844E-07	0,381220915	0,406	0,078	0,006073803	1,302443613	0,767787557	5,205128205
MRPL36	8,46482E-11	0,38099372	0,5	0,074	1,78616E-06	1,302238521	0,767908477	6,756756757
LIMK1	5,97464E-09	0,380860794	0,25	0,02	0,000126071	1,302118542	0,767979234	12,5
ATP2B1	1,95035E-10	0,380744156	0,531	0,086	4,11544E-06	1,302013273	0,768041326	6,174418605
RPL18A	0,002170887	0,380714766	1	0,793	1	1,301986749	0,768056972	1,261034048
DDA1	6,33541E-08	0,380474409	0,406	0,07	0,001336834	1,301769853	0,768184943	5,8
ARL6IP5	0,000447869	0,380408738	0,75	0,328	1	1,301710599	0,768219911	2,286585366
MRPL28	5,43746E-10	0,380188231	0,469	0,07	1,14736E-05	1,301511655	0,768337338	6,7
CDC123	1,7434E-09	0,379738725	0,438	0,066	3,67875E-05	1,301106202	0,768576769	6,636363636
GSDMD	9,01089E-08	0,379489621	0,438	0,082	0,001901388	1,300881564	0,768709487	5,341463415

Supplementary Table 1

Differentially expressed genes_adjacent-skin-XL_ comparing SC-Keloid vs. (SC-Myel, SC-Nonmyel)								
**gene**	**p_val**	**avg_log2FC**	**pct.1**	**pct.2**	**p_val_adj**	**Foldchange_UP**	**Foldchange_DOWN**	**Ratio_pct1_pct2**
BSCL2	9,16263E-06	0,37932465	0,438	0,109	0,19334064	1,300732818	0,768797394	4,018348624
MDK	0,011117037	0,379076574	0,344	0,145	1	1,300509172	0,768929602	2,372413793
CIAO2A	5,02386E-14	0,379043157	0,375	0,023	1,06008E-09	1,300479049	0,768947413	16,30434783
DTD1	5,92885E-07	0,378547478	0,5	0,117	0,012510463	1,300032309	0,769211652	4,273504274
ATP6V1D	6,38968E-06	0,378260908	0,438	0,109	0,134828681	1,299774102	0,76936446	4,018348624
TM9SF2	7,15869E-08	0,378195828	0,531	0,113	0,001510555	1,29971547	0,769399167	4,699115044
ERCC8	2,52232E-10	0,377956008	0,188	0,004	5,32235E-06	1,299499436	0,769527075	47
CNIH3	9,87864E-16	0,377672035	0,281	0,004	2,08449E-11	1,299243674	0,76967856	70,25
VPS28	3,55638E-05	0,377577187	0,656	0,227	0,750431063	1,29915826	0,769729163	2,889867841
RRP15	4,90429E-05	0,37704658	0,375	0,098	1	1,298680532	0,770012313	3,826530612
SLC39A7	2,46918E-07	0,376935165	0,594	0,148	0,005210227	1,298580243	0,770071781	4,013513514
HPRT1	2,11462E-10	0,376914722	0,438	0,059	4,46205E-06	1,298561842	0,770082693	7,423728814
CALM2	0,003225786	0,376810987	0,969	0,848	1	1,298468474	0,770138067	1,142688679
MEGF8	2,29201E-06	0,376530801	0,344	0,066	0,048363718	1,298216323	0,77028765	5,212121212
MRPL54	7,64549E-09	0,37520557	0,469	0,082	0,000161327	1,297024355	0,770995546	5,719512195
MLLT11	3,15447E-10	0,374927852	0,375	0,043	6,65625E-06	1,296774703	0,771143976	8,720930233
GOT2	7,37666E-07	0,374789001	0,188	0,016	0,015565494	1,296649901	0,771218198	11,75
TNIP1	1,6211E-08	0,374507203	0,438	0,074	0,000342069	1,296396655	0,771368853	5,918918919
PPP1CA	1,71675E-09	0,374056156	0,469	0,074	3,62252E-05	1,29599141	0,771610053	6,337837838
FASTK	1,78074E-06	0,373529325	0,406	0,09	0,037575365	1,295518238	0,771891874	4,511111111
KRT10	8,56866E-05	0,373494831	0,906	0,449	1	1,295487263	0,77191033	2,017817372
FAM174C	2,07713E-07	0,373468502	0,406	0,078	0,004382957	1,295463621	0,771924417	5,205128205
JMJD7	0,000486824	0,373291435	0,094	0,008	1	1,295304634	0,772019164	11,75
MRPS26	4,36552E-07	0,372872951	0,469	0,102	0,00921169	1,294928958	0,772243136	4,598039216
SSBP1	4,65446E-06	0,372536093	0,656	0,203	0,098213786	1,294626637	0,772423471	3,231527094
PPP2R1A	3,11676E-05	0,372518176	0,531	0,164	0,657667304	1,294610559	0,772433063	3,237804878
TSG101	2,60416E-06	0,372495373	0,5	0,129	0,054950464	1,294590097	0,772445272	3,875968992
REX1BD	3,93675E-07	0,372419377	0,656	0,172	0,008306936	1,294521904	0,772485963	3,813953488
MT1F	1,6383E-10	0,372364441	0,469	0,066	3,45698E-06	1,294472611	0,772515379	7,106060606
LMF2	2,95078E-12	0,371936449	0,531	0,07	6,22644E-08	1,294088648	0,772744589	7,585714286

Supplementary Table 1

Differentially expressed genes_adjacent-skin-XL_ comparing SC-Keloid vs. (SC-Myel, SC-Nonmyel)								
**gene**	**p_val**	**avg_log2FC**	**pct.1**	**pct.2**	**p_val_adj**	**Foldchange_UP**	**Foldchange_DOWN**	**Ratio_pct1_pct2**
AMOTL1	1,43796E-05	0,371620028	0,375	0,09	0,303422967	1,293804852	0,77291409	4,166666667
RPL21	0,009607627	0,371374252	0,938	0,766	1	1,293584459	0,773045775	1,224543081
YAF2	0,000426252	0,371214617	0,312	0,09	1	1,293441332	0,773131317	3,466666667
CENPF	0,013054404	0,371012873	0,062	0,008	1	1,293260472	0,773239438	7,75
OXCT1	1,41616E-06	0,370118017	0,281	0,043	0,02988246	1,292458554	0,773719201	6,534883721
C1QTNF3	1,57978E-10	0,369928453	0,219	0,008	3,33348E-06	1,292288741	0,773820871	27,375
SNW1	1,87015E-07	0,369847189	0,594	0,148	0,003946205	1,292215952	0,77386446	4,013513514
F2R	6,00279E-06	0,369786264	0,25	0,039	0,1266649	1,292161382	0,773897142	6,41025641
HOXD8	6,91427E-09	0,369242482	0,188	0,008	0,000145898	1,291674431	0,774188894	23,5
NAA20	1,28255E-06	0,369100103	0,469	0,109	0,027062998	1,291546963	0,774265303	4,302752294
SUCLG1	2,84806E-06	0,368937192	0,375	0,078	0,060097016	1,291401128	0,774352739	4,807692308
LGI1	0,000633004	0,367857151	0,188	0,039	1	1,290434711	0,774932658	4,820512821
ZNF506	0,027767885	0,367679309	0,188	0,07	1	1,290275648	0,77502819	2,685714286
FAM104B	2,84493E-08	0,367665365	0,312	0,039	0,000600308	1,290263177	0,775035681	8
SRP9	4,64131E-06	0,367600167	0,75	0,242	0,097936273	1,290204869	0,775070707	3,099173554
PSMD13	4,13014E-08	0,367578612	0,656	0,156	0,000871501	1,290185593	0,775082287	4,205128205
IAH1	1,16993E-05	0,367555812	0,656	0,219	0,24686625	1,290165203	0,775094536	2,99543379
MLX	3,65877E-09	0,367551948	0,375	0,051	7,72037E-05	1,290161748	0,775096612	7,352941176
PRKAB1	1,55244E-05	0,367502934	0,188	0,023	0,32758078	1,290117917	0,775122946	8,173913043
RPL7	0,009573799	0,367296046	0,969	0,73	1	1,289932922	0,775234109	1,32739726
AGPS	3,14761E-12	0,367186381	0,375	0,031	6,64178E-08	1,289834873	0,77529304	12,09677419
PRR13	6,33541E-08	0,366820386	0,406	0,07	0,001336834	1,289507698	0,775489748	5,8
LYPLA1	8,66868E-17	0,366766006	0,469	0,031	1,82918E-12	1,289459093	0,775518979	15,12903226
RER1	1,96381E-05	0,366734113	0,562	0,176	0,414384298	1,289430588	0,775536124	3,193181818
ATP5PB	2,6776E-07	0,36664669	0,625	0,16	0,005650005	1,289352455	0,77558312	3,90625
MSI2	1,17927E-11	0,366448614	0,344	0,027	2,48837E-07	1,289175445	0,775689612	12,74074074
SMIM7	8,26201E-05	0,366197264	0,531	0,176	1	1,28895086	0,775824766	3,017045455
GLB1	3,13778E-10	0,365384755	0,312	0,027	6,62103E-06	1,288225143	0,776261825	11,55555556
ERLEC1	7,00443E-08	0,365316793	0,562	0,125	0,001478005	1,288164459	0,776298393	4,496
TMEM87B	1,80314E-07	0,365289575	0,25	0,027	0,003804814	1,288140157	0,776313039	9,259259259

Supplementary Table 1

Differentially expressed genes_adjacent-skin-XL_ comparing SC-Keloid vs. (SC-Myel, SC-Nonmyel)								
**gene**	**p_val**	**avg_log2FC**	**pct.1**	**pct.2**	**p_val_adj**	**Foldchange_UP**	**Foldchange_DOWN**	**Ratio_pct1_pct2**
ILF2	7,45939E-05	0,365244768	0,625	0,223	1	1,288100151	0,77633715	2,802690583
DNAJC8	1,4543E-05	0,365202581	0,75	0,25	0,306871073	1,288062485	0,776359852	3
PRTFDC1	9,32316E-16	0,364812055	0,281	0,004	1,96728E-11	1,287713864	0,776570035	70,25
THAP2	5,34526E-05	0,364598698	0,188	0,027	1	1,28752344	0,776684889	6,962962963
NOL7	4,63255E-05	0,364516176	0,688	0,246	0,977514618	1,287449796	0,776729316	2,796747967
NANS	1,66398E-06	0,364051589	0,438	0,098	0,035111722	1,287035269	0,776979484	4,469387755
ADAM12	6,59591E-09	0,363986553	0,188	0,008	0,00013918	1,286977252	0,777014511	23,5
RAB1B	8,67864E-06	0,363009615	0,5	0,137	0,183127984	1,286106055	0,777540854	3,649635036
PBDC1	2,71813E-09	0,36300886	0,406	0,059	5,73552E-05	1,286105382	0,777541261	6,881355932
CLPP	1,07506E-06	0,362743286	0,344	0,062	0,022684808	1,285868655	0,777684405	5,548387097
MLF2	0,000318367	0,362097609	0,688	0,27	1	1,285293294	0,778032535	2,548148148
SHMT2	1,75258E-09	0,361530585	0,375	0,047	3,69813E-05	1,284788233	0,778338386	7,978723404
DNAJC9	6,28034E-07	0,360846908	0,25	0,031	0,013252142	1,284179531	0,778707319	8,064516129
NSA2	4,64355E-05	0,36081701	0,594	0,203	0,97983605	1,284152918	0,778723457	2,926108374
DCTN1	5,02498E-07	0,360498502	0,562	0,137	0,010603201	1,283869443	0,778895398	4,102189781
PIEZO1	2,01277E-07	0,360485885	0,344	0,055	0,00424715	1,283858216	0,778902209	6,254545455
RPSA	0,039722648	0,36020764	0,906	0,688	1	1,283610628	0,779052446	1,316860465
ARL16	7,27095E-11	0,360107755	0,438	0,055	1,53424E-06	1,283521761	0,779106386	7,963636364
BLMH	1,44034E-09	0,360034483	0,344	0,039	3,03927E-05	1,283456574	0,779145956	8,820512821
PSMD7	1,28427E-05	0,359656032	0,562	0,164	0,270994399	1,283119939	0,77935037	3,426829268
KRT16	0,023665185	0,359215995	0,125	0,035	1	1,282728634	0,779588117	3,571428571
INPPL1	8,0086E-05	0,35920084	0,281	0,062	1	1,282715159	0,779596306	4,532258065
MMP24OS	2,28626E-09	0,359149627	0,594	0,121	4,82423E-05	1,282669626	0,779623981	4,909090909
DRAXIN	1,0613E-19	0,359049535	0,312	0	2,23945E-15	1,28258064	0,779678072	#DIV/0!
FKBP9	1,0912E-09	0,358303527	0,688	0,141	2,30254E-05	1,281917597	0,780081342	4,879432624
RWDD1	5,11752E-06	0,358189009	0,719	0,234	0,107984844	1,281815845	0,780143266	3,072649573
SDF2L1	7,10248E-08	0,358024846	0,469	0,094	0,001498694	1,281669997	0,780232043	4,989361702
CREB3L1	5,75596E-16	0,357990874	0,25	0	1,21456E-11	1,281639817	0,780250416	#DIV/0!
C11orf49	4,44048E-14	0,356951963	0,375	0,023	9,36986E-10	1,280717217	0,780812491	16,30434783
ZMAT2	1,7738E-07	0,356783543	0,438	0,086	0,003742891	1,280567715	0,780903648	5,093023256

Supplementary Table 1

Differentially expressed genes_adjacent-skin-XL_ comparing SC-Keloid vs. (SC-Myel, SC-Nonmyel)								
**gene**	**p_val**	**avg_log2FC**	**pct.1**	**pct.2**	**p_val_adj**	**Foldchange_UP**	**Foldchange_DOWN**	**Ratio_pct1_pct2**
METTL2A	8,58517E-09	0,356524523	0,281	0,027	0,000181156	1,280337824	0,781043863	10,40740741
MACROH2A1	1,2102E-05	0,356471469	0,5	0,145	0,255365055	1,280290741	0,781072586	3,448275862
BUD23	1,02E-08	0,356449495	0,5	0,094	0,00021523	1,280271241	0,781084483	5,319148936
NDUFC1	0,00042243	0,356014694	0,781	0,359	1	1,27988545	0,781319922	2,175487465
ALG14	0,001159845	0,355928238	0,281	0,082	1	1,279808752	0,781366746	3,426829268
UBE2D1	5,57612E-08	0,35588188	0,469	0,09	0,001176618	1,279767629	0,781391854	5,211111111
ESYT2	1,8799E-07	0,355807059	0,688	0,184	0,003966775	1,279701259	0,781432379	3,739130435
SPAST	0,003333189	0,355740154	0,125	0,023	1	1,279641914	0,781468619	5,434782609
HINT2	4,09636E-07	0,355565473	0,5	0,113	0,008643726	1,279486985	0,781563245	4,424778761
CISD3	2,254E-11	0,3552175	0,406	0,043	4,75617E-07	1,279178415	0,781751778	9,441860465
PPCS	6,39045E-07	0,355088029	0,375	0,07	0,013484484	1,279063624	0,781821937	5,357142857
SF3B6	0,003648123	0,355034136	0,719	0,344	1	1,279015844	0,781851143	2,090116279
PRCC	1,83211E-08	0,354976195	0,375	0,055	0,000386594	1,278964477	0,781882545	6,818181818
PPP1R2	8,32881E-12	0,354938768	0,625	0,102	1,75746E-07	1,278931298	0,781902829	6,12745098
COMMD7	5,52128E-08	0,354715471	0,5	0,102	0,001165045	1,278733364	0,782023859	4,901960784
GTF2H5	3,13818E-05	0,353290133	0,656	0,227	0,662187226	1,277470639	0,782796856	2,889867841
UTP11	3,05972E-07	0,353280461	0,375	0,066	0,00645632	1,277462075	0,782802104	5,681818182
KARS1	5,87105E-09	0,35326892	0,438	0,07	0,000123885	1,277451855	0,782808367	6,257142857
UBE2D4	1,09475E-11	0,35322096	0,344	0,027	2,31003E-07	1,27740939	0,78283439	12,74074074
BUD31	3,92826E-06	0,352834706	0,656	0,203	0,082890191	1,277067433	0,783044007	3,231527094
C12orf75	4,03182E-13	0,352546554	0,375	0,027	8,50753E-09	1,276812388	0,783200421	13,88888889
NIFK	0,000398237	0,352456121	0,344	0,102	1	1,276732355	0,783249517	3,37254902
MRPS23	1,75258E-09	0,352301337	0,375	0,047	3,69813E-05	1,276595385	0,783333554	7,978723404
CTSK	1,08341E-05	0,35198348	0,312	0,062	0,228609655	1,276314154	0,783506159	5,032258065
NUCB1	0,000214794	0,351855392	0,75	0,285	1	1,276200843	0,783575724	2,631578947
PEX10	2,60611E-07	0,351376402	0,375	0,066	0,005499158	1,275777201	0,783835923	5,681818182
NAT10	1,90127E-05	0,350459171	0,156	0,016	0,401186735	1,27496635	0,784334426	9,75
PITPNA-AS1	0,000142816	0,350073595	0,188	0,031	1	1,274625647	0,784544076	6,064516129
COPRS	2,82414E-06	0,349800528	0,469	0,117	0,059592171	1,274384415	0,784692584	4,008547009
LIX1L	2,69906E-09	0,349583351	0,438	0,066	5,69529E-05	1,274192589	0,784810718	6,636363636

Supplementary Table 1

Differentially expressed genes_adjacent-skin-XL_ comparing SC-Keloid vs. (SC-Myel, SC-Nonmyel)								
**gene**	**p_val**	**avg_log2FC**	**pct.1**	**pct.2**	**p_val_adj**	**Foldchange_UP**	**Foldchange_DOWN**	**Ratio_pct1_pct2**
TEAD2	0,000655683	0,349314279	0,156	0,027	1	1,273954966	0,784957104	5,777777778
COMTD1	3,72083E-08	0,3488788	0,281	0,031	0,000785132	1,273570479	0,78519408	9,064516129
FANCE	0,000486824	0,348780812	0,094	0,008	1	1,273483981	0,785247412	11,75
CMAS	8,60164E-07	0,348389586	0,312	0,051	0,018150325	1,273138688	0,785460382	6,117647059
TSPAN4	7,0889E-05	0,34810712	0,438	0,125	1	1,272889444	0,785614183	3,504
JKAMP	1,03527E-07	0,348032044	0,469	0,094	0,002184527	1,272823206	0,785655066	4,989361702
WNT16	6,3574E-05	0,347851448	0,062	0	1	1,272663885	0,78575342	#DIV/0!
ADIPOR1	4,93566E-07	0,347457061	0,5	0,113	0,010414737	1,272316026	0,78596825	4,424778761
MAGOH	5,8376E-06	0,347421688	0,594	0,18	0,123179255	1,272284831	0,785987521	3,3
ABHD14B	4,32897E-07	0,347257894	0,406	0,078	0,009134556	1,272140392	0,786076762	5,205128205
BRMS1	6,60624E-08	0,347247302	0,344	0,051	0,001393983	1,272131052	0,786082533	6,745098039
ZNF322	3,25531E-10	0,346718606	0,406	0,051	6,86904E-06	1,271664948	0,786370657	7,960784314
SMIM19	2,98808E-06	0,346618591	0,562	0,156	0,063051576	1,271576792	0,786425174	3,602564103
SNRPF	6,11663E-06	0,34657946	0,594	0,168	0,129066942	1,271542303	0,786446505	3,535714286
NELFE	1,75209E-05	0,346577328	0,375	0,09	0,369707907	1,271540424	0,786447667	4,166666667
DHRS7	0,000161513	0,346003543	0,469	0,152	1	1,271034811	0,786760513	3,085526316
PSMD3	2,15233E-10	0,345912185	0,531	0,086	4,54164E-06	1,270954325	0,786810336	6,174418605
CLSPN	4,5807E-05	0,345648425	0,094	0,004	0,966573495	1,270721986	0,786954197	23,5
IRAK1	3,36857E-10	0,345625685	0,312	0,027	7,10802E-06	1,270701956	0,786966602	11,55555556
DHX29	6,3373E-07	0,34540228	0,5	0,121	0,013372347	1,2705052	0,787088475	4,132231405
RRBP1	0,000238018	0,344971927	0,625	0,25	1	1,270126267	0,787323297	2,5
TPMT	3,07079E-10	0,344954712	0,406	0,051	6,47967E-06	1,270111111	0,787332692	7,960784314
RAB31	1,64036E-09	0,344857877	0,562	0,105	3,46132E-05	1,270025863	0,78738554	5,352380952
HELLS	3,64608E-05	0,344812046	0,219	0,035	0,769358508	1,269985518	0,787410554	6,257142857
ZNF580	3,05456E-06	0,344755521	0,406	0,09	0,064454248	1,269935761	0,787441405	4,511111111
PHC2	1,77849E-06	0,344663657	0,531	0,137	0,037527896	1,2698549	0,787491547	3,875912409
B3GALT6	3,15172E-15	0,344103976	0,375	0,02	6,65044E-11	1,269362367	0,787797107	18,75
RPS12	0,001767624	0,343830509	1	0,832	1	1,269121778	0,78794645	1,201923077
CALHM2	8,46963E-16	0,343479277	0,312	0,008	1,78718E-11	1,268812841	0,788138304	39
PCDHB5	0,004841742	0,343352824	0,031	0	1	1,268701634	0,788207387	#DIV/0!

Supplementary Table 1

Differentially expressed genes_adjacent-skin-XL_ comparing SC-Keloid vs. (SC-Myel, SC-Nonmyel)								
**gene**	**p_val**	**avg_log2FC**	**pct.1**	**pct.2**	**p_val_adj**	**Foldchange_UP**	**Foldchange_DOWN**	**Ratio_pct1_pct2**
NCK2	1,92396E-11	0,343167918	0,438	0,051	4,05974E-07	1,268539038	0,788308416	8,588235294
TPD52L2	1,10416E-05	0,342587343	0,438	0,109	0,232989384	1,26802865	0,788625714	4,018348624
ARNT2	1,18109E-12	0,342514302	0,281	0,012	2,49221E-08	1,267964455	0,788665641	23,41666667
SRP19	3,88259E-10	0,341735578	0,562	0,098	8,19265E-06	1,267280229	0,789091455	5,734693878
CCS	9,21037E-08	0,34105292	0,438	0,082	0,001943481	1,266680716	0,789464928	5,341463415
SMPDL3A	5,39277E-11	0,340914973	0,25	0,012	1,13793E-06	1,266559606	0,789540418	20,83333333
ITGA7	1,56895E-06	0,340660708	0,375	0,074	0,033106457	1,266336403	0,789679581	5,067567568
TECR	0,000184294	0,34059503	0,812	0,344	1	1,266278754	0,789715532	2,360465116
LSM2	8,77573E-05	0,340295558	0,594	0,199	1	1,26601593	0,789879477	2,984924623
UBE2C	0,004841742	0,340012375	0,031	0	1	1,265767451	0,790034535	#DIV/0!
UCK2	1,76518E-09	0,339811261	0,312	0,031	3,7247E-05	1,265591014	0,790144675	10,06451613
KDELR2	0,000261715	0,339655033	0,688	0,277	1	1,265453972	0,790230243	2,483754513
KIFC3	9,65054E-06	0,339523707	0,344	0,074	0,203636023	1,265338784	0,79030218	4,648648649
ENOPH1	7,96316E-05	0,33927305	0,438	0,133	1	1,265118961	0,790439501	3,293233083
MARCHF2	1,33906E-06	0,338389872	0,406	0,086	0,028255507	1,264344728	0,790923534	4,720930233
ETFA	1,77074E-05	0,338158936	0,5	0,141	0,373642817	1,264142356	0,79105015	3,546099291
ATP5MK	0,002046656	0,33809465	0,938	0,461	1	1,264086027	0,7910854	2,034707158
DPYSL3	4,21575E-08	0,33763212	0,281	0,031	0,000889566	1,263680825	0,791339063	9,064516129
COMMD10	1,27017E-11	0,337471867	0,344	0,027	2,68019E-07	1,263540465	0,791426969	12,74074074
NDUFB6	1,25402E-06	0,337246117	0,656	0,188	0,026461019	1,263342764	0,79155082	3,489361702
DCK	5,62455E-11	0,337235949	0,312	0,023	1,18684E-06	1,26333386	0,791556398	13,56521739
COL11A1	7,70517E-07	0,337206667	0,25	0,031	0,01625868	1,263308218	0,791572465	8,064516129
APH1A	1,30473E-05	0,337107268	0,469	0,133	0,275312012	1,263221182	0,791627004	3,526315789
GGH	8,75235E-12	0,336826765	0,344	0,027	1,84683E-07	1,262975598	0,791780935	12,74074074
TRPV2	9,87864E-16	0,336740535	0,281	0,004	2,08449E-11	1,262900112	0,791828261	70,25
PLD3	3,3259E-07	0,336689206	0,656	0,176	0,007017973	1,262855181	0,791856434	3,727272727
RPN1	1,31281E-06	0,336676176	0,562	0,148	0,027701698	1,262843775	0,791863586	3,797297297
ITPRIPL2	1,00375E-05	0,336520981	0,344	0,074	0,21180149	1,262707934	0,791948774	4,648648649
SAE1	8,57361E-06	0,336029959	0,344	0,074	0,180911771	1,262278244	0,79221836	4,648648649
ATP6V0E1	0,001632141	0,335955533	0,719	0,316	1	1,262213127	0,79225923	2,275316456

Supplementary Table 1

Differentially expressed genes_adjacent-skin-XL_ comparing SC-Keloid vs. (SC-Myel, SC-Nonmyel)								
**gene**	**p_val**	**avg_log2FC**	**pct.1**	**pct.2**	**p_val_adj**	**Foldchange_UP**	**Foldchange_DOWN**	**Ratio_pct1_pct2**
CPLX1	0,079594089	0,335096261	0,031	0,004	1	1,261461575	0,792731241	7,75
MAGOHB	6,47688E-06	0,334721424	0,25	0,039	0,136668547	1,261133868	0,792937233	6,41025641
RAD51C	3,25118E-10	0,334551069	0,312	0,027	6,86031E-06	1,260984961	0,79303087	11,55555556
PNO1	6,85679E-07	0,334540104	0,25	0,031	0,014468514	1,260975376	0,793036897	8,064516129
EIF4A1	0,006267916	0,334351757	0,969	0,68	1	1,260810764	0,793140437	1,425
EBPL	4,26856E-12	0,333906508	0,406	0,039	9,00709E-08	1,260421709	0,793385256	10,41025641
CPNE2	1,89579E-09	0,333113583	0,469	0,074	4,0003E-05	1,259729154	0,793821431	6,337837838
SMDT1	2,63979E-06	0,332811113	0,656	0,191	0,05570226	1,259465072	0,793987878	3,434554974
CHP1	0,000328667	0,332288937	0,344	0,102	1	1,259009298	0,79427531	3,37254902
SEC61A1	2,79215E-07	0,33209989	0,531	0,121	0,005891712	1,258844331	0,794379396	4,388429752
TMEM240	0,016562311	0,33190249	0,094	0,02	1	1,258672098	0,794488097	4,7
C1GALT1C1	2,79642E-10	0,331838294	0,438	0,059	5,90073E-06	1,258616092	0,79452345	7,423728814
RAB8A	0,000100594	0,331067387	0,344	0,09	1	1,257943728	0,794948119	3,822222222
HARS1	2,14284E-09	0,33089104	0,438	0,066	4,5216E-05	1,257789973	0,795045295	6,636363636
PRMT1	0,003338449	0,330746086	0,594	0,277	1	1,257663604	0,79512518	2,144404332
NUTF2	2,72369E-06	0,330575398	0,406	0,09	0,057472657	1,257514816	0,795219259	4,511111111
LINC00920	1,12672E-12	0,330487732	0,281	0,012	2,3775E-08	1,257438405	0,795267582	23,41666667
MMADHC	1,53903E-06	0,330260141	0,406	0,086	0,032475139	1,257240054	0,795393049	4,720930233
CDK7	1,85141E-05	0,329947471	0,188	0,023	0,390665998	1,256967607	0,79556545	8,173913043
SUPT3H	0,030543175	0,329868912	0,188	0,07	1	1,256899163	0,795608772	2,685714286
BTBD6	3,80295E-07	0,329787292	0,438	0,09	0,008024611	1,256828057	0,795653785	4,866666667
ANKRD9	0,003488919	0,329771625	0,125	0,023	1	1,256814408	0,795662425	5,434782609
MIR503HG	2,52232E-10	0,329449497	0,188	0,004	5,32235E-06	1,256533816	0,795840102	47
KRT10-AS1	1,74709E-07	0,329252872	0,25	0,027	0,003686534	1,256362574	0,795948575	9,259259259
DCTN6	0,000260329	0,328874045	0,594	0,227	1	1,256032718	0,796157604	2,616740088
DUSP1	0,000268886	0,328688622	0,938	0,492	1	1,255871296	0,796259938	1,906504065
NOVA1	3,21766E-06	0,328674538	0,438	0,102	0,067895903	1,255859036	0,796267711	4,294117647
NBPF1	0,033585021	0,328605892	0,094	0,023	1	1,255799281	0,7963056	4,086956522
INTS8	3,28579E-06	0,328548217	0,219	0,027	0,069333472	1,255749079	0,796337435	8,111111111
SEC14L2	7,87421E-05	0,328507857	0,25	0,051	1	1,255713949	0,796359713	4,901960784

Supplementary Table 1

Differentially expressed genes_adjacent-skin-XL_ comparing SC-Keloid vs. (SC-Myel, SC-Nonmyel)								
**gene**	**p_val**	**avg_log2FC**	**pct.1**	**pct.2**	**p_val_adj**	**Foldchange_UP**	**Foldchange_DOWN**	**Ratio_pct1_pct2**
OPRL1	0,079594089	0,32841833	0,031	0,004	1	1,255636028	0,796409132	7,75
ANO4	1,23802E-12	0,32828442	0,281	0,012	2,61235E-08	1,255519486	0,796483058	23,41666667
PPP1R12A	7,37255E-07	0,328281604	0,75	0,23	0,015556819	1,255517036	0,796484613	3,260869565
DERL2	3,79079E-09	0,327961982	0,469	0,078	7,99894E-05	1,255238913	0,796661089	6,012820513
CCNI	0,011301623	0,327699451	0,844	0,48	1	1,255010515	0,796806073	1,758333333
MTHFS	2,03907E-07	0,32764846	0,219	0,02	0,004302644	1,254966157	0,796834237	10,95
RPS8	0,014300261	0,327449406	0,969	0,863	1	1,254793017	0,796944186	1,122827346
EIF3H	0,000504555	0,32744096	0,719	0,312	1	1,254785671	0,796948852	2,304487179
HCFC1R1	5,37316E-05	0,327301476	0,719	0,258	1	1,254664361	0,797025907	2,786821705
CHMP1B	1,94E-07	0,327244261	0,469	0,098	0,004093585	1,254614604	0,797057516	4,785714286
ADGRG1	3,59796E-11	0,326866821	0,531	0,078	7,59206E-07	1,254286412	0,797266071	6,807692308
C5orf34	1,39097E-05	0,326549249	0,125	0,008	0,293508116	1,254010345	0,797441588	15,625
DCUN1D5	6,73458E-11	0,326522659	0,469	0,062	1,42106E-06	1,253987232	0,797456285	7,564516129
EIF2B3	6,93869E-11	0,326471053	0,344	0,031	1,46413E-06	1,253942378	0,797484811	11,09677419
GFPT1	3,88452E-06	0,325984887	0,281	0,047	0,081967219	1,253519889	0,797753597	5,978723404
FAM50A	5,25599E-08	0,325963431	0,594	0,137	0,001109067	1,253501246	0,797765461	4,335766423
UBE2I	9,45231E-08	0,325861636	0,719	0,195	0,001994531	1,253412804	0,797821753	3,687179487
RAMAC	5,09162E-13	0,325269194	0,312	0,016	1,07438E-08	1,252898197	0,798149445	19,5
ZDHHC12	3,3871E-08	0,325037373	0,281	0,031	0,000714712	1,252696889	0,798277707	9,064516129
MRPS33	2,82997E-06	0,324869989	0,406	0,09	0,059715213	1,252551557	0,79837033	4,511111111
ABI1	0,00784636	0,324643728	0,312	0,121	1	1,252355132	0,79849555	2,578512397
SCOC	1,03758E-05	0,324554606	0,688	0,219	0,218939381	1,252277772	0,798544878	3,141552511
SYT11	3,91798E-05	0,324001796	0,688	0,242	0,826732185	1,251798017	0,798850922	2,842975207
TMEM121	1,43243E-10	0,323972829	0,219	0,008	3,02257E-06	1,251772883	0,798866962	27,375
G6PC3	3,45065E-10	0,323932912	0,406	0,051	7,28122E-06	1,251738249	0,798889065	7,960784314
UBE2Q2	8,02793E-12	0,323800245	0,531	0,074	1,69397E-07	1,251623147	0,798962533	7,175675676
GCSH	5,33942E-05	0,323769215	0,531	0,164	1	1,251596227	0,798979717	3,237804878
DCTPP1	1,68138E-09	0,32366262	0,344	0,039	3,54787E-05	1,251503755	0,799038753	8,820512821
ATP5MC1	4,28417E-05	0,323259101	0,656	0,227	0,904002969	1,251153761	0,799262274	2,889867841
NUDT8	6,6835E-09	0,322970762	0,25	0,02	0,000141028	1,250903729	0,799422031	12,5

Supplementary Table 1

Differentially expressed genes_adjacent-skin-XL_ comparing SC-Keloid vs. (SC-Myel, SC-Nonmyel)								
**gene**	**p_val**	**avg_log2FC**	**pct.1**	**pct.2**	**p_val_adj**	**Foldchange_UP**	**Foldchange_DOWN**	**Ratio_pct1_pct2**
FDPS	5,2444E-07	0,32292321	0,594	0,152	0,011066205	1,250862499	0,799448381	3,907894737
C8orf76	2,17326E-05	0,322814444	0,156	0,016	0,458580025	1,250768199	0,799508655	9,75
RGL2	1,30054E-06	0,322447763	0,438	0,098	0,027442635	1,250450339	0,799711887	4,469387755
GOLGA2	6,77313E-08	0,32234398	0,719	0,184	0,001429197	1,250360389	0,799769417	3,907608696
GNA12	6,53221E-07	0,322073379	0,375	0,07	0,013783618	1,250125885	0,799919442	5,357142857
ASAP2	0,000289532	0,321972224	0,625	0,238	1	1,250038236	0,79997553	2,62605042
SELENOT	0,000437405	0,321609438	0,688	0,281	1	1,249723935	0,80017672	2,448398577
GOLGA5	1,79804E-05	0,321396795	0,188	0,023	0,379403857	1,249539748	0,80029467	8,173913043
FBXO7	3,57882E-06	0,321261052	0,5	0,129	0,075516664	1,249422185	0,800369972	3,875968992
MRPL9	5,14469E-12	0,321241838	0,5	0,062	1,08558E-07	1,249405545	0,800380632	8,064516129
MGAT4B	6,73211E-07	0,321081847	0,594	0,152	0,014205428	1,249266997	0,800469397	3,907894737
NFIL3	0,000180694	0,320783505	0,688	0,27	1	1,249008681	0,800634947	2,548148148
GSTO1	5,07141E-06	0,320459155	0,625	0,188	0,107011727	1,248727908	0,800814968	3,324468085
GPSM2	5,87627E-10	0,320414023	0,469	0,07	1,23995E-05	1,248688845	0,800840021	6,7
DPY30	7,98674E-07	0,320292226	0,531	0,129	0,016852812	1,248583431	0,800907633	4,11627907
SCARB2	0,000398693	0,32022355	0,875	0,441	1	1,248523997	0,800945759	1,984126984
GABRA2	8,59744E-08	0,320211066	0,688	0,18	0,001814146	1,248513193	0,80095269	3,822222222
POLR3GL	6,04459E-06	0,319962852	0,406	0,094	0,127546957	1,248298406	0,801090504	4,319148936
BCKDHA	4,7449E-08	0,319832587	0,375	0,059	0,001001222	1,248185699	0,801162841	6,355932203
DTYMK	1,09317E-07	0,31949891	0,375	0,062	0,002306702	1,247897042	0,801348161	6,048387097
MCUB	0,001371418	0,319239116	0,125	0,02	1	1,247672348	0,801492477	6,25
ADRM1	0,001284837	0,319198668	0,469	0,176	1	1,247637367	0,801514948	2,664772727
SDHAF2	1,80411E-09	0,318875387	0,469	0,074	3,80685E-05	1,247357826	0,801694573	6,337837838
NRM	1,4529E-06	0,31839786	0,281	0,043	0,030657605	1,246945024	0,801959975	6,534883721
WDR18	1,04183E-07	0,317854966	0,375	0,062	0,002198366	1,24647588	0,802261814	6,048387097
MLH1	3,30275E-05	0,317306624	0,219	0,035	0,696912488	1,246002207	0,802566796	6,257142857
PITPNA	2,58449E-06	0,31726357	0,438	0,102	0,054535414	1,245965023	0,802590748	4,294117647
ANKRD54	1,7341E-09	0,317207772	0,344	0,039	3,65911E-05	1,245916835	0,80262179	8,820512821
CNKSR1	0,216675772	0,317104338	0,031	0,008	1	1,245827513	0,802679335	3,875
NDRG4	0,216675772	0,316834843	0,031	0,008	1	1,245594814	0,80282929	3,875

Supplementary Table 1

Differentially expressed genes_adjacent-skin-XL_ comparing SC-Keloid vs. (SC-Myel, SC-Nonmyel)								
gene	p_val	avg_log2FC	pct.1	pct.2	p_val_adj	Foldchange_UP	Foldchange_DOWN	Ratio_pct1_pct2
FAU	0,005295918	0,316797532	1	0,832	1	1,245562601	0,802850053	1,201923077
RPS21	0,00924843	0,316607774	1	0,719	1	1,245398783	0,802955659	1,390820584
OSTM1	5,18265E-06	0,316454871	0,375	0,082	0,109359146	1,245266796	0,803040764	4,573170732
GREB1	1,39097E-05	0,316070232	0,125	0,008	0,293508116	1,244934839	0,803254892	15,625
CARHSP1	0,000184559	0,31576346	0,312	0,082	1	1,244670146	0,803425713	3,804878049
NPL	5,89503E-14	0,315520276	0,25	0,004	1,24391E-09	1,24446036	0,803561152	62,5
SIDT2	4,18583E-08	0,315504515	0,25	0,023	0,000883252	1,244446764	0,803569931	10,86956522
SUSD4	9,06462E-07	0,3153256	0,125	0,004	0,019127258	1,244292445	0,803669591	31,25
YIF1B	2,25587E-09	0,314469599	0,438	0,066	4,7601E-05	1,243554381	0,804146578	6,636363636
STK10	4,50007E-07	0,313945841	0,281	0,039	0,009495599	1,243103002	0,804438569	7,205128205
NUDT22	2,30976E-11	0,313157229	0,438	0,051	4,87383E-07	1,242423677	0,804878415	8,588235294
GORAB	7,59646E-09	0,312593911	0,188	0,008	0,000160293	1,241938653	0,805192751	23,5
TSEN15	1,1764E-13	0,312428071	0,406	0,031	2,48233E-09	1,241795898	0,805285315	13,09677419
PTGR1	1,5926E-11	0,312136218	0,469	0,059	3,36054E-07	1,241544711	0,805448238	7,949152542
SPAG7	0,000214295	0,312092738	0,375	0,109	1	1,241507294	0,805472513	3,440366972
POLG2	2,03294E-05	0,311779761	0,156	0,016	0,428970333	1,241237992	0,80564727	9,75
GTPBP2	1,92051E-07	0,311526977	0,25	0,027	0,004052478	1,241020526	0,805788445	9,259259259
DCSTAMP	1,33967E-08	0,311376777	0,125	0	0,000282684	1,240891329	0,805872341	#DIV/0!
VCP	1,74429E-07	0,311309282	0,625	0,152	0,003680625	1,240833277	0,805910044	4,111842105
ARPC1A	7,09663E-05	0,311116583	0,594	0,199	1	1,240667551	0,806017695	2,984924623
EFTUD2	7,99636E-09	0,310911541	0,312	0,035	0,000168731	1,240491234	0,806132258	8,914285714
CSK	3,79454E-10	0,31066467	0,375	0,043	8,00686E-06	1,240278983	0,806270213	8,720930233
PFN2	7,20697E-09	0,310626342	0,5	0,09	0,000152074	1,240246032	0,806291634	5,555555556
RGS20	1,33967E-08	0,310469159	0,125	0	0,000282684	1,240110914	0,806379485	#DIV/0!
RFC4	4,5807E-05	0,310087534	0,094	0,004	0,966573495	1,23978292	0,806592818	23,5
BCAP29	2,4257E-05	0,309986939	0,594	0,191	0,511847132	1,239696477	0,806649062	3,109947644
DERL1	7,69876E-13	0,30984512	0,406	0,035	1,62452E-08	1,239574619	0,806728361	11,6
DBNL	4,11565E-08	0,309606911	0,5	0,098	0,000868444	1,239369965	0,806861573	5,102040816
CENPW	2,86276E-12	0,309004159	0,188	0	6,04072E-08	1,238852269	0,807198747	#DIV/0!
TRIP6	5,6068E-07	0,308911164	0,594	0,152	0,011830901	1,238772416	0,80725078	3,907894737

Supplementary Table 1

Differentially expressed genes_adjacent-skin-XL_ comparing SC-Keloid vs. (SC-Myel, SC-Nonmyel)								
**gene**	**p_val**	**avg_log2FC**	**pct.1**	**pct.2**	**p_val_adj**	**Foldchange_UP**	**Foldchange_DOWN**	**Ratio_pct1_pct2**
ENY2	9,43521E-06	0,308629823	0,812	0,285	0,199092297	1,238530866	0,807408219	2,849122807
CHST1	2,66394E-10	0,308291334	0,188	0,004	5,62118E-06	1,238240312	0,807597677	47
TK1	4,5807E-05	0,308211714	0,094	0,004	0,966573495	1,238171978	0,807642248	23,5
IFT20	2,30034E-15	0,308075421	0,531	0,051	4,85396E-11	1,238055012	0,807718551	10,41176471
MPC1	8,25323E-05	0,307451652	0,656	0,227	1	1,237519838	0,808067854	2,889867841
MCM3	3,52698E-08	0,307420619	0,25	0,023	0,000744228	1,237493219	0,808085236	10,86956522
AUP1	5,41025E-07	0,307289789	0,594	0,148	0,011416173	1,237381002	0,808158521	4,013513514
ACADM	2,01277E-07	0,307218585	0,344	0,055	0,00424715	1,237319933	0,808198408	6,254545455
TWF2	6,92349E-09	0,307040579	0,344	0,043	0,000146093	1,237167276	0,808298133	8
TIMM22	4,04517E-08	0,306872533	0,25	0,023	0,000853571	1,237023179	0,80839229	10,86956522
HAUS1	2,66329E-14	0,306775127	0,312	0,012	5,61981E-10	1,236939661	0,808446872	26
VAC14	6,06826E-11	0,306608233	0,312	0,023	1,28046E-06	1,236796579	0,8085404	13,56521739
DPP7	3,80125E-07	0,306032306	0,625	0,16	0,00802102	1,236302945	0,808863236	3,90625
PELO	0,247238819	0,305841135	0,219	0,129	1	1,236139133	0,808970425	1,697674419
RNF187	2,08401E-05	0,305672627	0,594	0,188	0,439745967	1,23599476	0,809064919	3,159574468
FSTL3	0,000150814	0,305487016	0,781	0,32	1	1,235835753	0,809169016	2,440625
LIN52	0,036851882	0,3048389	0,125	0,039	1	1,235280691	0,809532609	3,205128205
PSMB9	2,78278E-07	0,3045081	0,469	0,098	0,00587194	1,234997482	0,80971825	4,785714286
IGSF8	0,000288619	0,304385497	0,469	0,156	1	1,234892534	0,809787065	3,006410256
NDUFS2	4,67267E-09	0,304248153	0,562	0,109	9,85981E-05	1,234774978	0,80986416	5,155963303
MCM4	8,54581E-07	0,304052232	0,219	0,023	0,018032515	1,234607304	0,809974149	9,52173913
PCDHGC3	6,466E-11	0,303820228	0,25	0,012	1,36439E-06	1,234408779	0,810104413	20,83333333
NUBP2	4,7968E-08	0,303728214	0,469	0,09	0,001012173	1,234330052	0,810156083	5,211111111
TRIM58	8,22802E-07	0,303594648	0,188	0,016	0,01736194	1,234215782	0,810231091	11,75
LINC00665	8,49478E-10	0,303497273	0,25	0,016	1,79248E-05	1,234132482	0,81028578	15,625
IDH2	4,88462E-07	0,303484418	0,281	0,039	0,01030703	1,234121485	0,810293	7,205128205
DHRS4-AS1	2,65168E-08	0,303437015	0,219	0,016	0,00055953	1,234080936	0,810319624	13,6875
RPL36AL	0,006015039	0,303180306	0,875	0,473	1	1,233861366	0,810463823	1,849894292
RMDN1	6,30011E-07	0,303136415	0,344	0,059	0,013293862	1,23382383	0,81048848	5,830508475
FAF2	0,000268309	0,302937073	0,281	0,07	1	1,23365336	0,810600475	4,014285714

Supplementary Table 1

Differentially expressed genes_adjacent-skin-XL_ comparing SC-Keloid vs. (SC-Myel, SC-Nonmyel)								
**gene**	**p_val**	**avg_log2FC**	**pct.1**	**pct.2**	**p_val_adj**	**Foldchange_UP**	**Foldchange_DOWN**	**Ratio_pct1_pct2**
KLHL36	1,79492E-05	0,302606801	0,281	0,055	0,378745038	1,233370975	0,810786066	5,109090909
LDLRAP1	4,58437E-12	0,302572726	0,438	0,047	9,67347E-08	1,233341845	0,810805215	9,319148936
ATXN10	1,91697E-05	0,301962751	0,438	0,117	0,404500592	1,232820495	0,811148098	3,743589744
COX7B	0,002900042	0,301309278	0,75	0,359	1	1,232262211	0,811515594	2,08913649
PSMA3	0,000413037	0,300998918	0,625	0,254	1	1,23199715	0,81169019	2,460629921
IARS2	9,33487E-05	0,300815098	0,281	0,062	1	1,231840185	0,811793617	4,532258065
TPRN	0,003590182	0,300772733	0,156	0,035	1	1,231804012	0,811817456	4,457142857
NFIX	0,00010839	0,300685448	0,688	0,25	1	1,231729489	0,811866573	2,752
PBXIP1	0,000632975	0,300486291	0,25	0,062	1	1,231559467	0,811978656	4,032258065
CENPH	1,59484E-07	0,300181854	0,219	0,02	0,003365275	1,231299611	0,812150017	10,95
CAPNS1	0,000129179	0,300172562	0,719	0,262	1	1,23129168	0,812155248	2,744274809
MRPS36	2,01009E-07	0,300142908	0,562	0,133	0,004241491	1,231266372	0,812171942	4,22556391
ASB3	0,000390215	0,299752936	0,25	0,059	1	1,230933596	0,812391508	4,237288136
VANGL1	2,8258E-09	0,299180108	0,219	0,012	5,96273E-05	1,230444945	0,812714135	18,25
PTTG1IP	1,08578E-05	0,298965778	0,75	0,258	0,229109575	1,230262162	0,812834883	2,906976744
POLR2H	5,56188E-08	0,298862841	0,562	0,129	0,001173613	1,230174385	0,812892881	4,356589147
COMMD6	0,002311566	0,298808715	0,812	0,395	1	1,230128233	0,812923379	2,055696203
TAF6	1,45029E-07	0,298793604	0,281	0,035	0,003060257	1,230115348	0,812931894	8,028571429
RPL39L	6,24833E-11	0,298664593	0,344	0,031	1,31846E-06	1,230005352	0,813004593	11,09677419
TOR1AIP2	6,45154E-06	0,298412999	0,5	0,133	0,136133995	1,229790868	0,813146386	3,759398496
POFUT1	2,76082E-06	0,298310895	0,25	0,035	0,058256123	1,229703835	0,813203937	7,142857143
SLFN5	1,31816E-11	0,298025708	0,344	0,027	2,78145E-07	1,229460776	0,813364704	12,74074074
DHRS7B	1,63999E-07	0,29709402	0,25	0,027	0,003460546	1,228667049	0,813890143	9,259259259
THAP11	8,3444E-11	0,296923284	0,375	0,039	1,76075E-06	1,228521651	0,813986468	9,615384615
ETHE1	0,000161827	0,296864145	0,375	0,105	1	1,228471293	0,814019835	3,571428571
SNRNP25	8,87823E-09	0,296858829	0,281	0,027	0,00018734	1,228466766	0,814022835	10,40740741
CYP27C1	3,86379E-06	0,296799681	0,156	0,012	0,081529835	1,228416402	0,81405621	13
ALYREF	1,15762E-05	0,296669994	0,406	0,102	0,244269533	1,228305982	0,81412939	3,980392157
PPP1R9B	0,000142816	0,296243935	0,188	0,031	1	1,22794329	0,814369855	6,064516129
MRC2	2,64255E-12	0,296193455	0,25	0,008	5,57605E-08	1,227900325	0,814398351	31,25

Supplementary Table 1

Differentially expressed genes_adjacent-skin-XL_ comparing SC-Keloid vs. (SC-Myel, SC-Nonmyel)								
**gene**	**p_val**	**avg_log2FC**	**pct.1**	**pct.2**	**p_val_adj**	**Foldchange_UP**	**Foldchange_DOWN**	**Ratio_pct1_pct2**
RASGEF1C	0,020165934	0,296174683	0,344	0,148	1	1,227884348	0,814408948	2,324324324
FAM184B	1,96814E-10	0,296093666	0,156	0	4,15296E-06	1,227815397	0,814454683	#DIV/0!
SPOCD1	3,21675E-07	0,295766059	0,156	0,008	0,006787672	1,227536616	0,814639651	19,5
ANTKMT	5,76305E-06	0,295662429	0,5	0,133	0,12160622	1,227448444	0,814698169	3,759398496
ISY1	6,27223E-13	0,295406501	0,438	0,043	1,3235E-08	1,227230719	0,814842706	10,18604651
ATP6AP2	0,006998667	0,295366006	0,844	0,473	1	1,227196273	0,814865578	1,78435518
FAM104A	1,75619E-11	0,295352115	0,281	0,016	3,70573E-07	1,227184457	0,814873424	17,5625
ATP5ME	0,003760038	0,295293457	0,969	0,543	1	1,227134562	0,814906556	1,784530387
BRCA1	0,004607686	0,295095176	0,188	0,051	1	1,226965919	0,815018563	3,68627451
TIGAR	5,10479E-14	0,294912275	0,281	0,008	1,07716E-09	1,226810377	0,815121896	35,125
PFDN2	0,001687355	0,29488257	0,75	0,352	1	1,226785118	0,815138679	2,130681818
ATIC	7,43981E-11	0,294785865	0,344	0,031	1,56987E-06	1,226702887	0,81519332	11,09677419
RPS27	0,004104589	0,294723188	1	0,914	1	1,226649596	0,815228736	1,094091904
XYLT1	5,0197E-07	0,294614962	0,281	0,039	0,010592072	1,22655758	0,815289895	7,205128205
PCLAF	9,12354E-07	0,29426096	0,094	0	0,019251588	1,22625665	0,815489971	#DIV/0!
HGS	5,70869E-06	0,294001122	0,375	0,082	0,120459106	1,226035814	0,815636859	4,573170732
C20orf96	1,44979E-05	0,293867172	0,125	0,008	0,305919948	1,225921985	0,815712592	15,625
MED31	5,05391E-10	0,293732976	0,344	0,035	1,06643E-05	1,225807958	0,815788471	9,828571429
ANKH	2,41218E-06	0,292466638	0,25	0,035	0,050899452	1,224732467	0,816504851	7,142857143
PBRM1	5,95191E-05	0,292398116	0,531	0,164	1	1,224674298	0,816543632	3,237804878
WDR27	0,031169948	0,292277921	0,094	0,023	1	1,224572271	0,816611664	4,086956522
IFT27	1,04462E-08	0,292217949	0,406	0,062	0,000220426	1,224521367	0,816645611	6,548387097
RPE65	2,66394E-10	0,292189097	0,188	0,004	5,62118E-06	1,224496879	0,816661943	47
DUSP14	8,57488E-08	0,291725289	0,344	0,051	0,001809386	1,224103282	0,816924531	6,745098039
CLPTM1L	1,14064E-09	0,291682751	0,406	0,055	2,40686E-05	1,22406719	0,816948619	7,381818182
MLST8	8,16642E-07	0,291585482	0,25	0,031	0,01723197	1,223984665	0,8170037	8,064516129
HNRNPR	0,000384717	0,291440474	0,719	0,281	1	1,223861645	0,817085823	2,558718861
KLHL3	0,013349129	0,291428978	0,125	0,031	1	1,223851894	0,817092334	4,032258065
ESD	0,000155415	0,291270348	0,625	0,23	1	1,223717333	0,817182182	2,717391304
IPO4	7,82681E-10	0,291227918	0,25	0,016	1,65154E-05	1,223681344	0,817206216	15,625

Supplementary Table 1

Differentially expressed genes_adjacent-skin-XL_ comparing SC-Keloid vs. (SC-Myel, SC-Nonmyel)								
**gene**	**p_val**	**avg_log2FC**	**pct.1**	**pct.2**	**p_val_adj**	**Foldchange_UP**	**Foldchange_DOWN**	**Ratio_pct1_pct2**
ECH1	2,23905E-08	0,291133871	0,531	0,105	0,000472461	1,223601576	0,81725949	5,057142857
RSL24D1	0,002722867	0,291081827	0,688	0,293	1	1,223557437	0,817288972	2,348122867
IMMP1L	1,95325E-11	0,291065596	0,469	0,059	4,12155E-07	1,223543671	0,817298167	7,949152542
OAZ1	0,009661003	0,290909844	0,969	0,668	1	1,223411586	0,817386406	1,450598802
SLC39A1	1,7972E-07	0,290858824	0,562	0,129	0,003792262	1,223368322	0,817415313	4,356589147
EEF2	0,053411174	0,290857668	0,938	0,688	1	1,223367342	0,817415968	1,363372093
MZT1	9,63836E-06	0,290820728	0,406	0,098	0,203379102	1,223336017	0,817436899	4,142857143
STX10	1,97144E-07	0,290512959	0,438	0,086	0,004159927	1,223075072	0,8176113	5,093023256
SNX22	6,3574E-05	0,290181868	0,062	0	1	1,222794415	0,817798959	#DIV/0!
SDF2	3,9745E-07	0,29010833	0,406	0,078	0,008386593	1,222732087	0,817840646	5,205128205
KCTD15	1,79804E-05	0,290033738	0,188	0,023	0,379403857	1,22266887	0,817882932	8,173913043
LEPROTL1	1,73305E-06	0,289903818	0,406	0,086	0,036569061	1,222558769	0,817956589	4,720930233
MANEA-DT	0,000451818	0,289451339	0,094	0,008	1	1,222175393	0,818213168	11,75
ZGPAT	0,12647311	0,289120809	0,094	0,035	1	1,221895417	0,818400647	2,685714286
ZNF32	2,47596E-08	0,288830983	0,438	0,074	0,000522452	1,221649972	0,818565074	5,918918919
RPA3	5,02919E-06	0,288620476	0,344	0,07	0,106120986	1,221471732	0,818684521	4,914285714
ST8SIA2	2,86276E-12	0,288557244	0,188	0	6,04072E-08	1,221418197	0,818720404	#DIV/0!
RALYL	9,12354E-07	0,28848993	0,094	0	0,019251588	1,221361209	0,818758605	#DIV/0!
PGM3	6,24909E-08	0,288244749	0,562	0,125	0,00131862	1,221153661	0,818897762	4,496
CSNK1A1	0,004870538	0,288009159	0,844	0,418	1	1,220954264	0,819031498	2,019138756
CDK5	1,18109E-12	0,287967012	0,281	0,012	2,49221E-08	1,220918596	0,819055426	23,41666667
B4GALT7	1,69471E-11	0,287724923	0,375	0,035	3,57601E-07	1,220713739	0,819192877	10,71428571
PPP4R1	5,31565E-06	0,287709839	0,406	0,094	0,11216551	1,220700975	0,819201443	4,319148936
KDM2B	0,00079237	0,287378534	0,156	0,027	1	1,220420682	0,819389588	5,777777778
CCDC106	2,81727E-07	0,287364527	0,312	0,047	0,005944715	1,220408833	0,819397544	6,638297872
NMI	0,000189737	0,287309361	0,25	0,055	1	1,220362168	0,819428877	4,545454545
ANPEP	2,86276E-12	0,287234493	0,188	0	6,04072E-08	1,220298839	0,819471401	#DIV/0!
NUDT5	5,26847E-08	0,287189695	0,438	0,078	0,001111699	1,220260948	0,819496847	5,615384615
ATG101	7,84831E-07	0,286797997	0,531	0,129	0,016560709	1,219929686	0,819719375	4,11627907
NSMCE1	2,11379E-11	0,286760938	0,406	0,043	4,46031E-07	1,21989835	0,819740431	9,441860465

Supplementary Table 1

Differentially expressed genes_adjacent-skin-XL_ comparing SC-Keloid vs. (SC-Myel, SC-Nonmyel)								
**gene**	**p_val**	**avg_log2FC**	**pct.1**	**pct.2**	**p_val_adj**	**Foldchange_UP**	**Foldchange_DOWN**	**Ratio_pct1_pct2**
PAK1IP1	3,88452E-06	0,286571592	0,281	0,047	0,081967219	1,219738256	0,819848025	5,978723404
FAM114A2	9,0771E-08	0,285874552	0,188	0,012	0,001915359	1,219149079	0,820244232	15,66666667
DNMT1	1,07328E-06	0,285738571	0,531	0,129	0,022647178	1,219034174	0,820321547	4,11627907
KIF7	0,039886012	0,285622941	0,062	0,012	1	1,218936475	0,820387297	5,166666667
MED16	7,66065E-05	0,285511521	0,219	0,039	1	1,218842339	0,820450659	5,615384615
CDK18	4,38117E-05	0,284591151	0,281	0,059	0,924471165	1,218065024	0,820974234	4,762711864
TXNDC9	5,01789E-11	0,284509308	0,312	0,023	1,05883E-06	1,217995927	0,821020808	13,56521739
HMG20B	0,000109069	0,284260889	0,75	0,285	1	1,217786217	0,821162193	2,631578947
SLC39A13	7,27202E-05	0,284257947	0,375	0,098	1	1,217783733	0,821163868	3,826530612
SPDL1	5,34526E-05	0,284209886	0,188	0,027	1	1,217743165	0,821191224	6,962962963
PCNX2	1,51098E-05	0,284030544	0,125	0,008	0,318831333	1,217591796	0,821293313	15,625
GPX4	0,010556548	0,283811156	0,75	0,426	1	1,217406654	0,821418215	1,76056338
CTXN1	4,09886E-14	0,283517708	0,219	0	8,649E-10	1,217159055	0,82158531	#DIV/0!
SLC35A2	5,25184E-05	0,28273047	0,281	0,059	1	1,216495067	0,822033748	4,762711864
DHODH	9,0771E-08	0,282345924	0,188	0,012	0,001915359	1,216170857	0,822252888	15,66666667
FH	2,99054E-07	0,282316069	0,375	0,066	0,006310343	1,21614569	0,822269904	5,681818182
TUBG1	1,77239E-07	0,282312126	0,219	0,02	0,00373992	1,216142366	0,822272151	10,95
POLRMT	0,060046712	0,282235308	0,156	0,062	1	1,216077613	0,822315935	2,516129032
FMOD	0,034213216	0,281937369	0,094	0,023	1	1,2158265	0,822485774	4,086956522
PUF60	0,000136604	0,281648933	0,75	0,277	1	1,215583446	0,822650229	2,707581227
NDUFAF4	0,004972449	0,281638417	0,219	0,066	1	1,215574586	0,822656225	3,318181818
CDC42EP1	3,35661E-05	0,281586525	0,75	0,281	0,708279251	1,215530863	0,822685816	2,669039146
PFDN4	1,03996E-05	0,281501511	0,5	0,133	0,219441092	1,215459238	0,822734296	3,759398496
C15orf61	3,59137E-08	0,281376523	0,312	0,039	0,000757815	1,215353941	0,822805576	8
COX14	3,25025E-05	0,28104458	0,531	0,164	0,68583617	1,215074338	0,822994914	3,237804878
CUL2	1,31608E-06	0,281002536	0,344	0,062	0,027770673	1,215038928	0,823018899	5,548387097
RPS29	0,006923322	0,280933242	1	0,832	1	1,21498057	0,82305843	1,201923077
UBTD1	1,08519E-08	0,280899559	0,281	0,027	0,000228985	1,214952203	0,823077646	10,40740741
RPL15	0,018541127	0,280661666	0,969	0,832	1	1,21475188	0,823213379	1,164663462
ATP11B	7,53121E-05	0,280081691	0,344	0,086	1	1,214263639	0,823544384	4

Supplementary Table 1

Differentially expressed genes_adjacent-skin-XL_ comparing SC-Keloid vs. (SC-Myel, SC-Nonmyel)								
**gene**	**p_val**	**avg_log2FC**	**pct.1**	**pct.2**	**p_val_adj**	**Foldchange_UP**	**Foldchange_DOWN**	**Ratio_pct1_pct2**
TOR3A	1,68113E-05	0,279964702	0,25	0,043	0,354735265	1,214165178	0,823611168	5,813953488
GPI	6,67693E-07	0,27947085	0,375	0,07	0,014088981	1,213749625	0,823893149	5,357142857
TTLL1	4,27234E-12	0,279290744	0,219	0,004	9,01506E-08	1,21359811	0,82399601	54,75
TXNDC12	1,51302E-05	0,279136095	0,438	0,113	0,319262325	1,213468026	0,824084342	3,876106195
SLC25A4	4,8636E-06	0,279124532	0,438	0,105	0,102626823	1,2134583	0,824090947	4,171428571
ARF1	0,007847613	0,278966274	0,812	0,488	1	1,213325196	0,824181352	1,663934426
MTA1	4,97968E-06	0,278890288	0,469	0,117	0,105076243	1,213261293	0,824224762	4,008547009
UPF3B	4,12646E-12	0,278867453	0,406	0,039	8,70723E-08	1,213242089	0,824237808	10,41025641
RTF2	0,000151745	0,27885791	0,562	0,199	1	1,213234064	0,82424326	2,824120603
ID3	0,000169991	0,278848981	0,75	0,293	1	1,213226555	0,824248362	2,559726962
EIF3E	0,000646176	0,27872378	0,812	0,359	1	1,213121273	0,824319895	2,26183844
PIP4P2	7,4957E-05	0,278569462	0,312	0,074	1	1,212991518	0,824408073	4,216216216
ITGAE	6,97568E-07	0,278529026	0,375	0,07	0,014719382	1,212957521	0,82443118	5,357142857
C9orf64	1,86093E-07	0,278478276	0,25	0,027	0,003926759	1,212914853	0,824460182	9,259259259
TMTC3	5,30076E-07	0,278194149	0,281	0,039	0,011185134	1,212676002	0,824622569	7,205128205
TOR1A	4,62713E-14	0,277994256	0,375	0,023	9,7637E-10	1,212507991	0,824736832	16,30434783
CYB561A3	8,24168E-09	0,277920551	0,344	0,043	0,000173908	1,212446048	0,824778968	8
BLOC1S4	2,04525E-07	0,27757477	0,25	0,027	0,00431569	1,212155487	0,824976672	9,259259259
NOSIP	5,54069E-05	0,277522241	0,344	0,082	1	1,212111353	0,82500671	4,195121951
UBE4A	0,000292494	0,277355235	0,281	0,07	1	1,211971048	0,825102218	4,014285714
ROGDI	7,24756E-09	0,277213019	0,188	0,008	0,000152931	1,211851582	0,825183558	23,5
TUBA1C	0,001879994	0,277078393	0,5	0,195	1	1,211738502	0,825260564	2,564102564
UBA6-DT	7,24756E-09	0,276619012	0,188	0,008	0,000152931	1,211352723	0,825523384	23,5
FNBP1L	9,95832E-07	0,276577493	0,312	0,051	0,021013058	1,211317862	0,825547143	6,117647059
SIL1	2,77715E-07	0,276221364	0,562	0,133	0,005860059	1,211018885	0,825750954	4,22556391
MDH1	6,81779E-06	0,27619918	0,594	0,172	0,143862188	1,211000265	0,825763651	3,453488372
IMMT	1,91188E-05	0,275945768	0,344	0,078	0,403425253	1,210787569	0,825908711	4,41025641
PDCL	3,59137E-08	0,275640738	0,312	0,039	0,000757815	1,210531598	0,826083352	8
APBB1	3,48389E-06	0,275591028	0,219	0,027	0,073513559	1,210489889	0,826111816	8,111111111
H2AJ	0,038034675	0,275442596	0,781	0,414	1	1,210365353	0,826196815	1,88647343

Supplementary Table 1

Differentially expressed genes_adjacent-skin-XL_ comparing SC-Keloid vs. (SC-Myel, SC-Nonmyel)								
**gene**	**p_val**	**avg_log2FC**	**pct.1**	**pct.2**	**p_val_adj**	**Foldchange_UP**	**Foldchange_DOWN**	**Ratio_pct1_pct2**
NDUFAF1	2,24215E-10	0,275403455	0,281	0,02	4,73116E-06	1,210332516	0,82621923	14,05
HOMER1	3,91499E-06	0,275074046	0,219	0,027	0,082610119	1,210056193	0,826407902	8,111111111
MRPS16	7,3383E-09	0,274774116	0,344	0,043	0,000154846	1,209804654	0,826579727	8
ZBED1	0,0555007	0,274483403	0,125	0,043	1	1,209560895	0,826746305	2,906976744
RALB	4,27613E-06	0,27426051	0,281	0,047	0,090230617	1,209374035	0,826874045	5,978723404
AREG	0,433572992	0,274053582	0,062	0,035	1	1,209200585	0,826992653	1,771428571
RPL22L1	0,002313981	0,273925461	0,75	0,359	1	1,209093205	0,827066099	2,08913649
DDX23	6,03826E-06	0,273819409	0,312	0,059	0,127413405	1,209004328	0,827126899	5,288135593
RAB5C	5,46517E-06	0,273677852	0,656	0,195	0,115320473	1,208885706	0,82720806	3,364102564
OXSM	8,18806E-07	0,273531961	0,125	0,004	0,017277634	1,208763465	0,827291715	31,25
TFPT	1,88604E-09	0,273506394	0,312	0,031	3,97972E-05	1,208742045	0,827306376	10,06451613
TOP1MT	1,77239E-07	0,27340643	0,219	0,02	0,00373992	1,208658293	0,827363702	10,95
MED24	9,05158E-09	0,273249875	0,312	0,035	0,000190997	1,208527142	0,827453489	8,914285714
WLS	1,25535E-06	0,27324912	0,625	0,172	0,026489135	1,20852651	0,827453921	3,63372093
ENDOD1	0,009341004	0,27311313	0,531	0,242	1	1,208412598	0,827531922	2,194214876
RAB11A	6,43341E-05	0,272996324	0,656	0,223	1	1,208314765	0,827598925	2,941704036
EXOSC5	7,16496E-06	0,27281971	0,25	0,039	0,151187773	1,208166852	0,827700245	6,41025641
SULF1	0,000486824	0,272629266	0,094	0,008	1	1,208007378	0,827809514	11,75
SEC24D	2,00542E-05	0,272611436	0,406	0,102	0,423163655	1,207992449	0,827819744	3,980392157
ZNF160	0,000157414	0,272390569	0,188	0,031	1	1,207807527	0,827946488	6,064516129
INTS12	0,000392856	0,272254285	0,219	0,047	1	1,207693437	0,828024704	4,659574468
AGPAT1	3,32737E-07	0,27216029	0,469	0,098	0,007021076	1,207614755	0,828078653	4,785714286
LAGE3	2,04693E-11	0,272158335	0,406	0,043	4,31922E-07	1,207613119	0,828079775	9,441860465
TMEM223	3,9091E-08	0,272017486	0,25	0,023	0,000824859	1,207495227	0,828160623	10,86956522
LPCAT2	3,72126E-05	0,271840125	0,469	0,129	0,785222778	1,207346789	0,828262442	3,635658915
TCF4	1,41746E-05	0,27173235	0,719	0,246	0,299098567	1,2072566	0,828324318	2,922764228
CARM1	5,65549E-06	0,271428454	0,312	0,059	0,11933641	1,207002324	0,828498819	5,288135593
GLRX3	2,2623E-10	0,271422381	0,469	0,066	4,77367E-06	1,206997243	0,828502306	7,106060606
UBR4	5,72121E-06	0,271405399	0,406	0,094	0,120723203	1,206983036	0,828512059	4,319148936
PSMG2	9,82992E-07	0,271265998	0,594	0,152	0,020742108	1,206866416	0,828592118	3,907894737

Supplementary Table 1

Differentially expressed genes_adjacent-skin-XL_ comparing SC-Keloid vs. (SC-Myel, SC-Nonmyel)								
**gene**	**p_val**	**avg_log2FC**	**pct.1**	**pct.2**	**p_val_adj**	**Foldchange_UP**	**Foldchange_DOWN**	**Ratio_pct1_pct2**
DGAT1	3,25118E-10	0,271218619	0,312	0,027	6,86031E-06	1,206826782	0,82861933	11,55555556
TPM1	3,72621E-07	0,270965031	0,438	0,09	0,007862681	1,206614673	0,828764992	4,866666667
EPB41L4A	4,5807E-05	0,270959513	0,094	0,004	0,966573495	1,206610058	0,828768162	23,5
ZNF417	0,082317662	0,270950975	0,031	0,004	1	1,206602916	0,828773067	7,75
PPIL3	2,72573E-08	0,27088498	0,344	0,047	0,000575156	1,206547723	0,82881098	7,319148936
ZWINT	1,33443E-05	0,270766214	0,125	0,008	0,281577496	1,206448401	0,828879212	15,625
TSPAN11	0,000818349	0,270643931	0,562	0,219	1	1,206346147	0,828949471	2,566210046
MAPKAP1	1,22749E-05	0,270434937	0,312	0,062	0,259013251	1,206171404	0,829069564	5,032258065
MAD2L1	3,44546E-06	0,270308526	0,156	0,012	0,072702732	1,206065722	0,829142211	13
PIGT	3,53787E-05	0,270279642	0,469	0,133	0,746526569	1,206041576	0,829158812	3,526315789
ECI1	5,4927E-06	0,270082221	0,5	0,129	0,11590148	1,20587655	0,829272283	3,875968992
SUCLG2	1,07156E-05	0,270054364	0,469	0,121	0,226109558	1,205853266	0,829288296	3,876033058
TMEM161A	4,64156E-06	0,270046971	0,312	0,059	0,097941569	1,205847086	0,829292546	5,288135593
DYRK4	0,000168807	0,269622221	0,25	0,055	1	1,205492121	0,829536737	4,545454545
SPG21	1,38976E-05	0,269095125	0,312	0,062	0,293252516	1,205051769	0,829839867	5,032258065
PCDHB2	8,61561E-07	0,268879659	0,125	0,004	0,018179807	1,204871808	0,829963813	31,25
KDM1A	1,65075E-06	0,268849285	0,281	0,043	0,034832408	1,204846441	0,829981287	6,534883721
C2orf81	0,00050526	0,26884513	0,094	0,008	1	1,204842971	0,829983678	11,75
GSTA4	2,58449E-06	0,26861788	0,438	0,102	0,054535414	1,204653202	0,830114425	4,294117647
TMSB15A	0,00050526	0,26857011	0,094	0,008	1	1,204613315	0,830141912	11,75
CRAT	3,74455E-07	0,268558116	0,312	0,047	0,007901365	1,2046033	0,830148813	6,638297872
TRAF7	4,68061E-05	0,268438336	0,312	0,07	0,987655199	1,204503292	0,830217739	4,457142857
EPRS1	2,48397E-07	0,26821883	0,438	0,086	0,005241431	1,204320041	0,830344067	5,093023256
ENDOG	0,000183387	0,268206058	0,281	0,066	1	1,204309379	0,830351418	4,257575758
CWC15	6,96722E-05	0,268025538	0,469	0,141	1	1,204158697	0,830455323	3,326241135
ULK4	0,000486824	0,267944412	0,094	0,008	1	1,204090986	0,830502023	11,75
METAP2	0,000197292	0,267685415	0,594	0,211	1	1,203874843	0,830651131	2,815165877
NEFM	3,82664E-06	0,267665913	0,188	0,02	0,080745856	1,20385857	0,830662359	9,4
LRP4	0,64910682	0,267568304	0,031	0,02	1	1,203777123	0,830718562	1,55
ARHGDIB	1,29854E-10	0,267390929	0,219	0,008	2,74005E-06	1,20362913	0,830820703	27,375

Supplementary Table 1

Differentially expressed genes_adjacent-skin-XL_ comparing SC-Keloid vs. (SC-Myel, SC-Nonmyel)								
**gene**	**p_val**	**avg_log2FC**	**pct.1**	**pct.2**	**p_val_adj**	**Foldchange_UP**	**Foldchange_DOWN**	**Ratio_pct1_pct2**
DGCR6L	7,40549E-11	0,267383175	0,562	0,09	1,56263E-06	1,203622662	0,830825168	6,244444444
METTL5	6,46751E-10	0,267154376	0,531	0,09	1,36471E-05	1,203431793	0,83095694	5,9
PDE4A	2,11157E-07	0,267104506	0,219	0,02	0,00445563	1,203390194	0,830985664	10,95
CCDC34	1,99284E-10	0,267024776	0,281	0,02	4,2051E-06	1,203323691	0,83103159	14,05
THYN1	4,70256E-09	0,266571938	0,469	0,078	9,92287E-05	1,202946047	0,831292478	6,012820513
BBOX1	1,33967E-08	0,266369685	0,125	0	0,000282684	1,202777417	0,831409026	#DIV/0!
PSMC2	3,33903E-05	0,265849577	0,406	0,105	0,704567964	1,20234388	0,831708812	3,866666667
RND3	0,040432475	0,265060083	0,75	0,445	1	1,201686094	0,832164078	1,685393258
AIFM1	8,22802E-07	0,264937418	0,188	0,016	0,01736194	1,201583925	0,832234835	11,75
STK25	0,00011675	0,264928088	0,375	0,102	1	1,201576154	0,832240217	3,676470588
ELMOD1	6,3574E-05	0,264814075	0,062	0	1	1,201481201	0,83230599	#DIV/0!
TIMM23B	0,05712057	0,264670353	0,094	0,027	1	1,201361514	0,832388909	3,481481481
AMZ2	1,69117E-07	0,264520606	0,344	0,055	0,003568535	1,201236823	0,832475312	6,254545455
ZC3HC1	0,000486824	0,264506057	0,094	0,008	1	1,201224709	0,832483708	11,75
MCTS1	7,45803E-08	0,264412725	0,406	0,07	0,001573719	1,201147002	0,832537565	5,8
USP14	9,30016E-08	0,264324522	0,562	0,125	0,001962427	1,201073568	0,832588466	4,496
MICALL1	1,57978E-10	0,264098427	0,219	0,008	3,33348E-06	1,200885355	0,832718957	27,375
RPL5	0,103548686	0,26386582	0,938	0,746	1	1,20069175	0,832853228	1,257372654
TSPAN3	0,001215659	0,26376377	0,594	0,234	1	1,200606821	0,832912143	2,538461538
DDX11	4,36691E-05	0,2636901	0,094	0,004	0,921461827	1,200545515	0,832954676	23,5
PSMD9	9,51361E-07	0,263641304	0,594	0,152	0,02007467	1,20050491	0,832982849	3,907894737
MFSD3	1,96898E-07	0,263581921	0,219	0,02	0,00415474	1,200455496	0,833017136	10,95
PARD6A	1,33967E-08	0,263579644	0,125	0	0,000282684	1,200453602	0,833018451	#DIV/0!
CLTC	7,21599E-06	0,263566389	0,562	0,16	0,152264664	1,200442573	0,833026104	3,5125
MYEOV	4,09886E-14	0,263547785	0,219	0	8,649E-10	1,200427093	0,833036846	#DIV/0!
RPL30	0,014566126	0,263331247	1	0,883	1	1,200246931	0,833161889	1,132502831
ZNF544	1,02698E-07	0,263272101	0,188	0,012	0,002167028	1,200197725	0,833196047	15,66666667
TMX1	2,43867E-05	0,263236879	0,438	0,117	0,51458445	1,200168424	0,833216389	3,743589744
ERVMER34-1	2,86276E-12	0,263126483	0,188	0	6,04072E-08	1,20007659	0,833280149	#DIV/0!
CKS2	5,79556E-08	0,263043969	0,375	0,059	0,001222921	1,200007954	0,83332781	6,355932203

Supplementary Table 1

Differentially expressed genes_adjacent-skin-XL_ comparing SC-Keloid vs. (SC-Myel, SC-Nonmyel)								
**gene**	**p_val**	**avg_log2FC**	**pct.1**	**pct.2**	**p_val_adj**	**Foldchange_UP**	**Foldchange_DOWN**	**Ratio_pct1_pct2**
DCTN5	8,59347E-05	0,262782245	0,219	0,039	1	1,199790277	0,833479	5,615384615
IMP4	1,60198E-07	0,262652417	0,375	0,062	0,003380331	1,199682312	0,833554008	6,048387097
REEP6	0,000452457	0,262605308	0,125	0,016	1	1,19964314	0,833581227	7,8125
SEC62	0,000955886	0,262447343	0,875	0,391	1	1,199511794	0,833672503	2,237851662
COQ3	3,57983E-06	0,262426522	0,156	0,012	0,075538003	1,199494483	0,833684535	13
ADAMTS12	9,12354E-07	0,262416048	0,094	0	0,019251588	1,199485775	0,833690587	#DIV/0!
ST3GAL2	5,94561E-05	0,262233544	0,188	0,027	1	1,199334047	0,833796058	6,962962963
ARMC10	3,02618E-05	0,262145523	0,406	0,105	0,638555129	1,199260876	0,83384693	3,866666667
AIF1L	2,86075E-06	0,261265994	0,312	0,055	0,060364658	1,198529977	0,834355435	5,672727273
HS3ST1	1,96814E-10	0,261223087	0,156	0	4,15296E-06	1,198494332	0,834380249	#DIV/0!
YWHAZ	0,002852144	0,261161745	0,875	0,453	1	1,198443375	0,834415727	1,931567329
LPGAT1	1,35983E-06	0,261004797	0,531	0,137	0,028693682	1,198313006	0,834506506	3,875912409
NDUFB5	1,05193E-06	0,260970888	0,469	0,105	0,022196825	1,198284841	0,834526121	4,466666667
RNF150	0,002168448	0,260965428	0,062	0,004	1	1,198280306	0,834529279	15,5
EIF3G	0,00029595	0,260923282	0,781	0,309	1	1,198245301	0,834553659	2,527508091
HEATR1	0,001808729	0,260670401	0,156	0,031	1	1,198035286	0,834699955	5,032258065
HMGN4	6,30011E-07	0,260651828	0,344	0,059	0,013293862	1,198019863	0,834710701	5,830508475
MRPS7	9,80955E-10	0,260289879	0,5	0,082	2,06991E-05	1,197719337	0,834920143	6,097560976
GPX8	1,86296E-06	0,260254321	0,438	0,098	0,03931032	1,197689817	0,834940721	4,469387755
LEO1	1,17927E-11	0,26023504	0,344	0,027	2,48837E-07	1,197673811	0,83495188	12,74074074
FBXO21	3,59278E-05	0,260103412	0,375	0,094	0,758112671	1,197564542	0,835028063	3,989361702
CSE1L	4,70556E-08	0,259606028	0,438	0,078	0,00099292	1,197151741	0,835315997	5,615384615
SPATC1L	1,38853E-07	0,259551468	0,312	0,043	0,002929933	1,197106468	0,835347588	7,255813953
CDC37L1	2,30501E-05	0,25936821	0,344	0,078	0,486379331	1,196954415	0,835453704	4,41025641
P4HA3	1,33967E-08	0,25932721	0,125	0	0,000282684	1,196920399	0,835477447	#DIV/0!
TMEM250	9,0771E-08	0,25882614	0,188	0,012	0,001915359	1,196504763	0,835767671	15,66666667
DDX18	0,000130369	0,258794396	0,75	0,281	1	1,196478436	0,835786061	2,669039146
GNG7	5,59696E-07	0,258726637	0,281	0,039	0,01181015	1,196422243	0,835825317	7,205128205
GFUS	2,03458E-08	0,25865502	0,375	0,055	0,000429317	1,196362852	0,835866809	6,818181818
FBH1	0,005194433	0,25848939	0,219	0,066	1	1,196225511	0,835962777	3,318181818

Supplementary Table 1

Differentially expressed genes_adjacent-skin-XL_ comparing SC-Keloid vs. (SC-Myel, SC-Nonmyel)								
**gene**	**p_val**	**avg_log2FC**	**pct.1**	**pct.2**	**p_val_adj**	**Foldchange_UP**	**Foldchange_DOWN**	**Ratio_pct1_pct2**
PIGU	3,64929E-07	0,258341514	0,312	0,047	0,007700373	1,196102904	0,836048467	6,638297872
DENR	6,77744E-08	0,258279396	0,5	0,102	0,001430107	1,196051405	0,836084466	4,901960784
POLDIP2	8,77574E-09	0,258045703	0,312	0,035	0,000185177	1,195857679	0,836219909	8,914285714
DOCK2	2,86276E-12	0,257886988	0,188	0	6,04072E-08	1,195726127	0,836311909	#DIV/0!
RAI1	0,000290171	0,257581141	0,156	0,023	1	1,195472664	0,836489223	6,782608696
HSPBP1	6,72473E-09	0,25745552	0,344	0,043	0,000141898	1,195368574	0,836562063	8
TMEM14C	6,1983E-05	0,257423463	0,531	0,164	1	1,195342013	0,836580652	3,237804878
IDH1	5,01789E-11	0,257282535	0,312	0,023	1,05883E-06	1,195225253	0,836662376	13,56521739
CACNA2D4	2,86577E-08	0,257042616	0,219	0,016	0,000604707	1,195026504	0,836801524	13,6875
ZNF138	0,00703765	0,256972271	0,125	0,027	1	1,194968237	0,836842327	4,62962963
PDCL3	3,30101E-08	0,256808039	0,344	0,047	0,000696546	1,194832213	0,836937596	7,319148936
BMERB1	3,79454E-10	0,256472865	0,375	0,043	8,00686E-06	1,194554656	0,83713206	8,720930233
INSYN2A	1,33967E-08	0,256324217	0,125	0	0,000282684	1,194431582	0,837218318	#DIV/0!
TRMO	2,86577E-08	0,256126905	0,219	0,016	0,000604707	1,194268235	0,837332829	13,6875
SEPTIN2	0,022059085	0,25602693	0,75	0,512	1	1,194185478	0,837390856	1,46484375
MAP4	0,027371938	0,255801834	0,906	0,52	1	1,19399917	0,83752152	1,742307692
KCNC2	6,3574E-05	0,255712355	0,062	0	1	1,193925118	0,837573466	#DIV/0!
POLR3H	1,03471E-06	0,255680348	0,219	0,023	0,021833489	1,19389863	0,837592049	9,52173913
CHKB	0,007888732	0,255609121	0,312	0,117	1	1,193839689	0,837633402	2,666666667
PSMB5	0,000230571	0,255355154	0,562	0,199	1	1,193629548	0,837780869	2,824120603
GPATCH1	0,081392519	0,255213129	0,062	0,016	1	1,193512048	0,837863348	3,875
LHFPL6	2,27444E-08	0,255151971	0,469	0,086	0,000479931	1,193461454	0,837898867	5,453488372
TRADD	1,56846E-09	0,254937445	0,281	0,023	3,30962E-05	1,193284001	0,838023471	12,2173913
MAPK3	1,15611E-08	0,254559367	0,406	0,062	0,000243951	1,192971326	0,838243115	6,548387097
POLR2C	8,75711E-07	0,25436887	0,406	0,082	0,018478373	1,192813814	0,838353806	4,951219512
ABHD16A	9,25608E-07	0,254316379	0,312	0,051	0,019531255	1,192770415	0,838384309	6,117647059
ACLY	2,22056E-06	0,253958545	0,312	0,055	0,046856037	1,192474607	0,83859228	5,672727273
CABIN1	0,000362786	0,253932025	0,25	0,059	1	1,192452687	0,838607696	4,237288136
NIP7	4,33123E-08	0,253684773	0,25	0,023	0,000913933	1,192248339	0,838751431	10,86956522
SRSF9	0,000822741	0,253591559	0,594	0,238	1	1,192171309	0,838805625	2,495798319

Supplementary Table 1

Differentially expressed genes_adjacent-skin-XL_ comparing SC-Keloid vs. (SC-Myel, SC-Nonmyel)								
**gene**	**p_val**	**avg_log2FC**	**pct.1**	**pct.2**	**p_val_adj**	**Foldchange_UP**	**Foldchange_DOWN**	**Ratio_pct1_pct2**
ACTR1A	4,2816E-07	0,253591547	0,469	0,102	0,009034597	1,1921713	0,838805632	4,598039216
DIRAS1	4,09886E-14	0,253274426	0,219	0	8,649E-10	1,191909275	0,838990031	#DIV/0!
SMIM15	1,42389E-09	0,253227206	0,406	0,055	3,00454E-05	1,191870264	0,839017492	7,381818182
TP53I13	8,59395E-08	0,253146354	0,625	0,145	0,00181341	1,191803471	0,839064514	4,310344828
ATP6V0D1	5,15424E-07	0,253123658	0,438	0,09	0,010875957	1,191784722	0,839077714	4,866666667
MEOX1	6,3574E-05	0,252927537	0,062	0	1	1,191622721	0,839191786	#DIV/0!
SNF8	1,93845E-05	0,252516307	0,562	0,168	0,409031717	1,191283106	0,839431026	3,345238095
EI24	7,10867E-06	0,252464496	0,562	0,16	0,150000027	1,191240324	0,839461173	3,5125
MED10	3,31861E-06	0,25228736	0,594	0,164	0,070026036	1,191094071	0,839564249	3,62195122
RBBP4	1,71496E-06	0,252233596	0,625	0,176	0,036187473	1,191049684	0,839595538	3,551136364
TIMM17A	2,23409E-07	0,252185039	0,406	0,074	0,004714163	1,191009598	0,839623796	5,486486486
PRAG1	3,57983E-06	0,251854438	0,156	0,012	0,075538003	1,190736703	0,839816223	13
MOGS	2,11052E-07	0,25165503	0,25	0,027	0,004453418	1,190572131	0,83993231	9,259259259
PSMG3	6,03985E-05	0,251599668	0,281	0,059	1	1,190526446	0,839964541	4,762711864
CSNK1G3	3,65452E-05	0,251563127	0,375	0,094	0,771140872	1,190496292	0,839985817	3,989361702
PDHA1	0,000182503	0,250921218	0,25	0,055	1	1,189966713	0,840359641	4,545454545
ETV4	1,96814E-10	0,250818879	0,156	0	4,15296E-06	1,189882305	0,840419255	#DIV/0!
EFCAB2	2,33183E-10	0,250805791	0,281	0,02	4,92039E-06	1,18987151	0,840426879	14,05
MEA1	1,2796E-07	0,250763737	0,312	0,043	0,002700092	1,189836826	0,840451378	7,255813953
KIF13B	8,4498E-07	0,250706395	0,438	0,094	0,017829924	1,189789536	0,840484783	4,659574468
LIMD2	1,30636E-05	0,250647169	0,219	0,031	0,275655185	1,189740693	0,840519288	7,064516129
ITGA5	4,75304E-07	0,250509962	0,281	0,039	0,010029393	1,189627549	0,840599229	7,205128205
WDR46	9,10679E-05	0,250443727	0,25	0,051	1	1,189572934	0,840637822	4,901960784
HOGA1	4,09886E-14	0,250187043	0,219	0	8,649E-10	1,189361304	0,840787401	#DIV/0!
RNF128	4,27234E-12	0,250081005	0,219	0,004	9,01506E-08	1,189273889	0,840849202	54,75
MSRB1	4,03265E-08	0,249587858	0,312	0,039	0,000850929	1,188867437	0,841136673	8
RBM3	0,024130258	0,249580873	0,719	0,438	1	1,18886168	0,841140745	1,641552511
ARPIN	8,59347E-05	0,249456085	0,219	0,039	1	1,188758853	0,841213504	5,615384615
CNFN	0,00737039	0,24944426	0,094	0,016	1	1,188749109	0,841220399	5,875
PSMA5	7,85583E-06	0,249397106	0,594	0,168	0,165765954	1,188710256	0,841247895	3,535714286

Supplementary Table 1

Differentially expressed genes_adjacent-skin-XL_ comparing SC-Keloid vs. (SC-Myel, SC-Nonmyel)								
**gene**	**p_val**	**avg_log2FC**	**pct.1**	**pct.2**	**p_val_adj**	**Foldchange_UP**	**Foldchange_DOWN**	**Ratio_pct1_pct2**
BDH2	0,001492226	0,249217879	0,406	0,141	1	1,188562591	0,84135241	2,879432624
HTRA2	0,001066258	0,249014675	0,312	0,094	1	1,188395193	0,841470923	3,319148936
INO80C	1,01508E-08	0,248909881	0,281	0,027	0,000214192	1,188308874	0,841532048	10,40740741
RCAN1	0,000729331	0,248883038	0,906	0,477	1	1,188286764	0,841547706	1,899371069
RPE	6,40895E-07	0,248867643	0,281	0,039	0,013523521	1,188274084	0,841556686	7,205128205
SAMD1	7,07015E-08	0,248864754	0,375	0,059	0,001491872	1,188271705	0,841558371	6,355932203
RNASEH2B	4,15798E-09	0,24846155	0,438	0,066	8,77376E-05	1,187939654	0,841793602	6,636363636
DDX1	8,95258E-06	0,248398591	0,469	0,121	0,188908433	1,187887813	0,841830339	3,876033058
LRP12	0,001037136	0,248346551	0,25	0,066	1	1,187844965	0,841860705	3,787878788
SNX7	5,39891E-08	0,248224149	0,5	0,098	0,001139224	1,187744189	0,841932134	5,102040816
UPRT	3,5572E-05	0,248158573	0,219	0,035	0,75060449	1,187690203	0,841970404	6,257142857
SLC35C2	1,69331E-06	0,248033231	0,281	0,043	0,035730553	1,187587021	0,842043558	6,534883721
C18orf32	0,000163694	0,247875675	0,594	0,207	1	1,187457332	0,842135522	2,869565217
HSD17B11	1,75258E-09	0,247765833	0,375	0,047	3,69813E-05	1,187366926	0,842199642	7,978723404
SH3BGR	1,71675E-09	0,247601459	0,469	0,074	3,62252E-05	1,187231651	0,842295603	6,337837838
COQ8B	1,58275E-07	0,247570514	0,281	0,035	0,00333977	1,187206186	0,84231367	8,028571429
MRTO4	1,21163E-07	0,247534715	0,312	0,043	0,002556662	1,187176726	0,842334572	7,255813953
LMNB2	0,001625689	0,247531888	0,156	0,031	1	1,1871744	0,842336223	5,032258065
FAM229B	1,3835E-07	0,247504606	0,469	0,094	0,002919331	1,18715195	0,842352152	4,989361702
ARSA	0,001935026	0,247359452	0,25	0,07	1	1,187032514	0,842436907	3,571428571
ARHGAP1	6,15428E-07	0,246947461	0,344	0,059	0,012986147	1,186693581	0,842677517	5,830508475
AIFM2	2,03907E-07	0,24692012	0,219	0,02	0,004302644	1,186671092	0,842693487	10,95
UBE2T	2,81241E-07	0,246746664	0,156	0,008	0,005934461	1,186528426	0,842794811	19,5
RAP1GDS1	0,000874229	0,246651578	0,375	0,121	1	1,186450226	0,84285036	3,099173554
COPS4	1,31496E-07	0,246641858	0,312	0,043	0,002774696	1,186442233	0,842856038	7,255813953
NRSN2	2,37592E-09	0,246567239	0,312	0,031	5,01343E-05	1,186380869	0,842899633	10,06451613
UQCRFS1	3,99577E-07	0,246273561	0,5	0,109	0,008431478	1,186139392	0,843071233	4,587155963
THEM4	1,33352E-05	0,246077289	0,312	0,062	0,281386118	1,185978034	0,843185937	5,032258065
GNA11	9,51595E-05	0,246031796	0,375	0,102	1	1,185940637	0,843212526	3,676470588
CNPPD1	0,004239973	0,245974988	0,281	0,094	1	1,18589394	0,843245729	2,989361702

Supplementary Table 1

Differentially expressed genes_adjacent-skin-XL_ comparing SC-Keloid vs. (SC-Myel, SC-Nonmyel)								
**gene**	**p_val**	**avg_log2FC**	**pct.1**	**pct.2**	**p_val_adj**	**Foldchange_UP**	**Foldchange_DOWN**	**Ratio_pct1_pct2**
CCDC91	2,55882E-05	0,245867025	0,312	0,066	0,539936516	1,185805198	0,843308835	4,727272727
HOXB5	6,3574E-05	0,245842191	0,062	0	1	1,185784785	0,843323352	#DIV/0!
RRAD	0,025463646	0,245768333	0,188	0,066	1	1,185724081	0,843366527	2,848484848
SARNP	5,5641E-05	0,245523372	0,688	0,238	1	1,18552277	0,843509737	2,890756303
RFC5	5,90558E-11	0,245445872	0,25	0,012	1,24614E-06	1,185459086	0,843555051	20,83333333
GASK1B	8,23459E-06	0,245418044	0,406	0,094	0,173758165	1,18543622	0,843571323	4,319148936
PFDN6	0,000109253	0,245025988	0,344	0,09	1	1,185114119	0,843800596	3,822222222
IFRD2	5,23791E-05	0,244932163	0,312	0,07	1	1,185037048	0,843855474	4,457142857
ARL2BP	1,17879E-05	0,244807781	0,594	0,18	0,248737486	1,184934884	0,84392823	3,3
JOSD2	4,35448E-06	0,244710267	0,625	0,176	0,091883945	1,184854796	0,843985275	3,551136364
METTL26	0,000184234	0,244654248	0,594	0,207	1	1,184808789	0,844018047	2,869565217
DYNC1I2	0,00124038	0,243842782	0,781	0,359	1	1,184142563	0,844492911	2,175487465
GNL2	0,000102664	0,243527895	0,281	0,062	1	1,183884136	0,844677253	4,532258065
VDR	0,000486824	0,24342488	0,094	0,008	1	1,183799605	0,844737569	11,75
PPP2R3C	2,95926E-08	0,243304733	0,344	0,047	0,000624432	1,183701022	0,844807921	7,319148936
HMBS	1,50434E-10	0,243257849	0,219	0,008	3,17431E-06	1,183662556	0,844835376	27,375
SLC49A3	7,96166E-09	0,243177173	0,188	0,008	0,000167999	1,183596367	0,844882621	23,5
PRPF31	3,63521E-08	0,242853588	0,406	0,066	0,000767065	1,183330926	0,845072142	6,151515152
TP53TG1	0,000182503	0,24282753	0,25	0,055	1	1,183309552	0,845087406	4,545454545
TOR1AIP1	2,23409E-07	0,242554661	0,406	0,074	0,004714163	1,183085764	0,84524726	5,486486486
TINF2	0,000273883	0,242306984	0,312	0,082	1	1,182882673	0,845392381	3,804878049
C14orf132	7,47389E-09	0,242165179	0,25	0,02	0,000157707	1,182766411	0,845475481	12,5
LYRM2	8,93857E-07	0,242131553	0,406	0,082	0,018861285	1,182738844	0,845495187	4,951219512
MPND	2,70596E-09	0,24195763	0,219	0,012	5,70985E-05	1,182596268	0,845597121	18,25
NOC2L	6,59509E-05	0,241852302	0,375	0,098	1	1,182509933	0,845658858	3,826530612
SURF2	2,22228E-07	0,241640268	0,344	0,055	0,004689228	1,182336151	0,845783155	6,254545455
XRCC5	0,000291348	0,241409277	0,781	0,312	1	1,182146862	0,845918585	2,503205128
TTC4	9,7095E-07	0,241374925	0,219	0,023	0,020488018	1,182118714	0,845938727	9,52173913
OCEL1	1,08519E-08	0,241235896	0,281	0,027	0,000228985	1,182004802	0,846020252	10,40740741
CCDC90B	0,000465198	0,241051441	0,469	0,16	1	1,181853687	0,846128426	2,93125

Supplementary Table 1

Differentially expressed genes_adjacent-skin-XL_ comparing SC-Keloid vs. (SC-Myel, SC-Nonmyel)								
**gene**	**p_val**	**avg_log2FC**	**pct.1**	**pct.2**	**p_val_adj**	**Foldchange_UP**	**Foldchange_DOWN**	**Ratio_pct1_pct2**
NUDT9	0,001590978	0,240904878	0,281	0,086	1	1,181733628	0,846214389	3,26744186
MRPL12	3,38123E-08	0,240858617	0,406	0,066	0,000713474	1,181695736	0,846241523	6,151515152
EIF2B2	6,40752E-06	0,240799527	0,375	0,082	0,135204977	1,181647337	0,846276184	4,573170732
RANGRF	3,36842E-06	0,240670997	0,344	0,066	0,071077053	1,181542068	0,846351583	5,212121212
CDK1	6,3574E-05	0,240583745	0,062	0	1	1,181470613	0,84640277	#DIV/0!
PRKAR2A	0,00051005	0,240518728	0,344	0,102	1	1,18141737	0,846440915	3,37254902
PPOX	0,036309636	0,240476587	0,125	0,039	1	1,181382861	0,846465641	3,205128205
DDRGK1	1,84398E-06	0,240464927	0,469	0,109	0,038909776	1,181373313	0,846472482	4,302752294
RPL18	0,048110318	0,240320619	1	0,836	1	1,18125515	0,846557156	1,196172249
SMC1A	7,66099E-06	0,240258165	0,562	0,16	0,16165448	1,181204015	0,846593804	3,5125
TBC1D22A	5,94561E-05	0,240121294	0,188	0,027	1	1,181091957	0,846674125	6,962962963
CBY1	0,00030797	0,240022309	0,281	0,07	1	1,181010923	0,846732219	4,014285714
USE1	4,13472E-05	0,239862093	0,25	0,047	0,872467404	1,180879776	0,846826256	5,319148936
STING1	1,4529E-06	0,239743679	0,281	0,043	0,030657605	1,180782855	0,846895766	6,534883721
CFAP97	0,000159854	0,239291876	0,594	0,207	1	1,180413132	0,847161026	2,869565217
CPXM1	2,86276E-12	0,238785385	0,188	0	6,04072E-08	1,179998794	0,847458493	#DIV/0!
TOMM5	1,54008E-05	0,238653883	0,625	0,199	0,32497171	1,179891242	0,847535743	3,140703518
ANKRD6	1,17365E-05	0,238653329	0,219	0,031	0,247652593	1,179890789	0,847536068	7,064516129
TMEM60	3,9608E-08	0,238511234	0,281	0,031	0,000835768	1,179774584	0,847619549	9,064516129
P4HTM	0,00037456	0,23846655	0,406	0,125	1	1,179738043	0,847645802	3,248
NME3	0,000697293	0,238428184	0,719	0,289	1	1,179706671	0,847668344	2,487889273
GFER	2,41218E-06	0,238356202	0,25	0,035	0,050899452	1,179647812	0,847710639	7,142857143
MUL1	1,83571E-07	0,23806812	0,219	0,02	0,003873533	1,17941228	0,847879929	10,95
ETV5	4,5807E-05	0,238048923	0,094	0,004	0,966573495	1,179396586	0,847891211	23,5
USP45	0,000778441	0,238026504	0,188	0,039	1	1,179378259	0,847904388	4,820512821
GTF2H4	4,74973E-05	0,237978957	0,281	0,059	1	1,179339391	0,847932332	4,762711864
ZNF101	9,12354E-07	0,237917302	0,094	0	0,019251588	1,179288992	0,84796857	#DIV/0!
FOCAD	0,002370322	0,237671075	0,094	0,012	1	1,179087737	0,848113307	7,833333333
HSPA8	0,001881736	0,237633338	1	0,676	1	1,179056896	0,848135492	1,479289941
CDR2L	1,64657E-05	0,236829145	0,188	0,023	0,347443783	1,178399844	0,848608395	8,173913043

Supplementary Table 1

Differentially expressed genes_adjacent-skin-XL_ comparing SC-Keloid vs. (SC-Myel, SC-Nonmyel)								
**gene**	**p_val**	**avg_log2FC**	**pct.1**	**pct.2**	**p_val_adj**	**Foldchange_UP**	**Foldchange_DOWN**	**Ratio_pct1_pct2**
ALDOA	3,66632E-06	0,236784676	0,344	0,066	0,077363122	1,178363522	0,848634552	5,212121212
UCHL5	2,34772E-06	0,236661668	0,25	0,035	0,049539231	1,178263056	0,848706912	7,142857143
LDHA	0,000867274	0,236638321	0,875	0,414	1	1,178243989	0,848720646	2,11352657
HSPB3	2,52232E-10	0,236552509	0,188	0,004	5,32235E-06	1,178173909	0,84877113	47
CD81	0,038034898	0,236462312	0,969	0,84	1	1,178100251	0,848824197	1,153571429
NDUFS3	4,60659E-07	0,236410199	0,562	0,137	0,009720359	1,178057697	0,848854859	4,102189781
SPATS2L	0,000285018	0,236248727	0,719	0,277	1	1,177925852	0,848949871	2,59566787
RAB3D	0,081392519	0,235983285	0,062	0,016	1	1,177709145	0,849106084	3,875
GAL3ST4	2,97902E-08	0,235929683	0,219	0,016	0,000628603	1,177665389	0,849137632	13,6875
SDC1	6,26921E-05	0,235709763	0,188	0,027	1	1,177485882	0,849267082	6,962962963
CCT8	0,000102655	0,235702864	0,531	0,172	1	1,177480252	0,849271143	3,087209302
POLR3D	4,56192E-05	0,235637506	0,281	0,059	0,962611179	1,17742691	0,849309618	4,762711864
CEP112	0,003651243	0,235585556	0,125	0,023	1	1,177384513	0,849340202	5,434782609
LAMTOR1	0,000257417	0,235404595	0,625	0,223	1	1,17723684	0,849446743	2,802690583
STK11	0,001515665	0,235291119	0,312	0,098	1	1,177144247	0,84951356	3,183673469
NUCKS1	0,055411361	0,235269069	0,969	0,684	1	1,177126256	0,849526544	1,416666667
JMJD8	0,000105708	0,235023902	0,344	0,09	1	1,176926236	0,849670922	3,822222222
IGBP1	4,5658E-07	0,234985644	0,438	0,09	0,009634302	1,176895026	0,849693454	4,866666667
KLHL9	1,73693E-06	0,234978287	0,281	0,043	0,036650933	1,176889024	0,849697787	6,534883721
RPS27A	0,032662414	0,234960867	1	0,898	1	1,176874814	0,849708047	1,113585746
MACO1	1,04547E-06	0,2349574	0,312	0,051	0,022060425	1,176871986	0,849710089	6,117647059
PTPN14	1,70371E-07	0,234899356	0,531	0,117	0,003594989	1,176824638	0,849744276	4,538461538
PTPA	3,62649E-11	0,234798853	0,562	0,086	7,65225E-07	1,176742659	0,849803475	6,534883721
ULBP2	2,86276E-12	0,234773699	0,188	0	6,04072E-08	1,176722142	0,849818291	#DIV/0!
TMEM150A	0,001444236	0,234460645	0,125	0,02	1	1,17646683	0,850002715	6,25
MMP17	7,86208E-09	0,234311855	0,562	0,113	0,000165898	1,176345503	0,850090384	4,973451327
COL9A2	0,013108072	0,234267243	0,125	0,031	1	1,176309128	0,850116671	4,032258065
TBL2	0,007479227	0,234241203	0,125	0,027	1	1,176287897	0,850132015	4,62962963
ZNF420	0,000466094	0,232778715	0,125	0,016	1	1,175096075	0,850994247	7,8125
SRRT	0,005168596	0,232619501	0,281	0,098	1	1,1749664	0,851088167	2,867346939

Supplementary Table 1

Differentially expressed genes_adjacent-skin-XL_ comparing SC-Keloid vs. (SC-Myel, SC-Nonmyel)								
**gene**	**p_val**	**avg_log2FC**	**pct.1**	**pct.2**	**p_val_adj**	**Foldchange_UP**	**Foldchange_DOWN**	**Ratio_pct1_pct2**
CENPM	9,12354E-07	0,232418798	0,094	0	0,019251588	1,174802954	0,851206576	#DIV/0!
PCGF2	0,001066258	0,232002471	0,312	0,094	1	1,174463983	0,851452249	3,319148936
GDE1	8,16984E-11	0,231921337	0,438	0,055	1,72392E-06	1,174397935	0,851500135	7,963636364
CLNS1A	2,28454E-07	0,231708623	0,594	0,141	0,004820618	1,174224792	0,851625691	4,212765957
RNF182	1,96814E-10	0,231646752	0,156	0	4,15296E-06	1,174174436	0,851662214	#DIV/0!
POLR3K	0,000297876	0,2314477	0,156	0,023	1	1,174012443	0,851779728	6,782608696
CCL28	0,007186669	0,231426748	0,094	0,016	1	1,173995394	0,851792098	5,875
THAP8	1,96814E-10	0,231173265	0,156	0	4,15296E-06	1,173789139	0,851941773	#DIV/0!
FAM50B	2,10199E-05	0,231134774	0,156	0,016	0,443540004	1,173757824	0,851964502	9,75
GET3	5,47524E-07	0,230557953	0,438	0,09	0,0115533	1,173288624	0,852305204	4,866666667
TRABD	2,22437E-05	0,230502452	0,281	0,055	0,469363894	1,173243487	0,852337994	5,109090909
SRD5A1	2,33183E-10	0,230414951	0,281	0,02	4,92039E-06	1,173172331	0,85238969	14,05
DENND6B	0,000755915	0,230232963	0,156	0,027	1	1,173024351	0,852497222	5,777777778
NR1H2	4,34527E-06	0,230214401	0,469	0,117	0,091689461	1,173009259	0,85250819	4,008547009
ADA2	8,22802E-07	0,230115891	0,188	0,016	0,01736194	1,172929166	0,852566403	11,75
IGFBP6	1,07001E-07	0,230061611	0,188	0,012	0,002257818	1,172885037	0,85259848	15,66666667
INO80B	0,003403052	0,229986712	0,219	0,062	1	1,172824147	0,852642745	3,532258065
SOX11	9,12354E-07	0,229676854	0,094	0	0,019251588	1,172572278	0,852825893	#DIV/0!
TRAK2	1,11223E-05	0,229602188	0,219	0,031	0,234690635	1,172511594	0,852870032	7,064516129
RPF1	5,2276E-12	0,229160032	0,406	0,039	1,10307E-07	1,172152298	0,853131459	10,41025641
TWF1	1,03141E-06	0,228842149	0,406	0,082	0,021763787	1,171894055	0,853319458	4,951219512
PRPF19	2,54624E-06	0,228831989	0,25	0,035	0,053728244	1,171885802	0,853325468	7,142857143
CIAPIN1	8,24168E-09	0,228645209	0,344	0,043	0,000173908	1,171734092	0,853435952	8
EXOSC1	0,00287441	0,228229262	0,188	0,047	1	1,171396316	0,853682043	4
COQ10B	0,000281007	0,228099754	0,406	0,125	1	1,171291166	0,85375868	3,248
ENDOV	6,8971E-08	0,228085727	0,375	0,059	0,001455356	1,171279778	0,853766981	6,355932203
RPS28	0,045384033	0,228027612	1	0,898	1	1,171232597	0,853801373	1,113585746
SH2B3	9,40504E-07	0,228025278	0,219	0,023	0,019845575	1,171230703	0,853802754	9,52173913
MAP9	1,28694E-06	0,228015915	0,344	0,062	0,027155825	1,171223102	0,853808296	5,548387097
PRPF40A	0,000192736	0,227324242	0,594	0,211	1	1,170661715	0,854217736	2,815165877

Supplementary Table 1

Differentially expressed genes_adjacent-skin-XL_ comparing SC-Keloid vs. (SC-Myel, SC-Nonmyel)								
**gene**	**p_val**	**avg_log2FC**	**pct.1**	**pct.2**	**p_val_adj**	**Foldchange_UP**	**Foldchange_DOWN**	**Ratio_pct1_pct2**
ALG6	1,27946E-05	0,227057428	0,312	0,062	0,269978646	1,170445231	0,854375731	5,032258065
TRAM2	6,78692E-06	0,226879125	0,375	0,082	0,143210806	1,170300584	0,85448133	4,573170732
KCTD17	1,56846E-09	0,22664934	0,281	0,023	3,30962E-05	1,1701142	0,854617438	12,2173913
RABIF	1,36009E-12	0,226120353	0,281	0,012	2,86993E-08	1,169685238	0,854930854	23,41666667
SP110	0,003590182	0,225934847	0,156	0,035	1	1,169534846	0,855040791	4,457142857
RAB12	1,2136E-05	0,225815248	0,281	0,051	0,256081924	1,169437896	0,855111677	5,509803922
ACAT2	2,7063E-07	0,225802459	0,344	0,055	0,00571057	1,169427529	0,855119257	6,254545455
RFTN1	0,002370322	0,225794624	0,094	0,012	1	1,169421178	0,855123901	7,833333333
CHST11	3,57983E-06	0,225446016	0,156	0,012	0,075538003	1,169138638	0,855330555	13
RTN2	1,96814E-10	0,225408885	0,156	0	4,15296E-06	1,169108548	0,855352569	#DIV/0!
NHLRC3	0,000205019	0,225172248	0,25	0,055	1	1,168916801	0,85549288	4,545454545
PIK3R4	0,007631848	0,225002425	0,125	0,027	1	1,168779213	0,855593588	4,62962963
CDIPT	1,69566E-06	0,224968625	0,438	0,098	0,03578021	1,168751831	0,855613633	4,469387755
OGFOD3	2,43556E-06	0,224873557	0,312	0,055	0,051392661	1,168674817	0,855670017	5,672727273
MRM3	8,65685E-05	0,224760272	0,156	0,02	1	1,168583052	0,855737209	7,8
GLRX2	0,003712381	0,224416387	0,25	0,078	1	1,168304539	0,855941209	3,205128205
TMEM258	0,02145433	0,22437319	0,875	0,566	1	1,168269559	0,855966838	1,545936396
POLE3	9,13181E-09	0,2243661	0,438	0,07	0,00019269	1,168263817	0,855971045	6,257142857
FAM210B	6,61533E-07	0,224180221	0,594	0,148	0,013959011	1,168113306	0,856081336	4,013513514
PRDM10	0,000112833	0,224120272	0,125	0,012	1	1,168064768	0,85611691	10,41666667
SEMA4D	0,003660239	0,224110608	0,156	0,035	1	1,168056944	0,856122645	4,457142857
REXO2	1,34284E-05	0,223984053	0,594	0,172	0,283351994	1,167954485	0,856197748	3,453488372
GRB10	4,64456E-05	0,223909451	0,344	0,082	0,980048497	1,167894091	0,856242024	4,195121951
HOXB8	2,86276E-12	0,22355314	0,188	0	6,04072E-08	1,167605684	0,856453521	#DIV/0!
RAD23B	0,001005729	0,223536834	0,531	0,203	1	1,167592488	0,856463201	2,615763547
EFR3B	0,000169271	0,22347887	0,188	0,031	1	1,167545578	0,856497612	6,064516129
ITPK1	0,00078188	0,223434068	0,625	0,258	1	1,167509321	0,856524211	2,42248062
BEX1	0,169508224	0,223340091	0,094	0,039	1	1,167433272	0,856580007	2,41025641
RAB18	3,22581E-05	0,222994297	0,562	0,176	0,680677248	1,167153488	0,856785342	3,193181818
PPP1R37	2,33183E-10	0,222993153	0,281	0,02	4,92039E-06	1,167152563	0,856786021	14,05

Supplementary Table 1

Differentially expressed genes_adjacent-skin-XL_ comparing SC-Keloid vs. (SC-Myel, SC-Nonmyel)								
**gene**	**p_val**	**avg_log2FC**	**pct.1**	**pct.2**	**p_val_adj**	**Foldchange_UP**	**Foldchange_DOWN**	**Ratio_pct1_pct2**
STOML2	0,000113152	0,222915206	0,375	0,102	1	1,167089505	0,856832313	3,676470588
TRAPPC6A	0,00082778	0,222857761	0,188	0,039	1	1,167043035	0,856866431	4,820512821
AC097381.1	0,079594089	0,222807451	0,031	0,004	1	1,167002338	0,856896312	7,75
DDR1	0,000528986	0,222731247	0,406	0,133	1	1,166940698	0,856941575	3,052631579
CSNK1G2	3,80295E-07	0,22253909	0,438	0,09	0,008024611	1,166785279	0,857055722	4,866666667
ZDHHC13	0,002168448	0,222122871	0,062	0,004	1	1,166448709	0,857303019	15,5
TRPV4	1,33967E-08	0,221817429	0,125	0	0,000282684	1,166201779	0,857484543	#DIV/0!
STYXL1	2,41715E-14	0,221760791	0,312	0,012	5,10043E-10	1,166155997	0,857518207	26
SMIM12	8,43723E-08	0,221680318	0,438	0,082	0,001780339	1,166090951	0,857566041	5,341463415
PIAS2	5,30076E-07	0,221490985	0,281	0,039	0,011185134	1,165937928	0,857678591	7,205128205
STXBP4	9,73699E-05	0,221344395	0,156	0,02	1	1,165819465	0,857765743	7,8
CLN5	0,000133984	0,221212249	0,438	0,129	1	1,165712685	0,857844315	3,395348837
LMLN	1,57978E-10	0,221039614	0,219	0,008	3,33348E-06	1,165573203	0,857946972	27,375
SP2	0,010532596	0,220943546	0,156	0,043	1	1,16549559	0,858004104	3,627906977
TSNAX	3,12413E-07	0,220657802	0,438	0,086	0,006592226	1,165264772	0,85817406	5,093023256
RAB2A	0,001297108	0,220509468	0,875	0,395	1	1,165144969	0,858262299	2,215189873
RPAP2	0,010194	0,22036533	0,156	0,043	1	1,165028566	0,858348052	3,627906977
ADSS1	0,000486824	0,22027881	0,094	0,008	1	1,164958701	0,858399529	11,75
POP4	2,33183E-10	0,220076655	0,281	0,02	4,92039E-06	1,164795474	0,85851982	14,05
CHD9	1,31206E-05	0,21984917	0,844	0,297	0,276858651	1,164611823	0,858655202	2,841750842
IMPDH1	6,43857E-09	0,219745837	0,25	0,02	0,00013586	1,164528411	0,858716705	12,5
HMCN1	1,36009E-12	0,219634271	0,281	0,012	2,86993E-08	1,164438359	0,858783114	23,41666667
AHNAK2	7,47389E-09	0,219238008	0,25	0,02	0,000157707	1,164118569	0,859019027	12,5
WDR41	1,67748E-07	0,219159063	0,281	0,035	0,00353966	1,164054869	0,859066034	8,028571429
S100A9	0,00737039	0,219081274	0,094	0,016	1	1,163992106	0,859112355	5,875
ALDH7A1	5,36615E-07	0,218568911	0,438	0,09	0,011323121	1,163578796	0,859417518	4,866666667
LRP10	4,24827E-05	0,21852948	0,625	0,203	0,896427428	1,163546993	0,859441007	3,078817734
PTMA	0,021331787	0,218443967	1	0,965	1	1,163478029	0,85949195	1,03626943
ELP4	0,000774932	0,218161481	0,219	0,051	1	1,163250237	0,859660259	4,294117647
NPTN	2,06076E-07	0,21813478	0,625	0,156	0,00434842	1,163228708	0,85967617	4,006410256

Supplementary Table 1

Differentially expressed genes_adjacent-skin-XL_ comparing SC-Keloid vs. (SC-Myel, SC-Nonmyel)								
**gene**	**p_val**	**avg_log2FC**	**pct.1**	**pct.2**	**p_val_adj**	**Foldchange_UP**	**Foldchange_DOWN**	**Ratio_pct1_pct2**
BMS1	4,56192E-05	0,218049594	0,281	0,059	0,962611179	1,163160026	0,859726932	4,762711864
COMMD8	1,92244E-06	0,217996872	0,281	0,043	0,040565506	1,16311752	0,85975835	6,534883721
C4orf3	0,018070651	0,217948691	0,812	0,422	1	1,163078676	0,859787064	1,924170616
FDX2	1,06047E-05	0,217931128	0,281	0,051	0,22377069	1,163064517	0,859797531	5,509803922
MAN1C1	2,66394E-10	0,217888087	0,188	0,004	5,62118E-06	1,16302982	0,859823182	47
ADAM10	3,28502E-06	0,217785489	0,625	0,176	0,069317282	1,162947113	0,859884331	3,551136364
CAPN5	1,69331E-06	0,217656068	0,281	0,043	0,035730553	1,162842792	0,859961473	6,534883721
HSPA13	0,000127721	0,217292496	0,5	0,156	1	1,162549782	0,860178218	3,205128205
IL7R	1,57978E-10	0,217274619	0,219	0,008	3,33348E-06	1,162535376	0,860188877	27,375
FBXL7	0,171460184	0,217203169	0,094	0,039	1	1,162477803	0,860231479	2,41025641
LYAR	5,25179E-09	0,217194888	0,375	0,051	0,000110818	1,162471131	0,860236417	7,352941176
SOAT1	9,29699E-05	0,217159474	0,25	0,051	1	1,162442595	0,860257534	4,901960784
OTUD6B	6,60944E-05	0,217141022	0,188	0,027	1	1,162427728	0,860268536	6,962962963
IL27RA	1,5116E-08	0,217124316	0,156	0,004	0,000318962	1,162414267	0,860278498	39
MRPS18A	8,40691E-07	0,217100123	0,25	0,031	0,017739423	1,162394775	0,860292924	8,064516129
ASB1	0,000773943	0,216968806	0,156	0,027	1	1,162288977	0,860371233	5,777777778
PXN-AS1	7,65055E-07	0,216951864	0,188	0,016	0,016143431	1,162275327	0,860381337	11,75
PEX13	3,86379E-06	0,216689249	0,156	0,012	0,081529835	1,162063777	0,860537967	13
PGAP6	4,62825E-08	0,21659633	0,281	0,031	0,000976607	1,161988934	0,860593393	9,064516129
DRG1	3,59137E-08	0,216434868	0,312	0,039	0,000757815	1,161858895	0,860689714	8
PA2G4	0,000228794	0,216352926	0,75	0,285	1	1,161792906	0,8607386	2,631578947
KNOP1	0,001003745	0,216216519	0,25	0,066	1	1,161683064	0,860819987	3,787878788
CHRNB1	9,7095E-07	0,215895064	0,219	0,023	0,020488018	1,161424251	0,861011813	9,52173913
COPS5	7,93315E-05	0,215845975	0,406	0,113	1	1,161384733	0,86104111	3,592920354
CTCF	7,44809E-07	0,215649168	0,375	0,07	0,015716207	1,161226313	0,861158578	5,357142857
TYMS	9,12354E-07	0,215569093	0,094	0	0,019251588	1,161161862	0,861206377	#DIV/0!
ZMAT3	0,000939929	0,215345305	0,25	0,066	1	1,160981759	0,861339976	3,787878788
TMEM184B	0,045493752	0,215277649	0,25	0,113	1	1,160927316	0,861380369	2,212389381
EVA1A	8,61561E-07	0,215233435	0,125	0,004	0,018179807	1,160891737	0,861406768	31,25
DHFR	9,40504E-07	0,215058964	0,219	0,023	0,019845575	1,160751354	0,861510949	9,52173913

Supplementary Table 1

Differentially expressed genes_adjacent-skin-XL_ comparing SC-Keloid vs. (SC-Myel, SC-Nonmyel)								
**gene**	**p_val**	**avg_log2FC**	**pct.1**	**pct.2**	**p_val_adj**	**Foldchange_UP**	**Foldchange_DOWN**	**Ratio_pct1_pct2**
ESYT1	1,92242E-06	0,215027253	0,281	0,043	0,040564886	1,160725841	0,861529885	6,534883721
YTHDF2	1,06747E-05	0,215026959	0,438	0,109	0,225246221	1,160725604	0,861530061	4,018348624
NDUFA2	2,05856E-05	0,214985673	0,594	0,18	0,434377207	1,160692388	0,861554715	3,3
ZNF282	0,006378008	0,214965414	0,156	0,039	1	1,160676089	0,861566814	4
SMYD5	0,00737039	0,214955638	0,094	0,016	1	1,160668224	0,861572652	5,875
ATAD2B	9,89074E-05	0,214837132	0,25	0,051	1	1,160572888	0,861643426	4,901960784
KIAA0232	4,92519E-05	0,214829874	0,406	0,105	1	1,16056705	0,861647761	3,866666667
ABI2	0,003920049	0,214795217	0,438	0,176	1	1,16053917	0,86166846	2,488636364
PIGP	5,48122E-08	0,214744114	0,531	0,109	0,001156592	1,160498063	0,861698983	4,871559633
RBM15B	0,006378008	0,214567033	0,156	0,039	1	1,160355628	0,861804757	4
METTL21A	9,17361E-07	0,214467077	0,188	0,016	0,019357231	1,160275237	0,861864468	11,75
HMGN2	0,010063632	0,214394948	0,781	0,398	1	1,160217228	0,86190756	1,962311558
GIGYF2	2,62475E-05	0,214363554	0,344	0,078	0,553847608	1,160191982	0,861926315	4,41025641
NUDT2	8,90851E-07	0,214311283	0,25	0,031	0,018797848	1,160149947	0,861957545	8,064516129
RGS19	2,66394E-10	0,214294585	0,188	0,004	5,62118E-06	1,160136519	0,861967521	47
CCDC47	0,000543438	0,214170299	0,594	0,223	1	1,16003658	0,862041781	2,66367713
CTPS1	9,48524E-05	0,214166051	0,125	0,012	1	1,160033164	0,86204432	10,41666667
ZDHHC4	7,70517E-07	0,214059743	0,25	0,031	0,01625868	1,159947688	0,862107844	8,064516129
OLFML3	8,91574E-05	0,21382213	0,156	0,02	1	1,15975666	0,862249845	7,8
APEX2	1,59386E-08	0,213742197	0,156	0,004	0,000336319	1,159692404	0,86229762	39
COMMD3	5,80052E-06	0,213663448	0,438	0,105	0,122396669	1,159629105	0,862344689	4,171428571
NCAPD3	9,41597E-05	0,213650302	0,219	0,039	1	1,159618538	0,862352547	5,615384615
TMEM127	0,003453714	0,213486533	0,156	0,035	1	1,15948691	0,862450444	4,457142857
BIRC5	0,004841742	0,213453635	0,031	0	1	1,159460471	0,86247011	#DIV/0!
SPRYD7	0,000200254	0,213451306	0,219	0,043	1	1,159458599	0,862471502	5,093023256
TMPO	2,22454E-09	0,213277546	0,312	0,031	4,69401E-05	1,15931896	0,862575386	10,06451613
EHD2	3,76932E-06	0,213242409	0,375	0,078	0,079536364	1,159290726	0,862596394	4,807692308
POLR1D	5,53501E-05	0,213174224	0,625	0,207	1	1,159235936	0,862637164	3,019323671
EIF4H	0,0024417	0,212804961	0,688	0,297	1	1,158939263	0,862857987	2,316498316
CFDP1	2,40826E-05	0,212640828	0,562	0,172	0,508167664	1,158807421	0,862956158	3,26744186

Supplementary Table 1

Differentially expressed genes_adjacent-skin-XL_ comparing SC-Keloid vs. (SC-Myel, SC-Nonmyel)								
**gene**	**p_val**	**avg_log2FC**	**pct.1**	**pct.2**	**p_val_adj**	**Foldchange_UP**	**Foldchange_DOWN**	**Ratio_pct1_pct2**
SLC25A24	1,63458E-09	0,212404913	0,406	0,055	3,44913E-05	1,158617943	0,863097284	7,381818182
ARL15	1,44979E-05	0,212310657	0,125	0,008	0,305919948	1,158542249	0,863153675	15,625
PGS1	0,024054161	0,212003231	0,125	0,035	1	1,1582954	0,863337625	3,571428571
QDPR	0,000153915	0,211710123	0,5	0,16	1	1,158060097	0,863513045	3,125
TMEM214	0,000109604	0,211449018	0,25	0,051	1	1,157850525	0,863669341	4,901960784
TUSC2	4,03182E-13	0,211402866	0,375	0,027	8,50753E-09	1,157813486	0,86369697	13,88888889
C22orf39	0,007863882	0,211374498	0,219	0,07	1	1,15779072	0,863713953	3,128571429
PAICS	1,54041E-05	0,211242772	0,312	0,062	0,325042363	1,157685012	0,863792819	5,032258065
GTF2E2	9,47138E-06	0,211187393	0,281	0,051	0,19985553	1,157640575	0,863825977	5,509803922
DCBLD1	2,92389E-12	0,210687041	0,25	0,008	6,1697E-08	1,157239154	0,864125619	31,25
UBE2L6	0,000205019	0,210629726	0,25	0,055	1	1,15719318	0,864159949	4,545454545
MICAL2	2,33183E-10	0,210456666	0,281	0,02	4,92039E-06	1,157054376	0,864263617	14,05
PRIM2	2,86577E-08	0,210447535	0,219	0,016	0,000604707	1,157047053	0,864269087	13,6875
LRRC75A	6,93747E-09	0,210435576	0,25	0,02	0,000146388	1,157037462	0,864276251	12,5
CASP14	0,004841742	0,210432726	0,031	0	1	1,157035176	0,864277959	#DIV/0!
GABRA4	0,004841742	0,210432726	0,031	0	1	1,157035176	0,864277959	#DIV/0!
ELMOD3	0,000305772	0,210351516	0,156	0,023	1	1,156970048	0,864326611	6,782608696
WRAP73	8,65685E-05	0,210257255	0,156	0,02	1	1,156894458	0,864383085	7,8
ATG7	0,000760719	0,21024659	0,219	0,051	1	1,156885906	0,864389474	4,294117647
NTN1	6,02104E-12	0,210188295	0,312	0,02	1,2705E-07	1,15683916	0,864424402	15,6
FAM136A	8,55048E-11	0,210152441	0,344	0,031	1,80424E-06	1,156810411	0,864445885	11,09677419
ERI3	4,21482E-06	0,21012464	0,406	0,09	0,088937012	1,156788119	0,864462544	4,511111111
CALML4	0,013054404	0,209738751	0,062	0,008	1	1,156478745	0,864693799	7,75
SIVA1	0,001381319	0,209584669	0,656	0,273	1	1,156355238	0,864786155	2,402930403
CROT	1,58275E-07	0,209030607	0,281	0,035	0,00333977	1,155911229	0,865118337	8,028571429
SIPA1	0,005141746	0,208857539	0,344	0,129	1	1,155772573	0,865222124	2,666666667
FXYD3	0,000209017	0,208603933	0,25	0,055	1	1,155569422	0,865374231	4,545454545
LARS1	1,38862E-06	0,208551291	0,562	0,145	0,029301308	1,155527257	0,865405809	3,875862069
MEAK7	1,11477E-07	0,208546651	0,188	0,012	0,002352284	1,155523541	0,865408592	15,66666667
RAC1	0,076956059	0,208312388	0,938	0,656	1	1,155335924	0,865549127	1,429878049

Supplementary Table 1

Differentially expressed genes_adjacent-skin-XL_ comparing SC-Keloid vs. (SC-Myel, SC-Nonmyel)								
**gene**	**p_val**	**avg_log2FC**	**pct.1**	**pct.2**	**p_val_adj**	**Foldchange_UP**	**Foldchange_DOWN**	**Ratio_pct1_pct2**
UQCC3	1,56439E-07	0,208295528	0,375	0,062	0,00330101	1,155322421	0,865559243	6,048387097
FARP1	0,000305772	0,208075671	0,156	0,023	1	1,155146372	0,865691158	6,782608696
NAGA	1,51026E-05	0,208065333	0,344	0,074	0,318679081	1,155138094	0,865697361	4,648648649
S100A1	0,112120623	0,208039997	0,188	0,09	1	1,155117808	0,865712565	2,088888889
RUFY1	2,02073E-05	0,207910722	0,188	0,023	0,426394579	1,155014307	0,865790141	8,173913043
LRWD1	7,59646E-09	0,207902758	0,188	0,008	0,000160293	1,155007931	0,865794921	23,5
ZNF362	9,51212E-08	0,207892052	0,344	0,051	0,002007153	1,15499936	0,865801345	6,745098039
ST7-AS1	1,33967E-08	0,20775428	0,125	0	0,000282684	1,154889067	0,865884031	#DIV/0!
SLC35E4	4,79742E-08	0,207717011	0,25	0,023	0,001012303	1,154859233	0,865906399	10,86956522
MAPRE1	0,000531572	0,207706327	0,625	0,23	1	1,154850681	0,865912812	2,717391304
PRCP	0,001606083	0,207398282	0,375	0,129	1	1,154604123	0,866097722	2,906976744
FAIM	5,35812E-05	0,207179469	0,281	0,059	1	1,154429018	0,866229092	4,762711864
FBXO8	9,25608E-07	0,207156888	0,312	0,051	0,019531255	1,154410949	0,866242651	6,117647059
SH3KBP1	5,02394E-07	0,207008372	0,406	0,078	0,010601017	1,154292116	0,866331829	5,205128205
SUPV3L1	1,89132E-05	0,206853318	0,25	0,043	0,399087485	1,154168065	0,866424943	5,813953488
RBX1	0,004016773	0,206347158	0,875	0,449	1	1,153763203	0,866728976	1,948775056
RPAIN	3,92965E-05	0,206132795	0,5	0,148	0,829196265	1,153591784	0,866857769	3,378378378
TMEM220	0,000452457	0,205857218	0,125	0,016	1	1,153371452	0,867023368	7,8125
SLC12A4	6,66992E-05	0,205705839	0,281	0,059	1	1,153250437	0,867114347	4,762711864
ADGRA3	9,82165E-05	0,205534408	0,125	0,012	1	1,153113408	0,86721739	10,41666667
CHCHD3	0,00028292	0,205289944	0,312	0,082	1	1,15291803	0,867364352	3,804878049
DOCK8	0,000112833	0,20513752	0,125	0,012	1	1,152796228	0,867455996	10,41666667
KIF3B	9,48524E-05	0,205019892	0,125	0,012	1	1,152702241	0,867526725	10,41666667
CDC5L	7,23811E-06	0,204995125	0,5	0,129	0,152731424	1,152682452	0,867541618	3,875968992
GINS2	1,39097E-05	0,204673929	0,125	0,008	0,293508116	1,152425852	0,867734786	15,625
GPS1	1,16412E-07	0,204573196	0,531	0,117	0,002456417	1,152345388	0,867795376	4,538461538
ABHD6	1,77239E-07	0,204285382	0,219	0,02	0,00373992	1,152115522	0,867968516	10,95
SNRNP40	0,001177356	0,204261175	0,312	0,094	1	1,15209619	0,86798308	3,319148936
RAB11B	1,26978E-05	0,20404627	0,625	0,188	0,267937201	1,151924586	0,868112385	3,324468085
PRKACA	0,000164053	0,204036305	0,312	0,078	1	1,15191663	0,868118381	4

Supplementary Table 1

Differentially expressed genes_adjacent-skin-XL_ comparing SC-Keloid vs. (SC-Myel, SC-Nonmyel)								
**gene**	**p_val**	**avg_log2FC**	**pct.1**	**pct.2**	**p_val_adj**	**Foldchange_UP**	**Foldchange_DOWN**	**Ratio_pct1_pct2**
SNAPC5	1,73693E-06	0,203929225	0,281	0,043	0,036650933	1,151831135	0,868182818	6,534883721
DDX49	7,11318E-06	0,203844897	0,344	0,07	0,150095314	1,151763811	0,868233566	4,914285714
TSSC4	0,000501385	0,203789052	0,438	0,145	1	1,151719228	0,868267174	3,020689655
KLHDC3	1,25841E-06	0,203755428	0,344	0,062	0,026553766	1,151692386	0,868287411	5,548387097
DCAF4	0,013054404	0,203663556	0,062	0,008	1	1,151619047	0,868342706	7,75
MAP7D3	6,07138E-07	0,203622607	0,281	0,039	0,012811221	1,151586361	0,868367353	7,205128205
MRFAP1L1	9,62117E-08	0,2035475	0,406	0,07	0,002030164	1,15152641	0,868412562	5,8
HYOU1	0,000765876	0,203456757	0,312	0,094	1	1,151453984	0,868467185	3,319148936
MBLAC1	0,01346698	0,203453993	0,062	0,008	1	1,151451778	0,868468849	7,75
HACD3	5,96633E-09	0,203368571	0,469	0,078	0,000125895	1,151383602	0,868520273	6,012820513
PRR7	4,18583E-08	0,203337756	0,25	0,023	0,000883252	1,15135901	0,868538824	10,86956522
STAT1	0,000100965	0,202786986	0,25	0,051	1	1,150919545	0,868870465	4,901960784
CDK5RAP3	4,18627E-05	0,202506805	0,375	0,094	0,883345867	1,150696051	0,869039221	3,989361702
ORMDL3	4,0305E-06	0,202250439	0,219	0,027	0,085047498	1,150491591	0,869193663	8,111111111
NOA1	6,40895E-07	0,202233506	0,281	0,039	0,013523521	1,150478088	0,869203865	7,205128205
BCOR	1,13457E-05	0,202183831	0,281	0,051	0,239406468	1,150438475	0,869233793	5,509803922
MRPS35	1,78163E-06	0,202183139	0,281	0,043	0,037594075	1,150437924	0,86923421	6,534883721
GTF2F1	8,65547E-05	0,202145412	0,312	0,074	1	1,150407839	0,869256942	4,216216216
PFKFB4	7,93423E-07	0,202050216	0,188	0,016	0,016742011	1,150331932	0,869314301	11,75
CPSF2	0,000417537	0,201928805	0,188	0,035	1	1,15023513	0,869387462	5,371428571
EMD	0,000191745	0,201849047	0,5	0,16	1	1,150171542	0,869435526	3,125
UNC93B1	4,48152E-08	0,201848571	0,25	0,023	0,000945646	1,150171162	0,869435813	10,86956522
TRPV3	2,91341E-06	0,201777945	0,25	0,035	0,061475767	1,150114858	0,869478377	7,142857143
ANKRD13C	9,48469E-07	0,201677506	0,312	0,051	0,020013643	1,150034791	0,869538911	6,117647059
DPY19L1	6,17128E-06	0,201636407	0,312	0,059	0,130220119	1,15000203	0,869563683	5,288135593
AP3M1	1,10934E-05	0,201594306	0,281	0,051	0,23408214	1,14996847	0,869589059	5,509803922
NDFIP1	0,005933351	0,201542568	0,656	0,312	1	1,149927231	0,869620245	2,102564103
RNF26	0,000486824	0,201334872	0,094	0,008	1	1,149761694	0,869745448	11,75
MDP1	2,86075E-06	0,201184788	0,312	0,055	0,060364658	1,149642091	0,869835933	5,672727273
TNIK	3,51691E-07	0,201112127	0,156	0,008	0,007421029	1,149584191	0,869879743	19,5

Supplementary Table 1

Differentially expressed genes_adjacent-skin-XL_ comparing SC-Keloid vs. (SC-Myel, SC-Nonmyel)								
**gene**	**p_val**	**avg_log2FC**	**pct.1**	**pct.2**	**p_val_adj**	**Foldchange_UP**	**Foldchange_DOWN**	**Ratio_pct1_pct2**
GTSE1	9,12354E-07	0,200993646	0,094	0	0,019251588	1,149489785	0,869951184	#DIV/0!
PEBP1	0,039700085	0,200705746	0,969	0,711	1	1,149260419	0,870124807	1,362869198
CCDC50	1,82828E-06	0,200542732	0,438	0,098	0,038578545	1,149130568	0,87022313	4,469387755
POLE4	9,40178E-08	0,200422569	0,406	0,07	0,001983869	1,149034861	0,870295614	5,8
PRKAR1A	0,012411259	0,20037827	0,594	0,285	1	1,14899958	0,870322337	2,084210526
DENND2B	0,004081618	0,200308464	0,344	0,121	1	1,148943985	0,87036445	2,842975207
STT3A	1,50482E-06	0,200280243	0,344	0,062	0,031753276	1,148921511	0,870381476	5,548387097
PRKAG1	1,2719E-05	0,200126569	0,219	0,031	0,268384055	1,148799136	0,870474192	7,064516129
SELENOS	0,000417809	0,200087054	0,656	0,238	1	1,148767671	0,870498035	2,756302521
CLPTM1	3,09835E-08	0,199186929	0,594	0,129	0,000653783	1,148051156	0,871041325	4,604651163
RAD17	0,000466094	0,199099462	0,125	0,016	1	1,147981555	0,871094135	7,8125
TOM1L1	4,35951E-06	0,198800539	0,188	0,02	0,091990018	1,147743721	0,871274643	9,4
PLA2G4A	5,94561E-05	0,198611677	0,188	0,027	1	1,14759348	0,871388708	6,962962963
EXOSC2	0,003651243	0,198559137	0,125	0,023	1	1,147551688	0,871420443	5,434782609
MRPL45	2,65168E-08	0,198417597	0,219	0,016	0,00055953	1,14743911	0,87150594	13,6875
MRPL35	1,08519E-08	0,198237551	0,281	0,027	0,000228985	1,14729592	0,871614709	10,40740741
ALPK3	0,079594089	0,198201531	0,031	0,004	1	1,147267275	0,871636472	7,75
ADCK5	1,02698E-07	0,198161245	0,188	0,012	0,002167028	1,147235239	0,871660812	15,66666667
PTGES2	5,55781E-06	0,197781752	0,281	0,047	0,117275301	1,146933505	0,871890128	5,978723404
MAP3K20	0,002679274	0,197654716	0,188	0,047	1	1,146832517	0,871966905	4
ARL2	0,001768797	0,197567999	0,719	0,316	1	1,146763585	0,872019318	2,275316456
ZW10	1,2719E-05	0,197305424	0,219	0,031	0,268384055	1,14655489	0,872178043	7,064516129
CYSTM1	0,000354409	0,196829619	0,719	0,281	1	1,146176816	0,872465737	2,558718861
RNF181	5,83159E-07	0,196618643	0,625	0,16	0,01230523	1,146009214	0,872593333	3,90625
NREP	1,34164E-05	0,196595995	0,375	0,086	0,283099549	1,145991223	0,872607032	4,360465116
UROS	8,14044E-08	0,196456447	0,344	0,051	0,001717714	1,14588038	0,872691441	6,745098039
ELOVL1	1,28694E-06	0,196204685	0,344	0,062	0,027155825	1,145680432	0,872843746	5,548387097
FBXL4	0,000738278	0,196166287	0,156	0,027	1	1,145649939	0,872866978	5,777777778
NR2F6	6,07138E-07	0,196022733	0,281	0,039	0,012811221	1,145535949	0,872953835	7,205128205
CDC73	0,005295635	0,196001811	0,375	0,148	1	1,145519336	0,872966496	2,533783784

Supplementary Table 1

Differentially expressed genes_adjacent-skin-XL_ comparing SC-Keloid vs. (SC-Myel, SC-Nonmyel)								
**gene**	**p_val**	**avg_log2FC**	**pct.1**	**pct.2**	**p_val_adj**	**Foldchange_UP**	**Foldchange_DOWN**	**Ratio_pct1_pct2**
DRAM2	4,62825E-08	0,195911295	0,281	0,031	0,000976607	1,145447468	0,873021267	9,064516129
TIPIN	0,000305772	0,195809305	0,156	0,023	1	1,145366494	0,873082987	6,782608696
HPF1	8,67326E-07	0,195692643	0,375	0,07	0,018301437	1,145273879	0,873153591	5,357142857
MUS81	2,38912E-05	0,19562638	0,25	0,043	0,50412882	1,145221278	0,873193696	5,813953488
WASL	2,39175E-05	0,195579832	0,531	0,156	0,504682659	1,145184328	0,87322187	3,403846154
ZADH2	4,33123E-08	0,195542209	0,25	0,023	0,000913933	1,145154464	0,873244642	10,86956522
SEMA4F	4,5807E-05	0,195223812	0,094	0,004	0,966573495	1,144901761	0,873437385	23,5
TACC1	0,000804532	0,194984281	0,656	0,27	1	1,144711689	0,873582414	2,42962963
NOL9	0,000173407	0,194923251	0,188	0,031	1	1,144663265	0,87361937	6,064516129
SEPTIN11	4,19272E-05	0,194819008	0,406	0,105	0,884705188	1,14458056	0,873682496	3,866666667
ZNF283	0,368323117	0,194655059	0,031	0,012	1	1,144450496	0,873781787	2,583333333
PSME2	0,000149073	0,194584604	0,531	0,176	1	1,144394607	0,87382446	3,017045455
TRAPPC6B	4,70541E-06	0,194553731	0,281	0,047	0,099288927	1,144370118	0,87384316	5,978723404
CUTC	0,000417537	0,19422848	0,188	0,035	1	1,144112152	0,874040188	5,371428571
REPIN1	0,000641668	0,194138456	0,312	0,09	1	1,144040762	0,874094729	3,466666667
SHROOM2	3,3636E-07	0,194059102	0,156	0,008	0,007097534	1,143977837	0,874142809	19,5
MPHOSPH9	0,007631848	0,193964911	0,125	0,027	1	1,143903151	0,874199883	4,62962963
MYL5	7,96166E-09	0,193853884	0,188	0,008	0,000167999	1,143815122	0,874267161	23,5
SYTL2	5,78487E-05	0,193820099	0,375	0,098	1	1,143788336	0,874287635	3,826530612
CTDNEP1	5,13089E-07	0,193702944	0,688	0,184	0,010826683	1,143695458	0,874358635	3,739130435
RBM42	0,000499558	0,193397517	0,5	0,18	1	1,143453356	0,874543762	2,777777778
ATP2C1	3,11336E-06	0,193394318	0,406	0,09	0,065694924	1,143450821	0,874545701	4,511111111
POU3F2	1,34171E-05	0,19338996	0,219	0,031	0,283114022	1,143447367	0,874548343	7,064516129
FHOD1	2,8258E-09	0,193003147	0,219	0,012	5,96273E-05	1,143140829	0,874782857	18,25
DGKZ	0,00010081	0,192987096	0,219	0,039	1	1,143128111	0,874792589	5,615384615
PPP1R11	8,37038E-06	0,192960756	0,375	0,082	0,176623288	1,14310724	0,874808561	4,573170732
IRF2BP1	5,90909E-07	0,192959416	0,281	0,039	0,012468772	1,143106179	0,874809374	7,205128205
RND2	3,86379E-06	0,192825503	0,156	0,012	0,081529835	1,143000079	0,874890578	13
GAN	1,39097E-05	0,192410945	0,125	0,008	0,293508116	1,142671685	0,875142014	15,625
TRUB1	0,001770672	0,192353929	0,156	0,031	1	1,142626527	0,875176601	5,032258065

Supplementary Table 1

Differentially expressed genes_adjacent-skin-XL_ comparing SC-Keloid vs. (SC-Myel, SC-Nonmyel)								
**gene**	**p_val**	**avg_log2FC**	**pct.1**	**pct.2**	**p_val_adj**	**Foldchange_UP**	**Foldchange_DOWN**	**Ratio_pct1_pct2**
ETFRF1	3,59126E-05	0,19235181	0,469	0,133	0,757792512	1,142624849	0,875177886	3,526315789
MED27	1,31182E-08	0,192280595	0,406	0,062	0,000276808	1,142568447	0,875221088	6,548387097
PTRHD1	1,68619E-09	0,192268179	0,281	0,023	3,55804E-05	1,142558614	0,875228621	12,2173913
ZSCAN12	0,007750521	0,192263924	0,094	0,016	1	1,142555245	0,875231202	5,875
TRAPPC4	1,31726E-05	0,192261948	0,375	0,086	0,277954243	1,14255368	0,875232401	4,360465116
AKT1S1	1,3161E-06	0,192210987	0,344	0,062	0,027771004	1,142513322	0,875263317	5,548387097
NTHL1	1,28978E-06	0,192195457	0,469	0,105	0,027215712	1,142501023	0,875272739	4,466666667
CLN3	1,31496E-07	0,191869719	0,312	0,043	0,002774696	1,142243093	0,875470385	7,255813953
PLCG1	4,14927E-06	0,191805429	0,219	0,027	0,087553714	1,142192193	0,875509398	8,111111111
ARMC5	0,012262652	0,191741842	0,062	0,008	1	1,142141852	0,875547987	7,75
SMIM30	1,64044E-07	0,191735264	0,375	0,062	0,003461494	1,142136644	0,87555198	6,048387097
SYMPK	7,9257E-05	0,191685766	0,344	0,086	1	1,142097459	0,87558202	4
CYP51A1	3,2565E-07	0,191582099	0,438	0,086	0,006871539	1,142015395	0,875644938	5,093023256
RNF208	0,007945492	0,191543252	0,125	0,027	1	1,141984644	0,875668517	4,62962963
ZNF124	0,039886012	0,19153622	0,062	0,012	1	1,141979078	0,875672785	5,166666667
SAR1B	2,18521E-07	0,191203604	0,406	0,074	0,004611011	1,141715823	0,875874696	5,486486486
ZNF45	0,079594089	0,19117576	0,031	0,004	1	1,141693788	0,875891601	7,75
YIF1A	2,08573E-05	0,191148147	0,531	0,148	0,440110734	1,141671937	0,875908365	3,587837838
PAQR4	1,64044E-07	0,190888734	0,375	0,062	0,003461494	1,14146667	0,876065878	6,048387097
PNPLA4	3,86379E-06	0,190869752	0,156	0,012	0,081529835	1,141451651	0,876077405	13
SOCS2	6,42992E-05	0,190792519	0,625	0,207	1	1,141390547	0,876124306	3,019323671
NTAN1	5,09918E-07	0,190765671	0,344	0,059	0,010759779	1,141369306	0,87614061	5,830508475
ANXA8L1	0,004841742	0,190727061	0,031	0	1	1,141338761	0,876164058	#DIV/0!
LORICRIN	0,004841742	0,190727061	0,031	0	1	1,141338761	0,876164058	#DIV/0!
PTPRH	0,004841742	0,190727061	0,031	0	1	1,141338761	0,876164058	#DIV/0!
TENT5C	0,004841742	0,190727061	0,031	0	1	1,141338761	0,876164058	#DIV/0!
TLR6	0,004841742	0,190727061	0,031	0	1	1,141338761	0,876164058	#DIV/0!
RBM33	0,019943735	0,190718454	0,281	0,113	1	1,141331951	0,876169286	2,486725664
NKAIN4	0,520968078	0,190608619	0,031	0,016	1	1,141245063	0,876235992	1,9375
UGGT2	1,64044E-07	0,190552772	0,375	0,062	0,003461494	1,141200886	0,876269912	6,048387097

Supplementary Table 1

Differentially expressed genes_adjacent-skin-XL_ comparing SC-Keloid vs. (SC-Myel, SC-Nonmyel)								
**gene**	**p_val**	**avg_log2FC**	**pct.1**	**pct.2**	**p_val_adj**	**Foldchange_UP**	**Foldchange_DOWN**	**Ratio_pct1_pct2**
DLD	7,1112E-09	0,190552645	0,375	0,051	0,000150053	1,141200786	0,876269989	7,352941176
PIGC	2,06459E-05	0,190497827	0,438	0,113	0,435650061	1,141157424	0,876303285	3,876106195
PKNOX2	1,2719E-05	0,190472916	0,219	0,031	0,268384055	1,14113772	0,876318417	7,064516129
SMIM10	0,000200254	0,190408439	0,219	0,043	1	1,141086721	0,876357582	5,093023256
FAM167B	9,12354E-07	0,190205375	0,094	0	0,019251588	1,140926121	0,876480941	#DIV/0!
TMEM98	5,9331E-06	0,190128705	0,375	0,082	0,125194427	1,140865489	0,876527522	4,573170732
DYM	5,44692E-07	0,190084413	0,281	0,039	0,011493547	1,140830464	0,876554432	7,205128205
TBX3	0,000105286	0,190071864	0,125	0,012	1	1,140820541	0,876562057	10,41666667
FILIP1L	0,001557595	0,189779173	0,312	0,098	1	1,140589118	0,87673991	3,183673469
DYNC1LI2	0,000991155	0,189772957	0,719	0,285	1	1,140584203	0,876743687	2,522807018
LRRC41	0,000576308	0,189255376	0,281	0,074	1	1,14017508	0,877058284	3,797297297
BRD9	3,29746E-05	0,189032532	0,469	0,129	0,695796475	1,139998978	0,877193769	3,635658915
RNASEH1	2,83613E-06	0,188861141	0,25	0,035	0,059845173	1,139863556	0,877297984	7,142857143
CHMP4A	0,000321385	0,18883973	0,469	0,152	1	1,139846639	0,877311004	3,085526316
TRIB1	0,000916506	0,188786584	0,188	0,039	1	1,13980465	0,877343323	4,820512821
DRG2	1,72688E-07	0,188726004	0,281	0,035	0,003643896	1,13975679	0,877380164	8,028571429
SLTM	9,74729E-06	0,188599127	0,625	0,188	0,205677645	1,139656559	0,877457329	3,324468085
MIS18BP1	0,000390703	0,188477508	0,188	0,035	1	1,13956049	0,877531302	5,371428571
HIVEP3	3,58718E-06	0,188430479	0,219	0,027	0,075693187	1,139523344	0,877559907	8,111111111
ZEB1	1,45331E-05	0,188379083	0,219	0,031	0,306663142	1,139482749	0,877591171	7,064516129
EXOC6	0,007631848	0,18835418	0,125	0,027	1	1,13946308	0,87760632	4,62962963
YIPF6	0,005779495	0,188106803	0,375	0,145	1	1,139267714	0,877756815	2,586206897
UBA3	0,000428394	0,188024703	0,312	0,086	1	1,139202884	0,877806767	3,627906977
ATPAF1	1,96898E-07	0,187734787	0,219	0,02	0,00415474	1,138973979	0,877983184	10,95
PBK	6,3574E-05	0,187723195	0,062	0	1	1,138964827	0,877990239	#DIV/0!
DOHH	0,000408406	0,187504123	0,188	0,035	1	1,138791889	0,87812357	5,371428571
ZBED4	7,93423E-07	0,187258939	0,188	0,016	0,016742011	1,138598369	0,878272819	11,75
ERG28	1,37797E-05	0,18715948	0,219	0,031	0,29076515	1,138519878	0,878333369	7,064516129
NEIL2	2,17326E-05	0,187101546	0,156	0,016	0,458580025	1,138474159	0,878368641	9,75
ATF7	0,011968519	0,187062562	0,25	0,09	1	1,138443396	0,878392376	2,777777778

Supplementary Table 1

Differentially expressed genes_adjacent-skin-XL_ comparing SC-Keloid vs. (SC-Myel, SC-Nonmyel)								
**gene**	**p_val**	**avg_log2FC**	**pct.1**	**pct.2**	**p_val_adj**	**Foldchange_UP**	**Foldchange_DOWN**	**Ratio_pct1_pct2**
MAGED1	1,03141E-06	0,187047886	0,406	0,082	0,021763787	1,138431816	0,878401312	4,951219512
GLMP	2,27357E-09	0,186868844	0,375	0,047	4,79746E-05	1,138290542	0,87851033	7,978723404
NKIRAS1	0,017290773	0,186730523	0,094	0,02	1	1,138181412	0,878594563	4,7
TRAPPC3	3,92226E-06	0,186423138	0,375	0,078	0,082763591	1,137938933	0,878781779	4,807692308
MFN1	4,59733E-07	0,186238652	0,312	0,047	0,009700817	1,137793427	0,878894161	6,638297872
PGRMC2	0,014854014	0,186018376	0,406	0,184	1	1,137619718	0,879028364	2,206521739
ARHGAP4	0,221475664	0,185985968	0,031	0,008	1	1,137594163	0,879048111	3,875
MANBAL	1,03113E-06	0,185743859	0,375	0,07	0,021757888	1,137403271	0,879195642	5,357142857
DGUOK-AS1	3,67695E-07	0,185710818	0,156	0,008	0,007758733	1,137377222	0,879215778	19,5
ZNF341	0,079594089	0,185660745	0,031	0,004	1	1,137337747	0,879246295	7,75
PPP1CB	0,006321051	0,185523657	0,75	0,363	1	1,13722968	0,879329847	2,066115702
GPS2	0,002382773	0,185484655	0,469	0,184	1	1,137198937	0,879353618	2,548913043
RFXANK	7,80539E-08	0,185451089	0,375	0,059	0,001647015	1,137172479	0,879374078	6,355932203
TMCC1	6,4372E-05	0,185432097	0,188	0,027	1	1,137157508	0,879385655	6,962962963
WAC-AS1	2,03294E-05	0,185383574	0,156	0,016	0,428970333	1,137119263	0,879415232	9,75
CRTC1	0,039090354	0,185236063	0,125	0,039	1	1,137003002	0,879505154	3,205128205
PET100	0,00395393	0,185149349	0,625	0,277	1	1,136934663	0,879558019	2,25631769
PDE7A	0,000773943	0,185118387	0,156	0,027	1	1,136910264	0,879576895	5,777777778
RGL3	0,079594089	0,184961113	0,031	0,004	1	1,136786331	0,879672787	7,75
GOLIM4	0,000218057	0,184884009	0,406	0,121	1	1,136725578	0,879719802	3,355371901
CKMT2-AS1	0,082995684	0,184820331	0,062	0,016	1	1,136675407	0,879758631	3,875
PNKP	3,77747E-08	0,184733473	0,25	0,023	0,000797083	1,136606974	0,879811599	10,86956522
SS18L2	0,000248368	0,184728328	0,312	0,082	1	1,136602921	0,879814737	3,804878049
SCAMP3	2,7063E-07	0,184546549	0,344	0,055	0,00571057	1,136459718	0,8799256	6,254545455
C19orf73	3,67695E-07	0,184526001	0,156	0,008	0,007758733	1,136443532	0,879938133	19,5
URI1	8,37081E-05	0,184377109	0,5	0,16	1	1,136326252	0,880028951	3,125
ARFGAP3	1,65634E-05	0,18428557	0,594	0,176	0,349505	1,136254155	0,880084791	3,375
AP3D1	0,000255579	0,184077068	0,375	0,109	1	1,136089952	0,880211992	3,440366972
NPRL3	8,08168E-09	0,183725373	0,438	0,07	0,000170532	1,135813034	0,880426593	6,257142857
CD4	0,000362786	0,183622329	0,25	0,059	1	1,135731912	0,880489479	4,237288136

Supplementary Table 1

Differentially expressed genes_adjacent-skin-XL_ comparing SC-Keloid vs. (SC-Myel, SC-Nonmyel)								
**gene**	**p_val**	**avg_log2FC**	**pct.1**	**pct.2**	**p_val_adj**	**Foldchange_UP**	**Foldchange_DOWN**	**Ratio_pct1_pct2**
SMARCC1	1,79728E-06	0,183310097	0,344	0,062	0,037924443	1,135486141	0,880680058	5,548387097
ZNF626	0,000811202	0,18324056	0,156	0,027	1	1,135431413	0,880722507	5,777777778
ACBD6	5,25917E-07	0,183139943	0,438	0,09	0,011097368	1,135352227	0,880783933	4,866666667
MED4	6,07559E-05	0,183118885	0,438	0,121	1	1,135335656	0,880796789	3,619834711
HADHA	1,47773E-05	0,183085683	0,625	0,188	0,311815116	1,135309527	0,88081706	3,324468085
SMYD2	4,79742E-08	0,183013607	0,25	0,023	0,001012303	1,135252809	0,880861066	10,86956522
PMM1	6,4022E-08	0,182998893	0,375	0,059	0,001350927	1,135241231	0,88087005	6,355932203
SDK1	0,00737039	0,182975162	0,094	0,016	1	1,135222557	0,88088454	5,875
PLPBP	0,001819944	0,182966218	0,25	0,07	1	1,135215519	0,880890001	3,571428571
HKDC1	3,86379E-06	0,1827802	0,156	0,012	0,081529835	1,135069157	0,881003588	13
CYC1	2,07373E-05	0,182676971	0,656	0,207	0,437578288	1,134987942	0,881066629	3,169082126
AARS1	7,34732E-05	0,182624903	0,406	0,113	1	1,13494698	0,881098428	3,592920354
ATAD3A	5,98372E-09	0,182574851	0,344	0,043	0,000126263	1,134907606	0,881128996	8
COPB1	3,85019E-06	0,182531488	0,531	0,137	0,081242781	1,134873494	0,881155481	3,875912409
NDUFA5	0,002685982	0,182514868	0,562	0,23	1	1,134860421	0,881165632	2,443478261
INSC	0,023224909	0,182450784	0,375	0,168	1	1,134810012	0,881204774	2,232142857
HNRNPD	0,014648368	0,182401791	0,719	0,375	1	1,134771475	0,8812347	1,917333333
CDC26	8,41178E-05	0,182339799	0,375	0,098	1	1,134722716	0,881272566	3,826530612
MTHFD2L	9,7095E-07	0,182219655	0,219	0,023	0,020488018	1,134628223	0,88134596	9,52173913
BAG2	1,33967E-08	0,182144622	0,125	0	0,000282684	1,134569214	0,881391799	#DIV/0!
ARMT1	1,00234E-06	0,181934354	0,219	0,023	0,021150457	1,134403865	0,881520269	9,52173913
SPG11	0,00082778	0,18171337	0,188	0,039	1	1,134230117	0,881655305	4,820512821
SLC35A4	2,28053E-05	0,181397039	0,25	0,043	0,481213619	1,133981449	0,881848642	5,813953488
MSRA	4,50344E-06	0,181301694	0,188	0,02	0,095027166	1,133906509	0,881906923	9,4
PIGM	0,001520602	0,181271603	0,125	0,02	1	1,133882859	0,881925317	6,25
FAAP100	8,91574E-05	0,181202033	0,156	0,02	1	1,133828181	0,881967847	7,8
TMEM161B	0,000108998	0,181189143	0,125	0,012	1	1,133818051	0,881975727	10,41666667
LMAN1	0,000842458	0,181179293	0,719	0,293	1	1,13381031	0,881981749	2,453924915
CC2D2A	1,37797E-05	0,181029653	0,219	0,031	0,29076515	1,133692715	0,882073235	7,064516129
ARMC1	1,52765E-07	0,180997135	0,375	0,062	0,003223491	1,133667161	0,882093117	6,048387097

Supplementary Table 1

Differentially expressed genes_adjacent-skin-XL_ comparing SC-Keloid vs. (SC-Myel, SC-Nonmyel)								
**gene**	**p_val**	**avg_log2FC**	**pct.1**	**pct.2**	**p_val_adj**	**Foldchange_UP**	**Foldchange_DOWN**	**Ratio_pct1_pct2**
ERAP2	1,53871E-06	0,180950282	0,344	0,062	0,032468255	1,133630345	0,882121764	5,548387097
AIMP2	9,93063E-09	0,180922689	0,312	0,035	0,000209546	1,133608664	0,882138636	8,914285714
MRS2	4,02048E-10	0,180814172	0,312	0,027	8,48362E-06	1,133523399	0,882204991	11,55555556
LRRC55	0,004841742	0,180578281	0,031	0	1	1,133338074	0,88234925	#DIV/0!
SLFN12	4,41731E-05	0,180545504	0,25	0,047	0,932096561	1,133312326	0,882369296	5,319148936
ARL1	0,000109835	0,180537773	0,531	0,176	1	1,133306253	0,882374025	3,017045455
BLOC1S1	2,05332E-05	0,180337283	0,844	0,293	0,433271519	1,13314877	0,882496656	2,880546075
DARS2	0,002594281	0,180336029	0,094	0,012	1	1,133147784	0,882497424	7,833333333
RCC2	2,25732E-05	0,180302891	0,406	0,102	0,476317656	1,133121757	0,882517694	3,980392157
VDAC3	4,01187E-05	0,180295487	0,469	0,129	0,846544503	1,133115942	0,882522223	3,635658915
TTL	9,9101E-06	0,180290698	0,281	0,051	0,209112923	1,133112181	0,882525152	5,509803922
EEF1A1	0,008132866	0,180147266	1	0,973	1	1,132999532	0,882612897	1,027749229
NAA30	0,003136549	0,18014644	0,188	0,047	1	1,132998884	0,882613403	4
ODF2L	4,08635E-08	0,180124772	0,281	0,031	0,00086226	1,132981867	0,882626659	9,064516129
AIMP1	5,12068E-06	0,179754673	0,625	0,184	0,108051397	1,132691258	0,882853111	3,39673913
GCC1	4,50344E-06	0,179650596	0,188	0,02	0,095027166	1,132609547	0,882916803	9,4
IK	4,00808E-05	0,179593546	0,625	0,191	0,84574561	1,13256476	0,882951718	3,272251309
PYCR3	2,70596E-09	0,179588324	0,219	0,012	5,70985E-05	1,13256066	0,882954914	18,25
ZNF439	2,24684E-05	0,179267171	0,156	0,016	0,474104712	1,132308573	0,883151487	9,75
SACS	3,91499E-06	0,179025169	0,219	0,027	0,082610119	1,132118653	0,883299641	8,111111111
FOXC2	0,01346698	0,17900061	0,062	0,008	1	1,132099381	0,883314678	7,75
RWDD3	2,55882E-05	0,17835821	0,312	0,066	0,539936516	1,131595394	0,883708086	4,727272727
FN3KRP	2,54624E-06	0,178354078	0,25	0,035	0,053728244	1,131592154	0,883710617	7,142857143
NOL3	2,59108E-09	0,17825487	0,219	0,012	5,46744E-05	1,131514341	0,883771388	18,25
THAP4	2,91341E-06	0,178104952	0,25	0,035	0,061475767	1,131396766	0,88386323	7,142857143
KIAA1143	2,31819E-05	0,178095993	0,312	0,066	0,489161611	1,13138974	0,883868719	4,727272727
CISD1	5,14755E-05	0,17806718	0,281	0,059	1	1,131367144	0,883886372	4,762711864
MAF1	0,000175504	0,178006579	0,531	0,176	1	1,131319622	0,8839235	3,017045455
TMEM218	6,23796E-07	0,178000846	0,281	0,039	0,013162728	1,131315126	0,883927013	7,205128205
SURF1	0,00022545	0,177922607	0,438	0,137	1	1,131253775	0,88397495	3,197080292

Supplementary Table 1

Differentially expressed genes_adjacent-skin-XL_ comparing SC-Keloid vs. (SC-Myel, SC-Nonmyel)								
**gene**	**p_val**	**avg_log2FC**	**pct.1**	**pct.2**	**p_val_adj**	**Foldchange_UP**	**Foldchange_DOWN**	**Ratio_pct1_pct2**
G6PD	6,4372E-05	0,177857547	0,188	0,027	1	1,131202762	0,884014815	6,962962963
B3GALT4	7,20082E-09	0,177774612	0,25	0,02	0,000151945	1,131137735	0,884065635	12,5
ALG8	2,17664E-05	0,177768791	0,25	0,043	0,45929188	1,131133171	0,884069202	5,813953488
LACC1	4,80439E-05	0,177643808	0,094	0,004	1	1,131035183	0,884145794	23,5
TMEM203	4,59438E-06	0,177345919	0,281	0,047	0,096945964	1,13080167	0,884328372	5,978723404
TYSND1	0,001808729	0,177183303	0,156	0,031	1	1,130674217	0,884428056	5,032258065
USP5	4,22524E-05	0,177119242	0,219	0,035	0,891567246	1,130624012	0,884467329	6,257142857
XRN2	2,08946E-06	0,176911416	0,562	0,145	0,044089784	1,130461152	0,88459475	3,875862069
MRM2	4,48152E-08	0,176718927	0,25	0,023	0,000945646	1,130310333	0,884712783	10,86956522
HDAC1	0,000100733	0,176605492	0,281	0,062	1	1,130221464	0,884782348	4,532258065
TAF1B	0,011239937	0,176209728	0,156	0,043	1	1,12991146	0,885025097	3,627906977
DEGS1	0,000482217	0,175933142	0,719	0,293	1	1,12969486	0,885194786	2,453924915
SLC50A1	1,13961E-07	0,175777406	0,344	0,051	0,002404685	1,129572919	0,885290346	6,745098039
ANLN	1,33967E-08	0,175737702	0,125	0	0,000282684	1,129541832	0,88531471	#DIV/0!
SMIM4	3,9894E-06	0,175725805	0,344	0,066	0,084180249	1,129532518	0,885322011	5,212121212
PCOLCE2	1,33967E-08	0,175662262	0,125	0	0,000282684	1,129482769	0,885361005	#DIV/0!
GNB1L	4,80439E-05	0,175311332	0,094	0,004	1	1,12920806	0,885576392	23,5
SEZ6L2	7,96166E-09	0,175261686	0,188	0,008	0,000167999	1,129169203	0,885606867	23,5
TMEM39B	0,00050526	0,175227582	0,094	0,008	1	1,12914251	0,885627802	11,75
CPT2	4,80439E-05	0,175178702	0,094	0,004	1	1,129104254	0,885657809	23,5
BACE1	0,000209017	0,175160099	0,25	0,055	1	1,129089695	0,885669229	4,545454545
DPM1	3,15362E-05	0,174997108	0,406	0,102	0,665445267	1,128962142	0,885769295	3,980392157
EIF4G1	0,002569616	0,17490818	0,5	0,199	1	1,128892554	0,885823896	2,512562814
TMUB1	2,08505E-09	0,174890959	0,375	0,047	4,39966E-05	1,128879079	0,885834469	7,978723404
WDR54	1,30636E-05	0,174872464	0,219	0,031	0,275655185	1,128864607	0,885845826	7,064516129
TRMU	8,40691E-07	0,174817101	0,25	0,031	0,017739423	1,128821289	0,88587982	8,064516129
MAP1LC3A	0,001178868	0,174740357	0,656	0,266	1	1,128761243	0,885926946	2,466165414
KATNB1	2,66394E-10	0,174547215	0,188	0,004	5,62118E-06	1,128610139	0,886045558	47
ANAPC2	0,006725366	0,174463524	0,156	0,039	1	1,12854467	0,886096959	4
MMP11	2,66394E-10	0,174454706	0,188	0,004	5,62118E-06	1,128537772	0,886102375	47

Supplementary Table 1

Differentially expressed genes_adjacent-skin-XL_ comparing SC-Keloid vs. (SC-Myel, SC-Nonmyel)								
**gene**	**p_val**	**avg_log2FC**	**pct.1**	**pct.2**	**p_val_adj**	**Foldchange_UP**	**Foldchange_DOWN**	**Ratio_pct1_pct2**
LTBP2	1,88366E-07	0,174374007	0,281	0,035	0,003974718	1,128474647	0,886151942	8,028571429
ZNF485	9,12354E-07	0,174373288	0,094	0	0,019251588	1,128474085	0,886152383	#DIV/0!
NAGK	1,57893E-06	0,174343756	0,469	0,105	0,033317012	1,128450986	0,886170523	4,466666667
HPCAL1	0,000244445	0,174250165	0,438	0,137	1	1,128377783	0,886228013	3,197080292
LYRM4	0,000922105	0,173898905	0,281	0,078	1	1,128103084	0,886443814	3,602564103
PUSL1	9,17361E-07	0,173894749	0,188	0,016	0,019357231	1,128099834	0,886446367	11,75
TRAM2-AS1	0,003321984	0,173795728	0,156	0,035	1	1,128022409	0,886507211	4,457142857
MVB12B	2,43721E-05	0,173779294	0,344	0,078	0,514276546	1,12800956	0,88651731	4,41025641
POGLUT3	0,000196043	0,173711652	0,219	0,043	1	1,127956673	0,886558876	5,093023256
TOR4A	9,12354E-07	0,173644147	0,094	0	0,019251588	1,127903896	0,88660036	#DIV/0!
CCDC74B	7,96166E-09	0,173558453	0,188	0,008	0,000167999	1,127836902	0,886653024	23,5
CLP1	0,082995684	0,173481933	0,062	0,016	1	1,127777084	0,886700053	3,875
PHETA2	1,57978E-10	0,173264924	0,219	0,008	3,33348E-06	1,127607457	0,88683344	27,375
MAFG-DT	0,013054404	0,173245809	0,062	0,008	1	1,127592517	0,88684519	7,75
MPV17	3,55058E-06	0,17319955	0,375	0,078	0,074920718	1,127556362	0,886873627	4,807692308
CCDC86	4,35951E-06	0,172919798	0,188	0,02	0,091990018	1,12733774	0,887045616	9,4
DYNC1H1	0,006256954	0,172913679	0,812	0,402	1	1,127332958	0,887049379	2,019900498
SNX8	4,80509E-06	0,172794704	0,188	0,02	0,101392141	1,127239994	0,887122534	9,4
PTRH1	4,14927E-06	0,172595025	0,219	0,027	0,087553714	1,127083987	0,887245326	8,111111111
ROPN1B	3,31594E-06	0,172538325	0,156	0,012	0,069969667	1,127039692	0,887280197	13
CBX1	6,66882E-07	0,172518503	0,469	0,102	0,014071873	1,127024207	0,887292388	4,598039216
C3orf14	5,26316E-05	0,17242834	0,25	0,047	1	1,126953774	0,887347842	5,319148936
HS3ST3A1	0,005333601	0,17233841	0,188	0,051	1	1,126883528	0,887403157	3,68627451
PACC1	0,000157414	0,172316481	0,188	0,031	1	1,126866399	0,887416645	6,064516129
GGCT	0,000119568	0,172080387	0,438	0,129	1	1,126682006	0,887561881	3,395348837
SEC16A	0,004999094	0,171734168	0,188	0,051	1	1,126411656	0,887774904	3,68627451
RRAGB	0,001793335	0,171624443	0,188	0,043	1	1,12632599	0,887842427	4,372093023
AAMDC	8,13591E-07	0,171310169	0,562	0,137	0,017167592	1,126080659	0,888035854	4,102189781
BPHL	1,37797E-05	0,171169197	0,219	0,031	0,29076515	1,125970631	0,888122632	7,064516129
SCT	9,12354E-07	0,171136011	0,094	0	0,019251588	1,12594473	0,888143062	#DIV/0!

Supplementary Table 1

Differentially expressed genes_adjacent-skin-XL_ comparing SC-Keloid vs. (SC-Myel, SC-Nonmyel)								
**gene**	**p_val**	**avg_log2FC**	**pct.1**	**pct.2**	**p_val_adj**	**Foldchange_UP**	**Foldchange_DOWN**	**Ratio_pct1_pct2**
ZNHIT3	1,39804E-06	0,17106725	0,406	0,082	0,029500136	1,125891067	0,888185393	4,951219512
SPRY4	1,51098E-05	0,171034276	0,125	0,008	0,318831333	1,125865335	0,888205693	15,625
ITGB5	0,001481966	0,170716707	0,125	0,02	1	1,125617534	0,888401229	6,25
EREG	0,004841742	0,170607404	0,031	0	1	1,125532257	0,888468539	#DIV/0!
NRAS	1,01508E-08	0,170424051	0,281	0,027	0,000214192	1,125389221	0,888581462	10,40740741
TRAPPC12	2,60971E-05	0,170368877	0,312	0,066	0,550675877	1,125346183	0,888615446	4,727272727
RABEP2	1,00234E-06	0,170345843	0,219	0,023	0,021150457	1,125328216	0,888629633	9,52173913
MIB1	0,002509791	0,17032331	0,344	0,121	1	1,12531064	0,888643512	2,842975207
GLOD4	1,42359E-11	0,170194827	0,5	0,066	3,00392E-07	1,125210427	0,888722657	7,575757576
CD109	3,86379E-06	0,170057837	0,156	0,012	0,081529835	1,125103589	0,888807048	13
CACNA1D	9,12354E-07	0,170040751	0,094	0	0,019251588	1,125090264	0,888817575	#DIV/0!
TPX2	4,80439E-05	0,169987852	0,094	0,004	1	1,125049012	0,888850165	23,5
H1-4	9,45561E-05	0,169726397	0,156	0,02	1	1,124845141	0,889011264	7,8
ELAC2	4,0305E-06	0,169585746	0,219	0,027	0,085047498	1,124735483	0,889097939	8,111111111
NUSAP1	0,01346698	0,169554476	0,062	0,008	1	1,124711104	0,889117211	7,75
UBALD2	7,16027E-05	0,169548093	0,688	0,23	1	1,124706129	0,889121144	2,991304348
TOP2A	0,079594089	0,169367314	0,031	0,004	1	1,124565205	0,889232563	7,75
MYBL1	0,000466094	0,169303358	0,125	0,016	1	1,124515353	0,889271985	7,8125
TSPAN6	2,33422E-05	0,169277103	0,25	0,043	0,492544426	1,124494888	0,889288169	5,813953488
LMO4	0,003066018	0,169236936	0,719	0,344	1	1,124463581	0,889312929	2,090116279
EBAG9	1,26917E-05	0,169069367	0,281	0,051	0,26780859	1,124332982	0,889416228	5,509803922
ORC6	0,039886012	0,168897364	0,062	0,012	1	1,124198943	0,889522273	5,166666667
PMS2	0,003731537	0,168893281	0,156	0,035	1	1,124195761	0,889524791	4,457142857
ARHGAP22	2,66394E-10	0,168851482	0,188	0,004	5,62118E-06	1,124163191	0,889550563	47
TAF9	7,40997E-10	0,168555019	0,469	0,07	1,56358E-05	1,123932208	0,889733378	6,7
SHROOM4	0,003660239	0,168538874	0,156	0,035	1	1,12391963	0,889743335	4,457142857
SLURP1	0,082317662	0,168497362	0,031	0,004	1	1,123887291	0,889768937	7,75
TNFSF12	0,000107903	0,168438089	0,562	0,18	1	1,123841117	0,889805493	3,122222222
FAM8A1	0,115638666	0,168364691	0,156	0,07	1	1,123783942	0,889850764	2,228571429
RABGGTB	6,4529E-05	0,168354255	0,438	0,121	1	1,123775814	0,889857201	3,619834711

Supplementary Table 1

Differentially expressed genes_adjacent-skin-XL_ comparing SC-Keloid vs. (SC-Myel, SC-Nonmyel)								
**gene**	**p_val**	**avg_log2FC**	**pct.1**	**pct.2**	**p_val_adj**	**Foldchange_UP**	**Foldchange_DOWN**	**Ratio_pct1_pct2**
AKR1B1	7,61677E-06	0,16835301	0,531	0,141	0,160721394	1,123774844	0,889857969	3,765957447
TICAM1	3,57983E-06	0,168267263	0,156	0,012	0,075538003	1,123708054	0,88991086	13
TMEM164	3,67695E-07	0,168163232	0,156	0,008	0,007758733	1,123627027	0,889975033	19,5
DNAJC7	6,43053E-05	0,167597685	0,531	0,168	1	1,123186643	0,890323978	3,160714286
ANTXR1	0,004132388	0,167458289	0,281	0,094	1	1,123078124	0,890410006	2,989361702
CENPK	9,06462E-07	0,167111362	0,125	0,004	0,019127258	1,122808088	0,890624151	31,25
NTAQ1	3,86379E-06	0,167069708	0,156	0,012	0,081529835	1,122775671	0,890649865	13
ASL	0,000689826	0,16682173	0,25	0,062	1	1,122582699	0,890802968	4,032258065
CDR2	1,16035E-05	0,166780093	0,281	0,051	0,244846285	1,122550301	0,890828677	5,509803922
ZNF688	1,74824E-09	0,166653105	0,281	0,023	3,68897E-05	1,122451497	0,890907093	12,2173913
STXBP3	8,37038E-06	0,166521833	0,375	0,082	0,176623288	1,122349368	0,890988161	4,573170732
PRECSIT	4,5807E-05	0,166472053	0,094	0,004	0,966573495	1,122310643	0,891018905	23,5
NBL1	7,96166E-09	0,166208745	0,188	0,008	0,000167999	1,122105827	0,891181541	23,5
DDX54	1,54779E-07	0,166166817	0,312	0,043	0,003265999	1,122073217	0,891207441	7,255813953
BOLA1	2,11157E-07	0,166115497	0,219	0,02	0,00445563	1,122033303	0,891239143	10,95
BICC1	1,51098E-05	0,166080344	0,125	0,008	0,318831333	1,122005964	0,89126086	15,625
CCNB1	9,06462E-07	0,166074115	0,125	0,004	0,019127258	1,122001119	0,891264708	31,25
TMEM101	1,23864E-06	0,165772102	0,312	0,051	0,026136469	1,121766265	0,891451304	6,117647059
DSCC1	1,59386E-08	0,165715698	0,156	0,004	0,000336319	1,121722409	0,891486158	39
ALG1	1,11477E-07	0,165493297	0,188	0,012	0,002352284	1,121549501	0,891623597	15,66666667
FBXL5	1,98911E-06	0,165482261	0,406	0,086	0,04197219	1,121540922	0,891630417	4,720930233
ZKSCAN7	6,3574E-05	0,165458835	0,062	0	1	1,12152271	0,891644896	#DIV/0!
PHYH	0,000576308	0,165423669	0,281	0,074	1	1,121495374	0,89166663	3,797297297
FMNL3	1,59772E-07	0,165374351	0,469	0,094	0,003371341	1,121457036	0,891697112	4,989361702
PCDHGA12	9,12354E-07	0,165239729	0,094	0	0,019251588	1,121352395	0,891780322	#DIV/0!
HAUS4	0,000263702	0,165087304	0,281	0,07	1	1,121233927	0,891874546	4,014285714
BBIP1	0,00015856	0,165043498	0,312	0,078	1	1,121199882	0,891901628	4
SLC39A10	6,1182E-08	0,165038379	0,5	0,102	0,001291001	1,121195904	0,891904792	4,901960784
MSANTD4	6,93747E-09	0,164979603	0,25	0,02	0,000146388	1,121150227	0,89194113	12,5
MRPL50	1,8738E-08	0,164852004	0,469	0,082	0,000395392	1,121051071	0,892020021	5,719512195

Supplementary Table 1

Differentially expressed genes_adjacent-skin-XL_ comparing SC-Keloid vs. (SC-Myel, SC-Nonmyel)								
**gene**	**p_val**	**avg_log2FC**	**pct.1**	**pct.2**	**p_val_adj**	**Foldchange_UP**	**Foldchange_DOWN**	**Ratio_pct1_pct2**
TMEM177	1,57978E-10	0,164661146	0,219	0,008	3,33348E-06	1,120902774	0,892138036	27,375
KLHL4	9,12354E-07	0,164471826	0,094	0	0,019251588	1,120755692	0,892255116	#DIV/0!
C19orf48	1,54041E-05	0,164398443	0,312	0,062	0,325042363	1,120698686	0,892300502	5,032258065
NAXE	0,000540084	0,164341931	0,281	0,074	1	1,120654788	0,892335455	3,797297297
MSH2	1,08924E-08	0,164336596	0,312	0,035	0,000229841	1,120650643	0,892338755	8,914285714
NDUFAF2	3,23417E-06	0,164309456	0,406	0,09	0,068244172	1,120629562	0,892355542	4,511111111
MAMDC2	0,057775912	0,16424958	0,125	0,043	1	1,120583053	0,892392578	2,906976744
LNPK	0,000292494	0,164108804	0,281	0,07	1	1,120473714	0,892479661	4,014285714
ZNF444	0,000301839	0,164080606	0,312	0,082	1	1,120451814	0,892497105	3,804878049
METTL1	3,57983E-06	0,164070492	0,156	0,012	0,075538003	1,120443959	0,892503362	13
ARSD	0,033163773	0,163935732	0,188	0,07	1	1,120339305	0,892586733	2,685714286
FBXO32	0,038934481	0,163853079	0,062	0,012	1	1,120275122	0,892637871	5,166666667
HNRNPAB	2,24349E-06	0,163684773	0,688	0,195	0,04733988	1,120144437	0,892742013	3,528205128
PLIN3	9,68728E-06	0,163593051	0,531	0,145	0,204411223	1,120073224	0,892798773	3,662068966
SLC35F5	5,03836E-05	0,163586387	0,25	0,047	1	1,120068051	0,892802896	5,319148936
A2ML1	0,004841742	0,163375166	0,031	0	1	1,119904077	0,892933619	#DIV/0!
NCCRP1	0,004841742	0,163375166	0,031	0	1	1,119904077	0,892933619	#DIV/0!
WDR72	0,004841742	0,163375166	0,031	0	1	1,119904077	0,892933619	#DIV/0!
BABAM2	0,002478714	0,163259629	0,219	0,059	1	1,119814394	0,893005131	3,711864407
INKA1	9,12354E-07	0,163253611	0,094	0	0,019251588	1,119809722	0,893008857	#DIV/0!
C2orf72	1,33967E-08	0,163243023	0,125	0	0,000282684	1,119801504	0,893015411	#DIV/0!
FBXW5	5,29769E-05	0,163172111	0,375	0,094	1	1,119746465	0,893059305	3,989361702
TECPR2	0,000417537	0,163063608	0,188	0,035	1	1,119662253	0,893126474	5,371428571
IDI1	1,94445E-07	0,162984109	0,531	0,117	0,004102974	1,119600556	0,893175691	4,538461538
ARG2	1,39097E-05	0,162922591	0,125	0,008	0,293508116	1,119552817	0,893213777	15,625
SCGB1B2P	6,3574E-05	0,162917228	0,062	0	1	1,119548654	0,893217098	#DIV/0!
PHTF1	4,50344E-06	0,162761762	0,188	0,02	0,095027166	1,119428018	0,893313356	9,4
CUL1	0,016716716	0,162722376	0,188	0,062	1	1,119397457	0,893337745	3,032258065
IKBKG	0,00019555	0,162595444	0,188	0,031	1	1,119298975	0,893416346	6,064516129
FIS1	0,007532433	0,162513062	0,75	0,355	1	1,119235061	0,893467364	2,112676056

Supplementary Table 1

Differentially expressed genes_adjacent-skin-XL_ comparing SC-Keloid vs. (SC-Myel, SC-Nonmyel)								
**gene**	**p_val**	**avg_log2FC**	**pct.1**	**pct.2**	**p_val_adj**	**Foldchange_UP**	**Foldchange_DOWN**	**Ratio_pct1_pct2**
RPP30	5,14086E-05	0,162486727	0,312	0,07	1	1,119214631	0,893483674	4,457142857
LMNB1	1,44979E-05	0,162457644	0,125	0,008	0,305919948	1,119192069	0,893501685	15,625
PCCB	2,83613E-06	0,162416407	0,25	0,035	0,059845173	1,119160079	0,893527225	7,142857143
IPO13	1,90629E-05	0,162039241	0,188	0,023	0,402246146	1,118867533	0,893760852	8,173913043
HSF1	0,001325703	0,161987844	0,281	0,082	1	1,118827674	0,893792694	3,426829268
HDDC2	9,70761E-05	0,161937664	0,344	0,086	1	1,11878876	0,893823782	4
FADD	1,33967E-08	0,161606988	0,125	0	0,000282684	1,118532354	0,894028676	#DIV/0!
SAMM50	2,15242E-09	0,16154305	0,312	0,031	4,54182E-05	1,118482784	0,894068299	10,06451613
SLC39A4	2,9927E-06	0,161542022	0,25	0,035	0,063148942	1,118481987	0,894068936	7,142857143
PKMYT1	9,12354E-07	0,16150357	0,094	0	0,019251588	1,118452177	0,894092766	#DIV/0!
MTFR1L	0,000204257	0,161445463	0,281	0,066	1	1,11840713	0,894128778	4,257575758
DNAJB5	0,00292518	0,161419866	0,188	0,047	1	1,118387286	0,894144642	4
CFAP36	1,39438E-05	0,161244398	0,406	0,098	0,294228514	1,118251271	0,894253399	4,142857143
POMGNT2	4,5807E-05	0,1611377	0,094	0,004	0,966573495	1,118168571	0,894319538	23,5
RAD50	2,60711E-07	0,160930753	0,406	0,074	0,005501273	1,118008187	0,894447833	5,486486486
RASSF4	0,001275639	0,160766637	0,781	0,324	1	1,117881014	0,894549588	2,410493827
GRID2	0,079594089	0,160569263	0,031	0,004	1	1,117728088	0,894671979	7,75
FUT11	0,001560165	0,160545492	0,125	0,02	1	1,117709671	0,89468672	6,25
SPATA5L1	1,51098E-05	0,160397451	0,125	0,008	0,318831333	1,117594984	0,894778533	15,625
GALNT11	0,000390215	0,160381006	0,25	0,059	1	1,117582245	0,894788732	4,237288136
ZDHHC24	4,92471E-08	0,160376322	0,281	0,031	0,001039163	1,117578617	0,894791637	9,064516129
SDSL	2,86276E-12	0,160198119	0,188	0	6,04072E-08	1,117440581	0,89490217	#DIV/0!
NFU1	8,87757E-06	0,160186387	0,344	0,07	0,187325628	1,117431494	0,894909447	4,914285714
PHF19	3,51435E-06	0,160038359	0,344	0,066	0,074156248	1,117316845	0,895001274	5,212121212
NFE2L3	1,51098E-05	0,159993995	0,125	0,008	0,318831333	1,117282487	0,895028797	15,625
TRIB2	7,25857E-06	0,159991402	0,344	0,07	0,153163084	1,11728048	0,895030405	4,914285714
ACSL1	0,032396086	0,159969546	0,188	0,07	1	1,117263553	0,895043964	2,685714286
PDIA5	2,24684E-05	0,159928337	0,156	0,016	0,474104712	1,11723164	0,895069531	9,75
PKP1	4,80509E-06	0,159853808	0,188	0,02	0,101392141	1,117173926	0,895115771	9,4
TRAF5	0,002263143	0,159808605	0,062	0,004	1	1,117138923	0,895143817	15,5

Supplementary Table 1

Differentially expressed genes_adjacent-skin-XL_ comparing SC-Keloid vs. (SC-Myel, SC-Nonmyel)								
**gene**	**p_val**	**avg_log2FC**	**pct.1**	**pct.2**	**p_val_adj**	**Foldchange_UP**	**Foldchange_DOWN**	**Ratio_pct1_pct2**
WFS1	0,000313864	0,159736429	0,156	0,023	1	1,117083036	0,895188601	6,782608696
LIG3	3,71921E-06	0,159318357	0,156	0,012	0,078479117	1,116759368	0,895448051	13
HMGB3	4,82268E-05	0,159314912	0,25	0,047	1	1,116756701	0,89545019	5,319148936
EML1	2,02073E-05	0,158794994	0,188	0,023	0,426394579	1,116354317	0,89577295	8,173913043
VPS41	1,03682E-05	0,158784602	0,281	0,051	0,218779044	1,116346276	0,895779402	5,509803922
DNTTIP1	1,32717E-05	0,158595785	0,281	0,051	0,280046571	1,116200181	0,895896648	5,509803922
RPS6KA1	0,001808729	0,158536614	0,156	0,031	1	1,116154402	0,895933393	5,032258065
WDR13	0,000896083	0,158337449	0,5	0,18	1	1,116000327	0,896057085	2,777777778
APEH	1,99574E-07	0,158206873	0,281	0,035	0,004211201	1,115899323	0,89613819	8,028571429
ZNF720	0,007945492	0,158011944	0,125	0,027	1	1,115748559	0,89625928	4,62962963
SMAD7	8,53227E-07	0,157881429	0,188	0,016	0,018003947	1,115647627	0,896340364	11,75
KIF1C	9,69747E-05	0,15780716	0,281	0,062	1	1,115590195	0,896386509	4,532258065
MOCS2	3,10173E-05	0,157638218	0,5	0,145	0,654496234	1,115459566	0,896491483	3,448275862
MEX3A	4,65192E-06	0,157564023	0,188	0,02	0,098160247	1,115402201	0,896537589	9,4
PLA2G15	7,96166E-09	0,157263479	0,188	0,008	0,000167999	1,115169863	0,896724376	23,5
CD58	1,85164E-05	0,15716048	0,312	0,062	0,39071388	1,11509025	0,896788399	5,032258065
GMPPA	9,73699E-05	0,157078866	0,156	0,02	1	1,115027171	0,896839132	7,8
CDK20	2,10199E-05	0,156941949	0,156	0,016	0,443540004	1,114921356	0,896924249	9,75
POMGNT1	8,00274E-06	0,156908193	0,312	0,059	0,168865745	1,11489527	0,896945235	5,288135593
ABCC10	0,216675772	0,15690461	0,031	0,008	1	1,1148925	0,896947463	3,875
YKT6	1,38976E-05	0,15679026	0,312	0,062	0,293252516	1,114804136	0,897018559	5,032258065
AHCY	2,7748E-05	0,156768626	0,375	0,09	0,585509559	1,114787419	0,897032011	4,166666667
ENTR1	1,41893E-05	0,15669152	0,281	0,051	0,29940858	1,11472784	0,897079955	5,509803922
CD164	0,000524773	0,156688862	0,625	0,242	1	1,114725786	0,897081607	2,582644628
STX7	0,000349957	0,156622664	0,406	0,125	1	1,114674638	0,897122771	3,248
SLC31A1	3,51691E-07	0,15658294	0,156	0,008	0,007421029	1,114643946	0,897147473	19,5
LRFN4	0,000104629	0,156105421	0,281	0,062	1	1,114275071	0,89744447	4,532258065
CEP135	0,001808729	0,156067962	0,156	0,031	1	1,114246139	0,897467772	5,032258065
PCNX4	0,000208933	0,156016905	0,219	0,043	1	1,114206706	0,897499534	5,093023256
HDAC2	0,001051807	0,155894533	0,344	0,109	1	1,114112202	0,897575665	3,155963303

Supplementary Table 1

Differentially expressed genes_adjacent-skin-XL_ comparing SC-Keloid vs. (SC-Myel, SC-Nonmyel)								
**gene**	**p_val**	**avg_log2FC**	**pct.1**	**pct.2**	**p_val_adj**	**Foldchange_UP**	**Foldchange_DOWN**	**Ratio_pct1_pct2**
RUVBL1	4,25761E-07	0,155687231	0,312	0,047	0,008983981	1,113952125	0,897704648	6,638297872
E2F5	5,49744E-05	0,155596546	0,25	0,047	1	1,113882107	0,897761077	5,319148936
MRPS15	6,85015E-05	0,155230046	0,406	0,109	1	1,113599174	0,897989172	3,724770642
IFTAP	7,07015E-08	0,15515597	0,375	0,059	0,001491872	1,113541997	0,898035281	6,355932203
KANSL1	0,000693449	0,155065963	0,406	0,133	1	1,113472527	0,898091309	3,052631579
COL9A3	0,00717229	0,15489348	0,875	0,434	1	1,113339413	0,898198688	2,016129032
UTP4	0,000173407	0,154634698	0,188	0,031	1	1,113139727	0,898359816	6,064516129
DCAF15	1,06809E-06	0,154466956	0,219	0,023	0,022537726	1,11301031	0,898464274	9,52173913
AUH	0,057775912	0,154435442	0,125	0,043	1	1,112985997	0,898483901	2,906976744
HPS4	0,002478699	0,154296035	0,219	0,059	1	1,112878455	0,898570725	3,711864407
EIF2AK1	3,39387E-07	0,154246745	0,469	0,098	0,007161415	1,112840434	0,898601425	4,785714286
ANAPC5	0,000141727	0,154214458	0,531	0,176	1	1,112815529	0,898621536	3,017045455
KCND3	4,5807E-05	0,154057899	0,094	0,004	0,966573495	1,112694775	0,898719058	23,5
SLC2A1	0,024054161	0,153870925	0,125	0,035	1	1,112550579	0,89883554	3,571428571
POGLUT2	9,06462E-07	0,15380567	0,125	0,004	0,019127258	1,112500258	0,898876196	31,25
POPDC3	4,50344E-06	0,153713069	0,188	0,02	0,095027166	1,112428853	0,898933894	9,4
MIEN1	0,00016416	0,153711362	0,438	0,133	1	1,112427537	0,898934957	3,293233083
CSDE1	0,007675956	0,15356966	0,844	0,402	1	1,112318279	0,899023255	2,099502488
CZIB	1,46573E-06	0,153372865	0,562	0,141	0,030928364	1,11216656	0,899145898	3,985815603
TUBB6	4,67306E-06	0,153209086	0,531	0,141	0,098606208	1,112040311	0,899247977	3,765957447
STYX	0,000100263	0,153186982	0,156	0,02	1	1,112023273	0,899261755	7,8
C16orf91	4,70541E-06	0,153092748	0,281	0,047	0,099288927	1,11195064	0,899320495	5,978723404
MRPL55	3,70548E-08	0,153092571	0,5	0,098	0,000781893	1,111950504	0,899320605	5,102040816
TMEM199	0,000100263	0,153084072	0,156	0,02	1	1,111943953	0,899325903	7,8
MYCBP	7,96166E-09	0,152971385	0,188	0,008	0,000167999	1,111857105	0,899396151	23,5
ZFYVE9	0,000811202	0,152735414	0,156	0,027	1	1,111675261	0,899543271	5,777777778
PSME1	0,001777154	0,152716424	0,812	0,348	1	1,111660628	0,899555111	2,333333333
SNX9	0,000205019	0,152659593	0,25	0,055	1	1,111616839	0,899590547	4,545454545
TIMELESS	1,59386E-08	0,152596723	0,156	0,004	0,000336319	1,111568397	0,899629751	39
RNASEL	0,002517564	0,15250064	0,094	0,012	1	1,11149437	0,899689668	7,833333333

Supplementary Table 1

Differentially expressed genes_adjacent-skin-XL_ comparing SC-Keloid vs. (SC-Myel, SC-Nonmyel)								
**gene**	**p_val**	**avg_log2FC**	**pct.1**	**pct.2**	**p_val_adj**	**Foldchange_UP**	**Foldchange_DOWN**	**Ratio_pct1_pct2**
PSMD6	5,67461E-05	0,152378167	0,5	0,145	1	1,111400017	0,899766047	3,448275862
FBLIM1	4,80439E-05	0,152333719	0,094	0,004	1	1,111365776	0,899793768	23,5
AAMP	9,1055E-05	0,15233333	0,438	0,125	1	1,111365477	0,899794011	3,504
VPS53	0,00151347	0,152277442	0,188	0,043	1	1,111322424	0,899828869	4,372093023
OSER1-DT	0,000112833	0,152215654	0,125	0,012	1	1,111274829	0,899867408	10,41666667
GLB1L	6,3574E-05	0,152204898	0,062	0	1	1,111266544	0,899874117	#DIV/0!
HMOX2	4,71723E-06	0,15220079	0,406	0,09	0,099538323	1,11126338	0,899876679	4,511111111
RAD23A	0,008100744	0,152162964	0,562	0,25	1	1,111234244	0,899900273	2,248
PHKG2	0,000173407	0,152052549	0,188	0,031	1	1,111149201	0,899969148	6,064516129
TBC1D7	3,51435E-06	0,151576108	0,344	0,066	0,074156248	1,110782311	0,900266407	5,212121212
NOP56	0,000280811	0,151519123	0,406	0,121	1	1,110738438	0,900301967	3,355371901
PGM2	8,84735E-07	0,151447326	0,188	0,016	0,018668785	1,110683162	0,900346773	11,75
MRPL22	2,16766E-06	0,151370996	0,469	0,109	0,045739869	1,1106244	0,900394409	4,302752294
ZNF281	0,006607735	0,151355547	0,156	0,039	1	1,110612506	0,900404051	4
RRP7A	6,73191E-06	0,151115715	0,312	0,059	0,142049988	1,110427894	0,900553746	5,288135593
UPK3BL1	0,00011859	0,150981967	0,375	0,102	1	1,110324955	0,900637237	3,676470588
KIF3A	0,001385681	0,150823743	0,281	0,082	1	1,110203189	0,900736018	3,426829268
ODF2	2,17326E-05	0,150705738	0,156	0,016	0,458580025	1,110112384	0,900809696	9,75
SNX4	9,38646E-05	0,150662721	0,344	0,086	1	1,110079284	0,900836557	4
SLC4A3	0,039886012	0,15062022	0,062	0,012	1	1,110046583	0,900863095	5,166666667
ENAH	0,00079237	0,150464385	0,156	0,027	1	1,109926686	0,900960408	5,777777778
TTC1	2,1104E-05	0,150421903	0,469	0,125	0,445315492	1,109894003	0,900986939	3,752
RDH11	6,07698E-05	0,150046627	0,375	0,098	1	1,109605334	0,901221335	3,826530612
ZNF593	1,82474E-09	0,149893332	0,406	0,055	3,85039E-05	1,109487437	0,9013171	7,381818182
CATSPER1	6,3574E-05	0,149861183	0,062	0	1	1,109462714	0,901337186	#DIV/0!
DCLK1	0,216675772	0,14981673	0,031	0,008	1	1,109428529	0,901364959	3,875
TMEM9B	0,000513154	0,149808127	0,375	0,113	1	1,109421913	0,901370333	3,318584071
COA8	9,55566E-06	0,149331276	0,375	0,082	0,201634002	1,109055279	0,901668311	4,573170732
POLE	0,000108998	0,149117564	0,125	0,012	1	1,108891003	0,901801888	10,41666667
TOMM40	0,006373067	0,149040876	0,312	0,117	1	1,10883206	0,901849826	2,666666667

Supplementary Table 1

Differentially expressed genes_adjacent-skin-XL_ comparing SC-Keloid vs. (SC-Myel, SC-Nonmyel)								
**gene**	**p_val**	**avg_log2FC**	**pct.1**	**pct.2**	**p_val_adj**	**Foldchange_UP**	**Foldchange_DOWN**	**Ratio_pct1_pct2**
MARK2	0,01796492	0,148926456	0,219	0,078	1	1,108744122	0,901921354	2,807692308
SLC46A3	9,06462E-07	0,148820576	0,125	0,004	0,019127258	1,108662754	0,901987549	31,25
RAB4B	0,000196043	0,148634156	0,219	0,043	1	1,108519505	0,902104109	5,093023256
SEPHS2	5,82109E-10	0,148559094	0,375	0,043	1,22831E-05	1,108461832	0,902151045	8,720930233
MGMT	1,29375E-05	0,148271173	0,344	0,074	0,272994515	1,108240636	0,902331107	4,648648649
SCG5	1,59386E-08	0,148185245	0,156	0,004	0,000336319	1,108174631	0,902384852	39
TRMT12	0,039886012	0,148142418	0,062	0,012	1	1,108141734	0,90241164	5,166666667
SNAPIN	7,5014E-06	0,147972334	0,312	0,059	0,158287075	1,1080111	0,902518034	5,288135593
LINC01137	7,96166E-09	0,147963817	0,188	0,008	0,000167999	1,108004559	0,902523362	23,5
UTP18	5,26316E-05	0,147893312	0,25	0,047	1	1,107950412	0,90256747	5,319148936
TMX4	2,23171E-05	0,147851342	0,562	0,168	0,470913836	1,10791818	0,902593727	3,345238095
ACP2	2,40115E-05	0,147810201	0,156	0,016	0,506667402	1,107886586	0,902619467	9,75
NINJ1	9,41785E-05	0,147373227	0,625	0,207	1	1,107551072	0,902892901	3,019323671
DHPS	1,99857E-05	0,14726964	0,438	0,113	0,421717445	1,107471552	0,902957732	3,876106195
TASOR	0,000595256	0,147106226	0,281	0,074	1	1,107346116	0,903060015	3,797297297
BIN3	0,000105292	0,147095551	0,312	0,074	1	1,107337922	0,903066697	4,216216216
ADGRL4	3,67695E-07	0,146962544	0,156	0,008	0,007758733	1,107235838	0,903149958	19,5
YARS2	1,07001E-07	0,146791351	0,188	0,012	0,002257818	1,107104458	0,903257134	15,66666667
NCAPG	6,3574E-05	0,146750007	0,062	0	1	1,107072732	0,903283019	#DIV/0!
GCNT1	1,59386E-08	0,146594399	0,156	0,004	0,000336319	1,10695333	0,903380452	39
UBA5	0,00022736	0,146514849	0,219	0,043	1	1,106892295	0,903430266	5,093023256
KCNE4	0,004180266	0,14651157	0,125	0,023	1	1,106889779	0,903432319	5,434782609
ZFAND3	0,000253739	0,146453388	0,5	0,16	1	1,106845141	0,903468754	3,125
RSBN1	0,001170364	0,146415406	0,344	0,109	1	1,106816001	0,90349254	3,155963303
MVD	9,25578E-07	0,146230351	0,375	0,07	0,019530631	1,106674038	0,903608439	5,357142857
SSC5D	1,93924E-05	0,146229282	0,5	0,137	0,409199075	1,106673219	0,903609108	3,649635036
RIOK1	0,000446084	0,146220563	0,188	0,035	1	1,106666531	0,903614569	5,371428571
DCLRE1C	2,39232E-07	0,145814392	0,25	0,027	0,005048036	1,106355008	0,903869005	9,259259259
PLEKHO1	0,000314345	0,145783033	0,562	0,195	1	1,10633096	0,903888652	2,882051282
USP22	1,79699E-06	0,145758758	0,656	0,184	0,03791833	1,106312344	0,903903861	3,565217391

Supplementary Table 1

Differentially expressed genes_adjacent-skin-XL_ comparing SC-Keloid vs. (SC-Myel, SC-Nonmyel)								
**gene**	**p_val**	**avg_log2FC**	**pct.1**	**pct.2**	**p_val_adj**	**Foldchange_UP**	**Foldchange_DOWN**	**Ratio_pct1_pct2**
FAHD2B	0,000297876	0,145756368	0,156	0,023	1	1,106310511	0,903905359	6,782608696
RAB11B-AS1	9,06462E-07	0,14575451	0,125	0,004	0,019127258	1,106309086	0,903906523	31,25
ZNF576	0,002370322	0,145685028	0,094	0,012	1	1,106255807	0,903950057	7,833333333
ALG3	9,85458E-05	0,145628959	0,219	0,039	1	1,106212814	0,903985189	5,615384615
TSEN2	0,520968078	0,145594814	0,031	0,016	1	1,106186633	0,904006584	1,9375
TRMT1	0,001503327	0,145472984	0,219	0,055	1	1,106093224	0,904082928	3,981818182
COPB2	0,000902011	0,145365203	0,406	0,137	1	1,106010593	0,904150472	2,96350365
CD302	0,011423236	0,145345522	0,156	0,043	1	1,105995505	0,904162807	3,627906977
SUGP1	0,000439193	0,145306611	0,125	0,016	1	1,105965675	0,904187194	7,8125
GINM1	7,02651E-06	0,145294545	0,625	0,184	0,148266402	1,105956426	0,904194756	3,39673913
FKBP2	0,00858429	0,145077524	0,281	0,102	1	1,105790072	0,904330781	2,754901961
SLC35F6	2,08038E-05	0,145050626	0,188	0,023	0,438980454	1,105769456	0,904347642	8,173913043
SLC45A3	0,37426516	0,144956081	0,031	0,012	1	1,105696993	0,904406909	2,583333333
DHCR24	1,59386E-08	0,14491775	0,156	0,004	0,000336319	1,105667616	0,904430939	39
CCDC88A	4,80509E-06	0,144858335	0,188	0,02	0,101392141	1,105622082	0,904468187	9,4
NAE1	0,000103442	0,144784751	0,312	0,074	1	1,105565691	0,904514321	4,216216216
TTC19	7,10311E-07	0,144725551	0,438	0,09	0,014988265	1,105520326	0,904551437	4,866666667
PTPN9	2,32278E-05	0,144517969	0,156	0,016	0,490128791	1,10536127	0,904681598	9,75
TOR1B	0,000112833	0,144497112	0,125	0,012	1	1,10534529	0,904694677	10,41666667
DLK2	0,004841742	0,14437834	0,031	0	1	1,105254294	0,90476916	#DIV/0!
KCNH2	0,004841742	0,14437834	0,031	0	1	1,105254294	0,90476916	#DIV/0!
SPATA2L	0,007089636	0,144376113	0,156	0,039	1	1,105252588	0,904770557	4
AKAP1	0,000494525	0,144343671	0,125	0,016	1	1,105227735	0,904790903	7,8125
CCNDBP1	0,000561747	0,144285211	0,375	0,117	1	1,105182951	0,904827567	3,205128205
FBXO22	2,29902E-09	0,144111067	0,312	0,031	4,85116E-05	1,105049555	0,904936793	10,06451613
CTPS2	0,000509339	0,144038424	0,125	0,016	1	1,104993915	0,904982359	7,8125
DIRAS3	0,002168448	0,144017939	0,062	0,004	1	1,104978224	0,90499521	15,5
NCBP1	2,75671E-08	0,144015375	0,219	0,016	0,000581693	1,104976261	0,904996818	13,6875
MSTN	0,002168448	0,144013907	0,062	0,004	1	1,104975136	0,904997739	15,5
COMMD5	4,14985E-07	0,143854789	0,312	0,047	0,008756602	1,104853273	0,905097559	6,638297872

Supplementary Table 1

Differentially expressed genes_adjacent-skin-XL_ comparing SC-Keloid vs. (SC-Myel, SC-Nonmyel)								
**gene**	**p_val**	**avg_log2FC**	**pct.1**	**pct.2**	**p_val_adj**	**Foldchange_UP**	**Foldchange_DOWN**	**Ratio_pct1_pct2**
MLLT1	0,02536082	0,143437805	0,25	0,102	1	1,104533982	0,905359198	2,450980392
VPS45	0,008576675	0,143424671	0,188	0,055	1	1,104523927	0,90536744	3,418181818
TAF11	4,45925E-06	0,14300005	0,406	0,09	0,094094732	1,104198886	0,905633951	4,511111111
SRP54	6,95585E-05	0,14295571	0,438	0,121	1	1,10416495	0,905661786	3,619834711
TMOD3	1,66591E-06	0,142648173	0,406	0,086	0,035152316	1,103929602	0,905854865	4,720930233
DZIP1	0,033585021	0,142630313	0,094	0,023	1	1,103915935	0,905866079	4,086956522
SSUH2	0,00819789	0,142615151	0,188	0,055	1	1,103904333	0,9058756	3,418181818
STX18-AS1	0,216675772	0,142412158	0,031	0,008	1	1,103749021	0,906003069	3,875
NAGLU	0,000789392	0,142305506	0,219	0,051	1	1,103667428	0,906070048	4,294117647
NOTCH3	2,40115E-05	0,142207469	0,156	0,016	0,506667402	1,103592433	0,906131621	9,75
TCTN3	0,001201874	0,141868093	0,344	0,109	1	1,103332857	0,906344802	3,155963303
RPL23	0,070616355	0,141659381	0,906	0,613	1	1,103173251	0,906475931	1,477977162
ZDHHC1	2,24684E-05	0,141511136	0,156	0,016	0,474104712	1,103059899	0,906569082	9,75
GMPPB	5,12603E-06	0,141486628	0,188	0,02	0,108164307	1,103041162	0,906584482	9,4
PPIL1	8,84735E-07	0,141459566	0,188	0,016	0,018668785	1,103020471	0,906601488	11,75
LPCAT1	0,002594281	0,141357445	0,094	0,012	1	1,102942396	0,906665664	7,833333333
FURIN	0,00259611	0,141249841	0,25	0,074	1	1,102860136	0,90673329	3,378378378
CAAP1	2,24684E-05	0,141242403	0,156	0,016	0,474104712	1,10285445	0,906737965	9,75
LAPTM4A	0,073021569	0,141156169	0,938	0,57	1	1,102788531	0,906792165	1,645614035
FKBP14	5,908E-06	0,141069091	0,312	0,059	0,124664638	1,102721972	0,906846899	5,288135593
AGA	2,67934E-05	0,140915574	0,375	0,09	0,565368181	1,102604637	0,906943402	4,166666667
USO1	1,06763E-06	0,140598182	0,438	0,094	0,022527994	1,102362092	0,90714295	4,659574468
GPSM3	2,39232E-07	0,140458223	0,25	0,027	0,005048036	1,102255154	0,907230959	9,259259259
TM2D3	1,64883E-06	0,140452808	0,5	0,117	0,034791873	1,102251017	0,907234364	4,273504274
MED8	5,12603E-06	0,140298393	0,188	0,02	0,108164307	1,102133047	0,907331472	9,4
MPHOSPH10	9,87205E-05	0,140225892	0,344	0,086	1	1,102077662	0,90737707	4
SCG2	0,002263143	0,140211556	0,062	0,004	1	1,102066711	0,907386087	15,5
NPDC1	0,004150422	0,140143672	0,531	0,227	1	1,102014856	0,907428784	2,339207048
SCGB1D2	0,082317662	0,140052841	0,031	0,004	1	1,101945476	0,907485917	7,75
TAPBP	2,98435E-05	0,139913467	0,688	0,223	0,629727684	1,101839025	0,90757359	3,085201794

Supplementary Table 1

Differentially expressed genes_adjacent-skin-XL_ comparing SC-Keloid vs. (SC-Myel, SC-Nonmyel)								
**gene**	**p_val**	**avg_log2FC**	**pct.1**	**pct.2**	**p_val_adj**	**Foldchange_UP**	**Foldchange_DOWN**	**Ratio_pct1_pct2**
CLTB	0,003928859	0,139865405	0,688	0,297	1	1,101802319	0,907603826	2,316498316
SLC27A4	2,33183E-10	0,139809569	0,281	0,02	4,92039E-06	1,101759678	0,907638953	14,05
SLC25A1	0,00010663	0,139349039	0,281	0,062	1	1,101408035	0,907928731	4,532258065
HAUS6	0,000494525	0,139303323	0,125	0,016	1	1,101373135	0,907957502	7,8125
RFT1	0,001696757	0,139292125	0,156	0,031	1	1,101364586	0,907964549	5,032258065
MTERF3	0,000305772	0,139244501	0,156	0,023	1	1,10132823	0,907994522	6,782608696
HEXIM2	3,86379E-06	0,139115868	0,156	0,012	0,081529835	1,101230038	0,908075484	13
VPS39	2,1417E-05	0,139110892	0,188	0,023	0,451919526	1,10122624	0,908078616	8,173913043
FAF1	1,4531E-05	0,139064964	0,344	0,074	0,306618748	1,101191184	0,908107525	4,648648649
LRRC23	0,000789392	0,13899374	0,219	0,051	1	1,10113682	0,908152358	4,294117647
KANSL1-AS1	1,51098E-05	0,138664363	0,125	0,008	0,318831333	1,100885452	0,90835972	15,625
E2F7	9,06462E-07	0,138629672	0,125	0,004	0,019127258	1,100858981	0,908381562	31,25
EXOSC7	3,13462E-07	0,138500182	0,344	0,055	0,006614354	1,100760176	0,908463098	6,254545455
RNF126	0,036468388	0,138271182	0,312	0,141	1	1,100585466	0,908607311	2,212765957
FANCF	3,71921E-06	0,138229608	0,156	0,012	0,078479117	1,100553751	0,908633494	13
FOXD2	0,079594089	0,138180242	0,031	0,004	1	1,100516093	0,908664586	7,75
SLC25A34	0,082317662	0,138177072	0,031	0,004	1	1,100513675	0,908666583	7,75
CDC34	1,05494E-07	0,137935822	0,406	0,07	0,00222602	1,10032966	0,908818544	5,8
CCDC113	0,002263143	0,137478428	0,062	0,004	1	1,099980866	0,909106723	15,5
CRYBB2	1,33967E-08	0,137276601	0,125	0	0,000282684	1,099826993	0,909233912	#DIV/0!
SCML2	0,079594089	0,137186979	0,031	0,004	1	1,099758673	0,909290396	7,75
HAS2-AS1	0,088161845	0,137164524	0,094	0,031	1	1,099741556	0,909304549	3,032258065
PXYLP1	0,001560165	0,13713671	0,125	0,02	1	1,099720354	0,90932208	6,25
DCPS	1,60002E-05	0,137132218	0,344	0,074	0,337621055	1,09971693	0,909324912	4,648648649
CASP3	0,000103442	0,137114582	0,312	0,074	1	1,099703487	0,909336028	4,216216216
RRP36	8,54054E-06	0,137095415	0,25	0,039	0,18021385	1,099688877	0,909348109	6,41025641
RCC1	9,17361E-07	0,137090132	0,188	0,016	0,019357231	1,09968485	0,909351438	11,75
LIG4	2,32278E-05	0,13694204	0,156	0,016	0,490128791	1,099571973	0,909444788	9,75
B4GALT2	6,07138E-07	0,136882771	0,281	0,039	0,012811221	1,099526802	0,90948215	7,205128205
PALM2AKAP2	1,21997E-06	0,136814313	0,875	0,277	0,025742608	1,099474628	0,909525308	3,158844765

Supplementary Table 1

Differentially expressed genes_adjacent-skin-XL_ comparing SC-Keloid vs. (SC-Myel, SC-Nonmyel)								
**gene**	**p_val**	**avg_log2FC**	**pct.1**	**pct.2**	**p_val_adj**	**Foldchange_UP**	**Foldchange_DOWN**	**Ratio_pct1_pct2**
KIRREL1	8,54831E-05	0,136696775	0,375	0,098	1	1,099385057	0,909599411	3,826530612
RAPH1	5,74157E-05	0,136542142	0,25	0,047	1	1,099267227	0,90969691	5,319148936
CXorf38	5,74157E-05	0,136524772	0,25	0,047	1	1,099253992	0,909707863	5,319148936
ATP7A	0,006844876	0,136458365	0,156	0,039	1	1,099203395	0,909749737	4
LONP1	9,71531E-07	0,136445575	0,25	0,031	0,020500283	1,09919365	0,909757803	8,064516129
CCDC32	0,000217964	0,136420662	0,219	0,043	1	1,099174669	0,909773513	5,093023256
RINL	6,3574E-05	0,136312304	0,062	0	1	1,099092116	0,909841847	#DIV/0!
FNTA	0,004207321	0,136298654	0,531	0,219	1	1,099081716	0,909850456	2,424657534
ECSIT	2,07726E-05	0,136292467	0,25	0,043	0,438322831	1,099077003	0,909854357	5,813953488
PTS	2,22069E-05	0,136034231	0,344	0,078	0,468587791	1,09888029	0,910017232	4,41025641
ELOVL6	9,06462E-07	0,135896365	0,125	0,004	0,019127258	1,098775285	0,910104198	31,25
NINL	0,079594089	0,135807863	0,031	0,004	1	1,098707882	0,910160031	7,75
ENTPD6	1,00234E-06	0,135781986	0,219	0,023	0,021150457	1,098688176	0,910176356	9,52173913
GTF2H3	4,123E-05	0,135731579	0,219	0,035	0,869994633	1,098649789	0,910208158	6,257142857
MCRS1	2,82309E-05	0,135723019	0,312	0,066	0,595699315	1,098643271	0,910213558	4,727272727
PCK2	1,51098E-05	0,135673979	0,125	0,008	0,318831333	1,098605926	0,910244498	15,625
OXTR	9,12354E-07	0,135652768	0,094	0	0,019251588	1,098589774	0,910257881	#DIV/0!
VPS18	0,207161335	0,135520702	0,062	0,023	1	1,098489212	0,910341211	2,695652174
CDKN2AIPNL	9,18193E-05	0,135498559	0,156	0,02	1	1,098472352	0,910355184	7,8
SIRT2	0,002824026	0,13548169	0,5	0,203	1	1,098459508	0,910365828	2,463054187
PSMC4	7,09557E-07	0,13541173	0,562	0,137	0,014972355	1,098406243	0,910409975	4,102189781
GOLM1	5,26316E-05	0,135328018	0,25	0,047	1	1,09834251	0,910462803	5,319148936
TMEM234	9,17361E-07	0,13528504	0,188	0,016	0,019357231	1,098309791	0,910489926	11,75
SCN1B	5,03836E-05	0,135238421	0,25	0,047	1	1,0982743	0,910519348	5,319148936
NFKBIL1	4,39767E-08	0,135221661	0,312	0,039	0,000927952	1,098261542	0,910529926	8
MTMR14	1,06809E-06	0,13518685	0,219	0,023	0,022537726	1,098235042	0,910551896	9,52173913
EID2B	0,204327649	0,135037023	0,062	0,023	1	1,098120994	0,910646464	2,695652174
ICAM3	6,466E-11	0,13486558	0,25	0,012	1,36439E-06	1,097990506	0,910754687	20,83333333
MACROD1	9,71531E-07	0,134815415	0,25	0,031	0,020500283	1,097952328	0,910786356	8,064516129
ATG9A	0,000850121	0,134766292	0,156	0,027	1	1,097914944	0,910817368	5,777777778

Supplementary Table 1

Differentially expressed genes_adjacent-skin-XL_ comparing SC-Keloid vs. (SC-Myel, SC-Nonmyel)								
**gene**	**p_val**	**avg_log2FC**	**pct.1**	**pct.2**	**p_val_adj**	**Foldchange_UP**	**Foldchange_DOWN**	**Ratio_pct1_pct2**
DHCR7	1,33967E-08	0,134724443	0,125	0	0,000282684	1,097883096	0,91084379	#DIV/0!
EDC4	0,01198915	0,134703882	0,156	0,043	1	1,09786745	0,910856771	3,627906977
DBR1	0,002517564	0,13469954	0,094	0,012	1	1,097864146	0,910859512	7,833333333
GALNS	0,002442928	0,134571875	0,094	0,012	1	1,097766999	0,910940119	7,833333333
SNAP29	0,00819789	0,134455233	0,188	0,055	1	1,097678248	0,911013771	3,418181818
NUS1	2,33422E-05	0,134446883	0,25	0,043	0,492544426	1,097671895	0,911019043	5,813953488
ADPRM	0,000100263	0,134255115	0,156	0,02	1	1,097525999	0,911140147	7,8
ATE1	0,000451131	0,134162911	0,219	0,047	1	1,097455857	0,911198381	4,659574468
JAM3	0,000452944	0,134123393	0,469	0,156	1	1,097425796	0,911223341	3,006410256
FES	0,090719467	0,133840868	0,094	0,031	1	1,097210906	0,911401804	3,032258065
NUDT18	9,12354E-07	0,133660172	0,094	0	0,019251588	1,09707349	0,911515964	#DIV/0!
SARAF	0,025723822	0,133606175	0,812	0,434	1	1,09703243	0,911550081	1,870967742
ZNF582	0,079594089	0,133546009	0,031	0,004	1	1,096986681	0,911588096	7,75
TAF9B	8,53227E-07	0,133511021	0,188	0,016	0,018003947	1,096960077	0,911610204	11,75
TBCC	0,024533893	0,133440528	0,188	0,066	1	1,096906478	0,911654749	2,848484848
PCYT2	0,00022736	0,133302153	0,219	0,043	1	1,096801275	0,911742193	5,093023256
U2SURP	0,001102349	0,133229161	0,656	0,262	1	1,096745784	0,911788324	2,503816794
REEP2	1,53257E-05	0,133210694	0,219	0,031	0,323387973	1,096731746	0,911799994	7,064516129
SEMA6A	2,40115E-05	0,13314884	0,156	0,016	0,506667402	1,096684726	0,911839088	9,75
DAZAP2	0,000911191	0,133123842	0,594	0,227	1	1,096665723	0,911854888	2,616740088
ATR	0,014352989	0,133099845	0,125	0,031	1	1,096647482	0,911870055	4,032258065
INTS14	0,017290773	0,133041106	0,094	0,02	1	1,096602833	0,911907183	4,7
ACAP2	0,000482061	0,132768019	0,5	0,172	1	1,096395277	0,912079814	2,906976744
ETAA1	0,00050526	0,132611628	0,094	0,008	1	1,096276432	0,91217869	11,75
TTC13	0,000494525	0,132523186	0,125	0,016	1	1,096209229	0,912234611	7,8125
GNS	0,024931667	0,132511349	0,219	0,082	1	1,096200235	0,912242096	2,670731707
COQ5	9,49112E-05	0,132444161	0,25	0,051	1	1,096149185	0,912284581	4,901960784
FAM229A	0,000850121	0,13238774	0,156	0,027	1	1,096106317	0,91232026	5,777777778
MRGBP	2,03907E-07	0,132309028	0,219	0,02	0,004302644	1,096046517	0,912370036	10,95
SMC3	0,001213208	0,132267738	0,531	0,199	1	1,096015148	0,912396148	2,668341709

Supplementary Table 1

Differentially expressed genes_adjacent-skin-XL_ comparing SC-Keloid vs. (SC-Myel, SC-Nonmyel)								
**gene**	**p_val**	**avg_log2FC**	**pct.1**	**pct.2**	**p_val_adj**	**Foldchange_UP**	**Foldchange_DOWN**	**Ratio_pct1_pct2**
UBB	6,55085E-05	0,131906213	0,906	0,387	1	1,095740532	0,912624815	2,341085271
DGKQ	0,37426516	0,131792415	0,031	0,012	1	1,095654105	0,912696804	2,583333333
SFRP2	0,221475664	0,131720028	0,031	0,008	1	1,095599132	0,9127426	3,875
DOK4	1,51098E-05	0,131541168	0,125	0,008	0,318831333	1,095463312	0,912855765	15,625
ANO1	0,000524345	0,131486061	0,094	0,008	1	1,095421469	0,912890635	11,75
SMIM27	0,000341249	0,131400349	0,281	0,07	1	1,095356391	0,912944872	4,014285714
ABHD13	6,26921E-05	0,131310315	0,188	0,027	1	1,095288035	0,913001848	6,962962963
LRTOMT	0,002168448	0,131143897	0,062	0,004	1	1,095161698	0,913107171	15,5
KIF20B	0,038520178	0,131061275	0,125	0,039	1	1,095098981	0,913159465	3,205128205
KAT5	0,000764302	0,130961653	0,25	0,062	1	1,095023365	0,913222523	4,032258065
PEX3	6,6835E-09	0,130905905	0,25	0,02	0,000141028	1,094981052	0,913257812	12,5
CCDC43	0,000777418	0,130706189	0,25	0,062	1	1,094829482	0,913384245	4,032258065
GTF2F2	3,40485E-06	0,13066874	0,5	0,121	0,071845702	1,094801063	0,913407955	4,132231405
ZNF239	0,079594089	0,130631475	0,031	0,004	1	1,094772784	0,913431549	7,75
SV2A	1,33967E-08	0,130552796	0,125	0	0,000282684	1,094713081	0,913481365	#DIV/0!
TNFSF13B	6,3574E-05	0,130481427	0,062	0	1	1,094658927	0,913526556	#DIV/0!
NDUFAF3	0,00530393	0,130246947	0,75	0,332	1	1,094481028	0,913675043	2,259036145
QSOX1	8,47889E-08	0,130233354	0,5	0,102	0,00178913	1,094470716	0,913683651	4,901960784
METTL6	1,96272E-05	0,130200599	0,188	0,023	0,414152777	1,094445868	0,913704395	8,173913043
RPS6KB2	5,1496E-05	0,130040962	0,25	0,047	1	1,094324772	0,913805504	5,319148936
AARD	9,12354E-07	0,13001606	0,094	0	0,019251588	1,094305883	0,913821278	#DIV/0!
PLXNA4	7,15271E-05	0,129994808	0,188	0,027	1	1,094289763	0,913834739	6,962962963
TBRG1	0,000345222	0,129803236	0,406	0,125	1	1,094144465	0,913956093	3,248
NOL8	0,000213404	0,129787729	0,219	0,043	1	1,094132704	0,913965917	5,093023256
RPIA	9,18193E-05	0,129611904	0,156	0,02	1	1,093999367	0,914077311	7,8
VPS26B	8,75655E-06	0,129593143	0,25	0,039	0,184771949	1,093985141	0,914089198	6,41025641
EEFSEC	7,15271E-05	0,129493161	0,188	0,027	1	1,093909328	0,914152549	6,962962963
WDR83	0,058054176	0,129295097	0,094	0,027	1	1,093759158	0,914278059	3,481481481
SCAP	1,63815E-05	0,129250449	0,312	0,062	0,345665807	1,093725309	0,914306354	5,032258065
PSME3IP1	1,16035E-05	0,129168561	0,281	0,051	0,244846285	1,093663231	0,914358252	5,509803922

Supplementary Table 1

Differentially expressed genes_adjacent-skin-XL_ comparing SC-Keloid vs. (SC-Myel, SC-Nonmyel)								
**gene**	**p_val**	**avg_log2FC**	**pct.1**	**pct.2**	**p_val_adj**	**Foldchange_UP**	**Foldchange_DOWN**	**Ratio_pct1_pct2**
FBXW2	0,008767839	0,129147617	0,219	0,07	1	1,093647354	0,914371526	3,128571429
PXMP4	5,12603E-06	0,129079988	0,188	0,02	0,108164307	1,093596089	0,91441439	9,4
AK1	0,000346284	0,129074288	0,531	0,18	1	1,093591768	0,914418003	2,95
PRADC1	0,000408406	0,128942999	0,188	0,035	1	1,093492253	0,914501221	5,371428571
C3orf18	0,140820961	0,128921767	0,062	0,02	1	1,09347616	0,91451468	3,1
CHPF2	0,003191522	0,128910355	0,188	0,047	1	1,09346751	0,914521914	4
CTSH	0,380266129	0,12884451	0,031	0,012	1	1,093417605	0,914563654	2,583333333
IREB2	0,002438935	0,128810376	0,219	0,059	1	1,093391736	0,914585292	3,711864407
SLC35B4	2,08038E-05	0,128720605	0,188	0,023	0,438980454	1,093323702	0,914642203	8,173913043
DSG2	9,12354E-07	0,128575452	0,094	0	0,019251588	1,093213706	0,914734233	#DIV/0!
PTBP1	0,000324159	0,128518392	0,531	0,184	1	1,093170468	0,914770412	2,885869565
LIN7B	0,003731537	0,128513712	0,156	0,035	1	1,093166922	0,91477338	4,457142857
TFAP4	0,000524345	0,128486026	0,094	0,008	1	1,093145945	0,914790934	11,75
STK19	6,58444E-07	0,128397875	0,281	0,039	0,013893836	1,093079154	0,914846831	7,205128205
SF3B2	0,000178536	0,128211977	0,625	0,215	1	1,092938314	0,914964721	2,906976744
TRMT61A	5,1496E-05	0,127906342	0,25	0,047	1	1,092706799	0,915158577	5,319148936
TSFM	9,17361E-07	0,127799931	0,188	0,016	0,019357231	1,092626206	0,91522608	11,75
VTI1B	1,70052E-05	0,127734466	0,375	0,086	0,358825913	1,092576628	0,915267611	4,360465116
TEX264	2,99389E-05	0,127709962	0,312	0,066	0,631740799	1,09255807	0,915283157	4,727272727
FNDC4	1,11477E-07	0,127671849	0,188	0,012	0,002352284	1,092529207	0,915307338	15,66666667
BCKDHB	0,011239937	0,127616598	0,156	0,043	1	1,092487368	0,915342392	3,627906977
APOBEC3C	2,4664E-06	0,127499288	0,406	0,086	0,052043428	1,092398538	0,915416824	4,720930233
H4C11	0,013054404	0,127420737	0,062	0,008	1	1,092339061	0,915466668	7,75
NUP188	0,024247489	0,127260017	0,25	0,098	1	1,092217378	0,915568659	2,551020408
ZNF317	0,000112833	0,127215493	0,125	0,012	1	1,092183671	0,915596915	10,41666667
HIVEP1	0,024371641	0,127183443	0,219	0,082	1	1,092159408	0,915617256	2,670731707
LAMA5	0,000907991	0,12700936	0,281	0,078	1	1,092027631	0,915727745	3,602564103
CENPO	0,079594089	0,126987377	0,031	0,004	1	1,092010991	0,915741699	7,75
SUZ12	0,001221806	0,126841495	0,375	0,121	1	1,091900575	0,915834301	3,099173554
ANKRD35	6,3574E-05	0,126784193	0,062	0	1	1,091857207	0,915870677	#DIV/0!

Supplementary Table 1

Differentially expressed genes_adjacent-skin-XL_ comparing SC-Keloid vs. (SC-Myel, SC-Nonmyel)								
**gene**	**p_val**	**avg_log2FC**	**pct.1**	**pct.2**	**p_val_adj**	**Foldchange_UP**	**Foldchange_DOWN**	**Ratio_pct1_pct2**
MIER1	4,01482E-06	0,126736689	0,469	0,113	0,084716761	1,091821255	0,915900835	4,150442478
KANK2	0,00502496	0,126725743	0,438	0,18	1	1,091812971	0,915907785	2,433333333
NR2C2AP	0,000100263	0,126629597	0,156	0,02	1	1,091740212	0,915968826	7,8
HSD11B1L	0,000486824	0,1262323	0,094	0,008	1	1,091439605	0,916221104	11,75
ATP5IF1	0,010876165	0,126193117	0,812	0,387	1	1,091409962	0,916245989	2,098191214
MRPL24	1,82968E-05	0,126070868	0,344	0,074	0,38608009	1,091317483	0,916323632	4,648648649
RBM18	2,02073E-05	0,126018966	0,188	0,023	0,426394579	1,091278223	0,916356598	8,173913043
DPH2	3,71921E-06	0,126008886	0,156	0,012	0,078479117	1,091270599	0,916363	13
TRAF4	2,45533E-09	0,125916693	0,312	0,031	5,18099E-05	1,091200865	0,916421561	10,06451613
ATAD2	1,51098E-05	0,125897311	0,125	0,008	0,318831333	1,091186205	0,916433873	15,625
PIGH	3,1256E-08	0,125879635	0,344	0,047	0,000659534	1,091172836	0,916445101	7,319148936
FLYWCH2	3,45071E-05	0,125861291	0,469	0,129	0,728133774	1,091158962	0,916456754	3,635658915
ZNF629	0,000108998	0,125846925	0,125	0,012	1	1,091148096	0,91646588	10,41666667
TSHZ1	1,45331E-05	0,12571442	0,219	0,031	0,306663142	1,091047884	0,916550057	7,064516129
GPATCH4	0,003453714	0,125638886	0,156	0,035	1	1,090990762	0,916598045	4,457142857
CYB561D2	5,12603E-06	0,125341553	0,188	0,02	0,108164307	1,090765937	0,916786972	9,4
ZNF746	0,002594281	0,125259603	0,094	0,012	1	1,09070398	0,91683905	7,833333333
RHNO1	7,96166E-09	0,125201207	0,188	0,008	0,000167999	1,090659832	0,916876161	23,5
ENG	2,96463E-05	0,125171742	0,469	0,129	0,625566052	1,090637557	0,916894888	3,635658915
MTDH	0,004851532	0,125151664	0,781	0,359	1	1,090622379	0,916907648	2,175487465
PAFAH2	0,00050526	0,125073339	0,094	0,008	1	1,09056317	0,916957429	11,75
GCDH	0,000426857	0,125042157	0,188	0,035	1	1,090539599	0,916977248	5,371428571
DYNC2LI1	0,001642148	0,124968476	0,125	0,02	1	1,090483904	0,917024081	6,25
ABI3BP	0,00018472	0,124921194	0,312	0,078	1	1,090448166	0,917054135	4
GDAP1	0,016270711	0,12485248	0,125	0,031	1	1,090396231	0,917097814	4,032258065
EXOSC4	1,96272E-05	0,124816343	0,188	0,023	0,414152777	1,090368919	0,917120786	8,173913043
CHRAC1	1,70052E-05	0,124513843	0,375	0,086	0,358825913	1,090140317	0,917313106	4,360465116
C19orf25	2,1264E-05	0,12447344	0,25	0,043	0,448690756	1,090109788	0,917338796	5,813953488
UXT	0,000237591	0,124429328	0,5	0,164	1	1,090076457	0,917366844	3,048780488
RPTN	0,004841742	0,124232911	0,031	0	1	1,089928058	0,917491749	#DIV/0!

Supplementary Table 1

Differentially expressed genes_adjacent-skin-XL_ comparing SC-Keloid vs. (SC-Myel, SC-Nonmyel)								
**gene**	**p_val**	**avg_log2FC**	**pct.1**	**pct.2**	**p_val_adj**	**Foldchange_UP**	**Foldchange_DOWN**	**Ratio_pct1_pct2**
EPB41	0,000313864	0,12416382	0,156	0,023	1	1,089875862	0,917535689	6,782608696
PITPNM3	9,12354E-07	0,124137136	0,094	0	0,019251588	1,089855704	0,91755266	#DIV/0!
SYF2	0,001137823	0,124127047	0,594	0,23	1	1,089848082	0,917559077	2,582608696
ORMDL2	1,5064E-07	0,124122495	0,312	0,043	0,003178646	1,089844644	0,917561972	7,255813953
FAM218A	0,002168448	0,124111152	0,062	0,004	1	1,089836075	0,917569186	15,5
ZFP14	0,287158009	0,123901153	0,062	0,027	1	1,08967745	0,917702757	2,296296296
ALG9	9,17361E-07	0,123746544	0,188	0,016	0,019357231	1,089560679	0,91780111	11,75
ZNF691	0,002442928	0,123737937	0,094	0,012	1	1,089554178	0,917806585	7,833333333
XRCC4	4,80509E-06	0,123735887	0,188	0,02	0,101392141	1,08955263	0,917807889	9,4
UNC119B	0,000217964	0,123490137	0,219	0,043	1	1,089367051	0,917964243	5,093023256
USP18	0,886800848	0,123399	0,031	0,027	1	1,089298237	0,918022233	1,148148148
IFT74	6,4372E-05	0,123341358	0,188	0,027	1	1,089254715	0,918058914	6,962962963
FAM78B	9,06462E-07	0,123288424	0,125	0,004	0,019127258	1,08921475	0,918092599	31,25
COG2	0,034851427	0,12322727	0,094	0,023	1	1,08916858	0,918131516	4,086956522
TUBB2A	0,003624359	0,123173616	0,656	0,297	1	1,089128075	0,918165662	2,208754209
NDN	0,000801432	0,12306421	0,312	0,09	1	1,089045484	0,918235294	3,466666667
ZFPM1	0,002686655	0,122777762	0,219	0,059	1	1,088829275	0,918417628	3,711864407
SERP2	0,002517564	0,122757996	0,094	0,012	1	1,088814358	0,918430211	7,833333333
LBX2-AS1	3,86379E-06	0,122661215	0,156	0,012	0,081529835	1,088741319	0,918491824	13
EMC9	0,008322455	0,122554235	0,188	0,055	1	1,088660588	0,918559936	3,418181818
SGSM3	8,00274E-06	0,122470828	0,312	0,059	0,168865745	1,088597651	0,918613043	5,288135593
SUB1	0,010998166	0,122435898	0,844	0,441	1	1,088571295	0,918635283	1,9138322
COG4	1,5064E-07	0,122371818	0,312	0,043	0,003178646	1,088522944	0,918676088	7,255813953
ACADS	5,49744E-05	0,122326376	0,25	0,047	1	1,088488659	0,918705025	5,319148936
FGFBP3	0,00696629	0,122325243	0,156	0,039	1	1,088487804	0,918705746	4
MTOR	2,40115E-05	0,122234662	0,156	0,016	0,506667402	1,088419465	0,918763429	9,75
B3GALNT2	8,19238E-06	0,121954831	0,344	0,07	0,172867368	1,088208371	0,918941654	4,914285714
CRLS1	9,85458E-05	0,121598884	0,219	0,039	1	1,087939917	0,919168407	5,615384615
ABCA7	6,3574E-05	0,121587789	0,062	0	1	1,08793155	0,919175476	#DIV/0!
AL031590.1	0,004841742	0,121387998	0,031	0	1	1,087780899	0,919302776	#DIV/0!

Supplementary Table 1

Differentially expressed genes_adjacent-skin-XL_ comparing SC-Keloid vs. (SC-Myel, SC-Nonmyel)								
**gene**	**p_val**	**avg_log2FC**	**pct.1**	**pct.2**	**p_val_adj**	**Foldchange_UP**	**Foldchange_DOWN**	**Ratio_pct1_pct2**
MOSPD1	0,000186392	0,121250078	0,188	0,031	1	1,087676913	0,919390665	6,064516129
COQ9	4,14927E-06	0,121207397	0,219	0,027	0,087553714	1,087644735	0,919417865	8,111111111
SMARCD2	0,05712057	0,121122073	0,094	0,027	1	1,087580412	0,919472242	3,481481481
PRICKLE2	4,24942E-06	0,121040398	0,344	0,066	0,089667107	1,087518843	0,919524297	5,212121212
TMEM14B	0,000685336	0,120906796	0,5	0,172	1	1,087418137	0,919609455	2,906976744
HENMT1	0,082317662	0,120872276	0,031	0,004	1	1,087392118	0,919631459	7,75
GFOD2	2,03907E-07	0,12086599	0,219	0,02	0,004302644	1,08738738	0,919635466	10,95
CHST7	0,136324108	0,120764855	0,062	0,02	1	1,087311155	0,919699936	3,1
MMGT1	0,005043469	0,120695001	0,219	0,062	1	1,08725851	0,919744468	3,532258065
MRPL21	5,08352E-06	0,120651209	0,406	0,09	0,107267254	1,087225508	0,919772386	4,511111111
COL16A1	0,002128196	0,120636073	0,75	0,336	1	1,087214101	0,919782037	2,232142857
OXLD1	0,007558313	0,120547342	0,094	0,016	1	1,087147235	0,919838608	5,875
FAM214B	1,12327E-08	0,120484652	0,312	0,035	0,000237021	1,087099996	0,919878579	8,914285714
DERA	6,8795E-06	0,120373101	0,312	0,059	0,14516429	1,087015944	0,919949708	5,288135593
REP15	6,3574E-05	0,120335535	0,062	0	1	1,086987639	0,919973663	#DIV/0!
SMNDC1	0,002497467	0,120215261	0,375	0,133	1	1,086897024	0,920050362	2,819548872
GPALPP1	0,123276773	0,120199514	0,094	0,035	1	1,08688516	0,920060404	2,685714286
AVEN	2,24684E-05	0,120127094	0,156	0,016	0,474104712	1,086830602	0,920106591	9,75
ATAT1	0,000862268	0,11995858	0,188	0,039	1	1,086703663	0,92021407	4,820512821
ALDH3B1	2,11157E-07	0,119896744	0,219	0,02	0,00445563	1,086657086	0,920253512	10,95
CDT1	9,12354E-07	0,119767952	0,094	0	0,019251588	1,086560083	0,920335669	#DIV/0!
EXOC7	0,001096967	0,119720487	0,312	0,094	1	1,086524335	0,920365948	3,319148936
FMNL1	0,216675772	0,119342815	0,031	0,008	1	1,08623994	0,920606915	3,875
GOLGA7B	1,33967E-08	0,119221628	0,125	0	0,000282684	1,086148698	0,92068425	#DIV/0!
FNDC10	9,12354E-07	0,119211923	0,094	0	0,019251588	1,086141392	0,920690443	#DIV/0!
MAPKAPK3	9,17361E-07	0,119195682	0,188	0,016	0,019357231	1,086129165	0,920700808	11,75
SGO2	8,53227E-07	0,119000006	0,188	0,016	0,018003947	1,085981861	0,920825693	11,75
ATXN7L3	0,003907631	0,118987971	0,125	0,023	1	1,085972801	0,920833375	5,434782609
ARV1	0,001520602	0,118982512	0,125	0,02	1	1,085968692	0,92083686	6,25
DAP3	0,00019011	0,118869071	0,281	0,066	1	1,085883305	0,920909269	4,257575758

Supplementary Table 1

Differentially expressed genes_adjacent-skin-XL_ comparing SC-Keloid vs. (SC-Myel, SC-Nonmyel)								
**gene**	**p_val**	**avg_log2FC**	**pct.1**	**pct.2**	**p_val_adj**	**Foldchange_UP**	**Foldchange_DOWN**	**Ratio_pct1_pct2**
TFB1M	0,000524345	0,11884519	0,094	0,008	1	1,08586533	0,920924513	11,75
BCKDK	0,000235567	0,118808075	0,281	0,066	1	1,085837396	0,920948205	4,257575758
CKAP2L	6,3574E-05	0,118773451	0,062	0	1	1,085811336	0,920970307	#DIV/0!
CDCA5	6,3574E-05	0,118750856	0,062	0	1	1,085794331	0,920984731	#DIV/0!
POLR1E	0,039090354	0,118702899	0,125	0,039	1	1,085758238	0,921015347	3,205128205
GOPC	3,20633E-05	0,118616773	0,406	0,102	0,676566707	1,085693422	0,921070331	3,980392157
SRPK1	0,006263449	0,118543463	0,219	0,066	1	1,085638255	0,921117136	3,318181818
CTU2	4,02312E-05	0,118472998	0,219	0,035	0,848917917	1,08558523	0,921162127	6,257142857
ADAM15	9,0753E-05	0,118452452	0,344	0,086	1	1,085569771	0,921175246	4
NT5DC1	3,67695E-07	0,118144395	0,156	0,008	0,007758733	1,085337995	0,921371964	19,5
C7orf50	0,000107103	0,118078564	0,656	0,227	1	1,085288471	0,921414008	2,889867841
NXPH3	9,06462E-07	0,118071213	0,125	0,004	0,019127258	1,085282941	0,921418703	31,25
MRPL33	0,01098539	0,118044065	0,781	0,387	1	1,085262519	0,921436042	2,018087855
GDI1	0,000552365	0,118034426	0,688	0,266	1	1,085255268	0,921442198	2,586466165
SYCE1L	4,80439E-05	0,117996409	0,094	0,004	1	1,085226671	0,92146648	23,5
MANEA	4,15544E-10	0,117963814	0,344	0,035	8,7684E-06	1,085202152	0,921487299	9,828571429
TMEM233	0,004841742	0,117956024	0,031	0	1	1,085196292	0,921492275	#DIV/0!
TMEM51	3,51691E-07	0,11794196	0,156	0,008	0,007421029	1,085185714	0,921501257	19,5
RHBDD2	1,28546E-05	0,117774086	0,469	0,117	0,271244228	1,085059447	0,921608491	4,008547009
RRM2	0,004841742	0,117755654	0,031	0	1	1,085045584	0,921620266	#DIV/0!
RRP1B	0,004267025	0,117598072	0,156	0,035	1	1,084927074	0,921720938	4,457142857
FCGRT	0,030702441	0,117595747	0,594	0,297	1	1,084925325	0,921722423	2
CRELD2	2,35573E-05	0,117545393	0,469	0,129	0,497083296	1,084887459	0,921754594	3,635658915
ARL10	0,000417537	0,117513731	0,188	0,035	1	1,08486365	0,921774824	5,371428571
USP40	3,64935E-07	0,117481367	0,312	0,047	0,007700495	1,084839314	0,921795502	6,638297872
ARL6	9,06462E-07	0,117372197	0,125	0,004	0,019127258	1,084757227	0,921865257	31,25
ACD	0,000208933	0,117242451	0,219	0,043	1	1,084659675	0,921948168	5,093023256
BTN3A3	1,33967E-08	0,117185974	0,125	0	0,000282684	1,084617215	0,92198426	#DIV/0!
CPSF3	6,78604E-05	0,11717318	0,188	0,027	1	1,084607596	0,921992436	6,962962963
APLF	6,3574E-05	0,11709406	0,062	0	1	1,084548116	0,922043001	#DIV/0!

Supplementary Table 1

Differentially expressed genes_adjacent-skin-XL_ comparing SC-Keloid vs. (SC-Myel, SC-Nonmyel)								
**gene**	**p_val**	**avg_log2FC**	**pct.1**	**pct.2**	**p_val_adj**	**Foldchange_UP**	**Foldchange_DOWN**	**Ratio_pct1_pct2**
TES	3,92553E-05	0,117072978	0,219	0,035	0,828326361	1,084532268	0,922056475	6,257142857
TAF1A	6,3574E-05	0,117006176	0,062	0	1	1,084482051	0,922099171	#DIV/0!
C14orf93	0,000305772	0,11680624	0,156	0,023	1	1,084331768	0,922226969	6,782608696
MTG2	5,30026E-06	0,116798737	0,281	0,047	0,111840702	1,084326129	0,922231765	5,978723404
MEIS3	0,003734954	0,116669128	0,125	0,023	1	1,08422872	0,92231462	5,434782609
MECR	0,000100263	0,116664123	0,156	0,02	1	1,084224959	0,92231782	7,8
PDGFB	4,32037E-12	0,116628276	0,5	0,062	9,11641E-08	1,084198019	0,922340737	8,064516129
BCAT1	1,33967E-08	0,116604134	0,125	0	0,000282684	1,084179876	0,922356172	#DIV/0!
SPCS3	0,003180406	0,116575868	0,438	0,168	1	1,084158635	0,922374243	2,607142857
TMBIM1	0,055551272	0,116498697	0,375	0,191	1	1,084100644	0,922423583	1,963350785
CFAP298	3,52995E-09	0,11631048	0,625	0,129	7,44854E-05	1,083959219	0,922543932	4,84496124
PLAA	0,003660239	0,116284412	0,156	0,035	1	1,083939633	0,922560602	4,457142857
MNS1	3,73706E-05	0,116243233	0,219	0,035	0,788556861	1,083908695	0,922586935	6,257142857
ERC1	0,07109236	0,116162993	0,25	0,117	1	1,083848411	0,922638249	2,136752137
DNAJC17	9,91727E-06	0,116140099	0,25	0,039	0,209264362	1,083831212	0,92265289	6,41025641
CASD1	4,43697E-05	0,116098957	0,219	0,035	0,936244219	1,083800304	0,922679202	6,257142857
MTHFD1	0,000419572	0,116053371	0,25	0,059	1	1,083766059	0,922708357	4,237288136
SLC1A4	0,025666014	0,115883106	0,125	0,035	1	1,083638162	0,92281726	3,571428571
CHST12	5,07391E-05	0,11581868	0,344	0,082	1	1,083589771	0,922858472	4,195121951
COG8	0,143111738	0,115782068	0,062	0,02	1	1,083562273	0,922881891	3,1
ZNF777	0,985101745	0,115732726	0,031	0,031	1	1,083525214	0,922913456	1
IL15	0,038520178	0,115664469	0,125	0,039	1	1,083473951	0,922957122	3,205128205
HSPB11	2,56424E-06	0,115653286	0,406	0,086	0,054108077	1,083465553	0,922964276	4,720930233
NDUFAF5	8,53227E-07	0,115519218	0,188	0,016	0,018003947	1,083364873	0,92305005	11,75
TAMM41	0,217761179	0,115514607	0,094	0,043	1	1,08336141	0,923053001	2,186046512
IPO11	0,007558313	0,11550652	0,094	0,016	1	1,083355337	0,923058175	5,875
PHKA2	0,007558313	0,115428037	0,094	0,016	1	1,083296404	0,923108391	5,875
OGFOD1	6,94943E-07	0,115374311	0,281	0,039	0,014663999	1,083256063	0,923142768	7,205128205
MASTL	0,003907631	0,115248166	0,125	0,023	1	1,08316135	0,923223489	5,434782609
CYHR1	8,94972E-05	0,115105504	0,5	0,152	1	1,083054246	0,923314786	3,289473684

Supplementary Table 1

Differentially expressed genes_adjacent-skin-XL_ comparing SC-Keloid vs. (SC-Myel, SC-Nonmyel)								
**gene**	**p_val**	**avg_log2FC**	**pct.1**	**pct.2**	**p_val_adj**	**Foldchange_UP**	**Foldchange_DOWN**	**Ratio_pct1_pct2**
MRPS18C	0,00024412	0,1150116	0,406	0,121	1	1,082983753	0,923374886	3,355371901
PIGV	0,000112833	0,114994111	0,125	0,012	1	1,082970625	0,92338608	10,41666667
PINK1	0,024095672	0,114889756	0,219	0,082	1	1,082892292	0,923452874	2,670731707
CCDC25	0,000751392	0,114680291	0,25	0,062	1	1,082735079	0,92358696	4,032258065
KEAP1	5,49744E-05	0,114640011	0,25	0,047	1	1,082704849	0,923612747	5,319148936
CSF3	0,079594089	0,114429281	0,031	0,004	1	1,082546714	0,923747666	7,75
SLC9A5	0,004841742	0,114392539	0,031	0	1	1,082519144	0,923771192	#DIV/0!
GP1BB	6,3574E-05	0,114377582	0,062	0	1	1,082507922	0,923780769	#DIV/0!
OTUD3	0,001600673	0,11427028	0,125	0,02	1	1,082427411	0,923849479	6,25
CAVIN1	0,00598963	0,114221907	0,812	0,41	1	1,082391119	0,923880456	1,980487805
TIMM50	3,83019E-05	0,114089518	0,219	0,035	0,808209445	1,082291798	0,92396524	6,257142857
STRN3	0,001160855	0,114042607	0,312	0,094	1	1,082256607	0,923995284	3,319148936
LOXL1-AS1	9,06462E-07	0,114033921	0,125	0,004	0,019127258	1,082250091	0,924000847	31,25
SNX6	0,000164547	0,113999626	0,625	0,215	1	1,082224364	0,924022812	2,906976744
SAMD12	0,00050526	0,113914146	0,094	0,008	1	1,082160244	0,924077562	11,75
SLC48A1	0,163752339	0,113616323	0,094	0,039	1	1,081936871	0,924268344	2,41025641
UNG	6,10538E-05	0,113606115	0,188	0,027	1	1,081929215	0,924274884	6,962962963
BMF	0,64910682	0,113374158	0,031	0,02	1	1,081755277	0,924423501	1,55
ZBTB8OS	1,39237E-10	0,113247484	0,5	0,074	2,93803E-06	1,081660299	0,924504672	6,756756757
KIF5B	0,006594757	0,113137968	0,781	0,379	1	1,081578192	0,924574855	2,060686016
SLC25A37	0,00192833	0,113118706	0,406	0,148	1	1,081563752	0,924587199	2,743243243
SLC35A3	0,001280476	0,112962734	0,25	0,066	1	1,081446829	0,924687163	3,787878788
ATP6V1E1	0,000461786	0,112931017	0,406	0,129	1	1,081423053	0,924707493	3,147286822
PPA2	1,87436E-06	0,112857651	0,281	0,043	0,039550805	1,081368061	0,924754518	6,534883721
RMI1	1,96814E-10	0,11277735	0,156	0	4,15296E-06	1,081307874	0,924805991	#DIV/0!
ETV1	1,51098E-05	0,112689986	0,125	0,008	0,318831333	1,081242395	0,924861996	15,625
UCN2	1,33967E-08	0,112677366	0,125	0	0,000282684	1,081232937	0,924870086	#DIV/0!
AC003102.1	0,007750521	0,112622339	0,094	0,016	1	1,081191698	0,924905363	5,875
FUBP3	0,065708526	0,112552564	0,219	0,098	1	1,081139408	0,924950097	2,234693878
C8orf33	4,97256E-06	0,112536954	0,375	0,078	0,104925968	1,08112771	0,924960105	4,807692308

Supplementary Table 1

Differentially expressed genes_adjacent-skin-XL_ comparing SC-Keloid vs. (SC-Myel, SC-Nonmyel)								
**gene**	**p_val**	**avg_log2FC**	**pct.1**	**pct.2**	**p_val_adj**	**Foldchange_UP**	**Foldchange_DOWN**	**Ratio_pct1_pct2**
RBFOX2	1,1972E-07	0,112525006	0,469	0,094	0,002526204	1,081118756	0,924967765	4,989361702
ACYP1	5,30026E-06	0,112523628	0,281	0,047	0,111840702	1,081117724	0,924968649	5,978723404
CEP41	0,000105286	0,112505023	0,125	0,012	1	1,081103782	0,924980577	10,41666667
LGMN	2,72358E-05	0,112480894	0,344	0,078	0,574702915	1,081085701	0,924996048	4,41025641
ZNF454	0,079594089	0,112471924	0,031	0,004	1	1,081078979	0,925001799	7,75
DUSP4	0,002370322	0,112387178	0,094	0,012	1	1,081015477	0,925056136	7,833333333
ATG4D	3,71921E-06	0,112306504	0,156	0,012	0,078479117	1,080955029	0,925107866	13
SPTLC2	5,03836E-05	0,112282537	0,25	0,047	1	1,080937072	0,925123234	5,319148936
P4HA1	0,000138718	0,112133278	0,281	0,062	1	1,080825245	0,925218951	4,532258065
MED14OS	0,039886012	0,112094855	0,062	0,012	1	1,08079646	0,925243593	5,166666667
P3H4	9,17361E-07	0,112012054	0,188	0,016	0,019357231	1,080734431	0,925296697	11,75
CANT1	9,17361E-07	0,111909746	0,188	0,016	0,019357231	1,080657795	0,925362316	11,75
NXN	9,06462E-07	0,111866676	0,125	0,004	0,019127258	1,080625533	0,925389942	31,25
NAA60	0,001827205	0,111854396	0,188	0,043	1	1,080616335	0,925397819	4,372093023
AATF	0,000420635	0,111462232	0,344	0,098	1	1,080322634	0,925649402	3,510204082
PKN3	0,039886012	0,111386878	0,062	0,012	1	1,080266209	0,925697751	5,166666667
SUSD6	0,009253741	0,110945632	0,219	0,07	1	1,079935862	0,925980917	3,128571429
ZDHHC3	3,04133E-08	0,110923428	0,344	0,047	0,00064175	1,079919241	0,925995169	7,319148936
VSTM2B	0,226351275	0,110779664	0,031	0,008	1	1,079811633	0,926087449	3,875
TERF2	0,000509339	0,110747641	0,125	0,016	1	1,079787664	0,926108005	7,8125
DXO	0,003361753	0,110745596	0,188	0,047	1	1,079786134	0,926109317	4
ZNF18	0,004841742	0,110701721	0,031	0	1	1,079753296	0,926137483	#DIV/0!
AC004637.1	1,33967E-08	0,11066474	0,125	0	0,000282684	1,079725619	0,926161223	#DIV/0!
POLD2	0,001464368	0,110581213	0,344	0,109	1	1,079663109	0,926214846	3,155963303
DNLZ	1,44979E-05	0,11046168	0,125	0,008	0,305919948	1,079573658	0,92629159	15,625
ACTR1B	4,50124E-07	0,110447212	0,375	0,066	0,009498056	1,079562831	0,926300879	5,681818182
L3HYPDH	0,000181965	0,110390423	0,188	0,031	1	1,079520338	0,926337342	6,064516129
DHX30	0,000359124	0,11037519	0,281	0,07	1	1,079508939	0,926347123	4,014285714
XPO7	0,01796492	0,110369319	0,219	0,078	1	1,079504546	0,926350893	2,807692308
TFAP2A	0,005023211	0,110358083	0,562	0,238	1	1,079496139	0,926358107	2,361344538

Supplementary Table 1

Differentially expressed genes_adjacent-skin-XL_ comparing SC-Keloid vs. (SC-Myel, SC-Nonmyel)								
**gene**	**p_val**	**avg_log2FC**	**pct.1**	**pct.2**	**p_val_adj**	**Foldchange_UP**	**Foldchange_DOWN**	**Ratio_pct1_pct2**
TP53I3	4,62254E-08	0,110352595	0,406	0,066	0,000975403	1,079492033	0,926361631	6,151515152
ASPM	0,002263143	0,110319964	0,062	0,004	1	1,079467617	0,926382584	15,5
C6orf226	0,009528382	0,110309348	0,25	0,086	1	1,079459673	0,926389401	2,906976744
PHTF2	0,006844876	0,110232841	0,156	0,039	1	1,07940243	0,926438529	4
VAMP4	0,011609182	0,110213297	0,156	0,043	1	1,079387808	0,926451079	3,627906977
ANKRD34A	6,3574E-05	0,110081515	0,062	0	1	1,079289217	0,926535709	#DIV/0!
SFI1	0,002442928	0,110051396	0,094	0,012	1	1,079266685	0,926555053	7,833333333
PDE4B	1,11477E-07	0,11001538	0,188	0,012	0,002352284	1,079239742	0,926578184	15,66666667
ZNF674	0,002263143	0,109970912	0,062	0,004	1	1,079206477	0,926606744	15,5
FLNB	9,41286E-05	0,109949706	0,375	0,098	1	1,079190614	0,926620365	3,826530612
ARHGEF35	6,3574E-05	0,109890898	0,062	0	1	1,079146624	0,926658137	#DIV/0!
ZFP1	0,034213216	0,109884044	0,094	0,023	1	1,079141497	0,926662539	4,086956522
FASN	5,70421E-07	0,109882458	0,406	0,078	0,012036463	1,079140311	0,926663558	5,205128205
TMEM242	0,004026393	0,109836851	0,219	0,062	1	1,079106197	0,926692852	3,532258065
BTN3A1	9,06462E-07	0,109744122	0,125	0,004	0,019127258	1,079036841	0,926752417	31,25
UBTD2	0,000397528	0,109671393	0,281	0,07	1	1,078982446	0,926799138	4,014285714
PSMG3-AS1	0,040857201	0,109575466	0,062	0,012	1	1,078910705	0,926860764	5,166666667
ANP32E	0,009340743	0,109472945	0,312	0,121	1	1,078834038	0,926926631	2,578512397
FBL	7,10604E-05	0,109440585	0,5	0,148	1	1,078809839	0,926947423	3,378378378
MBNL1-AS1	0,173428922	0,109439811	0,094	0,039	1	1,078809261	0,92694792	2,41025641
GPRASP2	6,3574E-05	0,109434084	0,062	0	1	1,078804978	0,9269516	#DIV/0!
MYO1F	0,079594089	0,109347569	0,031	0,004	1	1,078740287	0,927007188	7,75
VASP	0,000278367	0,109303506	0,312	0,082	1	1,07870734	0,927035502	3,804878049
BAALC	6,3574E-05	0,109282055	0,062	0	1	1,078691301	0,927049286	#DIV/0!
PIN4	0,002116036	0,109256026	0,469	0,176	1	1,07867184	0,927066011	2,664772727
HNRNPA1	0,132238451	0,109194273	0,938	0,652	1	1,07862567	0,927105694	1,438650307
PYGO2	0,015149007	0,109177633	0,125	0,031	1	1,078613228	0,927116388	4,032258065
HIRIP3	0,017650698	0,10916843	0,156	0,047	1	1,078606348	0,927122301	3,319148936
MEPE	0,004841742	0,108944248	0,031	0	1	1,078438755	0,92726638	#DIV/0!
CHCHD6	9,73699E-05	0,108890337	0,156	0,02	1	1,078398456	0,927301031	7,8

Supplementary Table 1

Differentially expressed genes_adjacent-skin-XL_ comparing SC-Keloid vs. (SC-Myel, SC-Nonmyel)								
**gene**	**p_val**	**avg_log2FC**	**pct.1**	**pct.2**	**p_val_adj**	**Foldchange_UP**	**Foldchange_DOWN**	**Ratio_pct1_pct2**
BORCS6	0,000322156	0,108679205	0,156	0,023	1	1,078240649	0,927436747	6,782608696
NCKAP1	0,003444709	0,108621101	0,5	0,207	1	1,078197225	0,927474099	2,415458937
PI4KA	0,000497591	0,108520752	0,219	0,047	1	1,078122232	0,927538614	4,659574468
RTKN2	9,06462E-07	0,108418583	0,125	0,004	0,019127258	1,078045883	0,927604303	31,25
GLYCTK	9,06462E-07	0,108367747	0,125	0,004	0,019127258	1,078007897	0,927636989	31,25
UBAC1	0,002864354	0,10832414	0,219	0,059	1	1,077975314	0,927665028	3,711864407
PNPLA6	0,003804096	0,108291151	0,156	0,035	1	1,077950664	0,927686241	4,457142857
CACUL1	0,000216206	0,108275271	0,344	0,094	1	1,0779388	0,927696452	3,659574468
GOLPH3L	0,000100263	0,108230886	0,156	0,02	1	1,077905637	0,927724993	7,8
NMRAL1	1,41893E-05	0,108206587	0,281	0,051	0,29940858	1,077887482	0,927740619	5,509803922
LINC00630	0,221475664	0,10805473	0,031	0,008	1	1,077774031	0,927838277	3,875
ETV2	9,12354E-07	0,107970764	0,094	0	0,019251588	1,077711306	0,92789228	#DIV/0!
EEF1AKMT1	5,12603E-06	0,107967066	0,188	0,02	0,108164307	1,077708542	0,927894659	9,4
MSRB3	7,33388E-07	0,107710074	0,281	0,039	0,015475215	1,077516584	0,928059962	7,205128205
BCL2L11	0,000954402	0,10728368	0,188	0,039	1	1,077198167	0,928334294	4,820512821
MED1	9,91727E-06	0,107141059	0,25	0,039	0,209264362	1,077091684	0,928426071	6,41025641
MTIF2	0,000100263	0,10707484	0,156	0,02	1	1,077042246	0,928468687	7,8
ECD	0,002976767	0,107018676	0,188	0,047	1	1,077000318	0,928504833	4
SCLY	9,73699E-05	0,106941755	0,156	0,02	1	1,076942897	0,92855434	7,8
COMMD9	7,25857E-06	0,106775533	0,344	0,07	0,153163084	1,076818823	0,92866133	4,914285714
AK2	0,000812022	0,10676179	0,438	0,148	1	1,076808565	0,928670176	2,959459459
G3BP2	0,001103208	0,106634831	0,375	0,121	1	1,076713809	0,928751904	3,099173554
GPC2	0,002263143	0,106543934	0,062	0,004	1	1,076645972	0,928810422	15,5
PFKP	0,014273854	0,106500737	0,25	0,09	1	1,076613736	0,928838233	2,777777778
EPG5	0,084624339	0,106288606	0,062	0,016	1	1,076455445	0,928974817	3,875
NUP93	4,14927E-06	0,106260555	0,219	0,027	0,087553714	1,076434514	0,92899288	8,111111111
CEP250	0,000339359	0,106199474	0,156	0,023	1	1,076388941	0,929032213	6,782608696
KIAA1614	0,018047186	0,106131484	0,094	0,02	1	1,076338216	0,929075996	4,7
PEX2	0,000215381	0,10612998	0,375	0,105	1	1,076337094	0,929076965	3,571428571
FUCA1	0,000103236	0,106104936	0,156	0,02	1	1,076318409	0,929093093	7,8

Supplementary Table 1

Differentially expressed genes_adjacent-skin-XL_ comparing SC-Keloid vs. (SC-Myel, SC-Nonmyel)								
**gene**	**p_val**	**avg_log2FC**	**pct.1**	**pct.2**	**p_val_adj**	**Foldchange_UP**	**Foldchange_DOWN**	**Ratio_pct1_pct2**
ZNF667-AS1	0,000187864	0,105996536	0,312	0,078	1	1,076237541	0,929162905	4
PPT2	2,16801E-07	0,105625908	0,344	0,055	0,004574713	1,075961091	0,929401637	6,254545455
FOXK2	2,93017E-05	0,105603579	0,281	0,055	0,618294606	1,075944438	0,929416022	5,109090909
ADPRS	2,44525E-05	0,105600241	0,25	0,043	0,515972154	1,075941949	0,929418173	5,813953488
IFT52	1,50482E-06	0,105439404	0,344	0,062	0,031753276	1,075822006	0,929521793	5,548387097
ALDH18A1	4,02312E-05	0,10532534	0,219	0,035	0,848917917	1,075736951	0,929595287	6,257142857
LPP	0,001531692	0,105229596	0,531	0,203	1	1,075665562	0,929656982	2,615763547
STON2	0,000480113	0,105177453	0,125	0,016	1	1,075626686	0,929690582	7,8125
RNPEPL1	0,000212852	0,105115243	0,344	0,094	1	1,075580305	0,929730673	3,659574468
SCP2	0,00826133	0,105016062	0,531	0,238	1	1,075506364	0,929794591	2,231092437
DPH6	0,000425232	0,104892933	0,219	0,047	1	1,075414578	0,929873949	4,659574468
HMG20A	0,213283768	0,10486847	0,094	0,043	1	1,075396343	0,929889716	2,186046512
PTK2	0,017923128	0,10486251	0,562	0,273	1	1,0753919	0,929893558	2,058608059
PMF1	3,31424E-05	0,104859059	0,406	0,102	0,699337059	1,075389327	0,929895782	3,980392157
SUCLA2	0,00013642	0,104747985	0,375	0,102	1	1,075306536	0,929967378	3,676470588
ZNF91	0,030940914	0,104646454	0,219	0,086	1	1,075230863	0,930032828	2,546511628
CHD4	2,99875E-05	0,104607496	0,656	0,211	0,632766515	1,075201828	0,930057943	3,109004739
ASH2L	0,00083045	0,104462878	0,156	0,027	1	1,075094053	0,930151178	5,777777778
DNPEP	6,58735E-06	0,104396568	0,312	0,059	0,138999658	1,07504464	0,930193931	5,288135593
PGAP3	0,138558481	0,104316015	0,062	0,02	1	1,074984617	0,93024587	3,1
TFDP1	2,82595E-05	0,104201703	0,344	0,078	0,596303572	1,074899444	0,930319581	4,41025641
RAC2	9,12354E-07	0,1041945	0,094	0	0,019251588	1,074894077	0,930324226	#DIV/0!
CD2AP	6,41077E-05	0,104170164	0,281	0,059	1	1,074875946	0,930339919	4,762711864
PABPC5	0,000480113	0,104081145	0,125	0,016	1	1,074809624	0,930397325	7,8125
FBXW9	2,97902E-08	0,104041033	0,219	0,016	0,000628603	1,074779741	0,930423194	13,6875
USP35	0,000524345	0,104003142	0,094	0,008	1	1,074751513	0,930447631	11,75
TTC23	0,173428922	0,103962474	0,094	0,039	1	1,074721218	0,93047386	2,41025641
GCC2	0,002247723	0,103894994	0,469	0,18	1	1,074670951	0,930517383	2,605555556
PHF5A	0,000121256	0,103888258	0,312	0,074	1	1,074665932	0,930521728	4,216216216
MRPL37	1,14716E-07	0,103879577	0,312	0,043	0,002420617	1,074659466	0,930527326	7,255813953

Supplementary Table 1

Differentially expressed genes_adjacent-skin-XL_ comparing SC-Keloid vs. (SC-Myel, SC-Nonmyel)								
**gene**	**p_val**	**avg_log2FC**	**pct.1**	**pct.2**	**p_val_adj**	**Foldchange_UP**	**Foldchange_DOWN**	**Ratio_pct1_pct2**
MRPS27	0,000466117	0,103878359	0,188	0,035	1	1,074658559	0,930528112	5,371428571
ALKBH6	0,007750521	0,10382845	0,094	0,016	1	1,074621383	0,930560304	5,875
SLC17A9	4,52605E-06	0,103800004	0,219	0,027	0,095504122	1,074600195	0,930578652	8,111111111
SLC25A14	0,000108998	0,103772448	0,125	0,012	1	1,074579669	0,930596426	10,41666667
ZNF662	0,002263143	0,103094638	0,062	0,004	1	1,074074927	0,931033744	15,5
EN1	0,039886012	0,103088692	0,062	0,012	1	1,0740705	0,931037581	5,166666667
MED12	0,057775912	0,103082322	0,125	0,043	1	1,074065757	0,931041692	2,906976744
INTS5	1,51098E-05	0,103022329	0,125	0,008	0,318831333	1,074021095	0,931080409	15,625
KRCC1	9,55574E-06	0,102949692	0,375	0,082	0,201635701	1,073967021	0,931127288	4,573170732
UGGT1	8,14044E-08	0,10293233	0,344	0,051	0,001717714	1,073954096	0,931138494	6,745098039
CIBAR1	0,000478496	0,102895576	0,219	0,047	1	1,073926736	0,931162216	4,659574468
MTG1	1,05359E-06	0,10286096	0,375	0,07	0,022231775	1,073900969	0,931184559	5,357142857
DNAJC5	0,00094841	0,102766722	0,219	0,051	1	1,073830824	0,931245386	4,294117647
ELMOD2	2,50263E-05	0,10275453	0,25	0,043	0,528079884	1,073821749	0,931253256	5,813953488
DHRS4	0,001625689	0,102704894	0,156	0,031	1	1,073784805	0,931285296	5,032258065
ERI1	0,000524345	0,102604335	0,094	0,008	1	1,073709962	0,931350211	11,75
GHDC	0,01179781	0,102298152	0,156	0,043	1	1,073482113	0,931547893	3,627906977
ASF1A	0,000105488	0,102188285	0,219	0,039	1	1,073400366	0,931618837	5,615384615
FMN1	4,80439E-05	0,102183927	0,094	0,004	1	1,073397123	0,931621651	23,5
CYB5RL	0,082317662	0,102131032	0,031	0,004	1	1,073357769	0,931655808	7,75
GOLGA3	6,15412E-05	0,102089666	0,375	0,094	1	1,073326993	0,931682522	3,989361702
ABCB8	0,000850121	0,102015539	0,156	0,027	1	1,073271846	0,931730394	5,777777778
VRK2	4,96307E-06	0,101945568	0,188	0,02	0,104725812	1,073219793	0,931775584	9,4
WIZ	0,000181963	0,101760499	0,188	0,031	1	1,07308213	0,93189512	6,064516129
GOSR2	0,000114181	0,101753126	0,25	0,051	1	1,073076646	0,931899883	4,901960784
RRP1	0,000466117	0,10172953	0,188	0,035	1	1,073059095	0,931915124	5,371428571
LRRC28	0,015983649	0,101605196	0,125	0,031	1	1,072966621	0,931995442	4,032258065
CENPU	1,51098E-05	0,101588928	0,125	0,008	0,318831333	1,072954522	0,932005951	15,625
ECI2	0,002463233	0,101499555	0,281	0,086	1	1,072888056	0,932063689	3,26744186
PPP1R26	0,082317662	0,101493869	0,031	0,004	1	1,072883828	0,932067363	7,75

Supplementary Table 1

Differentially expressed genes_adjacent-skin-XL_ comparing SC-Keloid vs. (SC-Myel, SC-Nonmyel)								
**gene**	**p_val**	**avg_log2FC**	**pct.1**	**pct.2**	**p_val_adj**	**Foldchange_UP**	**Foldchange_DOWN**	**Ratio_pct1_pct2**
BMPER	6,3574E-05	0,101441373	0,062	0	1	1,072844789	0,932101279	#DIV/0!
MACROD2	0,00050526	0,101368846	0,094	0,008	1	1,072790856	0,932148139	11,75
GLUD2	0,002263143	0,101345674	0,062	0,004	1	1,072773625	0,932163111	15,5
DPP3	1,51098E-05	0,101337881	0,125	0,008	0,318831333	1,072767831	0,932168146	15,625
HMGCL	0,000103124	0,101250386	0,219	0,039	1	1,072702773	0,932224681	5,615384615
DEPP1	0,000604947	0,101155389	0,281	0,074	1	1,072632141	0,932286067	3,797297297
SUPT20H	0,000500143	0,101152315	0,312	0,086	1	1,072629856	0,932288053	3,627906977
PEMT	4,61578E-05	0,101128946	0,25	0,047	0,973975249	1,072612481	0,932303155	5,319148936
EPHA2	0,012263219	0,101111962	0,219	0,074	1	1,072599854	0,93231413	2,959459459
VPS72	3,05607E-05	0,101092656	0,281	0,055	0,644860382	1,0725855	0,932326607	5,109090909
ABCB7	9,71531E-07	0,101055729	0,25	0,031	0,020500283	1,072558047	0,932350471	8,064516129
DIAPH1	2,80921E-05	0,100903524	0,281	0,055	0,592771218	1,072444898	0,932448839	5,109090909
SH3BP4	0,000480113	0,100838984	0,125	0,016	1	1,072396923	0,932490554	7,8125
TMEM185B	0,01179781	0,100729037	0,156	0,043	1	1,072315199	0,932561621	3,627906977
FARSA	0,000114181	0,100694387	0,25	0,051	1	1,072289444	0,932584019	4,901960784
TPGS2	1,02794E-07	0,100597458	0,344	0,051	0,00216905	1,072217404	0,932646678	6,745098039
RCE1	2,11157E-07	0,100579136	0,219	0,02	0,00445563	1,072203787	0,932658523	10,95
EXO5	9,12354E-07	0,10045556	0,094	0	0,019251588	1,07211195	0,932738414	#DIV/0!
DOK3	0,002263143	0,100412207	0,062	0,004	1	1,072079733	0,932766444	15,5
H2AZ1	0,049504971	0,100246533	0,812	0,469	1	1,071956627	0,932873565	1,731343284
AMDHD2	4,92471E-08	0,100216301	0,281	0,031	0,001039163	1,071934164	0,932893114	9,064516129
UBL7	4,80509E-06	0,100202265	0,188	0,02	0,101392141	1,071923735	0,93290219	9,4
METTL13	0,000108998	0,100161609	0,125	0,012	1	1,071893528	0,93292848	10,41666667
NDST2	0,002263143	0,100147438	0,062	0,004	1	1,071882999	0,932937644	15,5
DDX55	0,000850121	0,100035904	0,156	0,027	1	1,071800136	0,933009772	5,777777778
LIN9	0,000524345	0,100005012	0,094	0,008	1	1,071777186	0,93302975	11,75
NPR2	0,040857201	0,09995049	0,062	0,012	1	1,071736682	0,933065012	5,166666667
AC093323.1	2,44525E-05	0,099916492	0,25	0,043	0,515972154	1,071711427	0,933087	5,813953488
ARL6IP6	4,2156E-05	0,099847254	0,531	0,156	0,889534105	1,071659994	0,933131782	3,403846154
IMPA2	3,67695E-07	0,099796531	0,156	0,008	0,007758733	1,071622317	0,93316459	19,5

Supplementary Table 1

Differentially expressed genes_adjacent-skin-XL_ comparing SC-Keloid vs. (SC-Myel, SC-Nonmyel)								
**gene**	**p_val**	**avg_log2FC**	**pct.1**	**pct.2**	**p_val_adj**	**Foldchange_UP**	**Foldchange_DOWN**	**Ratio_pct1_pct2**
ZDHHC21	3,92553E-05	0,099609622	0,219	0,035	0,828326361	1,071483491	0,933285495	6,257142857
ELOF1	0,001857229	0,099524	0,312	0,098	1	1,071419902	0,933340886	3,183673469
TTC26	4,80439E-05	0,099416375	0,094	0,004	1	1,071339977	0,933410515	23,5
ENO2	0,073365787	0,099392255	0,156	0,062	1	1,071322066	0,933426121	2,516129032
PTK7	0,002594281	0,099293143	0,094	0,012	1	1,071248469	0,933490249	7,833333333
ALG1L	6,3574E-05	0,0992758	0,062	0	1	1,071235591	0,933501471	#DIV/0!
UPF3A	0,006484811	0,099223372	0,375	0,145	1	1,071196663	0,933535395	2,586206897
TMUB2	3,2432E-06	0,099206015	0,25	0,035	0,068434669	1,071183776	0,933546626	7,142857143
MAP3K3	0,023284222	0,099156349	0,219	0,082	1	1,0711469	0,933578765	2,670731707
SEC61A2	0,01346698	0,098827564	0,062	0,008	1	1,070902817	0,933791549	7,75
GAB2	0,662254058	0,098682556	0,031	0,02	1	1,070795185	0,93388541	1,55
RASL12	0,011486777	0,098664601	0,281	0,105	1	1,070781858	0,933897033	2,676190476
LINC00926	1,33967E-08	0,098663412	0,125	0	0,000282684	1,070780976	0,933897802	#DIV/0!
TRIT1	0,000509339	0,098642288	0,125	0,016	1	1,070765297	0,933911477	7,8125
MAP6	0,003953072	0,098626232	0,156	0,035	1	1,070753381	0,93392187	4,457142857
FUT8	0,000514321	0,098391206	0,281	0,074	1	1,070578961	0,934074026	3,797297297
PDCD6	0,00035569	0,098332828	0,625	0,223	1	1,070535641	0,934111824	2,802690583
ST8SIA4	4,80439E-05	0,098230537	0,094	0,004	1	1,07045974	0,934178057	23,5
RBMX	0,001165359	0,098195494	0,75	0,309	1	1,070433739	0,934200748	2,427184466
ZBTB42	0,37426516	0,097890154	0,031	0,012	1	1,07020721	0,934398489	2,583333333
CPSF4	0,002442928	0,097873391	0,094	0,012	1	1,070194775	0,934409346	7,833333333
MPV17L2	3,86379E-06	0,097804254	0,156	0,012	0,081529835	1,070143491	0,934454126	13
PRELID3B	3,23695E-05	0,097790432	0,312	0,066	0,68302947	1,070133238	0,934463078	4,727272727
DPCD	9,17361E-07	0,097713675	0,188	0,016	0,019357231	1,070076304	0,934512797	11,75
LAMA2	0,000901035	0,097701538	0,312	0,09	1	1,070067302	0,934520659	3,466666667
NDE1	0,036392125	0,09765727	0,188	0,07	1	1,070034468	0,934549334	2,685714286
NUDT16L1	1,97172E-06	0,097544598	0,281	0,043	0,041605191	1,069950903	0,934622324	6,534883721
SUMF2	0,002089818	0,097530846	0,438	0,16	1	1,069940705	0,934631233	2,7375
ACAA2	6,4022E-08	0,097505259	0,375	0,059	0,001350927	1,069921729	0,934647809	6,355932203
SFXN3	2,90967E-07	0,09744837	0,406	0,074	0,006139701	1,06987954	0,934684665	5,486486486

Supplementary Table 1

Differentially expressed genes_adjacent-skin-XL_ comparing SC-Keloid vs. (SC-Myel, SC-Nonmyel)								
**gene**	**p_val**	**avg_log2FC**	**pct.1**	**pct.2**	**p_val_adj**	**Foldchange_UP**	**Foldchange_DOWN**	**Ratio_pct1_pct2**
SRPRA	4,89131E-08	0,097396059	0,594	0,129	0,001032116	1,069840748	0,934718557	4,604651163
CD68	0,000108998	0,097392613	0,125	0,012	1	1,069838192	0,93472079	10,41666667
URB1-AS1	0,034213216	0,097385499	0,094	0,023	1	1,069832917	0,934725399	4,086956522
ZNF92	0,001560165	0,097338839	0,125	0,02	1	1,069798317	0,93475563	6,25
MUC12	0,000105286	0,097296445	0,125	0,012	1	1,069766881	0,934783098	10,41666667
CENPS	0,000112833	0,097159993	0,125	0,012	1	1,069665706	0,934871516	10,41666667
VPS4A	3,5393E-05	0,097125459	0,375	0,09	0,746827544	1,069640101	0,934893895	4,166666667
NTPCR	0,000983579	0,097034353	0,219	0,051	1	1,069572556	0,934952935	4,294117647
SLC6A9	0,084624339	0,097031019	0,062	0,016	1	1,069570084	0,934955095	3,875
NCBP2AS2	3,37262E-07	0,097010143	0,344	0,055	0,007116571	1,069554607	0,934968624	6,254545455
TEX12	6,3574E-05	0,096959664	0,062	0	1	1,069517185	0,935001339	#DIV/0!
PAM	3,15754E-06	0,096883513	0,25	0,035	0,066627295	1,069460734	0,935050693	7,142857143
LMF1	0,006087048	0,096863185	0,219	0,066	1	1,069445664	0,935063869	3,318181818
TPP1	0,000745105	0,096859323	0,375	0,117	1	1,069442801	0,935066372	3,205128205
SEC31A	0,000104423	0,096815295	0,625	0,211	1	1,069410165	0,935094908	2,962085308
TGFBR1	0,00011187	0,096689186	0,25	0,051	1	1,06931669	0,93517665	4,901960784
CLSTN1	0,001883587	0,096660793	0,719	0,309	1	1,069295645	0,935195055	2,326860841
YY2	0,002263143	0,096611818	0,062	0,004	1	1,069259347	0,935226803	15,5
PCDHGA7	6,3574E-05	0,096607795	0,062	0	1	1,069256365	0,935229411	#DIV/0!
KRT23	0,002263143	0,096530603	0,062	0,004	1	1,069199155	0,935279452	15,5
NEK8	0,221475664	0,096464711	0,031	0,008	1	1,069150323	0,93532217	3,875
SLC27A5	9,12354E-07	0,096417983	0,094	0	0,019251588	1,069115694	0,935352465	#DIV/0!
EMC3	0,000149678	0,096415769	0,375	0,102	1	1,069114054	0,9353539	3,676470588
R3HDM1	9,17361E-07	0,096399679	0,188	0,016	0,019357231	1,06910213	0,935364332	11,75
ANKRD7	0,004841742	0,096343869	0,031	0	1	1,069060773	0,935400517	#DIV/0!
BEX2	0,004841742	0,096343869	0,031	0	1	1,069060773	0,935400517	#DIV/0!
C10orf143	0,01346698	0,096317794	0,062	0,008	1	1,069041451	0,935417423	7,75
CLCN7	0,0028755	0,09616958	0,25	0,074	1	1,06893163	0,935513527	3,378378378
COX11	3,31426E-05	0,096166241	0,406	0,102	0,699341562	1,068929157	0,935515692	3,980392157
ERGIC1	1,08914E-05	0,096145316	0,469	0,121	0,2298184	1,068913653	0,935529261	3,876033058

Supplementary Table 1

Differentially expressed genes_adjacent-skin-XL_ comparing SC-Keloid vs. (SC-Myel, SC-Nonmyel)								
gene	p_val	avg_log2FC	pct.1	pct.2	p_val_adj	Foldchange_UP	Foldchange_DOWN	Ratio_pct1_pct2
DHX40	0,000105201	0,096115242	0,25	0,051	1	1,068891371	0,935548763	4,901960784
TMED10	0,03856194	0,096089503	0,844	0,5	1	1,0688723	0,935565455	1,688
SPRYD3	0,000849771	0,095894278	0,219	0,051	1	1,068727671	0,935692064	4,294117647
CHMP7	0,000103236	0,095887536	0,156	0,02	1	1,068722677	0,935696436	7,8
KMT2B	0,323722708	0,095886521	0,094	0,051	1	1,068721925	0,935697095	1,843137255
AKIP1	7,15271E-05	0,09588551	0,188	0,027	1	1,068721175	0,935697751	6,962962963
YEATS4	1,70654E-05	0,095816327	0,312	0,062	0,360097761	1,068669928	0,935742622	5,032258065
NFYB	0,02256494	0,095712453	0,281	0,117	1	1,068592986	0,935809998	2,401709402
SLC66A1	0,040857201	0,095710662	0,062	0,012	1	1,06859166	0,93581116	5,166666667
SYNRG	0,000677	0,095640496	0,281	0,074	1	1,06853969	0,935856674	3,797297297
REST	7,43798E-05	0,095618339	0,562	0,18	1	1,068523279	0,935871047	3,122222222
FAM174A	0,000253224	0,095592625	0,25	0,055	1	1,068504234	0,935887728	4,545454545
UBE3D	6,3574E-05	0,095546511	0,062	0	1	1,068470081	0,935917643	#DIV/0!
NPEPL1	3,56492E-06	0,095498962	0,438	0,098	0,075223275	1,068434866	0,93594849	4,469387755
MAT2B	0,005508322	0,095497835	0,188	0,051	1	1,068434032	0,935949221	3,68627451
KIFBP	0,000112852	0,095421737	0,281	0,062	1	1,068377677	0,935998591	4,532258065
ELK1	0,000870223	0,095390615	0,156	0,027	1	1,06835463	0,936018783	5,777777778
DNASE2	4,6586E-08	0,095389311	0,312	0,039	0,000983011	1,068353664	0,936019629	8
DMWD	0,000313821	0,095389218	0,406	0,121	1	1,068353596	0,936019689	3,355371901
TPRKB	6,44927E-07	0,095332924	0,344	0,059	0,013608598	1,068311909	0,936056213	5,830508475
SPATA33	0,000297876	0,095318274	0,156	0,023	1	1,068301061	0,936065719	6,782608696
SYNJ2	0,000524345	0,095302057	0,094	0,008	1	1,068289052	0,936076241	11,75
ZNF559	0,000870223	0,095271027	0,156	0,027	1	1,068266075	0,936096375	5,777777778
DNAJC11	0,212914042	0,095181859	0,062	0,023	1	1,068200051	0,936154233	2,695652174
DENND3	0,01346698	0,095181657	0,062	0,008	1	1,068199902	0,936154364	7,75
GNPNAT1	4,39695E-06	0,095137575	0,219	0,027	0,092780105	1,068167264	0,936182969	8,111111111
SKAP1	0,002263143	0,095116511	0,062	0,004	1	1,068151668	0,936196638	15,5
UCK1	0,002560056	0,095039434	0,219	0,059	1	1,068094602	0,936246656	3,711864407
ARRB2	2,40115E-05	0,094914763	0,156	0,016	0,506667402	1,068002307	0,936327565	9,75
COMT	0,211033137	0,094844819	0,969	0,652	1	1,067950529	0,936372962	1,486196319

Supplementary Table 1

Differentially expressed genes_adjacent-skin-XL_ comparing SC-Keloid vs. (SC-Myel, SC-Nonmyel)								
**gene**	**p_val**	**avg_log2FC**	**pct.1**	**pct.2**	**p_val_adj**	**Foldchange_UP**	**Foldchange_DOWN**	**Ratio_pct1_pct2**
B3GNTL1	6,3574E-05	0,094700907	0,062	0	1	1,067844004	0,936466371	#DIV/0!
N6AMT1	0,000100263	0,094646801	0,156	0,02	1	1,067803957	0,936501493	7,8
GLUD1	0,025397909	0,094539312	0,281	0,117	1	1,067724403	0,93657127	2,401709402
TRMT6	0,001847537	0,094527608	0,156	0,031	1	1,067715741	0,936578868	5,032258065
CCHCR1	1,03471E-06	0,094414838	0,219	0,023	0,021833489	1,067632285	0,93665208	9,52173913
NONO	0,000106876	0,094388521	0,656	0,219	1	1,06761281	0,936669166	2,99543379
CNPY4	0,007342225	0,094371676	0,156	0,039	1	1,067600344	0,936680103	4
FSD1	4,80439E-05	0,094298866	0,094	0,004	1	1,067546466	0,936727376	23,5
COA6	3,67604E-06	0,094021253	0,312	0,055	0,077568042	1,067341061	0,936907645	5,672727273
COMMD4	6,16792E-05	0,093968215	0,438	0,121	1	1,067301823	0,936942089	3,619834711
FAM193A	0,000751392	0,093777924	0,25	0,062	1	1,067161056	0,937065679	4,032258065
PANK4	2,40115E-05	0,093579874	0,156	0,016	0,506667402	1,067014568	0,937194327	9,75
MTPAP	0,008106602	0,093563301	0,125	0,027	1	1,067002311	0,937205093	4,62962963
NFIC	0,067903822	0,093532176	0,844	0,48	1	1,066979291	0,937225313	1,758333333
NPM3	2,40115E-05	0,093479623	0,156	0,016	0,506667402	1,066940425	0,937259454	9,75
ARSB	1,51098E-05	0,093442085	0,125	0,008	0,318831333	1,066912665	0,937283841	15,625
ZNF684	0,000524345	0,093440105	0,094	0,008	1	1,066911201	0,937285127	11,75
ECE1	0,002464759	0,093367584	0,531	0,207	1	1,066857571	0,937332243	2,565217391
ECSCR	6,3574E-05	0,093356367	0,062	0	1	1,066849276	0,937339531	#DIV/0!
GPR108	1,41872E-05	0,093207268	0,312	0,062	0,299363242	1,066739026	0,937436408	5,032258065
APTX	2,11424E-07	0,09295797	0,281	0,035	0,004461255	1,066554708	0,937598411	8,028571429
ITGB3	0,002263143	0,092929097	0,062	0,004	1	1,066533364	0,937617176	15,5
MFSD4B	0,040857201	0,092922706	0,062	0,012	1	1,066528639	0,937621329	5,166666667
PI4K2B	0,040857201	0,092892834	0,062	0,012	1	1,066506556	0,937640743	5,166666667
FBXL8	6,3574E-05	0,092795047	0,062	0	1	1,066434269	0,9377043	#DIV/0!
ATP13A2	4,96307E-06	0,092748169	0,188	0,02	0,104725812	1,066399618	0,937734769	9,4
TTLL12	9,17361E-07	0,092698295	0,188	0,016	0,019357231	1,066362753	0,937767187	11,75
BYSL	0,00083045	0,092657262	0,156	0,027	1	1,066332424	0,93779386	5,777777778
DNAJC30	0,000177637	0,092559507	0,188	0,031	1	1,066260174	0,937857406	6,064516129
CLIC6	0,004841742	0,092545383	0,031	0	1	1,066249735	0,937866587	#DIV/0!

Supplementary Table 1

Differentially expressed genes_adjacent-skin-XL_ comparing SC-Keloid vs. (SC-Myel, SC-Nonmyel)								
**gene**	**p_val**	**avg_log2FC**	**pct.1**	**pct.2**	**p_val_adj**	**Foldchange_UP**	**Foldchange_DOWN**	**Ratio_pct1_pct2**
CPVL	0,004841742	0,092545383	0,031	0	1	1,066249735	0,937866587	#DIV/0!
GDPD2	0,004841742	0,092545383	0,031	0	1	1,066249735	0,937866587	#DIV/0!
HSF2BP	0,004841742	0,092545383	0,031	0	1	1,066249735	0,937866587	#DIV/0!
PCDHGB3	0,004841742	0,092545383	0,031	0	1	1,066249735	0,937866587	#DIV/0!
PPFIA2	0,004841742	0,092545383	0,031	0	1	1,066249735	0,937866587	#DIV/0!
ZNF671	0,004841742	0,092545383	0,031	0	1	1,066249735	0,937866587	#DIV/0!
MYEF2	0,035499775	0,092507298	0,094	0,023	1	1,066221588	0,937891346	4,086956522
PJA2	0,024164687	0,09247289	0,688	0,348	1	1,066196159	0,937913715	1,977011494
TBC1D20	0,018732203	0,092419814	0,156	0,047	1	1,066156935	0,937948221	3,319148936
BLOC1S2	1,90086E-05	0,092419179	0,344	0,074	0,401099502	1,066156466	0,937948633	4,648648649
ASTE1	0,002263143	0,092416342	0,062	0,004	1	1,066154369	0,937950478	15,5
BTBD10	0,00019555	0,092298396	0,188	0,031	1	1,06606721	0,938027162	6,064516129
WNK1	0,002024853	0,092239781	0,5	0,191	1	1,066023898	0,938065274	2,617801047
PTGES3	0,098211925	0,092159853	0,875	0,516	1	1,06596484	0,938117246	1,695736434
DNAAF5	1,51098E-05	0,092148583	0,125	0,008	0,318831333	1,065956513	0,938124574	15,625
FAM174B	0,000322156	0,092129176	0,156	0,023	1	1,065942174	0,938137194	6,782608696
BORCS8	1,24109E-05	0,092067077	0,281	0,051	0,261882629	1,065896293	0,938177576	5,509803922
COQ4	0,000199866	0,092037461	0,375	0,105	1	1,065874412	0,938196835	3,571428571
CYTH2	0,00367496	0,091973145	0,344	0,121	1	1,065826896	0,938238661	2,842975207
TCHP	0,000480113	0,091957058	0,125	0,016	1	1,065815011	0,938249123	7,8125
MRPS17	4,81902E-06	0,091576314	0,281	0,047	0,101686073	1,065533767	0,938496771	5,978723404
LINC01060	9,12354E-07	0,091558987	0,094	0	0,019251588	1,06552097	0,938508042	#DIV/0!
CDK5R1	9,12354E-07	0,091453362	0,094	0	0,019251588	1,065442962	0,938576757	#DIV/0!
ATF7IP2	0,110558038	0,091448672	0,125	0,051	1	1,065439499	0,938579807	2,450980392
DECR2	2,32278E-05	0,091339153	0,156	0,016	0,490128791	1,065358621	0,938651061	9,75
ZNF48	9,12354E-07	0,09133164	0,094	0	0,019251588	1,065353074	0,938655949	#DIV/0!
MCM10	6,3574E-05	0,091257987	0,062	0	1	1,065298686	0,93870387	#DIV/0!
RMI2	6,3574E-05	0,091257987	0,062	0	1	1,065298686	0,93870387	#DIV/0!
CHEK1	6,3574E-05	0,091234957	0,062	0	1	1,065281681	0,938718855	#DIV/0!
NEFH	9,12354E-07	0,091102152	0,094	0	0,019251588	1,065183623	0,938805271	#DIV/0!

Supplementary Table 1

Differentially expressed genes_adjacent-skin-XL_ comparing SC-Keloid vs. (SC-Myel, SC-Nonmyel)								
**gene**	**p_val**	**avg_log2FC**	**pct.1**	**pct.2**	**p_val_adj**	**Foldchange_UP**	**Foldchange_DOWN**	**Ratio_pct1_pct2**
ZNF20	3,86379E-06	0,090947562	0,156	0,012	0,081529835	1,06506949	0,938905873	13
NUP155	0,017290773	0,090820879	0,094	0,02	1	1,06497597	0,938988323	4,7
FAM91A1	0,001637922	0,090820149	0,219	0,055	1	1,064975432	0,938988797	3,981818182
CIPC	0,287158009	0,090817112	0,062	0,027	1	1,06497319	0,938990774	2,296296296
ZNF837	6,3574E-05	0,090813277	0,062	0	1	1,064970359	0,93899327	#DIV/0!
AKNAD1	0,380266129	0,090657345	0,031	0,012	1	1,064855259	0,939094765	2,583333333
SNX14	3,39075E-07	0,090508149	0,406	0,074	0,007154826	1,064745143	0,939191887	5,486486486
SETMAR	0,002263143	0,090494244	0,062	0,004	1	1,064734881	0,939200939	15,5
ICAM2	0,000524345	0,090419119	0,094	0,008	1	1,064679439	0,939249847	11,75
PLEKHG4	0,002517564	0,09040814	0,094	0,012	1	1,064671337	0,939256995	7,833333333
ELOC	0,003199976	0,0903436	0,719	0,312	1	1,064623709	0,939299014	2,304487179
KIF15	6,3574E-05	0,090243631	0,062	0	1	1,06454994	0,939364103	#DIV/0!
ARHGAP11A	6,3574E-05	0,090220585	0,062	0	1	1,064532934	0,939379109	#DIV/0!
RTEL1	0,01346698	0,09017173	0,062	0,008	1	1,064496886	0,939410921	7,75
PLXNB2	0,000135099	0,090113103	0,469	0,145	1	1,064453629	0,939449097	3,234482759
HECTD2	0,003820406	0,090079978	0,125	0,023	1	1,064429189	0,939470667	5,434782609
UROD	7,33388E-07	0,090057297	0,281	0,039	0,015475215	1,064412455	0,939485437	7,205128205
FBXO16	4,80439E-05	0,089977265	0,094	0,004	1	1,06435341	0,939537555	23,5
HP1BP3	0,023702854	0,08995558	0,781	0,41	1	1,064337412	0,939551677	1,904878049
DHX32	0,00019555	0,089881007	0,188	0,031	1	1,064282397	0,939600244	6,064516129
TSPAN31	0,00038432	0,089769215	0,281	0,07	1	1,064199931	0,939673055	4,014285714
APCDD1L	9,12354E-07	0,0897445	0,094	0	0,019251588	1,0641817	0,939689153	#DIV/0!
MFSD11	0,001477662	0,089702738	0,219	0,055	1	1,064150896	0,939716354	3,981818182
UFM1	0,003052977	0,089694632	0,531	0,219	1	1,064144916	0,939721634	2,424657534
IFI44	0,00024217	0,08933215	0,219	0,043	1	1,06387758	0,939957772	5,093023256
FBXO28	0,001637922	0,089322968	0,219	0,055	1	1,063870809	0,939963755	3,981818182
KNL1	0,004841742	0,089205499	0,031	0	1	1,063784188	0,940040293	#DIV/0!
MKI67	0,004841742	0,089205499	0,031	0	1	1,063784188	0,940040293	#DIV/0!
CARD6	0,00050526	0,089123976	0,094	0,008	1	1,063724079	0,940093413	11,75
B9D1	1,51098E-05	0,089029677	0,125	0,008	0,318831333	1,063654553	0,940154863	15,625

Supplementary Table 1

Differentially expressed genes_adjacent-skin-XL_ comparing SC-Keloid vs. (SC-Myel, SC-Nonmyel)								
**gene**	**p_val**	**avg_log2FC**	**pct.1**	**pct.2**	**p_val_adj**	**Foldchange_UP**	**Foldchange_DOWN**	**Ratio_pct1_pct2**
MAML3	0,000486986	0,088914518	0,188	0,035	1	1,063569653	0,940229911	5,371428571
HHLA3	0,000509339	0,088858582	0,125	0,016	1	1,063528417	0,940266366	7,8125
TRNT1	3,86379E-06	0,088808932	0,156	0,012	0,081529835	1,063491817	0,940298725	13
RNF217	0,00022736	0,088679282	0,219	0,043	1	1,063396248	0,940383231	5,093023256
HDDC3	0,000186392	0,088617204	0,188	0,031	1	1,063350492	0,940423696	6,064516129
FAM120A	5,72167E-05	0,088564323	0,531	0,16	1	1,063311517	0,940458167	3,31875
ADI1	0,002659405	0,088476583	0,5	0,195	1	1,063246852	0,940515364	2,564102564
ZNF776	4,80439E-05	0,088468551	0,094	0,004	1	1,063240932	0,940520601	23,5
NSD2	0,090719467	0,088372352	0,094	0,031	1	1,063170038	0,940583316	3,032258065
AP1M1	1,5064E-07	0,088352394	0,312	0,043	0,003178646	1,06315533	0,940596328	7,255813953
B3GALT2	0,000112833	0,08834627	0,125	0,012	1	1,063150817	0,940600321	10,41666667
PYURF	7,42194E-05	0,088337008	0,781	0,273	1	1,063143992	0,94060636	2,860805861
MT-ATP6	0,852540647	0,088279267	0,906	0,883	1	1,063101442	0,940644007	1,026047565
CD200	0,002818951	0,088247279	0,219	0,059	1	1,063077871	0,940664863	3,711864407
XPNPEP1	0,000181965	0,088206291	0,188	0,031	1	1,063047669	0,940691588	6,064516129
LTC4S	0,000112833	0,088167322	0,125	0,012	1	1,063018955	0,940716998	10,41666667
CAPZA1	0,000119405	0,088150474	0,562	0,18	1	1,063006541	0,940727984	3,122222222
ABAT	0,221475664	0,088137597	0,031	0,008	1	1,062997052	0,940736381	3,875
PUS7L	0,000777418	0,088089893	0,25	0,062	1	1,062961905	0,940767487	4,032258065
TUBD1	0,007750521	0,08807864	0,094	0,016	1	1,062953614	0,940774825	5,875
DIAPH3	0,002263143	0,087912849	0,062	0,004	1	1,062831469	0,940882943	15,5
C1orf94	6,3574E-05	0,087870453	0,062	0	1	1,062800236	0,940910593	#DIV/0!
DAB2	4,80439E-05	0,087856302	0,094	0,004	1	1,062789811	0,940919822	23,5
FAM120AOS	0,017756423	0,087832461	0,25	0,094	1	1,062772248	0,940935371	2,659574468
DCAKD	8,90851E-07	0,087827704	0,25	0,031	0,018797848	1,062768744	0,940938474	8,064516129
RNF40	0,00022736	0,087768643	0,219	0,043	1	1,062725237	0,940976995	5,093023256
BIN1	2,37907E-06	0,087759048	0,5	0,121	0,050200786	1,06271817	0,940983253	4,132231405
SAMD9L	0,000916506	0,08771029	0,188	0,039	1	1,062682254	0,941015056	4,820512821
UTP23	4,123E-05	0,087666244	0,219	0,035	0,869994633	1,06264981	0,941043785	6,257142857
DUSP28	0,036158386	0,087612455	0,094	0,023	1	1,062610191	0,941078872	4,086956522

Supplementary Table 1

Differentially expressed genes_adjacent-skin-XL_ comparing SC-Keloid vs. (SC-Myel, SC-Nonmyel)								
**gene**	**p_val**	**avg_log2FC**	**pct.1**	**pct.2**	**p_val_adj**	**Foldchange_UP**	**Foldchange_DOWN**	**Ratio_pct1_pct2**
P2RX7	0,00234362	0,087555371	0,312	0,102	1	1,062568147	0,941116109	3,058823529
COX7C	0,029773256	0,08751842	0,844	0,488	1	1,062540933	0,941140213	1,729508197
LEPR	0,082995684	0,087466839	0,062	0,016	1	1,062502944	0,941173863	3,875
CTNNBIP1	0,000110741	0,087441002	0,281	0,062	1	1,062483916	0,941190718	4,532258065
PLBD1	0,002517564	0,087408818	0,094	0,012	1	1,062460214	0,941211715	7,833333333
SARS1	0,008866644	0,087356989	0,531	0,234	1	1,062422046	0,941245528	2,269230769
UBA7	4,39687E-06	0,087155437	0,219	0,027	0,092778258	1,06227363	0,941377034	8,111111111
BST1	1,51098E-05	0,086877226	0,125	0,008	0,318831333	1,0620688	0,941558588	15,625
ELOVL4	0,01346698	0,086791096	0,062	0,008	1	1,062005396	0,941614802	7,75
C10orf67	3,67695E-07	0,086646657	0,156	0,008	0,007758733	1,061899076	0,941709078	19,5
IL3RA	0,004841742	0,086622355	0,031	0	1	1,061881188	0,941724942	#DIV/0!
INCENP	0,004841742	0,086622355	0,031	0	1	1,061881188	0,941724942	#DIV/0!
IZUMO1	0,004841742	0,086622355	0,031	0	1	1,061881188	0,941724942	#DIV/0!
MAGIX	0,004841742	0,086622355	0,031	0	1	1,061881188	0,941724942	#DIV/0!
PDGFRA	0,004841742	0,086622355	0,031	0	1	1,061881188	0,941724942	#DIV/0!
SLC47A1	0,004841742	0,086622355	0,031	0	1	1,061881188	0,941724942	#DIV/0!
TMEM72	0,004841742	0,086622355	0,031	0	1	1,061881188	0,941724942	#DIV/0!
ST6GAL2	9,12354E-07	0,086594208	0,094	0	0,019251588	1,061860471	0,941743315	#DIV/0!
ZNF827	0,001444236	0,086401968	0,125	0,02	1	1,061718987	0,941868811	6,25
SFT2D3	0,002442928	0,086370963	0,094	0,012	1	1,06169617	0,941889053	7,833333333
TRMT61B	5,86746E-05	0,086296469	0,25	0,047	1	1,06164135	0,941937689	5,319148936
ABCC3	9,12354E-07	0,086263813	0,094	0	0,019251588	1,06161732	0,94195901	#DIV/0!
SSR3	2,27194E-05	0,08616409	0,844	0,312	0,479402614	1,061543941	0,942024123	2,705128205
FAM72B	0,143111738	0,086132906	0,062	0,02	1	1,061520995	0,942044486	3,1
DLGAP1-AS1	0,000524345	0,08611526	0,094	0,008	1	1,061508012	0,942056008	11,75
ARMC9	9,06462E-07	0,086099069	0,125	0,004	0,019127258	1,061496099	0,94206658	31,25
POMT2	9,17E-07	0,086057045	0,25	0,031	0,01934961	1,061465179	0,942094023	8,064516129
RTL8C	0,000710949	0,085910595	0,531	0,191	1	1,061357434	0,942189661	2,780104712
LRRC34	0,013891036	0,08569931	0,062	0,008	1	1,061202008	0,942327655	7,75
RAB43	0,034851427	0,085559779	0,094	0,023	1	1,061099378	0,942418798	4,086956522

Supplementary Table 1

Differentially expressed genes_adjacent-skin-XL_ comparing SC-Keloid vs. (SC-Myel, SC-Nonmyel)								
**gene**	**p_val**	**avg_log2FC**	**pct.1**	**pct.2**	**p_val_adj**	**Foldchange_UP**	**Foldchange_DOWN**	**Ratio_pct1_pct2**
GIMAP4	0,082317662	0,085548854	0,031	0,004	1	1,061091343	0,942425934	7,75
DDR2	7,95124E-07	0,085501387	0,375	0,07	0,016777907	1,061056431	0,942456942	5,357142857
MTF1	0,777381931	0,085373644	0,031	0,023	1	1,060962485	0,942540396	1,347826087
TSTD2	0,005420294	0,085351599	0,188	0,051	1	1,060946273	0,942554798	3,68627451
DNAJC16	0,000509339	0,085310309	0,125	0,016	1	1,060915909	0,942581774	7,8125
ACO1	2,33422E-05	0,085308036	0,25	0,043	0,492544426	1,060914238	0,942583259	5,813953488
BDKRB1	0,079594089	0,085300391	0,031	0,004	1	1,060908616	0,942588254	7,75
TIMM17B	3,18992E-05	0,085231482	0,375	0,09	0,673105308	1,060857943	0,942633277	4,166666667
SDHAF3	7,93488E-09	0,085044584	0,375	0,051	0,000167434	1,06072052	0,942755401	7,352941176
ENTPD5	0,018047186	0,085034639	0,094	0,02	1	1,060713209	0,9427619	4,7
WDR3	0,000100263	0,085002427	0,156	0,02	1	1,060689526	0,94278295	7,8
SEC11C	0,000546674	0,084948386	0,375	0,117	1	1,060649795	0,942818265	3,205128205
SLC35B3	0,000466117	0,084802355	0,188	0,035	1	1,060542441	0,942913703	5,371428571
CENPBD1	0,002263143	0,084666168	0,062	0,004	1	1,060442332	0,943002716	15,5
MED14	0,002113111	0,084655178	0,281	0,086	1	1,060434254	0,9430099	3,26744186
TRMT11	4,48128E-07	0,084376957	0,312	0,047	0,009455951	1,060229771	0,943191775	6,638297872
TMEM267	6,78604E-05	0,084237609	0,188	0,027	1	1,06012737	0,943282881	6,962962963
H1-1	0,002263143	0,084099958	0,062	0,004	1	1,060026226	0,943372886	15,5
CEACAM6	0,004841742	0,083998986	0,031	0	1	1,059952038	0,943438914	#DIV/0!
LCN2	0,004841742	0,083998986	0,031	0	1	1,059952038	0,943438914	#DIV/0!
MYO5B	0,004841742	0,083998986	0,031	0	1	1,059952038	0,943438914	#DIV/0!
FAHD1	0,000509339	0,083996656	0,125	0,016	1	1,059950326	0,943440438	7,8125
TMEM119	6,3574E-05	0,083962888	0,062	0	1	1,059925518	0,94346252	#DIV/0!
CCDC134	6,3574E-05	0,083817773	0,062	0	1	1,059818909	0,943557424	#DIV/0!
KIAA1328	0,027421825	0,083797258	0,156	0,051	1	1,059803839	0,943570842	3,058823529
ABCA3	0,38835216	0,083786527	0,094	0,055	1	1,059795956	0,94357786	1,709090909
F8A1	0,002096899	0,083747867	0,156	0,031	1	1,059767557	0,943603145	5,032258065
CC2D1A	0,011609182	0,083653611	0,156	0,043	1	1,059698321	0,943664796	3,627906977
ANKAR	0,128095294	0,083541693	0,094	0,035	1	1,059616117	0,943738005	2,685714286
CCNG1	0,000446084	0,083516535	0,188	0,035	1	1,05959764	0,943754461	5,371428571

Supplementary Table 1

Differentially expressed genes_adjacent-skin-XL_ comparing SC-Keloid vs. (SC-Myel, SC-Nonmyel)								
**gene**	**p_val**	**avg_log2FC**	**pct.1**	**pct.2**	**p_val_adj**	**Foldchange_UP**	**Foldchange_DOWN**	**Ratio_pct1_pct2**
PSMG1	0,003540212	0,083507449	0,188	0,047	1	1,059590966	0,943760406	4
CAMK1D	0,000487956	0,083484299	0,219	0,047	1	1,059573964	0,943775549	4,659574468
CHCHD10	0,001490764	0,083410007	0,562	0,219	1	1,059519402	0,943824151	2,566210046
RALGPS1	0,143111738	0,083351756	0,062	0,02	1	1,059476623	0,94386226	3,1
MAPKAPK5	2,63664E-05	0,08326555	0,281	0,055	0,556356432	1,059413318	0,94391866	5,109090909
GALK1	0,001019952	0,083226227	0,219	0,051	1	1,059384442	0,943944389	4,294117647
SLC30A5	3,21209E-07	0,083098145	0,344	0,055	0,006777833	1,059290395	0,944028196	6,254545455
EIF3F	0,033344813	0,083007994	0,656	0,336	1	1,059224204	0,944087188	1,952380952
CLCC1	0,00122005	0,082970999	0,25	0,066	1	1,059197043	0,944111397	3,787878788
MIA2	1,37797E-05	0,082880839	0,219	0,031	0,29076515	1,059130851	0,944170401	7,064516129
SEMA3D	0,229249342	0,082836199	0,094	0,043	1	1,05909808	0,944199615	2,186046512
ZNF10	0,035499775	0,082754425	0,094	0,023	1	1,05903805	0,944253136	4,086956522
NAP1L1	0,22259565	0,082723375	0,656	0,484	1	1,059015257	0,944273459	1,355371901
ZNF568	8,84735E-07	0,08270126	0,188	0,016	0,018668785	1,058999024	0,944287933	11,75
CST6	6,3574E-05	0,082694036	0,062	0	1	1,058993721	0,944292662	#DIV/0!
UCHL3	1,21952E-05	0,082643239	0,375	0,082	0,257330394	1,058956435	0,944325911	4,573170732
CCDC102A	0,000509339	0,082567796	0,125	0,016	1	1,058901061	0,944375293	7,8125
RHBDD3	0,000339359	0,08253301	0,156	0,023	1	1,058875529	0,944398064	6,782608696
FAM111B	4,80439E-05	0,082500963	0,094	0,004	1	1,058852008	0,944419043	23,5
COG3	0,01179781	0,082471947	0,156	0,043	1	1,058830712	0,944438038	3,627906977
BVES	0,000524345	0,082403257	0,094	0,008	1	1,0587803	0,944483005	11,75
JAGN1	8,52679E-07	0,082377578	0,344	0,059	0,017992372	1,058761455	0,944499817	5,830508475
UBAC2	0,00025286	0,082312709	0,281	0,066	1	1,05871385	0,944542286	4,257575758
PPP3CC	0,000862411	0,082267925	0,312	0,09	1	1,058680986	0,944571607	3,466666667
ZNF641	0,008437622	0,082263291	0,125	0,027	1	1,058677585	0,944574641	4,62962963
FAM47E	6,3574E-05	0,082258265	0,062	0	1	1,058673897	0,944577932	#DIV/0!
ST3GAL1	0,175414505	0,082257002	0,094	0,039	1	1,05867297	0,944578759	2,41025641
KLHL18	0,00083045	0,08218628	0,156	0,027	1	1,058621075	0,944625063	5,777777778
IQGAP1	0,002600504	0,082184802	0,625	0,258	1	1,05861999	0,944626031	2,42248062
USP51	0,179574158	0,082139803	0,125	0,059	1	1,058586971	0,944655496	2,118644068

Supplementary Table 1

Differentially expressed genes_adjacent-skin-XL_ comparing SC-Keloid vs. (SC-Myel, SC-Nonmyel)								
**gene**	**p_val**	**avg_log2FC**	**pct.1**	**pct.2**	**p_val_adj**	**Foldchange_UP**	**Foldchange_DOWN**	**Ratio_pct1_pct2**
SEPTIN5	4,80439E-05	0,082052559	0,094	0,004	1	1,058522958	0,944712623	23,5
SFSWAP	0,0002964	0,081951109	0,531	0,18	1	1,058448525	0,944779058	2,95
C9orf40	1,51098E-05	0,081774759	0,125	0,008	0,318831333	1,058319151	0,944894552	15,625
ENTPD4	0,006536686	0,081739009	0,219	0,066	1	1,058292927	0,944917966	3,318181818
CKAP2	0,000509339	0,081724584	0,125	0,016	1	1,058282345	0,944927414	7,8125
CCNB2	9,12354E-07	0,081675527	0,094	0	0,019251588	1,058246361	0,944959545	#DIV/0!
SGCE	0,082320805	0,081666166	0,719	0,438	1	1,058239494	0,944965677	1,641552511
RAB22A	3,87818E-06	0,081464538	0,469	0,113	0,081833425	1,058091607	0,945097753	4,150442478
TUT7	0,003304112	0,081435522	0,188	0,047	1	1,058070327	0,945116761	4
POGLUT1	0,004180266	0,081424448	0,125	0,023	1	1,058062205	0,945124016	5,434782609
FASTKD2	0,038025501	0,081326424	0,156	0,055	1	1,057990317	0,945188235	2,836363636
LINC00960	0,002263143	0,081279683	0,062	0,004	1	1,05795604	0,945218858	15,5
ALG12	0,003820406	0,081217731	0,125	0,023	1	1,057910611	0,945259448	5,434782609
CCNC	0,000242168	0,081132742	0,219	0,043	1	1,057848291	0,945315135	5,093023256
FIP1L1	5,4442E-05	0,080980663	0,344	0,082	1	1,057736786	0,945414789	4,195121951
AASDHPPT	0,000125591	0,080977103	0,312	0,074	1	1,057734176	0,945417121	4,216216216
DPF2	0,167341781	0,080902071	0,156	0,078	1	1,057679167	0,945466292	2
AC010976.2	0,004841742	0,080886111	0,031	0	1	1,057667466	0,945476751	#DIV/0!
CADPS2	0,004841742	0,080886111	0,031	0	1	1,057667466	0,945476751	#DIV/0!
LINC00865	0,004841742	0,080886111	0,031	0	1	1,057667466	0,945476751	#DIV/0!
TWNK	0,004841742	0,080886111	0,031	0	1	1,057667466	0,945476751	#DIV/0!
WFIKKN1	0,079594089	0,08077911	0,031	0,004	1	1,057589025	0,945546877	7,75
PAPSS1	0,002435762	0,080742375	0,438	0,16	1	1,057562096	0,945570954	2,7375
SPACA9	0,007750521	0,080712923	0,094	0,016	1	1,057540506	0,945590258	5,875
RAB40C	0,000103236	0,080635966	0,156	0,02	1	1,057484096	0,9456407	7,8
FBXO36	9,06462E-07	0,080617243	0,125	0,004	0,019127258	1,057470372	0,945652972	31,25
PAXIP1-AS2	0,002263143	0,080616181	0,062	0,004	1	1,057469593	0,945653668	15,5
SEMA6C	0,018047186	0,080553931	0,094	0,02	1	1,057423967	0,945694472	4,7
CHAMP1	0,060931358	0,080549313	0,094	0,027	1	1,057420581	0,9456975	3,481481481
PAGR1	4,10016E-08	0,080474171	0,406	0,066	0,000865174	1,057365508	0,945746757	6,151515152

Supplementary Table 1

Differentially expressed genes_adjacent-skin-XL_ comparing SC-Keloid vs. (SC-Myel, SC-Nonmyel)								
**gene**	**p_val**	**avg_log2FC**	**pct.1**	**pct.2**	**p_val_adj**	**Foldchange_UP**	**Foldchange_DOWN**	**Ratio_pct1_pct2**
PIK3CD	0,002594281	0,080463818	0,094	0,012	1	1,05735792	0,945753544	7,833333333
ZNF738	0,000494525	0,080366816	0,125	0,016	1	1,057286829	0,945817135	7,8125
CDKL5	0,29055378	0,080311011	0,062	0,027	1	1,057245933	0,945853721	2,296296296
RARS1	6,94943E-07	0,080298719	0,281	0,039	0,014663999	1,057236925	0,94586178	7,205128205
NDUFA10	3,62687E-05	0,080279173	0,562	0,168	0,765305132	1,057222602	0,945874595	3,345238095
LRRC8A	0,000113954	0,080164665	0,375	0,098	1	1,057138692	0,945949673	3,826530612
ESS2	0,002517564	0,079813049	0,094	0,012	1	1,056881076	0,946180249	7,833333333
LDOC1	0,129733608	0,079798088	0,094	0,035	1	1,056870116	0,946190061	2,685714286
HSD17B14	0,039886012	0,079780842	0,062	0,012	1	1,056857483	0,946201372	5,166666667
HCG27	0,226351275	0,079619604	0,031	0,008	1	1,056739373	0,946307127	3,875
DDX60	0,140820961	0,07959983	0,062	0,02	1	1,056724889	0,946320098	3,1
FHL2	5,12603E-06	0,079595221	0,188	0,02	0,108164307	1,056721513	0,946323121	9,4
ANO10	1,26889E-06	0,079471638	0,312	0,051	0,026774874	1,056630997	0,946404187	6,117647059
SPAG1	0,001600673	0,079444786	0,125	0,02	1	1,056611331	0,946421802	6,25
GLRB	4,80439E-05	0,07939217	0,094	0,004	1	1,056572796	0,946456319	23,5
CCR1	6,3574E-05	0,079375314	0,062	0	1	1,056560451	0,946467378	#DIV/0!
MAPK9	0,005248131	0,079363727	0,188	0,051	1	1,056551966	0,946474979	3,68627451
AC093690.1	0,082317662	0,079314124	0,031	0,004	1	1,05651564	0,946507522	7,75
BRF1	0,142206469	0,079307796	0,125	0,055	1	1,056511006	0,946511673	2,272727273
CEP55	0,002263143	0,07926762	0,062	0,004	1	1,056481584	0,946538032	15,5
H1-3	0,040857201	0,079250415	0,062	0,012	1	1,056468985	0,946549321	5,166666667
DAXX	0,00083045	0,079236421	0,156	0,027	1	1,056458738	0,946558502	5,777777778
UBE3B	0,035499775	0,079231998	0,094	0,023	1	1,056455499	0,946561404	4,086956522
AGFG1	0,001103204	0,079209322	0,375	0,121	1	1,056438894	0,946576281	3,099173554
RBBP9	4,80439E-05	0,078904451	0,094	0,004	1	1,05621567	0,946776334	23,5
USP46	0,001452401	0,078862113	0,219	0,055	1	1,056184674	0,946804119	3,981818182
HSD17B4	5,07132E-06	0,078859274	0,375	0,078	0,107009836	1,056182596	0,946805982	4,807692308
CUL7	0,082995684	0,078667034	0,062	0,016	1	1,056041869	0,946932152	3,875
PPP6R1	0,025952325	0,078637925	0,156	0,051	1	1,056020561	0,946951259	3,058823529
WSB2	2,00071E-05	0,078616604	0,375	0,086	0,42217062	1,056004955	0,946965254	4,360465116

Supplementary Table 1

Differentially expressed genes_adjacent-skin-XL_ comparing SC-Keloid vs. (SC-Myel, SC-Nonmyel)								
**gene**	**p_val**	**avg_log2FC**	**pct.1**	**pct.2**	**p_val_adj**	**Foldchange_UP**	**Foldchange_DOWN**	**Ratio_pct1_pct2**
PRMT5-AS1	0,002263143	0,078607272	0,062	0,004	1	1,055998124	0,946971379	15,5
ADGRE5	0,000634905	0,078581995	0,281	0,074	1	1,055979622	0,946987971	3,797297297
TMEM109	0,000897631	0,078561486	0,625	0,234	1	1,055964611	0,947001433	2,670940171
YIPF2	0,000432429	0,078547723	0,281	0,07	1	1,055954537	0,947010467	4,014285714
AC011484.1	0,004841742	0,078341451	0,031	0	1	1,055803571	0,947145877	#DIV/0!
CNTNAP5	0,004841742	0,078341451	0,031	0	1	1,055803571	0,947145877	#DIV/0!
GNG3	0,004841742	0,078341451	0,031	0	1	1,055803571	0,947145877	#DIV/0!
PSORS1C1	0,004841742	0,078341451	0,031	0	1	1,055803571	0,947145877	#DIV/0!
SORL1	0,004841742	0,078341451	0,031	0	1	1,055803571	0,947145877	#DIV/0!
GTF2H2C	0,129733608	0,078322752	0,094	0,035	1	1,055789887	0,947158154	2,685714286
BZW1	0,000853194	0,07832207	0,719	0,281	1	1,055789388	0,947158602	2,558718861
GSK3A	0,010257922	0,078278057	0,25	0,086	1	1,055757179	0,947187497	2,906976744
EXOSC9	1,32717E-05	0,078270838	0,281	0,051	0,280046571	1,055751896	0,947192237	5,509803922
ATP9A	0,002594281	0,078161424	0,094	0,012	1	1,055671831	0,947264075	7,833333333
BTN3A2	3,86379E-06	0,078144266	0,156	0,012	0,081529835	1,055659276	0,947275341	13
MTMR4	0,00019555	0,078058032	0,188	0,031	1	1,055596178	0,947331964	6,064516129
MRPS22	0,000239783	0,078043209	0,281	0,066	1	1,055585332	0,947341697	4,257575758
TTLL7	0,00273635	0,077981543	0,469	0,188	1	1,055540213	0,947382191	2,494680851
RIN1	0,000112833	0,077889342	0,125	0,012	1	1,055472757	0,947442739	10,41666667
EPS8L1	6,3574E-05	0,077856125	0,062	0	1	1,055448456	0,947464553	#DIV/0!
SAYSD1	0,000200286	0,077809269	0,188	0,031	1	1,055414177	0,947495326	6,064516129
ATL2	0,000897835	0,077807877	0,219	0,051	1	1,055413159	0,94749624	4,294117647
NPHP1	0,000100263	0,077780833	0,156	0,02	1	1,055393376	0,947514001	7,8
ZUP1	0,082317662	0,077670021	0,031	0,004	1	1,055312315	0,947586782	7,75
GAREM1	0,015983649	0,07765547	0,125	0,031	1	1,055301671	0,947596339	4,032258065
ZFYVE16	0,000684407	0,077651104	0,469	0,16	1	1,055298478	0,947599206	2,93125
DNAH5	1,33967E-08	0,077628488	0,125	0	0,000282684	1,055281934	0,947614062	#DIV/0!
VEGFA	0,001643475	0,077574778	0,5	0,191	1	1,055242648	0,947649341	2,617801047
TCF7	0,000339359	0,07745034	0,156	0,023	1	1,055151633	0,947731083	6,782608696
ALDH1B1	4,80439E-05	0,077447105	0,094	0,004	1	1,055149268	0,947733208	23,5

Supplementary Table 1

Differentially expressed genes_adjacent-skin-XL_ comparing SC-Keloid vs. (SC-Myel, SC-Nonmyel)								
**gene**	**p_val**	**avg_log2FC**	**pct.1**	**pct.2**	**p_val_adj**	**Foldchange_UP**	**Foldchange_DOWN**	**Ratio_pct1_pct2**
CTTN	0,000485886	0,077413546	0,75	0,277	1	1,055124723	0,947755254	2,707581227
SMARCD1	0,000313264	0,077359619	0,344	0,094	1	1,055085284	0,947790681	3,659574468
TIMM8A	0,007750521	0,077256673	0,094	0,016	1	1,055009999	0,947858315	5,875
C15orf48	0,079594089	0,077167581	0,031	0,004	1	1,05494485	0,947916851	7,75
CEBPG	0,005165859	0,077028757	0,25	0,078	1	1,054843343	0,948008069	3,205128205
ZNF616	0,013891036	0,07700614	0,062	0,008	1	1,054826806	0,948022931	7,75
MRPL46	1,51098E-05	0,076992157	0,125	0,008	0,318831333	1,054816583	0,948032119	15,625
DOLPP1	2,40115E-05	0,076963317	0,156	0,016	0,506667402	1,054795497	0,94805107	9,75
SF3A3	3,5672E-05	0,076820082	0,312	0,066	0,752713874	1,054690779	0,9481452	4,727272727
SRPK2	0,006504289	0,076658045	0,406	0,16	1	1,054572327	0,948251698	2,5375
RAP1B	0,000709604	0,076577963	0,812	0,32	1	1,054513791	0,948304336	2,5375
IRAK4	2,32278E-05	0,07649244	0,156	0,016	0,490128791	1,054451281	0,948360553	9,75
PEX11G	0,002594281	0,076375794	0,094	0,012	1	1,05436603	0,948437233	7,833333333
TMEM251	0,000466117	0,076292362	0,188	0,035	1	1,054305056	0,948492084	5,371428571
C15orf65	4,80439E-05	0,076287002	0,094	0,004	1	1,05430114	0,948495608	23,5
COX18	0,079594089	0,076282901	0,031	0,004	1	1,054298142	0,948498304	7,75
MAN1A2	2,22383E-06	0,07614847	0,531	0,133	0,046924959	1,054199907	0,948586689	3,992481203
DPAGT1	5,12603E-06	0,076146897	0,188	0,02	0,108164307	1,054198758	0,948587724	9,4
DOLK	1,51098E-05	0,076144569	0,125	0,008	0,318831333	1,054197056	0,948589255	15,625
IFIT5	0,283294123	0,076092196	0,094	0,047	1	1,054158787	0,948623691	2
RFC1	0,005718938	0,07608101	0,375	0,145	1	1,054150614	0,948631046	2,586206897
E2F1	6,3574E-05	0,076020136	0,062	0	1	1,054106135	0,948671074	#DIV/0!
AAGAB	2,11157E-07	0,075971019	0,219	0,02	0,00445563	1,054070249	0,948703372	10,95
TMX3	9,10554E-05	0,075950789	0,438	0,125	1	1,054055468	0,948716676	3,504
FAM83D	6,3574E-05	0,075764323	0,062	0	1	1,053919242	0,948839304	#DIV/0!
GINS1	6,3574E-05	0,075764323	0,062	0	1	1,053919242	0,948839304	#DIV/0!
SETD6	1,51098E-05	0,075749328	0,125	0,008	0,318831333	1,053908288	0,948849166	15,625
CCDC137	0,001520602	0,075674945	0,125	0,02	1	1,053853952	0,948898088	6,25
CCDC88B	6,3574E-05	0,075621367	0,062	0	1	1,053814815	0,948933328	#DIV/0!
ZMYND15	6,3574E-05	0,075621367	0,062	0	1	1,053814815	0,948933328	#DIV/0!

Supplementary Table 1

Differentially expressed genes_adjacent-skin-XL_ comparing SC-Keloid vs. (SC-Myel, SC-Nonmyel)								
**gene**	**p_val**	**avg_log2FC**	**pct.1**	**pct.2**	**p_val_adj**	**Foldchange_UP**	**Foldchange_DOWN**	**Ratio_pct1_pct2**
B3GNT7	0,001642148	0,075573381	0,125	0,02	1	1,053779764	0,948964892	6,25
ATRX	0,000491157	0,075548347	0,844	0,336	1	1,053761479	0,948981358	2,511904762
LIPH	0,079594089	0,075449312	0,031	0,004	1	1,053689145	0,949046504	7,75
HCG18	0,044664395	0,075435771	0,188	0,074	1	1,053679256	0,949055412	2,540540541
TFB2M	0,082995684	0,075425312	0,062	0,016	1	1,053671617	0,949062292	3,875
CCNA2	0,013891036	0,075406541	0,062	0,008	1	1,053657907	0,949074641	7,75
ESCO1	0,003877934	0,075373062	0,156	0,035	1	1,053633456	0,949096666	4,457142857
SLC2A6	9,12354E-07	0,075322703	0,094	0	0,019251588	1,053596679	0,949129795	#DIV/0!
ULK3	0,00819789	0,075279151	0,188	0,055	1	1,053564874	0,949158448	3,418181818
FAM86C1P	0,013891036	0,075271958	0,062	0,008	1	1,053559621	0,94916318	7,75
ELOVL5	0,002083508	0,075270634	0,344	0,113	1	1,053558654	0,949164051	3,044247788
AMPH	6,3574E-05	0,075202982	0,062	0	1	1,05350925	0,949208562	#DIV/0!
C3AR1	6,3574E-05	0,075137816	0,062	0	1	1,053461665	0,949251438	#DIV/0!
FAM172A	0,000199867	0,075133993	0,375	0,105	1	1,053458873	0,949253953	3,571428571
SBSN	0,002263143	0,075106396	0,062	0,004	1	1,053438723	0,949272111	15,5
ZNF280C	0,221475664	0,075104247	0,031	0,008	1	1,053437153	0,949273525	3,875
ERGIC3	0,001313187	0,075099587	0,5	0,188	1	1,05343375	0,949276592	2,659574468
TMEM69	0,001600673	0,075094286	0,125	0,02	1	1,05342988	0,94928008	6,25
STARD3NL	3,90616E-06	0,075085114	0,344	0,066	0,082423904	1,053423183	0,949286115	5,212121212
LINC01117	0,040857201	0,075077369	0,062	0,012	1	1,053417527	0,949291211	5,166666667
ZKSCAN1	0,000119144	0,074997475	0,312	0,074	1	1,053359193	0,949343782	4,216216216
LPIN3	0,036158386	0,074865965	0,094	0,023	1	1,053263177	0,949430324	4,086956522
RAB35	3,35667E-06	0,074767321	0,312	0,055	0,070829145	1,053191163	0,949495244	5,672727273
ARID3A	0,007947097	0,074766701	0,094	0,016	1	1,05319071	0,949495652	5,875
RIMS2	0,004841742	0,074736776	0,031	0	1	1,053168864	0,949515347	#DIV/0!
EHD4	1,46747E-07	0,074727728	0,625	0,148	0,003096511	1,05316226	0,949521302	4,222972973
CDC20	0,004841742	0,074715715	0,031	0	1	1,05315349	0,949529208	#DIV/0!
STC1	0,133058992	0,074707403	0,094	0,035	1	1,053147423	0,949534679	2,685714286
GPR137	0,00010081	0,074654261	0,219	0,039	1	1,05310863	0,949569656	5,615384615
BAG6	7,067E-05	0,074587677	0,469	0,141	1	1,053060028	0,949613482	3,326241135

Supplementary Table 1

Differentially expressed genes_adjacent-skin-XL_ comparing SC-Keloid vs. (SC-Myel, SC-Nonmyel)								
**gene**	**p_val**	**avg_log2FC**	**pct.1**	**pct.2**	**p_val_adj**	**Foldchange_UP**	**Foldchange_DOWN**	**Ratio_pct1_pct2**
SCO2	5,12603E-06	0,07458487	0,188	0,02	0,108164307	1,053057979	0,94961533	9,4
MROH6	0,002263143	0,074560146	0,062	0,004	1	1,053039932	0,949631604	15,5
RUNDC3B	0,082317662	0,074428382	0,031	0,004	1	1,052943761	0,949718339	7,75
LPCAT3	0,12647311	0,074411273	0,094	0,035	1	1,052931274	0,949729602	2,685714286
VPS26C	0,041443265	0,074377882	0,125	0,039	1	1,052906905	0,949751584	3,205128205
UBXN4	0,024661405	0,074360855	0,625	0,324	1	1,052894478	0,949762793	1,929012346
ENKD1	6,96709E-05	0,074269061	0,188	0,027	1	1,052827487	0,949823225	6,962962963
NPTXR	0,000524345	0,074200379	0,094	0,008	1	1,052777367	0,949868445	11,75
NUAK2	0,002594281	0,073973697	0,094	0,012	1	1,052611963	0,950017704	7,833333333
ADPRH	0,002263143	0,073952741	0,062	0,004	1	1,052596674	0,950031503	15,5
MCAT	9,06462E-07	0,07388259	0,125	0,004	0,019127258	1,052545493	0,950077699	31,25
PEX1	0,129733608	0,073789606	0,094	0,035	1	1,052477656	0,950138935	2,685714286
TLCD3A	1,51098E-05	0,073697702	0,125	0,008	0,318831333	1,052410612	0,950199464	15,625
EMC1	0,000881541	0,073658877	0,219	0,051	1	1,052382291	0,950225036	4,294117647
BCL2L12	0,000103236	0,073622846	0,156	0,02	1	1,052356008	0,950248768	7,8
GGPS1	0,007214939	0,073593337	0,156	0,039	1	1,052334484	0,950268204	4
WASHC5	0,000112833	0,073579304	0,125	0,012	1	1,052324248	0,950277448	10,41666667
TCF12	0,000172259	0,07350612	0,344	0,09	1	1,052270868	0,950325653	3,822222222
AP5S1	3,86379E-06	0,073490716	0,156	0,012	0,081529835	1,052259633	0,9503358	13
HES5	0,082317662	0,073457354	0,031	0,004	1	1,052235299	0,950357777	7,75
ZNF714	0,002263143	0,073440709	0,062	0,004	1	1,052223159	0,950368742	15,5
TUBGCP5	0,000103236	0,073381514	0,156	0,02	1	1,052179986	0,950407737	7,8
CWC27	0,001582806	0,073348325	0,219	0,055	1	1,052155781	0,950429602	3,981818182
MYO9B	0,002990404	0,073219252	0,531	0,211	1	1,052061653	0,950514637	2,516587678
NT5C3A	0,000862006	0,07314507	0,375	0,117	1	1,052007559	0,950563512	3,205128205
SVBP	0,178976513	0,073000807	0,156	0,078	1	1,051902368	0,950658569	2
TEP1	0,040857201	0,072870018	0,062	0,012	1	1,051807011	0,950744756	5,166666667
RNF121	0,000190919	0,072865636	0,188	0,031	1	1,051803816	0,950747644	6,064516129
MAP2K5	0,039090354	0,072841761	0,125	0,039	1	1,05178641	0,950763378	3,205128205
FREM1	6,3574E-05	0,072710725	0,062	0	1	1,051690884	0,950849737	#DIV/0!

Supplementary Table 1

Differentially expressed genes_adjacent-skin-XL_ comparing SC-Keloid vs. (SC-Myel, SC-Nonmyel)								
**gene**	**p_val**	**avg_log2FC**	**pct.1**	**pct.2**	**p_val_adj**	**Foldchange_UP**	**Foldchange_DOWN**	**Ratio_pct1_pct2**
UNC79	0,082317662	0,07256341	0,031	0,004	1	1,051583499	0,950946835	7,75
GCFC2	0,000931266	0,072535737	0,219	0,051	1	1,051563329	0,950965075	4,294117647
CBL	0,01641551	0,072423629	0,25	0,094	1	1,051481618	0,951038975	2,659574468
CD79B	0,082317662	0,072182032	0,031	0,004	1	1,051305549	0,951198251	7,75
SMU1	0,007571116	0,072145619	0,312	0,113	1	1,051279015	0,951222259	2,761061947
C12orf76	5,63881E-05	0,072040868	0,344	0,082	1	1,051202686	0,951291328	4,195121951
RPGRIP1L	3,86379E-06	0,072032975	0,156	0,012	0,081529835	1,051196935	0,951296533	13
MRPL27	3,54056E-05	0,071889559	0,406	0,102	0,747094241	1,051092443	0,951391104	3,980392157
B3GNT5	0,000881541	0,071858119	0,219	0,051	1	1,051069537	0,951411838	4,294117647
CNOT6L	0,004569341	0,071833157	0,25	0,078	1	1,051051351	0,9514283	3,205128205
PTPRB	0,082317662	0,071687861	0,031	0,004	1	1,050945504	0,951524124	7,75
PSMB8	0,000342424	0,071598492	0,469	0,152	1	1,050880404	0,951583069	3,085526316
GPATCH2	5,85504E-05	0,071514999	0,375	0,094	1	1,050819588	0,951638141	3,989361702
AP4E1	3,67695E-07	0,071475232	0,156	0,008	0,007758733	1,050790624	0,951664373	19,5
HIBADH	9,43886E-07	0,071424193	0,25	0,031	0,019916948	1,05075345	0,951698041	8,064516129
ALDH1L2	0,000112833	0,07140308	0,125	0,012	1	1,050738072	0,951711969	10,41666667
PDE6D	0,000804279	0,071399872	0,25	0,062	1	1,050735736	0,951714085	4,032258065
CCDC28A	0,211308821	0,071316689	0,156	0,082	1	1,050675154	0,951768961	1,902439024
ASAH1	0,004580593	0,071315395	0,562	0,246	1	1,050674212	0,951769814	2,284552846
SP140L	0,007342225	0,071246945	0,156	0,039	1	1,050624363	0,951814973	4
L3MBTL3	0,177416998	0,071214656	0,094	0,039	1	1,050600849	0,951836276	2,41025641
TOR2A	1,51098E-05	0,071161057	0,125	0,008	0,318831333	1,050561818	0,951871639	15,625
UBE2Q1	0,01390071	0,071148305	0,188	0,059	1	1,050552532	0,951880053	3,186440678
EEA1	0,000610173	0,071120748	0,562	0,207	1	1,050532466	0,951898235	2,714975845
ZNF260	0,001896716	0,070920879	0,188	0,043	1	1,050386937	0,952030119	4,372093023
RAB11FIP3	0,029760483	0,070914776	0,156	0,051	1	1,050382493	0,952034146	3,058823529
RRP9	0,000487956	0,070856195	0,219	0,047	1	1,050339843	0,952072805	4,659574468
L3MBTL1	0,013891036	0,070794892	0,062	0,008	1	1,050295213	0,952113261	7,75
GALE	0,002263143	0,070692192	0,062	0,004	1	1,050220449	0,952181041	15,5
FBXO9	0,000452946	0,070684004	0,375	0,113	1	1,050214488	0,952186445	3,318584071

Supplementary Table 1

Differentially expressed genes_adjacent-skin-XL_ comparing SC-Keloid vs. (SC-Myel, SC-Nonmyel)								
**gene**	**p_val**	**avg_log2FC**	**pct.1**	**pct.2**	**p_val_adj**	**Foldchange_UP**	**Foldchange_DOWN**	**Ratio_pct1_pct2**
CRPPA	0,000494525	0,070626647	0,125	0,016	1	1,050172736	0,952224301	7,8125
COL2A1	0,082317662	0,070514376	0,031	0,004	1	1,050091015	0,952298407	7,75
DGKI	6,3574E-05	0,070485611	0,062	0	1	1,050070078	0,952317394	#DIV/0!
PPIG	0,015875905	0,070444376	0,531	0,254	1	1,050040065	0,952344613	2,090551181
TMED8	0,000112833	0,070240318	0,125	0,012	1	1,049891556	0,952479325	10,41666667
WDR77	0,000509339	0,070208374	0,125	0,016	1	1,049868309	0,952500415	7,8125
EGFL7	6,3574E-05	0,070183318	0,062	0	1	1,049850076	0,952516958	#DIV/0!
CRABP1	0,002594281	0,070141709	0,094	0,012	1	1,049819797	0,95254443	7,833333333
SLC46A1	6,3574E-05	0,07012926	0,062	0	1	1,049810738	0,952552649	#DIV/0!
LCMT1	4,0305E-06	0,070063581	0,219	0,027	0,085047498	1,049762947	0,952596015	8,111111111
ZFP37	0,00050526	0,070025094	0,094	0,008	1	1,049734942	0,952621428	11,75
GSDMB	0,655667102	0,069975342	0,031	0,02	1	1,049698742	0,95265428	1,55
FZD6	4,80439E-05	0,069790174	0,094	0,004	1	1,049564024	0,95277656	23,5
MMS19	9,17361E-07	0,069764238	0,188	0,016	0,019357231	1,049545155	0,952793689	11,75
ANKRD13D	8,70131E-06	0,069727582	0,344	0,07	0,183606386	1,049518489	0,952817897	4,914285714
CMIP	0,008971031	0,069724488	0,188	0,055	1	1,049516238	0,952819941	3,418181818
PCMT1	0,004326724	0,069721414	0,719	0,312	1	1,049514002	0,952821971	2,304487179
EIF4ENIF1	0,041848356	0,069704464	0,062	0,012	1	1,049501671	0,952833166	5,166666667
EMB	9,12354E-07	0,069687359	0,094	0	0,019251588	1,049489228	0,952844463	#DIV/0!
SLC5A6	9,06462E-07	0,069610833	0,125	0,004	0,019127258	1,049433561	0,952895007	31,25
IMPACT	6,94943E-07	0,069597745	0,281	0,039	0,014663999	1,04942404	0,952903652	7,205128205
DUSP23	0,000390562	0,069578442	0,344	0,098	1	1,049409999	0,952916401	3,510204082
WDTC1	4,02312E-05	0,069551675	0,219	0,035	0,848917917	1,049390529	0,952934082	6,257142857
OCLN	4,80439E-05	0,069504332	0,094	0,004	1	1,049356093	0,952965353	23,5
DNAH14	6,3574E-05	0,069434399	0,062	0	1	1,049305228	0,953011548	#DIV/0!
MRPL34	3,31291E-06	0,069377	0,438	0,098	0,069905783	1,049263482	0,953049465	4,469387755
PACS1	0,000118938	0,069369738	0,25	0,051	1	1,0492582	0,953054262	4,901960784
RIDA	0,002517564	0,06935818	0,094	0,012	1	1,049249794	0,953061898	7,833333333
LENG9	0,53887511	0,06932648	0,062	0,039	1	1,04922674	0,953082839	1,58974359
CCDC61	0,000524345	0,069163922	0,094	0,008	1	1,049108523	0,953190235	11,75

Supplementary Table 1

Differentially expressed genes_adjacent-skin-XL_ comparing SC-Keloid vs. (SC-Myel, SC-Nonmyel)								
**gene**	**p_val**	**avg_log2FC**	**pct.1**	**pct.2**	**p_val_adj**	**Foldchange_UP**	**Foldchange_DOWN**	**Ratio_pct1_pct2**
FAM234A	4,90537E-05	0,06916208	0,469	0,133	1	1,049107183	0,953191453	3,526315789
AGPAT5	1,12327E-08	0,069160498	0,312	0,035	0,000237021	1,049106033	0,953192498	8,914285714
EIF2B1	6,71476E-05	0,069116989	0,344	0,082	1	1,049074394	0,953221245	4,195121951
MVK	0,059959447	0,069107877	0,094	0,027	1	1,049067768	0,953227265	3,481481481
RRM1	9,85458E-05	0,068989487	0,219	0,039	1	1,048981684	0,953305492	5,615384615
B3GNT8	0,002263143	0,068988504	0,062	0,004	1	1,048980969	0,953306142	15,5
CALM3	0,005935912	0,06897016	0,469	0,195	1	1,048967631	0,953318263	2,405128205
TBC1D5	0,000202883	0,068828083	0,375	0,105	1	1,048864333	0,953412151	3,571428571
RNF19B	0,001968597	0,068702634	0,156	0,031	1	1,048773134	0,953495058	5,032258065
RAD1	0,001560165	0,068669316	0,125	0,02	1	1,048748914	0,953517078	6,25
FAP	6,3574E-05	0,068605064	0,062	0	1	1,048702207	0,953559546	#DIV/0!
STARD4	4,80439E-05	0,068598415	0,094	0,004	1	1,048697374	0,95356394	23,5
VRK1	0,004087528	0,068524129	0,125	0,023	1	1,048643377	0,953613042	5,434782609
IDE	0,001600673	0,068524036	0,125	0,02	1	1,04864331	0,953613103	6,25
MARS1	2,08038E-05	0,068478158	0,188	0,023	0,438980454	1,048609963	0,953643429	8,173913043
MFAP3	0,040857201	0,068475278	0,062	0,012	1	1,048607869	0,953645332	5,166666667
SERPINB1	0,000634905	0,068468501	0,281	0,074	1	1,048602944	0,953649812	3,797297297
CLCN3	0,001284108	0,068448091	0,344	0,109	1	1,048588109	0,953663303	3,155963303
TAF6L	0,000524345	0,068173021	0,094	0,008	1	1,0483882	0,95384515	11,75
RPF2	0,000174506	0,068160596	0,375	0,102	1	1,048379171	0,953853365	3,676470588
UBE2J2	0,00052751	0,068149416	0,375	0,113	1	1,048371047	0,953860757	3,318584071
GMDS	0,002594281	0,068104665	0,094	0,012	1	1,048338528	0,953890345	7,833333333
RNF14	6,60944E-05	0,068092568	0,188	0,027	1	1,048329737	0,953898344	6,962962963
ABHD15	0,007947097	0,06800476	0,094	0,016	1	1,048265934	0,953956403	5,875
AL137026.1	0,002594281	0,067959592	0,094	0,012	1	1,048233116	0,95398627	7,833333333
USP31	0,008148126	0,067959463	0,094	0,016	1	1,048233022	0,953986356	5,875
ABCF2	0,00010081	0,0678413	0,219	0,039	1	1,048147171	0,954064494	5,615384615
SPNS1	1,48343E-05	0,067684603	0,281	0,051	0,313018834	1,048033333	0,954168125	5,509803922
ASMTL	0,000390872	0,067574449	0,281	0,07	1	1,047953316	0,954240981	4,014285714
ZNF384	1,41516E-05	0,067489375	0,219	0,031	0,298613258	1,047891521	0,954297253	7,064516129

Supplementary Table 1

Differentially expressed genes_adjacent-skin-XL_ comparing SC-Keloid vs. (SC-Myel, SC-Nonmyel)								
**gene**	**p_val**	**avg_log2FC**	**pct.1**	**pct.2**	**p_val_adj**	**Foldchange_UP**	**Foldchange_DOWN**	**Ratio_pct1_pct2**
BCO2	0,082317662	0,067467802	0,031	0,004	1	1,047875852	0,954311523	7,75
MARCHF9	0,000818029	0,067393042	0,25	0,062	1	1,047821552	0,954360976	4,032258065
BGLAP	9,12354E-07	0,067291141	0,094	0	0,019251588	1,047747545	0,954428387	#DIV/0!
SZRD1	0,000266861	0,067253391	0,5	0,16	1	1,04772013	0,954453362	3,125
RAB6C	0,002263143	0,067131109	0,062	0,004	1	1,047631329	0,954534264	15,5
PTHLH	0,00050526	0,067096974	0,094	0,008	1	1,047606542	0,95455685	11,75
CCT2	0,003509282	0,067009636	0,656	0,277	1	1,047543124	0,954614638	2,368231047
AHCYL2	0,001560165	0,066992871	0,125	0,02	1	1,047530951	0,954625731	6,25
LINC00167	0,082317662	0,066983982	0,031	0,004	1	1,047524497	0,954631613	7,75
UBXN1	0,038714022	0,066969453	0,625	0,316	1	1,047513947	0,954641227	1,977848101
LYSMD1	4,80439E-05	0,066943572	0,094	0,004	1	1,047495156	0,954658353	23,5
CPNE1	0,001013249	0,06692401	0,5	0,18	1	1,047480952	0,954671298	2,777777778
AIDA	0,025898836	0,066865089	0,281	0,117	1	1,047438173	0,954710288	2,401709402
DIPK1A	0,082317662	0,066827166	0,031	0,004	1	1,047410641	0,954735384	7,75
AP1G2	0,002263143	0,066804126	0,062	0,004	1	1,047393913	0,954750632	15,5
KRT6A	0,018436147	0,066752597	0,094	0,02	1	1,047356504	0,954784733	4,7
KLF11	0,000881541	0,066729475	0,219	0,051	1	1,047339718	0,954800035	4,294117647
HOXB6	9,12354E-07	0,066676114	0,094	0	0,019251588	1,047300981	0,954835351	#DIV/0!
ACAD8	0,000339359	0,066670202	0,156	0,023	1	1,047296689	0,954839264	6,782608696
RAC3	3,67695E-07	0,066646528	0,156	0,008	0,007758733	1,047279503	0,954854933	19,5
CCDC92	0,000412046	0,066620571	0,25	0,059	1	1,047260661	0,954872112	4,237288136
PROCR	9,12354E-07	0,06660197	0,094	0	0,019251588	1,047247159	0,954884424	#DIV/0!
MFAP1	0,003191522	0,066500018	0,188	0,047	1	1,047173155	0,954951906	4
EIF3B	5,27955E-05	0,066495756	0,469	0,133	1	1,047170061	0,954954727	3,526315789
XKR8	6,3574E-05	0,06642765	0,062	0	1	1,047120628	0,954999809	#DIV/0!
LKAAEAR1	6,3574E-05	0,066422091	0,062	0	1	1,047116594	0,955003489	#DIV/0!
DHRS1	0,013145712	0,066384107	0,188	0,059	1	1,047089025	0,955028633	3,186440678
ZNF577	5,12603E-06	0,066291032	0,188	0,02	0,108164307	1,047021474	0,955090248	9,4
KIF11	0,002263143	0,066272423	0,062	0,004	1	1,047007969	0,955102568	15,5
LRRC20	1,51098E-05	0,066190611	0,125	0,008	0,318831333	1,046948597	0,955156731	15,625

Supplementary Table 1

Differentially expressed genes_adjacent-skin-XL_ comparing SC-Keloid vs. (SC-Myel, SC-Nonmyel)								
**gene**	**p_val**	**avg_log2FC**	**pct.1**	**pct.2**	**p_val_adj**	**Foldchange_UP**	**Foldchange_DOWN**	**Ratio_pct1_pct2**
CNOT11	0,000509339	0,06609672	0,125	0,016	1	1,046880464	0,955218895	7,8125
LINC00839	9,06462E-07	0,066032404	0,125	0,004	0,019127258	1,046833794	0,95526148	31,25
CPE	0,210023432	0,066026129	0,062	0,023	1	1,046829241	0,955265635	2,695652174
POU5F2	0,082317662	0,06597914	0,031	0,004	1	1,046795146	0,955296749	7,75
C5orf63	6,3574E-05	0,065925372	0,062	0	1	1,046756134	0,955332353	#DIV/0!
PLAGL1	0,002594281	0,065824889	0,094	0,012	1	1,04668323	0,955398893	7,833333333
RPUSD1	2,40115E-05	0,06581748	0,156	0,016	0,506667402	1,046677855	0,9554038	9,75
STRAP	0,024641865	0,065607803	0,562	0,277	1	1,046525745	0,955542666	2,028880866
MMP1	0,013891036	0,06560159	0,062	0,008	1	1,046521238	0,95554678	7,75
ACP6	0,000103236	0,065576501	0,156	0,02	1	1,046503039	0,955563398	7,8
ACACA	0,002053304	0,065509995	0,156	0,031	1	1,046454798	0,955607449	5,032258065
FICD	0,001642148	0,065436818	0,125	0,02	1	1,04640172	0,955655921	6,25
TMEM154	0,002263143	0,065420396	0,062	0,004	1	1,04638981	0,955666799	15,5
IWS1	0,000931266	0,065386423	0,219	0,051	1	1,046365169	0,955689304	4,294117647
ZNF195	0,000103236	0,065285703	0,156	0,02	1	1,046292121	0,955756026	7,8
DMXL2	0,00399666	0,065197473	0,125	0,023	1	1,046228136	0,955814479	5,434782609
NSUN2	0,000237136	0,065165148	0,219	0,043	1	1,046204694	0,955835895	5,093023256
GRK6	4,123E-05	0,065125819	0,219	0,035	0,869994633	1,046176174	0,955861952	6,257142857
KRT77	0,082317662	0,065059146	0,031	0,004	1	1,046127827	0,955906127	7,75
HYKK	0,008148126	0,064994349	0,094	0,016	1	1,046080842	0,955949062	5,875
SHKBP1	5,05415E-06	0,064991084	0,281	0,047	0,106647708	1,046078475	0,955951225	5,978723404
AGRN	0,000257484	0,064928935	0,594	0,211	1	1,046033413	0,955992407	2,815165877
CHUK	0,00097389	0,06481087	0,188	0,039	1	1,045947813	0,956070645	4,820512821
CASP6	0,002594281	0,064769811	0,094	0,012	1	1,045918045	0,956097856	7,833333333
FZD1	0,084624339	0,064764845	0,062	0,016	1	1,045914445	0,956101146	3,875
ZNF44	0,002594281	0,064730223	0,094	0,012	1	1,045889345	0,956124092	7,833333333
ZNF350	0,000524345	0,06469612	0,094	0,008	1	1,045864622	0,956146693	11,75
ASB7	0,061916284	0,064619463	0,094	0,027	1	1,045809052	0,956197499	3,481481481
B4GALT3	0,000252542	0,064617365	0,219	0,043	1	1,045807532	0,956198889	5,093023256
ZMYND10	0,082317662	0,064376554	0,031	0,004	1	1,045632982	0,956358509	7,75

Supplementary Table 1

Differentially expressed genes_adjacent-skin-XL_ comparing SC-Keloid vs. (SC-Myel, SC-Nonmyel)								
**gene**	**p_val**	**avg_log2FC**	**pct.1**	**pct.2**	**p_val_adj**	**Foldchange_UP**	**Foldchange_DOWN**	**Ratio_pct1_pct2**
AP3B1	4,24942E-06	0,064372342	0,344	0,066	0,089667107	1,04562993	0,956361301	5,212121212
TIAL1	0,007349966	0,064289168	0,469	0,195	1	1,045569649	0,956416439	2,405128205
SLC2A11	0,00399666	0,064196379	0,125	0,023	1	1,045502404	0,956477954	5,434782609
AACS	0,086278754	0,063944878	0,062	0,016	1	1,04532016	0,956644708	3,875
VEGFC	6,3574E-05	0,063886901	0,062	0	1	1,045278153	0,956683154	#DIV/0!
ZNF581	0,00010081	0,063852321	0,219	0,039	1	1,045253099	0,956706085	5,615384615
DDI2	0,041443265	0,063784514	0,125	0,039	1	1,045203973	0,956751051	3,205128205
B4GALNT3	1,11477E-07	0,063776781	0,188	0,012	0,002352284	1,045198371	0,95675618	15,66666667
ANKRD40	0,000124121	0,063640943	0,531	0,168	1	1,045099964	0,956846267	3,160714286
BTD	0,001666138	0,0635519	0,219	0,055	1	1,045035462	0,956905326	3,981818182
GTF2IRD2	0,142206469	0,063526599	0,125	0,055	1	1,045017136	0,956922108	2,272727273
MNAT1	0,000117065	0,063378667	0,312	0,074	1	1,044909986	0,957020234	4,216216216
MRPS18B	0,095443971	0,063327331	0,219	0,105	1	1,044872806	0,957054289	2,085714286
PRKRIP1	0,00950559	0,063291407	0,219	0,07	1	1,044846788	0,95707812	3,128571429
NUDC	0,005706333	0,063280148	0,594	0,266	1	1,044838634	0,95708559	2,233082707
ABR	1,47837E-05	0,063270936	0,312	0,062	0,311950941	1,044831962	0,957091701	5,032258065
EXTL1	0,060931358	0,06315322	0,094	0,027	1	1,044746714	0,957169797	3,481481481
TBC1D23	0,023239872	0,063144616	0,25	0,098	1	1,044740482	0,957175506	2,551020408
APOBEC3F	0,002594281	0,063123751	0,094	0,012	1	1,044725373	0,957189349	7,833333333
ALK	0,226351275	0,063007993	0,031	0,008	1	1,04464155	0,957266155	3,875
UFSP2	2,82528E-05	0,062978546	0,562	0,16	0,596163152	1,044620228	0,957285694	3,5125
NDUFV2	0,000327108	0,062937876	0,594	0,211	1	1,04459078	0,95731268	2,815165877
MYD88	4,80439E-05	0,062924503	0,094	0,004	1	1,044581098	0,957321554	23,5
GEMIN2	7,15271E-05	0,062880594	0,188	0,027	1	1,044549306	0,957350691	6,962962963
BCL10	0,000408355	0,062837465	0,344	0,098	1	1,04451808	0,957379311	3,510204082
AC022182.1	0,004841742	0,062737938	0,031	0	1	1,044446025	0,95744536	#DIV/0!
CABLES1	0,004841742	0,062737938	0,031	0	1	1,044446025	0,95744536	#DIV/0!
CDH4	0,004841742	0,062737938	0,031	0	1	1,044446025	0,95744536	#DIV/0!
GABRR2	0,004841742	0,062737938	0,031	0	1	1,044446025	0,95744536	#DIV/0!
GASK1A	0,004841742	0,062737938	0,031	0	1	1,044446025	0,95744536	#DIV/0!

Supplementary Table 1

Differentially expressed genes_adjacent-skin-XL_ comparing SC-Keloid vs. (SC-Myel, SC-Nonmyel)								
**gene**	**p_val**	**avg_log2FC**	**pct.1**	**pct.2**	**p_val_adj**	**Foldchange_UP**	**Foldchange_DOWN**	**Ratio_pct1_pct2**
KCTD14	0,004841742	0,062737938	0,031	0	1	1,044446025	0,95744536	#DIV/0!
LINC00240	0,004841742	0,062737938	0,031	0	1	1,044446025	0,95744536	#DIV/0!
SHISA3	0,004841742	0,062737938	0,031	0	1	1,044446025	0,95744536	#DIV/0!
ACAA1	6,599E-05	0,062704123	0,344	0,082	1	1,044421544	0,957467802	4,195121951
ZNF493	0,02693544	0,062605901	0,125	0,035	1	1,04435044	0,95753299	3,571428571
TTC3	0,000329944	0,062495723	0,781	0,293	1	1,044270686	0,95760612	2,66552901
MTCL1	6,3574E-05	0,06246842	0,062	0	1	1,044250924	0,957624242	#DIV/0!
DGKE	0,662254058	0,062461724	0,031	0,02	1	1,044246078	0,957628687	1,55
TMEM249	0,082317662	0,062404204	0,031	0,004	1	1,044204444	0,957666868	7,75
OSBPL7	0,226917872	0,062344547	0,094	0,043	1	1,044161266	0,95770647	2,186046512
ARCN1	1,79207E-05	0,062193773	0,438	0,109	0,37814398	1,044052148	0,957806563	4,018348624
SLC25A44	0,886800848	0,0621274	0,031	0,027	1	1,044004116	0,95785063	1,148148148
L1TD1	0,079594089	0,062122434	0,031	0,004	1	1,044000522	0,957853927	7,75
INSIG1	0,00041116	0,062085911	0,281	0,07	1	1,043974093	0,957878176	4,014285714
SLC22A18	3,33107E-06	0,062066501	0,25	0,035	0,070289013	1,043960047	0,957891064	7,142857143
ST3GAL6	0,003034006	0,062032908	0,312	0,105	1	1,043935739	0,957913368	2,971428571
DPH5	0,000100263	0,061998175	0,156	0,02	1	1,043910607	0,95793643	7,8
KLHL5	0,285925963	0,061996162	0,094	0,047	1	1,04390915	0,957937767	2
EEF2KMT	0,001600673	0,061959757	0,125	0,02	1	1,043882809	0,957961939	6,25
SNX11	0,215833268	0,061936957	0,062	0,023	1	1,043866311	0,957977079	2,695652174
S100A3	0,000509339	0,061912537	0,125	0,016	1	1,043848642	0,957993295	7,8125
NUDT19	0,002594281	0,061894078	0,094	0,012	1	1,043835286	0,958005552	7,833333333
RAB24	0,042049888	0,061785025	0,125	0,039	1	1,043756387	0,95807797	3,205128205
SMYD3	0,004107327	0,061759761	0,156	0,035	1	1,043738109	0,958094748	4,457142857
PPHLN1	0,000408355	0,061732146	0,344	0,098	1	1,04371813	0,958113087	3,510204082
TMBIM6	0,127999557	0,061714793	0,875	0,543	1	1,043705576	0,958124612	1,611418048
NDFIP2	3,73485E-05	0,061650478	0,438	0,117	0,788091641	1,04365905	0,958167325	3,743589744
MCC	5,12603E-06	0,061587288	0,188	0,02	0,108164307	1,043613338	0,958209294	9,4
NAA40	0,013891036	0,061504046	0,062	0,008	1	1,043553124	0,958264583	7,75
SHISAL1	6,3574E-05	0,061445571	0,062	0	1	1,043510828	0,958303424	#DIV/0!

Supplementary Table 1

Differentially expressed genes_adjacent-skin-XL_ comparing SC-Keloid vs. (SC-Myel, SC-Nonmyel)								
**gene**	**p_val**	**avg_log2FC**	**pct.1**	**pct.2**	**p_val_adj**	**Foldchange_UP**	**Foldchange_DOWN**	**Ratio_pct1_pct2**
ARNTL	0,00844871	0,061396305	0,188	0,055	1	1,043475194	0,95833615	3,418181818
ISOC2	0,000851711	0,061327711	0,375	0,121	1	1,043425583	0,958381715	3,099173554
MAP3K7CL	0,084624339	0,061324342	0,062	0,016	1	1,043423146	0,958383954	3,875
PARP11	0,084624339	0,061308814	0,062	0,016	1	1,043411916	0,958394269	3,875
SGTA	0,00862775	0,061213664	0,25	0,086	1	1,043343102	0,95845748	2,906976744
DALRD3	0,004267025	0,061203831	0,156	0,035	1	1,043335991	0,958464012	4,457142857
KLHL11	0,013891036	0,0611933	0,062	0,008	1	1,043328375	0,958471009	7,75
MOAP1	0,003518189	0,061154693	0,25	0,074	1	1,043300456	0,958496658	3,378378378
TESK2	0,145431	0,061148153	0,062	0,02	1	1,043295726	0,958501003	3,1
AP1S3	0,004841742	0,061138222	0,031	0	1	1,043288544	0,958507601	#DIV/0!
CDC25A	0,004841742	0,061138222	0,031	0	1	1,043288544	0,958507601	#DIV/0!
CDC6	0,004841742	0,061138222	0,031	0	1	1,043288544	0,958507601	#DIV/0!
KCNH1	0,004841742	0,061138222	0,031	0	1	1,043288544	0,958507601	#DIV/0!
LINC00028	0,004841742	0,061138222	0,031	0	1	1,043288544	0,958507601	#DIV/0!
LINC01561	0,004841742	0,061138222	0,031	0	1	1,043288544	0,958507601	#DIV/0!
LRRC56	0,004841742	0,061138222	0,031	0	1	1,043288544	0,958507601	#DIV/0!
NRIP3	0,004841742	0,061138222	0,031	0	1	1,043288544	0,958507601	#DIV/0!
POLQ	0,004841742	0,061138222	0,031	0	1	1,043288544	0,958507601	#DIV/0!
TMEM171	0,004841742	0,061138222	0,031	0	1	1,043288544	0,958507601	#DIV/0!
ZNF730	0,004841742	0,061138222	0,031	0	1	1,043288544	0,958507601	#DIV/0!
PALB2	0,002263143	0,061137636	0,062	0,004	1	1,043288121	0,95850799	15,5
SYNGR1	0,000113517	0,061133327	0,562	0,18	1	1,043285005	0,958510853	3,122222222
SPC24	6,3574E-05	0,061114705	0,062	0	1	1,043271538	0,958523225	#DIV/0!
EIF2S1	0,001211009	0,061094379	0,312	0,094	1	1,04325684	0,95853673	3,319148936
TTC12	0,013891036	0,061046264	0,062	0,008	1	1,043222047	0,958568699	7,75
STN1	0,000524345	0,061037431	0,094	0,008	1	1,043215659	0,958574568	11,75
ZNF35	0,082317662	0,060955854	0,031	0,004	1	1,043156673	0,958628772	7,75
BNIP2	0,001210407	0,060954397	0,438	0,152	1	1,043155619	0,95862974	2,881578947
PGPEP1	0,004029529	0,060918538	0,156	0,035	1	1,043129691	0,958653567	4,457142857
CDCA2	0,002263143	0,060829829	0,062	0,004	1	1,043065553	0,958712515	15,5

Supplementary Table 1

Differentially expressed genes_adjacent-skin-XL_ comparing SC-Keloid vs. (SC-Myel, SC-Nonmyel)								
**gene**	**p_val**	**avg_log2FC**	**pct.1**	**pct.2**	**p_val_adj**	**Foldchange_UP**	**Foldchange_DOWN**	**Ratio_pct1_pct2**
ZBTB41	0,004760864	0,060781738	0,25	0,078	1	1,043030784	0,958744474	3,205128205
ANKRD39	2,0222E-06	0,060762371	0,281	0,043	0,042670449	1,043016782	0,958757344	6,534883721
GPR89A	2,1417E-05	0,06064783	0,188	0,023	0,451919526	1,042933976	0,958833467	8,173913043
ZNF480	0,000524345	0,060534611	0,094	0,008	1	1,042852133	0,958908716	11,75
SPOP	0,000198951	0,06052186	0,406	0,117	1	1,042842916	0,958917192	3,47008547
GSDME	0,013891036	0,060483107	0,062	0,008	1	1,042814904	0,95894295	7,75
FBXO4	0,040844069	0,060451168	0,125	0,039	1	1,042791818	0,95896418	3,205128205
COMP	0,226351275	0,060440531	0,031	0,008	1	1,04278413	0,95897125	3,875
PLRG1	0,00023142	0,060394276	0,281	0,066	1	1,042750696	0,959001997	4,257575758
PCDHGB6	0,002442928	0,060367215	0,094	0,012	1	1,042731138	0,959019985	7,833333333
BRCA2	0,002263143	0,060233227	0,062	0,004	1	1,0426343	0,959109057	15,5
CENPE	0,013891036	0,060227346	0,062	0,008	1	1,04263005	0,959112966	7,75
TMEM18	0,023239872	0,060221767	0,25	0,098	1	1,042626018	0,959116676	2,551020408
AFAP1	0,041554521	0,060182386	0,156	0,055	1	1,042597558	0,959142856	2,836363636
CSNK1G1	0,001968597	0,060156532	0,156	0,031	1	1,042578874	0,959160045	5,032258065
SLC25A26	3,2432E-06	0,060145205	0,25	0,035	0,068434669	1,042570689	0,959167575	7,142857143
LTK	0,040857201	0,0601251	0,062	0,012	1	1,042556159	0,959180943	5,166666667
NSUN5	1,53257E-05	0,060114336	0,219	0,031	0,323387973	1,042548381	0,959188099	7,064516129
ATP6V1B1	6,3574E-05	0,060090646	0,062	0	1	1,042531262	0,95920385	#DIV/0!
LIPT2	6,3574E-05	0,060090646	0,062	0	1	1,042531262	0,95920385	#DIV/0!
MAX	0,025944296	0,060083412	0,375	0,168	1	1,042526035	0,959208659	2,232142857
HJURP	0,004841742	0,060078925	0,031	0	1	1,042522792	0,959211643	#DIV/0!
RACGAP1	0,004841742	0,060078925	0,031	0	1	1,042522792	0,959211643	#DIV/0!
TROAP	0,004841742	0,060078925	0,031	0	1	1,042522792	0,959211643	#DIV/0!
DAP	0,000135123	0,059984291	0,594	0,203	1	1,04245441	0,959274564	2,926108374
PCYOX1	0,000669326	0,059883439	0,344	0,102	1	1,042381539	0,959341625	3,37254902
CAMKK1	0,002263143	0,05983702	0,062	0,004	1	1,042348001	0,959372492	15,5
ACOX3	0,013891036	0,059825873	0,062	0,008	1	1,042339947	0,959379905	7,75
CREBBP	0,075628693	0,05980219	0,281	0,137	1	1,042322837	0,959395654	2,051094891
CDHR3	0,177883766	0,059761739	0,125	0,059	1	1,042293612	0,959422555	2,118644068

Supplementary Table 1

Differentially expressed genes_adjacent-skin-XL_ comparing SC-Keloid vs. (SC-Myel, SC-Nonmyel)								
**gene**	**p_val**	**avg_log2FC**	**pct.1**	**pct.2**	**p_val_adj**	**Foldchange_UP**	**Foldchange_DOWN**	**Ratio_pct1_pct2**
UCHL1	0,004507068	0,059731917	0,25	0,078	1	1,042272067	0,959442387	3,205128205
ZSCAN2	9,12354E-07	0,059727462	0,094	0	0,019251588	1,042268848	0,95944535	#DIV/0!
MGAT2	2,11157E-07	0,059677474	0,219	0,02	0,00445563	1,042232736	0,959478594	10,95
CCDC189	4,80439E-05	0,059657099	0,094	0,004	1	1,042218016	0,959492145	23,5
BFAR	0,000967853	0,059611786	0,344	0,105	1	1,042185282	0,959522282	3,276190476
TRIM16	0,000339359	0,059596654	0,156	0,023	1	1,042174351	0,959532346	6,782608696
AC009506.1	0,000524345	0,059568273	0,094	0,008	1	1,042153849	0,959551222	11,75
TNFAIP1	0,000103124	0,059514747	0,219	0,039	1	1,042115185	0,959586824	5,615384615
CILK1	0,000103124	0,059470621	0,219	0,039	1	1,042083311	0,959616174	5,615384615
CEP57	0,000466383	0,059418175	0,344	0,098	1	1,042045429	0,959651059	3,510204082
TSR1	0,000486986	0,059365543	0,188	0,035	1	1,042007414	0,95968607	5,371428571
HCG11	0,059959447	0,059357059	0,094	0,027	1	1,042001286	0,959691713	3,481481481
NEMF	0,001081512	0,059345988	0,312	0,094	1	1,04199329	0,959699078	3,319148936
KRBOX1	0,002594281	0,059279442	0,094	0,012	1	1,041945228	0,959743346	7,833333333
PRR14L	2,1417E-05	0,059257507	0,188	0,023	0,451919526	1,041929387	0,959757938	8,173913043
CPNE8	0,057775912	0,059215776	0,125	0,043	1	1,041899248	0,959785701	2,906976744
KRT80	6,3574E-05	0,059152451	0,062	0	1	1,041853517	0,95982783	#DIV/0!
FKBP15	0,000509339	0,059121195	0,125	0,016	1	1,041830945	0,959848625	7,8125
GTPBP6	0,000113241	0,059110714	0,438	0,125	1	1,041823377	0,959855598	3,504
TRAF2	0,004107327	0,059046775	0,156	0,035	1	1,041777205	0,959898139	4,457142857
LSM5	0,002529014	0,058834204	0,406	0,148	1	1,041623718	0,960039583	2,743243243
CSRNP3	0,002168448	0,058814111	0,062	0,004	1	1,041609211	0,960052954	15,5
EIF2S3	2,2573E-05	0,058746343	0,625	0,195	0,476312484	1,041560284	0,960098052	3,205128205
MRPL1	0,001637922	0,058578021	0,219	0,055	1	1,04143877	0,960210075	3,981818182
RILPL1	0,001555893	0,05848588	0,219	0,055	1	1,041372259	0,960271403	3,981818182
DARS1	0,01976798	0,058437201	0,219	0,078	1	1,041337122	0,960303805	2,807692308
IFI6	0,0012511	0,05835059	0,719	0,305	1	1,041274608	0,960361458	2,357377049
EDN2	0,004841742	0,058329758	0,031	0	1	1,041259572	0,960375325	#DIV/0!
SRGAP2-AS1	0,004841742	0,058329758	0,031	0	1	1,041259572	0,960375325	#DIV/0!
LRCH4	0,089433293	0,058328283	0,094	0,031	1	1,041258507	0,960376307	3,032258065

Supplementary Table 1

Differentially expressed genes_adjacent-skin-XL_ comparing SC-Keloid vs. (SC-Myel, SC-Nonmyel)								
**gene**	**p_val**	**avg_log2FC**	**pct.1**	**pct.2**	**p_val_adj**	**Foldchange_UP**	**Foldchange_DOWN**	**Ratio_pct1_pct2**
LETM2	0,004841742	0,058134176	0,031	0	1	1,041118421	0,960505529	#DIV/0!
NRG2	0,004841742	0,058134176	0,031	0	1	1,041118421	0,960505529	#DIV/0!
OAZ3	0,004841742	0,058134176	0,031	0	1	1,041118421	0,960505529	#DIV/0!
RIN3	0,004841742	0,058134176	0,031	0	1	1,041118421	0,960505529	#DIV/0!
GRIPAP1	0,000537931	0,05812826	0,219	0,047	1	1,041114152	0,960509468	4,659574468
TCP10L	0,002263143	0,058064566	0,062	0,004	1	1,041068188	0,960551875	15,5
CENATAC	0,022031631	0,058042734	0,25	0,098	1	1,041052434	0,960566411	2,551020408
ZC3HAV1L	0,013891036	0,057915908	0,062	0,008	1	1,04096092	0,960650857	7,75
ADCK1	9,12354E-07	0,057884458	0,094	0	0,019251588	1,040938228	0,960671799	#DIV/0!
FUNDC1	0,019236133	0,05771312	0,094	0,02	1	1,040814611	0,960785897	4,7
TPRA1	0,003191522	0,057696601	0,188	0,047	1	1,040802694	0,960796899	4
NCK1	0,001177356	0,05767649	0,312	0,094	1	1,040788185	0,960810292	3,319148936
RFWD3	0,000494525	0,057522753	0,125	0,016	1	1,040677282	0,960912684	7,8125
RNF144B	0,380266129	0,057472266	0,031	0,012	1	1,040640864	0,960946311	2,583333333
ZNF658	6,3574E-05	0,057424925	0,062	0	1	1,040606717	0,960977844	#DIV/0!
KRT8	0,082317662	0,057403086	0,031	0,004	1	1,040590965	0,960992392	7,75
TMA16	4,32987E-05	0,057346863	0,219	0,035	0,913646714	1,040550413	0,961029843	6,257142857
RNF170	0,008780679	0,057280975	0,125	0,027	1	1,040502892	0,961073734	4,62962963
ZNF689	4,80439E-05	0,057007297	0,094	0,004	1	1,040305528	0,961256067	23,5
PITPNC1	0,000509339	0,056849224	0,125	0,016	1	1,04019155	0,961361395	7,8125
MAMDC4	0,002263143	0,056826218	0,062	0,004	1	1,040174963	0,961376726	15,5
ABCB6	0,040857201	0,056825331	0,062	0,012	1	1,040174324	0,961377317	5,166666667
DEXI	7,04762E-05	0,056815157	0,312	0,07	1	1,040166988	0,961384096	4,457142857
SAC3D1	0,000112833	0,056756793	0,125	0,012	1	1,040124909	0,96142299	10,41666667
POLD1	0,013891036	0,056706981	0,062	0,008	1	1,040088998	0,961456185	7,75
KLHL2	0,004087481	0,056694826	0,125	0,023	1	1,040080234	0,961464286	5,434782609
SASS6	0,004029529	0,056602159	0,156	0,035	1	1,04001343	0,961526045	4,457142857
ACTR3	0,005699711	0,056589748	0,531	0,223	1	1,040004484	0,961534316	2,381165919
TFCP2	0,278079807	0,05648718	0,094	0,047	1	1,039930548	0,961602679	2
CHAF1A	3,67695E-07	0,056422218	0,156	0,008	0,007758733	1,039883723	0,961645979	19,5

Supplementary Table 1

Differentially expressed genes_adjacent-skin-XL_ comparing SC-Keloid vs. (SC-Myel, SC-Nonmyel)								
**gene**	**p_val**	**avg_log2FC**	**pct.1**	**pct.2**	**p_val_adj**	**Foldchange_UP**	**Foldchange_DOWN**	**Ratio_pct1_pct2**
IL32	0,000322156	0,056361261	0,156	0,023	1	1,039839785	0,961686612	6,782608696
TMEM11	0,000126424	0,056341647	0,25	0,051	1	1,039825649	0,961699686	4,901960784
UTP15	0,000524345	0,056331341	0,094	0,008	1	1,03981822	0,961706556	11,75
ZNF781	0,000494525	0,056315185	0,125	0,016	1	1,039806576	0,961717326	7,8125
PGAP2	0,000509339	0,05630413	0,125	0,016	1	1,039798609	0,961724695	7,8125
ETFB	0,006569644	0,056251614	0,406	0,16	1	1,039760759	0,961759704	2,5375
OSTF1	0,001267483	0,056223697	0,531	0,191	1	1,039740639	0,961778315	2,780104712
GEN1	0,000112833	0,05618019	0,125	0,012	1	1,039709285	0,961807319	10,41666667
TMEM138	0,000509339	0,056151119	0,125	0,016	1	1,039688335	0,9618267	7,8125
PIGB	0,002594281	0,056140658	0,094	0,012	1	1,039680795	0,961833675	7,833333333
CDCA4	0,013891036	0,056116247	0,062	0,008	1	1,039663204	0,961849949	7,75
THAP1	9,17361E-07	0,056109086	0,188	0,016	0,019357231	1,039658044	0,961854723	11,75
THRAP3	2,5478E-05	0,056053079	0,562	0,164	0,537612072	1,039617684	0,961892064	3,426829268
AMT	0,147778936	0,056007027	0,062	0,02	1	1,039584499	0,961922769	3,1
PCGF5	0,255362483	0,055921677	0,281	0,176	1	1,039522999	0,961979679	1,596590909
PTK6	0,082317662	0,055884154	0,031	0,004	1	1,039495962	0,962004699	7,75
SLC2A4RG	1,96669E-05	0,055858202	0,531	0,148	0,414992103	1,039477263	0,962022004	3,587837838
METTL16	0,020463101	0,055842699	0,156	0,047	1	1,039466093	0,962032342	3,319148936
WDR91	0,145431	0,055810735	0,062	0,02	1	1,039443063	0,962053657	3,1
RABGGTA	0,001968597	0,055718533	0,156	0,031	1	1,039376635	0,962115143	5,032258065
RAD51AP1	1,51098E-05	0,055579483	0,125	0,008	0,318831333	1,039276463	0,962207878	15,625
BATF3	0,002594281	0,055549084	0,094	0,012	1	1,039254564	0,962228153	7,833333333
SLC37A1	0,000524345	0,05552371	0,094	0,008	1	1,039236286	0,962245077	11,75
AGBL2	0,004841742	0,055500237	0,031	0	1	1,039219378	0,962260733	#DIV/0!
AJM1	0,004841742	0,055500237	0,031	0	1	1,039219378	0,962260733	#DIV/0!
ATP13A4	0,004841742	0,055500237	0,031	0	1	1,039219378	0,962260733	#DIV/0!
COL5A1-AS1	0,004841742	0,055500237	0,031	0	1	1,039219378	0,962260733	#DIV/0!
DCTN1-AS1	0,004841742	0,055500237	0,031	0	1	1,039219378	0,962260733	#DIV/0!
LINC00393	0,004841742	0,055500237	0,031	0	1	1,039219378	0,962260733	#DIV/0!
LRRC46	0,004841742	0,055500237	0,031	0	1	1,039219378	0,962260733	#DIV/0!

Supplementary Table 1

Differentially expressed genes_adjacent-skin-XL_ comparing SC-Keloid vs. (SC-Myel, SC-Nonmyel)								
**gene**	**p_val**	**avg_log2FC**	**pct.1**	**pct.2**	**p_val_adj**	**Foldchange_UP**	**Foldchange_DOWN**	**Ratio_pct1_pct2**
MC5R	0,004841742	0,055500237	0,031	0	1	1,039219378	0,962260733	#DIV/0!
MGAT5B	0,004841742	0,055500237	0,031	0	1	1,039219378	0,962260733	#DIV/0!
PCDHGA11	0,004841742	0,055500237	0,031	0	1	1,039219378	0,962260733	#DIV/0!
RLBP1	0,004841742	0,055500237	0,031	0	1	1,039219378	0,962260733	#DIV/0!
SLC1A1	0,004841742	0,055500237	0,031	0	1	1,039219378	0,962260733	#DIV/0!
ARHGEF33	0,004841742	0,055445626	0,031	0	1	1,03918004	0,962297159	#DIV/0!
CCNO	0,004841742	0,055445626	0,031	0	1	1,03918004	0,962297159	#DIV/0!
LINC01549	0,004841742	0,055445626	0,031	0	1	1,03918004	0,962297159	#DIV/0!
MMP16	0,004841742	0,055445626	0,031	0	1	1,03918004	0,962297159	#DIV/0!
NDST3	0,004841742	0,055445626	0,031	0	1	1,03918004	0,962297159	#DIV/0!
NME5	0,004841742	0,055445626	0,031	0	1	1,03918004	0,962297159	#DIV/0!
ZBTB3	0,004841742	0,055445626	0,031	0	1	1,03918004	0,962297159	#DIV/0!
ZNRF3-AS1	0,004841742	0,055445626	0,031	0	1	1,03918004	0,962297159	#DIV/0!
FBXL17	0,083160445	0,05543421	0,125	0,047	1	1,039171817	0,962304773	2,659574468
SLC4A1AP	0,000107903	0,05535192	0,219	0,039	1	1,039112546	0,962359664	5,615384615
OGFR	0,024369817	0,055160042	0,344	0,152	1	1,038974353	0,962487666	2,263157895
GON7	0,000509339	0,055153434	0,125	0,016	1	1,038969594	0,962492075	7,8125
JOSD1	0,001318433	0,055116797	0,344	0,109	1	1,03894321	0,962516517	3,155963303
SEC22C	0,000509247	0,055056727	0,344	0,098	1	1,038899952	0,962556595	3,510204082
SLC19A1	0,013891036	0,0550237	0,062	0,008	1	1,038876169	0,96257863	7,75
AC002454.1	6,3574E-05	0,054999204	0,062	0	1	1,03885853	0,962594974	#DIV/0!
AMBRA1	0,899307354	0,054991886	0,031	0,027	1	1,03885326	0,962599857	1,148148148
NCDN	0,000112833	0,054881383	0,125	0,012	1	1,038773693	0,96267359	10,41666667
ABHD4	0,000339359	0,054836758	0,156	0,023	1	1,038741563	0,962703367	6,782608696
SNRPN	0,000463482	0,054783378	0,75	0,281	1	1,03870313	0,962738988	2,669039146
ZNF75A	0,018832425	0,054754571	0,094	0,02	1	1,03868239	0,962758212	4,7
HOXB-AS1	0,143670631	0,054659592	0,125	0,055	1	1,03861401	0,962821597	2,272727273
DDX39B	0,007060784	0,054619009	0,719	0,328	1	1,038584794	0,962848682	2,192073171
SRRD	0,000486986	0,054617963	0,188	0,035	1	1,038584042	0,962849379	5,371428571
PPIE	9,73764E-06	0,054614288	0,375	0,082	0,205473989	1,038581396	0,962851832	4,573170732

Supplementary Table 1

Differentially expressed genes_adjacent-skin-XL_ comparing SC-Keloid vs. (SC-Myel, SC-Nonmyel)								
**gene**	**p_val**	**avg_log2FC**	**pct.1**	**pct.2**	**p_val_adj**	**Foldchange_UP**	**Foldchange_DOWN**	**Ratio_pct1_pct2**
ARSK	0,226917872	0,054605566	0,094	0,043	1	1,038575117	0,962857653	2,186046512
IFT81	0,001013976	0,054572766	0,188	0,039	1	1,038551505	0,962879544	4,820512821
CASTOR2	0,008956837	0,054531657	0,125	0,027	1	1,038521913	0,962906981	4,62962963
KLHL29	0,000881541	0,054527424	0,219	0,051	1	1,038518866	0,962909807	4,294117647
ZNF227	0,000509339	0,054484742	0,125	0,016	1	1,038488141	0,962938295	7,8125
PSMC3IP	0,147778936	0,054414324	0,062	0,02	1	1,038437454	0,962985297	3,1
FKBP11	0,004186451	0,054321068	0,156	0,035	1	1,038370331	0,963047546	4,457142857
SLC35G1	0,221475664	0,054259305	0,031	0,008	1	1,038325879	0,963088777	3,875
DIS3L	0,000100263	0,054216248	0,156	0,02	1	1,038294891	0,96311752	7,8
FAM117A	0,013891036	0,054197933	0,062	0,008	1	1,038281709	0,963129747	7,75
PPIF	0,287158009	0,054186509	0,062	0,027	1	1,038273488	0,963137374	2,296296296
EMILIN2	0,018436147	0,054127289	0,094	0,02	1	1,03823087	0,963176909	4,7
ZNF19	0,533973819	0,054118302	0,031	0,016	1	1,038224402	0,963182909	1,9375
HEATR3	0,29055378	0,05411404	0,062	0,027	1	1,038221335	0,963185754	2,296296296
AGER	0,002517564	0,054084104	0,094	0,012	1	1,038199792	0,963205741	7,833333333
ACSL4	7,5014E-06	0,05407657	0,312	0,059	0,158287075	1,038194371	0,963210771	5,288135593
CAD	0,001600673	0,054064068	0,125	0,02	1	1,038185374	0,963219118	6,25
RESF1	0,177416998	0,053878345	0,094	0,039	1	1,038051734	0,963343124	2,41025641
WNK3	0,002594281	0,053533226	0,094	0,012	1	1,037803442	0,963573601	7,833333333
CTNNB1	0,006642536	0,053517657	0,844	0,398	1	1,037792243	0,963584	2,120603015
CTDP1	0,002594281	0,053459489	0,094	0,012	1	1,037750401	0,963622851	7,833333333
TSR2	0,061168803	0,053366887	0,25	0,113	1	1,037683793	0,963684705	2,212389381
CAND1	0,000319167	0,053366349	0,438	0,137	1	1,037683406	0,963685065	3,197080292
CCDC140	0,002263143	0,053293123	0,062	0,004	1	1,037630738	0,963733979	15,5
THAP9	0,082317662	0,053283164	0,031	0,004	1	1,037623576	0,963740632	7,75
CGRRF1	0,082895194	0,053281019	0,188	0,082	1	1,037622033	0,963742065	2,292682927
SAMD14	6,3574E-05	0,053096923	0,062	0	1	1,037489635	0,963865051	#DIV/0!
TLCD1	6,3574E-05	0,053096923	0,062	0	1	1,037489635	0,963865051	#DIV/0!
MESP1	0,002594281	0,052982743	0,094	0,012	1	1,037407527	0,963941338	7,833333333
MED20	4,80439E-05	0,052968605	0,094	0,004	1	1,037397361	0,963950784	23,5

Supplementary Table 1

Differentially expressed genes_adjacent-skin-XL_ comparing SC-Keloid vs. (SC-Myel, SC-Nonmyel)								
**gene**	**p_val**	**avg_log2FC**	**pct.1**	**pct.2**	**p_val_adj**	**Foldchange_UP**	**Foldchange_DOWN**	**Ratio_pct1_pct2**
PES1	7,15271E-05	0,052961619	0,188	0,027	1	1,037392338	0,963955452	6,962962963
FAM210A	0,035499775	0,052945104	0,094	0,023	1	1,037380462	0,963966487	4,086956522
RELCH	0,004180266	0,052892803	0,125	0,023	1	1,037342856	0,964001434	5,434782609
THAP12	6,58444E-07	0,052841564	0,281	0,039	0,013893836	1,037306014	0,964035672	7,205128205
C3orf52	0,01346698	0,052790547	0,062	0,008	1	1,037269333	0,964069763	7,75
PPAT	0,015701049	0,052753467	0,125	0,031	1	1,037242673	0,964094542	4,032258065
CELSR1	0,002263143	0,052749084	0,062	0,004	1	1,037239523	0,964097471	15,5
SPATA18	6,3574E-05	0,052715157	0,062	0	1	1,03721513	0,964120143	#DIV/0!
ACOT11	0,007750521	0,05258881	0,094	0,016	1	1,037124298	0,964204582	5,875
VARS2	0,007947097	0,052585159	0,094	0,016	1	1,037121674	0,964207022	5,875
RASL10A	0,079594089	0,052486514	0,031	0,004	1	1,037050762	0,964272952	7,75
CCDC80	2,6901E-06	0,052486231	0,531	0,137	0,056763861	1,037050559	0,964273141	3,875912409
XRCC2	0,082317662	0,052460405	0,031	0,004	1	1,037031995	0,964290403	7,75
MPI	9,06462E-07	0,052407319	0,125	0,004	0,019127258	1,036993836	0,964325886	31,25
ZNF587	0,079594089	0,052290932	0,031	0,004	1	1,036910182	0,964403685	7,75
EFNB1	0,007089636	0,052268455	0,156	0,039	1	1,036894027	0,96441871	4
GCLC	0,373006089	0,05212464	0,062	0,031	1	1,03679067	0,964514853	2
ABCE1	0,005187435	0,05210879	0,531	0,215	1	1,036779279	0,964525449	2,469767442
DLG2	0,937761989	0,052020562	0,031	0,035	1	1,036715877	0,964584437	0,885714286
METTL22	0,000103236	0,052002904	0,156	0,02	1	1,036703188	0,964596243	7,8
TRIM35	0,008148126	0,051897612	0,094	0,016	1	1,036627529	0,964666645	5,875
BICD2	0,000531863	0,051824745	0,312	0,086	1	1,036575173	0,964715369	3,627906977
ZNF596	0,013891036	0,051802215	0,062	0,008	1	1,036558985	0,964730435	7,75
CCNQ	0,007471523	0,051801061	0,156	0,039	1	1,036558156	0,964731206	4
PHF14	1,74332E-05	0,051749254	0,5	0,137	0,36785828	1,036520934	0,96476585	3,649635036
WDR31	0,013891036	0,0517387	0,062	0,008	1	1,036513352	0,964772908	7,75
SOX5	0,003896088	0,051723366	0,344	0,121	1	1,036502335	0,964783162	2,842975207
CCZ1	1,54378E-05	0,05167424	0,438	0,109	0,325753636	1,03646704	0,964816015	4,018348624
TMX2	0,01299974	0,05166946	0,25	0,09	1	1,036463606	0,964819212	2,777777778
ZNF365	6,3574E-05	0,051636764	0,062	0	1	1,036440117	0,964841078	#DIV/0!

Supplementary Table 1

Differentially expressed genes_adjacent-skin-XL_ comparing SC-Keloid vs. (SC-Myel, SC-Nonmyel)								
**gene**	**p_val**	**avg_log2FC**	**pct.1**	**pct.2**	**p_val_adj**	**Foldchange_UP**	**Foldchange_DOWN**	**Ratio_pct1_pct2**
VAMP3	0,00023321	0,051576142	0,594	0,203	1	1,036396567	0,964881621	2,926108374
ILVBL	0,058043615	0,051534875	0,156	0,059	1	1,036366922	0,964909221	2,644067797
TCIRG1	0,000114181	0,051502367	0,25	0,051	1	1,03634357	0,964930964	4,901960784
SMC6	5,55781E-06	0,051498133	0,281	0,047	0,117275301	1,036340529	0,964933795	5,978723404
ZNF354B	0,770919596	0,051470129	0,031	0,023	1	1,036320413	0,964952526	1,347826087
MSL3	0,00011037	0,051455317	0,219	0,039	1	1,036309773	0,964962433	5,615384615
MAN1B1	3,60095E-05	0,051152358	0,375	0,09	0,759836844	1,036092176	0,965165092	4,166666667
ZNF747	0,000112833	0,051144461	0,125	0,012	1	1,036086505	0,965170375	10,41666667
ZNF598	0,019329956	0,051105223	0,188	0,062	1	1,036058326	0,965196626	3,032258065
FOXS1	6,3574E-05	0,051080942	0,062	0	1	1,036040889	0,96521287	#DIV/0!
LINC00629	6,3574E-05	0,051080942	0,062	0	1	1,036040889	0,96521287	#DIV/0!
BBS12	0,212914042	0,051017722	0,062	0,023	1	1,03599549	0,965255168	2,695652174
SH3BP1	0,000494525	0,050936579	0,125	0,016	1	1,035937223	0,965309459	7,8125
POU2F3	0,079594089	0,050889184	0,031	0,004	1	1,035903191	0,965341171	7,75
FARS2	0,058551336	0,050799898	0,125	0,043	1	1,035839082	0,965400917	2,906976744
PSEN2	0,002594281	0,050711416	0,094	0,012	1	1,035775556	0,965460128	7,833333333
TSPAN17	0,000112833	0,050704255	0,125	0,012	1	1,035770414	0,96546492	10,41666667
NEK3	0,212914042	0,050630922	0,062	0,023	1	1,035717767	0,965513996	2,695652174
CDKN3	0,01346698	0,050528333	0,062	0,008	1	1,03564412	0,965582656	7,75
ASB16-AS1	0,145431	0,050482593	0,062	0,02	1	1,035611286	0,965613269	3,1
CIP2A	4,80439E-05	0,050474348	0,094	0,004	1	1,035605367	0,965618788	23,5
AAR2	0,01346698	0,050460145	0,062	0,008	1	1,035595173	0,965628294	7,75
UBL7-DT	0,002263143	0,050452879	0,062	0,004	1	1,035589957	0,965633158	15,5
ZNF775	0,000466117	0,050394721	0,188	0,035	1	1,035548211	0,965672085	5,371428571
SNUPN	0,000390872	0,050388593	0,281	0,07	1	1,035543812	0,965676187	4,014285714
TIMM29	4,123E-05	0,050276082	0,219	0,035	0,869994633	1,035463057	0,9657515	6,257142857
HERC6	0,133058992	0,050239392	0,094	0,035	1	1,035436724	0,965776061	2,685714286
TBC1D1	0,004186485	0,050218337	0,156	0,035	1	1,035421613	0,965790155	4,457142857
ARPC5L	2,18864E-05	0,050208398	0,375	0,086	0,461825118	1,03541448	0,965796809	4,360465116
PGBD1	4,80439E-05	0,050185908	0,094	0,004	1	1,035398338	0,965811865	23,5

Supplementary Table 1

Differentially expressed genes_adjacent-skin-XL_ comparing SC-Keloid vs. (SC-Myel, SC-Nonmyel)								
**gene**	**p_val**	**avg_log2FC**	**pct.1**	**pct.2**	**p_val_adj**	**Foldchange_UP**	**Foldchange_DOWN**	**Ratio_pct1_pct2**
CYB561D1	0,002263143	0,050091962	0,062	0,004	1	1,035330917	0,965874759	15,5
NUF2	0,082317662	0,049925751	0,031	0,004	1	1,035211645	0,965986042	7,75
ZNF558	0,094667448	0,049922866	0,094	0,031	1	1,035209575	0,965987974	3,032258065
NSMCE2	1,67202E-05	0,049711432	0,312	0,062	0,352813205	1,03505787	0,966129555	5,032258065
SPATA6L	0,079594089	0,049656993	0,031	0,004	1	1,035018814	0,966166012	7,75
KLKB1	0,226351275	0,049498526	0,031	0,008	1	1,034905133	0,966272142	3,875
ISCA1	7,63763E-05	0,049442505	0,406	0,109	1	1,034864948	0,966309664	3,724770642
TMEM14A	0,000217056	0,04944141	0,531	0,172	1	1,034864162	0,966310398	3,087209302
CDC42SE2	0,000666239	0,049419626	0,281	0,074	1	1,034848537	0,966324988	3,797297297
SLC37A4	0,008607623	0,049347868	0,125	0,027	1	1,034797065	0,966373054	4,62962963
COX10	0,013891036	0,049267327	0,062	0,008	1	1,034739297	0,966427005	7,75
SRSF2	0,018901273	0,04924444	0,844	0,426	1	1,034722883	0,966442336	1,981220657
ZNF326	0,00344676	0,049235561	0,375	0,133	1	1,034716515	0,966448284	2,819548872
MINDY3	0,084174094	0,049186039	0,125	0,047	1	1,034680998	0,966481459	2,659574468
ERCC2	0,041848356	0,049149762	0,062	0,012	1	1,03465498	0,966505762	5,166666667
TOMM40L	0,000509339	0,049103101	0,125	0,016	1	1,034621518	0,966537022	7,8125
PLPP7	0,000524345	0,049095055	0,094	0,008	1	1,034615747	0,966542412	11,75
CHRNE	0,226351275	0,049077684	0,031	0,008	1	1,034603289	0,96655405	3,875
CCDC112	0,000338057	0,049029843	0,344	0,094	1	1,034568982	0,966586102	3,659574468
RTL8B	0,000993746	0,049009445	0,188	0,039	1	1,034554355	0,966599769	4,820512821
ANO7	0,226351275	0,048851154	0,031	0,008	1	1,034440851	0,966705829	3,875
TSPAN9	0,00094841	0,048817685	0,219	0,051	1	1,034416853	0,966728256	4,294117647
MRPL32	5,38695E-05	0,048723706	0,375	0,094	1	1,034349472	0,966791232	3,989361702
S100PBP	9,91727E-06	0,048667873	0,25	0,039	0,209264362	1,034309443	0,966828648	6,41025641
GEMIN6	0,013891036	0,048665501	0,062	0,008	1	1,034307742	0,966830237	7,75
MYPOP	0,000466094	0,048565652	0,125	0,016	1	1,03423616	0,966897154	7,8125
H2AC20	0,02693544	0,048551548	0,125	0,035	1	1,034226049	0,966906607	3,571428571
MDC1	0,008607623	0,048489251	0,125	0,027	1	1,034181391	0,96694836	4,62962963
BBOF1	0,008148126	0,048451875	0,094	0,016	1	1,034154599	0,966973411	5,875
IDS	0,000311395	0,048305345	0,625	0,223	1	1,034049569	0,967071628	2,802690583

Supplementary Table 1

Differentially expressed genes_adjacent-skin-XL_ comparing SC-Keloid vs. (SC-Myel, SC-Nonmyel)								
**gene**	**p_val**	**avg_log2FC**	**pct.1**	**pct.2**	**p_val_adj**	**Foldchange_UP**	**Foldchange_DOWN**	**Ratio_pct1_pct2**
MIA3	0,005262184	0,048266064	0,281	0,094	1	1,034021414	0,96709796	2,989361702
MOV10	0,061916284	0,048241166	0,094	0,027	1	1,03400357	0,96711465	3,481481481
CARD19	0,000812022	0,048156263	0,438	0,148	1	1,03394272	0,967171566	2,959459459
SHARPIN	0,000242575	0,048145139	0,375	0,105	1	1,033934748	0,967179024	3,571428571
MAGEF1	0,000591923	0,047994993	0,312	0,086	1	1,033827148	0,967279687	3,627906977
NOL10	0,000911755	0,047979945	0,156	0,027	1	1,033816365	0,967289776	5,777777778
AFG1L	0,013891036	0,047912595	0,062	0,008	1	1,033768104	0,967334933	7,75
SCRN2	0,006263449	0,047733022	0,219	0,066	1	1,033639438	0,967455346	3,318181818
ZWILCH	0,004087528	0,047725022	0,125	0,023	1	1,033633707	0,96746071	5,434782609
FGFR4	0,004841742	0,047706206	0,031	0	1	1,033620226	0,967473328	#DIV/0!
STEAP3	0,004841742	0,047706206	0,031	0	1	1,033620226	0,967473328	#DIV/0!
PIP4K2C	0,018436147	0,047569063	0,094	0,02	1	1,033521974	0,967565301	4,7
C17orf75	0,000108998	0,047559421	0,125	0,012	1	1,033515067	0,967571768	10,41666667
TAS2R30	0,082317662	0,047503445	0,031	0,004	1	1,033474967	0,96760931	7,75
ZNF71	0,008148126	0,047437658	0,094	0,016	1	1,033427842	0,967653434	5,875
R3HDM2	0,001092769	0,047420108	0,469	0,168	1	1,033415271	0,967665205	2,791666667
ZNF185	0,082317662	0,047391357	0,031	0,004	1	1,033394677	0,967684489	7,75
GABPA	0,00721637	0,047335217	0,219	0,066	1	1,033354465	0,967722146	3,318181818
KIAA1191	0,001907981	0,047313753	0,281	0,082	1	1,033339091	0,967736544	3,426829268
ADCY3	0,149643094	0,047277405	0,125	0,055	1	1,033313056	0,967760926	2,272727273
GEMIN8	0,143670631	0,047277137	0,125	0,055	1	1,033312865	0,967761105	2,272727273
RAB14	0,000457512	0,047255827	0,625	0,223	1	1,033297602	0,9677754	2,802690583
TARS1	0,001640115	0,047169826	0,406	0,141	1	1,033236008	0,967833092	2,879432624
TBC1D9B	0,012186755	0,047136036	0,312	0,121	1	1,033211808	0,96785576	2,578512397
CNOT8	0,00011289	0,047110673	0,219	0,039	1	1,033193644	0,967872776	5,615384615
PLAAT4	0,060931358	0,047099357	0,094	0,027	1	1,03318554	0,967880367	3,481481481
CYREN	0,000911755	0,04708958	0,156	0,027	1	1,033178538	0,967886927	5,777777778
RNF135	0,008270631	0,046942188	0,125	0,027	1	1,033072989	0,967985815	4,62962963
BBS1	0,45783772	0,046936039	0,062	0,035	1	1,033068586	0,967989941	1,771428571
LBX2	0,002263143	0,046923378	0,062	0,004	1	1,03305952	0,967998436	15,5

Supplementary Table 1

Differentially expressed genes_adjacent-skin-XL_ comparing SC-Keloid vs. (SC-Myel, SC-Nonmyel)								
**gene**	**p_val**	**avg_log2FC**	**pct.1**	**pct.2**	**p_val_adj**	**Foldchange_UP**	**Foldchange_DOWN**	**Ratio_pct1_pct2**
CCDC22	2,62125E-05	0,046919969	0,25	0,043	0,553110895	1,033057079	0,968000724	5,813953488
GPR89B	0,002010541	0,046905064	0,156	0,031	1	1,033046407	0,968010724	5,032258065
ADAMTS15	0,002594281	0,046791738	0,094	0,012	1	1,032965262	0,968086766	7,833333333
PECR	0,082317662	0,046780031	0,031	0,004	1	1,03295688	0,968094622	7,75
POMC	0,000524345	0,04668558	0,094	0,008	1	1,032889256	0,968158004	11,75
BRSK1	0,002881213	0,04655946	0,312	0,102	1	1,032798965	0,968242643	3,058823529
IGSF9B	0,147778936	0,046556341	0,062	0,02	1	1,032796732	0,968244737	3,1
COP1	0,000190919	0,046542916	0,188	0,031	1	1,032787121	0,968253747	6,064516129
ZNF212	0,226351275	0,046507869	0,031	0,008	1	1,032762033	0,968277268	3,875
COLGALT1	0,007861788	0,046502478	0,25	0,082	1	1,032758173	0,968280887	3,048780488
ZNF100	0,041848356	0,046392901	0,062	0,012	1	1,032679735	0,968354434	5,166666667
ZMIZ2	0,02818312	0,04634496	0,156	0,051	1	1,03264542	0,968386613	3,058823529
BRIP1	6,3574E-05	0,046338562	0,062	0	1	1,03264084	0,968390907	#DIV/0!
MACROH2A2	6,3574E-05	0,046338562	0,062	0	1	1,03264084	0,968390907	#DIV/0!
MESP2	6,3574E-05	0,046338562	0,062	0	1	1,03264084	0,968390907	#DIV/0!
NPAS1	6,3574E-05	0,046338562	0,062	0	1	1,03264084	0,968390907	#DIV/0!
POC1A	6,3574E-05	0,046338562	0,062	0	1	1,03264084	0,968390907	#DIV/0!
DNAAF11	0,082317662	0,046318131	0,031	0,004	1	1,032626216	0,968404622	7,75
ABHD11	2,1417E-05	0,04630075	0,188	0,023	0,451919526	1,032613776	0,968416288	8,173913043
TEX10	0,026506403	0,046274974	0,125	0,035	1	1,032595327	0,968433591	3,571428571
RIPOR3	0,004841742	0,046193245	0,031	0	1	1,032536832	0,968488454	#DIV/0!
KBTBD4	0,018436147	0,045949204	0,094	0,02	1	1,032362186	0,968652294	4,7
SERPINB6	0,003311505	0,045878196	0,594	0,238	1	1,032311376	0,968699971	2,495798319
RNF8	7,13916E-07	0,045820627	0,281	0,039	0,015064347	1,032270184	0,968738627	7,205128205
KSR2	6,3574E-05	0,045698772	0,062	0	1	1,032182998	0,968820454	#DIV/0!
GMNN	4,96307E-06	0,04566867	0,188	0,02	0,104725812	1,032161461	0,968840668	9,4
SPICE1	0,018436147	0,045558458	0,094	0,02	1	1,032082615	0,968914684	4,7
ANKFY1	0,02857055	0,045511208	0,156	0,051	1	1,032048813	0,968946417	3,058823529
FKBP7	1,9707E-05	0,045463456	0,406	0,098	0,41583782	1,032014654	0,968978489	4,142857143
POLR2I	0,002434679	0,045440371	0,594	0,238	1	1,03199814	0,968993994	2,495798319

Supplementary Table 1

Differentially expressed genes_adjacent-skin-XL_ comparing SC-Keloid vs. (SC-Myel, SC-Nonmyel)								
gene	p_val	avg_log2FC	pct.1	pct.2	p_val_adj	Foldchange_UP	Foldchange_DOWN	Ratio_pct1_pct2
ATRIP	0,527450503	0,045430249	0,031	0,016	1	1,0319909	0,969000793	1,9375
PRPF40B	0,143111738	0,045407689	0,062	0,02	1	1,031974762	0,969015945	3,1
CCDC138	0,002263143	0,045404213	0,062	0,004	1	1,031972276	0,96901828	15,5
CFAP20DC	0,002263143	0,045351551	0,062	0,004	1	1,031934607	0,969053653	15,5
CDCA3	0,004841742	0,045292115	0,031	0	1	1,031892094	0,969093576	#DIV/0!
DLGAP5	0,004841742	0,045292115	0,031	0	1	1,031892094	0,969093576	#DIV/0!
ESCO2	0,004841742	0,045292115	0,031	0	1	1,031892094	0,969093576	#DIV/0!
HMMR	0,004841742	0,045292115	0,031	0	1	1,031892094	0,969093576	#DIV/0!
KIF14	0,004841742	0,045292115	0,031	0	1	1,031892094	0,969093576	#DIV/0!
PIMREG	0,004841742	0,045292115	0,031	0	1	1,031892094	0,969093576	#DIV/0!
SPC25	0,004841742	0,045292115	0,031	0	1	1,031892094	0,969093576	#DIV/0!
MKS1	0,01346698	0,045282751	0,062	0,008	1	1,031885396	0,969099867	7,75
LINC00847	0,001642148	0,045207468	0,125	0,02	1	1,031831552	0,969150438	6,25
PLEK2	4,80439E-05	0,045149985	0,094	0,004	1	1,03179044	0,969189053	23,5
IFT43	0,000484576	0,045105681	0,25	0,059	1	1,031758755	0,969218817	4,237288136
POLE2	0,082317662	0,044927849	0,031	0,004	1	1,031631585	0,969338294	7,75
TOPBP1	0,000466117	0,044909854	0,188	0,035	1	1,031618717	0,969350384	5,371428571
GPAT4	0,011239937	0,044909568	0,156	0,043	1	1,031618512	0,969350577	3,627906977
GSTCD	0,041848356	0,044762069	0,062	0,012	1	1,031513047	0,969449686	5,166666667
FAM177A1	0,000778225	0,044747397	0,719	0,273	1	1,031502557	0,969459546	2,633699634
BST2	0,470250215	0,044721157	0,062	0,035	1	1,031483796	0,969477179	1,771428571
VPS37D	0,082317662	0,044686866	0,031	0,004	1	1,031459279	0,969500222	7,75
TRIP4	0,0016948	0,044594114	0,219	0,055	1	1,031392967	0,969562554	3,981818182
COG6	6,10931E-06	0,044539974	0,281	0,047	0,128912454	1,031354264	0,969598939	5,978723404
MYBL2	6,3574E-05	0,044530159	0,062	0	1	1,031347247	0,969605536	#DIV/0!
PCDHGA8	0,082317662	0,044387311	0,031	0,004	1	1,031245134	0,969701546	7,75
PIK3R2	0,004029529	0,044363202	0,156	0,035	1	1,0312279	0,969717751	4,457142857
GLIPR2	0,000371827	0,044291149	0,312	0,082	1	1,031176398	0,969766183	3,804878049
INHBA	0,082317662	0,04424159	0,031	0,004	1	1,031140976	0,969799497	7,75
LRRC61	0,003907631	0,044234389	0,125	0,023	1	1,03113583	0,969804337	5,434782609

Supplementary Table 1

Differentially expressed genes_adjacent-skin-XL_ comparing SC-Keloid vs. (SC-Myel, SC-Nonmyel)								
**gene**	**p_val**	**avg_log2FC**	**pct.1**	**pct.2**	**p_val_adj**	**Foldchange_UP**	**Foldchange_DOWN**	**Ratio_pct1_pct2**
DNAAF2	0,000954402	0,044223414	0,188	0,039	1	1,031127986	0,969811715	4,820512821
GAK	0,150636831	0,044185597	0,156	0,074	1	1,031100957	0,969837137	2,108108108
GLE1	0,000509339	0,04406156	0,125	0,016	1	1,031012311	0,969920523	7,8125
TWIST1	0,380266129	0,044028391	0,031	0,012	1	1,030988607	0,969942823	2,583333333
PRPH	0,004841742	0,043986939	0,031	0	1	1,030958986	0,969970691	#DIV/0!
DAAM1	0,000659904	0,043942762	0,344	0,102	1	1,030927417	0,970000394	3,37254902
RHOD	6,3574E-05	0,043837747	0,062	0	1	1,030852377	0,970071003	#DIV/0!
SLC16A2	0,040857201	0,043787902	0,062	0,012	1	1,030816762	0,97010452	5,166666667
BAK1	0,000436372	0,043776486	0,188	0,035	1	1,030808605	0,970112196	5,371428571
ZNF469	0,004841742	0,04368854	0,031	0	1	1,030745769	0,970171336	#DIV/0!
PREB	0,000850121	0,043621507	0,156	0,027	1	1,030697878	0,970216415	5,777777778
NPB	0,380266129	0,043436106	0,031	0,012	1	1,030565432	0,970341105	2,583333333
CSNK1D	0,01231509	0,043400133	0,312	0,121	1	1,030539736	0,9703653	2,578512397
FDFT1	7,61493E-08	0,043329024	0,375	0,059	0,001606825	1,030488942	0,97041313	6,355932203
MAP3K10	0,002594281	0,043290304	0,094	0,012	1	1,030461286	0,970439175	7,833333333
B3GNT9	0,002594281	0,043286227	0,094	0,012	1	1,030458374	0,970441917	7,833333333
UPP1	0,001318364	0,043128408	0,375	0,121	1	1,030345656	0,970548082	3,099173554
SPIN3	0,084624339	0,043104303	0,062	0,016	1	1,030328441	0,970564298	3,875
TLE2	0,004107327	0,04306958	0,156	0,035	1	1,030303643	0,970587658	4,457142857
STIMATE	0,059959447	0,043038428	0,094	0,027	1	1,030281396	0,970608616	3,481481481
DDX46	5,9111E-05	0,043023731	0,625	0,199	1	1,030270901	0,970618504	3,140703518
LINC00702	0,004841742	0,043005039	0,031	0	1	1,030257552	0,970631079	#DIV/0!
TSHZ3	0,013891036	0,042967315	0,062	0,008	1	1,030230613	0,97065646	7,75
DIRC3	0,002263143	0,042960115	0,062	0,004	1	1,030225472	0,970661304	15,5
PDCD2	0,000756965	0,042940594	0,281	0,074	1	1,030211532	0,970674438	3,797297297
WDR53	0,002594281	0,042882044	0,094	0,012	1	1,030169722	0,970713833	7,833333333
DHX33	0,003953072	0,042760886	0,156	0,035	1	1,030083212	0,970795357	4,457142857
CISD2	0,001125066	0,042724311	0,281	0,078	1	1,030057098	0,970819969	3,602564103
AP1M2	0,004841742	0,042610748	0,031	0	1	1,029976019	0,970896391	#DIV/0!
BCL2A1	0,004841742	0,042610748	0,031	0	1	1,029976019	0,970896391	#DIV/0!

Supplementary Table 1

Differentially expressed genes_adjacent-skin-XL_ comparing SC-Keloid vs. (SC-Myel, SC-Nonmyel)								
**gene**	**p_val**	**avg_log2FC**	**pct.1**	**pct.2**	**p_val_adj**	**Foldchange_UP**	**Foldchange_DOWN**	**Ratio_pct1_pct2**
BTBD16	0,004841742	0,042610748	0,031	0	1	1,029976019	0,970896391	#DIV/0!
CASP8	0,004841742	0,042610748	0,031	0	1	1,029976019	0,970896391	#DIV/0!
CD79A	0,004841742	0,042610748	0,031	0	1	1,029976019	0,970896391	#DIV/0!
CEACAM5	0,004841742	0,042610748	0,031	0	1	1,029976019	0,970896391	#DIV/0!
CKMT1A	0,004841742	0,042610748	0,031	0	1	1,029976019	0,970896391	#DIV/0!
EEF1A2	0,004841742	0,042610748	0,031	0	1	1,029976019	0,970896391	#DIV/0!
FAM66B	0,004841742	0,042610748	0,031	0	1	1,029976019	0,970896391	#DIV/0!
FLG	0,004841742	0,042610748	0,031	0	1	1,029976019	0,970896391	#DIV/0!
HSD11B2	0,004841742	0,042610748	0,031	0	1	1,029976019	0,970896391	#DIV/0!
LINC00460	0,004841742	0,042610748	0,031	0	1	1,029976019	0,970896391	#DIV/0!
NOTUM	0,004841742	0,042610748	0,031	0	1	1,029976019	0,970896391	#DIV/0!
PAQR5	0,004841742	0,042610748	0,031	0	1	1,029976019	0,970896391	#DIV/0!
SPTSSB	0,004841742	0,042610748	0,031	0	1	1,029976019	0,970896391	#DIV/0!
TMPRSS11E	0,004841742	0,042610748	0,031	0	1	1,029976019	0,970896391	#DIV/0!
ZBTB8B	0,004841742	0,042610748	0,031	0	1	1,029976019	0,970896391	#DIV/0!
EFHD1	0,041848356	0,042587571	0,062	0,012	1	1,029959473	0,970911989	5,166666667
ZNF875	0,062914349	0,042418724	0,094	0,027	1	1,029838938	0,971025626	3,481481481
DCAF13	0,001144569	0,042400085	0,312	0,094	1	1,029825633	0,971038172	3,319148936
POLR1H	0,028962559	0,042257826	0,156	0,051	1	1,029724091	0,971133927	3,058823529
ZNF682	0,013891036	0,042236426	0,062	0,008	1	1,029708816	0,971148332	7,75
ERFE	0,002263143	0,042204433	0,062	0,004	1	1,029685982	0,971169869	15,5
FANCG	1,51098E-05	0,042110654	0,125	0,008	0,318831333	1,029619052	0,971233	15,625
NUP42	3,33107E-06	0,042015232	0,25	0,035	0,070289013	1,029550953	0,971297241	7,142857143
SMPD1	0,000502235	0,042015049	0,25	0,059	1	1,029550823	0,971297364	4,237288136
VPS33A	0,019236133	0,041915117	0,094	0,02	1	1,02947951	0,971364646	4,7
TEAD4	0,004180266	0,041911889	0,125	0,023	1	1,029477207	0,971366819	5,434782609
PIP	0,082317662	0,041820201	0,031	0,004	1	1,029411783	0,971428554	7,75
SLC35E3	0,012905641	0,041818062	0,219	0,074	1	1,029410257	0,971429995	2,959459459
FRAT2	0,221757959	0,041809874	0,062	0,023	1	1,029404414	0,971435508	2,695652174
RGP1	0,036158386	0,041780737	0,094	0,023	1	1,029383624	0,971455128	4,086956522

Supplementary Table 1

Differentially expressed genes_adjacent-skin-XL_ comparing SC-Keloid vs. (SC-Myel, SC-Nonmyel)								
**gene**	**p_val**	**avg_log2FC**	**pct.1**	**pct.2**	**p_val_adj**	**Foldchange_UP**	**Foldchange_DOWN**	**Ratio_pct1_pct2**
ATP6V1A	0,03766997	0,041776056	0,188	0,07	1	1,029380284	0,97145828	2,685714286
DPF1	6,3574E-05	0,041755134	0,062	0	1	1,029365356	0,971472368	#DIV/0!
VLDLR	6,3574E-05	0,041755134	0,062	0	1	1,029365356	0,971472368	#DIV/0!
TNFAIP8	9,17361E-07	0,041739326	0,188	0,016	0,019357231	1,029354077	0,971483013	11,75
ZFP69B	0,002263143	0,041731076	0,062	0,004	1	1,029348191	0,971488568	15,5
C12orf43	0,041848356	0,041721687	0,062	0,012	1	1,029341492	0,97149489	5,166666667
MDFI	0,000524345	0,04165707	0,094	0,008	1	1,029295389	0,971538404	11,75
ZNF300	0,041848356	0,041650946	0,062	0,012	1	1,02929102	0,971542528	5,166666667
MMAB	1,93892E-07	0,041571307	0,281	0,035	0,004091308	1,029234204	0,97159616	8,028571429
PGD	0,002601637	0,041570409	0,219	0,059	1	1,029233563	0,971596765	3,711864407
ZNF668	0,002263143	0,041569758	0,062	0,004	1	1,029233099	0,971597203	15,5
POLD3	7,15271E-05	0,041539567	0,188	0,027	1	1,02921156	0,971617536	6,962962963
MBLAC2	0,000509339	0,041454213	0,125	0,016	1	1,029150671	0,971675021	7,8125
CFAP69	0,013891036	0,041423561	0,062	0,008	1	1,029128806	0,971695666	7,75
METTL7B	0,002263143	0,041404947	0,062	0,004	1	1,029115528	0,971708203	15,5
APOM	4,80439E-05	0,04130825	0,094	0,004	1	1,029046553	0,971773334	23,5
PEX19	0,060128123	0,041303866	0,125	0,043	1	1,029043427	0,971776287	2,906976744
GPR180	0,00010081	0,041271693	0,219	0,039	1	1,029020478	0,971797959	5,615384615
PRICKLE1	0,018832425	0,04125514	0,094	0,02	1	1,029008672	0,971809109	4,7
NIT2	5,84452E-05	0,041209923	0,438	0,117	1	1,028976421	0,971839568	3,743589744
TRAPPC14	0,041848356	0,04114678	0,062	0,012	1	1,028931386	0,971882103	5,166666667
ZSCAN16	6,3574E-05	0,040995724	0,062	0	1	1,028823659	0,971983869	#DIV/0!
CCDC150	0,082317662	0,040986045	0,031	0,004	1	1,028816756	0,97199039	7,75
POLR3C	0,036827385	0,040985195	0,094	0,023	1	1,02881615	0,971990962	4,086956522
MRPL10	4,52605E-06	0,040959454	0,219	0,027	0,095504122	1,028797794	0,972008305	8,111111111
FYTTD1	0,000308767	0,040845197	0,531	0,176	1	1,028716319	0,972085289	3,017045455
ZCCHC24	2,34925E-05	0,040827018	0,625	0,195	0,49571452	1,028703357	0,972097537	3,205128205
ZNF546	0,082317662	0,040743213	0,031	0,004	1	1,028643602	0,972154007	7,75
SPECC1L	0,006630119	0,040726234	0,219	0,066	1	1,028631496	0,972165449	3,318181818
STIL	0,002263143	0,040530203	0,062	0,004	1	1,028491737	0,972297554	15,5

Supplementary Table 1

Differentially expressed genes_adjacent-skin-XL_ comparing SC-Keloid vs. (SC-Myel, SC-Nonmyel)								
**gene**	**p_val**	**avg_log2FC**	**pct.1**	**pct.2**	**p_val_adj**	**Foldchange_UP**	**Foldchange_DOWN**	**Ratio_pct1_pct2**
RRP8	0,007471523	0,040402771	0,156	0,039	1	1,028400895	0,97238344	4
BAIAP2L2	0,004841742	0,040399781	0,031	0	1	1,028398764	0,972385455	#DIV/0!
CACNG4	0,004841742	0,040399781	0,031	0	1	1,028398764	0,972385455	#DIV/0!
CFAP45	0,004841742	0,040399781	0,031	0	1	1,028398764	0,972385455	#DIV/0!
EAF1-AS1	0,004841742	0,040399781	0,031	0	1	1,028398764	0,972385455	#DIV/0!
GPC6	0,004841742	0,040399781	0,031	0	1	1,028398764	0,972385455	#DIV/0!
GPR183	0,004841742	0,040399781	0,031	0	1	1,028398764	0,972385455	#DIV/0!
LINC01545	0,004841742	0,040399781	0,031	0	1	1,028398764	0,972385455	#DIV/0!
LYPD6	0,004841742	0,040399781	0,031	0	1	1,028398764	0,972385455	#DIV/0!
ODAM	0,004841742	0,040399781	0,031	0	1	1,028398764	0,972385455	#DIV/0!
LCORL	0,028438047	0,040337379	0,188	0,066	1	1,028354283	0,972427515	2,848484848
KLF16	0,029498271	0,040254371	0,219	0,082	1	1,028295116	0,972483467	2,670731707
SLC26A11	0,45783772	0,040244449	0,062	0,035	1	1,028288044	0,972490155	1,771428571
CIZ1	0,000478496	0,040215388	0,219	0,047	1	1,028267331	0,972509745	4,659574468
MUCL1	0,013891036	0,040206493	0,062	0,008	1	1,028260991	0,972515741	7,75
VIRMA	0,008607623	0,040201835	0,125	0,027	1	1,028257672	0,972518881	4,62962963
MPZL1	0,037506898	0,040196336	0,094	0,023	1	1,028253752	0,972522588	4,086956522
PPP1R42	0,002263143	0,040183789	0,062	0,004	1	1,02824481	0,972531046	15,5
C1orf112	0,040857201	0,040059265	0,062	0,012	1	1,028156062	0,972614992	5,166666667
AFF2	0,004841742	0,040033841	0,031	0	1	1,028137943	0,972632132	#DIV/0!
BEAN1	0,004841742	0,040033841	0,031	0	1	1,028137943	0,972632132	#DIV/0!
FAM225B	0,004841742	0,040033841	0,031	0	1	1,028137943	0,972632132	#DIV/0!
FSCN2	0,004841742	0,040033841	0,031	0	1	1,028137943	0,972632132	#DIV/0!
H2AC21	0,004841742	0,040033841	0,031	0	1	1,028137943	0,972632132	#DIV/0!
INPP5B	0,004841742	0,040033841	0,031	0	1	1,028137943	0,972632132	#DIV/0!
LGALS8-AS1	0,004841742	0,040033841	0,031	0	1	1,028137943	0,972632132	#DIV/0!
LINC00954	0,004841742	0,040033841	0,031	0	1	1,028137943	0,972632132	#DIV/0!
LRFN3	0,004841742	0,040033841	0,031	0	1	1,028137943	0,972632132	#DIV/0!
NCF1	0,004841742	0,040033841	0,031	0	1	1,028137943	0,972632132	#DIV/0!
NUDT11	0,004841742	0,040033841	0,031	0	1	1,028137943	0,972632132	#DIV/0!

Supplementary Table 1

Differentially expressed genes_adjacent-skin-XL_ comparing SC-Keloid vs. (SC-Myel, SC-Nonmyel)								
**gene**	**p_val**	**avg_log2FC**	**pct.1**	**pct.2**	**p_val_adj**	**Foldchange_UP**	**Foldchange_DOWN**	**Ratio_pct1_pct2**
SOX18	0,004841742	0,040033841	0,031	0	1	1,028137943	0,972632132	#DIV/0!
TMEM145	0,004841742	0,040033841	0,031	0	1	1,028137943	0,972632132	#DIV/0!
ZNF492	0,004841742	0,040033841	0,031	0	1	1,028137943	0,972632132	#DIV/0!
CASP9	0,02857055	0,039967284	0,156	0,051	1	1,028090512	0,972677005	3,058823529
KAT14	0,086278754	0,039959426	0,062	0,016	1	1,028084913	0,972682302	3,875
TTC38	0,040857201	0,039929441	0,062	0,012	1	1,028063545	0,972702519	5,166666667
CEP97	0,000588759	0,039899975	0,375	0,113	1	1,028042548	0,972722386	3,318584071
CD6	6,3574E-05	0,039889012	0,062	0	1	1,028034736	0,972729778	#DIV/0!
MILR1	6,3574E-05	0,039889012	0,062	0	1	1,028034736	0,972729778	#DIV/0!
GPN2	0,001642148	0,039838624	0,125	0,02	1	1,027998831	0,972763752	6,25
LRRC29	6,3574E-05	0,039739396	0,062	0	1	1,027928128	0,972830661	#DIV/0!
ATMIN	0,007089636	0,039707337	0,156	0,039	1	1,027905286	0,972852279	4
KREMEN1	0,008148126	0,039627251	0,094	0,016	1	1,027848227	0,972906285	5,875
ANAPC4	0,00019555	0,039584967	0,188	0,031	1	1,027818102	0,9729348	6,064516129
SUPT7L	0,000466117	0,039561222	0,188	0,035	1	1,027801186	0,972950814	5,371428571
SCRG1	0,003547823	0,039503443	0,344	0,121	1	1,027760024	0,97298978	2,842975207
SEPSECS	0,002263143	0,039443577	0,062	0,004	1	1,027717377	0,973030156	15,5
AGPAT3	0,000311736	0,039418985	0,312	0,082	1	1,027699859	0,973046743	3,804878049
FANCD2	0,082317662	0,039407994	0,031	0,004	1	1,027692029	0,973054156	7,75
CCDC85B	0,001081065	0,039315342	0,656	0,262	1	1,027626032	0,973116649	2,503816794
PXK	0,065622259	0,039264011	0,188	0,078	1	1,027589469	0,973151273	2,41025641
ARID3B	2,40115E-05	0,039232875	0,156	0,016	0,506667402	1,027567293	0,973172275	9,75
MIS18A	0,000339359	0,039225572	0,156	0,023	1	1,027562091	0,973177202	6,782608696
COPS7A	6,30883E-05	0,039212446	0,312	0,07	1	1,027552742	0,973186056	4,457142857
WDHD1	9,06462E-07	0,039211754	0,125	0,004	0,019127258	1,027552249	0,973186522	31,25
SS18	0,017996779	0,039150993	0,281	0,113	1	1,027508973	0,97322751	2,486725664
ABCC8	0,226351275	0,039150171	0,031	0,008	1	1,027508388	0,973228065	3,875
TMEM243	1,21952E-05	0,039105466	0,375	0,082	0,257330394	1,027476548	0,973258223	4,573170732
OSBPL3	0,221475664	0,039104402	0,031	0,008	1	1,027475791	0,973258941	3,875
KIAA0895L	0,002594281	0,039071951	0,094	0,012	1	1,02745268	0,973280833	7,833333333

Supplementary Table 1

Differentially expressed genes_adjacent-skin-XL_ comparing SC-Keloid vs. (SC-Myel, SC-Nonmyel)								
**gene**	**p_val**	**avg_log2FC**	**pct.1**	**pct.2**	**p_val_adj**	**Foldchange_UP**	**Foldchange_DOWN**	**Ratio_pct1_pct2**
GSTM4	0,018436147	0,039000142	0,094	0,02	1	1,027401541	0,973329278	4,7
ATG3	0,001601928	0,038965507	0,406	0,141	1	1,027376876	0,973352645	2,879432624
CERK	0,041848356	0,038927375	0,062	0,012	1	1,027349722	0,973378372	5,166666667
SH3GL1	0,001385781	0,038912635	0,438	0,148	1	1,027339225	0,973388318	2,959459459
GMIP	0,002263143	0,038912446	0,062	0,004	1	1,027339091	0,973388445	15,5
FBLL1	0,002263143	0,038880029	0,062	0,004	1	1,027316007	0,973410317	15,5
SLC9B1	0,082317662	0,038812237	0,031	0,004	1	1,027267735	0,973456059	7,75
PAK1	0,004180266	0,03880875	0,125	0,023	1	1,027265252	0,973458411	5,434782609
TMEM104	4,80439E-05	0,038757658	0,094	0,004	1	1,027228872	0,973492886	23,5
PLTP	0,000291314	0,038733219	0,375	0,109	1	1,027211471	0,973509377	3,440366972
SLMAP	0,000247303	0,038728151	0,219	0,043	1	1,027207863	0,973512797	5,093023256
CSTA	0,041848356	0,038633522	0,062	0,012	1	1,027140489	0,973576654	5,166666667
EXOC3-AS1	0,000524345	0,038618982	0,094	0,008	1	1,027130137	0,973586466	11,75
COMMD2	0,001245548	0,038408833	0,312	0,094	1	1,026980532	0,973728293	3,319148936
RELL1	0,007214939	0,03836489	0,156	0,039	1	1,026949251	0,973757952	4
CDCA8	0,082317662	0,038335982	0,031	0,004	1	1,026928674	0,973777464	7,75
GGACT	6,3574E-05	0,038294453	0,062	0	1	1,026899114	0,973805495	#DIV/0!
SPA17	6,3574E-05	0,038294453	0,062	0	1	1,026899114	0,973805495	#DIV/0!
ALPK2	6,3574E-05	0,038290028	0,062	0	1	1,026895964	0,973808482	#DIV/0!
GTPBP8	6,26098E-05	0,038211092	0,25	0,047	1	1,02683978	0,973861765	5,319148936
CREBL2	0,26859919	0,038138865	0,125	0,066	1	1,026788373	0,973910522	1,893939394
RPS11	0,270238431	0,038120052	0,938	0,793	1	1,026774984	0,973923221	1,182849937
CDC37	0,007884792	0,038000916	0,656	0,293	1	1,026690197	0,97400365	2,23890785
KLRG1	0,082317662	0,037996402	0,031	0,004	1	1,026686985	0,974006698	7,75
FECH	0,008148126	0,037872719	0,094	0,016	1	1,02659897	0,974090204	5,875
C21orf62	0,004841742	0,037852288	0,031	0	1	1,026584432	0,974103998	#DIV/0!
CDYL2	0,004841742	0,037852288	0,031	0	1	1,026584432	0,974103998	#DIV/0!
FAM149A	0,004841742	0,037852288	0,031	0	1	1,026584432	0,974103998	#DIV/0!
GALR3	0,004841742	0,037852288	0,031	0	1	1,026584432	0,974103998	#DIV/0!
OAS2	0,004841742	0,037852288	0,031	0	1	1,026584432	0,974103998	#DIV/0!

Supplementary Table 1

Differentially expressed genes_adjacent-skin-XL_ comparing SC-Keloid vs. (SC-Myel, SC-Nonmyel)								
**gene**	**p_val**	**avg_log2FC**	**pct.1**	**pct.2**	**p_val_adj**	**Foldchange_UP**	**Foldchange_DOWN**	**Ratio_pct1_pct2**
RGPD8	0,004841742	0,037852288	0,031	0	1	1,026584432	0,974103998	#DIV/0!
RGS4	0,004841742	0,037852288	0,031	0	1	1,026584432	0,974103998	#DIV/0!
TMEM72-AS1	0,004841742	0,037852288	0,031	0	1	1,026584432	0,974103998	#DIV/0!
WDR5B	0,004841742	0,037852288	0,031	0	1	1,026584432	0,974103998	#DIV/0!
ZNF154	0,004841742	0,037852288	0,031	0	1	1,026584432	0,974103998	#DIV/0!
ETFBKMT	0,000524345	0,037843382	0,094	0,008	1	1,026578095	0,974110012	11,75
PLD2	0,002594281	0,037784523	0,094	0,012	1	1,026536214	0,974149754	7,833333333
NUP43	0,084624339	0,037733638	0,062	0,016	1	1,026500008	0,974184113	3,875
ZBTB37	0,00083045	0,037724929	0,156	0,027	1	1,026493811	0,974189995	5,777777778
TDP1	0,018436147	0,037698396	0,094	0,02	1	1,026474932	0,974207912	4,7
SHLD3	0,041848356	0,037693435	0,062	0,012	1	1,026471403	0,974211261	5,166666667
THAP7	0,001622495	0,037660809	0,312	0,098	1	1,02644819	0,974233293	3,183673469
EIF5A2	0,019871239	0,03763399	0,156	0,047	1	1,026429109	0,974251403	3,319148936
LINC00973	0,002263143	0,037596526	0,062	0,004	1	1,026402455	0,974276703	15,5
C9orf116	6,3574E-05	0,037528792	0,062	0	1	1,026354267	0,974322446	#DIV/0!
VAX2	6,3574E-05	0,037528792	0,062	0	1	1,026354267	0,974322446	#DIV/0!
PDCD10	0,007118477	0,037514938	0,375	0,145	1	1,026344411	0,974331803	2,586206897
GAS8	0,041848356	0,037483513	0,062	0,012	1	1,026322055	0,974353026	5,166666667
CCDC181	0,079594089	0,037440311	0,031	0,004	1	1,026291322	0,974382204	7,75
PCSK7	0,003091583	0,037381152	0,25	0,074	1	1,026249239	0,97442216	3,378378378
ACOT8	0,000339359	0,037348865	0,156	0,023	1	1,026226272	0,974443967	6,782608696
FAM110A	0,000524345	0,037301327	0,094	0,008	1	1,026192458	0,974476077	11,75
SMARCAD1	0,000537931	0,037292001	0,219	0,047	1	1,026185824	0,974482376	4,659574468
RAN	0,009740348	0,037269281	0,844	0,457	1	1,026169663	0,974497723	1,846827133
CNOT9	0,000486115	0,037190727	0,281	0,07	1	1,026113791	0,974550785	4,014285714
STARD8	6,3574E-05	0,037185666	0,062	0	1	1,026110191	0,974554204	#DIV/0!
YAE1	0,000103236	0,037136797	0,156	0,02	1	1,026075434	0,974587215	7,8
LINC00937	0,226351275	0,037040612	0,031	0,008	1	1,026007027	0,974652194	3,875
KCNN3	0,226351275	0,036981668	0,031	0,008	1	1,025965108	0,974692016	3,875
PRSS27	0,087959196	0,036959839	0,062	0,016	1	1,025949585	0,974706764	3,875

Supplementary Table 1

Differentially expressed genes_adjacent-skin-XL_ comparing SC-Keloid vs. (SC-Myel, SC-Nonmyel)								
**gene**	**p_val**	**avg_log2FC**	**pct.1**	**pct.2**	**p_val_adj**	**Foldchange_UP**	**Foldchange_DOWN**	**Ratio_pct1_pct2**
METTL4	0,060128123	0,036951212	0,125	0,043	1	1,02594345	0,974712592	2,906976744
NUDT16	8,35459E-06	0,036928842	0,312	0,059	0,176290113	1,025927542	0,974727706	5,288135593
KLC2	0,000200286	0,03689435	0,188	0,031	1	1,025903014	0,974751011	6,064516129
ABCF1	0,006606659	0,036858665	0,312	0,113	1	1,02587764	0,974775121	2,761061947
RHEBL1	0,002594281	0,036835483	0,094	0,012	1	1,025861155	0,974790785	7,833333333
PGM1	0,000112833	0,036830086	0,125	0,012	1	1,025857318	0,974794431	10,41666667
FAM168A	0,0162314	0,03682506	0,25	0,094	1	1,025853744	0,974797827	2,659574468
BTF3	0,453673081	0,036824398	0,938	0,703	1	1,025853273	0,974798274	1,33428165
TMOD1	0,226351275	0,036797972	0,031	0,008	1	1,025834483	0,97481613	3,875
ZNF737	0,221757959	0,036717307	0,062	0,023	1	1,025777127	0,974870636	2,695652174
BAG5	0,000112855	0,036712123	0,344	0,086	1	1,025773441	0,974874139	4
CSMD2	0,002594281	0,036624482	0,094	0,012	1	1,025711129	0,974933362	7,833333333
APBA3	0,790352492	0,036624155	0,031	0,023	1	1,025710896	0,974933584	1,347826087
FREM2	0,01796492	0,036608583	0,219	0,078	1	1,025699825	0,974944107	2,807692308
CHFR	0,000426857	0,036464724	0,188	0,035	1	1,025597552	0,975041328	5,371428571
LRRC59	0,000172863	0,036463186	0,406	0,113	1	1,025596459	0,975042368	3,592920354
PAX8	0,380266129	0,036398007	0,031	0,012	1	1,025550125	0,97508642	2,583333333
PEX11B	0,041848356	0,036396746	0,062	0,012	1	1,025549229	0,975087272	5,166666667
PIGW	4,80439E-05	0,036362654	0,094	0,004	1	1,025524994	0,975110315	23,5
COL19A1	6,3574E-05	0,036269439	0,062	0	1	1,025458735	0,97517332	#DIV/0!
ZBTB9	6,3574E-05	0,036269439	0,062	0	1	1,025458735	0,97517332	#DIV/0!
NIPA1	5,12603E-06	0,036261822	0,188	0,02	0,108164307	1,025453322	0,975178469	9,4
SANBR	0,002096899	0,036255534	0,156	0,031	1	1,025448852	0,975182719	5,032258065
CENPA	0,082317662	0,036238347	0,031	0,004	1	1,025436636	0,975194337	7,75
SATB1	0,000371522	0,036231507	0,281	0,07	1	1,025431774	0,97519896	4,014285714
SCCPDH	0,009328862	0,036226929	0,75	0,352	1	1,02542852	0,975202055	2,130681818
CALML5	0,221475664	0,036172797	0,031	0,008	1	1,025390045	0,975238647	3,875
CYB5D2	1,49244E-05	0,035978404	0,219	0,031	0,314919708	1,02525189	0,975370062	7,064516129
SGPP1	0,059959447	0,035882245	0,094	0,027	1	1,025183558	0,975435074	3,481481481
RPL27A	0,371142822	0,035817396	0,969	0,734	1	1,025137477	0,975478921	1,320163488

Supplementary Table 1

Differentially expressed genes_adjacent-skin-XL_ comparing SC-Keloid vs. (SC-Myel, SC-Nonmyel)								
**gene**	**p_val**	**avg_log2FC**	**pct.1**	**pct.2**	**p_val_adj**	**Foldchange_UP**	**Foldchange_DOWN**	**Ratio_pct1_pct2**
SLC7A11	0,002263143	0,035795081	0,062	0,004	1	1,02512162	0,97549401	15,5
NDOR1	0,002594281	0,03579081	0,094	0,012	1	1,025118585	0,975496898	7,833333333
PIFO	0,004841742	0,035779411	0,031	0	1	1,025110486	0,975504605	#DIV/0!
AC009228.1	0,004841742	0,035773733	0,031	0	1	1,025106451	0,975508445	#DIV/0!
EFNB3	0,004841742	0,035773733	0,031	0	1	1,025106451	0,975508445	#DIV/0!
ENO4	0,004841742	0,035773733	0,031	0	1	1,025106451	0,975508445	#DIV/0!
IDH1-AS1	0,004841742	0,035773733	0,031	0	1	1,025106451	0,975508445	#DIV/0!
LHB	0,004841742	0,035773733	0,031	0	1	1,025106451	0,975508445	#DIV/0!
MEDAG	0,004841742	0,035773733	0,031	0	1	1,025106451	0,975508445	#DIV/0!
MGST1	0,004841742	0,035773733	0,031	0	1	1,025106451	0,975508445	#DIV/0!
PLA2R1	0,004841742	0,035773733	0,031	0	1	1,025106451	0,975508445	#DIV/0!
STXBP5L	0,004841742	0,035773733	0,031	0	1	1,025106451	0,975508445	#DIV/0!
TLR7	0,004841742	0,035773733	0,031	0	1	1,025106451	0,975508445	#DIV/0!
TM6SF2	0,004841742	0,035773733	0,031	0	1	1,025106451	0,975508445	#DIV/0!
TRIM7	0,004841742	0,035773733	0,031	0	1	1,025106451	0,975508445	#DIV/0!
KPNA2	0,001701754	0,035729131	0,375	0,125	1	1,02507476	0,975538604	3
PGF	0,040857201	0,035720231	0,062	0,012	1	1,025068436	0,975544622	5,166666667
VPS4B	0,001700084	0,035710592	0,281	0,082	1	1,025061588	0,97555114	3,426829268
COPG1	0,00052757	0,035709326	0,219	0,047	1	1,025060688	0,975551996	4,659574468
C7orf25	0,008148126	0,035692271	0,094	0,016	1	1,02504857	0,975563528	5,875
SLC6A6	0,098751938	0,035645528	0,094	0,031	1	1,025015359	0,975595137	3,032258065
NACC1	0,000112833	0,03553901	0,125	0,012	1	1,024939683	0,97566717	10,41666667
MTMR9	0,013518555	0,035504627	0,188	0,059	1	1,024915256	0,975690423	3,186440678
CHCHD4	0,527450503	0,035368155	0,031	0,016	1	1,024818308	0,975782724	1,9375
PEX12	0,082317662	0,03534806	0,031	0,004	1	1,024804034	0,975796315	7,75
TGFB2	0,015983649	0,035319882	0,125	0,031	1	1,024784019	0,975815374	4,032258065
HSF4	0,082317662	0,035276443	0,031	0,004	1	1,024753163	0,975844755	7,75
DAZAP1	0,00575081	0,035273663	0,344	0,125	1	1,024751188	0,975846636	2,752
SENP6	0,000301561	0,035254289	0,469	0,152	1	1,024737427	0,975859741	3,085526316
ZNF724	0,041848356	0,035212942	0,062	0,012	1	1,024708059	0,975887709	5,166666667

Supplementary Table 1

Differentially expressed genes_adjacent-skin-XL_ comparing SC-Keloid vs. (SC-Myel, SC-Nonmyel)								
**gene**	**p_val**	**avg_log2FC**	**pct.1**	**pct.2**	**p_val_adj**	**Foldchange_UP**	**Foldchange_DOWN**	**Ratio_pct1_pct2**
CMTR2	0,000476445	0,03514806	0,188	0,035	1	1,024661976	0,975931598	5,371428571
DTX2	0,004180266	0,035046288	0,125	0,023	1	1,024589696	0,976000446	5,434782609
RAB28	0,001700084	0,035027423	0,281	0,082	1	1,024576298	0,976013208	3,426829268
ATAD5	0,386325862	0,034976422	0,031	0,012	1	1,024540079	0,976047712	2,583333333
EOLA2	0,001600673	0,034794502	0,125	0,02	1	1,024410895	0,976170797	6,25
ETNK2	0,221475664	0,03463651	0,031	0,008	1	1,024298716	0,976277705	3,875
FBP1	0,082317662	0,034592944	0,031	0,004	1	1,024267785	0,976307187	7,75
MAD1L1	0,02818312	0,03456987	0,156	0,051	1	1,024251404	0,976322802	3,058823529
CLSTN3	0,852091481	0,034540518	0,031	0,039	1	1,024230565	0,976342665	0,794871795
GZF1	0,004180266	0,03448242	0,125	0,023	1	1,02418932	0,976381984	5,434782609
CEP78	0,002053304	0,034479183	0,156	0,031	1	1,024187022	0,976384175	5,032258065
BCL9	0,003304112	0,034392505	0,188	0,047	1	1,02412549	0,976442838	4
DNAJC22	0,082317662	0,034348557	0,031	0,004	1	1,024094293	0,976472584	7,75
BAZ1A	0,000954735	0,034266344	0,344	0,105	1	1,024035936	0,97652823	3,276190476
RANBP6	0,041848356	0,033998236	0,062	0,012	1	1,023845649	0,976709723	5,166666667
FAM76A	0,026750634	0,033971318	0,188	0,066	1	1,023826545	0,976727947	2,848484848
C8orf88	0,000190919	0,033967929	0,188	0,031	1	1,02382414	0,976730242	6,064516129
BTN2A2	0,000112833	0,033756642	0,125	0,012	1	1,02367421	0,976873297	10,41666667
HNRNPLL	0,003601587	0,033736382	0,188	0,047	1	1,023659834	0,976887015	4
ST13	0,046407115	0,033646093	0,719	0,379	1	1,023595772	0,976948154	1,897097625
PMPCA	2,87896E-05	0,033549179	0,312	0,066	0,607490199	1,023527014	0,977013783	4,727272727
GCAT	0,040857201	0,033532566	0,062	0,012	1	1,023515227	0,977025035	5,166666667
RHOBTB2	0,002594281	0,033519434	0,094	0,012	1	1,023505911	0,977033928	7,833333333
AC007879.2	0,004841742	0,033490869	0,031	0	1	1,023485646	0,977053273	#DIV/0!
BDNF	0,004841742	0,033490869	0,031	0	1	1,023485646	0,977053273	#DIV/0!
BNC2	0,004841742	0,033490869	0,031	0	1	1,023485646	0,977053273	#DIV/0!
CRYBA2	0,004841742	0,033490869	0,031	0	1	1,023485646	0,977053273	#DIV/0!
EPSTI1	0,004841742	0,033490869	0,031	0	1	1,023485646	0,977053273	#DIV/0!
IQUB	0,004841742	0,033490869	0,031	0	1	1,023485646	0,977053273	#DIV/0!
LINC00871	0,004841742	0,033490869	0,031	0	1	1,023485646	0,977053273	#DIV/0!

Supplementary Table 1

Differentially expressed genes_adjacent-skin-XL_ comparing SC-Keloid vs. (SC-Myel, SC-Nonmyel)								
**gene**	**p_val**	**avg_log2FC**	**pct.1**	**pct.2**	**p_val_adj**	**Foldchange_UP**	**Foldchange_DOWN**	**Ratio_pct1_pct2**
POU2F2	0,004841742	0,033490869	0,031	0	1	1,023485646	0,977053273	#DIV/0!
RAD54B	0,004841742	0,033490869	0,031	0	1	1,023485646	0,977053273	#DIV/0!
TLL1	0,004841742	0,033490869	0,031	0	1	1,023485646	0,977053273	#DIV/0!
TPRG1	0,004841742	0,033490869	0,031	0	1	1,023485646	0,977053273	#DIV/0!
ULBP3	0,004841742	0,033490869	0,031	0	1	1,023485646	0,977053273	#DIV/0!
ZNF304	0,004841742	0,033490869	0,031	0	1	1,023485646	0,977053273	#DIV/0!
WSCD1	0,082317662	0,033441065	0,031	0,004	1	1,023450314	0,977087003	7,75
MRPS11	0,000245551	0,033342837	0,312	0,078	1	1,023380634	0,977153531	4
MED25	0,093336433	0,033306113	0,094	0,031	1	1,023354584	0,977178405	3,032258065
CDK14	0,036158386	0,033274102	0,094	0,023	1	1,023331877	0,977200088	4,086956522
MED7	0,001642148	0,033267059	0,125	0,02	1	1,023326882	0,977204858	6,25
TTI2	0,008148126	0,033191788	0,094	0,016	1	1,023273492	0,977255844	5,875
FAM66C	0,221475664	0,033191722	0,031	0,008	1	1,023273445	0,977255889	3,875
PIGN	0,028712073	0,033158039	0,125	0,035	1	1,023249555	0,977278705	3,571428571
RIOK2	0,0057801	0,033110993	0,188	0,051	1	1,023216188	0,977310574	3,68627451
PFKM	0,002010541	0,033019398	0,156	0,031	1	1,023151226	0,977372625	5,032258065
SNX12	0,016562293	0,032999425	0,125	0,031	1	1,023137062	0,977386156	4,032258065
ERCC3	0,002005544	0,032865975	0,188	0,043	1	1,023042426	0,977476569	4,372093023
ZNF623	0,461954663	0,032660667	0,062	0,035	1	1,022896848	0,977615682	1,771428571
FGFBP1	0,777381931	0,032645293	0,031	0,023	1	1,022885948	0,9776261	1,347826087
KCTD5	0,000911755	0,032590134	0,156	0,027	1	1,02284684	0,977663478	5,777777778
ORMDL1	0,179626713	0,032573541	0,438	0,254	1	1,022835076	0,977674723	1,724409449
PARN	0,01390071	0,03254649	0,188	0,059	1	1,022815898	0,977693055	3,186440678
SPATS2	0,043915019	0,032522016	0,125	0,039	1	1,022798547	0,977709641	3,205128205
LRRTM2	0,179436467	0,032480803	0,094	0,039	1	1,02276933	0,977737571	2,41025641
AP5B1	0,150155729	0,032436117	0,062	0,02	1	1,022737651	0,977767856	3,1
CDH13	0,000623439	0,032367814	0,344	0,102	1	1,022689231	0,977814149	3,37254902
ZNF395	0,133058992	0,032341835	0,094	0,035	1	1,022670816	0,977831757	2,685714286
GJA4	0,082317662	0,032324022	0,031	0,004	1	1,022658189	0,97784383	7,75
RCVRN	6,3574E-05	0,032299906	0,062	0	1	1,022641094	0,977860176	#DIV/0!

Supplementary Table 1

Differentially expressed genes_adjacent-skin-XL_ comparing SC-Keloid vs. (SC-Myel, SC-Nonmyel)								
**gene**	**p_val**	**avg_log2FC**	**pct.1**	**pct.2**	**p_val_adj**	**Foldchange_UP**	**Foldchange_DOWN**	**Ratio_pct1_pct2**
KDM5B	0,017516805	0,032249573	0,281	0,109	1	1,022605417	0,977894292	2,577981651
TOP3B	0,041848356	0,032210366	0,062	0,012	1	1,022577626	0,977920868	5,166666667
NUP54	0,00014425	0,032192337	0,344	0,09	1	1,022564847	0,977933089	3,822222222
SMPD2	0,041848356	0,032093231	0,062	0,012	1	1,022494605	0,97800027	5,166666667
SLC35F2	0,226351275	0,032087443	0,031	0,008	1	1,022490503	0,978004194	3,875
KRBA2	0,221475664	0,032043602	0,031	0,008	1	1,022459432	0,978033914	3,875
GMEB1	0,226917872	0,031964087	0,094	0,043	1	1,022403079	0,978087821	2,186046512
ZNF217	0,05670394	0,031818179	0,156	0,059	1	1,022299683	0,978186745	2,644067797
WDR55	0,001642148	0,031803996	0,125	0,02	1	1,022289634	0,978196362	6,25
GBA2	1,53257E-05	0,031802975	0,219	0,031	0,323387973	1,02228891	0,978197054	7,064516129
FANCA	0,002263143	0,03176711	0,062	0,004	1	1,022263496	0,978221372	15,5
TYW1	0,087959196	0,031697567	0,062	0,016	1	1,022214221	0,978268527	3,875
MARK4	0,041443265	0,031671771	0,125	0,039	1	1,022195943	0,978286019	3,205128205
TRIB3	0,000524345	0,031600241	0,094	0,008	1	1,022145264	0,978334524	11,75
MATN3	0,000339359	0,031535338	0,156	0,023	1	1,022099281	0,978378538	6,782608696
STAU1	0,004796303	0,031481677	0,5	0,199	1	1,022061265	0,978414929	2,512562814
SUFU	4,80439E-05	0,031441994	0,094	0,004	1	1,022033152	0,978441842	23,5
BORA	0,004841742	0,031409513	0,031	0	1	1,022010142	0,978463871	#DIV/0!
CDCA7	0,004841742	0,031409513	0,031	0	1	1,022010142	0,978463871	#DIV/0!
DDIAS	0,004841742	0,031409513	0,031	0	1	1,022010142	0,978463871	#DIV/0!
DSN1	0,004841742	0,031409513	0,031	0	1	1,022010142	0,978463871	#DIV/0!
EFCAB13	0,004841742	0,031409513	0,031	0	1	1,022010142	0,978463871	#DIV/0!
GALNT15	0,004841742	0,031409513	0,031	0	1	1,022010142	0,978463871	#DIV/0!
GINS4	0,004841742	0,031409513	0,031	0	1	1,022010142	0,978463871	#DIV/0!
GPR162	0,004841742	0,031409513	0,031	0	1	1,022010142	0,978463871	#DIV/0!
H3C1	0,004841742	0,031409513	0,031	0	1	1,022010142	0,978463871	#DIV/0!
KHDC1	0,004841742	0,031409513	0,031	0	1	1,022010142	0,978463871	#DIV/0!
KIFC1	0,004841742	0,031409513	0,031	0	1	1,022010142	0,978463871	#DIV/0!
PROB1	0,004841742	0,031409513	0,031	0	1	1,022010142	0,978463871	#DIV/0!
RASSF7	0,004841742	0,031409513	0,031	0	1	1,022010142	0,978463871	#DIV/0!

Supplementary Table 1

Differentially expressed genes_adjacent-skin-XL_ comparing SC-Keloid vs. (SC-Myel, SC-Nonmyel)								
**gene**	**p_val**	**avg_log2FC**	**pct.1**	**pct.2**	**p_val_adj**	**Foldchange_UP**	**Foldchange_DOWN**	**Ratio_pct1_pct2**
RNF39	0,004841742	0,031409513	0,031	0	1	1,022010142	0,978463871	#DIV/0!
SGO1	0,004841742	0,031409513	0,031	0	1	1,022010142	0,978463871	#DIV/0!
ZNF257	0,004841742	0,031409513	0,031	0	1	1,022010142	0,978463871	#DIV/0!
ZNF488	0,004841742	0,031409513	0,031	0	1	1,022010142	0,978463871	#DIV/0!
ZNF7	0,000911755	0,031373381	0,156	0,027	1	1,021984546	0,978488377	5,777777778
LSM12	0,001801849	0,031282566	0,344	0,109	1	1,021920216	0,978549973	3,155963303
HDGFL3	0,000237136	0,031220699	0,219	0,043	1	1,021876394	0,978591937	5,093023256
DLG3	0,668867248	0,031163776	0,031	0,02	1	1,021836076	0,978630549	1,55
SLC39A11	0,008148126	0,03107802	0,094	0,016	1	1,021775338	0,978688722	5,875
MBTPS2	0,008607623	0,031064839	0,125	0,027	1	1,021766003	0,978697664	4,62962963
SLC45A4	0,002263143	0,031049819	0,062	0,004	1	1,021755366	0,978707853	15,5
ALKBH8	0,086278754	0,030954459	0,062	0,016	1	1,021687831	0,978772546	3,875
ABCB9	0,004841742	0,030945964	0,031	0	1	1,021681815	0,97877831	#DIV/0!
ADM2	0,004841742	0,030945964	0,031	0	1	1,021681815	0,97877831	#DIV/0!
C2CD6	0,004841742	0,030945964	0,031	0	1	1,021681815	0,97877831	#DIV/0!
COCH	0,004841742	0,030945964	0,031	0	1	1,021681815	0,97877831	#DIV/0!
MAPK12	0,004841742	0,030945964	0,031	0	1	1,021681815	0,97877831	#DIV/0!
PLCB2	0,004841742	0,030945964	0,031	0	1	1,021681815	0,97877831	#DIV/0!
SNX32	0,004841742	0,030945964	0,031	0	1	1,021681815	0,97877831	#DIV/0!
STKLD1	0,004841742	0,030945964	0,031	0	1	1,021681815	0,97877831	#DIV/0!
TNN	0,004841742	0,030945964	0,031	0	1	1,021681815	0,97877831	#DIV/0!
TNNI3	0,004841742	0,030945964	0,031	0	1	1,021681815	0,97877831	#DIV/0!
ZNF174	0,004841742	0,030945964	0,031	0	1	1,021681815	0,97877831	#DIV/0!
TBRG4	0,00347979	0,030922577	0,188	0,047	1	1,021665253	0,978794176	4
PRIM1	0,008148126	0,030864319	0,094	0,016	1	1,021623998	0,978833702	5,875
C6orf89	0,00041116	0,03076718	0,281	0,07	1	1,021555213	0,978899611	4,014285714
ZNF628	0,008607623	0,030747605	0,125	0,027	1	1,021541352	0,978912893	4,62962963
MTMR6	0,002043075	0,03067287	0,188	0,043	1	1,021488435	0,978963605	4,372093023
TAF3	0,002025682	0,030660726	0,25	0,07	1	1,021479836	0,978971845	3,571428571
CCNJ	0,002263143	0,030656271	0,062	0,004	1	1,021476682	0,978974868	15,5

Supplementary Table 1

Differentially expressed genes_adjacent-skin-XL_ comparing SC-Keloid vs. (SC-Myel, SC-Nonmyel)								
**gene**	**p_val**	**avg_log2FC**	**pct.1**	**pct.2**	**p_val_adj**	**Foldchange_UP**	**Foldchange_DOWN**	**Ratio_pct1_pct2**
OAZ2	0,003916378	0,030607778	0,375	0,137	1	1,021442348	0,979007774	2,737226277
SCAMP4	6,83245E-05	0,030581787	0,344	0,082	1	1,021423946	0,979025412	4,195121951
C9orf78	0,000581767	0,03053573	0,438	0,141	1	1,021391339	0,979056667	3,106382979
NDUFA4L2	0,041848356	0,030371354	0,062	0,012	1	1,021274971	0,979168224	5,166666667
BUB1	0,004841742	0,030352177	0,031	0	1	1,021261396	0,97918124	#DIV/0!
BUB1B	0,004841742	0,030352177	0,031	0	1	1,021261396	0,97918124	#DIV/0!
CORT	0,004841742	0,030352177	0,031	0	1	1,021261396	0,97918124	#DIV/0!
H2AC12	0,004841742	0,030352177	0,031	0	1	1,021261396	0,97918124	#DIV/0!
KIF2C	0,004841742	0,030352177	0,031	0	1	1,021261396	0,97918124	#DIV/0!
MND1	0,004841742	0,030352177	0,031	0	1	1,021261396	0,97918124	#DIV/0!
NEXN	0,004841742	0,030352177	0,031	0	1	1,021261396	0,97918124	#DIV/0!
PLK4	0,004841742	0,030352177	0,031	0	1	1,021261396	0,97918124	#DIV/0!
RBPMS2	0,004841742	0,030352177	0,031	0	1	1,021261396	0,97918124	#DIV/0!
SUV39H1	0,004841742	0,030352177	0,031	0	1	1,021261396	0,97918124	#DIV/0!
TBC1D19	0,000190919	0,030341949	0,188	0,031	1	1,021254155	0,979188182	6,064516129
DENND1B	0,004180266	0,030293525	0,125	0,023	1	1,021219878	0,979221049	5,434782609
PANK2	0,007761791	0,030198304	0,25	0,082	1	1,021152478	0,979285681	3,048780488
ZNF697	0,150155729	0,030198133	0,062	0,02	1	1,021152357	0,979285798	3,1
CEP131	0,065070981	0,030184395	0,125	0,043	1	1,021142633	0,979295123	2,906976744
NAP1L5	0,004180266	0,030177881	0,125	0,023	1	1,021138022	0,979299545	5,434782609
FLRT1	0,039505443	0,030081876	0,156	0,055	1	1,021070072	0,979364715	2,836363636
HMSD	4,80439E-05	0,030062219	0,094	0,004	1	1,02105616	0,979378059	23,5
GPBP1L1	0,000475965	0,030041662	0,25	0,059	1	1,021041611	0,979392014	4,237288136
FAM219B	0,040519001	0,02997637	0,156	0,055	1	1,020995403	0,97943634	2,836363636
EHHADH	0,226351275	0,029970161	0,031	0,008	1	1,020991009	0,979440555	3,875
IFT140	0,380266129	0,029918917	0,031	0,012	1	1,020954744	0,979475345	2,583333333
EPN1	0,001123583	0,029915101	0,344	0,105	1	1,020952043	0,979477936	3,276190476
GEMIN7	0,008607623	0,029870744	0,125	0,027	1	1,020920654	0,979508051	4,62962963
SYT17	0,226351275	0,029818755	0,031	0,008	1	1,020883864	0,97954335	3,875
IL4R	4,80439E-05	0,029799497	0,094	0,004	1	1,020870237	0,979556425	23,5

Supplementary Table 1

Differentially expressed genes_adjacent-skin-XL_ comparing SC-Keloid vs. (SC-Myel, SC-Nonmyel)								
**gene**	**p_val**	**avg_log2FC**	**pct.1**	**pct.2**	**p_val_adj**	**Foldchange_UP**	**Foldchange_DOWN**	**Ratio_pct1_pct2**
INTS7	0,226351275	0,029682339	0,031	0,008	1	1,020787338	0,979635976	3,875
EVL	8,97778E-06	0,029613335	0,25	0,039	0,189440096	1,020738515	0,979682833	6,41025641
DEPDC1	6,3574E-05	0,029582288	0,062	0	1	1,020716549	0,979703916	#DIV/0!
KLHL23	0,082317662	0,029567451	0,031	0,004	1	1,020706051	0,979713992	7,75
NDC80	0,082317662	0,029466032	0,031	0,004	1	1,0206343	0,979782866	7,75
RNF19A	0,000163225	0,029405246	0,438	0,129	1	1,020591298	0,979824149	3,395348837
POLR2D	0,00399666	0,029349282	0,125	0,023	1	1,020551709	0,979862158	5,434782609
DMAP1	3,63697E-05	0,029329728	0,312	0,066	0,767438061	1,020537877	0,979875439	4,727272727
C6orf223	0,082317662	0,029281811	0,031	0,004	1	1,020503981	0,979907985	7,75
GNA15	0,082317662	0,029280113	0,031	0,004	1	1,02050278	0,979909138	7,75
OGFOD2	0,002263143	0,029272229	0,062	0,004	1	1,020497203	0,979914493	15,5
L3MBTL2	0,002043075	0,029254252	0,188	0,043	1	1,020484488	0,979926703	4,372093023
IPO9	0,043285695	0,029185303	0,125	0,039	1	1,020435718	0,979973537	3,205128205
KIF23	0,082317662	0,029160625	0,031	0,004	1	1,020418263	0,9799903	7,75
TICAM2	0,037506898	0,029073016	0,094	0,023	1	1,020356299	0,980049813	4,086956522
BTBD1	0,000258097	0,029065521	0,25	0,055	1	1,020350998	0,980054905	4,545454545
ZFYVE19	0,019236133	0,029055201	0,094	0,02	1	1,020343699	0,980061915	4,7
ZNF625	0,002263143	0,029013091	0,062	0,004	1	1,020313918	0,980090522	15,5
ECHDC3	0,041848356	0,029006714	0,062	0,012	1	1,020309407	0,980094854	5,166666667
PCDHB10	0,082317662	0,028995705	0,031	0,004	1	1,020301622	0,980102333	7,75
SIRT6	0,000537931	0,028965837	0,219	0,047	1	1,020280499	0,980122624	4,659574468
DHX58	0,300902654	0,028871007	0,062	0,027	1	1,020213436	0,980187051	2,296296296
BNIP1	0,002096899	0,028840053	0,156	0,031	1	1,020191548	0,980208082	5,032258065
PARP3	0,001600673	0,028839136	0,125	0,02	1	1,020190899	0,980208705	6,25
PRMT3	0,008956837	0,028817635	0,125	0,027	1	1,020175695	0,980223314	4,62962963
PDZK1	0,002263143	0,028809947	0,062	0,004	1	1,020170258	0,980228537	15,5
CGREF1	0,082317662	0,02879852	0,031	0,004	1	1,020162178	0,980236301	7,75
NQO1	0,000103236	0,028785752	0,156	0,02	1	1,02015315	0,980244976	7,8
KCNC3	0,002263143	0,028753629	0,062	0,004	1	1,020130435	0,980266803	15,5
RTKN	0,001887375	0,02871455	0,406	0,141	1	1,020102803	0,980293356	2,879432624

Supplementary Table 1

Differentially expressed genes_adjacent-skin-XL_ comparing SC-Keloid vs. (SC-Myel, SC-Nonmyel)								
**gene**	**p_val**	**avg_log2FC**	**pct.1**	**pct.2**	**p_val_adj**	**Foldchange_UP**	**Foldchange_DOWN**	**Ratio_pct1_pct2**
AP4M1	0,008148126	0,028695873	0,094	0,016	1	1,020089597	0,980306047	5,875
ZNF277	0,000248428	0,028683237	0,281	0,066	1	1,020080663	0,980314633	4,257575758
STXBP1	0,020365114	0,028630848	0,188	0,062	1	1,020043621	0,980350232	3,032258065
ITGA4	0,013891036	0,028566227	0,062	0,008	1	1,019997932	0,980394144	7,75
SSH3	0,036158386	0,028459008	0,094	0,023	1	1,01992213	0,980467009	4,086956522
SMIM3	0,013891036	0,028376424	0,062	0,008	1	1,019863748	0,980523135	7,75
ATP6V0A2	0,003663929	0,028331599	0,188	0,047	1	1,019832062	0,980553601	4
RSAD1	0,007736261	0,028284972	0,156	0,039	1	1,019799101	0,980585293	4
NCAPG2	0,000509339	0,02827546	0,125	0,016	1	1,019792377	0,980591758	7,8125
LINC00663	0,082317662	0,028206557	0,031	0,004	1	1,019743674	0,980638592	7,75
RSRC1	0,010891946	0,028146009	0,375	0,152	1	1,019700878	0,980679748	2,467105263
SCAMP2	0,000231507	0,028140662	0,438	0,133	1	1,019697098	0,980683383	3,293233083
GBA	0,000954402	0,028019234	0,188	0,039	1	1,019611277	0,980765928	4,820512821
EIPR1	0,000890764	0,027996737	0,156	0,027	1	1,019595377	0,980781222	5,777777778
SERPINA5	0,082317662	0,027993413	0,031	0,004	1	1,019593028	0,980783482	7,75
TEX2	0,026679107	0,027833923	0,219	0,082	1	1,019480318	0,980891913	2,670731707
RXRB	0,007020064	0,027830472	0,281	0,098	1	1,019477879	0,98089426	2,867346939
MORN4	2,40115E-05	0,027753074	0,156	0,016	0,506667402	1,019423188	0,980946884	9,75
SARS2	0,018832425	0,02773321	0,094	0,02	1	1,019409151	0,980960391	4,7
DNASE1L1	0,002263143	0,027719765	0,062	0,004	1	1,019399652	0,980969533	15,5
SPIN4	0,041848356	0,027619209	0,062	0,012	1	1,019328602	0,981037909	5,166666667
LINC00672	0,226351275	0,027530338	0,031	0,008	1	1,019265812	0,981098344	3,875
TACC2	0,013145712	0,027524983	0,188	0,059	1	1,019262029	0,981101985	3,186440678
SLC25A20	0,040857201	0,027500946	0,062	0,012	1	1,019245047	0,981118332	5,166666667
LRR1	0,001642148	0,02749423	0,125	0,02	1	1,019240302	0,981122899	6,25
CCT4	0,004661349	0,027351489	0,688	0,301	1	1,019139463	0,981219976	2,285714286
NIPSNAP2	0,000596865	0,027263613	0,375	0,113	1	1,019077388	0,981279745	3,318584071
TCN2	0,002263143	0,027237309	0,062	0,004	1	1,019058808	0,981297637	15,5
LIG1	0,001642148	0,027228643	0,125	0,02	1	1,019052686	0,981303531	6,25
RPS6KC1	0,004087528	0,027189657	0,125	0,023	1	1,019025149	0,98133005	5,434782609

Supplementary Table 1

Differentially expressed genes_adjacent-skin-XL_ comparing SC-Keloid vs. (SC-Myel, SC-Nonmyel)								
**gene**	**p_val**	**avg_log2FC**	**pct.1**	**pct.2**	**p_val_adj**	**Foldchange_UP**	**Foldchange_DOWN**	**Ratio_pct1_pct2**
MAP4K4	0,000524001	0,02717899	0,594	0,215	1	1,019017614	0,981337305	2,762790698
PEBP4	0,082317662	0,026964268	0,031	0,004	1	1,018865961	0,981483373	7,75
SPATA5	0,094667448	0,026929822	0,094	0,031	1	1,018841635	0,981506807	3,032258065
TMEM79	0,013891036	0,026918745	0,062	0,008	1	1,018833813	0,981514343	7,75
C11orf24	0,000910029	0,026888669	0,5	0,176	1	1,018812573	0,981534805	2,840909091
CMPK2	0,013891036	0,026843873	0,062	0,008	1	1,018780939	0,981565282	7,75
CCAR1	1,0872E-05	0,026818455	0,531	0,141	0,229410591	1,01876299	0,981582576	3,765957447
FAM89A	0,016270711	0,026802618	0,125	0,031	1	1,018751807	0,981593351	4,032258065
CYRIB	6,26098E-05	0,026771245	0,25	0,047	1	1,018729653	0,981614697	5,319148936
LRRC14	0,018832425	0,026743724	0,094	0,02	1	1,01871022	0,981633423	4,7
ZNF107	0,226351275	0,026731986	0,031	0,008	1	1,018701931	0,98164141	3,875
GAR1	0,000486986	0,026691575	0,188	0,035	1	1,018673397	0,981668906	5,371428571
SERP1	0,003582014	0,026589165	0,656	0,27	1	1,018601089	0,981738593	2,42962963
FAM200A	0,002263143	0,026512233	0,062	0,004	1	1,018546774	0,981790945	15,5
DZIP1L	0,041848356	0,026500854	0,062	0,012	1	1,01853874	0,981798689	5,166666667
ZNF324B	0,082317662	0,026456137	0,031	0,004	1	1,01850717	0,981829121	7,75
BID	0,018832425	0,02641551	0,094	0,02	1	1,018478489	0,98185677	4,7
UBE2J1	0,000104629	0,026411024	0,281	0,062	1	1,018475322	0,981859823	4,532258065
ZSCAN21	0,002594281	0,026391339	0,094	0,012	1	1,018461426	0,981873221	7,833333333
OCIAD1	0,01039994	0,026298279	0,688	0,32	1	1,018395733	0,981936558	2,15
PSMB8-AS1	4,80439E-05	0,026290667	0,094	0,004	1	1,018390359	0,981941739	23,5
ANO8	0,226351275	0,026256371	0,031	0,008	1	1,01836615	0,981965082	3,875
TBC1D10A	0,000237396	0,026254248	0,344	0,094	1	1,018364652	0,981966527	3,659574468
UGDH-AS1	0,380266129	0,026216014	0,031	0,012	1	1,018337663	0,981992551	2,583333333
DLST	0,000619548	0,026186375	0,312	0,086	1	1,018316743	0,982012725	3,627906977
ZFYVE1	0,001642148	0,02602359	0,125	0,02	1	1,018201848	0,982123536	6,25
C12orf60	0,087959196	0,025839118	0,062	0,016	1	1,018071663	0,982249125	3,875
CWC22	0,010298205	0,025780243	0,219	0,07	1	1,018030118	0,98228921	3,128571429
DENND6A	0,006917668	0,025699015	0,219	0,066	1	1,017972801	0,982344517	3,318181818
CDC7	0,386325862	0,025646438	0,031	0,012	1	1,017935703	0,982380318	2,583333333

Supplementary Table 1

Differentially expressed genes_adjacent-skin-XL_ comparing SC-Keloid vs. (SC-Myel, SC-Nonmyel)								
**gene**	**p_val**	**avg_log2FC**	**pct.1**	**pct.2**	**p_val_adj**	**Foldchange_UP**	**Foldchange_DOWN**	**Ratio_pct1_pct2**
TKT	0,002673937	0,025636713	0,406	0,148	1	1,017928841	0,982386941	2,743243243
USF3	0,06592651	0,025622529	0,125	0,043	1	1,017918833	0,982396599	2,906976744
RGMB-AS1	0,082317662	0,025566269	0,031	0,004	1	1,017879139	0,982434909	7,75
CMBL	0,082317662	0,025525392	0,031	0,004	1	1,017850299	0,982462746	7,75
TRAP1	0,005236307	0,025517167	0,25	0,078	1	1,017844496	0,982468348	3,205128205
SYNJ1	0,145431	0,025499336	0,062	0,02	1	1,017831916	0,98248049	3,1
ZNF337	0,177416998	0,025474994	0,094	0,039	1	1,017814743	0,982497067	2,41025641
SCFD2	0,013891036	0,025427097	0,062	0,008	1	1,017780953	0,982529686	7,75
ARHGAP42	0,012579781	0,02539949	0,156	0,043	1	1,017761477	0,982548488	3,627906977
PMPCB	0,000142904	0,025351326	0,375	0,102	1	1,0177275	0,982581291	3,676470588
ACO2	0,000887986	0,02528525	0,312	0,09	1	1,017680888	0,982626294	3,466666667
TXNDC11	0,000911755	0,025247328	0,156	0,027	1	1,017654138	0,982652124	5,777777778
RNF157	0,041848356	0,025203045	0,062	0,012	1	1,017622903	0,982682286	5,166666667
NCAPH	0,082317662	0,025181741	0,031	0,004	1	1,017607875	0,982696797	7,75
MRPL58	0,016270711	0,025111478	0,125	0,031	1	1,017558316	0,982744658	4,032258065
CASP2	0,090719467	0,025058147	0,094	0,031	1	1,017520702	0,982780987	3,032258065
FAM27C	0,013891036	0,025052152	0,062	0,008	1	1,017516473	0,982785071	7,75
MTIF3	0,000336489	0,025030412	0,375	0,109	1	1,017501141	0,982799881	3,440366972
MCM9	0,013891036	0,025011871	0,062	0,008	1	1,017488064	0,982812511	7,75
SUPT5H	0,031534081	0,024957269	0,344	0,156	1	1,017449556	0,982849709	2,205128205
GUCD1	0,01390071	0,02491284	0,188	0,059	1	1,017418223	0,982879977	3,186440678
OCRL	0,013891036	0,024880518	0,062	0,008	1	1,01739543	0,982901998	7,75
UHMK1	0,056043813	0,024867717	0,156	0,059	1	1,017386402	0,982910719	2,644067797
MTFP1	0,013891036	0,02485794	0,062	0,008	1	1,017379507	0,98291738	7,75
OSCP1	0,002263143	0,024803662	0,062	0,004	1	1,017341232	0,982954361	15,5
GSS	0,000173407	0,024733452	0,188	0,031	1	1,017291723	0,983002198	6,064516129
SLU7	0,000657584	0,024705989	0,406	0,129	1	1,017272358	0,983020911	3,147286822
ITPR3	0,001087388	0,024640337	0,469	0,164	1	1,017226067	0,983065646	2,859756098
ZBED5	0,018341076	0,024576733	0,188	0,062	1	1,017181222	0,983108987	3,032258065
BOLA3-AS1	0,084624339	0,024561557	0,062	0,016	1	1,017170522	0,983119328	3,875

Supplementary Table 1

Differentially expressed genes_adjacent-skin-XL_ comparing SC-Keloid vs. (SC-Myel, SC-Nonmyel)								
**gene**	**p_val**	**avg_log2FC**	**pct.1**	**pct.2**	**p_val_adj**	**Foldchange_UP**	**Foldchange_DOWN**	**Ratio_pct1_pct2**
ZNF251	0,019236133	0,024548591	0,094	0,02	1	1,01716138	0,983128164	4,7
ZNF468	0,226351275	0,024487481	0,031	0,008	1	1,017118296	0,983169808	3,875
DGKH	0,013708456	0,024450951	0,188	0,059	1	1,017092541	0,983194704	3,186440678
VASH2	0,002263143	0,024377983	0,062	0,004	1	1,017041101	0,983244433	15,5
FOXQ1	0,082317662	0,024316491	0,031	0,004	1	1,016997752	0,983286342	7,75
EHD3	0,082317662	0,024308762	0,031	0,004	1	1,016992304	0,98329161	7,75
ZNF470	0,041848356	0,024257564	0,062	0,012	1	1,016956214	0,983326505	5,166666667
RMC1	0,018436147	0,024172611	0,094	0,02	1	1,016896332	0,98338441	4,7
MTRES1	0,131388145	0,024165947	0,094	0,035	1	1,016891635	0,983388953	2,685714286
APOBEC3H	0,082317662	0,024148678	0,031	0,004	1	1,016879463	0,983400724	7,75
MRPS5	4,10457E-05	0,024112578	0,406	0,102	0,866106149	1,016854019	0,983425331	3,980392157
CANX	0,044634338	0,024112359	0,781	0,418	1	1,016853864	0,983425481	1,868421053
AL133481.1	0,004841742	0,024050284	0,031	0	1	1,016810113	0,983467795	#DIV/0!
ATP2A1	0,004841742	0,024050284	0,031	0	1	1,016810113	0,983467795	#DIV/0!
CD1D	0,004841742	0,024050284	0,031	0	1	1,016810113	0,983467795	#DIV/0!
CMTM8	0,004841742	0,024050284	0,031	0	1	1,016810113	0,983467795	#DIV/0!
CORO2A	0,004841742	0,024050284	0,031	0	1	1,016810113	0,983467795	#DIV/0!
ELF3	0,004841742	0,024050284	0,031	0	1	1,016810113	0,983467795	#DIV/0!
FAAP24	0,004841742	0,024050284	0,031	0	1	1,016810113	0,983467795	#DIV/0!
GLB1L2	0,004841742	0,024050284	0,031	0	1	1,016810113	0,983467795	#DIV/0!
LINC00265	0,004841742	0,024050284	0,031	0	1	1,016810113	0,983467795	#DIV/0!
NOMO2	0,004841742	0,024050284	0,031	0	1	1,016810113	0,983467795	#DIV/0!
POLR3B	0,004841742	0,024050284	0,031	0	1	1,016810113	0,983467795	#DIV/0!
RNF165	0,004841742	0,024050284	0,031	0	1	1,016810113	0,983467795	#DIV/0!
STAMBPL1	0,004841742	0,024050284	0,031	0	1	1,016810113	0,983467795	#DIV/0!
WNT10A	0,004841742	0,024050284	0,031	0	1	1,016810113	0,983467795	#DIV/0!
SMAD2	0,00076551	0,023959543	0,438	0,145	1	1,016746161	0,983529654	3,020689655
CCNJL	0,000103236	0,023940488	0,156	0,02	1	1,016732732	0,983542645	7,8
DCUN1D2	0,008148126	0,023912472	0,094	0,016	1	1,016712988	0,983561745	5,875
TBCCD1	0,041848356	0,023899039	0,062	0,012	1	1,016703521	0,983570903	5,166666667

Supplementary Table 1

Differentially expressed genes_adjacent-skin-XL_ comparing SC-Keloid vs. (SC-Myel, SC-Nonmyel)								
**gene**	**p_val**	**avg_log2FC**	**pct.1**	**pct.2**	**p_val_adj**	**Foldchange_UP**	**Foldchange_DOWN**	**Ratio_pct1_pct2**
LY6K	0,226351275	0,023891253	0,031	0,008	1	1,016698034	0,983576211	3,875
VCPIP1	0,019329956	0,023872779	0,188	0,062	1	1,016685015	0,983588806	3,032258065
HK1	0,002012651	0,02386129	0,312	0,098	1	1,016676918	0,983596639	3,183673469
IBA57	0,226351275	0,023785765	0,031	0,008	1	1,016623697	0,983648132	3,875
ITGA11	0,082317662	0,023733304	0,031	0,004	1	1,01658673	0,9836839	7,75
KYNU	0,082317662	0,023670908	0,031	0,004	1	1,016542764	0,983726445	7,75
SIKE1	1,38769E-05	0,023663377	0,281	0,051	0,292816941	1,016537457	0,983731581	5,509803922
KNSTRN	0,000524345	0,023638913	0,094	0,008	1	1,01652022	0,983748262	11,75
MIEF2	0,082317662	0,023534485	0,031	0,004	1	1,016446643	0,983819473	7,75
SLC26A2	0,019580952	0,023484581	0,156	0,047	1	1,016411484	0,983853504	3,319148936
TMEM237	0,001642148	0,023433528	0,125	0,02	1	1,016375517	0,98388832	6,25
HCCS	0,214636476	0,023369842	0,156	0,082	1	1,016330651	0,983931754	1,902439024
NIF3L1	0,008148126	0,023342606	0,094	0,016	1	1,016311465	0,983950329	5,875
RNF34	5,86746E-05	0,023311562	0,25	0,047	1	1,016289596	0,983971502	5,319148936
KLHDC4	0,003420308	0,023290562	0,188	0,047	1	1,016274802	0,983985825	4
APOO	0,008956837	0,023282895	0,125	0,027	1	1,016269402	0,983991054	4,62962963
BAIAP3	0,004841742	0,023281496	0,031	0	1	1,016268416	0,983992009	#DIV/0!
C17orf97	0,004841742	0,023281496	0,031	0	1	1,016268416	0,983992009	#DIV/0!
DLX5	0,004841742	0,023281496	0,031	0	1	1,016268416	0,983992009	#DIV/0!
FAM86B1	0,004841742	0,023281496	0,031	0	1	1,016268416	0,983992009	#DIV/0!
HOXD4	0,004841742	0,023281496	0,031	0	1	1,016268416	0,983992009	#DIV/0!
RHPN1-AS1	0,004841742	0,023281496	0,031	0	1	1,016268416	0,983992009	#DIV/0!
SAMD15	0,004841742	0,023281496	0,031	0	1	1,016268416	0,983992009	#DIV/0!
SMAGP	0,004841742	0,023281496	0,031	0	1	1,016268416	0,983992009	#DIV/0!
TMEM229B	0,004841742	0,023281496	0,031	0	1	1,016268416	0,983992009	#DIV/0!
ZNF134	0,004841742	0,023281496	0,031	0	1	1,016268416	0,983992009	#DIV/0!
ZNF229	0,004841742	0,023281496	0,031	0	1	1,016268416	0,983992009	#DIV/0!
CYP26B1	0,226351275	0,023269257	0,031	0,008	1	1,016259795	0,984000356	3,875
REXO4	0,000965846	0,023124119	0,219	0,051	1	1,016157562	0,984099354	4,294117647
MAK16	0,037506898	0,0231013	0,094	0,023	1	1,01614149	0,984114919	4,086956522

Supplementary Table 1

Differentially expressed genes_adjacent-skin-XL_ comparing SC-Keloid vs. (SC-Myel, SC-Nonmyel)								
**gene**	**p_val**	**avg_log2FC**	**pct.1**	**pct.2**	**p_val_adj**	**Foldchange_UP**	**Foldchange_DOWN**	**Ratio_pct1_pct2**
SLC25A22	0,000509339	0,02307094	0,125	0,016	1	1,016120106	0,984135629	7,8125
RAB9B	4,80439E-05	0,023057727	0,094	0,004	1	1,0161108	0,984144642	23,5
RAMP2	0,082317662	0,023016362	0,031	0,004	1	1,016081667	0,98417286	7,75
RARRES1	0,226351275	0,02294984	0,031	0,008	1	1,016034816	0,984218241	3,875
ZFP90	0,043285695	0,022901043	0,125	0,039	1	1,016000452	0,984251531	3,205128205
EAF1	0,00011037	0,022892861	0,219	0,039	1	1,015994689	0,984257113	5,615384615
LINGO1	0,082317662	0,022876991	0,031	0,004	1	1,015983513	0,98426794	7,75
DNAJC21	0,008685186	0,022857272	0,406	0,164	1	1,015969627	0,984281394	2,475609756
MR1	0,000524345	0,022824358	0,094	0,008	1	1,015946448	0,98430385	11,75
VPS11	0,097375089	0,022804161	0,094	0,031	1	1,015932226	0,984317629	3,032258065
FAM151B	0,082317662	0,022745288	0,031	0,004	1	1,015890768	0,984357798	7,75
ZNF800	0,037823916	0,022741254	0,25	0,105	1	1,015887928	0,98436055	2,380952381
MRTFB	0,183526587	0,022695354	0,094	0,039	1	1,015855607	0,984391869	2,41025641
DLG1	0,000788946	0,022691208	0,469	0,16	1	1,015852689	0,984394697	2,93125
POP1	0,226351275	0,022683056	0,031	0,008	1	1,015846949	0,98440026	3,875
TACO1	0,013891036	0,022660246	0,062	0,008	1	1,015830887	0,984415824	7,75
ZNF780B	0,150155009	0,022624616	0,062	0,02	1	1,015805799	0,984440137	3,1
ELP6	0,000128726	0,022584013	0,281	0,062	1	1,015777211	0,984467843	4,532258065
QSER1	0,011715823	0,022566485	0,344	0,137	1	1,01576487	0,984479804	2,510948905
SLX4	0,082317662	0,022524001	0,031	0,004	1	1,015734959	0,984508794	7,75
TP53INP1	0,087959196	0,022484052	0,062	0,016	1	1,015706833	0,984536057	3,875
HMGN3-AS1	0,041848356	0,022479899	0,062	0,012	1	1,015703909	0,984538891	5,166666667
TMSB15B	0,013891036	0,022446693	0,062	0,008	1	1,015680531	0,984561552	7,75
PIGG	0,000126424	0,022391951	0,25	0,051	1	1,015641993	0,984598911	4,901960784
NSFL1C	0,000305244	0,02233037	0,406	0,121	1	1,015598641	0,984640939	3,355371901
RBBP7	0,000350429	0,022244977	0,531	0,18	1	1,01553853	0,984699222	2,95
A4GALT	0,004841742	0,022161105	0,031	0	1	1,015479493	0,984756469	#DIV/0!
AC140912.1	0,004841742	0,022161105	0,031	0	1	1,015479493	0,984756469	#DIV/0!
ANKRD2	0,004841742	0,022161105	0,031	0	1	1,015479493	0,984756469	#DIV/0!
ARHGAP28	0,004841742	0,022161105	0,031	0	1	1,015479493	0,984756469	#DIV/0!

Supplementary Table 1

Differentially expressed genes_adjacent-skin-XL_ comparing SC-Keloid vs. (SC-Myel, SC-Nonmyel)								
**gene**	**p_val**	**avg_log2FC**	**pct.1**	**pct.2**	**p_val_adj**	**Foldchange_UP**	**Foldchange_DOWN**	**Ratio_pct1_pct2**
C1orf53	0,004841742	0,022161105	0,031	0	1	1,015479493	0,984756469	#DIV/0!
CRMP1	0,004841742	0,022161105	0,031	0	1	1,015479493	0,984756469	#DIV/0!
DDIT4L	0,004841742	0,022161105	0,031	0	1	1,015479493	0,984756469	#DIV/0!
FAM182B	0,004841742	0,022161105	0,031	0	1	1,015479493	0,984756469	#DIV/0!
FGF22	0,004841742	0,022161105	0,031	0	1	1,015479493	0,984756469	#DIV/0!
GAREM2	0,004841742	0,022161105	0,031	0	1	1,015479493	0,984756469	#DIV/0!
HOXA11	0,004841742	0,022161105	0,031	0	1	1,015479493	0,984756469	#DIV/0!
HOXD1	0,004841742	0,022161105	0,031	0	1	1,015479493	0,984756469	#DIV/0!
LINC02872	0,004841742	0,022161105	0,031	0	1	1,015479493	0,984756469	#DIV/0!
LINC02901	0,004841742	0,022161105	0,031	0	1	1,015479493	0,984756469	#DIV/0!
MCHR1	0,004841742	0,022161105	0,031	0	1	1,015479493	0,984756469	#DIV/0!
SHOX2	0,004841742	0,022161105	0,031	0	1	1,015479493	0,984756469	#DIV/0!
STK33	0,004841742	0,022161105	0,031	0	1	1,015479493	0,984756469	#DIV/0!
SMAP1	0,001108522	0,022013587	0,344	0,105	1	1,015375664	0,984857167	3,276190476
AC092171.2	0,004841742	0,022009639	0,031	0	1	1,015372885	0,984859863	#DIV/0!
AP001437.1	0,004841742	0,022009639	0,031	0	1	1,015372885	0,984859863	#DIV/0!
APOL4	0,004841742	0,022009639	0,031	0	1	1,015372885	0,984859863	#DIV/0!
FCRLB	0,004841742	0,022009639	0,031	0	1	1,015372885	0,984859863	#DIV/0!
GPR35	0,004841742	0,022009639	0,031	0	1	1,015372885	0,984859863	#DIV/0!
H4C9	0,004841742	0,022009639	0,031	0	1	1,015372885	0,984859863	#DIV/0!
IKBKE	0,004841742	0,022009639	0,031	0	1	1,015372885	0,984859863	#DIV/0!
JPH2	0,004841742	0,022009639	0,031	0	1	1,015372885	0,984859863	#DIV/0!
KLHL35	0,004841742	0,022009639	0,031	0	1	1,015372885	0,984859863	#DIV/0!
NANOS3	0,004841742	0,022009639	0,031	0	1	1,015372885	0,984859863	#DIV/0!
PHETA1	0,004841742	0,022009639	0,031	0	1	1,015372885	0,984859863	#DIV/0!
PNPLA3	0,004841742	0,022009639	0,031	0	1	1,015372885	0,984859863	#DIV/0!
PODNL1	0,004841742	0,022009639	0,031	0	1	1,015372885	0,984859863	#DIV/0!
PRICKLE4	0,004841742	0,022009639	0,031	0	1	1,015372885	0,984859863	#DIV/0!
RGS8	0,004841742	0,022009639	0,031	0	1	1,015372885	0,984859863	#DIV/0!
TBC1D2	0,004841742	0,022009639	0,031	0	1	1,015372885	0,984859863	#DIV/0!

Supplementary Table 1

Differentially expressed genes_adjacent-skin-XL_ comparing SC-Keloid vs. (SC-Myel, SC-Nonmyel)								
**gene**	**p_val**	**avg_log2FC**	**pct.1**	**pct.2**	**p_val_adj**	**Foldchange_UP**	**Foldchange_DOWN**	**Ratio_pct1_pct2**
MAPK8IP3	0,08132778	0,021992152	0,188	0,082	1	1,015360577	0,9848718	2,292682927
EEF1AKMT3	0,002263143	0,02196952	0,062	0,004	1	1,01534465	0,98488725	15,5
HAUS3	0,027811527	0,021963367	0,125	0,035	1	1,015340319	0,984891451	3,571428571
LINC01547	0,002263143	0,021960452	0,062	0,004	1	1,015338267	0,984893441	15,5
CYB5R4	0,027811527	0,021910856	0,125	0,035	1	1,015303363	0,9849273	3,571428571
TMLHE	0,007947097	0,021812442	0,094	0,016	1	1,015234107	0,984994489	5,875
C1QTNF1	0,001560165	0,021808845	0,125	0,02	1	1,015231575	0,984996945	6,25
SFXN4	0,087959196	0,021782659	0,062	0,016	1	1,015213148	0,985014823	3,875
RPA2	0,000253221	0,021779867	0,25	0,055	1	1,015211183	0,98501673	4,545454545
SPRYD4	0,002263143	0,021757143	0,062	0,004	1	1,015195193	0,985032245	15,5
GTF2A2	0,00023336	0,021713112	0,531	0,176	1	1,01516421	0,985062309	3,017045455
HOXB4	0,002263143	0,021673375	0,062	0,004	1	1,015136249	0,985089441	15,5
PASK	0,386325862	0,021657914	0,031	0,012	1	1,01512537	0,985099998	2,583333333
MBD4	0,000764302	0,021419898	0,25	0,062	1	1,014957909	0,985262533	4,032258065
ZNF561	0,008148126	0,021413392	0,094	0,016	1	1,014953331	0,985266977	5,875
XPO5	0,041848356	0,021410167	0,062	0,012	1	1,014951062	0,985269179	5,166666667
NPW	0,041848356	0,021319839	0,062	0,012	1	1,014887518	0,985330869	5,166666667
ZNF710	0,226351275	0,021281723	0,031	0,008	1	1,014860704	0,985356902	3,875
RNASEH1-AS1	0,002594281	0,02126304	0,094	0,012	1	1,014847562	0,985369663	7,833333333
CELSR2	0,002560056	0,021134007	0,219	0,059	1	1,0147568	0,985457797	3,711864407
PRRT3	0,000509339	0,021015026	0,125	0,016	1	1,014673115	0,985539072	7,8125
LYRM1	0,016270711	0,020977211	0,125	0,031	1	1,014646519	0,985564905	4,032258065
COIL	0,149643094	0,020963647	0,125	0,055	1	1,014636979	0,985574171	2,272727273
MRPL39	0,000517394	0,020887045	0,219	0,047	1	1,014583107	0,985626503	4,659574468
MT-ND6	0,002263143	0,020884831	0,062	0,004	1	1,01458155	0,985628016	15,5
POLR3F	0,226351275	0,020788142	0,031	0,008	1	1,014513556	0,985694074	3,875
RAB20	0,013891036	0,020778577	0,062	0,008	1	1,014506829	0,98570061	7,75
NOB1	0,030583271	0,020771122	0,188	0,066	1	1,014501587	0,985705703	2,848484848
MED17	0,35458226	0,02076559	0,156	0,098	1	1,014497697	0,985709483	1,591836735
MCM8	0,002594281	0,020660979	0,094	0,012	1	1,014424137	0,98578096	7,833333333

Supplementary Table 1

Differentially expressed genes_adjacent-skin-XL_ comparing SC-Keloid vs. (SC-Myel, SC-Nonmyel)								
**gene**	**p_val**	**avg_log2FC**	**pct.1**	**pct.2**	**p_val_adj**	**Foldchange_UP**	**Foldchange_DOWN**	**Ratio_pct1_pct2**
NGRN	0,001462129	0,020456155	0,594	0,23	1	1,014280127	0,985920924	2,582608696
TRPS1	0,013247448	0,020439868	0,312	0,121	1	1,014268676	0,985932055	2,578512397
TBC1D9	0,380266129	0,020395678	0,031	0,012	1	1,014237609	0,985962255	2,583333333
ZBED6CL	0,226351275	0,020325758	0,031	0,008	1	1,014188456	0,98601004	3,875
CXCL16	4,22524E-05	0,020320274	0,219	0,035	0,891567246	1,014184601	0,986013788	6,257142857
CDKN2C	0,000828263	0,020303244	0,406	0,133	1	1,014172629	0,986025427	3,052631579
DHRS4L2	1,63815E-05	0,020300066	0,312	0,062	0,345665807	1,014170395	0,9860276	5,032258065
ZFYVE27	0,040252231	0,020215719	0,125	0,039	1	1,014111104	0,986085249	3,205128205
KRR1	0,037105165	0,020183452	0,312	0,141	1	1,014088423	0,986107304	2,212765957
SIRT5	0,008148126	0,020059147	0,094	0,016	1	1,01400105	0,986192272	5,875
MOCS3	0,082317662	0,020055355	0,031	0,004	1	1,013998385	0,986194864	7,75
WWC1	1,51098E-05	0,019918264	0,125	0,008	0,318831333	1,013902036	0,986288581	15,625
DAGLB	0,013891036	0,019897519	0,062	0,008	1	1,013887456	0,986302763	7,75
ZCCHC9	3,15754E-06	0,019880654	0,25	0,035	0,066627295	1,013875604	0,986314293	7,142857143
SUSD5	0,082317662	0,019815825	0,031	0,004	1	1,013830046	0,986358615	7,75
CDC42EP5	0,033341196	0,019740807	0,688	0,363	1	1,013777329	0,986409906	1,895316804
PSAP	0,147637695	0,019667276	1	0,773	1	1,01372566	0,986460183	1,293661061
MYL6B	0,000597516	0,019656488	0,344	0,098	1	1,013718081	0,986467559	3,510204082
ZNF286A	0,188205529	0,019635283	0,125	0,059	1	1,013703181	0,986482058	2,118644068
AC007365.1	0,082317662	0,019619122	0,031	0,004	1	1,013691826	0,986493108	7,75
MCCC2	0,000983579	0,019608882	0,219	0,051	1	1,01368463	0,986500111	4,294117647
DOCK8-AS1	0,082317662	0,019528316	0,031	0,004	1	1,013628024	0,986555202	7,75
RAPGEF4	0,082317662	0,019528316	0,031	0,004	1	1,013628024	0,986555202	7,75
CENPJ	0,000112833	0,019522262	0,125	0,012	1	1,01362377	0,986559342	10,41666667
MEPCE	0,041554521	0,019502173	0,156	0,055	1	1,013609656	0,98657308	2,836363636
DHDDS	0,008956837	0,019395968	0,125	0,027	1	1,013535041	0,98664571	4,62962963
PRRC1	0,003420308	0,019390466	0,188	0,047	1	1,013531175	0,986649473	4
CSNK2A3	0,082317662	0,019390218	0,031	0,004	1	1,013531002	0,986649642	7,75
HAPLN2	0,226351275	0,019336226	0,031	0,008	1	1,013493071	0,986686568	3,875
CABCOCO1	0,013891036	0,019304626	0,062	0,008	1	1,013470873	0,98670818	7,75

Supplementary Table 1

Differentially expressed genes_adjacent-skin-XL_ comparing SC-Keloid vs. (SC-Myel, SC-Nonmyel)								
**gene**	**p_val**	**avg_log2FC**	**pct.1**	**pct.2**	**p_val_adj**	**Foldchange_UP**	**Foldchange_DOWN**	**Ratio_pct1_pct2**
SNRK-AS1	0,082317662	0,019225457	0,031	0,004	1	1,013415259	0,986762327	7,75
CDC42	0,18062686	0,01919876	0,812	0,512	1	1,013396506	0,986780588	1,5859375
SLC30A7	0,000905215	0,019153457	0,25	0,062	1	1,013364684	0,986811575	4,032258065
PHYHD1	0,002010541	0,019125777	0,156	0,031	1	1,013345242	0,986830508	5,032258065
AMN1	0,003953072	0,019094444	0,156	0,035	1	1,013323233	0,986851941	4,457142857
SPRTN	0,00011289	0,019069918	0,219	0,039	1	1,013306007	0,986868718	5,615384615
MTRNR2L1	0,062914349	0,019062444	0,094	0,027	1	1,013300758	0,98687383	3,481481481
INTS6-AS1	0,540537634	0,01902189	0,031	0,016	1	1,013272274	0,986901572	1,9375
MFSD8	0,013891036	0,018995879	0,062	0,008	1	1,013254006	0,986919365	7,75
AP5M1	0,003361753	0,018925737	0,188	0,047	1	1,013204744	0,986967349	4
TEDC1	0,139907281	0,018924346	0,094	0,035	1	1,013203767	0,9869683	2,685714286
C4orf46	0,004180266	0,018918116	0,125	0,023	1	1,013199392	0,986972563	5,434782609
TRO	0,013891036	0,018904361	0,062	0,008	1	1,013189732	0,986981972	7,75
COQ2	0,013891036	0,018858264	0,062	0,008	1	1,013157359	0,987013509	7,75
BEND6	0,019236133	0,018851453	0,094	0,02	1	1,013152576	0,987018169	4,7
ERGIC2	0,000596933	0,018837637	0,562	0,207	1	1,013142873	0,987027621	2,714975845
PPP2R5A	0,00208125	0,018780702	0,188	0,043	1	1,013102891	0,987066574	4,372093023
FAM120C	0,015983649	0,018732204	0,125	0,031	1	1,013068835	0,987099756	4,032258065
PDCD2L	0,224763613	0,01867947	0,062	0,023	1	1,013031806	0,987135838	2,695652174
LINC00526	0,145431	0,018645379	0,062	0,02	1	1,013007868	0,987159164	3,1
ACYP2	0,003953072	0,018547748	0,156	0,035	1	1,012939317	0,98722597	4,457142857
ARL14EP	0,131388145	0,01853765	0,094	0,035	1	1,012932227	0,98723288	2,685714286
RWDD2A	0,082317662	0,018503508	0,031	0,004	1	1,012908256	0,987256244	7,75
DGLUCY	0,150155729	0,018443501	0,062	0,02	1	1,012866126	0,987297308	3,1
RASAL2-AS1	0,226351275	0,018383336	0,031	0,008	1	1,012823887	0,987338483	3,875
ARMC6	0,002096899	0,018317545	0,156	0,031	1	1,0127777	0,987383509	5,032258065
NOX4	0,082317662	0,018223644	0,031	0,004	1	1,012711784	0,987447777	7,75
B3GALNT1	0,082317662	0,018166163	0,031	0,004	1	1,012671436	0,987487121	7,75
DNM3OS	0,082317662	0,018116502	0,031	0,004	1	1,012636577	0,987521113	7,75
SHQ1	0,150155729	0,018111201	0,062	0,02	1	1,012632856	0,987524742	3,1

Supplementary Table 1

Differentially expressed genes_adjacent-skin-XL_ comparing SC-Keloid vs. (SC-Myel, SC-Nonmyel)								
**gene**	**p_val**	**avg_log2FC**	**pct.1**	**pct.2**	**p_val_adj**	**Foldchange_UP**	**Foldchange_DOWN**	**Ratio_pct1_pct2**
ACTR3C	0,004841742	0,018000621	0,031	0	1	1,012555243	0,987600436	#DIV/0!
ADTRP	0,004841742	0,018000621	0,031	0	1	1,012555243	0,987600436	#DIV/0!
ASB2	0,004841742	0,018000621	0,031	0	1	1,012555243	0,987600436	#DIV/0!
AVIL	0,004841742	0,018000621	0,031	0	1	1,012555243	0,987600436	#DIV/0!
B3GAT2	0,004841742	0,018000621	0,031	0	1	1,012555243	0,987600436	#DIV/0!
C7orf31	0,004841742	0,018000621	0,031	0	1	1,012555243	0,987600436	#DIV/0!
C9orf135-DT	0,004841742	0,018000621	0,031	0	1	1,012555243	0,987600436	#DIV/0!
CCL26	0,004841742	0,018000621	0,031	0	1	1,012555243	0,987600436	#DIV/0!
DEFB124	0,004841742	0,018000621	0,031	0	1	1,012555243	0,987600436	#DIV/0!
DPYSL4	0,004841742	0,018000621	0,031	0	1	1,012555243	0,987600436	#DIV/0!
EOMES	0,004841742	0,018000621	0,031	0	1	1,012555243	0,987600436	#DIV/0!
HOXD3	0,004841742	0,018000621	0,031	0	1	1,012555243	0,987600436	#DIV/0!
HRH1	0,004841742	0,018000621	0,031	0	1	1,012555243	0,987600436	#DIV/0!
ITGB2	0,004841742	0,018000621	0,031	0	1	1,012555243	0,987600436	#DIV/0!
KRT79	0,004841742	0,018000621	0,031	0	1	1,012555243	0,987600436	#DIV/0!
LINC00519	0,004841742	0,018000621	0,031	0	1	1,012555243	0,987600436	#DIV/0!
LINC00923	0,004841742	0,018000621	0,031	0	1	1,012555243	0,987600436	#DIV/0!
LRRC73	0,004841742	0,018000621	0,031	0	1	1,012555243	0,987600436	#DIV/0!
METTL27	0,004841742	0,018000621	0,031	0	1	1,012555243	0,987600436	#DIV/0!
NAPA-AS1	0,004841742	0,018000621	0,031	0	1	1,012555243	0,987600436	#DIV/0!
PITX1-AS1	0,004841742	0,018000621	0,031	0	1	1,012555243	0,987600436	#DIV/0!
SNPH	0,004841742	0,018000621	0,031	0	1	1,012555243	0,987600436	#DIV/0!
SPRED3	0,004841742	0,018000621	0,031	0	1	1,012555243	0,987600436	#DIV/0!
TNFSF4	0,004841742	0,018000621	0,031	0	1	1,012555243	0,987600436	#DIV/0!
ZNF772	0,004841742	0,018000621	0,031	0	1	1,012555243	0,987600436	#DIV/0!
ZSCAN1	0,004841742	0,018000621	0,031	0	1	1,012555243	0,987600436	#DIV/0!
ZNF563	0,226351275	0,017971227	0,031	0,008	1	1,012534613	0,987620558	3,875
RBPMS	0,140867233	0,017969842	0,25	0,133	1	1,012533641	0,987621507	1,879699248
ZFPL1	0,122440788	0,017937062	0,156	0,07	1	1,012510635	0,987643947	2,228571429
LINC00887	0,226351275	0,017936837	0,031	0,008	1	1,012510478	0,987644101	3,875

Supplementary Table 1

Differentially expressed genes_adjacent-skin-XL_ comparing SC-Keloid vs. (SC-Myel, SC-Nonmyel)								
**gene**	**p_val**	**avg_log2FC**	**pct.1**	**pct.2**	**p_val_adj**	**Foldchange_UP**	**Foldchange_DOWN**	**Ratio_pct1_pct2**
C8orf82	0,003070984	0,01787635	0,312	0,102	1	1,012468027	0,98768551	3,058823529
DNAJB14	9,23407E-05	0,01783182	0,5	0,148	1	1,012436777	0,987715997	3,378378378
ZNF586	0,226351275	0,017793413	0,031	0,008	1	1,012409825	0,987742291	3,875
AGAP1	0,015772854	0,017781032	0,344	0,145	1	1,012401136	0,987750768	2,372413793
DDB1	6,02217E-05	0,017765493	0,469	0,133	1	1,012390232	0,987761407	3,526315789
CCDC127	0,003601563	0,017717913	0,188	0,047	1	1,012356844	0,987793984	4
RNF20	0,000509339	0,017679517	0,125	0,016	1	1,012329902	0,987820273	7,8125
RALGAPB	0,002774205	0,017673626	0,219	0,059	1	1,012325768	0,987824307	3,711864407
PSMD6-AS2	0,386325862	0,017647537	0,031	0,012	1	1,012307461	0,987842171	2,583333333
ST7	0,041848356	0,017572588	0,062	0,012	1	1,012254873	0,987893491	5,166666667
ANKRD16	0,082317662	0,017516927	0,031	0,004	1	1,01221582	0,987931606	7,75
ENTPD7	0,041848356	0,01742236	0,062	0,012	1	1,012149472	0,987996366	5,166666667
MIX23	0,008270631	0,017373069	0,125	0,027	1	1,012114892	0,988030122	4,62962963
PHF7	0,386325862	0,017357411	0,031	0,012	1	1,012103907	0,988040846	2,583333333
OSBPL8	0,000480998	0,017354847	0,5	0,168	1	1,012102108	0,988042601	2,976190476
DTL	0,082317662	0,017344618	0,031	0,004	1	1,012094932	0,988049607	7,75
ZNRF1	4,43697E-05	0,017337092	0,219	0,035	0,936244219	1,012089652	0,988054761	6,257142857
CLEC18A	0,082317662	0,017336258	0,031	0,004	1	1,012089068	0,988055332	7,75
MINDY4	0,082317662	0,017336258	0,031	0,004	1	1,012089068	0,988055332	7,75
ZDHHC20	6,53913E-05	0,017281054	0,281	0,059	1	1,012050341	0,988093141	4,762711864
LINC01001	0,540537634	0,017260964	0,031	0,016	1	1,012036248	0,9881069	1,9375
SAMD10	0,226351275	0,017248336	0,031	0,008	1	1,01202739	0,988115549	3,875
MAGT1	0,001210407	0,017238157	0,438	0,152	1	1,01202025	0,988122521	2,881578947
PGBD4	0,226351275	0,017118203	0,031	0,008	1	1,011936108	0,988204682	3,875
SHC1	1,09993E-06	0,017103653	0,375	0,07	0,023209524	1,011925902	0,988214649	5,357142857
ELAPOR2	0,226351275	0,017033132	0,031	0,008	1	1,011876439	0,988262955	3,875
CAMK2A	0,082317662	0,017021542	0,031	0,004	1	1,01186831	0,988270895	7,75
CENPL	0,29055378	0,017008026	0,062	0,027	1	1,01185883	0,988280153	2,296296296
RNF123	0,212914042	0,016974286	0,062	0,023	1	1,011835166	0,988303267	2,695652174
DROSHA	0,087959196	0,016947062	0,062	0,016	1	1,011816073	0,988321916	3,875

Supplementary Table 1

Differentially expressed genes_adjacent-skin-XL_ comparing SC-Keloid vs. (SC-Myel, SC-Nonmyel)								
**gene**	**p_val**	**avg_log2FC**	**pct.1**	**pct.2**	**p_val_adj**	**Foldchange_UP**	**Foldchange_DOWN**	**Ratio_pct1_pct2**
CAMK2N2	0,082317662	0,016943322	0,031	0,004	1	1,01181345	0,988324478	7,75
ZBTB46	0,226351275	0,016938781	0,031	0,008	1	1,011810265	0,988327589	3,875
ZNF396	0,082317662	0,016839202	0,031	0,004	1	1,01174043	0,988395809	7,75
ALKBH4	0,001642148	0,016757972	0,125	0,02	1	1,011683466	0,988451461	6,25
TUBGCP3	0,000200286	0,016755143	0,188	0,031	1	1,011681482	0,9884534	6,064516129
BET1	0,003791564	0,016732164	0,188	0,047	1	1,011665368	0,988469144	4
TTC32	0,006444438	0,016567296	0,219	0,066	1	1,011549764	0,98858211	3,318181818
LAD1	0,082317662	0,016416927	0,031	0,004	1	1,011444338	0,988685153	7,75
FPGT	0,004087528	0,016392873	0,125	0,023	1	1,011427474	0,988701638	5,434782609
PSPN	0,226351275	0,016380541	0,031	0,008	1	1,011418829	0,988710089	3,875
GSK3B	0,003663003	0,016371498	0,375	0,137	1	1,011412489	0,988716286	2,737226277
ST3GAL3	0,037506898	0,016356281	0,094	0,023	1	1,011401821	0,988726715	4,086956522
ZNF569	0,041848356	0,0163221	0,062	0,012	1	1,011377859	0,988750141	5,166666667
ENPEP	0,082317662	0,016317861	0,031	0,004	1	1,011374887	0,988753046	7,75
TRIM41	0,175414505	0,016310858	0,094	0,039	1	1,011369977	0,988757846	2,41025641
BHLHE41	0,093336433	0,016266981	0,094	0,031	1	1,011339219	0,988787917	3,032258065
IPO5	5,26466E-05	0,016232859	0,531	0,156	1	1,0113153	0,988811304	3,403846154
POLH	0,226351275	0,016212755	0,031	0,008	1	1,011301207	0,988825083	3,875
FBXL20	0,004107327	0,016172683	0,156	0,035	1	1,011273118	0,988852549	4,457142857
CORO1B	6,63363E-07	0,0161531	0,5	0,113	0,013997633	1,011259391	0,988865971	4,424778761
ERI2	0,002263143	0,016151528	0,062	0,004	1	1,011258289	0,988867049	15,5
SLC25A40	0,041848356	0,016128685	0,062	0,012	1	1,011242277	0,988882706	5,166666667
SLF1	2,24716E-07	0,01612481	0,25	0,027	0,004741729	1,011239561	0,988885362	9,259259259
ABHD8	0,036827385	0,016074994	0,094	0,023	1	1,011204644	0,988919509	4,086956522
PCMTD2	0,021796651	0,016055779	0,25	0,098	1	1,011191176	0,98893268	2,551020408
RAB27B	0,082317662	0,016036504	0,031	0,004	1	1,011177666	0,988945893	7,75
ZNF213	0,036827385	0,016015187	0,094	0,023	1	1,011162725	0,988960506	4,086956522
ROCK1	0,000290654	0,015990863	0,531	0,176	1	1,011145677	0,98897718	3,017045455
EARS2	0,002263143	0,015966517	0,062	0,004	1	1,011128614	0,988993869	15,5
POLD4	0,001260548	0,015913404	0,469	0,164	1	1,011091389	0,98903028	2,859756098

Supplementary Table 1

Differentially expressed genes_adjacent-skin-XL_ comparing SC-Keloid vs. (SC-Myel, SC-Nonmyel)								
**gene**	**p_val**	**avg_log2FC**	**pct.1**	**pct.2**	**p_val_adj**	**Foldchange_UP**	**Foldchange_DOWN**	**Ratio_pct1_pct2**
GPATCH3	0,000509339	0,015864745	0,125	0,016	1	1,011057288	0,989063639	7,8125
C5	0,380671495	0,01585748	0,062	0,031	1	1,011052197	0,989068619	2
ZNF66	4,80439E-05	0,01585307	0,094	0,004	1	1,011049106	0,989071643	23,5
AMMECR1L	0,016858455	0,01579318	0,125	0,031	1	1,011007136	0,989112702	4,032258065
DPY19L4	0,000211329	0,015761575	0,312	0,078	1	1,010984988	0,989134371	4
ADAT1	0,000911755	0,015704286	0,156	0,027	1	1,010944843	0,98917365	5,777777778
INKA2	0,088329108	0,015661099	0,125	0,047	1	1,010914581	0,989203261	2,659574468
FAS	0,001968597	0,015657716	0,156	0,031	1	1,01091221	0,989205581	5,032258065
ZNF814	0,013891036	0,01562841	0,062	0,008	1	1,010891675	0,989225675	7,75
FEZ1	0,001667472	0,01549717	0,656	0,258	1	1,01079972	0,989315668	2,542635659
SF3A1	0,016972927	0,015480644	0,281	0,109	1	1,010788142	0,989327	2,577981651
MIS12	0,000509339	0,015452843	0,125	0,016	1	1,010768664	0,989346065	7,8125
C18orf54	0,008148126	0,015264825	0,094	0,016	1	1,010636945	0,989475009	5,875
NFX1	0,012579781	0,015260482	0,156	0,043	1	1,010633902	0,989477988	3,627906977
HROB	0,002263143	0,015257995	0,062	0,004	1	1,01063216	0,989479693	15,5
ADRA1B	0,004841742	0,015255908	0,031	0	1	1,010630698	0,989481125	#DIV/0!
APOBEC3B	0,004841742	0,015255908	0,031	0	1	1,010630698	0,989481125	#DIV/0!
ARHGEF39	0,004841742	0,015255908	0,031	0	1	1,010630698	0,989481125	#DIV/0!
BTN1A1	0,004841742	0,015255908	0,031	0	1	1,010630698	0,989481125	#DIV/0!
C2orf66	0,004841742	0,015255908	0,031	0	1	1,010630698	0,989481125	#DIV/0!
CD70	0,004841742	0,015255908	0,031	0	1	1,010630698	0,989481125	#DIV/0!
CDC45	0,004841742	0,015255908	0,031	0	1	1,010630698	0,989481125	#DIV/0!
CNIH2	0,004841742	0,015255908	0,031	0	1	1,010630698	0,989481125	#DIV/0!
E2F8	0,004841742	0,015255908	0,031	0	1	1,010630698	0,989481125	#DIV/0!
FBLN7	0,004841742	0,015255908	0,031	0	1	1,010630698	0,989481125	#DIV/0!
FSCN3	0,004841742	0,015255908	0,031	0	1	1,010630698	0,989481125	#DIV/0!
H3C2	0,004841742	0,015255908	0,031	0	1	1,010630698	0,989481125	#DIV/0!
H3C8	0,004841742	0,015255908	0,031	0	1	1,010630698	0,989481125	#DIV/0!
IQCH	0,004841742	0,015255908	0,031	0	1	1,010630698	0,989481125	#DIV/0!
IQGAP3	0,004841742	0,015255908	0,031	0	1	1,010630698	0,989481125	#DIV/0!

Supplementary Table 1

Differentially expressed genes_adjacent-skin-XL_ comparing SC-Keloid vs. (SC-Myel, SC-Nonmyel)								
**gene**	**p_val**	**avg_log2FC**	**pct.1**	**pct.2**	**p_val_adj**	**Foldchange_UP**	**Foldchange_DOWN**	**Ratio_pct1_pct2**
KIF20A	0,004841742	0,015255908	0,031	0	1	1,010630698	0,989481125	#DIV/0!
KTN1-AS1	0,004841742	0,015255908	0,031	0	1	1,010630698	0,989481125	#DIV/0!
NEK2	0,004841742	0,015255908	0,031	0	1	1,010630698	0,989481125	#DIV/0!
PLEKHS1	0,004841742	0,015255908	0,031	0	1	1,010630698	0,989481125	#DIV/0!
PRSS36	0,004841742	0,015255908	0,031	0	1	1,010630698	0,989481125	#DIV/0!
PSTPIP1	0,004841742	0,015255908	0,031	0	1	1,010630698	0,989481125	#DIV/0!
RAB39B	0,004841742	0,015255908	0,031	0	1	1,010630698	0,989481125	#DIV/0!
RAD51	0,004841742	0,015255908	0,031	0	1	1,010630698	0,989481125	#DIV/0!
SLC24A1	0,004841742	0,015255908	0,031	0	1	1,010630698	0,989481125	#DIV/0!
SLC26A4	0,004841742	0,015255908	0,031	0	1	1,010630698	0,989481125	#DIV/0!
THBS4	0,004841742	0,015255908	0,031	0	1	1,010630698	0,989481125	#DIV/0!
TONSL	0,004841742	0,015255908	0,031	0	1	1,010630698	0,989481125	#DIV/0!
TRIM61	0,004841742	0,015255908	0,031	0	1	1,010630698	0,989481125	#DIV/0!
TSPAN2	0,004841742	0,015255908	0,031	0	1	1,010630698	0,989481125	#DIV/0!
TTK	0,004841742	0,015255908	0,031	0	1	1,010630698	0,989481125	#DIV/0!
WNT5B	0,004841742	0,015255908	0,031	0	1	1,010630698	0,989481125	#DIV/0!
XPO4	0,036158386	0,015238858	0,094	0,023	1	1,010618755	0,989492819	4,086956522
NDUFV3	0,000479192	0,01521685	0,406	0,121	1	1,010603338	0,989507913	3,355371901
MCM2	0,082317662	0,01517925	0,031	0,004	1	1,010576999	0,989533703	7,75
ZNF584	0,082317662	0,01517925	0,031	0,004	1	1,010576999	0,989533703	7,75
SYT1	0,082317662	0,015168485	0,031	0,004	1	1,010569459	0,989541086	7,75
KCTD13	0,030992783	0,015161683	0,156	0,051	1	1,010564694	0,989545752	3,058823529
GIT2	0,081162818	0,015130939	0,125	0,047	1	1,010543159	0,989566839	2,659574468
FTSJ3	0,036827385	0,015117482	0,094	0,023	1	1,010533733	0,98957607	4,086956522
NPPC	0,226351275	0,015095753	0,031	0,008	1	1,010518514	0,989590974	3,875
PCDHGA9	0,226351275	0,015074692	0,031	0,008	1	1,010503762	0,98960542	3,875
ZNF620	0,082317662	0,015072939	0,031	0,004	1	1,010502534	0,989606623	7,75
ZNF511	0,013783696	0,014934776	0,25	0,09	1	1,010405765	0,9897014	2,777777778
PPP5D1P	0,082317662	0,014928169	0,031	0,004	1	1,010401138	0,989705932	7,75
MEX3B	5,12603E-06	0,014920724	0,188	0,02	0,108164307	1,010395923	0,98971104	9,4

Supplementary Table 1

Differentially expressed genes_adjacent-skin-XL_ comparing SC-Keloid vs. (SC-Myel, SC-Nonmyel)								
**gene**	**p_val**	**avg_log2FC**	**pct.1**	**pct.2**	**p_val_adj**	**Foldchange_UP**	**Foldchange_DOWN**	**Ratio_pct1_pct2**
KAZALD1	0,226351275	0,014914435	0,031	0,008	1	1,010391519	0,989715354	3,875
GPATCH11	0,001034587	0,014888593	0,188	0,039	1	1,010373421	0,989733082	4,820512821
PLA2G4E	0,082317662	0,014764647	0,031	0,004	1	1,010286621	0,989818116	7,75
NUP37	0,026506403	0,014710936	0,125	0,035	1	1,010249009	0,989854968	3,571428571
SYS1	0,003361753	0,014656211	0,188	0,047	1	1,010210689	0,989892516	4
AL445989.1	0,386325862	0,014651505	0,031	0,012	1	1,010207393	0,989895745	2,583333333
AURKA	0,002263143	0,014647794	0,062	0,004	1	1,010204795	0,989898291	15,5
PLEKHG1	0,011423236	0,014641634	0,156	0,043	1	1,010200481	0,989902518	3,627906977
BTC	0,082317662	0,014594539	0,031	0,004	1	1,010167505	0,989934833	7,75
DHRS12	0,003420308	0,01453668	0,188	0,047	1	1,010126993	0,989974535	4
ADORA1	0,004841742	0,014477918	0,031	0	1	1,010085851	0,990014858	#DIV/0!
ADORA2A	0,004841742	0,014477918	0,031	0	1	1,010085851	0,990014858	#DIV/0!
AK7	0,004841742	0,014477918	0,031	0	1	1,010085851	0,990014858	#DIV/0!
ARHGAP45	0,004841742	0,014477918	0,031	0	1	1,010085851	0,990014858	#DIV/0!
BFSP1	0,004841742	0,014477918	0,031	0	1	1,010085851	0,990014858	#DIV/0!
CCDC125	0,004841742	0,014477918	0,031	0	1	1,010085851	0,990014858	#DIV/0!
CHRNA5	0,004841742	0,014477918	0,031	0	1	1,010085851	0,990014858	#DIV/0!
CSF1R	0,004841742	0,014477918	0,031	0	1	1,010085851	0,990014858	#DIV/0!
GRAMD1C	0,004841742	0,014477918	0,031	0	1	1,010085851	0,990014858	#DIV/0!
GREM1	0,004841742	0,014477918	0,031	0	1	1,010085851	0,990014858	#DIV/0!
H2AC13	0,004841742	0,014477918	0,031	0	1	1,010085851	0,990014858	#DIV/0!
ITPRIPL1	0,004841742	0,014477918	0,031	0	1	1,010085851	0,990014858	#DIV/0!
PLAC8L1	0,004841742	0,014477918	0,031	0	1	1,010085851	0,990014858	#DIV/0!
PSD2	0,004841742	0,014477918	0,031	0	1	1,010085851	0,990014858	#DIV/0!
PTGER1	0,004841742	0,014477918	0,031	0	1	1,010085851	0,990014858	#DIV/0!
ROBO4	0,004841742	0,014477918	0,031	0	1	1,010085851	0,990014858	#DIV/0!
SCG3	0,004841742	0,014477918	0,031	0	1	1,010085851	0,990014858	#DIV/0!
TSNAXIP1	0,004841742	0,014477918	0,031	0	1	1,010085851	0,990014858	#DIV/0!
WDR89	0,004841742	0,014477918	0,031	0	1	1,010085851	0,990014858	#DIV/0!
ZSCAN22	0,004841742	0,014477918	0,031	0	1	1,010085851	0,990014858	#DIV/0!

Supplementary Table 1

Differentially expressed genes_adjacent-skin-XL_ comparing SC-Keloid vs. (SC-Myel, SC-Nonmyel)								
**gene**	**p_val**	**avg_log2FC**	**pct.1**	**pct.2**	**p_val_adj**	**Foldchange_UP**	**Foldchange_DOWN**	**Ratio_pct1_pct2**
TEX261	7,83217E-06	0,014467781	0,312	0,059	0,165266588	1,010078753	0,990021814	5,288135593
MRPL18	0,000139969	0,014431979	0,469	0,141	1	1,010053687	0,990046383	3,326241135
CYFIP1	0,000280812	0,014340487	0,406	0,121	1	1,009989635	0,990109171	3,355371901
ACTR3B	0,041848356	0,014326423	0,062	0,012	1	1,009979789	0,990118823	5,166666667
DUSP12	0,000252542	0,014322062	0,219	0,043	1	1,009976736	0,990121816	5,093023256
FGF5	0,082317662	0,014279815	0,031	0,004	1	1,009947161	0,990150811	7,75
ZBTB11-AS1	0,041848356	0,014266383	0,062	0,012	1	1,009937758	0,990160029	5,166666667
MOCS1	0,150155729	0,01423006	0,062	0,02	1	1,009912331	0,990184959	3,1
CDK16	0,001125066	0,014188248	0,281	0,078	1	1,009883062	0,990213657	3,602564103
SMURF2	0,082895042	0,01414728	0,188	0,082	1	1,009854385	0,990241776	2,292682927
CNNM2	0,036827385	0,014076445	0,094	0,023	1	1,009804804	0,990290397	4,086956522
NOC3L	0,006724752	0,014018127	0,219	0,066	1	1,009763985	0,990330428	3,318181818
ZNF3	0,236345884	0,013989971	0,094	0,043	1	1,009744278	0,990349757	2,186046512
GFM2	0,001642148	0,013932472	0,125	0,02	1	1,009704036	0,990389228	6,25
WDPCP	0,086278754	0,0139205	0,062	0,016	1	1,009695656	0,990397447	3,875
PRKAR2B	0,002517564	0,013820927	0,094	0,012	1	1,009625971	0,990465805	7,833333333
TMEM256	0,010318402	0,013789627	0,469	0,203	1	1,009604067	0,990487294	2,310344828
PCDHGA1	0,226351275	0,013729723	0,031	0,008	1	1,009562147	0,990528422	3,875
GAS2L1	0,000537931	0,013677632	0,219	0,047	1	1,009525696	0,990564187	4,659574468
TIGD6	0,086278754	0,013676528	0,062	0,016	1	1,009524923	0,990564946	3,875
FBXL14	0,082317662	0,013590628	0,031	0,004	1	1,009464816	0,990623927	7,75
VWA8	0,086278754	0,013588948	0,062	0,016	1	1,009463641	0,99062508	3,875
EXOC2	0,008148126	0,013573071	0,094	0,016	1	1,009452531	0,990635982	5,875
TRIM6	0,082317662	0,013555002	0,031	0,004	1	1,009439888	0,99064839	7,75
HTRA3	0,082317662	0,013537203	0,031	0,004	1	1,009427435	0,990660612	7,75
DDX10	0,038302319	0,013531797	0,219	0,086	1	1,009423652	0,990664324	2,546511628
ZNF711	0,036827385	0,013340283	0,094	0,023	1	1,009289663	0,99079584	4,086956522
STX5	0,000103595	0,013306358	0,375	0,098	1	1,00926593	0,990819139	3,826530612
HAUS8	0,019236133	0,013297016	0,094	0,02	1	1,009259395	0,990825555	4,7
HHAT	0,386325862	0,013263164	0,031	0,012	1	1,009235713	0,990848804	2,583333333

Supplementary Table 1

Differentially expressed genes_adjacent-skin-XL_ comparing SC-Keloid vs. (SC-Myel, SC-Nonmyel)								
**gene**	**p_val**	**avg_log2FC**	**pct.1**	**pct.2**	**p_val_adj**	**Foldchange_UP**	**Foldchange_DOWN**	**Ratio_pct1_pct2**
ARFRP1	0,014633666	0,013118447	0,281	0,105	1	1,009134481	0,990948202	2,676190476
GYS1	0,002594281	0,013109062	0,094	0,012	1	1,009127917	0,990954648	7,833333333
KCNJ2	0,082317662	0,013050652	0,031	0,004	1	1,009087061	0,99099477	7,75
IMMP2L	0,019871239	0,013021668	0,156	0,047	1	1,009066789	0,991014679	3,319148936
PCDH18	0,380266129	0,013001183	0,031	0,012	1	1,009052461	0,991028751	2,583333333
RDH13	0,386325862	0,012864067	0,031	0,012	1	1,008956564	0,991122944	2,583333333
FDXR	0,001642148	0,012791172	0,125	0,02	1	1,008905585	0,991173024	6,25
TRIM59	0,002263143	0,012772329	0,062	0,004	1	1,008892408	0,991185969	15,5
MED22	0,003907631	0,012753111	0,125	0,023	1	1,008878969	0,991199173	5,434782609
GRK4	0,008148126	0,012731957	0,094	0,016	1	1,008864177	0,991213707	5,875
NCAPH2	8,72119E-06	0,012729278	0,312	0,059	0,184025872	1,008862303	0,991215547	5,288135593
LRMDA	0,000425228	0,012708985	0,281	0,07	1	1,008848112	0,99122949	4,014285714
ZSCAN9	0,226351275	0,012704925	0,031	0,008	1	1,008845273	0,99123228	3,875
GLCE	0,783859705	0,012693488	0,031	0,023	1	1,008837276	0,991240138	1,347826087
RBM34	0,386325862	0,012685954	0,031	0,012	1	1,008832007	0,991245314	2,583333333
CFAP46	0,082317662	0,012656571	0,031	0,004	1	1,008811461	0,991265503	7,75
H3C4	0,002263143	0,012639884	0,062	0,004	1	1,008799793	0,991276968	15,5
C2orf74	0,036158386	0,012607986	0,094	0,023	1	1,008777488	0,991298886	4,086956522
ANKRD37	0,243500153	0,012548714	0,188	0,105	1	1,008736044	0,991339613	1,79047619
KCNAB3	0,147778936	0,012435851	0,062	0,02	1	1,008657133	0,99141717	3,1
ANG	2,40115E-05	0,012411871	0,156	0,016	0,506667402	1,008640368	0,991433649	9,75
BANK1	0,041848356	0,012373436	0,062	0,012	1	1,008613497	0,991460062	5,166666667
ABHD18	0,668867248	0,012363179	0,031	0,02	1	1,008606326	0,991467111	1,55
NUDCD1	0,057370526	0,01230925	0,156	0,059	1	1,008568624	0,991504174	2,644067797
DBF4B	0,013891036	0,012264421	0,062	0,008	1	1,008537286	0,991534983	7,75
TTPA	0,082317662	0,012152548	0,031	0,004	1	1,008459082	0,991611874	7,75
ZNF843	0,082317662	0,0121165	0,031	0,004	1	1,008433884	0,991636651	7,75
PSPH	0,019236133	0,012065513	0,094	0,02	1	1,008398246	0,991671698	4,7
TMEM241	0,226351275	0,012049586	0,031	0,008	1	1,008387113	0,991682645	3,875
STAC3	0,082317662	0,012016854	0,031	0,004	1	1,008364235	0,991705145	7,75

Supplementary Table 1

Differentially expressed genes_adjacent-skin-XL_ comparing SC-Keloid vs. (SC-Myel, SC-Nonmyel)								
**gene**	**p_val**	**avg_log2FC**	**pct.1**	**pct.2**	**p_val_adj**	**Foldchange_UP**	**Foldchange_DOWN**	**Ratio_pct1_pct2**
NF2	0,027370459	0,012013309	0,125	0,035	1	1,008361757	0,991707582	3,571428571
ACOT9	0,003973246	0,011977267	0,281	0,09	1	1,008336566	0,991732358	3,122222222
ANGEL1	0,380266129	0,011952914	0,031	0,012	1	1,008319545	0,991749098	2,583333333
NLE1	0,000524345	0,011914053	0,094	0,008	1	1,008292385	0,991775813	11,75
ZNF358	0,007341735	0,011833461	0,375	0,145	1	1,008236062	0,991831217	2,586206897
MTRNR2L12	0,008437622	0,011815606	0,125	0,027	1	1,008223583	0,991843493	4,62962963
IFT122	0,229249342	0,011782118	0,094	0,043	1	1,008200181	0,991866515	2,186046512
APOC1	0,082317662	0,011772094	0,031	0,004	1	1,008193175	0,991873407	7,75
ADCY1	0,013891036	0,011763564	0,062	0,008	1	1,008187215	0,991879272	7,75
ZNF415	0,000509339	0,011677717	0,125	0,016	1	1,008127224	0,991938295	7,8125
LEMD2	0,004751692	0,0116733	0,219	0,062	1	1,008124138	0,991941331	3,532258065
PEX11A	0,013891036	0,011631084	0,062	0,008	1	1,008094639	0,991970358	7,75
NAP1L4	0,000273658	0,011607468	0,5	0,16	1	1,008078137	0,991986596	3,125
ZNF570	0,533973819	0,011597756	0,031	0,016	1	1,008071351	0,991993274	1,9375
PLEC	0,036246184	0,011587862	0,75	0,418	1	1,008064438	0,992000077	1,794258373
SNTB2	0,001206362	0,011488807	0,375	0,121	1	1,007995227	0,99206819	3,099173554
FZD3	0,016205151	0,011475907	0,25	0,09	1	1,007986213	0,992077061	2,777777778
C9orf135	0,082317662	0,011468582	0,031	0,004	1	1,007981096	0,992082098	7,75
NIPSNAP1	0,036827385	0,011364594	0,094	0,023	1	1,007908444	0,992153608	4,086956522
MCEE	0,000911755	0,011364472	0,156	0,027	1	1,007908359	0,992153693	5,777777778
EPHA3	0,143111738	0,011352707	0,062	0,02	1	1,007900139	0,992161784	3,1
LINC01069	0,082317662	0,011328774	0,031	0,004	1	1,00788342	0,992178242	7,75
MUC5B	0,082317662	0,011287235	0,031	0,004	1	1,0078544	0,99220681	7,75
ZNF805	0,008148126	0,011274868	0,094	0,016	1	1,007845761	0,992215316	5,875
PPIH	0,000804279	0,01118279	0,25	0,062	1	1,007781439	0,992278644	4,032258065
BECN1	8,86008E-06	0,011170429	0,375	0,082	0,186956528	1,007772804	0,992287146	4,573170732
MELTF-AS1	0,098751938	0,011138105	0,094	0,031	1	1,007750224	0,99230938	3,032258065
KIF18A	0,082317662	0,011095212	0,031	0,004	1	1,007720263	0,992338882	7,75
NUDT6	0,041848356	0,01099507	0,062	0,012	1	1,007650317	0,992407766	5,166666667
MCRIP2	0,007736261	0,010948184	0,156	0,039	1	1,00761757	0,992440018	4

Supplementary Table 1

Differentially expressed genes_adjacent-skin-XL_ comparing SC-Keloid vs. (SC-Myel, SC-Nonmyel)								
**gene**	**p_val**	**avg_log2FC**	**pct.1**	**pct.2**	**p_val_adj**	**Foldchange_UP**	**Foldchange_DOWN**	**Ratio_pct1_pct2**
PMVK	0,000580757	0,010785553	0,375	0,113	1	1,00750399	0,9925519	3,318584071
FANCI	0,013891036	0,010766958	0,062	0,008	1	1,007491005	0,992564693	7,75
HYAL3	0,002263143	0,010712216	0,062	0,004	1	1,007452777	0,992602356	15,5
ZNF391	0,082317662	0,010677822	0,031	0,004	1	1,00742876	0,99262602	7,75
C19orf54	0,226351275	0,010659924	0,031	0,008	1	1,007416262	0,992638334	3,875
SPON2	0,187685368	0,010656323	0,094	0,039	1	1,007413747	0,992640812	2,41025641
SKA2	0,000330614	0,010584783	0,625	0,215	1	1,007363793	0,992690036	2,906976744
ZNF770	0,008956837	0,010559446	0,125	0,027	1	1,007346102	0,99270747	4,62962963
KRT7	0,380266129	0,010544953	0,031	0,012	1	1,007335982	0,992717443	2,583333333
GOLGA8N	0,682170546	0,010524475	0,031	0,02	1	1,007321683	0,992731534	1,55
PSMD12	0,004853928	0,010482316	0,406	0,152	1	1,007292247	0,992760545	2,671052632
PRUNE2	0,221475664	0,010344969	0,031	0,008	1	1,007196356	0,992855061	3,875
UBE2G2	0,003530962	0,010290612	0,406	0,152	1	1,007158409	0,99289247	2,671052632
VAMP8	0,019236133	0,010130916	0,094	0,02	1	1,007046929	0,993002382	4,7
KXD1	0,000734952	0,010130644	0,469	0,16	1	1,007046739	0,99300257	2,93125
ATP10B	0,082317662	0,010097438	0,031	0,004	1	1,007023561	0,993025425	7,75
SELPLG	0,013891036	0,010086591	0,062	0,008	1	1,00701599	0,993032891	7,75
NKAPD1	5,86746E-05	-0,010076384	0,25	0,047	1	0,993039917	1,007008865	5,319148936
ENOX1	0,082317662	-0,010086186	0,031	0,004	1	0,99303317	1,007015707	7,75
PWP1	0,000215327	-0,010221494	0,344	0,09	1	0,99294004	1,007110158	3,822222222
SCO1	0,036158129	-0,010241758	0,094	0,023	1	0,992926093	1,007124304	4,086956522
COPG2	0,150155729	-0,010256246	0,062	0,02	1	0,992916122	1,007134417	3,1
SPOCK1	0,226351275	-0,010398146	0,031	0,008	1	0,992818466	1,007233481	3,875
FBXW8	0,226351275	-0,010399973	0,031	0,008	1	0,992817208	1,007234758	3,875
ZC2HC1A	0,013247448	-0,010419954	0,312	0,121	1	0,992803459	1,007248707	2,578512397
CRCP	0,086231137	-0,010553997	0,125	0,047	1	0,99271122	1,007342296	2,659574468
PRRG2	0,226351275	-0,010569996	0,031	0,008	1	0,992700211	1,007353468	3,875
MRPS25	0,000537931	-0,01059715	0,219	0,047	1	0,992681527	1,007372428	4,659574468
METAP1	0,06222129	-0,010618064	0,156	0,059	1	0,992667137	1,007387031	2,644067797
CASP1	0,226351275	-0,01065939	0,031	0,008	1	0,992638702	1,007415888	3,875

Supplementary Table 1

Differentially expressed genes_adjacent-skin-XL_ comparing SC-Keloid vs. (SC-Myel, SC-Nonmyel)								
**gene**	**p_val**	**avg_log2FC**	**pct.1**	**pct.2**	**p_val_adj**	**Foldchange_UP**	**Foldchange_DOWN**	**Ratio_pct1_pct2**
TCF19	0,226351275	-0,010675902	0,031	0,008	1	0,992627341	1,007427419	3,875
BANP	0,000870223	-0,010714652	0,156	0,027	1	0,99260068	1,007454478	5,777777778
DEPTOR	0,150155729	-0,010784048	0,062	0,02	1	0,992552935	1,007502939	3,1
BEX5	0,226351275	-0,010786042	0,031	0,008	1	0,992551563	1,007504332	3,875
UBR1	0,067638366	-0,010809034	0,188	0,078	1	0,992535745	1,007520389	2,41025641
HSDL2	0,019329956	-0,010846138	0,188	0,062	1	0,992510219	1,007546301	3,032258065
NOL11	0,009522001	-0,010881857	0,188	0,055	1	0,992485647	1,007571247	3,418181818
NAPG	0,002910424	-0,010918726	0,219	0,059	1	0,992460283	1,007596996	3,711864407
NLRX1	0,041848356	-0,010964428	0,062	0,012	1	0,992428844	1,007628915	5,166666667
THEM6	0,371729093	-0,010971844	0,125	0,074	1	0,992423743	1,007634095	1,689189189
FXN	0,000509339	-0,011004003	0,125	0,016	1	0,992401621	1,007656556	7,8125
RAB9A	0,000374084	-0,011132592	0,531	0,176	1	0,992313171	1,007746374	3,017045455
KDM4C	0,004107327	-0,011171261	0,156	0,035	1	0,992286574	1,007773385	4,457142857
CBX8	0,082317662	-0,01117306	0,031	0,004	1	0,992285337	1,007774642	7,75
SAPCD1	0,013891036	-0,011195829	0,062	0,008	1	0,992269676	1,007790547	7,75
FUT10	0,008148126	-0,011287361	0,094	0,016	1	0,992206724	1,007854488	5,875
OAS3	0,087959196	-0,011288066	0,062	0,016	1	0,992206239	1,007854981	3,875
CASZ1	0,226351275	-0,01130198	0,031	0,008	1	0,99219667	1,007864701	3,875
FAM219A	0,004087528	-0,011331701	0,125	0,023	1	0,99217623	1,007885465	5,434782609
AKT2	0,00029018	-0,011372489	0,344	0,094	1	0,992148179	1,00791396	3,659574468
R3HDM4	0,000428874	-0,011449906	0,312	0,082	1	0,992094941	1,007968047	3,804878049
YPEL1	0,019236133	-0,011465498	0,094	0,02	1	0,992084218	1,007978941	4,7
RALGPS2	0,164073652	-0,011509207	0,188	0,098	1	0,992054162	1,00800948	1,918367347
TIPARP-AS1	0,226351275	-0,011543658	0,031	0,008	1	0,992030473	1,008033551	3,875
CAMKK2	9,37709E-06	-0,011582949	0,375	0,082	0,197865964	0,992003455	1,008061005	4,573170732
MOSPD3	0,009242853	-0,011779843	0,188	0,055	1	0,99186808	1,008198591	3,418181818
TTI1	0,002594281	-0,011901865	0,094	0,012	1	0,991784191	1,008283867	7,833333333
CCDC121	0,087959196	-0,011936702	0,062	0,016	1	0,991760243	1,008308215	3,875
PRR16	0,221757959	-0,011983219	0,062	0,023	1	0,991728266	1,008340726	2,695652174
NRTN	0,224763613	-0,012023365	0,062	0,023	1	0,991700669	1,008368786	2,695652174

Supplementary Table 1

Differentially expressed genes_adjacent-skin-XL_ comparing SC-Keloid vs. (SC-Myel, SC-Nonmyel)								
**gene**	**p_val**	**avg_log2FC**	**pct.1**	**pct.2**	**p_val_adj**	**Foldchange_UP**	**Foldchange_DOWN**	**Ratio_pct1_pct2**
BBS4	0,000476445	-0,012144065	0,188	0,035	1	0,991617705	1,008453152	5,371428571
BTBD8	0,226351275	-0,012156312	0,031	0,008	1	0,991609287	1,008461713	3,875
H2BC5	0,540537634	-0,01217688	0,031	0,016	1	0,99159515	1,00847609	1,9375
NNT-AS1	0,006360213	-0,012216757	0,188	0,051	1	0,991567742	1,008503966	3,68627451
TTC30A	0,386325862	-0,012263753	0,031	0,012	1	0,991535442	1,008536818	2,583333333
GINS3	0,000339359	-0,012306205	0,156	0,023	1	0,991506266	1,008566496	6,782608696
MON1A	0,386325862	-0,012334632	0,031	0,012	1	0,991486729	1,008586369	2,583333333
MEF2B	0,386325862	-0,012336546	0,031	0,012	1	0,991485414	1,008587707	2,583333333
ZNF599	0,013891036	-0,012345663	0,062	0,008	1	0,991479149	1,00859408	7,75
CRACR2B	0,226351275	-0,012403121	0,031	0,008	1	0,991439662	1,00863425	3,875
DFFA	0,12647311	-0,012467064	0,094	0,035	1	0,991395721	1,008678956	2,685714286
CDC23	0,000931266	-0,012467451	0,219	0,051	1	0,991395454	1,008679227	4,294117647
ZNF430	0,098751938	-0,012503095	0,094	0,031	1	0,99137096	1,008704148	3,032258065
LSG1	0,000993746	-0,012633575	0,188	0,039	1	0,991281303	1,008795381	4,820512821
GOLT1B	1,48183E-06	-0,012657194	0,438	0,094	0,03126817	0,991265075	1,008811896	4,659574468
LINC01088	0,013891036	-0,012676905	0,062	0,008	1	0,991251532	1,008825679	7,75
PAQR7	0,008956837	-0,012747842	0,125	0,027	1	0,991202793	1,008875284	4,62962963
ZC4H2	0,004180266	-0,013011822	0,125	0,023	1	0,991021443	1,009059902	5,434782609
MRPL30	0,004823168	-0,01303788	0,219	0,062	1	0,991003543	1,009078128	3,532258065
NBR1	3,42564E-05	-0,013048098	0,406	0,102	0,722843467	0,990996524	1,009085275	3,980392157
PNPT1	0,003568756	-0,013124766	0,25	0,074	1	0,990943862	1,009138901	3,378378378
TCF3	0,000233604	-0,013273881	0,312	0,078	1	0,990841445	1,00924321	4
SRD5A3	0,016858455	-0,01327505	0,125	0,031	1	0,990840642	1,009244028	4,032258065
BTN2A1	0,004611611	-0,013340409	0,219	0,062	1	0,990795754	1,009289751	3,532258065
BRF2	0,041848356	-0,013390582	0,062	0,012	1	0,990761297	1,009324852	5,166666667
RAP1GAP2	0,062914349	-0,013466761	0,094	0,027	1	0,990708983	1,009378149	3,481481481
KIF4A	0,386325862	-0,013524062	0,031	0,012	1	0,990669635	1,009418241	2,583333333
SLC38A5	0,226351275	-0,013559176	0,031	0,008	1	0,990645524	1,009442809	3,875
GPR107	0,089393291	-0,01356442	0,125	0,047	1	0,990641922	1,009446479	2,659574468
PMS1	0,020165275	-0,013577778	0,156	0,047	1	0,99063275	1,009455825	3,319148936

Supplementary Table 1

Differentially expressed genes_adjacent-skin-XL_ comparing SC-Keloid vs. (SC-Myel, SC-Nonmyel)								
**gene**	**p_val**	**avg_log2FC**	**pct.1**	**pct.2**	**p_val_adj**	**Foldchange_UP**	**Foldchange_DOWN**	**Ratio_pct1_pct2**
GBP3	0,000507403	-0,013618023	0,219	0,047	1	0,990605116	1,009483985	4,659574468
QRICH2	0,150155729	-0,013675767	0,062	0,02	1	0,990565468	1,00952439	3,1
BLZF1	0,014828336	-0,013798656	0,219	0,074	1	0,990481095	1,009610386	2,959459459
TRAM1	0,000173589	-0,013799776	0,688	0,238	1	0,990480326	1,009611169	2,890756303
TBC1D8	0,540537634	-0,013881476	0,031	0,016	1	0,990424237	1,009668345	1,9375
DSC2	0,226351275	-0,013940207	0,031	0,008	1	0,990383918	1,009709449	3,875
GLTP	0,319787484	-0,013953897	0,219	0,133	1	0,99037452	1,00971903	1,646616541
EPS15	0,018087948	-0,014010764	0,312	0,125	1	0,990335483	1,009758831	2,496
FAM43A	0,386325862	-0,014016329	0,031	0,012	1	0,990331663	1,009762726	2,583333333
PLCB3	3,86379E-06	-0,014073559	0,156	0,012	0,081529835	0,990292378	1,009802783	13
MALT1	0,150155729	-0,014086795	0,062	0,02	1	0,990283293	1,009812048	3,1
PHGDH	0,020463101	-0,014136474	0,156	0,047	1	0,990249193	1,009846821	3,319148936
MLPH	0,226351275	-0,014294983	0,031	0,008	1	0,9901404	1,009957779	3,875
CLIC3	0,540537634	-0,014302246	0,031	0,016	1	0,990135416	1,009962864	1,9375
BCL7A	0,037506898	-0,014329764	0,094	0,023	1	0,99011653	1,009982128	4,086956522
ZNF446	0,796859864	-0,014342013	0,031	0,023	1	0,990108124	1,009990703	1,347826087
NDUFAF7	0,138170297	-0,014352177	0,094	0,035	1	0,990101149	1,009997818	2,685714286
OXCT2	0,082317662	-0,014379587	0,031	0,004	1	0,990082337	1,010017008	7,75
CBWD6	0,386325862	-0,014457036	0,031	0,012	1	0,990029188	1,01007123	2,583333333
PCSK5	0,06592651	-0,014513571	0,125	0,043	1	0,989990392	1,010110813	2,906976744
C11orf65	0,386325862	-0,014561412	0,031	0,012	1	0,989957564	1,01014431	2,583333333
TIGD1	0,540537634	-0,014658285	0,031	0,016	1	0,989891093	1,010212141	1,9375
MYO5A	0,021727091	-0,014768683	0,219	0,078	1	0,989815348	1,010289447	2,807692308
LANCL2	0,063925678	-0,014821852	0,094	0,027	1	0,989778869	1,010326681	3,481481481
STOML1	0,001055584	-0,014832108	0,188	0,039	1	0,989771833	1,010333863	4,820512821
MGST2	0,668867248	-0,014860835	0,031	0,02	1	0,989752125	1,010353981	1,55
LRCH2	0,675506228	-0,014946147	0,031	0,02	1	0,989693599	1,010413729	1,55
PAXIP1	0,087959196	-0,014964384	0,062	0,016	1	0,989681089	1,010426502	3,875
SRR	0,224763613	-0,015013002	0,062	0,023	1	0,989647737	1,010460553	2,695652174
ROR1	0,082317662	-0,015029507	0,031	0,004	1	0,989636415	1,010472113	7,75

Supplementary Table 1

Differentially expressed genes_adjacent-skin-XL_ comparing SC-Keloid vs. (SC-Myel, SC-Nonmyel)								
**gene**	**p_val**	**avg_log2FC**	**pct.1**	**pct.2**	**p_val_adj**	**Foldchange_UP**	**Foldchange_DOWN**	**Ratio_pct1_pct2**
SAAL1	0,004180266	-0,015040727	0,125	0,023	1	0,989628719	1,010479972	5,434782609
PROSER1	0,150155729	-0,015053865	0,062	0,02	1	0,989619707	1,010489174	3,1
IRX2	0,386325862	-0,015097701	0,031	0,012	1	0,989589638	1,010519878	2,583333333
NT5M	0,041848356	-0,015137609	0,062	0,012	1	0,989562264	1,010547831	5,166666667
LRP2BP	0,796859864	-0,01533443	0,031	0,023	1	0,989427271	1,010685706	1,347826087
DUOX1	0,675506228	-0,015348792	0,031	0,02	1	0,989417422	1,010695767	1,55
YPEL4	0,226351275	-0,015384842	0,031	0,008	1	0,989392698	1,010721023	3,875
FASTKD5	0,540537634	-0,015420756	0,031	0,016	1	0,989368069	1,010746183	1,9375
ZNF653	0,668867248	-0,015439251	0,031	0,02	1	0,989355386	1,010759141	1,55
PHKG1	0,043692883	-0,015537914	0,156	0,055	1	0,989287728	1,010828267	2,836363636
STRN	0,007635721	-0,015549478	0,281	0,098	1	0,989279799	1,010836369	2,867346939
WASHC3	0,000345651	-0,015749602	0,406	0,121	1	0,98914258	1,010976598	3,355371901
ZNF22	6,52388E-06	-0,015815363	0,562	0,148	0,137660362	0,989097494	1,011022681	3,797297297
MFSD1	0,01460911	-0,015837658	0,25	0,09	1	0,989082208	1,011038306	2,777777778
TDRD7	0,540537634	-0,015842788	0,031	0,016	1	0,989078691	1,011041901	1,9375
RSPRY1	0,045949521	-0,015859814	0,219	0,09	1	0,989067019	1,011053832	2,433333333
LFNG	0,041848356	-0,015872172	0,062	0,012	1	0,989058547	1,011062493	5,166666667
TAF2	0,183526587	-0,015885307	0,094	0,039	1	0,989049542	1,011071698	2,41025641
DBF4	0,386325862	-0,015902769	0,031	0,012	1	0,98903757	1,011083936	2,583333333
ZBTB45	0,000112833	-0,015940249	0,125	0,012	1	0,989011877	1,011110204	10,41666667
TCAF1	4,00575E-05	-0,016015633	0,312	0,066	0,845253588	0,9889602	1,011163038	4,727272727
JRKL	0,008956837	-0,016051335	0,125	0,027	1	0,988935726	1,011188061	4,62962963
MANEAL	0,226351275	-0,016083862	0,031	0,008	1	0,988913431	1,011210859	3,875
CRYBG3	0,127492976	-0,016099115	0,188	0,09	1	0,988902975	1,011221551	2,088888889
PLXNA1	0,224763613	-0,016114919	0,062	0,023	1	0,988892142	1,011232628	2,695652174
KCNC4	0,675506228	-0,016160298	0,031	0,02	1	0,988861038	1,011264436	1,55
HCG25	0,226351275	-0,016238891	0,031	0,008	1	0,988807169	1,011319528	3,875
ZNF487	0,039667683	-0,016297358	0,125	0,039	1	0,988767097	1,011360514	3,205128205
C19orf44	0,218781208	-0,016343596	0,062	0,023	1	0,988735408	1,011392928	2,695652174
C12orf73	0,000466117	-0,016479213	0,188	0,035	1	0,988642469	1,011488006	5,371428571

Supplementary Table 1

Differentially expressed genes_adjacent-skin-XL_ comparing SC-Keloid vs. (SC-Myel, SC-Nonmyel)								
**gene**	**p_val**	**avg_log2FC**	**pct.1**	**pct.2**	**p_val_adj**	**Foldchange_UP**	**Foldchange_DOWN**	**Ratio_pct1_pct2**
VAPB	0,000605519	-0,016484063	0,406	0,125	1	0,988639146	1,011491406	3,248
DBP	0,076632198	-0,016505438	0,156	0,062	1	0,988624498	1,011506393	2,516129032
ZNF692	0,001642148	-0,016511833	0,125	0,02	1	0,988620116	1,011510876	6,25
RNGTT	0,016562293	-0,016578162	0,125	0,031	1	0,988574664	1,011557382	4,032258065
SCHIP1	0,063387391	-0,016615621	0,125	0,043	1	0,988548997	1,011583647	2,906976744
DHRSX	0,016858455	-0,016639244	0,125	0,031	1	0,98853281	1,011600212	4,032258065
ATP6V0E2	0,002538449	-0,01672599	0,406	0,145	1	0,988473374	1,011661039	2,8
NASP	0,010412431	-0,016804844	0,594	0,27	1	0,988419348	1,011716335	2,2
HARS2	0,019236133	-0,016819173	0,094	0,02	1	0,988409531	1,011726384	4,7
AC147651.4	0,014828336	-0,016832439	0,219	0,074	1	0,988400442	1,011735687	2,959459459
HOMER3	0,00036468	-0,016879763	0,344	0,094	1	0,988368021	1,011768874	3,659574468
RAB3IL1	0,540537634	-0,016971458	0,031	0,016	1	0,988305204	1,011833183	1,9375
ZNF830	0,019580952	-0,017041738	0,156	0,047	1	0,98825706	1,011882475	3,319148936
OPA3	0,224763613	-0,017089896	0,062	0,023	1	0,988224072	1,011916253	2,695652174
NUP88	0,000509339	-0,017215701	0,125	0,016	1	0,988137901	1,012004497	7,8125
ZNF43	0,019236133	-0,01726264	0,094	0,02	1	0,988105752	1,012037424	4,7
KIF2A	0,018075901	-0,017273851	0,281	0,109	1	0,988098074	1,012045288	2,577981651
MYOF	0,041641305	-0,017344608	0,25	0,105	1	0,988049614	1,012094925	2,380952381
NT5C	0,001507967	-0,017390214	0,531	0,195	1	0,988018381	1,012126919	2,723076923
MAOB	0,386325862	-0,017402646	0,031	0,012	1	0,988009866	1,012135642	2,583333333
NIT1	0,000476445	-0,017428447	0,188	0,035	1	0,987992197	1,012153743	5,371428571
SNAPC1	0,093336433	-0,017496298	0,094	0,031	1	0,987945732	1,012201346	3,032258065
FBXO45	0,141661018	-0,017528851	0,094	0,035	1	0,98792344	1,012224186	2,685714286
MTA2	0,291239073	-0,017529596	0,094	0,047	1	0,98792293	1,012224708	2
P2RY1	0,145146326	-0,017629822	0,125	0,055	1	0,9878543	1,012295032	2,272727273
RGMA	0,540537634	-0,017748493	0,031	0,016	1	0,987773047	1,012378302	1,9375
RNF25	0,000911755	-0,01775522	0,156	0,027	1	0,98776844	1,012383023	5,777777778
PSMD10	0,184716953	-0,017824394	0,125	0,059	1	0,98772108	1,012431566	2,118644068
PMM2	0,297426083	-0,017838025	0,062	0,027	1	0,987711748	1,012441131	2,296296296
UBL4A	0,00013166	-0,017840296	0,25	0,051	1	0,987710193	1,012442725	4,901960784

Supplementary Table 1

Differentially expressed genes_adjacent-skin-XL_ comparing SC-Keloid vs. (SC-Myel, SC-Nonmyel)								
**gene**	**p_val**	**avg_log2FC**	**pct.1**	**pct.2**	**p_val_adj**	**Foldchange_UP**	**Foldchange_DOWN**	**Ratio_pct1_pct2**
RBKS	0,226351275	-0,017877254	0,031	0,008	1	0,987684891	1,012468662	3,875
MRPL2	0,000693143	-0,017879809	0,375	0,113	1	0,987683142	1,012470455	3,318584071
C3	0,087959196	-0,017929006	0,062	0,016	1	0,987649462	1,012504981	3,875
PDSS2	0,386325862	-0,017958996	0,031	0,012	1	0,987628931	1,012526029	2,583333333
ADAMTS1	0,000365428	-0,017962838	0,562	0,195	1	0,987626301	1,012528726	2,882051282
H2AC8	0,082317662	-0,018022353	0,031	0,004	1	0,98758556	1,012570496	7,75
USP4	0,021379715	-0,018034742	0,156	0,047	1	0,987577079	1,012579192	3,319148936
BCL7B	0,000189615	-0,018112209	0,344	0,09	1	0,987524052	1,012633564	3,822222222
C17orf58	0,540537634	-0,018141793	0,031	0,016	1	0,987503801	1,01265433	1,9375
ZNF883	0,041848356	-0,018212241	0,062	0,012	1	0,987455582	1,01270378	5,166666667
GABPB1	0,002096899	-0,018238535	0,156	0,031	1	0,987437585	1,012722237	5,032258065
BTBD19	0,226351275	-0,018241341	0,031	0,008	1	0,987435665	1,012724206	3,875
PML	0,001753489	-0,018257474	0,219	0,055	1	0,987424623	1,012735531	3,981818182
C19orf47	0,226351275	-0,01827382	0,031	0,008	1	0,987413435	1,012747006	3,875
SEC23A	0,00245484	-0,01829515	0,312	0,098	1	0,987398836	1,01276198	3,183673469
TXLNA	0,009633791	-0,018319478	0,219	0,07	1	0,987382186	1,012779058	3,128571429
KLHDC10	0,001783529	-0,018366147	0,219	0,055	1	0,987350246	1,01281182	3,981818182
EIF5AL1	0,386325862	-0,01841842	0,031	0,012	1	0,987314472	1,012848518	2,583333333
ACOT4	0,226351275	-0,018444783	0,031	0,008	1	0,987296431	1,012867026	3,875
ZNF592	0,063387391	-0,018450021	0,125	0,043	1	0,987292846	1,012870704	2,906976744
ZNF624	0,019236133	-0,018462274	0,094	0,02	1	0,987284461	1,012879306	4,7
ATXN2L	0,034171291	-0,01854361	0,312	0,137	1	0,987228802	1,012936412	2,277372263
R3HCC1L	0,008148126	-0,018583155	0,094	0,016	1	0,987201742	1,012964177	5,875
CLK2	0,226351275	-0,018685872	0,031	0,008	1	0,987131457	1,013036301	3,875
ZNF484	0,041848356	-0,018686167	0,062	0,012	1	0,987131256	1,013036508	5,166666667
CERS2	0,002500955	-0,018728283	0,594	0,234	1	0,987102439	1,013066081	2,538461538
NACAD	0,093753684	-0,018732479	0,125	0,047	1	0,987099568	1,013069028	2,659574468
TTC37	0,000193306	-0,018792453	0,406	0,117	1	0,987058534	1,013111143	3,47008547
ZNF525	0,087959196	-0,018824603	0,062	0,016	1	0,987036538	1,01313372	3,875
ZNF513	0,547500804	-0,018827346	0,062	0,039	1	0,987034662	1,013135646	1,58974359

Supplementary Table 1

Differentially expressed genes_adjacent-skin-XL_ comparing SC-Keloid vs. (SC-Myel, SC-Nonmyel)								
**gene**	**p_val**	**avg_log2FC**	**pct.1**	**pct.2**	**p_val_adj**	**Foldchange_UP**	**Foldchange_DOWN**	**Ratio_pct1_pct2**
SLC38A10	7,14053E-05	-0,018939845	0,375	0,094	1	0,986957697	1,013214652	3,989361702
HOTAIRM1	0,226351275	-0,018988685	0,031	0,008	1	0,986924286	1,013248953	3,875
RAD52	0,036827385	-0,019057911	0,094	0,023	1	0,986876931	1,013297574	4,086956522
DTWD2	0,226351275	-0,019096092	0,031	0,008	1	0,986850814	1,013324391	3,875
PPM1L	0,226351275	-0,019103358	0,031	0,008	1	0,986845844	1,013329495	3,875
ASCC2	0,000469207	-0,019125084	0,219	0,047	1	0,986830982	1,013344755	4,659574468
SPRED2	0,037506634	-0,019153038	0,094	0,023	1	0,986811861	1,01336439	4,086956522
RPUSD2	0,150155729	-0,019334935	0,062	0,02	1	0,986687451	1,013492164	3,1
AGO1	0,063925678	-0,019421439	0,094	0,027	1	0,986628291	1,013552935	3,481481481
UBA1	0,00307185	-0,019499438	0,5	0,191	1	0,98657495	1,013607734	2,617801047
MFSD9	0,082317662	-0,019555336	0,031	0,004	1	0,986536725	1,013647008	7,75
TLCD5	0,243596382	-0,019574795	0,094	0,043	1	0,98652342	1,01366068	2,186046512
PBX4	0,041848356	-0,019682401	0,062	0,012	1	0,986449841	1,013736288	5,166666667
SYNGR2	0,106283856	-0,019703546	0,219	0,105	1	0,986435383	1,013751146	2,085714286
RBBP8	0,091552625	-0,019811194	0,125	0,047	1	0,986361782	1,013826791	2,659574468
PBLD	0,001642148	-0,019840416	0,125	0,02	1	0,986341803	1,013847327	6,25
SMAD1	0,019236133	-0,019854163	0,094	0,02	1	0,986332404	1,013856987	4,7
OGG1	0,001034587	-0,020010958	0,188	0,039	1	0,986225214	1,013967181	4,820512821
INAVA	0,226351275	-0,020026264	0,031	0,008	1	0,98621475	1,013977939	3,875
ZSCAN5A	0,226351275	-0,020048473	0,031	0,008	1	0,986199569	1,013993548	3,875
PARD6G	0,918113127	-0,020139756	0,031	0,027	1	0,986137172	1,014057708	1,148148148
UBE2N	0,005554076	-0,020176873	0,625	0,266	1	0,986111801	1,014083798	2,34962406
NARF	0,000110396	-0,020480414	0,375	0,098	1	0,985904346	1,014297182	3,826530612
B3GLCT	0,150155729	-0,020551564	0,062	0,02	1	0,985855725	1,014347206	3,1
BCDIN3D	0,002594281	-0,020559945	0,094	0,012	1	0,985849998	1,014353099	7,833333333
ARL5A	0,000608099	-0,020567711	0,531	0,188	1	0,985844691	1,014358559	2,824468085
MFF	0,005275462	-0,02057596	0,406	0,152	1	0,985839054	1,014364359	2,671052632
CRYZ	0,004180266	-0,020599154	0,125	0,023	1	0,985823205	1,014380666	5,434782609
LINC02897	0,226351275	-0,020679021	0,031	0,008	1	0,985768632	1,014436824	3,875
PTPN18	0,124769281	-0,020728312	0,125	0,051	1	0,985734953	1,014471483	2,450980392

Supplementary Table 1

Differentially expressed genes_adjacent-skin-XL_ comparing SC-Keloid vs. (SC-Myel, SC-Nonmyel)								
**gene**	**p_val**	**avg_log2FC**	**pct.1**	**pct.2**	**p_val_adj**	**Foldchange_UP**	**Foldchange_DOWN**	**Ratio_pct1_pct2**
ZNF254	0,27733921	-0,020767838	0,125	0,066	1	0,985707947	1,014499278	1,893939394
UBTF	4,77841E-05	-0,020839555	0,531	0,156	1	0,985658948	1,014549711	3,403846154
CCDC157	0,386325862	-0,020846462	0,031	0,012	1	0,985654229	1,014554567	2,583333333
FBXO33	0,041554521	-0,020856309	0,156	0,055	1	0,985647502	1,014561492	2,836363636
MARCHF7	0,061210167	-0,02088253	0,281	0,133	1	0,985629587	1,014579932	2,112781955
LXN	0,540537634	-0,021000671	0,031	0,016	1	0,985548878	1,014663019	1,9375
RILP	0,682170546	-0,021074335	0,031	0,02	1	0,985498558	1,014714828	1,55
HAPLN3	0,386325862	-0,021077014	0,031	0,012	1	0,985496728	1,014716713	2,583333333
PSMG4	0,010298205	-0,021145825	0,219	0,07	1	0,985449724	1,014765112	3,128571429
ZNF768	0,911838915	-0,021149681	0,031	0,027	1	0,98544709	1,014767824	1,148148148
MOB2	0,002169795	-0,021222133	0,375	0,125	1	0,985397603	1,014818787	3
AKAP10	0,40679988	-0,021238819	0,094	0,055	1	0,985386206	1,014830525	1,709090909
OSGEPL1	0,533973819	-0,021258012	0,031	0,016	1	0,985373097	1,014844025	1,9375
PHKB	0,150636831	-0,02136522	0,156	0,074	1	0,985299876	1,014919442	2,108108108
MIPEP	0,221475664	-0,021378291	0,031	0,008	1	0,985290949	1,014928637	3,875
POLR2J2	0,226351275	-0,021391014	0,031	0,008	1	0,985282259	1,014937588	3,875
ZNF335	0,185597364	-0,021397118	0,094	0,039	1	0,985278091	1,014941882	2,41025641
IARS1	0,019838208	-0,021418765	0,25	0,094	1	0,985263308	1,014957111	2,659574468
INVS	0,224763613	-0,021491219	0,062	0,023	1	0,985213827	1,015008085	2,695652174
SH3BGRL	0,032224112	-0,021545287	0,656	0,32	1	0,985176905	1,015046125	2,05
IPCEF1	0,087959196	-0,021554847	0,062	0,016	1	0,985170377	1,015052851	3,875
ANKRD26	0,000235567	-0,021555419	0,281	0,066	1	0,985169986	1,015053254	4,257575758
PRKD2	0,38454022	-0,021562092	0,062	0,031	1	0,985165429	1,015057949	2
AGTPBP1	0,224763613	-0,021606116	0,062	0,023	1	0,985135367	1,015088924	2,695652174
TCTA	0,044241765	-0,021687165	0,156	0,055	1	0,985080025	1,015145952	2,836363636
STK17B	0,016858455	-0,021764276	0,125	0,031	1	0,985027375	1,015200212	4,032258065
DKC1	0,004681177	-0,021809519	0,219	0,062	1	0,984996485	1,01523205	3,532258065
LINC00649	0,533973819	-0,021847172	0,031	0,016	1	0,984970778	1,015258546	1,9375
AGFG2	0,540537634	-0,021947648	0,031	0,016	1	0,984902182	1,015329256	1,9375
IFFO2	0,003663929	-0,022001514	0,188	0,047	1	0,98486541	1,015367166	4

Supplementary Table 1

Differentially expressed genes_adjacent-skin-XL_ comparing SC-Keloid vs. (SC-Myel, SC-Nonmyel)								
**gene**	**p_val**	**avg_log2FC**	**pct.1**	**pct.2**	**p_val_adj**	**Foldchange_UP**	**Foldchange_DOWN**	**Ratio_pct1_pct2**
CEP63	0,063655955	-0,022023203	0,188	0,078	1	0,984850603	1,015382431	2,41025641
FERMT2	0,150139851	-0,022034311	0,781	0,488	1	0,98484302	1,015390249	1,600409836
SERHL2	0,002594281	-0,022034882	0,094	0,012	1	0,98484263	1,015390651	7,833333333
BRCC3	0,000322156	-0,022073146	0,156	0,023	1	0,984816511	1,015417582	6,782608696
TRIM14	0,221757959	-0,022227723	0,062	0,023	1	0,984710999	1,015526384	2,695652174
TUT1	0,061916284	-0,022245486	0,094	0,027	1	0,984698874	1,015538888	3,481481481
OTUD5	0,138170297	-0,022258846	0,094	0,035	1	0,984689756	1,015548292	2,685714286
FRS3	0,386325862	-0,022282548	0,031	0,012	1	0,984673578	1,015564977	2,583333333
PRELID3A	0,304406192	-0,022329049	0,062	0,027	1	0,984641841	1,015597712	2,296296296
RHOBTB1	0,063387391	-0,022431454	0,125	0,043	1	0,984571952	1,015669803	2,906976744
BDH1	0,386325862	-0,022514201	0,031	0,012	1	0,984515482	1,015728059	2,583333333
ORAI3	0,008148126	-0,022521089	0,094	0,016	1	0,984510782	1,015732908	5,875
NOCT	0,396289917	-0,022569082	0,062	0,031	1	0,984478031	1,015766699	2
GRIK4	0,226351275	-0,022695926	0,031	0,008	1	0,984391478	1,01585601	3,875
SMARCAL1	0,096013629	-0,022700382	0,094	0,031	1	0,984388438	1,015859148	3,032258065
PTPRS	0,037506898	-0,022740259	0,094	0,023	1	0,98436123	1,015887227	4,086956522
SLC16A7	0,041848356	-0,022746726	0,062	0,012	1	0,984356817	1,015891781	5,166666667
MTFR1	0,236345884	-0,022796482	0,094	0,043	1	0,984322869	1,015926818	2,186046512
KATNAL1	1,43353E-05	-0,022799857	0,469	0,121	0,302489408	0,984320566	1,015929195	3,876033058
RHBDD1	0,38454022	-0,022830927	0,062	0,031	1	0,984299368	1,015951074	2
SLC8B1	0,096013629	-0,022870116	0,094	0,031	1	0,984272631	1,015978672	3,032258065
C11orf71	0,396289917	-0,022870803	0,062	0,031	1	0,984272162	1,015979155	2
NDC1	0,041848356	-0,022887094	0,062	0,012	1	0,984261048	1,015990628	5,166666667
TMEM53	0,019236133	-0,022916906	0,094	0,02	1	0,984240709	1,016011623	4,7
BUB3	0,000246192	-0,022953198	0,375	0,105	1	0,98421595	1,016037181	3,571428571
GRWD1	0,066791287	-0,022968912	0,125	0,043	1	0,98420523	1,016048248	2,906976744
HEXA	0,00033983	-0,023013932	0,469	0,148	1	0,984174518	1,016079955	3,168918919
NOTCH4	0,564954469	-0,023022381	0,062	0,039	1	0,984168754	1,016085906	1,58974359
SLC2A13	0,045949521	-0,023038323	0,219	0,09	1	0,984157879	1,016097134	2,433333333
POLM	0,224763613	-0,023129397	0,062	0,023	1	0,984095754	1,016161279	2,695652174

Supplementary Table 1

Differentially expressed genes_adjacent-skin-XL_ comparing SC-Keloid vs. (SC-Myel, SC-Nonmyel)								
**gene**	**p_val**	**avg_log2FC**	**pct.1**	**pct.2**	**p_val_adj**	**Foldchange_UP**	**Foldchange_DOWN**	**Ratio_pct1_pct2**
GOT1	0,001642148	-0,0231689	0,125	0,02	1	0,984068808	1,016189104	6,25
METTL8	0,016270711	-0,023170377	0,125	0,031	1	0,9840678	1,016190144	4,032258065
RAPGEF6	0,735890209	-0,023219984	0,094	0,074	1	0,984033964	1,016225087	1,27027027
TRIM65	0,087959196	-0,023242413	0,062	0,016	1	0,984018666	1,016240885	3,875
BRME1	0,226351275	-0,023268118	0,031	0,008	1	0,984001133	1,016258992	3,875
NOL12	0,160548057	-0,023270376	0,125	0,055	1	0,983999593	1,016260583	2,272727273
KDM5A	0,000120335	-0,023282593	0,469	0,141	1	0,98399126	1,016269189	3,326241135
PLXNA2	0,386325862	-0,02330183	0,031	0,012	1	0,98397814	1,01628274	2,583333333
ICA1	0,226351275	-0,023322818	0,031	0,008	1	0,983963825	1,016297525	3,875
QSOX2	0,008148126	-0,023330814	0,094	0,016	1	0,983958372	1,016303158	5,875
FBXO27	0,386325862	-0,023340534	0,031	0,012	1	0,983951743	1,016310005	2,583333333
DMAC2	0,017209641	-0,02334731	0,219	0,074	1	0,983947121	1,016314778	2,959459459
FGD5	0,543117005	-0,023429862	0	0,012	1	0,98389082	1,016372934	0
TCOF1	0,001814041	-0,02344259	0,219	0,055	1	0,983882141	1,016381901	3,981818182
TEX30	0,004180266	-0,023467371	0,125	0,023	1	0,983865241	1,016399359	5,434782609
FBXL18	0,062914349	-0,023472881	0,094	0,027	1	0,983861483	1,016403241	3,481481481
ZKSCAN2	0,386325862	-0,023555636	0,031	0,012	1	0,983805049	1,016461545	2,583333333
PPIL6	0,226351275	-0,023566395	0,031	0,008	1	0,983797712	1,016469125	3,875
LRRC47	0,064224632	-0,023568383	0,125	0,043	1	0,983796356	1,016470526	2,906976744
SLC4A7	6,66992E-05	-0,023584143	0,281	0,059	1	0,98378561	1,01648163	4,762711864
GLRX	0,147778936	-0,023615476	0,062	0,02	1	0,983764243	1,016503707	3,1
SCRN3	0,016562293	-0,023627314	0,125	0,031	1	0,983756172	1,016512047	4,032258065
TBC1D4	0,226351275	-0,023638074	0,031	0,008	1	0,983748834	1,016519629	3,875
DDX19B	0,029760483	-0,023644796	0,156	0,051	1	0,983744251	1,016524365	3,058823529
MAP1S	0,386325862	-0,023645108	0,031	0,012	1	0,983744038	1,016524585	2,583333333
SLC22A11	0,082317662	-0,023680314	0,031	0,004	1	0,983720032	1,016549392	7,75
TSEN54	0,224763613	-0,023709506	0,062	0,023	1	0,983700127	1,016569961	2,695652174
OSBPL6	0,226351275	-0,023785674	0,031	0,008	1	0,983648193	1,016623633	3,875
DUS4L	0,087959196	-0,023790986	0,062	0,016	1	0,983644572	1,016627376	3,875
ZNF287	0,000509339	-0,023807809	0,125	0,016	1	0,983633101	1,016639231	7,8125

Supplementary Table 1

Differentially expressed genes_adjacent-skin-XL_ comparing SC-Keloid vs. (SC-Myel, SC-Nonmyel)								
**gene**	**p_val**	**avg_log2FC**	**pct.1**	**pct.2**	**p_val_adj**	**Foldchange_UP**	**Foldchange_DOWN**	**Ratio_pct1_pct2**
PACRGL	0,031413167	-0,02383034	0,156	0,051	1	0,98361774	1,016655108	3,058823529
FAM3A	6,05286E-05	-0,023862298	0,375	0,094	1	0,983595951	1,016677629	3,989361702
TRAF3IP1	0,002096899	-0,024019207	0,156	0,031	1	0,983488981	1,01678821	5,032258065
TMEM181	0,00011289	-0,024140367	0,219	0,039	1	0,983406389	1,016873605	5,615384615
PIM2	0,082317662	-0,024167706	0,031	0,004	1	0,983387753	1,016892875	7,75
UMPS	0,041848356	-0,024190458	0,062	0,012	1	0,983372245	1,016908912	5,166666667
COG5	0,080880928	-0,024209286	0,156	0,062	1	0,983359411	1,016922184	2,516129032
UQCRC2	0,001131816	-0,024231405	0,375	0,121	1	0,983344335	1,016937774	3,099173554
RFX7	0,300902654	-0,024447372	0,062	0,027	1	0,983197143	1,017090018	2,296296296
FTSJ1	0,028712073	-0,024451057	0,125	0,035	1	0,983194631	1,017092616	3,571428571
LARP1	0,005471065	-0,024451223	0,469	0,184	1	0,983194518	1,017092733	2,548913043
AMPD2	0,020764757	-0,024490161	0,156	0,047	1	0,983167982	1,017120185	3,319148936
NBPF3	0,386325862	-0,024563631	0,031	0,012	1	0,983117915	1,017171983	2,583333333
SPECC1	6,91883E-05	-0,024593371	0,312	0,07	1	0,98309765	1,017192952	4,457142857
TMEM238	0,386325862	-0,024613854	0,031	0,012	1	0,983083692	1,017207394	2,583333333
FAM98B	0,001055584	-0,024634465	0,188	0,039	1	0,983069647	1,017221926	4,820512821
EPHB4	0,087959196	-0,024648467	0,062	0,016	1	0,983060106	1,017231799	3,875
COL24A1	0,226351275	-0,024768892	0,031	0,008	1	0,982978051	1,017316713	3,875
ZDHHC11	0,221475664	-0,024813026	0,031	0,008	1	0,982947981	1,017347835	3,875
EID2	0,006461838	-0,024927961	0,188	0,051	1	0,982869676	1,017428887	3,68627451
MRFAP1	0,183520991	-0,024975968	0,875	0,488	1	0,98283697	1,017462744	1,793032787
ARMCX3	0,010754958	-0,02523561	0,625	0,281	1	0,982660105	1,017645872	2,224199288
PDK1	0,041848356	-0,025241859	0,062	0,012	1	0,982655848	1,017650281	5,166666667
ZNF274	0,065070981	-0,025260531	0,125	0,043	1	0,982643131	1,017663452	2,906976744
PUS3	0,386325862	-0,025476346	0,031	0,012	1	0,982496146	1,017815697	2,583333333
C1QTNF6	3,86379E-06	-0,025479202	0,156	0,012	0,081529835	0,982494202	1,017817712	13
ROM1	0,543117005	-0,025532804	0	0,012	1	0,982457698	1,017855529	0
SLC11A2	0,511066664	-0,025619695	0,188	0,133	1	0,982398528	1,017916834	1,413533835
LACTB2	0,044552052	-0,025675597	0,125	0,039	1	0,982360463	1,017956277	3,205128205
LRCH1	0,346466678	-0,025757304	0,094	0,051	1	0,982304829	1,018013931	1,843137255

Supplementary Table 1

Differentially expressed genes_adjacent-skin-XL_ comparing SC-Keloid vs. (SC-Myel, SC-Nonmyel)								
**gene**	**p_val**	**avg_log2FC**	**pct.1**	**pct.2**	**p_val_adj**	**Foldchange_UP**	**Foldchange_DOWN**	**Ratio_pct1_pct2**
TAF5	0,226351275	-0,025849521	0,031	0,008	1	0,982242041	1,018079005	3,875
SPOUT1	0,087959196	-0,025862549	0,062	0,016	1	0,982233172	1,018088198	3,875
SNRPA1	0,020683076	-0,025907323	0,281	0,109	1	0,982202689	1,018119795	2,577981651
DHRS11	0,041848356	-0,025961393	0,062	0,012	1	0,982165878	1,018157953	5,166666667
AIP	0,000403093	-0,025990024	0,562	0,191	1	0,982146387	1,018178159	2,942408377
TAOK2	0,281781087	-0,026102945	0,125	0,066	1	0,982069516	1,018257856	1,893939394
ZNF527	0,386325862	-0,026112207	0,031	0,012	1	0,982063212	1,018264393	2,583333333
DCAF7	0,047830565	-0,026150905	0,219	0,09	1	0,98203687	1,018291707	2,433333333
MDM1	0,14813251	-0,026191123	0,125	0,055	1	0,982009494	1,018320094	2,272727273
CBARP	0,386325862	-0,026255404	0,031	0,012	1	0,98196574	1,018365467	2,583333333
TRIM62	0,226351275	-0,026284144	0,031	0,008	1	0,981946179	1,018385755	3,875
ELP3	0,682170546	-0,026312856	0,031	0,02	1	0,981926636	1,018406022	1,55
ZNF880	0,224763613	-0,026345494	0,062	0,023	1	0,981904423	1,018429062	2,695652174
PIGX	0,000983571	-0,02636661	0,219	0,051	1	0,981890051	1,018443969	4,294117647
ZNHIT6	0,416216544	-0,026507404	0,094	0,055	1	0,981794232	1,018543364	1,709090909
ARMC2	0,540537634	-0,026543082	0,031	0,016	1	0,981769953	1,018568553	1,9375
TANGO2	0,002096899	-0,026544216	0,156	0,031	1	0,981769181	1,018569354	5,032258065
ZNF169	0,533973819	-0,02658708	0,031	0,016	1	0,981740012	1,018599617	1,9375
LARP1B	0,000343233	-0,026597281	0,344	0,094	1	0,98173307	1,01860682	3,659574468
C6orf47	7,15271E-05	-0,026620796	0,188	0,027	1	0,981717068	1,018623422	6,962962963
KDM4A	0,147778936	-0,026653033	0,062	0,02	1	0,981695132	1,018646184	3,1
STX18	0,007318479	-0,026682948	0,219	0,066	1	0,981674777	1,018667306	3,318181818
STARD5	0,918113127	-0,026703046	0,031	0,027	1	0,981661101	1,018681497	1,148148148
TXNRD3	0,540537634	-0,026751519	0,031	0,016	1	0,981628119	1,018715724	1,9375
ZNF839	0,803381388	-0,02675545	0,031	0,023	1	0,981625444	1,0187185	1,347826087
FUZ	0,008956837	-0,026779347	0,125	0,027	1	0,981609185	1,018735374	4,62962963
CEP126	0,138170297	-0,026802408	0,094	0,035	1	0,981593494	1,018751659	2,685714286
ATXN7L3B	0,000632526	-0,026817562	0,406	0,129	1	0,981583183	1,01876236	3,147286822
FANCC	0,082317662	-0,026817966	0,031	0,004	1	0,981582908	1,018762645	7,75
FAM72A	0,150155729	-0,026917248	0,062	0,02	1	0,981515361	1,018832755	3,1

Supplementary Table 1

Differentially expressed genes_adjacent-skin-XL_ comparing SC-Keloid vs. (SC-Myel, SC-Nonmyel)								
**gene**	**p_val**	**avg_log2FC**	**pct.1**	**pct.2**	**p_val_adj**	**Foldchange_UP**	**Foldchange_DOWN**	**Ratio_pct1_pct2**
AL592183.1	0,386325862	-0,026933826	0,031	0,012	1	0,981504083	1,018844463	2,583333333
MSH5	0,082317662	-0,026934478	0,031	0,004	1	0,981503639	1,018844923	7,75
PABPC1L	0,224763613	-0,026945633	0,062	0,023	1	0,98149605	1,018852801	2,695652174
MKKS	0,000252542	-0,026950157	0,219	0,043	1	0,981492973	1,018855996	5,093023256
ZFP82	0,386325862	-0,027021541	0,031	0,012	1	0,98144441	1,01890641	2,583333333
ZBTB48	0,139907281	-0,027041071	0,094	0,035	1	0,981431124	1,018920203	2,685714286
F11R	0,226351275	-0,027093123	0,031	0,008	1	0,981395715	1,018956966	3,875
PDIK1L	0,041848356	-0,027098522	0,062	0,012	1	0,981392042	1,018960779	5,166666667
CHDH	0,675506228	-0,027294315	0,031	0,02	1	0,981258863	1,019099075	1,55
DIAPH2	0,150155729	-0,027434976	0,062	0,02	1	0,981163196	1,019198441	3,1
EML4	0,000112855	-0,027481009	0,344	0,086	1	0,98113189	1,019230962	4
RPAP1	0,226351275	-0,027502046	0,031	0,008	1	0,981117584	1,019245824	3,875
CTU1	0,224763613	-0,027512971	0,062	0,023	1	0,981110154	1,019253543	2,695652174
ATF2	0,00145178	-0,027552134	0,406	0,137	1	0,981083521	1,019281211	2,96350365
CARD8	0,087959196	-0,027562406	0,062	0,016	1	0,981076536	1,019288469	3,875
ZNF268	0,019236133	-0,0276119	0,094	0,02	1	0,981042879	1,019323437	4,7
PAPPA2	0,082317662	-0,027624251	0,031	0,004	1	0,98103448	1,019332164	7,75
RAP2C	0,019580952	-0,027700408	0,156	0,047	1	0,980982695	1,019385974	3,319148936
SHISA5	0,002067673	-0,02774971	0,375	0,125	1	0,980949172	1,019420811	3
SUSD1	0,226351275	-0,027844014	0,031	0,008	1	0,980885052	1,019487449	3,875
NIPA2	0,001963481	-0,027889236	0,281	0,082	1	0,980854307	1,019519405	3,426829268
PTDSS1	0,019236133	-0,02811176	0,094	0,02	1	0,980703029	1,019676671	4,7
MBIP	0,01057519	-0,028119857	0,219	0,07	1	0,980697526	1,019682393	3,128571429
TAFAZZIN	0,006070525	-0,028174875	0,25	0,078	1	0,980660127	1,01972128	3,205128205
ACVRL1	0,543117005	-0,02828215	0	0,012	1	0,980587211	1,019797106	0
CYP4V2	0,008956837	-0,028321333	0,125	0,027	1	0,980560578	1,019824805	4,62962963
YARS1	0,014643669	-0,028329774	0,219	0,074	1	0,980554841	1,019830772	2,959459459
UCN	0,082317662	-0,028450744	0,031	0,004	1	0,980472625	1,019916287	7,75
PSMC6	0,000280867	-0,028457664	0,469	0,145	1	0,980467922	1,01992118	3,234482759
GLIS2	0,540537634	-0,028479791	0,031	0,016	1	0,980452884	1,019936823	1,9375

Supplementary Table 1

Differentially expressed genes_adjacent-skin-XL_ comparing SC-Keloid vs. (SC-Myel, SC-Nonmyel)								
**gene**	**p_val**	**avg_log2FC**	**pct.1**	**pct.2**	**p_val_adj**	**Foldchange_UP**	**Foldchange_DOWN**	**Ratio_pct1_pct2**
FGF13	0,226351275	-0,028483845	0,031	0,008	1	0,98045013	1,019939688	3,875
SHLD2	0,066791287	-0,028488497	0,125	0,043	1	0,980446968	1,019942978	2,906976744
STPG1	0,386325862	-0,028540535	0,031	0,012	1	0,980411604	1,019979767	2,583333333
CAPN2	0,003873867	-0,028547095	0,438	0,168	1	0,980407146	1,019984405	2,607142857
ZNF175	0,668867248	-0,028578779	0,031	0,02	1	0,980385615	1,020006806	1,55
ZNF680	0,016858455	-0,028672437	0,125	0,031	1	0,980321972	1,020073026	4,032258065
ZNF224	0,062559189	-0,028786161	0,125	0,043	1	0,980244698	1,020153439	2,906976744
C21orf58	0,041848356	-0,028790222	0,062	0,012	1	0,980241939	1,020156311	5,166666667
ATF6B	0,004352817	-0,028790249	0,281	0,09	1	0,98024192	1,02015633	3,122222222
HACD1	0,150155729	-0,028798345	0,062	0,02	1	0,98023642	1,020162055	3,1
TRAPPC13	0,000965846	-0,028823201	0,219	0,051	1	0,980219531	1,020179631	4,294117647
TMED4	0,013290145	-0,028839829	0,562	0,262	1	0,980208234	1,020191389	2,145038168
AASDH	0,22892808	-0,028959053	0,125	0,062	1	0,980127233	1,020275701	2,016129032
TET3	0,087959196	-0,028961953	0,062	0,016	1	0,980125263	1,020277752	3,875
KCNG1	0,022581316	-0,028971071	0,188	0,062	1	0,980119069	1,0202842	3,032258065
ZNF408	0,019236133	-0,029117517	0,094	0,02	1	0,980019583	1,020387773	4,7
EIF3J	0,012558306	-0,029308493	0,562	0,254	1	0,979889862	1,020522856	2,212598425
L3MBTL4	0,540537634	-0,029326479	0,031	0,016	1	0,979877646	1,020535578	1,9375
IRGQ	0,224763613	-0,029355656	0,062	0,023	1	0,979857829	1,020556217	2,695652174
LEPROT	0,012926041	-0,029398382	0,719	0,336	1	0,979828811	1,020586442	2,139880952
EXOSC6	0,000460086	-0,029406842	0,219	0,047	1	0,979823065	1,020592427	4,659574468
IL1RAPL1	0,318241523	-0,029406877	0,156	0,094	1	0,979823041	1,020592452	1,659574468
SH3BP5L	0,911838915	-0,029449697	0,031	0,027	1	0,97979396	1,020622744	1,148148148
SLC30A6	0,224763613	-0,029453335	0,062	0,023	1	0,979791489	1,020625318	2,695652174
GTPBP10	0,150155729	-0,029466191	0,062	0,02	1	0,979782758	1,020634413	3,1
CEPT1	0,063925678	-0,029492482	0,094	0,027	1	0,979764903	1,020653012	3,481481481
SEC22A	0,002053304	-0,029548359	0,156	0,031	1	0,979726957	1,020692544	5,032258065
CA12	0,543117005	-0,029565934	0	0,012	1	0,979715022	1,020704978	0
RIC1	0,224763613	-0,029573986	0,062	0,023	1	0,979709554	1,020710675	2,695652174
PPP2R5D	0,001013976	-0,029580662	0,188	0,039	1	0,97970502	1,020715399	4,820512821

Supplementary Table 1

Differentially expressed genes_adjacent-skin-XL_ comparing SC-Keloid vs. (SC-Myel, SC-Nonmyel)								
**gene**	**p_val**	**avg_log2FC**	**pct.1**	**pct.2**	**p_val_adj**	**Foldchange_UP**	**Foldchange_DOWN**	**Ratio_pct1_pct2**
HUWE1	0,029364342	-0,029607009	0,375	0,172	1	0,979687129	1,020734039	2,180232558
GK5	0,087959196	-0,029668855	0,062	0,016	1	0,979645132	1,020777797	3,875
TNFAIP8L3	0,041848356	-0,029676128	0,062	0,012	1	0,979640193	1,020782943	5,166666667
EXPH5	0,543117005	-0,029700381	0	0,012	1	0,979623725	1,020800104	0
ZBTB14	0,087959196	-0,029702255	0,062	0,016	1	0,979622452	1,02080143	3,875
TNPO1	0,000728803	-0,029719992	0,438	0,145	1	0,979610408	1,02081398	3,020689655
PPP1R12C	0,475301513	-0,029724765	0,156	0,105	1	0,979607168	1,020817357	1,485714286
KTI12	0,087959196	-0,029729641	0,062	0,016	1	0,979603857	1,020820807	3,875
AGAP5	0,226351275	-0,029731017	0,031	0,008	1	0,979602923	1,020821781	3,875
KCTD20	0,024505233	-0,029767547	0,25	0,098	1	0,979578118	1,020847629	2,551020408
E2F4	0,003004599	-0,029773932	0,219	0,059	1	0,979573783	1,020852147	3,711864407
FAM20B	0,014828336	-0,029778818	0,219	0,074	1	0,979570466	1,020855604	2,959459459
ZNF93	0,041848356	-0,029790355	0,062	0,012	1	0,979562632	1,020863768	5,166666667
ABHD14A	0,031706938	-0,029798246	0,188	0,066	1	0,979557274	1,020869352	2,848484848
BAIAP2-DT	0,141661018	-0,029805414	0,094	0,035	1	0,979552407	1,020874424	2,685714286
PAXIP1-DT	0,291239073	-0,029812576	0,094	0,047	1	0,979547544	1,020879492	2
RAB30	0,178976513	-0,029897584	0,156	0,078	1	0,979489829	1,020939647	2
LGALS3BP	0,018884013	-0,029932792	0,875	0,438	1	0,979465925	1,020964563	1,997716895
INSYN1	0,80991663	-0,02993557	0,031	0,023	1	0,979464039	1,020966529	1,347826087
LINC01140	0,226351275	-0,02994528	0,031	0,008	1	0,979457447	1,0209734	3,875
ASNS	0,002594281	-0,02996069	0,094	0,012	1	0,979446985	1,020984306	7,833333333
KYAT1	0,543117005	-0,029994466	0	0,012	1	0,979424054	1,021008209	0
ZNF548	0,991060715	-0,030011146	0,031	0,031	1	0,979412731	1,021020014	1
NEMP2	0,036827385	-0,030107272	0,094	0,023	1	0,979347475	1,021088046	4,086956522
PI16	0,682170546	-0,030122623	0,031	0,02	1	0,979337055	1,021098911	1,55
DDX60L	0,150155729	-0,030164821	0,062	0,02	1	0,97930841	1,021128778	3,1
NAA15	0,031755723	-0,030200646	0,25	0,102	1	0,979284092	1,021154135	2,450980392
LRP11	0,13644998	-0,030276182	0,094	0,035	1	0,979232821	1,021207602	2,685714286
PAXBP1-AS1	0,386325862	-0,030285054	0,031	0,012	1	0,979226798	1,021213882	2,583333333
TMEM17	0,002096899	-0,030321024	0,156	0,031	1	0,979202384	1,021239343	5,032258065

Supplementary Table 1

Differentially expressed genes_adjacent-skin-XL_ comparing SC-Keloid vs. (SC-Myel, SC-Nonmyel)								
**gene**	**p_val**	**avg_log2FC**	**pct.1**	**pct.2**	**p_val_adj**	**Foldchange_UP**	**Foldchange_DOWN**	**Ratio_pct1_pct2**
CBFB	0,000103352	-0,0303993	0,531	0,16	1	0,979149258	1,021294754	3,31875
TENT5B	0,150155729	-0,030410668	0,062	0,02	1	0,979141542	1,021302801	3,1
RNF180	0,150155729	-0,030430883	0,062	0,02	1	0,979127822	1,021317112	3,1
ANGPTL1	0,478627389	-0,030439435	0,062	0,035	1	0,979122018	1,021323166	1,771428571
IL17RE	0,002096899	-0,030448199	0,156	0,031	1	0,979116071	1,02132937	5,032258065
LAT	0,533973819	-0,030474654	0,031	0,016	1	0,979098117	1,021348099	1,9375
SNAPC4	0,540537634	-0,030476525	0,031	0,016	1	0,979096847	1,021349424	1,9375
RCC1L	0,092647906	-0,030567058	0,125	0,047	1	0,979035408	1,021413518	2,659574468
RXYLT1	7,44701E-05	-0,03057176	0,312	0,07	1	0,979032217	1,021416847	4,457142857
APOL2	0,128344705	-0,030574964	0,156	0,07	1	0,979030043	1,021419115	2,228571429
MIR181A1HG	0,098751938	-0,030588303	0,094	0,031	1	0,979020991	1,021428559	3,032258065
RCBTB2	0,037506898	-0,030657234	0,094	0,023	1	0,978974215	1,021477364	4,086956522
RPA4	0,082317662	-0,030670581	0,031	0,004	1	0,978965158	1,021486814	7,75
TIAM1	0,543117005	-0,030858141	0	0,012	1	0,978837894	1,021619623	0
SPSB1	0,021070281	-0,030858826	0,156	0,047	1	0,978837429	1,021620108	3,319148936
GPER1	0,150155729	-0,030875445	0,062	0,02	1	0,978826153	1,021631877	3,1
AIG1	0,013467327	-0,030915427	0,344	0,137	1	0,978799027	1,02166019	2,510948905
TEFM	0,396289917	-0,03092546	0,062	0,031	1	0,978792221	1,021667294	2
MANSC1	0,543117005	-0,030970626	0	0,012	1	0,978761579	1,02169928	0
RTL6	0,224763613	-0,030971436	0,062	0,023	1	0,978761028	1,021699854	2,695652174
RHBDF2	0,062914349	-0,030981738	0,094	0,027	1	0,97875404	1,021707149	3,481481481
MIF4GD	0,00208125	-0,031008304	0,188	0,043	1	0,978736017	1,021725964	4,372093023
FLOT1	0,008322664	-0,031025853	0,594	0,254	1	0,978724111	1,021738392	2,338582677
B4GALT6	0,000204358	-0,03104337	0,312	0,078	1	0,978712228	1,021750798	4
ANKRD23	0,226351275	-0,031116462	0,031	0,008	1	0,978662644	1,021802565	3,875
CCDC77	0,019236133	-0,031126969	0,094	0,02	1	0,978655517	1,021810006	4,7
TMEM131	0,021727091	-0,031140664	0,219	0,078	1	0,978646227	1,021819706	2,807692308
DGKG	0,540537634	-0,031166014	0,031	0,016	1	0,978629031	1,021837661	1,9375
TNNI3K	0,543117005	-0,031200956	0	0,012	1	0,978605329	1,02186241	0
C1orf50	0,133058992	-0,031211083	0,094	0,035	1	0,97859846	1,021869583	2,685714286

Supplementary Table 1

Differentially expressed genes_adjacent-skin-XL_ comparing SC-Keloid vs. (SC-Myel, SC-Nonmyel)								
**gene**	**p_val**	**avg_log2FC**	**pct.1**	**pct.2**	**p_val_adj**	**Foldchange_UP**	**Foldchange_DOWN**	**Ratio_pct1_pct2**
ZBTB1	0,3182413	-0,031321138	0,156	0,094	1	0,978523811	1,021947539	1,659574468
WDR19	0,098751938	-0,03139988	0,094	0,031	1	0,978470405	1,022003318	3,032258065
ZNF749	0,028258709	-0,031537007	0,125	0,035	1	0,978377406	1,022100463	3,571428571
TBL3	0,183526587	-0,031546148	0,094	0,039	1	0,978371207	1,022106939	2,41025641
HUNK	0,004180266	-0,031568462	0,125	0,023	1	0,978356075	1,022122748	5,434782609
RETREG3	0,010405093	-0,031619202	0,188	0,055	1	0,978321666	1,022158697	3,418181818
EPB41L1	0,000339359	-0,031631226	0,156	0,023	1	0,978313513	1,022167216	6,782608696
OR7A5	0,386325862	-0,031650863	0,031	0,012	1	0,978300197	1,022181129	2,583333333
INTS4	0,002043075	-0,031682508	0,188	0,043	1	0,978278738	1,022203551	4,372093023
AP4B1	0,087959196	-0,031712739	0,062	0,016	1	0,978258239	1,022224971	3,875
MPP2	0,087959196	-0,031720931	0,062	0,016	1	0,978252685	1,022230775	3,875
ATG16L1	0,028712073	-0,031766452	0,125	0,035	1	0,978221818	1,02226303	3,571428571
C1orf216	0,918113127	-0,031769825	0,031	0,027	1	0,978219531	1,02226542	1,148148148
TM9SF4	0,006260028	-0,031799	0,188	0,051	1	0,978199749	1,022286093	3,68627451
STX2	0,001959565	-0,031829969	0,312	0,098	1	0,978178752	1,022308038	3,183673469
MCU	0,304406192	-0,031855648	0,062	0,027	1	0,97816134	1,022326235	2,296296296
URGCP	0,092647906	-0,031867459	0,125	0,047	1	0,978153333	1,022334604	2,659574468
CACYBP	0,000180922	-0,031899817	0,656	0,23	1	0,978131394	1,022357534	2,852173913
ACSS3	0,098751938	-0,031949102	0,094	0,031	1	0,97809798	1,02239246	3,032258065
LIAS	0,016858455	-0,032114647	0,125	0,031	1	0,977985753	1,022509783	4,032258065
SKP2	0,540537634	-0,032148676	0,031	0,016	1	0,977962685	1,022533902	1,9375
GANAB	0,046537241	-0,032153789	0,344	0,164	1	0,977959219	1,022537526	2,097560976
DPP9	0,080553141	-0,032155366	0,188	0,082	1	0,97795815	1,022538643	2,292682927
PPID	0,029760483	-0,032190446	0,156	0,051	1	0,97793437	1,022563508	3,058823529
CYP2R1	0,226351275	-0,032267477	0,031	0,008	1	0,977882156	1,022618107	3,875
UBP1	0,197139196	-0,032298975	0,125	0,059	1	0,977860807	1,022640434	2,118644068
ZMYM6	0,015314414	-0,032306914	0,188	0,059	1	0,977855425	1,022646062	3,186440678
ZNF771	0,147778936	-0,032316288	0,062	0,02	1	0,977849072	1,022652707	3,1
MAST2	0,016562293	-0,032347371	0,125	0,031	1	0,977828005	1,02267474	4,032258065
MATN4	0,386325862	-0,032358739	0,031	0,012	1	0,977820299	1,022682798	2,583333333

Supplementary Table 1

Differentially expressed genes_adjacent-skin-XL_ comparing SC-Keloid vs. (SC-Myel, SC-Nonmyel)								
**gene**	**p_val**	**avg_log2FC**	**pct.1**	**pct.2**	**p_val_adj**	**Foldchange_UP**	**Foldchange_DOWN**	**Ratio_pct1_pct2**
EFS	0,000712309	-0,032382453	0,531	0,184	1	0,977804227	1,022699608	2,885869565
ECRG4	0,082317662	-0,032388655	0,031	0,004	1	0,977800024	1,022704005	7,75
SLC25A15	0,226351275	-0,032430006	0,031	0,008	1	0,977771998	1,022733319	3,875
CPM	0,662254058	-0,0324437	0,031	0,02	1	0,977762717	1,022743026	1,55
PET117	0,008148126	-0,032448347	0,094	0,016	1	0,977759567	1,022746321	5,875
ACAT1	0,020057665	-0,032459623	0,25	0,094	1	0,977751925	1,022754314	2,659574468
ZNF517	0,386325862	-0,032491439	0,031	0,012	1	0,977730363	1,022776869	2,583333333
IRAK2	0,019236133	-0,032499191	0,094	0,02	1	0,977725109	1,022782365	4,7
SLC7A6OS	0,008780679	-0,032510991	0,125	0,027	1	0,977717113	1,02279073	4,62962963
HEBP2	0,000566964	-0,032519849	0,406	0,125	1	0,97771111	1,02279701	3,248
SUGP2	0,000190541	-0,032552025	0,406	0,117	1	0,977689304	1,022819822	3,47008547
LZTS1	0,040857201	-0,032617238	0,062	0,012	1	0,977645112	1,022866056	5,166666667
CCDC6	0,005195348	-0,032635905	0,219	0,062	1	0,977632462	1,022879292	3,532258065
BVES-AS1	0,226351275	-0,032692346	0,031	0,008	1	0,977594216	1,02291931	3,875
SULT1A1	0,915203992	-0,032734531	0,031	0,035	1	0,977565631	1,022949221	0,885714286
LGALS7	0,543117005	-0,032881228	0	0,012	1	0,977466235	1,023053242	0
OPTN	0,025330018	-0,032891759	0,594	0,285	1	0,977459099	1,02306071	2,084210526
HGSNAT	0,15375596	-0,032938244	0,219	0,113	1	0,977427606	1,023093674	1,938053097
WDR24	0,543117005	-0,032952293	0	0,012	1	0,977418087	1,023103637	0
ZFAND1	0,04173482	-0,032957372	0,188	0,07	1	0,977414646	1,023107239	2,685714286
PCSK1N	0,540537634	-0,032977908	0,031	0,016	1	0,977400733	1,023121803	1,9375
PAK4	0,007871757	-0,032997431	0,156	0,039	1	0,977387507	1,023135648	4
AMMECR1	0,226351275	-0,033045858	0,031	0,008	1	0,9773547	1,023169992	3,875
LARP4	0,029285816	-0,033073544	0,344	0,152	1	0,977335944	1,023189627	2,263157895
HUS1B	0,543117005	-0,033086127	0	0,012	1	0,977327419	1,023198552	0
RBMS3-AS3	0,682170546	-0,033135368	0,031	0,02	1	0,977294063	1,023233475	1,55
ZNF652	0,133804381	-0,033196794	0,281	0,148	1	0,977252453	1,023277043	1,898648649
TMEM254	0,041848356	-0,03324291	0,062	0,012	1	0,977221216	1,023309752	5,166666667
ICE1	0,00464375	-0,033314478	0,281	0,09	1	0,977172739	1,023360517	3,122222222
IFT80	0,001013976	-0,033356578	0,188	0,039	1	0,977144225	1,02339038	4,820512821

Supplementary Table 1

Differentially expressed genes_adjacent-skin-XL_ comparing SC-Keloid vs. (SC-Myel, SC-Nonmyel)								
**gene**	**p_val**	**avg_log2FC**	**pct.1**	**pct.2**	**p_val_adj**	**Foldchange_UP**	**Foldchange_DOWN**	**Ratio_pct1_pct2**
SLC25A19	0,386325862	-0,03337337	0,031	0,012	1	0,977132851	1,023402292	2,583333333
ZNF471	0,150155729	-0,033373868	0,062	0,02	1	0,977132514	1,023402646	3,1
ZNF764	0,540537634	-0,033388336	0,031	0,016	1	0,977122715	1,023412909	1,9375
NCOA4	0,007536877	-0,033417065	0,344	0,129	1	0,977103257	1,023433289	2,666666667
ZFP62	0,041848356	-0,033466257	0,062	0,012	1	0,977069941	1,023468186	5,166666667
INPP5F	0,000651351	-0,033483985	0,438	0,141	1	0,977057935	1,023480762	3,106382979
KLHL7-DT	0,543117005	-0,033532166	0	0,012	1	0,977025305	1,023514943	0
FBXO15	0,543117005	-0,033590818	0	0,012	1	0,976985585	1,023556555	0
PIP5K1C	0,044552052	-0,033592501	0,125	0,039	1	0,976984446	1,023557749	3,205128205
SPATA6	0,007993973	-0,033599449	0,656	0,297	1	0,976979741	1,023562678	2,208754209
FITM2	0,307936041	-0,033699415	0,062	0,027	1	0,976912047	1,023633604	2,296296296
TDRD9	0,013891036	-0,033712508	0,062	0,008	1	0,976903181	1,023642895	7,75
STIM2	0,152699481	-0,033722158	0,125	0,055	1	0,976896647	1,023649742	2,272727273
TSLP	0,386325862	-0,033745315	0,031	0,012	1	0,976880967	1,023666172	2,583333333
SNRNP48	0,13644998	-0,033777842	0,094	0,035	1	0,976858942	1,023689252	2,685714286
IQCG	0,044552052	-0,033788057	0,125	0,039	1	0,976852025	1,0236965	3,205128205
UBXN11	0,008148126	-0,033806535	0,094	0,016	1	0,976839514	1,023709612	5,875
PTCD3	0,001853924	-0,033877973	0,281	0,082	1	0,976791145	1,023760305	3,426829268
CHST10	0,682170546	-0,033947646	0,031	0,02	1	0,976743973	1,023809747	1,55
SEMA6D	0,030992783	-0,033967042	0,156	0,051	1	0,976730842	1,023823512	3,058823529
CTF1	0,392349482	-0,03406316	0,062	0,031	1	0,976665771	1,023891724	2
PIK3C3	0,02818312	-0,034067655	0,156	0,051	1	0,976662727	1,023894915	3,058823529
CBWD3	0,037506898	-0,034085612	0,094	0,023	1	0,976650571	1,023907659	4,086956522
USP20	0,911838915	-0,03417149	0,031	0,027	1	0,976592436	1,02396861	1,148148148
PDE12	0,007871757	-0,034223321	0,156	0,039	1	0,976557352	1,024005398	4
AGAP9	0,386325862	-0,034232286	0,031	0,012	1	0,976551283	1,024011762	2,583333333
SLC44A4	0,543117005	-0,034264201	0	0,012	1	0,976529681	1,024034415	0
UBFD1	0,000105488	-0,034272556	0,219	0,039	1	0,976524025	1,024040346	5,615384615
ZNF112	0,386325862	-0,034281774	0,031	0,012	1	0,976517786	1,024046888	2,583333333
PRRT3-AS1	0,063925678	-0,034304554	0,094	0,027	1	0,976502367	1,024063058	3,481481481

Supplementary Table 1

Differentially expressed genes_adjacent-skin-XL_ comparing SC-Keloid vs. (SC-Myel, SC-Nonmyel)								
**gene**	**p_val**	**avg_log2FC**	**pct.1**	**pct.2**	**p_val_adj**	**Foldchange_UP**	**Foldchange_DOWN**	**Ratio_pct1_pct2**
C3orf38	0,010253031	-0,034311936	0,188	0,055	1	0,97649737	1,024068298	3,418181818
THOC6	0,147986647	-0,034317778	0,156	0,074	1	0,976493416	1,024072445	2,108108108
MRPL49	0,029826788	-0,034319956	0,219	0,082	1	0,976491942	1,024073991	2,670731707
ELAC1	0,008956837	-0,03432179	0,125	0,027	1	0,976490701	1,024075292	4,62962963
SNIP1	0,285925963	-0,034333456	0,094	0,047	1	0,976482805	1,024083573	2
MED15	0,024538024	-0,034376967	0,406	0,188	1	0,976453355	1,02411446	2,159574468
LIPT1	0,543117005	-0,034397437	0	0,012	1	0,9764395	1,024128991	0
FNDC1	0,386325862	-0,034418365	0,031	0,012	1	0,976425336	1,024143847	2,583333333
PTPRG	0,543117005	-0,034477673	0	0,012	1	0,976385196	1,02418595	0
NSUN3	0,041848356	-0,034478518	0,062	0,012	1	0,976384624	1,02418655	5,166666667
DCAF11	0,007471523	-0,034516384	0,156	0,039	1	0,976358998	1,024213432	4
GRAMD1B	0,543117005	-0,034542276	0	0,012	1	0,976341476	1,024231813	0
HIC2	0,147778936	-0,034560802	0,062	0,02	1	0,976328939	1,024244966	3,1
SHB	0,682170546	-0,034564479	0,031	0,02	1	0,97632645	1,024247576	1,55
SATB2	0,226351275	-0,034593768	0,031	0,008	1	0,976306629	1,02426837	3,875
SNX1	0,003987873	-0,03460905	0,344	0,121	1	0,976296287	1,02427922	2,842975207
PSTK	0,224763613	-0,034685793	0,062	0,023	1	0,976244355	1,024333707	2,695652174
KPNA1	0,011181716	-0,03471187	0,312	0,117	1	0,97622671	1,024352222	2,666666667
PFKL	0,003182741	-0,034743681	0,375	0,133	1	0,976205185	1,024374809	2,819548872
HOXC-AS1	0,226351275	-0,034787949	0,031	0,008	1	0,976175231	1,024406242	3,875
SORD	0,098751938	-0,034939365	0,094	0,031	1	0,976072783	1,024513763	3,032258065
ACBD5	0,000801432	-0,034964367	0,312	0,09	1	0,976055868	1,024531518	3,466666667
ING4	0,005967942	-0,034968883	0,188	0,051	1	0,976052813	1,024534725	3,68627451
MPLKIP	0,001190114	-0,035042254	0,438	0,148	1	0,976003175	1,024586831	2,959459459
VMAC	0,087959196	-0,035049429	0,062	0,016	1	0,975998321	1,024591927	3,875
GIN1	0,028712073	-0,035102009	0,125	0,035	1	0,975962751	1,024629269	3,571428571
GORASP1	0,094667448	-0,035121158	0,094	0,031	1	0,975949797	1,024642869	3,032258065
MRPS10	0,005602602	-0,035163902	0,281	0,094	1	0,975920882	1,024673227	2,989361702
METTL25	0,150155729	-0,035173291	0,062	0,02	1	0,975914531	1,024679896	3,1
FGD1	0,226351275	-0,03518143	0,031	0,008	1	0,975909025	1,024685677	3,875

Supplementary Table 1

Differentially expressed genes_adjacent-skin-XL_ comparing SC-Keloid vs. (SC-Myel, SC-Nonmyel)								
**gene**	**p_val**	**avg_log2FC**	**pct.1**	**pct.2**	**p_val_adj**	**Foldchange_UP**	**Foldchange_DOWN**	**Ratio_pct1_pct2**
STK16	0,008956837	-0,03523651	0,125	0,027	1	0,975871767	1,024724799	4,62962963
H2AC11	0,543117005	-0,035253999	0	0,012	1	0,975859937	1,024737221	0
ZNF583	0,386325862	-0,035284098	0,031	0,012	1	0,975839578	1,0247586	2,583333333
ASPHD1	0,682170546	-0,035330532	0,031	0,02	1	0,975808171	1,024791583	1,55
PCDHB14	0,540537634	-0,035351206	0,031	0,016	1	0,975794187	1,024806269	1,9375
SPINT1	0,543117005	-0,03537745	0	0,012	1	0,975776437	1,024824911	0
OTUD7A	0,087959196	-0,035389228	0,062	0,016	1	0,975768471	1,024833278	3,875
BRD7	0,002023444	-0,035417546	0,406	0,141	1	0,975749318	1,024853394	2,879432624
UVRAG	0,102525773	-0,035455481	0,156	0,066	1	0,975723661	1,024880343	2,363636364
SPINDOC	0,123421204	-0,035462465	0,125	0,051	1	0,975718938	1,024885304	2,450980392
ATG12	0,02226642	-0,035479072	0,344	0,145	1	0,975707706	1,024897102	2,372413793
NNAT	0,543117005	-0,035517596	0	0,012	1	0,975681652	1,024924469	0
DRD4	0,386325862	-0,035532347	0,031	0,012	1	0,975671676	1,024934949	2,583333333
CEP19	0,226351275	-0,035627264	0,031	0,008	1	0,975607488	1,025002383	3,875
PSRC1	0,087959196	-0,035678367	0,062	0,016	1	0,97557293	1,025038691	3,875
MT1G	0,041848356	-0,035710945	0,062	0,012	1	0,975550901	1,025061838	5,166666667
AC093157.1	0,063925678	-0,035776033	0,094	0,027	1	0,975506889	1,025108086	3,481481481
GALM	0,016562293	-0,035781968	0,125	0,031	1	0,975502876	1,025112303	4,032258065
TARS2	0,002594281	-0,035809022	0,094	0,012	1	0,975484583	1,025131526	7,833333333
TFPI	0,150155729	-0,035894191	0,062	0,02	1	0,975426998	1,025192046	3,1
CLINT1	0,584692171	-0,035923421	0,188	0,137	1	0,975407236	1,025212817	1,372262774
TNNI2	0,543117005	-0,035966129	0	0,012	1	0,975378361	1,025243167	0
DLX1	0,386325862	-0,036038015	0,031	0,012	1	0,975329761	1,025294254	2,583333333
ZNF385D	0,226351275	-0,036111813	0,031	0,008	1	0,975279872	1,025346702	3,875
SMCO4	0,224763613	-0,036126166	0,062	0,023	1	0,975270169	1,025356903	2,695652174
COX15	0,000476445	-0,036154374	0,188	0,035	1	0,9752511	1,025376952	5,371428571
ZNF354A	0,246047587	-0,036221265	0,094	0,043	1	0,975205883	1,025424495	2,186046512
AC144652.1	0,386325862	-0,036302291	0,031	0,012	1	0,975151115	1,025482087	2,583333333
WASHC2A	0,118142322	-0,036367452	0,125	0,051	1	0,975107072	1,025528405	2,450980392
SLC35E2B	0,146633603	-0,036367609	0,125	0,055	1	0,975106966	1,025528516	2,272727273

Supplementary Table 1

Differentially expressed genes_adjacent-skin-XL_ comparing SC-Keloid vs. (SC-Myel, SC-Nonmyel)								
**gene**	**p_val**	**avg_log2FC**	**pct.1**	**pct.2**	**p_val_adj**	**Foldchange_UP**	**Foldchange_DOWN**	**Ratio_pct1_pct2**
AP2B1	0,000273539	-0,036402083	0,531	0,176	1	0,975083665	1,025553022	3,017045455
RPS6KL1	0,386325862	-0,036491	0,031	0,012	1	0,97502357	1,025616232	2,583333333
ACSS1	0,077467066	-0,036527617	0,156	0,062	1	0,974998823	1,025642264	2,516129032
MYO19	0,098751938	-0,036639373	0,094	0,031	1	0,9749233	1,025721716	3,032258065
NOMO1	3,05607E-05	-0,036667666	0,281	0,055	0,644860382	0,97490418	1,025741832	5,109090909
DMC1	0,386325862	-0,036722059	0,031	0,012	1	0,974867425	1,025780505	2,583333333
MED19	0,000146687	-0,036756534	0,281	0,062	1	0,97484413	1,025805018	4,532258065
SYNGAP1	0,087959196	-0,036769479	0,062	0,016	1	0,974835382	1,025814223	3,875
TGFB3	0,543117005	-0,036777127	0	0,012	1	0,974830215	1,02581966	0
C15orf40	0,015105004	-0,036927112	0,188	0,059	1	0,974728875	1,025926312	3,186440678
SLC16A8	0,226351275	-0,036969422	0,031	0,008	1	0,97470029	1,0259564	3,875
TNS3	0,019510793	-0,036972076	0,594	0,289	1	0,974698496	1,025958287	2,055363322
ERBIN	0,077894802	-0,036994693	0,406	0,215	1	0,974683216	1,025974371	1,888372093
GEMIN4	0,386325862	-0,036995692	0,031	0,012	1	0,974682542	1,025975081	2,583333333
BBS10	0,221757959	-0,037013933	0,062	0,023	1	0,974670218	1,025988054	2,695652174
TRIM45	0,386325862	-0,037038375	0,031	0,012	1	0,974653705	1,026005437	2,583333333
TGFBRAP1	0,141661018	-0,037042244	0,094	0,035	1	0,974651091	1,026008188	2,685714286
MIR181A2HG	0,226351275	-0,03706628	0,031	0,008	1	0,974634853	1,026025282	3,875
SLC20A2	0,012009804	-0,037095335	0,281	0,105	1	0,974615225	1,026045946	2,676190476
RELA	0,008282358	-0,037118992	0,312	0,113	1	0,974599244	1,02606277	2,761061947
RPP25	0,386325862	-0,037120994	0,031	0,012	1	0,974597891	1,026064194	2,583333333
NEURL1B	0,004267025	-0,037124896	0,156	0,035	1	0,974595255	1,026066969	4,457142857
DNAAF10	0,224763613	-0,037163543	0,062	0,023	1	0,974569148	1,026094456	2,695652174
MUC1	0,543117005	-0,03721724	0	0,012	1	0,974532876	1,026132648	0
FBXL6	0,001034587	-0,03723123	0,188	0,039	1	0,974523425	1,026142598	4,820512821
CORO1A	0,226351275	-0,037289131	0,031	0,008	1	0,974484315	1,026183782	3,875
SPATA20	0,06901054	-0,037291147	0,188	0,078	1	0,974482953	1,026185217	2,41025641
LDAH	0,082317662	-0,037294082	0,031	0,004	1	0,974480971	1,026187304	7,75
RPP40	0,008148126	-0,037358742	0,094	0,016	1	0,974437296	1,026233298	5,875
NFXL1	0,008956837	-0,037375296	0,125	0,027	1	0,974426115	1,026245073	4,62962963

Supplementary Table 1

Differentially expressed genes_adjacent-skin-XL_ comparing SC-Keloid vs. (SC-Myel, SC-Nonmyel)								
**gene**	**p_val**	**avg_log2FC**	**pct.1**	**pct.2**	**p_val_adj**	**Foldchange_UP**	**Foldchange_DOWN**	**Ratio_pct1_pct2**
MIER2	0,041848356	-0,037389911	0,062	0,012	1	0,974416244	1,026255469	5,166666667
TP53	0,004681177	-0,037412686	0,219	0,062	1	0,974400862	1,02627167	3,532258065
FASTKD3	0,226351275	-0,037429898	0,031	0,008	1	0,974389237	1,026283914	3,875
OTUB1	0,001535057	-0,037492306	0,312	0,094	1	0,974347088	1,02632831	3,319148936
EDC3	0,386325862	-0,037540435	0,031	0,012	1	0,974314583	1,02636255	2,583333333
MEN1	0,087959196	-0,037551439	0,062	0,016	1	0,974307152	1,026370379	3,875
MTRR	0,540537634	-0,037652238	0,031	0,016	1	0,974239081	1,026442092	1,9375
PCAT6	0,540537634	-0,037676253	0,031	0,016	1	0,974222863	1,026459178	1,9375
ASAH2B	0,082317662	-0,037719141	0,031	0,004	1	0,974193903	1,026489693	7,75
CDON	0,543117005	-0,037729997	0	0,012	1	0,974186572	1,026497417	0
CHD1L	0,543117005	-0,038003713	0	0,012	1	0,974001762	1,026692188	0
FAR2	0,019236133	-0,038043668	0,094	0,02	1	0,973974788	1,026720622	4,7
KCNK6	0,386325862	-0,038076872	0,031	0,012	1	0,973952371	1,026744253	2,583333333
GCHFR	0,543117005	-0,03808296	0	0,012	1	0,973948262	1,026748585	0
TNPO3	0,000257882	-0,038112285	0,219	0,043	1	0,973928465	1,026769456	5,093023256
CAPS	0,0501673	-0,038123163	0,281	0,129	1	0,973921122	1,026777198	2,178294574
EXTL3	0,392349482	-0,038132341	0,062	0,031	1	0,973914925	1,02678373	2
HAUS7	0,009522001	-0,038141521	0,188	0,055	1	0,973908728	1,026790264	3,418181818
TM2D2	0,006461838	-0,03817132	0,188	0,051	1	0,973888613	1,026811472	3,68627451
PPIP5K1	0,543117005	-0,038319539	0	0,012	1	0,973788562	1,02691697	0
SRP68	0,040359751	-0,038349556	0,219	0,086	1	0,973768302	1,026938336	2,546511628
HSPBAP1	0,578217897	-0,038556967	0,062	0,039	1	0,973628317	1,027085986	1,58974359
FCSK	0,540537634	-0,038609291	0,031	0,016	1	0,973593006	1,027123237	1,9375
NKAPL	0,543117005	-0,038733975	0	0,012	1	0,973508867	1,02721201	0
ING2	0,000257365	-0,038787419	0,281	0,066	1	0,973472805	1,027250063	4,257575758
PLEKHH1	0,139907281	-0,038828106	0,094	0,035	1	0,973445351	1,027279034	2,685714286
CAMK1	0,000257882	-0,038864875	0,219	0,043	1	0,973420542	1,027305216	5,093023256
TYW5	0,803381388	-0,038940852	0,031	0,023	1	0,97336928	1,027359319	1,347826087
PLEKHM2	0,003622253	-0,038964787	0,375	0,137	1	0,973353131	1,027376363	2,737226277
OPLAH	0,543117005	-0,038981611	0	0,012	1	0,973341781	1,027388344	0

Supplementary Table 1

Differentially expressed genes_adjacent-skin-XL_ comparing SC-Keloid vs. (SC-Myel, SC-Nonmyel)								
**gene**	**p_val**	**avg_log2FC**	**pct.1**	**pct.2**	**p_val_adj**	**Foldchange_UP**	**Foldchange_DOWN**	**Ratio_pct1_pct2**
NECAB3	0,037506898	-0,03903078	0,094	0,023	1	0,973308608	1,027423359	4,086956522
CXADR	0,540537634	-0,039075561	0,031	0,016	1	0,973278397	1,027455251	1,9375
LENG1	0,011446319	-0,039162042	0,219	0,07	1	0,973220057	1,027516843	3,128571429
PRKCZ	0,087959196	-0,039168829	0,062	0,016	1	0,973215479	1,027521676	3,875
PTGES	0,226351275	-0,039313277	0,031	0,008	1	0,973118041	1,027624561	3,875
EYA2	0,480650464	-0,03939156	0	0,016	1	0,97306524	1,027680323	0
B9D2	0,008148126	-0,039470341	0,094	0,016	1	0,973012105	1,027736443	5,875
SENP1	0,019236133	-0,039476779	0,094	0,02	1	0,973007763	1,027741029	4,7
TERF2IP	0,00629843	-0,039542182	0,688	0,297	1	0,972963654	1,027787622	2,316498316
ADGRG6	0,000132242	-0,039567384	0,5	0,152	1	0,972946658	1,027805576	3,289473684
TRIM34	0,540537634	-0,039663887	0,031	0,016	1	0,972881579	1,027874329	1,9375
NSDHL	0,098751938	-0,039768376	0,094	0,031	1	0,972811119	1,027948777	3,032258065
STRIP1	0,098751938	-0,039772713	0,094	0,031	1	0,972808195	1,027951867	3,032258065
CCDC97	0,540537634	-0,039793488	0,031	0,016	1	0,972794186	1,02796667	1,9375
SLC17A5	0,540537634	-0,039814813	0,031	0,016	1	0,972779807	1,027981864	1,9375
MAP6D1	0,386325862	-0,039914836	0,031	0,012	1	0,972712366	1,028053137	2,583333333
CCDC180	0,226351275	-0,039948409	0,031	0,008	1	0,97268973	1,028077062	3,875
CCDC3	0,285925963	-0,039950524	0,094	0,047	1	0,972688305	1,028078569	2
GPN3	0,005677526	-0,039957988	0,25	0,078	1	0,972683272	1,028083888	3,205128205
SETD1B	0,30793671	-0,040025988	0,062	0,027	1	0,972637427	1,028132347	2,296296296
ICMT	0,000484576	-0,040047873	0,25	0,059	1	0,972622672	1,028147943	4,237288136
HK2	0,041848356	-0,040144121	0,062	0,012	1	0,972557787	1,028216537	5,166666667
EXOG	0,030992783	-0,040206807	0,156	0,051	1	0,97251553	1,028261215	3,058823529
DUS1L	0,006917668	-0,040216614	0,219	0,066	1	0,972508918	1,028268205	3,318181818
ZSCAN31	0,543117005	-0,040421726	0	0,012	1	0,972370664	1,028414407	0
SLC35A5	0,093753684	-0,040441995	0,125	0,047	1	0,972357003	1,028428855	2,659574468
NMNAT3	0,543117005	-0,040462078	0	0,012	1	0,972343468	1,028443172	0
PFAS	0,226351275	-0,04053868	0,031	0,008	1	0,972291841	1,02849778	3,875
DUS2	0,540537634	-0,040649077	0,031	0,016	1	0,972217443	1,028576485	1,9375
ADCK2	0,001013976	-0,040661523	0,188	0,039	1	0,972209056	1,028585358	4,820512821

Supplementary Table 1

Differentially expressed genes_adjacent-skin-XL_ comparing SC-Keloid vs. (SC-Myel, SC-Nonmyel)								
**gene**	**p_val**	**avg_log2FC**	**pct.1**	**pct.2**	**p_val_adj**	**Foldchange_UP**	**Foldchange_DOWN**	**Ratio_pct1_pct2**
SGF29	0,037506898	-0,04069555	0,094	0,023	1	0,972186125	1,028609619	4,086956522
SNX21	0,009664375	-0,040755251	0,188	0,055	1	0,972145896	1,028652185	3,418181818
WDR62	0,386325862	-0,04078563	0,031	0,012	1	0,972125425	1,028673846	2,583333333
ACSL3	0,001076629	-0,040804881	0,406	0,133	1	0,972112454	1,028687572	3,052631579
HYPK	0,27733921	-0,040857569	0,125	0,066	1	0,972076952	1,028725141	1,893939394
PTPN3	0,540537634	-0,040883633	0,031	0,016	1	0,972059391	1,028743727	1,9375
NUB1	0,000314728	-0,040927402	0,469	0,148	1	0,9720299	1,028774938	3,168918919
EBNA1BP2	0,00027287	-0,04093556	0,344	0,094	1	0,972024404	1,028780755	3,659574468
GNG12-AS1	0,543117005	-0,04093857	0	0,012	1	0,972022376	1,028782901	0
TRIM3	0,063387391	-0,040939097	0,125	0,043	1	0,97202202	1,028783277	2,906976744
ODF3L1	0,540537634	-0,040955676	0,031	0,016	1	0,972010851	1,028795099	1,9375
FBXO6	0,082317662	-0,040961452	0,031	0,004	1	0,972006959	1,028799219	7,75
TNFRSF10B	0,18979066	-0,040972075	0,094	0,039	1	0,971999802	1,028806794	2,41025641
ZBTB5	0,564954469	-0,041040839	0,062	0,039	1	0,971953474	1,028855832	1,58974359
PAIP2	0,016755261	-0,041086214	0,688	0,32	1	0,971922905	1,028888191	2,15
ASPG	0,226351275	-0,041106692	0,031	0,008	1	0,971909109	1,028902796	3,875
ZNRD2	0,000383854	-0,041131918	0,312	0,082	1	0,971892115	1,028920787	3,804878049
XRCC3	0,543117005	-0,041198083	0	0,012	1	0,971847543	1,028967976	0
ZRANB3	0,386325862	-0,041204288	0,031	0,012	1	0,971843364	1,028972402	2,583333333
DRAM1	0,785705341	-0,041230349	0,062	0,051	1	0,971825808	1,028990989	1,215686275
NUP85	0,000257882	-0,041321635	0,219	0,043	1	0,971764318	1,029056101	5,093023256
FLAD1	0,002005544	-0,041325268	0,188	0,043	1	0,971761872	1,029058692	4,372093023
FCRLA	0,543117005	-0,041363847	0	0,012	1	0,971735886	1,02908621	0
RNF216	0,010903421	-0,041392171	0,25	0,086	1	0,971716808	1,029106414	2,906976744
TEN1	0,560566066	-0,041453586	0,062	0,039	1	0,971675443	1,029150224	1,58974359
FBF1	0,803381388	-0,041463313	0,031	0,023	1	0,971668892	1,029157162	1,347826087
IL10RB	0,015394705	-0,041471178	0,219	0,074	1	0,971663595	1,029162774	2,959459459
TRMT2A	0,003151082	-0,041489836	0,219	0,059	1	0,971651029	1,029176083	3,711864407
ZNF615	0,543117005	-0,041532933	0	0,012	1	0,971622004	1,029206827	0
SOX17	0,533973819	-0,041567046	0,031	0,016	1	0,97159903	1,029231164	1,9375

Supplementary Table 1

Differentially expressed genes_adjacent-skin-XL_ comparing SC-Keloid vs. (SC-Myel, SC-Nonmyel)								
**gene**	**p_val**	**avg_log2FC**	**pct.1**	**pct.2**	**p_val_adj**	**Foldchange_UP**	**Foldchange_DOWN**	**Ratio_pct1_pct2**
SYCP2	0,224763613	-0,041576793	0,062	0,023	1	0,971592465	1,029238118	2,695652174
PDE7B	0,682170546	-0,041581962	0,031	0,02	1	0,971588985	1,029241805	1,55
OMA1	0,063925678	-0,041605471	0,094	0,027	1	0,971573152	1,029258577	3,481481481
NUDT14	0,008956837	-0,04189755	0,125	0,027	1	0,971376473	1,029466976	4,62962963
EYA3	0,150155729	-0,041901633	0,062	0,02	1	0,971373724	1,029469889	3,1
ARAP3	0,540537634	-0,041935584	0,031	0,016	1	0,971350865	1,029494116	1,9375
NOD1	0,682170546	-0,041936148	0,031	0,02	1	0,971350486	1,029494518	1,55
STRADB	0,30793671	-0,041980579	0,062	0,027	1	0,971320571	1,029526224	2,296296296
IL1RAPL2	0,037506898	-0,042016715	0,094	0,023	1	0,971296242	1,029552012	4,086956522
FBXL12	0,000467498	-0,042024933	0,25	0,059	1	0,97129071	1,029557876	4,237288136
GMPR2	0,030583271	-0,04202603	0,188	0,066	1	0,97128997	1,02955866	2,848484848
GTF3C1	0,000475965	-0,042035072	0,25	0,059	1	0,971283883	1,029565112	4,237288136
SURF6	0,004475279	-0,042047445	0,219	0,062	1	0,971275553	1,029573942	3,532258065
AP001816.1	0,004107327	-0,042208395	0,156	0,035	1	0,971167202	1,02968881	4,457142857
ZNF606	0,013891036	-0,042223952	0,062	0,008	1	0,97115673	1,029699913	7,75
DHX9	0,017693233	-0,042255474	0,406	0,176	1	0,971135511	1,029722412	2,306818182
WDR20	0,199149424	-0,04229383	0,156	0,078	1	0,971109692	1,029749789	2
SPATA22	0,543117005	-0,042390587	0	0,012	1	0,971044565	1,029818853	0
MFSD5	0,386325862	-0,042484149	0,031	0,012	1	0,970981592	1,029885641	2,583333333
PABPC4	0,03539645	-0,042566047	0,312	0,137	1	0,970926474	1,029944107	2,277372263
KATNAL2	0,386325862	-0,042580317	0,031	0,012	1	0,97091687	1,029954294	2,583333333
ZNF709	0,543117005	-0,042627073	0	0,012	1	0,970885405	1,029987674	0
ZC3H13	0,019816745	-0,04267733	0,438	0,191	1	0,970851584	1,030023555	2,293193717
CHP2	0,480650464	-0,042717749	0	0,016	1	0,970824384	1,030052413	0
ICA1L	0,224763613	-0,042726126	0,062	0,023	1	0,970818748	1,030058394	2,695652174
ADAP1	0,366622408	-0,042765195	0,125	0,074	1	0,970792458	1,030086289	1,689189189
SWSAP1	0,386325862	-0,042779949	0,031	0,012	1	0,97078253	1,030096823	2,583333333
MAP3K8	0,236345884	-0,042814006	0,094	0,043	1	0,970759613	1,030121141	2,186046512
ZBTB34	0,543117005	-0,042877985	0	0,012	1	0,970716564	1,030166824	0
OSBPL2	0,191913299	-0,042883008	0,094	0,039	1	0,970713184	1,030170411	2,41025641

Supplementary Table 1

Differentially expressed genes_adjacent-skin-XL_ comparing SC-Keloid vs. (SC-Myel, SC-Nonmyel)								
**gene**	**p_val**	**avg_log2FC**	**pct.1**	**pct.2**	**p_val_adj**	**Foldchange_UP**	**Foldchange_DOWN**	**Ratio_pct1_pct2**
RAD9A	0,000920564	-0,042924336	0,25	0,062	1	0,970685377	1,030199922	4,032258065
RMDN3	0,044149392	-0,042950114	0,188	0,07	1	0,970668033	1,03021833	2,685714286
CLEC2D	0,285925963	-0,043014878	0,094	0,047	1	0,97062446	1,030264578	2
C6orf120	0,138170297	-0,043031942	0,094	0,035	1	0,97061298	1,030276764	2,685714286
TAF15	0,023441759	-0,043140456	0,281	0,113	1	0,970539977	1,030354261	2,486725664
SLC27A6	0,543117005	-0,043144394	0	0,012	1	0,970537327	1,030357073	0
MEIS1	0,480650464	-0,04346028	0	0,016	1	0,970324846	1,0305827	0
TMEM116	0,087959196	-0,043483489	0,062	0,016	1	0,970309237	1,030599279	3,875
DUOX2	0,543117005	-0,043487924	0	0,012	1	0,970306254	1,030602447	0
ZBED8	0,540537634	-0,043548829	0,031	0,016	1	0,970265292	1,030645957	1,9375
SNAI2	0,087959196	-0,043596387	0,062	0,016	1	0,970233308	1,030679932	3,875
MMAA	0,540537634	-0,043614605	0,031	0,016	1	0,970221056	1,030692947	1,9375
PAFAH1B2	0,011766264	-0,043676464	0,375	0,152	1	0,970179457	1,030737141	2,467105263
SMAD9	0,226351275	-0,043682798	0,031	0,008	1	0,970175197	1,030741667	3,875
SLC35A1	0,012380103	-0,043695313	0,156	0,043	1	0,970166781	1,030750608	3,627906977
PLEKHH2	0,543117005	-0,043728283	0	0,012	1	0,97014461	1,030774165	0
SOCS5	0,028258709	-0,043736471	0,125	0,035	1	0,970139104	1,030780014	3,571428571
MORF4L1	0,474416485	-0,043831464	0,875	0,668	1	0,970075228	1,030847888	1,30988024
ATP7B	0,543117005	-0,043883936	0	0,012	1	0,970039946	1,030885381	0
ZNF425	0,543117005	-0,043990111	0	0,012	1	0,969968559	1,030961252	0
SUPT16H	0,004077882	-0,043992289	0,438	0,168	1	0,969967095	1,030962808	2,607142857
RBL1	0,045196862	-0,044125302	0,125	0,039	1	0,96987767	1,031057865	3,205128205
C1orf131	0,019236133	-0,044147515	0,094	0,02	1	0,969862737	1,03107374	4,7
ZNF23	0,036827385	-0,044172376	0,094	0,023	1	0,969846025	1,031091508	4,086956522
PPARG	0,386325862	-0,044199665	0,031	0,012	1	0,969827679	1,031111012	2,583333333
RSPH4A	0,543117005	-0,044260118	0	0,012	1	0,969787042	1,031154219	0
TUBGCP6	0,224763613	-0,044297071	0,062	0,023	1	0,969762202	1,031180631	2,695652174
HINT3	0,187685368	-0,044339477	0,094	0,039	1	0,969733698	1,031210942	2,41025641
BCAN	0,543117005	-0,044356709	0	0,012	1	0,969722115	1,031223259	0
CORO6	0,543117005	-0,044368515	0	0,012	1	0,969714179	1,031231698	0

Supplementary Table 1

Differentially expressed genes_adjacent-skin-XL_ comparing SC-Keloid vs. (SC-Myel, SC-Nonmyel)								
**gene**	**p_val**	**avg_log2FC**	**pct.1**	**pct.2**	**p_val_adj**	**Foldchange_UP**	**Foldchange_DOWN**	**Ratio_pct1_pct2**
DNER	0,543117005	-0,044369355	0	0,012	1	0,969713615	1,031232299	0
GMEB2	0,803381388	-0,044395312	0,031	0,023	1	0,969696168	1,031250852	1,347826087
PROSER3	0,540537634	-0,044427622	0,031	0,016	1	0,969674451	1,031273948	1,9375
MTERF1	0,540537634	-0,044542246	0,031	0,016	1	0,969597412	1,031355888	1,9375
KLHL7	0,004186485	-0,04459756	0,156	0,035	1	0,969560238	1,031395431	4,457142857
CXXC1	0,062941189	-0,044603232	0,156	0,059	1	0,969556427	1,031399486	2,644067797
QRSL1	0,396289328	-0,044647824	0,062	0,031	1	0,969526459	1,031431366	2
CLDN15	0,063925678	-0,044739892	0,094	0,027	1	0,969464589	1,031497191	3,481481481
KLK11	0,543117005	-0,044761309	0	0,012	1	0,969450197	1,031512503	0
YIPF1	0,000911755	-0,044843802	0,156	0,027	1	0,969394766	1,031571487	5,777777778
STARD4-AS1	0,682170546	-0,044927799	0,031	0,02	1	0,969338327	1,031631549	1,55
ABCG1	0,682170546	-0,044978634	0,031	0,02	1	0,969304172	1,0316679	1,55
KMT5C	0,609434411	-0,044989651	0,094	0,066	1	0,96929677	1,031675779	1,424242424
VSIG10	0,019236133	-0,044990965	0,094	0,02	1	0,969295887	1,031676719	4,7
SKIV2L	0,063925678	-0,045123284	0,094	0,027	1	0,969206991	1,031771345	3,481481481
RBM10	0,014095341	-0,045169317	0,188	0,059	1	0,969176066	1,031804267	3,186440678
THOC3	0,028712073	-0,045199685	0,125	0,035	1	0,969155666	1,031825985	3,571428571
ANKMY1	0,543117005	-0,045200121	0	0,012	1	0,969155373	1,031826297	0
IFITM10	0,386325862	-0,045204165	0,031	0,012	1	0,969152656	1,03182919	2,583333333
MFHAS1	0,028712073	-0,045215422	0,125	0,035	1	0,969145094	1,031837241	3,571428571
C1orf162	0,480650464	-0,04521791	0	0,016	1	0,969143423	1,03183902	0
MGAT5	0,224763613	-0,04524352	0,062	0,023	1	0,969126219	1,031857338	2,695652174
KIF9-AS1	0,543117005	-0,045293207	0	0,012	1	0,969092843	1,031892875	0
AQR	0,002053304	-0,045295976	0,156	0,031	1	0,969090983	1,031894856	5,032258065
NOP53	0,095825261	-0,045313993	0,812	0,438	1	0,96907888	1,031907743	1,853881279
H4C5	0,540537634	-0,04531609	0,031	0,016	1	0,969077472	1,031909243	1,9375
TBCK	0,185597364	-0,045351027	0,094	0,039	1	0,969054005	1,031934232	2,41025641
RASL11A	0,543117005	-0,045355353	0	0,012	1	0,969051098	1,031937327	0
CASTOR1	0,037506898	-0,045505262	0,094	0,023	1	0,968950411	1,03204456	4,086956522
HIP1	0,34617705	-0,045527904	0,156	0,094	1	0,968935204	1,032060757	1,659574468

Supplementary Table 1

Differentially expressed genes_adjacent-skin-XL_ comparing SC-Keloid vs. (SC-Myel, SC-Nonmyel)								
**gene**	**p_val**	**avg_log2FC**	**pct.1**	**pct.2**	**p_val_adj**	**Foldchange_UP**	**Foldchange_DOWN**	**Ratio_pct1_pct2**
ZDHHC14	0,212914042	-0,04554269	0,062	0,023	1	0,968925273	1,032071335	2,695652174
SMARCA4	7,44701E-05	-0,045587064	0,312	0,07	1	0,968895472	1,032103079	4,457142857
ZNF574	0,028712073	-0,045638824	0,125	0,035	1	0,968860711	1,032140109	3,571428571
IL18	0,543117005	-0,045647039	0	0,012	1	0,968855194	1,032145986	0
KIAA1549	0,543117005	-0,045659374	0	0,012	1	0,968846911	1,032154811	0
SALL2	0,087959196	-0,045661542	0,062	0,016	1	0,968845455	1,032156362	3,875
TBP	0,150155729	-0,045671491	0,062	0,02	1	0,968838774	1,03216348	3,1
CLN6	0,682170546	-0,04570753	0,031	0,02	1	0,968814572	1,032189264	1,55
RNASEH2A	0,540537634	-0,045759326	0,031	0,016	1	0,96877979	1,032226322	1,9375
CCDC78	0,543117005	-0,045769089	0	0,012	1	0,968773234	1,032233308	0
ACAD10	0,087959196	-0,045771286	0,062	0,016	1	0,968771759	1,03223488	3,875
USP13	0,062914349	-0,045775507	0,094	0,027	1	0,968768924	1,0322379	3,481481481
BEND5	0,80991663	-0,045794572	0,031	0,023	1	0,968756122	1,032251541	1,347826087
FAM204A	0,096306182	-0,04579477	0,312	0,16	1	0,968755989	1,032251683	1,95
SAP30L	0,437565433	-0,045867883	0,156	0,102	1	0,968706896	1,032303997	1,529411765
TSC22D1	0,0082127	-0,045947223	0,5	0,211	1	0,968653624	1,032360769	2,369668246
CLCN5	0,300902654	-0,045976749	0,062	0,027	1	0,9686338	1,032381897	2,296296296
MSRB2	0,028531331	-0,046143679	0,219	0,082	1	0,968521729	1,032501358	2,670731707
FAM234B	0,480650464	-0,046155667	0	0,016	1	0,968513681	1,032509938	0
SYN3	0,540537634	-0,046214727	0,031	0,016	1	0,968474034	1,032552206	1,9375
ELAVL1	0,00181928	-0,046217547	0,406	0,137	1	0,96847214	1,032554225	2,96350365
ZFP64	0,386325862	-0,046220494	0,031	0,012	1	0,968470162	1,032556334	2,583333333
UTP25	0,543117005	-0,046282332	0	0,012	1	0,968428652	1,032600593	0
CNBD2	0,003004599	-0,046310308	0,219	0,059	1	0,968409872	1,032620617	3,711864407
ACSS2	0,682170546	-0,046390142	0,031	0,02	1	0,968356285	1,03267776	1,55
NEU3	0,682170546	-0,046408983	0,031	0,02	1	0,968343639	1,032691247	1,55
KLHL8	0,668126075	-0,046438757	0,062	0,043	1	0,968323655	1,032712559	1,441860465
API5	0,005679953	-0,046489878	0,312	0,109	1	0,968289344	1,032749153	2,862385321
ITPKB	0,152699481	-0,046534787	0,125	0,055	1	0,968259202	1,032781303	2,272727273
ONECUT1	0,226351275	-0,046550306	0,031	0,008	1	0,968248787	1,032792412	3,875

Supplementary Table 1

Differentially expressed genes_adjacent-skin-XL_ comparing SC-Keloid vs. (SC-Myel, SC-Nonmyel)								
**gene**	**p_val**	**avg_log2FC**	**pct.1**	**pct.2**	**p_val_adj**	**Foldchange_UP**	**Foldchange_DOWN**	**Ratio_pct1_pct2**
STK24	0,112358923	-0,046612434	0,188	0,086	1	0,968207091	1,032836889	2,186046512
TATDN2	0,021379715	-0,046692265	0,156	0,047	1	0,968153518	1,032894042	3,319148936
DNA2	0,543117005	-0,046705054	0	0,012	1	0,968144935	1,032903198	0
PANX2	0,480650464	-0,046705387	0	0,016	1	0,968144712	1,032903437	0
FKBP1B	0,001642148	-0,04684075	0,125	0,02	1	0,968053878	1,033000355	6,25
CNEP1R1	0,304406192	-0,046866905	0,062	0,027	1	0,968036328	1,033019083	2,296296296
PSKH1	0,540537634	-0,046917804	0,031	0,016	1	0,968002176	1,033055529	1,9375
STAU2	0,000890115	-0,047001374	0,25	0,062	1	0,967946105	1,033115371	4,032258065
SLF2	0,000539834	-0,047018778	0,344	0,098	1	0,967934428	1,033127835	3,510204082
GNB4	0,011883017	-0,047100605	0,375	0,148	1	0,967879531	1,033186433	2,533783784
TRIM32	0,480650464	-0,047104851	0	0,016	1	0,967876682	1,033189474	0
FEZ2	0,009140921	-0,047120538	0,469	0,195	1	0,967866158	1,033200708	2,405128205
TMEM186	0,543117005	-0,047176341	0	0,012	1	0,967828722	1,033240673	0
LIN37	0,654515658	-0,04718386	0,062	0,043	1	0,967823678	1,033246058	1,441860465
NRL	0,543117005	-0,047216966	0	0,012	1	0,967801469	1,033269769	0
MYRIP	0,543117005	-0,047250022	0	0,012	1	0,967779294	1,033293444	0
TOMM34	0,063925678	-0,047261667	0,094	0,027	1	0,967771483	1,033301784	3,481481481
ZNF319	0,150155729	-0,047308979	0,062	0,02	1	0,967739746	1,033335671	3,1
RAP2C-AS1	0,150155729	-0,047374679	0,062	0,02	1	0,967695676	1,03338273	3,1
EPN2	0,014109694	-0,047390375	0,344	0,141	1	0,967685148	1,033393973	2,439716312
RAB3B	0,543117005	-0,047408355	0	0,012	1	0,967673088	1,033406852	0
HRCT1	0,480650464	-0,04741583	0	0,016	1	0,967668074	1,033412207	0
SBF2-AS1	0,543117005	-0,047559506	0	0,012	1	0,96757171	1,033515128	0
CCL21	0,480650464	-0,047756546	0	0,016	1	0,967439571	1,033656292	0
CRYBG1	0,386325862	-0,047799878	0,031	0,012	1	0,967410514	1,033687339	2,583333333
RANBP9	0,000327928	-0,047816813	0,344	0,094	1	0,967399158	1,033699474	3,659574468
RPP38	0,001055584	-0,047906139	0,188	0,039	1	0,967339262	1,033763478	4,820512821
GCA	0,007565208	-0,047926071	0,25	0,082	1	0,967325898	1,03377776	3,048780488
SPIN2A	0,543117005	-0,048053777	0	0,012	1	0,967240275	1,033869273	0
GLIS3	0,012493801	-0,048084339	0,438	0,191	1	0,967219785	1,033891175	2,293193717

Supplementary Table 1

Differentially expressed genes_adjacent-skin-XL_ comparing SC-Keloid vs. (SC-Myel, SC-Nonmyel)								
**gene**	**p_val**	**avg_log2FC**	**pct.1**	**pct.2**	**p_val_adj**	**Foldchange_UP**	**Foldchange_DOWN**	**Ratio_pct1_pct2**
MRRF	0,065070981	-0,048112798	0,125	0,043	1	0,967200705	1,03391157	2,906976744
FAM13A	0,285925963	-0,048135895	0,094	0,047	1	0,967185221	1,033928123	2
RIOX2	0,037506898	-0,048169572	0,094	0,023	1	0,967162644	1,033952258	4,086956522
TAPBPL	0,062914349	-0,048185285	0,094	0,027	1	0,967152111	1,033963519	3,481481481
BET1L	0,238745564	-0,048210833	0,094	0,043	1	0,967134984	1,03398183	2,186046512
SIN3B	0,083687879	-0,048227964	0,188	0,082	1	0,9671235	1,033994108	2,292682927
PTGIR	0,543117005	-0,04830998	0	0,012	1	0,967068521	1,034052891	0
REM1	0,682170546	-0,048385339	0,031	0,02	1	0,967018008	1,034106906	1,55
RAB11FIP4	0,480650464	-0,04848239	0	0,016	1	0,966952958	1,034176473	0
CTSS	0,543117005	-0,048490457	0	0,012	1	0,966947551	1,034182256	0
LRRC27	0,386325862	-0,048495444	0,031	0,012	1	0,966944209	1,034185831	2,583333333
DPT	0,543117005	-0,048499362	0	0,012	1	0,966941583	1,034188639	0
PRR14	7,07365E-05	-0,048616227	0,344	0,082	1	0,966863259	1,034272417	4,195121951
LRRN3	0,543117005	-0,048673147	0	0,012	1	0,966825113	1,034313224	0
METAP1D	0,010405093	-0,048698301	0,188	0,055	1	0,966808256	1,034331258	3,418181818
ATP8B2	0,191913299	-0,048736991	0,094	0,039	1	0,966782329	1,034358997	2,41025641
CCDC85C	0,543117005	-0,048835499	0	0,012	1	0,966716319	1,034429626	0
NFATC3	0,043915019	-0,048849156	0,125	0,039	1	0,966707168	1,034439418	3,205128205
RALBP1	0,001989868	-0,04895953	0,625	0,242	1	0,966633212	1,034518561	2,582644628
RAB6B	0,543117005	-0,049102461	0	0,012	1	0,96653745	1,034621058	0
SH3BP5-AS1	0,386325862	-0,049207004	0,031	0,012	1	0,966467414	1,034696034	2,583333333
DOC2B	0,543117005	-0,04924786	0	0,012	1	0,966440045	1,034725336	0
IFT57	0,014096701	-0,049284092	0,375	0,152	1	0,966415774	1,034751322	2,467105263
IGFLR1	0,480650464	-0,049337915	0	0,016	1	0,96637972	1,034789927	0
NPY1R	0,543117005	-0,049376108	0	0,012	1	0,966354138	1,034817321	0
ARMH3	0,918113127	-0,049384809	0,031	0,027	1	0,966348309	1,034823563	1,148148148
CAMTA2	0,224763613	-0,049413247	0,062	0,023	1	0,966329261	1,034843961	2,695652174
SLX4IP	0,540537634	-0,049429123	0,031	0,016	1	0,966318627	1,034855349	1,9375
PICK1	0,152699481	-0,049495771	0,125	0,055	1	0,966273988	1,034903157	2,272727273
AC132192.1	0,087959196	-0,049501538	0,062	0,016	1	0,966270125	1,034907294	3,875

Supplementary Table 1

Differentially expressed genes_adjacent-skin-XL_ comparing SC-Keloid vs. (SC-Myel, SC-Nonmyel)								
**gene**	**p_val**	**avg_log2FC**	**pct.1**	**pct.2**	**p_val_adj**	**Foldchange_UP**	**Foldchange_DOWN**	**Ratio_pct1_pct2**
NLGN1	0,540537634	-0,049524927	0,031	0,016	1	0,96625446	1,034924072	1,9375
RSBN1L	0,018003408	-0,049555868	0,406	0,176	1	0,966233737	1,034946268	2,306818182
PCDHB11	0,540537634	-0,049557265	0,031	0,016	1	0,966232801	1,03494727	1,9375
IDH3A	0,002020461	-0,049576342	0,281	0,082	1	0,966220025	1,034960956	3,426829268
BAP1	0,178474769	-0,049608755	0,188	0,098	1	0,966198317	1,034984208	1,918367347
PEX7	0,191913299	-0,049738492	0,094	0,039	1	0,966111434	1,035077285	2,41025641
LRIG3	0,386325862	-0,04975345	0,031	0,012	1	0,966101417	1,035088017	2,583333333
MFN2	0,016858455	-0,049794998	0,125	0,031	1	0,966073595	1,035117827	4,032258065
MGAT4A	0,543117005	-0,049823593	0	0,012	1	0,966054447	1,035138343	0
TMEM68	0,004186485	-0,049876255	0,156	0,035	1	0,966019184	1,03517613	4,457142857
IRF3	3,3603E-05	-0,049964935	0,375	0,09	0,709056871	0,965959806	1,035239762	4,166666667
ANKLE2	0,000130697	-0,049964965	0,469	0,141	1	0,965959786	1,035239783	3,326241135
UBE2V2	0,01375067	-0,049978631	0,406	0,168	1	0,965950636	1,03524959	2,416666667
ISOC1	0,065070981	-0,049980734	0,125	0,043	1	0,965949228	1,035251099	2,906976744
FAM178B	0,803381388	-0,050090044	0,031	0,023	1	0,965876043	1,035329541	1,347826087
RPUSD4	0,191913299	-0,050172635	0,094	0,039	1	0,96582075	1,035388813	2,41025641
LRRC8D	0,300902654	-0,050236239	0,062	0,027	1	0,965778171	1,035434461	2,296296296
LGALS9	0,543117005	-0,050330397	0	0,012	1	0,965715142	1,035502041	0
NOP14	0,098751938	-0,050337285	0,094	0,031	1	0,965710531	1,035506985	3,032258065
FGF4	0,543117005	-0,050405304	0	0,012	1	0,965665002	1,035555807	0
ARHGEF2	0,013138017	-0,050621015	0,406	0,168	1	0,965520627	1,035710654	2,416666667
NUP133	0,65904021	-0,05066278	0,062	0,043	1	0,965492676	1,035740638	1,441860465
APLP1	0,141661018	-0,050691034	0,094	0,035	1	0,965473768	1,035760922	2,685714286
ALDH4A1	0,087959196	-0,050700249	0,062	0,016	1	0,965467601	1,035767538	3,875
FOXD1	0,041848356	-0,050796522	0,062	0,012	1	0,965403176	1,035836658	5,166666667
SLC8A2	0,543117005	-0,050812694	0	0,012	1	0,965392354	1,03584827	0
GUF1	0,30793671	-0,050835087	0,062	0,027	1	0,96537737	1,035864348	2,296296296
PAPLN	0,041848356	-0,050859144	0,062	0,012	1	0,965361272	1,035881621	5,166666667
PAF1	0,049390616	-0,050997124	0,25	0,109	1	0,965268949	1,035980698	2,293577982
LRRC49	0,002263143	-0,051116627	0,062	0,004	1	0,965188996	1,036066516	15,5

Supplementary Table 1

Differentially expressed genes_adjacent-skin-XL_ comparing SC-Keloid vs. (SC-Myel, SC-Nonmyel)								
**gene**	**p_val**	**avg_log2FC**	**pct.1**	**pct.2**	**p_val_adj**	**Foldchange_UP**	**Foldchange_DOWN**	**Ratio_pct1_pct2**
GRM5-AS1	0,543117005	-0,051159411	0	0,012	1	0,965160373	1,036097241	0
PICALM	9,01646E-05	-0,051179577	0,469	0,133	1	0,965146882	1,036111724	3,526315789
ZNF266	0,037506898	-0,051204759	0,094	0,023	1	0,965130036	1,036129809	4,086956522
F2RL1	0,543117005	-0,05120582	0	0,012	1	0,965129326	1,036130571	0
ITGB7	0,543117005	-0,051213565	0	0,012	1	0,965124145	1,036136134	0
KIAA1586	0,682170546	-0,0513225	0,031	0,02	1	0,965051273	1,036214373	1,55
LINC02875	0,543117005	-0,051324605	0	0,012	1	0,965049865	1,036215885	0
CYB561	0,087959196	-0,051353454	0,062	0,016	1	0,965030567	1,036236606	3,875
PWWP2A	0,002873288	-0,051357617	0,344	0,117	1	0,965027783	1,036239596	2,94017094
HSDL1	0,540537634	-0,051371908	0,031	0,016	1	0,965018224	1,036249861	1,9375
CCL20	0,013891036	-0,051383083	0,062	0,008	1	0,965010749	1,036257888	7,75
DAPL1	0,80991663	-0,051388467	0,031	0,023	1	0,965007147	1,036261755	1,347826087
ERLIN1	0,185597364	-0,051404407	0,094	0,039	1	0,964996485	1,036273204	2,41025641
ERMP1	0,386325862	-0,051477789	0,031	0,012	1	0,964947403	1,036325915	2,583333333
AGPAT4	0,236345884	-0,051501694	0,094	0,043	1	0,964931414	1,036343087	2,186046512
LRRC37A3	0,543117005	-0,051546035	0	0,012	1	0,964901757	1,036374939	0
CAPZA2	0,018429421	-0,05156467	0,562	0,258	1	0,964889294	1,036388326	2,178294574
MTA3	0,001753489	-0,051570896	0,219	0,055	1	0,96488513	1,036392798	3,981818182
ZNF667	0,480650464	-0,051578258	0	0,016	1	0,964880206	1,036398087	0
TMEM70	0,000229745	-0,051624028	0,312	0,078	1	0,964849595	1,036430968	4
MITD1	0,000831993	-0,051653146	0,25	0,062	1	0,964830122	1,036451886	4,032258065
FAH	0,386325862	-0,051662486	0,031	0,012	1	0,964823875	1,036458597	2,583333333
RNF167	0,001091363	-0,051668285	0,281	0,078	1	0,964819997	1,036462763	3,602564103
IRAK1BP1	0,304406192	-0,05167652	0,062	0,027	1	0,96481449	1,036468679	2,296296296
ERMAP	0,150155729	-0,051723736	0,062	0,02	1	0,964782915	1,0365026	3,1
SLC35G2	0,682170546	-0,051734504	0,031	0,02	1	0,964775714	1,036510337	1,55
ADAM19	0,000503273	-0,051750428	0,406	0,125	1	0,964765065	1,036521777	3,248
MATN1	0,543117005	-0,051859649	0	0,012	1	0,964692029	1,036600251	0
ZNF607	0,428872689	-0,051873282	0	0,02	1	0,964682913	1,036610047	0
HIRA	0,224763613	-0,051904717	0,062	0,023	1	0,964661894	1,036632634	2,695652174

Supplementary Table 1

Differentially expressed genes_adjacent-skin-XL_ comparing SC-Keloid vs. (SC-Myel, SC-Nonmyel)								
**gene**	**p_val**	**avg_log2FC**	**pct.1**	**pct.2**	**p_val_adj**	**Foldchange_UP**	**Foldchange_DOWN**	**Ratio_pct1_pct2**
JAZF1	0,000103236	-0,051957569	0,156	0,02	1	0,964626555	1,036670611	7,8
MRPL14	0,003593442	-0,051964312	0,438	0,164	1	0,964622046	1,036675457	2,670731707
ZNF512B	0,682170546	-0,051966365	0,031	0,02	1	0,964620674	1,036676931	1,55
PPP1R14A	0,543117005	-0,051986436	0	0,012	1	0,964607253	1,036691354	0
LURAP1	0,478627389	-0,052018377	0,062	0,035	1	0,964585897	1,036714307	1,771428571
ACSL6	0,543117005	-0,052020841	0	0,012	1	0,96458425	1,036716077	0
TMEM135	0,138170297	-0,052136131	0,094	0,035	1	0,96450717	1,036798928	2,685714286
ACBD4	0,002594281	-0,052154936	0,094	0,012	1	0,964494599	1,036812442	7,833333333
TMEM231	0,30793671	-0,052192377	0,062	0,027	1	0,964469568	1,03683935	2,296296296
C2CD4B	0,543117005	-0,052261891	0	0,012	1	0,964423098	1,03688931	0
MORC2-AS1	0,386325862	-0,052279743	0,031	0,012	1	0,964411164	1,03690214	2,583333333
AC147651.1	0,480650464	-0,052291463	0	0,016	1	0,964403329	1,036910564	0
MORN1	0,543117005	-0,052357889	0	0,012	1	0,964358927	1,036958307	0
CYP20A1	0,27733921	-0,052400047	0,125	0,066	1	0,964330747	1,036988609	1,893939394
CAPRIN1	0,000170904	-0,052430139	0,531	0,168	1	0,964310633	1,037010239	3,160714286
TATDN3	0,543117005	-0,05246113	0	0,012	1	0,964289918	1,037032516	0
TMEM92	0,543117005	-0,052513214	0	0,012	1	0,964255106	1,037069955	0
WWC2-AS2	0,480650464	-0,052534043	0	0,016	1	0,964241185	1,037084928	0
RAB36	0,543117005	-0,052546035	0	0,012	1	0,96423317	1,037093549	0
CALHM5	0,041848356	-0,052569909	0,062	0,012	1	0,964217214	1,03711071	5,166666667
EGLN1	0,062247505	-0,052662701	0,25	0,113	1	0,964155199	1,037177418	2,212389381
SMYD4	0,224763613	-0,052663116	0,062	0,023	1	0,964154921	1,037177717	2,695652174
FHIT	0,480650464	-0,052683377	0	0,016	1	0,964141381	1,037192283	0
ZNF26	0,087959196	-0,052685248	0,062	0,016	1	0,964140131	1,037193628	3,875
SHPK	0,80991663	-0,052786801	0,031	0,023	1	0,964072266	1,03726664	1,347826087
MROH1	0,540537634	-0,052819532	0,031	0,016	1	0,964050394	1,037290173	1,9375
PEG3	0,682170546	-0,05282753	0,031	0,02	1	0,96404505	1,037295923	1,55
C2orf42	0,087959196	-0,052852698	0,062	0,016	1	0,964028232	1,03731402	3,875
MYCL	0,480650464	-0,052852703	0	0,016	1	0,964028228	1,037314023	0
CGGBP1	0,001121182	-0,052857904	0,625	0,238	1	0,964024753	1,037317763	2,62605042

Supplementary Table 1

Differentially expressed genes_adjacent-skin-XL_ comparing SC-Keloid vs. (SC-Myel, SC-Nonmyel)								
**gene**	**p_val**	**avg_log2FC**	**pct.1**	**pct.2**	**p_val_adj**	**Foldchange_UP**	**Foldchange_DOWN**	**Ratio_pct1_pct2**
CCDC144A	0,918113127	-0,052862854	0,031	0,027	1	0,964021446	1,037321322	1,148148148
HBS1L	0,000278482	-0,052945593	0,25	0,055	1	0,96396616	1,037380814	4,545454545
ALAS1	0,037506898	-0,052953527	0,094	0,023	1	0,963960859	1,037386519	4,086956522
EPS15L1	0,001019952	-0,052978333	0,219	0,051	1	0,963944285	1,037404356	4,294117647
ZNF177	0,543117005	-0,053036353	0	0,012	1	0,963905519	1,037446078	0
TGDS	0,000911755	-0,053077435	0,156	0,027	1	0,963878071	1,037475621	5,777777778
NOM1	0,04261236	-0,053083387	0,156	0,055	1	0,963874095	1,037479901	2,836363636
TGOLN2	0,015503587	-0,053172246	0,625	0,277	1	0,963814729	1,037543804	2,25631769
DHX57	0,019236133	-0,053197795	0,094	0,02	1	0,963797661	1,037562178	4,7
TTN-AS1	0,063925678	-0,053198969	0,094	0,027	1	0,963796877	1,037563022	3,481481481
ERCC4	0,000486986	-0,053199138	0,188	0,035	1	0,963796764	1,037563144	5,371428571
MMUT	0,005043469	-0,053214347	0,219	0,062	1	0,963786603	1,037574082	3,532258065
LIMD1	0,008148126	-0,05326443	0,094	0,016	1	0,963753147	1,037610101	5,875
LRFN5	0,480650464	-0,053280355	0	0,016	1	0,963742508	1,037621555	0
DRC3	0,543117005	-0,053326275	0	0,012	1	0,963711833	1,037654583	0
PLGLB1	0,543117005	-0,053351037	0	0,012	1	0,963695293	1,037672393	0
DAGLA	0,682170546	-0,05335151	0,031	0,02	1	0,963694977	1,037672733	1,55
PGAM5	0,224763613	-0,053400402	0,062	0,023	1	0,963662318	1,0377079	2,695652174
KIN	0,001845033	-0,053457403	0,219	0,055	1	0,963624244	1,0377489	3,981818182
AC104532.2	0,540537634	-0,053484613	0,031	0,016	1	0,96360607	1,037768473	1,9375
KATNIP	0,183526587	-0,0535575	0,094	0,039	1	0,963557389	1,037820903	2,41025641
CEP104	0,141661018	-0,053573287	0,094	0,035	1	0,963546845	1,037832261	2,685714286
BCAS2	0,076019609	-0,053575024	0,25	0,117	1	0,963545684	1,03783351	2,136752137
CRTAP	0,000604154	-0,053608039	0,438	0,141	1	0,963523635	1,03785726	3,106382979
GPKOW	0,138170297	-0,05369056	0,094	0,035	1	0,963468524	1,037916627	2,685714286
TIMM23	0,016858455	-0,053787522	0,125	0,031	1	0,963403772	1,037986386	4,032258065
HOMEZ	0,386325862	-0,053795784	0,031	0,012	1	0,963398255	1,03799233	2,583333333
CEP162	0,063925678	-0,053816602	0,094	0,027	1	0,963384354	1,038007309	3,481481481
SNTB1	0,922888178	-0,053895507	0,062	0,066	1	0,963331665	1,038064082	0,939393939
LONRF2	0,543117005	-0,054053078	0	0,012	1	0,963226455	1,038177466	0

Supplementary Table 1

Differentially expressed genes_adjacent-skin-XL_ comparing SC-Keloid vs. (SC-Myel, SC-Nonmyel)								
**gene**	**p_val**	**avg_log2FC**	**pct.1**	**pct.2**	**p_val_adj**	**Foldchange_UP**	**Foldchange_DOWN**	**Ratio_pct1_pct2**
ZNF500	0,226351275	-0,054103625	0,031	0,008	1	0,963192708	1,03821384	3,875
PRPSAP2	0,013518555	-0,054117563	0,188	0,059	1	0,963183403	1,03822387	3,186440678
JADE2	0,924392325	-0,054129756	0,031	0,027	1	0,963175262	1,038232645	1,148148148
TMEM204	0,480650464	-0,054132737	0	0,016	1	0,963173272	1,03823479	0
PCDHB6	0,543117005	-0,054152118	0	0,012	1	0,963160333	1,038248738	0
MDFIC	5,82732E-06	-0,054205229	0,281	0,047	0,12296218	0,963124876	1,03828696	5,978723404
EML2	0,015105004	-0,054230081	0,188	0,059	1	0,963108285	1,038304846	3,186440678
TWIST2	0,540537634	-0,054431218	0,031	0,016	1	0,96297402	1,038449614	1,9375
SPINK5	0,682170546	-0,054469145	0,031	0,02	1	0,962948705	1,038476914	1,55
PFN4	0,386325862	-0,054469917	0,031	0,012	1	0,96294819	1,03847747	2,583333333
CCNK	0,976455087	-0,05455801	0,062	0,062	1	0,962889393	1,038540883	1
RCHY1	0,141661018	-0,054585358	0,094	0,035	1	0,96287114	1,03856057	2,685714286
SLC7A1	0,470250215	-0,054614093	0,062	0,035	1	0,962851962	1,038581256	1,771428571
CLDN12	0,008148126	-0,054684433	0,094	0,016	1	0,962805019	1,038631894	5,875
AVL9	0,682170546	-0,054712771	0,031	0,02	1	0,962786107	1,038652296	1,55
KLHL42	0,004542982	-0,054754444	0,219	0,062	1	0,962758297	1,038682297	3,532258065
ZSWIM8-AS1	0,543117005	-0,054802909	0	0,012	1	0,962725955	1,038717191	0
BTRC	0,097375089	-0,054853931	0,094	0,031	1	0,962691908	1,038753927	3,032258065
FHIP1A	0,543117005	-0,05489875	0	0,012	1	0,962662001	1,038786198	0
CDC25B	0,002053304	-0,054964677	0,156	0,031	1	0,962618012	1,038833668	5,032258065
CHAC2	0,226351275	-0,054974447	0,031	0,008	1	0,962611493	1,038840703	3,875
KDM4A-AS1	0,543117005	-0,054994628	0	0,012	1	0,962598028	1,038855235	0
HABP4	0,027355643	-0,055008637	0,312	0,133	1	0,962588681	1,038865322	2,345864662
GNAL	0,924392442	-0,055034093	0,031	0,027	1	0,962571696	1,038883654	1,148148148
HLA-DQB1	0,540537634	-0,055055287	0,031	0,016	1	0,962557555	1,038898915	1,9375
LIPC	0,480650464	-0,055109591	0	0,016	1	0,962521325	1,038938021	0
ZBED6	0,480650464	-0,055163286	0	0,016	1	0,962485502	1,038976689	0
UBC	0,056469408	-0,055216511	1	0,91	1	0,962449993	1,039015021	1,098901099
PPFIA3	0,543117005	-0,055260005	0	0,012	1	0,962420979	1,039046345	0
APC	0,02529276	-0,055304035	0,25	0,098	1	0,962391606	1,039078057	2,551020408

Supplementary Table 1

Differentially expressed genes_adjacent-skin-XL_ comparing SC-Keloid vs. (SC-Myel, SC-Nonmyel)								
**gene**	**p_val**	**avg_log2FC**	**pct.1**	**pct.2**	**p_val_adj**	**Foldchange_UP**	**Foldchange_DOWN**	**Ratio_pct1_pct2**
WDR36	0,191913299	-0,05534114	0,094	0,039	1	0,962366855	1,039104781	2,41025641
NAV2-AS3	0,480650464	-0,055345955	0	0,016	1	0,962363643	1,039108249	0
TMCC2	0,543117005	-0,055351286	0	0,012	1	0,962360087	1,039112089	0
CYB5R1	0,097228337	-0,055390695	0,188	0,082	1	0,962333799	1,039140474	2,292682927
LINC00900	0,082317662	-0,055439313	0,031	0,004	1	0,96230137	1,039175493	7,75
FAM72D	0,226351275	-0,055454304	0,031	0,008	1	0,96229137	1,039186291	3,875
BMI1	0,003103243	-0,055465714	0,406	0,145	1	0,96228376	1,039194509	2,8
PPP1R8	0,004180266	-0,055542547	0,125	0,023	1	0,962232513	1,039249855	5,434782609
SDK2	0,80991663	-0,055568853	0,031	0,023	1	0,962214968	1,039268805	1,347826087
NIFK-AS1	0,224763613	-0,055584998	0,062	0,023	1	0,9622042	1,039280435	2,695652174
TLK1	1,04156E-05	-0,055598456	0,344	0,07	0,219780613	0,962195224	1,03929013	4,914285714
FIZ1	0,543117005	-0,055608323	0	0,012	1	0,962188644	1,039297238	0
GLI4	0,098751938	-0,055638299	0,094	0,031	1	0,962168652	1,039318833	3,032258065
VXN	0,543117005	-0,055644863	0	0,012	1	0,962164274	1,039323562	0
DNAJC25	0,063925678	-0,055667485	0,094	0,027	1	0,962149187	1,039339859	3,481481481
RAVER1	0,241162386	-0,055678589	0,094	0,043	1	0,962141781	1,039347858	2,186046512
FERMT3	0,30793671	-0,055691274	0,062	0,027	1	0,962133322	1,039356997	2,296296296
CCDC183	0,543117005	-0,05569596	0	0,012	1	0,962130197	1,039360373	0
TMEM183A	0,037342574	-0,055769515	0,281	0,121	1	0,962081145	1,039413365	2,32231405
FAM131A	0,573780511	-0,055795336	0,062	0,039	1	0,962063926	1,039431968	1,58974359
HPDL	0,480650464	-0,055815134	0	0,016	1	0,962050724	1,039446232	0
RTN4IP1	0,480650464	-0,055844844	0	0,016	1	0,962030912	1,039467639	0
MORC2	0,016858455	-0,055855667	0,125	0,031	1	0,962023695	1,039475437	4,032258065
ENTHD1	0,543117005	-0,055863109	0	0,012	1	0,962018732	1,039480799	0
MCF2L2	0,543117005	-0,055899918	0	0,012	1	0,961994187	1,03950732	0
TNFAIP8L1	0,428872689	-0,055907815	0	0,02	1	0,961988921	1,039513011	0
ITGA2	0,000492493	-0,055911723	0,312	0,086	1	0,961986316	1,039515826	3,627906977
EEF1AKMT2	0,006502031	-0,05595475	0,281	0,094	1	0,961957626	1,039546829	2,989361702
PCGF6	0,063925678	-0,055969627	0,094	0,027	1	0,961947706	1,039557549	3,481481481
UNC119	0,918113127	-0,056025795	0,031	0,027	1	0,961910256	1,039598023	1,148148148

Supplementary Table 1

Differentially expressed genes_adjacent-skin-XL_ comparing SC-Keloid vs. (SC-Myel, SC-Nonmyel)								
**gene**	**p_val**	**avg_log2FC**	**pct.1**	**pct.2**	**p_val_adj**	**Foldchange_UP**	**Foldchange_DOWN**	**Ratio_pct1_pct2**
NUAK1	0,386325862	-0,056117559	0,031	0,012	1	0,961849074	1,03966415	2,583333333
PEX5	0,183526162	-0,056159053	0,094	0,039	1	0,961821411	1,039694052	2,41025641
ZNF512	0,028712073	-0,056160837	0,125	0,035	1	0,961820221	1,039695338	3,571428571
TESK1	0,012987709	-0,056180154	0,156	0,043	1	0,961807343	1,039709259	3,627906977
FBXL2	0,000911755	-0,056196205	0,156	0,027	1	0,961796642	1,039720827	5,777777778
DTX3L	0,123421204	-0,056318107	0,125	0,051	1	0,961715378	1,039808683	2,450980392
DUSP15	0,013407295	-0,056415883	0,156	0,043	1	0,961650201	1,039879156	3,627906977
INPP5K	0,002042841	-0,056485727	0,375	0,125	1	0,961603647	1,0399295	3
FYCO1	0,028712073	-0,056485957	0,125	0,035	1	0,961603494	1,039929666	3,571428571
YTHDF3-DT	0,30793671	-0,056511191	0,062	0,027	1	0,961586675	1,039947855	2,296296296
TRIM73	0,150155729	-0,056515333	0,062	0,02	1	0,961583914	1,039950841	3,1
FOLH1	0,428872689	-0,056516024	0	0,02	1	0,961583454	1,039951339	0
TMEM86B	0,543117005	-0,05652467	0	0,012	1	0,961577691	1,039957571	0
ISCA2	0,015740914	-0,056526656	0,188	0,059	1	0,961576367	1,039959003	3,186440678
SFXN2	0,543117005	-0,056546469	0	0,012	1	0,961563162	1,039973285	0
HUS1	0,00208125	-0,056553426	0,188	0,043	1	0,961558525	1,0399783	4,372093023
ZSWIM9	0,682170546	-0,056580203	0,031	0,02	1	0,961540678	1,039997603	1,55
ATP6V1H	0,001195376	-0,056626434	0,281	0,078	1	0,961509866	1,04003093	3,602564103
SLFN13	0,150155729	-0,056685147	0,062	0,02	1	0,961470737	1,040073257	3,1
VHL	0,043149764	-0,056777745	0,156	0,055	1	0,961409027	1,040140015	2,836363636
BCAS4	0,013891036	-0,056825199	0,062	0,008	1	0,961377404	1,040174229	7,75
RILPL2	0,396289917	-0,056844351	0,062	0,031	1	0,961364642	1,040188037	2
KIF3C	0,482846351	-0,056868211	0,062	0,035	1	0,961348743	1,04020524	1,771428571
ZNF250	0,543117005	-0,056904569	0	0,012	1	0,961324516	1,040231455	0
PCBD2	0,082317662	-0,056914791	0,031	0,004	1	0,961317704	1,040238826	7,75
MAP4K1	0,480650464	-0,056915024	0	0,016	1	0,961317549	1,040238993	0
MSTO1	0,091552625	-0,056978653	0,125	0,047	1	0,961275152	1,040284874	2,659574468
TRIP13	0,082317662	-0,057145942	0,031	0,004	1	0,961163693	1,040405508	7,75
ZNF503-AS1	0,386325862	-0,057188332	0,031	0,012	1	0,961135452	1,040436078	2,583333333
DGKA	0,392349482	-0,057193014	0,062	0,031	1	0,961132332	1,040439455	2

Supplementary Table 1

Differentially expressed genes_adjacent-skin-XL_ comparing SC-Keloid vs. (SC-Myel, SC-Nonmyel)								
**gene**	**p_val**	**avg_log2FC**	**pct.1**	**pct.2**	**p_val_adj**	**Foldchange_UP**	**Foldchange_DOWN**	**Ratio_pct1_pct2**
ERN1	0,060102361	-0,057240417	0,156	0,059	1	0,961100753	1,040473641	2,644067797
SLC36A2	0,480650464	-0,057252716	0	0,016	1	0,96109256	1,040482511	0
RAB7A	0,057320859	-0,057338739	0,75	0,398	1	0,961035255	1,040544554	1,884422111
LANCL1	0,185597364	-0,057369985	0,094	0,039	1	0,961014441	1,04056709	2,41025641
PARP6	0,000511287	-0,057423294	0,25	0,059	1	0,960978931	1,040605541	4,237288136
TSTD3	0,098751938	-0,057445568	0,094	0,031	1	0,960964095	1,040621606	3,032258065
CFB	0,480650464	-0,057511273	0	0,016	1	0,96092033	1,040669001	0
ADD1	0,007483769	-0,057574855	0,656	0,281	1	0,960877982	1,040714866	2,334519573
APBB1IP	0,543117005	-0,057632437	0	0,012	1	0,960839631	1,040756405	0
ZNF571	0,540537634	-0,057648098	0,031	0,016	1	0,960829201	1,040767703	1,9375
SMARCA2	0,000266603	-0,057685344	0,281	0,066	1	0,960804396	1,040794572	4,257575758
ABITRAM	0,304406192	-0,057741148	0,062	0,027	1	0,960767232	1,040834831	2,296296296
LSR	0,30793671	-0,057755336	0,062	0,027	1	0,960757783	1,040845068	2,296296296
RNPEP	0,005379813	-0,057811759	0,25	0,078	1	0,960720209	1,040885775	3,205128205
GRIK5	0,480650464	-0,057844614	0	0,016	1	0,960698331	1,04090948	0
NARS2	0,30793671	-0,057855445	0,062	0,027	1	0,960691119	1,040917295	2,296296296
BAG1	0,038195773	-0,057912851	0,375	0,176	1	0,960652893	1,040958714	2,130681818
PTCH2	0,543117005	-0,057927666	0	0,012	1	0,960643027	1,040969404	0
TCEAL3	0,105996737	-0,057977576	0,344	0,18	1	0,960609794	1,041005417	1,911111111
RNF111	0,009808643	-0,058045215	0,188	0,055	1	0,960564759	1,041054224	3,418181818
ZNF518B	0,480650464	-0,058047957	0	0,016	1	0,960562933	1,041056203	0
SLC27A3	0,386325862	-0,058105101	0,031	0,012	1	0,960524887	1,041097439	2,583333333
ZNF407-AS1	0,224763613	-0,05820783	0,062	0,023	1	0,960456494	1,041171575	2,695652174
HVCN1	0,543117005	-0,058271646	0	0,012	1	0,96041401	1,041217631	0
ZNF490	0,815054348	-0,058278305	0,031	0,039	1	0,960409577	1,041222437	0,794871795
MGAT1	0,009644385	-0,058291039	0,344	0,133	1	0,9604011	1,041231627	2,586466165
MRPL53	0,037506898	-0,058298957	0,094	0,023	1	0,960395829	1,041237342	4,086956522
ARHGEF19	0,682170546	-0,05831565	0,031	0,02	1	0,960384716	1,04124939	1,55
ZNF184	0,087959196	-0,058352289	0,062	0,016	1	0,960360326	1,041275834	3,875
NPIPA5	0,480650464	-0,058400732	0	0,016	1	0,96032808	1,041310798	0

Supplementary Table 1

Differentially expressed genes_adjacent-skin-XL_ comparing SC-Keloid vs. (SC-Myel, SC-Nonmyel)								
**gene**	**p_val**	**avg_log2FC**	**pct.1**	**pct.2**	**p_val_adj**	**Foldchange_UP**	**Foldchange_DOWN**	**Ratio_pct1_pct2**
CDKN2B	0,002594281	-0,058428798	0,094	0,012	1	0,960309398	1,041331056	7,833333333
ZGRF1	0,150155729	-0,058454455	0,062	0,02	1	0,96029232	1,041349575	3,1
TC2N	0,480650464	-0,058468976	0	0,016	1	0,960282655	1,041360057	0
ZNF436	0,543117005	-0,058476792	0	0,012	1	0,960277452	1,041365699	0
NFS1	0,06592651	-0,058490177	0,125	0,043	1	0,960268543	1,04137536	2,906976744
CNGA1	0,543117005	-0,058499816	0	0,012	1	0,960262127	1,041382318	0
TBC1D25	0,291239073	-0,058515572	0,094	0,047	1	0,96025164	1,041393691	2
LRRC4B	0,480650464	-0,058531954	0	0,016	1	0,960240736	1,041405517	0
HSPB8	0,543117005	-0,058567681	0	0,012	1	0,960216957	1,041431306	0
SUCO	0,003724448	-0,058571142	0,25	0,074	1	0,960214653	1,041433805	3,378378378
VPS37A	0,090467775	-0,058632211	0,125	0,047	1	0,960174009	1,04147789	2,659574468
MRPL11	0,005962766	-0,058632604	0,281	0,094	1	0,960173747	1,041478173	2,989361702
GHR	0,543117005	-0,058700759	0	0,012	1	0,960128388	1,041527375	0
CDKL1	0,924392442	-0,058717481	0,031	0,027	1	0,96011726	1,041539447	1,148148148
C2orf49	0,188089808	-0,058738812	0,156	0,078	1	0,960103064	1,041554847	2
SF3B4	0,008201485	-0,058783937	0,281	0,098	1	0,960073034	1,041587425	2,867346939
HAS3	0,543117005	-0,058832727	0	0,012	1	0,960040566	1,041622651	0
KCNJ10	0,543117005	-0,05886107	0	0,012	1	0,960021706	1,041643115	0
SLC26A6	0,224763613	-0,05891201	0,062	0,023	1	0,959987809	1,041679895	2,695652174
AC019117.2	0,803381388	-0,058982911	0,031	0,023	1	0,959940631	1,04173109	1,347826087
TLR1	0,480650464	-0,058985543	0	0,016	1	0,95993888	1,04173299	0
SLC4A2	0,002246141	-0,058998508	0,344	0,113	1	0,959930254	1,041742352	3,044247788
ARFIP2	0,020165275	-0,059008133	0,156	0,047	1	0,95992385	1,041749302	3,319148936
PIM1	0,139907281	-0,059017972	0,094	0,035	1	0,959917303	1,041756406	2,685714286
SLC33A1	0,307008662	-0,059047637	0,188	0,113	1	0,959897565	1,041777827	1,663716814
NOP2	0,480650464	-0,059058091	0	0,016	1	0,95989061	1,041785376	0
NME6	0,80991663	-0,05907129	0,031	0,023	1	0,959881828	1,041794907	1,347826087
SEMA7A	0,803381388	-0,059073631	0,031	0,023	1	0,95988027	1,041796598	1,347826087
CEP43	0,905570195	-0,059075826	0,031	0,027	1	0,95987881	1,041798183	1,148148148
GNL3L	0,080880928	-0,059122312	0,156	0,062	1	0,959847881	1,041831752	2,516129032

Supplementary Table 1

Differentially expressed genes_adjacent-skin-XL_ comparing SC-Keloid vs. (SC-Myel, SC-Nonmyel)								
**gene**	**p_val**	**avg_log2FC**	**pct.1**	**pct.2**	**p_val_adj**	**Foldchange_UP**	**Foldchange_DOWN**	**Ratio_pct1_pct2**
SLC39A9	0,150155729	-0,059311745	0,062	0,02	1	0,959721857	1,041968559	3,1
GLT8D2	0,543117005	-0,05936573	0	0,012	1	0,959685945	1,042007549	0
CRABP2	0,150155729	-0,059477954	0,062	0,02	1	0,959611296	1,042088608	3,1
NELFB	0,00014443	-0,059507226	0,312	0,074	1	0,959591826	1,042109752	4,216216216
AIFM3	0,543117005	-0,059508888	0	0,012	1	0,959590721	1,042110952	0
FRRS1L	0,543117005	-0,059557995	0	0,012	1	0,959558059	1,042146425	0
EHBP1L1	0,013783696	-0,059706408	0,25	0,09	1	0,959459352	1,042253638	2,777777778
HECTD4	0,361559695	-0,05973785	0,125	0,074	1	0,959438442	1,042276353	1,689189189
SPR	0,543117005	-0,059745067	0	0,012	1	0,959433642	1,042281567	0
DIABLO	0,480650464	-0,059808132	0	0,016	1	0,959391703	1,04232713	0
FSIP1	0,543117005	-0,059968013	0	0,012	1	0,959285388	1,042442648	0
PKNOX1	0,028712073	-0,06001155	0,125	0,035	1	0,959256439	1,042474107	3,571428571
TDG	0,038104316	-0,060018487	0,188	0,07	1	0,959251827	1,04247912	2,685714286
ZNF398	0,226351275	-0,060060865	0,031	0,008	1	0,95922365	1,042509742	3,875
GLMN	0,641019742	-0,060148712	0,062	0,043	1	0,959165244	1,042573223	1,441860465
ZNF845	0,543117005	-0,060154242	0	0,012	1	0,959161567	1,042577219	0
VPS50	0,008780679	-0,06017377	0,125	0,027	1	0,959148584	1,042591332	4,62962963
STAG3	0,543117005	-0,060224195	0	0,012	1	0,959115061	1,042627772	0
FOXO6	0,428872689	-0,060333371	0	0,02	1	0,959042483	1,042706677	0
APOBEC3D	0,428872689	-0,060333597	0	0,02	1	0,959042332	1,04270684	0
CD34	0,428872689	-0,060341976	0	0,02	1	0,959036763	1,042712896	0
CAP2	0,480650464	-0,060406907	0	0,016	1	0,9589936	1,042759826	0
PKIA	0,044796455	-0,060469861	0,156	0,055	1	0,958951754	1,04280533	2,836363636
ZNF678	0,224763613	-0,060471802	0,062	0,023	1	0,958950464	1,042806732	2,695652174
LINC01106	0,543117005	-0,060493189	0	0,012	1	0,958936248	1,042822192	0
WDR11	0,003791564	-0,06050717	0,188	0,047	1	0,958926956	1,042832297	4
PRMT7	0,569359326	-0,060516615	0,062	0,039	1	0,958920678	1,042839124	1,58974359
FLI1	0,543117005	-0,06054749	0	0,012	1	0,958900156	1,042861443	0
ZNF367	0,682170546	-0,060605402	0,031	0,02	1	0,958861665	1,042903305	1,55
EPOR	0,543117005	-0,060609931	0	0,012	1	0,958858655	1,042906579	0

Supplementary Table 1

Differentially expressed genes_adjacent-skin-XL_ comparing SC-Keloid vs. (SC-Myel, SC-Nonmyel)								
**gene**	**p_val**	**avg_log2FC**	**pct.1**	**pct.2**	**p_val_adj**	**Foldchange_UP**	**Foldchange_DOWN**	**Ratio_pct1_pct2**
OLFM2	0,682170546	-0,060635804	0,031	0,02	1	0,958841459	1,042925283	1,55
NUDT7	0,682170546	-0,060636435	0,031	0,02	1	0,95884104	1,042925739	1,55
RCOR1	0,06222129	-0,060669843	0,156	0,059	1	0,958818837	1,04294989	2,644067797
INPP5J	0,226351275	-0,060675525	0,031	0,008	1	0,95881506	1,042953997	3,875
CPSF6	0,004374246	-0,060678646	0,375	0,137	1	0,958812986	1,042956253	2,737226277
NUP210	0,480650464	-0,060694324	0	0,016	1	0,958802567	1,042967588	0
PYCR2	0,000246494	-0,060708458	0,406	0,117	1	0,958793173	1,042977806	3,47008547
CHKB-DT	0,682170546	-0,060721269	0,031	0,02	1	0,958784659	1,042987067	1,55
LBR	0,00187651	-0,060721651	0,219	0,055	1	0,958784405	1,042987344	3,981818182
TRAIP	0,087959196	-0,060734486	0,062	0,016	1	0,958775876	1,042996622	3,875
ZDHHC18	0,682170546	-0,060806812	0,031	0,02	1	0,958727811	1,043048912	1,55
CHM	0,045949521	-0,06098374	0,219	0,09	1	0,958610243	1,043176836	2,433333333
TIMMDC1	0,020837881	-0,061022582	0,406	0,176	1	0,958584434	1,043204923	2,306818182
PRRT2	0,028712073	-0,061041621	0,125	0,035	1	0,958571784	1,04321869	3,571428571
CD248	0,428872689	-0,0610608	0	0,02	1	0,958559041	1,043232558	0
ESRRG	0,543117005	-0,061113069	0	0,012	1	0,958524312	1,043270355	0
ZNF180	0,428872689	-0,061156329	0	0,02	1	0,958495571	1,043301639	0
ZNF467	0,543117005	-0,06118588	0	0,012	1	0,958475939	1,043323009	0
KPNA4	0,003395537	-0,061228586	0,438	0,16	1	0,958447566	1,043353894	2,7375
CYB5D1	0,540537634	-0,061284864	0,031	0,016	1	0,958410179	1,043394594	1,9375
RNF10	0,03126913	-0,061298354	0,406	0,184	1	0,958401217	1,043404351	2,206521739
RPA1	1,51672E-05	-0,061299968	0,281	0,051	0,32004324	0,958400145	1,043405518	5,509803922
ATPAF2	0,30793671	-0,061337282	0,062	0,027	1	0,958375358	1,043432505	2,296296296
BRD3	0,011655001	-0,061373034	0,375	0,148	1	0,958351608	1,043458363	2,533783784
MCPH1	0,299332476	-0,061384993	0,094	0,047	1	0,958343664	1,043467013	2
ALS2CL	0,543117005	-0,061440608	0	0,012	1	0,958306721	1,043507238	0
H2BC9	0,543117005	-0,061451861	0	0,012	1	0,958299246	1,043515378	0
DEDD	0,300902654	-0,061457487	0,062	0,027	1	0,958295509	1,043519447	2,296296296
MCOLN2	0,480650464	-0,061457897	0	0,016	1	0,958295237	1,043519743	0
SLAIN2	0,006238542	-0,061470674	0,375	0,141	1	0,95828675	1,043528985	2,659574468

Supplementary Table 1

Differentially expressed genes_adjacent-skin-XL_ comparing SC-Keloid vs. (SC-Myel, SC-Nonmyel)								
**gene**	**p_val**	**avg_log2FC**	**pct.1**	**pct.2**	**p_val_adj**	**Foldchange_UP**	**Foldchange_DOWN**	**Ratio_pct1_pct2**
LYPLA2	0,001896716	-0,061476578	0,188	0,043	1	0,958282829	1,043533256	4,372093023
AOC3	0,480650464	-0,06148889	0	0,016	1	0,95827465	1,043542162	0
KBTBD8	0,543117005	-0,061514962	0	0,012	1	0,958257333	1,04356102	0
FANCM	0,682170546	-0,06163423	0,031	0,02	1	0,958178116	1,043647296	1,55
RNF41	0,011149113	-0,061680605	0,219	0,07	1	0,958147317	1,043680844	3,128571429
GXYLT2	0,013196028	-0,061755999	0,156	0,043	1	0,958097246	1,043735387	3,627906977
TNFRSF21	0,543117005	-0,061772915	0	0,012	1	0,958086012	1,043747625	0
MTCH1	0,078203165	-0,061835411	0,656	0,352	1	0,95804451	1,04379284	1,863636364
SLC16A5	0,543117005	-0,061842588	0	0,012	1	0,958039744	1,043798033	0
BRPF1	0,097375089	-0,061845319	0,094	0,031	1	0,95803793	1,043800009	3,032258065
OXNAD1	0,543117005	-0,061857483	0	0,012	1	0,958029853	1,043808809	0
PNMA1	0,002052921	-0,061882529	0,469	0,172	1	0,958013221	1,043826931	2,726744186
PARVA	0,000869817	-0,061920896	0,5	0,176	1	0,957987744	1,04385469	2,840909091
KCNIP2	0,480650464	-0,061931718	0	0,016	1	0,957980558	1,043862521	0
SMCO3	0,540537634	-0,062047933	0,031	0,016	1	0,957903392	1,043946611	1,9375
ALAD	0,020165275	-0,062075165	0,156	0,047	1	0,957885311	1,043966317	3,319148936
ARSJ	0,741950345	-0,062102326	0,062	0,047	1	0,957867277	1,043985971	1,319148936
LRRC45	0,224763613	-0,062142015	0,062	0,023	1	0,957840927	1,044014692	2,695652174
RHOU	0,480650464	-0,06215008	0	0,016	1	0,957835572	1,044020529	0
MPC2	0,000223656	-0,06220179	0,5	0,156	1	0,957801241	1,04405795	3,205128205
LZTFL1	0,428872689	-0,062221724	0	0,02	1	0,957788007	1,044072376	0
PPP1R14C	0,480650464	-0,06228652	0	0,016	1	0,957744991	1,044119269	0
TMEM80	0,026654007	-0,062406809	0,25	0,098	1	0,957665139	1,04420633	2,551020408
KCNAB2	0,543117005	-0,062407139	0	0,012	1	0,95766492	1,044206569	0
KICS2	0,80991663	-0,062420694	0,031	0,023	1	0,957655922	1,04421638	1,347826087
ZNF140	0,682170546	-0,062479169	0,031	0,02	1	0,957617108	1,044258704	1,55
ZBTB47	0,18979066	-0,06252012	0,094	0,039	1	0,957589926	1,044288346	2,41025641
CEP128	0,540537634	-0,062560479	0,031	0,016	1	0,957563138	1,04431756	1,9375
PRPF8	0,000425789	-0,062580876	0,438	0,137	1	0,9575496	1,044332325	3,197080292
WDCP	0,543117005	-0,062600818	0	0,012	1	0,957536364	1,044346761	0

Supplementary Table 1

Differentially expressed genes_adjacent-skin-XL_ comparing SC-Keloid vs. (SC-Myel, SC-Nonmyel)								
**gene**	**p_val**	**avg_log2FC**	**pct.1**	**pct.2**	**p_val_adj**	**Foldchange_UP**	**Foldchange_DOWN**	**Ratio_pct1_pct2**
LMO7	0,275136254	-0,062684332	0,125	0,066	1	0,957480937	1,044407217	1,893939394
HOMER2	0,682170546	-0,062709449	0,031	0,02	1	0,957464267	1,0444254	1,55
CTNNBL1	0,007318479	-0,062824985	0,219	0,066	1	0,957387593	1,044509045	3,318181818
SRBD1	0,30757457	-0,062885089	0,094	0,047	1	0,957347708	1,04455256	2
POLR2F	0,054996801	-0,062901256	0,688	0,359	1	0,95733698	1,044564266	1,91643454
AJAP1	0,480650464	-0,062904389	0	0,016	1	0,957334901	1,044566534	0
IQSEC2	0,037506898	-0,062954001	0,094	0,023	1	0,957301981	1,044602456	4,086956522
CLASP1	0,000121681	-0,062975429	0,281	0,062	1	0,957287762	1,044617972	4,532258065
MTMR11	0,226351275	-0,063020166	0,031	0,008	1	0,957258077	1,044650365	3,875
ZFP28	0,428872689	-0,06303538	0	0,02	1	0,957247983	1,044661381	0
TRIM47	0,001391765	-0,063076521	0,344	0,105	1	0,957220686	1,044691172	3,276190476
SMIM1	0,480650464	-0,063129285	0	0,016	1	0,957185678	1,04472938	0
CDK3	0,480650464	-0,063140402	0	0,016	1	0,957178302	1,044737431	0
TRIM36	0,428872689	-0,063142964	0	0,02	1	0,957176602	1,044739286	0
ZSWIM4	0,815054348	-0,06322162	0,031	0,039	1	0,957124418	1,044796247	0,794871795
SLC35E2A	0,386325862	-0,063229722	0,031	0,012	1	0,957119043	1,044802115	2,583333333
CYP4F11	0,480650464	-0,063248577	0	0,016	1	0,957106534	1,04481577	0
NEURL4	0,480650464	-0,063368477	0	0,016	1	0,957026994	1,044902606	0
GABRQ	0,480650464	-0,063483165	0	0,016	1	0,956950917	1,044985675	0
TRIM16L	0,386325862	-0,063524634	0,031	0,012	1	0,956923411	1,045015712	2,583333333
WDR44	0,045196862	-0,06353117	0,125	0,039	1	0,956919076	1,045020447	3,205128205
METTL15	0,126128812	-0,06364912	0,125	0,051	1	0,956840845	1,045105887	2,450980392
S1PR2	0,191913299	-0,063652418	0,094	0,039	1	0,956838657	1,045108277	2,41025641
MBNL3	0,480650464	-0,063660533	0	0,016	1	0,956833275	1,045114155	0
UBE2H	0,015370253	-0,063676918	0,656	0,285	1	0,956822408	1,045126025	2,301754386
TRIM29	0,224763613	-0,063688257	0,062	0,023	1	0,956814888	1,045134239	2,695652174
PDHX	0,803381388	-0,063712066	0,031	0,023	1	0,956799098	1,045151487	1,347826087
WBP4	0,014231146	-0,063720494	0,406	0,172	1	0,956793508	1,045157593	2,360465116
SDCBP2-AS1	0,543117005	-0,063769136	0	0,012	1	0,956761249	1,045192832	0
RBPMS-AS1	0,543117005	-0,063790685	0	0,012	1	0,956746959	1,045208444	0

Supplementary Table 1

Differentially expressed genes_adjacent-skin-XL_ comparing SC-Keloid vs. (SC-Myel, SC-Nonmyel)								
**gene**	**p_val**	**avg_log2FC**	**pct.1**	**pct.2**	**p_val_adj**	**Foldchange_UP**	**Foldchange_DOWN**	**Ratio_pct1_pct2**
METTL25B	0,924392442	-0,063795425	0,031	0,027	1	0,956743816	1,045211878	1,148148148
GULP1	0,043054743	-0,063831944	0,625	0,312	1	0,956719597	1,045238336	2,003205128
TMCO4	0,30793671	-0,06383515	0,062	0,027	1	0,956717471	1,045240659	2,296296296
ANKRD18B	0,543117005	-0,063874763	0	0,012	1	0,956691203	1,045269359	0
BACH1	0,01094829	-0,063925676	0,656	0,316	1	0,956657441	1,045306247	2,075949367
SLC25A23	0,098751938	-0,063928615	0,094	0,031	1	0,956655493	1,045308377	3,032258065
MSX2	0,30793671	-0,064098787	0,062	0,027	1	0,956542658	1,045431682	2,296296296
PCTP	0,098751938	-0,064106563	0,094	0,031	1	0,956537502	1,045437317	3,032258065
TRIM17	0,480650464	-0,064128702	0	0,016	1	0,956522823	1,045453361	0
EMCN	0,543117005	-0,064132712	0	0,012	1	0,956520165	1,045456266	0
TMCC3	0,918113127	-0,064248195	0,031	0,027	1	0,956443602	1,045539955	1,148148148
SLC9A8	0,428872689	-0,064251441	0	0,02	1	0,95644145	1,045542307	0
USP19	0,898329073	-0,064268686	0,031	0,035	1	0,956430017	1,045554805	0,885714286
PAN3-AS1	0,543117005	-0,064279676	0	0,012	1	0,956422731	1,04556277	0
AQP8	0,543117005	-0,064355084	0	0,012	1	0,956372741	1,045617422	0
EPCAM	0,480650464	-0,064449739	0	0,016	1	0,956309996	1,045686026	0
ATAD3C	0,480650464	-0,064521136	0	0,016	1	0,956262671	1,045737777	0
CTSL	0,008090256	-0,064531528	0,5	0,207	1	0,956255783	1,04574531	2,415458937
CDPF1	0,480650464	-0,064531622	0	0,016	1	0,95625572	1,045745379	0
GPD1L	0,38481627	-0,064543738	0	0,023	1	0,95624769	1,045754161	0
TCEAL9	0,011189289	-0,064559034	0,75	0,371	1	0,956237551	1,045765248	2,021563342
PIGZ	0,480650464	-0,064574906	0	0,016	1	0,956227031	1,045776754	0
TMEM131L	0,682170546	-0,064628821	0,031	0,02	1	0,956191297	1,045815836	1,55
TECPR1	0,020764757	-0,064630207	0,156	0,047	1	0,956190378	1,04581684	3,319148936
DUSP2	0,985101745	-0,064650106	0,031	0,031	1	0,956177189	1,045831266	1
FAM102B	0,304406192	-0,064665486	0,062	0,027	1	0,956166996	1,045842415	2,296296296
ZMYND19	0,224763613	-0,064690974	0,062	0,023	1	0,956150103	1,045860892	2,695652174
DZANK1	0,543117005	-0,064736726	0	0,012	1	0,956119782	1,04589406	0
SEC16B	0,480650464	-0,064777508	0	0,016	1	0,956092755	1,045923625	0
GRB2	0,001068223	-0,065015005	0,469	0,16	1	0,955935375	1,04609582	2,93125

Supplementary Table 1

Differentially expressed genes_adjacent-skin-XL_ comparing SC-Keloid vs. (SC-Myel, SC-Nonmyel)								
**gene**	**p_val**	**avg_log2FC**	**pct.1**	**pct.2**	**p_val_adj**	**Foldchange_UP**	**Foldchange_DOWN**	**Ratio_pct1_pct2**
CAPNS2	0,543117005	-0,065036815	0	0,012	1	0,955920924	1,046111635	0
RFXAP	0,063925678	-0,065037166	0,094	0,027	1	0,955920692	1,046111889	3,481481481
GK	0,020764757	-0,065044232	0,156	0,047	1	0,95591601	1,046117012	3,319148936
PLD1	0,480650464	-0,065085564	0	0,016	1	0,955888624	1,046146983	0
MGA	0,007871757	-0,065092976	0,156	0,039	1	0,955883713	1,046152358	4
TTC7B	0,097375089	-0,065107657	0,094	0,031	1	0,955873985	1,046163004	3,032258065
HEPH	0,428970186	-0,065232661	0,094	0,055	1	0,955791166	1,046253654	1,709090909
THOC5	0,016858455	-0,06523456	0,125	0,031	1	0,955789908	1,046255031	4,032258065
NCAPD2	0,682170546	-0,065259784	0,031	0,02	1	0,955773197	1,046273324	1,55
APOBEC3G	1,66267E-05	-0,065298491	0,344	0,074	0,35084016	0,955747554	1,046301396	4,648648649
PXMP2	0,001637922	-0,065302117	0,219	0,055	1	0,955745153	1,046304025	3,981818182
ZBED5-AS1	0,480650464	-0,065418431	0	0,016	1	0,955668101	1,046388384	0
BTBD9	0,924392442	-0,065502444	0,031	0,027	1	0,955612451	1,04644932	1,148148148
THBS3	0,030577237	-0,065508775	0,156	0,051	1	0,955608257	1,046453913	3,058823529
ANAPC13	0,020683123	-0,065595489	0,281	0,109	1	0,955550821	1,046516813	2,577981651
GTPBP3	0,004180266	-0,065600809	0,125	0,023	1	0,955547298	1,046520671	5,434782609
CHTF18	0,008956837	-0,065619683	0,125	0,027	1	0,955534797	1,046534362	4,62962963
TNFSF10	0,540537634	-0,065622376	0,031	0,016	1	0,955533013	1,046536317	1,9375
ZNF696	0,543117005	-0,065725352	0	0,012	1	0,955464813	1,046611018	0
TCFL5	0,008956837	-0,065747368	0,125	0,027	1	0,955450232	1,04662699	4,62962963
SLC43A1	0,543117005	-0,065759341	0	0,012	1	0,955442303	1,046635676	0
TMEM33	0,002391142	-0,065777973	0,312	0,098	1	0,955429963	1,046649193	3,183673469
DYNLT2	0,540537634	-0,065803702	0,031	0,016	1	0,955412924	1,046667859	1,9375
TAOK3	0,109417123	-0,065837403	0,188	0,086	1	0,955390606	1,04669231	2,186046512
EGFR	0,480650464	-0,065902967	0	0,016	1	0,955347189	1,046739878	0
PEAR1	0,543117005	-0,065987731	0	0,012	1	0,95529106	1,04680138	0
LMOD3	0,543117005	-0,066013761	0	0,012	1	0,955273824	1,046820267	0
UEVLD	0,80991663	-0,066016284	0,031	0,023	1	0,955272154	1,046822098	1,347826087
SEC24C	0,236932254	-0,066045876	0,125	0,062	1	0,95525256	1,04684357	2,016129032
SPIN2B	0,388432899	-0,066065875	0,062	0,031	1	0,955239318	1,046858082	2

Supplementary Table 1

Differentially expressed genes_adjacent-skin-XL_ comparing SC-Keloid vs. (SC-Myel, SC-Nonmyel)								
**gene**	**p_val**	**avg_log2FC**	**pct.1**	**pct.2**	**p_val_adj**	**Foldchange_UP**	**Foldchange_DOWN**	**Ratio_pct1_pct2**
SLC25A35	0,543117005	-0,066105741	0	0,012	1	0,955212923	1,046887009	0
CASTOR3	0,540537634	-0,066122197	0,031	0,016	1	0,955202027	1,046898951	1,9375
RNF146	0,045240417	-0,06614559	0,438	0,207	1	0,955186539	1,046915926	2,115942029
APOLD1	0,013407295	-0,066166518	0,156	0,043	1	0,955172683	1,046931113	3,627906977
LAMC2	0,480650464	-0,066217866	0	0,016	1	0,955138687	1,046968376	0
ZDHHC16	0,682170546	-0,066219841	0,031	0,02	1	0,955137379	1,04696981	1,55
INAFM2	0,767893042	-0,066365627	0,125	0,102	1	0,955040866	1,047075612	1,225490196
WDR76	0,543117005	-0,066368852	0	0,012	1	0,955038731	1,047077953	0
PPARA	0,80991663	-0,066396438	0,031	0,023	1	0,95502047	1,047097975	1,347826087
DFFB	0,543117005	-0,066475063	0	0,012	1	0,954968424	1,047155042	0
SNAP47	0,191913299	-0,066476144	0,094	0,039	1	0,954967709	1,047155826	2,41025641
ZC3H10	0,300902654	-0,06649709	0,062	0,027	1	0,954953844	1,04717103	2,296296296
ATAD3B	0,01071611	-0,066539088	0,219	0,07	1	0,954926045	1,047201514	3,128571429
CFAP70	0,480650464	-0,066568918	0	0,016	1	0,9549063	1,047223167	0
CCDC15	0,480650464	-0,066584233	0	0,016	1	0,954896164	1,047234283	0
FDX1	0,000973816	-0,066633475	0,469	0,16	1	0,954863572	1,047270028	2,93125
LAMB4	0,543117005	-0,066678733	0	0,012	1	0,954833618	1,047302882	0
ALG2	0,021379715	-0,066763325	0,156	0,047	1	0,954777633	1,047364292	3,319148936
GEMIN5	0,041848356	-0,066763923	0,062	0,012	1	0,954777238	1,047364726	5,166666667
CDIN1	0,543117005	-0,066977348	0	0,012	1	0,954636003	1,04751968	0
SINHCAF	0,300902654	-0,066981382	0,062	0,027	1	0,954633334	1,047522609	2,296296296
SPAG16	0,005195348	-0,06698988	0,219	0,062	1	0,954627711	1,047528779	3,532258065
SLC7A6	0,30793671	-0,067008924	0,062	0,027	1	0,954615109	1,047542607	2,296296296
BBS9	1,49244E-05	-0,067037365	0,219	0,031	0,314919708	0,954596291	1,047563258	7,064516129
SETD9	0,150155729	-0,067041541	0,062	0,02	1	0,954593527	1,04756629	3,1
CSTF2	0,386325862	-0,067096438	0,031	0,012	1	0,954557204	1,047606152	2,583333333
DNAH10OS	0,480650464	-0,067123258	0	0,016	1	0,954539459	1,047625628	0
MUC20	0,543117005	-0,067147725	0	0,012	1	0,95452327	1,047643395	0
KLK7	0,543117005	-0,067179926	0	0,012	1	0,954501966	1,047666779	0
TBX19	0,428872689	-0,06731182	0	0,02	1	0,954414707	1,047762563	0

Supplementary Table 1

Differentially expressed genes_adjacent-skin-XL_ comparing SC-Keloid vs. (SC-Myel, SC-Nonmyel)								
**gene**	**p_val**	**avg_log2FC**	**pct.1**	**pct.2**	**p_val_adj**	**Foldchange_UP**	**Foldchange_DOWN**	**Ratio_pct1_pct2**
CFAP410	0,009052775	-0,067355171	0,312	0,113	1	0,954386029	1,047794047	2,761061947
MAMLD1	0,063925678	-0,067368052	0,094	0,027	1	0,954377508	1,047803402	3,481481481
ZBTB22	0,004267025	-0,067375392	0,156	0,035	1	0,954372652	1,047808733	4,457142857
TTC21A	0,543117005	-0,067408874	0	0,012	1	0,954350503	1,047833051	0
LRRC3	0,540537634	-0,067458098	0,031	0,016	1	0,954317942	1,047868804	1,9375
JHY	0,001642148	-0,067507305	0,125	0,02	1	0,954285393	1,047904544	6,25
CCL14	0,985101745	-0,067575881	0,031	0,031	1	0,954240034	1,047954356	1
PRDX3	0,021863554	-0,067605223	0,344	0,145	1	0,954220626	1,04797567	2,372413793
PHF11	0,065872266	-0,067665212	0,219	0,094	1	0,95418095	1,048019247	2,329787234
UAP1L1	0,045196862	-0,067722553	0,125	0,039	1	0,954143026	1,048060902	3,205128205
MAP2K4	0,031413167	-0,067772147	0,156	0,051	1	0,954110226	1,048096931	3,058823529
GSPT1	0,009644385	-0,067835617	0,344	0,133	1	0,954068252	1,048143042	2,586466165
PNMA8B	0,386325862	-0,067850066	0,031	0,012	1	0,954058697	1,048153539	2,583333333
SCYL3	0,803381388	-0,067868029	0,031	0,023	1	0,954046818	1,04816659	1,347826087
CDKL3	0,18979066	-0,067884947	0,094	0,039	1	0,954035631	1,048178881	2,41025641
CCDC146	0,480650464	-0,06791121	0	0,016	1	0,954018263	1,048197963	0
FAM24B	0,226351275	-0,067917194	0,031	0,008	1	0,954014306	1,04820231	3,875
MGRN1	0,002818951	-0,068154739	0,219	0,059	1	0,953857237	1,048374915	3,711864407
KCNK7	0,543117005	-0,068156965	0	0,012	1	0,953855765	1,048376533	0
YWHAG	0,012821205	-0,06818637	0,5	0,219	1	0,953836324	1,048397901	2,283105023
SHC3	0,543117005	-0,068186677	0	0,012	1	0,953836121	1,048398124	0
ZCWPW2	0,150155729	-0,068199107	0,062	0,02	1	0,953827903	1,048407157	3,1
TTC5	0,097375089	-0,068343067	0,094	0,031	1	0,95373273	1,048511777	3,032258065
SPHK2	0,089393291	-0,068439757	0,125	0,047	1	0,953668812	1,048582052	2,659574468
ZFTA	0,918113127	-0,06851172	0,031	0,027	1	0,953621243	1,048634358	1,148148148
ESYT3	0,082317662	-0,068540426	0,031	0,004	1	0,953602269	1,048655223	7,75
HIF1AN	0,150155729	-0,068548882	0,062	0,02	1	0,95359668	1,048661369	3,1
ZNF891	0,150155729	-0,068568151	0,062	0,02	1	0,953583944	1,048675375	3,1
LMBR1	0,682170546	-0,068611001	0,031	0,02	1	0,953555621	1,048706523	1,55
ERP44	0,012057574	-0,068624756	0,438	0,18	1	0,95354653	1,048716522	2,433333333

Supplementary Table 1

Differentially expressed genes_adjacent-skin-XL_ comparing SC-Keloid vs. (SC-Myel, SC-Nonmyel)								
**gene**	**p_val**	**avg_log2FC**	**pct.1**	**pct.2**	**p_val_adj**	**Foldchange_UP**	**Foldchange_DOWN**	**Ratio_pct1_pct2**
FLVCR2	0,543117005	-0,068652298	0	0,012	1	0,953528326	1,048736542	0
NFYA	0,480650464	-0,068681748	0	0,016	1	0,953508862	1,04875795	0
STK4	0,002865711	-0,068715045	0,281	0,086	1	0,953486855	1,048782156	3,26744186
SGCB	0,007191855	-0,068736084	0,688	0,289	1	0,953472951	1,048797451	2,380622837
CEP76	0,540537634	-0,068797544	0,031	0,016	1	0,953432333	1,048842131	1,9375
GABBR1	0,901189191	-0,068908627	0,125	0,113	1	0,953358924	1,048922892	1,10619469
CEP83	0,224763613	-0,068928379	0,062	0,023	1	0,953345872	1,048937253	2,695652174
C1orf174	0,246047587	-0,068964275	0,094	0,043	1	0,953322152	1,048963352	2,186046512
EPOP	0,543117005	-0,068993827	0	0,012	1	0,953302624	1,048984839	0
EHMT2	0,47210696	-0,069094438	0,094	0,059	1	0,953236145	1,049057996	1,593220339
ANAPC7	0,01489812	-0,069102724	0,188	0,059	1	0,95323067	1,049064021	3,186440678
HYLS1	0,533973819	-0,0691119	0,031	0,016	1	0,953224607	1,049070694	1,9375
ANXA2R	0,540537634	-0,069170149	0,031	0,016	1	0,953186121	1,049113051	1,9375
BLVRA	0,008594651	-0,069198112	0,25	0,082	1	0,953167646	1,049133386	3,048780488
TMEM268	0,150155729	-0,069205447	0,062	0,02	1	0,953162801	1,049138719	3,1
AQP1	0,016795238	-0,06923015	0,375	0,168	1	0,95314648	1,049156684	2,232142857
LY6D	0,560566066	-0,069271897	0,062	0,039	1	0,953118899	1,049187044	1,58974359
ZNF579	0,540537634	-0,069309264	0,031	0,016	1	0,953094213	1,049214219	1,9375
BHLHE40-AS1	0,543117005	-0,069328168	0	0,012	1	0,953081724	1,049227967	0
PYCARD	0,150155729	-0,069338944	0,062	0,02	1	0,953074606	1,049235804	3,1
RBM19	0,091552625	-0,069359416	0,125	0,047	1	0,953061081	1,049250693	2,659574468
KPTN	0,019236133	-0,069478034	0,094	0,02	1	0,952982724	1,049336965	4,7
AGAP4	0,682170546	-0,069483624	0,031	0,02	1	0,952979031	1,049341032	1,55
NKIRAS2	0,080880928	-0,069581404	0,156	0,062	1	0,952914445	1,049412154	2,516129032
TVP23B	0,001783529	-0,069621514	0,219	0,055	1	0,952887952	1,04944133	3,981818182
MPP4	0,082317662	-0,069633797	0,031	0,004	1	0,95287984	1,049450265	7,75
YJU2	0,224763613	-0,069650439	0,062	0,023	1	0,952868848	1,049462371	2,695652174
ADCY2	0,543117005	-0,069664467	0	0,012	1	0,952859583	1,049472575	0
PEA15	0,010386793	-0,069671865	0,625	0,289	1	0,952854696	1,049477957	2,162629758
ERAL1	0,00208125	-0,069679259	0,188	0,043	1	0,952849813	1,049483336	4,372093023

Supplementary Table 1

Differentially expressed genes_adjacent-skin-XL_ comparing SC-Keloid vs. (SC-Myel, SC-Nonmyel)								
**gene**	**p_val**	**avg_log2FC**	**pct.1**	**pct.2**	**p_val_adj**	**Foldchange_UP**	**Foldchange_DOWN**	**Ratio_pct1_pct2**
PRPS1	0,24262347	-0,069725201	0,156	0,082	1	0,95281947	1,049516757	1,902439024
TMEM144	0,480650464	-0,069799145	0	0,016	1	0,952770635	1,04957055	0
GLI1	0,428872689	-0,069813825	0	0,02	1	0,952760941	1,04958123	0
MAST4	0,041848356	-0,069821612	0,062	0,012	1	0,952755798	1,049586895	5,166666667
OPN3	0,012987709	-0,069919728	0,156	0,043	1	0,952691005	1,049658279	3,627906977
CCDC107	0,075619507	-0,069961883	0,375	0,184	1	0,952663168	1,04968895	2,038043478
MYH7B	0,543117005	-0,069987451	0	0,012	1	0,952646285	1,049707553	0
BOLA2	0,010903421	-0,070034265	0,25	0,086	1	0,952615372	1,049741616	2,906976744
C16orf46	0,087959196	-0,070122447	0,062	0,016	1	0,952557147	1,049805781	3,875
ROPN1	0,38481627	-0,070167027	0	0,023	1	0,952527713	1,049838221	0
HS3ST2	0,226351275	-0,070168561	0,031	0,008	1	0,952526701	1,049839337	3,875
LSP1	0,480650464	-0,070170489	0	0,016	1	0,952525428	1,04984074	0
CDR1	0,543117005	-0,070243288	0	0,012	1	0,952477364	1,049893717	0
SLC25A42	0,80991663	-0,070258562	0,031	0,023	1	0,95246728	1,049904832	1,347826087
ZNF853	0,191913299	-0,070268506	0,094	0,039	1	0,952460715	1,049912069	2,41025641
GTF2E1	0,540537634	-0,070325016	0,031	0,016	1	0,952423408	1,049953195	1,9375
SLC7A8	0,543117005	-0,070329671	0	0,012	1	0,952420335	1,049956583	0
LRRC17	0,543117005	-0,070335699	0	0,012	1	0,952416356	1,049960969	0
EMSY	0,055416701	-0,070339548	0,188	0,074	1	0,952413815	1,04996377	2,540540541
USP27X	0,682170546	-0,070404602	0,031	0,02	1	0,952370869	1,050011117	1,55
PHYHIP	0,540537634	-0,070437547	0,031	0,016	1	0,952349122	1,050035094	1,9375
FAHD2A	0,286270979	-0,070457742	0,125	0,066	1	0,95233579	1,050049793	1,893939394
DTX4	0,480650464	-0,070559408	0	0,016	1	0,952268682	1,050123792	0
BOLA2B	0,224763613	-0,070575431	0,062	0,023	1	0,952258106	1,050135456	2,695652174
ZNF77	0,041848356	-0,070633056	0,062	0,012	1	0,952220071	1,050177401	5,166666667
MOB1A	0,000171537	-0,07064838	0,562	0,18	1	0,952209957	1,050188556	3,122222222
PPP1R1B	0,480650464	-0,070713528	0	0,016	1	0,952166959	1,050235981	0
VPS26A	0,000636209	-0,070730666	0,562	0,195	1	0,952155648	1,050248456	2,882051282
SCEL	0,226351275	-0,070854485	0,031	0,008	1	0,952073933	1,050338598	3,875
SPEG	0,480650464	-0,070869017	0	0,016	1	0,952064343	1,050349178	0

Supplementary Table 1

Differentially expressed genes_adjacent-skin-XL_ comparing SC-Keloid vs. (SC-Myel, SC-Nonmyel)								
**gene**	**p_val**	**avg_log2FC**	**pct.1**	**pct.2**	**p_val_adj**	**Foldchange_UP**	**Foldchange_DOWN**	**Ratio_pct1_pct2**
IQSEC1	0,089411268	-0,070904326	0,188	0,082	1	0,952041042	1,050374885	2,292682927
PIGQ	0,00187651	-0,070939018	0,219	0,055	1	0,952018149	1,050400143	3,981818182
TBC1D15	0,015301152	-0,070945832	0,312	0,121	1	0,952013653	1,050405104	2,578512397
HMGXB4	0,015203835	-0,070958005	0,219	0,074	1	0,95200562	1,050413967	2,959459459
GJC2	0,310355006	-0,070982824	0,094	0,047	1	0,951989243	1,050432038	2
TOP3A	0,150155729	-0,071067656	0,062	0,02	1	0,951933266	1,050493807	3,1
HPR	0,543117005	-0,071157961	0	0,012	1	0,951873682	1,050559564	0
ZNF614	0,682170546	-0,071161073	0,031	0,02	1	0,951871629	1,05056183	1,55
ZBTB25	0,24851603	-0,071211274	0,094	0,043	1	0,951838507	1,050598387	2,186046512
CLPX	0,021448302	-0,071214083	0,188	0,062	1	0,951836654	1,050600432	3,032258065
FAM227B	0,30793671	-0,071290421	0,062	0,027	1	0,95178629	1,050656025	2,296296296
DDX59	0,044552052	-0,071342942	0,125	0,039	1	0,951751642	1,050694274	3,205128205
NSMCE4A	0,001308481	-0,071349005	0,281	0,078	1	0,951747642	1,05069869	3,602564103
GDF10	0,543117005	-0,071370064	0	0,012	1	0,951733749	1,050714027	0
CERS5	0,371729093	-0,071384392	0,125	0,074	1	0,951724297	1,050724462	1,689189189
KLHL12	0,386325862	-0,07143713	0,031	0,012	1	0,951689508	1,050762872	2,583333333
ZNF345	0,540537634	-0,071551771	0,031	0,016	1	0,951613886	1,050846372	1,9375
PKDCC	0,191913299	-0,071584892	0,094	0,039	1	0,95159204	1,050870497	2,41025641
DDAH1	0,682170546	-0,071621604	0,031	0,02	1	0,951567824	1,05089724	1,55
SELENBP1	0,480650464	-0,071653565	0	0,016	1	0,951546744	1,050920521	0
ZNF808	0,224763613	-0,071857075	0,062	0,023	1	0,951412527	1,051068776	2,695652174
PUS1	0,487085433	-0,071893633	0,062	0,035	1	0,951388418	1,051095411	1,771428571
CCND3	0,00555836	-0,071932997	0,344	0,121	1	0,95136246	1,05112409	2,842975207
FUOM	0,046495836	-0,072115339	0,156	0,055	1	0,951242225	1,05125695	2,836363636
TMEM187	0,480650464	-0,072139496	0	0,016	1	0,951226297	1,051274553	0
STRADA	0,478627389	-0,072204955	0,062	0,035	1	0,951183138	1,051322253	1,771428571
ANGPT2	0,540537634	-0,07221581	0,031	0,016	1	0,951175981	1,051330164	1,9375
SOX3	0,480650464	-0,072232964	0	0,016	1	0,951164671	1,051342664	0
MTX1	0,002826784	-0,072269391	0,281	0,086	1	0,951140655	1,051369211	3,26744186
GDPD1	0,386325862	-0,072309351	0,031	0,012	1	0,951114311	1,051398332	2,583333333

Supplementary Table 1

Differentially expressed genes_adjacent-skin-XL_ comparing SC-Keloid vs. (SC-Myel, SC-Nonmyel)								
**gene**	**p_val**	**avg_log2FC**	**pct.1**	**pct.2**	**p_val_adj**	**Foldchange_UP**	**Foldchange_DOWN**	**Ratio_pct1_pct2**
AC011043.1	0,300902654	-0,072372122	0,062	0,027	1	0,951072929	1,051444079	2,296296296
EAF2	0,480650464	-0,072386142	0	0,016	1	0,951063687	1,051454296	0
TRIM52	0,150155729	-0,072467358	0,062	0,02	1	0,951010149	1,051513489	3,1
RNF152	0,543117005	-0,072633989	0	0,012	1	0,950900314	1,051634946	0
PROCA1	0,543117005	-0,072658916	0	0,012	1	0,950883884	1,051653116	0
ESRRA	0,083520674	-0,072765802	0,156	0,062	1	0,950813438	1,051731034	2,516129032
CNOT3	0,059034985	-0,072805181	0,188	0,074	1	0,950787486	1,051759741	2,540540541
DUSP18	0,004107327	-0,072923462	0,156	0,035	1	0,950709537	1,051845975	4,457142857
HECTD3	0,087959196	-0,072945747	0,062	0,016	1	0,950694852	1,051862223	3,875
IRF6	0,428872689	-0,072966054	0	0,02	1	0,95068147	1,051877029	0
FBXL19-AS1	0,543117005	-0,072990234	0	0,012	1	0,950665536	1,051894659	0
ZNF550	0,480650464	-0,073069935	0	0,016	1	0,950613019	1,051952771	0
ELMO2	0,470250215	-0,073111979	0,062	0,035	1	0,950585316	1,051983429	1,771428571
TMEM209	0,098751938	-0,073287445	0,094	0,031	1	0,950469709	1,052111383	3,032258065
POFUT2	0,005262184	-0,073326922	0,281	0,094	1	0,950443702	1,052140172	2,989361702
CTRL	0,428872689	-0,07338276	0	0,02	1	0,950406916	1,052180895	0
GPR157	0,543117005	-0,073459135	0	0,012	1	0,950356604	1,052236598	0
ZNF823	0,480650464	-0,07346258	0	0,016	1	0,950354335	1,052239111	0
CAVIN4	0,388432899	-0,073487555	0,062	0,031	1	0,950337883	1,052257326	2
TRAPPC10	0,090467775	-0,073530634	0,125	0,047	1	0,950309506	1,052288748	2,659574468
CCAR2	0,482846351	-0,07357149	0,062	0,035	1	0,950282594	1,052318548	1,771428571
SPATA2	0,138170297	-0,073621668	0,094	0,035	1	0,950249544	1,052355149	2,685714286
ANKRD65	0,000278482	-0,073622866	0,25	0,055	1	0,950248754	1,052356023	4,545454545
PTCD2	0,480650464	-0,073631436	0	0,016	1	0,95024311	1,052362274	0
PDK2	0,016562293	-0,073711539	0,125	0,031	1	0,950190351	1,052420706	4,032258065
CDK5RAP2	0,00606391	-0,073736571	0,188	0,051	1	0,950173864	1,052438967	3,68627451
MYOZ1	0,682170546	-0,073760117	0,031	0,02	1	0,950158356	1,052456144	1,55
AGGF1	0,002005544	-0,073771698	0,188	0,043	1	0,950150729	1,052464593	4,372093023
DACT1	0,396289917	-0,073812142	0,062	0,031	1	0,950124094	1,052494097	2
SPTLC3	0,80991663	-0,073876028	0,031	0,023	1	0,950082021	1,052540705	1,347826087

Supplementary Table 1

Differentially expressed genes_adjacent-skin-XL_ comparing SC-Keloid vs. (SC-Myel, SC-Nonmyel)								
**gene**	**p_val**	**avg_log2FC**	**pct.1**	**pct.2**	**p_val_adj**	**Foldchange_UP**	**Foldchange_DOWN**	**Ratio_pct1_pct2**
ZCWPW1	0,028712073	-0,073916577	0,125	0,035	1	0,950055318	1,052570288	3,571428571
MORC3	0,079867096	-0,07397743	0,281	0,137	1	0,950015245	1,052614687	2,051094891
CCDC117	0,006461838	-0,07404185	0,188	0,051	1	0,949972826	1,05266169	3,68627451
HPGD	0,543117005	-0,074152076	0	0,012	1	0,949900248	1,05274212	0
ABCA6	0,543117005	-0,074198172	0	0,012	1	0,949869898	1,052775756	0
WDR7	0,682170546	-0,074226902	0,031	0,02	1	0,949850982	1,052796722	1,55
EIF2S2	0,006896051	-0,074235307	0,656	0,285	1	0,949845448	1,052802855	2,301754386
ZNF783	0,924392442	-0,074254255	0,031	0,027	1	0,949832973	1,052816683	1,148148148
WDR25	0,578217897	-0,074256686	0,062	0,039	1	0,949831373	1,052818457	1,58974359
PODXL	0,533973819	-0,074327026	0,031	0,016	1	0,949785064	1,052869789	1,9375
FAM124A	0,543117005	-0,074348159	0	0,012	1	0,949771151	1,052885212	0
GTF2H2	0,979143607	-0,074464556	0,031	0,031	1	0,949694527	1,052970163	1
PTPRN2	0,682170546	-0,074510572	0,031	0,02	1	0,949664236	1,053003749	1,55
PACSIN2	0,008489906	-0,074584895	0,406	0,16	1	0,949615313	1,053057997	2,5375
SUPT6H	0,01602055	-0,074688416	0,25	0,09	1	0,949547176	1,053133563	2,777777778
FCHO2	0,002686655	-0,07473828	0,219	0,059	1	0,949514357	1,053169963	3,711864407
HEPN1	0,38481627	-0,07473907	0	0,023	1	0,949513837	1,053170539	0
DOP1B	0,224763613	-0,074745294	0,062	0,023	1	0,949509741	1,053175083	2,695652174
SPAG8	0,543115367	-0,074748024	0	0,012	1	0,949507944	1,053177076	0
LMCD1	0,162153727	-0,074763343	0,125	0,055	1	0,949497862	1,053188259	2,272727273
MMRN2	0,480650464	-0,074796216	0	0,016	1	0,949476227	1,053212257	0
ZC3H3	0,480650464	-0,07482932	0	0,016	1	0,949454441	1,053236424	0
ARFIP1	0,015776025	-0,074841034	0,312	0,121	1	0,949446731	1,053244976	2,578512397
RNF139-AS1	0,543117005	-0,074845528	0	0,012	1	0,949443774	1,053248257	0
OSGEP	0,008065251	-0,074881855	0,25	0,082	1	0,949419868	1,053274778	3,048780488
DHFR2	0,480650464	-0,074944102	0	0,016	1	0,949378905	1,053320224	0
FUCA2	0,001578028	-0,074962198	0,312	0,094	1	0,949366996	1,053333436	3,319148936
ZNF426	0,392349482	-0,075000652	0,062	0,031	1	0,949341692	1,053361512	2
IL16	0,38481627	-0,075012364	0	0,023	1	0,949333985	1,053370063	0
TADA2A	0,396289917	-0,075110347	0,062	0,031	1	0,949269512	1,053441607	2

Supplementary Table 1

Differentially expressed genes_adjacent-skin-XL_ comparing SC-Keloid vs. (SC-Myel, SC-Nonmyel)								
**gene**	**p_val**	**avg_log2FC**	**pct.1**	**pct.2**	**p_val_adj**	**Foldchange_UP**	**Foldchange_DOWN**	**Ratio_pct1_pct2**
ZNF567	0,396289917	-0,075120593	0,062	0,031	1	0,94926277	1,053449089	2
AEN	0,045196862	-0,075161495	0,125	0,039	1	0,949235858	1,053478956	3,205128205
GTF3C3	0,007871757	-0,0751851	0,156	0,039	1	0,949220327	1,053496192	4
FZR1	0,011296848	-0,075239971	0,219	0,07	1	0,949184225	1,053536262	3,128571429
TP53BP1	0,023464878	-0,075297528	0,188	0,062	1	0,949146358	1,053578294	3,032258065
H2BU1	0,480650464	-0,075326952	0	0,016	1	0,949127	1,053599782	0
DMPK	0,249776756	-0,075339787	0,156	0,086	1	0,949118556	1,053609155	1,813953488
ARHGAP20	0,480650464	-0,075339892	0	0,016	1	0,949118487	1,053609232	0
LINC01550	0,30793671	-0,075364281	0,062	0,027	1	0,949102442	1,053627044	2,296296296
DNMT3A	0,044241765	-0,075379034	0,156	0,055	1	0,949092737	1,053637818	2,836363636
LDHD	0,396289917	-0,075455348	0,062	0,031	1	0,949042534	1,053693553	2
RPS6KA4	0,020165275	-0,075546966	0,156	0,047	1	0,948982267	1,05376047	3,319148936
LOX	0,386325862	-0,075553232	0,031	0,012	1	0,948978145	1,053765047	2,583333333
KIF22	0,00711554	-0,075570323	0,219	0,066	1	0,948966903	1,05377753	3,318181818
GOLGA8B	0,044241765	-0,075574164	0,156	0,055	1	0,948964377	1,053780336	2,836363636
DNAJC27-AS1	0,924392442	-0,075576837	0,031	0,027	1	0,948962619	1,053782288	1,148148148
RSPH9	0,226351275	-0,075592436	0,031	0,008	1	0,948952358	1,053793683	3,875
OR2AT4	0,543117005	-0,075595061	0	0,012	1	0,948950631	1,0537956	0
ZBTB33	0,224763613	-0,075638208	0,062	0,023	1	0,948922251	1,053827117	2,695652174
TRAF3	0,991060715	-0,075643078	0,031	0,031	1	0,948919048	1,053830674	1
ZNF763	0,543117005	-0,075724524	0	0,012	1	0,94886548	1,053890168	0
RAI14	0,00052757	-0,075804705	0,219	0,047	1	0,948812746	1,053948742	4,659574468
DACT3	0,30757457	-0,075871076	0,094	0,047	1	0,948769096	1,053997231	2
LINC01011	0,682170546	-0,076027354	0,031	0,02	1	0,948666328	1,054111409	1,55
KCTD6	0,241162386	-0,076101489	0,094	0,043	1	0,94861758	1,054165578	2,186046512
TMEM9B-AS1	0,543117005	-0,076180232	0	0,012	1	0,948565806	1,054223116	0
PLEKHG5	0,38481627	-0,076213233	0	0,023	1	0,948544108	1,054247231	0
ACACB	0,918113127	-0,076264616	0,031	0,027	1	0,948510325	1,05428478	1,148148148
CD72	0,226351275	-0,076272786	0,031	0,008	1	0,948504954	1,054290751	3,875
FAM241B	0,480650464	-0,076284875	0	0,016	1	0,948497006	1,054299585	0

Supplementary Table 1

Differentially expressed genes_adjacent-skin-XL_ comparing SC-Keloid vs. (SC-Myel, SC-Nonmyel)								
**gene**	**p_val**	**avg_log2FC**	**pct.1**	**pct.2**	**p_val_adj**	**Foldchange_UP**	**Foldchange_DOWN**	**Ratio_pct1_pct2**
VMA21	0,005322289	-0,076493833	0,375	0,137	1	0,948359637	1,054452299	2,737226277
MRPS28	0,000277006	-0,076524884	0,375	0,105	1	0,948339226	1,054474994	3,571428571
PRPSAP1	0,002969767	-0,076549285	0,438	0,156	1	0,948323186	1,05449283	2,807692308
BCAR1	0,001503035	-0,076550268	0,438	0,148	1	0,94832254	1,054493548	2,959459459
VSNL1	0,543117005	-0,0765554	0	0,012	1	0,948319166	1,054497299	0
MAML1	0,569359326	-0,076562807	0,062	0,039	1	0,948314298	1,054502713	1,58974359
PIWIL4	0,480650464	-0,076563819	0	0,016	1	0,948313632	1,054503453	0
THUMPD3	0,001038602	-0,076750527	0,219	0,051	1	0,948190913	1,054639932	4,294117647
TMEM184C	0,098751938	-0,076793511	0,094	0,031	1	0,948162663	1,054671354	3,032258065
EIF2B5	0,015740914	-0,076894279	0,188	0,059	1	0,948096439	1,054745022	3,186440678
IRX5	0,480650464	-0,076949348	0	0,016	1	0,94806025	1,054785284	0
GDAP2	0,30793671	-0,076974792	0,062	0,027	1	0,94804353	1,054803886	2,296296296
CNOT6	0,106706178	-0,077033368	0,156	0,066	1	0,948005039	1,054846714	2,363636364
ZNF639	0,000520492	-0,077110333	0,25	0,059	1	0,947954465	1,05490299	4,237288136
NLN	0,020764757	-0,077183337	0,156	0,047	1	0,947906498	1,054956372	3,319148936
TENT2	0,154245377	-0,077222984	0,125	0,055	1	0,947880449	1,054985364	2,272727273
ARHGAP33	0,682170546	-0,077375001	0,031	0,02	1	0,947780576	1,055096534	1,55
FBXO5	0,80991663	-0,077428218	0,031	0,023	1	0,947745616	1,055135454	1,347826087
FAM83B	0,480650464	-0,077469157	0	0,016	1	0,947718722	1,055165396	0
CIITA	0,543117005	-0,077561791	0	0,012	1	0,947657872	1,055233149	0
FAXC	0,226351275	-0,077563195	0,031	0,008	1	0,94765695	1,055234176	3,875
CTNNA2	0,543117005	-0,077775479	0	0,012	1	0,947517517	1,055389459	0
GSR	0,484885847	-0,07780696	0,125	0,082	1	0,947496842	1,055412489	1,524390244
TAB3	0,233963311	-0,07783604	0,094	0,043	1	0,947477744	1,055433762	2,186046512
SRC	0,012011479	-0,077843978	0,25	0,086	1	0,947472531	1,055439569	2,906976744
SOSTDC1	0,428872689	-0,077849157	0	0,02	1	0,947469129	1,055443359	0
MICAL1	0,007871757	-0,077884068	0,156	0,039	1	0,947446202	1,055468899	4
FKBPL	0,008956837	-0,077897014	0,125	0,027	1	0,947437701	1,05547837	4,62962963
AR	0,480650464	-0,077938457	0	0,016	1	0,947410485	1,05550869	0
FZD8	0,150155729	-0,077958192	0,062	0,02	1	0,947397525	1,055523129	3,1

Supplementary Table 1

Differentially expressed genes_adjacent-skin-XL_ comparing SC-Keloid vs. (SC-Myel, SC-Nonmyel)								
**gene**	**p_val**	**avg_log2FC**	**pct.1**	**pct.2**	**p_val_adj**	**Foldchange_UP**	**Foldchange_DOWN**	**Ratio_pct1_pct2**
SELENOK	0,030598955	-0,077995816	0,812	0,395	1	0,947372818	1,055550656	2,055696203
FADS2	0,115570145	-0,078028671	0,125	0,051	1	0,947351243	1,055574695	2,450980392
CDV3	0,149055219	-0,078064725	0,438	0,25	1	0,947327569	1,055601075	1,752
GRIA3	0,480650464	-0,078064875	0	0,016	1	0,94732747	1,055601185	0
RPTOR	0,540537634	-0,07808398	0,031	0,016	1	0,947314925	1,055615164	1,9375
EIF3C	0,063925678	-0,078178748	0,094	0,027	1	0,9472527	1,055684507	3,481481481
ESAM	0,480650464	-0,078233433	0	0,016	1	0,947216796	1,055724523	0
CFAP91	0,480650464	-0,078237298	0	0,016	1	0,947214258	1,055727352	0
RBM24	0,38481627	-0,078276595	0	0,023	1	0,947188457	1,055756109	0
GGNBP2	0,03177037	-0,078278619	0,406	0,184	1	0,947187129	1,05575759	2,206521739
H4-16	0,428872689	-0,078289227	0	0,02	1	0,947180164	1,055765353	0
BTBD2	0,002185475	-0,078298747	0,25	0,07	1	0,947173914	1,055772319	3,571428571
ZNF649	0,004180266	-0,078314155	0,125	0,023	1	0,947163798	1,055783596	5,434782609
LSM6	0,030181651	-0,078333371	0,25	0,098	1	0,947151182	1,055797658	2,551020408
TOMM7	0,680787953	-0,078351234	0,781	0,625	1	0,947139455	1,055810731	1,2496
EOLA1	0,80991663	-0,0784409	0,031	0,023	1	0,947080591	1,055876353	1,347826087
SNAPC3	0,985101745	-0,078460286	0,031	0,031	1	0,947067865	1,055890541	1
NCSTN	0,010265241	-0,078493435	0,375	0,148	1	0,947046103	1,055914803	2,533783784
SLC12A7	0,480650464	-0,078503601	0	0,016	1	0,94703943	1,055922244	0
ZKSCAN8	0,480650464	-0,078515136	0	0,016	1	0,947031859	1,055930686	0
FANCL	0,096013629	-0,078660841	0,094	0,031	1	0,946936218	1,056037335	3,032258065
TRNAU1AP	0,106706178	-0,078678404	0,156	0,066	1	0,94692469	1,056050191	2,363636364
CDC42BPG	0,428872689	-0,078760864	0	0,02	1	0,946870568	1,056110554	0
RNFT1	0,224763613	-0,078768056	0,062	0,023	1	0,946865848	1,056115818	2,695652174
GSTT2B	0,116850675	-0,078781046	0,125	0,051	1	0,946857323	1,056125328	2,450980392
ZNF433	0,019236133	-0,078825179	0,094	0,02	1	0,946828358	1,056157636	4,7
ADPGK	0,004475279	-0,078916111	0,219	0,062	1	0,946768682	1,056224207	3,532258065
CLIP1	0,00356589	-0,078995651	0,375	0,133	1	0,946716485	1,056282441	2,819548872
CEP20	0,093753684	-0,079002773	0,125	0,047	1	0,946711812	1,056287655	2,659574468
MPHOSPH6	0,80991663	-0,079002812	0,031	0,023	1	0,946711786	1,056287684	1,347826087

Supplementary Table 1

Differentially expressed genes_adjacent-skin-XL_ comparing SC-Keloid vs. (SC-Myel, SC-Nonmyel)								
**gene**	**p_val**	**avg_log2FC**	**pct.1**	**pct.2**	**p_val_adj**	**Foldchange_UP**	**Foldchange_DOWN**	**Ratio_pct1_pct2**
LYPLAL1	0,030583271	-0,079008225	0,188	0,066	1	0,946708234	1,056291648	2,848484848
TMEM62	0,682170546	-0,07902164	0,031	0,02	1	0,946699431	1,056301469	1,55
CAMSAP1	0,953848385	-0,079035762	0,062	0,059	1	0,946690164	1,056311809	1,050847458
IGF2R	0,000951976	-0,079052276	0,25	0,062	1	0,946679328	1,056323901	4,032258065
GALNT4	0,540537634	-0,079134263	0,031	0,016	1	0,94662553	1,056383932	1,9375
FAM185A	0,985101745	-0,079160514	0,031	0,031	1	0,946608306	1,056403154	1
THADA	0,540537634	-0,079266592	0,031	0,016	1	0,946538706	1,056480832	1,9375
WDR6	0,001195376	-0,079347365	0,281	0,078	1	0,946485714	1,056539983	3,602564103
S100P	0,543117005	-0,079400848	0	0,012	1	0,946450626	1,056579152	0
BTBD3	0,020165275	-0,079407656	0,156	0,047	1	0,94644616	1,056584138	3,319148936
NEPRO	0,009954827	-0,079431995	0,188	0,055	1	0,946430193	1,056601963	3,418181818
SS18L1	0,150155729	-0,079528854	0,062	0,02	1	0,946366655	1,056672902	3,1
LRRC57	0,379471368	-0,079534982	0,125	0,074	1	0,946362635	1,056677391	1,689189189
DDX19A	0,011750536	-0,079698497	0,219	0,07	1	0,94625538	1,056797162	3,128571429
LASP1	0,031172497	-0,079711069	0,219	0,082	1	0,946247134	1,056806371	2,670731707
TCEANC	0,543117005	-0,079765501	0	0,012	1	0,946211434	1,056846244	0
ZDBF2	0,134746241	-0,079832526	0,094	0,035	1	0,946167475	1,056895345	2,685714286
KLHL15	0,243596382	-0,079877346	0,094	0,043	1	0,946138081	1,05692818	2,186046512
SORT1	0,00732945	-0,079915763	0,312	0,109	1	0,946112887	1,056956324	2,862385321
NRF1	0,183526587	-0,079979931	0,094	0,039	1	0,946070807	1,057003337	2,41025641
SERPINB7	0,480650464	-0,080099583	0	0,016	1	0,945992347	1,057091005	0
LCTL	0,682170546	-0,080143174	0,031	0,02	1	0,945963764	1,057122945	1,55
CLHC1	0,013891036	-0,080152468	0,062	0,008	1	0,94595767	1,057129755	7,75
GM2A	0,979143607	-0,080207418	0,031	0,031	1	0,945921641	1,05717002	1
PPME1	0,361186003	-0,080227403	0,094	0,051	1	0,945908537	1,057184665	1,843137255
FEN1	0,063925678	-0,080318841	0,094	0,027	1	0,945848587	1,057251671	3,481481481
BACE2	0,000280868	-0,080350877	0,469	0,145	1	0,945827585	1,057275148	3,234482759
CCDC82	0,013950247	-0,080355115	0,469	0,203	1	0,945824806	1,057278254	2,310344828
P2RX4	0,191913299	-0,080375959	0,094	0,039	1	0,945811141	1,05729353	2,41025641
GALK2	0,126128812	-0,080402607	0,125	0,051	1	0,945793671	1,057313059	2,450980392

Supplementary Table 1

Differentially expressed genes_adjacent-skin-XL_ comparing SC-Keloid vs. (SC-Myel, SC-Nonmyel)								
**gene**	**p_val**	**avg_log2FC**	**pct.1**	**pct.2**	**p_val_adj**	**Foldchange_UP**	**Foldchange_DOWN**	**Ratio_pct1_pct2**
ACVR2A	0,191913299	-0,080487169	0,094	0,039	1	0,945738236	1,057375034	2,41025641
CRNKL1	0,233614723	-0,080498911	0,156	0,082	1	0,945730539	1,05738364	1,902439024
CASKIN1	0,480650464	-0,080533101	0	0,016	1	0,945708127	1,057408699	0
IRAK3	0,480650464	-0,080627072	0	0,016	1	0,945646529	1,057477577	0
LATS2-AS1	0,543117005	-0,080637751	0	0,012	1	0,945639529	1,057485405	0
PITPNB	0,000238705	-0,080670847	0,562	0,18	1	0,945617836	1,057509664	3,122222222
ZNF549	0,543117005	-0,080737925	0	0,012	1	0,945573871	1,057558834	0
POU6F1	0,480650464	-0,080798842	0	0,016	1	0,945533945	1,05760349	0
RMRP	0,004180266	-0,080961287	0,125	0,023	1	0,945427486	1,05772258	5,434782609
MDN1	0,310355006	-0,080964824	0,094	0,047	1	0,945425168	1,057725174	2
ZNF181	0,38481627	-0,080977161	0	0,023	1	0,945417083	1,057734219	0
ALG10B	0,428872689	-0,081057902	0	0,02	1	0,945364174	1,057793417	0
PKHD1	0,480650464	-0,081142945	0	0,016	1	0,945308449	1,057855773	0
SMIM14	0,010944771	-0,081200264	0,312	0,117	1	0,945270893	1,057897802	2,666666667
ERRFI1	0,015740914	-0,081217617	0,188	0,059	1	0,945259523	1,057910527	3,186440678
SESN2	0,428872689	-0,081271237	0	0,02	1	0,945224391	1,057949847	0
HLF	0,803381388	-0,0813058	0,031	0,023	1	0,945201746	1,057975193	1,347826087
TCF20	0,243596382	-0,081329725	0,094	0,043	1	0,945186072	1,057992738	2,186046512
BRD3OS	0,480650464	-0,081344546	0	0,016	1	0,945176362	1,058003607	0
PPCDC	0,478627389	-0,081368067	0,062	0,035	1	0,945160952	1,058020857	1,771428571
RFX5	0,428872689	-0,081420591	0	0,02	1	0,945126542	1,058059376	0
GRK5	0,540537634	-0,081473632	0,031	0,016	1	0,945091795	1,058098277	1,9375
OXER1	0,543117005	-0,081481452	0	0,012	1	0,945086672	1,058104012	0
FKTN	0,30793671	-0,081490489	0,062	0,027	1	0,945080753	1,05811064	2,296296296
SLCO3A1	0,198962585	-0,081539695	0,125	0,059	1	0,945048519	1,05814673	2,118644068
POLA1	0,428872689	-0,081585726	0	0,02	1	0,945018367	1,058180492	0
MMS22L	0,087959196	-0,081624302	0,062	0,016	1	0,944993098	1,058208787	3,875
ZNF334	0,540537634	-0,081639479	0,031	0,016	1	0,944983158	1,058219919	1,9375
MTFMT	0,185597364	-0,081705278	0,094	0,039	1	0,944940059	1,058268184	2,41025641
TRMT44	0,543117005	-0,081740757	0	0,012	1	0,944916821	1,058294209	0

Supplementary Table 1

Differentially expressed genes_adjacent-skin-XL_ comparing SC-Keloid vs. (SC-Myel, SC-Nonmyel)								
**gene**	**p_val**	**avg_log2FC**	**pct.1**	**pct.2**	**p_val_adj**	**Foldchange_UP**	**Foldchange_DOWN**	**Ratio_pct1_pct2**
DPY19L3	0,020900593	-0,08174968	0,188	0,062	1	0,944910977	1,058300755	3,032258065
ZNF189	0,682170546	-0,081781172	0,031	0,02	1	0,944890351	1,058323856	1,55
MOB3A	0,045196862	-0,081939705	0,125	0,039	1	0,944786526	1,058440158	3,205128205
GYG1	0,00187651	-0,081952518	0,219	0,055	1	0,944778135	1,058449559	3,981818182
ZNF223	0,480650464	-0,082016355	0	0,016	1	0,944736331	1,058496394	0
MANBA	0,224763613	-0,082079205	0,062	0,023	1	0,944695175	1,058542508	2,695652174
PNMA6A	0,480650464	-0,082142444	0	0,016	1	0,944653766	1,058588909	0
LIMK2	0,80991663	-0,082300516	0,031	0,023	1	0,944550269	1,058704902	1,347826087
C17orf100	0,386325862	-0,082318298	0,031	0,012	1	0,944538627	1,058717951	2,583333333
EBF1	0,428872689	-0,082329891	0	0,02	1	0,944531037	1,058726458	0
NOP16	0,924392442	-0,082414677	0,031	0,027	1	0,944475529	1,058788681	1,148148148
UBE2E1	0,005526861	-0,082502431	0,25	0,078	1	0,944418082	1,058853085	3,205128205
ZFAT	0,480650464	-0,082527683	0	0,016	1	0,944401552	1,058871619	0
ARVCF	0,80991663	-0,082605841	0,031	0,023	1	0,94435039	1,058928985	1,347826087
NUDT17	0,540537634	-0,082628438	0,031	0,016	1	0,944335599	1,058945571	1,9375
TCP11L1	0,141661018	-0,082630409	0,094	0,035	1	0,944334309	1,058947018	2,685714286
ANKRD46	0,487085433	-0,082655742	0,062	0,035	1	0,944317727	1,058965612	1,771428571
FAM111A	0,000642175	-0,082664162	0,344	0,098	1	0,944312216	1,058971793	3,510204082
PAX3	0,12425419	-0,082774605	0,25	0,125	1	0,944239928	1,059052864	2
ADGRL1	0,037506898	-0,08282616	0,094	0,023	1	0,944206186	1,05909071	4,086956522
PCIF1	0,003101546	-0,082849894	0,219	0,059	1	0,944190653	1,059108133	3,711864407
MCM3AP	0,076632198	-0,082865903	0,156	0,062	1	0,944180176	1,059119886	2,516129032
JMJD4	0,540537634	-0,082970065	0,031	0,016	1	0,944112009	1,059196357	1,9375
CTIF	0,428872689	-0,083084723	0	0,02	1	0,944036978	1,05928054	0
ACTR5	0,480650464	-0,083148476	0	0,016	1	0,943995262	1,059327351	0
STEAP1B	0,162153727	-0,083216366	0,125	0,055	1	0,943950841	1,059377201	2,272727273
ZNF343	0,682170546	-0,083344822	0,031	0,02	1	0,943866796	1,059471531	1,55
TSPYL5	0,480650464	-0,083356736	0	0,016	1	0,943859002	1,05948028	0
KLK1	0,543117005	-0,083488116	0	0,012	1	0,943773053	1,059576767	0
ATP2B2	0,30793671	-0,083618077	0,062	0,027	1	0,943688039	1,05967222	2,296296296

Supplementary Table 1

Differentially expressed genes_adjacent-skin-XL_ comparing SC-Keloid vs. (SC-Myel, SC-Nonmyel)								
**gene**	**p_val**	**avg_log2FC**	**pct.1**	**pct.2**	**p_val_adj**	**Foldchange_UP**	**Foldchange_DOWN**	**Ratio_pct1_pct2**
RBM26-AS1	0,428872689	-0,083708673	0	0,02	1	0,943628781	1,059738766	0
ZFAND4	0,480650464	-0,08371905	0	0,016	1	0,943621994	1,059746389	0
DNAJC18	0,141661018	-0,08379292	0,094	0,035	1	0,943573679	1,059800652	2,685714286
KATNA1	0,081753212	-0,083801649	0,156	0,062	1	0,94356797	1,059807064	2,516129032
PIDD1	0,428872689	-0,083852868	0	0,02	1	0,943534471	1,059844691	0
SMIM8	0,031413167	-0,083895353	0,156	0,051	1	0,943506686	1,059875902	3,058823529
MALSU1	0,033543351	-0,084020326	0,281	0,117	1	0,943424959	1,059967717	2,401709402
GRAMD1A	0,004584175	-0,084020414	0,281	0,09	1	0,943424902	1,059967781	3,122222222
PDZRN3	0,793660383	-0,084035603	0,125	0,105	1	0,943414969	1,059978941	1,19047619
ZNF337-AS1	0,480650464	-0,084049874	0	0,016	1	0,943405637	1,059989426	0
GALC	0,012579781	-0,084053765	0,156	0,043	1	0,943403092	1,059992286	3,627906977
VTI1A	0,392349482	-0,08408197	0,062	0,031	1	0,943384649	1,060013008	2
DDX20	0,224763613	-0,084104995	0,062	0,023	1	0,943369593	1,060029927	2,695652174
KLHL20	0,065070981	-0,084131252	0,125	0,043	1	0,943352424	1,060049219	2,906976744
MRPS30	0,191913299	-0,084166226	0,094	0,039	1	0,943329555	1,060074917	2,41025641
ZNF324	0,487085433	-0,08417386	0,062	0,035	1	0,943324564	1,060080526	1,771428571
HMBOX1	0,004241146	-0,084180152	0,281	0,09	1	0,94332045	1,06008515	3,122222222
RNF207	0,543117005	-0,084194871	0	0,012	1	0,943310825	1,060095965	0
ZNF789	0,675506228	-0,08429425	0,031	0,02	1	0,943245849	1,060168992	1,55
WDR5	0,000237136	-0,084303444	0,219	0,043	1	0,943239837	1,060175748	5,093023256
NDUFAF6	0,021379715	-0,084314367	0,156	0,047	1	0,943232695	1,060183775	3,319148936
ACADVL	0,032365765	-0,084344155	0,719	0,348	1	0,943213221	1,060205665	2,066091954
RIPPLY2	0,543117005	-0,084344505	0	0,012	1	0,943212991	1,060205923	0
PRICKLE3	0,924392442	-0,084345794	0,031	0,027	1	0,943212149	1,06020687	1,148148148
SLC29A4	0,428872689	-0,084378854	0	0,02	1	0,943190535	1,060231165	0
PABIR2	0,299332476	-0,08452178	0,094	0,047	1	0,943097099	1,060336206	2
TMEM50B	0,001247106	-0,084551719	0,531	0,195	1	0,943077528	1,060358211	2,723076923
SEC23IP	0,241162386	-0,08458392	0,094	0,043	1	0,943056479	1,060381878	2,186046512
MAGEH1	0,00455806	-0,08467241	0,406	0,152	1	0,942998637	1,06044692	2,671052632
CCZ1B	0,804543977	-0,084722482	0,031	0,039	1	0,942965908	1,060483727	0,794871795

Supplementary Table 1

Differentially expressed genes_adjacent-skin-XL_ comparing SC-Keloid vs. (SC-Myel, SC-Nonmyel)								
**gene**	**p_val**	**avg_log2FC**	**pct.1**	**pct.2**	**p_val_adj**	**Foldchange_UP**	**Foldchange_DOWN**	**Ratio_pct1_pct2**
FRZB	0,543117005	-0,084776914	0	0,012	1	0,942930331	1,060523739	0
PRKCD	0,006461838	-0,084792083	0,188	0,051	1	0,942920417	1,060534889	3,68627451
POLR1C	0,141661018	-0,084796765	0,094	0,035	1	0,942917357	1,060538331	2,685714286
MSH6	0,869419867	-0,084824216	0,094	0,082	1	0,942899416	1,060558511	1,146341463
USF1	0,004180266	-0,084849624	0,125	0,023	1	0,94288281	1,060577189	5,434782609
PDIA2	0,428872689	-0,084920833	0	0,02	1	0,942836272	1,060629538	0
SLC41A3	0,010102949	-0,08498892	0,188	0,055	1	0,942791776	1,060679596	3,418181818
IGSF3	0,30793671	-0,085029853	0,062	0,027	1	0,942765028	1,06070969	2,296296296
NDRG3	0,077467066	-0,085037547	0,156	0,062	1	0,94276	1,060715347	2,516129032
FKRP	0,094667448	-0,085072795	0,094	0,031	1	0,942736966	1,060741263	3,032258065
TTC8	0,181472976	-0,08513374	0,094	0,039	1	0,942697142	1,060786073	2,41025641
MLH3	0,004107327	-0,085139881	0,156	0,035	1	0,942693129	1,060790589	4,457142857
PHAF1	0,428872689	-0,085143445	0	0,02	1	0,942690801	1,060793209	0
UBOX5	0,543117005	-0,085164594	0	0,012	1	0,942676982	1,06080876	0
SEL1L	0,013651996	-0,085276478	0,438	0,188	1	0,942603878	1,060891031	2,329787234
ASB8	0,030992783	-0,085298377	0,156	0,051	1	0,94258957	1,060907134	3,058823529
GRIA2	0,38481627	-0,085389803	0	0,023	1	0,942529839	1,060974368	0
WASF2	0,059010151	-0,085405192	0,656	0,348	1	0,942519785	1,060985685	1,885057471
CCDC173	0,480650464	-0,08543414	0	0,016	1	0,942500873	1,061006975	0
TOMM70	0,044149392	-0,085436566	0,188	0,07	1	0,942499288	1,061008759	2,685714286
UBE3C	0,0001347	-0,085512997	0,312	0,074	1	0,942449358	1,06106497	4,216216216
SYPL1	0,02802379	-0,085518241	0,656	0,324	1	0,942445932	1,061068827	2,024691358
SLX1A	0,386325862	-0,085531642	0,031	0,012	1	0,942437178	1,061078683	2,583333333
ARL17A	0,428872689	-0,085567069	0	0,02	1	0,942414036	1,061104739	0
PRKAA2	0,38481627	-0,085596444	0	0,023	1	0,942394847	1,061126346	0
FLNC	0,062914349	-0,085704598	0,094	0,027	1	0,942324202	1,061205897	3,481481481
IQCE	0,221757959	-0,085731646	0,062	0,023	1	0,942306535	1,061225794	2,695652174
NAGPA	0,569359326	-0,085745646	0,062	0,039	1	0,942297391	1,061236091	1,58974359
PAOX	0,226351275	-0,085805927	0,031	0,008	1	0,942258019	1,061280435	3,875
NPIPB4	0,346686972	-0,085825752	0	0,027	1	0,942245071	1,061295018	0

Supplementary Table 1

Differentially expressed genes_adjacent-skin-XL_ comparing SC-Keloid vs. (SC-Myel, SC-Nonmyel)								
**gene**	**p_val**	**avg_log2FC**	**pct.1**	**pct.2**	**p_val_adj**	**Foldchange_UP**	**Foldchange_DOWN**	**Ratio_pct1_pct2**
GRAMD2A	0,540537634	-0,085825916	0,031	0,016	1	0,942244964	1,061295139	1,9375
LINC01560	0,428872689	-0,085844284	0	0,02	1	0,942232968	1,061308651	0
IQCK	0,30793671	-0,085931412	0,062	0,027	1	0,942176065	1,061372749	2,296296296
ASNSD1	0,084415749	-0,085947287	0,156	0,062	1	0,942165698	1,061384428	2,516129032
KMT2D	0,979143607	-0,085983029	0,031	0,031	1	0,942142357	1,061410723	1
PDPN	0,028712073	-0,086031405	0,125	0,035	1	0,942110766	1,061446315	3,571428571
LIPA	0,000478083	-0,086050276	0,281	0,07	1	0,942098443	1,061460199	4,014285714
CCNE1	0,540537634	-0,086331717	0,031	0,016	1	0,941914676	1,061667289	1,9375
FOXP4	0,803745164	-0,086415826	0,062	0,051	1	0,941859764	1,061729186	1,215686275
TMEM140	0,38481627	-0,086442049	0	0,023	1	0,941842644	1,061748484	0
CPT1B	0,480650464	-0,086477401	0	0,016	1	0,941819566	1,061774502	0
LINC01136	0,543117005	-0,08655504	0	0,012	1	0,941768882	1,061831643	0
SMARCC2	0,036658896	-0,086642284	0,312	0,137	1	0,941711933	1,061895857	2,277372263
ZFHX2	0,428872689	-0,086643013	0	0,02	1	0,941711457	1,061896394	0
GTF3C2	0,016858455	-0,086685253	0,125	0,031	1	0,941683885	1,061927485	4,032258065
RANBP3	0,032268627	-0,086889972	0,156	0,051	1	0,94155027	1,062078183	3,058823529
RARB	0,480650464	-0,086950451	0	0,016	1	0,9415108	1,062122708	0
ZNF76	0,098751938	-0,086982294	0,094	0,031	1	0,941490019	1,062146151	3,032258065
SYTL4	0,487085433	-0,086993877	0,062	0,035	1	0,941482461	1,062154678	1,771428571
RFK	0,007632632	-0,08706945	0,219	0,066	1	0,941433144	1,062210319	3,318181818
TMC7	0,80991663	-0,08721337	0,031	0,023	1	0,941339233	1,062316288	1,347826087
SOX1-OT	0,38481627	-0,087222272	0	0,023	1	0,941333425	1,062322843	0
GON4L	0,138088965	-0,087232772	0,312	0,172	1	0,941326574	1,062330575	1,813953488
CFAP44	0,428872689	-0,087353156	0	0,02	1	0,941248029	1,062419223	0
DDX31	0,80991663	-0,087372351	0,031	0,023	1	0,941235506	1,062433359	1,347826087
GJB1	0,480650464	-0,087580032	0	0,016	1	0,941100022	1,062586311	0
PDLIM3	0,008622858	-0,087591443	0,406	0,168	1	0,941092578	1,062594715	2,416666667
LPCAT4	0,141661018	-0,087593451	0,094	0,035	1	0,941091268	1,062596194	2,685714286
PTBP3	0,011597498	-0,08759551	0,219	0,07	1	0,941089925	1,062597711	3,128571429
SIRT3	0,087959196	-0,087613135	0,062	0,016	1	0,941078428	1,062610693	3,875

Supplementary Table 1

Differentially expressed genes_adjacent-skin-XL_ comparing SC-Keloid vs. (SC-Myel, SC-Nonmyel)								
**gene**	**p_val**	**avg_log2FC**	**pct.1**	**pct.2**	**p_val_adj**	**Foldchange_UP**	**Foldchange_DOWN**	**Ratio_pct1_pct2**
F2RL2	0,480650464	-0,08761628	0	0,016	1	0,941076376	1,062613009	0
NGLY1	0,003791564	-0,087641626	0,188	0,047	1	0,941059844	1,062631677	4
GCN1	0,020463101	-0,087666877	0,156	0,047	1	0,941043373	1,062650276	3,319148936
HDX	0,682170546	-0,08767791	0,031	0,02	1	0,941036176	1,062658403	1,55
PHOSPHO2	0,480650464	-0,08771295	0	0,016	1	0,94101332	1,062684213	0
EXOSC3	0,887104347	-0,087728171	0,031	0,035	1	0,941003392	1,062695425	0,885714286
GCH1	0,38481627	-0,087744568	0	0,023	1	0,940992697	1,062707504	0
EFNA3	0,087959196	-0,087775833	0,062	0,016	1	0,940972305	1,062730534	3,875
PEX14	0,063925678	-0,087839989	0,094	0,027	1	0,940930461	1,062777794	3,481481481
SPTB	0,543117005	-0,087883463	0	0,012	1	0,940902108	1,06280982	0
ZNF383	0,898329199	-0,087927451	0,031	0,035	1	0,94087342	1,062842225	0,885714286
CDC14A	0,150155729	-0,088001893	0,062	0,02	1	0,940824873	1,062897069	3,1
ROBO1	0,141661018	-0,088025726	0,094	0,035	1	0,940809331	1,062914628	2,685714286
ZEB2-AS1	0,985101745	-0,088053723	0,031	0,031	1	0,940791074	1,062935255	1
MFSD6	0,019236133	-0,088116002	0,094	0,02	1	0,940750462	1,062981142	4,7
LAMB3	0,80991663	-0,088167298	0,031	0,023	1	0,940717013	1,063018937	1,347826087
AP5Z1	0,028712073	-0,088167884	0,125	0,035	1	0,940716632	1,063019369	3,571428571
RWDD2B	0,682170546	-0,08825566	0,031	0,02	1	0,940659398	1,063084047	1,55
CDK8	0,027811527	-0,088358948	0,125	0,035	1	0,940592056	1,06316016	3,571428571
PRR4	0,682170546	-0,088399351	0,031	0,02	1	0,940565715	1,063189934	1,55
YDJC	0,540537634	-0,088399776	0,031	0,016	1	0,940565437	1,063190247	1,9375
KCNK1	0,480650464	-0,088430319	0	0,016	1	0,940545525	1,063212756	0
BUD13	7,15271E-05	-0,088553305	0,188	0,027	1	0,940465349	1,063303396	6,962962963
MIR155HG	0,480650464	-0,088553807	0	0,016	1	0,940465022	1,063303766	0
TRIM39	0,80991663	-0,088610852	0,031	0,023	1	0,940427837	1,06334581	1,347826087
LARP6	0,006112579	-0,088623158	0,281	0,094	1	0,940419815	1,063354881	2,989361702
NHEJ1	0,682170546	-0,088639817	0,031	0,02	1	0,940408956	1,063367159	1,55
THNSL2	0,006360213	-0,088706209	0,188	0,051	1	0,94036568	1,063416096	3,68627451
FAM161B	0,682170546	-0,088746977	0,031	0,02	1	0,940339107	1,063446147	1,55
PRXL2B	0,020165275	-0,088784325	0,156	0,047	1	0,940314764	1,063473677	3,319148936

Supplementary Table 1

Differentially expressed genes_adjacent-skin-XL_ comparing SC-Keloid vs. (SC-Myel, SC-Nonmyel)								
**gene**	**p_val**	**avg_log2FC**	**pct.1**	**pct.2**	**p_val_adj**	**Foldchange_UP**	**Foldchange_DOWN**	**Ratio_pct1_pct2**
OAF	0,000191716	-0,088832959	0,469	0,145	1	0,940283066	1,063509528	3,234482759
LYSMD3	0,015740914	-0,088851773	0,188	0,059	1	0,940270804	1,063523398	3,186440678
SDHAF1	0,056601693	-0,08907061	0,188	0,074	1	0,940128188	1,063684732	2,540540541
SLC9A1	0,682170546	-0,089112702	0,031	0,02	1	0,94010076	1,063715766	1,55
HCFC1	0,033260329	-0,089134074	0,188	0,066	1	0,940086833	1,063731524	2,848484848
FGGY	0,396289917	-0,089175626	0,062	0,031	1	0,940059757	1,063762162	2
USP21	0,543117005	-0,089246895	0	0,012	1	0,94001332	1,063814712	0
PPWD1	0,06592651	-0,089296158	0,125	0,043	1	0,939981222	1,063851039	2,906976744
TSNARE1	0,428872689	-0,089296648	0	0,02	1	0,939980903	1,0638514	0
GATD3A	0,924392442	-0,089504913	0,031	0,027	1	0,939845219	1,064004987	1,148148148
GDF11	0,031413167	-0,08953786	0,156	0,051	1	0,939823756	1,064029286	3,058823529
ITIH3	0,428872689	-0,089585391	0	0,02	1	0,939792793	1,064064342	0
ZNF436-AS1	0,887104347	-0,089604705	0,031	0,035	1	0,939780211	1,064078588	0,885714286
TMEM88	0,480650464	-0,089735118	0	0,016	1	0,939695263	1,06417478	0
ZNF8	0,428872689	-0,089783436	0	0,02	1	0,939663792	1,064210421	0
SAMD9	0,30793671	-0,089816303	0,062	0,027	1	0,939642385	1,064234666	2,296296296
ST6GALNAC5	0,458984864	-0,089823187	0,031	0,066	1	0,939637902	1,064239744	0,46969697
MTARC1	0,480650464	-0,089857604	0	0,016	1	0,939615486	1,064265133	0
GTPBP4	0,021727091	-0,089866282	0,219	0,078	1	0,939609834	1,064271534	2,807692308
TTLL9	0,428872689	-0,089872461	0	0,02	1	0,93960581	1,064276093	0
SCNM1	0,163771487	-0,089901435	0,125	0,055	1	0,93958694	1,064297467	2,272727273
ASAP1	0,001026737	-0,089913649	0,312	0,09	1	0,939578985	1,064306478	3,466666667
ZNF595	0,480650464	-0,08991465	0	0,016	1	0,939578333	1,064307216	0
CLEC16A	0,38481627	-0,089938157	0	0,023	1	0,939563024	1,064324558	0
ZNF57	0,924392442	-0,08993937	0,031	0,027	1	0,939562234	1,064325453	1,148148148
NUBPL	0,682170546	-0,089969223	0,031	0,02	1	0,939542792	1,064347477	1,55
NUP214	0,409924554	-0,090013805	0,094	0,055	1	0,939513759	1,064380367	1,709090909
RRN3	0,633153567	-0,09007477	0,031	0,051	1	0,939474058	1,064425347	0,607843137
RSAD2	0,543117005	-0,090107958	0	0,012	1	0,939452447	1,064449833	0
AURKB	0,979143607	-0,090113795	0,031	0,031	1	0,939448646	1,06445414	1

Supplementary Table 1

Differentially expressed genes_adjacent-skin-XL_ comparing SC-Keloid vs. (SC-Myel, SC-Nonmyel)								
**gene**	**p_val**	**avg_log2FC**	**pct.1**	**pct.2**	**p_val_adj**	**Foldchange_UP**	**Foldchange_DOWN**	**Ratio_pct1_pct2**
NCOA3	0,073703017	-0,090129028	0,281	0,133	1	0,939438726	1,064465379	2,112781955
UBE2QL1	0,543117005	-0,090154501	0	0,012	1	0,939422139	1,064484174	0
S100A7	0,428872689	-0,090178025	0	0,02	1	0,939406821	1,064501532	0
MCM6	0,480650464	-0,090337764	0	0,016	1	0,939302814	1,064619402	0
PTPN4	0,008956837	-0,090451525	0,125	0,027	1	0,93922875	1,064703354	4,62962963
SERGEF	0,279554151	-0,090531115	0,125	0,066	1	0,939176936	1,064762093	1,893939394
ZC3H8	0,141661018	-0,090538725	0,094	0,035	1	0,939171982	1,06476771	2,685714286
SETD4	0,352307957	-0,09058456	0,094	0,051	1	0,939142145	1,064801538	1,843137255
ABTB1	0,355251835	-0,090638781	0,094	0,051	1	0,939106849	1,064841557	1,843137255
GNAZ	0,480650464	-0,090639554	0	0,016	1	0,939106347	1,064842128	0
ALG10	0,80991663	-0,090697274	0,031	0,023	1	0,939068775	1,064884731	1,347826087
FCF1	0,044552052	-0,090721167	0,125	0,039	1	0,939053223	1,064902367	3,205128205
CCT6B	0,008148126	-0,090738788	0,094	0,016	1	0,939041753	1,064915374	5,875
RAB5A	0,009784256	-0,090774705	0,5	0,211	1	0,939018375	1,064941887	2,369668246
FMC1	0,001797983	-0,090804817	0,406	0,137	1	0,938998776	1,064964114	2,96350365
APBA1	0,30793671	-0,090885848	0,062	0,027	1	0,938946038	1,065023931	2,296296296
TBC1D22B	0,540537634	-0,090904757	0,031	0,016	1	0,938933731	1,06503789	1,9375
CTR9	0,24851603	-0,09095277	0,094	0,043	1	0,938902484	1,065073335	2,186046512
RSKR	0,480650464	-0,091022171	0	0,016	1	0,938857319	1,065124572	0
PRDX2	0,015844528	-0,091070952	0,438	0,184	1	0,938825574	1,065160587	2,380434783
DUS3L	0,578217897	-0,091094011	0,062	0,039	1	0,938810569	1,065177612	1,58974359
TEPSIN	0,80991663	-0,091193225	0,031	0,023	1	0,938746009	1,065250867	1,347826087
HPS3	0,191913299	-0,091206912	0,094	0,039	1	0,938737104	1,065260972	2,41025641
PPP1R1C	0,346686972	-0,091272335	0	0,027	1	0,938694535	1,065309281	0
NLRC5	0,682170546	-0,091303744	0,031	0,02	1	0,938674099	1,065332474	1,55
PAQR3	0,198962585	-0,091309432	0,125	0,059	1	0,938670398	1,065336674	2,118644068
RFNG	0,019958062	-0,09131923	0,312	0,125	1	0,938664023	1,065343909	2,496
CORO7	0,794711529	-0,091453834	0,062	0,051	1	0,93857645	1,065443311	1,215686275
ZNF646	0,543117005	-0,091477233	0	0,012	1	0,938561227	1,065460592	0
SPATA7	0,150155729	-0,091491694	0,062	0,02	1	0,938551819	1,065471271	3,1

Supplementary Table 1

Differentially expressed genes_adjacent-skin-XL_ comparing SC-Keloid vs. (SC-Myel, SC-Nonmyel)								
**gene**	**p_val**	**avg_log2FC**	**pct.1**	**pct.2**	**p_val_adj**	**Foldchange_UP**	**Foldchange_DOWN**	**Ratio_pct1_pct2**
ARHGEF9	0,351727998	-0,091605083	0,125	0,07	1	0,938478056	1,065555015	1,785714286
STRIP2	0,543117005	-0,091643178	0	0,012	1	0,938453275	1,065583153	0
CCDC122	0,979143607	-0,091678626	0,031	0,031	1	0,938430217	1,065609335	1
MTERF2	0,191913299	-0,091701467	0,094	0,039	1	0,93841536	1,065626206	2,41025641
COPS3	0,006012768	-0,091756346	0,344	0,121	1	0,938379664	1,065666742	2,842975207
TLCD2	0,428872689	-0,091768038	0	0,02	1	0,938372059	1,065675379	0
UBALD1	0,004895615	-0,091788751	0,219	0,062	1	0,938358587	1,065690679	3,532258065
PRR3	0,396289917	-0,091821295	0,062	0,031	1	0,93833742	1,065714719	2
AZI2	0,004409647	-0,091837077	0,281	0,09	1	0,938327155	1,065726377	3,122222222
C2CD2	0,224763613	-0,091853008	0,062	0,023	1	0,938316794	1,065738146	2,695652174
MYO1G	0,543117005	-0,091872568	0	0,012	1	0,938304073	1,065752595	0
WWC2	0,01489812	-0,09189341	0,188	0,059	1	0,938290517	1,065767992	3,186440678
ZBTB18	0,38481627	-0,091909267	0	0,023	1	0,938280205	1,065779705	0
LRIF1	0,141661018	-0,092044023	0,094	0,035	1	0,938192567	1,065879261	2,685714286
LRRC69	0,480650464	-0,09213213	0	0,016	1	0,938135273	1,065944356	0
CHCHD7	0,007958785	-0,092164526	0,219	0,066	1	0,938114207	1,065968293	3,318181818
TKFC	0,80991663	-0,092203011	0,031	0,023	1	0,938089183	1,065996729	1,347826087
GPRC5B	0,428872689	-0,09221424	0	0,02	1	0,938081881	1,066005026	0
CSTF3	0,012380103	-0,092346514	0,156	0,043	1	0,937995877	1,066102767	3,627906977
FAM117B	0,480650464	-0,092476508	0	0,016	1	0,937911363	1,066198833	0
CLDN1	0,540537634	-0,092533613	0,031	0,016	1	0,937874239	1,066241036	1,9375
RTN4RL2	0,428872689	-0,092559389	0	0,02	1	0,937857482	1,066260086	0
FRMD1	0,38481627	-0,092611593	0	0,023	1	0,937823546	1,06629867	0
MIR22HG	0,067638227	-0,092626524	0,188	0,078	1	0,937813841	1,066309705	2,41025641
SCARF2	0,428872689	-0,092658212	0	0,02	1	0,937793242	1,066333126	0
GSAP	0,80991663	-0,092662484	0,031	0,023	1	0,937790465	1,066336284	1,347826087
QPRT	0,924392442	-0,092723612	0,031	0,027	1	0,937750731	1,066381466	1,148148148
PISD	0,063925678	-0,092836165	0,094	0,027	1	0,937677574	1,066464665	3,481481481
KLHDC9	0,480650464	-0,092853055	0	0,016	1	0,937666597	1,06647715	0
CHST6	0,682170546	-0,092998347	0,031	0,02	1	0,937572171	1,066584559	1,55

Supplementary Table 1

Differentially expressed genes_adjacent-skin-XL_ comparing SC-Keloid vs. (SC-Myel, SC-Nonmyel)								
**gene**	**p_val**	**avg_log2FC**	**pct.1**	**pct.2**	**p_val_adj**	**Foldchange_UP**	**Foldchange_DOWN**	**Ratio_pct1_pct2**
MIOS	0,06592651	-0,093034259	0,125	0,043	1	0,937548833	1,066611109	2,906976744
RAB2B	0,737388712	-0,093143075	0,062	0,047	1	0,93747812	1,066691562	1,319148936
ZNF263	0,641781411	-0,093169767	0,031	0,051	1	0,937460776	1,066711297	0,607843137
SH3PXD2A	0,031803489	-0,093193662	0,625	0,324	1	0,937445249	1,066728965	1,929012346
TTC9C	0,139907281	-0,093227828	0,094	0,035	1	0,937423048	1,066754228	2,685714286
TELO2	0,304406192	-0,093282365	0,062	0,027	1	0,937387613	1,066794554	2,296296296
TSPAN7	0,663576959	-0,093292999	0,062	0,043	1	0,937380703	1,066802417	1,441860465
PRDM4	0,755689148	-0,093353578	0,062	0,047	1	0,937341343	1,066847213	1,319148936
NFYC	0,01158477	-0,093429521	0,25	0,086	1	0,937292003	1,066903374	2,906976744
PCDHGB7	0,386325862	-0,09344237	0,031	0,012	1	0,937283655	1,066912876	2,583333333
RABL2A	0,478627389	-0,093443661	0,062	0,035	1	0,937282816	1,066913831	1,771428571
CTDSPL	0,00660872	-0,093470655	0,312	0,109	1	0,937265279	1,066933793	2,862385321
FBXO46	0,428872689	-0,093470747	0	0,02	1	0,93726522	1,066933861	0
SNRNP35	0,24851603	-0,093486859	0,094	0,043	1	0,937254753	1,066945777	2,186046512
CCDC191	0,80991663	-0,09354217	0,031	0,023	1	0,93721882	1,066986683	1,347826087
SEC14L5	0,38481627	-0,093547657	0	0,023	1	0,937215255	1,066990741	0
ZMAT5	0,00187651	-0,093558738	0,219	0,055	1	0,937208057	1,066998936	3,981818182
MAN2A1	0,021653988	-0,093587707	0,25	0,094	1	0,937189238	1,067020362	2,659574468
DMTN	0,480650464	-0,093588049	0	0,016	1	0,937189016	1,067020615	0
CDHR1	0,543117005	-0,093643266	0	0,012	1	0,937153147	1,067061454	0
PLIN2	0,927044416	-0,093655869	0,062	0,066	1	0,93714496	1,067070776	0,939393939
THAP7-AS1	0,30793671	-0,093657381	0,062	0,027	1	0,937143979	1,067071894	2,296296296
CAVIN2	0,428872689	-0,093710049	0	0,02	1	0,937109767	1,06711085	0
TMEM191C	0,480650464	-0,093729408	0	0,016	1	0,937097193	1,067125169	0
PELP1	0,002053304	-0,093757325	0,156	0,031	1	0,937079059	1,067145819	5,032258065
POLL	0,236345884	-0,093800303	0,094	0,043	1	0,937051144	1,06717761	2,186046512
NPFFR1	0,543117005	-0,093806819	0	0,012	1	0,937046912	1,06718243	0
FIG4	0,746521062	-0,094064933	0,062	0,047	1	0,936879279	1,067373378	1,319148936
CMTM4	0,480650464	-0,094075209	0	0,016	1	0,936872605	1,06738098	0
GKAP1	0,979143607	-0,094088889	0,031	0,031	1	0,936863722	1,067391101	1

Supplementary Table 1

Differentially expressed genes_adjacent-skin-XL_ comparing SC-Keloid vs. (SC-Myel, SC-Nonmyel)								
**gene**	**p_val**	**avg_log2FC**	**pct.1**	**pct.2**	**p_val_adj**	**Foldchange_UP**	**Foldchange_DOWN**	**Ratio_pct1_pct2**
DDX39A	0,005265092	-0,09409083	0,375	0,137	1	0,936862461	1,067392537	2,737226277
C2orf92	0,540537634	-0,094135877	0,031	0,016	1	0,936833209	1,067425866	1,9375
USP3	0,654515972	-0,094258236	0,062	0,043	1	0,936753757	1,067516402	1,441860465
SSX2IP	0,386325862	-0,094260903	0,031	0,012	1	0,936752025	1,067518375	2,583333333
IFT172	0,80991663	-0,094297407	0,031	0,023	1	0,936728323	1,067545386	1,347826087
RAE1	0,00372725	-0,09430568	0,188	0,047	1	0,936722952	1,067551508	4
ZNF14	0,428872689	-0,094439563	0	0,02	1	0,936636028	1,067650582	0
SLC38A9	0,428872689	-0,094504247	0	0,02	1	0,936594034	1,067698452	0
FAM228B	0,264732918	-0,094514961	0,156	0,086	1	0,936587079	1,067706381	1,813953488
ATL3	0,000702579	-0,094675863	0,375	0,113	1	0,936482627	1,067825468	3,318584071
KCND1	0,386325862	-0,094707134	0,031	0,012	1	0,936462329	1,067848614	2,583333333
LETM1	0,002540231	-0,094725431	0,25	0,07	1	0,936450453	1,067862156	3,571428571
TRMT9B	0,803381388	-0,094772912	0,031	0,023	1	0,936419633	1,067897302	1,347826087
PPP1R13B	0,682170546	-0,094814617	0,031	0,02	1	0,936392564	1,067928173	1,55
SEPTIN3	0,38481627	-0,095021196	0	0,023	1	0,936258492	1,0680811	0
HES6	0,011438702	-0,095043492	0,281	0,102	1	0,936244023	1,068097606	2,754901961
E2F6	0,141661018	-0,095130711	0,094	0,035	1	0,936187423	1,068162181	2,685714286
CDC20B	0,588139692	-0,095212991	0,094	0,066	1	0,936134032	1,068223103	1,424242424
C2CD2L	0,150155729	-0,095235429	0,062	0,02	1	0,936119472	1,068239717	3,1
ZNF441	0,30793671	-0,09523618	0,062	0,027	1	0,936118985	1,068240272	2,296296296
IQCC	0,543117005	-0,095240893	0	0,012	1	0,936115927	1,068243762	0
SHC2	0,38481627	-0,095285744	0	0,023	1	0,936086825	1,068276973	0
STAT6	0,841804635	-0,095321335	0,094	0,105	1	0,936063732	1,068303327	0,895238095
CSTF1	0,224763613	-0,09534086	0,062	0,023	1	0,936051064	1,068317786	2,695652174
TMEM120B	0,346686972	-0,09542148	0	0,027	1	0,935998758	1,068377486	0
MRAS	0,246047587	-0,095445777	0,094	0,043	1	0,935982994	1,068395479	2,186046512
DCAF6	0,46012646	-0,095490604	0,156	0,105	1	0,935953912	1,068428677	1,485714286
PYGO1	0,918113127	-0,095633022	0,031	0,027	1	0,935861522	1,068534154	1,148148148
IDH3G	0,000101021	-0,095636248	0,469	0,133	1	0,93585943	1,068536543	3,526315789
PDE8A	0,000957468	-0,095744295	0,375	0,117	1	0,935789343	1,068616572	3,205128205

Supplementary Table 1

Differentially expressed genes_adjacent-skin-XL_ comparing SC-Keloid vs. (SC-Myel, SC-Nonmyel)								
**gene**	**p_val**	**avg_log2FC**	**pct.1**	**pct.2**	**p_val_adj**	**Foldchange_UP**	**Foldchange_DOWN**	**Ratio_pct1_pct2**
SRCAP	0,958239549	-0,095744882	0,062	0,059	1	0,935788963	1,068617006	1,050847458
FAM83H	0,428872689	-0,095822509	0	0,02	1	0,935738612	1,068674507	0
BSDC1	0,077284458	-0,095939235	0,281	0,133	1	0,935662906	1,068760975	2,112781955
MPV17L	0,428872689	-0,095955689	0	0,02	1	0,935652235	1,068773164	0
NBPF14	0,428872689	-0,095966394	0	0,02	1	0,935645292	1,068781095	0
NCOA5	0,396289917	-0,095981876	0,062	0,031	1	0,935635251	1,068792565	2
CCDC81	0,543117005	-0,096068342	0	0,012	1	0,935579178	1,068856623	0
RTL10	0,80991663	-0,096136863	0,031	0,023	1	0,935534743	1,06890739	1,347826087
RINT1	0,979143607	-0,096201943	0,031	0,031	1	0,935492542	1,068955609	1
BIVM	0,307936041	-0,096260007	0,062	0,027	1	0,935454892	1,068998632	2,296296296
CEP152	0,808271912	-0,096405186	0,062	0,051	1	0,935360762	1,069106211	1,215686275
SGPL1	0,130767324	-0,096407868	0,156	0,07	1	0,935359023	1,069108199	2,228571429
PHF21A	0,309574808	-0,096413362	0,156	0,09	1	0,935355461	1,06911227	1,733333333
HEATR6	0,979143607	-0,09641512	0,031	0,031	1	0,935354321	1,069113573	1
CTDSPL2	0,055416701	-0,096506899	0,188	0,074	1	0,935294819	1,069181588	2,540540541
CHAC1	0,428872689	-0,096749038	0	0,02	1	0,935137854	1,069361052	0
ELOVL7	0,38481627	-0,096865349	0	0,023	1	0,935062466	1,069447268	0
ZBTB4	0,092820674	-0,096880909	0,188	0,082	1	0,935052381	1,069458803	2,292682927
CA11	0,090467775	-0,096918588	0,125	0,047	1	0,935027961	1,069486734	2,659574468
PTPRU	0,592641159	-0,096922816	0,031	0,055	1	0,93502522	1,069489869	0,563636364
SMIM13	0,158804529	-0,096961398	0,156	0,074	1	0,935000215	1,069518471	2,108108108
MEMO1	0,924392442	-0,09697894	0,031	0,027	1	0,934988846	1,069531475	1,148148148
ZBTB7B	0,578217897	-0,097053153	0,062	0,039	1	0,934940751	1,069586494	1,58974359
MUTYH	0,241007682	-0,097058588	0,125	0,062	1	0,934937229	1,069590523	2,016129032
WWC3	0,107772109	-0,097118409	0,156	0,066	1	0,934898463	1,069634874	2,363636364
AGL	0,478627389	-0,097149302	0,062	0,035	1	0,934878444	1,069657779	1,771428571
IPP	0,138170297	-0,09714986	0,094	0,035	1	0,934878082	1,069658193	2,685714286
ASXL3	0,543117005	-0,097194341	0	0,012	1	0,934849259	1,069691173	0
COX7A1	0,025744792	-0,097202514	0,406	0,18	1	0,934843963	1,069697233	2,255555556
B3GAT1	0,979143607	-0,097257547	0,031	0,031	1	0,934808303	1,069738038	1

Supplementary Table 1

Differentially expressed genes_adjacent-skin-XL_ comparing SC-Keloid vs. (SC-Myel, SC-Nonmyel)								
**gene**	**p_val**	**avg_log2FC**	**pct.1**	**pct.2**	**p_val_adj**	**Foldchange_UP**	**Foldchange_DOWN**	**Ratio_pct1_pct2**
KANK3	0,38481627	-0,097340841	0	0,023	1	0,934754333	1,069799801	0
LRP5	0,803381388	-0,097436912	0,031	0,023	1	0,934692089	1,069871043	1,347826087
HMGA2	0,480650464	-0,097449487	0	0,016	1	0,934683942	1,069880369	0
ZNF28	0,041848356	-0,097482259	0,062	0,012	1	0,93466271	1,069904672	5,166666667
HMGCR	0,246047587	-0,097504272	0,094	0,043	1	0,934648448	1,069920997	2,186046512
CCL19	0,543117005	-0,097522925	0	0,012	1	0,934636364	1,069934831	0
CBFA2T2	0,482846351	-0,097563963	0,062	0,035	1	0,934609779	1,069965265	1,771428571
LUM	0,428872689	-0,097587648	0	0,02	1	0,934594435	1,069982832	0
RBSN	0,361186003	-0,097623204	0,094	0,051	1	0,934571402	1,070009202	1,843137255
H2AC19	0,428872689	-0,097640323	0	0,02	1	0,934560313	1,070021899	0
ZC3H14	0,315872824	-0,097656675	0,156	0,09	1	0,934549719	1,070034027	1,733333333
C18orf25	0,425948605	-0,097674821	0,125	0,078	1	0,934537965	1,070047486	1,602564103
GMCL1	0,133225265	-0,097702767	0,156	0,07	1	0,934519863	1,070068214	2,228571429
WASF1	0,38481627	-0,097777807	0	0,023	1	0,934471256	1,070123873	0
RABL2B	0,30206331	-0,097789066	0,094	0,047	1	0,934463963	1,070132225	2
NXT2	0,924392442	-0,097827736	0,031	0,027	1	0,934438916	1,070160909	1,148148148
KRI1	0,139907281	-0,097865999	0,094	0,035	1	0,934414133	1,070189292	2,685714286
LINC01588	0,396289917	-0,097883059	0,062	0,031	1	0,934403084	1,070201947	2
RGS5	0,543117005	-0,097920128	0	0,012	1	0,934379075	1,070229446	0
AKT3	0,008008107	-0,097936513	0,375	0,141	1	0,934368464	1,0702416	2,659574468
NKAP	0,000524333	-0,097945201	0,344	0,098	1	0,934362837	1,070248046	3,510204082
AP3S2	0,432193743	-0,097953156	0,094	0,055	1	0,934357685	1,070253947	1,709090909
MSLN	0,543117005	-0,097986561	0	0,012	1	0,93433605	1,070278729	0
BCL9L	0,335252921	-0,097990129	0,156	0,094	1	0,934333739	1,070281376	1,659574468
TRIO	0,028002326	-0,098009762	0,281	0,113	1	0,934321025	1,07029594	2,486725664
ZHX3	0,682170546	-0,098058071	0,031	0,02	1	0,93428974	1,07033178	1,55
CDH15	0,428872689	-0,098061755	0	0,02	1	0,934287353	1,070334514	0
FHIP2A	0,392349482	-0,098081539	0,062	0,031	1	0,934274541	1,070349191	2
KLHL17	0,224763613	-0,098082367	0,062	0,023	1	0,934274005	1,070349806	2,695652174
NFKBIE	0,031707035	-0,098101759	0,188	0,066	1	0,934261447	1,070364193	2,848484848

Supplementary Table 1

Differentially expressed genes_adjacent-skin-XL_ comparing SC-Keloid vs. (SC-Myel, SC-Nonmyel)								
**gene**	**p_val**	**avg_log2FC**	**pct.1**	**pct.2**	**p_val_adj**	**Foldchange_UP**	**Foldchange_DOWN**	**Ratio_pct1_pct2**
EMG1	0,012303637	-0,098147292	0,25	0,086	1	0,934231962	1,070397975	2,906976744
TOM1	0,251411476	-0,098186116	0,125	0,062	1	0,934206821	1,070426781	2,016129032
FLOT2	0,186547601	-0,098187573	0,156	0,078	1	0,934205877	1,070427862	2
MPP1	0,80991663	-0,098196538	0,031	0,023	1	0,934200072	1,070434514	1,347826087
PIGS	0,054252457	-0,098220389	0,188	0,074	1	0,934184628	1,070452211	2,540540541
AGK	0,001055584	-0,098224954	0,188	0,039	1	0,934181672	1,070455597	4,820512821
NKRF	0,80991663	-0,098258483	0,031	0,023	1	0,934159962	1,070480475	1,347826087
NLRP1	0,045196862	-0,098269491	0,125	0,039	1	0,934152834	1,070488644	3,205128205
PAPOLG	0,979143607	-0,098287011	0,031	0,031	1	0,934141489	1,070501644	1
PARG	0,098751938	-0,098308736	0,094	0,031	1	0,934127423	1,070517764	3,032258065
GSN	0,19131494	-0,098327394	0,938	0,766	1	0,934115342	1,070531609	1,224543081
STK40	0,253529282	-0,098507127	0,125	0,062	1	0,933998976	1,070664986	2,016129032
LMTK2	0,38481627	-0,0985773	0	0,023	1	0,933953547	1,070717065	0
KCTD1	0,797009215	-0,098580031	0,062	0,074	1	0,933951779	1,070719091	0,837837838
RIPOR1	0,010903421	-0,098635022	0,25	0,086	1	0,93391618	1,070759905	2,906976744
MET	0,487085433	-0,098640943	0,062	0,035	1	0,933912347	1,070764299	1,771428571
DICER1-AS1	0,924392442	-0,098655834	0,031	0,027	1	0,933902708	1,070775351	1,148148148
AARS2	0,226351275	-0,098655853	0,031	0,008	1	0,933902696	1,070775365	3,875
LRRC40	0,045196862	-0,098694826	0,125	0,039	1	0,933877468	1,070804291	3,205128205
VPS9D1	0,304810673	-0,098744663	0,094	0,047	1	0,933845208	1,070841282	2
LSAMP	0,010737013	-0,098757551	0,438	0,184	1	0,933836866	1,070850848	2,380434783
PILRB	0,101501297	-0,098854057	0,156	0,066	1	0,933774401	1,070922483	2,363636364
KLK6	0,815054348	-0,098930938	0,031	0,039	1	0,933724641	1,070979554	0,794871795
TXNDC5	0,006092904	-0,098937245	0,312	0,109	1	0,933720559	1,070984236	2,862385321
DOCK11	0,428872689	-0,098946656	0	0,02	1	0,933714469	1,070991222	0
PLEKHM1	0,815054348	-0,09907121	0,031	0,039	1	0,933633861	1,071083689	0,794871795
PHKA1	0,543117005	-0,099253898	0	0,012	1	0,933515642	1,071219329	0
DNHD1	0,480650464	-0,099299188	0	0,016	1	0,933486337	1,071252958	0
ANKRD20A1	0,428872689	-0,099352317	0	0,02	1	0,933451961	1,071292409	0
AC008914.1	0,480650464	-0,099370949	0	0,016	1	0,933439906	1,071306244	0

Supplementary Table 1

Differentially expressed genes_adjacent-skin-XL_ comparing SC-Keloid vs. (SC-Myel, SC-Nonmyel)								
gene	p_val	avg_log2FC	pct.1	pct.2	p_val_adj	Foldchange_UP	Foldchange_DOWN	Ratio_pct1_pct2
IL20RB	0,38481627	-0,099371378	0	0,023	1	0,933439628	1,071306563	0
AL627171.1	0,924392442	-0,099467357	0,031	0,027	1	0,933377531	1,071377837	1,148148148
PPP2R5C	0,003190249	-0,099555749	0,312	0,102	1	0,933320346	1,071443481	3,058823529
DHX8	0,093753684	-0,099625366	0,125	0,047	1	0,93327531	1,071495184	2,659574468
MED9	0,480650464	-0,099654783	0	0,016	1	0,93325628	1,071517032	0
PI4K2A	0,198962585	-0,099671604	0,125	0,059	1	0,933245399	1,071529526	2,118644068
SENP7	0,003256235	-0,099677127	0,375	0,133	1	0,933241826	1,071533628	2,819548872
CHSY1	0,062941189	-0,09977646	0,156	0,059	1	0,933177573	1,071607408	2,644067797
TCEAL1	0,003190249	-0,099791564	0,312	0,102	1	0,933167803	1,071618627	3,058823529
WASF3	0,428872689	-0,099799038	0	0,02	1	0,933162969	1,071624179	0
RBBP5	0,804543977	-0,099821222	0,031	0,039	1	0,93314862	1,071640657	0,794871795
DET1	0,428872689	-0,099823143	0	0,02	1	0,933147377	1,071642084	0
HR	0,257702735	-0,099842706	0,031	0,094	1	0,933134724	1,071656615	0,329787234
SEMA3E	0,543117005	-0,09986564	0	0,012	1	0,93311989	1,071673652	0
FRY	0,428872689	-0,099889503	0	0,02	1	0,933104456	1,071691378	0
ZNF790	0,682170546	-0,099967989	0,031	0,02	1	0,933053694	1,071749682	1,55
MTM1	0,663576959	-0,100079731	0,062	0,043	1	0,932981428	1,071832696	1,441860465
PATJ	0,313292814	-0,100080822	0	0,031	1	0,932980723	1,071833506	0
ATP11C	0,122084526	-0,100093118	0,125	0,051	1	0,932972771	1,071842642	2,450980392
C1RL-AS1	0,480650464	-0,100113062	0	0,016	1	0,932959874	1,071857459	0
NAP1L2	0,480650464	-0,100230049	0	0,016	1	0,932884224	1,071944379	0
PHF6	0,006461838	-0,100337463	0,188	0,051	1	0,93281477	1,072024192	3,68627451
HDGF	0,073207175	-0,100391318	0,531	0,27	1	0,932779949	1,072064211	1,966666667
WDR82	0,006785527	-0,100483796	0,344	0,125	1	0,932720159	1,072132933	2,752
PON3	0,80991663	-0,100549578	0,031	0,023	1	0,932677631	1,07218182	1,347826087
PARP12	0,746971464	-0,100567334	0,094	0,074	1	0,932666152	1,072195016	1,27027027
CEP170B	0,097375089	-0,100571186	0,094	0,031	1	0,932663662	1,072197879	3,032258065
TBKBP1	0,80991663	-0,100605155	0,031	0,023	1	0,932641702	1,072223125	1,347826087
CARD10	0,24851603	-0,100640577	0,094	0,043	1	0,932618804	1,072249451	2,186046512
PPARD	0,093753684	-0,100691473	0,125	0,047	1	0,932585903	1,072287279	2,659574468

Supplementary Table 1

Differentially expressed genes_adjacent-skin-XL_ comparing SC-Keloid vs. (SC-Myel, SC-Nonmyel)								
**gene**	**p_val**	**avg_log2FC**	**pct.1**	**pct.2**	**p_val_adj**	**Foldchange_UP**	**Foldchange_DOWN**	**Ratio_pct1_pct2**
OPA1	0,040359751	-0,100712204	0,219	0,086	1	0,932572502	1,072302687	2,546511628
CPTP	0,30793671	-0,100744719	0,062	0,027	1	0,932551484	1,072326855	2,296296296
FLRT3	0,026365081	-0,10077786	0,312	0,133	1	0,932530062	1,072351488	2,345864662
XPNPEP3	0,093753684	-0,100805151	0,125	0,047	1	0,932512422	1,072371774	2,659574468
ZNF718	0,480650464	-0,100807098	0	0,016	1	0,932511164	1,072373221	0
GABARAPL2	0,595233492	-0,100821627	0,844	0,555	1	0,932501773	1,07238402	1,520720721
LY96	0,682170546	-0,100890057	0,031	0,02	1	0,932457543	1,072434887	1,55
GPRC5A	0,480650464	-0,100894682	0	0,016	1	0,932454554	1,072438325	0
LDLRAD2	0,480650464	-0,101000507	0	0,016	1	0,932386159	1,072516994	0
DNAJC14	0,098751938	-0,101038934	0,094	0,031	1	0,932361325	1,072545561	3,032258065
DTNBP1	0,560566066	-0,101058435	0,062	0,039	1	0,932348721	1,072560059	1,58974359
AXL	0,012799595	-0,101060863	0,469	0,199	1	0,932347153	1,072561864	2,35678392
PLCD3	0,396289917	-0,10109051	0,062	0,031	1	0,932327993	1,072583905	2
ALKBH3	0,007871757	-0,101093839	0,156	0,039	1	0,932325842	1,07258638	4
BEGAIN	0,38481627	-0,101175657	0	0,023	1	0,93227297	1,07264721	0
TOLLIP	0,005793368	-0,101212122	0,375	0,141	1	0,932249406	1,072674322	2,659574468
ZXDB	0,682170546	-0,101261964	0,031	0,02	1	0,932217199	1,072711382	1,55
PCNT	0,003791564	-0,101297415	0,188	0,047	1	0,932194293	1,072737741	4
TINAGL1	0,480650464	-0,101341041	0	0,016	1	0,932166104	1,072770181	0
ADARB2	0,428872689	-0,101367098	0	0,02	1	0,932149268	1,072789557	0
ZNF793	0,226351275	-0,101373685	0,031	0,008	1	0,932145012	1,072794455	3,875
CRLF3	0,682170546	-0,101471518	0,031	0,02	1	0,932081803	1,072867206	1,55
EIF2AK2	0,014668447	-0,101501503	0,344	0,141	1	0,932062431	1,072889505	2,439716312
AKNA	0,224763613	-0,101526966	0,062	0,023	1	0,932045981	1,072908441	2,695652174
CIAO3	0,578217897	-0,101558526	0,062	0,039	1	0,932025592	1,072931912	1,58974359
MAGEE1	0,428872689	-0,101597856	0	0,02	1	0,932000184	1,072961162	0
FOXRED2	0,480650464	-0,101630761	0	0,016	1	0,931978927	1,072985634	0
PRDM5	0,141661018	-0,101743711	0,094	0,035	1	0,931905964	1,073069643	2,685714286
ARRB1	0,80991663	-0,101749768	0,031	0,023	1	0,931902051	1,073074148	1,347826087
PRXL2C	0,007018614	-0,101825566	0,5	0,203	1	0,931853092	1,073130528	2,463054187

Supplementary Table 1

Differentially expressed genes_adjacent-skin-XL_ comparing SC-Keloid vs. (SC-Myel, SC-Nonmyel)								
**gene**	**p_val**	**avg_log2FC**	**pct.1**	**pct.2**	**p_val_adj**	**Foldchange_UP**	**Foldchange_DOWN**	**Ratio_pct1_pct2**
SEH1L	0,20079827	-0,101847035	0,125	0,059	1	0,931839225	1,073146497	2,118644068
TTC9	0,480650464	-0,101883365	0	0,016	1	0,931815759	1,073173522	0
YRDC	0,487085433	-0,101885804	0,062	0,035	1	0,931814184	1,073175336	1,771428571
CERS4	0,284294126	-0,101997055	0,188	0,109	1	0,931742331	1,073258095	1,724770642
TNKS1BP1	0,062941189	-0,102005222	0,156	0,059	1	0,931737056	1,073264172	2,644067797
CPSF1	0,076632198	-0,102006178	0,156	0,062	1	0,931736439	1,073264882	2,516129032
SPSB2	0,487085433	-0,102089324	0,062	0,035	1	0,931682743	1,073326739	1,771428571
ZCCHC10	0,057807698	-0,10214782	0,188	0,074	1	0,931644967	1,073370259	2,540540541
CLBA1	0,013891036	-0,102176741	0,062	0,008	1	0,931626291	1,073391777	7,75
BRMS1L	0,376291412	-0,102197413	0,094	0,051	1	0,931612942	1,073407158	1,843137255
ZNF766	0,80991663	-0,102217847	0,031	0,023	1	0,931599747	1,073422361	1,347826087
ZNF611	0,38481627	-0,102239412	0	0,023	1	0,931585822	1,073438406	0
RNASE1	0,310355006	-0,102314649	0,094	0,047	1	0,93153724	1,073494388	2
PPP1R3D	0,428872689	-0,10239924	0	0,02	1	0,931482623	1,073557333	0
C16orf87	0,063895026	-0,102415705	0,25	0,113	1	0,931471992	1,073569585	2,212389381
CDH3	0,543117005	-0,10251885	0	0,012	1	0,931405399	1,073646342	0
APTR	0,638347525	-0,102584977	0,094	0,066	1	0,931362708	1,073695555	1,424242424
SEC31B	0,543117005	-0,102626908	0	0,012	1	0,931335639	1,073726762	0
PTPRF	0,000161906	-0,10265792	0,438	0,125	1	0,93131562	1,073749842	3,504
PPAN	0,578217897	-0,102673468	0,062	0,039	1	0,931305583	1,073761415	1,58974359
KLHDC8A	0,815054348	-0,10269925	0,031	0,039	1	0,93128894	1,073780603	0,794871795
MTSS1	0,38481627	-0,102714085	0	0,023	1	0,931279363	1,073791646	0
MAK	0,480650464	-0,102770726	0	0,016	1	0,931242802	1,073833804	0
CARMIL1	0,543117005	-0,102806827	0	0,012	1	0,9312195	1,073860675	0
SGMS2	0,741950345	-0,102810347	0,062	0,047	1	0,931217228	1,073863295	1,319148936
ZNF582-DT	0,428872689	-0,102935304	0	0,02	1	0,931136575	1,07395631	0
POLR1A	0,540537634	-0,102973446	0,031	0,016	1	0,931111958	1,073984704	1,9375
LRPPRC	0,008804476	-0,103044011	0,281	0,098	1	0,931066416	1,074037236	2,867346939
EVI5L	0,80991663	-0,10317868	0,031	0,023	1	0,930979509	1,074137497	1,347826087
ST6GALNAC2	0,38481627	-0,103189371	0	0,023	1	0,93097261	1,074145457	0

Supplementary Table 1

Differentially expressed genes_adjacent-skin-XL_ comparing SC-Keloid vs. (SC-Myel, SC-Nonmyel)								
**gene**	**p_val**	**avg_log2FC**	**pct.1**	**pct.2**	**p_val_adj**	**Foldchange_UP**	**Foldchange_DOWN**	**Ratio_pct1_pct2**
CR1	0,428872689	-0,103304449	0	0,02	1	0,930898353	1,074231141	0
CD14	0,543117005	-0,103383263	0	0,012	1	0,9308475	1,074289827	0
RAP1A	0,040061847	-0,103439287	0,625	0,301	1	0,930811353	1,074331546	2,07641196
ELP1	0,482846351	-0,103458145	0,062	0,035	1	0,930799187	1,074345589	1,771428571
PCDHB15	0,80991663	-0,103524077	0,031	0,023	1	0,930756649	1,074394688	1,347826087
DCLK3	0,38481627	-0,103589874	0	0,023	1	0,930714201	1,074443689	0
PLEKHF1	0,246047587	-0,103619537	0,094	0,043	1	0,930695065	1,074465781	2,186046512
ZNF566	0,346686972	-0,103700582	0	0,027	1	0,930642784	1,074526142	0
SLC25A10	0,018832425	-0,103711914	0,094	0,02	1	0,930635474	1,074534582	4,7
GSTM2	0,924392442	-0,103809563	0,031	0,027	1	0,930572486	1,074607314	1,148148148
BAHD1	0,907442332	-0,103838783	0,062	0,055	1	0,930553639	1,074629079	1,127272727
VPS13B	0,060102361	-0,103858125	0,156	0,059	1	0,930541163	1,074643487	2,644067797
ANKRD13B	0,185597364	-0,103966849	0,094	0,039	1	0,930471038	1,074724477	2,41025641
STK32C	0,000137099	-0,104085937	0,25	0,051	1	0,930394235	1,074813194	4,901960784
HEPACAM	0,346686972	-0,104090891	0	0,027	1	0,93039104	1,074816885	0
PTPRM	0,119445142	-0,104096002	0,125	0,051	1	0,930387745	1,074820693	2,450980392
MST1	0,38481627	-0,104245218	0	0,023	1	0,930291521	1,074931866	0
H3-2	0,388432899	-0,104298773	0,062	0,031	1	0,930256987	1,07497177	2
TMTC4	0,80991663	-0,104373144	0,031	0,023	1	0,930209034	1,075027186	1,347826087
TMEM216	0,428872689	-0,104441857	0	0,02	1	0,930164731	1,075078389	0
GALNT12	0,082317662	-0,10444613	0,031	0,004	1	0,930161976	1,075081573	7,75
ANKMY2	0,997020183	-0,104448704	0,031	0,031	1	0,930160316	1,075083491	1
ZNF197	0,396289917	-0,104483761	0,062	0,031	1	0,930137714	1,075109616	2
ZNF672	0,373239546	-0,104508661	0,094	0,051	1	0,930121661	1,075128171	1,843137255
TIMM9	0,01679085	-0,104579751	0,219	0,074	1	0,930075829	1,075181151	2,959459459
FRA10AC1	0,064651165	-0,104601639	0,219	0,094	1	0,930061718	1,075197463	2,329787234
RAB29	0,008148126	-0,104752299	0,094	0,016	1	0,929964598	1,075309751	5,875
PRH1	0,000493332	-0,10483339	0,25	0,059	1	0,929912327	1,075370194	4,237288136
NAAA	0,38481627	-0,10483856	0	0,023	1	0,929908995	1,075374047	0
DDX27	0,003195191	-0,104902133	0,281	0,086	1	0,929868019	1,075421435	3,26744186

Supplementary Table 1

Differentially expressed genes_adjacent-skin-XL_ comparing SC-Keloid vs. (SC-Myel, SC-Nonmyel)								
**gene**	**p_val**	**avg_log2FC**	**pct.1**	**pct.2**	**p_val_adj**	**Foldchange_UP**	**Foldchange_DOWN**	**Ratio_pct1_pct2**
ERCC5	0,015958056	-0,104916212	0,188	0,059	1	0,929858945	1,07543193	3,186440678
RNF220	0,001968647	-0,104916954	0,188	0,043	1	0,929858467	1,075432483	4,372093023
ITPK1-AS1	0,543117005	-0,104977497	0	0,012	1	0,929819446	1,075477615	0
CATSPER2	0,543117005	-0,105004116	0	0,012	1	0,92980229	1,075497459	0
MERTK	0,313292814	-0,105101641	0	0,031	1	0,929739438	1,075570164	0
TOLLIP-DT	0,428872689	-0,105225845	0	0,02	1	0,929659399	1,075662766	0
DUSP3	0,656644882	-0,105296892	0,125	0,094	1	0,929613618	1,075715739	1,329787234
FRMD5	0,006829528	-0,105354357	0,281	0,094	1	0,92957659	1,075758588	2,989361702
NHSL1	0,543117005	-0,10537675	0	0,012	1	0,929562162	1,075775285	0
KLF8	0,38481627	-0,105413913	0	0,023	1	0,929538218	1,075802997	0
ZKSCAN5	0,540537634	-0,105516832	0,031	0,016	1	0,929471908	1,075879745	1,9375
MB21D2	0,881459974	-0,105529295	0,062	0,066	1	0,929463879	1,07588904	0,939393939
ZZEF1	0,396289917	-0,105563746	0,062	0,031	1	0,929441684	1,075914732	2
ZNF449	0,428872689	-0,105597414	0	0,02	1	0,929419994	1,075939841	0
KPNA6	0,006920774	-0,10564716	0,312	0,109	1	0,929387947	1,075976941	2,862385321
CECR2	0,085298168	-0,105673803	0,219	0,098	1	0,929370783	1,075996812	2,234693878
LEAP2	0,428872689	-0,105756751	0	0,02	1	0,929317351	1,076058678	0
ATRN	0,108846454	-0,105767326	0,156	0,066	1	0,929310539	1,076066566	2,363636364
HNRNPM	0,025188216	-0,105916529	0,812	0,406	1	0,929214435	1,076177858	2
LRRK1	0,480650464	-0,10594639	0	0,016	1	0,929195202	1,076200132	0
RAB3IP	0,371729093	-0,105972365	0,125	0,074	1	0,929178473	1,076219509	1,689189189
TRERF1	0,543117005	-0,106065512	0	0,012	1	0,929118482	1,076288998	0
ANAPC10	0,079159031	-0,106112724	0,156	0,062	1	0,929088078	1,076324219	2,516129032
HELQ	0,979143607	-0,106187055	0,031	0,031	1	0,92904021	1,076379676	1
ZNF81	0,682170546	-0,106311632	0,031	0,02	1	0,928959991	1,076472625	1,55
MRPL4	0,022674894	-0,106385993	0,281	0,109	1	0,928912111	1,076528111	2,577981651
SULT1A2	0,480650464	-0,106416613	0	0,016	1	0,928892396	1,07655096	0
NTMT1	0,075260589	-0,106435367	0,344	0,168	1	0,928880321	1,076564954	2,047619048
TRAF3IP2-AS1	0,512685318	-0,106455978	0,094	0,059	1	0,92886705	1,076580335	1,593220339
ATP13A1	0,063925678	-0,106469124	0,094	0,027	1	0,928858587	1,076590145	3,481481481

Supplementary Table 1

Differentially expressed genes_adjacent-skin-XL_ comparing SC-Keloid vs. (SC-Myel, SC-Nonmyel)								
**gene**	**p_val**	**avg_log2FC**	**pct.1**	**pct.2**	**p_val_adj**	**Foldchange_UP**	**Foldchange_DOWN**	**Ratio_pct1_pct2**
CDC42EP2	0,540537634	-0,106517101	0,031	0,016	1	0,928827697	1,076625948	1,9375
LRRN1	0,979143607	-0,106574996	0,031	0,031	1	0,928790424	1,076669154	1
PTGER4	0,543117005	-0,106595289	0	0,012	1	0,92877736	1,076684298	0
ESF1	3,06659E-06	-0,106604796	0,531	0,137	0,064708125	0,92877124	1,076691393	3,875912409
GTF3C5	0,006461838	-0,106619986	0,188	0,051	1	0,928761461	1,076702729	3,68627451
ZNF442	0,428872689	-0,106638589	0	0,02	1	0,928749485	1,076716613	0
ALDH5A1	0,543117005	-0,106696225	0	0,012	1	0,928712382	1,076759629	0
CDK12	0,003481349	-0,106705941	0,344	0,117	1	0,928706128	1,076766881	2,94017094
CWF19L2	0,424410493	-0,106724856	0,344	0,23	1	0,928693952	1,076780998	1,495652174
ZNF451	0,083810509	-0,106931988	0,219	0,098	1	0,928560626	1,076935606	2,234693878
NOTCH2NLA	0,38481627	-0,106980178	0	0,023	1	0,92852961	1,076971579	0
MYO1B	0,487085433	-0,107029592	0,062	0,035	1	0,928497807	1,077008467	1,771428571
TMEM175	0,103558458	-0,107090063	0,156	0,066	1	0,928458889	1,077053612	2,363636364
CXCR4	0,924392442	-0,10709078	0,031	0,027	1	0,928458428	1,077054146	1,148148148
PTDSS2	0,010405093	-0,107129464	0,188	0,055	1	0,928433533	1,077083026	3,418181818
ST6GALNAC6	0,049283458	-0,107179039	0,219	0,09	1	0,92840163	1,077120039	2,433333333
CLUAP1	0,004823168	-0,107300681	0,219	0,062	1	0,928323354	1,077210861	3,532258065
TBCD	0,304810673	-0,107318971	0,094	0,047	1	0,928311586	1,077224517	2
MAP2	0,428872689	-0,107345235	0	0,02	1	0,928294686	1,077244128	0
C2orf69	0,682170546	-0,107347435	0,031	0,02	1	0,92829327	1,077245771	1,55
MFSD13A	0,38481627	-0,10749824	0	0,023	1	0,928196241	1,077358381	0
MICU3	0,001478724	-0,10749905	0,25	0,066	1	0,92819572	1,077358987	3,787878788
EHD1	0,002010541	-0,107508104	0,156	0,031	1	0,928189895	1,077365748	5,032258065
GAMT	0,001328266	-0,107547442	0,281	0,078	1	0,928164586	1,077395124	3,602564103
PCOLCE	0,097375089	-0,107603683	0,094	0,031	1	0,928128404	1,077437126	3,032258065
C1orf109	0,396289917	-0,107660133	0,062	0,031	1	0,928092089	1,077479285	2
RMND1	0,004087528	-0,107715293	0,125	0,023	1	0,928056605	1,077520482	5,434782609
CUL3	0,007287546	-0,107732796	0,406	0,156	1	0,928045346	1,077533555	2,602564103
FRMD4A	0,022295068	-0,107821527	0,594	0,285	1	0,927988269	1,077599829	2,084210526
HNRNPUL1	0,00432798	-0,107860137	0,5	0,203	1	0,927963434	1,077628668	2,463054187

Supplementary Table 1

Differentially expressed genes_adjacent-skin-XL_ comparing SC-Keloid vs. (SC-Myel, SC-Nonmyel)								
**gene**	**p_val**	**avg_log2FC**	**pct.1**	**pct.2**	**p_val_adj**	**Foldchange_UP**	**Foldchange_DOWN**	**Ratio_pct1_pct2**
PHAX	0,006037254	-0,107888927	0,281	0,094	1	0,927944916	1,077650173	2,989361702
COL9A1	0,480650464	-0,107989157	0	0,016	1	0,92788045	1,077725045	0
TENM4	0,428872689	-0,107997281	0	0,02	1	0,927875225	1,077731114	0
PLCG2	0,80991663	-0,108012149	0,031	0,023	1	0,927865663	1,07774222	1,347826087
ILDR2	0,80991663	-0,108049655	0,031	0,023	1	0,927841541	1,077770239	1,347826087
H2BC11	0,38481627	-0,108053628	0	0,023	1	0,927838986	1,077773207	0
HEMK1	0,396289917	-0,10808197	0,062	0,031	1	0,927820759	1,077794381	2
HDAC6	0,246047587	-0,108108868	0,094	0,043	1	0,92780346	1,077814476	2,186046512
TAF13	0,011597542	-0,108161617	0,219	0,07	1	0,927769538	1,077853884	3,128571429
TMEM178B	0,224763613	-0,108208094	0,062	0,023	1	0,92773965	1,077888608	2,695652174
VCL	0,02913176	-0,108274146	0,688	0,336	1	0,927697176	1,077937959	2,047619048
GATA3	0,543117005	-0,10827466	0	0,012	1	0,927696845	1,077938343	0
CUL4A	0,022242508	-0,108478428	0,219	0,078	1	0,927565825	1,078090604	2,807692308
PARP9	0,30757457	-0,108492743	0,094	0,047	1	0,927556622	1,0781013	2
PEDS1	0,016381033	-0,108499148	0,219	0,074	1	0,927552504	1,078106087	2,959459459
RADX	0,474428645	-0,108542185	0,062	0,035	1	0,927524834	1,078138249	1,771428571
TMEM19	0,191913299	-0,108545306	0,094	0,039	1	0,927522828	1,078140581	2,41025641
SPRING1	0,244454149	-0,10855475	0,156	0,082	1	0,927516756	1,078147639	1,902439024
SLC12A9	0,093753684	-0,108575246	0,125	0,047	1	0,927503579	1,078162956	2,659574468
TRIM21	0,30793671	-0,108610712	0,062	0,027	1	0,927480779	1,07818946	2,296296296
IQCB1	0,008956837	-0,108635714	0,125	0,027	1	0,927464705	1,078208146	4,62962963
LMBRD2	0,65904021	-0,108647107	0,062	0,043	1	0,927457381	1,07821666	1,441860465
C10orf90	0,396289917	-0,108670426	0,062	0,031	1	0,92744239	1,078234088	2
DCP1B	0,037506898	-0,108734496	0,094	0,023	1	0,927401204	1,078281973	4,086956522
P2RY11	0,480650464	-0,108752395	0	0,016	1	0,927389698	1,078295352	0
DENND4A	0,663576959	-0,108793748	0,062	0,043	1	0,927363116	1,07832626	1,441860465
FUNDC2	0,014652896	-0,108830597	0,531	0,234	1	0,927339429	1,078353803	2,269230769
LAPTM5	0,480650464	-0,108873822	0	0,016	1	0,927311645	1,078386112	0
HACE1	0,041848356	-0,108885841	0,062	0,012	1	0,92730392	1,078395096	5,166666667
KCTD10	0,487085433	-0,108887332	0,062	0,035	1	0,927302962	1,07839621	1,771428571

Supplementary Table 1

Differentially expressed genes_adjacent-skin-XL_ comparing SC-Keloid vs. (SC-Myel, SC-Nonmyel)								
**gene**	**p_val**	**avg_log2FC**	**pct.1**	**pct.2**	**p_val_adj**	**Foldchange_UP**	**Foldchange_DOWN**	**Ratio_pct1_pct2**
SLC2A10	0,386325862	-0,108887961	0,031	0,012	1	0,927302558	1,07839668	2,583333333
SIAE	0,480650464	-0,108919467	0	0,016	1	0,927282307	1,078420231	0
GLCCI1	0,30793671	-0,109043218	0,062	0,027	1	0,92720277	1,078512739	2,296296296
GPD2	0,241007682	-0,109084201	0,125	0,062	1	0,927176431	1,078543378	2,016129032
ANKRD29	0,682170546	-0,10924564	0,031	0,02	1	0,927072685	1,078664075	1,55
RHOF	0,65904021	-0,109248201	0,062	0,043	1	0,92707104	1,078665989	1,441860465
TMEM67	0,062941189	-0,109316404	0,156	0,059	1	0,927027214	1,078716984	2,644067797
CES2	0,396289917	-0,109505115	0,062	0,031	1	0,926905962	1,078858095	2
EIF4B	0,082312017	-0,109522073	0,5	0,258	1	0,926895067	1,078870776	1,937984496
WDR90	0,428872689	-0,109786307	0	0,02	1	0,926725319	1,079068392	0
ACOT2	0,138248252	-0,109801909	0,156	0,07	1	0,926715297	1,079080062	2,228571429
VPS54	0,045196862	-0,110042016	0,125	0,039	1	0,926561077	1,079259668	3,205128205
ZNF555	0,540537634	-0,11006842	0,031	0,016	1	0,926544119	1,07927942	1,9375
TBC1D10B	0,00257846	-0,11010962	0,25	0,07	1	0,92651766	1,079310242	3,571428571
MAP3K12	0,011905317	-0,110163069	0,219	0,07	1	0,926483335	1,07935023	3,128571429
RIPK1	0,063925678	-0,110164652	0,094	0,027	1	0,926482318	1,079351414	3,481481481
TBC1D2B	0,202646283	-0,110206707	0,125	0,059	1	0,926455311	1,079382878	2,118644068
TRMT5	0,38481627	-0,110304966	0	0,023	1	0,926392214	1,079456395	0
FAM193B	0,022371373	-0,110416826	0,25	0,094	1	0,926320389	1,079540094	2,659574468
NME7	0,18979066	-0,110474039	0,094	0,039	1	0,926283654	1,079582907	2,41025641
PKP4	0,746521062	-0,110511809	0,062	0,047	1	0,926259405	1,07961117	1,319148936
TANK	0,030205778	-0,110589187	0,375	0,164	1	0,926209727	1,079669076	2,286585366
MED6	0,204032042	-0,110611577	0,156	0,078	1	0,926195352	1,079685833	2
SMG8	0,428872689	-0,110666028	0	0,02	1	0,926160396	1,079726583	0
MARCHF3	0,887104347	-0,110788216	0,031	0,035	1	0,926081959	1,079818033	0,885714286
RGS10	0,009783331	-0,110795705	0,281	0,098	1	0,926077152	1,079823639	2,867346939
LINC00342	0,232905777	-0,110878989	0,125	0,062	1	0,926023693	1,079885977	2,016129032
FAM189A2	0,480650464	-0,110910434	0	0,016	1	0,926003509	1,079909515	0
UBE2O	0,38481627	-0,110937698	0	0,023	1	0,92598601	1,079929923	0
ATP10D	0,013407295	-0,111144188	0,156	0,043	1	0,925853485	1,080084502	3,627906977

Supplementary Table 1

Differentially expressed genes_adjacent-skin-XL_ comparing SC-Keloid vs. (SC-Myel, SC-Nonmyel)								
**gene**	**p_val**	**avg_log2FC**	**pct.1**	**pct.2**	**p_val_adj**	**Foldchange_UP**	**Foldchange_DOWN**	**Ratio_pct1_pct2**
ABTB2	0,924392442	-0,111183989	0,031	0,027	1	0,925827943	1,0801143	1,148148148
INSIG2	0,370203052	-0,11125504	0,094	0,051	1	0,925782348	1,080167495	1,843137255
TMEM74	0,38481627	-0,111274252	0	0,023	1	0,92577002	1,080181879	0
NAIF1	0,38481627	-0,111278437	0	0,023	1	0,925767334	1,080185013	0
TRIM25	0,924392442	-0,111291207	0,031	0,027	1	0,92575914	1,080194574	1,148148148
TPH1	0,480650464	-0,111318151	0	0,016	1	0,92574185	1,080214748	0
SLC29A1	0,924392442	-0,111413666	0,031	0,027	1	0,925680563	1,080286268	1,148148148
SPINT2	0,313292814	-0,111430399	0	0,031	1	0,925669827	1,080298797	0
ZNF562	0,569359326	-0,111438162	0,062	0,039	1	0,925664846	1,08030461	1,58974359
JUP	0,482008211	-0,111497447	0,125	0,082	1	0,925626808	1,080349004	1,524390244
EGFLAM	0,492171826	-0,111558904	0,094	0,059	1	0,925587378	1,080395027	1,593220339
ZMYM3	0,141661018	-0,111591867	0,094	0,035	1	0,92556623	1,080419712	2,685714286
DEFB1	0,924392442	-0,111593232	0,031	0,027	1	0,925565354	1,080420735	1,148148148
CPD	0,241007682	-0,111596763	0,125	0,062	1	0,925563089	1,080423378	2,016129032
OSR2	0,226351275	-0,111613638	0,031	0,008	1	0,925552263	1,080436016	3,875
LINC00842	0,480650464	-0,111732801	0	0,016	1	0,925475818	1,080525261	0
ARHGAP6	0,428872689	-0,111775822	0	0,02	1	0,925448221	1,080557483	0
ZNF621	0,422565177	-0,111777072	0,094	0,055	1	0,925447419	1,080558419	1,709090909
QTRT1	0,000497147	-0,111782775	0,438	0,137	1	0,92544376	1,080562691	3,197080292
PEX26	0,141661018	-0,11202258	0,094	0,035	1	0,925289946	1,080742317	2,685714286
DCAF12	0,003101546	-0,112031313	0,219	0,059	1	0,925284345	1,080748859	3,711864407
HOOK3	0,060183489	-0,112034116	0,438	0,223	1	0,925282547	1,080750959	1,964125561
DIS3	0,000890115	-0,112165988	0,25	0,062	1	0,925197974	1,080849751	4,032258065
TFDP2	0,00311028	-0,112179278	0,312	0,102	1	0,925189451	1,080859708	3,058823529
N4BP2	0,924392442	-0,112324516	0,031	0,027	1	0,925096316	1,080968525	1,148148148
DTWD1	0,001337371	-0,112379536	0,344	0,105	1	0,925061036	1,081009751	3,276190476
PMAIP1	0,100484991	-0,112463706	0,156	0,066	1	0,925007068	1,081072821	2,363636364
GOLGA6L10	0,80991663	-0,112656331	0,031	0,023	1	0,924883572	1,081217172	1,347826087
ZNF687	0,346686972	-0,112751696	0	0,027	1	0,924822437	1,081288646	0
ARHGAP10	0,058265316	-0,112835997	0,219	0,094	1	0,924768398	1,08135183	2,329787234

Supplementary Table 1

Differentially expressed genes_adjacent-skin-XL_ comparing SC-Keloid vs. (SC-Myel, SC-Nonmyel)								
**gene**	**p_val**	**avg_log2FC**	**pct.1**	**pct.2**	**p_val_adj**	**Foldchange_UP**	**Foldchange_DOWN**	**Ratio_pct1_pct2**
SLC10A7	0,313292064	-0,112934701	0	0,031	1	0,924705131	1,081425815	0
NYNRIN	0,30793671	-0,11299012	0,062	0,027	1	0,924669611	1,081467357	2,296296296
EVC2	0,428872689	-0,113043131	0	0,02	1	0,924635635	1,081507096	0
SORCS2	0,191913299	-0,113064425	0,094	0,039	1	0,924621987	1,081523059	2,41025641
TUFT1	0,008148126	-0,113155102	0,094	0,016	1	0,924563875	1,081591037	5,875
FBXO38	0,141661018	-0,113176934	0,094	0,035	1	0,924549883	1,081607405	2,685714286
ETV3	0,820034503	-0,11321077	0,094	0,078	1	0,9245282	1,081632773	1,205128205
FNIP1	0,066548207	-0,113212109	0,406	0,203	1	0,924527342	1,081633777	2
PEX6	0,304810673	-0,113219494	0,094	0,047	1	0,924522609	1,081639313	2
HSCB	0,663576959	-0,113226405	0,062	0,043	1	0,92451818	1,081644495	1,441860465
GBE1	0,001119353	-0,113271321	0,312	0,09	1	0,924489397	1,081678171	3,466666667
RNF185	0,396289917	-0,113290512	0,062	0,031	1	0,924477099	1,08169256	2
LYL1	0,037506898	-0,113295332	0,094	0,023	1	0,924474011	1,081696173	4,086956522
PRKAA1	0,005195348	-0,113317984	0,219	0,062	1	0,924459496	1,081713157	3,532258065
MAPK7	0,682170546	-0,113362375	0,031	0,02	1	0,924431051	1,081746442	1,55
SFXN5	0,160548057	-0,113457824	0,125	0,055	1	0,924369892	1,081818013	2,272727273
ZNF519	0,480650464	-0,113467875	0	0,016	1	0,924363452	1,08182555	0
SHMT1	0,898459048	-0,113547934	0,062	0,055	1	0,924312159	1,081885584	1,127272727
NAALADL2	0,033260329	-0,113584427	0,188	0,066	1	0,924288778	1,081912951	2,848484848
MTREX	0,054252457	-0,113610405	0,188	0,074	1	0,924272135	1,081932433	2,540540541
EFCAB7	0,396289917	-0,113729869	0,062	0,031	1	0,924195603	1,082022027	2
ATAD1	0,047008515	-0,113749116	0,312	0,137	1	0,924183273	1,082036463	2,277372263
CCDC174	0,123421204	-0,113841017	0,125	0,051	1	0,924124404	1,082105391	2,450980392
HPS1	0,061508217	-0,113867443	0,156	0,059	1	0,924107477	1,082125212	2,644067797
STK38	0,663576959	-0,113873026	0,062	0,043	1	0,924103901	1,082129401	1,441860465
MCOLN3	0,428872689	-0,113877658	0	0,02	1	0,924100934	1,082132875	0
HIF3A	0,38481627	-0,113999951	0	0,023	1	0,924022604	1,082224608	0
ZNF234	0,428872689	-0,114000386	0	0,02	1	0,924022325	1,082224934	0
XPA	0,008804476	-0,114011637	0,281	0,098	1	0,924015119	1,082233374	2,867346939
MBTD1	0,098751938	-0,114016416	0,094	0,031	1	0,924012059	1,082236959	3,032258065

Supplementary Table 1

Differentially expressed genes_adjacent-skin-XL_ comparing SC-Keloid vs. (SC-Myel, SC-Nonmyel)								
**gene**	**p_val**	**avg_log2FC**	**pct.1**	**pct.2**	**p_val_adj**	**Foldchange_UP**	**Foldchange_DOWN**	**Ratio_pct1_pct2**
PCBP2	0,163555638	-0,114061227	0,781	0,438	1	0,923983358	1,082270575	1,783105023
CINP	0,013407295	-0,114123197	0,156	0,043	1	0,92394367	1,082317064	3,627906977
OSBPL10	0,016858455	-0,114205787	0,125	0,031	1	0,923890778	1,082379025	4,032258065
IL17RC	0,351727998	-0,114288453	0,125	0,07	1	0,923837842	1,082441046	1,785714286
SLC40A1	0,346686972	-0,114314017	0	0,027	1	0,923821471	1,082460227	0
CEACAM1	0,480650464	-0,114319549	0	0,016	1	0,923817929	1,082464378	0
C1QTNF2	0,38481627	-0,114345307	0	0,023	1	0,923801436	1,082483704	0
MEIS2	0,480650464	-0,114525525	0	0,016	1	0,923686044	1,082618934	0
UCP2	0,80991663	-0,114577892	0,031	0,023	1	0,923652516	1,082658232	1,347826087
NMRK1	0,005831886	-0,11466612	0,25	0,078	1	0,923596032	1,082724444	3,205128205
MAP3K7	0,016999114	-0,114709106	0,219	0,074	1	0,923568513	1,082756705	2,959459459
TMEM97	0,746521062	-0,114765947	0,062	0,047	1	0,923532126	1,082799365	1,319148936
STXBP6	0,18979066	-0,114886925	0,094	0,039	1	0,923454686	1,082890168	2,41025641
ZNF431	0,060929624	-0,114939564	0,125	0,043	1	0,923420993	1,082929679	2,906976744
RAB11FIP2	0,064224632	-0,114950742	0,125	0,043	1	0,923413838	1,08293807	2,906976744
GATC	0,735253332	-0,114968819	0,031	0,043	1	0,923402268	1,08295164	0,720930233
ZNF429	0,346686972	-0,11502551	0	0,027	1	0,923365983	1,082994195	0
KRBA1	0,924392442	-0,115172721	0,031	0,027	1	0,923271769	1,083104708	1,148148148
CDIP1	0,681846175	-0,115355591	0,031	0,047	1	0,923154746	1,083242007	0,659574468
TEPP	0,543117005	-0,11535573	0	0,012	1	0,923154657	1,083242111	0
PCCA	0,098751938	-0,115413826	0,094	0,031	1	0,923117484	1,083285733	3,032258065
TMEM170B	0,38481627	-0,115532948	0	0,023	1	0,923041266	1,083375183	0
PNPO	0,924392442	-0,115615215	0,031	0,027	1	0,922988632	1,083436962	1,148148148
ARL6IP1	0,115222133	-0,115670187	0,625	0,352	1	0,922953464	1,083478246	1,775568182
ATP2B1-AS1	0,224763613	-0,115755251	0,062	0,023	1	0,922899046	1,083542132	2,695652174
OTULIN	0,085318957	-0,115780673	0,156	0,062	1	0,922882784	1,083561225	2,516129032
GATD1	0,552606916	-0,115782286	0,094	0,062	1	0,922881752	1,083562436	1,516129032
VPS51	0,022218422	-0,115782836	0,281	0,109	1	0,9228814	1,083562849	2,577981651
PJVK	0,540537634	-0,115787393	0,031	0,016	1	0,922878485	1,083566272	1,9375
MCFD2	0,006829551	-0,115799267	0,281	0,094	1	0,92287089	1,08357519	2,989361702

Supplementary Table 1

Differentially expressed genes_adjacent-skin-XL_ comparing SC-Keloid vs. (SC-Myel, SC-Nonmyel)								
**gene**	**p_val**	**avg_log2FC**	**pct.1**	**pct.2**	**p_val_adj**	**Foldchange_UP**	**Foldchange_DOWN**	**Ratio_pct1_pct2**
ZNF445	0,396289917	-0,115800833	0,062	0,031	1	0,922869888	1,083576366	2
TMEM263	0,010366655	-0,115814488	0,375	0,148	1	0,922861153	1,083586623	2,533783784
KIAA0040	0,979143607	-0,115837111	0,031	0,031	1	0,922846682	1,083603614	1
DENND5A	0,001278728	-0,115847382	0,406	0,133	1	0,922840111	1,083611329	3,052631579
ZNF600	0,682170546	-0,1159235	0,031	0,02	1	0,922791423	1,083668503	1,55
PARD6B	0,573780511	-0,115972582	0,062	0,039	1	0,922760029	1,083705371	1,58974359
FHIP2B	0,638347525	-0,116036213	0,094	0,066	1	0,922719332	1,083753169	1,424242424
DPY19L2	0,480650464	-0,11614588	0	0,016	1	0,922649193	1,083835554	0
ABCD1	0,480650464	-0,116182648	0	0,016	1	0,922625679	1,083863177	0
FAM81A	0,428872689	-0,116191312	0	0,02	1	0,922620138	1,083869687	0
MTBP	0,543117005	-0,116229897	0	0,012	1	0,922595463	1,083898675	0
CLYBL	0,80991663	-0,116249207	0,031	0,023	1	0,922583115	1,083913183	1,347826087
APPL1	0,002946987	-0,116270868	0,344	0,113	1	0,922569263	1,083929457	3,044247788
AHSA1	0,003069747	-0,116319462	0,656	0,266	1	0,922538188	1,083965967	2,466165414
ZNF713	0,682170546	-0,116334737	0,031	0,02	1	0,922528421	1,083977445	1,55
SMAD3	0,979143607	-0,116342302	0,031	0,031	1	0,922523583	1,083983129	1
ZNF521	0,38481627	-0,116356707	0	0,023	1	0,922514372	1,083993952	0
ENOX2	0,428872689	-0,116474087	0	0,02	1	0,922439318	1,084082151	0
OXSR1	0,051520095	-0,116599695	0,312	0,145	1	0,92235901	1,08417654	2,151724138
C4orf33	0,141661018	-0,116621122	0,094	0,035	1	0,922345311	1,084192643	2,685714286
SNX27	0,067298158	-0,116635156	0,25	0,113	1	0,922336339	1,084203189	2,212389381
KIFC2	0,985101745	-0,116673055	0,031	0,031	1	0,92231211	1,084231671	1
GPHN	0,979143607	-0,116779107	0,031	0,031	1	0,922244313	1,084311376	1
KIAA1671	0,578217897	-0,116883326	0,062	0,039	1	0,922177693	1,084389708	1,58974359
CSNK2A1	0,006146292	-0,11689552	0,344	0,125	1	0,922169899	1,084398874	2,752
CKS1B	0,122113558	-0,116909038	0,344	0,184	1	0,922161259	1,084409034	1,869565217
E2F3	0,540537634	-0,117054468	0,031	0,016	1	0,922068305	1,084518353	1,9375
SH3YL1	0,016962377	-0,117138905	0,25	0,09	1	0,922014341	1,084581829	2,777777778
NSG1	0,480650464	-0,117144221	0	0,016	1	0,922010943	1,084585825	0
PIK3C2B	0,38481627	-0,117215297	0	0,023	1	0,921965521	1,08463926	0

Supplementary Table 1

Differentially expressed genes_adjacent-skin-XL_ comparing SC-Keloid vs. (SC-Myel, SC-Nonmyel)								
**gene**	**p_val**	**avg_log2FC**	**pct.1**	**pct.2**	**p_val_adj**	**Foldchange_UP**	**Foldchange_DOWN**	**Ratio_pct1_pct2**
SLC38A7	0,063925678	-0,117227272	0,094	0,027	1	0,921957868	1,084648263	3,481481481
GGA2	0,677260831	-0,117253508	0,031	0,047	1	0,921941102	1,084667988	0,659574468
P2RX6	0,082317662	-0,117292893	0,031	0,004	1	0,921915933	1,0846976	7,75
RBM41	0,682170546	-0,117304367	0,031	0,02	1	0,921908601	1,084706227	1,55
PRTG	0,480650464	-0,117330467	0	0,016	1	0,921891923	1,08472585	0
HDHD3	0,346686972	-0,117371002	0	0,027	1	0,921866022	1,084756327	0
CNTROB	0,38454022	-0,117389162	0,062	0,031	1	0,921854417	1,084769982	2
PDCD4	0,021783242	-0,117409742	0,312	0,125	1	0,921841267	1,084785457	2,496
C21orf91	0,80991663	-0,117463919	0,031	0,023	1	0,921806651	1,084826194	1,347826087
ZNF211	0,428872689	-0,117476915	0	0,02	1	0,921798346	1,084835967	0
FBXO31	0,578217897	-0,117579607	0,062	0,039	1	0,921732734	1,084913189	1,58974359
BPNT1	0,30793671	-0,117583509	0,062	0,027	1	0,921730242	1,084916122	2,296296296
NIM1K	0,032866024	-0,117819988	0,188	0,066	1	0,921579169	1,085093971	2,848484848
DCLK2	0,979143607	-0,117829145	0,031	0,031	1	0,92157332	1,085100858	1
ZNF22-AS1	0,543117005	-0,117849731	0	0,012	1	0,92156017	1,085116342	0
TANGO6	0,540537634	-0,117896349	0,031	0,016	1	0,921530391	1,085151406	1,9375
SUGCT	0,543117005	-0,117923465	0	0,012	1	0,921513071	1,085171802	0
GNPAT	0,005452889	-0,117945466	0,25	0,078	1	0,921499018	1,085188351	3,205128205
MAN2B2	0,045196862	-0,118005634	0,125	0,039	1	0,921460587	1,08523361	3,205128205
PPP1R35	0,010105466	-0,118111279	0,312	0,113	1	0,921393114	1,085313082	2,761061947
ZNF25	0,195328071	-0,11813859	0,125	0,059	1	0,921375672	1,085333628	2,118644068
LOH12CR2	0,428872689	-0,118159554	0	0,02	1	0,921362283	1,085349399	0
PHYHIPL	0,428872689	-0,11816295	0	0,02	1	0,921360114	1,085351954	0
LIPE	0,80991663	-0,11817879	0,031	0,023	1	0,921349998	1,08536387	1,347826087
RRAS2	0,438682776	-0,118190735	0,094	0,055	1	0,92134237	1,085372857	1,709090909
SETD7	0,023853062	-0,118271408	0,219	0,078	1	0,921290852	1,08543355	2,807692308
TMEM259	0,107936107	-0,118284877	0,594	0,32	1	0,92128225	1,085443684	1,85625
MLKL	0,38481627	-0,118335107	0	0,023	1	0,921250175	1,085481476	0
LINC01023	0,38481627	-0,118336965	0	0,023	1	0,921248988	1,085482874	0
QRICH1	0,24851603	-0,118435876	0,094	0,043	1	0,92118583	1,085557298	2,186046512

Supplementary Table 1

Differentially expressed genes_adjacent-skin-XL_ comparing SC-Keloid vs. (SC-Myel, SC-Nonmyel)								
**gene**	**p_val**	**avg_log2FC**	**pct.1**	**pct.2**	**p_val_adj**	**Foldchange_UP**	**Foldchange_DOWN**	**Ratio_pct1_pct2**
INTS9	0,682170546	-0,118466724	0,031	0,02	1	0,921166133	1,085580509	1,55
NTRK2	0,985101745	-0,118482854	0,031	0,031	1	0,921155834	1,085592647	1
HCFC2	0,011905272	-0,11851401	0,219	0,07	1	0,921135941	1,085616091	3,128571429
IFITM1	0,487085433	-0,118573451	0,062	0,035	1	0,92109799	1,085660821	1,771428571
COA7	0,543117005	-0,118580426	0	0,012	1	0,921093537	1,08566607	0
ALG5	0,000404212	-0,118864989	0,438	0,137	1	0,920911875	1,085880232	3,197080292
SLC7A5	0,037506898	-0,118909748	0,094	0,023	1	0,920883304	1,085913922	4,086956522
ARHGAP31	0,299332476	-0,119051797	0,094	0,047	1	0,920792637	1,086020847	2
RSPH3	0,428872689	-0,119094643	0	0,02	1	0,920765292	1,0860531	0
CIDEB	0,80991663	-0,119105289	0,031	0,023	1	0,920758497	1,086061115	1,347826087
CCDC115	0,219587526	-0,11920021	0,188	0,102	1	0,920697918	1,086132574	1,843137255
IRX3	0,428872689	-0,119275123	0	0,02	1	0,920650112	1,086188974	0
AC159540.2	0,543117005	-0,119280186	0	0,012	1	0,920646881	1,086192786	0
N4BP1	0,032927619	-0,119297747	0,219	0,082	1	0,920635675	1,086206007	2,670731707
METRN	0,040081648	-0,119354099	0,781	0,402	1	0,920599715	1,086248435	1,94278607
EXD2	0,098751938	-0,119395348	0,094	0,031	1	0,920573394	1,086279494	3,032258065
DEPDC5	0,346686972	-0,119436121	0	0,027	1	0,920547377	1,086310194	0
LPAR6	0,441948175	-0,119458998	0,094	0,055	1	0,92053278	1,08632742	1,709090909
EIF1AD	0,063925678	-0,119544771	0,094	0,027	1	0,920478053	1,086392008	3,481481481
C21orf62-AS1	0,480650464	-0,119553241	0	0,016	1	0,920472649	1,086398386	0
KCNIP4	0,480650464	-0,119598708	0	0,016	1	0,92044364	1,086432625	0
AS3MT	0,38481627	-0,119625187	0	0,023	1	0,920426747	1,086452565	0
USPL1	0,020764757	-0,119794946	0,156	0,047	1	0,920318449	1,086580413	3,319148936
MON1B	0,24851603	-0,119799725	0,094	0,043	1	0,9203154	1,086584013	2,186046512
MCMDC2	0,543117005	-0,119920347	0	0,012	1	0,920238457	1,086674864	0
ALKBH1	0,979143607	-0,120182729	0,031	0,031	1	0,920071109	1,086872515	1
MYNN	0,396289917	-0,120218295	0,062	0,031	1	0,920048427	1,086899309	2
HDAC5	0,001493165	-0,120256861	0,312	0,094	1	0,920023833	1,086928365	3,319148936
VAMP7	0,055416701	-0,120328398	0,188	0,074	1	0,919978214	1,086982262	2,540540541
RNF138	0,187685368	-0,120387777	0,094	0,039	1	0,91994035	1,087027001	2,41025641

Supplementary Table 1

Differentially expressed genes_adjacent-skin-XL_ comparing SC-Keloid vs. (SC-Myel, SC-Nonmyel)								
**gene**	**p_val**	**avg_log2FC**	**pct.1**	**pct.2**	**p_val_adj**	**Foldchange_UP**	**Foldchange_DOWN**	**Ratio_pct1_pct2**
THAP9-AS1	0,230628924	-0,120422393	0,188	0,102	1	0,919918277	1,087053084	1,843137255
TTC39B	0,428872689	-0,120451372	0	0,02	1	0,919899799	1,087074919	0
UBE2B	0,112897195	-0,120497157	0,688	0,395	1	0,919870606	1,087109419	1,741772152
PDLIM2	0,070258445	-0,12052547	0,281	0,133	1	0,919852553	1,087130754	2,112781955
TMEM59	0,540894585	-0,120532358	0,75	0,52	1	0,919848162	1,087135944	1,442307692
PDZD8	0,111371471	-0,120552582	0,188	0,086	1	0,919835267	1,087151184	2,186046512
DNAJB12	0,007848695	-0,120614144	0,219	0,066	1	0,919796017	1,087197576	3,318181818
CPNE3	0,001139882	-0,120643322	0,562	0,203	1	0,919777415	1,087219564	2,768472906
SLC25A32	0,158954432	-0,120668874	0,125	0,055	1	0,919761125	1,08723882	2,272727273
ZBTB20-AS2	0,540537634	-0,120801605	0,031	0,016	1	0,919676508	1,087338853	1,9375
GAS1	0,346686972	-0,120927576	0	0,027	1	0,919596209	1,0874338	0
OSMR	0,30757457	-0,121018413	0,094	0,047	1	0,91953831	1,087502271	2
ATF5	0,013407295	-0,121100087	0,156	0,043	1	0,919486255	1,087563838	3,627906977
AXIN2	0,924392442	-0,12121891	0,031	0,027	1	0,919410527	1,087653415	1,148148148
ALKBH2	0,00187651	-0,121294877	0,219	0,055	1	0,919362115	1,087710689	3,981818182
TTLL3	0,792332287	-0,121327962	0,094	0,105	1	0,919341032	1,087735633	0,895238095
DSE	0,560566066	-0,121343188	0,062	0,039	1	0,91933133	1,087747113	1,58974359
FNTB	0,013407295	-0,121354487	0,156	0,043	1	0,919324129	1,087755633	3,627906977
VSIG8	0,480650464	-0,121488029	0	0,016	1	0,919239037	1,087856324	0
XPC	0,00434487	-0,121529297	0,312	0,105	1	0,919212743	1,087887443	2,971428571
CDC27	0,004889051	-0,121569171	0,281	0,09	1	0,919187338	1,08791751	3,122222222
FAM222A	0,907442332	-0,121586024	0,062	0,055	1	0,9191766	1,08793022	1,127272727
KAT6B	0,000123326	-0,121648937	0,375	0,098	1	0,919136517	1,087977663	3,826530612
ADGRL2	0,428872689	-0,121716975	0	0,02	1	0,919093172	1,088028973	0
RTTN	0,139907281	-0,121727166	0,094	0,035	1	0,91908668	1,088036659	2,685714286
ZNF654	0,445305691	-0,121729969	0,125	0,078	1	0,919084894	1,088038773	1,602564103
MTARC2	0,663576959	-0,121736029	0,062	0,043	1	0,919081033	1,088043344	1,441860465
FGFR3	0,682170546	-0,121892329	0,031	0,02	1	0,918981466	1,088161227	1,55
AKAP6	0,283789128	-0,121932164	0	0,035	1	0,918956092	1,088191273	0
LRSAM1	0,346686972	-0,121959139	0	0,027	1	0,91893891	1,08821162	0

Supplementary Table 1

Differentially expressed genes_adjacent-skin-XL_ comparing SC-Keloid vs. (SC-Myel, SC-Nonmyel)								
**gene**	**p_val**	**avg_log2FC**	**pct.1**	**pct.2**	**p_val_adj**	**Foldchange_UP**	**Foldchange_DOWN**	**Ratio_pct1_pct2**
NR1D2	0,013194542	-0,122094622	0,375	0,152	1	0,918852617	1,088313818	2,467105263
DNAJA3	0,578217897	-0,122128611	0,062	0,039	1	0,91883097	1,088339459	1,58974359
DCTN4	0,232905777	-0,122154821	0,125	0,062	1	0,918814277	1,088359232	2,016129032
ANAPC1	0,668126075	-0,122206162	0,062	0,043	1	0,91878158	1,088397963	1,441860465
ZPR1	0,195328071	-0,122226296	0,125	0,059	1	0,918768757	1,088413153	2,118644068
RUNX2	0,243596382	-0,122229492	0,094	0,043	1	0,918766722	1,088415564	2,186046512
C16orf74	0,38481627	-0,122353188	0	0,023	1	0,918687951	1,088508888	0
TSTD1	0,740158863	-0,12236091	0,031	0,043	1	0,918683034	1,088514715	0,720930233
DOP1A	0,24851603	-0,122365562	0,094	0,043	1	0,918680071	1,088518224	2,186046512
ZNF674-AS1	0,428872689	-0,122383314	0	0,02	1	0,918668767	1,088531619	0
LTO1	0,569358921	-0,122424833	0,062	0,039	1	0,918642329	1,088562946	1,58974359
GNPDA1	0,170843838	-0,122433197	0,188	0,094	1	0,918637004	1,088569257	2
AC083843.3	0,346686972	-0,122479225	0	0,027	1	0,918607695	1,088603987	0
GGA1	0,005754239	-0,122535607	0,25	0,078	1	0,918571797	1,088646531	3,205128205
LPXN	0,543117005	-0,122584689	0	0,012	1	0,918540546	1,088683569	0
WDR4	0,543117005	-0,122645729	0	0,012	1	0,918501684	1,088729632	0
RGS9	0,428970186	-0,122754854	0,094	0,055	1	0,918432211	1,088811987	1,709090909
PWWP2B	0,578217897	-0,122848245	0,062	0,039	1	0,91837276	1,088882471	1,58974359
GXYLT1	0,376291412	-0,122874543	0,094	0,051	1	0,918356019	1,08890232	1,843137255
VRK3	0,487085433	-0,122921593	0,062	0,035	1	0,91832607	1,088937833	1,771428571
MMACHC	0,543117005	-0,122925928	0	0,012	1	0,918323311	1,088941104	0
TRIM37	0,139907281	-0,122939763	0,094	0,035	1	0,918314504	1,088951547	2,685714286
PSMB4	0,000128916	-0,122950748	0,625	0,207	1	0,918307512	1,088959839	3,019323671
SGCD	0,355251835	-0,123045717	0,094	0,051	1	0,918247064	1,089031525	1,843137255
UNC45A	0,015105004	-0,123063978	0,188	0,059	1	0,918235441	1,08904531	3,186440678
TUBGCP2	0,000207816	-0,123077586	0,312	0,078	1	0,91822678	1,089055581	4
LRP3	0,313292814	-0,123148056	0	0,031	1	0,91818193	1,089108779	0
PLXNB1	0,892714086	-0,123167499	0,031	0,035	1	0,918169555	1,089123457	0,885714286
FGF14-AS2	0,487085433	-0,123172858	0,062	0,035	1	0,918166145	1,089127502	1,771428571
CHRDL1	0,346686972	-0,123254519	0	0,027	1	0,918114175	1,089189152	0

Supplementary Table 1

Differentially expressed genes_adjacent-skin-XL_ comparing SC-Keloid vs. (SC-Myel, SC-Nonmyel)								
**gene**	**p_val**	**avg_log2FC**	**pct.1**	**pct.2**	**p_val_adj**	**Foldchange_UP**	**Foldchange_DOWN**	**Ratio_pct1_pct2**
ALDH9A1	0,003355836	-0,123296513	0,312	0,102	1	0,918087451	1,089220857	3,058823529
C12orf4	0,06592651	-0,12337101	0,125	0,043	1	0,918040044	1,089277103	2,906976744
TMEM25	0,428872689	-0,123411148	0	0,02	1	0,918014504	1,089307408	0
CEP295	0,021379715	-0,123482298	0,156	0,047	1	0,917969231	1,089361132	3,319148936
AC016831.7	0,257548576	-0,123489013	0	0,039	1	0,917964958	1,089366202	0
RABEPK	0,007602859	-0,123507512	0,156	0,039	1	0,917953187	1,089380171	4
PUDP	0,346686972	-0,123512358	0	0,027	1	0,917950104	1,08938383	0
OASL	0,480650464	-0,123623289	0	0,016	1	0,917879524	1,089467598	0
AJUBA	0,428872689	-0,123646437	0	0,02	1	0,917864797	1,089485078	0
ADK	0,00397851	-0,123652699	0,531	0,219	1	0,917860813	1,089489807	2,424657534
MAPK8	0,400593325	-0,123721554	0,094	0,055	1	0,917817008	1,089541806	1,709090909
MBOAT1	0,996386478	-0,123796124	0,094	0,09	1	0,917769569	1,089598124	1,044444444
ALS2	0,124769281	-0,124072416	0,125	0,051	1	0,917593822	1,089806814	2,450980392
RNF4	0,563155042	-0,124095825	0,094	0,062	1	0,917578934	1,089824496	1,516129032
SULT1C4	0,80991663	-0,124164962	0,031	0,023	1	0,917534963	1,089876725	1,347826087
ZNF669	0,38481627	-0,124197263	0	0,023	1	0,91751442	1,089901127	0
TDRP	0,313292814	-0,124243662	0	0,031	1	0,917484912	1,08993618	0
FCHSD1	0,80991663	-0,12438498	0,031	0,023	1	0,917395045	1,090042949	1,347826087
ZBED3-AS1	0,38481627	-0,124402892	0	0,023	1	0,917383655	1,090056483	0
VWC2	0,38481627	-0,124423784	0	0,023	1	0,91737037	1,090072268	0
AQP11	0,346686972	-0,124620041	0	0,027	1	0,917245584	1,090220566	0
EXOC6B	0,24851603	-0,124737822	0,094	0,043	1	0,917170703	1,090309575	2,186046512
RING1	0,103889562	-0,124760179	0,219	0,102	1	0,91715649	1,090326472	2,147058824
GOLGA1	0,578217897	-0,124782889	0,062	0,039	1	0,917142053	1,090343635	1,58974359
ZNF346	0,887104347	-0,12478695	0,031	0,035	1	0,917139472	1,090346704	0,885714286
CLSTN2	0,346686972	-0,124971839	0	0,027	1	0,917021943	1,090486446	0
TRAPPC2	0,924392442	-0,124981805	0,031	0,027	1	0,917015609	1,090493979	1,148148148
FZD5	0,681846175	-0,125032733	0,031	0,047	1	0,916983238	1,090532475	0,659574468
SCNN1D	0,38481627	-0,125043051	0	0,023	1	0,916976679	1,090540275	0
MN1	0,887104347	-0,125059929	0,031	0,035	1	0,916965952	1,090553033	0,885714286

Supplementary Table 1

Differentially expressed genes_adjacent-skin-XL_ comparing SC-Keloid vs. (SC-Myel, SC-Nonmyel)								
**gene**	**p_val**	**avg_log2FC**	**pct.1**	**pct.2**	**p_val_adj**	**Foldchange_UP**	**Foldchange_DOWN**	**Ratio_pct1_pct2**
DNAJC24	0,171678436	-0,125093832	0,156	0,074	1	0,916944404	1,090578661	2,108108108
SYTL1	0,38481627	-0,12510871	0	0,023	1	0,916934947	1,090589908	0
PINX1	0,984608494	-0,125125762	0,062	0,059	1	0,91692411	1,090602798	1,050847458
ANKRD49	0,046495836	-0,125143372	0,156	0,055	1	0,916912918	1,09061611	2,836363636
CRTAC1	0,38481627	-0,125193202	0	0,023	1	0,916881249	1,09065378	0
CATSPERG	0,480650464	-0,125212304	0	0,016	1	0,916869109	1,090668221	0
ISG20L2	0,191913299	-0,125222815	0,094	0,039	1	0,916862429	1,090676167	2,41025641
COQ6	0,021726813	-0,125223475	0,188	0,062	1	0,91686201	1,090676666	3,032258065
CLIP3	0,300029356	-0,125226089	0,125	0,066	1	0,916860348	1,090678643	1,893939394
PHF20	0,129999673	-0,125250524	0,312	0,168	1	0,91684482	1,090697115	1,857142857
KDM3B	0,613016608	-0,125363705	0,094	0,066	1	0,916772895	1,090782685	1,424242424
SKIL	0,002392549	-0,125434022	0,531	0,207	1	0,916728212	1,090835851	2,565217391
C6orf136	0,224763613	-0,125545598	0,062	0,023	1	0,916657317	1,090920218	2,695652174
NAP1L3	0,924392442	-0,125595269	0,031	0,027	1	0,916625757	1,090957779	1,148148148
ATG10	0,124769281	-0,125599803	0,125	0,051	1	0,916622876	1,090961207	2,450980392
MAGI3	0,730358576	-0,125673599	0,031	0,043	1	0,916575991	1,091017013	0,720930233
SLC16A12	0,480650464	-0,125680562	0	0,016	1	0,916571567	1,091022279	0
SCN5A	0,38481627	-0,125691635	0	0,023	1	0,916564532	1,091030653	0
MPPE1	0,06440165	-0,12577736	0,156	0,059	1	0,916510072	1,091095483	2,644067797
RP9	0,031413167	-0,125823235	0,156	0,051	1	0,916480929	1,091130179	3,058823529
SNX29	0,016858455	-0,125853219	0,125	0,031	1	0,916461881	1,091152857	4,032258065
PLAGL2	0,428872689	-0,125886662	0	0,02	1	0,916440637	1,091178151	0
APC2	0,428872689	-0,125889463	0	0,02	1	0,916438858	1,091180269	0
SNURF	0,428872689	-0,12600173	0	0,02	1	0,916367546	1,091265186	0
SLC12A6	0,979143607	-0,1260087	0,031	0,031	1	0,916363119	1,091270458	1
PTGR2	0,578217897	-0,126013066	0,062	0,039	1	0,916360346	1,09127376	1,58974359
EXOC4	0,021379715	-0,12604142	0,156	0,047	1	0,916342336	1,091295208	3,319148936
CCDC102B	0,924392442	-0,126117193	0,031	0,027	1	0,916294209	1,091352526	1,148148148
UPF1	0,138620038	-0,126159056	0,188	0,09	1	0,916267621	1,091384195	2,088888889
CHEK2	0,80991663	-0,126172746	0,031	0,023	1	0,916258927	1,091394551	1,347826087

Supplementary Table 1

Differentially expressed genes_adjacent-skin-XL_ comparing SC-Keloid vs. (SC-Myel, SC-Nonmyel)								
**gene**	**p_val**	**avg_log2FC**	**pct.1**	**pct.2**	**p_val_adj**	**Foldchange_UP**	**Foldchange_DOWN**	**Ratio_pct1_pct2**
TNFRSF1A	0,028631721	-0,126214334	0,406	0,184	1	0,916232515	1,091426012	2,206521739
ZNF618	0,38481627	-0,126299904	0	0,023	1	0,916178172	1,09149075	0
ST8SIA1	0,428872689	-0,126304939	0	0,02	1	0,916174974	1,091494559	0
RBM45	0,313292814	-0,126353228	0	0,031	1	0,916144309	1,091531094	0
AP4S1	0,346686972	-0,126353633	0	0,027	1	0,916144052	1,0915314	0
RBMS2	0,062854872	-0,126363231	0,219	0,094	1	0,916137958	1,091538662	2,329787234
S100A8	0,38481627	-0,126365021	0	0,023	1	0,916136821	1,091540016	0
MED30	0,003355836	-0,126491824	0,312	0,102	1	0,916056302	1,091635959	3,058823529
STAMBP	0,009014103	-0,126504182	0,281	0,098	1	0,916048455	1,091645311	2,867346939
BLOC1S6	0,033217489	-0,126543343	0,469	0,219	1	0,91602359	1,091674943	2,141552511
PC	0,672687415	-0,126563424	0,031	0,047	1	0,91601084	1,091690138	0,659574468
PRKAG2	0,139907281	-0,126586711	0,094	0,035	1	0,915996054	1,091707759	2,685714286
PRKN	0,38481627	-0,126590155	0	0,023	1	0,915993867	1,091710366	0
SMO	0,682170546	-0,126617064	0,031	0,02	1	0,915976783	1,091730728	1,55
DCST2	0,480650464	-0,126619382	0	0,016	1	0,915975311	1,091732482	0
HLCS	0,480650464	-0,126736319	0	0,016	1	0,91590107	1,091820976	0
NUPR1	0,123421204	-0,126909372	0,125	0,051	1	0,915791213	1,091951949	2,450980392
SLC25A45	0,428872689	-0,126911191	0	0,02	1	0,915790059	1,091953325	0
ATF6	0,003827364	-0,126915235	0,344	0,117	1	0,915787491	1,091956387	2,94017094
TGIF2	0,605861551	-0,12694965	0,094	0,066	1	0,915765646	1,091982435	1,424242424
C9orf50	0,396289917	-0,126974253	0,062	0,031	1	0,915750029	1,092001057	2
CACNA1A	0,346686972	-0,126976992	0	0,027	1	0,91574829	1,092003131	0
RAB11FIP5	0,310355006	-0,127005767	0,094	0,047	1	0,915730026	1,092024911	2
PBX3	0,002617214	-0,127136755	0,25	0,07	1	0,915646887	1,092124065	3,571428571
COX19	0,329441973	-0,127152114	0,125	0,07	1	0,915637139	1,092135691	1,785714286
TPST1	0,012061903	-0,127191826	0,219	0,07	1	0,915611935	1,092165754	3,128571429
TLNRD1	0,02386822	-0,127243249	0,25	0,094	1	0,9155793	1,092204684	2,659574468
KCTD18	0,313292814	-0,127257621	0	0,031	1	0,915570179	1,092215564	0
TOE1	0,150155729	-0,127294681	0,062	0,02	1	0,91554666	1,092243622	3,1
GTF2IRD1	0,06592651	-0,127367883	0,125	0,043	1	0,915500207	1,092299043	2,906976744

Supplementary Table 1

Differentially expressed genes_adjacent-skin-XL_ comparing SC-Keloid vs. (SC-Myel, SC-Nonmyel)								
**gene**	**p_val**	**avg_log2FC**	**pct.1**	**pct.2**	**p_val_adj**	**Foldchange_UP**	**Foldchange_DOWN**	**Ratio_pct1_pct2**
BLOC1S3	0,887104347	-0,127406872	0,031	0,035	1	0,915475466	1,092328563	0,885714286
MAD2L1BP	0,016858455	-0,127496027	0,125	0,031	1	0,915418893	1,092396069	4,032258065
TCEA3	0,887104347	-0,127612222	0,031	0,035	1	0,915345168	1,092484054	0,885714286
SRPK3	0,682170546	-0,12771703	0,031	0,02	1	0,915278673	1,092563423	1,55
CRISPLD1	0,346686972	-0,127720165	0	0,027	1	0,915276684	1,092565797	0
ATP6V0C	0,361013281	-0,127741703	0,844	0,543	1	0,91526302	1,092582108	1,554327808
PWP2	0,480650464	-0,127762009	0	0,016	1	0,915250138	1,092597486	0
WWOX	0,191913299	-0,12777838	0,094	0,039	1	0,915239752	1,092609885	2,41025641
DVL1	0,053678031	-0,127833838	0,188	0,074	1	0,91520457	1,092651886	2,540540541
DIPK2A	0,122084526	-0,127860905	0,125	0,051	1	0,9151874	1,092672386	2,450980392
DNMBP	0,38481627	-0,127869612	0	0,023	1	0,915181876	1,092678981	0
ARNT	0,163771487	-0,127870346	0,125	0,055	1	0,915181411	1,092679537	2,272727273
TANC2	0,170423334	-0,127951696	0,25	0,137	1	0,915129808	1,092741151	1,824817518
PTER	0,346686972	-0,127984122	0	0,027	1	0,91510924	1,092765712	0
MPP3	0,38481627	-0,128000963	0	0,023	1	0,915098557	1,092778469	0
AC123768.4	0,543117005	-0,128114352	0	0,012	1	0,915026638	1,092864359	0
RAB5B	0,059034985	-0,128122138	0,188	0,074	1	0,9150217	1,092870257	2,540540541
ADAM17	0,016947037	-0,128194263	0,5	0,223	1	0,914975956	1,092924895	2,242152466
OSGIN1	0,38481627	-0,128278817	0	0,023	1	0,914922332	1,092988951	0
ROBO3	0,032917376	-0,128398627	0,281	0,117	1	0,914846355	1,093079723	2,401709402
TRIM27	0,206379422	-0,128568646	0,125	0,059	1	0,914738548	1,093208548	2,118644068
AAAS	0,157372807	-0,128592373	0,125	0,055	1	0,914723504	1,093226528	2,272727273
IL6	0,924392442	-0,128653413	0,031	0,027	1	0,914684803	1,093272782	1,148148148
NOP14-AS1	0,346686972	-0,128726882	0	0,027	1	0,914638224	1,093328459	0
RNF115	0,020832552	-0,12878678	0,344	0,141	1	0,914600251	1,093373853	2,439716312
EGOT	0,087959196	-0,12885948	0,062	0,016	1	0,914554164	1,093428951	3,875
MRPS14	0,01032863	-0,128918462	0,312	0,113	1	0,914516775	1,093473655	2,761061947
LIN54	0,804543977	-0,129052153	0,031	0,039	1	0,914432032	1,09357499	0,794871795
ZBTB24	0,150155729	-0,129162656	0,062	0,02	1	0,914361995	1,093658754	3,1
IPMK	0,313292814	-0,129196904	0	0,031	1	0,914340289	1,093684717	0

Supplementary Table 1

Differentially expressed genes_adjacent-skin-XL_ comparing SC-Keloid vs. (SC-Myel, SC-Nonmyel)								
**gene**	**p_val**	**avg_log2FC**	**pct.1**	**pct.2**	**p_val_adj**	**Foldchange_UP**	**Foldchange_DOWN**	**Ratio_pct1_pct2**
TAF1C	0,735253332	-0,129199503	0,031	0,043	1	0,914338641	1,093686688	0,720930233
NR3C2	0,38481627	-0,1292127	0	0,023	1	0,914330278	1,093696692	0
MALRD1	0,480650464	-0,129293733	0	0,016	1	0,914278923	1,093758124	0
FTX	0,400032689	-0,129297482	0,219	0,145	1	0,914276547	1,093760967	1,510344828
TRRAP	0,082633113	-0,129414293	0,156	0,062	1	0,914202524	1,093849529	2,516129032
TRAPPC11	0,30793671	-0,129443083	0,062	0,027	1	0,914184281	1,093871357	2,296296296
SLC44A3	0,682170546	-0,12947356	0,031	0,02	1	0,914164968	1,093894466	1,55
YJU2B	0,00036468	-0,12947428	0,344	0,094	1	0,914164512	1,093895012	3,659574468
TNK1	0,80991663	-0,129532077	0,031	0,023	1	0,91412789	1,093938836	1,347826087
RBM12	0,435431286	-0,129600026	0,094	0,055	1	0,914084837	1,09399036	1,709090909
CCDC153	0,480650464	-0,129604137	0	0,016	1	0,914082232	1,093993477	0
VOPP1	0,157372807	-0,129629201	0,125	0,055	1	0,914066352	1,094012484	2,272727273
CCNE2	0,346686972	-0,129649233	0	0,027	1	0,91405366	1,094027674	0
SMAD4	0,046142047	-0,129686845	0,344	0,16	1	0,91402983	1,094056197	2,15
POMZP3	0,428872689	-0,129733519	0	0,02	1	0,91400026	1,094091592	0
TPCN2	0,123421204	-0,130053617	0,125	0,051	1	0,913797489	1,094334371	2,450980392
SART1	0,348388786	-0,130195639	0,156	0,094	1	0,913707537	1,094442105	1,659574468
MFSD10	0,068218414	-0,130275713	0,469	0,238	1	0,913656825	1,094502851	1,970588235
TCERG1	0,062955122	-0,130385006	0,281	0,129	1	0,913587612	1,09458577	2,178294574
SMARCD3	0,351727998	-0,130443503	0,125	0,07	1	0,91355057	1,094630153	1,785714286
RABL3	0,540537634	-0,130559647	0,031	0,016	1	0,913477027	1,09471828	1,9375
CCDC7	0,918113127	-0,13057358	0,031	0,027	1	0,913468205	1,094728852	1,148148148
TBPL1	0,032475728	-0,130621314	0,188	0,066	1	0,913437982	1,094765074	2,848484848
CDC42EP4	0,023800873	-0,130641266	0,375	0,16	1	0,91342535	1,094780214	2,34375
AMOT	0,313292814	-0,13065278	0	0,031	1	0,91341806	1,094788952	0
OPCML	0,38481627	-0,130658331	0	0,023	1	0,913414546	1,094793164	0
MTHFSD	0,150155729	-0,130689734	0,062	0,02	1	0,913394663	1,094816994	3,1
CSTF2T	0,480650464	-0,130746392	0	0,016	1	0,913358793	1,094859991	0
MOSPD2	0,395713974	-0,130758238	0,156	0,098	1	0,913351293	1,094868981	1,591836735
HAS2	0,014442227	-0,13078925	0,281	0,113	1	0,913331661	1,094892516	2,486725664

Supplementary Table 1

Differentially expressed genes_adjacent-skin-XL_ comparing SC-Keloid vs. (SC-Myel, SC-Nonmyel)								
**gene**	**p_val**	**avg_log2FC**	**pct.1**	**pct.2**	**p_val_adj**	**Foldchange_UP**	**Foldchange_DOWN**	**Ratio_pct1_pct2**
TMEM8B	0,045653963	-0,130796574	0,188	0,07	1	0,913327024	1,094898074	2,685714286
PAN2	0,799224988	-0,130827064	0,062	0,051	1	0,913307722	1,094921215	1,215686275
PPP1R13L	0,979143607	-0,130875551	0,031	0,031	1	0,913277027	1,094958014	1
JAKMIP2	0,141661018	-0,130927298	0,094	0,035	1	0,91324427	1,094997289	2,685714286
RECQL5	0,924392442	-0,130970895	0,031	0,027	1	0,913216673	1,09503038	1,148148148
PEX5L	0,38481627	-0,131102976	0	0,023	1	0,91313307	1,095130636	0
AC002451.1	0,428872689	-0,131104058	0	0,02	1	0,913132386	1,095131457	0
CTNS	0,06592651	-0,131113075	0,125	0,043	1	0,913126679	1,095138302	2,906976744
PUM2	0,003195191	-0,131126896	0,281	0,086	1	0,913117931	1,095148793	3,26744186
CREB3L4	0,682170546	-0,131154227	0,031	0,02	1	0,913100633	1,09516954	1,55
RHOG	0,00640202	-0,131162115	0,438	0,168	1	0,91309564	1,095175528	2,607142857
DCAF17	0,346686972	-0,131332892	0	0,027	1	0,91298756	1,095305176	0
TRPC1	0,804543977	-0,131475079	0,031	0,039	1	0,912897584	1,09541313	0,794871795
KPNB1	0,020415783	-0,131491575	0,531	0,242	1	0,912887145	1,095425656	2,194214876
ZNF461	0,540537634	-0,131540165	0,031	0,016	1	0,9128564	1,095462551	1,9375
ZNF486	0,38481627	-0,131577702	0	0,023	1	0,912832649	1,095491053	0
ERCC6	0,80991663	-0,131613986	0,031	0,023	1	0,912809691	1,095518605	1,347826087
EPB41L5	0,924392442	-0,131622576	0,031	0,027	1	0,912804257	1,095525128	1,148148148
WHRN	0,482846351	-0,131709021	0,062	0,035	1	0,912749564	1,095590773	1,771428571
RSL1D1	0,002393139	-0,131735371	0,438	0,152	1	0,912732893	1,095610783	2,881578947
EXT2	0,021654043	-0,131805892	0,25	0,094	1	0,912688278	1,09566434	2,659574468
NBPF11	0,80991663	-0,131810378	0,031	0,023	1	0,91268544	1,095667747	1,347826087
ZNF460	0,304810673	-0,131822319	0,094	0,047	1	0,912677887	1,095676815	2
SGSM2	0,30793671	-0,13184139	0,062	0,027	1	0,912665822	1,095691299	2,296296296
ACCS	0,224763613	-0,131858654	0,062	0,023	1	0,9126549	1,095704411	2,695652174
ANXA3	0,349205649	-0,131875587	0,125	0,07	1	0,912644188	1,095717272	1,785714286
ZNF700	0,924392442	-0,131965899	0,031	0,027	1	0,912587059	1,095785865	1,148148148
ZNF605	0,80991663	-0,132051343	0,031	0,023	1	0,912533012	1,095850765	1,347826087
ATG2A	0,663576959	-0,132157974	0,062	0,043	1	0,912465569	1,095931763	1,441860465
DBT	0,916437471	-0,132202216	0,062	0,055	1	0,912437587	1,095965372	1,127272727

Supplementary Table 1

Differentially expressed genes_adjacent-skin-XL_ comparing SC-Keloid vs. (SC-Myel, SC-Nonmyel)								
**gene**	**p_val**	**avg_log2FC**	**pct.1**	**pct.2**	**p_val_adj**	**Foldchange_UP**	**Foldchange_DOWN**	**Ratio_pct1_pct2**
CDC42BPA	0,014364265	-0,132210958	0,375	0,152	1	0,912432059	1,095972013	2,467105263
STK39	0,730358576	-0,132284787	0,031	0,043	1	0,912385367	1,0960281	0,720930233
PRC1	0,304406192	-0,132334537	0,062	0,027	1	0,912353904	1,096065896	2,296296296
PCDHGA10	0,396289917	-0,132358341	0,062	0,031	1	0,912338851	1,096083981	2
ALG11	0,224763613	-0,132450958	0,062	0,023	1	0,912280283	1,096154349	2,695652174
NACC2	0,264732918	-0,132550395	0,156	0,086	1	0,912217407	1,096229904	1,813953488
ATG4A	0,396289917	-0,132559695	0,062	0,031	1	0,912211527	1,09623697	2
UHRF1BP1L	0,632215601	-0,132620382	0,125	0,09	1	0,912173155	1,096283084	1,388888889
SACM1L	0,24851603	-0,13267781	0,094	0,043	1	0,912136846	1,096326724	2,186046512
TCP11L2	0,480650464	-0,132735903	0	0,016	1	0,912100118	1,096370871	0
DHX37	0,979143607	-0,132771973	0,031	0,031	1	0,912077314	1,096398282	1
WDR35	0,487085433	-0,132793351	0,062	0,035	1	0,912063798	1,096414529	1,771428571
ECT2	0,089393291	-0,132889919	0,125	0,047	1	0,912002751	1,096487921	2,659574468
BRWD3	0,032569978	-0,132952734	0,219	0,082	1	0,911963043	1,096535663	2,670731707
LGALSL	0,741950345	-0,132969683	0,062	0,047	1	0,911952329	1,096548545	1,319148936
MTO1	0,098751938	-0,132984308	0,094	0,031	1	0,911943084	1,096559662	3,032258065
PPM1A	0,008258336	-0,133205146	0,375	0,141	1	0,911803501	1,096727528	2,659574468
MAN1A1	0,924392442	-0,13329325	0,031	0,027	1	0,91174782	1,096794506	1,148148148
HDGFL2	0,011438702	-0,133340562	0,281	0,102	1	0,911717921	1,096830475	2,754901961
RGL1	0,979143607	-0,133535321	0,031	0,031	1	0,91159485	1,096978554	1
FBXO30	0,237189349	-0,133573772	0,156	0,082	1	0,911570554	1,097007791	1,902439024
WDR17	0,313292814	-0,13361724	0	0,031	1	0,911543089	1,097040844	0
TULP4	0,052408043	-0,133676709	0,281	0,125	1	0,911505516	1,097086066	2,248
MAPK6	0,016558745	-0,133762121	0,406	0,172	1	0,911451553	1,097151019	2,360465116
SH2B1	0,505798338	-0,133836403	0,094	0,059	1	0,911404625	1,097207511	1,593220339
ZNF318	0,38481627	-0,134007957	0	0,023	1	0,911296255	1,09733799	0
CSRNP2	0,920939129	-0,134049279	0,062	0,055	1	0,911270153	1,09736942	1,127272727
UBQLN2	0,016391618	-0,134049625	0,25	0,09	1	0,911269935	1,097369684	2,777777778
PRRC2C	0,048544154	-0,134207047	0,75	0,387	1	0,911170505	1,097489431	1,937984496
NTN4	0,304810673	-0,134237386	0,094	0,047	1	0,911151344	1,097512511	2

Supplementary Table 1

Differentially expressed genes_adjacent-skin-XL_ comparing SC-Keloid vs. (SC-Myel, SC-Nonmyel)								
**gene**	**p_val**	**avg_log2FC**	**pct.1**	**pct.2**	**p_val_adj**	**Foldchange_UP**	**Foldchange_DOWN**	**Ratio_pct1_pct2**
SLC22A4	0,682170546	-0,134240079	0,031	0,02	1	0,911149643	1,09751456	1,55
ETV7	0,224763613	-0,134412481	0,062	0,023	1	0,911040767	1,097645721	2,695652174
PAAF1	0,067739324	-0,134640749	0,219	0,094	1	0,910896631	1,097819408	2,329787234
RASA4	0,428872689	-0,134868658	0	0,02	1	0,910752744	1,097992848	0
MSANTD2	0,924392442	-0,134874881	0,031	0,027	1	0,910748816	1,097997585	1,148148148
CHD3	7,38464E-05	-0,134901571	0,438	0,121	1	0,910731967	1,098017898	3,619834711
GORASP2	0,000741516	-0,134912812	0,375	0,113	1	0,910724871	1,098026453	3,318584071
CDC16	0,00295717	-0,134930795	0,219	0,059	1	0,910713519	1,09804014	3,711864407
TAF4	0,38481627	-0,134942026	0	0,023	1	0,910706429	1,098048689	0
L2HGDH	0,38481627	-0,134956046	0	0,023	1	0,910697579	1,098059359	0
THBD	0,346686972	-0,135051233	0	0,027	1	0,910637495	1,09813181	0
ZNF524	0,017209641	-0,135087221	0,219	0,074	1	0,910614779	1,098159203	2,959459459
CSNK2A2	0,015052808	-0,135173891	0,375	0,152	1	0,910560075	1,098225177	2,467105263
SSTR1	0,428872689	-0,135184267	0	0,02	1	0,910553526	1,098233076	0
CFH	0,428872689	-0,135199821	0	0,02	1	0,91054371	1,098244916	0
CBLL1	0,283789128	-0,135212655	0	0,035	1	0,910535609	1,098254686	0
EPHA10	0,38481627	-0,135214403	0	0,023	1	0,910534506	1,098256017	0
NAXD	0,376291412	-0,135230142	0,094	0,051	1	0,910524573	1,098267998	1,843137255
UMAD1	0,13644998	-0,135242326	0,094	0,035	1	0,910516883	1,098277273	2,685714286
LINC00324	0,682170546	-0,135349282	0,031	0,02	1	0,910449383	1,098358699	1,55
CCDC71L	0,023035563	-0,135351304	0,219	0,078	1	0,910448107	1,098360238	2,807692308
CUEDC2	0,002518612	-0,135355242	0,562	0,219	1	0,910445623	1,098363236	2,566210046
ADNP	0,042949063	-0,135364156	0,375	0,176	1	0,910439997	1,098370022	2,130681818
SMN1	0,979143607	-0,135368785	0,031	0,031	1	0,910437076	1,098373547	1
ACER3	0,440039837	-0,135381358	0,156	0,102	1	0,910429141	1,098383119	1,529411765
ASRGL1	0,730358576	-0,135419919	0,031	0,043	1	0,910404808	1,098412477	0,720930233
SPIDR	0,124769281	-0,135461389	0,125	0,051	1	0,910378638	1,098444051	2,450980392
SMURF1	0,943031489	-0,135692598	0,094	0,086	1	0,910232751	1,098620104	1,093023256
CCDC14	0,094709571	-0,135698965	0,219	0,102	1	0,910228734	1,098624953	2,147058824
MKRN2	0,030493266	-0,135750505	0,219	0,082	1	0,910196217	1,098664202	2,670731707

Supplementary Table 1

Differentially expressed genes_adjacent-skin-XL_ comparing SC-Keloid vs. (SC-Myel, SC-Nonmyel)								
**gene**	**p_val**	**avg_log2FC**	**pct.1**	**pct.2**	**p_val_adj**	**Foldchange_UP**	**Foldchange_DOWN**	**Ratio_pct1_pct2**
RAB33A	0,313292814	-0,135827261	0	0,031	1	0,910147793	1,098722656	0
NBEA	0,283788463	-0,135941265	0	0,035	1	0,910075874	1,098809482	0
DDX47	0,257548576	-0,135989942	0	0,039	1	0,910045168	1,098846557	0
ZNF665	0,80991663	-0,136019447	0,031	0,023	1	0,910026557	1,098869029	1,347826087
SYDE1	0,010405093	-0,136039455	0,188	0,055	1	0,910013937	1,09888427	3,418181818
RNF139	0,163012044	-0,136107453	0,156	0,074	1	0,909971046	1,098936064	2,108108108
H2AW	0,600062	-0,136142066	0,188	0,137	1	0,909949215	1,09896243	1,372262774
HIBCH	0,022445649	-0,136173301	0,281	0,109	1	0,909929514	1,098986223	2,577981651
MYBBP1A	0,578217897	-0,136202536	0,062	0,039	1	0,909911075	1,099008493	1,58974359
RANGAP1	0,202549436	-0,136229722	0,188	0,098	1	0,909893929	1,099029203	1,918367347
ANKRD42	0,668126075	-0,13626663	0,062	0,043	1	0,909870652	1,09905732	1,441860465
SDHB	0,000138929	-0,136313504	0,469	0,137	1	0,90984109	1,099093029	3,423357664
PRKAB2	0,066791287	-0,13637147	0,125	0,043	1	0,909804534	1,099137191	2,906976744
LINC00115	0,804543977	-0,136444398	0,031	0,039	1	0,909758545	1,099192754	0,794871795
GGCX	0,485433146	-0,136544553	0,094	0,059	1	0,90969539	1,099269064	1,593220339
KLHL34	0,313292814	-0,136554997	0	0,031	1	0,909688804	1,099277022	0
PPP1R21	0,376291412	-0,136590687	0,094	0,051	1	0,9096663	1,099304217	1,843137255
PCDHB16	0,428872689	-0,136614841	0	0,02	1	0,909651071	1,099322622	0
ISYNA1	0,434183714	-0,136641746	0,125	0,078	1	0,909634107	1,099343123	1,602564103
USP28	0,924392442	-0,136687298	0,031	0,027	1	0,909605386	1,099377835	1,148148148
TNFRSF6B	0,428872689	-0,136718086	0	0,02	1	0,909585975	1,099401296	0
SCLT1	0,076971853	-0,136892117	0,188	0,078	1	0,909476259	1,099533925	2,41025641
DCAF1	0,573780511	-0,136946972	0,062	0,039	1	0,909441679	1,099575733	1,58974359
TTF1	0,017422452	-0,136985323	0,219	0,074	1	0,909417503	1,099604963	2,959459459
TSGA10	0,38481627	-0,137038382	0	0,023	1	0,909384058	1,099645405	0
C3orf62	0,80991663	-0,137057793	0,031	0,023	1	0,909371823	1,0996602	1,347826087
FBXW4	0,415867271	-0,137062877	0,031	0,07	1	0,909368618	1,099664075	0,442857143
FXR2	0,06440165	-0,137151973	0,156	0,059	1	0,90931246	1,099731988	2,644067797
C12orf29	0,017377488	-0,137159395	0,281	0,105	1	0,909307782	1,099737646	2,676190476
CX3CL1	0,480650464	-0,137332848	0	0,016	1	0,909198464	1,099869874	0

Supplementary Table 1

Differentially expressed genes_adjacent-skin-XL_ comparing SC-Keloid vs. (SC-Myel, SC-Nonmyel)								
**gene**	**p_val**	**avg_log2FC**	**pct.1**	**pct.2**	**p_val_adj**	**Foldchange_UP**	**Foldchange_DOWN**	**Ratio_pct1_pct2**
TMEM39A	0,920939129	-0,137432413	0,062	0,055	1	0,909135719	1,099945782	1,127272727
ARFGEF1	0,086229766	-0,137435076	0,156	0,062	1	0,909134041	1,099947812	2,516129032
MRPL48	0,003997708	-0,137462053	0,25	0,074	1	0,909117042	1,09996838	3,378378378
GOLPH3	0,013381021	-0,137507948	0,281	0,102	1	0,909088121	1,100003373	2,754901961
ZNF133	0,150155729	-0,137525074	0,062	0,02	1	0,909077329	1,100016432	3,1
METTL3	0,043169771	-0,137568034	0,188	0,07	1	0,90905026	1,100049188	2,685714286
UHRF1BP1	0,979143607	-0,137599526	0,031	0,031	1	0,909030417	1,1000732	1
TRAF6	0,755689148	-0,13766183	0,062	0,047	1	0,90899116	1,100120709	1,319148936
RNF214	0,578217897	-0,137687216	0,062	0,039	1	0,908975166	1,100140067	1,58974359
PHF12	0,158954432	-0,137751294	0,125	0,055	1	0,908934794	1,100188932	2,272727273
THOC2	0,073890297	-0,138016904	0,375	0,188	1	0,908767469	1,100391502	1,994680851
NCOR2	0,011768519	-0,138027945	0,375	0,148	1	0,908760513	1,100399924	2,533783784
CISH	0,480650464	-0,138106926	0	0,016	1	0,908710764	1,100460168	0
UBE2Q2L	0,283789128	-0,138520521	0	0,035	1	0,908450291	1,100775695	0
C11orf91	0,578217897	-0,138528449	0,062	0,039	1	0,908445298	1,100781744	1,58974359
AP3M2	0,045196862	-0,138616537	0,125	0,039	1	0,908389832	1,100848958	3,205128205
MRPS9	0,373239546	-0,138623855	0,094	0,051	1	0,908385225	1,100854541	1,843137255
RABGAP1L	0,384687297	-0,138742832	0,125	0,074	1	0,908310314	1,100945331	1,689189189
CALCOCO1	0,00581471	-0,138790989	0,312	0,109	1	0,908279996	1,100982081	2,862385321
F10	0,346686972	-0,13888985	0	0,027	1	0,908217758	1,101057529	0
NSMF	0,021379715	-0,139008386	0,156	0,047	1	0,908143139	1,101147999	3,319148936
NCKIPSD	0,840130893	-0,139011404	0,062	0,051	1	0,908141239	1,101150302	1,215686275
TRPM4	0,38481627	-0,13903958	0	0,023	1	0,908123503	1,101171808	0
CUBN	0,191913299	-0,13907184	0,094	0,039	1	0,908103197	1,101196431	2,41025641
USP2	0,346686972	-0,13911642	0	0,027	1	0,908075136	1,10123046	0
SCAF1	0,086049964	-0,139254739	0,219	0,098	1	0,907988078	1,101336046	2,234693878
NSL1	0,003355836	-0,13931659	0,312	0,102	1	0,907949153	1,101383263	3,058823529
STAM2	0,367181952	-0,139322289	0,094	0,051	1	0,907945566	1,101387614	1,843137255
MNT	0,885008839	-0,139322535	0,062	0,055	1	0,907945411	1,101387801	1,127272727
BMP4	0,38481627	-0,139342593	0	0,023	1	0,907932788	1,101403114	0

Supplementary Table 1

Differentially expressed genes_adjacent-skin-XL_ comparing SC-Keloid vs. (SC-Myel, SC-Nonmyel)								
**gene**	**p_val**	**avg_log2FC**	**pct.1**	**pct.2**	**p_val_adj**	**Foldchange_UP**	**Foldchange_DOWN**	**Ratio_pct1_pct2**
TCAIM	0,551839113	-0,139348704	0,062	0,039	1	0,907928942	1,10140778	1,58974359
PAPOLA	0,050044716	-0,139483803	0,625	0,312	1	0,907843924	1,101510924	2,003205128
BAZ1B	0,049054766	-0,139665436	0,312	0,145	1	0,907729635	1,101649611	2,151724138
STXBP5	0,370203052	-0,139757714	0,094	0,051	1	0,907671577	1,101720078	1,843137255
EPHA5	0,480650464	-0,139763384	0	0,016	1	0,90766801	1,101724407	0
USP1	0,012712505	-0,139823977	0,375	0,148	1	0,907629888	1,101770681	2,533783784
CYP2D6	0,543117005	-0,139862838	0	0,012	1	0,907605441	1,101800359	0
HPS6	0,38481627	-0,139993113	0	0,023	1	0,907523487	1,101899856	0
ZNF205	0,578217897	-0,140036007	0,062	0,039	1	0,907496506	1,101932618	1,58974359
IPO8	0,016179439	-0,140082093	0,219	0,074	1	0,907467517	1,101967819	2,959459459
GTF3C4	0,817344545	-0,140104873	0,062	0,051	1	0,907453188	1,101985219	1,215686275
GATB	0,346686972	-0,140171099	0	0,027	1	0,907411533	1,102035806	0
TTC7A	0,578217897	-0,140180239	0,062	0,039	1	0,907405784	1,102042788	1,58974359
CETN3	0,118142322	-0,140247936	0,125	0,051	1	0,907363206	1,102094501	2,450980392
GGA3	0,924392325	-0,140302263	0,031	0,027	1	0,907329038	1,102136003	1,148148148
LIPE-AS1	0,804543977	-0,140602637	0,031	0,039	1	0,907140149	1,102365496	0,794871795
LLGL2	0,924392442	-0,140674702	0,031	0,027	1	0,907094837	1,102420562	1,148148148
MARF1	0,428970186	-0,140807066	0,094	0,055	1	0,907011617	1,102521711	1,709090909
RETREG2	0,006746293	-0,140821642	0,281	0,094	1	0,907002453	1,10253285	2,989361702
LONRF3	0,38481627	-0,140832853	0	0,023	1	0,906995405	1,102541418	0
ZHX2	0,979143607	-0,14087312	0,031	0,031	1	0,90697009	1,102572192	1
ZNF529	0,979143607	-0,140881222	0,031	0,031	1	0,906964997	1,102578383	1
CES4A	0,346686972	-0,140884469	0	0,027	1	0,906962955	1,102580865	0
RCCD1	0,573780511	-0,1409502	0,062	0,039	1	0,906921634	1,102631101	1,58974359
MAPKAPK5-AS1	0,7917997	-0,140997518	0,094	0,074	1	0,906891889	1,102667266	1,27027027
ATG5	0,074729729	-0,141035981	0,188	0,078	1	0,906867711	1,102696664	2,41025641
MRNIP	0,580948071	-0,141076879	0,094	0,062	1	0,906842004	1,102727924	1,516129032
EXT1	0,346694817	-0,141091677	0,125	0,07	1	0,906832702	1,102739235	1,785714286
RANBP10	0,682170546	-0,141184824	0,031	0,02	1	0,906774155	1,102810435	1,55
FBXL19	0,480650464	-0,141258402	0	0,016	1	0,90672791	1,102866681	0

Supplementary Table 1

Differentially expressed genes_adjacent-skin-XL_ comparing SC-Keloid vs. (SC-Myel, SC-Nonmyel)								
**gene**	**p_val**	**avg_log2FC**	**pct.1**	**pct.2**	**p_val_adj**	**Foldchange_UP**	**Foldchange_DOWN**	**Ratio_pct1_pct2**
TRPM7	0,012515483	-0,14131221	0,281	0,102	1	0,906694093	1,102907815	2,754901961
ZNF836	0,38481627	-0,14132407	0	0,023	1	0,906686639	1,102916881	0
PARP10	0,161825316	-0,141370182	0,219	0,113	1	0,906657659	1,102952134	1,938053097
RADIL	0,346686972	-0,141377328	0	0,027	1	0,906653169	1,102957597	0
RRM2B	0,191913299	-0,141411646	0,094	0,039	1	0,906631602	1,102983834	2,41025641
GFPT2	0,224763613	-0,141427125	0,062	0,023	1	0,906621874	1,102995668	2,695652174
LAYN	0,158954432	-0,141447242	0,125	0,055	1	0,906609232	1,103011048	2,272727273
TRIM5	0,38481627	-0,141520996	0	0,023	1	0,906562886	1,103067438	0
ADRB2	0,887104347	-0,14152129	0,031	0,035	1	0,906562701	1,103067664	0,885714286
RO60	0,006717589	-0,141601809	0,562	0,242	1	0,906512105	1,103129229	2,32231405
H3C10	0,480650464	-0,141644836	0	0,016	1	0,90648507	1,103162129	0
RCL1	0,396289917	-0,141707146	0,062	0,031	1	0,90644592	1,103209776	2
SPTBN4	0,38481627	-0,141763737	0	0,023	1	0,906410365	1,103253051	0
GTDC1	0,219587526	-0,141971038	0,188	0,102	1	0,906280132	1,103411589	1,843137255
ARRDC1-AS1	0,376291412	-0,142021724	0,094	0,051	1	0,906248292	1,103450356	1,843137255
DYRK1B	0,428872689	-0,142035217	0	0,02	1	0,906239816	1,103460676	0
TTC28	0,955618714	-0,142048035	0,094	0,094	1	0,906231765	1,10347048	1
NFATC2IP	0,016391618	-0,142124323	0,25	0,09	1	0,906183845	1,103528831	2,777777778
EEF2K	0,668126075	-0,142208634	0,062	0,043	1	0,90613089	1,103593323	1,441860465
FAM107A	0,313292814	-0,142217633	0	0,031	1	0,906125237	1,103600207	0
ARHGEF37	0,257548576	-0,142419147	0	0,039	1	0,90599868	1,103754367	0
UNK	0,12714655	-0,142427872	0,156	0,07	1	0,9059932	1,103761043	2,228571429
SCAF4	0,077757581	-0,142449133	0,25	0,113	1	0,905979849	1,103777309	2,212389381
TMEFF1	0,663576959	-0,142471882	0,031	0,047	1	0,905965563	1,103794714	0,659574468
HDHD2	0,376291412	-0,14248305	0,094	0,051	1	0,90595855	1,103803259	1,843137255
NAT9	0,248144547	-0,142493385	0,156	0,082	1	0,90595206	1,103811167	1,902439024
ABRAXAS1	0,376291412	-0,14255711	0,094	0,051	1	0,905912045	1,103859923	1,843137255
USP54	0,980211723	-0,142655753	0,062	0,059	1	0,905850106	1,103935401	1,050847458
ATP2A2	0,130846492	-0,142715461	0,312	0,168	1	0,905812617	1,10398109	1,857142857
GATAD1	0,00430374	-0,142873819	0,344	0,117	1	0,905713195	1,104102276	2,94017094

Supplementary Table 1

Differentially expressed genes_adjacent-skin-XL_ comparing SC-Keloid vs. (SC-Myel, SC-Nonmyel)								
**gene**	**p_val**	**avg_log2FC**	**pct.1**	**pct.2**	**p_val_adj**	**Foldchange_UP**	**Foldchange_DOWN**	**Ratio_pct1_pct2**
BOC	0,313292814	-0,142921311	0	0,031	1	0,905683381	1,104138622	0
PDP2	0,313292814	-0,142932271	0	0,031	1	0,9056765	1,10414701	0
TMEM126B	0,125283821	-0,142963146	0,375	0,199	1	0,905657118	1,10417064	1,884422111
UST	0,578217897	-0,142996391	0,062	0,039	1	0,905636249	1,104196085	1,58974359
NUDT12	0,093753684	-0,143074889	0,125	0,047	1	0,905586973	1,104256167	2,659574468
KIAA1549L	0,38481627	-0,143109824	0	0,023	1	0,905565045	1,104282907	0
FGFRL1	0,804543977	-0,143157239	0,031	0,039	1	0,905535284	1,1043192	0,794871795
ARSL	0,141661018	-0,143174439	0,094	0,035	1	0,905524488	1,104332366	2,685714286
CREB1	0,013381021	-0,143227894	0,281	0,102	1	0,905490937	1,104373285	2,754901961
SAMD5	0,514167295	-0,143408764	0,125	0,082	1	0,905377423	1,104511748	1,524390244
KTN1	0,131259472	-0,143413078	0,875	0,512	1	0,905374716	1,104515051	1,708984375
AADAT	0,573780511	-0,143439727	0,062	0,039	1	0,905357992	1,104535453	1,58974359
AMY2B	0,38481627	-0,143579008	0	0,023	1	0,905270591	1,104642093	0
ARHGEF1	0,010405093	-0,143629674	0,188	0,055	1	0,905238799	1,104680888	3,418181818
KCTD2	0,487085433	-0,143923292	0,062	0,035	1	0,905054583	1,104905736	1,771428571
MAFA	0,428872689	-0,143940768	0	0,02	1	0,90504362	1,10491912	0
ALMS1	0,979143607	-0,143952556	0,031	0,031	1	0,905036225	1,104928148	1
NAA50	0,05005634	-0,143975337	0,375	0,176	1	0,905021934	1,104945595	2,130681818
TAB1	0,032268627	-0,144014326	0,156	0,051	1	0,904997476	1,104975457	3,058823529
PHLDB3	0,80991663	-0,144037575	0,031	0,023	1	0,904982892	1,104993264	1,347826087
PPP6C	0,000886095	-0,144141172	0,344	0,102	1	0,90491791	1,105072614	3,37254902
CYFIP2	0,898329199	-0,144180116	0,031	0,035	1	0,904893483	1,105102444	0,885714286
SLK	0,00294808	-0,144217695	0,375	0,129	1	0,904869913	1,10513123	2,906976744
FSBP	0,428872689	-0,144371907	0	0,02	1	0,904773195	1,105249366	0
PDP1	0,221404772	-0,144413362	0,156	0,082	1	0,904747197	1,105281125	1,902439024
RBM27	0,097146104	-0,144427659	0,219	0,102	1	0,904738231	1,105292079	2,147058824
VPS16	0,002096899	-0,144446713	0,156	0,031	1	0,904726282	1,105306676	5,032258065
MLYCD	0,979143607	-0,14447092	0,031	0,031	1	0,904711102	1,105325223	1
WWP2	0,313292814	-0,144522399	0	0,031	1	0,90467882	1,105364664	0
MIGA2	0,918113127	-0,144561467	0,031	0,027	1	0,904654322	1,105394598	1,148148148

Supplementary Table 1

Differentially expressed genes_adjacent-skin-XL_ comparing SC-Keloid vs. (SC-Myel, SC-Nonmyel)								
**gene**	**p_val**	**avg_log2FC**	**pct.1**	**pct.2**	**p_val_adj**	**Foldchange_UP**	**Foldchange_DOWN**	**Ratio_pct1_pct2**
TIMM10B	0,516147046	-0,144715612	0,094	0,059	1	0,904557669	1,10551271	1,593220339
CRAMP1	0,668126075	-0,144963557	0,062	0,043	1	0,904402223	1,105702722	1,441860465
NQO2	0,011445553	-0,144978757	0,25	0,086	1	0,904392694	1,105714372	2,906976744
C18orf21	0,313292814	-0,144986791	0	0,031	1	0,904387658	1,10572053	0
NECAB1	0,38481627	-0,145066915	0	0,023	1	0,904337432	1,105781941	0
EIF3A	0,147059932	-0,145135085	0,562	0,316	1	0,904294701	1,105834192	1,778481013
EFCAB5	0,543117005	-0,145166348	0	0,012	1	0,904275105	1,105858156	0
NEK4	0,313292814	-0,145314349	0	0,031	1	0,904182344	1,105971607	0
DTD2	0,243596382	-0,145363755	0,094	0,043	1	0,90415138	1,106009483	2,186046512
PLEKHA8	0,445227443	-0,14536454	0,094	0,055	1	0,904150888	1,106010085	1,709090909
SLC25A43	0,346686972	-0,145365445	0	0,027	1	0,904150321	1,106010778	0
ZDHHC17	0,009336902	-0,145370087	0,281	0,098	1	0,904147412	1,106014337	2,867346939
CORO2B	0,163022941	-0,145372935	0,188	0,094	1	0,904145627	1,10601652	2
EPC1	0,073415381	-0,145392937	0,5	0,254	1	0,904133092	1,106031855	1,968503937
CRADD	0,445227443	-0,145411809	0,094	0,055	1	0,904121265	1,106046323	1,709090909
HIF1A	0,025560092	-0,14542645	0,25	0,098	1	0,904112089	1,106057548	2,551020408
GPR153	0,376291412	-0,145446847	0,094	0,051	1	0,904099307	1,106073185	1,843137255
C14orf28	0,428872689	-0,145490669	0	0,02	1	0,904071845	1,106106782	0
CYP1B1	0,979143607	-0,145524657	0,031	0,031	1	0,904050547	1,106132842	1
TBCEL	0,313292814	-0,145793384	0	0,031	1	0,903882167	1,106338897	0
ZNF33B	0,480650464	-0,145820485	0	0,016	1	0,903865188	1,10635968	0
LY6G5B	0,38481627	-0,145974963	0	0,023	1	0,903768411	1,106478151	0
HSD17B8	0,141661018	-0,146018052	0,094	0,035	1	0,903741419	1,106511198	2,685714286
MAP3K1	0,238963839	-0,146069011	0,125	0,062	1	0,903709497	1,106550283	2,016129032
TTC33	0,293095985	-0,146096102	0,125	0,066	1	0,903692527	1,106571062	1,893939394
CTH	0,80991663	-0,14613524	0,031	0,023	1	0,903668012	1,106601082	1,347826087
NR4A3	0,880532593	-0,146137639	0,062	0,055	1	0,903666509	1,106602922	1,127272727
SCFD1	0,022224283	-0,146199983	0,344	0,141	1	0,90362746	1,106650744	2,439716312
BRIX1	0,039525798	-0,146220707	0,219	0,086	1	0,903614479	1,106666641	2,546511628
PKIB	0,346686972	-0,146260839	0	0,027	1	0,903589343	1,106697426	0

Supplementary Table 1

Differentially expressed genes_adjacent-skin-XL_ comparing SC-Keloid vs. (SC-Myel, SC-Nonmyel)								
**gene**	**p_val**	**avg_log2FC**	**pct.1**	**pct.2**	**p_val_adj**	**Foldchange_UP**	**Foldchange_DOWN**	**Ratio_pct1_pct2**
ATXN7L1	0,924392442	-0,146431473	0,031	0,027	1	0,903482478	1,106828328	1,148148148
MYRF	0,480650464	-0,146487143	0	0,016	1	0,903447616	1,106871038	0
AZIN1	0,034279306	-0,146569607	0,531	0,25	1	0,903395976	1,106934308	2,124
LINC01006	0,205678783	-0,146590678	0,156	0,078	1	0,903382782	1,106950476	2
EPHA7	0,346686972	-0,146621925	0	0,027	1	0,903363216	1,106974451	0
EDEM1	0,191913299	-0,146669079	0,094	0,039	1	0,903333691	1,107010632	2,41025641
COG1	0,141661018	-0,146871658	0,094	0,035	1	0,903206855	1,107166087	2,685714286
EGLN2	0,033696287	-0,146893713	0,312	0,133	1	0,903193048	1,107183013	2,345864662
GFOD1	0,979143607	-0,146911164	0,031	0,031	1	0,903182123	1,107196406	1
TANC1	0,038148997	-0,146932772	0,406	0,191	1	0,903168596	1,107212989	2,12565445
SYNC	0,695004519	-0,146964652	0,094	0,07	1	0,903148638	1,107237455	1,342857143
AHRR	0,346686972	-0,147138178	0	0,027	1	0,903040015	1,107370641	0
BRD8	0,086035399	-0,147149326	0,25	0,121	1	0,903033037	1,107379198	2,066115702
PINLYP	0,38481627	-0,147179492	0	0,023	1	0,903014155	1,107402353	0
CAND2	0,38481627	-0,147187727	0	0,023	1	0,903009001	1,107408673	0
LINC02693	0,80991663	-0,1472351	0,031	0,023	1	0,90297935	1,107445038	1,347826087
PNPLA7	0,38481627	-0,147262862	0	0,023	1	0,902961973	1,107466349	0
ABHD3	0,730358576	-0,147285954	0,031	0,043	1	0,902947521	1,107484075	0,720930233
PITPNM2	0,480650464	-0,147332439	0	0,016	1	0,902918428	1,10751976	0
CNOT7	0,003573812	-0,14735065	0,312	0,102	1	0,90290703	1,10753374	3,058823529
PDXDC1	0,44252225	-0,147474201	0,156	0,102	1	0,902829709	1,107628592	1,529411765
NAT8L	0,346686972	-0,147588449	0	0,027	1	0,902758217	1,107716309	0
POLR3E	0,663576959	-0,147623667	0,031	0,047	1	0,902736179	1,107743351	0,659574468
KLHL26	0,709822531	-0,147658981	0,094	0,07	1	0,902714082	1,107770467	1,342857143
CNTLN	0,06366796	-0,147683189	0,156	0,059	1	0,902698935	1,107789054	2,644067797
ME1	0,38481627	-0,147718164	0	0,023	1	0,902677051	1,107815911	0
IL1R1	0,979143607	-0,147774757	0,031	0,031	1	0,902641643	1,107859368	1
ZDHHC6	0,142110321	-0,147843404	0,156	0,07	1	0,902598694	1,107912084	2,228571429
CCL27	0,482846351	-0,147852925	0,062	0,035	1	0,902592738	1,107919395	1,771428571
LYRM7	0,209000952	-0,147977703	0,156	0,078	1	0,902514676	1,108015223	2

Supplementary Table 1

Differentially expressed genes_adjacent-skin-XL_ comparing SC-Keloid vs. (SC-Myel, SC-Nonmyel)								
**gene**	**p_val**	**avg_log2FC**	**pct.1**	**pct.2**	**p_val_adj**	**Foldchange_UP**	**Foldchange_DOWN**	**Ratio_pct1_pct2**
SEC23B	0,373239546	-0,148003411	0,094	0,051	1	0,902498594	1,108034968	1,843137255
UGP2	0,002500836	-0,148381038	0,312	0,102	1	0,902262395	1,108325035	3,058823529
AKAP8	0,042072475	-0,148410085	0,219	0,086	1	0,902244229	1,10834735	2,546511628
NEO1	0,80991663	-0,148462267	0,031	0,023	1	0,902211595	1,10838744	1,347826087
GSTK1	0,006409259	-0,148494577	0,562	0,234	1	0,90219139	1,108412263	2,401709402
RANBP2	0,032749577	-0,148706157	0,375	0,168	1	0,902059088	1,10857483	2,232142857
CDYL	0,024125962	-0,14872558	0,25	0,094	1	0,902046943	1,108589755	2,659574468
MTERF4	0,031413167	-0,148774736	0,156	0,051	1	0,902016209	1,108627528	3,058823529
PPIC	0,887104347	-0,148877532	0,031	0,035	1	0,90195194	1,108706524	0,885714286
LCLAT1	0,480650464	-0,148961193	0	0,016	1	0,901899638	1,108770818	0
UNC50	0,0332995	-0,149017936	0,344	0,152	1	0,901864166	1,108814429	2,263157895
PIK3R3	0,046495836	-0,14932125	0,156	0,055	1	0,901674577	1,109047572	2,836363636
CTDSP1	0,001326358	-0,149356886	0,406	0,133	1	0,901652305	1,109074966	3,052631579
PPP1R12B	0,087744784	-0,149365473	0,188	0,082	1	0,901646938	1,109081568	2,292682927
VIPAS39	0,037506898	-0,149561481	0,094	0,023	1	0,901524446	1,109232261	4,086956522
CLCN2	0,487085433	-0,149600803	0,062	0,035	1	0,901499875	1,109262494	1,771428571
UTP20	0,093753684	-0,149603285	0,125	0,047	1	0,901498324	1,109264402	2,659574468
TRIM13	0,112512668	-0,14963088	0,219	0,105	1	0,901481081	1,10928562	2,085714286
VPS52	0,80991663	-0,149637284	0,031	0,023	1	0,901477079	1,109290544	1,347826087
NPIPB15	0,663576959	-0,14967522	0,031	0,047	1	0,901453375	1,109319713	0,659574468
GNG2	0,102932724	-0,149715565	0,781	0,434	1	0,901428166	1,109350736	1,799539171
TMEM201	0,979143607	-0,149785313	0,031	0,031	1	0,901384587	1,10940437	1
MAP4K2	0,346686972	-0,149813736	0	0,027	1	0,901366829	1,109426226	0
APOOL	0,187685368	-0,149852511	0,094	0,039	1	0,901342604	1,109456045	2,41025641
NTRK3	0,578217897	-0,150031789	0,062	0,039	1	0,901230605	1,109593921	1,58974359
ERV3-1	0,191913299	-0,150039573	0,094	0,039	1	0,901225742	1,109599908	2,41025641
ZNF84	0,487085433	-0,150091869	0,062	0,035	1	0,901193074	1,10964013	1,771428571
NOC4L	0,170210335	-0,150397981	0,156	0,074	1	0,901001878	1,109875599	2,108108108
DZIP3	0,65904021	-0,15041492	0,062	0,043	1	0,9009913	1,109888631	1,441860465
FBXO34	0,502372892	-0,150455117	0,094	0,059	1	0,900966196	1,109919555	1,593220339

Supplementary Table 1

Differentially expressed genes_adjacent-skin-XL_ comparing SC-Keloid vs. (SC-Myel, SC-Nonmyel)								
**gene**	**p_val**	**avg_log2FC**	**pct.1**	**pct.2**	**p_val_adj**	**Foldchange_UP**	**Foldchange_DOWN**	**Ratio_pct1_pct2**
BCL2L1	0,046495836	-0,150527774	0,156	0,055	1	0,900920823	1,109975455	2,836363636
BCLAF3	0,122617152	-0,150622577	0,188	0,086	1	0,900861623	1,110048396	2,186046512
FXYD6	0,000699188	-0,150681961	0,312	0,086	1	0,900824543	1,110094089	3,627906977
LMOD1	0,38481627	-0,150693261	0	0,023	1	0,900817487	1,110102783	0
MAP3K5	0,428872689	-0,150765627	0	0,02	1	0,900772303	1,110158468	0
FAM13A-AS1	0,428872689	-0,150769748	0	0,02	1	0,90076973	1,11016164	0
HERC3	0,283789128	-0,150820961	0	0,035	1	0,900737755	1,110201049	0
EAPP	0,028096732	-0,150846605	0,469	0,215	1	0,900721744	1,110220783	2,181395349
FAN1	0,552606916	-0,150890941	0,094	0,062	1	0,900694064	1,110254902	1,516129032
SAP130	0,487085433	-0,150895354	0,062	0,035	1	0,900691309	1,110258298	1,771428571
TSPOAP1	0,38481627	-0,150991913	0	0,023	1	0,900631028	1,11033261	0
RXRA	0,028370624	-0,151219406	0,25	0,098	1	0,900489022	1,110507708	2,551020408
CBLB	0,820322169	-0,151286338	0,031	0,039	1	0,900447246	1,110559229	0,794871795
UNC13D	0,38481627	-0,15140733	0	0,023	1	0,900371734	1,11065237	0
PCDHB4	0,346686972	-0,151463317	0	0,027	1	0,900336793	1,110695472	0
KIF13A	0,011597542	-0,151572501	0,219	0,07	1	0,900268658	1,110779534	3,128571429
BRSK2	0,346686972	-0,151576909	0	0,027	1	0,900265907	1,110782928	0
PDXK	0,016831044	-0,151618943	0,281	0,105	1	0,900239677	1,110815292	2,676190476
NCEH1	0,682170546	-0,151640405	0,031	0,02	1	0,900226285	1,110831817	1,55
IZUMO4	0,313292814	-0,151676567	0	0,031	1	0,900203721	1,110859661	0
ATF7IP	0,008555004	-0,151801261	0,375	0,145	1	0,900125918	1,110955678	2,586206897
INIP	0,505287368	-0,151843199	0,125	0,082	1	0,900099753	1,110987973	1,524390244
RNPS1	0,002880026	-0,15188956	0,594	0,234	1	0,900070829	1,111023675	2,538461538
USP49	0,313292814	-0,151970094	0	0,031	1	0,900020586	1,111085697	0
C1orf35	0,222039877	-0,151976151	0,219	0,121	1	0,900016807	1,111090362	1,809917355
C19orf81	0,30793671	-0,151978527	0,062	0,027	1	0,900015325	1,111092191	2,296296296
UBLCP1	0,001348326	-0,152012696	0,281	0,078	1	0,899994009	1,111118507	3,602564103
PLPP2	0,346686972	-0,152063354	0	0,027	1	0,899962409	1,111157522	0
ZNF333	0,840130893	-0,1520702	0,062	0,051	1	0,899958138	1,111162795	1,215686275
IFIH1	0,918113127	-0,152081082	0,031	0,027	1	0,899951349	1,111171177	1,148148148

Supplementary Table 1

Differentially expressed genes_adjacent-skin-XL_ comparing SC-Keloid vs. (SC-Myel, SC-Nonmyel)								
**gene**	**p_val**	**avg_log2FC**	**pct.1**	**pct.2**	**p_val_adj**	**Foldchange_UP**	**Foldchange_DOWN**	**Ratio_pct1_pct2**
MAP3K13	0,006343661	-0,152129372	0,281	0,094	1	0,899921227	1,11120837	2,989361702
ZFPM2	0,428872689	-0,152143508	0	0,02	1	0,899912409	1,111219258	0
INTS2	0,487085433	-0,15220712	0,062	0,035	1	0,89987273	1,111268256	1,771428571
USP36	0,548211541	-0,152308245	0,156	0,109	1	0,899809656	1,111346153	1,431192661
MRPL43	0,00546859	-0,152584485	0,469	0,18	1	0,899637382	1,111558968	2,605555556
CCDC152	0,578217897	-0,152594274	0,062	0,039	1	0,899631278	1,11156651	1,58974359
AFG3L2	0,030492951	-0,152662627	0,25	0,098	1	0,899588655	1,111619176	2,551020408
TDP2	0,668126075	-0,152721525	0,062	0,043	1	0,89955193	1,111664559	1,441860465
MTRF1L	0,066791287	-0,152725242	0,125	0,043	1	0,899549613	1,111667423	2,906976744
NFE2L1	0,000589142	-0,152771838	0,438	0,141	1	0,89952056	1,111703328	3,106382979
NIPAL3	0,887104347	-0,152785654	0,031	0,035	1	0,899511946	1,111713974	0,885714286
ARHGEF3	0,924392442	-0,152797148	0,031	0,027	1	0,899504779	1,111722831	1,148148148
CIART	0,979143607	-0,15280955	0,031	0,031	1	0,899497047	1,111732388	1
CA13	0,668126075	-0,152921619	0,062	0,043	1	0,899427176	1,111818751	1,441860465
H4C8	0,543117005	-0,153068956	0	0,012	1	0,899335326	1,111932303	0
DHX38	0,012156741	-0,153166001	0,25	0,086	1	0,899274833	1,112007101	2,906976744
LSMEM2	0,346686972	-0,1531949	0	0,027	1	0,899256819	1,112029377	0
INPP5A	0,510608096	-0,153313166	0,031	0,059	1	0,899183105	1,11212054	0,525423729
FARP2	0,242059311	-0,153413475	0,188	0,102	1	0,899120588	1,112197867	1,843137255
DENND4B	0,310355006	-0,153436601	0,094	0,047	1	0,899106176	1,112215695	2
CPPED1	7,0737E-05	-0,153458639	0,344	0,082	1	0,899092441	1,112232685	4,195121951
PCNX3	0,979143607	-0,153484966	0,031	0,031	1	0,899076034	1,112252982	1
POGK	0,021726813	-0,153541295	0,188	0,062	1	0,899040931	1,112296409	3,032258065
KIF16B	0,580948071	-0,153558994	0,094	0,062	1	0,899029902	1,112310055	1,516129032
EED	0,804543977	-0,153725673	0,031	0,039	1	0,898926041	1,112438571	0,794871795
PAFAH1B1	0,021721731	-0,153796329	0,625	0,289	1	0,898882017	1,112493054	2,162629758
ADM	0,797009215	-0,153838366	0,062	0,074	1	0,898855825	1,11252547	0,837837838
ABCB4	0,487085433	-0,153846437	0,062	0,035	1	0,898850797	1,112531695	1,771428571
ZNF330	0,376881888	-0,153912505	0,188	0,117	1	0,898809635	1,112582644	1,606837607
BMP2K	0,569030388	-0,153912841	0,25	0,184	1	0,898809426	1,112582903	1,358695652

Supplementary Table 1

Differentially expressed genes_adjacent-skin-XL_ comparing SC-Keloid vs. (SC-Myel, SC-Nonmyel)								
**gene**	**p_val**	**avg_log2FC**	**pct.1**	**pct.2**	**p_val_adj**	**Foldchange_UP**	**Foldchange_DOWN**	**Ratio_pct1_pct2**
WRAP53	0,028712073	-0,153935545	0,125	0,035	1	0,898795281	1,112600412	3,571428571
ID1	0,014643669	-0,153967856	0,219	0,074	1	0,898775152	1,11262533	2,959459459
MGAT3	0,924392442	-0,153985373	0,031	0,027	1	0,898764239	1,11263884	1,148148148
SUMO4	0,924392442	-0,154087416	0,031	0,027	1	0,898700671	1,112717541	1,148148148
NAALAD2	0,38481627	-0,154154033	0	0,023	1	0,898659174	1,112768922	0
SIRT7	0,001626207	-0,154223822	0,25	0,066	1	0,898615703	1,112822752	3,787878788
TMEM185A	0,007848729	-0,154357422	0,219	0,066	1	0,898532491	1,112925809	3,318181818
LNP1	0,480650464	-0,154391066	0	0,016	1	0,898511537	1,112951764	0
TMEM167B	0,102169822	-0,154498033	0,219	0,102	1	0,898444921	1,113034285	2,147058824
ATP1B1	0,004295141	-0,154528399	0,688	0,285	1	0,89842601	1,113057713	2,414035088
GNA13	0,030158445	-0,154556084	0,219	0,082	1	0,89840877	1,113079072	2,670731707
BRPF3	0,907442332	-0,154558866	0,062	0,055	1	0,898407037	1,113081219	1,127272727
EMC2	0,069682133	-0,154572504	0,438	0,223	1	0,898398545	1,113091741	1,964125561
NPAT	0,668126075	-0,154654439	0,062	0,043	1	0,898347524	1,113154958	1,441860465
ZNF407	0,804543977	-0,15469663	0,031	0,039	1	0,898321252	1,113187512	0,794871795
ENHO	0,283789128	-0,154744865	0	0,035	1	0,898291218	1,113224732	0
PIGL	0,38481627	-0,154751967	0	0,023	1	0,898286796	1,113230211	0
GNPTG	0,033118365	-0,15478699	0,344	0,148	1	0,898264989	1,113257237	2,324324324
KAZN	0,085318957	-0,154840345	0,156	0,062	1	0,89823177	1,113298409	2,516129032
RNF166	0,013196028	-0,154870268	0,156	0,043	1	0,898213139	1,1133215	3,627906977
TMEM63B	0,302364586	-0,155011073	0,125	0,066	1	0,89812548	1,113430164	1,893939394
HEY2	0,313292814	-0,155022526	0	0,031	1	0,89811835	1,113439003	0
INTS1	0,082632923	-0,155053107	0,156	0,062	1	0,898099312	1,113462605	2,516129032
CXCL8	0,157961013	-0,15506211	0,188	0,094	1	0,898093708	1,113469554	2
ARID1A	0,111390099	-0,15506771	0,312	0,16	1	0,898090222	1,113473875	1,95
NR1H3	0,746521062	-0,155192984	0,062	0,047	1	0,898012241	1,113570567	1,319148936
DLX2	0,668126075	-0,155360233	0,062	0,043	1	0,897908142	1,113699668	1,441860465
TSN	0,060916409	-0,155405416	0,188	0,074	1	0,897880022	1,113734548	2,540540541
PLEKHA2	0,257548576	-0,155427722	0	0,039	1	0,897866139	1,113751768	0
TUBGCP4	0,668126075	-0,155734098	0,062	0,043	1	0,897675485	1,113988313	1,441860465

Supplementary Table 1

Differentially expressed genes_adjacent-skin-XL_ comparing SC-Keloid vs. (SC-Myel, SC-Nonmyel)								
**gene**	**p_val**	**avg_log2FC**	**pct.1**	**pct.2**	**p_val_adj**	**Foldchange_UP**	**Foldchange_DOWN**	**Ratio_pct1_pct2**
CUL9	0,30793671	-0,155742801	0,062	0,027	1	0,89767007	1,113995034	2,296296296
RPP25L	0,578217499	-0,155891006	0,062	0,039	1	0,897577859	1,114109478	1,58974359
NCOA6	0,505798338	-0,155919373	0,094	0,059	1	0,89756021	1,114131385	1,593220339
PHIP	0,018057075	-0,155977028	0,531	0,242	1	0,897524342	1,11417591	2,194214876
NFKBID	0,603353082	-0,156029614	0,031	0,051	1	0,897491628	1,114216522	0,607843137
MGME1	0,032268627	-0,156069742	0,156	0,051	1	0,897466665	1,114247514	3,058823529
DIDO1	0,032866024	-0,156090739	0,188	0,066	1	0,897453603	1,114263731	2,848484848
LPIN2	0,653016738	-0,156150408	0,094	0,066	1	0,897416485	1,114309817	1,424242424
CEP164	0,06222129	-0,156158549	0,156	0,059	1	0,897411422	1,114316105	2,644067797
SLC25A30-AS1	0,480650464	-0,15617117	0	0,016	1	0,897403571	1,114325853	0
CCDC59	0,012153845	-0,15623965	0,469	0,199	1	0,897360975	1,114378748	2,35678392
RASA2	0,505798338	-0,156248858	0,094	0,059	1	0,897355248	1,114385861	1,593220339
ADPRHL1	0,313292814	-0,15625778	0	0,031	1	0,897349698	1,114392752	0
GRIN2B	0,313292814	-0,1562875	0	0,031	1	0,897331213	1,114415709	0
SOCS1	0,804543977	-0,156293695	0,031	0,039	1	0,89732736	1,114420495	0,794871795
MIR4458HG	0,015526375	-0,156856978	0,188	0,059	1	0,896977078	1,114855691	3,186440678
TRIP12	0,005677323	-0,156857067	0,375	0,137	1	0,896977022	1,11485576	2,737226277
EPHB6	0,487085433	-0,15686511	0,062	0,035	1	0,896972022	1,114861976	1,771428571
PSMD5	0,093753684	-0,156899122	0,125	0,047	1	0,896950875	1,11488826	2,659574468
SENP3	0,804543977	-0,156985213	0,031	0,039	1	0,896897353	1,114954791	0,794871795
GSN-AS1	0,226351275	-0,157003175	0,031	0,008	1	0,896886186	1,114968673	3,875
UNKL	0,967025549	-0,157007994	0,062	0,059	1	0,89688319	1,114972397	1,050847458
YPEL2	0,159667137	-0,15703885	0,25	0,129	1	0,896864008	1,114996244	1,937984496
SGMS1	0,039103141	-0,15713771	0,25	0,102	1	0,896802553	1,115072651	2,450980392
CEBPZOS	0,023853062	-0,157207477	0,219	0,078	1	0,896759186	1,115126576	2,807692308
MOB4	0,061583228	-0,157311186	0,438	0,215	1	0,896694724	1,115206741	2,037209302
XRCC1	0,202646283	-0,157382836	0,125	0,059	1	0,896650191	1,115262128	2,118644068
SCRIB	0,137474745	-0,157492767	0,188	0,09	1	0,896581871	1,115347112	2,088888889
OGT	0,879936477	-0,157655985	0,156	0,152	1	0,896480442	1,115473303	1,026315789
GOLGA6L4	0,313292814	-0,157682012	0	0,031	1	0,896464269	1,115493427	0

Supplementary Table 1

Differentially expressed genes_adjacent-skin-XL_ comparing SC-Keloid vs. (SC-Myel, SC-Nonmyel)								
**gene**	**p_val**	**avg_log2FC**	**pct.1**	**pct.2**	**p_val_adj**	**Foldchange_UP**	**Foldchange_DOWN**	**Ratio_pct1_pct2**
HEG1	0,804543977	-0,157737261	0,031	0,039	1	0,89642994	1,115536146	0,794871795
ACVR1B	0,487085433	-0,157756486	0,062	0,035	1	0,896417994	1,115551012	1,771428571
KPNA5	0,997801078	-0,157991023	0,062	0,059	1	0,896272276	1,11573238	1,050847458
PPIL4	0,026160842	-0,158112835	0,594	0,281	1	0,896196604	1,115826589	2,113879004
PLA2G4B	0,346686972	-0,158113626	0	0,027	1	0,896196113	1,115827201	0
FZD4	0,892714086	-0,15811692	0,031	0,035	1	0,896194067	1,115829748	0,885714286
SPTY2D1	0,054396443	-0,158186082	0,219	0,09	1	0,896151105	1,115883242	2,433333333
MTX3	0,487085433	-0,158218679	0,062	0,035	1	0,896130857	1,115908455	1,771428571
FTO	0,126128812	-0,158354503	0,125	0,051	1	0,896046494	1,116013518	2,450980392
COPS7B	0,370203052	-0,158407051	0,094	0,051	1	0,896013857	1,116054168	1,843137255
EXOC8	0,887104347	-0,158424179	0,031	0,035	1	0,896003219	1,116067419	0,885714286
SNX33	0,887104347	-0,158444591	0,031	0,035	1	0,895990542	1,11608321	0,885714286
MDM2	0,238991084	-0,158497945	0,156	0,082	1	0,895957407	1,116124485	1,902439024
PRPF38B	0,105080828	-0,158661366	0,375	0,195	1	0,895855924	1,116250921	1,923076923
ZNF865	0,141661018	-0,158865594	0,094	0,035	1	0,895729115	1,116408949	2,685714286
FHIP1B	0,01489812	-0,158893202	0,188	0,059	1	0,895711974	1,116430313	3,186440678
ASB6	0,098751938	-0,158912205	0,094	0,031	1	0,895700176	1,116445019	3,032258065
ABCB10	0,428872689	-0,158982488	0	0,02	1	0,895656542	1,116499409	0
ABHD17B	0,99780108	-0,158982863	0,062	0,059	1	0,895656309	1,116499699	1,050847458
ACVR1	0,892714086	-0,159101864	0,031	0,035	1	0,895582434	1,116591798	0,885714286
SEPTIN4	0,313292814	-0,159136869	0	0,031	1	0,895560704	1,116618891	0
CDCA7L	0,804543977	-0,159261401	0,031	0,039	1	0,895483404	1,11671528	0,794871795
PIGK	0,001591384	-0,159266922	0,375	0,121	1	0,895479976	1,116719554	3,099173554
PDPK1	0,17621177	-0,159290283	0,188	0,094	1	0,895465476	1,116737637	2
DLGAP1-AS2	0,38481627	-0,159317677	0	0,023	1	0,895448473	1,116758842	0
DNASE1	0,283789128	-0,159347046	0	0,035	1	0,895430245	1,116781576	0
ZFAND2B	0,023577809	-0,159662241	0,219	0,078	1	0,895234636	1,117025593	2,807692308
NFKBIB	0,188205529	-0,159663885	0,125	0,059	1	0,895233615	1,117026866	2,118644068
STK11IP	0,840130893	-0,159695438	0,062	0,051	1	0,895214036	1,117051297	1,215686275
C19orf12	0,516147046	-0,159954315	0,094	0,059	1	0,895053414	1,117251758	1,593220339

Supplementary Table 1

Differentially expressed genes_adjacent-skin-XL_ comparing SC-Keloid vs. (SC-Myel, SC-Nonmyel)								
**gene**	**p_val**	**avg_log2FC**	**pct.1**	**pct.2**	**p_val_adj**	**Foldchange_UP**	**Foldchange_DOWN**	**Ratio_pct1_pct2**
ANKRD52	0,016858455	-0,159989039	0,125	0,031	1	0,895031871	1,117278649	4,032258065
HSPA14	0,668126075	-0,160040815	0,062	0,043	1	0,89499975	1,117318747	1,441860465
DOCK10	0,283789128	-0,160115752	0	0,035	1	0,894953263	1,117376785	0
NUBP1	0,046495836	-0,160129295	0,156	0,055	1	0,894944862	1,117387274	2,836363636
RAVER2	0,80991663	-0,160156113	0,031	0,023	1	0,894928226	1,117408045	1,347826087
SLC12A2	0,035794044	-0,160160733	0,25	0,102	1	0,89492536	1,117411624	2,450980392
ZNF394	0,005195348	-0,160196977	0,219	0,062	1	0,894902878	1,117439696	3,532258065
GPR137B	0,016314912	-0,160239672	0,406	0,168	1	0,894876395	1,117472766	2,416666667
CCSER2	0,047107985	-0,16024164	0,531	0,262	1	0,894875174	1,11747429	2,026717557
ADAMTSL4	0,38481627	-0,160245637	0	0,023	1	0,894872695	1,117477386	0
KCTD7	0,224763613	-0,160249456	0,062	0,023	1	0,894870326	1,117480345	2,695652174
ZNF117	0,428872689	-0,160320894	0	0,02	1	0,894826016	1,11753568	0
HEXD	0,045356999	-0,16044754	0,156	0,055	1	0,894747467	1,117633787	2,836363636
MALINC1	0,428872689	-0,160524117	0	0,02	1	0,894699976	1,117693111	0
NECTIN3	0,202646283	-0,160673866	0,125	0,059	1	0,894607113	1,117809132	2,118644068
NSRP1	0,001571041	-0,160721782	0,406	0,133	1	0,894577401	1,117846258	3,052631579
GAS7	0,598297014	-0,16084176	0,938	0,629	1	0,894503009	1,117939224	1,491255962
MME	0,313292814	-0,160890103	0	0,031	1	0,894473036	1,117976686	0
NUP35	0,283789128	-0,161094434	0	0,035	1	0,894346359	1,118135038	0
LLGL1	0,682170546	-0,161177817	0,031	0,02	1	0,89429467	1,118199665	1,55
TFIP11	0,979143607	-0,161210633	0,031	0,031	1	0,894274329	1,118225099	1
UBE2V1	0,061399135	-0,161222166	0,594	0,301	1	0,89426718	1,118234038	1,973421927
MAP3K6	0,093753684	-0,161294993	0,125	0,047	1	0,894222038	1,118290488	2,659574468
KLHDC1	0,38481627	-0,161338815	0	0,023	1	0,894194876	1,118324457	0
XRRA1	0,175031227	-0,161513583	0,281	0,152	1	0,894086561	1,118459939	1,848684211
RREB1	0,204506657	-0,161633785	0,125	0,059	1	0,894012071	1,11855313	2,118644068
TM7SF3	0,017987005	-0,16168237	0,312	0,121	1	0,893981963	1,1185908	2,578512397
SUOX	0,428872689	-0,161685015	0	0,02	1	0,893980325	1,118592851	0
PRKD1	0,32461626	-0,161721677	0,125	0,07	1	0,893957607	1,118621277	1,785714286
DONSON	0,037506898	-0,161725945	0,094	0,023	1	0,893954962	1,118624587	4,086956522

Supplementary Table 1

Differentially expressed genes_adjacent-skin-XL_ comparing SC-Keloid vs. (SC-Myel, SC-Nonmyel)								
**gene**	**p_val**	**avg_log2FC**	**pct.1**	**pct.2**	**p_val_adj**	**Foldchange_UP**	**Foldchange_DOWN**	**Ratio_pct1_pct2**
GPM6A	0,283789128	-0,16172718	0	0,035	1	0,893954197	1,118625544	0
SLC22A5	0,428872689	-0,161780374	0	0,02	1	0,893921236	1,11866679	0
GBP4	0,616047684	-0,161818676	0,031	0,051	1	0,893897504	1,11869649	0,607843137
AKAP8L	0,057218654	-0,162061333	0,312	0,145	1	0,893747166	1,118884667	2,151724138
NTNG2	0,313292814	-0,162097327	0	0,031	1	0,893724868	1,118912582	0
HOXC4	0,979143607	-0,162230665	0,031	0,031	1	0,893642271	1,119016	1
KLHL22	0,428872689	-0,162241267	0	0,02	1	0,893635704	1,119024224	0
B4GALT4	0,663576959	-0,162343955	0,031	0,047	1	0,893572099	1,119103876	0,659574468
IGIP	0,80991663	-0,16235158	0,031	0,023	1	0,893567377	1,119109791	1,347826087
C5orf24	0,033637365	-0,162408052	0,531	0,258	1	0,8935324	1,119153598	2,058139535
AK4	0,38481627	-0,162512409	0	0,023	1	0,893467769	1,119234554	0
ZNF585A	0,187685368	-0,162530215	0,094	0,039	1	0,893456741	1,119248368	2,41025641
GABRB3	0,283789128	-0,162604613	0	0,035	1	0,893410668	1,119306088	0
PLCL1	0,313292814	-0,162657516	0	0,031	1	0,893377908	1,119347133	0
LDLR	0,106706178	-0,162678983	0,156	0,066	1	0,893364615	1,119363789	2,363636364
MIPOL1	0,668126075	-0,162712848	0,062	0,043	1	0,893343644	1,119390065	1,441860465
DCUN1D1	0,023853062	-0,162726401	0,219	0,078	1	0,893335253	1,11940058	2,807692308
SLC22A23	0,668126075	-0,162729505	0,062	0,043	1	0,89333333	1,119402989	1,441860465
DNAJC2	0,001963472	-0,162750811	0,281	0,082	1	0,893320137	1,119419521	3,426829268
PLAG1	0,346686972	-0,162778271	0	0,027	1	0,893303135	1,119440827	0
ZNF660	0,226351275	-0,162779288	0,031	0,008	1	0,893302505	1,119441617	3,875
POT1	0,607571545	-0,162794894	0,031	0,051	1	0,893292841	1,119453726	0,607843137
SARM1	0,578217897	-0,162937865	0,062	0,039	1	0,893204321	1,119564669	1,58974359
PRKCE	0,257548576	-0,162976757	0	0,039	1	0,893180242	1,119594851	0
SIAH2	0,055470426	-0,163028894	0,219	0,09	1	0,893147964	1,119635313	2,433333333
H2AZ2	0,036060551	-0,163068937	0,688	0,336	1	0,893123175	1,119666389	2,047619048
CHIC1	0,346686972	-0,163070292	0	0,027	1	0,893122336	1,11966744	0
FAT1	0,031984901	-0,163145979	0,406	0,188	1	0,893075482	1,119726183	2,159574468
HSPA1L	0,887104347	-0,163154063	0,031	0,035	1	0,893070478	1,119732457	0,885714286
PPP5C	0,191913299	-0,163190774	0,094	0,039	1	0,893047753	1,119760949	2,41025641

Supplementary Table 1

Differentially expressed genes_adjacent-skin-XL_ comparing SC-Keloid vs. (SC-Myel, SC-Nonmyel)								
gene	p_val	avg_log2FC	pct.1	pct.2	p_val_adj	Foldchange_UP	Foldchange_DOWN	Ratio_pct1_pct2
NR2C2	0,046495836	-0,163207541	0,156	0,055	1	0,893037374	1,119773964	2,836363636
NBPF9	0,540537634	-0,163384894	0,031	0,016	1	0,892927598	1,119911628	1,9375
CEACAM19	0,924392442	-0,163393507	0,031	0,027	1	0,892922267	1,119918314	1,148148148
ARHGAP5-AS1	0,804543977	-0,163757023	0,031	0,039	1	0,892697306	1,120200535	0,794871795
KCNIP3	0,480650464	-0,163790304	0	0,016	1	0,892676713	1,120226377	0
SIPA1L3	0,019236133	-0,163843751	0,094	0,02	1	0,892643643	1,120267879	4,7
NEDD4	0,441948175	-0,163881637	0,094	0,055	1	0,892620202	1,120297298	1,709090909
ANKS3	0,007871757	-0,163884545	0,156	0,039	1	0,892618402	1,120299556	4
ZSCAN32	0,428872689	-0,164031049	0	0,02	1	0,892527763	1,120413327	0
CPOX	0,283789128	-0,164100614	0	0,035	1	0,892484727	1,120467354	0
SART3	0,010405093	-0,164125287	0,188	0,055	1	0,892469464	1,120486516	3,418181818
PPP2R5E	0,026679107	-0,164199765	0,219	0,082	1	0,892423392	1,120544361	2,670731707
TADA2B	0,682170546	-0,164203656	0,031	0,02	1	0,892420985	1,120547383	1,55
U2AF1L4	0,062941189	-0,164320916	0,156	0,059	1	0,892348453	1,120638464	2,644067797
TPBG	0,38481627	-0,164351293	0	0,023	1	0,892329665	1,12066206	0
GDNF	0,887104347	-0,164406642	0,031	0,035	1	0,892295431	1,120705055	0,885714286
COG7	0,924392442	-0,164614553	0,031	0,027	1	0,892166849	1,120866574	1,148148148
ARMCX5	0,396289917	-0,164696548	0,062	0,031	1	0,892116145	1,12093028	2
FAM120B	0,835562242	-0,164699623	0,062	0,051	1	0,892114243	1,120932669	1,215686275
SLC25A13	0,000137099	-0,164723594	0,25	0,051	1	0,89209942	1,120951294	4,901960784
ZMYM1	0,38481627	-0,164755374	0	0,023	1	0,892079769	1,120975987	0
ZBTB17	0,163771186	-0,164833405	0,125	0,055	1	0,89203152	1,121036619	2,272727273
XRN1	0,219379907	-0,164840199	0,25	0,141	1	0,89202732	1,121041898	1,773049645
TMEM91	0,162153727	-0,164850008	0,125	0,055	1	0,892021255	1,121049521	2,272727273
CNR1	0,428872689	-0,165011912	0	0,02	1	0,891921155	1,121175335	0
KIF27	0,735253332	-0,165038547	0,031	0,043	1	0,891904688	1,121196035	0,720930233
TEX41	0,428872689	-0,165226028	0	0,02	1	0,891788791	1,121341746	0
AHCYL1	0,019312158	-0,1653081	0,469	0,199	1	0,891738061	1,121405538	2,35678392
INPP4B	0,38481627	-0,165319257	0	0,023	1	0,891731164	1,121414211	0
LRP1	0,012154915	-0,165410174	0,75	0,344	1	0,89167497	1,121484883	2,180232558

Supplementary Table 1

Differentially expressed genes_adjacent-skin-XL_ comparing SC-Keloid vs. (SC-Myel, SC-Nonmyel)								
**gene**	**p_val**	**avg_log2FC**	**pct.1**	**pct.2**	**p_val_adj**	**Foldchange_UP**	**Foldchange_DOWN**	**Ratio_pct1_pct2**
RMND5B	0,755689148	-0,16567891	0,062	0,047	1	0,89150889	1,121693806	1,319148936
ANKRD20A4P	0,283789128	-0,165723901	0	0,035	1	0,891481089	1,121728786	0
HECTD1	0,008160896	-0,165744814	0,469	0,191	1	0,891468166	1,121745047	2,455497382
SMG5	0,24851603	-0,165787264	0,094	0,043	1	0,891441936	1,121778054	2,186046512
FILIP1	0,257548576	-0,16578785	0	0,039	1	0,891441574	1,12177851	0
MOB3C	0,283789128	-0,165940436	0	0,035	1	0,891347296	1,12189716	0
UBE2E2	0,13446757	-0,166064997	0,156	0,07	1	0,891270341	1,121994028	2,228571429
SLC25A27	0,346686972	-0,166111048	0	0,027	1	0,891241892	1,122029842	0
SPRY2	0,002659034	-0,166184834	0,625	0,25	1	0,891196311	1,12208723	2,5
IKZF2	0,480650464	-0,166214992	0	0,016	1	0,891177682	1,122110686	0
FRMD4B	0,283788463	-0,166376772	0	0,035	1	0,891077753	1,122236524	0
TPR	0,015662533	-0,16647999	0,469	0,211	1	0,891014003	1,122316818	2,222748815
WRNIP1	0,045147709	-0,166532346	0,188	0,07	1	0,890981668	1,122357547	2,685714286
RASSF8-AS1	0,050259834	-0,166537924	0,25	0,105	1	0,890978224	1,122361887	2,380952381
NTM	0,193723669	-0,166590707	0,938	0,574	1	0,890945627	1,122402951	1,634146341
YTHDF1	0,846196088	-0,166658577	0,094	0,078	1	0,890903714	1,122455754	1,205128205
BOD1	0,024242212	-0,166683314	0,312	0,129	1	0,890888438	1,122475	2,418604651
POLR2B	0,00211817	-0,166697179	0,375	0,125	1	0,890879876	1,122485788	3
CYRIA	0,578217897	-0,166726114	0,062	0,039	1	0,890862009	1,122508301	1,58974359
SNX25	0,428872689	-0,166880278	0	0,02	1	0,890766818	1,122628257	0
ANKRD30B	0,38481627	-0,166912946	0	0,023	1	0,890746648	1,122653678	0
ZNF740	0,487085433	-0,166973351	0,062	0,035	1	0,890709354	1,122700683	1,771428571
LDAF1	0,516147046	-0,167053813	0,094	0,059	1	0,890659678	1,122763301	1,593220339
CTBP1-DT	0,346686972	-0,167205739	0	0,027	1	0,89056589	1,122881542	0
PER2	0,310355006	-0,167296722	0,094	0,047	1	0,890509729	1,122952358	2
PIR	0,313292814	-0,167316197	0	0,031	1	0,890497708	1,122967517	0
C9orf72	0,38481627	-0,167323261	0	0,023	1	0,890493348	1,122973016	0
SETD1A	0,032268627	-0,167349113	0,156	0,051	1	0,890477391	1,122993139	3,058823529
NSUN4	0,428872689	-0,16741049	0	0,02	1	0,890439508	1,123040915	0
TMEM178A	0,38481627	-0,167484637	0	0,023	1	0,890393745	1,123098635	0

Supplementary Table 1

Differentially expressed genes_adjacent-skin-XL_ comparing SC-Keloid vs. (SC-Myel, SC-Nonmyel)								
**gene**	**p_val**	**avg_log2FC**	**pct.1**	**pct.2**	**p_val_adj**	**Foldchange_UP**	**Foldchange_DOWN**	**Ratio_pct1_pct2**
KLC1	0,174462513	-0,167494792	0,531	0,309	1	0,890387478	1,123106541	1,718446602
METTL17	0,157372807	-0,167495336	0,125	0,055	1	0,890387142	1,123106964	2,272727273
DVL2	0,516147046	-0,167724775	0,094	0,059	1	0,890245551	1,123285592	1,593220339
DSG3	0,428872689	-0,167963448	0	0,02	1	0,890098284	1,123471439	0
RRAGD	0,234088337	-0,168108845	0	0,043	1	0,890008583	1,12358467	0
DNALI1	0,191913299	-0,168149277	0,094	0,039	1	0,889983641	1,123616159	2,41025641
GABPB2	0,141661018	-0,168158351	0,094	0,035	1	0,889978043	1,123623226	2,685714286
GJB6	0,283789128	-0,168271206	0	0,035	1	0,889908428	1,123711124	0
PHLPP2	0,979143607	-0,16828917	0,031	0,031	1	0,889897347	1,123725117	1
ABT1	0,396289917	-0,168297998	0,062	0,031	1	0,889891902	1,123731993	2
MCCC1	0,668126075	-0,16851801	0,062	0,043	1	0,889756202	1,123903376	1,441860465
DGKD	0,396289917	-0,168553864	0,062	0,031	1	0,88973409	1,123931308	2
PHC3	0,22427219	-0,168679	0,188	0,102	1	0,88965692	1,1240288	1,843137255
DHTKD1	0,257548576	-0,16920993	0	0,039	1	0,889329576	1,124442532	0
ING3	0,247212967	-0,169263554	0,125	0,062	1	0,88929652	1,124484328	2,016129032
DPH7	0,300029356	-0,169310927	0,125	0,066	1	0,88926732	1,124521252	1,893939394
XYLT2	0,577368489	-0,169425365	0,094	0,062	1	0,889196784	1,124610455	1,516129032
EBF4	0,569359326	-0,169465332	0,062	0,039	1	0,889172151	1,124641611	1,58974359
TMEM158	0,141661018	-0,169529925	0,094	0,035	1	0,889132341	1,124691965	2,685714286
WDR37	0,924392442	-0,169700259	0,031	0,027	1	0,88902737	1,124824762	1,148148148
POLG	0,249306044	-0,169722603	0,125	0,062	1	0,889013602	1,124842183	2,016129032
UVSSA	0,141661018	-0,169722965	0,094	0,035	1	0,889013379	1,124842465	2,685714286
PTPN13	0,171744666	-0,169736909	0,281	0,152	1	0,889004786	1,124853337	1,848684211
TBCE	0,373239546	-0,169969064	0,094	0,051	1	0,888861741	1,12503436	1,843137255
SLC16A1	0,123421204	-0,170094044	0,125	0,051	1	0,888784743	1,125131825	2,450980392
BIRC7	0,346686972	-0,170101978	0	0,027	1	0,888779855	1,125138013	0
TNPO2	0,012061903	-0,170297682	0,219	0,07	1	0,888659299	1,12529065	3,128571429
PYROXD2	0,480650464	-0,170396888	0	0,016	1	0,888598192	1,125368033	0
RNF44	0,668126075	-0,170404332	0,062	0,043	1	0,888593608	1,125373839	1,441860465
XPO6	0,074729729	-0,170406574	0,188	0,078	1	0,888592227	1,125375588	2,41025641

Supplementary Table 1

Differentially expressed genes_adjacent-skin-XL_ comparing SC-Keloid vs. (SC-Myel, SC-Nonmyel)								
**gene**	**p_val**	**avg_log2FC**	**pct.1**	**pct.2**	**p_val_adj**	**Foldchange_UP**	**Foldchange_DOWN**	**Ratio_pct1_pct2**
DSG1	0,573780511	-0,170558201	0,062	0,039	1	0,888498841	1,125493871	1,58974359
GIGYF1	0,192364395	-0,170635549	0,188	0,098	1	0,888451206	1,125554215	1,918367347
DENND1A	0,924392442	-0,170659681	0,031	0,027	1	0,888436345	1,125573042	1,148148148
TFPI2	0,016858455	-0,170685143	0,125	0,031	1	0,888420666	1,125592907	4,032258065
LTA4H	0,916437471	-0,170689839	0,062	0,055	1	0,888417773	1,125596572	1,127272727
ENSA	0,006587322	-0,170730285	0,5	0,199	1	0,888392867	1,125628128	2,512562814
TVP23C	0,482846351	-0,171004423	0,062	0,035	1	0,888224073	1,125842038	1,771428571
ERMN	0,428872689	-0,17107929	0	0,02	1	0,888177981	1,125900463	0
FLRT2	0,346686972	-0,171111144	0	0,027	1	0,888158371	1,125925323	0
LINC00461	0,257548576	-0,171135512	0	0,039	1	0,888143369	1,125944341	0
TTPAL	0,428872689	-0,17115666	0	0,02	1	0,88813035	1,125960846	0
ZNF34	0,376291412	-0,171276945	0,094	0,051	1	0,888056305	1,126054727	1,843137255
PAIP2B	0,313292814	-0,171340435	0	0,031	1	0,888017224	1,126104284	0
CEP44	0,933712959	-0,171513693	0,062	0,062	1	0,887910586	1,126239529	1
LTN1	0,003618924	-0,171541534	0,312	0,102	1	0,887893451	1,126261263	3,058823529
GRPEL1	0,014142285	-0,171649964	0,281	0,102	1	0,887826721	1,126345914	2,754901961
SH3BP5	0,050263346	-0,171655405	0,469	0,227	1	0,887823373	1,126350162	2,066079295
ASIC3	0,480650464	-0,171658059	0	0,016	1	0,88782174	1,126352234	0
CRISPLD2	0,021983485	-0,171699364	0,219	0,078	1	0,887796321	1,126384482	2,807692308
SERAC1	0,804543977	-0,171778168	0,031	0,039	1	0,887747829	1,12644601	0,794871795
AREL1	0,283789128	-0,171954895	0	0,035	1	0,887639088	1,126584006	0
TTC17	0,169441886	-0,172080879	0,281	0,156	1	0,887561578	1,12668239	1,801282051
TSPOAP1-AS1	0,428872689	-0,172135821	0	0,02	1	0,887527778	1,126725298	0
OFD1	0,08709749	-0,172137964	0,344	0,176	1	0,887526459	1,126726972	1,954545455
MSMO1	0,004241146	-0,172323303	0,281	0,09	1	0,887412449	1,126871729	3,122222222
PKP2	0,257548576	-0,172460356	0	0,039	1	0,887328151	1,126978783	0
CLK3	0,12985323	-0,17275061	0,219	0,105	1	0,887149648	1,127205542	2,085714286
FAM221A	0,573799329	-0,172879246	0,094	0,062	1	0,88707055	1,127306052	1,516129032
WIPI2	0,001193685	-0,172917717	0,375	0,117	1	0,887046896	1,127336114	3,205128205
TASOR2	0,078496688	-0,172969033	0,188	0,078	1	0,887015344	1,127376213	2,41025641

Supplementary Table 1

Differentially expressed genes_adjacent-skin-XL_ comparing SC-Keloid vs. (SC-Myel, SC-Nonmyel)								
**gene**	**p_val**	**avg_log2FC**	**pct.1**	**pct.2**	**p_val_adj**	**Foldchange_UP**	**Foldchange_DOWN**	**Ratio_pct1_pct2**
ASMTL-AS1	0,480650464	-0,172985753	0	0,016	1	0,887005065	1,127389279	0
CLPB	0,924392442	-0,173110746	0,031	0,027	1	0,886928219	1,127486959	1,148148148
VANGL2	0,556776127	-0,17320077	0,031	0,055	1	0,886872876	1,127557316	0,563636364
COL11A2	0,38481627	-0,173202405	0	0,023	1	0,886871871	1,127558594	0
DTX3	0,134082625	-0,17322414	0,188	0,09	1	0,88685851	1,127575581	2,088888889
POLR2M	0,069649574	-0,173226835	0,219	0,094	1	0,886856854	1,127577687	2,329787234
KIF5C	0,835562242	-0,173234228	0,062	0,051	1	0,886852309	1,127583466	1,215686275
HLA-DMA	0,38481627	-0,173256698	0	0,023	1	0,886838496	1,127601028	0
GPNMB	0,288533951	-0,173339468	0,125	0,066	1	0,886787618	1,127665722	1,893939394
SRGAP3	0,887104347	-0,173373336	0,031	0,035	1	0,886766801	1,127692195	0,885714286
RSF1	0,219892848	-0,173435704	0,469	0,277	1	0,886728467	1,127740946	1,693140794
FOXP1	0,008087959	-0,173509075	0,625	0,273	1	0,886683371	1,127798301	2,289377289
DVL3	0,071823511	-0,173567009	0,188	0,078	1	0,886647766	1,127843591	2,41025641
RERG	0,213026321	-0,173696708	0	0,047	1	0,886568059	1,127944989	0
SLC9A6	0,578217897	-0,173755428	0,062	0,039	1	0,886531975	1,1279909	1,58974359
GLYR1	0,001644566	-0,173811659	0,312	0,094	1	0,886497422	1,128034865	3,319148936
KIAA2026	0,014142248	-0,173842994	0,281	0,102	1	0,886478168	1,128059366	2,754901961
LDB1	0,578217897	-0,173966665	0,062	0,039	1	0,88640218	1,12815607	1,58974359
ACAP1	0,213026321	-0,173981707	0	0,047	1	0,886392938	1,128167832	0
CDH1	0,283789128	-0,174183199	0	0,035	1	0,88626915	1,128325407	0
FAM135A	0,663576959	-0,17422209	0,031	0,047	1	0,886245259	1,128355824	0,659574468
LRRC42	0,917697681	-0,174223513	0,094	0,082	1	0,886244385	1,128356937	1,146341463
RAD54L2	0,060283688	-0,17429408	0,188	0,074	1	0,886201037	1,12841213	2,540540541
SLC30A9	0,003201338	-0,17436827	0,219	0,059	1	0,886155465	1,12847016	3,711864407
ADGRB1	0,80991663	-0,174413557	0,031	0,023	1	0,886127649	1,128505584	1,347826087
POLR3A	0,283789128	-0,174561834	0	0,035	1	0,886036579	1,128621575	0
LRRC24	0,979143607	-0,174572115	0,031	0,031	1	0,886030265	1,128629618	1
THSD7B	0,38481627	-0,174622645	0	0,023	1	0,885999233	1,128669149	0
CS	0,656705388	-0,174689453	0,094	0,066	1	0,885958206	1,128721416	1,424242424
NBPF20	0,480650464	-0,174714217	0	0,016	1	0,885942998	1,128740791	0

Supplementary Table 1

Differentially expressed genes_adjacent-skin-XL_ comparing SC-Keloid vs. (SC-Myel, SC-Nonmyel)								
**gene**	**p_val**	**avg_log2FC**	**pct.1**	**pct.2**	**p_val_adj**	**Foldchange_UP**	**Foldchange_DOWN**	**Ratio_pct1_pct2**
KIF5A	0,38481627	-0,174759166	0	0,023	1	0,885915396	1,128775959	0
ZNF721	0,024411868	-0,174766289	0,219	0,078	1	0,885911021	1,128781532	2,807692308
EXOC5	0,144457051	-0,174849057	0,188	0,09	1	0,885860198	1,128846292	2,088888889
STK38L	0,283789128	-0,174924615	0	0,035	1	0,885813804	1,128905414	0
ORC2	0,482846351	-0,175031935	0,062	0,035	1	0,885747912	1,128989395	1,771428571
FAM53C	0,085298168	-0,175120977	0,219	0,098	1	0,885693246	1,129059078	2,234693878
CLN8	0,615516182	-0,175195641	0,156	0,113	1	0,88564741	1,129117512	1,380530973
NUFIP1	0,663576959	-0,175198325	0,031	0,047	1	0,885645762	1,129119612	0,659574468
MYH14	0,681846175	-0,175361012	0,031	0,047	1	0,885545897	1,129246946	0,659574468
WDSUB1	0,603353082	-0,175369884	0,031	0,051	1	0,885540451	1,129253891	0,607843137
TMEM192	0,840130893	-0,175429198	0,062	0,051	1	0,885504045	1,129300319	1,215686275
S100A14	0,911938491	-0,175444676	0,062	0,055	1	0,885494545	1,129312435	1,127272727
FOXC1	0,150155729	-0,175475059	0,062	0,02	1	0,885475896	1,129336218	3,1
SETD2	0,020351656	-0,175481014	0,312	0,125	1	0,885472241	1,12934088	2,496
PSMF1	0,00354177	-0,175508642	0,438	0,16	1	0,885455284	1,129362507	2,7375
TBX2	0,395250692	-0,175535722	0,125	0,074	1	0,885438665	1,129383706	1,689189189
EIF4G3	0,019190685	-0,175662471	0,312	0,125	1	0,885360877	1,129482933	2,496
C17orf67	0,283789128	-0,175849005	0	0,035	1	0,885246411	1,12962898	0
SLC4A5	0,924392442	-0,175872135	0,031	0,027	1	0,885232219	1,12964709	1,148148148
BDNF-AS	0,80991663	-0,175927405	0,031	0,023	1	0,885198306	1,129690368	1,347826087
ZNF496	0,163771487	-0,175989456	0,125	0,055	1	0,885160234	1,129738958	2,272727273
CYP46A1	0,234087804	-0,176147836	0	0,043	1	0,885063066	1,129862988	0
BCCIP	0,045356999	-0,17616643	0,156	0,055	1	0,885051659	1,12987755	2,836363636
BCAR3	0,037171106	-0,176175657	0,281	0,117	1	0,885045998	1,129884776	2,401709402
CEBPZ	0,00891214	-0,176187314	0,406	0,16	1	0,885038847	1,129893906	2,5375
CTBP2	0,053220599	-0,176208736	0,312	0,145	1	0,885025706	1,129910683	2,151724138
COQ8A	0,804543977	-0,176252488	0,031	0,039	1	0,884998866	1,12994495	0,794871795
MRI1	0,243596382	-0,176273751	0,094	0,043	1	0,884985823	1,129961604	2,186046512
SETDB1	0,428872689	-0,176332868	0	0,02	1	0,884949559	1,130007908	0
MAOA	0,540537634	-0,176397133	0,031	0,016	1	0,88491014	1,130058245	1,9375

Supplementary Table 1

Differentially expressed genes_adjacent-skin-XL_ comparing SC-Keloid vs. (SC-Myel, SC-Nonmyel)								
**gene**	**p_val**	**avg_log2FC**	**pct.1**	**pct.2**	**p_val_adj**	**Foldchange_UP**	**Foldchange_DOWN**	**Ratio_pct1_pct2**
UBE4B	0,588138226	-0,176425072	0,094	0,062	1	0,884893003	1,13008013	1,516129032
HACL1	0,864972765	-0,176434905	0,062	0,066	1	0,884886973	1,130087831	0,939393939
HAUS2	0,663576959	-0,176461018	0,031	0,047	1	0,884870956	1,130108286	0,659574468
ERO1A	0,112120373	-0,176529256	0,156	0,066	1	0,884829103	1,130161741	2,363636364
LARP7	0,00377279	-0,176634062	0,375	0,133	1	0,884764827	1,130243845	2,819548872
FLII	0,011699892	-0,176655266	0,281	0,102	1	0,884751823	1,130260458	2,754901961
RNASET2	0,804543977	-0,176748697	0,031	0,039	1	0,884694527	1,130333657	0,794871795
MICA	0,0600426	-0,176945785	0,281	0,125	1	0,884573676	1,130488084	2,248
PLA2G6	0,346686972	-0,177113963	0	0,027	1	0,884470565	1,130619875	0
NSMAF	0,234088337	-0,177307269	0	0,043	1	0,884352064	1,130771376	0
TIFA	0,65904021	-0,177338617	0,062	0,043	1	0,884332848	1,130795947	1,441860465
LSM14B	0,253529282	-0,177350365	0,125	0,062	1	0,884325647	1,130805155	2,016129032
PCYOX1L	0,487085433	-0,177388618	0,062	0,035	1	0,884302199	1,130835138	1,771428571
KBTBD7	0,755689148	-0,177501886	0,062	0,047	1	0,884232774	1,130923925	1,319148936
CNDP2	0,046495687	-0,177597126	0,156	0,055	1	0,884174403	1,130998587	2,836363636
MCM7	0,293095687	-0,177774378	0,125	0,066	1	0,884065778	1,131137552	1,893939394
SOGA3	0,668126075	-0,177844141	0,062	0,043	1	0,88402303	1,13119225	1,441860465
SV2C	0,313292814	-0,177968611	0	0,031	1	0,883946763	1,131289849	0
SMIM43	0,38481627	-0,177988427	0	0,023	1	0,883934622	1,131305388	0
LINC01003	0,465191913	-0,177997012	0,031	0,062	1	0,883929362	1,13131212	0,5
ANKS1B	0,283789128	-0,17799979	0	0,035	1	0,88392766	1,131314298	0
BRAT1	0,512685318	-0,178114482	0,094	0,059	1	0,883857391	1,13140424	1,593220339
ZNF432	0,30793671	-0,178157023	0,062	0,027	1	0,88383133	1,131437602	2,296296296
ARHGAP29	0,191913299	-0,17826219	0,094	0,039	1	0,883766904	1,131520082	2,41025641
C1QTNF4	0,804543977	-0,178283182	0,031	0,039	1	0,883754045	1,131536547	0,794871795
PIP4P1	0,294412282	-0,178285251	0,156	0,086	1	0,883752778	1,131538169	1,813953488
TMEM65	0,04735424	-0,178386769	0,219	0,09	1	0,883690593	1,131617795	2,433333333
KLHL13	0,346686972	-0,178399648	0	0,027	1	0,883682704	1,131627897	0
C3orf33	0,396289917	-0,178498212	0,062	0,031	1	0,883622333	1,131705212	2
KDM4B	0,045923444	-0,178625042	0,156	0,055	1	0,883544656	1,131804707	2,836363636

Supplementary Table 1

Differentially expressed genes_adjacent-skin-XL_ comparing SC-Keloid vs. (SC-Myel, SC-Nonmyel)								
**gene**	**p_val**	**avg_log2FC**	**pct.1**	**pct.2**	**p_val_adj**	**Foldchange_UP**	**Foldchange_DOWN**	**Ratio_pct1_pct2**
TIAM2	0,313292814	-0,178684619	0	0,031	1	0,88350817	1,131851446	0
PIK3CA	0,078068641	-0,178717805	0,219	0,098	1	0,883487847	1,131877482	2,234693878
CAMK2G	0,126128812	-0,178788456	0,125	0,051	1	0,883444582	1,131932914	2,450980392
TRIM44	0,017176388	-0,178803257	0,344	0,137	1	0,883435519	1,131944526	2,510948905
FBXO48	0,313292814	-0,178839607	0	0,031	1	0,883413261	1,131973047	0
PRMT5	0,920939129	-0,178903984	0,062	0,055	1	0,883373841	1,13202356	1,127272727
DBNDD2	0,143778766	-0,178909509	0,219	0,109	1	0,883370458	1,132027895	2,009174312
MYO6	0,575944473	-0,17897855	0,125	0,086	1	0,883328185	1,13208207	1,453488372
COQ7	0,924392442	-0,179028111	0,031	0,027	1	0,88329784	1,132120962	1,148148148
DENND10	0,06222129	-0,179068764	0,156	0,059	1	0,88327295	1,132152864	2,644067797
ZNF253	0,730358576	-0,179154895	0,031	0,043	1	0,883220219	1,132220457	0,720930233
NAB1	0,032927619	-0,179196263	0,219	0,082	1	0,883194894	1,132252923	2,670731707
GAS2	0,313292814	-0,179288137	0	0,031	1	0,883138652	1,132325029	0
MATN1-AS1	0,543117005	-0,179362448	0	0,012	1	0,883093164	1,132383355	0
NEK6	0,106706178	-0,179470356	0,156	0,066	1	0,883027115	1,132468056	2,363636364
LDLRAD3	0,38481627	-0,179732882	0	0,023	1	0,882866446	1,132674149	0
H6PD	0,084415941	-0,179882274	0,156	0,062	1	0,882775029	1,132791444	2,516129032
OXA1L	0,013247139	-0,179884062	0,312	0,117	1	0,882773935	1,132792848	2,666666667
IRF8	0,730358576	-0,179951615	0,031	0,043	1	0,882732601	1,132845891	0,720930233
C5orf51	0,887104347	-0,179981649	0,031	0,035	1	0,882714224	1,132869475	0,885714286
TUBB4A	0,257548576	-0,180063197	0	0,039	1	0,88266433	1,132933512	0
CCNB1IP1	0,228559925	-0,180101555	0,281	0,16	1	0,882640863	1,132963634	1,75625
IL17RA	0,924392442	-0,18031164	0,031	0,027	1	0,882512342	1,133128629	1,148148148
ARSG	0,428872689	-0,180332304	0	0,02	1	0,882499702	1,133144859	0
MYH10	0,840130893	-0,180341168	0,062	0,051	1	0,882494279	1,133151821	1,215686275
RNF43	0,38481627	-0,180416621	0	0,023	1	0,882448126	1,133211087	0
PYROXD1	0,668126075	-0,180459555	0,062	0,043	1	0,882421866	1,133244811	1,441860465
CENPC	0,317829628	-0,180634029	0,25	0,152	1	0,882315155	1,13338187	1,644736842
ASCC3	0,093923791	-0,180815673	0,25	0,121	1	0,882204073	1,133524578	2,066115702
KRT6B	0,487085433	-0,180858113	0,062	0,035	1	0,882178122	1,133557923	1,771428571

Supplementary Table 1

Differentially expressed genes_adjacent-skin-XL_ comparing SC-Keloid vs. (SC-Myel, SC-Nonmyel)								
**gene**	**p_val**	**avg_log2FC**	**pct.1**	**pct.2**	**p_val_adj**	**Foldchange_UP**	**Foldchange_DOWN**	**Ratio_pct1_pct2**
U2AF1	0,804543977	-0,181099099	0,031	0,039	1	0,882030776	1,133747288	0,794871795
ACAP3	0,566708997	-0,18137443	0,125	0,086	1	0,881862461	1,133963678	1,453488372
FRAS1	0,38481627	-0,181404095	0	0,023	1	0,881844328	1,133986995	0
PCMTD1	0,043619105	-0,181487115	0,375	0,176	1	0,881793584	1,134052252	2,130681818
HEYL	0,234088337	-0,181492433	0	0,043	1	0,881790333	1,134056433	0
FCGR2A	0,283789128	-0,181547638	0	0,035	1	0,881756593	1,134099828	0
SLC10A3	0,668126075	-0,181801192	0,031	0,047	1	0,881601637	1,134299164	0,659574468
KIAA0753	0,887104347	-0,181822686	0,031	0,035	1	0,881588503	1,134316063	0,885714286
OPHN1	0,135718813	-0,181932279	0,156	0,07	1	0,881521536	1,134402234	2,228571429
LRP1B	0,887104347	-0,181964025	0,031	0,035	1	0,881502139	1,134427197	0,885714286
RETSAT	0,668126075	-0,182120841	0,062	0,043	1	0,881406328	1,134550512	1,441860465
IDH3B	0,051511579	-0,182157346	0,281	0,121	1	0,881384026	1,13457922	2,32231405
SYBU	0,283789128	-0,182200569	0	0,035	1	0,88135762	1,134613212	0
ARHGAP17	0,045356999	-0,182232107	0,156	0,055	1	0,881338353	1,134638016	2,836363636
RFFL	0,234088337	-0,182234267	0	0,043	1	0,881337033	1,134639714	0
ARMCX2	0,817344545	-0,182249026	0,062	0,051	1	0,881328017	1,134651322	1,215686275
ME3	0,286287755	-0,182372802	0,156	0,086	1	0,881252407	1,134748674	1,813953488
DELE1	0,702399386	-0,182378487	0,094	0,07	1	0,881248935	1,134753145	1,342857143
ARRDC1	0,741950345	-0,182382444	0,062	0,047	1	0,881246517	1,134756258	1,319148936
TSPAN13	0,548952158	-0,182393063	0,031	0,055	1	0,881240031	1,13476461	0,563636364
CYP19A1	0,38481627	-0,182415736	0	0,023	1	0,881226182	1,134782444	0
SENP2	0,552857355	-0,18242172	0,031	0,055	1	0,881222527	1,13478715	0,563636364
CMTR1	0,24851603	-0,182423664	0,094	0,043	1	0,881221339	1,134788679	2,186046512
BLVRB	0,000478633	-0,18246427	0,562	0,191	1	0,881196537	1,13482062	2,942408377
COL15A1	0,225044228	-0,182574184	0,5	0,301	1	0,881129404	1,134907081	1,661129568
ZNF142	0,346686972	-0,182690968	0	0,027	1	0,881058081	1,134998954	0
DUSP22	0,584538006	-0,182894638	0,094	0,062	1	0,880933707	1,135159197	1,516129032
CDK19	0,237112684	-0,183038119	0,188	0,102	1	0,8808461	1,135272098	1,843137255
CSGALNACT1	0,234088337	-0,183110684	0	0,043	1	0,880801796	1,135329201	0
THAP5	0,079734103	-0,183171613	0,25	0,113	1	0,880764598	1,135377151	2,212389381

Supplementary Table 1

Differentially expressed genes_adjacent-skin-XL_ comparing SC-Keloid vs. (SC-Myel, SC-Nonmyel)								
**gene**	**p_val**	**avg_log2FC**	**pct.1**	**pct.2**	**p_val_adj**	**Foldchange_UP**	**Foldchange_DOWN**	**Ratio_pct1_pct2**
TP53RK	0,002502521	-0,183216639	0,25	0,07	1	0,88073711	1,135412586	3,571428571
NCKAP5L	0,920939129	-0,183256754	0,062	0,055	1	0,880712621	1,135444157	1,127272727
UNC13B	0,804543977	-0,18333224	0,031	0,039	1	0,880666541	1,135503568	0,794871795
ZBED3	0,887104347	-0,183348337	0,031	0,035	1	0,880656715	1,135516238	0,885714286
ADHFE1	0,283789128	-0,183463323	0	0,035	1	0,880586528	1,135606745	0
LMCD1-AS1	0,257548576	-0,183490355	0	0,039	1	0,880570028	1,135628023	0
SZT2	0,804543977	-0,183524629	0,031	0,039	1	0,880549108	1,135655003	0,794871795
PHF3	0,103229209	-0,183841558	0,344	0,18	1	0,880355692	1,135904509	1,911111111
NXT1	0,166191736	-0,183901172	0,25	0,133	1	0,880319316	1,135951447	1,879699248
STARD10	0,730358576	-0,183902303	0,031	0,043	1	0,880318625	1,135952338	0,720930233
EPM2AIP1	0,102169822	-0,183925213	0,219	0,102	1	0,880304646	1,135970377	2,147058824
LINC00639	0,38481627	-0,183952938	0	0,023	1	0,880287729	1,135992207	0
BICD1	0,639978691	-0,183976642	0,25	0,184	1	0,880273265	1,136010872	1,358695652
SLC25A46	0,505798338	-0,184003317	0,094	0,059	1	0,880256989	1,136031877	1,593220339
OARD1	0,653016738	-0,184090906	0,094	0,066	1	0,880203549	1,13610085	1,424242424
HERC5	0,730358576	-0,18426141	0,031	0,043	1	0,880099529	1,136235127	0,720930233
DNM2	0,000588939	-0,184340877	0,344	0,098	1	0,880051052	1,136297715	3,510204082
MAPK14	0,093753684	-0,184345498	0,125	0,047	1	0,880048233	1,136301355	2,659574468
DGCR2	0,140813864	-0,184368801	0,156	0,07	1	0,880034018	1,136319709	2,228571429
HSPA4	0,043081122	-0,184496541	0,25	0,102	1	0,879956102	1,136420326	2,450980392
ANO3	0,313292814	-0,184561658	0	0,031	1	0,879916385	1,13647162	0
CACNA2D2	0,887104347	-0,184598696	0,031	0,035	1	0,879893796	1,136500797	0,885714286
SNX18	0,251411476	-0,184625681	0,125	0,062	1	0,879877338	1,136522055	2,016129032
TYK2	0,020728744	-0,184697717	0,25	0,094	1	0,879833405	1,136578805	2,659574468
AC087239.1	0,578217897	-0,184815892	0,062	0,039	1	0,879761339	1,136671909	1,58974359
DLG5	0,021379617	-0,184843843	0,156	0,047	1	0,879744294	1,136693931	3,319148936
PIKFYVE	0,660402423	-0,184883975	0,094	0,066	1	0,879719822	1,136725551	1,424242424
ZSCAN18	0,504051677	-0,18496647	0,188	0,129	1	0,87966952	1,136790552	1,457364341
CHST15	0,313292814	-0,18498441	0	0,031	1	0,879658582	1,136804689	0
RNF13	0,027793664	-0,184998682	0,594	0,27	1	0,879649879	1,136815935	2,2

Supplementary Table 1

Differentially expressed genes_adjacent-skin-XL_ comparing SC-Keloid vs. (SC-Myel, SC-Nonmyel)								
**gene**	**p_val**	**avg_log2FC**	**pct.1**	**pct.2**	**p_val_adj**	**Foldchange_UP**	**Foldchange_DOWN**	**Ratio_pct1_pct2**
PRKCI	0,24262347	-0,185029585	0,156	0,082	1	0,879631037	1,136840286	1,902439024
EBLN2	0,804543977	-0,185058464	0,031	0,039	1	0,87961343	1,136863043	0,794871795
G2E3	0,445227443	-0,185083032	0,094	0,055	1	0,879598451	1,136882402	1,709090909
CCDC93	0,840130893	-0,185290802	0,062	0,051	1	0,879471784	1,137046143	1,215686275
TWSG1	0,003131851	-0,185341192	0,344	0,113	1	0,879441067	1,137085858	3,044247788
RABEP1	0,017377488	-0,185350686	0,281	0,105	1	0,87943528	1,137093341	2,676190476
WNT2B	0,38481627	-0,185424917	0	0,023	1	0,879390031	1,137151849	0
TOP2B	0,075667481	-0,18548136	0,312	0,152	1	0,879355627	1,137196339	2,052631579
CNN3	0,924341381	-0,18549705	0,938	0,777	1	0,879346064	1,137208707	1,207207207
TAX1BP1	0,102973716	-0,185501675	0,5	0,266	1	0,879343245	1,137212352	1,879699248
PI4KB	0,017422395	-0,185722211	0,219	0,074	1	0,879208835	1,137386205	2,959459459
IMP3	0,059405355	-0,185927776	0,281	0,129	1	0,879083568	1,137548279	2,178294574
PRPF38A	0,011905317	-0,185954595	0,219	0,07	1	0,879067227	1,137569426	3,128571429
CD40	0,584537751	-0,185977937	0,094	0,062	1	0,879053004	1,137587831	1,516129032
CRBN	0,041373501	-0,186093554	0,344	0,152	1	0,87898256	1,137679	2,263157895
TBC1D13	0,098751938	-0,186103499	0,094	0,031	1	0,878976501	1,137686843	3,032258065
GPRASP1	0,730358576	-0,186190699	0,031	0,043	1	0,878923375	1,137755609	0,720930233
ZNF627	0,682170546	-0,186195097	0,031	0,02	1	0,878920695	1,137759078	1,55
DDB2	0,012061903	-0,186314788	0,219	0,07	1	0,878847781	1,137853474	3,128571429
PNPLA8	0,100519352	-0,186381577	0,344	0,18	1	0,878807096	1,137906151	1,911111111
TPD52	0,194053346	-0,186429171	0	0,051	1	0,878778105	1,137943691	0
NPHP4	0,887104347	-0,186544812	0,031	0,035	1	0,878707667	1,138034909	0,885714286
STX4	0,163003711	-0,186572611	0,219	0,113	1	0,878690736	1,138056838	1,938053097
RBM48	0,755689148	-0,186665333	0,062	0,047	1	0,878634264	1,138129983	1,319148936
CCDC142	0,804543977	-0,186768222	0,031	0,039	1	0,878571605	1,138211154	0,794871795
SUMF1	0,108846454	-0,186793413	0,156	0,066	1	0,878556264	1,138231028	2,363636364
ELOA	0,993403306	-0,186881565	0,062	0,059	1	0,878502584	1,138300579	1,050847458
GPAM	0,187685368	-0,186902301	0,094	0,039	1	0,878489957	1,13831694	2,41025641
HERC4	0,222039877	-0,186922761	0,219	0,121	1	0,878477499	1,138333084	1,809917355
HCN1	0,313292814	-0,186929537	0	0,031	1	0,878473373	1,13833843	0

Supplementary Table 1

Differentially expressed genes_adjacent-skin-XL_ comparing SC-Keloid vs. (SC-Myel, SC-Nonmyel)								
**gene**	**p_val**	**avg_log2FC**	**pct.1**	**pct.2**	**p_val_adj**	**Foldchange_UP**	**Foldchange_DOWN**	**Ratio_pct1_pct2**
RIN2	0,023464878	-0,187224626	0,188	0,062	1	0,878293708	1,138571289	3,032258065
RBAK	0,313292814	-0,187262659	0	0,031	1	0,878270554	1,138601306	0
SCAI	0,663576959	-0,187288418	0,062	0,043	1	0,878254873	1,138621635	1,441860465
ZNF12	0,755689148	-0,187311825	0,062	0,047	1	0,878240625	1,138640108	1,319148936
SLC25A16	0,310355006	-0,187374773	0,094	0,047	1	0,878202305	1,138689791	2
UQCC1	0,578217897	-0,187494602	0,062	0,039	1	0,878129366	1,138784374	1,58974359
GNL1	0,024125962	-0,187522118	0,25	0,094	1	0,878112618	1,138806094	2,659574468
HFE	0,428872689	-0,187593711	0	0,02	1	0,878069043	1,138862608	0
PREP	0,066791287	-0,187606236	0,125	0,043	1	0,87806142	1,138872495	2,906976744
GPR37L1	0,234088337	-0,187611493	0	0,043	1	0,87805822	1,138876645	0
PTRH2	0,435431286	-0,187690274	0,094	0,055	1	0,878010274	1,138938837	1,709090909
STAT5A	0,663576959	-0,18770521	0,031	0,047	1	0,878001184	1,138950629	0,659574468
TRMT1L	0,283789128	-0,187845584	0	0,035	1	0,877915758	1,139061454	0
SPEF2	0,487085433	-0,188028112	0,062	0,035	1	0,877804693	1,139205575	1,771428571
MAEA	0,086049964	-0,188117401	0,219	0,098	1	0,877750367	1,139276083	2,234693878
SDCBP	0,007292486	-0,18817866	0,594	0,246	1	0,877713097	1,13932446	2,414634146
NEMP1	0,979143607	-0,188364326	0,031	0,031	1	0,877600148	1,139471094	1
TMEM215	0,346686972	-0,188399944	0	0,027	1	0,877578482	1,139499225	0
CAPN3	0,283789128	-0,188409356	0	0,035	1	0,877572756	1,139506659	0
DUBR	0,30793671	-0,18846772	0,062	0,027	1	0,877537255	1,139552759	2,296296296
ZNF275	0,809794875	-0,188501271	0,031	0,039	1	0,877516847	1,13957926	0,794871795
SLC24A4	0,346686972	-0,188600215	0	0,027	1	0,877456667	1,139657418	0
TBXA2R	0,578217897	-0,188712859	0,062	0,039	1	0,877388159	1,139746405	1,58974359
AK9	0,933712959	-0,189016633	0,062	0,062	1	0,877203435	1,139986416	1
ERMARD	0,578217897	-0,189054287	0,062	0,039	1	0,877180541	1,14001617	1,58974359
CPED1	0,257548576	-0,189083263	0	0,039	1	0,877162923	1,140039067	0
AL035681.1	0,283789128	-0,189085913	0	0,035	1	0,877161312	1,14004116	0
CNNM4	0,730358576	-0,189125944	0,031	0,043	1	0,877136974	1,140072794	0,720930233
CNTNAP3B	0,38481627	-0,189171712	0	0,023	1	0,877109148	1,140108962	0
PTGFRN	0,313292814	-0,189181487	0	0,031	1	0,877103205	1,140116687	0

Supplementary Table 1

Differentially expressed genes_adjacent-skin-XL_ comparing SC-Keloid vs. (SC-Myel, SC-Nonmyel)								
**gene**	**p_val**	**avg_log2FC**	**pct.1**	**pct.2**	**p_val_adj**	**Foldchange_UP**	**Foldchange_DOWN**	**Ratio_pct1_pct2**
SLC35E1	0,108846454	-0,189197994	0,156	0,066	1	0,877093169	1,140129732	2,363636364
FBXO17	0,05263413	-0,189202744	0,25	0,105	1	0,877090281	1,140133486	2,380952381
DEK	0,103213032	-0,189233539	0,656	0,363	1	0,87707156	1,140157823	1,807162534
PTPN1	0,046647979	-0,189277258	0,25	0,105	1	0,877044981	1,140192375	2,380952381
SCYL2	0,112120373	-0,189338224	0,156	0,066	1	0,87700792	1,140240558	2,363636364
NHS	0,428872689	-0,189413633	0	0,02	1	0,87696208	1,14030016	0
COQ10A	0,396289917	-0,189443508	0,062	0,031	1	0,87694392	1,140323773	2
LTV1	0,082633113	-0,189443645	0,156	0,062	1	0,876943837	1,140323882	2,516129032
MARCHF8	0,257548576	-0,189478207	0	0,039	1	0,876922829	1,1403512	0
ZNF106	0,829394279	-0,189580928	0,312	0,25	1	0,876860394	1,140432396	1,248
SECISBP2	0,072066314	-0,189608136	0,25	0,113	1	0,876843857	1,140453905	2,212389381
EFNB2	0,603353082	-0,189610886	0,031	0,051	1	0,876842186	1,140456078	0,607843137
LRRTM4	0,234088337	-0,189666118	0	0,043	1	0,876808617	1,14049974	0
ARHGAP35	0,012941817	-0,189727331	0,281	0,102	1	0,876771415	1,140548132	2,754901961
PAQR8	0,084415941	-0,189750277	0,156	0,062	1	0,87675747	1,140566273	2,516129032
PRDM8	0,313292814	-0,189977756	0	0,031	1	0,876619238	1,140746127	0
CYP2J2	0,313292814	-0,190029124	0	0,031	1	0,876588025	1,140786745	0
TRIM11	0,804543977	-0,190066343	0,031	0,039	1	0,876565411	1,140816175	0,794871795
CNOT10	0,009808643	-0,19012574	0,188	0,055	1	0,876529323	1,140863145	3,418181818
SNX19	0,24851603	-0,190188739	0,094	0,043	1	0,876491048	1,140912965	2,186046512
LIME1	0,924392442	-0,190236081	0,031	0,027	1	0,876462286	1,140950404	1,148148148
ULK2	0,093753684	-0,190247623	0,125	0,047	1	0,876455274	1,140959533	2,659574468
GRK3	0,283789128	-0,190267332	0	0,035	1	0,876443301	1,140975119	0
MSANTD3	0,001998184	-0,190336661	0,406	0,137	1	0,876401184	1,141029951	2,96350365
NEIL1	0,603353082	-0,190364619	0,031	0,051	1	0,876384201	1,141052063	0,607843137
SPTBN5	0,668126075	-0,190462026	0,031	0,047	1	0,876325031	1,141129107	0,659574468
YES1	0,126128812	-0,190571512	0,125	0,051	1	0,87625853	1,14121571	2,450980392
SYNPO	0,037601582	-0,190708477	0,25	0,102	1	0,876175345	1,141324058	2,450980392
RBM22	0,017193636	-0,191216885	0,281	0,105	1	0,875866634	1,141726333	2,676190476
ZNF701	0,924392442	-0,191295976	0,031	0,027	1	0,875818618	1,141788927	1,148148148

Supplementary Table 1

Differentially expressed genes_adjacent-skin-XL_ comparing SC-Keloid vs. (SC-Myel, SC-Nonmyel)								
**gene**	**p_val**	**avg_log2FC**	**pct.1**	**pct.2**	**p_val_adj**	**Foldchange_UP**	**Foldchange_DOWN**	**Ratio_pct1_pct2**
SYT10	0,603353082	-0,1913098	0,031	0,051	1	0,875810226	1,141799868	0,607843137
WEE1	0,901760626	-0,191330632	0,125	0,125	1	0,87579758	1,141816355	1
CEP170	0,008124757	-0,191335708	0,438	0,18	1	0,875794498	1,141820372	2,433333333
DDX56	0,015958056	-0,191372125	0,188	0,059	1	0,875772392	1,141849195	3,186440678
USP6NL	0,147069022	-0,191384602	0,219	0,109	1	0,875764818	1,14185907	2,009174312
CNTN4	0,804543977	-0,191438613	0,031	0,039	1	0,875732032	1,141901819	0,794871795
CLCN6	0,920939129	-0,19150916	0,062	0,055	1	0,87568921	1,141957659	1,127272727
PARD3	0,346694817	-0,191601472	0,125	0,07	1	0,87563318	1,14203073	1,785714286
RUFY3	0,099952249	-0,191625953	0,188	0,082	1	0,875618321	1,14205011	2,292682927
TBK1	0,536449576	-0,191693451	0,031	0,059	1	0,875577356	1,142103543	0,525423729
JAK1	0,278206222	-0,191905037	0,281	0,172	1	0,875448953	1,142271056	1,63372093
ICE2	0,754388196	-0,191917446	0,094	0,074	1	0,875441423	1,142280881	1,27027027
HS1BP3	0,033260329	-0,192147391	0,188	0,066	1	0,875301901	1,142462959	2,848484848
ABCA5	0,668126075	-0,19216295	0,062	0,043	1	0,875292461	1,142475281	1,441860465
ZBTB49	0,150155729	-0,192281512	0,062	0,02	1	0,875220532	1,142569174	3,1
LRBA	0,107772109	-0,192372125	0,156	0,066	1	0,875165562	1,142640939	2,363636364
USP15	0,383262858	-0,19249852	0,188	0,117	1	0,875088892	1,14274105	1,606837607
SREK1IP1	0,000334896	-0,192539575	0,375	0,105	1	0,87506399	1,14277357	3,571428571
VPS35L	0,045196862	-0,192546629	0,125	0,039	1	0,875059712	1,142779158	3,205128205
SREBF2	0,175145722	-0,192559372	0,219	0,113	1	0,875051983	1,142789251	1,938053097
BLOC1S5	0,313292814	-0,192805563	0	0,031	1	0,87490267	1,142984281	0
ZSCAN30	0,663576959	-0,192957045	0,062	0,043	1	0,874810812	1,1431043	1,441860465
QTRT2	0,887104347	-0,193053754	0,031	0,035	1	0,874752171	1,143180929	0,885714286
DAPK3	0,551476222	-0,193079234	0,125	0,086	1	0,874736722	1,143201119	1,453488372
HS6ST1	0,840130759	-0,193230142	0,062	0,051	1	0,874645228	1,143320706	1,215686275
VPS35	0,017012333	-0,193562198	0,344	0,137	1	0,87444394	1,143583888	2,510948905
SPP1	0,682170546	-0,193569131	0,031	0,02	1	0,874439738	1,143589383	1,55
IKZF4	0,346686972	-0,193580551	0	0,027	1	0,874432816	1,143598436	0
FAM199X	0,288304724	-0,193608904	0,156	0,086	1	0,874415631	1,14362091	1,813953488
KBTBD6	0,746521062	-0,193734205	0,062	0,047	1	0,87433969	1,143720241	1,319148936

Supplementary Table 1

Differentially expressed genes_adjacent-skin-XL_ comparing SC-Keloid vs. (SC-Myel, SC-Nonmyel)								
**gene**	**p_val**	**avg_log2FC**	**pct.1**	**pct.2**	**p_val_adj**	**Foldchange_UP**	**Foldchange_DOWN**	**Ratio_pct1_pct2**
AC144831.1	0,428872689	-0,193784897	0	0,02	1	0,874308969	1,143760428	0
HDAC10	0,310355006	-0,193847023	0,094	0,047	1	0,874271319	1,143809683	2
ERO1B	0,109929252	-0,193883366	0,156	0,066	1	0,874249296	1,143838496	2,363636364
METTL14	0,730358576	-0,1938957	0,031	0,043	1	0,874241822	1,143848275	0,720930233
CLIP2	0,788035309	-0,193930005	0,094	0,074	1	0,874221034	1,143875475	1,27027027
ZCCHC3	0,093753684	-0,193944235	0,125	0,047	1	0,874212411	1,143886758	2,659574468
MFSD14C	0,057671435	-0,194005136	0,219	0,09	1	0,874175508	1,143935046	2,433333333
CHD8	0,000845623	-0,194127509	0,281	0,074	1	0,874101362	1,144032081	3,797297297
HLTF	0,024411938	-0,194190901	0,219	0,078	1	0,874062955	1,144082351	2,807692308
MEGF10	0,346686972	-0,194192775	0	0,027	1	0,874061819	1,144083837	0
ST3GAL6-AS1	0,99780108	-0,194195844	0,062	0,059	1	0,87405996	1,144086271	1,050847458
PTP4A2	0,12899053	-0,194221637	0,531	0,293	1	0,874044333	1,144106725	1,812286689
HSD17B7	0,730358576	-0,194455458	0,031	0,043	1	0,873902686	1,144292169	0,720930233
KCNA2	0,741950345	-0,194523924	0,062	0,047	1	0,873861214	1,144346475	1,319148936
PTPN2	0,830197375	-0,194548277	0,125	0,102	1	0,873846464	1,144365791	1,225490196
LRATD2	0,313292814	-0,194622135	0	0,031	1	0,873801728	1,144424379	0
UTP6	0,003327396	-0,194623398	0,281	0,086	1	0,873800964	1,14442538	3,26744186
BMP2	0,376291412	-0,194633393	0,094	0,051	1	0,87379491	1,144433309	1,843137255
ZNF143	0,532167601	-0,194687427	0,125	0,082	1	0,873762184	1,144476172	1,524390244
RIC8B	0,499735776	-0,194758869	0,031	0,059	1	0,873718917	1,144532848	0,525423729
SNX15	0,006461838	-0,194773744	0,188	0,051	1	0,873709908	1,144544649	3,68627451
KIAA0586	0,441948175	-0,194774889	0,094	0,055	1	0,873709214	1,144545558	1,709090909
TRMT10C	0,140813864	-0,194986829	0,156	0,07	1	0,873580871	1,14471371	2,228571429
ZC3H7A	0,064653778	-0,195073296	0,25	0,109	1	0,873528515	1,14478232	2,293577982
HAT1	0,006622186	-0,195157886	0,469	0,184	1	0,873477299	1,144849444	2,548913043
ADAMTS10	0,346686972	-0,19537241	0	0,027	1	0,873347425	1,145019692	0
ADO	0,663576959	-0,195563109	0,031	0,047	1	0,873231992	1,145171053	0,659574468
EIF2B4	0,032569978	-0,195621339	0,219	0,082	1	0,873196747	1,145217276	2,670731707
ENTPD1	0,283789128	-0,195837819	0	0,035	1	0,873065732	1,145389131	0
LRP6	0,012798323	-0,196085038	0,281	0,102	1	0,872916137	1,145585421	2,754901961

Supplementary Table 1

Differentially expressed genes_adjacent-skin-XL_ comparing SC-Keloid vs. (SC-Myel, SC-Nonmyel)								
**gene**	**p_val**	**avg_log2FC**	**pct.1**	**pct.2**	**p_val_adj**	**Foldchange_UP**	**Foldchange_DOWN**	**Ratio_pct1_pct2**
AP1B1	0,902949137	-0,196262801	0,062	0,055	1	0,872808586	1,145726584	1,127272727
HAUS5	0,38481627	-0,196318891	0	0,023	1	0,872774653	1,145771129	0
CACTIN	0,24851603	-0,196364143	0,094	0,043	1	0,872747278	1,145807068	2,186046512
HMCES	0,887104347	-0,196382928	0,031	0,035	1	0,872735914	1,145821988	0,885714286
FAM200B	0,109275924	-0,19639625	0,312	0,156	1	0,872727855	1,145832569	2
RDH14	0,097803604	-0,196414932	0,25	0,117	1	0,872716554	1,145847407	2,136752137
EIF4E3	0,603353082	-0,196519826	0,031	0,051	1	0,872653104	1,145930721	0,607843137
APOA1	0,283789128	-0,196537351	0	0,035	1	0,872642503	1,145944641	0
KIAA1522	0,804543977	-0,196733958	0,031	0,039	1	0,87252359	1,146100818	0,794871795
RNF5	0,21844902	-0,197110371	0,344	0,195	1	0,87229597	1,146399886	1,764102564
HLA-DPB1	0,130767324	-0,197195092	0,156	0,07	1	0,872244746	1,146467209	2,228571429
SCAF8	0,963619739	-0,19731346	0,062	0,062	1	0,872173185	1,146561276	1
CHML	0,38731203	-0,197348138	0,125	0,074	1	0,872152221	1,146588836	1,689189189
CHMP4B	0,116434951	-0,197498637	0,438	0,234	1	0,872061244	1,146708453	1,871794872
TNS4	0,38481627	-0,197511537	0	0,023	1	0,872053447	1,146718706	0
NUP160	0,632215601	-0,197570113	0,125	0,09	1	0,872018041	1,146765266	1,388888889
ACY1	0,989005733	-0,197859198	0,062	0,059	1	0,871843324	1,146995076	1,050847458
DDHD1	0,274387967	-0,197949585	0,156	0,086	1	0,871788704	1,147066939	1,813953488
NOXA1	0,887104347	-0,197996393	0,031	0,035	1	0,871760419	1,147104156	0,885714286
CEP120	0,924392442	-0,198114662	0,031	0,027	1	0,871688957	1,147198197	1,148148148
TMEM129	0,18309726	-0,198175558	0,188	0,094	1	0,871652164	1,147246621	2
DSC3	0,313292814	-0,198183182	0	0,031	1	0,871647558	1,147252684	0
CAPN15	0,213026321	-0,198218824	0	0,047	1	0,871626024	1,147281028	0
PHLPP1	0,730358576	-0,198350957	0,031	0,043	1	0,871546197	1,147386109	0,720930233
PFKFB2	0,887104347	-0,198427426	0,031	0,035	1	0,871500003	1,147446927	0,885714286
CAPN7	0,204730373	-0,198479125	0,219	0,117	1	0,871468773	1,147488046	1,871794872
MAU2	0,438682776	-0,198597335	0,094	0,055	1	0,871397371	1,147582072	1,709090909
ZFR	0,002789771	-0,198607534	0,594	0,227	1	0,871391211	1,147590184	2,616740088
CACNB1	0,887104347	-0,198712576	0,031	0,035	1	0,871327767	1,147673743	0,885714286
SFT2D1	0,248412105	-0,198877291	0,438	0,262	1	0,871228292	1,147804782	1,671755725

Supplementary Table 1

Differentially expressed genes_adjacent-skin-XL_ comparing SC-Keloid vs. (SC-Myel, SC-Nonmyel)								
**gene**	**p_val**	**avg_log2FC**	**pct.1**	**pct.2**	**p_val_adj**	**Foldchange_UP**	**Foldchange_DOWN**	**Ratio_pct1_pct2**
TRIM24	0,709822531	-0,198996706	0,094	0,07	1	0,871156181	1,147899793	1,342857143
ZNF17	0,313292814	-0,198998704	0	0,031	1	0,871154975	1,147901382	0
METTL2B	0,505798338	-0,199107221	0,094	0,059	1	0,871089451	1,147987729	1,593220339
NUP107	0,022581316	-0,199169317	0,188	0,062	1	0,871051958	1,148037142	3,032258065
ATXN2	0,055924511	-0,199206574	0,312	0,141	1	0,871029464	1,148066789	2,212765957
LNPEP	0,054236211	-0,199218728	0,281	0,125	1	0,871022126	1,148076461	2,248
TRIOBP	0,021783242	-0,199278147	0,312	0,125	1	0,870986252	1,148123747	2,496
MTF2	0,079268503	-0,199298826	0,188	0,078	1	0,870973768	1,148140204	2,41025641
ZNF33A	0,299332476	-0,199328182	0,094	0,047	1	0,870956046	1,148163567	2
NCLN	0,010253031	-0,199331143	0,188	0,055	1	0,870954259	1,148165923	3,418181818
RENBP	0,735253332	-0,199360869	0,031	0,043	1	0,870936313	1,148189581	0,720930233
CDRT4	0,38481627	-0,199389403	0	0,023	1	0,870919087	1,14821229	0
IDUA	0,730358576	-0,199437586	0,031	0,043	1	0,870890001	1,148250638	0,720930233
LONP2	0,163022941	-0,199471884	0,188	0,094	1	0,870869297	1,148277937	2
DMAC2L	0,022218422	-0,199553243	0,281	0,109	1	0,870820187	1,148342694	2,577981651
ATP6AP1L	0,480650464	-0,199563183	0	0,016	1	0,870814187	1,148350607	0
ANGPTL4	0,796888971	-0,199586383	0,094	0,109	1	0,870800184	1,148369073	0,862385321
CHPT1	0,210631511	-0,199587814	0,281	0,16	1	0,87079932	1,148370213	1,75625
UBE3A	0,0160657	-0,19970405	0,375	0,152	1	0,870729164	1,148462738	2,467105263
MAP2K1	0,302364288	-0,199715821	0,125	0,066	1	0,870722059	1,148472109	1,893939394
PORCN	0,979143607	-0,199717413	0,031	0,031	1	0,870721099	1,148473376	1
CTBP1	0,007912882	-0,199756861	0,469	0,184	1	0,870697291	1,14850478	2,548913043
SELENOI	0,085310408	-0,199843106	0,25	0,117	1	0,870645241	1,148573441	2,136752137
KLHL28	0,512685017	-0,199909846	0,094	0,059	1	0,870604966	1,148626575	1,593220339
COASY	0,085318957	-0,200051442	0,156	0,062	1	0,870519523	1,148739315	2,516129032
IL13RA1	0,441948175	-0,200084297	0,094	0,055	1	0,870499699	1,148765475	1,709090909
ENTPD3-AS1	0,445227443	-0,20018271	0,094	0,055	1	0,870440319	1,148843841	1,709090909
KRTDAP	0,755688935	-0,200253121	0,062	0,047	1	0,870397839	1,148899912	1,319148936
SUDS3	0,013247171	-0,200390293	0,312	0,117	1	0,870315085	1,149009155	2,666666667
ACVR2B	0,755689148	-0,200557436	0,062	0,047	1	0,870214261	1,149142281	1,319148936

Supplementary Table 1

Differentially expressed genes_adjacent-skin-XL_ comparing SC-Keloid vs. (SC-Myel, SC-Nonmyel)								
**gene**	**p_val**	**avg_log2FC**	**pct.1**	**pct.2**	**p_val_adj**	**Foldchange_UP**	**Foldchange_DOWN**	**Ratio_pct1_pct2**
KANK1	0,821890137	-0,200588521	0,062	0,051	1	0,870195511	1,149167041	1,215686275
PXN	0,048878828	-0,200597808	0,25	0,105	1	0,870189909	1,149174439	2,380952381
MIER3	0,730358576	-0,200779329	0,031	0,043	1	0,870080428	1,149319038	0,720930233
CRIPT	0,048249119	-0,200834825	0,312	0,141	1	0,870046959	1,149363249	2,212765957
SP1	0,165863407	-0,200972039	0,156	0,074	1	0,869964213	1,14947257	2,108108108
TMEM41B	0,296467269	-0,201029125	0,156	0,086	1	0,86992979	1,149518054	1,813953488
MIB2	0,162153727	-0,201163185	0,125	0,055	1	0,869848958	1,149624876	2,272727273
TTF2	0,063925678	-0,201193339	0,094	0,027	1	0,869830777	1,149648905	3,481481481
CDK11A	0,176138873	-0,201291977	0,156	0,074	1	0,869771308	1,14972751	2,108108108
FBLN1	0,165863407	-0,201582561	0,156	0,074	1	0,869596138	1,149959108	2,108108108
RB1	0,000761729	-0,201601172	0,375	0,113	1	0,869584921	1,149973943	3,318584071
ENPP4	0,487085433	-0,201754191	0,062	0,035	1	0,869492693	1,150095921	1,771428571
SNORC	0,38481627	-0,201779972	0	0,023	1	0,869477155	1,150116474	0
OSMR-DT	0,234088337	-0,201835314	0	0,043	1	0,869443803	1,150160593	0
DNAJB2	0,021766407	-0,202278155	0,562	0,25	1	0,869176965	1,150513693	2,248
NELFA	0,021379715	-0,202363521	0,156	0,047	1	0,869125536	1,150581773	3,319148936
MZF1	0,445227443	-0,202391518	0,094	0,055	1	0,86910867	1,150604101	1,709090909
BORCS7	0,171556582	-0,202417697	0,312	0,172	1	0,869092899	1,15062498	1,813953488
MAN2B1	0,020727365	-0,20249627	0,219	0,078	1	0,869045567	1,150687648	2,807692308
TIMM44	0,010665855	-0,202541698	0,344	0,129	1	0,869018203	1,150723882	2,666666667
BRD1	0,003804594	-0,202611239	0,312	0,102	1	0,868976316	1,15077935	3,058823529
BCR	0,376291412	-0,202652087	0,094	0,051	1	0,868951712	1,150811934	1,843137255
ARRDC4	0,965065266	-0,20266582	0,094	0,086	1	0,868943441	1,150822888	1,093023256
KNTC1	0,396289917	-0,202695218	0,062	0,031	1	0,868925734	1,150846339	2
LIFR	0,804543977	-0,202853636	0,031	0,039	1	0,868830325	1,150972717	0,794871795
MTR	0,109465145	-0,202868617	0,344	0,184	1	0,868821303	1,150984669	1,869565217
TCF25	0,138316184	-0,202919656	0,656	0,371	1	0,868790567	1,151025388	1,76819407
OXR1	0,023564102	-0,202984566	0,312	0,129	1	0,868751479	1,151077177	2,418604651
ALDH16A1	0,663576959	-0,202986556	0,031	0,047	1	0,868750281	1,151078765	0,659574468
IL17D	0,234088337	-0,203031345	0	0,043	1	0,86872331	1,151114501	0

Supplementary Table 1

Differentially expressed genes_adjacent-skin-XL_ comparing SC-Keloid vs. (SC-Myel, SC-Nonmyel)								
**gene**	**p_val**	**avg_log2FC**	**pct.1**	**pct.2**	**p_val_adj**	**Foldchange_UP**	**Foldchange_DOWN**	**Ratio_pct1_pct2**
EXOSC8	0,015596966	-0,203257751	0,344	0,137	1	0,86858699	1,151295163	2,510948905
GET1	0,042510141	-0,203390111	0,219	0,086	1	0,868507305	1,151400793	2,546511628
NMB	0,438682776	-0,203423654	0,094	0,055	1	0,868487112	1,151427564	1,709090909
ZNF414	0,354261856	-0,203594341	0,125	0,07	1	0,868384367	1,151563799	1,785714286
SFMBT1	0,257548576	-0,203612371	0	0,039	1	0,868373514	1,15157819	0
PCDH7	0,310355006	-0,203813648	0,094	0,047	1	0,868252372	1,151738863	2
GMFB	0,294914241	-0,203872081	0,281	0,168	1	0,868217206	1,151785512	1,672619048
SMAD5	0,133024088	-0,203885554	0,312	0,164	1	0,868209098	1,151796269	1,902439024
CDK17	0,66298871	-0,203907785	0,125	0,094	1	0,86819572	1,151814017	1,329787234
SGSH	0,804543977	-0,203980116	0,031	0,039	1	0,868152193	1,151871767	0,794871795
COL14A1	0,059009985	-0,204252804	0,656	0,348	1	0,867988116	1,152089506	1,885057471
ZNF670	0,663576959	-0,204351571	0,031	0,047	1	0,867928696	1,152168381	0,659574468
TNFSF13	0,840130893	-0,204363144	0,062	0,051	1	0,867921733	1,152177623	1,215686275
XPR1	0,668126075	-0,204443299	0,062	0,043	1	0,867873514	1,152241639	1,441860465
FCHSD2	0,346686972	-0,204512596	0	0,027	1	0,867831828	1,152296986	0
SLC15A4	0,359363779	-0,204613283	0,125	0,07	1	0,867771264	1,152377409	1,785714286
NCL	0,491718361	-0,204810227	0,719	0,5	1	0,867652812	1,152534731	1,438
ZNF146	0,015980029	-0,205032064	0,219	0,074	1	0,867519406	1,152711966	2,959459459
DCUN1D4	0,204506657	-0,205099194	0,125	0,059	1	0,86747904	1,152765604	2,118644068
DNAAF9	0,49382256	-0,205162781	0,156	0,102	1	0,867440807	1,152816413	1,529411765
VCPKMT	0,730358576	-0,205163449	0,031	0,043	1	0,867440406	1,152816947	0,720930233
BMPR2	0,000564972	-0,205183424	0,438	0,137	1	0,867428396	1,152832908	3,197080292
CADM1	0,60167888	-0,205424724	0,844	0,605	1	0,867283324	1,153025744	1,395041322
CHURC1	0,048262835	-0,205653959	0,594	0,289	1	0,86714553	1,153208966	2,055363322
CYP27A1	0,132721028	-0,205795786	0,25	0,125	1	0,867060287	1,15332234	2
RUSF1	0,121559505	-0,205839888	0,188	0,086	1	0,867033783	1,153357597	2,186046512
KIDINS220	0,051676732	-0,205877201	0,562	0,277	1	0,867011358	1,153387427	2,028880866
USP44	0,194053346	-0,205877563	0	0,051	1	0,867011141	1,153387717	0
SEPTIN8	0,478106391	-0,205907613	0,156	0,102	1	0,866993082	1,153411741	1,529411765
INPP1	0,467546197	-0,205946805	0,219	0,145	1	0,86696953	1,153443075	1,510344828

Supplementary Table 1

Differentially expressed genes_adjacent-skin-XL_ comparing SC-Keloid vs. (SC-Myel, SC-Nonmyel)								
**gene**	**p_val**	**avg_log2FC**	**pct.1**	**pct.2**	**p_val_adj**	**Foldchange_UP**	**Foldchange_DOWN**	**Ratio_pct1_pct2**
ZRSR2	0,131179297	-0,205985523	0,219	0,109	1	0,866946263	1,15347403	2,009174312
MARVELD1	0,015008421	-0,206011735	0,531	0,234	1	0,866930512	1,153494987	2,269230769
BCL2L2	0,376291412	-0,206022511	0,094	0,051	1	0,866924036	1,153503604	1,843137255
RAPGEFL1	0,346686972	-0,206251501	0	0,027	1	0,866786446	1,153686706	0
PCDH1	0,176138873	-0,206270303	0,156	0,074	1	0,866775149	1,153701742	2,108108108
TTLL5	0,045196862	-0,206316666	0,125	0,039	1	0,866747295	1,153738818	3,205128205
SCAPER	0,910865542	-0,206530655	0,125	0,113	1	0,866618743	1,153909961	1,10619469
GID4	0,755689148	-0,206745397	0,062	0,047	1	0,866489759	1,15408173	1,319148936
ME2	0,008636262	-0,206765112	0,438	0,168	1	0,866477918	1,154097501	2,607142857
DENND4C	0,031838434	-0,20684689	0,156	0,051	1	0,866428804	1,154162922	3,058823529
TFRC	0,755689148	-0,206889868	0,062	0,047	1	0,866402993	1,154197306	1,319148936
SLC30A1	0,023464878	-0,207020007	0,188	0,062	1	0,866324842	1,154301425	3,032258065
SRXN1	0,755689148	-0,207109677	0,062	0,047	1	0,866270998	1,154373172	1,319148936
PRDX1	0,267197802	-0,207141403	0,781	0,465	1	0,866251948	1,154398558	1,679569892
ZNF514	0,804543977	-0,207300579	0,031	0,039	1	0,866156378	1,154525933	0,794871795
MCOLN1	0,920939129	-0,207391782	0,062	0,055	1	0,866101624	1,154598921	1,127272727
SLC16A1-AS1	0,730358576	-0,207423991	0,031	0,043	1	0,866082287	1,154624699	0,720930233
MSN	0,022864713	-0,207601197	0,719	0,348	1	0,865975914	1,154766529	2,066091954
RUSC1	0,111020545	-0,207620177	0,156	0,066	1	0,865964521	1,154781722	2,363636364
GTF2A1	0,096333668	-0,207681774	0,188	0,082	1	0,865927548	1,154831027	2,292682927
DMKN	0,449278612	-0,207684796	0,031	0,066	1	0,865925735	1,154833446	0,46969697
NAPB	0,234088337	-0,207753395	0	0,043	1	0,865884561	1,154888359	0
SGK3	0,663576959	-0,207776965	0,031	0,047	1	0,865870416	1,154907226	0,659574468
APBB3	0,840130893	-0,207781312	0,062	0,051	1	0,865867807	1,154910706	1,215686275
H4C3	0,48019894	-0,207934658	0,188	0,129	1	0,865775777	1,15503347	1,457364341
ARMCX6	0,118408245	-0,208385587	0,25	0,121	1	0,865505212	1,155394544	2,066115702
ALDOC	0,573799329	-0,20844463	0,094	0,062	1	0,865469792	1,15544183	1,516129032
HOOK2	0,721008876	-0,208568957	0,094	0,07	1	0,865395211	1,155541407	1,342857143
RNF2	0,033260329	-0,208697798	0,188	0,066	1	0,86531793	1,155644608	2,848484848
RHOT1	0,403286918	-0,208861659	0,125	0,074	1	0,865219653	1,155775873	1,689189189

Supplementary Table 1

Differentially expressed genes_adjacent-skin-XL_ comparing SC-Keloid vs. (SC-Myel, SC-Nonmyel)								
**gene**	**p_val**	**avg_log2FC**	**pct.1**	**pct.2**	**p_val_adj**	**Foldchange_UP**	**Foldchange_DOWN**	**Ratio_pct1_pct2**
PROX1	0,313292814	-0,208879038	0	0,031	1	0,86520923	1,155789796	0
MIIP	0,211587583	-0,208926328	0,188	0,098	1	0,86518087	1,155827682	1,918367347
CACNA2D1	0,924392442	-0,209013645	0,031	0,027	1	0,865128508	1,155897639	1,148148148
STAT5B	0,603353082	-0,209064128	0,031	0,051	1	0,865098236	1,155938087	0,607843137
AKTIP	0,588138226	-0,209164511	0,094	0,062	1	0,865038044	1,15601852	1,516129032
ELF1	0,023456761	-0,209335633	0,406	0,176	1	0,864935446	1,156155647	2,306818182
C2CD3	0,603353082	-0,209423675	0,031	0,051	1	0,864882664	1,156226205	0,607843137
PIBF1	0,1480486	-0,209460751	0,188	0,09	1	0,864860437	1,156255919	2,088888889
TUBE1	0,30757457	-0,209650855	0,094	0,047	1	0,864746483	1,156408289	2
HSD17B12	0,001785675	-0,209807734	0,438	0,148	1	0,864652455	1,156534044	2,959459459
SSPN	0,008602824	-0,210004206	0,375	0,141	1	0,864534711	1,156691556	2,659574468
NT5C2	0,30793671	-0,210051746	0,062	0,027	1	0,864506223	1,156729672	2,296296296
ARL4D	0,007632599	-0,210119638	0,219	0,066	1	0,864465541	1,156784108	3,318181818
UBAP2L	0,095516294	-0,210185432	0,219	0,102	1	0,864426118	1,156836864	2,147058824
KYAT3	0,120509006	-0,21038703	0,188	0,086	1	0,864305334	1,156998529	2,186046512
ACOX1	0,126128812	-0,210507862	0,125	0,051	1	0,864232948	1,157095436	2,450980392
ASPSCR1	0,011905317	-0,210533541	0,219	0,07	1	0,864217565	1,157116032	3,128571429
RNF122	0,313292814	-0,210551113	0	0,031	1	0,864207039	1,157130126	0
GOLGA7	0,102160818	-0,210619399	0,406	0,211	1	0,864166135	1,157184897	1,924170616
UBXN7	0,477649336	-0,210633187	0,188	0,125	1	0,864157876	1,157195956	1,504
FOXK1	0,470914834	-0,210752114	0,125	0,078	1	0,864086643	1,157291353	1,602564103
BOD1L1	0,016618948	-0,211010874	0,562	0,246	1	0,863931676	1,157498941	2,284552846
TXNL4B	0,257548576	-0,211115235	0	0,039	1	0,863869183	1,157582675	0
HNRNPUL2	0,006343661	-0,211380775	0,281	0,094	1	0,863710196	1,157795757	2,989361702
HEBP1	0,755689148	-0,211449735	0,062	0,047	1	0,863668912	1,1578511	1,319148936
SDHD	0,076126362	-0,211524609	0,438	0,211	1	0,86362409	1,157911193	2,075829384
RPS6KA2	0,194053346	-0,211565218	0	0,051	1	0,863599781	1,157943786	0
BMT2	0,804543977	-0,211566813	0,031	0,039	1	0,863598826	1,157945067	0,794871795
DYNC1LI1	0,048731044	-0,211824153	0,344	0,156	1	0,863444795	1,158151633	2,205128205
WBP1	0,077107676	-0,211905101	0,25	0,113	1	0,86339635	1,158216617	2,212389381

Supplementary Table 1

Differentially expressed genes_adjacent-skin-XL_ comparing SC-Keloid vs. (SC-Myel, SC-Nonmyel)								
**gene**	**p_val**	**avg_log2FC**	**pct.1**	**pct.2**	**p_val_adj**	**Foldchange_UP**	**Foldchange_DOWN**	**Ratio_pct1_pct2**
HNRNPF	0,20307892	-0,2119921	0,688	0,398	1	0,863344286	1,158286464	1,728643216
FN3K	0,213026321	-0,212117729	0	0,047	1	0,86326911	1,158387331	0
PRRX2	0,887104347	-0,212124568	0,031	0,035	1	0,863265018	1,158392822	0,885714286
ANKRD44	0,300029356	-0,212329744	0,125	0,066	1	0,863142255	1,158557577	1,893939394
DTNA	0,920939129	-0,212358421	0,062	0,055	1	0,863125098	1,158580607	1,127272727
NBPF15	0,283789128	-0,21246245	0	0,035	1	0,863062863	1,158664151	0
ZFHX4	0,809794875	-0,212579815	0,031	0,039	1	0,862992654	1,158758415	0,794871795
TTC31	0,815054348	-0,212780253	0,031	0,039	1	0,862872765	1,158919415	0,794871795
CDK2AP2	0,010493526	-0,212898172	0,625	0,27	1	0,86280224	1,159014144	2,314814815
AFDN	0,499735776	-0,212899205	0,031	0,059	1	0,862801623	1,159014973	0,525423729
IKZF5	0,173155757	-0,212952193	0,156	0,074	1	0,862769934	1,159057543	2,108108108
CEP350	0,129906113	-0,212968678	0,281	0,145	1	0,862760075	1,159070787	1,937931034
ACIN1	0,021818636	-0,213145951	0,344	0,141	1	0,862654069	1,159213219	2,439716312
PLEKHF2	0,035154895	-0,21321565	0,344	0,148	1	0,862612394	1,159269224	2,324324324
MEX3C	0,01270143	-0,213262419	0,312	0,117	1	0,86258443	1,159306805	2,666666667
TMEM117	0,213026321	-0,213279205	0	0,047	1	0,862574394	1,159320294	0
RYK	0,031443381	-0,213315569	0,25	0,098	1	0,862552653	1,159349515	2,551020408
RBM4	0,071728066	-0,213579132	0,5	0,25	1	0,862395089	1,159561334	2
STX17	0,098798214	-0,213584157	0,219	0,102	1	0,862392085	1,159565373	2,147058824
CCNYL1	0,283789128	-0,213621203	0	0,035	1	0,862369941	1,159595149	0
JPX	0,209711856	-0,21363768	0,375	0,215	1	0,862360092	1,159608393	1,744186047
COL8A2	0,283789128	-0,213674184	0	0,035	1	0,862338272	1,159637734	0
NDST1	0,344129332	-0,21372712	0,156	0,09	1	0,862306631	1,159680285	1,733333333
PPP2R5B	0,293095985	-0,213755635	0,125	0,066	1	0,862289588	1,159703207	1,893939394
EFNA5	0,253529282	-0,21377924	0,125	0,062	1	0,86227548	1,159722181	2,016129032
ZNF236	0,394496075	-0,213872366	0,156	0,094	1	0,862219821	1,159797044	1,659574468
SNHG18	0,416896114	-0,21393677	0,125	0,074	1	0,862181332	1,15984882	1,689189189
PDPR	0,751100713	-0,213990763	0,062	0,047	1	0,862149065	1,159892228	1,319148936
CRTC3	0,007165916	-0,214042943	0,344	0,125	1	0,862117883	1,159934181	2,752
ZNF736	0,80991663	-0,214096977	0,031	0,023	1	0,862085594	1,159977625	1,347826087

Supplementary Table 1

Differentially expressed genes_adjacent-skin-XL_ comparing SC-Keloid vs. (SC-Myel, SC-Nonmyel)								
**gene**	**p_val**	**avg_log2FC**	**pct.1**	**pct.2**	**p_val_adj**	**Foldchange_UP**	**Foldchange_DOWN**	**Ratio_pct1_pct2**
IVD	0,004170278	-0,214345833	0,25	0,074	1	0,861936903	1,160177731	3,378378378
CAV2	0,10309818	-0,214363751	0,438	0,227	1	0,861926198	1,16019214	1,929515419
FAM83F	0,346686972	-0,214373054	0	0,027	1	0,86192064	1,160199621	0
RAD51D	0,376291412	-0,214400384	0,094	0,051	1	0,861904312	1,1602216	1,843137255
DCP2	0,937980621	-0,2144033	0,062	0,062	1	0,86190257	1,160223945	1
PPP6R3	0,001193685	-0,214482772	0,375	0,117	1	0,861855093	1,160287859	3,205128205
GANC	0,993403301	-0,214593812	0,062	0,059	1	0,861788761	1,160377166	1,050847458
SYT8	0,313292814	-0,214661504	0	0,031	1	0,861748326	1,160431613	0
SLC25A51	0,804543977	-0,214949782	0,031	0,039	1	0,861576149	1,160663513	0,794871795
SLC2A8	0,382636471	-0,214986948	0,156	0,094	1	0,861553954	1,160693413	1,659574468
NUP58	0,197902332	-0,215016706	0,219	0,117	1	0,861536183	1,160717355	1,871794872
TMTC2	0,234088337	-0,215017882	0	0,043	1	0,861535481	1,160718301	0
OGDH	0,022768525	-0,215073506	0,219	0,078	1	0,861502265	1,160763054	2,807692308
SRCIN1	0,695157794	-0,215139552	0,156	0,121	1	0,861462827	1,160816195	1,289256198
MTAP	0,296467032	-0,215166108	0,156	0,086	1	0,861446969	1,160837562	1,813953488
MCM5	0,376291412	-0,215318185	0,094	0,051	1	0,861356167	1,160959935	1,843137255
HHIPL1	0,283789128	-0,215410196	0	0,035	1	0,861301234	1,16103398	0
MMP28	0,857462101	-0,215657685	0,094	0,078	1	0,861153494	1,161233168	1,205128205
LUZP1	0,110090245	-0,215701182	0,219	0,102	1	0,861127531	1,16126818	2,147058824
PDRG1	0,06440165	-0,215938401	0,156	0,059	1	0,860985949	1,16145914	2,644067797
TFAP2B	0,150480606	-0,215970126	0,188	0,09	1	0,860967016	1,161484681	2,088888889
RPP14	0,191913299	-0,216032796	0,094	0,039	1	0,860929617	1,161535136	2,41025641
NISCH	0,323961112	-0,216140043	0,188	0,109	1	0,86086562	1,161621485	1,724770642
USP8	0,039103141	-0,216151283	0,25	0,102	1	0,860858913	1,161630536	2,450980392
MADD	0,24851603	-0,21639385	0,094	0,043	1	0,860714185	1,161825862	2,186046512
DSP	0,563155042	-0,216435686	0,094	0,062	1	0,860689225	1,161859554	1,516129032
KRT15	0,887940756	-0,2165191	0,094	0,082	1	0,860639464	1,161926733	1,146341463
CPEB3	0,677260831	-0,216814541	0,031	0,047	1	0,860463236	1,162164701	0,659574468
CNTF	0,283789128	-0,216867825	0	0,035	1	0,860431457	1,162207625	0
LGALS7B	0,989005733	-0,21695859	0,062	0,059	1	0,860377326	1,162280746	1,050847458

Supplementary Table 1

Differentially expressed genes_adjacent-skin-XL_ comparing SC-Keloid vs. (SC-Myel, SC-Nonmyel)								
**gene**	**p_val**	**avg_log2FC**	**pct.1**	**pct.2**	**p_val_adj**	**Foldchange_UP**	**Foldchange_DOWN**	**Ratio_pct1_pct2**
CAMK2B	0,257548576	-0,2170577	0	0,039	1	0,860318222	1,162360595	0
BASP1	0,663576959	-0,217065274	0,031	0,047	1	0,860313706	1,162366697	0,659574468
ZNF267	0,015958056	-0,217218318	0,188	0,059	1	0,860222446	1,16249001	3,186440678
SFR1	0,836825729	-0,217345128	0,125	0,102	1	0,860146838	1,162592195	1,225490196
KRAS	0,105466572	-0,217357027	0,438	0,23	1	0,860139744	1,162601783	1,904347826
CDK2	0,204035935	-0,217411823	0,188	0,098	1	0,860107074	1,162645942	1,918367347
AFMID	0,660402423	-0,217487008	0,094	0,066	1	0,860062252	1,162706534	1,424242424
THOC1	0,024131097	-0,217516121	0,219	0,078	1	0,860044896	1,162729998	2,807692308
KMT5B	0,094564098	-0,217578584	0,188	0,082	1	0,860007661	1,16278034	2,292682927
ARFGAP1	0,12985323	-0,217997863	0,219	0,105	1	0,85975776	1,163118319	2,085714286
TEF	0,548952158	-0,218172047	0,031	0,055	1	0,859653963	1,163258757	0,563636364
SOX13	0,286287991	-0,218251633	0,156	0,086	1	0,859606542	1,163322929	1,813953488
LYNX1	0,420869875	-0,218436533	0,031	0,066	1	0,859496379	1,163472034	0,46969697
SNRPA	0,00587965	-0,218528783	0,344	0,121	1	0,859441422	1,163546431	2,842975207
NRIP2	0,414703427	-0,218567005	0,031	0,066	1	0,859418653	1,163577258	0,46969697
RARG	0,660402423	-0,218569235	0,094	0,066	1	0,859417324	1,163579057	1,424242424
OBSCN	0,346686972	-0,21872714	0	0,027	1	0,859323265	1,163706419	0
CDC42BPB	0,470352044	-0,21876635	0,156	0,102	1	0,859299911	1,163738047	1,529411765
RHOB	0,162099104	-0,218784526	1	0,676	1	0,859289084	1,163752709	1,479289941
PAPPA	0,80991663	-0,218899718	0,031	0,023	1	0,859220477	1,163845632	1,347826087
ALPK1	0,804543977	-0,218902562	0,031	0,039	1	0,859218784	1,163847927	0,794871795
ACSF3	0,376291412	-0,218959944	0,094	0,051	1	0,85918461	1,163894218	1,843137255
CIAO1	0,055641998	-0,218976323	0,281	0,125	1	0,859174855	1,163907433	2,248
TBL1XR1	0,101791565	-0,219030981	0,344	0,172	1	0,859142305	1,163951529	2
LZTS3	0,663576959	-0,219053707	0,031	0,047	1	0,859128771	1,163969865	0,659574468
CNTNAP1	0,234088337	-0,219229517	0	0,043	1	0,859024083	1,164111717	0
CARS1	0,025727869	-0,219287853	0,344	0,145	1	0,858989349	1,164158789	2,372413793
TARBP1	0,197541013	-0,219288877	0,156	0,078	1	0,858988739	1,164159615	2
TXNDC16	0,354261856	-0,219439576	0,125	0,07	1	0,858899017	1,164281226	1,785714286
LYPD3	0,607571545	-0,219456068	0,031	0,051	1	0,858889198	1,164294535	0,607843137

Supplementary Table 1

Differentially expressed genes_adjacent-skin-XL_ comparing SC-Keloid vs. (SC-Myel, SC-Nonmyel)								
**gene**	**p_val**	**avg_log2FC**	**pct.1**	**pct.2**	**p_val_adj**	**Foldchange_UP**	**Foldchange_DOWN**	**Ratio_pct1_pct2**
NSD3	0,111217613	-0,219607102	0,562	0,301	1	0,858799287	1,16441643	1,867109635
ASAP3	0,804543977	-0,219621225	0,031	0,039	1	0,858790879	1,16442783	0,794871795
FAM149B1	0,283789128	-0,219823241	0	0,035	1	0,858670634	1,164590892	0
SIPA1L1	0,635386876	-0,220044913	0,125	0,09	1	0,858538708	1,164769847	1,388888889
FSD1L	0,234088337	-0,220057572	0	0,043	1	0,858531175	1,164780067	0
TGIF1	0,042403887	-0,220171483	0,344	0,152	1	0,858463391	1,164872038	2,263157895
C1RL	0,979143607	-0,220413832	0,031	0,031	1	0,858319195	1,165067734	1
EVC	0,313292814	-0,220676592	0	0,031	1	0,858162882	1,165279949	0
DENND2C	0,38481627	-0,220701679	0	0,023	1	0,85814796	1,165300212	0
ZMYM4	0,233614723	-0,220820482	0,156	0,082	1	0,858077296	1,165396176	1,902439024
MFSD14A	0,462289318	-0,220822061	0,125	0,078	1	0,858076357	1,165397452	1,602564103
NUDCD3	0,010248684	-0,220936034	0,281	0,098	1	0,858008572	1,165489522	2,867346939
RAD21	0,023981535	-0,220977583	0,688	0,309	1	0,857983862	1,165523087	2,226537217
TBC1D17	0,193795898	-0,221001031	0,188	0,098	1	0,857969917	1,165542031	1,918367347
NPRL2	0,668126075	-0,221163294	0,062	0,043	1	0,857873425	1,16567313	1,441860465
SLC30A4	0,730358576	-0,221233715	0,031	0,043	1	0,857831552	1,16573003	0,720930233
PIK3CB	0,176914647	-0,221455068	0	0,055	1	0,857699944	1,165908902	0
NGDN	0,691830399	-0,221557142	0,125	0,094	1	0,857639262	1,165991396	1,329787234
KHSRP	0,093171637	-0,221638115	0,344	0,172	1	0,857591128	1,16605684	2
ZNF24	0,039672007	-0,221646441	0,438	0,207	1	0,857586178	1,166063569	2,115942029
INPP4A	0,979143607	-0,221666979	0,031	0,031	1	0,85757397	1,166080169	1
ERLIN2	0,180319513	-0,221705943	0,188	0,094	1	0,857550809	1,166111663	2
B3GNT2	0,603353082	-0,221823518	0,031	0,051	1	0,857480924	1,166206702	0,607843137
NBEAL2	0,313292814	-0,221898354	0	0,031	1	0,857436445	1,166267197	0
ITGA6	0,566482884	-0,221907918	0,75	0,551	1	0,857430761	1,166274929	1,361161525
MAF	0,037284913	-0,22195374	0,625	0,297	1	0,857403529	1,166311971	2,104377104
ELP2	0,002806326	-0,222024696	0,344	0,113	1	0,85736136	1,166369336	3,044247788
NUMB	0,034609483	-0,222123285	0,312	0,133	1	0,857302773	1,166449044	2,345864662
RNF144A	0,730358576	-0,222125836	0,031	0,043	1	0,857301257	1,166451107	0,720930233
SAFB	0,082278968	-0,222240485	0,281	0,133	1	0,857233131	1,166543806	2,112781955

Supplementary Table 1

Differentially expressed genes_adjacent-skin-XL_ comparing SC-Keloid vs. (SC-Myel, SC-Nonmyel)								
**gene**	**p_val**	**avg_log2FC**	**pct.1**	**pct.2**	**p_val_adj**	**Foldchange_UP**	**Foldchange_DOWN**	**Ratio_pct1_pct2**
HOXC6	0,044299501	-0,222276815	0,219	0,086	1	0,857211545	1,166573183	2,546511628
HDAC3	0,205678783	-0,222377106	0,156	0,078	1	0,857151956	1,166654282	2
YJEFN3	0,346686972	-0,222428501	0	0,027	1	0,857121421	1,166695844	0
OSBPL11	0,588138226	-0,222583456	0,094	0,062	1	0,857029366	1,166821162	1,516129032
C2CD5	0,191913299	-0,22271049	0,094	0,039	1	0,856953905	1,166923908	2,41025641
CDK10	0,045653963	-0,222919122	0,188	0,07	1	0,856829987	1,167092673	2,685714286
ZYG11B	0,060916409	-0,223021354	0,188	0,074	1	0,856769273	1,167175378	2,540540541
TRIM28	0,186249045	-0,223168053	0,438	0,258	1	0,856682158	1,167294067	1,697674419
EDEM3	0,001195376	-0,223241384	0,281	0,078	1	0,856638615	1,1673534	3,602564103
RAB4A	0,052320766	-0,223504938	0,344	0,156	1	0,856482137	1,167566674	2,205128205
FAM3C	0,056829833	-0,223537294	0,688	0,336	1	0,856462928	1,16759286	2,047619048
CD47	0,047527418	-0,223688223	0,562	0,277	1	0,856373333	1,167715015	2,028880866
KRIT1	0,341900128	-0,223705389	0,156	0,09	1	0,856363143	1,16772891	1,733333333
PACRG	0,979143607	-0,223714477	0,031	0,031	1	0,856357749	1,167736266	1
PPIL2	0,40869837	-0,223811756	0,125	0,074	1	0,856300008	1,167815007	1,689189189
SNHG32	0,314485574	-0,223882849	0,594	0,359	1	0,856257812	1,167872556	1,6545961
ZNF410	0,05263413	-0,224063163	0,25	0,105	1	0,856150801	1,168018531	2,380952381
CSKMT	0,128167284	-0,224307351	0,219	0,109	1	0,856005902	1,168216245	2,009174312
PREX2	0,283789128	-0,224314923	0	0,035	1	0,856001409	1,168222376	0
DDX50	0,007259728	-0,224326181	0,281	0,094	1	0,85599473	1,168231492	2,989361702
CTDSP2	0,23387633	-0,22438456	0,281	0,16	1	0,855960092	1,168278766	1,75625
MINDY1	0,161397126	-0,224402847	0	0,059	1	0,855949242	1,168293575	0
FBRS	0,098129635	-0,224633209	0,188	0,082	1	0,85581258	1,168480136	2,292682927
UHRF2	0,057671435	-0,224939595	0,219	0,09	1	0,85563085	1,168728314	2,433333333
PIP5K1A	0,828111391	-0,225017613	0,062	0,07	1	0,85558458	1,168791518	0,885714286
LAMTOR3	0,144457051	-0,225051322	0,188	0,09	1	0,855564589	1,168818827	2,088888889
WDR48	0,499735776	-0,225061947	0,031	0,059	1	0,855558289	1,168827435	0,525423729
GABARAPL1	0,649827026	-0,225073081	0,156	0,113	1	0,855551686	1,168836456	1,380530973
FBXO42	0,556776127	-0,225114577	0,031	0,055	1	0,855527078	1,168870075	0,563636364
WAPL	0,017764736	-0,225291315	0,344	0,141	1	0,855422278	1,169013277	2,439716312

Supplementary Table 1

Differentially expressed genes_adjacent-skin-XL_ comparing SC-Keloid vs. (SC-Myel, SC-Nonmyel)								
**gene**	**p_val**	**avg_log2FC**	**pct.1**	**pct.2**	**p_val_adj**	**Foldchange_UP**	**Foldchange_DOWN**	**Ratio_pct1_pct2**
TOX4	0,021818636	-0,225539038	0,344	0,141	1	0,855275407	1,169214023	2,439716312
SHROOM3	0,257548576	-0,225581101	0	0,039	1	0,855250471	1,169248113	0
TLCD4	0,668126075	-0,22570905	0,031	0,047	1	0,855174624	1,169351816	0,659574468
LAS1L	0,400597364	-0,225712875	0,125	0,074	1	0,855172357	1,169354916	1,689189189
NAPEPLD	0,560708386	-0,225794628	0,031	0,055	1	0,855123898	1,169421182	0,563636364
SLC41A2	0,942250066	-0,225977909	0,062	0,062	1	0,85501527	1,169569755	1
DNAJC3	0,12924202	-0,225993997	0,469	0,254	1	0,855005736	1,169582797	1,846456693
AC011752.1	0,213026321	-0,226185068	0	0,047	1	0,854892506	1,169737708	0
ABRAXAS2	0,30793671	-0,22618964	0,062	0,027	1	0,854889797	1,169741414	2,296296296
PLEKHH3	0,511198329	-0,226261539	0,125	0,082	1	0,854847193	1,169799712	1,524390244
PARP8	0,726697784	-0,226327937	0,062	0,078	1	0,854807851	1,169853552	0,794871795
GOSR1	0,000315631	-0,226448238	0,469	0,145	1	0,854736574	1,169951105	3,234482759
RALGAPA2	0,283789128	-0,226552323	0	0,035	1	0,854674911	1,170035516	0
ADAT2	0,603353082	-0,226660502	0,031	0,051	1	0,854610826	1,170123253	0,607843137
PRKX	0,99780108	-0,226669989	0,062	0,059	1	0,854605206	1,170130948	1,050847458
PLAU	0,310355006	-0,226682862	0,094	0,047	1	0,854597581	1,170141389	2
CXCL2	0,283789128	-0,226832665	0	0,035	1	0,854508848	1,170262897	0
CROCC	0,588138226	-0,227010687	0,094	0,062	1	0,854403412	1,170407311	1,516129032
LZTR1	0,031838434	-0,227070584	0,156	0,051	1	0,85436794	1,170455905	3,058823529
FEM1A	0,840130893	-0,227273751	0,062	0,051	1	0,854247632	1,170620745	1,215686275
CCP110	0,202394917	-0,227342898	0,156	0,078	1	0,85420669	1,170676854	2
ARL17B	0,234088337	-0,227420608	0	0,043	1	0,85416068	1,170739913	0
SPIRE1	0,984065256	-0,227622955	0,156	0,148	1	0,854040887	1,170904128	1,054054054
ADAMTS7	0,037113331	-0,227897469	0,438	0,207	1	0,853878396	1,171126948	2,115942029
DSTYK	0,145646739	-0,228134935	0,188	0,09	1	0,853737861	1,171319729	2,088888889
LIN7C	0,076677794	-0,228152666	0,281	0,133	1	0,853727368	1,171334125	2,112781955
ARTN	0,963619739	-0,228273456	0,062	0,062	1	0,853655893	1,1714322	1
SLC25A48	0,234088337	-0,228523503	0	0,043	1	0,85350795	1,171635249	0
TOGARAM1	0,031451095	-0,228566892	0,281	0,113	1	0,853482282	1,171670486	2,486725664
STEEP1	0,804543977	-0,228627438	0,031	0,039	1	0,853446464	1,17171966	0,794871795

Supplementary Table 1

Differentially expressed genes_adjacent-skin-XL_ comparing SC-Keloid vs. (SC-Myel, SC-Nonmyel)								
**gene**	**p_val**	**avg_log2FC**	**pct.1**	**pct.2**	**p_val_adj**	**Foldchange_UP**	**Foldchange_DOWN**	**Ratio_pct1_pct2**
ALKBH5	0,081948379	-0,228695142	0,312	0,148	1	0,853406414	1,171774648	2,108108108
PRRG3	0,257548576	-0,228894429	0	0,039	1	0,853288537	1,171936522	0
JMY	0,400599564	-0,229034383	0,188	0,117	1	0,853205764	1,172050217	1,606837607
MLF1	0,004528707	-0,229310338	0,344	0,121	1	0,853042581	1,172274424	2,842975207
WFDC2	0,213026321	-0,229444967	0	0,047	1	0,85296298	1,172383824	0
PDZD4	0,836825729	-0,22946239	0,125	0,102	1	0,85295268	1,172397982	1,225490196
FLCN	0,920205922	-0,229554844	0,094	0,094	1	0,85289802	1,172473117	1
GALT	0,212362312	-0,229602397	0,156	0,078	1	0,852869908	1,172511763	2
CCM2	0,003196702	-0,229642135	0,375	0,129	1	0,852846417	1,17254406	2,906976744
ATG14	0,240402432	-0,229773425	0,188	0,102	1	0,852768808	1,17265077	1,843137255
GNE	0,887104347	-0,229832453	0,031	0,035	1	0,852733918	1,172698751	0,885714286
ARHGAP39	0,401035597	-0,229978427	0,031	0,07	1	0,852647642	1,172817412	0,442857143
CA2	0,887104347	-0,230010595	0,031	0,035	1	0,85262863	1,172843562	0,885714286
HOPX	0,428872689	-0,230012647	0	0,02	1	0,852627417	1,172845231	0
LGALS8	0,445227443	-0,230225172	0,094	0,055	1	0,852501825	1,173018016	1,709090909
SCAMP1	0,050620571	-0,230242995	0,281	0,121	1	0,852491293	1,173032508	2,32231405
LYRM9	0,597765819	-0,230306772	0,125	0,086	1	0,852453609	1,173084365	1,453488372
XPOT	0,050727376	-0,230313855	0,25	0,105	1	0,852449423	1,173090124	2,380952381
MBOAT7	0,804306744	-0,23038847	0,062	0,07	1	0,852405336	1,173150797	0,885714286
TAPT1	0,457583888	-0,23043634	0,156	0,102	1	0,852377053	1,173189725	1,529411765
PLEKHM3	0,257548576	-0,230480053	0	0,039	1	0,852351227	1,173225272	0
UBR7	0,097228337	-0,230634286	0,188	0,082	1	0,85226011	1,173350704	2,292682927
GFM1	0,556776127	-0,230906405	0,031	0,055	1	0,852099373	1,17357204	0,563636364
CAMTA1	0,165926701	-0,231082089	0,594	0,348	1	0,851995615	1,17371496	1,706896552
MAP7D1	0,270700266	-0,231185356	0,344	0,207	1	0,851934632	1,173798977	1,661835749
WDR75	0,302364586	-0,231211609	0,125	0,066	1	0,851919129	1,173820337	1,893939394
TLE3	0,080620365	-0,231329941	0,5	0,262	1	0,851849257	1,17391662	1,908396947
TBC1D32	0,887104347	-0,231347509	0,031	0,035	1	0,851838884	1,173930914	0,885714286
STX12	0,202385763	-0,23147405	0,344	0,195	1	0,851764171	1,174033886	1,764102564
C16orf72	0,229370318	-0,231658427	0,219	0,121	1	0,851655322	1,174183938	1,809917355

Supplementary Table 1

Differentially expressed genes_adjacent-skin-XL_ comparing SC-Keloid vs. (SC-Myel, SC-Nonmyel)								
**gene**	**p_val**	**avg_log2FC**	**pct.1**	**pct.2**	**p_val_adj**	**Foldchange_UP**	**Foldchange_DOWN**	**Ratio_pct1_pct2**
ADRA2A	0,283789128	-0,231843482	0	0,035	1	0,851546087	1,174334561	0
DCAF16	0,458482361	-0,231972634	0,031	0,062	1	0,851469859	1,174439693	0,5
PRPF4B	0,263115464	-0,232064759	0,656	0,387	1	0,851415489	1,174514691	1,695090439
CC2D1B	0,548952158	-0,232093061	0,031	0,055	1	0,851398786	1,174537732	0,563636364
ARHGDIG	0,234088337	-0,232157791	0	0,043	1	0,851360587	1,174590432	0
CBX4	0,176138873	-0,232162004	0,156	0,074	1	0,851358101	1,174593862	2,108108108
TTLL4	0,30793671	-0,232328919	0,062	0,027	1	0,851259607	1,174729767	2,296296296
PDLIM5	0,048024678	-0,232412412	0,281	0,121	1	0,851210344	1,174797754	2,32231405
UQCR10	0,101636686	-0,232653114	0,625	0,352	1	0,851068339	1,174993775	1,775568182
ARHGEF25	0,730358576	-0,232666801	0,031	0,043	1	0,851060264	1,175004923	0,720930233
MAP3K2	0,008215074	-0,232705046	0,375	0,145	1	0,851037703	1,175036072	2,586206897
ITM2A	0,503346174	-0,232813571	0,031	0,059	1	0,850973687	1,175124466	0,525423729
FBXW7	0,278218567	-0,232819945	0,281	0,168	1	0,850969928	1,175129658	1,672619048
RIPK2	0,185335556	-0,232852903	0,219	0,113	1	0,850950488	1,175156503	1,938053097
MOSMO	0,598076973	-0,23302229	0,156	0,109	1	0,850850584	1,175294487	1,431192661
CWF19L1	0,38481627	-0,233050242	0	0,023	1	0,850834098	1,175317259	0
FMN2	0,234088337	-0,23332413	0	0,043	1	0,850672588	1,175540407	0
TMC6	0,283789128	-0,233388001	0	0,035	1	0,850634928	1,175592452	0
PSPC1	0,795568952	-0,233485046	0,094	0,074	1	0,85057771	1,175671532	1,27027027
RAP2B	0,40598726	-0,233523208	0,125	0,074	1	0,850555211	1,175702631	1,689189189
RNMT	0,061685881	-0,233704949	0,406	0,203	1	0,850448071	1,175850748	2
TRIP11	0,03715015	-0,233710965	0,344	0,152	1	0,850444524	1,175855651	2,263157895
MOK	0,007740005	-0,233713234	0,219	0,066	1	0,850443187	1,175857501	3,318181818
ENOSF1	0,093753684	-0,233797405	0,125	0,047	1	0,850393571	1,175926105	2,659574468
PSD3	0,144822002	-0,233816656	0,25	0,129	1	0,850382224	1,175941797	1,937984496
RAB33B	0,538237415	-0,233828246	0,125	0,082	1	0,850375392	1,175951244	1,524390244
UBR2	0,142100035	-0,233892662	0,188	0,09	1	0,850337424	1,176003751	2,088888889
NLGN2	0,906381059	-0,233930318	0,125	0,109	1	0,850315229	1,176034447	1,146788991
CAPS2	0,543117005	-0,234179658	0	0,012	1	0,850168282	1,176237718	0
NPC1	0,998160744	-0,234250889	0,094	0,086	1	0,850126308	1,176295794	1,093023256

Supplementary Table 1

Differentially expressed genes_adjacent-skin-XL_ comparing SC-Keloid vs. (SC-Myel, SC-Nonmyel)								
**gene**	**p_val**	**avg_log2FC**	**pct.1**	**pct.2**	**p_val_adj**	**Foldchange_UP**	**Foldchange_DOWN**	**Ratio_pct1_pct2**
YTHDC1	0,642293611	-0,234291644	0,438	0,316	1	0,850102293	1,176329024	1,386075949
CCDC171	0,283789128	-0,234658493	0	0,035	1	0,849886156	1,176628179	0
ANOS1	0,194053346	-0,234692799	0	0,051	1	0,849865947	1,176656158	0
NDUFS1	0,093751344	-0,234732765	0,281	0,137	1	0,849842403	1,176688755	2,051094891
ENGASE	0,213026321	-0,235109133	0	0,047	1	0,849620727	1,176995768	0
EIF4A3	0,097562195	-0,235195707	0,5	0,254	1	0,849569744	1,1770664	1,968503937
IQGAP2	0,692420524	-0,235230296	0,5	0,387	1	0,849549375	1,177094621	1,291989664
EID3	0,548952158	-0,235494711	0,031	0,055	1	0,849393686	1,177310377	0,563636364
IBTK	0,060245342	-0,235542009	0,25	0,109	1	0,849365839	1,177348975	2,293577982
SHFL	0,123752934	-0,235770008	0,312	0,164	1	0,849231619	1,177535054	1,902439024
BMPR1A	0,553563879	-0,235819504	0,062	0,09	1	0,849202484	1,177575454	0,688888889
PRPF18	0,300605972	-0,235941709	0,156	0,086	1	0,849130554	1,177675206	1,813953488
C7orf26	0,396289917	-0,236011881	0,062	0,031	1	0,849089254	1,177732488	2
UBN1	0,588138226	-0,236346678	0,094	0,062	1	0,848892234	1,178005829	1,516129032
USP11	0,170623218	-0,236349942	0,344	0,191	1	0,848890314	1,178008494	1,80104712
H2BC15	0,751100713	-0,236392039	0,062	0,047	1	0,848865544	1,178042868	1,319148936
UCKL1	0,036178394	-0,23640022	0,312	0,133	1	0,84886073	1,178049548	2,345864662
SMG6	0,163771487	-0,236712692	0,125	0,055	1	0,848676896	1,178304729	2,272727273
ODR4	0,060916409	-0,236750168	0,188	0,074	1	0,848654851	1,178335337	2,540540541
ST7L	0,173155757	-0,236852517	0,156	0,074	1	0,848594647	1,178418935	2,108108108
SNTA1	0,255641859	-0,236882928	0,156	0,082	1	0,84857676	1,178443775	1,902439024
HMGCS1	0,033860139	-0,236943591	0,281	0,117	1	0,848541079	1,178493328	2,401709402
TOB2	0,635380935	-0,236983595	0,25	0,188	1	0,84851755	1,178526006	1,329787234
TMEM74B	0,38481627	-0,237231486	0	0,023	1	0,848371766	1,178728524	0
DDX42	0,176396095	-0,237562018	0,219	0,113	1	0,848177421	1,17899861	1,938053097
CFL2	0,059539815	-0,237597075	0,281	0,125	1	0,84815681	1,17902726	2,248
SDE2	0,535198384	-0,237760429	0,125	0,082	1	0,84806078	1,179160767	1,524390244
RPRD2	0,516147046	-0,23780467	0,094	0,059	1	0,848034775	1,179196927	1,593220339
TFG	0,033363079	-0,237882042	0,625	0,289	1	0,847989295	1,179260169	2,162629758
KIAA0408	0,804543977	-0,237956252	0,031	0,039	1	0,847945677	1,17932083	0,794871795

Supplementary Table 1

Differentially expressed genes_adjacent-skin-XL_ comparing SC-Keloid vs. (SC-Myel, SC-Nonmyel)								
**gene**	**p_val**	**avg_log2FC**	**pct.1**	**pct.2**	**p_val_adj**	**Foldchange_UP**	**Foldchange_DOWN**	**Ratio_pct1_pct2**
RGS1	0,346686972	-0,238267084	0	0,027	1	0,847763005	1,179574945	0
PPP1CC	0,015859624	-0,238615415	0,438	0,18	1	0,847558342	1,179859782	2,433333333
MPRIP-AS1	0,799342981	-0,238743692	0,094	0,074	1	0,847482984	1,179964694	1,27027027
MED21	0,28927089	-0,238772536	0,219	0,129	1	0,847466041	1,179988285	1,697674419
ABHD10	0,376291412	-0,238881316	0,094	0,051	1	0,847402144	1,18007726	1,843137255
KANTR	0,313292814	-0,238977729	0	0,031	1	0,847345515	1,180156125	0
SDR42E2	0,603353082	-0,238996926	0,031	0,051	1	0,84733424	1,180171829	0,607843137
TAP2	0,054931246	-0,239023378	0,219	0,09	1	0,847318705	1,180193467	2,433333333
RBM11	0,497163605	-0,239051446	0,062	0,098	1	0,84730222	1,180216428	0,632653061
SYDE2	0,603353082	-0,239057768	0,031	0,051	1	0,847298507	1,1802216	0,607843137
NADK2	0,578217897	-0,239103788	0,062	0,039	1	0,84727148	1,180259248	1,58974359
RFC3	0,06440165	-0,239526914	0,156	0,059	1	0,847023021	1,180605456	2,644067797
LAMA3	0,283789128	-0,239792653	0	0,035	1	0,846867017	1,180822939	0
USP33	0,081640693	-0,239983815	0,281	0,133	1	0,846754812	1,180979412	2,112781955
PRPF6	0,072310646	-0,240064427	0,281	0,129	1	0,8467075	1,181045403	2,178294574
FNBP1	0,235357578	-0,240087898	0,219	0,121	1	0,846693725	1,181064617	1,809917355
HEATR5B	0,079268503	-0,240091307	0,188	0,078	1	0,846691724	1,181067408	2,41025641
SYVN1	0,548952158	-0,240277964	0,031	0,055	1	0,846582186	1,181220225	0,563636364
XPO1	0,058127092	-0,240441978	0,406	0,191	1	0,846485947	1,181354521	2,12565445
NBEAL1	0,195356281	-0,240634466	0,281	0,156	1	0,846373014	1,18151215	1,801282051
SLC25A38	0,516147046	-0,240857124	0,094	0,059	1	0,846242399	1,181694513	1,593220339
C2orf76	0,204506657	-0,240927017	0,125	0,059	1	0,846201403	1,181751763	2,118644068
DGCR6	0,283789128	-0,241457852	0	0,035	1	0,845890104	1,182186665	0
MKNK1	0,253529282	-0,241730639	0,125	0,062	1	0,845730177	1,182410215	2,016129032
SRFBP1	0,279787851	-0,241759961	0,188	0,105	1	0,845712987	1,182434248	1,79047619
FAM20C	0,035482716	-0,241801191	0,281	0,117	1	0,845688818	1,18246804	2,401709402
UBQLN1	0,039640256	-0,241920456	0,312	0,137	1	0,84561891	1,182565797	2,277372263
SLC44A2	0,597547605	-0,242012688	0,375	0,277	1	0,845564851	1,182641401	1,353790614
CNTRL	0,429615562	-0,242036508	0,156	0,098	1	0,84555089	1,182660928	1,591836735
LARP4B	0,579038679	-0,242166807	0,125	0,086	1	0,845474526	1,182767746	1,453488372

Supplementary Table 1

Differentially expressed genes_adjacent-skin-XL_ comparing SC-Keloid vs. (SC-Myel, SC-Nonmyel)								
**gene**	**p_val**	**avg_log2FC**	**pct.1**	**pct.2**	**p_val_adj**	**Foldchange_UP**	**Foldchange_DOWN**	**Ratio_pct1_pct2**
PDK3	0,213026321	-0,242196377	0	0,047	1	0,845457198	1,182791989	0
SCGB2A2	0,804543977	-0,242213467	0,031	0,039	1	0,845447182	1,182806	0,794871795
PNP	0,933712959	-0,24232062	0,062	0,062	1	0,845384391	1,182893854	1
POC5	0,887104347	-0,242511579	0,031	0,035	1	0,845272501	1,183050435	0,885714286
LINC00667	0,730213605	-0,242636612	0,156	0,121	1	0,845199248	1,18315297	1,289256198
LSS	0,359364064	-0,242762006	0,125	0,07	1	0,845125789	1,18325581	1,785714286
CDC40	0,086229766	-0,243043822	0,156	0,062	1	0,844960718	1,18348697	2,516129032
MLLT10	0,387353854	-0,243070425	0,156	0,094	1	0,844945137	1,183508794	1,659574468
RNF11	0,493233094	-0,243158319	0,25	0,168	1	0,844893662	1,183580899	1,488095238
EXD3	0,887104347	-0,243429878	0,031	0,035	1	0,844734643	1,183803705	0,885714286
C1D	0,045258158	-0,243514278	0,344	0,152	1	0,844685226	1,183872962	2,263157895
NRARP	0,436520656	-0,243542813	0,031	0,066	1	0,844668518	1,183896378	0,46969697
FLT3LG	0,887104347	-0,243542924	0,031	0,035	1	0,844668454	1,183896469	0,885714286
PEAK1	0,95305118	-0,243698514	0,094	0,09	1	0,844577364	1,184024155	1,044444444
SERPINB5	0,313292814	-0,243787266	0	0,031	1	0,844525409	1,184096996	0
POLDIP3	0,496843677	-0,24394137	0,188	0,125	1	0,844435204	1,184223485	1,504
MSH3	0,653016738	-0,243945977	0,094	0,066	1	0,844432507	1,184227267	1,424242424
DNAJC19	0,007902696	-0,24417978	0,5	0,199	1	0,84429567	1,184419198	2,512562814
RHOT2	0,308425288	-0,244438399	0,219	0,129	1	0,844144334	1,184631537	1,697674419
ZBTB11	0,217580187	-0,244458511	0,281	0,156	1	0,844132566	1,184648052	1,801282051
CMTM7	0,142110321	-0,244509884	0,156	0,07	1	0,844102508	1,184690236	2,228571429
LURAP1L	0,804543977	-0,244525565	0,031	0,039	1	0,844093333	1,184703114	0,794871795
SHISA9	0,663576959	-0,244648465	0,062	0,043	1	0,84402143	1,18480404	1,441860465
TK2	0,214664135	-0,244704952	0,188	0,098	1	0,843988384	1,184850431	1,918367347
MEG8	0,627437373	-0,244739813	0,094	0,066	1	0,84396799	1,184879061	1,424242424
CPQ	0,178680175	-0,244802926	0,344	0,191	1	0,84393107	1,184930897	1,80104712
USP10	0,138196896	-0,244993767	0,219	0,105	1	0,843819442	1,185087651	2,085714286
ZC3H12A	0,711312846	-0,245088637	0,125	0,094	1	0,843763955	1,185165584	1,329787234
NET1	0,038216718	-0,24512949	0,281	0,117	1	0,843740062	1,185199144	2,401709402
HSPA4L	0,176914647	-0,245284787	0	0,055	1	0,843649244	1,18532673	0

Supplementary Table 1

Differentially expressed genes_adjacent-skin-XL_ comparing SC-Keloid vs. (SC-Myel, SC-Nonmyel)								
**gene**	**p_val**	**avg_log2FC**	**pct.1**	**pct.2**	**p_val_adj**	**Foldchange_UP**	**Foldchange_DOWN**	**Ratio_pct1_pct2**
DDX51	0,804543977	-0,245442824	0,031	0,039	1	0,843556833	1,185456582	0,794871795
TMEM71	0,933712959	-0,245521893	0,062	0,062	1	0,843510602	1,185521554	1
CTSO	0,933712959	-0,245613675	0,062	0,062	1	0,843456941	1,185596978	1
CAT	0,236009066	-0,245776508	0,25	0,141	1	0,843361748	1,1857308	1,773049645
PELI2	0,234088337	-0,245870837	0	0,043	1	0,843306607	1,18580833	0
SMPD4	0,34004375	-0,245884487	0,188	0,113	1	0,843298628	1,18581955	1,663716814
RBM15	0,548952158	-0,246030632	0,031	0,055	1	0,843213206	1,18593968	0,563636364
GNPDA2	0,445227443	-0,246059202	0,094	0,055	1	0,843196509	1,185963165	1,709090909
SOCS7	0,603353082	-0,246115323	0,031	0,051	1	0,843163708	1,186009301	0,607843137
LMO7DN	0,428872689	-0,246266362	0	0,02	1	0,843075441	1,186133473	0
WWP1	0,965906126	-0,246320615	0,125	0,113	1	0,843043737	1,186178079	1,10619469
HLA-DQA1	0,480650464	-0,246352464	0	0,016	1	0,843025126	1,186204265	0
AC116407.2	0,663576959	-0,246459195	0,031	0,047	1	0,842962761	1,186292024	0,659574468
PGM5	0,38481627	-0,246467332	0	0,023	1	0,842958007	1,186298715	0
HNRNPK	0,906160641	-0,246478263	0,875	0,656	1	0,84295162	1,186307703	1,333841463
NSUN6	0,168752127	-0,246510655	0,156	0,074	1	0,842932694	1,186334339	2,108108108
TADA1	0,194053346	-0,246591235	0	0,051	1	0,842885614	1,186400602	0
IFIT1	0,739785061	-0,246918669	0,062	0,074	1	0,842694334	1,186669898	0,837837838
ATG4B	0,074729729	-0,247093985	0,188	0,078	1	0,842591937	1,186814111	2,41025641
HMGXB3	0,028002326	-0,247150017	0,281	0,113	1	0,842559212	1,186860205	2,486725664
GTF2I	0,090030079	-0,247161946	0,75	0,387	1	0,842552246	1,186870019	1,937984496
FGD4	0,31227644	-0,247260901	0,188	0,109	1	0,842494457	1,18695143	1,724770642
AP1AR	0,403286918	-0,247318208	0,125	0,074	1	0,842460992	1,186998579	1,689189189
ANKRD17	0,058657759	-0,247378104	0,25	0,109	1	0,842426016	1,187047861	2,293577982
URB1	0,99780108	-0,247481336	0,062	0,059	1	0,842365738	1,187132803	1,050847458
XAF1	0,062941189	-0,247495757	0,156	0,059	1	0,842357318	1,187144669	2,644067797
PTPN11	0,010105466	-0,247619964	0,312	0,113	1	0,8422848	1,18724688	2,761061947
TNS2	0,618344889	-0,247999292	0,156	0,113	1	0,842063367	1,187559083	1,380530973
ARRDC2	0,396289917	-0,248052135	0,062	0,031	1	0,842032524	1,187602582	2
MBD1	0,043657261	-0,248199477	0,188	0,07	1	0,841946532	1,187723877	2,685714286

Supplementary Table 1

Differentially expressed genes_adjacent-skin-XL_ comparing SC-Keloid vs. (SC-Myel, SC-Nonmyel)								
**gene**	**p_val**	**avg_log2FC**	**pct.1**	**pct.2**	**p_val_adj**	**Foldchange_UP**	**Foldchange_DOWN**	**Ratio_pct1_pct2**
ANKRA2	0,304711877	-0,248215107	0,125	0,066	1	0,841937411	1,187736745	1,893939394
WDFY1	0,445227443	-0,248250235	0,094	0,055	1	0,841916911	1,187765666	1,709090909
DUSP5	0,724751104	-0,248275594	0,094	0,07	1	0,841902112	1,187786544	1,342857143
NVL	0,213026321	-0,248292136	0	0,047	1	0,841892459	1,187800164	0
SCMH1	0,99780108	-0,248431581	0,062	0,059	1	0,841811089	1,187914977	1,050847458
KCTD4	0,257548576	-0,248461754	0	0,039	1	0,841793483	1,187939821	0
LZIC	0,018167812	-0,248490028	0,312	0,121	1	0,841776986	1,187963103	2,578512397
RHEB	0,327964386	-0,248742928	0,688	0,414	1	0,841629438	1,188171368	1,661835749
ATXN1	0,00243148	-0,248859852	0,406	0,141	1	0,84156123	1,188267667	2,879432624
SDAD1	0,516147046	-0,248920952	0,094	0,059	1	0,84152559	1,188317993	1,593220339
CSPP1	0,961390661	-0,248968793	0,094	0,086	1	0,841497685	1,188357399	1,093023256
SLC25A12	0,663576959	-0,248987762	0,031	0,047	1	0,84148662	1,188373024	0,659574468
EPHX1	0,588138226	-0,249166245	0,094	0,062	1	0,841382522	1,188520053	1,516129032
GTF2H1	0,517145205	-0,249167357	0,125	0,082	1	0,841381874	1,188520969	1,524390244
UGDH	0,113147688	-0,249263811	0,25	0,121	1	0,841325624	1,188600432	2,066115702
ERCC6L2	0,074729729	-0,24929154	0,188	0,078	1	0,841309453	1,188623278	2,41025641
MLLT3	0,887104347	-0,249403919	0,031	0,035	1	0,841243922	1,188715869	0,885714286
DNAL1	0,663576959	-0,249638333	0,031	0,047	1	0,841107245	1,188909032	0,659574468
CCDC159	0,902554286	-0,249686305	0,094	0,094	1	0,841079277	1,188948565	1
PBX1	0,773969012	-0,249722239	0,062	0,074	1	0,841058328	1,18897818	0,837837838
EIF4EBP2	0,86908937	-0,249780646	0,062	0,066	1	0,841024279	1,189026316	0,939393939
USP25	0,588138226	-0,249866304	0,094	0,062	1	0,840974346	1,189096915	1,516129032
BAMBI	0,234088337	-0,249967754	0	0,043	1	0,840915211	1,189180535	0
SWT1	0,979143607	-0,249979184	0,031	0,031	1	0,840908548	1,189189957	1
TRIM4	0,603353082	-0,250017549	0,031	0,051	1	0,840886186	1,189221581	0,607843137
FER	0,998397162	-0,25009856	0,125	0,117	1	0,84083897	1,18928836	1,068376068
LINS1	0,191913299	-0,250159632	0,094	0,039	1	0,840803377	1,189338706	2,41025641
SEPTIN1	0,933712959	-0,250219988	0,062	0,062	1	0,840768202	1,189388464	1
DGCR8	0,603353082	-0,25028894	0,031	0,051	1	0,840728019	1,189445311	0,607843137
UBXN2A	0,141002056	-0,250334217	0,312	0,164	1	0,840701634	1,189482641	1,902439024

Supplementary Table 1

Differentially expressed genes_adjacent-skin-XL_ comparing SC-Keloid vs. (SC-Myel, SC-Nonmyel)								
**gene**	**p_val**	**avg_log2FC**	**pct.1**	**pct.2**	**p_val_adj**	**Foldchange_UP**	**Foldchange_DOWN**	**Ratio_pct1_pct2**
LARGE1	0,339977961	-0,250391107	0,188	0,109	1	0,840668484	1,189529546	1,724770642
KAT7	0,057671435	-0,250482835	0,219	0,09	1	0,840615035	1,18960518	2,433333333
ZNF136	0,176914255	-0,25067062	0	0,055	1	0,840505625	1,189760033	0
YPEL3	0,065290575	-0,250715054	0,375	0,18	1	0,840479739	1,189796677	2,083333333
SF3B3	0,25187375	-0,250974458	0,156	0,082	1	0,84032863	1,190010628	1,902439024
GGT7	0,997801078	-0,251027965	0,062	0,059	1	0,840297464	1,190054764	1,050847458
CHST9	0,933712959	-0,251029573	0,062	0,062	1	0,840296527	1,190056091	1
TMEM87A	0,012740243	-0,251053086	0,562	0,242	1	0,840282832	1,190075486	2,32231405
THAP6	0,283789128	-0,251105431	0	0,035	1	0,840252345	1,190118666	0
SLC39A14	0,253529282	-0,251114274	0,125	0,062	1	0,840247195	1,190125961	2,016129032
COL23A1	0,213026321	-0,251216308	0	0,047	1	0,840187771	1,190210135	0
CASK	0,230628924	-0,25126976	0,188	0,102	1	0,840156642	1,190254233	1,843137255
RBM26	0,01480364	-0,251428186	0,375	0,148	1	0,840064388	1,190384945	2,533783784
PNPLA2	0,113235914	-0,25181352	0,344	0,176	1	0,839840042	1,190702932	1,954545455
MPST	0,05964868	-0,25219357	0,312	0,141	1	0,839618832	1,19101664	2,212765957
ATF1	0,864972765	-0,252248277	0,062	0,066	1	0,839586994	1,191061804	0,939393939
CNST	0,720482167	-0,252537782	0,094	0,109	1	0,839418531	1,191300838	0,862385321
CBR3	0,538237843	-0,252626722	0,125	0,082	1	0,839366784	1,191374283	1,524390244
UBN2	0,48331453	-0,252754245	0,156	0,102	1	0,839292594	1,191479595	1,529411765
HELB	0,194053346	-0,252801449	0	0,051	1	0,839265133	1,19151858	0
BIRC6	0,096332388	-0,252848375	0,312	0,152	1	0,839237835	1,191557337	2,052631579
ITFG1	0,077209699	-0,253020772	0,375	0,184	1	0,839137555	1,191699733	2,038043478
PRR12	0,732255237	-0,253136601	0,094	0,07	1	0,839070187	1,191795414	1,342857143
ZNRF3	0,663576959	-0,253183946	0,031	0,047	1	0,839042651	1,191834526	0,659574468
ZNFX1	0,638302587	-0,253355458	0,156	0,113	1	0,838942909	1,191976223	1,380530973
D2HGDH	0,803121702	-0,253394143	0,094	0,074	1	0,838920414	1,192008186	1,27027027
SHOC2	0,125272136	-0,253657177	0,375	0,195	1	0,838767474	1,192225534	1,923076923
LRRC4	0,234088337	-0,254083588	0	0,043	1	0,8385196	1,192577967	0
PPP6R2	0,321994138	-0,254117234	0,188	0,109	1	0,838500045	1,19260578	1,724770642
SYT14	0,161397126	-0,2544985	0	0,059	1	0,838278481	1,192920996	0

Supplementary Table 1

Differentially expressed genes_adjacent-skin-XL_ comparing SC-Keloid vs. (SC-Myel, SC-Nonmyel)								
**gene**	**p_val**	**avg_log2FC**	**pct.1**	**pct.2**	**p_val_adj**	**Foldchange_UP**	**Foldchange_DOWN**	**Ratio_pct1_pct2**
CYS1	0,213026321	-0,254688508	0	0,047	1	0,838168084	1,193078118	0
FAM168B	0,134661836	-0,254794025	0,25	0,125	1	0,838106784	1,193165381	2
U2AF2	0,07974974	-0,254809024	0,281	0,133	1	0,83809807	1,193177787	2,112781955
PPP2CA	0,089906165	-0,254815171	0,594	0,309	1	0,838094499	1,19318287	1,922330097
CRY2	0,607571545	-0,254826714	0,031	0,051	1	0,838087794	1,193192417	0,607843137
CBX6	0,414229703	-0,254940301	0,375	0,242	1	0,838021812	1,193286363	1,549586777
STAT2	0,616461443	-0,255086838	0,125	0,09	1	0,837936697	1,193407574	1,388888889
POR	0,219339222	-0,255321765	0,188	0,098	1	0,837800259	1,193601923	1,918367347
BORCS5	0,124754001	-0,255441767	0,188	0,086	1	0,837730574	1,19370121	2,186046512
DPM3	0,02809504	-0,255494946	0,438	0,191	1	0,837699695	1,193745212	2,293193717
SORCS3	0,257548576	-0,255589621	0	0,039	1	0,837644724	1,193823552	0
POLB	0,788035309	-0,255604245	0,094	0,074	1	0,837636234	1,193835653	1,27027027
TBL1X	0,990803913	-0,255681667	0,094	0,086	1	0,837591283	1,193899722	1,093023256
FXR1	0,279034725	-0,25577955	0,625	0,367	1	0,837534457	1,193980727	1,702997275
STS	0,257548576	-0,255878895	0	0,039	1	0,837476786	1,194062949	0
TMF1	0,276733609	-0,255921991	0,281	0,168	1	0,837451769	1,194098619	1,672619048
CLEC2B	0,864972765	-0,255954539	0,062	0,066	1	0,837432876	1,194125558	0,939393939
PAXX	0,262322083	-0,256004498	0,469	0,281	1	0,837403876	1,19416691	1,669039146
CTNNA3	0,257548576	-0,256139466	0	0,039	1	0,837325539	1,194278633	0
IFI35	0,287162697	-0,256312145	0,25	0,148	1	0,837225324	1,194421587	1,689189189
TRPV1	0,603353082	-0,256483632	0,031	0,051	1	0,837125813	1,194563571	0,607843137
INTS13	0,516147046	-0,256521368	0,094	0,059	1	0,837103916	1,194594818	1,593220339
FBXL3	0,01453664	-0,25672929	0,469	0,195	1	0,836983281	1,194766995	2,405128205
TSPAN8	0,346686972	-0,256793539	0	0,027	1	0,836946007	1,194820205	0
RCOR3	0,569779589	-0,25687327	0,125	0,086	1	0,836899755	1,194886238	1,453488372
LYST	0,002547096	-0,256900377	0,5	0,184	1	0,83688403	1,19490869	2,717391304
RORA	0,730358576	-0,256903326	0,031	0,043	1	0,83688232	1,194911132	0,720930233
RDH5	0,323924664	-0,2569246	0,031	0,078	1	0,836869979	1,194928753	0,397435897
CPLANE1	0,310355006	-0,257190123	0,094	0,047	1	0,83671597	1,195148695	2
SDHA	0,024139484	-0,257327671	0,438	0,184	1	0,836636201	1,195262647	2,380434783

Supplementary Table 1

Differentially expressed genes_adjacent-skin-XL_ comparing SC-Keloid vs. (SC-Myel, SC-Nonmyel)								
gene	p_val	avg_log2FC	pct.1	pct.2	p_val_adj	Foldchange_UP	Foldchange_DOWN	Ratio_pct1_pct2
ETV6	0,208543031	-0,257397753	0,188	0,098	1	0,83659556	1,195320711	1,918367347
LHFPL2	0,163771487	-0,257599195	0,125	0,055	1	0,836478755	1,195487624	2,272727273
ITPRIP	0,455148041	-0,257641475	0,031	0,062	1	0,836454242	1,19552266	0,5
CHTOP	0,025504336	-0,257736042	0,375	0,16	1	0,836399415	1,195601027	2,34375
LSMEM1	0,161397126	-0,257854616	0	0,059	1	0,836330675	1,195699297	0
ANKRD13A	0,603353082	-0,258041953	0,031	0,051	1	0,836222083	1,195854571	0,607843137
PATL1	0,512685318	-0,258275853	0,094	0,059	1	0,836086519	1,196048468	1,593220339
SSBP2	0,59146562	-0,258362678	0,312	0,223	1	0,836036203	1,19612045	1,399103139
MFSD2A	0,603353082	-0,258647586	0,031	0,051	1	0,835871116	1,196356688	0,607843137
APAF1	0,755689148	-0,258795096	0,062	0,047	1	0,835785656	1,196479017	1,319148936
TEAD3	0,250004291	-0,259043024	0,156	0,082	1	0,835642038	1,19668465	1,902439024
ARFGEF2	0,887104347	-0,259049327	0,031	0,035	1	0,835638387	1,196689878	0,885714286
ATPSCKMT	0,751100713	-0,259183325	0,062	0,047	1	0,835560777	1,196801032	1,319148936
NR2C1	0,152942762	-0,259218161	0,188	0,09	1	0,835540601	1,196829932	2,088888889
ARPP19	0,091991809	-0,259515271	0,688	0,352	1	0,835368547	1,197076433	1,954545455
NECTIN1	0,257548576	-0,259646122	0	0,039	1	0,835292783	1,197185012	0
RPS6KB1	0,206379422	-0,259715261	0,125	0,059	1	0,835252753	1,197242387	2,118644068
SAP25	0,979143607	-0,259889117	0,031	0,031	1	0,835152105	1,197386672	1
FAM13B	0,294412282	-0,260086982	0,156	0,086	1	0,835037573	1,197550904	1,813953488
PODN	0,346686972	-0,260201168	0	0,027	1	0,834971484	1,197645692	0
TST	0,872528346	-0,260297367	0,094	0,078	1	0,83491581	1,197725553	1,205128205
FHL1	0,064712409	-0,260386604	0,344	0,164	1	0,834864168	1,19779964	2,097560976
PIAS1	0,983447865	-0,260568929	0,094	0,086	1	0,834758666	1,197951025	1,093023256
OSBPL9	0,081388664	-0,260821158	0,438	0,219	1	0,834612736	1,198160484	2
ING1	0,005703422	-0,260845496	0,531	0,207	1	0,834598657	1,198180697	2,565217391
CBWD5	0,356807214	-0,260866258	0,125	0,07	1	0,834586646	1,19819794	1,785714286
STX11	0,771038875	-0,261080112	0,094	0,102	1	0,834462943	1,198375565	0,921568627
ARHGAP36	0,257548576	-0,261213193	0	0,039	1	0,834385972	1,198486113	0
MTRFR	0,40869837	-0,261214988	0,125	0,074	1	0,834384933	1,198487605	1,689189189
STAM	0,474951714	-0,261258311	0,188	0,121	1	0,834359877	1,198523595	1,553719008

Supplementary Table 1

Differentially expressed genes_adjacent-skin-XL_ comparing SC-Keloid vs. (SC-Myel, SC-Nonmyel)								
**gene**	**p_val**	**avg_log2FC**	**pct.1**	**pct.2**	**p_val_adj**	**Foldchange_UP**	**Foldchange_DOWN**	**Ratio_pct1_pct2**
CREG1	0,100873626	-0,261418775	0,188	0,082	1	0,834267081	1,198656908	2,292682927
SMIM29	0,098415875	-0,261556962	0,312	0,152	1	0,834187175	1,198771726	2,052631579
ZNF821	0,499735776	-0,261612553	0,031	0,059	1	0,834155032	1,198817919	0,525423729
PPP1R3B	0,234088337	-0,261650894	0	0,043	1	0,834132864	1,198849779	0
SELENOO	0,213026321	-0,261843597	0	0,047	1	0,834021455	1,199009922	0
GRK2	0,066489731	-0,261893534	0,219	0,094	1	0,833992587	1,199051424	2,329787234
ZNF121	0,257548576	-0,261925418	0	0,039	1	0,833974156	1,199077924	0
KHDRBS1	0,045833886	-0,26204295	0,594	0,285	1	0,833906218	1,199175614	2,084210526
TNFAIP3	0,015819636	-0,2620588	0,438	0,188	1	0,833897056	1,199188788	2,329787234
DOCK4	0,234088337	-0,262101807	0	0,043	1	0,833872198	1,199224536	0
CUL4B	0,011149113	-0,262113572	0,219	0,07	1	0,833865397	1,199234317	3,128571429
BEX4	0,113552381	-0,262135614	0,281	0,137	1	0,833852657	1,199252639	2,051094891
SNN	0,908376542	-0,262484208	0,125	0,105	1	0,833651201	1,199542445	1,19047619
PDS5A	0,514412332	-0,262680866	0,156	0,105	1	0,833537571	1,19970597	1,485714286
CEP290	0,030583271	-0,262753182	0,188	0,066	1	0,833495791	1,199766107	2,848484848
ARHGAP32	0,603353082	-0,262926793	0,031	0,051	1	0,833395495	1,199910494	0,607843137
POLI	0,933712959	-0,263086418	0,062	0,062	1	0,833303291	1,200043263	1
RFLNA	0,234088337	-0,263186788	0	0,043	1	0,833245319	1,200126754	0
DNAJA4	0,1480486	-0,263353878	0,188	0,09	1	0,833148819	1,200265759	2,088888889
RALGAPA1	0,260995138	-0,263544322	0,25	0,145	1	0,833038846	1,200424211	1,724137931
BCORL1	0,234088337	-0,263802308	0	0,043	1	0,832889894	1,200638893	0
NRCAM	0,377976391	-0,26380554	0,031	0,07	1	0,832888028	1,200641583	0,442857143
BTBD7	0,323405291	-0,263862909	0,219	0,133	1	0,832854909	1,200689327	1,646616541
FLYWCH1	0,920939129	-0,264168071	0,062	0,055	1	0,83267876	1,200943327	1,127272727
PLD6	0,344592621	-0,264203493	0,031	0,074	1	0,832658316	1,200972813	0,418918919
TNFRSF1B	0,234088337	-0,264299106	0	0,043	1	0,832603134	1,201052409	0
WIPI1	0,159627915	-0,26468513	0,219	0,109	1	0,832380383	1,20137382	2,009174312
POMT1	0,730358576	-0,264715698	0,031	0,043	1	0,832362747	1,201399274	0,720930233
NFRKB	0,672687415	-0,264857912	0,031	0,047	1	0,8322807	1,201517709	0,659574468
ORC4	0,728499918	-0,264883432	0,094	0,07	1	0,832265978	1,201538962	1,342857143

Supplementary Table 1

Differentially expressed genes_adjacent-skin-XL_ comparing SC-Keloid vs. (SC-Myel, SC-Nonmyel)								
**gene**	**p_val**	**avg_log2FC**	**pct.1**	**pct.2**	**p_val_adj**	**Foldchange_UP**	**Foldchange_DOWN**	**Ratio_pct1_pct2**
INO80	0,033288532	-0,265001845	0,219	0,082	1	0,83219767	1,201637586	2,670731707
ANKS1A	0,257548576	-0,265016501	0	0,039	1	0,832189216	1,201649794	0
FARSB	0,603353082	-0,265064602	0,031	0,051	1	0,832161471	1,201689858	0,607843137
CLUH	0,660402423	-0,265261643	0,094	0,066	1	0,832047823	1,201853994	1,424242424
TFAP2C	0,173512292	-0,265375141	0,188	0,094	1	0,831982368	1,201948549	2
SNX2	0,422212218	-0,265472549	0,156	0,098	1	0,831926196	1,202029705	1,591836735
PRDM11	0,283789128	-0,265520301	0	0,035	1	0,83189866	1,202069492	0
CHMP2B	0,00368802	-0,265581127	0,625	0,25	1	0,831863587	1,202120174	2,5
PARP2	0,112120373	-0,265668801	0,156	0,066	1	0,831813036	1,20219323	2,363636364
TOMM20	0,926891423	-0,265696369	0,688	0,504	1	0,831797141	1,202216203	1,365079365
CARF	0,376291412	-0,265740446	0,094	0,051	1	0,831771728	1,202252933	1,843137255
LCOR	0,598664857	-0,265804172	0,156	0,113	1	0,831734988	1,20230604	1,380530973
CLASP2	0,404264282	-0,26604443	0,25	0,16	1	0,831596488	1,202506281	1,5625
ATP6V1C1	0,663284905	-0,266079639	0,188	0,137	1	0,831576193	1,202535629	1,372262774
BICRA	0,505798338	-0,266108944	0,094	0,059	1	0,831559302	1,202560056	1,593220339
AKIRIN1	0,047804972	-0,266199187	0,312	0,137	1	0,831507288	1,20263528	2,277372263
ADNP2	0,99780108	-0,266237731	0,062	0,059	1	0,831485073	1,202667411	1,050847458
CAST	0,156386684	-0,26637252	0,562	0,324	1	0,831407392	1,20277978	1,734567901
MAP3K11	0,05488416	-0,266435738	0,406	0,191	1	0,831370961	1,202832487	2,12565445
ELK4	0,076071543	-0,266456052	0,344	0,164	1	0,831359255	1,202849423	2,097560976
PHACTR2	0,67254982	-0,266865086	0,125	0,094	1	0,83112358	1,203190504	1,329787234
RTL8A	0,005851444	-0,266946951	0,469	0,18	1	0,831076421	1,20325878	2,605555556
UBE2E3	0,34115731	-0,266979613	0,469	0,281	1	0,831057605	1,203286022	1,669039146
SESN1	0,109929252	-0,267448185	0,156	0,066	1	0,830787731	1,2036769	2,363636364
ITPR2	0,40598726	-0,267455671	0,125	0,074	1	0,83078342	1,203683146	1,689189189
CSGALNACT2	0,469579464	-0,267472469	0,281	0,191	1	0,830773746	1,203697161	1,471204188
PARL	0,60658408	-0,267533003	0,156	0,109	1	0,830738889	1,203747668	1,431192661
TMEM44-AS1	0,730358576	-0,267582772	0,031	0,043	1	0,830710231	1,203789194	0,720930233
NAIP	0,730358576	-0,267859368	0,031	0,043	1	0,830550981	1,204020009	0,720930233
WWTR1	0,001438879	-0,267914075	0,594	0,215	1	0,830519487	1,204065667	2,762790698

Supplementary Table 1

Differentially expressed genes_adjacent-skin-XL_ comparing SC-Keloid vs. (SC-Myel, SC-Nonmyel)								
**gene**	**p_val**	**avg_log2FC**	**pct.1**	**pct.2**	**p_val_adj**	**Foldchange_UP**	**Foldchange_DOWN**	**Ratio_pct1_pct2**
RBM28	0,142694599	-0,267977677	0,219	0,109	1	0,830482874	1,20411875	2,009174312
USP12	0,337948547	-0,268084028	0,188	0,109	1	0,830421656	1,204207517	1,724770642
SLC43A2	0,99780108	-0,268145836	0,062	0,059	1	0,830386079	1,204259109	1,050847458
DPYD	0,762521971	-0,268459949	0,062	0,074	1	0,830205302	1,204521337	0,837837838
KLF5	0,18833141	-0,268576185	0,281	0,156	1	0,830138416	1,204618387	1,801282051
FAT4	0,194053346	-0,268686256	0	0,051	1	0,830075083	1,204710297	0
LEMD3	0,47380962	-0,268748325	0,125	0,078	1	0,830039372	1,204762128	1,602564103
NTNG1	0,840130893	-0,268851051	0,062	0,051	1	0,829980272	1,204847915	1,215686275
PATZ1	0,499735776	-0,269016836	0,031	0,059	1	0,829884901	1,204986377	0,525423729
NABP1	0,414703427	-0,269068523	0,031	0,066	1	0,829855169	1,205029548	0,46969697
TOPORS	0,428423523	-0,269114477	0,188	0,121	1	0,829828737	1,205067932	1,553719008
FAM161A	0,213026321	-0,269205205	0	0,047	1	0,829776552	1,205143719	0
PPFIBP2	0,867816547	-0,269255788	0,094	0,098	1	0,829747459	1,205185974	0,959183673
EML3	0,516147046	-0,269446567	0,094	0,059	1	0,829637743	1,205345356	1,593220339
ZFP91	0,10042554	-0,269490837	0,344	0,172	1	0,829612285	1,205382344	2
LMNA	0,549394686	-0,269508029	1	0,836	1	0,829602399	1,205396707	1,196172249
WDR86	0,176914647	-0,26974256	0	0,055	1	0,829467546	1,205592678	0
PTPN23	0,202646283	-0,269770245	0,125	0,059	1	0,829451629	1,205615813	2,118644068
ITPKC	0,840130893	-0,269795631	0,062	0,051	1	0,829437034	1,205637027	1,215686275
MFAP3L	0,230078455	-0,269926499	0,031	0,098	1	0,829361798	1,205746397	0,316326531
PRXL2A	0,314221703	-0,270014482	0,031	0,078	1	0,829311221	1,205819932	0,397435897
CDK11B	0,073994318	-0,270106137	0,188	0,078	1	0,829258536	1,205896541	2,41025641
HNRNPA3	0,86445071	-0,27046883	0,844	0,578	1	0,829050087	1,206199741	1,460207612
YEATS2	0,213026321	-0,270514859	0	0,047	1	0,829023637	1,206238225	0
LUC7L	0,0527427	-0,270718194	0,344	0,16	1	0,828906801	1,206408246	2,15
PPP1R3E	0,234088337	-0,270751661	0	0,043	1	0,828887573	1,206436232	0
RBM12B	0,512685318	-0,270757673	0,094	0,059	1	0,828884119	1,206441258	1,593220339
UBE2Z	0,032277048	-0,270767141	0,344	0,148	1	0,828878679	1,206449177	2,324324324
TGS1	0,982137358	-0,270878136	0,125	0,113	1	0,828814911	1,206541999	1,10619469
RP2	0,949444396	-0,27101554	0,094	0,09	1	0,828735978	1,206656917	1,044444444

Supplementary Table 1

Differentially expressed genes_adjacent-skin-XL_ comparing SC-Keloid vs. (SC-Myel, SC-Nonmyel)								
**gene**	**p_val**	**avg_log2FC**	**pct.1**	**pct.2**	**p_val_adj**	**Foldchange_UP**	**Foldchange_DOWN**	**Ratio_pct1_pct2**
PLXNA3	0,03107935	-0,271133647	0,312	0,133	1	0,828668135	1,206755705	2,345864662
TRIM23	0,933712959	-0,271386513	0,062	0,062	1	0,828522905	1,206967236	1
TTBK2	0,804306744	-0,271408043	0,062	0,07	1	0,82851054	1,206985248	0,885714286
RHAG	0,161397126	-0,271430259	0	0,059	1	0,828497782	1,207003834	0
DNAJC13	0,588138226	-0,271733586	0,094	0,062	1	0,828323609	1,207257634	1,516129032
MIGA1	0,02361287	-0,271749692	0,25	0,094	1	0,828314362	1,207271111	2,659574468
KIAA0319L	0,441948175	-0,271877391	0,094	0,055	1	0,828241047	1,207377977	1,709090909
SOX4	0,010984362	-0,272104306	0,906	0,543	1	0,828110787	1,207567895	1,668508287
PNN	0,060413072	-0,272382817	0,594	0,293	1	0,827950937	1,207801037	2,027303754
BIRC2	0,023091141	-0,272420408	0,719	0,34	1	0,827929364	1,207832508	2,114705882
NUP50	0,086229766	-0,272670497	0,156	0,062	1	0,827785856	1,208041902	2,516129032
VPS8	0,445227443	-0,272733055	0,094	0,055	1	0,827749962	1,208094286	1,709090909
ABL1	0,790895329	-0,272739635	0,125	0,098	1	0,827746187	1,208099796	1,275510204
RSRP1	0,042956774	-0,272824139	0,656	0,324	1	0,827697705	1,208170561	2,024691358
NMT2	0,563646362	-0,272953141	0,125	0,086	1	0,827623697	1,208278598	1,453488372
ZSCAN16-AS1	0,01059166	-0,272990789	0,406	0,16	1	0,8276021	1,208310129	2,5375
TUBA4A	0,234088337	-0,27308296	0	0,043	1	0,827549228	1,208387328	0
RPAP3	0,482846351	-0,273231661	0,062	0,035	1	0,827463935	1,208511885	1,771428571
PDCD11	0,304711877	-0,273421246	0,125	0,066	1	0,827355205	1,208670706	1,893939394
SSBP3	0,561023118	-0,27376423	0,25	0,176	1	0,827158534	1,208958088	1,420454545
DDX41	0,830999134	-0,273988642	0,062	0,051	1	0,827029879	1,209146157	1,215686275
AKAP17A	0,598076973	-0,274019469	0,156	0,109	1	0,827012208	1,209171993	1,431192661
GARNL3	0,257548576	-0,274197436	0	0,039	1	0,826910196	1,209321163	0
ZNF438	0,38481627	-0,274218734	0	0,023	1	0,826897989	1,209339016	0
TBC1D16	0,211587385	-0,274260134	0,188	0,098	1	0,82687426	1,20937372	1,918367347
GPR161	0,804543977	-0,274360869	0,031	0,039	1	0,826816527	1,209458166	0,794871795
ARIH2	0,074669904	-0,274765252	0,281	0,129	1	0,826584805	1,209797221	2,178294574
KANSL2	0,084415941	-0,274813837	0,156	0,062	1	0,826556969	1,209837963	2,516129032
ARHGEF11	0,084505016	-0,275250691	0,25	0,113	1	0,826306722	1,210204363	2,212389381
PIP4K2B	0,877333124	-0,275329516	0,062	0,066	1	0,826261576	1,210270487	0,939393939

Supplementary Table 1

Differentially expressed genes_adjacent-skin-XL_ comparing SC-Keloid vs. (SC-Myel, SC-Nonmyel)								
**gene**	**p_val**	**avg_log2FC**	**pct.1**	**pct.2**	**p_val_adj**	**Foldchange_UP**	**Foldchange_DOWN**	**Ratio_pct1_pct2**
ZER1	0,194053346	-0,275461865	0	0,051	1	0,82618578	1,210381519	0
EPS8L2	0,663576959	-0,275530939	0,031	0,047	1	0,826146225	1,210439472	0,659574468
ATL1	0,043846305	-0,27567134	0,219	0,086	1	0,826065829	1,210557276	2,546511628
SNHG10	0,336324902	-0,27581154	0,031	0,078	1	0,825985557	1,210674923	0,397435897
AC018742.1	0,176914647	-0,275947168	0	0,055	1	0,825907909	1,210788744	0
MED28	0,375273435	-0,276183945	0,188	0,113	1	0,825772371	1,210987476	1,663716814
ZFAND2A	0,37314069	-0,276427294	0,188	0,113	1	0,825633095	1,211191758	1,663716814
RRP12	0,346686972	-0,276648164	0	0,027	1	0,825506703	1,211377201	0
ATXN7	0,131758782	-0,276757743	0,25	0,125	1	0,825444005	1,211469214	2
ATG2B	0,368897772	-0,276770569	0,188	0,113	1	0,825436667	1,211479984	1,663716814
AOPEP	0,256694147	-0,276807297	0,75	0,434	1	0,825415653	1,211510826	1,728110599
SMG9	0,023464878	-0,277003724	0,188	0,062	1	0,825303278	1,211675788	3,032258065
SLC19A2	0,376291412	-0,277023653	0,094	0,051	1	0,825291878	1,211692526	1,843137255
POU2F1	0,377954466	-0,277068586	0,156	0,094	1	0,825266174	1,211730265	1,659574468
CREB5	0,003714914	-0,277085281	0,688	0,285	1	0,825256624	1,211744287	2,414035088
TET2	0,449025168	-0,277194629	0,188	0,121	1	0,825194077	1,211836134	1,553719008
H2AX	0,134277149	-0,277470928	0,312	0,168	1	0,825036054	1,212068242	1,857142857
DENND5B	0,897998875	-0,27751157	0,062	0,066	1	0,825012813	1,212102387	0,939393939
NUMBL	0,701170822	-0,277603626	0,062	0,078	1	0,824960172	1,212179732	0,794871795
SLC35D2	0,485930104	-0,277846578	0,156	0,102	1	0,824821259	1,212383882	1,529411765
ZC3H15	0,106737226	-0,277974234	0,531	0,281	1	0,824748278	1,212491165	1,889679715
NUP205	0,603353082	-0,278210877	0,031	0,051	1	0,824613007	1,212690063	0,607843137
ANKZF1	0,257548576	-0,27837366	0	0,039	1	0,824519969	1,212826902	0
NOL4L	0,864972765	-0,278488594	0,062	0,066	1	0,824454285	1,212923528	0,939393939
PCID2	0,072798397	-0,278534483	0,438	0,215	1	0,824428062	1,212962109	2,037209302
GTPBP1	0,683139498	-0,278567028	0,062	0,078	1	0,824409464	1,212989471	0,794871795
ZDHHC7	0,006461838	-0,278614779	0,188	0,051	1	0,824382178	1,21302962	3,68627451
CEP68	0,176914647	-0,278729117	0	0,055	1	0,824316845	1,21312576	0
STX6	0,552857355	-0,279027093	0,031	0,055	1	0,824146608	1,213376346	0,563636364
FBXO25	0,048024678	-0,279114088	0,281	0,121	1	0,824096913	1,213449516	2,32231405

Supplementary Table 1

Differentially expressed genes_adjacent-skin-XL_ comparing SC-Keloid vs. (SC-Myel, SC-Nonmyel)								
**gene**	**p_val**	**avg_log2FC**	**pct.1**	**pct.2**	**p_val_adj**	**Foldchange_UP**	**Foldchange_DOWN**	**Ratio_pct1_pct2**
TERF1	0,159450688	-0,279116138	0,469	0,258	1	0,824095742	1,21345124	1,817829457
NBAS	0,282283175	-0,279515027	0,156	0,086	1	0,82386792	1,213786792	1,813953488
CYB5R2	0,480026373	-0,27956719	0,188	0,125	1	0,823838132	1,21383068	1,504
SHC4	0,00093582	-0,279621296	0,5	0,168	1	0,823807236	1,213876203	2,976190476
ANKRD36	0,0323681	-0,279873301	0,281	0,113	1	0,823663349	1,214088257	2,486725664
PABPN1	0,084307738	-0,279914454	0,562	0,289	1	0,823639854	1,21412289	1,944636678
SLC41A1	0,897998807	-0,279957339	0,062	0,066	1	0,823615372	1,21415898	0,939393939
A2M	0,641749481	-0,2799818	0,125	0,09	1	0,823601407	1,214179567	1,388888889
USP38	0,965440085	-0,280016667	0,125	0,109	1	0,823581503	1,214208912	1,146788991
PREPL	0,219339222	-0,280235943	0,188	0,098	1	0,823456336	1,214393474	1,918367347
RND1	0,213026321	-0,280358977	0	0,047	1	0,823386114	1,214497043	0
PITPNM1	0,92888861	-0,280403123	0,094	0,082	1	0,823360919	1,214534206	1,146341463
CTBS	0,532167601	-0,280413144	0,125	0,082	1	0,8233552	1,214542643	1,524390244
CPEB2	0,620208736	-0,280474136	0,094	0,066	1	0,823320392	1,21459399	1,424242424
NBN	0,039498851	-0,280587725	0,312	0,133	1	0,823255571	1,214689623	2,345864662
VAMP2	0,57400339	-0,280771532	0,656	0,434	1	0,823150691	1,214844392	1,511520737
EHMT1	0,445227443	-0,280825788	0,094	0,055	1	0,823119735	1,214890079	1,709090909
TYMP	0,755689148	-0,280844671	0,062	0,047	1	0,823108961	1,214905981	1,319148936
DNAJA2	0,083274923	-0,280934956	0,375	0,184	1	0,823057452	1,214982013	2,038043478
ANKDD1A	0,057114487	-0,281058921	0,219	0,09	1	0,822986733	1,215086416	2,433333333
SLC23A2	0,585250398	-0,281063156	0,125	0,086	1	0,822984317	1,215089983	1,453488372
PDE1C	0,296467269	-0,281356327	0,156	0,086	1	0,822817095	1,215336927	1,813953488
PODXL2	0,176914647	-0,281420756	0	0,055	1	0,82278035	1,215391204	0
PITRM1	0,416896114	-0,281463094	0,125	0,074	1	0,822756205	1,215426872	1,689189189
MAST3	0,257548576	-0,281573534	0	0,039	1	0,822693224	1,215519918	0
HIPK1	0,162173229	-0,281670546	0,281	0,148	1	0,822637905	1,215601656	1,898648649
OSER1	0,869020862	-0,281712439	0,219	0,18	1	0,822614018	1,215636956	1,216666667
NFKB1	0,018778522	-0,281719196	0,375	0,152	1	0,822610165	1,215642649	2,467105263
ITM2B	0,38991927	-0,281787516	0,938	0,82	1	0,82257121	1,215700218	1,143902439
DAB2IP	0,877924569	-0,28197572	0,094	0,094	1	0,822463911	1,21585882	1

Supplementary Table 1

Differentially expressed genes_adjacent-skin-XL_ comparing SC-Keloid vs. (SC-Myel, SC-Nonmyel)								
**gene**	**p_val**	**avg_log2FC**	**pct.1**	**pct.2**	**p_val_adj**	**Foldchange_UP**	**Foldchange_DOWN**	**Ratio_pct1_pct2**
GNRH1	0,442873529	-0,282120151	0,031	0,066	1	0,822381576	1,215980548	0,46969697
NADK	0,588138226	-0,282301702	0,094	0,062	1	0,822278093	1,216133579	1,516129032
CLCF1	0,548952158	-0,282596534	0,031	0,055	1	0,822110067	1,216382136	0,563636364
HDAC4	0,455148041	-0,28265252	0,031	0,062	1	0,822078165	1,216429341	0,5
TMEM205	0,042345745	-0,282705788	0,375	0,168	1	0,822047812	1,216474255	2,232142857
CDC14B	0,185335556	-0,282882734	0,219	0,113	1	0,821946995	1,216623464	1,938053097
RNF145	0,107553188	-0,282979438	0,438	0,227	1	0,821891901	1,216705017	1,929515419
WDR43	0,279787851	-0,283097789	0,188	0,105	1	0,821824481	1,216804833	1,79047619
CBX7	0,566708997	-0,283277798	0,125	0,086	1	0,821721946	1,216956667	1,453488372
DMTF1	0,933712959	-0,283356386	0,062	0,062	1	0,821677185	1,21702296	1
TMEM94	0,99780108	-0,28385413	0,062	0,059	1	0,821393747	1,217442918	1,050847458
CREB3L2	0,135673048	-0,283865476	0,531	0,289	1	0,821387288	1,217452492	1,837370242
CENPT	0,073603171	-0,283969089	0,219	0,094	1	0,821328298	1,217539931	2,329787234
KMT2E-AS1	0,720482167	-0,283972282	0,094	0,109	1	0,821326481	1,217542626	0,862385321
RNPC3	0,901654262	-0,284054822	0,156	0,137	1	0,821279492	1,217612286	1,138686131
EIF3J-DT	0,99780108	-0,284056863	0,062	0,059	1	0,82127833	1,217614009	1,050847458
CALCOCO2	0,095645788	-0,284107883	0,312	0,152	1	0,821249287	1,21765707	2,052631579
H1-0	0,006188545	-0,284267015	0,594	0,238	1	0,821158706	1,217791388	2,495798319
DIS3L2	0,445227443	-0,284403198	0,094	0,055	1	0,821081196	1,217906347	1,709090909
SF3A2	0,081340621	-0,284736001	0,312	0,148	1	0,82089181	1,218187327	2,108108108
MPP7	0,176914647	-0,284893021	0	0,055	1	0,820802471	1,218319919	0
PURA	0,294977518	-0,285009472	0,562	0,355	1	0,82073622	1,218418263	1,583098592
TJAP1	0,337469503	-0,28537279	0,156	0,09	1	0,820529558	1,21872514	1,733333333
TAF4B	0,730358576	-0,285463916	0,031	0,043	1	0,820477732	1,218802121	0,720930233
TRABD2B	0,685922766	-0,285583975	0,094	0,113	1	0,820409456	1,218903552	0,831858407
INPP5E	0,139907281	-0,285600967	0,094	0,035	1	0,820399793	1,218917909	2,685714286
FEM1C	0,004000281	-0,28566122	0,469	0,172	1	0,820365531	1,218968816	2,726744186
WDR47	0,189793134	-0,285744032	0,25	0,133	1	0,820318442	1,219038789	1,879699248
MAP2K7	0,940093621	-0,285871345	0,094	0,082	1	0,820246055	1,21914637	1,146341463
ARAF	0,905060272	-0,286071261	0,125	0,105	1	0,8201324	1,21931532	1,19047619

Supplementary Table 1

Differentially expressed genes_adjacent-skin-XL_ comparing SC-Keloid vs. (SC-Myel, SC-Nonmyel)								
**gene**	**p_val**	**avg_log2FC**	**pct.1**	**pct.2**	**p_val_adj**	**Foldchange_UP**	**Foldchange_DOWN**	**Ratio_pct1_pct2**
PIAS4	0,840130893	-0,286262267	0,062	0,051	1	0,820023826	1,219476762	1,215686275
PIGA	0,176914647	-0,286487653	0	0,055	1	0,819895727	1,219667291	0
METTL7A	0,283789128	-0,286519672	0	0,035	1	0,81987753	1,21969436	0
FUS	0,582606868	-0,286521935	0,844	0,566	1	0,819876244	1,219696274	1,491166078
EDA	0,283789128	-0,287315603	0	0,035	1	0,819425331	1,220367448	0
UFL1	0,644568418	-0,287595142	0,219	0,16	1	0,819266573	1,220603931	1,36875
ARMC8	0,544122042	-0,287652705	0,156	0,105	1	0,819233885	1,220652634	1,485714286
RHOJ	0,217772806	-0,287879815	0,188	0,098	1	0,819104931	1,220844805	1,918367347
WIPF2	0,163189943	-0,287940224	0,219	0,109	1	0,819070634	1,220895925	2,009174312
CCDC30	0,346686972	-0,287993501	0	0,027	1	0,819040387	1,220941013	0
FBXO2	0,194053346	-0,28828185	0	0,051	1	0,818876704	1,221185064	0
ADCY6	0,416896114	-0,288302181	0,125	0,074	1	0,818865164	1,221202274	1,689189189
LRRTM1	0,161397126	-0,288504252	0	0,059	1	0,818750478	1,221373333	0
SRSF1	0,061665482	-0,288807348	0,469	0,234	1	0,818578484	1,221629959	2,004273504
TMEM38B	0,826887692	-0,288863318	0,125	0,102	1	0,818546728	1,221677354	1,225490196
BBS2	0,466259327	-0,289030156	0,219	0,148	1	0,818452073	1,221818641	1,47972973
NUMA1	0,183857413	-0,289271509	0,344	0,191	1	0,818315163	1,22202306	1,80104712
ATP6V0A1	0,864371301	-0,289391173	0,094	0,098	1	0,818247292	1,222124424	0,959183673
MIR4500HG	0,257548576	-0,289398679	0	0,039	1	0,818243034	1,222130782	0
MPHOSPH8	0,06072671	-0,289526405	0,594	0,297	1	0,818170596	1,222238986	2
RPRD1B	0,194053346	-0,289846344	0	0,051	1	0,817989175	1,222510066	0
ST6GALNAC3	0,455148041	-0,289848138	0,031	0,062	1	0,817988158	1,222511586	0,5
TMCO3	0,538237843	-0,289914906	0,125	0,082	1	0,817950302	1,222568166	1,524390244
WASHC4	0,006677744	-0,290011453	0,406	0,152	1	0,817895566	1,222649984	2,671052632
HILPDA	0,588367824	-0,290023743	0,125	0,086	1	0,817888598	1,2226604	1,453488372
TJP1	0,261947676	-0,290154748	0,406	0,242	1	0,817814332	1,222771429	1,67768595
RPGR	0,33089308	-0,29077809	0,156	0,09	1	0,817461058	1,223299863	1,733333333
IFNAR2	0,067002187	-0,290992886	0,312	0,145	1	0,817339359	1,223482008	2,151724138
RNF6	0,160548057	-0,291015556	0,125	0,055	1	0,817326516	1,223501233	2,272727273
NUP62	0,045653963	-0,291094179	0,188	0,07	1	0,817281975	1,223567913	2,685714286

Supplementary Table 1

Differentially expressed genes_adjacent-skin-XL_ comparing SC-Keloid vs. (SC-Myel, SC-Nonmyel)								
**gene**	**p_val**	**avg_log2FC**	**pct.1**	**pct.2**	**p_val_adj**	**Foldchange_UP**	**Foldchange_DOWN**	**Ratio_pct1_pct2**
ZDHHC2	0,945716537	-0,291292777	0,125	0,109	1	0,817169477	1,223736358	1,146788991
KLHDC2	0,142790542	-0,291351916	0,25	0,129	1	0,81713598	1,223786523	1,937984496
FBXO44	0,359364064	-0,291369279	0,125	0,07	1	0,817126146	1,223801251	1,785714286
BEST1	0,864972765	-0,291469217	0,062	0,066	1	0,817069545	1,223886028	0,939393939
YOD1	0,723616565	-0,291763865	0,094	0,109	1	0,816902688	1,224136014	0,862385321
MXD4	0,224684614	-0,291796482	0,375	0,219	1	0,816884219	1,22416369	1,712328767
KCNK5	0,176914647	-0,292280714	0	0,055	1	0,816610083	1,224574642	0
ZFYVE26	0,283789128	-0,292332706	0	0,035	1	0,816580654	1,224618775	0
ALDH3A1	0,283789128	-0,292337122	0	0,035	1	0,816578155	1,224622523	0
GBF1	0,253529282	-0,292454796	0,125	0,062	1	0,816511553	1,224722414	2,016129032
RPRD1A	0,571446987	-0,292467526	0,188	0,129	1	0,816504348	1,22473322	1,457364341
SLC16A4	0,780949248	-0,292561244	0,125	0,098	1	0,816451309	1,224812783	1,275510204
APPBP2	0,045653963	-0,292979021	0,188	0,07	1	0,816214915	1,225167516	2,685714286
VPS36	0,280295079	-0,293142812	0,156	0,086	1	0,816122254	1,225306619	1,813953488
TMEM43	0,488339982	-0,293250453	0,219	0,148	1	0,816061365	1,225398044	1,47972973
SOX15	0,194053346	-0,293308227	0	0,051	1	0,816028686	1,225447117	0
PACSIN3	0,94223401	-0,293328573	0,094	0,09	1	0,816017178	1,225464399	1,044444444
GOLM2	0,111559249	-0,293597755	0,312	0,156	1	0,815864937	1,225693071	2
IL6R	0,314221703	-0,293707076	0,031	0,078	1	0,815803117	1,225785952	0,397435897
FBXO3	0,425400508	-0,293893702	0,219	0,141	1	0,815697592	1,225944529	1,553191489
ILF3-DT	0,411420229	-0,294031617	0,125	0,074	1	0,815619619	1,226061729	1,689189189
ZNF37A	0,06440165	-0,294070495	0,156	0,059	1	0,815597639	1,22609477	2,644067797
VSIR	0,820932332	-0,294094027	0,094	0,102	1	0,815584336	1,226114769	0,921568627
RBP7	0,161397126	-0,294122474	0	0,059	1	0,815568255	1,226138945	0
AGAP3	0,585250398	-0,294307032	0,125	0,086	1	0,815463929	1,22629581	1,453488372
CKAP5	0,017422452	-0,294419634	0,219	0,074	1	0,815400285	1,226391527	2,959459459
ELK3	0,045138424	-0,294519762	0,281	0,121	1	0,815343695	1,226476645	2,32231405
ZNF248	0,499735776	-0,294766097	0,031	0,059	1	0,81520449	1,226686079	0,525423729
PCED1A	0,214057198	-0,294824684	0,156	0,078	1	0,815171386	1,226735895	2
TRPM3	0,234088337	-0,294903322	0	0,043	1	0,815126954	1,226802764	0

Supplementary Table 1

Differentially expressed genes_adjacent-skin-XL_ comparing SC-Keloid vs. (SC-Myel, SC-Nonmyel)								
**gene**	**p_val**	**avg_log2FC**	**pct.1**	**pct.2**	**p_val_adj**	**Foldchange_UP**	**Foldchange_DOWN**	**Ratio_pct1_pct2**
CCNG2	0,344592621	-0,2949236	0,031	0,074	1	0,815115497	1,226820008	0,418918919
TBC1D14	0,414703427	-0,294984103	0,031	0,066	1	0,815081314	1,226871459	0,46969697
NAF1	0,286570971	-0,295006806	0,031	0,082	1	0,815068487	1,226890765	0,37804878
INTS6L	0,283789128	-0,295148968	0	0,035	1	0,814988175	1,227011668	0
ARHGAP26	0,414703427	-0,295203953	0,031	0,066	1	0,814957114	1,227058433	0,46969697
SLC27A1	0,080736527	-0,295221748	0,312	0,148	1	0,814947062	1,227073569	2,108108108
NR1D1	0,69506376	-0,295221864	0,125	0,094	1	0,814946997	1,227073667	1,329787234
PHF8	0,499735776	-0,295226733	0,031	0,059	1	0,814944246	1,227077809	0,525423729
DOCK9	0,603353082	-0,295447292	0,031	0,051	1	0,814819667	1,227265419	0,607843137
FST	0,089395838	-0,295498689	0,344	0,172	1	0,814790639	1,227309142	2
GOLGB1	0,043864058	-0,295504457	0,406	0,188	1	0,814787382	1,227314048	2,159574468
ABCD4	0,952287961	-0,295981833	0,125	0,109	1	0,81451782	1,227720224	1,146788991
EPHB3	0,933712959	-0,296006501	0,062	0,062	1	0,814503893	1,227741217	1
NPR3	0,38481627	-0,296009064	0	0,023	1	0,814502446	1,227743397	0
ISCU	0,014540929	-0,296119381	0,625	0,27	1	0,814440166	1,227837282	2,314814815
CPSF7	0,139772668	-0,296788436	0,188	0,09	1	0,814062555	1,228406827	2,088888889
OGFRL1	0,933712959	-0,297022164	0,062	0,062	1	0,813930681	1,228605855	1
PPRC1	0,864972765	-0,297182548	0,062	0,066	1	0,813840201	1,228742447	0,939393939
OTUD4	0,50170566	-0,297385524	0,188	0,125	1	0,813725708	1,228915334	1,504
TJP2	0,548952158	-0,29757679	0,031	0,055	1	0,813617836	1,229078268	0,563636364
SOCS6	0,253529282	-0,297680582	0,125	0,062	1	0,813559303	1,229166695	2,016129032
MOB1B	0,310355006	-0,297912725	0,094	0,047	1	0,813428404	1,229364495	2
MBD6	0,898658481	-0,298121437	0,125	0,125	1	0,813310736	1,229542358	1
PIAS3	1	-0,298175801	0,094	0,086	1	0,813280089	1,229588691	1,093023256
TLK2	0,233850664	-0,298205202	0,219	0,121	1	0,813263515	1,229613749	1,809917355
GSE1	0,588138226	-0,29822074	0,094	0,062	1	0,813254756	1,229626993	1,516129032
KIAA0100	0,109929252	-0,298410073	0,156	0,066	1	0,813148035	1,229788374	2,363636364
EIF2AK3	0,998160744	-0,298593609	0,094	0,086	1	0,813044595	1,229944834	1,093023256
HTT	0,442120615	-0,298595926	0,156	0,098	1	0,813043289	1,22994681	1,591836735
CPT1A	0,499735776	-0,298860255	0,031	0,059	1	0,812894338	1,23017218	0,525423729

Supplementary Table 1

Differentially expressed genes_adjacent-skin-XL_ comparing SC-Keloid vs. (SC-Myel, SC-Nonmyel)								
**gene**	**p_val**	**avg_log2FC**	**pct.1**	**pct.2**	**p_val_adj**	**Foldchange_UP**	**Foldchange_DOWN**	**Ratio_pct1_pct2**
MORC4	0,455148041	-0,298883322	0,031	0,062	1	0,812881341	1,230191849	0,5
TNKS	0,304070194	-0,299025523	0,219	0,125	1	0,812801223	1,23031311	1,752
THUMPD2	0,755689148	-0,299162593	0,062	0,047	1	0,812724002	1,230430008	1,319148936
MSX1	0,468029835	-0,299210479	0,125	0,078	1	0,812697027	1,230470848	1,602564103
SETDB2	0,234088337	-0,299586818	0	0,043	1	0,812485056	1,230791869	0
CHD7	0,094284344	-0,299687664	0,312	0,152	1	0,812428264	1,230877906	2,052631579
CLIP4	0,499735776	-0,299780397	0,031	0,059	1	0,812376044	1,230957027	0,525423729
ATG13	0,872528346	-0,299882398	0,094	0,078	1	0,81231861	1,23104406	1,205128205
TRAPPC8	0,445227443	-0,299915951	0,094	0,055	1	0,812299718	1,231072691	1,709090909
ORC3	0,170210568	-0,300158404	0,156	0,074	1	0,812163218	1,231279597	2,108108108
TFE3	0,92142629	-0,30029037	0,094	0,082	1	0,812088932	1,231392229	1,146341463
KAT2A	0,377976391	-0,300887327	0,031	0,07	1	0,811752976	1,231901859	0,442857143
ZNF264	0,663576959	-0,300906887	0,031	0,047	1	0,81174197	1,231918562	0,659574468
NEGR1	0,740472812	-0,300937937	0,094	0,105	1	0,8117245	1,231945076	0,895238095
HMGN5	0,653016738	-0,300958741	0,094	0,066	1	0,811712794	1,231962841	1,424242424
RPL38	0,721966631	-0,301022549	0,938	0,793	1	0,811676895	1,232017329	1,182849937
CGAS	0,399301517	-0,301084511	0,156	0,094	1	0,811642035	1,232070244	1,659574468
DCP1A	0,455618878	-0,301113693	0,25	0,168	1	0,811625618	1,232095166	1,488095238
SLC7A2	0,720482167	-0,30137924	0,094	0,109	1	0,811476241	1,23232197	0,862385321
TIMP3	0,611112677	-0,301403221	0,875	0,637	1	0,811462752	1,232342454	1,373626374
PTAR1	0,079303382	-0,301414206	0,406	0,203	1	0,811456574	1,232351838	2
NPIPB5	0,891985815	-0,301435717	0,094	0,094	1	0,811444475	1,232370212	1
CHKA	0,932622158	-0,301490433	0,094	0,082	1	0,811413701	1,232416952	1,146341463
OSGIN2	0,949001794	-0,301512955	0,125	0,109	1	0,811401033	1,232436192	1,146788991
H1-2	0,508238341	-0,301635615	0,125	0,082	1	0,81133205	1,232540981	1,524390244
PHRF1	0,499735776	-0,3016683	0,031	0,059	1	0,811313669	1,232568904	0,525423729
ZNF528	0,755689148	-0,30183696	0,062	0,047	1	0,811218827	1,232713008	1,319148936
PDGFRB	0,799342981	-0,301986262	0,094	0,074	1	0,81113488	1,232840585	1,27027027
UBE2K	0,062546936	-0,302261069	0,375	0,176	1	0,810980388	1,233075442	2,130681818
CCNH	0,664350968	-0,302268787	0,156	0,113	1	0,810976049	1,233082039	1,380530973

Supplementary Table 1

Differentially expressed genes_adjacent-skin-XL_ comparing SC-Keloid vs. (SC-Myel, SC-Nonmyel)								
**gene**	**p_val**	**avg_log2FC**	**pct.1**	**pct.2**	**p_val_adj**	**Foldchange_UP**	**Foldchange_DOWN**	**Ratio_pct1_pct2**
SOX12	0,134529645	-0,302429463	0	0,066	1	0,810885734	1,233219377	0
LINC00844	0,377976391	-0,302522531	0,031	0,07	1	0,810833426	1,233298934	0,442857143
SIRPA	0,257548576	-0,302805703	0	0,039	1	0,810674291	1,23354103	0
AC012368.1	0,147320294	-0,302915012	0	0,062	1	0,810612871	1,233634495	0
MTMR12	0,99780108	-0,302940342	0,062	0,059	1	0,810598639	1,233656155	1,050847458
LINC00327	0,603353082	-0,30296186	0,031	0,051	1	0,810586549	1,233674555	0,607843137
FOSL1	0,499122068	-0,303167445	0,156	0,102	1	0,810471048	1,233850367	1,529411765
SP3	0,084416338	-0,303234715	0,312	0,148	1	0,810433258	1,2339079	2,108108108
HSPA5	0,000295098	-0,303264739	0,875	0,496	1	0,810416393	1,233933579	1,764112903
ARFGAP2	0,040655239	-0,303366061	0,25	0,102	1	0,810359478	1,234020244	2,450980392
NF1	0,001574074	-0,30350795	0,438	0,148	1	0,810279783	1,234141615	2,959459459
PSME4	0,607026308	-0,30362658	0,062	0,086	1	0,810213158	1,2342431	0,720930233
THAP3	0,732255237	-0,303627877	0,094	0,07	1	0,81021243	1,234244209	1,342857143
MGP	0,528999683	-0,303860123	0,031	0,059	1	0,810082012	1,234442915	0,525423729
TMEM170A	0,501783108	-0,304506667	0,156	0,102	1	0,809719054	1,234996255	1,529411765
CLDND1	0,980897822	-0,304538853	0,531	0,398	1	0,80970099	1,235023808	1,334170854
PLEKHB1	0,77263384	-0,304989747	0,25	0,199	1	0,809447969	1,235409857	1,256281407
DNM1L	0,041853306	-0,305172128	0,25	0,102	1	0,809345648	1,235566044	2,450980392
ATP1B3	0,055021025	-0,305221565	0,469	0,223	1	0,809317914	1,235608384	2,103139013
PPP2R3B	1	-0,305296267	0,094	0,086	1	0,809276009	1,235672365	1,093023256
MID1IP1	0,025583591	-0,305501027	0,5	0,227	1	0,809161158	1,235847754	2,202643172
DYRK3	0,739785061	-0,305731461	0,062	0,074	1	0,809031925	1,236045166	0,837837838
NLK	0,234088337	-0,305746574	0	0,043	1	0,80902345	1,236058114	0
SPTAN1	0,103586669	-0,305783288	0,75	0,398	1	0,809002862	1,23608957	1,884422111
ZFX	0,606634787	-0,305897787	0,188	0,133	1	0,808938658	1,236187676	1,413533835
TMEM64	0,810386495	-0,306051129	0,125	0,102	1	0,808852682	1,236319076	1,225490196
DPP8	0,296411886	-0,30609491	0,188	0,105	1	0,808828136	1,236356594	1,79047619
AXIN1	0,933712959	-0,306309848	0,062	0,062	1	0,808707644	1,236540804	1
CCDC9	0,433914664	-0,306368552	0,062	0,105	1	0,808674738	1,236591121	0,59047619
WTIP	0,606583785	-0,306505758	0,094	0,117	1	0,808597833	1,236708732	0,803418803

Supplementary Table 1

Differentially expressed genes_adjacent-skin-XL_ comparing SC-Keloid vs. (SC-Myel, SC-Nonmyel)								
**gene**	**p_val**	**avg_log2FC**	**pct.1**	**pct.2**	**p_val_adj**	**Foldchange_UP**	**Foldchange_DOWN**	**Ratio_pct1_pct2**
AGO4	0,283789128	-0,306695436	0	0,035	1	0,80849153	1,236871338	0
KHNYN	0,739785061	-0,306900086	0,062	0,074	1	0,808376851	1,237046804	0,837837838
BARD1	0,099037596	-0,306994194	0,188	0,082	1	0,808324122	1,2371275	2,292682927
CCDC9B	0,110090391	-0,307078272	0,219	0,102	1	0,808277015	1,2371996	2,147058824
PAK2	0,140661932	-0,307394949	0,594	0,316	1	0,808099615	1,237471199	1,879746835
PTGS2	0,607571545	-0,307496758	0,031	0,051	1	0,80804259	1,23755853	0,607843137
KBTBD2	0,427470791	-0,307982747	0,188	0,117	1	0,807770438	1,237975486	1,606837607
KANSL3	0,499735776	-0,30807434	0,031	0,059	1	0,807719156	1,238054084	0,525423729
WDR59	0,414152817	-0,308101154	0,125	0,074	1	0,807704144	1,238077095	1,689189189
ZNF708	0,291015131	-0,308176854	0,031	0,082	1	0,807661764	1,23814206	0,37804878
LRRC75B	0,176914647	-0,308190555	0	0,055	1	0,807654094	1,238153819	0
IKBKB	0,803121702	-0,30825914	0,094	0,074	1	0,807615699	1,238212681	1,27027027
MARCHF1	0,325559719	-0,308291666	0,062	0,121	1	0,807597491	1,238240597	0,512396694
MED29	0,165597712	-0,308293441	0,219	0,109	1	0,807596498	1,238242121	2,009174312
PAPSS2	0,213026321	-0,308314917	0	0,047	1	0,807584476	1,238260554	0
ZBTB2	0,690331157	-0,308447767	0,062	0,078	1	0,807510113	1,238374583	0,794871795
NIN	0,732255237	-0,30888782	0,094	0,07	1	0,807263842	1,238752372	1,342857143
HEY1	0,079268503	-0,309148561	0,188	0,078	1	0,807117958	1,238976274	2,41025641
MOXD1	0,597765632	-0,309511723	0,125	0,086	1	0,806914812	1,239288194	1,453488372
BCAM	0,800356249	-0,309892708	0,062	0,07	1	0,806701751	1,239615507	0,885714286
TNXB	0,346686972	-0,310322464	0	0,027	1	0,806461483	1,239984824	0
DNAJC3-DT	0,993403306	-0,310383958	0,062	0,059	1	0,806427108	1,240037679	1,050847458
HSF2	0,908376542	-0,310442421	0,125	0,105	1	0,80639443	1,24008793	1,19047619
GLS	0,155491455	-0,310497684	0,438	0,234	1	0,806363542	1,240135433	1,871794872
PRRT1	0,864989089	-0,31054911	0,094	0,078	1	0,806334798	1,24017964	1,205128205
FHOD3	0,39210653	-0,310567299	0,156	0,094	1	0,806324633	1,240195275	1,659574468
NAPA	0,166915939	-0,310570692	0,406	0,223	1	0,806322736	1,240198193	1,820627803
CMTM6	0,403541193	-0,310774884	0,531	0,34	1	0,806208621	1,240373737	1,561764706
ATG16L2	0,81222265	-0,310826763	0,062	0,07	1	0,80617963	1,240418341	0,885714286
PVR	0,916437471	-0,310847982	0,062	0,055	1	0,806167774	1,240436585	1,127272727

Supplementary Table 1

Differentially expressed genes_adjacent-skin-XL_ comparing SC-Keloid vs. (SC-Myel, SC-Nonmyel)								
**gene**	**p_val**	**avg_log2FC**	**pct.1**	**pct.2**	**p_val_adj**	**Foldchange_UP**	**Foldchange_DOWN**	**Ratio_pct1_pct2**
ERAP1	0,304711877	-0,310886063	0,125	0,066	1	0,806146495	1,240469328	1,893939394
RIMKLB	0,380539324	-0,310965573	0,062	0,109	1	0,806102068	1,240537694	0,568807339
TM4SF1	0,131958685	-0,311071792	0,344	0,18	1	0,80604272	1,240629033	1,911111111
FRS2	0,088230697	-0,311163563	0,312	0,148	1	0,805991448	1,240707953	2,108108108
SH3PXD2B	0,405092563	-0,311180564	0,219	0,137	1	0,805981951	1,240722573	1,598540146
CCDC184	0,352472909	-0,311262209	0,031	0,074	1	0,805936339	1,240792791	0,418918919
FAM171A1	0,660402423	-0,311621886	0,094	0,066	1	0,805735437	1,24110217	1,424242424
STARD7	0,165597712	-0,311704122	0,219	0,109	1	0,80568951	1,241172917	2,009174312
CERT1	0,396866202	-0,311721794	0,219	0,137	1	0,805679642	1,241188121	1,598540146
ADAR	0,409657741	-0,311800426	0,281	0,184	1	0,80563573	1,241255772	1,527173913
CD99L2	0,22275641	-0,311870722	0,281	0,156	1	0,805596476	1,241316254	1,801282051
E4F1	0,603353082	-0,311955191	0,031	0,051	1	0,80554931	1,241388934	0,607843137
FBRSL1	0,799342981	-0,312116822	0,094	0,074	1	0,805459067	1,24152802	1,27027027
C2orf68	0,864972765	-0,312486566	0,062	0,066	1	0,805252664	1,241846249	0,939393939
GAPVD1	0,210314935	-0,313027747	0,219	0,117	1	0,804950656	1,242312174	1,871794872
ACKR1	0,134529645	-0,313174185	0	0,066	1	0,804868955	1,24243828	0
ZBTB8A	0,283789128	-0,313673972	0	0,035	1	0,804590176	1,242868767	0
RBM23	0,382714865	-0,313689998	0,219	0,137	1	0,804581239	1,242882573	1,598540146
ADCY9	0,548952158	-0,313756796	0,031	0,055	1	0,804543986	1,242940121	0,563636364
RFX3	0,664688786	-0,313867484	0,062	0,082	1	0,804482262	1,243035487	0,756097561
FANCB	0,603353082	-0,313972726	0,031	0,051	1	0,804423578	1,243126167	0,607843137
USP7	0,015076029	-0,314019571	0,406	0,164	1	0,804397459	1,243166533	2,475609756
TMEM41A	0,420870206	-0,31409743	0,031	0,066	1	0,804354048	1,243233626	0,46969697
CASP4	0,165379896	-0,314204922	0,219	0,113	1	0,80429412	1,24332626	1,938053097
SAMD8	0,420656361	-0,314301828	0,188	0,117	1	0,804240097	1,243409777	1,606837607
NCALD	0,213026321	-0,315458379	0	0,047	1	0,803595628	1,244406969	0
WIPF1	0,031021044	-0,315629607	0,406	0,184	1	0,803500258	1,244554672	2,206521739
MFSD14B	0,701547097	-0,315672969	0,125	0,094	1	0,803476109	1,244592078	1,329787234
AHDC1	0,682891358	-0,315763295	0,094	0,113	1	0,803425805	1,244670004	0,831858407
KIAA0930	0,561197551	-0,315903172	0,188	0,129	1	0,803347912	1,244790687	1,457364341

Supplementary Table 1

Differentially expressed genes_adjacent-skin-XL_ comparing SC-Keloid vs. (SC-Myel, SC-Nonmyel)								
**gene**	**p_val**	**avg_log2FC**	**pct.1**	**pct.2**	**p_val_adj**	**Foldchange_UP**	**Foldchange_DOWN**	**Ratio_pct1_pct2**
BLCAP	0,535198384	-0,316237291	0,062	0,09	1	0,803161884	1,245079006	0,688888889
LAMP5	0,194053346	-0,316241673	0	0,051	1	0,803159444	1,245082788	0
MAP1LC3B	0,079580072	-0,316486763	0,656	0,34	1	0,803023013	1,245294324	1,929411765
SMG7	0,688602647	-0,316921737	0,125	0,094	1	0,802780937	1,245669839	1,329787234
ABCD3	0,096829815	-0,317058486	0,375	0,188	1	0,802704847	1,245787918	1,994680851
MKRN1	0,331907101	-0,317308537	0,188	0,109	1	0,802565733	1,246003859	1,724770642
SCAF11	0,02508472	-0,317504263	0,594	0,273	1	0,802456858	1,246172912	2,175824176
VAV2	0,194053346	-0,317532104	0	0,051	1	0,802441373	1,246196961	0
CNKSR2	0,932622158	-0,317579323	0,094	0,082	1	0,80241511	1,246237749	1,146341463
PRMT2	0,124591345	-0,317759264	0,531	0,289	1	0,802315034	1,246393197	1,837370242
PHYKPL	0,830197375	-0,317896702	0,125	0,102	1	0,802238605	1,24651194	1,225490196
NXF1	0,899089282	-0,318223756	0,156	0,133	1	0,802056761	1,246794552	1,172932331
SH3RF3	0,535198384	-0,318529765	0,125	0,082	1	0,801886655	1,247059037	1,524390244
REPS1	0,721126199	-0,31856039	0,125	0,094	1	0,801869633	1,247085509	1,329787234
CITED4	0,283789128	-0,318903323	0	0,035	1	0,801679049	1,24738198	0
MSL2	0,377976391	-0,319022515	0,031	0,07	1	0,801612819	1,24748504	0,442857143
TRDMT1	0,800356249	-0,319030447	0,062	0,07	1	0,801608412	1,247491899	0,885714286
ARID1B	0,068226873	-0,319148875	0,469	0,23	1	0,801542612	1,247594308	2,039130435
MXD1	0,529145535	-0,319619714	0,125	0,082	1	0,801281063	1,248001539	1,524390244
ZNF302	0,573799329	-0,319798505	0,094	0,062	1	0,801181768	1,248156212	1,516129032
NUDT3	0,870756587	-0,320166622	0,188	0,16	1	0,800977365	1,248474731	1,175
ARHGEF17	0,60091357	-0,320255247	0,125	0,086	1	0,800928162	1,248551427	1,453488372
FXYD7	0,176914647	-0,320396276	0	0,055	1	0,800849872	1,248673484	0
PPP2R2D	0,594827904	-0,320407802	0,188	0,129	1	0,800843474	1,24868346	1,457364341
SLC66A3	0,49213523	-0,320426781	0,219	0,145	1	0,800832938	1,248699887	1,510344828
ETF1	0,223362355	-0,320661569	0,406	0,223	1	0,800702619	1,248903121	1,820627803
ZHX1	0,739069785	-0,320763724	0,156	0,121	1	0,800645925	1,248991556	1,289256198
CUEDC1	0,351104367	-0,320765127	0,25	0,152	1	0,800645146	1,248992771	1,644736842
TRIM33	0,049795909	-0,32100782	0,25	0,105	1	0,800510472	1,249202896	2,380952381
BICRAL	0,176914647	-0,321051518	0	0,055	1	0,800486225	1,249240735	0

Supplementary Table 1

Differentially expressed genes_adjacent-skin-XL_ comparing SC-Keloid vs. (SC-Myel, SC-Nonmyel)								
**gene**	**p_val**	**avg_log2FC**	**pct.1**	**pct.2**	**p_val_adj**	**Foldchange_UP**	**Foldchange_DOWN**	**Ratio_pct1_pct2**
SLFN11	0,341900128	-0,321069861	0,156	0,09	1	0,800476047	1,249256618	1,733333333
CNN1	0,348136214	-0,321277544	0,188	0,113	1	0,800360824	1,249436467	1,663716814
PTPN21	0,864989089	-0,321671643	0,094	0,078	1	0,80014222	1,249777821	1,205128205
SPSB3	0,759572792	-0,321736991	0,281	0,219	1	0,800105977	1,249834432	1,283105023
SQOR	0,377976391	-0,321906001	0,031	0,07	1	0,800012251	1,249980858	0,442857143
CDH7	0,194053346	-0,321968351	0	0,051	1	0,799977678	1,25003488	0
SYP	0,937980621	-0,322199738	0,062	0,062	1	0,799849383	1,250235383	1
SNHG7	0,600018946	-0,322823136	0,406	0,277	1	0,799503838	1,250775734	1,465703971
RAPGEF1	0,377976391	-0,322921665	0,031	0,07	1	0,799449238	1,250861159	0,442857143
DDAH2	0,115401198	-0,322923695	0,625	0,324	1	0,799448112	1,25086292	1,929012346
EDRF1	0,161397126	-0,323210061	0	0,059	1	0,799289443	1,251111233	0
TSPAN5	0,06801158	-0,323312756	0,469	0,234	1	0,799232549	1,251200293	2,004273504
TRIM9	0,499735776	-0,323328475	0,031	0,059	1	0,799223841	1,251213926	0,525423729
BEND7	0,804543977	-0,323451726	0,031	0,039	1	0,799155566	1,251320823	0,794871795
ASXL2	0,940093621	-0,323541532	0,094	0,082	1	0,799105821	1,251398718	1,146341463
C1GALT1	0,482162603	-0,323855828	0,188	0,121	1	0,798931752	1,25167137	1,553719008
SSH1	0,29527654	-0,323923732	0,188	0,109	1	0,798894149	1,251730284	1,724770642
APOL6	0,683139676	-0,32413139	0,062	0,078	1	0,798779166	1,251910468	0,794871795
APIP	0,138196896	-0,324205279	0,219	0,105	1	0,798738257	1,251974587	2,085714286
LYSMD2	0,588138226	-0,32422229	0,094	0,062	1	0,798728839	1,25198935	1,516129032
MCMBP	0,331907101	-0,324308978	0,188	0,109	1	0,798680847	1,252064581	1,724770642
STAG1	0,477497952	-0,32471006	0,281	0,188	1	0,798458838	1,252412714	1,494680851
RBM17	0,056992281	-0,324799199	0,406	0,195	1	0,798409505	1,252490099	2,082051282
FOXO4	0,414703427	-0,32516878	0,031	0,066	1	0,798204999	1,252810996	0,46969697
LETMD1	0,739785061	-0,325238922	0,062	0,074	1	0,798166193	1,252871907	0,837837838
POM121	0,739785061	-0,325257074	0,062	0,074	1	0,79815615	1,252887671	0,837837838
TAF8	0,516147046	-0,325384107	0,094	0,059	1	0,798085874	1,252997996	1,593220339
RC3H2	0,935028354	-0,325591464	0,094	0,09	1	0,797971174	1,253178101	1,044444444
YIPF4	0,270384651	-0,32561845	0,438	0,258	1	0,797956248	1,253201542	1,697674419
PCGF3	0,054396443	-0,325793385	0,219	0,09	1	0,797859497	1,253353509	2,433333333

Supplementary Table 1

Differentially expressed genes_adjacent-skin-XL_ comparing SC-Keloid vs. (SC-Myel, SC-Nonmyel)								
**gene**	**p_val**	**avg_log2FC**	**pct.1**	**pct.2**	**p_val_adj**	**Foldchange_UP**	**Foldchange_DOWN**	**Ratio_pct1_pct2**
EPS8	0,067162899	-0,325889825	0,344	0,164	1	0,797806164	1,253437295	2,097560976
CUL5	0,529145535	-0,325960051	0,125	0,082	1	0,79776733	1,25349831	1,524390244
USP42	0,25962289	-0,326387726	0,031	0,09	1	0,797530873	1,253869954	0,344444444
GABRE	0,147320294	-0,326701463	0	0,062	1	0,797357457	1,254142658	0
SUN1	0,64075889	-0,326811063	0,281	0,207	1	0,797296885	1,254237937	1,357487923
SF3B1	0,307065968	-0,327150444	0,656	0,406	1	0,79710935	1,25453302	1,615763547
GNPTAB	0,660402423	-0,327247746	0,094	0,066	1	0,797055591	1,254617634	1,424242424
TACSTD2	0,122891724	-0,327309943	0	0,07	1	0,797021229	1,254671724	0
PLEKHA3	0,070297141	-0,327389976	0,406	0,195	1	0,796977016	1,254741328	2,082051282
ZCCHC8	0,803121702	-0,327399464	0,094	0,074	1	0,796971774	1,254749581	1,27027027
CYCS	0,025025245	-0,32759758	0,719	0,34	1	0,796862339	1,254921899	2,114705882
LRIG2	0,804543977	-0,327674231	0,031	0,039	1	0,796820003	1,254988576	0,794871795
ELL2	0,270497466	-0,328062152	0,156	0,086	1	0,796605777	1,25532607	1,813953488
MGAT4C	0,283789128	-0,328296141	0	0,035	1	0,796476587	1,255529687	0
YTHDF3	0,054103199	-0,328440523	0,25	0,105	1	0,796396882	1,255655343	2,380952381
ZNF507	0,630272476	-0,3284866	0,062	0,082	1	0,796371446	1,255695448	0,756097561
MARK3	0,089247978	-0,328493932	0,406	0,203	1	0,796367399	1,25570183	2
PGAP1	0,99448227	-0,328533188	0,094	0,086	1	0,79634573	1,255735998	1,093023256
SSH2	0,429756478	-0,328869765	0,188	0,117	1	0,796159967	1,256028991	1,606837607
SBF2	0,943946826	-0,329056771	0,125	0,117	1	0,796056773	1,256191812	1,068376068
UBAP2	0,731875997	-0,329177407	0,125	0,141	1	0,795990211	1,256296857	0,886524823
TULP3	0,060233739	-0,329367441	0,406	0,191	1	0,795885368	1,25646235	2,12565445
EXOC3	0,135027649	-0,329497213	0,281	0,141	1	0,795813781	1,256575375	1,992907801
COL17A1	0,313292814	-0,32953394	0	0,031	1	0,795793522	1,256607364	0
AKR1C2	0,147320294	-0,329586671	0	0,062	1	0,795764436	1,256653294	0
ARID5B	0,037162106	-0,329633471	0,594	0,285	1	0,795738622	1,25669406	2,084210526
MARCKS	0,444380808	-0,329665638	1	0,879	1	0,79572088	1,25672208	1,137656428
PBX2	0,048449315	-0,329954653	0,281	0,121	1	0,79556149	1,256973865	2,32231405
CEP192	0,257548576	-0,330533997	0	0,039	1	0,795242079	1,25747873	0
PKIG	0,000606525	-0,330824021	0,688	0,25	1	0,795082229	1,257731545	2,752

Supplementary Table 1

Differentially expressed genes_adjacent-skin-XL_ comparing SC-Keloid vs. (SC-Myel, SC-Nonmyel)								
**gene**	**p_val**	**avg_log2FC**	**pct.1**	**pct.2**	**p_val_adj**	**Foldchange_UP**	**Foldchange_DOWN**	**Ratio_pct1_pct2**
GALNT18	0,234088337	-0,330866184	0	0,043	1	0,795058993	1,257768303	0
AMIGO2	0,213026321	-0,330882513	0	0,047	1	0,795049994	1,257782539	0
FAM76B	0,563799113	-0,331056741	0,094	0,125	1	0,794953985	1,257934445	0,752
AP2A2	0,255577342	-0,331282077	0,219	0,121	1	0,79482983	1,258130938	1,809917355
SERINC3	0,427421679	-0,331396468	0,406	0,266	1	0,794766811	1,258230699	1,526315789
PALLD	0,131096381	-0,331407513	0,594	0,32	1	0,794760726	1,258240332	1,85625
WNK2	0,234088337	-0,331565353	0	0,043	1	0,794673779	1,258377999	0
SPTLC1	0,753434371	-0,331731155	0,188	0,145	1	0,794582456	1,258522627	1,296551724
CDS2	0,683139676	-0,331895938	0,062	0,078	1	0,794491705	1,258666382	0,794871795
HLA-F	0,134099711	-0,331963606	0,281	0,141	1	0,794454441	1,25872542	1,992907801
GRINA	0,225377528	-0,332025233	0,281	0,156	1	0,794420505	1,258779189	1,801282051
NR6A1	0,093802134	-0,332154424	0	0,082	1	0,79434937	1,258891916	0
KLF7	0,229370488	-0,332383057	0,219	0,121	1	0,794223494	1,259091436	1,809917355
MITF	0,428872689	-0,332412745	0	0,02	1	0,79420715	1,259117347	0
ZNF83	0,036984609	-0,332496365	0,344	0,148	1	0,794161119	1,259190328	2,324324324
WDR45B	0,182101208	-0,332792336	0,406	0,223	1	0,793998212	1,25944868	1,820627803
CD83	0,06440165	-0,332844822	0,156	0,059	1	0,793969326	1,2594945	2,644067797
DIP2C	0,389095675	-0,333029236	0,094	0,145	1	0,793867843	1,259655506	0,648275862
MDGA1	0,176914647	-0,333119946	0	0,055	1	0,79381793	1,25973471	0
PRKD3	0,358820944	-0,333200825	0,312	0,188	1	0,793773429	1,259805334	1,659574468
STAG2	0,110573964	-0,333523115	0,531	0,277	1	0,793596124	1,260086799	1,916967509
RBM5	0,268932309	-0,333678043	0,344	0,199	1	0,793510906	1,260222125	1,728643216
PPP3R1	0,093052434	-0,334076831	0,281	0,133	1	0,793291595	1,260570522	2,112781955
TMEM9	0,482260664	-0,334138953	0,219	0,141	1	0,793257437	1,260624803	1,553191489
CXCL14	0,942250066	-0,3345764	0,062	0,062	1	0,793016946	1,261007101	1
KDM3A	0,960267559	-0,334899116	0,094	0,09	1	0,792839576	1,261289207	1,044444444
KDM6A	0,683139676	-0,334984451	0,062	0,078	1	0,792792681	1,261363814	0,794871795
XIAP	0,480873217	-0,335036512	0,219	0,145	1	0,792764073	1,261409333	1,510344828
TFEB	0,872528346	-0,335041851	0,094	0,078	1	0,792761139	1,261414	1,205128205
PLA2G4C	0,176914647	-0,335147634	0	0,055	1	0,792703013	1,261506495	0

Supplementary Table 1

Differentially expressed genes_adjacent-skin-XL_ comparing SC-Keloid vs. (SC-Myel, SC-Nonmyel)								
**gene**	**p_val**	**avg_log2FC**	**pct.1**	**pct.2**	**p_val_adj**	**Foldchange_UP**	**Foldchange_DOWN**	**Ratio_pct1_pct2**
VPS13C	0,146718253	-0,335187009	0,562	0,309	1	0,792681379	1,261540925	1,818770227
PLEKHG2	0,414703427	-0,335883446	0,031	0,066	1	0,792298818	1,26215006	0,46969697
ANKIB1	0,002108779	-0,335886548	0,281	0,082	1	0,792297114	1,262152773	3,426829268
ECPAS	0,37099797	-0,335943214	0,156	0,094	1	0,792265995	1,262202349	1,659574468
DDX6	0,0321176	-0,336367955	0,5	0,23	1	0,792032779	1,262574007	2,173913043
PHF20L1	0,046151963	-0,336584035	0,375	0,168	1	0,791914161	1,262763124	2,232142857
PANK3	0,028002326	-0,33669142	0,281	0,113	1	0,791855219	1,262857119	2,486725664
RGS12	0,517145205	-0,336986559	0,125	0,082	1	0,791693242	1,263115494	1,524390244
ITM2C	0,030109085	-0,337089418	0,75	0,355	1	0,791636799	1,263205553	2,112676056
MAPK1IP1L	0,273549367	-0,337260844	0,281	0,16	1	0,791542739	1,26335566	1,75625
CAV1	0,067594417	-0,337545783	0,531	0,27	1	0,791386421	1,263605204	1,966666667
VTA1	0,800356249	-0,337548293	0,062	0,07	1	0,791385044	1,263607402	0,885714286
KCMF1	0,178842486	-0,337618854	0,375	0,203	1	0,79134634	1,263669205	1,84729064
ILF3	0,273697301	-0,337643559	0,438	0,262	1	0,791332788	1,263690845	1,671755725
ZNF207	0,016864748	-0,338062861	0,594	0,262	1	0,79110283	1,264058175	2,267175573
HACD2	0,538657453	-0,338080089	0,156	0,105	1	0,791093384	1,264073269	1,485714286
ZNF655	0,163189943	-0,338443026	0,219	0,109	1	0,790894394	1,264391311	2,009174312
HLA-DPA1	0,924392442	-0,338443242	0,031	0,027	1	0,790894276	1,2643915	1,148148148
CNNM3	0,161397126	-0,33855003	0	0,059	1	0,790835736	1,264485094	0
SAP18	0,944368866	-0,33867764	0,719	0,523	1	0,790765788	1,264596945	1,374760994
YPEL5	0,086900572	-0,338686543	0,5	0,258	1	0,790760908	1,26460475	1,937984496
OLFML2B	0,416896114	-0,338790556	0,125	0,074	1	0,790703899	1,264695927	1,689189189
LGR4	0,960267559	-0,338813364	0,094	0,09	1	0,790691399	1,26471592	1,044444444
TCP1	0,333101677	-0,339198482	0,469	0,297	1	0,790480357	1,265053573	1,579124579
SHF	0,607571545	-0,339290314	0,031	0,051	1	0,790430042	1,2651341	0,607843137
RABGAP1	0,620881389	-0,3393152	0,156	0,109	1	0,790416407	1,265155924	1,431192661
SLC25A33	0,76088146	-0,339576569	0,125	0,133	1	0,790273223	1,265385149	0,939849624
TPRG1L	0,660402423	-0,339651954	0,094	0,066	1	0,79023193	1,265451271	1,424242424
LRRK2	0,316283338	-0,339728343	0,031	0,082	1	0,790190089	1,265518277	0,37804878
ZMYND11	0,320338862	-0,339857426	0,25	0,148	1	0,790119391	1,265631512	1,689189189

Supplementary Table 1

Differentially expressed genes_adjacent-skin-XL_ comparing SC-Keloid vs. (SC-Myel, SC-Nonmyel)								
**gene**	**p_val**	**avg_log2FC**	**pct.1**	**pct.2**	**p_val_adj**	**Foldchange_UP**	**Foldchange_DOWN**	**Ratio_pct1_pct2**
UIMC1	1	-0,340179261	0,094	0,086	1	0,789943152	1,26591388	1,093023256
MAPKBP1	0,920939129	-0,340219827	0,062	0,055	1	0,78992094	1,265949475	1,127272727
PALS1	0,16495238	-0,340633975	0,031	0,113	1	0,789694214	1,266312938	0,274336283
SIX5	0,730358576	-0,340803655	0,031	0,043	1	0,789601341	1,266461881	0,720930233
USP16	0,177046306	-0,341045202	0,344	0,184	1	0,789469151	1,26667394	1,869565217
EP300	0,657456412	-0,341153397	0,219	0,168	1	0,789409947	1,266768938	1,303571429
ALG13	0,97109805	-0,341238252	0,094	0,09	1	0,789363518	1,266843447	1,044444444
CCDC149	0,10262374	-0,341253052	0	0,078	1	0,78935542	1,266856444	0
GTF2IRD2B	0,213026321	-0,341317179	0	0,047	1	0,789320334	1,266912756	0
SEMA4A	0,492632793	-0,341453589	0,062	0,094	1	0,789245706	1,267032551	0,659574468
DENND2A	0,344592621	-0,341493728	0,031	0,074	1	0,789223748	1,267067803	0,418918919
AP1G1	0,860276283	-0,341723729	0,156	0,133	1	0,789097936	1,267269821	1,172932331
THBS1	0,056235689	-0,341786107	0,5	0,25	1	0,789063818	1,267324616	2
PIEZO2	0,10262374	-0,34188031	0	0,078	1	0,789012297	1,26740737	0
PEG10	0,001612855	-0,342053761	0,531	0,203	1	0,788917442	1,267559755	2,615763547
WHAMM	0,940093621	-0,342195974	0,094	0,082	1	0,788839679	1,267684711	1,146341463
REXO1	0,032569978	-0,342229179	0,219	0,082	1	0,788821523	1,267713889	2,670731707
ACADSB	0,499735776	-0,342334271	0,031	0,059	1	0,788764064	1,267806238	0,525423729
INTU	0,800356249	-0,342373975	0,062	0,07	1	0,788742357	1,267841129	0,885714286
PPFIA1	0,434592747	-0,342533767	0,156	0,098	1	0,788655001	1,267981562	1,591836735
ARHGEF10L	0,100873626	-0,342735311	0,188	0,082	1	0,788544834	1,268158711	2,292682927
ZNF131	0,976274084	-0,342889223	0,188	0,16	1	0,788460713	1,26829401	1,175
GNL3	0,004661673	-0,342918378	0,5	0,199	1	0,78844478	1,268319641	2,512562814
ZNF677	0,377976391	-0,342974586	0,031	0,07	1	0,788414062	1,268369056	0,442857143
TMEM165	0,022157242	-0,343098941	0,531	0,234	1	0,788346107	1,26847839	2,269230769
MPRIP	0,069948044	-0,343272852	0,5	0,25	1	0,788251081	1,268631308	2
WBP11	0,133974042	-0,34356338	0,469	0,242	1	0,78809236	1,268886809	1,938016529
MAPK10	0,28878729	-0,343685246	0,031	0,082	1	0,788025792	1,268993998	0,37804878
ZNF397	0,682891358	-0,34385333	0,094	0,113	1	0,787933987	1,269141853	0,831858407
SPPL3	0,138730892	-0,34398926	0,344	0,18	1	0,787859751	1,269261438	1,911111111

Supplementary Table 1

Differentially expressed genes_adjacent-skin-XL_ comparing SC-Keloid vs. (SC-Myel, SC-Nonmyel)								
**gene**	**p_val**	**avg_log2FC**	**pct.1**	**pct.2**	**p_val_adj**	**Foldchange_UP**	**Foldchange_DOWN**	**Ratio_pct1_pct2**
ZNF704	0,396895993	-0,34401813	0,062	0,109	1	0,787843985	1,269286837	0,568807339
TSC1	0,711312846	-0,344248605	0,125	0,094	1	0,787718135	1,269489625	1,329787234
SNX24	0,10262374	-0,344518341	0	0,078	1	0,787570872	1,269727	0
NBPF12	0,383663233	-0,344656598	0,031	0,07	1	0,7874954	1,269848687	0,442857143
NKX6-2	0,257548576	-0,344716273	0	0,039	1	0,787462827	1,269901214	0
TUSC1	0,090849937	-0,34478198	0,312	0,148	1	0,787426963	1,269959052	2,108108108
TCEA2	0,845147782	-0,344885163	0,281	0,219	1	0,787370648	1,270049884	1,283105023
EZH2	0,630272476	-0,344931086	0,062	0,082	1	0,787345585	1,270090312	0,756097561
ZCCHC14	0,1480486	-0,34495006	0,188	0,09	1	0,78733523	1,270107016	2,088888889
MAPKAPK2	0,38681383	-0,345837969	0,25	0,156	1	0,786850813	1,270888946	1,602564103
TIA1	0,26655819	-0,346051781	0,219	0,125	1	0,786734208	1,271077309	1,752
NOTCH1	0,0620791	-0,346245359	0,312	0,141	1	0,786628652	1,271247872	2,212765957
IRF9	0,309367063	-0,346414333	0,344	0,211	1	0,786536525	1,271396774	1,630331754
ETS2	0,212362312	-0,346710527	0,156	0,078	1	0,78637506	1,271657826	2
FEM1B	0,864989089	-0,346745058	0,094	0,078	1	0,786356239	1,271688264	1,205128205
KLF15	0,134529645	-0,347184497	0	0,066	1	0,786116755	1,272075674	0
PANX1	0,630272281	-0,347311388	0,062	0,082	1	0,786047615	1,272187563	0,756097561
CYB5A	0,216844992	-0,347339189	0,312	0,172	1	0,786032468	1,272212079	1,813953488
MTMR10	0,314221703	-0,347421523	0,031	0,078	1	0,785987611	1,272284685	0,397435897
TSC2	0,621083681	-0,347805948	0,25	0,18	1	0,785778202	1,272623748	1,388888889
OTUD1	0,704796968	-0,347914755	0,125	0,094	1	0,785718942	1,272719731	1,329787234
RNF130	0,300504859	-0,347996279	0,5	0,309	1	0,785674543	1,272791653	1,618122977
INTS3	0,165597712	-0,348183729	0,219	0,109	1	0,785572467	1,272957038	2,009174312
ZNF141	0,864989089	-0,34824401	0,094	0,078	1	0,785539643	1,273010228	1,205128205
ZNF532	0,264110461	-0,348737978	0,062	0,129	1	0,785270726	1,273446172	0,480620155
RASGRF1	0,283789128	-0,348878848	0	0,035	1	0,785194053	1,273570521	0
TENT4B	0,217772806	-0,349154219	0,188	0,098	1	0,785044196	1,273813634	1,918367347
FBLN5	0,10262374	-0,349372604	0	0,078	1	0,78492537	1,27400647	0
RBM14	0,790895329	-0,349493042	0,125	0,098	1	0,784859846	1,27411283	1,275510204
RUFY2	0,790895329	-0,349727368	0,125	0,098	1	0,784732378	1,274319791	1,275510204

Supplementary Table 1

Differentially expressed genes_adjacent-skin-XL_ comparing SC-Keloid vs. (SC-Myel, SC-Nonmyel)								
**gene**	**p_val**	**avg_log2FC**	**pct.1**	**pct.2**	**p_val_adj**	**Foldchange_UP**	**Foldchange_DOWN**	**Ratio_pct1_pct2**
SPIN1	0,526388413	-0,350650381	0,188	0,125	1	0,784230479	1,275135341	1,504
PKD1	0,291985575	-0,350717475	0,25	0,148	1	0,784194009	1,275194644	1,689189189
C1orf52	0,139693288	-0,350974183	0,312	0,16	1	0,784054485	1,275421568	1,95
KIF1B	0,276655885	-0,351291408	0,344	0,203	1	0,783882103	1,275702043	1,694581281
PCBP1	0,188666329	-0,351373128	0,781	0,414	1	0,783837702	1,275774306	1,88647343
RIPK4	0,603353082	-0,351376336	0,031	0,051	1	0,783835959	1,275777143	0,607843137
ZNF622	0,780949248	-0,351395402	0,094	0,102	1	0,7838256	1,275794002	0,921568627
IFT88	0,853446312	-0,35163611	0,125	0,102	1	0,783694833	1,276006882	1,225490196
MGLL	0,344592621	-0,351999655	0,031	0,074	1	0,783497374	1,276328464	0,418918919
SNAI1	0,392106312	-0,352599694	0,156	0,094	1	0,783171573	1,276859419	1,659574468
NAA25	0,587470736	-0,35273478	0,062	0,086	1	0,783098245	1,276978983	0,720930233
RBP1	0,194053346	-0,352812299	0	0,051	1	0,783056169	1,277047599	0
NT5DC2	0,050014543	-0,353579	0,438	0,207	1	0,782640135	1,277726449	2,115942029
SNRNP70	0,148541101	-0,353646516	0,719	0,387	1	0,782603509	1,277786247	1,857881137
CCNT1	0,932622158	-0,353803198	0,094	0,082	1	0,78251852	1,277925026	1,146341463
REV1	0,362589924	-0,353970207	0,188	0,113	1	0,782427939	1,27807297	1,663716814
TXLNG	0,432049072	-0,354004535	0,188	0,117	1	0,782409322	1,278103381	1,606837607
KIF19	0,176914647	-0,354010842	0	0,055	1	0,782405902	1,278108968	0
ZC3HAV1	0,180187128	-0,354048619	0,219	0,113	1	0,782385415	1,278142436	1,938053097
KDM5C	0,507127635	-0,354099972	0,156	0,102	1	0,782357566	1,278187933	1,529411765
CRKL	0,935028354	-0,354106346	0,094	0,09	1	0,782354109	1,27819358	1,044444444
PLK3	0,790895329	-0,354164791	0,094	0,102	1	0,782322416	1,278245362	0,921568627
NPIPB11	0,455148041	-0,354331288	0,031	0,062	1	0,782232136	1,278392888	0,5
ANKRD50	0,798617831	-0,354392778	0,219	0,176	1	0,782198797	1,278447377	1,244318182
ATP6V1E2	0,548952158	-0,354398445	0,031	0,055	1	0,782195724	1,278452399	0,563636364
TRHDE	0,176914647	-0,354561136	0	0,055	1	0,782107522	1,278596576	0
KHDRBS3	0,154902655	-0,354652241	0,75	0,391	1	0,782058134	1,278677322	1,918158568
FMR1	0,573788361	-0,354694715	0,094	0,121	1	0,78203511	1,278714967	0,776859504
APBB2	0,310355006	-0,354899229	0,094	0,047	1	0,781924258	1,278896249	2
VEZT	0,209609348	-0,35498805	0,25	0,137	1	0,78187612	1,278974987	1,824817518

Supplementary Table 1

Differentially expressed genes_adjacent-skin-XL_ comparing SC-Keloid vs. (SC-Myel, SC-Nonmyel)								
**gene**	**p_val**	**avg_log2FC**	**pct.1**	**pct.2**	**p_val_adj**	**Foldchange_UP**	**Foldchange_DOWN**	**Ratio_pct1_pct2**
PCNX1	0,874415164	-0,355485726	0,094	0,094	1	0,781606448	1,279416262	1
RNF38	0,736944569	-0,355752906	0,188	0,145	1	0,781461712	1,279653226	1,296551724
CRTC2	0,764452551	-0,355851249	0,125	0,098	1	0,781408444	1,279740458	1,275510204
SLC39A6	0,296464763	-0,356048425	0,281	0,168	1	0,781301655	1,279915374	1,672619048
POU3F1	0,000310707	-0,356145474	0,562	0,195	1	0,781249099	1,280001476	2,882051282
MAT2A	0,95305118	-0,356472828	0,094	0,09	1	0,78107185	1,280291947	1,044444444
RASSF1	0,250657258	-0,356775754	0,312	0,18	1	0,780907864	1,280560801	1,733333333
LINC00685	0,194053346	-0,356806824	0	0,051	1	0,780891047	1,28058838	0
RBPJ	0,037267708	-0,357053545	0,625	0,293	1	0,780757515	1,280807397	2,133105802
EIF1AX	0,663103381	-0,357187524	0,719	0,473	1	0,780685011	1,280926348	1,520084567
KRT17	0,177248199	-0,357211934	0,281	0,152	1	0,780671803	1,28094802	1,848684211
ANGEL2	0,359364064	-0,357805675	0,125	0,07	1	0,780350583	1,281475303	1,785714286
IRF7	0,127829684	-0,35781879	0,219	0,105	1	0,780343489	1,281486952	2,085714286
ZNF703	0,99780108	-0,357851196	0,062	0,059	1	0,780325961	1,281515738	1,050847458
TUBG2	0,908376542	-0,358108498	0,125	0,105	1	0,780186804	1,281744314	1,19047619
AAK1	0,070620647	-0,358155075	0,375	0,18	1	0,780161616	1,281785695	2,083333333
ULK1	0,288304724	-0,358317555	0,156	0,086	1	0,780073758	1,281930061	1,813953488
SIRT1	0,810386495	-0,358903275	0,125	0,102	1	0,779757119	1,282450619	1,225490196
ATXN1L	0,314221703	-0,35912495	0,031	0,078	1	0,779637317	1,282647686	0,397435897
SAFB2	0,171841867	-0,359156659	0,281	0,148	1	0,779620181	1,282675878	1,898648649
SEC63	0,235403752	-0,359233241	0,312	0,176	1	0,779578798	1,282743967	1,772727273
CREM	0,288320579	-0,359371025	0,219	0,125	1	0,779504348	1,282866482	1,752
RICTOR	0,824651962	-0,359671592	0,219	0,176	1	0,779341965	1,283133778	1,244318182
NAB2	0,250004291	-0,359753338	0,156	0,082	1	0,779297807	1,283206485	1,902439024
NECTIN2	0,195175128	-0,359813682	0,406	0,223	1	0,779265212	1,28326016	1,820627803
CTNNA1	0,801026191	-0,36011053	0,938	0,613	1	0,779104887	1,283524229	1,530179445
GARRE1	0,50638112	-0,360201528	0,094	0,125	1	0,779055747	1,28360519	0,752
DDX24	0,085816921	-0,361429578	0,75	0,363	1	0,778392882	1,284698284	2,066115702
ZFYVE28	0,455148041	-0,361518575	0,031	0,062	1	0,778344865	1,284777538	0,5
SOS2	0,11621502	-0,361526786	0,25	0,125	1	0,778340436	1,28478485	2

Supplementary Table 1

Differentially expressed genes_adjacent-skin-XL_ comparing SC-Keloid vs. (SC-Myel, SC-Nonmyel)								
**gene**	**p_val**	**avg_log2FC**	**pct.1**	**pct.2**	**p_val_adj**	**Foldchange_UP**	**Foldchange_DOWN**	**Ratio_pct1_pct2**
AK3	0,422250029	-0,361696205	0,281	0,18	1	0,778249039	1,284935734	1,561111111
PPP3CA	0,095565969	-0,361928682	0,5	0,258	1	0,778123641	1,285142807	1,937984496
BRWD1	0,144843861	-0,362696052	0,281	0,145	1	0,777709867	1,285826556	1,937931034
PPM1D	0,538237843	-0,362726666	0,062	0,09	1	0,777693364	1,285853841	0,688888889
AFF4	0,036152742	-0,362752213	0,531	0,242	1	0,777679593	1,285876611	2,194214876
KIAA1109	0,739069785	-0,362954393	0,156	0,121	1	0,777570616	1,286056827	1,289256198
GPR173	0,147320294	-0,36296622	0	0,062	1	0,777564242	1,28606737	0
POM121C	0,738281619	-0,363268898	0,125	0,098	1	0,777401126	1,286337216	1,275510204
DOK5	0,414703427	-0,363491861	0,031	0,066	1	0,777280991	1,28653603	0,46969697
DISP1	0,499735776	-0,363527551	0,031	0,059	1	0,777261762	1,286567857	0,525423729
ADGRA2	0,1480486	-0,36365711	0,188	0,09	1	0,777191964	1,286683401	2,088888889
GRAMD4	0,499735776	-0,363704631	0,031	0,059	1	0,777166365	1,286725784	0,525423729
G0S2	0,10262374	-0,363921188	0	0,078	1	0,777049716	1,286918943	0
RBM38	0,512685318	-0,364154878	0,094	0,059	1	0,776923859	1,287127417	1,593220339
CEP95	0,637094964	-0,364206034	0,062	0,082	1	0,776896311	1,287173058	0,756097561
OSBPL5	0,377976391	-0,36420965	0,031	0,07	1	0,776894363	1,287176284	0,442857143
PHLDB2	0,120847574	-0,364463725	0,344	0,176	1	0,776757556	1,28740299	1,954545455
TNIP2	0,194605586	-0,364527125	0,219	0,113	1	0,776723422	1,287459567	1,938053097
HELZ2	0,833510073	-0,364972823	0,125	0,102	1	0,776483502	1,287857369	1,225490196
RNF31	0,99780108	-0,365457366	0,062	0,059	1	0,776222756	1,288289981	1,050847458
EFR3A	0,783849734	-0,365514939	0,156	0,121	1	0,776191781	1,288341393	1,289256198
CDKAL1	0,176914647	-0,365876901	0	0,055	1	0,775997064	1,28866467	0
USP9X	0,449929502	-0,365953967	0,25	0,164	1	0,775955613	1,288733509	1,524390244
MMP19	0,191913299	-0,365975083	0,094	0,039	1	0,775944256	1,288752372	2,41025641
ITSN2	0,589624457	-0,366096596	0,094	0,117	1	0,775878903	1,288860924	0,803418803
H2AC6	0,377976391	-0,366416699	0,031	0,07	1	0,775706772	1,289146926	0,442857143
IL17B	0,147320294	-0,366418648	0	0,062	1	0,775705724	1,289148668	0
DNAJB9	0,27817043	-0,36678656	0,281	0,164	1	0,775507931	1,289477464	1,713414634
THY1	0,014401184	-0,366885719	0,312	0,117	1	0,775454631	1,289566095	2,666666667
HERPUD2	0,347206802	-0,367191169	0,031	0,074	1	0,775290467	1,289839153	0,418918919

Supplementary Table 1

Differentially expressed genes_adjacent-skin-XL_ comparing SC-Keloid vs. (SC-Myel, SC-Nonmyel)								
**gene**	**p_val**	**avg_log2FC**	**pct.1**	**pct.2**	**p_val_adj**	**Foldchange_UP**	**Foldchange_DOWN**	**Ratio_pct1_pct2**
KDM7A	0,112290358	-0,367660838	0	0,074	1	0,775038113	1,290259129	0
STX3	0,935028321	-0,367726816	0,094	0,09	1	0,775002669	1,290318137	1,044444444
EPAS1	0,840330301	-0,367732127	0,094	0,098	1	0,774999816	1,290322887	0,959183673
ZXDC	0,147320294	-0,367758472	0	0,062	1	0,774985664	1,29034645	0
PDZD2	0,134529645	-0,368089398	0	0,066	1	0,774807918	1,290642464	0
ARID2	0,528890372	-0,368184877	0,188	0,125	1	0,774756642	1,290727883	1,504
ZNF148	0,274790708	-0,368310879	0,219	0,125	1	0,774688979	1,290840617	1,752
MTHFR	0,633679827	-0,368564689	0,062	0,082	1	0,774552702	1,291067731	0,756097561
NCOA2	0,587470736	-0,368846503	0,062	0,086	1	0,774401417	1,291319951	0,720930233
SNX30	0,739785061	-0,368849372	0,062	0,074	1	0,774399877	1,29132252	0,837837838
ARAP1	0,99780108	-0,368963064	0,062	0,059	1	0,774338852	1,291424287	1,050847458
PPP2R3A	0,756564831	-0,369167995	0,094	0,105	1	0,774228868	1,291607743	0,895238095
ZRANB1	0,853446377	-0,36930997	0,125	0,102	1	0,774152679	1,291734856	1,225490196
ZC3H7B	0,185335556	-0,369506052	0,219	0,113	1	0,774047469	1,291910432	1,938053097
IFIT3	0,847430516	-0,369670094	0,188	0,156	1	0,77395946	1,292057338	1,205128205
ADIPOR2	0,908376542	-0,369707553	0,125	0,105	1	0,773939365	1,292090885	1,19047619
EPB41L4B	0,112290358	-0,369733414	0	0,074	1	0,773925492	1,292114047	0
DHX36	0,244023195	-0,370953155	0,531	0,312	1	0,773271445	1,29320694	1,701923077
POLR2A	0,639262178	-0,371051513	0,188	0,137	1	0,773218728	1,29329511	1,372262774
POLR2J3	0,165239067	-0,371141238	0,25	0,129	1	0,773170641	1,293375546	1,937984496
RASEF	0,194053346	-0,371170142	0	0,051	1	0,773155151	1,293401458	0
RYR1	0,122891457	-0,371247744	0	0,07	1	0,773113565	1,293471031	0
USP39	0,872528346	-0,371512161	0,094	0,078	1	0,772971881	1,293708121	1,205128205
OBSL1	0,633679827	-0,371519107	0,062	0,082	1	0,77296816	1,29371435	0,756097561
MAPK8IP1	0,906837565	-0,372180508	0,125	0,121	1	0,772613875	1,294307587	1,033057851
ARL8B	0,784946494	-0,372359412	0,219	0,168	1	0,772518072	1,294468099	1,303571429
REL	0,344060173	-0,372455095	0,188	0,109	1	0,772466838	1,294553954	1,724770642
PURB	0,158735078	-0,372939197	0,344	0,18	1	0,772207677	1,29498842	1,911111111
SP4	0,183854736	-0,373094562	0,031	0,102	1	0,772124522	1,295127886	0,303921569
PRRC2A	0,230685397	-0,37310719	0,281	0,156	1	0,772117764	1,295139222	1,801282051

Supplementary Table 1

Differentially expressed genes_adjacent-skin-XL_ comparing SC-Keloid vs. (SC-Myel, SC-Nonmyel)								
**gene**	**p_val**	**avg_log2FC**	**pct.1**	**pct.2**	**p_val_adj**	**Foldchange_UP**	**Foldchange_DOWN**	**Ratio_pct1_pct2**
LIN7A	0,933712959	-0,373461385	0,062	0,062	1	0,771928225	1,29545723	1
XAB2	0,633679827	-0,373511527	0,062	0,082	1	0,771901397	1,295502255	0,756097561
SERTAD3	0,540099312	-0,373848467	0,25	0,172	1	0,771721141	1,295804854	1,453488372
MAP1A	0,581227435	-0,373966631	0,094	0,117	1	0,771657935	1,295910991	0,803418803
TENT4A	0,122891724	-0,374065272	0	0,07	1	0,771605177	1,295999599	0
TRMT13	0,840130893	-0,374185327	0,062	0,051	1	0,77154097	1,296107452	1,215686275
UBL3	0,149799379	-0,37440934	0,375	0,203	1	0,771421179	1,296308719	1,84729064
REC8	0,581017609	-0,374476953	0,062	0,086	1	0,771385027	1,296369472	0,720930233
BAG4	0,997012247	-0,374926695	0,156	0,137	1	0,771144595	1,296773662	1,138686131
PRRG1	0,416531335	-0,375038102	0,062	0,102	1	0,771085048	1,296873805	0,607843137
CDC42EP3	0,00823539	-0,375069413	0,5	0,207	1	0,771068313	1,296901952	2,415458937
KANK4	0,134529645	-0,375392477	0	0,066	1	0,770895666	1,297192401	0
SELE	0,314221703	-0,375604919	0,031	0,078	1	0,770782157	1,297383431	0,397435897
PCM1	0,211486392	-0,375712882	0,656	0,375	1	0,770724479	1,297480523	1,749333333
GLA	0,956227111	-0,375934279	0,156	0,133	1	0,770606212	1,297679651	1,172932331
ARMH4	0,176914647	-0,376040936	0	0,055	1	0,770549244	1,29777559	0
TTC39C	0,795919922	-0,376070881	0,156	0,121	1	0,77053325	1,297802528	1,289256198
KAT8	0,783849734	-0,376736072	0,156	0,121	1	0,770178058	1,29840105	1,289256198
G3BP1	0,195794938	-0,376748989	0,438	0,242	1	0,770171163	1,298412675	1,809917355
VPS13A	0,255603817	-0,377006568	0,188	0,102	1	0,770033668	1,298644515	1,843137255
CDKN2D	0,127028438	-0,377081817	0,25	0,125	1	0,769993506	1,298712253	2
HECA	0,767743616	-0,37726334	0,094	0,102	1	0,769896629	1,29887567	0,921568627
AQP3	0,99780108	-0,377291161	0,062	0,059	1	0,769881783	1,298900717	1,050847458
FRMD6	0,089637552	-0,377296988	0,531	0,273	1	0,769878674	1,298905963	1,945054945
EVA1C	0,271686462	-0,377384598	0,031	0,086	1	0,769831923	1,298984844	0,360465116
SPG7	0,051511579	-0,377429748	0,281	0,121	1	0,769807831	1,299025497	2,32231405
SETD3	0,983740563	-0,377625284	0,156	0,141	1	0,769703502	1,299201573	1,106382979
SWAP70	0,147320294	-0,37793041	0	0,062	1	0,769540729	1,299476379	0
EP400	0,836907453	-0,378210491	0,094	0,098	1	0,769391347	1,299728681	0,959183673
RAB3GAP1	0,453327517	-0,378305129	0,219	0,141	1	0,769340878	1,299813943	1,553191489

Supplementary Table 1

Differentially expressed genes_adjacent-skin-XL_ comparing SC-Keloid vs. (SC-Myel, SC-Nonmyel)								
**gene**	**p_val**	**avg_log2FC**	**pct.1**	**pct.2**	**p_val_adj**	**Foldchange_UP**	**Foldchange_DOWN**	**Ratio_pct1_pct2**
ENPP2	0,683139676	-0,378463707	0,062	0,078	1	0,769256318	1,299956824	0,794871795
KLF3	0,115202665	-0,378514944	0,281	0,137	1	0,769228998	1,300002993	2,051094891
CCDC186	0,891985815	-0,378753946	0,094	0,094	1	0,769101576	1,300218373	1
CIC	0,122912861	-0,378806771	0,375	0,191	1	0,769073415	1,300265983	1,963350785
JDP2	0,322941334	-0,379060125	0,25	0,152	1	0,768938369	1,300494344	1,644736842
OTUD6B-AS1	0,000584485	-0,379112368	0,469	0,152	1	0,768910525	1,300541439	3,085526316
RAF1	0,551561351	-0,379369141	0,281	0,195	1	0,768773685	1,300772931	1,441025641
RHBDF1	0,067686507	-0,379474048	0,625	0,312	1	0,768717785	1,300867522	2,003205128
TCF7L1	0,093802134	-0,379631856	0	0,082	1	0,768633704	1,301009824	0
ZMYM2	0,705829061	-0,379718021	0,281	0,215	1	0,768587799	1,301087529	1,306976744
TTC14	0,090673844	-0,3797747	0,219	0,098	1	0,768557604	1,301138646	2,234693878
GLI3	0,62870361	-0,379789085	0,188	0,137	1	0,768549941	1,30115162	1,372262774
SLC2A3	0,535935517	-0,37992026	0,156	0,105	1	0,768480065	1,30126993	1,485714286
HSBP1L1	0,263419653	-0,379932761	0,031	0,086	1	0,768473406	1,301281206	0,360465116
PALM	0,147320294	-0,380288295	0	0,062	1	0,768284048	1,30160193	0
CHD6	0,191924078	-0,380516124	0,344	0,184	1	0,768162731	1,301807494	1,869565217
SNX13	0,378844198	-0,380610423	0,219	0,133	1	0,768112524	1,301892586	1,646616541
GNG12	0,661090749	-0,381071549	0,562	0,371	1	0,767867052	1,302308775	1,514824798
ARHGEF12	0,104361714	-0,381248288	0,531	0,266	1	0,767772989	1,302468326	1,996240602
GTF2B	0,150581114	-0,381388029	0,312	0,168	1	0,767698626	1,30259449	1,857142857
TMEM106B	0,048773235	-0,381404446	0,594	0,273	1	0,76768989	1,302609313	2,175824176
AVPI1	0,724493459	-0,381423245	0,094	0,105	1	0,767679887	1,302626286	0,895238095
KCNMB4	0,059738382	-0,381494884	0,562	0,273	1	0,767641767	1,302690972	2,058608059
SH3RF1	0,047008515	-0,381618181	0,312	0,137	1	0,767576165	1,302802308	2,277372263
SSU72	0,084485451	-0,381665225	0,438	0,211	1	0,767551137	1,302844791	2,075829384
BCAS3	0,864972765	-0,381852601	0,062	0,066	1	0,767451454	1,303014015	0,939393939
SIN3A	0,940300606	-0,381870679	0,156	0,137	1	0,767441837	1,303030342	1,138686131
DIP2A	0,421195014	-0,38212146	0,219	0,141	1	0,767308446	1,303256866	1,553191489
CNRIP1	0,761165734	-0,382521404	0,094	0,102	1	0,767095762	1,303618204	0,921568627
SRSF10	0,254882297	-0,382560888	0,5	0,297	1	0,767074768	1,303653882	1,683501684

Supplementary Table 1

Differentially expressed genes_adjacent-skin-XL_ comparing SC-Keloid vs. (SC-Myel, SC-Nonmyel)								
**gene**	**p_val**	**avg_log2FC**	**pct.1**	**pct.2**	**p_val_adj**	**Foldchange_UP**	**Foldchange_DOWN**	**Ratio_pct1_pct2**
CYP2U1	0,499735776	-0,382952313	0,031	0,059	1	0,766866678	1,304007632	0,525423729
MYO1C	0,582442201	-0,383064913	0,438	0,312	1	0,766806827	1,304109411	1,403846154
SERINC1	0,824329963	-0,38313766	0,75	0,477	1	0,766768162	1,304175172	1,572327044
CA5B	0,286570971	-0,383307374	0,031	0,082	1	0,766677967	1,3043286	0,37804878
PLEKHB2	0,127653578	-0,383361099	0,438	0,227	1	0,766649417	1,304377173	1,929515419
RAB30-DT	0,764452551	-0,38343445	0,094	0,102	1	0,76661044	1,304443493	0,921568627
PIP4K2A	0,759867431	-0,383481991	0,156	0,121	1	0,766585178	1,30448648	1,289256198
TSPYL1	0,381130252	-0,38355032	0,25	0,156	1	0,766548872	1,304548264	1,602564103
PSEN1	0,739785061	-0,383644146	0,062	0,074	1	0,766499021	1,304633108	0,837837838
SOS1	0,92888861	-0,383685358	0,094	0,082	1	0,766477125	1,304670377	1,146341463
DDX21	0,180917725	-0,383784361	0,531	0,297	1	0,766424529	1,304759911	1,787878788
FAM126B	0,932622158	-0,383848129	0,094	0,082	1	0,766390653	1,304817583	1,146341463
RGPD5	0,147320294	-0,384027494	0	0,062	1	0,766295376	1,304979817	0
JADE1	0,795919846	-0,384083593	0,156	0,121	1	0,76626558	1,305030562	1,289256198
PRDM2	0,460709201	-0,384295919	0,188	0,121	1	0,766152814	1,305222641	1,553719008
ZNF844	0,194053346	-0,384419166	0	0,051	1	0,766087366	1,305334149	0
SLC6A8	0,476871703	-0,384476235	0,125	0,164	1	0,766057062	1,305385785	0,762195122
GLG1	0,143447781	-0,384561148	0,656	0,344	1	0,766011975	1,30546262	1,906976744
NAV3	0,739785061	-0,384563799	0,062	0,074	1	0,766010568	1,305465018	0,837837838
GATAD2B	0,846196797	-0,384676923	0,125	0,125	1	0,765950506	1,305567385	1
TOX	0,180934293	-0,384822233	0,031	0,102	1	0,765873362	1,305698891	0,303921569
PM20D2	0,122891724	-0,384856489	0	0,07	1	0,765855178	1,305729894	0
REV3L	0,354261856	-0,38500438	0,125	0,07	1	0,765776674	1,305863751	1,785714286
ZCCHC2	0,093802134	-0,385383139	0	0,082	1	0,765575656	1,306206633	0
SDR39U1	0,430375867	-0,385590142	0,219	0,137	1	0,765465816	1,306394066	1,598540146
STOX2	0,590709689	-0,385652518	0,062	0,086	1	0,765432722	1,306450549	0,720930233
EIF5B	0,454104776	-0,385915974	0,5	0,328	1	0,765292956	1,306689147	1,524390244
WBP2	0,357140161	-0,386249496	0,312	0,188	1	0,765116056	1,306991263	1,659574468
RECK	0,344592621	-0,386750382	0,031	0,074	1	0,764850463	1,307445112	0,418918919
MEF2D	0,829082683	-0,38690827	0,219	0,211	1	0,764766763	1,307588206	1,037914692

Supplementary Table 1

Differentially expressed genes_adjacent-skin-XL_ comparing SC-Keloid vs. (SC-Myel, SC-Nonmyel)								
**gene**	**p_val**	**avg_log2FC**	**pct.1**	**pct.2**	**p_val_adj**	**Foldchange_UP**	**Foldchange_DOWN**	**Ratio_pct1_pct2**
COPS2	0,097309644	-0,387365941	0,625	0,32	1	0,764524192	1,308003082	1,953125
PHF23	0,727694156	-0,387566821	0,125	0,094	1	0,764417747	1,308185221	1,329787234
MAPRE2	0,606764606	-0,38766631	0,438	0,312	1	0,764365035	1,308275437	1,403846154
ZNF276	0,784260716	-0,387781212	0,094	0,102	1	0,76430416	1,308379638	0,921568627
PSAT1	0,65562104	-0,387934543	0,156	0,113	1	0,764222933	1,308518701	1,380530973
FGFR1OP2	0,216855361	-0,388094742	0,375	0,207	1	0,764138078	1,308664009	1,811594203
ZCCHC7	0,630272476	-0,388142597	0,062	0,082	1	0,764112731	1,308707419	0,756097561
ING5	0,784260716	-0,388470501	0,125	0,098	1	0,763939079	1,309004903	1,275510204
PDE4D	0,830197375	-0,388884479	0,125	0,102	1	0,7637199	1,309380572	1,225490196
TMEM100	0,933712959	-0,388955555	0,062	0,062	1	0,763682275	1,309445082	1
CNTFR	0,112290358	-0,389265965	0	0,074	1	0,763517979	1,309726853	0
ZFYVE21	0,399428356	-0,389566081	0,438	0,273	1	0,763359165	1,309999336	1,604395604
ASTN2	0,335280915	-0,389707779	0,062	0,117	1	0,763284194	1,310128008	0,52991453
TM2D1	0,518918986	-0,390002754	0,188	0,125	1	0,763128148	1,310395905	1,504
FAM53B	0,371015466	-0,390272093	0,188	0,113	1	0,762985691	1,310640568	1,663716814
PROS1	0,275671339	-0,390301899	0,312	0,18	1	0,762969928	1,310667646	1,733333333
LRIG1	0,929115609	-0,390348955	0,156	0,133	1	0,762945043	1,310710396	1,172932331
DUSP8	0,168918316	-0,390644616	0,469	0,258	1	0,762788704	1,310979037	1,817829457
MYADM	0,275690751	-0,391387705	0,656	0,383	1	0,762395916	1,311654456	1,712793734
DUSP7	0,633679827	-0,391762618	0,062	0,082	1	0,762197817	1,311995361	0,756097561
SRGAP2	0,351736754	-0,391822237	0,375	0,234	1	0,76216632	1,312049579	1,602564103
SREK1	0,120415954	-0,392645615	0,438	0,227	1	0,761731459	1,312798609	1,929515419
ZNF292	0,34004375	-0,393158794	0,188	0,113	1	0,761460553	1,313265665	1,663716814
ANKRD11	0,271175856	-0,393301566	0,531	0,309	1	0,761385201	1,313395635	1,718446602
MAN2C1	0,294412282	-0,394044779	0,156	0,086	1	0,76099307	1,314072413	1,813953488
CAB39	0,484579232	-0,394158046	0,188	0,121	1	0,760933326	1,314175586	1,553719008
CNOT1	0,256016384	-0,394401251	0,281	0,16	1	0,760805061	1,314397144	1,75625
AKIRIN2	0,483515905	-0,394602374	0,281	0,184	1	0,760699007	1,314580394	1,527173913
ALDH2	0,761165734	-0,395054583	0,094	0,102	1	0,760460605	1,314992511	0,921568627
RAP1GAP	0,286570971	-0,395245249	0,031	0,082	1	0,760360109	1,315166311	0,37804878

Supplementary Table 1

Differentially expressed genes_adjacent-skin-XL_ comparing SC-Keloid vs. (SC-Myel, SC-Nonmyel)								
**gene**	**p_val**	**avg_log2FC**	**pct.1**	**pct.2**	**p_val_adj**	**Foldchange_UP**	**Foldchange_DOWN**	**Ratio_pct1_pct2**
SNAP23	0,721126199	-0,395393294	0,125	0,094	1	0,760282088	1,315301276	1,329787234
CLDN11	0,54128594	-0,395413868	0,062	0,09	1	0,760271245	1,315320034	0,688888889
FAM107B	0,643001647	-0,395425086	0,281	0,207	1	0,760265334	1,315330261	1,357487923
WDR70	0,679002061	-0,395551319	0,156	0,113	1	0,760198815	1,315445355	1,380530973
UGT8	0,344592621	-0,39580923	0,031	0,074	1	0,760062926	1,315680539	0,418918919
FNDC3A	0,103443287	-0,395960007	0,406	0,211	1	0,759983496	1,315818048	1,924170616
ARHGEF28	0,286570971	-0,39599998	0,031	0,082	1	0,759962439	1,315854507	0,37804878
WDFY2	0,161996001	-0,396320018	0,219	0,109	1	0,759793872	1,31614644	2,009174312
PKN2	0,625841939	-0,396536187	0,344	0,242	1	0,759680036	1,316343662	1,421487603
SYNPO2	0,377976391	-0,396627776	0,031	0,07	1	0,759631809	1,316427232	0,442857143
C1orf56	0,533757264	-0,396868558	0,344	0,23	1	0,759505039	1,316646959	1,495652174
GALNT17	0,111920774	-0,397196193	0,281	0,137	1	0,759332576	1,316946002	2,051094891
PDE4DIP	0,99198594	-0,397408394	0,125	0,117	1	0,759220897	1,317139721	1,068376068
PLEKHO2	0,629239656	-0,397882067	0,094	0,113	1	0,758971666	1,317572242	0,831858407
ZBTB21	0,91311971	-0,39795255	0,188	0,18	1	0,758934587	1,317636614	1,044444444
LTBR	0,425192201	-0,397970118	0,188	0,117	1	0,758925346	1,317652659	1,606837607
COPA	0,104921292	-0,398154804	0,375	0,191	1	0,758828198	1,317821349	1,963350785
NSD1	0,041354995	-0,398180596	0,375	0,168	1	0,758814632	1,317844909	2,232142857
RIT1	0,09828181	-0,398284501	0,406	0,203	1	0,758759984	1,317939825	2
SEC24B	0,314221703	-0,398526689	0,031	0,078	1	0,75863262	1,318161089	0,397435897
ZNRF2	0,157750115	-0,398618587	0,438	0,23	1	0,758584297	1,318245057	1,904347826
TNFSF9	0,176914647	-0,398659316	0	0,055	1	0,758562882	1,318282273	0
ZFC3H1	0,518918986	-0,399140658	0,188	0,125	1	0,758309836	1,318722179	1,504
BMP8B	0,102705832	-0,399463229	0,375	0,188	1	0,758140305	1,319017064	1,994680851
RAB1A	0,209331887	-0,399961081	0,5	0,27	1	0,757878728	1,319472315	1,851851852
UTRN	0,599452816	-0,400239199	0,625	0,414	1	0,757732641	1,319726703	1,509661836
CDADC1	0,622425736	-0,400354329	0,125	0,141	1	0,757672175	1,319832024	0,886524823
NANOS1	0,134529645	-0,400567682	0	0,066	1	0,757560134	1,320027222	0
BRAF	0,748489035	-0,400821312	0,219	0,168	1	0,757426965	1,320259307	1,303571429
NOTCH2	0,933712959	-0,401079988	0,062	0,062	1	0,75729117	1,320496052	1

Supplementary Table 1

Differentially expressed genes_adjacent-skin-XL_ comparing SC-Keloid vs. (SC-Myel, SC-Nonmyel)								
**gene**	**p_val**	**avg_log2FC**	**pct.1**	**pct.2**	**p_val_adj**	**Foldchange_UP**	**Foldchange_DOWN**	**Ratio_pct1_pct2**
ODC1	0,320929119	-0,401274979	0,375	0,23	1	0,757188823	1,320674539	1,630434783
NRIP1	0,743682421	-0,401441333	0,094	0,105	1	0,757101519	1,320826832	0,895238095
TSIX	0,161397126	-0,401786432	0	0,059	1	0,756920438	1,321142817	0
DOT1L	0,888467101	-0,40248506	0,094	0,094	1	0,756553987	1,321782738	1
SUN2	0,139091795	-0,402575333	0,5	0,266	1	0,756506649	1,321865447	1,879699248
PYGL	0,676061779	-0,402751615	0,156	0,113	1	0,756414217	1,322026975	1,380530973
DYNLL2	0,074070998	-0,403613168	0,312	0,145	1	0,755962634	1,322816703	2,151724138
LMBR1L	0,804306744	-0,403689936	0,062	0,07	1	0,75592241	1,322887094	0,885714286
RELB	0,73148486	-0,403766453	0,156	0,117	1	0,755882318	1,322957259	1,333333333
SHROOM1	0,499735776	-0,404189492	0,031	0,059	1	0,755660705	1,323345245	0,525423729
PLEKHA5	0,093064088	-0,404590816	0,375	0,188	1	0,755450527	1,323713419	1,994680851
ALDH3A2	0,369291357	-0,404664497	0,281	0,172	1	0,755411946	1,323781026	1,63372093
PIK3IP1	0,894553025	-0,404683527	0,156	0,148	1	0,755401982	1,323798487	1,054054054
PHF2	0,891985815	-0,405071603	0,094	0,094	1	0,75519881	1,324154628	1
TNFRSF25	0,637094964	-0,405600709	0,062	0,082	1	0,754921893	1,324640349	0,756097561
YTHDC2	0,780840172	-0,405891356	0,156	0,121	1	0,754769821	1,324907239	1,289256198
CSNK1E	0,212322827	-0,405916742	0,594	0,34	1	0,75475654	1,324930553	1,747058824
IGFBP4	0,177714422	-0,406468494	0,5	0,273	1	0,754467942	1,325437363	1,831501832
BTG1	0,259697423	-0,406623955	0,906	0,559	1	0,754386647	1,325580196	1,620751342
AKR1C3	0,730358576	-0,40732641	0,031	0,043	1	0,754019422	1,326225785	0,720930233
ITCH	0,516441398	-0,407410343	0,188	0,125	1	0,753975556	1,326302944	1,504
ATF4	0,997181982	-0,407691393	0,781	0,531	1	0,753828689	1,326561346	1,470809793
NT5DC3	0,998160744	-0,40804922	0,094	0,086	1	0,753641743	1,326890409	1,093023256
RNF168	0,795919922	-0,408265417	0,156	0,121	1	0,753528813	1,327089266	1,289256198
LCAT	0,796107586	-0,408270939	0,125	0,133	1	0,753525929	1,327094346	0,939849624
MT-ND5	0,581668786	-0,40870256	0,844	0,531	1	0,753300525	1,327491442	1,589453861
LRRC8C	0,180934293	-0,408729587	0,031	0,102	1	0,753286413	1,327516311	0,303921569
PDE5A	0,935028354	-0,40889686	0,094	0,09	1	0,753199078	1,327670238	1,044444444
SYNJ2BP	0,376840213	-0,409229048	0,219	0,133	1	0,75302567	1,327975977	1,646616541
THUMPD1	0,563751658	-0,409510799	0,188	0,129	1	0,752878623	1,328235349	1,457364341

Supplementary Table 1

Differentially expressed genes_adjacent-skin-XL_ comparing SC-Keloid vs. (SC-Myel, SC-Nonmyel)								
**gene**	**p_val**	**avg_log2FC**	**pct.1**	**pct.2**	**p_val_adj**	**Foldchange_UP**	**Foldchange_DOWN**	**Ratio_pct1_pct2**
DNAJC27	0,874415164	-0,409517929	0,094	0,094	1	0,752874902	1,328241913	1
EFCAB14	0,778379363	-0,409638904	0,188	0,145	1	0,752811773	1,328353295	1,296551724
WARS1	0,081948379	-0,409667034	0,312	0,148	1	0,752797095	1,328379196	2,108108108
PPA1	0,109648938	-0,409810317	0,469	0,242	1	0,752722334	1,328511132	1,938016529
SASH1	0,039155566	-0,410009077	0,312	0,133	1	0,752618638	1,328694174	2,345864662
SLC25A28	0,684934253	-0,410018456	0,188	0,137	1	0,752613746	1,328702811	1,372262774
BNIP3	0,814811285	-0,410031488	0,188	0,145	1	0,752606947	1,328714814	1,296551724
NEDD1	0,998160744	-0,41030938	0,094	0,086	1	0,752461994	1,328970776	1,093023256
LRRC1	0,309789541	-0,410351206	0,062	0,121	1	0,752440179	1,329009305	0,512396694
PGGHG	0,161397126	-0,41040295	0	0,059	1	0,752413193	1,329056973	0
PPP4R3A	0,704239657	-0,410504699	0,188	0,141	1	0,752360129	1,32915071	1,333333333
EIF1	0,05695683	-0,410921704	1	0,945	1	0,752142694	1,329534951	1,058201058
ARIH1	0,046571605	-0,411076757	0,406	0,188	1	0,752061862	1,32967785	2,159574468
ARL4A	0,793909321	-0,411498652	0,156	0,16	1	0,751841965	1,330066752	0,975
MT-ND4	0,246764884	-0,41159891	0,875	0,879	1	0,751789718	1,330159187	0,995449374
ZDHHC8	0,723273635	-0,411866991	0,125	0,133	1	0,751650034	1,330406379	0,939849624
COBLL1	0,378729131	-0,411965752	0,219	0,137	1	0,751598581	1,330497456	1,598540146
MYO18A	0,935028354	-0,411992275	0,094	0,09	1	0,751584763	1,330521917	1,044444444
OSBPL1A	0,494404353	-0,412292101	0,219	0,145	1	0,751428583	1,330798459	1,510344828
FGF1	0,377976391	-0,412571552	0,031	0,07	1	0,751283045	1,331056261	0,442857143
PLEKHA7	0,112290358	-0,412690604	0	0,074	1	0,751221051	1,331166104	0
CNOT2	0,169219927	-0,412839914	0,312	0,164	1	0,751143308	1,331303879	1,902439024
POLK	0,54829256	-0,413439175	0,219	0,148	1	0,750831366	1,331856986	1,47972973
TMEM176B	0,74527186	-0,414554835	0,562	0,465	1	0,75025096	1,332887332	1,208602151
ILKAP	0,879520665	-0,415169295	0,219	0,18	1	0,749931488	1,333455144	1,216666667
INTS11	0,125718416	-0,416106408	0,25	0,121	1	0,749444523	1,334321581	2,066115702
KDM2A	0,162595876	-0,416308706	0,344	0,18	1	0,749339441	1,334508695	1,911111111
H1-10	0,246397264	-0,41634369	0,594	0,355	1	0,749321271	1,334541056	1,673239437
C11orf68	0,471015282	-0,416369652	0,219	0,141	1	0,749307786	1,334565072	1,553191489
ROCK2	0,199197135	-0,416456983	0,5	0,273	1	0,74926243	1,33464586	1,831501832

Supplementary Table 1

Differentially expressed genes_adjacent-skin-XL_ comparing SC-Keloid vs. (SC-Myel, SC-Nonmyel)								
**gene**	**p_val**	**avg_log2FC**	**pct.1**	**pct.2**	**p_val_adj**	**Foldchange_UP**	**Foldchange_DOWN**	**Ratio_pct1_pct2**
FZD7	0,112290358	-0,416512015	0	0,074	1	0,749233849	1,334696772	0
BMPR1B	0,286570971	-0,416515351	0,031	0,082	1	0,749232117	1,334699858	0,37804878
PTTG1	0,099632778	-0,416711854	0,219	0,102	1	0,749130074	1,334881664	2,147058824
CCSAP	0,122891724	-0,41712194	0	0,07	1	0,748917165	1,335261158	0
CRNDE	0,800356249	-0,417142928	0,062	0,07	1	0,748906269	1,335280583	0,885714286
MFAP4	0,078384714	-0,417516202	0	0,09	1	0,748712527	1,33562611	0
AKAP11	0,914087118	-0,417587877	0,156	0,133	1	0,748675331	1,335692467	1,172932331
PARD3B	0,122891724	-0,418081539	0	0,07	1	0,748419193	1,336149593	0
RTL5	0,147319971	-0,41811755	0	0,062	1	0,748400512	1,336182945	0
IGF1R	0,983447865	-0,418235321	0,094	0,086	1	0,74833942	1,336292026	1,093023256
KITLG	0,10262374	-0,418714743	0	0,078	1	0,748090781	1,336736163	0
SENP5	0,79289767	-0,41912952	0,156	0,121	1	0,747875735	1,337120532	1,289256198
STK3	0,775937953	-0,419156019	0,125	0,129	1	0,747861998	1,337145092	0,968992248
EVI5	0,113010074	-0,419650898	0,438	0,223	1	0,747605508	1,337603843	1,964125561
SDC4	0,386049732	-0,41969156	0,188	0,113	1	0,747584437	1,337641544	1,663716814
GPATCH2L	0,501783108	-0,419976389	0,156	0,102	1	0,747436857	1,337905659	1,529411765
PPM1B	0,249959309	-0,420097764	0,312	0,176	1	0,747373977	1,338018223	1,772727273
LAMP1	0,944623414	-0,420210294	0,812	0,527	1	0,747315684	1,338122591	1,540796964
NFATC4	0,26138036	-0,420265368	0,031	0,086	1	0,747287156	1,338173674	0,360465116
RHOQ	0,430724357	-0,420739487	0,438	0,293	1	0,747041612	1,338613517	1,494880546
CERS1	0,71496263	-0,420845954	0,094	0,105	1	0,746986485	1,338712306	0,895238095
TNKS2	0,709693094	-0,421023298	0,188	0,141	1	0,746894667	1,338876878	1,333333333
PCNP	0,443094633	-0,421049259	0,625	0,371	1	0,746881227	1,338900972	1,684636119
RBL2	0,416531335	-0,421556509	0,062	0,102	1	0,74661867	1,33937181	0,607843137
ZNF280D	0,250807401	-0,421810974	0,219	0,121	1	0,746486992	1,339608071	1,809917355
CXCL3	0,213026321	-0,421867788	0	0,047	1	0,746457596	1,339660827	0
FGD5-AS1	0,150020598	-0,422075694	0,406	0,215	1	0,746350032	1,339853898	1,888372093
TP53INP2	0,868757226	-0,422174142	0,094	0,078	1	0,746299103	1,339945332	1,205128205
ASPA	0,235102422	-0,422306925	0,594	0,34	1	0,746230418	1,340068664	1,747058824
DYNLRB1	0,982173576	-0,423250542	0,812	0,531	1	0,745742495	1,340945443	1,529190207

Supplementary Table 1

Differentially expressed genes_adjacent-skin-XL_ comparing SC-Keloid vs. (SC-Myel, SC-Nonmyel)								
**gene**	**p_val**	**avg_log2FC**	**pct.1**	**pct.2**	**p_val_adj**	**Foldchange_UP**	**Foldchange_DOWN**	**Ratio_pct1_pct2**
AL627171.2	0,504146063	-0,424877964	0,188	0,125	1	0,744901739	1,342458941	1,504
CHIC2	0,676784237	-0,42489987	0,188	0,137	1	0,744890429	1,342479325	1,372262774
TNRC6C	0,89455306	-0,424965244	0,156	0,148	1	0,744856676	1,342540159	1,054054054
SH3GLB2	0,773567054	-0,425039359	0,344	0,262	1	0,744818411	1,342609131	1,312977099
ZC3H4	0,507127635	-0,425318396	0,156	0,102	1	0,744674367	1,342868835	1,529411765
YLPM1	0,881436494	-0,425336698	0,094	0,094	1	0,74466492	1,342885871	1
ADAM33	0,161397126	-0,42534141	0	0,059	1	0,744662488	1,342890257	0
NPHP3	0,452272907	-0,425390504	0,156	0,098	1	0,744637148	1,342935955	1,591836735
MYCBP2	0,320338862	-0,425396886	0,25	0,148	1	0,744633854	1,342941896	1,689189189
NR2F2-AS1	0,686731872	-0,425578409	0,062	0,078	1	0,744540169	1,343110879	0,794871795
WDR26	0,358918639	-0,42560648	0,094	0,145	1	0,744525682	1,343137012	0,648275862
ZNF516	0,786862533	-0,426616887	0,156	0,121	1	0,744004428	1,344078022	1,289256198
RBMXL1	0,66795303	-0,426621209	0,094	0,109	1	0,744002199	1,344082049	0,862385321
EZH1	0,884950692	-0,426884046	0,094	0,094	1	0,743866666	1,344326942	1
KCNH8	0,286570971	-0,42692071	0,031	0,082	1	0,743847762	1,344361106	0,37804878
RUSC2	0,614662208	-0,426987766	0,094	0,113	1	0,743813189	1,344423593	0,831858407
CRIM1	0,677114428	-0,427804879	0,094	0,109	1	0,743392027	1,345185263	0,862385321
ANKRD12	0,214766065	-0,427806266	0,594	0,324	1	0,743391312	1,345186557	1,833333333
RHPN1	0,498361807	-0,427942865	0,062	0,094	1	0,743320929	1,345313929	0,659574468
DMXL1	0,89512162	-0,427991389	0,125	0,105	1	0,743295929	1,345359178	1,19047619
TRIM69	0,788750462	-0,42809388	0,188	0,148	1	0,743243126	1,345454758	1,27027027
PALMD	0,176914647	-0,428161517	0	0,055	1	0,743208282	1,345517837	0
TXNRD2	0,679002061	-0,428295828	0,156	0,113	1	0,743139094	1,345643108	1,380530973
PAN3	0,220157452	-0,428451574	0,281	0,156	1	0,743058873	1,345788384	1,801282051
HNRNPL	0,313250979	-0,428459085	0,531	0,305	1	0,743055004	1,345795391	1,740983607
NCAM2	0,869232693	-0,428649774	0,125	0,121	1	0,742956797	1,345973284	1,033057851
C11orf96	0,269438702	-0,428671372	0,281	0,176	1	0,742945675	1,345993433	1,596590909
GPSM1	0,372854694	-0,428736469	0,281	0,172	1	0,742912152	1,346054169	1,63372093
PDCD6IP	0,127653578	-0,428808009	0,438	0,227	1	0,742875314	1,346120918	1,929515419
ESPN	0,059869253	-0,42895401	0	0,102	1	0,742800139	1,346257153	0

Supplementary Table 1

Differentially expressed genes_adjacent-skin-XL_ comparing SC-Keloid vs. (SC-Myel, SC-Nonmyel)								
**gene**	**p_val**	**avg_log2FC**	**pct.1**	**pct.2**	**p_val_adj**	**Foldchange_UP**	**Foldchange_DOWN**	**Ratio_pct1_pct2**
USP24	0,935028354	-0,43023345	0,094	0,09	1	0,742141686	1,347451598	1,044444444
PHLDA3	0,08577078	-0,430256092	0,5	0,25	1	0,742130039	1,347472745	2
KIAA1217	0,864647308	-0,430538359	0,125	0,125	1	0,741984853	1,347736407	1
SLITRK2	0,199956022	-0,430992092	0,031	0,098	1	0,741751533	1,348160341	0,316326531
ALDH1A1	0,112290358	-0,431867663	0	0,074	1	0,7413015	1,348978789	0
YAP1	0,278057939	-0,432118361	0,281	0,16	1	0,741172695	1,349213221	1,75625
CLDND2	0,08574604	-0,43240253	0	0,086	1	0,74102672	1,349479003	0
BTG3	0,370524765	-0,432861211	0,625	0,383	1	0,740791161	1,349908116	1,631853786
IP6K1	0,975643601	-0,433473042	0,125	0,113	1	0,740477066	1,350480718	1,10619469
DAB1	0,112290358	-0,433692097	0	0,074	1	0,740364643	1,350685787	0
MAGI2	0,257984542	-0,43373008	0,25	0,145	1	0,74034515	1,350721349	1,724137931
ARHGAP23	0,764452445	-0,433881429	0,094	0,102	1	0,740267487	1,350863056	0,921568627
ITFG2	0,516147046	-0,433980812	0,094	0,059	1	0,740216494	1,350956117	1,593220339
MT-ATP8	0,38996635	-0,434663964	0,281	0,176	1	0,739866066	1,351595979	1,596590909
NKAIN2	0,112290358	-0,434792322	0	0,074	1	0,739800242	1,351716238	0
JMJD6	0,821728391	-0,434841644	0,125	0,125	1	0,739774951	1,35176245	1
TNFRSF14	0,474951714	-0,434914272	0,188	0,121	1	0,73973771	1,351830502	1,553719008
ATP11A	0,584240026	-0,434990455	0,062	0,086	1	0,739698649	1,351901889	0,720930233
TET1	0,826816092	-0,435020798	0,156	0,125	1	0,739683091	1,351930323	1,248
TOX2	0,630272476	-0,435109377	0,062	0,082	1	0,739637678	1,352013331	0,756097561
RARA	0,137266353	-0,435241402	0,344	0,176	1	0,739569994	1,352137063	1,954545455
TMEM35B	0,092805973	-0,435441052	0,469	0,227	1	0,739467654	1,352324195	2,066079295
TNRC6A	0,18989486	-0,435761515	0,312	0,168	1	0,739303416	1,352624617	1,857142857
MBD5	0,419048035	-0,436011966	0,062	0,102	1	0,739175085	1,352859452	0,607843137
FUBP1	0,381855801	-0,436074659	0,281	0,172	1	0,739142964	1,352918242	1,63372093
AFF1	0,453137599	-0,43638319	0,062	0,098	1	0,73898491	1,353207605	0,632653061
AHCTF1	0,768835141	-0,436441276	0,156	0,121	1	0,738955158	1,353262089	1,289256198
CYTH1	0,593956834	-0,436807533	0,062	0,086	1	0,738767583	1,353605685	0,720930233
NRG1	0,134529645	-0,436909803	0	0,066	1	0,738715215	1,353701644	0
HERPUD1	0,121574842	-0,437861637	0,531	0,285	1	0,738228	1,354595058	1,863157895

Supplementary Table 1

Differentially expressed genes_adjacent-skin-XL_ comparing SC-Keloid vs. (SC-Myel, SC-Nonmyel)								
**gene**	**p_val**	**avg_log2FC**	**pct.1**	**pct.2**	**p_val_adj**	**Foldchange_UP**	**Foldchange_DOWN**	**Ratio_pct1_pct2**
MRE11	0,935028354	-0,437870324	0,094	0,09	1	0,738223555	1,354603214	1,044444444
HMGA1	0,120752413	-0,437882713	0,375	0,203	1	0,738217216	1,354614846	1,84729064
SFN	0,263449854	-0,438690232	0,281	0,164	1	0,737804129	1,355373277	1,713414634
PKD2	0,826565871	-0,438902172	0,156	0,156	1	0,737695749	1,355572404	1
LRRC58	0,285844171	-0,438943133	0,312	0,18	1	0,737674805	1,355610891	1,733333333
CCNT2	0,611764646	-0,43940387	0,094	0,113	1	0,73743926	1,356043886	0,831858407
TDRD3	0,623390898	-0,4395783	0,094	0,113	1	0,737350105	1,356207849	0,831858407
SCD5	0,134529645	-0,439745505	0	0,066	1	0,737264652	1,35636504	0
RFX2	0,134529645	-0,439798946	0	0,066	1	0,737237343	1,356415284	0
TMEM255A	0,093802134	-0,439831932	0	0,082	1	0,737220487	1,356446298	0
PON2	0,319403387	-0,44026021	0,656	0,359	1	0,737001668	1,356849032	1,82729805
FKBP5	0,220913711	-0,440346433	0,188	0,098	1	0,736957622	1,356930127	1,918367347
AKAP13	0,328814009	-0,44040983	0,812	0,453	1	0,736925239	1,356989756	1,792494481
CIT	0,42035466	-0,440756858	0,281	0,18	1	0,736747999	1,357316208	1,561111111
SESTD1	0,684934253	-0,441490117	0,188	0,137	1	0,736373637	1,358006247	1,372262774
CRYZL1	0,194605756	-0,441664167	0,219	0,113	1	0,736284805	1,35817009	1,938053097
AGO3	0,516587316	-0,441814229	0,094	0,125	1	0,736208224	1,358311369	0,752
CPXM2	0,238418073	-0,441998599	0,031	0,09	1	0,736114146	1,358484966	0,344444444
RASAL2	0,855412171	-0,442355507	0,125	0,125	1	0,735932061	1,358821082	1
PARP4	0,253529282	-0,442778356	0,125	0,062	1	0,735716394	1,359219406	2,016129032
TUT4	0,122552691	-0,442896304	0,438	0,227	1	0,735656248	1,359330534	1,929515419
RCAN3	0,72064445	-0,44300868	0,188	0,141	1	0,735598947	1,359436421	1,333333333
SAV1	0,306674893	-0,444058787	0,125	0,188	1	0,735063715	1,360426286	0,664893617
RLIM	0,560183087	-0,444472276	0,094	0,121	1	0,73485307	1,360816252	0,776859504
CCDC12	0,245928411	-0,444479522	0,312	0,176	1	0,734849379	1,360823086	1,772727273
ST3GAL5	0,112290358	-0,444920686	0	0,074	1	0,734624702	1,361239279	0
ABHD5	0,676061901	-0,445401533	0,156	0,113	1	0,734379895	1,361693052	1,380530973
GPX3	0,924392442	-0,44669574	0,031	0,027	1	0,733721395	1,362915143	1,148148148
CSF1	0,220935523	-0,447264127	0,031	0,094	1	0,733432384	1,363452204	0,329787234
TAF7	0,445024927	-0,447627997	0,625	0,367	1	0,733247424	1,363796131	1,702997275

Supplementary Table 1

Differentially expressed genes_adjacent-skin-XL_ comparing SC-Keloid vs. (SC-Myel, SC-Nonmyel)								
**gene**	**p_val**	**avg_log2FC**	**pct.1**	**pct.2**	**p_val_adj**	**Foldchange_UP**	**Foldchange_DOWN**	**Ratio_pct1_pct2**
ARHGEF6	0,285257317	-0,448132288	0,062	0,121	1	0,732991164	1,364272926	0,512396694
HADH	0,799342981	-0,448536035	0,094	0,074	1	0,73278606	1,36465478	1,27027027
FA2H	0,180934293	-0,448828243	0,031	0,102	1	0,732637655	1,364931209	0,303921569
GSTM3	0,464378621	-0,449192994	0,25	0,164	1	0,732452448	1,365276344	1,524390244
SF1	0,334357247	-0,449321814	0,812	0,457	1	0,73238705	1,365398255	1,776805252
RELT	0,220935523	-0,449613112	0,031	0,094	1	0,732239186	1,365673975	0,329787234
SPOPL	0,683139498	-0,449745548	0,062	0,078	1	0,732171972	1,365799346	0,794871795
MICAL3	0,787831811	-0,450016119	0,156	0,125	1	0,732034669	1,366055519	1,248
FBXW11	0,400977729	-0,450529334	0,312	0,199	1	0,731774306	1,366541558	1,567839196
CIRBP	0,393323828	-0,450893246	0,812	0,656	1	0,731589743	1,366886304	1,237804878
ARHGEF40	0,4750123	-0,451862074	0,312	0,207	1	0,731098616	1,367804531	1,507246377
CLASRP	0,453137599	-0,452058224	0,062	0,098	1	0,730999222	1,367990512	0,632653061
DCXR	0,858704938	-0,45211266	0,188	0,148	1	0,73097164	1,36804213	1,27027027
NLGN3	0,352472909	-0,45268757	0,031	0,074	1	0,730680408	1,3685874	0,418918919
IER3	0,651974317	-0,453657966	0,75	0,574	1	0,730189098	1,369508258	1,306620209
DYNC2I1	0,792338755	-0,453702042	0,188	0,145	1	0,73016679	1,3695501	1,296551724
CBR4	0,683139676	-0,453708312	0,062	0,078	1	0,730163617	1,369556052	0,794871795
TUBB4B	0,547771204	-0,453801434	0,594	0,391	1	0,730116488	1,369644455	1,519181586
AFTPH	0,70409276	-0,454119052	0,188	0,137	1	0,729955767	1,369946024	1,372262774
ZNF608	0,388962014	-0,454192732	0,219	0,133	1	0,729918488	1,37001599	1,646616541
LTBP3	0,505862177	-0,454541794	0,656	0,422	1	0,729741904	1,370347508	1,55450237
FAXDC2	0,093802134	-0,45486224	0	0,082	1	0,729579835	1,370651918	0
METRNL	0,92470606	-0,455025384	0,219	0,191	1	0,729497337	1,370806924	1,146596859
DCN	0,714264918	-0,455605766	0,031	0,047	1	0,729203926	1,371358497	0,659574468
RFX1	0,936503132	-0,455767353	0,156	0,141	1	0,729122257	1,371512103	1,106382979
C1orf21	0,458526501	-0,456419087	0,438	0,285	1	0,728792952	1,37213182	1,536842105
C10orf82	0,217476928	-0,457696387	0,031	0,094	1	0,728147996	1,373347185	0,329787234
CNP	0,8930962	-0,457955171	0,656	0,488	1	0,728017396	1,373593551	1,344262295
CWC25	0,567357939	-0,458352271	0,094	0,117	1	0,727817038	1,373971683	0,803418803
NGF	0,933712959	-0,458359587	0,062	0,062	1	0,727813347	1,373978651	1

Supplementary Table 1

Differentially expressed genes_adjacent-skin-XL_ comparing SC-Keloid vs. (SC-Myel, SC-Nonmyel)								
**gene**	**p_val**	**avg_log2FC**	**pct.1**	**pct.2**	**p_val_adj**	**Foldchange_UP**	**Foldchange_DOWN**	**Ratio_pct1_pct2**
DDHD2	0,752148714	-0,458750419	0,125	0,133	1	0,727616206	1,374350918	0,939849624
TNK2	0,724407642	-0,459139707	0,125	0,094	1	0,727419897	1,374721814	1,329787234
TRAK1	0,311875867	-0,460210986	0,312	0,184	1	0,726879949	1,375742998	1,695652174
NFASC	0,147320294	-0,460611415	0	0,062	1	0,726678227	1,376124897	0
FAM241A	0,238418073	-0,46103909	0,031	0,09	1	0,726462841	1,376532899	0,344444444
MT-CYB	0,568438123	-0,46118783	0,875	0,805	1	0,726387947	1,376674825	1,086956522
CASC3	0,646129172	-0,461501455	0,156	0,172	1	0,726230057	1,37697413	0,906976744
CD9	0,040744367	-0,462007176	1	0,898	1	0,725975529	1,377456898	1,113585746
IFFO1	0,238418073	-0,462453829	0,031	0,09	1	0,725750804	1,37788342	0,344444444
CERS6	0,409017449	-0,462659117	0,062	0,105	1	0,725647541	1,378079499	0,59047619
ATXN3	0,838902498	-0,463093105	0,156	0,125	1	0,725429287	1,378494112	1,248
AMFR	0,260324649	-0,46384477	0,281	0,16	1	0,725051426	1,379212514	1,75625
MPDZ	0,295716939	-0,463915174	0,25	0,145	1	0,725016044	1,379279822	1,724137931
SPART	0,158963941	-0,463977892	0,281	0,145	1	0,724984526	1,379339784	1,937931034
CELF2	0,533456153	-0,464594736	0,594	0,363	1	0,724674615	1,379929667	1,636363636
PTPRJ	0,915013818	-0,46576293	0,125	0,105	1	0,724088062	1,38104749	1,19047619
SRSF6	0,058975014	-0,465976612	0,438	0,203	1	0,723980823	1,381252056	2,157635468
EIF4E	0,053592746	-0,465986373	0,531	0,258	1	0,723975925	1,381261401	2,058139535
MAVS	0,660994176	-0,466076306	0,125	0,09	1	0,723930795	1,381347508	1,388888889
POGZ	0,834073421	-0,466255719	0,25	0,227	1	0,723840773	1,381519302	1,101321586
CSAD	0,166418547	-0,466890784	0,281	0,148	1	0,723522213	1,382127572	1,898648649
YY1AP1	0,73994668	-0,467248096	0,188	0,141	1	0,723343041	1,382469925	1,333333333
ATP6V1B2	0,28568409	-0,467291243	0,281	0,16	1	0,723321408	1,382511272	1,75625
TRPC4AP	0,975310564	-0,467685547	0,125	0,109	1	0,723123744	1,382889178	1,146788991
LRRC8B	0,19837126	-0,468173253	0,031	0,098	1	0,722879332	1,383356746	0,316326531
EHBP1	0,699930189	-0,468285302	0,656	0,434	1	0,72282319	1,383464191	1,511520737
SERPINA3	0,548952158	-0,468313234	0,031	0,055	1	0,722809196	1,383490976	0,563636364
FSTL5	0,134529645	-0,468484207	0	0,066	1	0,722723541	1,383654943	0
EPC2	0,905539169	-0,468542054	0,156	0,129	1	0,722694563	1,383710424	1,209302326
NPC2	0,392955531	-0,469042835	0,625	0,355	1	0,722443748	1,384190814	1,76056338

Supplementary Table 1

Differentially expressed genes_adjacent-skin-XL_ comparing SC-Keloid vs. (SC-Myel, SC-Nonmyel)								
**gene**	**p_val**	**avg_log2FC**	**pct.1**	**pct.2**	**p_val_adj**	**Foldchange_UP**	**Foldchange_DOWN**	**Ratio_pct1_pct2**
MRPS31	0,416896114	-0,469113725	0,125	0,074	1	0,72240825	1,384258831	1,689189189
HERC1	0,945716537	-0,469221319	0,125	0,109	1	0,722354376	1,38436207	1,146788991
C7orf61	0,08574604	-0,469485428	0	0,086	1	0,72222215	1,384615524	0
MEG3	0,00329525	-0,469570095	0,719	0,285	1	0,722179766	1,384696785	2,522807018
CKAP4	0,122936942	-0,469828232	0,25	0,121	1	0,72205056	1,384944566	2,066115702
RBM25	0,455553576	-0,470020781	0,688	0,406	1	0,721954199	1,385129419	1,694581281
PLK2	0,527444083	-0,470167464	0,219	0,152	1	0,721880799	1,385270257	1,440789474
ANKRD18A	0,213026321	-0,47065802	0	0,047	1	0,721635381	1,385741367	0
BROX	0,811460203	-0,471280338	0,219	0,172	1	0,721324165	1,386339247	1,273255814
GATAD2A	0,536573979	-0,472089773	0,125	0,148	1	0,720919574	1,387117281	0,844594595
ELL	0,314221703	-0,472229915	0,031	0,078	1	0,720849549	1,387252031	0,397435897
TMEM161B-DT	0,455148041	-0,472365625	0,031	0,062	1	0,720781743	1,387382532	0,5
ADAMTS9	0,790538584	-0,472612344	0,219	0,172	1	0,720658491	1,387619812	1,273255814
TMEM128	0,353479652	-0,472644135	0,062	0,109	1	0,720642611	1,38765039	0,568807339
PHF1	0,63133634	-0,472845302	0,125	0,145	1	0,720542133	1,387843895	0,862068966
WBP1L	0,322870955	-0,47435123	0,219	0,129	1	0,719790402	1,389293323	1,697674419
ZNF219	0,726144259	-0,47504178	0,125	0,133	1	0,719445955	1,389958472	0,939849624
ZC3H6	0,475495989	-0,475365201	0,219	0,141	1	0,719284688	1,390270106	1,553191489
SMOX	0,270831373	-0,476224113	0,062	0,121	1	0,718856588	1,391098053	0,512396694
RBM7	0,616094331	-0,476301027	0,281	0,191	1	0,718818265	1,391172218	1,471204188
NRDE2	0,837002351	-0,477986737	0,125	0,125	1	0,717978856	1,392798676	1
TYRO3	0,08574604	-0,478859637	0	0,086	1	0,717544575	1,393641642	0
PRRC2B	0,520874723	-0,479281136	0,312	0,211	1	0,717334968	1,394048868	1,478672986
EGFL8	0,458755675	-0,480093362	0,844	0,656	1	0,716931228	1,394833928	1,286585366
KRT1	0,09097562	-0,480374208	0,031	0,141	1	0,716791677	1,395105484	0,219858156
FAM133B	0,521406761	-0,48037692	0,469	0,305	1	0,71679033	1,395108106	1,537704918
SRSF4	0,263679024	-0,480934036	0,375	0,211	1	0,716513585	1,39564695	1,777251185
RAB10	0,939107348	-0,481521264	0,5	0,359	1	0,716221999	1,396215143	1,39275766
IL11RA	0,73624035	-0,482343514	0,281	0,262	1	0,715813911	1,397011129	1,072519084
CCNL2	0,191876676	-0,482550462	0,406	0,227	1	0,715711238	1,397211539	1,788546256

Supplementary Table 1

Differentially expressed genes_adjacent-skin-XL_ comparing SC-Keloid vs. (SC-Myel, SC-Nonmyel)								
**gene**	**p_val**	**avg_log2FC**	**pct.1**	**pct.2**	**p_val_adj**	**Foldchange_UP**	**Foldchange_DOWN**	**Ratio_pct1_pct2**
SEC14L1	0,530052754	-0,48323046	0,312	0,203	1	0,715373975	1,397870253	1,536945813
SBNO2	0,560003828	-0,483325964	0,125	0,152	1	0,71532662	1,397962794	0,822368421
ANAPC16	0,135648283	-0,483341138	0,594	0,301	1	0,715319096	1,397977497	1,973421927
FKBP4	0,203993033	-0,483941055	0,406	0,23	1	0,715021707	1,39855894	1,765217391
NCOR1	0,044672041	-0,484114443	0,406	0,184	1	0,714935778	1,398727033	2,206521739
MARCHF6	0,937963083	-0,484218342	0,406	0,324	1	0,714884292	1,39882777	1,25308642
MICALL2	0,449453483	-0,484587281	0,469	0,301	1	0,714701499	1,399185537	1,558139535
SORBS2	0,47756212	-0,484656375	0,5	0,316	1	0,714667271	1,399252548	1,582278481
SPRY1	0,015693314	-0,485401731	0,344	0,141	1	0,71429814	1,399975646	2,439716312
PLEKHG3	0,059869253	-0,486227612	0	0,102	1	0,713889352	1,400777302	0
CLOCK	0,721126199	-0,486531796	0,125	0,094	1	0,713738848	1,401072679	1,329787234
PABPC1	0,908444375	-0,486846082	0,719	0,516	1	0,71358338	1,401377931	1,393410853
MTSS2	0,675735117	-0,487132761	0,125	0,137	1	0,713441597	1,401656427	0,912408759
MON2	0,799775352	-0,487235336	0,125	0,129	1	0,713390873	1,401756088	0,968992248
CDK9	0,964894825	-0,488092115	0,156	0,145	1	0,712967335	1,402588802	1,075862069
MED13	0,722343574	-0,488188281	0,312	0,227	1	0,712919812	1,402682298	1,374449339
SOX7	0,26138036	-0,488989207	0,031	0,086	1	0,712524138	1,403461226	0,360465116
TFAM	0,379561462	-0,489102993	0,188	0,113	1	0,712467943	1,403571923	1,663716814
CREBZF	0,922361175	-0,489274594	0,25	0,219	1	0,712383204	1,40373888	1,141552511
DHX15	0,890525449	-0,489535371	0,219	0,176	1	0,712254447	1,403992638	1,244318182
MID1	0,180934293	-0,489672144	0,031	0,102	1	0,712186926	1,404125748	0,303921569
GRAMD2B	0,140747594	-0,489689422	0,344	0,176	1	0,712178396	1,404142564	1,954545455
TMEM168	0,586985538	-0,489752827	0,188	0,129	1	0,712147098	1,404204277	1,457364341
NRDC	0,532574125	-0,490537173	0,312	0,207	1	0,711760032	1,404967904	1,507246377
KIAA1755	0,982137358	-0,49057596	0,125	0,113	1	0,711740896	1,405005677	1,10619469
CUX1	0,314654322	-0,491058403	0,375	0,219	1	0,711502927	1,405475595	1,712328767
JMJD1C	0,530326632	-0,491257104	0,531	0,348	1	0,711404939	1,405669183	1,525862069
PSIP1	0,258883021	-0,491318491	0,281	0,16	1	0,711374669	1,405728996	1,75625
NPIPA1	0,630272476	-0,491860392	0,062	0,082	1	0,711107515	1,406257111	0,756097561
PDE9A	0,071654957	-0,492199329	0	0,094	1	0,710940472	1,406587527	0

Supplementary Table 1

Differentially expressed genes_adjacent-skin-XL_ comparing SC-Keloid vs. (SC-Myel, SC-Nonmyel)								
**gene**	**p_val**	**avg_log2FC**	**pct.1**	**pct.2**	**p_val_adj**	**Foldchange_UP**	**Foldchange_DOWN**	**Ratio_pct1_pct2**
SIK2	0,198371032	-0,492706286	0,031	0,098	1	0,710690694	1,407081882	0,316326531
TNS1	0,99584873	-0,493364752	0,188	0,164	1	0,710366399	1,407724241	1,146341463
WNT6	0,180934079	-0,493457	0,031	0,102	1	0,710320979	1,407814256	0,303921569
KHDC4	0,594827904	-0,493963368	0,188	0,129	1	0,710071709	1,408308467	1,457364341
EZR	0,526054298	-0,493964304	0,562	0,355	1	0,710071248	1,408309381	1,583098592
NOLC1	0,188904652	-0,494058615	0,281	0,148	1	0,710024831	1,408401447	1,898648649
CAPRIN2	0,239501047	-0,494457995	0,062	0,129	1	0,709828303	1,408791388	0,480620155
ANKHD1	0,847556267	-0,494547602	0,25	0,223	1	0,709784216	1,408878892	1,121076233
CRY1	0,259540252	-0,494722713	0,25	0,141	1	0,709698069	1,409049909	1,773049645
TNFAIP2	0,507127635	-0,494779574	0,156	0,102	1	0,709670099	1,409105444	1,529411765
TOP1	0,510974938	-0,494813994	0,406	0,266	1	0,709653167	1,409139064	1,526315789
ZFHX3	0,496193648	-0,494876265	0,25	0,164	1	0,709622538	1,409199887	1,524390244
LMBRD1	0,197426112	-0,495557504	0,5	0,27	1	0,709287533	1,409865468	1,851851852
FRMD8	0,213436227	-0,496063998	0,062	0,133	1	0,709038564	1,410360523	0,466165414
TMED5	0,307253547	-0,496312389	0,312	0,184	1	0,708916499	1,410603367	1,695652174
RNF114	0,736411986	-0,496829516	0,406	0,285	1	0,708662436	1,411109082	1,424561404
DIXDC1	0,45890811	-0,496851086	0,156	0,191	1	0,708651841	1,41113018	0,816753927
NRP1	0,084505016	-0,496868178	0,25	0,113	1	0,708643445	1,411146898	2,212389381
SCD	0,43153954	-0,497253318	0,438	0,273	1	0,708454292	1,411523666	1,604395604
GALNT2	0,114455439	-0,497927216	0,625	0,316	1	0,708123443	1,412183158	1,977848101
C12orf57	0,419427842	-0,499063149	0,656	0,406	1	0,707566108	1,413295505	1,615763547
RASGEF1A	0,492632793	-0,499164784	0,062	0,094	1	0,707516263	1,413395072	0,659574468
KCTD3	0,977829359	-0,499530028	0,156	0,141	1	0,707337166	1,413752943	1,106382979
TARS3	0,926839124	-0,499788131	0,25	0,215	1	0,707210632	1,414005892	1,162790698
ARL8A	0,883675864	-0,499847725	0,406	0,293	1	0,70718142	1,414064301	1,385665529
AEBP2	0,192462217	-0,500743745	0,281	0,148	1	0,706742344	1,414942812	1,898648649
SAP30BP	0,205385164	-0,502817831	0,406	0,227	1	0,705727028	1,416978463	1,788546256
HELZ	0,139465797	-0,503534625	0,031	0,113	1	0,705376479	1,417682655	0,274336283
AFAP1L2	0,397154955	-0,504443339	0,469	0,293	1	0,704932322	1,418575896	1,600682594
CDKN1C	0,773665643	-0,505579211	0,312	0,262	1	0,704377529	1,419693218	1,190839695

Supplementary Table 1

Differentially expressed genes_adjacent-skin-XL_ comparing SC-Keloid vs. (SC-Myel, SC-Nonmyel)								
**gene**	**p_val**	**avg_log2FC**	**pct.1**	**pct.2**	**p_val_adj**	**Foldchange_UP**	**Foldchange_DOWN**	**Ratio_pct1_pct2**
BACH2	0,19837126	-0,505596422	0,031	0,098	1	0,704369125	1,419710155	0,316326531
BAZ2B	0,626135927	-0,506218528	0,219	0,152	1	0,704065459	1,420322482	1,440789474
B4GALT5	0,674055279	-0,506600999	0,094	0,109	1	0,70387883	1,420699071	0,862385321
AHI1	0,454428691	-0,507313266	0,406	0,254	1	0,703531406	1,421400652	1,598425197
MTMR3	0,968729744	-0,507322197	0,125	0,109	1	0,703527052	1,421409451	1,146788991
NHLRC2	0,708633239	-0,507624275	0,094	0,105	1	0,703379759	1,421707103	0,895238095
MEF2A	0,399497935	-0,507906762	0,406	0,25	1	0,703242048	1,421985508	1,624
TMEM248	0,589223109	-0,508246519	0,281	0,191	1	0,703076452	1,422320428	1,471204188
GAL3ST1	0,351280945	-0,508540799	0,062	0,109	1	0,702933054	1,42261058	0,568807339
RFTN2	0,549412664	-0,509037876	0,094	0,121	1	0,702690901	1,423100823	0,776859504
RNF24	0,682167253	-0,509326913	0,156	0,16	1	0,702550135	1,423385962	0,975
BMP7	0,078384714	-0,509579542	0	0,09	1	0,702427123	1,423635232	0
WDFY3	0,611764646	-0,510180374	0,094	0,113	1	0,702134648	1,424228249	0,831858407
DISC1	0,91169441	-0,51059941	0,125	0,105	1	0,701930739	1,424641982	1,19047619
SIAH1	0,807013741	-0,510825841	0,281	0,207	1	0,70182058	1,424865597	1,357487923
NXPE3	0,616164234	-0,511000843	0,219	0,152	1	0,701735453	1,425038447	1,440789474
PTPDC1	0,256488161	-0,511167713	0,094	0,164	1	0,701654291	1,425203284	0,573170732
TSPAN14	0,723273635	-0,512033513	0,125	0,133	1	0,701233336	1,426058843	0,939849624
NKD1	0,122891724	-0,512461801	0	0,07	1	0,701025194	1,426482255	0
TPCN1	0,26138036	-0,512703426	0,031	0,086	1	0,700907795	1,426721185	0,360465116
CEMIP2	0,674055279	-0,512808677	0,094	0,109	1	0,700856662	1,426825275	0,862385321
SELENOW	0,14489921	-0,513326399	0,719	0,582	1	0,7006052	1,427337394	1,235395189
GRB14	0,220935523	-0,51335968	0,031	0,094	1	0,700589038	1,427370321	0,329787234
TPP2	0,761165734	-0,513781244	0,094	0,102	1	0,700384352	1,427787468	0,921568627
ETFDH	0,491272981	-0,513808505	0,094	0,125	1	0,700371117	1,427814448	0,752
RAB11FIP1	0,19837126	-0,514040898	0,031	0,098	1	0,700258309	1,428044462	0,316326531
CD46	0,181261718	-0,514838642	0,656	0,348	1	0,699871205	1,428834323	1,885057471
HLA-DRA	0,530494223	-0,515798861	0,062	0,094	1	0,699405545	1,429785633	0,659574468
HDAC11	0,150480606	-0,516554389	0,031	0,109	1	0,699039368	1,430534597	0,28440367
ZNF791	0,81643733	-0,516851424	0,094	0,098	1	0,698895458	1,430829159	0,959183673

Supplementary Table 1

Differentially expressed genes_adjacent-skin-XL_ comparing SC-Keloid vs. (SC-Myel, SC-Nonmyel)								
**gene**	**p_val**	**avg_log2FC**	**pct.1**	**pct.2**	**p_val_adj**	**Foldchange_UP**	**Foldchange_DOWN**	**Ratio_pct1_pct2**
YME1L1	0,17903325	-0,517507161	0,406	0,227	1	0,698577867	1,43147965	1,788546256
RPS6KA3	0,270831373	-0,517709791	0,062	0,121	1	0,698479757	1,431680719	0,512396694
IGF2BP2	0,835877214	-0,517847892	0,156	0,125	1	0,698412898	1,431817772	1,248
DNAJC6	0,054716441	-0,518457537	0	0,105	1	0,69811783	1,432422948	0
RTF1	0,06208619	-0,518982438	0,5	0,238	1	0,697863877	1,432944207	2,100840336
SYNM	0,309093028	-0,519512505	0,094	0,148	1	0,697607519	1,433470788	0,635135135
SOX6	0,656036246	-0,520698081	0,375	0,262	1	0,697034476	1,434649268	1,43129771
ERBB3	0,294659479	-0,521225682	0,875	0,5	1	0,696779613	1,435174022	1,75
C11orf54	0,078384714	-0,52175605	0	0,09	1	0,696523508	1,435701723	0
SFT2D2	0,248051582	-0,521934131	0,062	0,125	1	0,696437537	1,435878951	0,496
ARGLU1	0,909089059	-0,52310997	0,656	0,457	1	0,695870151	1,437049712	1,435448578
LRRC7	0,093802134	-0,523158157	0	0,082	1	0,695846909	1,437097711	0
EWSR1	0,114855681	-0,523233948	0,562	0,285	1	0,695810354	1,43717321	1,971929825
KANSL1L	0,23950123	-0,523979934	0,062	0,129	1	0,695450659	1,437916533	0,480620155
HNRNPA2B1	0,07943437	-0,524347548	0,938	0,797	1	0,695273473	1,438282977	1,176913425
PRPF39	0,217476686	-0,524698026	0,031	0,094	1	0,695104589	1,438632425	0,329787234
STX16	0,393622993	-0,525264403	0,281	0,176	1	0,694831756	1,439197318	1,596590909
PDS5B	0,714048186	-0,525353187	0,312	0,223	1	0,694788997	1,439285889	1,399103139
PIK3C2A	0,907819917	-0,525449799	0,188	0,152	1	0,694742471	1,439382276	1,236842105
MINDY2	0,961283601	-0,525869348	0,25	0,211	1	0,694540464	1,439800922	1,184834123
RNF103	0,723273635	-0,526655012	0,125	0,133	1	0,694162333	1,440585224	0,939849624
TMEM176A	0,31715205	-0,526914793	0,312	0,352	1	0,694037349	1,440844649	0,886363636
NFATC1	0,389076766	-0,527352501	0,094	0,137	1	0,693826812	1,441281862	0,686131387
SBDS	0,491042257	-0,527518578	0,781	0,586	1	0,693746947	1,441447786	1,332764505
PLXDC2	0,901090659	-0,527658711	0,375	0,285	1	0,693679565	1,441587804	1,315789474
TAF1	0,112290358	-0,52768383	0	0,074	1	0,693667487	1,441612904	0
TAOK1	0,492895148	-0,527867284	0,5	0,312	1	0,693579285	1,441796232	1,602564103
ZNF331	0,249700501	-0,527933782	0,062	0,125	1	0,693547317	1,441862691	0,496
TLN2	0,472561141	-0,528011835	0,188	0,121	1	0,693509796	1,4419407	1,553719008
MYO1E	0,899476288	-0,52841952	0,156	0,129	1	0,693313847	1,44234823	1,209302326

Supplementary Table 1

Differentially expressed genes_adjacent-skin-XL_ comparing SC-Keloid vs. (SC-Myel, SC-Nonmyel)								
**gene**	**p_val**	**avg_log2FC**	**pct.1**	**pct.2**	**p_val_adj**	**Foldchange_UP**	**Foldchange_DOWN**	**Ratio_pct1_pct2**
SNHG12	0,829851616	-0,529682165	0,219	0,168	1	0,692707325	1,443611123	1,303571429
ANKRD36C	0,708251698	-0,530876499	0,156	0,16	1	0,692134105	1,444806711	0,975
SLC9A3R1	0,230125064	-0,53143839	0,062	0,129	1	0,69186459	1,445369534	0,480620155
NKTR	0,421783651	-0,531746314	0,469	0,285	1	0,691716937	1,445678061	1,645614035
KCNQ1OT1	0,600062	-0,531756457	0,125	0,145	1	0,691712073	1,445688226	0,862068966
ZMYND8	0,507127635	-0,532553274	0,156	0,102	1	0,691330138	1,446486916	1,529411765
RASGEF1B	0,345353593	-0,532922153	0,094	0,145	1	0,691153397	1,446856812	0,648275862
EXOC1	0,048449393	-0,532935518	0,281	0,121	1	0,691146994	1,446870216	2,32231405
SVIP	0,088519719	-0,533138986	0,375	0,184	1	0,691049526	1,447074287	2,038043478
ADAM23	0,78856501	-0,533448581	0,188	0,184	1	0,690901246	1,447384855	1,02173913
UBAP1	0,284187836	-0,533669979	0,094	0,152	1	0,690795227	1,44760699	0,618421053
IL34	0,071654957	-0,53371672	0	0,094	1	0,690772847	1,447653891	0
RNF149	0,270831373	-0,533949889	0,062	0,121	1	0,690661213	1,44788788	0,512396694
RLF	0,882939173	-0,534805909	0,219	0,195	1	0,690251532	1,448747236	1,123076923
HES4	0,897119629	-0,535813453	0,25	0,199	1	0,689769646	1,44975936	1,256281407
AKAP7	0,324349291	-0,536482012	0,062	0,113	1	0,689450074	1,450431349	0,548672566
MECP2	0,647779378	-0,537074249	0,375	0,262	1	0,689167107	1,451026883	1,43129771
USP47	0,367937279	-0,537188462	0,344	0,207	1	0,68911255	1,451141761	1,661835749
AGO2	0,947872509	-0,537396366	0,281	0,227	1	0,689013251	1,451350897	1,237885463
LINC00632	0,943740324	-0,537686942	0,219	0,188	1	0,688874489	1,451643246	1,164893617
TRIM56	0,059869253	-0,538285398	0	0,102	1	0,688588791	1,452245539	0
IFNGR1	0,250980166	-0,539107299	0,219	0,277	1	0,688196615	1,453073117	0,790613718
APOE	0,26138036	-0,540723225	0,031	0,086	1	0,687426215	1,454701578	0,360465116
SOD1	0,468461826	-0,541604548	0,844	0,645	1	0,687006404	1,455590507	1,308527132
GPATCH8	0,338687844	-0,541842176	0,281	0,168	1	0,686893256	1,455830279	1,672619048
TMEM132A	0,667452977	-0,542410162	0,125	0,137	1	0,68662288	1,456403549	0,912408759
PARP14	0,841792297	-0,543097163	0,188	0,148	1	0,686295993	1,457097243	1,27027027
KIF1A	0,10262374	-0,543204179	0	0,078	1	0,686245087	1,457205332	0
LUC7L2	0,745097108	-0,543924422	0,375	0,27	1	0,685902576	1,457933	1,388888889
CIR1	0,567878517	-0,544150158	0,281	0,188	1	0,685795262	1,458161138	1,494680851

Supplementary Table 1

Differentially expressed genes_adjacent-skin-XL_ comparing SC-Keloid vs. (SC-Myel, SC-Nonmyel)								
**gene**	**p_val**	**avg_log2FC**	**pct.1**	**pct.2**	**p_val_adj**	**Foldchange_UP**	**Foldchange_DOWN**	**Ratio_pct1_pct2**
USP32	0,803488864	-0,544227525	0,125	0,125	1	0,685758486	1,458239337	1
RBBP6	0,836660523	-0,544590524	0,344	0,254	1	0,685585963	1,458606294	1,354330709
MT-CO1	0,649932755	-0,545046839	0,781	0,719	1	0,685369151	1,459067714	1,086230876
SERINC5	0,930493752	-0,545299108	0,25	0,203	1	0,685249318	1,459322868	1,231527094
PHC1	0,611764646	-0,545460366	0,094	0,113	1	0,685172728	1,459485994	0,831858407
ADAMTS4	0,966010928	-0,546128274	0,156	0,141	1	0,684855595	1,460161832	1,106382979
MYSM1	0,574023156	-0,547108897	0,188	0,129	1	0,684390246	1,461154664	1,457364341
SNRNP27	0,172612804	-0,548242942	0,406	0,215	1	0,683852485	1,462303671	1,888372093
JAM2	0,19837126	-0,548425518	0,031	0,098	1	0,683765948	1,462488741	0,316326531
MEGF6	0,612285359	-0,54864432	0,156	0,109	1	0,683662254	1,462710562	1,431192661
SLC35F1	0,172513176	-0,548769625	0,5	0,262	1	0,683602877	1,462837611	1,908396947
NEDD4L	0,536573979	-0,549668448	0,125	0,148	1	0,683177114	1,463749266	0,844594595
CLK4	0,328400266	-0,549773897	0,094	0,145	1	0,683127182	1,463856258	0,648275862
RIF1	0,691490388	-0,551747041	0,312	0,223	1	0,682193521	1,465859714	1,399103139
WRN	0,730871257	-0,55222762	0,094	0,105	1	0,681966312	1,46634809	0,895238095
HEXIM1	0,09580422	-0,553027975	0,406	0,203	1	0,681588087	1,467161793	2
CTNND1	0,573208462	-0,553260432	0,312	0,297	1	0,681478274	1,467398212	1,050505051
ECHDC2	0,426651606	-0,553825732	0,062	0,102	1	0,681211298	1,467973304	0,607843137
PRNP	0,418711318	-0,554108034	1	0,824	1	0,681078014	1,468260581	1,213592233
NEK7	0,167651587	-0,554355896	0,062	0,145	1	0,680961011	1,468512857	0,427586207
SLC25A29	0,486291268	-0,554829145	0,094	0,125	1	0,680737672	1,468994653	0,752
PLCE1	0,082922884	-0,555489336	0,344	0,164	1	0,680426231	1,469667033	2,097560976
BBX	0,838249419	-0,556658535	0,375	0,277	1	0,679875018	1,470858574	1,353790614
ARMCX4	0,611764646	-0,557528782	0,094	0,113	1	0,679465035	1,471746077	0,831858407
CYBRD1	0,987437888	-0,558050254	0,188	0,16	1	0,679219482	1,472278146	1,175
DUSP16	0,915013818	-0,558155445	0,125	0,105	1	0,67916996	1,472385498	1,19047619
ALDH6A1	0,803488864	-0,558994262	0,125	0,125	1	0,678775189	1,473241827	1
JARID2	0,435228895	-0,559230904	0,094	0,133	1	0,678663861	1,473483498	0,706766917
IST1	0,144091573	-0,559382185	0,375	0,191	1	0,6785927	1,473638016	1,963350785
MAP4K3	0,486291098	-0,560742111	0,094	0,125	1	0,67795334	1,475027765	0,752

Supplementary Table 1

Differentially expressed genes_adjacent-skin-XL_ comparing SC-Keloid vs. (SC-Myel, SC-Nonmyel)								
**gene**	**p_val**	**avg_log2FC**	**pct.1**	**pct.2**	**p_val_adj**	**Foldchange_UP**	**Foldchange_DOWN**	**Ratio_pct1_pct2**
PAQR6	0,883363513	-0,561140385	0,156	0,145	1	0,677766208	1,475435021	1,075862069
INAFM1	0,227058461	-0,561285407	0,062	0,129	1	0,677698082	1,475583341	0,480620155
SREBF1	0,801438557	-0,561439648	0,312	0,238	1	0,677625632	1,475741107	1,31092437
NUP98	0,069020595	-0,562060078	0,031	0,145	1	0,677334282	1,476375885	0,213793103
PGM2L1	0,035646772	-0,562645373	0,531	0,242	1	0,677059546	1,476974965	2,194214876
SMCHD1	0,35121699	-0,562762504	0,344	0,203	1	0,677004579	1,477094885	1,694581281
NDEL1	0,60483255	-0,56381453	0,281	0,191	1	0,676511081	1,478172388	1,471204188
USP34	0,822879814	-0,564246293	0,375	0,277	1	0,676308648	1,478614835	1,353790614
HERC2	0,213440672	-0,564299033	0,375	0,207	1	0,676283925	1,478668889	1,811594203
HNRNPA0	0,644251391	-0,565373495	0,75	0,508	1	0,675780443	1,479770553	1,476377953
NUTM2A-AS1	0,598458377	-0,565534769	0,375	0,246	1	0,675704904	1,479935981	1,524390244
PPP1R16A	0,908273548	-0,56618434	0,188	0,168	1	0,675400737	1,48060247	1,119047619
RMND5A	0,103889562	-0,567001408	0,031	0,125	1	0,675018334	1,481441244	0,248
CNKSR3	0,629352429	-0,567276418	0,344	0,238	1	0,674889672	1,481723667	1,445378151
CPEB4	0,600062	-0,567430653	0,125	0,145	1	0,674817525	1,481882083	0,862068966
BTAF1	0,345353761	-0,567667061	0,094	0,145	1	0,674706955	1,482124932	0,648275862
ERICH1	0,783849734	-0,567744406	0,156	0,121	1	0,674670784	1,482204393	1,289256198
CLDN19	0,078384714	-0,567938558	0	0,09	1	0,674579996	1,482403875	0
RBMS1	0,142817233	-0,56977545	0,594	0,316	1	0,673721643	1,484292528	1,879746835
FAM214A	0,535198384	-0,569878454	0,062	0,09	1	0,673673542	1,484398506	0,688888889
MEX3D	0,950738707	-0,569926681	0,188	0,156	1	0,673651023	1,484448128	1,205128205
ZC3H12C	0,295532937	-0,569962529	0,062	0,117	1	0,673634284	1,484485014	0,52991453
KDSR	0,669580018	-0,570252191	0,469	0,316	1	0,673499047	1,484783096	1,484177215
BDP1	0,404521581	-0,572167702	0,312	0,199	1	0,672605414	1,486755798	1,567839196
SLC37A3	0,449469411	-0,572481299	0,094	0,129	1	0,672459227	1,487079008	0,728682171
PCDH17	0,071654957	-0,57289112	0	0,094	1	0,672268231	1,487501496	0
RDX	0,303707374	-0,573054763	0,844	0,68	1	0,672191981	1,487670232	1,241176471
SPRED1	0,975124463	-0,57439476	0,219	0,188	1	0,671567929	1,489052644	1,164893617
FCGR2B	0,314221703	-0,574438402	0,031	0,078	1	0,671547614	1,489097689	0,397435897
FRMD3	0,54873037	-0,57529378	0,125	0,148	1	0,671149569	1,489980841	0,844594595

Supplementary Table 1

Differentially expressed genes_adjacent-skin-XL_ comparing SC-Keloid vs. (SC-Myel, SC-Nonmyel)								
**gene**	**p_val**	**avg_log2FC**	**pct.1**	**pct.2**	**p_val_adj**	**Foldchange_UP**	**Foldchange_DOWN**	**Ratio_pct1_pct2**
FAR1	0,428423523	-0,575390696	0,094	0,133	1	0,671104485	1,490080937	0,706766917
CYP7B1	0,327050001	-0,576092861	0,094	0,148	1	0,670777936	1,490806341	0,635135135
LSM14A	0,598509491	-0,576576214	0,344	0,23	1	0,670553239	1,491305897	1,495652174
ARID4A	0,733675065	-0,577789633	0,156	0,156	1	0,669989489	1,492560729	1
KMT2A	0,367014331	-0,577980978	0,562	0,332	1	0,669900634	1,492758701	1,692771084
TARDBP	0,576684612	-0,578071989	0,188	0,199	1	0,669858375	1,492852873	0,944723618
H2BC21	0,19837126	-0,578215754	0,031	0,098	1	0,669791627	1,493001644	0,316326531
INTS10	0,511429095	-0,579573211	0,25	0,164	1	0,669161705	1,494407096	1,524390244
VPS37B	0,70704765	-0,580075693	0,25	0,184	1	0,66892868	1,49492768	1,358695652
CDKN1B	0,583331073	-0,580181352	0,188	0,199	1	0,668879692	1,495037168	0,944723618
IFIT2	0,771038875	-0,580533583	0,094	0,102	1	0,668716406	1,495402223	0,921568627
RUBCN	0,189930437	-0,580549439	0,062	0,137	1	0,668709056	1,495418659	0,452554745
MAPK1	0,414206269	-0,581247271	0,344	0,215	1	0,66838568	1,496142168	1,6
ZNF644	0,798298705	-0,582018994	0,344	0,289	1	0,668028244	1,496942695	1,190311419
PTPRA	0,236011314	-0,582278011	0,375	0,211	1	0,667908319	1,497211475	1,777251185
KSR1	0,078384714	-0,583256424	0	0,09	1	0,667455508	1,498227205	0
HIPK3	0,302084682	-0,583938836	0,469	0,273	1	0,667139868	1,498936052	1,717948718
TUBB2B	0,9102215	-0,584124541	0,531	0,43	1	0,667053999	1,499129009	1,234883721
DOCK7	0,088935428	-0,584308377	0,438	0,211	1	0,666969005	1,499320048	2,075829384
PAG1	0,087669454	-0,584331879	0,031	0,133	1	0,66695814	1,499344473	0,233082707
NEU1	0,309264374	-0,584426243	0,281	0,164	1	0,666914516	1,499442546	1,713414634
FYN	0,80210583	-0,584671583	0,312	0,27	1	0,666801113	1,499697557	1,155555556
NAV2	0,848801983	-0,58472823	0,156	0,148	1	0,666774932	1,499756443	1,054054054
PCDH10	0,337841113	-0,584850179	0,125	0,188	1	0,666718572	1,499883222	0,664893617
USP53	0,899977466	-0,584926999	0,469	0,348	1	0,666683072	1,499963089	1,347701149
CFD	0,344592621	-0,587517065	0,031	0,074	1	0,665487252	1,502658386	0,418918919
ATOH8	0,054716322	-0,588400034	0	0,105	1	0,66508008	1,503578336	0
NUDT4	0,014587152	-0,588683509	0,562	0,242	1	0,664949411	1,503873804	2,32231405
ARF6	0,461326013	-0,588837869	0,531	0,324	1	0,664878269	1,504034717	1,638888889
STIP1	0,874412152	-0,58892577	0,438	0,312	1	0,664837761	1,504126359	1,403846154

Supplementary Table 1

Differentially expressed genes_adjacent-skin-XL_ comparing SC-Keloid vs. (SC-Myel, SC-Nonmyel)								
**gene**	**p_val**	**avg_log2FC**	**pct.1**	**pct.2**	**p_val_adj**	**Foldchange_UP**	**Foldchange_DOWN**	**Ratio_pct1_pct2**
LITAF	0,764312379	-0,589833405	0,438	0,352	1	0,664419626	1,50507294	1,244318182
MRGPRF	0,031765882	-0,590077649	0	0,129	1	0,664307152	1,505327765	0
TTC21B	0,276181391	-0,590894074	0,062	0,121	1	0,663931325	1,506179875	0,512396694
NEK1	0,189930437	-0,591067119	0,062	0,137	1	0,663851694	1,506360546	0,452554745
SP100	0,331669544	-0,591690921	0,406	0,234	1	0,663564715	1,507012017	1,735042735
IRF2BPL	0,389151804	-0,591970919	0,281	0,172	1	0,663435943	1,507304526	1,63372093
HTRA1	0,424065451	-0,592165699	0,562	0,328	1	0,663346378	1,507508042	1,713414634
PCF11	0,61519101	-0,594079329	0,281	0,273	1	0,662467081	1,509508969	1,029304029
ARHGEF16	0,045682874	-0,594206032	0	0,113	1	0,662408903	1,509641546	0
BNIP3L	0,558819534	-0,595434137	0,281	0,277	1	0,661845262	1,510927186	1,014440433
MBNL1	0,676253127	-0,596176508	0,625	0,484	1	0,661504782	1,511704868	1,291322314
NAA16	0,065500116	-0,596347307	0	0,098	1	0,661426472	1,511883848	0
PLSCR1	0,346885347	-0,596483148	0,125	0,176	1	0,661364197	1,51202621	0,710227273
BAZ2A	0,227194215	-0,597226691	0,125	0,191	1	0,661023427	1,512805686	0,654450262
SHPRH	0,927665952	-0,600003022	0,156	0,141	1	0,659752574	1,515719741	1,106382979
ATP2B4	0,822100351	-0,600004106	0,312	0,27	1	0,659752078	1,515720881	1,155555556
STON1	0,312631271	-0,600806957	0,094	0,148	1	0,659385032	1,516564604	0,635135135
IFNGR2	0,84684372	-0,601658471	0,688	0,453	1	0,658995962	1,517459982	1,518763797
AC007952.4	0,915013818	-0,601881079	0,125	0,105	1	0,658894286	1,517694145	1,19047619
NOP58	0,911607561	-0,601938177	0,156	0,129	1	0,658868209	1,517754212	1,209302326
ATM	0,118585846	-0,60222515	0,344	0,172	1	0,658737164	1,518056145	2
SESN3	0,642128591	-0,602464987	0,25	0,176	1	0,658627663	1,518308532	1,420454545
MX1	0,177040152	-0,603458725	0,094	0,18	1	0,658174152	1,519354713	0,522222222
COL27A1	0,862675664	-0,60347826	0,281	0,254	1	0,65816524	1,519375287	1,106299213
DYRK1A	0,556088893	-0,604185129	0,125	0,148	1	0,657842842	1,520119908	0,844594595
MAGI1	0,325081758	-0,604293276	0,125	0,18	1	0,657793531	1,520233863	0,694444444
SH3D19	0,699543015	-0,605518328	0,312	0,281	1	0,657235208	1,521525305	1,110320285
THRA	0,768954653	-0,606004941	0,188	0,18	1	0,657013563	1,522038594	1,044444444
ELF2	0,698599064	-0,606255138	0,188	0,137	1	0,656899632	1,522302574	1,372262774
IRF2	0,427947953	-0,606299326	0,125	0,16	1	0,656879512	1,522349201	0,78125

Supplementary Table 1

Differentially expressed genes_adjacent-skin-XL_ comparing SC-Keloid vs. (SC-Myel, SC-Nonmyel)								
**gene**	**p_val**	**avg_log2FC**	**pct.1**	**pct.2**	**p_val_adj**	**Foldchange_UP**	**Foldchange_DOWN**	**Ratio_pct1_pct2**
AKR1C1	0,755689148	-0,606666017	0,062	0,047	1	0,656712574	1,522736186	1,319148936
CD59	0,370177778	-0,607045986	0,969	0,809	1	0,656539635	1,523137289	1,197775031
SPAG9	0,543313878	-0,607067088	0,625	0,375	1	0,656530032	1,523159568	1,666666667
L1CAM	0,37324344	-0,607681497	0,906	0,652	1	0,656250492	1,523808382	1,389570552
KLHL21	0,71468524	-0,608737828	0,125	0,133	1	0,655770165	1,524924513	0,939849624
CADM4	0,200355408	-0,609534304	0,438	0,469	1	0,655408231	1,525766619	0,933901919
TMEM123	0,921931644	-0,610217151	0,375	0,297	1	0,655098091	1,526488955	1,262626263
SEPTIN10	0,741468474	-0,610860974	0,5	0,328	1	0,654805809	1,527170324	1,524390244
CNBP	0,624285586	-0,611778833	0,688	0,477	1	0,654389347	1,528142237	1,442348008
DIP2B	0,775368776	-0,612640385	0,375	0,27	1	0,653998674	1,529055089	1,388888889
WDR74	0,507127635	-0,612771256	0,156	0,102	1	0,65393935	1,5291938	1,529411765
MYO15B	0,243695697	-0,612913253	0,062	0,133	1	0,65387499	1,529344318	0,466165414
ATP9B	0,10388942	-0,612913949	0,031	0,125	1	0,653874674	1,529345056	0,248
ERBB2	0,155166548	-0,613425793	0,438	0,23	1	0,653642732	1,529887738	1,904347826
AUTS2	0,659211148	-0,613680057	0,125	0,137	1	0,653527542	1,530157393	0,912408759
SPHK1	0,838254212	-0,61427677	0,5	0,336	1	0,653257293	1,530790412	1,488095238
EIF1B	0,892198632	-0,6143419	0,625	0,449	1	0,653227802	1,53085952	1,391982183
ZFAS1	0,602714118	-0,614466488	0,969	0,605	1	0,653171393	1,530991728	1,601652893
SLC25A36	0,69409843	-0,614979978	0,219	0,207	1	0,652938955	1,531536742	1,057971014
PHF10	0,938454639	-0,615872687	0,219	0,176	1	0,652535055	1,532484718	1,244318182
KLHL24	0,918500285	-0,616555988	0,188	0,164	1	0,65222607	1,533210717	1,146341463
RB1CC1	0,658940148	-0,618850235	0,312	0,215	1	0,651189691	1,535650847	1,451162791
DHH	0,189930437	-0,618922623	0,062	0,137	1	0,651157018	1,535727901	0,452554745
SH3BP2	0,353309248	-0,6194439	0,344	0,207	1	0,650921783	1,536282892	1,661835749
LZTS2	0,541420238	-0,620113591	0,125	0,148	1	0,650619699	1,536996192	0,844594595
GPCPD1	0,138332372	-0,620155959	0,031	0,113	1	0,650600592	1,53704133	0,274336283
ITGAV	0,438290863	-0,620272615	0,594	0,344	1	0,650547987	1,53716562	1,726744186
GPC4	0,078384714	-0,620537934	0	0,09	1	0,650428359	1,537448339	0
THUMPD3-AS1	0,984779541	-0,621012858	0,188	0,164	1	0,650214278	1,537954538	1,146341463
STOM	0,789751879	-0,621818635	0,312	0,273	1	0,649851221	1,53881376	1,142857143

Supplementary Table 1

Differentially expressed genes_adjacent-skin-XL_ comparing SC-Keloid vs. (SC-Myel, SC-Nonmyel)								
**gene**	**p_val**	**avg_log2FC**	**pct.1**	**pct.2**	**p_val_adj**	**Foldchange_UP**	**Foldchange_DOWN**	**Ratio_pct1_pct2**
HDAC7	0,382784766	-0,622568964	0,094	0,137	1	0,649513328	1,539614287	0,686131387
TMED7	0,154809432	-0,623044315	0,438	0,227	1	0,649299357	1,540121655	1,929515419
RAB40B	0,382784766	-0,62511509	0,094	0,137	1	0,648368052	1,54233386	0,686131387
ZNF638	0,75124878	-0,626299991	0,406	0,281	1	0,647835759	1,543601115	1,444839858
FBXO11	0,521878142	-0,627299262	0,312	0,203	1	0,647387196	1,544670648	1,536945813
CRK	0,81875474	-0,627905672	0,25	0,223	1	0,647115136	1,545320058	1,121076233
DNTTIP2	0,177396377	-0,627994307	0,094	0,172	1	0,647075381	1,545415001	0,546511628
EGR3	0,865226568	-0,628629065	0,25	0,227	1	0,646790742	1,546095104	1,101321586
INSR	0,137206058	-0,629160402	0,031	0,113	1	0,646552577	1,546664626	0,274336283
CLEC14A	0,065500116	-0,630091878	0	0,098	1	0,646135265	1,547663553	0
RORB	0,078496882	-0,630995184	0,031	0,137	1	0,645730831	1,548632886	0,226277372
NIPBL	0,707188953	-0,631019904	0,344	0,242	1	0,645719766	1,548659422	1,421487603
CKB	0,859329737	-0,631054206	0,5	0,383	1	0,645704414	1,548696243	1,305483029
HMGB2	0,566311275	-0,63111964	0,25	0,266	1	0,645675128	1,548766487	0,939849624
LPIN1	0,806521651	-0,631687122	0,125	0,125	1	0,645421203	1,549375812	1
KMT2C	0,671602961	-0,632350026	0,281	0,262	1	0,645124706	1,550087898	1,072519084
GCLM	0,32791849	-0,632563009	0,188	0,109	1	0,645029474	1,550316753	1,724770642
GIT1	0,983758836	-0,632646566	0,25	0,211	1	0,644992117	1,550406545	1,184834123
HLA-DRB1	0,314221703	-0,63323962	0,031	0,078	1	0,644727033	1,551044007	0,397435897
NPEPPS	0,638567716	-0,634045134	0,344	0,23	1	0,644367156	1,551910258	1,495652174
NR3C1	0,957983132	-0,634247681	0,625	0,441	1	0,644276697	1,552128154	1,41723356
RNF169	0,775527841	-0,635700183	0,25	0,188	1	0,643628367	1,55369162	1,329787234
BBS5	0,19837126	-0,63613824	0,031	0,098	1	0,643432967	1,554163451	0,316326531
AK5	0,041730427	-0,636607201	0	0,117	1	0,643223847	1,554668728	0
NMT1	0,541417165	-0,636715326	0,281	0,191	1	0,643175641	1,554785249	1,471204188
MAG	0,08574604	-0,636964282	0	0,086	1	0,643064662	1,555053572	0
KAT2B	0,355687038	-0,637666258	0,062	0,109	1	0,642751841	1,555810402	0,568807339
MBD2	0,429427742	-0,63842768	0,375	0,23	1	0,642412701	1,55663174	1,630434783
ATP8A1	0,050000177	-0,639263575	0	0,109	1	0,642040595	1,557533912	0
LACTB	0,251919686	-0,639943849	0,094	0,156	1	0,641737925	1,558268509	0,602564103

Supplementary Table 1

Differentially expressed genes_adjacent-skin-XL_ comparing SC-Keloid vs. (SC-Myel, SC-Nonmyel)								
**gene**	**p_val**	**avg_log2FC**	**pct.1**	**pct.2**	**p_val_adj**	**Foldchange_UP**	**Foldchange_DOWN**	**Ratio_pct1_pct2**
MLXIP	0,405500634	-0,640278854	0,375	0,23	1	0,641588926	1,558630393	1,630434783
PPP2CB	0,828245535	-0,640562494	0,656	0,449	1	0,6414628	1,558936856	1,461024499
DCAF8	0,82197282	-0,641265112	0,188	0,176	1	0,641150472	1,559696271	1,068181818
CELF1	0,915192996	-0,643126872	0,312	0,254	1	0,640323618	1,561710317	1,228346457
RAB8B	0,444667251	-0,643687085	0,344	0,211	1	0,640075022	1,562316863	1,630331754
ICAM1	0,238418073	-0,646909102	0,031	0,09	1	0,638647117	1,565809934	0,344444444
ANKRD28	0,427947953	-0,64730192	0,125	0,16	1	0,638473249	1,566236333	0,78125
HIPK2	0,413936113	-0,647737815	0,125	0,168	1	0,63828037	1,566709626	0,744047619
FOXN2	0,384875137	-0,647905873	0,094	0,137	1	0,638206022	1,566892141	0,686131387
AKAP9	0,814480554	-0,647962802	0,406	0,293	1	0,638180839	1,566953971	1,385665529
CHD1	0,666879428	-0,648571809	0,312	0,289	1	0,6379115	1,567615571	1,079584775
COBL	0,75872397	-0,648678237	0,438	0,301	1	0,637864442	1,567731219	1,455149502
CAHM	0,2285881	-0,649804366	0,062	0,129	1	0,637366737	1,568955426	0,480620155
SRGN	0,150480606	-0,651122972	0,031	0,109	1	0,636784457	1,570390088	0,28440367
ZSWIM8	0,453137599	-0,651835338	0,062	0,098	1	0,636470107	1,571165698	0,632653061
KLF6	0,31355846	-0,65189151	0,906	0,773	1	0,636445326	1,571226873	1,172056921
ANTXR2	0,307333706	-0,653236786	0,094	0,148	1	0,635852134	1,572692685	0,635135135
MRPS24	0,072634989	-0,653441702	0,656	0,328	1	0,635761826	1,572916081	2
LINC00472	0,145730596	-0,653462839	0,062	0,148	1	0,635752511	1,572939127	0,418918919
LENG8	0,989447626	-0,653846414	0,219	0,184	1	0,635583504	1,573357385	1,190217391
ARMCX1	0,074893791	-0,653953485	0,062	0,18	1	0,635536335	1,573474158	0,344444444
VEZF1	0,372432709	-0,653976849	0,5	0,289	1	0,635526043	1,573499641	1,730103806
MDM4	0,998932805	-0,65399363	0,438	0,324	1	0,635518651	1,573517943	1,351851852
EIF5	0,496471528	-0,654965407	0,531	0,441	1	0,635090719	1,574578197	1,204081633
HNRNPC	0,322341005	-0,656095244	0,844	0,594	1	0,634593547	1,5758118	1,420875421
TTYH2	0,086206764	-0,656369403	0,031	0,133	1	0,634472965	1,576111285	0,233082707
APLP2	0,54519988	-0,657275921	0,688	0,5	1	0,634074419	1,577101946	1,376
BPTF	0,877091832	-0,657684195	0,469	0,324	1	0,633895005	1,57754832	1,447530864
TMPRSS5	0,045682874	-0,657965484	0	0,113	1	0,633771424	1,577855931	0
EGR2	0,790895329	-0,658880671	0,094	0,102	1	0,633369512	1,578857177	0,921568627

Supplementary Table 1

Differentially expressed genes_adjacent-skin-XL_ comparing SC-Keloid vs. (SC-Myel, SC-Nonmyel)								
**gene**	**p_val**	**avg_log2FC**	**pct.1**	**pct.2**	**p_val_adj**	**Foldchange_UP**	**Foldchange_DOWN**	**Ratio_pct1_pct2**
CASKIN2	0,248051582	-0,658896719	0,062	0,125	1	0,633362467	1,578874739	0,496
SDC2	0,564559422	-0,658898343	0,156	0,172	1	0,633361754	1,578876516	0,906976744
HEATR5A	0,512526612	-0,660116303	0,156	0,176	1	0,632827279	1,580210007	0,886363636
NFAT5	0,495477494	-0,661826145	0,188	0,207	1	0,632077715	1,582083938	0,90821256
HSPA9	0,914697862	-0,662354063	0,594	0,391	1	0,631846464	1,582662969	1,519181586
MTUS1	0,673955066	-0,663589018	0,219	0,211	1	0,631305831	1,584018317	1,037914692
CCDC126	0,861530298	-0,664164166	0,188	0,148	1	0,631054203	1,584649931	1,27027027
UPF2	0,728981998	-0,664651336	0,312	0,273	1	0,630841145	1,585185126	1,142857143
CORO1C	0,683925113	-0,664956322	0,375	0,258	1	0,630707799	1,585520271	1,453488372
AMOTL2	0,356657717	-0,665457381	0,094	0,141	1	0,630488787	1,586071029	0,666666667
FOXJ3	0,779766547	-0,665799352	0,219	0,199	1	0,630339357	1,58644703	1,100502513
FGF2	0,506319067	-0,667138344	0,406	0,277	1	0,629754598	1,587920125	1,465703971
PPP2R2B	0,116282551	-0,667524993	0,062	0,16	1	0,629585844	1,588345752	0,3875
ZBTB7A	0,863911019	-0,668117625	0,406	0,309	1	0,629327275	1,588998348	1,313915858
MKNK2	0,227823267	-0,668157166	0,094	0,164	1	0,629310026	1,5890419	0,573170732
SRGAP1	0,254938178	-0,66815773	0,094	0,156	1	0,62930978	1,589042521	0,602564103
FOXN3	0,277642838	-0,668539038	0,094	0,152	1	0,629143474	1,589462564	0,618421053
FRYL	0,643920142	-0,669188782	0,219	0,219	1	0,628860192	1,590178569	1
ITSN1	0,158518263	-0,669316484	0,062	0,145	1	0,62880453	1,590319333	0,427586207
EMP1	0,121368453	-0,669614165	0,875	0,461	1	0,628674798	1,590647508	1,898047722
CFLAR	0,142451599	-0,669750051	0,094	0,184	1	0,628615587	1,590797335	0,510869565
INO80D	0,106159347	-0,670422104	0,062	0,168	1	0,628322825	1,591538552	0,369047619
RASD1	0,970129378	-0,671208699	0,156	0,137	1	0,627980341	1,592406537	1,138686131
HNRNPH2	0,561120066	-0,671941916	0,281	0,27	1	0,627661265	1,593216047	1,040740741
RBM43	0,454181181	-0,67239534	0,094	0,129	1	0,627464029	1,593716857	0,728682171
PROSER2	0,021993198	-0,672932255	0	0,145	1	0,627230555	1,594310087	0
S100A2	0,236273078	-0,673158726	0,156	0,227	1	0,627132101	1,594560378	0,68722467
DOK6	0,024121703	-0,67347707	0	0,141	1	0,626993734	1,594912271	0
RAB21	0,791451263	-0,673986559	0,375	0,305	1	0,62677235	1,595475615	1,229508197
SMIM5	0,323879795	-0,674113092	0,344	0,199	1	0,62671738	1,595615554	1,728643216

Supplementary Table 1

Differentially expressed genes_adjacent-skin-XL_ comparing SC-Keloid vs. (SC-Myel, SC-Nonmyel)								
**gene**	**p_val**	**avg_log2FC**	**pct.1**	**pct.2**	**p_val_adj**	**Foldchange_UP**	**Foldchange_DOWN**	**Ratio_pct1_pct2**
UACA	0,64821776	-0,674156111	0,25	0,242	1	0,626698693	1,595663134	1,033057851
SORBS3	0,347958339	-0,675175179	0,156	0,199	1	0,626256172	1,596790651	0,783919598
IGSF11	0,021993198	-0,675330931	0	0,145	1	0,626188565	1,596963049	0
H3-3B	0,001377561	-0,678473223	0,969	0,957	1	0,624826166	1,600445138	1,012539185
GAS6	0,173563398	-0,679422758	0,062	0,141	1	0,624415062	1,601498845	0,439716312
RBM6	0,119127359	-0,679425676	0,5	0,25	1	0,624413799	1,601502085	2
CTSF	0,792249622	-0,679956056	0,281	0,242	1	0,624184287	1,602090955	1,161157025
SELENOM	0,142710778	-0,680589626	0,812	0,645	1	0,623910232	1,602794679	1,258914729
CTTNBP2NL	0,360215275	-0,681070558	0,125	0,168	1	0,623702282	1,603329071	0,744047619
ATP1A1	0,859703745	-0,681728919	0,5	0,344	1	0,623417726	1,604060903	1,453488372
TOB1	0,529583804	-0,683643445	0,375	0,34	1	0,622590969	1,606190982	1,102941176
MSL1	0,722930988	-0,684164033	0,281	0,203	1	0,622366351	1,606770672	1,384236453
LRCH3	0,353488187	-0,686265181	0,125	0,172	1	0,621460594	1,609112485	0,726744186
ATP13A3	0,515145183	-0,686666131	0,188	0,203	1	0,621287903	1,609559747	0,926108374
KCNK12	0,071654957	-0,687005229	0	0,094	1	0,621141889	1,609938111	0
TRIM8	0,739656961	-0,687570278	0,375	0,258	1	0,62089866	1,610568786	1,453488372
HNRNPDL	0,045348966	-0,689481507	0,906	0,73	1	0,62007666	1,612703822	1,24109589
VWA1	0,157721486	-0,689515302	0,812	0,707	1	0,620062136	1,612741599	1,148514851
UBE2R2	0,856979723	-0,68978983	0,562	0,371	1	0,619944156	1,613048514	1,514824798
TP53BP2	0,298637683	-0,690635031	0,094	0,148	1	0,619581069	1,613993793	0,635135135
ZBTB38	0,316848011	-0,691161345	0,469	0,266	1	0,619355079	1,614582707	1,763157895
NPTX2	0,764452551	-0,692309592	0,094	0,102	1	0,618862328	1,615868272	0,921568627
GRIK2	0,840275242	-0,692490398	0,156	0,148	1	0,618784773	1,616070794	1,054054054
ASPH	0,794573296	-0,692579354	0,531	0,348	1	0,618746621	1,616170443	1,525862069
SFPQ	0,294095328	-0,692755358	0,688	0,559	1	0,61867114	1,616367622	1,230769231
PPP1R9A	0,384590169	-0,694817551	0,125	0,168	1	0,617787442	1,618679715	0,744047619
ZBTB10	0,464267129	-0,69517441	0,156	0,184	1	0,617634647	1,619080154	0,847826087
MYLIP	0,086206887	-0,695439067	0,031	0,133	1	0,617521355	1,619377196	0,233082707
SAP30	0,622996508	-0,695583773	0,281	0,258	1	0,617459419	1,619539632	1,089147287
ZBTB43	0,13585239	-0,69626128	0,062	0,152	1	0,617169521	1,620300365	0,407894737

Supplementary Table 1

Differentially expressed genes_adjacent-skin-XL_ comparing SC-Keloid vs. (SC-Myel, SC-Nonmyel)								
**gene**	**p_val**	**avg_log2FC**	**pct.1**	**pct.2**	**p_val_adj**	**Foldchange_UP**	**Foldchange_DOWN**	**Ratio_pct1_pct2**
ARID4B	0,66771677	-0,696497658	0,406	0,336	1	0,617068409	1,620565866	1,208333333
RPS6KA5	0,057628491	-0,696867074	0,031	0,152	1	0,616910423	1,62098088	0,203947368
GNAI1	0,013784176	-0,697124659	0	0,164	1	0,616800287	1,621270323	0
GADD45G	0,436655983	-0,697355872	0,188	0,117	1	0,616701443	1,621530176	1,606837607
MAP2K3	0,201268578	-0,698193304	0,125	0,195	1	0,616343574	1,622471689	0,641025641
NCOA1	0,493774078	-0,698460307	0,094	0,125	1	0,616229517	1,622771991	0,752
LAMC1	0,812864729	-0,698606698	0,531	0,391	1	0,616166991	1,622936663	1,358056266
HIVEP2	0,464246717	-0,699162279	0,125	0,156	1	0,615929751	1,623561776	0,801282051
RBM39	0,074220177	-0,699173846	0,781	0,664	1	0,615924813	1,623574792	1,176204819
PGRMC1	0,828309537	-0,699369922	0,719	0,484	1	0,615841108	1,623795467	1,48553719
RNF213	0,520207072	-0,70107434	0,406	0,258	1	0,615113976	1,625714973	1,573643411
TNRC18	0,611764646	-0,702935	0,094	0,113	1	0,614321168	1,627813028	0,831858407
DAAM2	0,21437948	-0,70379198	0,094	0,164	1	0,613956361	1,628780258	0,573170732
CDC42SE1	0,924457555	-0,704127246	0,469	0,34	1	0,613813701	1,629158811	1,379411765
MYO9A	0,414999889	-0,704936126	0,094	0,133	1	0,613469649	1,630072493	0,706766917
DEDD2	0,231069397	-0,705409241	0,094	0,16	1	0,613268502	1,630607144	0,5875
YY1	0,863516207	-0,706375767	0,625	0,422	1	0,612857783	1,631699927	1,481042654
EFNA1	0,054716441	-0,706565848	0	0,105	1	0,612777042	1,631914924	0
SERPINB2	0,314221703	-0,706692827	0,031	0,078	1	0,612723111	1,632058564	0,397435897
TRIM2	0,104758015	-0,708667913	0,031	0,125	1	0,611884851	1,634294423	0,248
IP6K2	0,069302474	-0,708851041	0,406	0,191	1	0,611807187	1,634501886	2,12565445
PELI1	0,58497922	-0,710024716	0,25	0,246	1	0,611309666	1,635832142	1,016260163
INF2	0,754571239	-0,710078442	0,375	0,309	1	0,611286901	1,635893061	1,213592233
LRRFIP2	0,567778851	-0,711692142	0,469	0,301	1	0,61060354	1,637723883	1,558139535
RNASE4	0,112311725	-0,712173324	0,094	0,191	1	0,610399919	1,638270204	0,492146597
HSPB1	0,418452272	-0,712188668	0,969	0,758	1	0,610393427	1,638287628	1,278364116
PTBP2	0,396332035	-0,712287728	0,188	0,223	1	0,610351517	1,638400122	0,843049327
OAT	0,400891593	-0,713109602	0,344	0,332	1	0,610003911	1,639333752	1,036144578
DICER1	0,872732285	-0,713870742	0,219	0,195	1	0,609682169	1,640198863	1,123076923
SAMD4B	0,381980579	-0,714166779	0,281	0,289	1	0,609557077	1,640535461	0,972318339

Supplementary Table 1

Differentially expressed genes_adjacent-skin-XL_ comparing SC-Keloid vs. (SC-Myel, SC-Nonmyel)								
gene	p_val	avg_log2FC	pct.1	pct.2	p_val_adj	Foldchange_UP	Foldchange_DOWN	Ratio_pct1_pct2
IRF2BP2	0,799941487	-0,714374707	0,5	0,34	1	0,609469231	1,64077192	1,470588235
FGF7	0,028989337	-0,714474518	0	0,133	1	0,609427067	1,640885439	0
MALAT1	3,06731E-05	-0,715658378	1	0,996	0,647232879	0,608927183	1,642232485	1,004016064
PLCB1	0,828430167	-0,716472777	0,25	0,188	1	0,608583541	1,643159783	1,329787234
DEPDC7	0,132006288	-0,718113963	0,062	0,152	1	0,60789162	1,645030078	0,407894737
EIF4A2	0,312636926	-0,718874341	0,875	0,594	1	0,607571313	1,645897326	1,473063973
SOX10	0,465907978	-0,720438295	0,812	0,457	1	0,606913032	1,647682529	1,776805252
ARHGEF26	0,910986121	-0,72101166	0,312	0,258	1	0,606671877	1,648337492	1,209302326
PTX3	0,009120578	-0,721587665	0,281	0,098	1	0,606429708	1,648995732	2,867346939
SLC22A17	0,960889512	-0,722067331	0,562	0,398	1	0,606228116	1,64954408	1,412060302
KLF13	0,787534153	-0,724227738	0,344	0,289	1	0,605320981	1,65201609	1,190311419
DIO2	0,065500116	-0,724660181	0	0,098	1	0,605139565	1,652511351	0
SERTAD1	0,767122349	-0,726879488	0,531	0,398	1	0,604209391	1,655055376	1,334170854
TMEM245	0,353796011	-0,727897276	0,156	0,195	1	0,603783285	1,656223391	0,8
PHF13	0,434202776	-0,729279923	0,125	0,16	1	0,60320491	1,65781144	0,78125
GPM6B	0,000330269	-0,729874982	1	0,926	1	0,602956161	1,658495367	1,079913607
MIDEAS	0,535149581	-0,730388075	0,25	0,254	1	0,602741759	1,659085314	0,984251969
BAIAP2	0,520630523	-0,731268152	0,188	0,199	1	0,602374185	1,660097703	0,944723618
PFKFB3	0,12036165	-0,73204648	0,062	0,156	1	0,602049294	1,66099356	0,397435897
NUFIP2	0,867579695	-0,733335643	0,438	0,309	1	0,601511555	1,662478454	1,417475728
GNAQ	0,963076802	-0,734248146	0,25	0,199	1	0,60113122	1,663530303	1,256281407
FOXD3	0,920892644	-0,734739517	0,688	0,492	1	0,600926514	1,664096985	1,398373984
STK32A	0,030461633	-0,735017267	0,031	0,176	1	0,600810834	1,66441739	0,176136364
SRSF5	0,965010947	-0,735171219	0,781	0,508	1	0,600746724	1,664595012	1,537401575
HNRNPH3	0,414440417	-0,735297116	0,594	0,473	1	0,600694302	1,664740279	1,255813953
SPEN	0,383902296	-0,736466222	0,219	0,242	1	0,600207719	1,66608987	0,904958678
IPO7	0,998818743	-0,736595828	0,312	0,242	1	0,600153801	1,666239551	1,289256198
PTGDS	0,19837126	-0,738444586	0,031	0,098	1	0,59938522	1,668376141	0,316326531
CNTN1	0,041730427	-0,739328293	0	0,117	1	0,599018185	1,6693984	0
UBE2D3	0,503682383	-0,740228312	0,656	0,473	1	0,598644607	1,670440172	1,386892178

Supplementary Table 1

Differentially expressed genes_adjacent-skin-XL_ comparing SC-Keloid vs. (SC-Myel, SC-Nonmyel)								
**gene**	**p_val**	**avg_log2FC**	**pct.1**	**pct.2**	**p_val_adj**	**Foldchange_UP**	**Foldchange_DOWN**	**Ratio_pct1_pct2**
ADAMTS9-AS2	0,038111671	-0,742139877	0	0,121	1	0,597851931	1,672654964	0
ZMAT1	0,684759296	-0,742950327	0,156	0,16	1	0,597516175	1,673594861	0,975
ATN1	0,109401783	-0,74384166	0,25	0,344	1	0,597147129	1,674629169	0,726744186
SGK1	0,749244773	-0,743991424	0,125	0,133	1	0,597085143	1,67480302	0,939849624
PLAAT3	0,863015651	-0,7444272	0,375	0,297	1	0,596904816	1,675308982	1,262626263
ZNF462	0,921952026	-0,744555914	0,188	0,152	1	0,596851564	1,675458455	1,236842105
PPP1R18	0,964067721	-0,745613446	0,406	0,305	1	0,596414217	1,67668706	1,331147541
CAMK2D	0,664432664	-0,745714666	0,344	0,23	1	0,596372374	1,6768047	1,495652174
HSP90AB1	0,119655413	-0,745793838	0,969	0,832	1	0,596339647	1,676896723	1,164663462
GFRA3	0,897038612	-0,745913366	0,75	0,48	1	0,596290242	1,67703566	1,5625
HLA-E	0,402826691	-0,746785888	0,719	0,531	1	0,595929724	1,678050214	1,354048964
PACS2	0,491361021	-0,747849678	0,125	0,152	1	0,595490469	1,679288003	0,822368421
UGCG	0,412460131	-0,748906237	0,344	0,215	1	0,595054521	1,680518281	1,6
PPTC7	0,092299385	-0,750119735	0,062	0,168	1	0,594554211	1,681932415	0,369047619
DUSP6	0,523236119	-0,750447049	0,125	0,156	1	0,594419336	1,682314049	0,801282051
GJC3	0,078384714	-0,750997386	0	0,09	1	0,594192629	1,682955915	0
TAB2	0,200105361	-0,752379498	0,125	0,195	1	0,593623661	1,684568971	0,641025641
ZMYM5	0,297710641	-0,753238449	0,156	0,207	1	0,593270335	1,685572227	0,753623188
PLPP3	0,428736122	-0,753855192	0,188	0,211	1	0,59301677	1,686292953	0,890995261
MKLN1	0,876663892	-0,754791241	0,188	0,172	1	0,592632134	1,687387408	1,093023256
PHLDB1	0,73708174	-0,755505632	0,469	0,32	1	0,592338748	1,688223173	1,465625
XBP1	0,461846537	-0,756621939	0,438	0,281	1	0,591880594	1,689529966	1,558718861
FNBP4	0,738086752	-0,75671973	0,281	0,25	1	0,591840476	1,689644492	1,124
GBP1	0,144763586	-0,757370593	0,438	0,227	1	0,591573531	1,690406937	1,929515419
BGN	0,137205524	-0,757491973	0,125	0,23	1	0,591523761	1,690549164	0,543478261
MAN2A2	0,07228439	-0,758286037	0,156	0,289	1	0,591198274	1,691479904	0,539792388
PPP4R2	0,937976735	-0,759300876	0,406	0,305	1	0,590782552	1,692670165	1,331147541
PREX1	0,054716441	-0,759729349	0	0,105	1	0,590607119	1,693172954	0
NR2F1	0,731305749	-0,760686839	0,219	0,16	1	0,590215274	1,694297054	1,36875
FGFBP2	0,054716441	-0,761031404	0	0,105	1	0,590074327	1,694701759	0

Supplementary Table 1

Differentially expressed genes_adjacent-skin-XL_ comparing SC-Keloid vs. (SC-Myel, SC-Nonmyel)								
**gene**	**p_val**	**avg_log2FC**	**pct.1**	**pct.2**	**p_val_adj**	**Foldchange_UP**	**Foldchange_DOWN**	**Ratio_pct1_pct2**
GOLGA8A	0,529345739	-0,764237638	0,125	0,148	1	0,588764407	1,698472239	0,844594595
ZRANB2	0,747475225	-0,764443571	0,375	0,305	1	0,588680371	1,698714699	1,229508197
KMT2E	0,831376578	-0,764663642	0,625	0,457	1	0,58859058	1,698973844	1,36761488
TMEM132B	0,038111838	-0,768787417	0	0,121	1	0,586910565	1,703837109	0
UBR3	0,078496882	-0,769741454	0,031	0,137	1	0,586522576	1,704964208	0,226277372
PRDM16	0,109682674	-0,771412611	0,062	0,16	1	0,585843567	1,706940311	0,3875
ARL13B	0,622936899	-0,771461171	0,219	0,215	1	0,585823848	1,706997767	1,018604651
NORAD	0,733068525	-0,771637315	0,562	0,41	1	0,585752327	1,707206193	1,370731707
GAB1	0,609762878	-0,771643207	0,25	0,246	1	0,585749934	1,707213166	1,016260163
ADGRL3	0,016635187	-0,772160576	0	0,156	1	0,585539915	1,707825504	0
ATP1B2	0,095797588	-0,773229897	0,062	0,168	1	0,585106075	1,709091808	0,369047619
SRSF11	0,099550522	-0,77512838	0,656	0,562	1	0,584336624	1,71134233	1,167259786
OGA	0,60416098	-0,775286529	0,344	0,305	1	0,584272572	1,711529939	1,127868852
TSPAN15	0,637426397	-0,775635118	0,438	0,355	1	0,584131415	1,711943535	1,233802817
NPNT	0,026447767	-0,776966053	0	0,137	1	0,583592783	1,713523589	0
ARHGEF7	0,764260824	-0,778267793	0,25	0,223	1	0,583066446	1,715070395	1,121076233
SETD5	0,35496866	-0,779286367	0,25	0,273	1	0,582654934	1,7162817	0,915750916
RSRC2	0,709651769	-0,779373944	0,781	0,445	1	0,582619566	1,716385887	1,75505618
PNRC2	0,562656319	-0,780134142	0,25	0,242	1	0,582312647	1,717290539	1,033057851
SMC5	0,800582307	-0,782624987	0,375	0,297	1	0,581308139	1,720258041	1,262626263
SRSF3	0,06174927	-0,782787617	0,781	0,676	1	0,581242614	1,720451971	1,155325444
PLEKHA4	0,585766432	-0,783589661	0,719	0,426	1	0,580919571	1,721408695	1,687793427
RIOK3	0,409820795	-0,786503157	0,25	0,262	1	0,579747598	1,724888561	0,954198473
VGLL4	0,995246059	-0,787365868	0,75	0,496	1	0,579401021	1,725920327	1,512096774
CITED2	0,557828792	-0,787733523	0,219	0,148	1	0,579253386	1,726360216	1,47972973
TXN	0,637446779	-0,790432079	0,688	0,578	1	0,578170907	1,729592388	1,190311419
CCN1	0,755531983	-0,790498168	0,844	0,613	1	0,578144422	1,729671621	1,376835237
RYBP	0,540963733	-0,790532099	0,344	0,32	1	0,578130825	1,729712303	1,075
SRRM1	0,285112879	-0,790613073	0,656	0,508	1	0,578098377	1,729809388	1,291338583
CDKN2AIP	0,189930272	-0,790781995	0,062	0,137	1	0,578030692	1,730011941	0,452554745

Supplementary Table 1

Differentially expressed genes_adjacent-skin-XL_ comparing SC-Keloid vs. (SC-Myel, SC-Nonmyel)								
**gene**	**p_val**	**avg_log2FC**	**pct.1**	**pct.2**	**p_val_adj**	**Foldchange_UP**	**Foldchange_DOWN**	**Ratio_pct1_pct2**
DCUN1D3	0,305722739	-0,791408264	0,156	0,203	1	0,577779826	1,730763096	0,768472906
MYOT	0,61252846	-0,79176852	0,438	0,301	1	0,577635566	1,73119534	1,455149502
HNRNPU	0,219334289	-0,792570328	0,781	0,605	1	0,577314622	1,732157755	1,290909091
PPP2R2A	0,26710439	-0,796517608	0,281	0,309	1	0,575737222	1,736903507	0,909385113
CRYL1	0,11399702	-0,797767517	0,125	0,223	1	0,575238636	1,738408961	0,560538117
ELMO1	0,034798894	-0,79834708	0	0,125	1	0,575007596	1,73910746	0
NFKB2	0,099894919	-0,798636471	0,062	0,164	1	0,574892267	1,739456344	0,37804878
CNOT4	0,708770049	-0,799509815	0,344	0,234	1	0,574544357	1,740509653	1,47008547
CAMSAP2	0,228167587	-0,800927637	0,188	0,242	1	0,573979996	1,742220996	0,776859504
RXRG	0,086802846	-0,803470986	0,125	0,234	1	0,572969009	1,745295092	0,534188034
GEM	0,836432406	-0,805342248	0,188	0,18	1	0,572226316	1,747560314	1,044444444
MLLT6	0,018098341	-0,805846913	0,031	0,203	1	0,572026182	1,74817173	0,15270936
PER3	0,986081827	-0,806107319	0,25	0,203	1	0,571922941	1,748487302	1,231527094
S1PR3	0,78964859	-0,806872476	0,219	0,199	1	0,571619693	1,749414887	1,100502513
APCDD1	0,324707387	-0,809622019	0,094	0,145	1	0,570531315	1,752752168	0,648275862
LHPP	0,068343209	-0,809979368	0,062	0,18	1	0,570390015	1,75318637	0,344444444
RALGDS	0,725641486	-0,811144679	0,156	0,156	1	0,569929479	1,754603047	1
KIF21A	0,06206671	-0,811201494	0,062	0,184	1	0,569907035	1,754672147	0,336956522
ARHGAP15	0,053282509	-0,812766637	0,062	0,203	1	0,569289092	1,756576779	0,305418719
KAT6A	0,164165532	-0,814013683	0,156	0,242	1	0,568797219	1,758095796	0,644628099
MAFG	0,575103826	-0,81436969	0,438	0,281	1	0,568656877	1,758529687	1,558718861
TAMALIN	0,553211263	-0,814539524	0,219	0,219	1	0,568589939	1,758736712	1
MAP3K4	0,090929152	-0,817446937	0,062	0,168	1	0,567445233	1,762284607	0,369047619
HPS5	0,075210866	-0,817937764	0,062	0,176	1	0,567252213	1,762884265	0,352272727
MBOAT2	0,293579025	-0,819693493	0,219	0,258	1	0,566562298	1,765030965	0,848837209
NKAIN3	0,01254006	-0,819820349	0	0,168	1	0,566512483	1,76518617	0
DDX5	0,03640077	-0,821682086	1	0,793	1	0,565781894	1,767465538	1,261034048
FAM181B	0,324707219	-0,822716265	0,094	0,145	1	0,565376465	1,768732979	0,648275862
ASH1L	0,82986017	-0,824137786	0,344	0,277	1	0,564819661	1,770476612	1,241877256
PHACTR4	0,870309538	-0,824215552	0,344	0,273	1	0,564789216	1,770572049	1,26007326

Supplementary Table 1

Differentially expressed genes_adjacent-skin-XL_ comparing SC-Keloid vs. (SC-Myel, SC-Nonmyel)								
**gene**	**p_val**	**avg_log2FC**	**pct.1**	**pct.2**	**p_val_adj**	**Foldchange_UP**	**Foldchange_DOWN**	**Ratio_pct1_pct2**
FNDC3B	0,996777288	-0,825773059	0,438	0,316	1	0,564179809	1,772484559	1,386075949
KCTD11	0,096946527	-0,828675597	0,094	0,199	1	0,563045885	1,776054185	0,472361809
NCOA7	0,090929152	-0,828946522	0,062	0,168	1	0,56294016	1,776387743	0,369047619
FAM126A	0,879492191	-0,836432596	0,375	0,289	1	0,560026656	1,785629289	1,297577855
SCML1	0,163572054	-0,83763844	0,125	0,207	1	0,559558766	1,78712239	0,603864734
MAFK	0,05293496	-0,837719659	0,094	0,23	1	0,559527266	1,787223002	0,408695652
MT-CO3	0,035466145	-0,838894846	0,844	0,82	1	0,559071673	1,788679427	1,029268293
ARHGAP19	0,024121756	-0,840443404	0	0,141	1	0,558471899	1,790600389	0
SMG1	0,707944663	-0,841628962	0,25	0,227	1	0,558013155	1,792072448	1,101321586
SON	0,007338614	-0,841732343	0,938	0,754	1	0,55797317	1,792200869	1,24403183
SECISBP2L	0,951390724	-0,842528899	0,594	0,414	1	0,557665181	1,793190671	1,434782609
ZSWIM6	0,159148124	-0,843936745	0,188	0,258	1	0,557121252	1,7949414	0,728682171
CHORDC1	0,531153812	-0,845908041	0,25	0,25	1	0,556360522	1,797395682	1
DRP2	0,078384714	-0,848819576	0	0,09	1	0,555238851	1,801026709	0
SNHG9	0,572382114	-0,849264806	0,375	0,246	1	0,555067525	1,801582608	1,524390244
ZBTB16	0,045682874	-0,849290444	0	0,113	1	0,555057661	1,801614625	0
NUP153	0,052320766	-0,849661428	0,062	0,191	1	0,554914949	1,802077963	0,32460733
DNAJB6	0,931529644	-0,84971339	0,656	0,434	1	0,554894962	1,80214287	1,511520737
GOLGA4	0,811215417	-0,850741624	0,469	0,316	1	0,55449962	1,803427747	1,484177215
IVNS1ABP	0,06213286	-0,851109865	0,125	0,258	1	0,554358104	1,803888122	0,484496124
CLK1	0,222595783	-0,852677657	0,562	0,496	1	0,553756005	1,805849492	1,133064516
VASN	0,150052517	-0,853323052	0,094	0,18	1	0,553508335	1,806657526	0,522222222
MARCHF5	0,520630631	-0,855937383	0,188	0,199	1	0,552506222	1,809934368	0,944723618
JUND	0,002323049	-0,856443444	1	0,922	1	0,552312451	1,810569358	1,084598698
SCRN1	0,281853519	-0,856851345	0,125	0,18	1	0,552156315	1,811081343	0,694444444
ABLIM1	0,358606521	-0,857303649	0,281	0,301	1	0,551983234	1,81164923	0,933554817
MTCO2P12	0,077128877	-0,859298936	0,812	0,777	1	0,551220353	1,814156524	1,045045045
NRBP2	0,715557304	-0,861395174	0,344	0,297	1	0,550420011	1,816794412	1,158249158
GFRA1	0,636959502	-0,862316997	0,312	0,273	1	0,550068427	1,81795564	1,142857143
NID2	0,020369679	-0,862818049	0,031	0,191	1	0,54987742	1,818587131	0,162303665

Supplementary Table 1

Differentially expressed genes_adjacent-skin-XL_ comparing SC-Keloid vs. (SC-Myel, SC-Nonmyel)								
**gene**	**p_val**	**avg_log2FC**	**pct.1**	**pct.2**	**p_val_adj**	**Foldchange_UP**	**Foldchange_DOWN**	**Ratio_pct1_pct2**
PAXBP1	0,107695716	-0,862872318	0,219	0,312	1	0,549856736	1,818655541	0,701923077
ADAMTS8	0,01514567	-0,864095441	0	0,16	1	0,549390763	1,820198059	0
BAG3	0,186594105	-0,865313606	0,188	0,266	1	0,548927071	1,821735624	0,706766917
THSD7A	0,110135177	-0,866214645	0,156	0,254	1	0,548584344	1,82287375	0,614173228
GJC1	0,8258646	-0,8665337	0,188	0,172	1	0,548463037	1,823276926	1,093023256
AASS	0,337299246	-0,867424409	0,156	0,203	1	0,548124524	1,824402951	0,768472906
MT-ND1	0,005740828	-0,868101962	0,844	0,82	1	0,547867162	1,825259972	1,029268293
TOM1L2	0,065525443	-0,871120959	0,094	0,215	1	0,546721888	1,829083527	0,437209302
DPYSL2	0,690228765	-0,871327665	0,375	0,328	1	0,546643561	1,829345613	1,143292683
HNRNPH1	0,031020966	-0,872653356	0,875	0,723	1	0,546141481	1,83102737	1,210235131
B4GALNT1	0,21834609	-0,875895603	0,125	0,191	1	0,544915486	1,835146964	0,654450262
ZMIZ1	0,244740217	-0,876316363	0,125	0,188	1	0,544756585	1,83568226	0,664893617
EDIL3	0,016384074	-0,877047155	0,031	0,203	1	0,544480711	1,836612354	0,15270936
SLITRK5	0,038111838	-0,878646695	0	0,121	1	0,54387737	1,838649766	0
GJA1	0,0301917	-0,880297571	0,031	0,176	1	0,543255368	1,840754937	0,176136364
AZGP1	0,137205883	-0,880596837	0,031	0,113	1	0,543142689	1,841136815	0,274336283
WTAP	0,867699789	-0,881020012	0,531	0,359	1	0,542983396	1,84167694	1,479108635
MIDN	0,267041892	-0,88277714	0,219	0,281	1	0,542322473	1,843921374	0,779359431
MX2	0,029129106	-0,883355961	0,031	0,184	1	0,542104932	1,84466132	0,168478261
TXNRD1	0,724070658	-0,884432305	0,156	0,16	1	0,541700638	1,846038069	0,975
BCAS1	0,071654957	-0,88601864	0	0,094	1	0,541105331	1,848069022	0
HSPE1	0,085349783	-0,886122652	0,75	0,629	1	0,541066321	1,848202264	1,192368839
SLC3A2	0,797175401	-0,888084896	0,625	0,434	1	0,540330904	1,850717759	1,440092166
CHD2	0,839210831	-0,888511098	0,375	0,297	1	0,540171303	1,851264581	1,262626263
MIR99AHG	0,048905975	-0,889928468	0,031	0,156	1	0,539640874	1,853084241	0,198717949
TAFA5	0,010365668	-0,892244857	0	0,176	1	0,538775123	1,856061941	0
CLMN	0,07764459	-0,893045156	0,094	0,215	1	0,538476334	1,85709183	0,437209302
C1R	0,606308094	-0,893640113	0,312	0,277	1	0,538254316	1,857857839	1,126353791
SEMA4B	0,048906052	-0,893738159	0,031	0,156	1	0,538217737	1,857984104	0,198717949
ETNK1	0,170586877	-0,895771767	0,25	0,309	1	0,537459605	1,860604946	0,809061489

Supplementary Table 1

Differentially expressed genes_adjacent-skin-XL_ comparing SC-Keloid vs. (SC-Myel, SC-Nonmyel)								
**gene**	**p_val**	**avg_log2FC**	**pct.1**	**pct.2**	**p_val_adj**	**Foldchange_UP**	**Foldchange_DOWN**	**Ratio_pct1_pct2**
HSPD1	0,764102597	-0,895849695	0,688	0,473	1	0,537430575	1,86070545	1,454545455
ID4	0,220694814	-0,896269413	0,25	0,301	1	0,537274245	1,861246857	0,830564784
DNAJA1	0,142311415	-0,897186956	0,906	0,652	1	0,536932651	1,862430973	1,389570552
SLC25A25	0,162901689	-0,897725073	0,156	0,23	1	0,536732416	1,863125779	0,67826087
TLE1	0,696643418	-0,900236064	0,594	0,418	1	0,535799053	1,866371347	1,421052632
BRD4	0,313957844	-0,901322553	0,344	0,348	1	0,535395696	1,867777434	0,988505747
LPAR1	0,124515681	-0,904227541	0,188	0,27	1	0,534318716	1,871542151	0,696296296
CEP85L	0,400977845	-0,904907111	0,188	0,215	1	0,534067089	1,872423934	0,874418605
RERE	0,154166227	-0,905653409	0,219	0,281	1	0,533790891	1,873392779	0,779359431
BCLAF1	0,309930687	-0,907246767	0,375	0,352	1	0,533201681	1,875462955	1,065340909
ARHGAP5	0,242222017	-0,907575883	0,344	0,355	1	0,533080057	1,875890846	0,969014085
FBLN2	0,204440837	-0,907833386	0,219	0,285	1	0,532984918	1,876225699	0,768421053
PIK3R1	0,407099813	-0,909390564	0,562	0,457	1	0,532409949	1,878251903	1,2297593
UBR5	0,442734228	-0,909655902	0,219	0,23	1	0,532312038	1,87859738	0,952173913
EPB41L4A-AS1	0,374462559	-0,91039405	0,312	0,301	1	0,532039753	1,879558801	1,03654485
MXI1	0,102252102	-0,912394872	0,094	0,195	1	0,531302398	1,882167302	0,482051282
HTATIP2	0,062957523	-0,913334549	0,125	0,246	1	0,530956455	1,883393621	0,508130081
CD44	0,533400398	-0,914920062	0,75	0,438	1	0,530373257	1,885464598	1,712328767
N4BP2L1	0,028814689	-0,916277998	0,062	0,215	1	0,529874279	1,887240126	0,288372093
KCTD12	0,576894575	-0,918451629	0,375	0,32	1	0,529076547	1,890085672	1,171875
PPM1K	0,00941818	-0,923732529	0	0,18	1	0,527143433	1,897016897	0
ERF	0,245298456	-0,924588188	0,406	0,391	1	0,526830878	1,898142347	1,038363171
LIMS2	0,562237259	-0,929937203	0,156	0,172	1	0,524881188	1,905193066	0,906976744
SOX8	0,045413282	-0,933212528	0,094	0,242	1	0,523690911	1,909523306	0,388429752
STARD9	0,038357172	-0,933784861	0,062	0,211	1	0,523483199	1,910280985	0,293838863
SERPINB9	0,038111671	-0,935392227	0	0,121	1	0,522900289	1,912410493	0
GATM	0,408165217	-0,935441332	0,406	0,242	1	0,522882492	1,912475587	1,67768595
PLEKHA1	0,963412599	-0,936614842	0,281	0,223	1	0,522457344	1,914031857	1,260089686
SNHG8	0,632765497	-0,939362768	0,594	0,445	1	0,521463158	1,91768102	1,334831461
ANK2	0,936472656	-0,940607031	0,281	0,234	1	0,521013612	1,919335652	1,200854701

Supplementary Table 1

Differentially expressed genes_adjacent-skin-XL_ comparing SC-Keloid vs. (SC-Myel, SC-Nonmyel)								
**gene**	**p_val**	**avg_log2FC**	**pct.1**	**pct.2**	**p_val_adj**	**Foldchange_UP**	**Foldchange_DOWN**	**Ratio_pct1_pct2**
GRIK3	0,016635187	-0,940711794	0	0,156	1	0,520975779	1,919475032	0
MAP4K5	0,61398632	-0,941568246	0,438	0,344	1	0,520666595	1,920614861	1,273255814
FADS3	0,141874315	-0,944433876	0,531	0,484	1	0,51963342	1,924433576	1,097107438
FOXO3	0,178072433	-0,944567851	0,188	0,254	1	0,519585166	1,924612297	0,74015748
PRKCA	0,542212256	-0,945340482	0,406	0,34	1	0,519306979	1,925643292	1,194117647
TTYH1	0,059703471	-0,945374593	0,188	0,305	1	0,5192947	1,925688823	0,616393443
NFKBIZ	0,735053311	-0,945764023	0,375	0,305	1	0,519154545	1,926208698	1,229508197
SYNE1	0,770265309	-0,948835192	0,312	0,266	1	0,518050559	1,930313525	1,172932331
RGS11	0,09743441	-0,95021914	0,156	0,254	1	0,517553841	1,932166125	0,614173228
DDX3X	0,223783767	-0,951247665	0,781	0,602	1	0,517184999	1,933544093	1,297342193
PTPN12	0,238436492	-0,951336671	0,375	0,363	1	0,517153093	1,933663386	1,033057851
SFRP1	0,132959812	-0,951661851	0,062	0,152	1	0,517036541	1,934099278	0,407894737
PUM1	0,168691752	-0,954753053	0,156	0,234	1	0,515929894	1,938247834	0,666666667
TRIP10	0,044250233	-0,956013706	0,156	0,297	1	0,515479262	1,93994225	0,525252525
OTUD7B	0,363446048	-0,956888033	0,25	0,262	1	0,515166957	1,941118284	0,954198473
LUC7L3	0,617147851	-0,958201067	0,594	0,43	1	0,514698304	1,94288575	1,381395349
KLF10	0,243091552	-0,959596463	0,312	0,332	1	0,51420072	1,944765848	0,939759036
VAPA	0,092311085	-0,960805395	0,688	0,59	1	0,513770017	1,946396182	1,166101695
ASXL1	0,076945647	-0,961531566	0,281	0,359	1	0,51351148	1,947376133	0,782729805
DCD	0,454084099	-0,961759465	0,156	0,184	1	0,513430368	1,94768378	0,847826087
TCF7L2	0,059560843	-0,966681081	0,094	0,223	1	0,511681834	1,954339463	0,421524664
SYNE3	0,039766082	-0,96735423	0,094	0,242	1	0,511443143	1,955251554	0,388429752
EMP2	0,117368323	-0,970250043	0,656	0,555	1	0,510417591	1,959180125	1,181981982
INTS6	0,435006104	-0,970533097	0,375	0,348	1	0,510317458	1,959564549	1,077586207
HBP1	0,552798543	-0,976961991	0,406	0,34	1	0,508048456	1,968316186	1,194117647
LRRC4C	0,056128986	-0,978862885	0,062	0,191	1	0,507379493	1,970911347	0,32460733
SBSPON	0,031103374	-0,980804897	0,062	0,211	1	0,506696969	1,973566179	0,293838863
QKI	0,000761518	-0,981209971	0,875	0,781	1	0,50655472	1,974120386	1,120358515
FTH1	0,055805446	-0,982930643	1	0,973	1	0,505950923	1,976476282	1,027749229
GPBP1	0,158601771	-0,98431859	0,75	0,594	1	0,505464407	1,978378669	1,262626263

Supplementary Table 1

Differentially expressed genes_adjacent-skin-XL_ comparing SC-Keloid vs. (SC-Myel, SC-Nonmyel)								
**gene**	**p_val**	**avg_log2FC**	**pct.1**	**pct.2**	**p_val_adj**	**Foldchange_UP**	**Foldchange_DOWN**	**Ratio_pct1_pct2**
VPS13D	0,090929152	-0,986725848	0,062	0,168	1	0,5046217	1,981682516	0,369047619
ARHGAP24	0,159966334	-0,98835152	0,312	0,359	1	0,504053397	1,983916794	0,86908078
IER2	0,714654675	-0,990343107	0,719	0,512	1	0,50335805	1,986657409	1,404296875
DLC1	0,143224237	-0,990745315	0,469	0,469	1	0,503217739	1,987211346	1
PCDH9	0,096496694	-0,993572037	0,125	0,242	1	0,502232733	1,991108773	0,516528926
ARL5B	0,14495731	-0,994768154	0,125	0,211	1	0,501816511	1,992760256	0,592417062
SOX2	0,891269757	-0,995097849	0,5	0,352	1	0,501701846	1,993215708	1,420454545
ISG20	0,011403548	-0,99512006	0	0,172	1	0,501694122	1,993246395	0
ZNF536	0,212510708	-0,996378709	0,188	0,246	1	0,50125662	1,99498612	0,764227642
GADD45A	0,366304996	-0,99654937	0,406	0,254	1	0,501197329	1,995222127	1,598425197
C1S	0,036660749	-0,99833483	0,031	0,168	1	0,500577437	1,997692916	0,18452381
RHOBTB3	0,285941193	-1,002547159	0,156	0,207	1	0,499118001	2,003534231	0,753623188
C1orf198	0,134977634	-1,005250662	0,562	0,512	1	0,498183567	2,007292224	1,09765625
ARRDC3	0,380496983	-1,006758152	0,219	0,238	1	0,49766328	2,009390766	0,920168067
ANKRD33B	0,02004569	-1,007961717	0	0,148	1	0,497248279	2,011067795	0
ABCA8	0,8414712	-1,007982159	0,281	0,234	1	0,497241233	2,011096291	1,200854701
PPP1R15B	0,098189816	-1,009676039	0,25	0,32	1	0,496657761	2,013458921	0,78125
SERTAD2	0,164353323	-1,012212815	0,312	0,344	1	0,495785226	2,01700242	0,906976744
SLC44A1	0,546832311	-1,013601003	0,375	0,328	1	0,495308401	2,018944152	1,143292683
RAPGEF2	0,025604089	-1,015340595	0,062	0,219	1	0,494711522	2,021380049	0,283105023
FOXO1	0,027571015	-1,019276323	0,188	0,344	1	0,493363769	2,02690198	0,546511628
F3	0,027165086	-1,021435583	0,062	0,227	1	0,492625911	2,029937886	0,273127753
CTSC	0,086604386	-1,022195281	0,312	0,387	1	0,492366571	2,031007097	0,80620155
CD74	0,248051582	-1,022495197	0,062	0,125	1	0,492264226	2,031429359	0,496
MAL	0,006578194	-1,023737823	0,625	0,656	1	0,49184041	2,033179829	0,952743902
MT-ND4L	0,446962539	-1,027545372	0,656	0,477	1	0,49054406	2,038552868	1,375262055
BRD2	0,155623357	-1,030808362	0,594	0,512	1	0,489435834	2,043168747	1,16015625
NRP2	0,201371388	-1,031591511	0,219	0,27	1	0,489170222	2,044278156	0,811111111
NCMAP	0,078384714	-1,032904756	0	0,09	1	0,488725147	2,046139853	0
CMTM5	0,102731791	-1,034945603	0,281	0,34	1	0,488034282	2,049036385	0,826470588

Supplementary Table 1

Differentially expressed genes_adjacent-skin-XL_ comparing SC-Keloid vs. (SC-Myel, SC-Nonmyel)								
**gene**	**p_val**	**avg_log2FC**	**pct.1**	**pct.2**	**p_val_adj**	**Foldchange_UP**	**Foldchange_DOWN**	**Ratio_pct1_pct2**
NNMT	0,051387074	-1,035659866	0,031	0,164	1	0,487792721	2,050051093	0,18902439
SRSF7	0,495427991	-1,036374176	0,625	0,469	1	0,487551264	2,051066369	1,332622601
ID2	0,3148935	-1,03638128	0,188	0,238	1	0,487548863	2,051076469	0,789915966
FAM102A	0,013608429	-1,039137284	0,062	0,242	1	0,486618379	2,054998419	0,256198347
CYLD	0,279143582	-1,040045434	0,281	0,297	1	0,486312158	2,056292411	0,946127946
DDX17	0,140775248	-1,046570034	0,906	0,637	1	0,484117773	2,065613069	1,422291994
IER5	0,072729672	-1,046601132	0,469	0,238	1	0,484107338	2,065657596	1,970588235
TGFBR3	0,00406764	-1,048238319	0,031	0,262	1	0,483558279	2,068003058	0,118320611
TRA2B	0,047453112	-1,052194555	0,594	0,535	1	0,482234056	2,07368183	1,110280374
KCTD9	0,093861404	-1,058286007	0,156	0,254	1	0,480202225	2,082455992	0,614173228
IFITM2	0,15311344	-1,062029733	0,625	0,527	1	0,478957738	2,087866885	1,185958254
SLC5A3	0,022046343	-1,062604376	0,062	0,23	1	0,478767001	2,088698672	0,269565217
BCL3	0,087194206	-1,063780986	0,125	0,23	1	0,478376695	2,090402835	0,543478261
FGFR1	0,057032565	-1,063922309	0,094	0,223	1	0,478329837	2,090607615	0,421524664
MLIP	0,028989337	-1,065686702	0	0,133	1	0,477745205	2,093165959	0
ZBTB44	0,056948738	-1,070464336	0,156	0,273	1	0,476165719	2,100109185	0,571428571
LONRF1	0,063837771	-1,071885851	0,094	0,219	1	0,475696775	2,102179483	0,429223744
ARC	0,78981908	-1,072871237	0,219	0,164	1	0,475371977	2,103615798	1,335365854
TRA2A	0,130099628	-1,080589986	0,469	0,441	1	0,472835419	2,114900787	1,063492063
FOSL2	0,17416129	-1,081266539	0,25	0,305	1	0,472613734	2,115892805	0,819672131
PNISR	0,006151966	-1,08613737	0,75	0,676	1	0,471020785	2,123048559	1,109467456
ENTPD2	0,05661993	-1,087601625	0,094	0,223	1	0,470542968	2,12520443	0,421524664
CYTH3	0,049701594	-1,094258858	0,125	0,254	1	0,468376677	2,135033723	0,492125984
PIM3	0,194372735	-1,100072699	0,25	0,305	1	0,466492988	2,143654944	0,819672131
PPP1R10	0,180063683	-1,100740546	0,312	0,336	1	0,466277091	2,144647506	0,928571429
NEAT1	0,000105444	-1,103530347	0,906	0,898	1	0,465376302	2,148798714	1,008908686
SRRM2	0,043994388	-1,105846523	0,656	0,578	1	0,464629763	2,152251276	1,134948097
DDIT3	0,052490891	-1,106985316	0,25	0,355	1	0,464263152	2,153950828	0,704225352
WAC	0,145418195	-1,107180743	0,344	0,363	1	0,464200267	2,154242622	0,947658402
FMNL2	0,001802033	-1,10810015	0,062	0,324	1	0,463904533	2,155615925	0,191358025

Supplementary Table 1

Differentially expressed genes_adjacent-skin-XL_ comparing SC-Keloid vs. (SC-Myel, SC-Nonmyel)								
**gene**	**p_val**	**avg_log2FC**	**pct.1**	**pct.2**	**p_val_adj**	**Foldchange_UP**	**Foldchange_DOWN**	**Ratio_pct1_pct2**
SKI	0,20422078	-1,109882183	0,312	0,332	1	0,463331867	2,15828021	0,939759036
SEMA4C	0,109682674	-1,110977736	0,062	0,16	1	0,462980156	2,159919787	0,3875
SAMD4A	0,083372458	-1,111045109	0,188	0,285	1	0,462958535	2,160020657	0,659649123
TEAD1	0,080248942	-1,111958987	0,312	0,383	1	0,462665366	2,161389359	0,81462141
RTN4	0,153276844	-1,115067617	0,688	0,543	1	0,461669517	2,166051608	1,267034991
TNRC6B	0,276339621	-1,116010457	0,5	0,426	1	0,461367902	2,167467644	1,17370892
FNIP2	0,267786202	-1,116379257	0,219	0,262	1	0,461249976	2,168021791	0,835877863
HSPG2	0,023485799	-1,117591325	0,906	0,738	1	0,460862624	2,169844001	1,227642276
ACKR3	0,020369679	-1,118546279	0,031	0,191	1	0,460557669	2,171280748	0,162303665
IRS2	0,012451411	-1,1218215	0,062	0,246	1	0,459513292	2,17621561	0,25203252
LGI4	0,07565944	-1,12297205	0,438	0,473	1	0,459146976	2,177951835	0,926004228
SIPA1L2	0,124344223	-1,127071993	0,5	0,473	1	0,457843995	2,184150081	1,057082452
TLE4	0,367743962	-1,127464561	0,281	0,297	1	0,45771943	2,184744486	0,946127946
C2orf88	0,015254011	-1,134285278	0,062	0,238	1	0,455560549	2,195097891	0,260504202
PRDM16-DT	0,00389118	-1,13573219	0,031	0,254	1	0,455103886	2,197300509	0,122047244
ITGB4	0,07574701	-1,13670966	0,5	0,488	1	0,454795644	2,198789751	1,024590164
KDM6B	0,091319012	-1,139317901	0,188	0,285	1	0,453974164	2,202768528	0,659649123
ARID5A	0,344662082	-1,14040283	0,281	0,293	1	0,453632897	2,204425666	0,959044369
PRELP	0,014940017	-1,140691216	0,031	0,211	1	0,453542227	2,204866361	0,146919431
IFITM3	6,36237E-05	-1,143757265	1	0,895	1	0,452579372	2,209557179	1,117318436
RBMS3	0,013639666	-1,144812181	0,031	0,207	1	0,452248562	2,211173423	0,149758454
SIK3	0,032087103	-1,147561441	0,125	0,27	1	0,451387558	2,215391146	0,462962963
LATS2	0,103995267	-1,148442933	0,188	0,277	1	0,451111843	2,216745172	0,678700361
SCN9A	0,096620596	-1,148931904	0,312	0,367	1	0,450958974	2,217496617	0,85013624
OLFM1	0,018871851	-1,151117954	0,094	0,27	1	0,450276174	2,220859236	0,348148148
LAMA4	0,095104602	-1,153862809	0,531	0,5	1	0,449420298	2,22508864	1,062
ARAP2	0,01723139	-1,158942509	0,062	0,234	1	0,447840681	2,23293694	0,264957265
N4BP2L2	0,032281593	-1,16731779	0,531	0,543	1	0,445248362	2,245937514	0,977900552
PLSCR4	0,019595462	-1,174044394	0,156	0,316	1	0,443177212	2,256433708	0,493670886
MYC	0,116778581	-1,178137555	0,125	0,219	1	0,441921628	2,262844668	0,570776256

Supplementary Table 1

Differentially expressed genes_adjacent-skin-XL_ comparing SC-Keloid vs. (SC-Myel, SC-Nonmyel)								
**gene**	**p_val**	**avg_log2FC**	**pct.1**	**pct.2**	**p_val_adj**	**Foldchange_UP**	**Foldchange_DOWN**	**Ratio_pct1_pct2**
IGFBP7	0,052065573	-1,181206889	0,75	0,742	1	0,440982439	2,267663995	1,010781671
ITPRID2	0,032617871	-1,186040599	0,375	0,445	1	0,439507412	2,275274484	0,842696629
FIGN	0,247448622	-1,186113165	0,406	0,379	1	0,439485306	2,27538893	1,071240106
ZNF503	0,023291609	-1,187262756	0,188	0,348	1	0,439135248	2,277202764	0,540229885
SORBS1	0,022850965	-1,19242944	0,219	0,367	1	0,437565397	2,285372669	0,596730245
PHLDA1	0,530059383	-1,194340596	0,625	0,48	1	0,436986133	2,288402135	1,302083333
FSTL1	0,040955199	-1,195926994	0,438	0,477	1	0,436505884	2,290919863	0,918238994
SFRP5	0,024121703	-1,19824523	0	0,141	1	0,435805035	2,294604053	0
OLFML2A	0,701078703	-1,198366988	0,719	0,449	1	0,435768256	2,294797718	1,601336303
MRPS6	0,039020606	-1,204985923	0,125	0,262	1	0,433773576	2,305350199	0,477099237
ITPR1	0,008167187	-1,209893608	0,031	0,227	1	0,432300495	2,313205774	0,136563877
NLGN4X	0,03387328	-1,210057222	0,156	0,305	1	0,432251471	2,313468126	0,51147541
CHADL	0,044630182	-1,210938075	0,219	0,328	1	0,431987635	2,314881071	0,667682927
STAT3	0,050088233	-1,211235076	0,406	0,441	1	0,431898713	2,315357673	0,920634921
AC009041.2	0,011487017	-1,212215971	0,062	0,25	1	0,431605163	2,31693243	0,248
PMP2	0,154061402	-1,214355133	0,406	0,441	1	0,430965673	2,32037042	0,920634921
MCL1	0,174101789	-1,215667914	0,594	0,48	1	0,430573694	2,322482802	1,2375
ARHGEF10	0,006744192	-1,215766412	0,188	0,391	1	0,430544298	2,322641373	0,480818414
SMTN	0,029264113	-1,220783077	0,156	0,301	1	0,429049772	2,330731921	0,518272425
CNTN2	0,003891944	-1,221690884	0	0,215	1	0,42877988	2,332198982	0
SEMA5A	0,03161618	-1,235083747	0,062	0,211	1	0,424817838	2,353950116	0,293838863
CREBRF	0,026925757	-1,239773293	0,188	0,324	1	0,423439191	2,361614187	0,580246914
GFRA2	0,155566833	-1,241150347	0,219	0,285	1	0,42303521	2,363869426	0,768421053
DNAJB4	0,189382021	-1,241517187	0,375	0,383	1	0,422927657	2,364470573	0,979112272
NEDD9	0,26704199	-1,251783001	0,25	0,277	1	0,419928904	2,381355486	0,902527076
ZBTB20	0,049601691	-1,252737111	0,594	0,527	1	0,419651281	2,38293089	1,127134725
PTEN	0,028740164	-1,254499412	0,375	0,449	1	0,419138975	2,3858435	0,83518931
HSPA2	0,078442739	-1,25798428	0,094	0,207	1	0,418127756	2,391613534	0,45410628
DDIT4	0,575177068	-1,261618946	0,344	0,316	1	0,417075668	2,397646462	1,088607595
NFE2L2	0,053452746	-1,262818711	0,781	0,586	1	0,416728967	2,399641206	1,332764505

Supplementary Table 1

Differentially expressed genes_adjacent-skin-XL_ comparing SC-Keloid vs. (SC-Myel, SC-Nonmyel)								
**gene**	**p_val**	**avg_log2FC**	**pct.1**	**pct.2**	**p_val_adj**	**Foldchange_UP**	**Foldchange_DOWN**	**Ratio_pct1_pct2**
SLIT2	0,001808419	-1,26312705	0,062	0,312	1	0,416639911	2,400154123	0,198717949
IER5L	0,881276048	-1,264300909	0,594	0,43	1	0,416301047	2,402107821	1,381395349
NR2F2	0,168957526	-1,265022582	0,406	0,418	1	0,416092854	2,403309717	0,971291866
B4GALNT4	0,006627143	-1,266002865	0,031	0,234	1	0,415810223	2,404943274	0,132478632
SPTBN1	7,6514E-06	-1,266905703	0,812	0,844	0,161452129	0,415550091	2,406448757	0,962085308
MEGF9	0,009125872	-1,268348499	0,281	0,43	1	0,41513472	2,408856578	0,653488372
ART3	0,000521148	-1,269305391	0	0,289	1	0,414859466	2,410454824	0
MED13L	0,059760021	-1,271281558	0,219	0,32	1	0,414291591	2,413758866	0,684375
GLDN	0,038111838	-1,277667763	0	0,121	1	0,412461749	2,424467246	0
ADGRB3	0,00233989	-1,27964665	0	0,234	1	0,41189638	2,427795071	0
LAMB1	0,151754339	-1,280968331	0,656	0,523	1	0,411519206	2,430020241	1,254302103
CTNNAL1	0,013141583	-1,281829675	0,25	0,414	1	0,411273586	2,43147149	0,603864734
MT1X	0,117391427	-1,292645948	0,594	0,297	1	0,408201688	2,449769391	2
KLF4	0,411365317	-1,295299521	0,594	0,457	1	0,407451566	2,454279439	1,299781182
NR4A1	0,099814455	-1,299962306	0,875	0,672	1	0,406136809	2,462224494	1,302083333
FOS	0,062805775	-1,302091138	0,938	0,82	1	0,405537958	2,465860419	1,143902439
NDRG1	0,006894276	-1,304635019	0,344	0,484	1	0,40482351	2,470212267	0,710743802
ZEB2	0,000190608	-1,30491606	0,906	0,797	1	0,404744657	2,470693518	1,136762861
SPATA13	0,004074523	-1,305154021	0,062	0,285	1	0,404677903	2,471101074	0,21754386
SERPING1	0,001607793	-1,306982411	0,031	0,285	1	0,404165362	2,474234792	0,10877193
TSPYL2	0,037320683	-1,313955585	0,094	0,238	1	0,402216568	2,486222795	0,394957983
COL3A1	0,40492022	-1,31716832	0,594	0,48	1	0,401321869	2,49176553	1,2375
GPR155	0,014158204	-1,321101176	0,344	0,469	1	0,400229336	2,498567469	0,73347548
PRIMA1	0,006627129	-1,322066328	0,031	0,234	1	0,399961676	2,500239551	0,132478632
SEMA3G	0,001387247	-1,323905225	0	0,254	1	0,399452199	2,503428453	0
BHLHE40	0,038478302	-1,33625316	0,406	0,457	1	0,396047902	2,524947097	0,888402626
RDH10	0,006553384	-1,34469807	0,062	0,27	1	0,393736381	2,539770382	0,22962963
LPL	0,003180406	-1,348440459	0	0,223	1	0,392716343	2,546367165	0
TIPARP	0,067184942	-1,349931289	0,219	0,324	1	0,392310733	2,548999851	0,675925926
PNRC1	0,001040003	-1,350580056	0,719	0,68	1	0,392134354	2,550146371	1,057352941

Supplementary Table 1

Differentially expressed genes_adjacent-skin-XL_ comparing SC-Keloid vs. (SC-Myel, SC-Nonmyel)								
**gene**	**p_val**	**avg_log2FC**	**pct.1**	**pct.2**	**p_val_adj**	**Foldchange_UP**	**Foldchange_DOWN**	**Ratio_pct1_pct2**
ETS1	0,143852793	-1,354460401	0,562	0,48	1	0,391081067	2,557014605	1,170833333
TMOD2	0,022143304	-1,358307161	0,281	0,398	1	0,390039689	2,563841652	0,706030151
CADM3	0,005697043	-1,363502494	0,188	0,402	1	0,388637631	2,573091024	0,467661692
NIBAN1	0,009452591	-1,365385148	0,156	0,355	1	0,388130807	2,576450987	0,43943662
SORCS1	0,009361352	-1,368022607	0,188	0,379	1	0,387421894	2,581165428	0,496042216
IFRD1	0,013620574	-1,376982811	0,312	0,445	1	0,385023174	2,59724626	0,701123596
GAS2L3	0,0092796	-1,377421201	0,125	0,316	1	0,384906196	2,598035603	0,39556962
SLC38A2	0,003055286	-1,377620205	0,375	0,547	1	0,384853106	2,598393998	0,685557587
HSPH1	0,845159753	-1,377733377	0,594	0,402	1	0,384822917	2,598597837	1,47761194
IGSF1	0,002110019	-1,381828568	0	0,238	1	0,383732119	2,605984617	0
SNHG15	0,08051557	-1,384312628	0,281	0,352	1	0,383071971	2,610475516	0,798295455
CSRNP1	0,033492656	-1,393769119	0,406	0,461	1	0,380569245	2,627642707	0,880694143
CLIC4	0,000104241	-1,396858265	0,906	0,77	1	0,379755229	2,63327513	1,176623377
GBP2	0,057556704	-1,399907448	0,156	0,277	1	0,378953452	2,638846528	0,563176895
FTL	0,006179769	-1,400975581	1	0,883	1	0,378672988	2,640800983	1,132502831
TSC22D2	0,014197513	-1,404046352	0,25	0,395	1	0,377867841	2,646427905	0,632911392
GLUL	0,028188895	-1,411896583	0,188	0,34	1	0,375817308	2,660867336	0,552941176
ABCA1	0,003082907	-1,414825929	0,094	0,332	1	0,375054998	2,66627563	0,28313253
RC3H1	0,016577215	-1,423093774	0,125	0,293	1	0,372911767	2,68159948	0,42662116
HMOX1	0,431765035	-1,425767617	0,062	0,102	1	0,372221265	2,686574075	0,607843137
ATP1A2	0,000888905	-1,428226816	0,031	0,312	1	0,371587322	2,691157479	0,099358974
TAF1D	0,098619939	-1,436709593	0,531	0,48	1	0,369408869	2,707027592	1,10625
AHNAK	4,63416E-06	-1,438159544	0,969	0,832	0,097785484	0,369037788	2,709749603	1,164663462
KRT5	0,003929166	-1,439396388	0,031	0,254	1	0,368721542	2,712073709	0,122047244
IL6ST	0,01282952	-1,441824678	0,5	0,531	1	0,368101446	2,716642412	0,941619586
SYNE2	0,032916555	-1,445331948	0,281	0,383	1	0,367207658	2,723254752	0,733681462
EPB41L2	0,010436886	-1,447561858	0,281	0,422	1	0,36664052	2,72746722	0,665876777
PRRG4	0,000805979	-1,451344223	0,031	0,309	1	0,365680544	2,7346273	0,100323625
SNCA	0,001108266	-1,452606805	0,156	0,426	1	0,365360657	2,737021571	0,366197183
BPGM	0,550310174	-1,45288584	0,406	0,332	1	0,365289999	2,737550996	1,222891566

Supplementary Table 1

Differentially expressed genes_adjacent-skin-XL_ comparing SC-Keloid vs. (SC-Myel, SC-Nonmyel)								
**gene**	**p_val**	**avg_log2FC**	**pct.1**	**pct.2**	**p_val_adj**	**Foldchange_UP**	**Foldchange_DOWN**	**Ratio_pct1_pct2**
NR4A2	0,023665888	-1,45728209	0,594	0,574	1	0,364178564	2,745905716	1,034843206
AMD1	0,001894494	-1,463046037	0,156	0,395	1	0,362726477	2,756898279	0,394936709
NRN1	0,043454745	-1,465940521	0,531	0,496	1	0,361999467	2,762435004	1,070564516
COL21A1	0,001541938	-1,46973062	0	0,25	1	0,361049708	2,76970173	0
KRT14	0,113824728	-1,471931522	0,281	0,348	1	0,360499329	2,77393027	0,807471264
MT-ND2	2,09488E-05	-1,472645502	0,844	0,82	0,442041378	0,360320964	2,775303409	1,029268293
CCND2	0,002446203	-1,474457763	0,062	0,305	1	0,359868626	2,778791835	0,203278689
TENT5A	0,08880514	-1,490600538	0,25	0,328	1	0,355864385	2,810059228	0,762195122
ABCA2	0,002572903	-1,493278288	0,062	0,301	1	0,355204487	2,81527975	0,205980066
JUN	6,45673E-05	-1,499359024	1	0,934	1	0,353710506	2,827170759	1,070663812
DHRS3	0,000465946	-1,500857715	0	0,293	1	0,353343257	2,830109189	0
LIMCH1	0,009059078	-1,510090553	0,031	0,223	1	0,351089182	2,848279162	0,139013453
ABL2	0,20096991	-1,527151597	0,406	0,387	1	0,346961719	2,882162337	1,049095607
MT1E	0,559141209	-1,531640436	0,469	0,383	1	0,345883851	2,891143937	1,224543081
SOCS3	0,063316032	-1,532406171	0,531	0,512	1	0,345700316	2,892678868	1,037109375
IFI16	0,045498933	-1,539194415	0,625	0,543	1	0,34407753	2,906321723	1,151012891
TSC22D3	0,902579767	-1,541950208	0,406	0,285	1	0,343420911	2,911878598	1,424561404
CEBPB	0,136626421	-1,555146378	0,781	0,566	1	0,340294001	2,93863541	1,379858657
XIST	7,51886E-06	-1,561103938	0,781	0,832	0,158655511	0,338891666	2,950795495	0,938701923
DKK3	0,000137148	-1,56559708	0,156	0,492	1	0,33783786	2,959999803	0,317073171
TGFBR2	0,008409367	-1,569874703	0,438	0,512	1	0,336837647	2,968789294	0,85546875
SQSTM1	0,034044879	-1,571731933	0,562	0,523	1	0,336404304	2,972613576	1,07456979
NFKBIA	0,001808384	-1,574392428	0,656	0,738	1	0,335784507	2,978100475	0,888888889
CRYAB	1,97264E-07	-1,57472901	1	0,961	0,004162477	0,335706178	2,978795348	1,040582726
HSP90AA1	0,005788735	-1,57701408	1	0,922	1	0,335174877	2,983517171	1,084598698
CCNL1	0,000159697	-1,578639547	0,656	0,68	1	0,334797452	2,986880557	0,964705882
NRXN3	0,031717738	-1,587949556	0,219	0,34	1	0,332643891	3,006217842	0,644117647
HSPA12A	0,002259963	-1,589792636	0,406	0,543	1	0,332219201	3,010060816	0,747697974
SOD3	0,009874943	-1,603375158	0,062	0,254	1	0,329106139	3,038533417	0,244094488
NAMPT	0,039410979	-1,604535339	0,25	0,352	1	0,328841586	3,040977915	0,710227273

Supplementary Table 1

Differentially expressed genes_adjacent-skin-XL_ comparing SC-Keloid vs. (SC-Myel, SC-Nonmyel)								
**gene**	**p_val**	**avg_log2FC**	**pct.1**	**pct.2**	**p_val_adj**	**Foldchange_UP**	**Foldchange_DOWN**	**Ratio_pct1_pct2**
CAB39L	0,000761101	-1,605552592	0,094	0,367	1	0,328609799	3,043122883	0,25613079
RETREG1	0,001149229	-1,607363473	0,25	0,477	1	0,328197585	3,04694503	0,524109015
VGLL3	0,00037172	-1,607404633	0	0,301	1	0,328188221	3,047031962	0
NFIA	0,000994878	-1,609824742	0,156	0,41	1	0,32763815	3,052147621	0,380487805
PMP22	6,09945E-09	-1,613049936	1	0,922	0,000128704	0,326906522	3,058978434	1,084598698
RAPGEF5	0,000146496	-1,613168503	0	0,332	1	0,326879656	3,059229844	0
SOWAHC	0,002513995	-1,619482706	0,031	0,27	1	0,325452137	3,072648434	0,114814815
TXNIP	0,011911683	-1,622226571	0,312	0,441	1	0,324833747	3,078497871	0,707482993
MT-ND3	1,02546E-05	-1,630133347	0,844	0,875	0,216382902	0,323058346	3,095416081	0,964571429
MAFF	0,002033328	-1,650080538	0,25	0,461	1	0,318622369	3,138511593	0,542299349
MT1A	0,033276355	-1,655692691	0,031	0,176	1	0,317385321	3,15074433	0,176136364
ALCAM	0,001254174	-1,656223648	0,156	0,398	1	0,317268534	3,151904118	0,391959799
ADD3	0,002628605	-1,663840289	0,219	0,422	1	0,315597944	3,168588451	0,518957346
CD55	0,000805983	-1,672426669	0,031	0,309	1	0,313725202	3,187502932	0,100323625
SOD2	0,642995769	-1,679068911	0,531	0,336	1	0,312284115	3,202212193	1,580357143
BCL6	0,003096624	-1,680673201	0,312	0,469	1	0,311937045	3,205775064	0,665245203
GADD45B	0,313831708	-1,682982858	0,812	0,672	1	0,311438054	3,210911404	1,208333333
NRXN1	1,30342E-05	-1,684621321	0,844	0,809	0,275034574	0,311084556	3,214560094	1,043263288
RELN	0,000654925	-1,688762978	0,094	0,371	1	0,310192782	3,223801641	0,253369272
HSPA1A	0,018784067	-1,69450398	0,969	0,879	1	0,308960866	3,236655863	1,102389078
FOSB	0,072783521	-1,698077637	0,812	0,652	1	0,308196496	3,244683226	1,245398773
NFIB	0,000112077	-1,701386428	0,094	0,422	1	0,307490463	3,25213338	0,222748815
MBNL2	0,00057158	-1,702101991	0,375	0,535	1	0,307337989	3,253746807	0,700934579
STARD13	0,002436583	-1,708931013	0,375	0,512	1	0,305886637	3,269184983	0,732421875
BTG2	0,176353344	-1,720731278	0,406	0,398	1	0,303394896	3,29603435	1,020100503
SELENOP	2,24418E-05	-1,727633043	0,031	0,418	0,473545264	0,30194694	3,311840153	0,074162679
NFATC2	0,00810324	-1,745194952	0,094	0,305	1	0,298293625	3,352401519	0,308196721
PLLP	0,000365502	-1,751173215	0,062	0,363	1	0,297060108	3,366322078	0,170798898
TSC22D4	0,002725343	-1,756689144	0,156	0,375	1	0,295926512	3,379217343	0,416
RGCC	0,004618309	-1,75803476	0,344	0,508	1	0,295650627	3,382370643	0,677165354

Supplementary Table 1

Differentially expressed genes_adjacent-skin-XL_ comparing SC-Keloid vs. (SC-Myel, SC-Nonmyel)								
**gene**	**p_val**	**avg_log2FC**	**pct.1**	**pct.2**	**p_val_adj**	**Foldchange_UP**	**Foldchange_DOWN**	**Ratio_pct1_pct2**
PER1	0,000850035	-1,782753536	0,094	0,363	1	0,290628172	3,440822656	0,258953168
HES1	0,000194163	-1,785515348	0,594	0,73	1	0,290072343	3,447415876	0,81369863
YBX3	0,00357947	-1,795772801	0,469	0,543	1	0,288017265	3,472014086	0,863720074
DNAJB1	0,008924643	-1,802144402	0,812	0,699	1	0,286748054	3,487381998	1,161659514
IRF1	0,009089875	-1,814850041	0,156	0,355	1	0,284233786	3,518230588	0,43943662
HSPA6	0,297875812	-1,832245392	0,219	0,129	1	0,280827205	3,560908567	1,697674419
LTBP4	1,0953E-05	-1,836550542	0,312	0,652	0,231119174	0,279990437	3,571550552	0,478527607
SAMHD1	0,000827252	-1,83689586	0,219	0,457	1	0,279923428	3,572405528	0,479212254
WSB1	5,21784E-06	-1,858434816	0,562	0,715	0,110101556	0,275775306	3,626140476	0,786013986
MTURN	0,005261806	-1,865240339	0,219	0,398	1	0,274477474	3,643286219	0,550251256
TCIM	0,208448506	-1,869798269	0,688	0,559	1	0,273611682	3,654814715	1,230769231
EGR1	0,231347142	-1,88571504	0,781	0,57	1	0,270609606	3,695360313	1,370175439
ZFAND5	0,001416688	-1,891698474	0,438	0,555	1	0,269489604	3,710718277	0,789189189
DMD	1,50201E-05	-1,899757876	0,125	0,508	0,316938305	0,267988338	3,731505665	0,246062992
PPP1R15A	3,91578E-08	-1,932853556	0,812	0,855	0,000826268	0,261910616	3,818096473	0,949707602
AHR	2,15528E-05	-1,948399263	0,719	0,75	0,454784973	0,259103559	3,859460689	0,958666667
PLP1	1,68409E-08	-1,953748789	0,75	0,828	0,000355359	0,25814458	3,873798169	0,905797101
ITGB8	0,021634511	-1,956565317	0,625	0,555	1	0,257641104	3,881368249	1,126126126
FXYD1	2,20406E-07	-1,990188155	0,594	0,781	0,004650794	0,251706058	3,97288809	0,76056338
HSPA1B	0,020498363	-1,994620708	1	0,805	1	0,2509339	3,985113207	1,242236025
NDRG2	8,62269E-07	-2,005298642	0,406	0,695	0,018194742	0,249083499	4,014717967	0,584172662
DOCK5	5,03745E-05	-2,01956677	0,219	0,52	1	0,246632227	4,054620164	0,421153846
NCAM1	3,15288E-05	-2,024930487	0,156	0,508	0,665288904	0,245716989	4,069722672	0,307086614
PRX	0,137206058	-2,031403162	0,031	0,113	1	0,244617045	4,088022573	0,274336283
RARRES2	3,22499E-06	-2,050922346	0,094	0,52	0,068050569	0,241329746	4,143708011	0,180769231
JUNB	8,88571E-05	-2,061404409	0,938	0,816	1	0,239582692	4,173924224	1,149509804
BCL2	1,70476E-05	-2,068597401	0,062	0,449	0,359722259	0,238391153	4,194786547	0,138084633
MT2A	0,125004246	-2,078061558	0,906	0,738	1	0,236832412	4,222395032	1,227642276
CCN5	0,018264327	-2,093454803	0	0,152	1	0,234318895	4,26768828	0
MATN2	0,000240492	-2,094002224	0,5	0,641	1	0,234230001	4,269307933	0,780031201

Supplementary Table 1

Differentially expressed genes_adjacent-skin-XL_ comparing SC-Keloid vs. (SC-Myel, SC-Nonmyel)								
**gene**	**p_val**	**avg_log2FC**	**pct.1**	**pct.2**	**p_val_adj**	**Foldchange_UP**	**Foldchange_DOWN**	**Ratio_pct1_pct2**
CDH19	7,49304E-10	-2,098682395	1	0,918	1,58111E-05	0,233471379	4,283180259	1,089324619
CDKN1A	4,46741E-05	-2,105185751	0,188	0,508	0,942668022	0,232421309	4,30253149	0,37007874
ZFP36L2	0,053704432	-2,128795911	0,656	0,559	1	0,228648615	4,373523093	1,17352415
CHL1	7,19449E-07	-2,146949616	0,031	0,516	0,015181093	0,225789513	4,428903662	0,060077519
MT3	0,00124733	-2,168135319	0	0,258	1	0,222498063	4,494421151	0
ZFP36L1	1,48319E-06	-2,249186985	0,625	0,746	0,031296798	0,210342607	4,754148557	0,837801609
ADIRF	1,19435E-06	-2,287653243	0,25	0,641	0,025202079	0,204808395	4,882612356	0,390015601
DST	1,6753E-09	-2,313876176	0,875	0,863	3,53504E-05	0,201119354	4,972171903	1,013904983
CADM2	1,75525E-07	-2,320422696	0,031	0,543	0,003703747	0,200208801	4,994785407	0,057090239
SCN7A	2,61179E-06	-2,330290234	0,656	0,746	0,055111356	0,198844114	5,029065118	0,879356568
XKR4	1,05817E-06	-2,333336095	0,062	0,527	0,022328418	0,198424752	5,039693848	0,117647059
PCSK2	7,47954E-07	-2,361331633	0,125	0,594	0,015782577	0,194611432	5,138444275	0,21043771
KLF9	4,40649E-07	-2,411156826	0,25	0,652	0,009298126	0,188005031	5,319006596	0,383435583
AATK	5,15953E-05	-2,496597927	0,156	0,488	1	0,177194051	5,643530327	0,319672131
SAT1	5,01889E-06	-2,499737087	0,531	0,688	0,105903628	0,176808914	5,655823453	0,771802326
ATF3	6,06014E-08	-2,524379074	0,625	0,777	0,001278751	0,17381457	5,753257623	0,804375804
VMP1	3,17903E-05	-2,726511972	0,625	0,684	0,670806983	0,151090832	6,618535262	0,91374269
ANK3	1,23746E-08	-2,7411253	0,125	0,645	0,000261117	0,14956813	6,685916329	0,19379845
CLU	8,26174E-10	-2,783778854	0,438	0,797	1,74331E-05	0,145210849	6,886537809	0,549560853
MT1M	0,045648135	-2,849449353	0,156	0,281	1	0,138749132	7,207252315	0,555160142
ZFP36	6,83522E-07	-3,060017338	0,531	0,746	0,014422992	0,119906574	8,339826314	0,711796247
BIRC3	0,000109392	-3,075533987	0,156	0,473	1	0,118623851	8,430007921	0,329809725
MBP	0,004823334	-3,131080632	0,031	0,246	1	0,114143402	8,760909396	0,12601626
CEBPD	0,000650306	-3,133043865	0,281	0,512	1	0,11398818	8,77283944	0,548828125
FGL2	2,59668E-07	-3,268276216	0,031	0,535	0,005479256	0,10378888	9,634943564	0,057943925
COL28A1	1,13234E-12	-3,342514488	0,031	0,77	2,38935E-08	0,098583192	10,14371694	0,04025974
PDK4	2,17807E-07	-3,346423805	0	0,516	0,004595936	0,09831642	10,17124097	0
MPZ	2,65263E-07	-3,703191866	0,312	0,703	0,005597309	0,076776475	13,02482303	0,443812233
CCL2	0,000262071	-4,072429784	0,156	0,441	1	0,059439682	16,82377767	0,353741497

Supplementary Table 2

Differentially expressed genes_keloid-XL_ comparing SC-Keloid vs. (SC-Myel, SC-Nonmyel, SC-Nonmyel2)								
**gene**	**p_val**	**avg_log2FC**	**pct.1**	**pct.2**	**p_val_adj**	**Foldchange_UP**	**Foldchange_DOWN**	**Ratio_pct1_pct2**
TGFBI	1,62559E-63	4,114407312	0,939	0,049	3,43016E-59	17,32048367	0,057735108	19,16326531
IBSP	1,76098E-49	4,02492376	0,744	0,025	3,71584E-45	16,27881479	0,061429534	29,76
IGFBP3	2,56146E-59	3,882741596	0,902	0,042	5,40493E-55	14,75100757	0,067791979	21,47619048
SPARCL1	1,80626E-38	3,198701767	0,951	0,281	3,81138E-34	9,181321159	0,108916787	3,384341637
ELN	1,45092E-46	3,00729267	0,915	0,126	3,06158E-42	8,040541529	0,124369733	7,261904762
TAGLN	2,86713E-39	2,872219187	0,768	0,088	6,04993E-35	7,321905671	0,136576466	8,727272727
COL7A1	3,01719E-45	2,7498591	0,902	0,126	6,36657E-41	6,726514348	0,148665408	7,158730159
MMP15	9,57764E-48	2,51027746	0,829	0,07	2,02098E-43	5,697296385	0,17552185	11,84285714
COL1A1	1,86368E-30	2,492821807	0,988	0,656	3,93254E-26	5,628778225	0,177658447	1,506097561
IGFBP5	6,04209E-33	2,463500531	1	0,702	1,27494E-28	5,515533849	0,181306112	1,424501425
FN1	3,93038E-29	2,438870099	0,963	0,488	8,29349E-25	5,422169072	0,184428037	1,973360656
CCN3	3,09733E-32	2,417916588	0,793	0,137	6,53567E-28	5,343987333	0,187126192	5,788321168
ITGB1	4,92765E-35	2,248125	1	0,765	1,03978E-30	4,750650259	0,210497499	1,307189542
PCDH20	1,48287E-30	2,235276058	0,841	0,193	3,12901E-26	4,708527835	0,212380607	4,357512953
APOD	8,17119E-31	2,16544267	0,854	0,204	1,7242E-26	4,486040577	0,222913722	4,18627451
COL6A2	8,30144E-29	2,148125105	0,951	0,502	1,75169E-24	4,432513745	0,225605617	1,894422311
LOXL2	2,83567E-49	2,073879796	0,817	0,049	5,98355E-45	4,210173817	0,237519885	16,67346939
NES	1,81968E-29	2,06620237	0,902	0,288	3,83971E-25	4,187828523	0,238787237	3,131944444
COL8A1	6,91195E-32	2,01267362	0,866	0,214	1,45849E-27	4,035293531	0,247813447	4,046728972
S100A16	4,14099E-31	2,004861574	0,829	0,2	8,73791E-27	4,013501882	0,249158971	4,145
COL1A2	2,89243E-25	1,978320612	0,976	0,723	6,10332E-21	3,940341342	0,25378512	1,349930844
MYL9	4,53715E-27	1,956163331	0,927	0,354	9,57385E-23	3,880286913	0,257712902	2,618644068
TNFAIP6	8,63425E-23	1,944135252	0,89	0,442	1,82191E-18	3,848070556	0,259870495	2,013574661
TNC	6,21347E-25	1,943263571	0,927	0,365	1,3111E-20	3,845746242	0,260027557	2,539726027
S100A6	4,10404E-33	1,939016777	1	0,954	8,65993E-29	3,834442343	0,260794116	1,048218029
COL6A1	1,67035E-25	1,92907186	0,976	0,565	3,52461E-21	3,808101309	0,262598056	1,727433628
PTN	1,35095E-27	1,900107448	1	0,596	2,85065E-23	3,732409936	0,267923411	1,677852349
ACTB	7,55083E-25	1,893136081	1	0,853	1,5933E-20	3,714417753	0,269221199	1,172332943
S100A11	4,39506E-23	1,873087428	0,976	0,695	9,27401E-19	3,663156728	0,272988593	1,404316547
GPC1	3,17181E-29	1,850565564	0,927	0,298	6,69284E-25	3,606415357	0,277283646	3,110738255

Supplementary Table 2

Differentially expressed genes_keloid-XL_ comparing SC-Keloid vs. (SC-Myel, SC-Nonmyel, SC-Nonmyel2)								
**gene**	**p_val**	**avg_log2FC**	**pct.1**	**pct.2**	**p_val_adj**	**Foldchange_UP**	**Foldchange_DOWN**	**Ratio_pct1_pct2**
TNFRSF12A	4,81504E-23	1,813453931	0,854	0,309	1,01602E-18	3,514827609	0,284508975	2,763754045
POSTN	1,64269E-10	1,767518868	0,415	0,109	3,46623E-06	3,404679197	0,293713429	3,80733945
COL5A1	2,20943E-31	1,757190004	0,878	0,189	4,66211E-27	3,380390709	0,295823793	4,645502646
MMP2	4,2307E-27	1,753113296	0,707	0,119	8,92719E-23	3,370852024	0,296660901	5,941176471
SH3BGRL3	2,26043E-23	1,750267321	0,878	0,323	4,76973E-19	3,364208967	0,297246696	2,718266254
SCARA3	1,22164E-31	1,734678751	0,732	0,105	2,57779E-27	3,328053788	0,300475913	6,971428571
COL12A1	1,59351E-21	1,72302214	0,756	0,214	3,36246E-17	3,301272295	0,302913517	3,53271028
CCN2	8,73775E-19	1,718610944	0,659	0,154	1,84375E-14	3,291193716	0,303841125	4,279220779
TPM2	5,2851E-27	1,703018669	0,622	0,077	1,11521E-22	3,255814872	0,307142771	8,077922078
S100A13	3,97585E-26	1,690629594	0,915	0,33	8,38945E-22	3,227975421	0,309791702	2,772727273
PDLIM7	1,10251E-28	1,681052191	0,805	0,175	2,32642E-24	3,206617318	0,311855111	4,6
HMCN2	5,90033E-45	1,646631677	0,695	0,021	1,24503E-40	3,131017727	0,319384969	33,0952381
MYL6	1,48839E-27	1,636808819	0,976	0,758	3,14064E-23	3,10977202	0,321566981	1,287598945
CD151	1,21178E-24	1,630034051	0,89	0,309	2,55697E-20	3,09520304	0,323080582	2,8802589
S100A4	6,69157E-09	1,56417428	0,817	0,596	0,000141199	2,957082062	0,338171204	1,370805369
CDH2	7,17837E-39	1,559836326	0,744	0,063	1,51471E-34	2,948203941	0,339189561	11,80952381
TMSB10	5,12584E-23	1,537081364	1	0,923	1,0816E-18	2,90206808	0,344581854	1,083423619
PMEPA1	8,79212E-18	1,531498495	0,902	0,604	1,85523E-13	2,890859503	0,345917883	1,493377483
CALR	9,47057E-19	1,517175727	0,878	0,456	1,99838E-14	2,86230166	0,349369186	1,925438596
PPP1R14B	4,432E-21	1,512759122	0,89	0,379	9,35197E-17	2,853552532	0,350440368	2,34828496
TUBB3	9,55507E-27	1,497262433	0,598	0,074	2,01622E-22	2,823065169	0,354224908	8,081081081
COL5A2	3,05732E-25	1,488710361	0,927	0,326	6,45124E-21	2,806379981	0,356330934	2,843558282
MMP14	1,2458E-20	1,479955532	0,89	0,316	2,62876E-16	2,789401355	0,358499862	2,816455696
CSRP2	5,96579E-21	1,479317957	0,549	0,091	1,25884E-16	2,788168897	0,35865833	6,032967033
ENC1	1,06132E-32	1,458627118	0,622	0,046	2,23949E-28	2,748466925	0,363839197	13,52173913
CCND1	3,18616E-15	1,455715169	0,744	0,305	6,72313E-11	2,742924989	0,364574315	2,439344262
SLC15A3	2,22243E-26	1,448380917	0,707	0,119	4,68954E-22	2,729016122	0,366432427	5,941176471
CDKN2A	1,76597E-22	1,437972217	0,427	0,028	3,72636E-18	2,709397779	0,369085709	15,25
PPIB	3,62907E-23	1,435850422	0,939	0,551	7,65771E-19	2,705415952	0,369628929	1,704174229
CFL1	6,82296E-24	1,433647643	0,988	0,709	1,43971E-19	2,701288339	0,370193728	1,393511989

Supplementary Table 2

Differentially expressed genes_keloid-XL_ comparing SC-Keloid vs. (SC-Myel, SC-Nonmyel, SC-Nonmyel2)								
**gene**	**p_val**	**avg_log2FC**	**pct.1**	**pct.2**	**p_val_adj**	**Foldchange_UP**	**Foldchange_DOWN**	**Ratio_pct1_pct2**
ISLR	1,88969E-37	1,427952535	0,512	0	3,98742E-33	2,690645893	0,371657974	#DIV/0!
ATOX1	2,77868E-25	1,426045969	0,768	0,193	5,86329E-21	2,68709247	0,372149456	3,979274611
ATP5F1E	2,55495E-21	1,416318029	0,963	0,635	5,39121E-17	2,66903464	0,374667299	1,516535433
MYL12A	1,01094E-16	1,397384056	0,866	0,582	2,13319E-12	2,634235003	0,379616852	1,487972509
LYPD1	4,35129E-32	1,389825846	0,646	0,056	9,18165E-28	2,620470461	0,381610865	11,53571429
CALB2	2,94856E-25	1,385608713	0,366	0,004	6,22175E-21	2,612821764	0,382727982	91,5
ACTG1	2,10965E-19	1,360547517	0,939	0,888	4,45157E-15	2,567826125	0,389434468	1,057432432
MYH9	9,80578E-19	1,359781339	0,72	0,204	2,06912E-14	2,566462782	0,389641341	3,529411765
S100A10	8,75227E-24	1,34296843	1	0,891	1,84682E-19	2,536727288	0,394208713	1,122334456
UQCR11	1,14514E-19	1,317434553	0,841	0,326	2,41637E-15	2,492225399	0,401247817	2,579754601
PHLDA2	1,18899E-25	1,313456336	0,72	0,13	2,50889E-21	2,485362579	0,40235578	5,538461538
COL4A2	1,18968E-17	1,303975734	0,976	0,719	2,51034E-13	2,469083683	0,405008549	1,35744089
TMED9	6,44674E-23	1,293342579	0,793	0,232	1,36033E-18	2,450952593	0,408004628	3,418103448
OST4	2,0745E-16	1,287697766	0,902	0,47	4,3774E-12	2,441381521	0,409604149	1,919148936
ADAMTSL1	5,12554E-28	1,281280043	0,732	0,112	1,08154E-23	2,430545333	0,411430302	6,535714286
RGS16	4,81023E-17	1,277923538	0,78	0,284	1,01501E-12	2,424897117	0,41238863	2,746478873
CDK2AP1	3,21289E-16	1,254193557	0,805	0,33	6,77952E-12	2,385337749	0,419227843	2,439393939
PRRX1	9,43329E-27	1,253395425	0,598	0,063	1,99052E-22	2,38401849	0,419459834	9,492063492
BSG	3,59957E-20	1,250317609	0,963	0,421	7,59545E-16	2,378937895	0,420355656	2,287410926
PFN1	2,12696E-17	1,249913788	0,89	0,561	4,4881E-13	2,378272106	0,420473333	1,586452763
COL4A1	1,37339E-14	1,242931285	0,951	0,635	2,89799E-10	2,366789312	0,422513316	1,497637795
COL6A3	8,11708E-18	1,24174266	0,354	0,028	1,71279E-13	2,364840136	0,422861565	12,64285714
CDH6	8,03257E-19	1,239794178	0,683	0,168	1,69495E-14	2,361648375	0,423433061	4,06547619
EMP3	1,23338E-16	1,23844335	0,915	0,47	2,60256E-12	2,359438144	0,423829717	1,946808511
ADAMTS16	1,03115E-34	1,234480386	0,573	0,025	2,17583E-30	2,352965856	0,424995542	22,92
TIMP4	4,83669E-23	1,22783714	0,659	0,112	1,02059E-18	2,34215595	0,426957052	5,883928571
NGFR	6,22554E-16	1,226256474	0,902	0,446	1,31365E-11	2,339591211	0,427425097	2,022421525
THBS2	2,33723E-20	1,220693936	0,646	0,13	4,93178E-16	2,330587916	0,429076283	4,969230769
TENM3	3,80701E-19	1,202970271	0,72	0,196	8,03316E-15	2,302131543	0,434380044	3,673469388
LOXL3	1,8427E-28	1,19256383	0,646	0,07	3,88828E-24	2,285585566	0,437524639	9,228571429

Supplementary Table 2

Differentially expressed genes_keloid-XL_ comparing SC-Keloid vs. (SC-Myel, SC-Nonmyel, SC-Nonmyel2)								
**gene**	**p_val**	**avg_log2FC**	**pct.1**	**pct.2**	**p_val_adj**	**Foldchange_UP**	**Foldchange_DOWN**	**Ratio_pct1_pct2**
P3H2	4,94023E-34	1,188752101	0,598	0,035	1,04244E-29	2,279554814	0,438682147	17,08571429
TPST2	1,56728E-31	1,183237268	0,585	0,042	3,30711E-27	2,270857642	0,440362258	13,92857143
CD276	2,14028E-25	1,181847037	0,634	0,095	4,5162E-21	2,26867042	0,440786811	6,673684211
FLNA	1,07565E-13	1,178181262	0,866	0,53	2,26974E-09	2,262913224	0,44190824	1,633962264
COL5A3	1,41652E-11	1,175518208	0,683	0,263	2,989E-07	2,258739991	0,442724707	2,596958175
DSEL	4,70947E-24	1,173977256	0,598	0,081	9,93745E-20	2,256328704	0,443197837	7,382716049
STMP1	1,16258E-20	1,168524364	0,78	0,211	2,45315E-16	2,247816653	0,444876142	3,696682464
ANGPTL2	1,33042E-19	1,16814382	0,707	0,168	2,80733E-15	2,247223818	0,444993504	4,208333333
RCAN1	8,01016E-08	1,167599034	0,793	0,512	0,001690224	2,246375387	0,445161573	1,548828125
PDIA4	3,35776E-21	1,162338444	0,72	0,158	7,08521E-17	2,238199204	0,446787756	4,556962025
PPFIBP1	1,01044E-14	1,160154914	0,805	0,326	2,13214E-10	2,234814234	0,447464485	2,469325153
TAGLN2	1,3442E-18	1,157733351	0,951	0,667	2,83641E-14	2,231066245	0,448216185	1,425787106
P4HB	3,08161E-17	1,15324973	0,805	0,316	6,50249E-13	2,224143281	0,449611322	2,547468354
CYTOR	2,73679E-18	1,152021859	0,598	0,13	5,7749E-14	2,222251128	0,449994147	4,6
CRIP2	5,09393E-16	1,1352815	0,878	0,407	1,07487E-11	2,196614192	0,45524608	2,157248157
GAPDH	9,14084E-13	1,129295436	0,963	0,761	1,92881E-08	2,18751883	0,457138922	1,26544021
FKBP10	4,16567E-24	1,128440052	0,695	0,123	8,78997E-20	2,18622222	0,457410043	5,650406504
GRN	9,37728E-25	1,126470611	0,744	0,137	1,9787E-20	2,183239817	0,458034886	5,430656934
ABHD2	1,82113E-19	1,121888852	0,646	0,14	3,84277E-15	2,176317209	0,459491841	4,614285714
KIRREL3	9,38378E-22	1,120760419	0,61	0,098	1,98007E-17	2,174615625	0,459851382	6,224489796
PLOD1	2,72906E-23	1,113425352	0,646	0,105	5,75859E-19	2,163587327	0,462195349	6,152380952
BBLN	7,83839E-16	1,112231867	0,78	0,26	1,65398E-11	2,161798217	0,462577863	3
APOC2	4,44145E-22	1,111506396	0,5	0,056	9,37191E-18	2,160711411	0,462810533	8,928571429
ANXA5	8,65981E-17	1,110886539	0,976	0,649	1,82731E-12	2,159783257	0,463009423	1,50385208
ELOB	1,75296E-14	1,110757793	0,854	0,432	3,69893E-10	2,159590526	0,463050744	1,976851852
CAP1	6,03086E-14	1,109323682	0,768	0,337	1,27257E-09	2,157444852	0,463511269	2,278931751
LGALS1	5,92638E-12	1,106029625	0,988	0,898	1,25053E-07	2,15252445	0,464570797	1,100222717
BMP1	1,70077E-23	1,104707308	0,659	0,109	3,5888E-19	2,150552436	0,4649968	6,04587156
APRT	3,49156E-21	1,095907891	0,732	0,172	7,36755E-17	2,137475511	0,467841617	4,255813953
LSM7	2,70165E-19	1,091575216	0,805	0,277	5,70076E-15	2,131065913	0,469248742	2,906137184

Supplementary Table 2

Differentially expressed genes_keloid-XL_ comparing SC-Keloid vs. (SC-Myel, SC-Nonmyel, SC-Nonmyel2)								
**gene**	**p_val**	**avg_log2FC**	**pct.1**	**pct.2**	**p_val_adj**	**Foldchange_UP**	**Foldchange_DOWN**	**Ratio_pct1_pct2**
CRLF1	7,62731E-11	1,086386213	0,378	0,084	1,60944E-06	2,123414786	0,470939548	4,5
DBNDD1	1,67922E-32	1,085994828	0,463	0,004	3,54332E-28	2,122838807	0,471067326	115,75
SERF2	1,57319E-15	1,080888041	0,988	0,856	3,31959E-11	2,115337763	0,472737743	1,154205607
PLS3	1,2554E-19	1,080113085	0,768	0,214	2,64902E-15	2,114201796	0,472991747	3,588785047
VAMP5	2,77017E-18	1,079660022	0,72	0,218	5,84533E-14	2,113537956	0,473140308	3,302752294
TRAF1	4,57707E-26	1,074681264	0,5	0,035	9,65808E-22	2,106256683	0,474775942	14,28571429
NDUFA4	1,01263E-14	1,073128757	0,878	0,502	2,13674E-10	2,103991326	0,47528713	1,749003984
TIMP1	3,42035E-15	1,072740781	0,976	0,653	7,21729E-11	2,103425587	0,475414964	1,494640123
CYTL1	6,40469E-25	1,069086869	0,573	0,067	1,35145E-20	2,098104984	0,476620573	8,552238806
SPARC	4,64894E-17	1,066926586	1	0,926	9,80973E-13	2,094965645	0,477334797	1,079913607
PDIA6	1,4584E-14	1,066890247	0,793	0,316	3,07737E-10	2,094912877	0,47734682	2,509493671
ARHGDIA	2,07492E-18	1,064881859	0,817	0,26	4,37829E-14	2,091998561	0,478011801	3,142307692
ECM1	5,26123E-18	1,058558764	0,646	0,144	1,11017E-13	2,082849739	0,480111446	4,486111111
NDUFB2	1,40457E-16	1,058438247	0,817	0,277	2,96377E-12	2,082675753	0,480151554	2,949458484
TXNDC17	1,76196E-21	1,050111493	0,683	0,137	3,71791E-17	2,070689867	0,482930842	4,98540146
CAPNS1	1,38345E-19	1,046388391	0,829	0,232	2,91922E-15	2,065353014	0,48417873	3,573275862
POLR2L	2,31601E-17	1,043107339	0,866	0,358	4,887E-13	2,060661219	0,485281128	2,418994413
PDLIM1	1,61542E-19	1,037855495	0,573	0,102	3,40869E-15	2,053173428	0,487050917	5,617647059
LAMB2	6,99846E-18	1,035362193	0,732	0,196	1,47675E-13	2,049628147	0,487893378	3,734693878
SCRG1	5,98067E-07	1,035312396	0,366	0,126	0,012619813	2,049557402	0,487910219	2,904761905
SEM1	1,33093E-15	1,033872991	0,829	0,365	2,80839E-11	2,047513538	0,488397259	2,271232877
HSBP1	9,92472E-15	1,028784106	0,915	0,432	2,09422E-10	2,040303972	0,490123047	2,118055556
TPM4	4,15742E-14	1,025928253	0,866	0,495	8,77258E-10	2,036269131	0,49109422	1,749494949
NCS1	8,47452E-24	1,024926172	0,537	0,06	1,78821E-19	2,034855251	0,491435447	8,95
CYBA	3,14133E-17	1,018306593	0,768	0,235	6,62852E-13	2,025540025	0,493695502	3,268085106
SSR4	1,6312E-15	1,017204361	0,89	0,386	3,44199E-11	2,023993085	0,494072834	2,305699482
FRMD4A	3,26816E-16	1,01454576	0,695	0,193	6,89614E-12	2,020266701	0,494984152	3,601036269
UQCRQ	6,8204E-15	1,010868812	0,768	0,274	1,43917E-10	2,015124272	0,49624731	2,802919708
VCAN	4,00491E-18	1,00951676	0,5	0,077	8,45076E-14	2,013236641	0,496712597	6,493506494
ANXA2	1,99468E-12	1,009063799	0,963	0,832	4,20898E-08	2,012604646	0,496868574	1,157451923

Supplementary Table 2

Differentially expressed genes_keloid-XL_ comparing SC-Keloid vs. (SC-Myel, SC-Nonmyel, SC-Nonmyel2)								
**gene**	**p_val**	**avg_log2FC**	**pct.1**	**pct.2**	**p_val_adj**	**Foldchange_UP**	**Foldchange_DOWN**	**Ratio_pct1_pct2**
SCX	6,24715E-16	1,007808137	0,293	0,018	1,31821E-11	2,010853721	0,497301216	16,27777778
DYNLT1	2,4264E-20	1,00406928	0,683	0,151	5,11994E-16	2,005649183	0,498591682	4,523178808
MAGED2	1,99518E-12	1,004029625	0,72	0,312	4,21003E-08	2,005594055	0,498605387	2,307692308
RAB13	1,00729E-14	1,001607342	0,817	0,337	2,12548E-10	2,002229492	0,499443248	2,424332344
ATP5MF	2,03747E-14	0,99891078	0,78	0,312	4,29927E-10	1,998490591	0,500377637	2,5
STX8	3,63073E-19	0,996126176	0,561	0,102	7,66121E-15	1,994636943	0,501344369	5,5
NDUFS5	7,91025E-13	0,991379512	0,829	0,428	1,66914E-08	1,988085099	0,502996577	1,936915888
APP	2,407E-13	0,990634012	0,951	0,54	5,07901E-09	1,987058039	0,503256563	1,761111111
FXYD5	4,36389E-18	0,986372999	0,695	0,172	9,20825E-14	1,981197903	0,504745134	4,040697674
MFGE8	6,3249E-14	0,98105308	0,72	0,235	1,33462E-09	1,973905715	0,50660981	3,063829787
PLAT	1,21971E-11	0,977582849	0,915	0,495	2,5737E-07	1,969163426	0,507829867	1,848484848
RHOC	3,56084E-13	0,973215112	0,829	0,389	7,51374E-09	1,963210829	0,509369643	2,131105398
BEX3	2,37171E-13	0,972839403	0,854	0,396	5,00455E-09	1,962699633	0,509502312	2,156565657
PERP	3,77597E-22	0,971764878	0,622	0,098	7,96768E-18	1,96123835	0,509881932	6,346938776
PTMS	8,4227E-12	0,9716285	0,927	0,632	1,77727E-07	1,961052964	0,509930134	1,466772152
PKM	3,06907E-12	0,971155154	0,744	0,344	6,47605E-08	1,96040965	0,510097469	2,162790698
CAMK2N1	2,55643E-13	0,966619342	0,671	0,221	5,39431E-09	1,954255831	0,511703731	3,036199095
PHPT1	1,36833E-14	0,961893377	0,744	0,256	2,88731E-10	1,947864575	0,513382713	2,90625
KCNMA1	6,30954E-21	0,960681885	0,72	0,151	1,33138E-16	1,946229557	0,513814003	4,768211921
SERPINE2	2,58298E-13	0,958848978	0,695	0,218	5,45035E-09	1,943758491	0,514467206	3,188073394
CD63	1,8822E-12	0,958002214	0,963	0,768	3,97162E-08	1,942617972	0,514769252	1,25390625
SDCCAG8	1,53642E-17	0,957897798	0,585	0,116	3,242E-13	1,94247738	0,51480651	5,043103448
GPR17	5,91491E-30	0,948342481	0,524	0,025	1,2481E-25	1,929654394	0,518227514	20,96
EMC10	9,49018E-16	0,945390446	0,793	0,277	2,00252E-11	1,925709983	0,519288994	2,862815884
CYB5R3	1,2062E-12	0,941679138	0,744	0,277	2,5452E-08	1,920762495	0,520626576	2,685920578
HLA-A	7,74157E-10	0,941426965	0,951	0,621	1,63355E-05	1,920426787	0,520717586	1,531400966
CTSB	5,99762E-17	0,93725039	0,537	0,102	1,26556E-12	1,914875227	0,522227237	5,264705882
PDIA3	7,06661E-13	0,933322318	0,866	0,449	1,49113E-08	1,909668626	0,52365106	1,928730512
ANXA6	2,31451E-16	0,932048543	0,512	0,095	4,88384E-12	1,907983299	0,524113602	5,389473684
ANAPC11	8,98081E-19	0,930209237	0,732	0,189	1,89504E-14	1,905552342	0,524782226	3,873015873

Supplementary Table 2

Differentially expressed genes_keloid-XL_ comparing SC-Keloid vs. (SC-Myel, SC-Nonmyel, SC-Nonmyel2)								
gene	p_val	avg_log2FC	pct.1	pct.2	p_val_adj	Foldchange_UP	Foldchange_DOWN	Ratio_pct1_pct2
COTL1	7,51593E-10	0,927957889	0,659	0,253	1,58594E-05	1,902581018	0,525601796	2,604743083
IL1RAP	9,08427E-16	0,925825804	0,61	0,147	1,91687E-11	1,899771366	0,526379131	4,149659864
ZYX	1,03062E-19	0,925783839	0,549	0,095	2,17471E-15	1,899716107	0,526394442	5,778947368
RPS26	2,58818E-10	0,911434282	0,805	0,446	5,46132E-06	1,880914515	0,531656273	1,804932735
PET100	8,35814E-17	0,910132754	0,744	0,218	1,76365E-12	1,879218412	0,532136123	3,412844037
PLOD3	1,74388E-19	0,908933624	0,695	0,154	3,67976E-15	1,877657105	0,532578604	4,512987013
CHPF	5,33508E-16	0,908081844	0,561	0,116	1,12575E-11	1,876548847	0,532893136	4,836206897
ZNF385A	2,03398E-24	0,906735798	0,439	0,025	4,29191E-20	1,874798828	0,533390562	17,56
EVA1B	3,40075E-23	0,903360178	0,61	0,084	7,17593E-19	1,870417299	0,534640051	7,261904762
SLIRP	1,17962E-13	0,900426541	0,561	0,151	2,48912E-09	1,866617777	0,535728317	3,715231788
ARF5	2,44054E-16	0,899238362	0,78	0,242	5,14979E-12	1,865081096	0,536169715	3,223140496
SHISA4	8,82195E-18	0,897446618	0,634	0,144	1,86152E-13	1,862766211	0,536836021	4,402777778
P4HA2	1,82312E-13	0,896586485	0,768	0,288	3,84697E-09	1,861655963	0,537156177	2,666666667
SEPTIN9	1,27153E-16	0,893258661	0,671	0,179	2,68305E-12	1,857366682	0,53839665	3,748603352
CD82	1,34986E-13	0,891355268	0,598	0,161	2,84833E-09	1,854917816	0,539107443	3,714285714
PDGFB	5,50193E-19	0,889679957	0,561	0,091	1,16096E-14	1,852765067	0,539733838	6,164835165
MYLK	3,63074E-26	0,887974269	0,476	0,028	7,66121E-22	1,850575851	0,540372339	17
TLE5	9,24541E-10	0,887629328	0,878	0,46	1,95087E-05	1,85013344	0,540501554	1,908695652
TMSB4X	3,99536E-13	0,870784216	1	0,912	8,43061E-09	1,828656646	0,546849515	1,096491228
MICOS13	7,2432E-13	0,870502663	0,61	0,186	1,52839E-08	1,828299804	0,546956247	3,279569892
TMCO1	1,17643E-15	0,869918559	0,695	0,207	2,48239E-11	1,827559731	0,547177738	3,357487923
FIBIN	1,20587E-12	0,865798401	0,561	0,158	2,54451E-08	1,822347892	0,548742644	3,550632911
PDLIM4	2,35977E-09	0,864493834	0,793	0,463	4,97935E-05	1,820700767	0,549239072	1,712742981
ARPC2	1,2248E-12	0,863781288	0,805	0,375	2,58446E-08	1,819801746	0,549510408	2,146666667
GUK1	3,37041E-12	0,861464405	0,78	0,351	7,11191E-08	1,816881598	0,550393598	2,222222222
RPN2	2,27221E-12	0,859516775	0,732	0,27	4,7946E-08	1,814430473	0,551137128	2,711111111
RSU1	8,13371E-19	0,858515075	0,573	0,102	1,71629E-14	1,813171105	0,55151993	5,617647059
ARL4C	3,56791E-22	0,858290988	0,598	0,084	7,52864E-18	1,812889495	0,551605601	7,119047619
CTSA	3,42944E-18	0,857684885	0,573	0,105	7,23646E-14	1,812128027	0,55183739	5,457142857
URM1	3,08305E-19	0,856940313	0,549	0,091	6,50555E-15	1,811193032	0,552122265	6,032967033

Supplementary Table 2

Differentially expressed genes_keloid-XL_ comparing SC-Keloid vs. (SC-Myel, SC-Nonmyel, SC-Nonmyel2)								
**gene**	**p_val**	**avg_log2FC**	**pct.1**	**pct.2**	**p_val_adj**	**Foldchange_UP**	**Foldchange_DOWN**	**Ratio_pct1_pct2**
DRAXIN	9,20887E-18	0,854681362	0,415	0,049	1,94316E-13	1,808359311	0,552987448	8,469387755
FSCN1	7,43725E-14	0,846966148	0,622	0,175	1,56933E-09	1,798714418	0,555952624	3,554285714
AP2S1	3,94473E-17	0,844919414	0,683	0,179	8,32378E-13	1,796164413	0,556741907	3,815642458
ATP5F1D	2,64692E-14	0,843742567	0,793	0,291	5,58527E-10	1,794699828	0,557196242	2,725085911
AQP1	2,18541E-12	0,841925839	0,561	0,158	4,61144E-08	1,792441257	0,557898339	3,550632911
HSP90B1	3,20682E-09	0,841652819	0,927	0,628	6,76672E-05	1,792102083	0,558003927	1,47611465
DNPH1	2,00334E-16	0,841029495	0,512	0,095	4,22725E-12	1,791327963	0,558245068	5,389473684
HLA-B	2,55208E-07	0,840464841	0,963	0,786	0,00538515	1,790626994	0,558463601	1,22519084
PLAC9	4,34098E-09	0,837939222	0,598	0,232	9,15989E-05	1,787495019	0,559442118	2,577586207
GSN	4,73998E-12	0,836296674	0,976	0,709	1,00018E-07	1,785461065	0,560079421	1,376586742
OS9	1,22853E-11	0,832599471	0,683	0,232	2,59231E-07	1,780891312	0,561516581	2,943965517
TIMM8B	4,53808E-15	0,831204055	0,585	0,137	9,5758E-11	1,779169615	0,562059958	4,270072993
CSPG4	2,05286E-14	0,830909508	0,439	0,077	4,33173E-10	1,778806409	0,562174723	5,701298701
SELENON	5,36583E-22	0,830107698	0,622	0,102	1,13224E-17	1,777818073	0,562487251	6,098039216
PLN	1,48819E-14	0,828904962	0,232	0,007	3,14022E-10	1,776336571	0,562956377	33,14285714
CLIC1	1,08167E-11	0,82736821	0,854	0,509	2,28244E-07	1,774445433	0,563556355	1,677799607
TIMP2	1,882E-08	0,826709309	0,963	0,635	0,000397121	1,773635202	0,563813798	1,516535433
GNG5	3,88886E-11	0,826677453	0,878	0,491	8,20589E-07	1,773596039	0,563826248	1,788187373
DDOST	1,3336E-16	0,82657897	0,61	0,137	2,81403E-12	1,773474971	0,563864738	4,452554745
RABAC1	1,35267E-13	0,82629854	0,866	0,365	2,85427E-09	1,773130278	0,563974352	2,37260274
TSPAN3	2,86913E-12	0,825928032	0,683	0,232	6,05415E-08	1,772674967	0,564119209	2,943965517
DAP	7,61574E-14	0,825857676	0,622	0,168	1,607E-09	1,772588521	0,56414672	3,702380952
TMEM54	1,29734E-21	0,821362452	0,378	0,018	2,73751E-17	1,767073993	0,565907259	21
ERGIC1	4,48645E-19	0,820169183	0,524	0,081	9,46686E-15	1,765613032	0,566375521	6,469135802
CHCHD2	3,72429E-10	0,819493382	0,793	0,456	7,85863E-06	1,76478616	0,56664089	1,739035088
FHDC1	2,31128E-17	0,818784223	0,476	0,07	4,87703E-13	1,763918889	0,566919492	6,8
NME2	1,9937E-09	0,816974836	0,915	0,593	4,20691E-05	1,76170802	0,567630952	1,543001686
RTN1	1,38412E-20	0,816559361	0,317	0,007	2,92063E-16	1,761200746	0,567794445	45,28571429
SERPINE1	3,47646E-14	0,815271163	0,451	0,084	7,33568E-10	1,759628853	0,568301661	5,369047619
RPL28	7,02416E-13	0,812978946	0,988	0,933	1,48217E-08	1,756835297	0,569205321	1,058949625

Supplementary Table 2

Differentially expressed genes_keloid-XL_ comparing SC-Keloid vs. (SC-Myel, SC-Nonmyel, SC-Nonmyel2)								
**gene**	**p_val**	**avg_log2FC**	**pct.1**	**pct.2**	**p_val_adj**	**Foldchange_UP**	**Foldchange_DOWN**	**Ratio_pct1_pct2**
ATP5PF	8,01045E-11	0,811054529	0,805	0,389	1,69028E-06	1,75449341	0,569965093	2,06940874
TMEM160	4,88517E-16	0,810074029	0,573	0,126	1,03082E-11	1,753301408	0,570352591	4,547619048
OSTC	1,24774E-13	0,809768479	0,61	0,179	2,63285E-09	1,752930113	0,570473399	3,407821229
RBM3	6,25709E-10	0,809521901	0,854	0,463	1,32031E-05	1,752630537	0,57057091	1,844492441
CHST3	9,08483E-15	0,808531958	0,585	0,14	1,91699E-10	1,751428336	0,570962556	4,178571429
CALU	2,28741E-10	0,801247308	0,841	0,432	4,82667E-06	1,742607078	0,573852828	1,946759259
FKBP1A	2,9835E-11	0,801152354	0,732	0,291	6,29549E-07	1,742492388	0,573890599	2,515463918
CAVIN3	3,9452E-09	0,797370779	0,878	0,572	8,32476E-05	1,737930969	0,575396847	1,534965035
BCL7C	1,76738E-14	0,792981077	0,634	0,165	3,72935E-10	1,732650986	0,577150279	3,842424242
PRTFDC1	1,77489E-21	0,792809286	0,329	0,007	3,7452E-17	1,732444681	0,577219008	47
HLA-C	4,8583E-06	0,790147963	0,951	0,67	0,102515065	1,729251806	0,57828478	1,419402985
FAAP20	1,73767E-17	0,7859519	0,622	0,133	3,66665E-13	1,724229603	0,579969163	4,676691729
BRK1	5,13362E-10	0,784857987	0,793	0,389	1,08324E-05	1,722922714	0,580409087	2,038560411
NID1	4,45846E-12	0,784413547	0,549	0,154	9,40779E-08	1,722392028	0,580587917	3,564935065
CALD1	3,58349E-09	0,781451222	0,78	0,453	7,56152E-05	1,718859022	0,581781279	1,721854305
DHRS7	9,03911E-15	0,781309239	0,549	0,126	1,90734E-10	1,718689868	0,581838538	4,357142857
C4orf48	1,16306E-12	0,781189928	0,573	0,161	2,45418E-08	1,718547739	0,581886658	3,559006211
CMTM3	4,18412E-15	0,778870831	0,683	0,196	8,82892E-11	1,715787435	0,582822779	3,484693878
PPP1CA	6,90533E-27	0,776780167	0,561	0,049	1,45709E-22	1,713302822	0,583667982	11,44897959
RCN2	3,09253E-14	0,775512401	0,707	0,211	6,52554E-10	1,711797921	0,584181104	3,3507109
GNAI2	2,56256E-10	0,774735853	0,902	0,509	5,40726E-06	1,710876773	0,584495631	1,772102161
ABLIM3	2,55859E-22	0,774450886	0,488	0,046	5,39889E-18	1,710538867	0,584611095	10,60869565
NDUFB3	1,1447E-14	0,772620519	0,61	0,154	2,41543E-10	1,708370059	0,58535327	3,961038961
DCTN2	7,82173E-17	0,771340457	0,671	0,168	1,65046E-12	1,706854944	0,585872867	3,994047619
PPIA	1,76878E-13	0,770271176	0,963	0,779	3,73229E-09	1,705590344	0,586307259	1,236200257
CTSD	2,25293E-09	0,770112053	0,841	0,418	4,75391E-05	1,705402236	0,58637193	2,011961722
GSTP1	6,14185E-10	0,769461295	0,793	0,358	1,29599E-05	1,704633152	0,586636485	2,215083799
ARPC3	9,65905E-12	0,769407	0,78	0,337	2,03816E-07	1,704569	0,586658563	2,314540059
RGS3	4,67365E-11	0,768462201	0,646	0,221	9,86186E-07	1,703453069	0,587042883	2,923076923
TPPP3	6,18389E-09	0,767412206	0,585	0,214	0,000130486	1,702213745	0,587470289	2,73364486

Supplementary Table 2

Differentially expressed genes_keloid-XL_ comparing SC-Keloid vs. (SC-Myel, SC-Nonmyel, SC-Nonmyel2)								
**gene**	**p_val**	**avg_log2FC**	**pct.1**	**pct.2**	**p_val_adj**	**Foldchange_UP**	**Foldchange_DOWN**	**Ratio_pct1_pct2**
FABP5	9,54174E-10	0,764334671	0,622	0,228	2,0134E-05	1,698586479	0,588724809	2,728070175
SLN	1,82136E-07	0,764042252	0,11	0,004	0,003843249	1,698242229	0,588844149	27,5
NDUFA13	3,93307E-10	0,763491016	0,805	0,414	8,29917E-06	1,697593475	0,589069182	1,944444444
C1QL1	8,43349E-13	0,761280353	0,634	0,186	1,77955E-08	1,694994219	0,589972514	3,408602151
RPL36A	5,76965E-09	0,757560189	0,951	0,8	0,000121745	1,690629102	0,591495792	1,18875
SRM	3,5492E-09	0,755832786	0,5	0,158	7,48918E-05	1,688606047	0,592204441	3,164556962
LTBP2	4,5483E-17	0,754699597	0,451	0,06	9,59737E-13	1,687280225	0,59266978	7,516666667
QPCT	5,55105E-24	0,75190268	0,427	0,021	1,17133E-19	1,684012305	0,593819889	20,33333333
C12orf75	1,80795E-17	0,751259386	0,39	0,039	3,81495E-13	1,683261575	0,594084731	10
PSMB3	9,33162E-15	0,750991957	0,695	0,204	1,96907E-10	1,682949582	0,594194865	3,406862745
MCRIP1	3,44343E-15	0,748278747	0,732	0,232	7,26598E-11	1,679787511	0,595313391	3,155172414
OPRD1	6,12815E-23	0,74769693	0,366	0,011	1,2931E-18	1,679110214	0,595553521	33,27272727
ZNHIT1	1,78906E-10	0,747668856	0,659	0,242	3,7751E-06	1,679077541	0,59556511	2,723140496
MLEC	5,78727E-12	0,746779001	0,561	0,154	1,22117E-07	1,678042204	0,595932568	3,642857143
RPL36	6,84888E-09	0,746646571	0,976	0,849	0,000144518	1,677888177	0,595987274	1,14958775
PXDN	1,57083E-11	0,745987824	0,476	0,116	3,3146E-07	1,677122214	0,596259468	4,103448276
EFEMP2	2,25925E-19	0,745515884	0,573	0,095	4,76725E-15	1,676573678	0,596454551	6,031578947
RPS2	9,85872E-08	0,74529298	1	0,916	0,002080288	1,676314658	0,596546713	1,091703057
SSR2	7,03046E-10	0,744582334	0,756	0,34	1,4835E-05	1,675489139	0,596840634	2,223529412
UBTD1	3,77206E-18	0,744136435	0,463	0,063	7,95942E-14	1,674971369	0,597025131	7,349206349
TPGS1	3,44593E-22	0,743632729	0,61	0,091	7,27126E-18	1,674386668	0,597233613	6,703296703
NDUFAF8	1,67423E-17	0,743469769	0,573	0,112	3,53279E-13	1,674197547	0,597301078	5,116071429
LINC00623	1,5587E-19	0,743216027	0,573	0,091	3,28902E-15	1,673903115	0,597406141	6,296703297
ANXA1	9,19237E-12	0,742604441	0,951	0,667	1,93968E-07	1,673193665	0,597659447	1,425787106
PGM3	2,66793E-12	0,741968513	0,5	0,13	5,62961E-08	1,672456298	0,597922948	3,846153846
NEDD8	4,99914E-12	0,740999285	0,817	0,361	1,05487E-07	1,67133309	0,598324778	2,263157895
MARCKSL1	1,58504E-12	0,739251417	0,695	0,221	3,34459E-08	1,669309446	0,599050106	3,14479638
LAMTOR2	2,48739E-17	0,739014799	0,585	0,119	5,24863E-13	1,669035682	0,599148365	4,915966387
NIBAN2	1,28044E-08	0,73678107	0,829	0,439	0,000270186	1,66645351	0,600076746	1,888382688
TAF10	2,82578E-17	0,736655101	0,537	0,098	5,96268E-13	1,666308009	0,600129144	5,479591837

Supplementary Table 2

Differentially expressed genes_keloid-XL_ comparing SC-Keloid vs. (SC-Myel, SC-Nonmyel, SC-Nonmyel2)								
**gene**	**p_val**	**avg_log2FC**	**pct.1**	**pct.2**	**p_val_adj**	**Foldchange_UP**	**Foldchange_DOWN**	**Ratio_pct1_pct2**
SELENOF	4,9864E-08	0,734854223	0,756	0,361	0,001052181	1,664229299	0,600878737	2,094182825
COPE	1,1654E-12	0,733691473	0,732	0,253	2,45911E-08	1,662888543	0,601363215	2,893280632
QSOX1	1,22983E-18	0,73038779	0,476	0,06	2,59507E-14	1,659084986	0,602741878	7,933333333
COX8A	3,82673E-10	0,730365782	0,805	0,354	8,07479E-06	1,659059677	0,602751073	2,274011299
ACTG2	1,25399E-12	0,728176638	0,171	0	2,64605E-08	1,656544131	0,603666381	#DIV/0!
TECR	4,4026E-10	0,725601991	0,768	0,347	9,28993E-06	1,653590483	0,604744651	2,213256484
HM13	7,45032E-14	0,721961638	0,524	0,123	1,57209E-09	1,649423237	0,606272531	4,260162602
TMEM47	1,54587E-13	0,721035453	0,634	0,175	3,26194E-09	1,648364677	0,606661872	3,622857143
MGAT4B	5,19197E-12	0,719608199	0,537	0,14	1,09556E-07	1,646734761	0,607262337	3,835714286
HOXB2	2,93499E-17	0,718050187	0,439	0,06	6,19313E-13	1,64495736	0,607918493	7,316666667
TRUB2	1,76436E-26	0,716123514	0,476	0,025	3,72298E-22	1,642762038	0,608730891	19,04
COX7A2	2,02429E-09	0,715442361	0,744	0,358	4,27145E-05	1,641986609	0,609018365	2,078212291
PRDX5	1,00769E-11	0,715429537	0,707	0,274	2,12632E-07	1,641972014	0,609023779	2,580291971
IFI27L2	7,21906E-13	0,715147727	0,683	0,225	1,52329E-08	1,641651308	0,609142755	3,035555556
COX6A1	2,40012E-09	0,714043948	0,841	0,439	5,06449E-05	1,640395792	0,609608976	1,91571754
PFKFB4	8,10509E-25	0,710157351	0,439	0,021	1,71026E-20	1,63598254	0,611253467	20,9047619
CTSZ	4,66042E-13	0,709265443	0,671	0,214	9,83395E-09	1,63497145	0,611631475	3,135514019
MYDGF	1,96692E-11	0,707284519	0,634	0,225	4,1504E-07	1,632728056	0,612471866	2,817777778
MICOS10	3,76744E-12	0,70609852	0,695	0,232	7,94968E-08	1,631386389	0,612975569	2,995689655
CTDNEP1	8,74844E-16	0,705950632	0,671	0,175	1,84601E-11	1,631219166	0,613038408	3,834285714
ECE1	2,66522E-10	0,704791082	0,695	0,26	5,62388E-06	1,629908619	0,613531328	2,673076923
ACTN1	3,08625E-14	0,703331291	0,744	0,228	6,51229E-10	1,628260231	0,614152444	3,263157895
NDUFA1	5,86662E-09	0,70247871	0,78	0,393	0,000123792	1,627298272	0,614515493	1,984732824
NDUFA7	2,19295E-15	0,700904691	0,537	0,109	4,62735E-11	1,625523813	0,615186312	4,926605505
MAZ	1,66128E-11	0,700585583	0,671	0,232	3,50546E-07	1,625164305	0,6153224	2,892241379
PGAM1	1,76128E-11	0,699630518	0,634	0,214	3,71648E-07	1,624088802	0,615729878	2,962616822
NDUFA11	2,98651E-11	0,697102675	0,768	0,302	6,30184E-07	1,621245618	0,616809686	2,543046358
AGRN	7,7145E-14	0,696062482	0,695	0,196	1,62784E-09	1,62007711	0,61725457	3,545918367
RPLP2	3,8604E-08	0,695895001	0,988	0,877	0,000814583	1,619889048	0,617326231	1,126567845
RPL37A	5,21782E-10	0,69498839	0,988	0,919	1,10101E-05	1,618871405	0,61771429	1,07508161

Supplementary Table 2

Differentially expressed genes_keloid-XL_ comparing SC-Keloid vs. (SC-Myel, SC-Nonmyel, SC-Nonmyel2)								
gene	p_val	avg_log2FC	pct.1	pct.2	p_val_adj	Foldchange_UP	Foldchange_DOWN	Ratio_pct1_pct2
LOXL1	1,24125E-21	0,694176253	0,378	0,018	2,61916E-17	1,617960349	0,618062118	21
C2orf27A	6,70436E-23	0,692020473	0,488	0,042	1,41469E-18	1,615544481	0,618986362	11,61904762
SDC3	7,10678E-08	0,690675708	0,878	0,484	0,001499601	1,614039302	0,6195636	1,814049587
COX6B1	1,20668E-07	0,690071063	0,829	0,498	0,002546219	1,613362986	0,619823319	1,664658635
KRT10	2,41436E-07	0,687743629	0,793	0,481	0,005094545	1,61076232	0,620824058	1,648648649
PSMD13	4,30261E-16	0,687443265	0,537	0,102	9,07893E-12	1,610426999	0,620953325	5,264705882
PLPP4	1,1595E-20	0,687034782	0,305	0,004	2,44667E-16	1,60997109	0,621129166	76,25
YWHAB	4,15882E-10	0,685737104	0,817	0,393	8,77552E-06	1,608523601	0,621688112	2,078880407
PLP2	5,98914E-07	0,684790116	0,524	0,228	0,01263768	1,607468109	0,622096323	2,298245614
CLDN4	1,23006E-16	0,683947094	0,293	0,014	2,59555E-12	1,606529078	0,622459944	20,92857143
HMGN1	6,28396E-09	0,683648011	0,817	0,404	0,000132598	1,606196065	0,622588999	2,022277228
MTPN	3,83693E-14	0,683417485	0,683	0,186	8,0963E-10	1,605939435	0,622688489	3,672043011
MFAP2	4,39331E-19	0,682666618	0,354	0,021	9,27032E-15	1,605103823	0,623012659	16,85714286
NUCB2	2,21931E-10	0,68230636	0,549	0,172	4,68297E-06	1,604703059	0,623168252	3,191860465
COX6C	6,66007E-10	0,681769539	0,829	0,425	1,40534E-05	1,604106067	0,623400173	1,950588235
NDUFB7	5,21249E-13	0,67985351	0,732	0,256	1,09989E-08	1,601977084	0,624228655	2,859375
RPLP1	8,6321E-08	0,679813629	1	0,975	0,001821459	1,6019328	0,624245911	1,025641026
GLRX2	1,21776E-20	0,678821286	0,476	0,053	2,5696E-16	1,600831306	0,62467544	8,981132075
SLC9A3R2	2,55941E-14	0,678474295	0,683	0,2	5,40062E-10	1,600446327	0,624825702	3,415
ADAM19	5,80179E-18	0,676353387	0,512	0,077	1,22424E-13	1,598095238	0,625744935	6,649350649
PDGFC	1,59628E-17	0,673229864	0,537	0,088	3,36831E-13	1,594639006	0,627101178	6,102272727
MRC2	4,68471E-21	0,673226914	0,341	0,011	9,88521E-17	1,594635745	0,627102461	31
CD81	5,66536E-10	0,67300548	0,976	0,775	1,19545E-05	1,594391009	0,62719872	1,259354839
REEP3	1,70628E-10	0,672365952	0,707	0,27	3,60043E-06	1,593684393	0,62747681	2,618518519
NRXN2	5,82173E-09	0,671158209	0,476	0,147	0,000122844	1,592350809	0,628002319	3,238095238
ATP5MJ	4,89408E-10	0,667816745	0,707	0,284	1,0327E-05	1,588666991	0,629458537	2,48943662
LAMTOR4	7,56379E-11	0,66534076	0,72	0,277	1,59604E-06	1,585942824	0,630539755	2,599277978
GLIPR1	1,02223E-22	0,665007624	0,378	0,014	2,15701E-18	1,585576653	0,630685371	27
ST8SIA2	1,51419E-20	0,66462616	0,378	0,021	3,19509E-16	1,585157464	0,630852154	18
TXNDC5	8,78383E-13	0,664167008	0,439	0,088	1,85348E-08	1,584653052	0,631052961	4,988636364

Supplementary Table 2

Differentially expressed genes_keloid-XL_ comparing SC-Keloid vs. (SC-Myel, SC-Nonmyel, SC-Nonmyel2)								
**gene**	**p_val**	**avg_log2FC**	**pct.1**	**pct.2**	**p_val_adj**	**Foldchange_UP**	**Foldchange_DOWN**	**Ratio_pct1_pct2**
ERH	1,57148E-10	0,661121992	0,768	0,316	3,31597E-06	1,581311941	0,632386295	2,430379747
CD99	3,17303E-10	0,659632697	0,902	0,488	6,69541E-06	1,579680394	0,633039445	1,848360656
PRDX4	4,5924E-15	0,659054066	0,598	0,144	9,69043E-11	1,579046948	0,633293394	4,152777778
ATP5MK	1,77669E-08	0,659034752	0,866	0,491	0,0003749	1,579025808	0,633301872	1,763747454
NDUFA3	4,58528E-10	0,658654651	0,561	0,186	9,67539E-06	1,578609844	0,633468747	3,016129032
RHOA	9,45889E-09	0,658515825	0,939	0,66	0,000199592	1,578457947	0,633529707	1,422727273
TAX1BP3	1,09569E-09	0,658142951	0,89	0,421	2,31201E-05	1,578050037	0,633693468	2,114014252
GABARAP	1,62862E-07	0,657588498	0,854	0,551	0,003436543	1,577443681	0,633937054	1,549909256
SHC1	2,09903E-12	0,656252886	0,463	0,098	4,42916E-08	1,575983998	0,634524209	4,724489796
CIAO2B	6,0758E-10	0,65527897	0,756	0,337	1,28205E-05	1,574920461	0,634952701	2,243323442
SEC61G	2,21687E-10	0,654176115	0,756	0,326	4,67782E-06	1,573716988	0,63543827	2,319018405
LAMTOR1	1,09888E-12	0,653664536	0,659	0,196	2,31875E-08	1,573159048	0,635663636	3,362244898
TMA7	6,65353E-09	0,653082031	0,976	0,695	0,000140396	1,572523995	0,635920344	1,404316547
IFI44	2,56014E-13	0,652545731	0,476	0,098	5,40215E-09	1,571939542	0,636156782	4,857142857
CAPG	6,048E-17	0,651020069	0,28	0,011	1,27619E-12	1,570278081	0,636829879	25,45454545
RAB34	2,4802E-13	0,649847427	0,537	0,13	5,23348E-09	1,569002257	0,637347713	4,130769231
COX7A1	3,80433E-12	0,648069477	0,622	0,186	8,02751E-08	1,567069839	0,638133653	3,344086022
DSTN	3,41124E-11	0,647443803	0,805	0,33	7,19806E-07	1,566390372	0,638410461	2,439393939
ITGA3	4,11197E-12	0,644977425	0,585	0,165	8,67666E-08	1,563714818	0,639502797	3,545454545
C7orf50	2,76136E-14	0,643543487	0,622	0,154	5,82675E-10	1,562161366	0,640138735	4,038961039
PRKCSH	5,18498E-13	0,643104678	0,549	0,14	1,09408E-08	1,561686293	0,640333468	3,921428571
RPN1	1,91564E-09	0,642417881	0,549	0,186	4,04219E-05	1,560943027	0,640638372	2,951612903
LMAN2	2,69601E-10	0,642235518	0,573	0,186	5,68885E-06	1,560745729	0,640719357	3,080645161
TGFB1I1	3,13671E-12	0,641895307	0,585	0,168	6,61877E-08	1,560377723	0,640870467	3,482142857
ALDH1A3	1,00185E-15	0,641535618	0,561	0,109	2,11399E-11	1,559988742	0,641030267	5,146788991
ATP5MG	5,8368E-10	0,641301443	0,841	0,446	1,23162E-05	1,559735549	0,641134326	1,885650224
MXRA8	2,47648E-10	0,637009955	0,841	0,365	5,22563E-06	1,555102802	0,643044305	2,304109589
LGALS3BP	5,58853E-09	0,635770536	0,744	0,319	0,000117924	1,553767387	0,643596981	2,332288401
SEC11A	2,62115E-08	0,633959726	0,744	0,354	0,000553088	1,551818387	0,644405304	2,101694915
COX5B	4,70758E-10	0,632851814	0,756	0,33	9,93347E-06	1,550627132	0,644900363	2,290909091

Supplementary Table 2

Differentially expressed genes_keloid-XL_ comparing SC-Keloid vs. (SC-Myel, SC-Nonmyel, SC-Nonmyel2)								
**gene**	**p_val**	**avg_log2FC**	**pct.1**	**pct.2**	**p_val_adj**	**Foldchange_UP**	**Foldchange_DOWN**	**Ratio_pct1_pct2**
NDUFS8	7,94916E-13	0,632420476	0,537	0,144	1,67735E-08	1,550163594	0,645093204	3,729166667
ATP5MC3	6,06014E-11	0,632368137	0,78	0,323	1,27875E-06	1,550107358	0,645116608	2,414860681
DRAP1	5,59097E-10	0,632183877	0,768	0,323	1,17975E-05	1,549909392	0,645199007	2,377708978
PLEC	5,20583E-07	0,629341868	0,793	0,432	0,010984831	1,546859183	0,646471257	1,835648148
MMP24OS	2,95866E-14	0,629279813	0,561	0,126	6,24307E-10	1,546792649	0,646499064	4,452380952
PKN1	7,82344E-11	0,628912401	0,561	0,168	1,65082E-06	1,546398776	0,64666373	3,339285714
CSRP1	1,891E-06	0,628316862	0,744	0,425	0,039902018	1,545760561	0,646930725	1,750588235
CALM1	7,45862E-07	0,627729464	0,89	0,782	0,015738442	1,545131327	0,647194178	1,138107417
GDI1	3,37058E-11	0,627670362	0,646	0,214	7,11226E-07	1,54506803	0,647220692	3,018691589
C17orf49	2,50602E-12	0,626356652	0,598	0,179	5,28796E-08	1,54366174	0,647810316	3,340782123
SPCS1	6,43093E-09	0,626120255	0,756	0,337	0,000135699	1,54340882	0,647916474	2,243323442
COL20A1	1,30395E-06	0,625612827	0,232	0,053	0,027514561	1,542866064	0,648144401	4,377358491
UQCC2	2,3792E-15	0,623959699	0,5	0,095	5,02035E-11	1,541099167	0,648887509	5,263157895
RPL4	7,95406E-08	0,623938165	0,927	0,653	0,001678386	1,541076164	0,648897195	1,419601838
SEMA3C	6,16035E-09	0,623877264	0,854	0,407	0,00012999	1,541011111	0,648924588	2,098280098
MIA	2,41272E-08	0,623496229	0,927	0,575	0,000509108	1,540604163	0,649096	1,612173913
COL18A1	1,15303E-09	0,623479402	1	0,832	2,433E-05	1,540586194	0,649103571	1,201923077
MANF	3,53666E-12	0,62344384	0,561	0,161	7,46271E-08	1,54054822	0,649119571	3,48447205
SFXN3	5,09478E-19	0,623383868	0,415	0,039	1,07505E-14	1,540484182	0,649146555	10,64102564
HEXB	7,66309E-13	0,623132179	0,402	0,074	1,61699E-08	1,540215456	0,649259814	5,432432432
ACTA2	6,88075E-06	0,619089125	0,122	0,014	0,145190748	1,53590515	0,651081872	8,714285714
EDF1	1,08409E-07	0,619060769	0,78	0,463	0,002287539	1,535874962	0,651094669	1,684665227
TMEM14C	6,83141E-14	0,618581828	0,659	0,175	1,4415E-09	1,535365172	0,651310853	3,765714286
PSMB1	7,77843E-09	0,617594884	0,683	0,284	0,000164133	1,534315192	0,651756565	2,404929577
MED4	2,33849E-18	0,6171626	0,585	0,105	4,93444E-14	1,533855525	0,651951885	5,571428571
CXXC5	5,89814E-08	0,616132372	0,829	0,421	0,001244567	1,532760589	0,65241761	1,96912114
ANKH	1,18414E-11	0,615473508	0,415	0,088	2,49866E-07	1,532060753	0,65271563	4,715909091
AP2M1	9,66228E-09	0,613954279	0,671	0,291	0,000203884	1,530448267	0,653403334	2,305841924
NME4	3,16137E-11	0,613485638	0,451	0,112	6,6708E-07	1,529951201	0,653615618	4,026785714
AURKAIP1	6,60256E-15	0,611955161	0,61	0,147	1,39321E-10	1,528329019	0,654309371	4,149659864

Supplementary Table 2

Differentially expressed genes_keloid-XL_ comparing SC-Keloid vs. (SC-Myel, SC-Nonmyel, SC-Nonmyel2)								
**gene**	**p_val**	**avg_log2FC**	**pct.1**	**pct.2**	**p_val_adj**	**Foldchange_UP**	**Foldchange_DOWN**	**Ratio_pct1_pct2**
LIMK1	2,10212E-11	0,611177085	0,28	0,035	4,43569E-07	1,527504981	0,65466235	8
SRPX	1,60128E-11	0,611127358	0,451	0,102	3,37886E-07	1,527452332	0,654684915	4,421568627
KCNN4	2,3339E-17	0,609376803	0,354	0,028	4,92477E-13	1,525600058	0,655479786	12,64285714
NAT14	2,51244E-13	0,609140083	0,549	0,13	5,30151E-09	1,525349755	0,655587347	4,223076923
SLC39A3	1,54109E-18	0,607905844	0,402	0,039	3,25186E-14	1,524045362	0,656148449	10,30769231
PPDPF	3,2218E-07	0,607466333	0,951	0,733	0,006798328	1,523581138	0,656348372	1,297407913
SSC5D	3,17917E-11	0,606635796	0,512	0,137	6,70837E-07	1,522704289	0,65672633	3,737226277
ITGB5	2,92623E-14	0,6063728	0,366	0,049	6,17464E-10	1,522426733	0,656846059	7,469387755
MRPL41	1,87039E-12	0,605410815	0,622	0,186	3,94672E-08	1,521411922	0,657284188	3,344086022
RPS27L	4,03838E-07	0,605221051	0,744	0,379	0,008521381	1,521211817	0,657370649	1,963060686
COX5A	4,89633E-13	0,605197367	0,598	0,168	1,03318E-08	1,521186844	0,657381441	3,55952381
SCG2	1,16201E-17	0,604043249	0,244	0	2,45196E-13	1,519970421	0,65790754	#DIV/0!
ILK	4,36213E-14	0,603988443	0,646	0,175	9,20452E-10	1,519912681	0,657932533	3,691428571
HOXC9	2,04999E-15	0,603300802	0,402	0,053	4,32569E-11	1,519188407	0,658246202	7,58490566
CAPS	1,5959E-08	0,602172809	0,451	0,14	0,00033675	1,518001071	0,658761064	3,221428571
PRSS23	4,28476E-08	0,602124329	0,659	0,298	0,000904126	1,517950062	0,658783201	2,211409396
TMEM59L	6,50115E-11	0,601262226	0,439	0,102	1,37181E-06	1,51704326	0,659176984	4,303921569
KDELR2	6,6943E-08	0,601248568	0,659	0,295	0,001412563	1,517028898	0,659183224	2,233898305
FKBP3	1,12791E-15	0,601026892	0,537	0,109	2,38E-11	1,516795819	0,659284518	4,926605505
PYGB	5,08566E-13	0,600752988	0,439	0,084	1,07313E-08	1,516507873	0,659409699	5,226190476
TADA3	1,31552E-18	0,600520615	0,476	0,06	2,77588E-14	1,516263631	0,659515918	7,933333333
VDAC1	1,95241E-12	0,598651222	0,646	0,204	4,11979E-08	1,514300183	0,660371049	3,166666667
COX4I1	4,68408E-08	0,598528892	0,829	0,477	0,000988389	1,514171787	0,660427046	1,737945493
PRELID1	7,57197E-12	0,597286315	0,598	0,175	1,59776E-07	1,512868209	0,660996109	3,417142857
PSMD8	7,1826E-10	0,597058394	0,61	0,232	1,5156E-05	1,51262922	0,661100544	2,629310345
PPP2R1A	3,75869E-13	0,597011754	0,573	0,144	7,93121E-09	1,51258032	0,661121916	3,979166667
ADARB1	1,76294E-20	0,594217688	0,451	0,042	3,71998E-16	1,509653742	0,662403551	10,73809524
SDF4	2,12483E-12	0,59411538	0,695	0,214	4,4836E-08	1,50954669	0,662450527	3,247663551
UQCRH	1,50106E-08	0,593497304	0,805	0,393	0,000316738	1,508900112	0,662734393	2,048346056
GLDC	9,28997E-16	0,592446609	0,305	0,021	1,96028E-11	1,507801601	0,663217229	14,52380952

Supplementary Table 2

Differentially expressed genes_keloid-XL_ comparing SC-Keloid vs. (SC-Myel, SC-Nonmyel, SC-Nonmyel2)								
**gene**	**p_val**	**avg_log2FC**	**pct.1**	**pct.2**	**p_val_adj**	**Foldchange_UP**	**Foldchange_DOWN**	**Ratio_pct1_pct2**
TM9SF2	9,73185E-11	0,592295806	0,5	0,133	2,05352E-06	1,507644001	0,663286558	3,759398496
PSMD1	2,10772E-14	0,592049845	0,476	0,091	4,4475E-10	1,507386988	0,66339965	5,230769231
ATP5PD	1,8292E-08	0,592022382	0,683	0,281	0,00038598	1,507358294	0,663412278	2,430604982
NME1	7,64002E-12	0,591762774	0,573	0,158	1,61212E-07	1,507087075	0,663531667	3,626582278
KIFC3	1,15306E-15	0,590936208	0,488	0,084	2,43308E-11	1,506223865	0,663911935	5,80952381
HMCN1	3,62547E-14	0,590319239	0,329	0,035	7,6501E-10	1,505579865	0,664195918	9,4
PIGT	1,3102E-14	0,588925597	0,451	0,077	2,76464E-10	1,504126178	0,664837841	5,857142857
DNAJC15	3,26664E-16	0,588425527	0,585	0,116	6,89294E-12	1,503604905	0,665068328	5,043103448
RPLP0	3,09259E-07	0,588292266	0,988	0,779	0,00652568	1,503466025	0,665129763	1,268292683
PRKAR1B	1,36386E-15	0,587339942	0,402	0,053	2,87788E-11	1,502473913	0,66556896	7,58490566
C19orf53	3,8239E-07	0,587223689	0,695	0,326	0,008068812	1,502352848	0,665622594	2,13190184
SUMO2	5,01602E-09	0,587136752	0,951	0,628	0,000105843	1,502262318	0,665662706	1,51433121
MIF	5,70801E-05	0,586233174	0,878	0,618	1	1,501321727	0,666079749	1,420711974
SNRPF	1,58147E-07	0,585352045	0,622	0,267	0,003337053	1,500405072	0,666486683	2,329588015
UBA52	5,83903E-09	0,584225052	0,963	0,758	0,000123209	1,499233455	0,667007527	1,270448549
NAA10	8,16659E-10	0,583463941	0,598	0,2	1,72323E-05	1,498442725	0,667359508	2,99
NAA38	3,93773E-09	0,583450892	0,598	0,218	8,309E-05	1,498429173	0,667365544	2,743119266
SNF8	1,40908E-13	0,58251623	0,5	0,109	2,97329E-09	1,497458717	0,667798043	4,587155963
NENF	4,31259E-10	0,581915889	0,72	0,263	9,09999E-06	1,496835716	0,668075988	2,737642586
LIMA1	7,06874E-10	0,581696612	0,659	0,246	1,49157E-05	1,496608228	0,668177537	2,678861789
DYRK2	5,24121E-12	0,581535269	0,476	0,109	1,10595E-07	1,496440866	0,668252266	4,366972477
TUSC3	1,30657E-18	0,580786709	0,329	0,018	2,75699E-14	1,49566462	0,668599087	18,27777778
MPG	1,46254E-12	0,579328281	0,634	0,182	3,08612E-08	1,494153408	0,66927532	3,483516484
EIF4EBP1	2,23711E-15	0,578808085	0,341	0,035	4,72053E-11	1,493614755	0,669516685	9,742857143
TGFB1	1,77711E-10	0,578753592	0,634	0,218	3,74988E-06	1,49355834	0,669541974	2,908256881
SPTSSA	1,15662E-06	0,576209363	0,78	0,428	0,024405822	1,490926733	0,670723771	1,822429907
PLPP1	1,34246E-07	0,573718198	0,841	0,54	0,002832731	1,488354506	0,671882939	1,557407407
RPL35	8,40074E-06	0,572877449	0,963	0,842	0,177264096	1,487487401	0,672274602	1,143705463
POMP	1,63041E-08	0,570398594	0,768	0,344	0,000344034	1,484933778	0,673430704	2,23255814
SPNS1	4,00112E-18	0,570180456	0,378	0,032	8,44275E-14	1,48470927	0,673532536	11,8125

Supplementary Table 2

Differentially expressed genes_keloid-XL_ comparing SC-Keloid vs. (SC-Myel, SC-Nonmyel, SC-Nonmyel2)								
**gene**	**p_val**	**avg_log2FC**	**pct.1**	**pct.2**	**p_val_adj**	**Foldchange_UP**	**Foldchange_DOWN**	**Ratio_pct1_pct2**
DBN1	5,36999E-19	0,569670562	0,524	0,074	1,13312E-14	1,48418462	0,673770626	7,081081081
MRPS21	2,31946E-10	0,567806416	0,732	0,256	4,89429E-06	1,482268103	0,674641786	2,859375
PSMC3	4,33351E-10	0,567671291	0,585	0,186	9,14413E-06	1,482129278	0,674704977	3,14516129
BLOC1S1	1,94053E-10	0,566786287	0,695	0,246	4,0947E-06	1,481220362	0,675118993	2,825203252
SEC61B	7,2489E-07	0,566614247	0,793	0,463	0,0152959	1,481043738	0,675199506	1,712742981
ACTR2	1,33841E-08	0,566468035	0,573	0,207	0,000282418	1,480893648	0,675267938	2,768115942
C14orf132	4,14014E-14	0,566234181	0,268	0,018	8,7361E-10	1,480653621	0,675377405	14,88888889
CHMP2A	8,50003E-12	0,565795532	0,695	0,225	1,79359E-07	1,480203499	0,675582783	3,088888889
PSMC5	1,12901E-11	0,565472684	0,512	0,133	2,38233E-07	1,479872295	0,675733983	3,84962406
SEPTIN2	5,46167E-08	0,564795001	0,878	0,46	0,001152467	1,479177311	0,676051473	1,908695652
DGUOK	1,95944E-12	0,564249296	0,646	0,2	4,13461E-08	1,478617913	0,67630724	3,23
NDUFS2	1,55212E-13	0,563904528	0,5	0,105	3,27512E-09	1,478264602	0,67646888	4,761904762
PFDN5	1,50806E-06	0,563828111	0,963	0,698	0,031821487	1,478186303	0,676504712	1,37965616
PEA15	4,02477E-08	0,562401094	0,78	0,351	0,000849267	1,476724904	0,677174197	2,222222222
CNIH3	6,0734E-21	0,562194378	0,354	0,014	1,28155E-16	1,476513326	0,677271232	25,28571429
DDR1	3,18849E-11	0,56209293	0,561	0,154	6,72803E-07	1,476409504	0,677318859	3,642857143
TXNL4A	1,17528E-14	0,561104405	0,598	0,144	2,47995E-10	1,475398225	0,677783112	4,152777778
TMEM219	1,26521E-12	0,560437557	0,683	0,211	2,66971E-08	1,474716419	0,678096472	3,236966825
ADGRL4	6,63347E-15	0,559663864	0,305	0,025	1,39973E-10	1,473925765	0,678460221	12,2
DNM3	6,65258E-14	0,559565065	0,415	0,067	1,40376E-09	1,47382483	0,678506685	6,194029851
MRPL52	3,68533E-11	0,559452275	0,585	0,175	7,7764E-07	1,473709611	0,678559733	3,342857143
PARK7	3,2398E-09	0,558950233	0,756	0,337	6,83631E-05	1,473196866	0,678795905	2,243323442
CCDC124	7,85015E-14	0,55805039	0,573	0,137	1,65646E-09	1,472278284	0,679219418	4,182481752
EIF6	7,2983E-13	0,557257994	0,512	0,116	1,54001E-08	1,471469862	0,67959258	4,413793103
KRTCAP2	2,52608E-09	0,557078073	0,854	0,428	5,33028E-05	1,471286364	0,679677338	1,995327103
NINJ1	2,51037E-10	0,557063868	0,5	0,14	5,29712E-06	1,471271878	0,679684031	3,571428571
TSPO	6,55783E-09	0,556103814	0,805	0,379	0,000138377	1,470293133	0,680136483	2,124010554
WDR1	1,36259E-08	0,556001928	0,598	0,246	0,00028752	1,470189302	0,680184517	2,430894309
SCAND1	5,15558E-12	0,555741299	0,646	0,204	1,08788E-07	1,469923729	0,680307407	3,166666667
NDUFA6	8,83242E-12	0,55512959	0,634	0,196	1,86373E-07	1,469300607	0,680595921	3,234693878

Supplementary Table 2

Differentially expressed genes_keloid-XL_ comparing SC-Keloid vs. (SC-Myel, SC-Nonmyel, SC-Nonmyel2)								
**gene**	**p_val**	**avg_log2FC**	**pct.1**	**pct.2**	**p_val_adj**	**Foldchange_UP**	**Foldchange_DOWN**	**Ratio_pct1_pct2**
EIF4E2	2,90186E-13	0,555109523	0,39	0,063	6,12322E-09	1,46928017	0,680605388	6,19047619
GIPC1	8,75995E-16	0,553270001	0,549	0,105	1,84844E-11	1,467407945	0,681473754	5,228571429
CAPZB	5,42221E-09	0,552870988	0,744	0,291	0,000114414	1,467002153	0,681662258	2,556701031
PSAP	6,77522E-06	0,551263405	0,976	0,779	0,142963907	1,465368395	0,682422252	1,252888318
TCEA2	6,69401E-15	0,550362822	0,549	0,116	1,4125E-10	1,464453944	0,682848378	4,732758621
MRPL51	1,4604E-08	0,549571103	0,695	0,291	0,00030816	1,463650505	0,683223213	2,388316151
AEBP1	0,000177795	0,549231504	0,707	0,425	1	1,463306013	0,683384057	1,663529412
FHL3	4,44758E-13	0,549088198	0,39	0,067	9,38484E-09	1,463160668	0,683451942	5,820895522
PDAP1	1,59756E-12	0,547798307	0,646	0,186	3,37102E-08	1,461853062	0,684063279	3,47311828
KCNS3	1,81086E-11	0,545829592	0,561	0,154	3,82109E-07	1,459859565	0,684997396	3,642857143
PDCD5	2,16792E-08	0,545788808	0,634	0,256	0,000457452	1,459818296	0,68501676	2,4765625
S100A1	8,21747E-05	0,544803532	0,28	0,102	1	1,458821666	0,685484746	2,745098039
CARD19	1,68739E-11	0,544734633	0,512	0,137	3,56056E-07	1,458751999	0,685517484	3,737226277
PEX10	8,88557E-14	0,54331072	0,378	0,056	1,87494E-09	1,457312949	0,686194411	6,75
GNG10	1,20301E-09	0,542845859	0,695	0,267	2,53847E-05	1,456843453	0,68641555	2,602996255
BANF1	1,42807E-07	0,542657408	0,671	0,323	0,003013378	1,456653167	0,686505218	2,077399381
C5orf15	3,67048E-12	0,541973436	0,561	0,14	7,74507E-08	1,45596274	0,686830763	4,007142857
RPS19BP1	5,7424E-13	0,541144569	0,659	0,186	1,2117E-08	1,455126491	0,687225479	3,543010753
COL9A3	7,17009E-08	0,540357469	0,793	0,396	0,001512961	1,454332825	0,687600515	2,002525253
FAM89B	1,62682E-12	0,539214835	0,488	0,112	3,43276E-08	1,45318143	0,68814532	4,357142857
COPS9	5,84179E-08	0,53807447	0,707	0,33	0,001232676	1,45203323	0,688689473	2,142424242
ARPC4	1,32333E-11	0,53775392	0,585	0,165	2,79236E-07	1,451710641	0,688842509	3,545454545
DIRC3	3,96988E-15	0,536960528	0,207	0	8,37685E-11	1,450912511	0,689221433	#DIV/0!
PIN1	1,1891E-11	0,535894176	0,561	0,154	2,50911E-07	1,449840481	0,689731052	3,642857143
TSC22D1	2,25E-07	0,535824541	0,622	0,267	0,00474772	1,449770503	0,689764344	2,329588015
LRRFIP1	5,76942E-11	0,535149428	0,488	0,126	1,2174E-06	1,449092238	0,690087197	3,873015873
UBL5	7,89652E-09	0,534778672	0,878	0,456	0,000166624	1,448719886	0,690264564	1,925438596
MEF2C	4,58428E-11	0,534583574	0,744	0,263	9,67329E-07	1,448523986	0,690357916	2,828897338
GASK1B	1,0418E-11	0,534554815	0,415	0,088	2,1983E-07	1,448495111	0,690371678	4,715909091
ARPC1B	1,88313E-06	0,533513502	0,817	0,484	0,039735942	1,447449989	0,690870156	1,688016529

Supplementary Table 2

Differentially expressed genes_keloid-XL_ comparing SC-Keloid vs. (SC-Myel, SC-Nonmyel, SC-Nonmyel2)								
**gene**	**p_val**	**avg_log2FC**	**pct.1**	**pct.2**	**p_val_adj**	**Foldchange_UP**	**Foldchange_DOWN**	**Ratio_pct1_pct2**
RRAS	1,60952E-13	0,533220557	0,634	0,165	3,39625E-09	1,447156108	0,691010455	3,842424242
TMEM167A	1,16397E-11	0,532650466	0,573	0,158	2,4561E-07	1,446584367	0,691283566	3,626582278
KRT1	0,111172911	0,532346008	0,159	0,095	1	1,44627912	0,691429466	1,673684211
SRP9	7,91235E-08	0,532301309	0,683	0,277	0,001669584	1,446234311	0,691450889	2,465703971
COL9A2	4,83658E-15	0,532033927	0,329	0,032	1,02057E-10	1,445966299	0,691579051	10,28125
ARPC5	1,07224E-07	0,529745995	0,841	0,463	0,002262542	1,443674996	0,692676678	1,816414687
BUD23	7,95141E-13	0,529397277	0,512	0,116	1,67783E-08	1,443326083	0,692844127	4,413793103
COX7A2L	7,92649E-10	0,529375538	0,524	0,168	1,67257E-05	1,443304335	0,692854567	3,119047619
ARHGAP21	1,56178E-13	0,528267294	0,512	0,112	3,2955E-09	1,442196048	0,693387006	4,571428571
HINT2	4,56861E-15	0,528042076	0,573	0,123	9,64023E-11	1,441970926	0,693495258	4,658536585
CSTB	3,28345E-10	0,526738906	0,683	0,242	6,9284E-06	1,440668998	0,694121968	2,82231405
MRPL20	1,23115E-10	0,526575908	0,549	0,161	2,59785E-06	1,440506238	0,694200395	3,409937888
PSMB7	1,29376E-09	0,525895094	0,512	0,161	2,72996E-05	1,439826617	0,694528069	3,180124224
CTXN1	4,26618E-21	0,525026139	0,293	0	9,00206E-17	1,438959651	0,694946519	#DIV/0!
SIL1	2,14463E-13	0,524462775	0,463	0,091	4,52538E-09	1,438397855	0,695217944	5,087912088
GPAA1	6,10336E-11	0,524438082	0,512	0,137	1,28787E-06	1,438373237	0,695229843	3,737226277
DNASE2	3,24039E-05	0,524174559	0,159	0,032	0,683754475	1,438110526	0,695356846	4,96875
MZT2B	2,9296E-07	0,524061988	0,829	0,442	0,006181748	1,437998318	0,695411106	1,875565611
TRAPPC1	6,49233E-17	0,522679097	0,5	0,081	1,36995E-12	1,436620589	0,69607801	6,172839506
SELENOS	6,28427E-10	0,521886388	0,598	0,196	1,32604E-05	1,435831435	0,696460584	3,051020408
SEC61A1	3,29148E-10	0,520830034	0,451	0,119	6,94535E-06	1,434780491	0,696970726	3,789915966
CNPY2	5,56976E-08	0,520229637	0,756	0,358	0,001175274	1,434183512	0,69726084	2,111731844
RNF128	2,28785E-19	0,518887085	0,317	0,011	4,82759E-15	1,432849502	0,697910003	28,81818182
COX17	2,82427E-08	0,518748309	0,549	0,207	0,000595949	1,432711679	0,69797714	2,652173913
CRIP1	1,45302E-05	0,518731267	0,707	0,372	0,306600939	1,432694755	0,697985385	1,900537634
EVA1A	2,88804E-17	0,518590616	0,256	0,004	6,09406E-13	1,432555086	0,698053436	64
CHID1	1,22162E-10	0,517729989	0,427	0,098	2,57775E-06	1,431700763	0,698469978	4,357142857
RPS19	0,001260254	0,517402633	0,976	0,888	1	1,431375938	0,698628483	1,099099099
FAM20C	1,72842E-10	0,515518518	0,439	0,109	3,64714E-06	1,429507825	0,699541466	4,027522936
CTNNB1	3,90452E-07	0,515492099	0,744	0,368	0,008238918	1,429481649	0,699554276	2,02173913

Supplementary Table 2

Differentially expressed genes_keloid-XL_ comparing SC-Keloid vs. (SC-Myel, SC-Nonmyel, SC-Nonmyel2)								
**gene**	**p_val**	**avg_log2FC**	**pct.1**	**pct.2**	**p_val_adj**	**Foldchange_UP**	**Foldchange_DOWN**	**Ratio_pct1_pct2**
TAPBP	8,09367E-07	0,515351845	0,671	0,288	0,017078443	1,429342686	0,699622288	2,329861111
COMMD7	2,67117E-13	0,514751182	0,402	0,07	5,63643E-09	1,428747706	0,699913635	5,742857143
PSMA7	2,44056E-08	0,514630208	0,768	0,368	0,000514983	1,428627906	0,699972327	2,086956522
UBE2D1	1,91763E-14	0,514229348	0,39	0,056	4,04639E-10	1,42823101	0,700166845	6,964285714
DCTN3	5,59295E-11	0,513982177	0,537	0,151	1,18017E-06	1,427986338	0,700286812	3,556291391
ERP29	1,88922E-09	0,513632517	0,537	0,168	3,98645E-05	1,427640285	0,700456558	3,196428571
POLR2G	3,74787E-13	0,513626076	0,573	0,137	7,90838E-09	1,427633912	0,700459685	4,182481752
CTTN	1,14731E-10	0,513599237	0,744	0,263	2,42093E-06	1,427607353	0,700472716	2,828897338
HIGD1A	2,80706E-10	0,513149312	0,512	0,144	5,92317E-06	1,427162203	0,700691203	3,555555556
NRBF2	1,36426E-11	0,513097178	0,402	0,081	2,87872E-07	1,42711063	0,700716524	4,962962963
ARPC1A	8,37722E-13	0,512503631	0,549	0,14	1,76768E-08	1,426523616	0,701004869	3,921428571
SCUBE3	7,88467E-10	0,512503073	0,146	0,004	1,66374E-05	1,426523064	0,70100514	36,5
ESD	4,86998E-10	0,512318209	0,622	0,211	1,02762E-05	1,426340284	0,701094971	2,947867299
MXRA5	1,75288E-13	0,511887929	0,293	0,028	3,69875E-09	1,425914945	0,701304102	10,46428571
HOXB7	1,1487E-16	0,511868809	0,39	0,042	2,42388E-12	1,425896048	0,701313396	9,285714286
MLF2	2,21824E-07	0,511733047	0,549	0,221	0,004680706	1,425761874	0,701379395	2,484162896
SCARB2	6,33607E-06	0,511254722	0,793	0,435	0,133697317	1,425289241	0,701611976	1,822988506
NPL	1,65879E-21	0,511222474	0,366	0,014	3,50022E-17	1,425257382	0,701627659	26,14285714
MRPL16	9,74773E-13	0,510150787	0,366	0,06	2,05687E-08	1,424199042	0,702149047	6,1
GTF3A	4,61872E-15	0,509432268	0,537	0,105	9,74597E-11	1,423489911	0,702498832	5,114285714
ATP5MC2	1,41291E-07	0,509371033	0,878	0,481	0,002981378	1,423429492	0,70252865	1,825363825
MORN2	1,05522E-16	0,509190716	0,439	0,06	2,22662E-12	1,423251594	0,702616462	7,316666667
NDUFB10	4,81236E-09	0,50915747	0,671	0,26	0,000101546	1,423218797	0,702632654	2,580769231
GNG7	1,32712E-14	0,508798875	0,402	0,056	2,80036E-10	1,422865087	0,702807321	7,178571429
BCAP31	1,87259E-09	0,508581298	0,524	0,168	3,95136E-05	1,422650517	0,702913321	3,119047619
CHMP5	4,24196E-07	0,50794282	0,61	0,256	0,008950961	1,422021049	0,703224471	2,3828125
NDUFB11	2,73624E-09	0,507910742	0,72	0,284	5,77373E-05	1,421989431	0,703240107	2,535211268
PSMB10	3,60856E-09	0,507713646	0,293	0,056	7,61441E-05	1,421795177	0,703336188	5,232142857
VKORC1	6,55493E-11	0,507087317	0,683	0,239	1,38316E-06	1,421178055	0,7036416	2,857740586
HYAL2	3,64835E-15	0,502980417	0,354	0,039	7,69837E-11	1,417138161	0,7056475	9,076923077

Supplementary Table 2

Differentially expressed genes_keloid-XL_ comparing SC-Keloid vs. (SC-Myel, SC-Nonmyel, SC-Nonmyel2)								
**gene**	**p_val**	**avg_log2FC**	**pct.1**	**pct.2**	**p_val_adj**	**Foldchange_UP**	**Foldchange_DOWN**	**Ratio_pct1_pct2**
B4GALT2	1,60536E-12	0,502390205	0,329	0,046	3,38747E-08	1,416558522	0,705936242	7,152173913
PSMA6	3,59759E-10	0,502359379	0,598	0,189	7,59128E-06	1,416528256	0,705951326	3,164021164
ITGA1	1,4869E-11	0,501848865	0,72	0,235	3,13751E-07	1,41602709	0,706201179	3,063829787
VAT1	6,75237E-06	0,501749817	0,561	0,232	0,142481733	1,415929875	0,706249665	2,418103448
CLU	0,002328052	0,501705561	0,671	0,421	1	1,415886441	0,70627133	1,593824228
GUSB	2,15211E-11	0,500982571	0,415	0,088	4,54117E-07	1,415177064	0,706625358	4,715909091
MXRA7	3,35389E-12	0,500472244	0,622	0,175	7,07704E-08	1,414676559	0,706875358	3,554285714
SHFL	1,83956E-08	0,500157593	0,427	0,123	0,000388165	1,414368053	0,707029545	3,471544715
MFSD10	4,02367E-12	0,49968058	0,598	0,161	8,49034E-08	1,413900483	0,707263356	3,714285714
MYL6B	1,92335E-13	0,499471484	0,451	0,088	4,05846E-09	1,413695575	0,70736587	5,125
TCEAL4	2,71236E-09	0,49943068	0,707	0,284	5,72335E-05	1,413655592	0,707385877	2,48943662
KCNMB1	3,1272E-20	0,498971382	0,28	0	6,59871E-16	1,413205611	0,707611116	#DIV/0!
P3H1	6,67394E-13	0,498516988	0,476	0,098	1,40827E-08	1,412760575	0,707834022	4,857142857
MYL12B	9,41296E-07	0,498133152	0,878	0,635	0,019862295	1,412384753	0,70802237	1,382677165
ANO6	1,42026E-08	0,498104305	0,39	0,105	0,000299689	1,412356512	0,708036527	3,714285714
EEF1G	1,30791E-05	0,49808317	0,89	0,656	0,275982128	1,412335822	0,7080469	1,356707317
RAB31	8,97243E-09	0,497561939	0,463	0,14	0,000189327	1,411825651	0,708302756	3,307142857
A1BG	1,08688E-12	0,496386875	0,561	0,144	2,29343E-08	1,410676198	0,708879898	3,895833333
NAP1L4	5,27423E-13	0,49350294	0,585	0,147	1,11292E-08	1,407859086	0,71029836	3,979591837
LSM3	1,83954E-09	0,493362954	0,524	0,158	3,88161E-05	1,407722487	0,710367284	3,316455696
SNU13	8,71124E-08	0,492874326	0,78	0,351	0,001838159	1,407245784	0,71060792	2,222222222
GNAS	4,74842E-06	0,491775787	0,988	0,839	0,100196424	1,406174646	0,711149218	1,177592372
MARVELD1	1,47809E-09	0,491036866	0,659	0,225	3,11892E-05	1,405454614	0,711513549	2,928888889
PSMB8	1,48167E-10	0,49062524	0,463	0,119	3,12648E-06	1,405053671	0,711716585	3,890756303
ACP1	1,70738E-13	0,489525501	0,5	0,109	3,60275E-09	1,403983033	0,71225932	4,587155963
YIF1A	5,47486E-12	0,489076557	0,488	0,116	1,15525E-07	1,403546204	0,712480998	4,206896552
RAB1B	2,25502E-11	0,487202974	0,512	0,137	4,75832E-07	1,401724645	0,713406876	3,737226277
SNRPE	8,93909E-09	0,486812393	0,585	0,207	0,000188624	1,401345207	0,713600043	2,826086957
LMF2	7,37157E-11	0,486741682	0,488	0,126	1,55547E-06	1,401276524	0,71363502	3,873015873
EMC7	6,7997E-10	0,486226881	0,671	0,232	1,4348E-05	1,400776593	0,713889713	2,892241379

Supplementary Table 2

Differentially expressed genes_keloid-XL_ comparing SC-Keloid vs. (SC-Myel, SC-Nonmyel, SC-Nonmyel2)								
**gene**	**p_val**	**avg_log2FC**	**pct.1**	**pct.2**	**p_val_adj**	**Foldchange_UP**	**Foldchange_DOWN**	**Ratio_pct1_pct2**
CHPF2	4,74931E-15	0,485190225	0,329	0,032	1,00215E-10	1,399770419	0,714402867	10,28125
PSMB9	3,97203E-10	0,484264461	0,439	0,112	8,38137E-06	1,398872487	0,71486144	3,919642857
SSBP1	8,78868E-09	0,484042822	0,561	0,196	0,00018545	1,398657597	0,714971271	2,862244898
GNA11	5,93049E-08	0,483464345	0,427	0,126	0,001251394	1,39809689	0,715258011	3,388888889
HDAC9	2,80761E-13	0,482129352	0,256	0,018	5,92433E-09	1,396803764	0,715920178	14,22222222
PRKACB	1,17895E-16	0,481978327	0,402	0,046	2,48769E-12	1,396657551	0,715995126	8,739130435
GET3	3,20665E-13	0,481968101	0,427	0,081	6,76635E-09	1,396647651	0,716000202	5,271604938
PTPRK	4,85419E-10	0,481777671	0,439	0,112	1,02428E-05	1,396463311	0,716094717	3,919642857
HOXC8	1,10755E-10	0,481544929	0,268	0,035	2,33705E-06	1,396238046	0,71621025	7,657142857
PLD3	2,74054E-07	0,481226655	0,537	0,2	0,005782814	1,395930054	0,716368271	2,685
MIR4435-2HG	4,1431E-13	0,480454277	0,378	0,06	8,74236E-09	1,395182914	0,716751897	6,3
COQ4	9,12065E-14	0,480104191	0,476	0,091	1,92455E-09	1,394844398	0,716925846	5,230769231
TOMM22	8,88293E-13	0,479663843	0,415	0,077	1,87439E-08	1,39441872	0,717144704	5,38961039
SMARCB1	1,67148E-13	0,479056253	0,488	0,102	3,52698E-09	1,393831585	0,717446793	4,784313725
ITGA2	3,48186E-07	0,478728959	0,415	0,133	0,007347078	1,393515412	0,717609573	3,120300752
APEX1	1,12247E-09	0,476706152	0,622	0,2	2,36852E-05	1,39156293	0,718616441	3,11
PSMB6	2,99536E-09	0,476420689	0,549	0,189	6,32051E-05	1,391287611	0,718758646	2,904761905
PPP4C	2,12934E-12	0,476260049	0,488	0,112	4,49312E-08	1,391132704	0,718838682	4,357142857
MMP11	1,15746E-13	0,475709118	0,22	0,007	2,44235E-09	1,390601564	0,719113242	31,42857143
KDELR3	1,44822E-21	0,475577837	0,366	0,014	3,05588E-17	1,39047503	0,719178682	26,14285714
GTF2A2	7,62807E-09	0,475050924	0,61	0,218	0,00016096	1,389967282	0,719441395	2,798165138
SH3GL1	6,10057E-11	0,47468022	0,488	0,126	1,28728E-06	1,389610172	0,719626281	3,873015873
LIMD2	2,03339E-16	0,474013489	0,354	0,032	4,29067E-12	1,388968122	0,719958928	11,0625
RCN3	8,04841E-10	0,472955871	0,354	0,074	1,6983E-05	1,387950264	0,720486912	4,783783784
ATP6AP1	3,58416E-09	0,472679843	0,537	0,172	7,56294E-05	1,387684736	0,720624775	3,122093023
RPS18	0,000107073	0,472495927	0,988	0,954	1	1,387507843	0,720716647	1,035639413
NOP10	5,08831E-10	0,472136269	0,598	0,207	1,07368E-05	1,387161986	0,720896341	2,888888889
CCDC167	1,4636E-13	0,47199398	0,366	0,053	3,08835E-09	1,387025181	0,720967444	6,905660377
SDF2L1	3,36549E-11	0,471706117	0,402	0,088	7,10152E-07	1,386748454	0,721111314	4,568181818
ATP5F1C	2,52329E-07	0,471339984	0,646	0,288	0,005324396	1,386396564	0,721294344	2,243055556

Supplementary Table 2

Differentially expressed genes_keloid-XL_ comparing SC-Keloid vs. (SC-Myel, SC-Nonmyel, SC-Nonmyel2)								
**gene**	**p_val**	**avg_log2FC**	**pct.1**	**pct.2**	**p_val_adj**	**Foldchange_UP**	**Foldchange_DOWN**	**Ratio_pct1_pct2**
PFDN4	1,53368E-07	0,470721765	0,439	0,144	0,003236208	1,385802597	0,721603497	3,048611111
STRAP	7,6657E-07	0,470126616	0,549	0,204	0,016175385	1,385231036	0,721901238	2,691176471
COL16A1	1,80022E-07	0,469954967	0,793	0,351	0,003798634	1,385066234	0,721987134	2,259259259
NDUFB8	6,52455E-06	0,469948881	0,671	0,309	0,137674618	1,38506039	0,72199018	2,171521036
WLS	7,09532E-10	0,469878731	0,512	0,144	1,49718E-05	1,384993044	0,722025287	3,555555556
GANAB	1,30973E-10	0,469837342	0,598	0,179	2,76367E-06	1,384953312	0,722046001	3,340782123
GADD45GIP1	7,91896E-09	0,469557075	0,549	0,196	0,000167098	1,384684288	0,722186284	2,801020408
HSPB2	1,40215E-05	0,468906552	0,488	0,211	0,29586708	1,384060063	0,722511997	2,312796209
WDR45	4,64998E-11	0,468661173	0,476	0,119	9,81192E-07	1,383824677	0,722634895	4
VPS28	1,89631E-09	0,468520142	0,707	0,274	4,00141E-05	1,383689408	0,722705539	2,580291971
RUNX1	1,9465E-11	0,468505575	0,61	0,175	4,1073E-07	1,383675437	0,722712837	3,485714286
PARVB	2,12765E-15	0,468498515	0,366	0,042	4,48956E-11	1,383668665	0,722716374	8,714285714
RCN1	1,78926E-07	0,46825288	0,512	0,189	0,003775523	1,383433101	0,722839434	2,708994709
CCDC50	2,87881E-12	0,467735225	0,451	0,098	6,07457E-08	1,382936798	0,723098844	4,602040816
SNX3	5,26077E-06	0,467466605	0,793	0,411	0,111007561	1,382679328	0,723233493	1,929440389
RPL7L1	2,43653E-14	0,467389772	0,5	0,095	5,14133E-10	1,382605694	0,72327201	5,263157895
MELTF	6,08531E-16	0,467369931	0,28	0,014	1,28406E-11	1,38258668	0,723281957	20
SYT11	9,01994E-06	0,467122785	0,573	0,263	0,190329858	1,382349851	0,723405873	2,178707224
C1orf122	2,89591E-11	0,467036979	0,646	0,196	6,11066E-07	1,382267636	0,7234489	3,295918367
RPL39L	1,23196E-15	0,466863294	0,366	0,039	2,59955E-11	1,382101236	0,723536	9,384615385
TRAPPC2L	1,43344E-12	0,465858572	0,402	0,074	3,02471E-08	1,381139047	0,724040061	5,432432432
MDH2	2,31743E-08	0,465642415	0,512	0,175	0,000489001	1,380932129	0,724148551	2,925714286
ENG	3,7574E-11	0,465315481	0,476	0,112	7,92848E-07	1,380619227	0,724312671	4,25
COPS6	1,90267E-11	0,464688314	0,598	0,172	4,01483E-07	1,380019176	0,724627612	3,476744186
VPS35	4,99319E-08	0,463576121	0,415	0,119	0,001053613	1,37895571	0,725186453	3,487394958
SHTN1	3,10104E-08	0,463564268	0,268	0,053	0,00065435	1,378944381	0,725192411	5,056603774
SHISA5	3,92488E-09	0,462790644	0,427	0,116	8,28189E-05	1,378205141	0,725581389	3,681034483
TRAPPC5	7,30031E-13	0,46258145	0,573	0,14	1,54044E-08	1,378005312	0,725686607	4,092857143
LARP7	3,36178E-15	0,462287063	0,512	0,098	7,0937E-11	1,377724155	0,725834701	5,224489796
UAP1	3,53724E-11	0,462189739	0,439	0,102	7,46394E-07	1,377631217	0,725883667	4,303921569

Supplementary Table 2

Differentially expressed genes_keloid-XL_ comparing SC-Keloid vs. (SC-Myel, SC-Nonmyel, SC-Nonmyel2)								
**gene**	**p_val**	**avg_log2FC**	**pct.1**	**pct.2**	**p_val_adj**	**Foldchange_UP**	**Foldchange_DOWN**	**Ratio_pct1_pct2**
PFN2	1,04185E-15	0,461896063	0,476	0,081	2,19841E-11	1,377350814	0,726031444	5,87654321
BOLA3	8,66745E-14	0,461825172	0,415	0,067	1,82892E-09	1,377283135	0,72606712	6,194029851
RBX1	9,65093E-07	0,461705977	0,854	0,481	0,020364437	1,377169349	0,726127111	1,775467775
ZNF428	3,02824E-09	0,460721245	0,793	0,316	6,38988E-05	1,376229664	0,726622908	2,509493671
FHL2	3,71441E-17	0,460678932	0,354	0,028	7,83778E-13	1,3761893	0,72664422	12,64285714
SUMO1	1,12111E-09	0,46043722	0,634	0,221	2,36566E-05	1,37595875	0,726765973	2,868778281
HS3ST1	1,85484E-13	0,460371668	0,268	0,021	3,9139E-09	1,375896231	0,726798996	12,76190476
LRPAP1	3,25121E-05	0,459875186	0,549	0,253	0,686036843	1,375422819	0,727049156	2,169960474
FAM114A1	2,42405E-10	0,459262412	0,585	0,175	5,11499E-06	1,374838743	0,72735803	3,342857143
SURF4	2,13827E-09	0,459046472	0,573	0,186	4,51196E-05	1,374632974	0,727466909	3,080645161
AP1S1	3,06104E-15	0,459010293	0,378	0,046	6,45909E-11	1,374598503	0,727485151	8,217391304
GSDMD	1,29724E-13	0,45895517	0,39	0,06	2,73731E-09	1,374545982	0,727512948	6,5
JTB	1,37655E-08	0,458916897	0,622	0,242	0,000290466	1,374509518	0,727532249	2,570247934
ACTN4	0,000101416	0,457687249	0,732	0,46	1	1,373338485	0,728152608	1,591304348
ATP5PB	5,48016E-12	0,457484416	0,512	0,13	1,15637E-07	1,373145417	0,728254989	3,938461538
EMC3	5,65978E-16	0,45726671	0,451	0,067	1,19427E-11	1,372938222	0,728364892	6,731343284
CANX	9,72965E-06	0,457139145	0,829	0,516	0,205305412	1,37281683	0,728429298	1,606589147
CUTA	5,9655E-07	0,456476296	0,646	0,302	0,012587796	1,372186232	0,728764053	2,139072848
SRI	2,93807E-11	0,456459151	0,622	0,189	6,19961E-07	1,372169925	0,728772714	3,291005291
MAP2K2	2,25452E-08	0,45624651	0,585	0,218	0,000475725	1,371967694	0,728880137	2,683486239
MRPL34	8,00009E-13	0,456121987	0,524	0,119	1,6881E-08	1,37184928	0,728943051	4,403361345
MMP17	2,60465E-08	0,455370927	0,561	0,2	0,000549607	1,371135288	0,729322634	2,805
COL11A1	1,31883E-09	0,454988657	0,268	0,042	2,78287E-05	1,370772028	0,729515908	6,380952381
ELOF1	2,77951E-12	0,454771559	0,439	0,091	5,86505E-08	1,370565768	0,729625694	4,824175824
PHC2	1,01133E-11	0,454018035	0,61	0,175	2,13401E-07	1,369850105	0,730006879	3,485714286
RPS10	0,001221381	0,453424021	0,939	0,747	1	1,369286199	0,730307514	1,257028112
PSMB4	7,37789E-09	0,45305206	0,671	0,256	0,000155681	1,368933211	0,730495829	2,62109375
PGAP6	3,36806E-11	0,452908326	0,341	0,06	7,10694E-07	1,368796833	0,730568611	5,683333333
SNRPC	6,14175E-15	0,452074773	0,415	0,06	1,29597E-10	1,368006204	0,730990837	6,916666667
ADH5	1,47209E-11	0,451314528	0,463	0,109	3,10626E-07	1,367285507	0,731376143	4,247706422

Supplementary Table 2

Differentially expressed genes_keloid-XL_ comparing SC-Keloid vs. (SC-Myel, SC-Nonmyel, SC-Nonmyel2)								
gene	p_val	avg_log2FC	pct.1	pct.2	p_val_adj	Foldchange_UP	Foldchange_DOWN	Ratio_pct1_pct2
LYRM4	1,83667E-11	0,451271357	0,39	0,077	3,87556E-07	1,367244593	0,731398029	5,064935065
SH3BGR	3,12104E-12	0,450848719	0,415	0,081	6,5857E-08	1,366844117	0,731612323	5,12345679
FABP7	2,12165E-13	0,449140099	0,268	0,021	4,4769E-09	1,365226287	0,732479304	12,76190476
DPM2	3,74157E-11	0,448980231	0,415	0,091	7,89509E-07	1,365075013	0,732560475	4,56043956
NOTCH3	1,15746E-13	0,448775385	0,22	0,007	2,44235E-09	1,364881201	0,732664498	31,42857143
ECHS1	9,22507E-09	0,447854471	0,427	0,116	0,000194658	1,364010236	0,733132328	3,681034483
AUP1	1,08097E-12	0,446410053	0,537	0,13	2,28096E-08	1,36264528	0,733866704	4,130769231
MVP	2,60569E-10	0,446140408	0,439	0,109	5,49826E-06	1,362390621	0,734003879	4,027522936
HYI	8,5149E-11	0,445765339	0,488	0,126	1,79673E-06	1,362036475	0,734194728	3,873015873
ST3GAL4	9,2128E-11	0,443649763	0,463	0,112	1,94399E-06	1,360040641	0,735272145	4,133928571
AGPAT2	1,9604E-15	0,443438303	0,451	0,07	4,13665E-11	1,35984131	0,735379924	6,442857143
FKBP8	5,29979E-07	0,443303523	0,683	0,302	0,011183078	1,359714277	0,735448628	2,261589404
SLC35B2	2,38506E-13	0,442573205	0,439	0,081	5,03271E-09	1,359026139	0,73582102	5,419753086
SNX17	2,63969E-12	0,44251757	0,439	0,091	5,57001E-08	1,358973732	0,735849396	4,824175824
ADAMTS2	1,15969E-12	0,441834206	0,28	0,028	2,44707E-08	1,358330177	0,73619803	10
POLR2I	6,58955E-11	0,441796882	0,561	0,158	1,39046E-06	1,358295035	0,736217077	3,550632911
UCN2	2,91183E-16	0,441723624	0,256	0,007	6,14425E-12	1,358226066	0,736254461	36,57142857
NDUFS6	1,87151E-08	0,440870441	0,622	0,232	0,000394907	1,357423073	0,736689997	2,681034483
CDIPT	2,99691E-09	0,440800981	0,415	0,105	6,32379E-05	1,35735772	0,736725467	3,952380952
FAM204A	3,80022E-09	0,4406657	0,488	0,147	8,01884E-05	1,357230447	0,736794552	3,319727891
SNRPD2	4,24574E-07	0,439530258	0,671	0,302	0,008958941	1,356162689	0,737374659	2,221854305
PRAF2	1,48875E-11	0,439255672	0,378	0,074	3,14141E-07	1,355904596	0,737515016	5,108108108
DAZAP2	3,50249E-09	0,43883576	0,61	0,204	7,39059E-05	1,355510003	0,737729709	2,990196078
CAPN2	1,9946E-08	0,438320019	0,463	0,144	0,000420881	1,355025516	0,737993483	3,215277778
SRP19	6,57128E-08	0,438061599	0,476	0,158	0,001386606	1,354782821	0,738125686	3,012658228
PLEKHG1	8,18343E-10	0,437534974	0,451	0,119	1,72679E-05	1,354288378	0,738395172	3,789915966
PIN4	6,82474E-10	0,437479301	0,5	0,14	1,44009E-05	1,354236117	0,738423667	3,571428571
MSRB2	3,29211E-09	0,436318546	0,305	0,06	6,94669E-05	1,353146972	0,739018023	5,083333333
UQCRC1	7,13739E-16	0,436193106	0,5	0,084	1,50606E-11	1,353029323	0,739082282	5,952380952
RPS17	0,000289034	0,436019825	0,902	0,611	1	1,352866821	0,739171058	1,476268412

Supplementary Table 2

Differentially expressed genes_keloid-XL_ comparing SC-Keloid vs. (SC-Myel, SC-Nonmyel, SC-Nonmyel2)								
gene	p_val	avg_log2FC	pct.1	pct.2	p_val_adj	Foldchange_UP	Foldchange_DOWN	Ratio_pct1_pct2
RPS6	3,30444E-05	0,435780557	0,976	0,874	0,697270777	1,35264247	0,739293658	1,116704805
DNM1	1,74966E-16	0,435566309	0,305	0,018	3,69196E-12	1,35244161	0,739403455	16,94444444
RRAGA	3,37443E-12	0,435124413	0,476	0,105	7,12039E-08	1,352027422	0,739629969	4,533333333
CCDC90B	1,36575E-11	0,434734563	0,463	0,105	2,88187E-07	1,351662122	0,739829861	4,40952381
HSPB11	1,05417E-11	0,434528562	0,439	0,095	2,2244E-07	1,351469133	0,739935508	4,621052632
HIGD2A	1,76145E-11	0,434134229	0,549	0,147	3,71684E-07	1,351099786	0,740137783	3,734693878
SELENOH	1,47518E-08	0,434028872	0,61	0,214	0,000311278	1,351001122	0,740191836	2,85046729
SPPL2A	2,73852E-06	0,433780108	0,829	0,439	0,057785494	1,350768188	0,740319478	1,888382688
MRPL17	2,26948E-17	0,433306182	0,512	0,081	4,78883E-13	1,350324533	0,740562713	6,320987654
DERL2	1,9255E-12	0,433240678	0,378	0,063	4,063E-08	1,350263224	0,740596338	6
RTRAF	4,31432E-06	0,43255821	0,695	0,333	0,091036566	1,349624632	0,740946761	2,087087087
IFI27L1	1,56982E-12	0,432017271	0,341	0,049	3,31247E-08	1,349118685	0,741224631	6,959183673
ZNF827	7,1255E-13	0,431751502	0,329	0,042	1,50355E-08	1,348870177	0,74136119	7,833333333
GPI	4,47709E-09	0,43144558	0,39	0,098	9,44711E-05	1,348584181	0,741518412	3,979591837
FBN1	1,56845E-09	0,431440275	0,415	0,105	3,30959E-05	1,348579222	0,741521139	3,952380952
DAD1	7,36312E-05	0,431134524	0,841	0,582	1	1,348293448	0,741678306	1,445017182
CCPG1	6,05575E-08	0,431128156	0,5	0,175	0,001277824	1,348287496	0,74168158	2,857142857
TTYH3	1,64454E-10	0,430725413	0,329	0,06	3,47014E-06	1,34791116	0,741888657	5,483333333
CCNDBP1	4,59782E-11	0,429062471	0,378	0,074	9,70185E-07	1,346358368	0,742744298	5,108108108
TGOLN2	1,42776E-09	0,427954498	0,72	0,256	3,01271E-05	1,345324776	0,743314936	2,8125
CCDC25	2,83078E-11	0,427573383	0,366	0,067	5,97323E-07	1,344969431	0,743511322	5,462686567
DTYMK	2,90131E-13	0,427339273	0,354	0,049	6,12205E-09	1,344751197	0,743631984	7,224489796
FNBP1L	7,34197E-15	0,427256783	0,329	0,032	1,54923E-10	1,34467431	0,743674504	10,28125
PLXNA4	1,37047E-13	0,427142653	0,341	0,042	2,89183E-09	1,344567938	0,743733337	8,119047619
MRPL54	2,22194E-13	0,426881743	0,476	0,095	4,68851E-09	1,344324796	0,743867853	5,010526316
RASSF8-AS1	1,43596E-10	0,42675849	0,463	0,116	3,03001E-06	1,344209952	0,743931406	3,99137931
CKAP5	5,19236E-08	0,426502704	0,305	0,07	0,00109564	1,343971648	0,744063315	4,357142857
ITFG1	4,58697E-10	0,426288589	0,427	0,105	9,67897E-06	1,3437722	0,744173752	4,066666667
RPL27	3,54524E-05	0,426011171	0,915	0,768	0,74808191	1,343513829	0,744316864	1,19140625
TUBB	0,000201891	0,425772536	0,829	0,625	1	1,343291617	0,744439991	1,3264

Supplementary Table 2

Differentially expressed genes_keloid-XL_ comparing SC-Keloid vs. (SC-Myel, SC-Nonmyel, SC-Nonmyel2)								
**gene**	**p_val**	**avg_log2FC**	**pct.1**	**pct.2**	**p_val_adj**	**Foldchange_UP**	**Foldchange_DOWN**	**Ratio_pct1_pct2**
RAB5C	9,51635E-10	0,425577442	0,622	0,204	2,00805E-05	1,343109978	0,744540668	3,049019608
COPS8	1,60824E-09	0,425399507	0,476	0,137	3,39356E-05	1,342944336	0,744632501	3,474452555
TWF2	3,955E-14	0,424988683	0,329	0,035	8,34544E-10	1,342561971	0,744844575	9,4
SAE1	1,8276E-12	0,424229871	0,39	0,07	3,85641E-08	1,341856012	0,745236442	5,571428571
AKAP12	5,83516E-08	0,424224412	0,451	0,144	0,001231278	1,341850934	0,745239262	3,131944444
NT5DC2	6,57128E-08	0,424204377	0,488	0,154	0,001386606	1,3418323	0,745249611	3,168831169
HPCAL1	2,03487E-05	0,424162191	0,39	0,154	0,429377418	1,341793064	0,745271403	2,532467532
GTF3C6	4,31819E-15	0,424063878	0,439	0,07	9,11181E-11	1,34170163	0,745322192	6,271428571
TMEM147	6,7852E-11	0,423982874	0,573	0,161	1,43174E-06	1,341626299	0,745364041	3,559006211
NT5E	8,96003E-09	0,423361056	0,195	0,021	0,000189066	1,341048167	0,745685371	9,285714286
TBCA	0,000111951	0,423296044	0,78	0,46	1	1,340987737	0,745718975	1,695652174
PLAUR	3,26819E-09	0,423228537	0,476	0,137	6,89621E-05	1,34092499	0,745753869	3,474452555
PGK1	1,48163E-08	0,422896128	0,5	0,158	0,000312639	1,340616066	0,745925717	3,164556962
DNAJC2	4,14681E-10	0,42289475	0,305	0,053	8,75017E-06	1,340614785	0,745926429	5,754716981
RAB5IF	9,94582E-11	0,422572601	0,5	0,133	2,09867E-06	1,340315463	0,746093011	3,759398496
MCAM	0,00017561	0,422434868	0,805	0,533	1	1,340187511	0,746164243	1,510318949
KDELR1	7,28297E-08	0,422330173	0,634	0,256	0,00153678	1,340090259	0,746218393	2,4765625
H2AJ	2,81543E-07	0,421410184	0,78	0,354	0,005940846	1,339235971	0,7466944	2,203389831
COX16	3,03105E-08	0,421330279	0,463	0,147	0,000639581	1,339161799	0,746735757	3,149659864
RBIS	3,34362E-08	0,420691274	0,744	0,333	0,000705537	1,338568783	0,747066578	2,234234234
LZIC	1,25072E-06	0,42059427	0,354	0,109	0,026391379	1,338478783	0,747116811	3,247706422
EIF3M	1,00468E-06	0,420311282	0,415	0,147	0,021199806	1,338216263	0,747263374	2,823129252
ELAVL1	1,65211E-09	0,420104748	0,439	0,119	3,48611E-05	1,3380247	0,747370359	3,68907563
AGA	6,96776E-14	0,419779408	0,366	0,049	1,47027E-09	1,337722997	0,747538917	7,469387755
CMSS1	1,13686E-13	0,419619793	0,415	0,07	2,39889E-09	1,337575005	0,747621626	5,928571429
SYTL2	3,51225E-17	0,419245032	0,402	0,042	7,41121E-13	1,337227595	0,747815857	9,571428571
MYOF	1,24034E-10	0,419220631	0,354	0,07	2,61724E-06	1,337204978	0,747828505	5,057142857
CERCAM	5,92658E-12	0,418946423	0,402	0,074	1,25057E-07	1,336950844	0,747970656	5,432432432
NDUFA2	7,99594E-07	0,418887261	0,463	0,175	0,016872231	1,33689602	0,748001329	2,645714286
PCBP4	7,23097E-07	0,41887374	0,671	0,302	0,015258069	1,33688349	0,74800834	2,221854305

Supplementary Table 2

Differentially expressed genes_keloid-XL_ comparing SC-Keloid vs. (SC-Myel, SC-Nonmyel, SC-Nonmyel2)								
**gene**	**p_val**	**avg_log2FC**	**pct.1**	**pct.2**	**p_val_adj**	**Foldchange_UP**	**Foldchange_DOWN**	**Ratio_pct1_pct2**
DCBLD2	3,44721E-09	0,418668638	0,561	0,179	7,27396E-05	1,336693445	0,748114689	3,134078212
PYURF	4,70625E-08	0,417882147	0,646	0,249	0,000993065	1,33596494	0,748522637	2,59437751
METAP2	4,1807E-07	0,416958547	0,549	0,211	0,008821686	1,335109941	0,749001988	2,601895735
UBE2Q2	2,63542E-10	0,416870969	0,439	0,109	5,561E-06	1,335028896	0,749047457	4,027522936
HMOX2	1,98654E-09	0,416592017	0,39	0,095	4,19179E-05	1,334770788	0,749192303	4,105263158
PSMD14	4,85505E-14	0,415694397	0,451	0,081	1,02446E-09	1,333940574	0,749658583	5,567901235
AP3S1	6,34682E-11	0,414632618	0,463	0,112	1,33924E-06	1,332959196	0,750210511	4,133928571
TCF3	5,03755E-12	0,414464402	0,463	0,102	1,06297E-07	1,332803784	0,75029799	4,539215686
PSME2	1,46499E-08	0,414351195	0,524	0,172	0,000309128	1,332699204	0,750356868	3,046511628
RPL3	0,000873564	0,414325357	0,976	0,905	1	1,332675336	0,750370306	1,078453039
RPE65	6,01275E-14	0,414016633	0,207	0,004	1,26875E-09	1,332390186	0,750530896	51,75
PBXIP1	1,30274E-09	0,413216717	0,305	0,056	2,74892E-05	1,331651635	0,75094715	5,446428571
ABCF2	4,29588E-10	0,412507104	0,268	0,039	9,06473E-06	1,330996802	0,751316606	6,871794872
PSENEN	1,31809E-11	0,412423833	0,451	0,102	2,7813E-07	1,330919979	0,751359973	4,421568627
RPL22L1	0,001021646	0,412339146	0,695	0,396	1	1,330841857	0,751404079	1,755050505
ERI3	1,32346E-09	0,412209075	0,329	0,063	2,79262E-05	1,330721875	0,751471828	5,222222222
PEMT	9,08828E-11	0,411420485	0,305	0,049	1,91772E-06	1,32999469	0,751882701	6,224489796
ERCC1	1,2443E-13	0,411399406	0,537	0,119	2,62559E-09	1,329975257	0,751893687	4,512605042
TRAPPC4	3,34596E-11	0,411375839	0,354	0,063	7,06031E-07	1,329953532	0,75190597	5,619047619
FAM32A	4,34691E-12	0,411303255	0,463	0,098	9,17242E-08	1,329886621	0,7519438	4,724489796
TCF12	1,41726E-11	0,4111948	0,415	0,084	2,99056E-07	1,329786651	0,75200033	4,94047619
COMMD3	9,93322E-12	0,410460809	0,439	0,091	2,09601E-07	1,329110275	0,752383018	4,824175824
ARL2	1,36908E-05	0,410445115	0,622	0,277	0,288889373	1,329095818	0,752391202	2,245487365
ERGIC3	2,16614E-08	0,41013125	0,561	0,2	0,000457078	1,328806697	0,752554907	2,805
ARL2BP	3,72516E-10	0,409993036	0,524	0,151	7,86046E-06	1,3286794	0,752627007	3,470198675
PLEKHG4	5,06939E-14	0,409080452	0,28	0,021	1,06969E-09	1,327839203	0,753103235	13,33333333
RANBP1	6,1262E-10	0,408968856	0,646	0,214	1,29269E-05	1,327736496	0,753161492	3,018691589
TCTN1	2,13666E-11	0,408637767	0,28	0,035	4,50856E-07	1,327431824	0,753334357	8
OCIAD2	9,50765E-13	0,408510498	0,268	0,025	2,00621E-08	1,327314728	0,753400817	10,72
ABI3BP	6,04745E-07	0,408063869	0,256	0,06	0,012760715	1,326903882	0,75363409	4,266666667

Supplementary Table 2

Differentially expressed genes_keloid-XL_ comparing SC-Keloid vs. (SC-Myel, SC-Nonmyel, SC-Nonmyel2)								
**gene**	**p_val**	**avg_log2FC**	**pct.1**	**pct.2**	**p_val_adj**	**Foldchange_UP**	**Foldchange_DOWN**	**Ratio_pct1_pct2**
ERLIN2	6,20937E-05	0,407484102	0,183	0,046	1	1,326370755	0,753937009	3,97826087
GLOD4	4,01678E-16	0,407360537	0,402	0,049	8,4758E-12	1,326257157	0,754001586	8,204081633
ADGRG1	7,46884E-13	0,407176135	0,5	0,109	1,576E-08	1,326087649	0,754097967	4,587155963
SH3BGRL	2,19172E-07	0,406753328	0,732	0,316	0,004624747	1,325699073	0,754319001	2,316455696
LBH	5,24774E-13	0,406745478	0,451	0,091	1,10733E-08	1,325691859	0,754323105	4,956043956
STK32B	1,36E-12	0,406377401	0,256	0,021	2,86973E-08	1,325353677	0,754515581	12,19047619
PGLS	6,35293E-09	0,406376251	0,476	0,14	0,000134053	1,32535262	0,754516183	3,4
IER3IP1	2,21851E-10	0,405628739	0,585	0,175	4,68127E-06	1,324666085	0,754907226	3,342857143
SSB	9,0389E-08	0,405300296	0,695	0,291	0,001907299	1,324364547	0,755079107	2,388316151
PARP3	1,90671E-10	0,405193845	0,171	0,007	4,02334E-06	1,324266831	0,755134824	24,42857143
POLR2J	2,70664E-08	0,405085572	0,561	0,204	0,000571129	1,32416745	0,755191498	2,75
HMGN3	2,56982E-05	0,404929962	0,707	0,368	0,542257973	1,324024632	0,755272958	1,921195652
CAND1	1,20762E-07	0,404715253	0,488	0,165	0,002548191	1,323827599	0,75538537	2,957575758
SWI5	1,61658E-12	0,404619505	0,451	0,091	3,41115E-08	1,323739743	0,755435504	4,956043956
THOC7	3,20723E-08	0,404592345	0,451	0,133	0,000676758	1,323714822	0,755449726	3,390977444
HYOU1	3,41916E-07	0,403417886	0,366	0,105	0,007214765	1,322637661	0,756064968	3,485714286
TPGS2	5,28081E-14	0,402373246	0,305	0,028	1,1143E-09	1,3216803	0,756612624	10,89285714
TUBB6	1,02945E-08	0,40178214	0,476	0,151	0,000217225	1,321138886	0,75692269	3,152317881
TMEM14B	2,75157E-10	0,401039368	0,549	0,158	5,80608E-06	1,320458873	0,757312492	3,474683544
UFC1	2,94865E-10	0,400953526	0,561	0,168	6,22195E-06	1,320380307	0,757357554	3,339285714
TOMM5	1,16748E-07	0,400559093	0,549	0,204	0,002463489	1,320019363	0,757564645	2,691176471
NTN1	1,09013E-06	0,400506585	0,195	0,035	0,023002751	1,319971321	0,757592217	5,571428571
TCEAL8	9,40622E-11	0,400199986	0,488	0,126	1,98481E-06	1,319690833	0,757753236	3,873015873
ATP6AP2	2,68794E-05	0,399990279	0,805	0,46	0,567181546	1,31949902	0,75786339	1,75
ZFAND2B	4,76008E-09	0,39892871	0,293	0,056	0,000100442	1,318528458	0,758421249	5,232142857
NPM3	1,15033E-06	0,398589853	0,195	0,035	0,024273087	1,318218802	0,758599406	5,571428571
ABRACL	3,52113E-12	0,398033395	0,293	0,035	7,42995E-08	1,317710453	0,75889206	8,371428571
NDUFB4	1,45658E-07	0,397499848	0,732	0,33	0,003073524	1,317223219	0,75917277	2,218181818
ITGAE	1,07491E-12	0,397150668	0,451	0,088	2,26817E-08	1,316904446	0,759356537	5,125
C3orf14	7,14958E-12	0,396678151	0,28	0,032	1,50863E-07	1,316473199	0,759605286	8,75

Supplementary Table 2

Differentially expressed genes_keloid-XL_ comparing SC-Keloid vs. (SC-Myel, SC-Nonmyel, SC-Nonmyel2)								
**gene**	**p_val**	**avg_log2FC**	**pct.1**	**pct.2**	**p_val_adj**	**Foldchange_UP**	**Foldchange_DOWN**	**Ratio_pct1_pct2**
ST3GAL2	1,41131E-09	0,396611277	0,22	0,025	2,978E-05	1,316412177	0,759640497	8,8
TMEM59	0,003044102	0,396274846	0,817	0,565	1	1,316105231	0,759817663	1,446017699
POLR2E	2,22008E-08	0,39623364	0,524	0,175	0,00046846	1,316067641	0,759839365	2,994285714
CLNS1A	1,20086E-08	0,395931098	0,427	0,119	0,000253394	1,315791682	0,759998724	3,588235294
CHN1	9,89953E-08	0,395876192	0,341	0,091	0,0020889	1,315741607	0,760027649	3,747252747
ATRAID	4,09648E-08	0,395626919	0,524	0,182	0,000864399	1,315514289	0,76015898	2,879120879
TALDO1	4,05758E-08	0,394451178	0,671	0,274	0,000856189	1,314442632	0,760778733	2,448905109
GARS1	2,54344E-10	0,394255976	0,329	0,06	5,36691E-06	1,314264796	0,760881676	5,483333333
RPL41	0,000563398	0,393398645	1	0,968	1	1,313484016	0,761333969	1,033057851
CCT3	1,19047E-07	0,393341	0,622	0,232	0,002512002	1,313431535	0,76136439	2,681034483
ZNF365	2,72638E-14	0,393202558	0,195	0	5,75293E-10	1,313305503	0,761437455	#DIV/0!
MRPL23	6,8254E-08	0,392922214	0,451	0,144	0,001440228	1,313050327	0,761585431	3,131944444
JMJD8	2,36857E-12	0,392800117	0,415	0,077	4,99792E-08	1,312939207	0,761649888	5,38961039
CXCL12	2,79416E-09	0,392185976	0,585	0,182	5,89595E-05	1,312380421	0,761974184	3,214285714
ACSBG1	4,73042E-11	0,391835881	0,366	0,067	9,98165E-07	1,312061988	0,762159112	5,462686567
NUDT1	6,5086E-10	0,391168288	0,39	0,088	1,37338E-05	1,311454984	0,762511876	4,431818182
SGCE	5,29058E-05	0,390730033	0,756	0,421	1	1,311056658	0,762743543	1,795724466
DNAJB12	1,33823E-09	0,39048736	0,305	0,056	2,8238E-05	1,310836146	0,762871853	5,446428571
DEAF1	4,00323E-11	0,389139158	0,244	0,025	8,44722E-07	1,309611738	0,763585092	9,76
ANP32B	3,61754E-06	0,38909956	0,695	0,33	0,076333779	1,309575793	0,763606051	2,106060606
ADAM15	2,79652E-08	0,389024482	0,268	0,053	0,000590093	1,309507645	0,76364579	5,056603774
ETS2	7,315E-07	0,388866338	0,329	0,095	0,01543538	1,309364109	0,763729503	3,463157895
SRP14	3,14544E-05	0,388803283	0,951	0,73	0,663718538	1,309306882	0,763762884	1,302739726
NABP2	3,33825E-09	0,388073387	0,232	0,032	7,04404E-05	1,308644638	0,764149388	7,25
NDUFC2	6,00762E-08	0,387812777	0,756	0,337	0,001267667	1,308408264	0,764287438	2,243323442
VPS25	1,57423E-15	0,387710106	0,366	0,039	3,32178E-11	1,308315153	0,764341831	9,384615385
EIF4G1	3,95711E-07	0,387525789	0,524	0,193	0,008349907	1,308148015	0,764439489	2,715025907
CDC123	2,99894E-13	0,38740944	0,402	0,067	6,32805E-09	1,308042522	0,764501141	6
FAM136A	7,09342E-12	0,386528939	0,268	0,028	1,49678E-07	1,307244444	0,764967871	9,571428571
SHMT2	2,39317E-12	0,386083736	0,305	0,039	5,04982E-08	1,306841103	0,76520397	7,820512821

Supplementary Table 2

Differentially expressed genes_keloid-XL_ comparing SC-Keloid vs. (SC-Myel, SC-Nonmyel, SC-Nonmyel2)								
**gene**	**p_val**	**avg_log2FC**	**pct.1**	**pct.2**	**p_val_adj**	**Foldchange_UP**	**Foldchange_DOWN**	**Ratio_pct1_pct2**
HOXB-AS1	6,82833E-06	0,385559444	0,134	0,018	0,144084492	1,306366268	0,765482105	7,444444444
RPL10A	0,000954574	0,385475844	0,939	0,832	1	1,30629057	0,765526463	1,128605769
R3HDM4	5,57388E-15	0,385171623	0,439	0,07	1,17614E-10	1,306015141	0,765687907	6,271428571
CST3	0,000154861	0,384992321	0,976	0,811	1	1,305852836	0,765783075	1,203452528
MACF1	2,76528E-06	0,38476674	0,61	0,263	0,058350086	1,305648668	0,765902823	2,319391635
RPL12	0,001213796	0,384761203	0,976	0,905	1	1,305643657	0,765905762	1,078453039
MDP1	1,9252E-13	0,384601558	0,354	0,049	4,06237E-09	1,305499186	0,76599052	7,224489796
SLC52A2	5,59403E-15	0,384504307	0,329	0,032	1,1804E-10	1,305411186	0,766042156	10,28125
JOSD2	6,72195E-11	0,384207875	0,634	0,182	1,4184E-06	1,30514299	0,766199572	3,483516484
ATP5F1A	2,39405E-07	0,383958217	0,598	0,235	0,005051683	1,304917155	0,766332174	2,544680851
MACIR	4,58367E-10	0,383625474	0,341	0,067	9,672E-06	1,304616224	0,766508941	5,089552239
NELFE	2,77699E-14	0,383501528	0,476	0,088	5,85973E-10	1,304504144	0,766574797	5,409090909
RP9	5,41276E-08	0,382412014	0,28	0,06	0,001142147	1,303519364	0,767153928	4,666666667
CKAP4	3,2372E-07	0,382272219	0,329	0,091	0,006830817	1,303393061	0,767228268	3,615384615
CNN2	6,51136E-16	0,382017158	0,293	0,018	1,37396E-11	1,303162648	0,767363922	16,27777778
PEBP1	5,93422E-05	0,381994581	0,951	0,702	1	1,303142254	0,767375931	1,354700855
COPZ1	1,75771E-15	0,381645909	0,427	0,063	3,70895E-11	1,302827348	0,767561413	6,777777778
YIF1B	9,15077E-13	0,381348918	0,293	0,032	1,9309E-08	1,302559177	0,767719439	9,15625
NPTN	3,51221E-10	0,381141909	0,622	0,2	7,41111E-06	1,30237229	0,767829604	3,11
NSMCE1	3,28035E-12	0,380329698	0,366	0,06	6,92187E-08	1,301639284	0,768262	6,1
KCTD13	1,77717E-09	0,380015825	0,256	0,039	3,75001E-05	1,30135613	0,768429162	6,564102564
COMMD6	2,94713E-06	0,37983626	0,841	0,414	0,062187406	1,301194167	0,76852481	2,031400966
SLC39A7	1,82427E-09	0,379767491	0,573	0,182	3,84939E-05	1,301132145	0,768561444	3,148351648
NDUFAF2	3,10031E-08	0,379563759	0,427	0,126	0,000654197	1,300948416	0,768669985	3,388888889
TMEM230	1,25809E-09	0,379540779	0,561	0,172	2,6547E-05	1,300927694	0,768682229	3,261627907
CFAP36	1,00353E-08	0,379508298	0,378	0,098	0,000211755	1,300898406	0,768699535	3,857142857
CNIH1	2,37849E-10	0,379198245	0,5	0,133	5,01886E-06	1,300618856	0,768864756	3,759398496
RNF7	2,57269E-08	0,378691679	0,561	0,193	0,000542863	1,300162257	0,769134771	2,906735751
IMPDH2	2,16186E-10	0,378559863	0,524	0,147	4,56174E-06	1,300043469	0,769205049	3,56462585
PPP1CB	2,65256E-05	0,378552255	0,659	0,326	0,559715858	1,300036614	0,769209105	2,021472393

Supplementary Table 2

Differentially expressed genes_keloid-XL_ comparing SC-Keloid vs. (SC-Myel, SC-Nonmyel, SC-Nonmyel2)								
**gene**	**p_val**	**avg_log2FC**	**pct.1**	**pct.2**	**p_val_adj**	**Foldchange_UP**	**Foldchange_DOWN**	**Ratio_pct1_pct2**
MGST3	2,54898E-07	0,378535275	0,671	0,302	0,005378609	1,300021312	0,769218159	2,221854305
SLC12A4	2,37306E-11	0,3781434	0,427	0,091	5,00739E-07	1,29966824	0,769427127	4,692307692
ANGPTL7	0,01184833	0,37780228	0,183	0,081	1	1,299360974	0,769609077	2,259259259
CPNE2	1,24048E-10	0,377739159	0,354	0,067	2,61754E-06	1,299304125	0,76964275	5,28358209
ATP5ME	0,001006057	0,377593464	0,866	0,604	1	1,299172918	0,769720478	1,433774834
CDC42EP1	3,34521E-08	0,377383205	0,61	0,225	0,000705874	1,29898359	0,769832666	2,711111111
PTPA	1,07931E-10	0,376888534	0,439	0,102	2,27744E-06	1,298538271	0,770096672	4,303921569
ACAP3	2,27189E-08	0,376215718	0,341	0,084	0,000479392	1,297932825	0,770455898	4,05952381
PSMG2	3,62589E-10	0,375770359	0,476	0,123	7,65099E-06	1,297532215	0,770693774	3,869918699
GFUS	2,11938E-08	0,3757419	0,305	0,067	0,000447211	1,29750662	0,770708977	4,552238806
MYO10	1,9211E-07	0,37546675	0,439	0,14	0,004053716	1,297259184	0,77085598	3,135714286
EID1	0,00021199	0,375317486	0,768	0,523	1	1,297124975	0,770935738	1,468451243
RND2	1,59334E-09	0,375295121	0,171	0,011	3,36211E-05	1,297104866	0,77094769	15,54545455
TEN1	2,93496E-14	0,375208699	0,293	0,025	6,19306E-10	1,297027168	0,770993873	11,72
MTX2	3,59032E-13	0,375131377	0,366	0,053	7,57593E-09	1,296957655	0,771035196	6,905660377
SEC23A	3,10586E-08	0,37493207	0,341	0,084	0,000655367	1,296778493	0,771141722	4,05952381
HSPA5	0,000238584	0,374360041	0,89	0,614	1	1,296264422	0,77144754	1,449511401
ANO4	1,03632E-16	0,374061925	0,28	0,011	2,18674E-12	1,295996592	0,771606967	25,45454545
KCND3	5,77944E-14	0,374059298	0,293	0,025	1,21952E-09	1,295994233	0,771608372	11,72
TMEM43	1,07915E-07	0,373772868	0,415	0,126	0,002277108	1,295736954	0,771761581	3,293650794
PSMB5	3,42032E-09	0,37345806	0,561	0,175	7,21722E-05	1,295454244	0,771930004	3,205714286
HOMER3	1,19365E-08	0,373321545	0,366	0,091	0,000251872	1,295331667	0,772003052	4,021978022
GORASP2	7,97067E-08	0,373315966	0,317	0,077	0,001681892	1,295326658	0,772006037	4,116883117
NAA20	3,25453E-15	0,373295359	0,488	0,084	6,86738E-11	1,295308157	0,772017064	5,80952381
PWP1	5,03921E-11	0,373250528	0,378	0,074	1,06332E-06	1,295267906	0,772041055	5,108108108
NDUFAB1	7,03012E-08	0,37315536	0,549	0,196	0,001483426	1,295182466	0,772091984	2,801020408
RRP36	1,45879E-09	0,372696536	0,329	0,067	3,07819E-05	1,294770621	0,772337574	4,910447761
CSNK2B	6,15555E-06	0,372488431	0,622	0,291	0,12988822	1,294583867	0,772448989	2,137457045
SPPL3	2,80165E-11	0,371765266	0,5	0,126	5,91175E-07	1,293935108	0,772836284	3,968253968
PPCS	7,76554E-10	0,371708111	0,366	0,081	1,63861E-05	1,293883846	0,772866902	4,518518519

Supplementary Table 2

Differentially expressed genes_keloid-XL_ comparing SC-Keloid vs. (SC-Myel, SC-Nonmyel, SC-Nonmyel2)								
**gene**	**p_val**	**avg_log2FC**	**pct.1**	**pct.2**	**p_val_adj**	**Foldchange_UP**	**Foldchange_DOWN**	**Ratio_pct1_pct2**
PSMB2	1,95991E-08	0,371643866	0,537	0,179	0,000413561	1,29382623	0,772901319	3
DBI	1,1564E-05	0,371577674	0,78	0,425	0,244012856	1,293766869	0,772936781	1,835294118
SUN2	9,64356E-06	0,371576565	0,561	0,253	0,203488708	1,293765875	0,772937376	2,217391304
SNRPG	1,22603E-05	0,370857411	0,634	0,319	0,258704685	1,293121119	0,773322765	1,987460815
TNIK	1,27282E-12	0,370759302	0,22	0,011	2,68577E-08	1,293033185	0,773375356	20
H3-3A	0,000124538	0,370684905	0,988	0,902	1	1,292966508	0,773415238	1,095343681
CYTH2	2,04829E-10	0,370478738	0,5	0,133	4,32211E-06	1,292781751	0,773525771	3,759398496
RER1	2,16313E-09	0,370346025	0,598	0,189	4,56442E-05	1,292662834	0,77359693	3,164021164
ANXA11	1,94158E-10	0,3703024	0,5	0,133	4,09693E-06	1,292623746	0,773620323	3,759398496
SNRPD3	1,97484E-05	0,370000246	0,561	0,246	0,416710651	1,292353051	0,773782365	2,280487805
BLOC1S2	9,63074E-15	0,369910442	0,415	0,06	2,03218E-10	1,292272608	0,773830532	6,916666667
RNF34	1,52946E-13	0,369908644	0,329	0,039	3,22731E-09	1,292270998	0,773831497	8,435897436
SF3A3	1,5558E-10	0,368483833	0,451	0,109	3,28289E-06	1,290995376	0,774596113	4,137614679
DGCR6L	9,8889E-14	0,36838477	0,415	0,067	2,08666E-09	1,290906733	0,774649303	6,194029851
SLC35A4	9,06611E-09	0,3683794	0,232	0,035	0,000191304	1,290901927	0,774652186	6,628571429
RRBP1	3,69864E-05	0,368339505	0,573	0,277	0,780450311	1,29086623	0,774673608	2,068592058
IGFBP6	1,73673E-12	0,36829483	0,256	0,021	3,66467E-08	1,290826257	0,774697597	12,19047619
IFT20	1,40427E-08	0,368013757	0,378	0,098	0,000296316	1,290574797	0,774848542	3,857142857
ANAPC15	1,18829E-13	0,36765755	0,329	0,039	2,50741E-09	1,290256188	0,775039879	8,435897436
RPL26	0,000641429	0,367457492	0,963	0,94	1	1,290077282	0,775147361	1,024468085
KHDRBS1	5,94858E-07	0,3670908	0,646	0,274	0,012552104	1,289749422	0,775344406	2,357664234
C3orf70	2,28809E-10	0,367065849	0,39	0,084	4,82811E-06	1,289727116	0,775357816	4,642857143
ETFA	8,6709E-08	0,367046105	0,427	0,13	0,001829646	1,289709466	0,775368427	3,284615385
CTHRC1	2,2064E-12	0,366321144	0,341	0,053	4,65572E-08	1,289061543	0,775758151	6,433962264
ABHD14B	1,25282E-08	0,366303377	0,329	0,074	0,000264357	1,289045669	0,775767705	4,445945946
PFDN1	2,13321E-08	0,366177287	0,659	0,249	0,000450128	1,288933013	0,775835509	2,646586345
ELAC2	1,60381E-05	0,365379997	0,171	0,035	0,338420451	1,288220895	0,776264385	4,885714286
ACTR10	7,53068E-11	0,365236345	0,634	0,186	1,58905E-06	1,28809263	0,776341683	3,408602151
IGSF8	7,16569E-07	0,365176335	0,439	0,151	0,015120312	1,288039052	0,776373976	2,907284768
SLC38A10	1,70793E-07	0,36513569	0,366	0,102	0,0036039	1,288002764	0,776395849	3,588235294

Supplementary Table 2

Differentially expressed genes_keloid-XL_ comparing SC-Keloid vs. (SC-Myel, SC-Nonmyel, SC-Nonmyel2)								
**gene**	**p_val**	**avg_log2FC**	**pct.1**	**pct.2**	**p_val_adj**	**Foldchange_UP**	**Foldchange_DOWN**	**Ratio_pct1_pct2**
ARL6IP4	1,30814E-05	0,364367204	0,683	0,34	0,276030552	1,287316862	0,776809525	2,008823529
DLX5	1,85659E-13	0,36434265	0,183	0	3,91758E-09	1,287294952	0,776822746	#DIV/0!
TSEN15	1,86004E-10	0,364290103	0,232	0,025	3,92487E-06	1,287248067	0,776851041	9,28
TMED3	2,25714E-13	0,364274124	0,439	0,081	4,76278E-09	1,287233809	0,776859645	5,419753086
MED10	1,76336E-10	0,364088119	0,488	0,123	3,72086E-06	1,287067858	0,776959811	3,967479675
MRPL57	2,95931E-10	0,363609275	0,512	0,14	6,24445E-06	1,286640739	0,777217734	3,657142857
RPL32	0,001980261	0,363573035	0,988	0,951	1	1,28660842	0,777237258	1,038906414
GSTO1	1,57445E-08	0,36312392	0,512	0,165	0,000332226	1,286207957	0,777479252	3,103030303
LIF	1,57118E-07	0,362877024	0,183	0,025	0,003315355	1,285987861	0,777612317	7,32
RPL13	0,003599224	0,362055281	0,988	0,944	1	1,285255584	0,778055363	1,046610169
DNTTIP1	4,51798E-12	0,361286237	0,293	0,035	9,5334E-08	1,284570648	0,778470224	8,371428571
NDUFS3	2,35169E-10	0,360612767	0,549	0,158	4,96231E-06	1,283971132	0,77883371	3,474683544
CPNE1	9,08402E-08	0,360412848	0,488	0,158	0,001916818	1,283793221	0,778941642	3,088607595
THYN1	4,45665E-11	0,360165638	0,341	0,06	9,40397E-07	1,283573258	0,779075128	5,683333333
MRPL55	2,96501E-12	0,360037479	0,476	0,105	6,25646E-08	1,28345924	0,779144338	4,533333333
SSRP1	1,38408E-07	0,359195233	0,378	0,109	0,002920538	1,282710174	0,779599336	3,467889908
TMEM208	9,33396E-09	0,359152215	0,402	0,109	0,000196956	1,282671927	0,779622582	3,688073394
PSMA1	7,15815E-08	0,358938919	0,598	0,214	0,00151044	1,282482303	0,779737855	2,794392523
DYNLT2B	8,03643E-15	0,358763594	0,329	0,032	1,69577E-10	1,282326458	0,779832619	10,28125
THOP1	3,08453E-10	0,35840444	0,22	0,021	6,50866E-06	1,282007267	0,78002678	10,47619048
LY6E	1,68362E-10	0,358182753	0,524	0,147	3,55261E-06	1,281810287	0,780146649	3,56462585
R3HCC1	1,12983E-12	0,358134998	0,354	0,053	2,38405E-08	1,281767858	0,780172473	6,679245283
CIBAR1	1,6417E-13	0,357959443	0,341	0,042	3,46415E-09	1,281611895	0,780267415	8,119047619
ZSCAN16-AS1	8,78222E-10	0,357951596	0,354	0,074	1,85314E-05	1,281604924	0,780271659	4,783783784
VEGFB	8,76533E-08	0,357705636	0,463	0,151	0,001849572	1,281386447	0,780404696	3,066225166
GLIPR2	1,89974E-15	0,357597028	0,305	0,021	4,00863E-11	1,281289986	0,780463448	14,52380952
DCTD	4,81332E-06	0,357592344	0,305	0,091	0,101565811	1,281285826	0,780465982	3,351648352
RPSA	0,016428521	0,357442625	0,878	0,705	1	1,281152865	0,780546981	1,245390071
UQCRB	6,5046E-05	0,357247659	0,841	0,519	1	1,280979741	0,780652471	1,620423892
BRSK1	3,63881E-07	0,357085739	0,293	0,074	0,00767826	1,280835979	0,780740092	3,959459459

Supplementary Table 2

Differentially expressed genes_keloid-XL_ comparing SC-Keloid vs. (SC-Myel, SC-Nonmyel, SC-Nonmyel2)								
**gene**	**p_val**	**avg_log2FC**	**pct.1**	**pct.2**	**p_val_adj**	**Foldchange_UP**	**Foldchange_DOWN**	**Ratio_pct1_pct2**
INTS1	7,73359E-05	0,356838676	0,268	0,091	1	1,280616653	0,780873806	2,945054945
CENPB	2,4019E-08	0,356637739	0,439	0,13	0,000506824	1,280438303	0,780982573	3,376923077
PDGFA	0,000450537	0,356603664	0,72	0,432	1	1,280408061	0,781001019	1,666666667
COMT	0,000436018	0,35655876	0,768	0,526	1	1,280368208	0,781025328	1,460076046
LTBP1	1,73695E-12	0,356550956	0,268	0,025	3,66514E-08	1,280361282	0,781029553	10,72
KARS1	1,74912E-09	0,356490944	0,305	0,056	3,69082E-05	1,280308024	0,781062042	5,446428571
AGAP2-AS1	3,32985E-08	0,356284937	0,232	0,039	0,000702632	1,280125218	0,781173581	5,948717949
SMARCE1	3,67792E-10	0,355807542	0,598	0,179	7,76077E-06	1,279701688	0,781432118	3,340782123
NAXE	3,07379E-10	0,35565217	0,329	0,06	6,486E-06	1,279563877	0,781516279	5,483333333
DARS1	9,90557E-14	0,35559294	0,354	0,046	2,09017E-09	1,279511345	0,781548365	7,695652174
COPS7A	3,44523E-11	0,35551578	0,317	0,049	7,26978E-07	1,279442915	0,781590166	6,469387755
NTPCR	1,59785E-07	0,355508361	0,28	0,063	0,003371631	1,279436336	0,781594185	4,444444444
WIPF1	1,59969E-07	0,355485846	0,573	0,218	0,003375505	1,279416368	0,781606383	2,628440367
ZSWIM7	3,4598E-13	0,355318434	0,427	0,074	7,30052E-09	1,279267912	0,781697087	5,77027027
EMILIN1	6,62118E-08	0,35523456	0,28	0,06	0,001397136	1,279193542	0,781742533	4,666666667
ATP6V1F	1,89993E-09	0,355101287	0,659	0,225	4,00904E-05	1,279075378	0,781814752	2,928888889
CRISPLD1	1,36536E-06	0,354608144	0,098	0,004	0,028810488	1,278638238	0,782082039	24,5
TUBA1B	0,000209396	0,354127447	0,89	0,814	1	1,278212275	0,782342667	1,093366093
BTBD10	3,20102E-10	0,35402138	0,28	0,042	6,75447E-06	1,278118304	0,782400187	6,666666667
LTBR	1,8675E-09	0,353347152	0,439	0,116	3,94061E-05	1,277521129	0,782765919	3,784482759
CDK5RAP3	6,72492E-09	0,353087275	0,39	0,098	0,000141902	1,277291026	0,782906933	3,979591837
MANBAL	3,86464E-11	0,352986457	0,39	0,077	8,15478E-07	1,277201769	0,782961646	5,064935065
CLTA	8,00289E-07	0,352633968	0,768	0,361	0,016886902	1,276889753	0,783152968	2,127423823
CHCHD1	4,20672E-15	0,352543266	0,378	0,046	8,87661E-11	1,276809478	0,783202206	8,217391304
UBE2L3	8,1892E-07	0,352257398	0,598	0,246	0,017280035	1,276556505	0,783357412	2,430894309
SERPINH1	0,000235943	0,352250937	0,793	0,467	1	1,276550788	0,78336092	1,698072805
CCT8	4,62102E-09	0,352211872	0,598	0,196	9,75081E-05	1,276516223	0,783382132	3,051020408
EPN1	2,43227E-09	0,352063508	0,463	0,126	5,13233E-05	1,276384955	0,783462698	3,674603175
TBCB	1,03624E-06	0,351504042	0,683	0,291	0,021865802	1,275890078	0,783766578	2,347079038
RPL7A	0,002877129	0,35148519	0,963	0,867	1	1,275873406	0,783776819	1,110726644

Supplementary Table 2

Differentially expressed genes_keloid-XL_ comparing SC-Keloid vs. (SC-Myel, SC-Nonmyel, SC-Nonmyel2)								
**gene**	**p_val**	**avg_log2FC**	**pct.1**	**pct.2**	**p_val_adj**	**Foldchange_UP**	**Foldchange_DOWN**	**Ratio_pct1_pct2**
PPWD1	1,0077E-05	0,351297048	0,244	0,067	0,212634949	1,27570703	0,783879038	3,641791045
EIF3I	6,41173E-09	0,350958952	0,598	0,207	0,000135294	1,275408103	0,784062762	2,888888889
ATP6V1E1	1,94871E-08	0,350739739	0,476	0,144	0,000411198	1,275214323	0,784181907	3,305555556
MRPL21	6,8079E-10	0,350628462	0,427	0,105	1,43653E-05	1,275115968	0,784242394	4,066666667
MRPL24	2,07162E-16	0,350149098	0,354	0,032	4,37133E-12	1,274692356	0,784503018	11,0625
ARHGAP1	3,07194E-11	0,349996009	0,378	0,07	6,48211E-07	1,274557101	0,784586268	5,4
CORO1B	9,42556E-11	0,349680528	0,463	0,112	1,98889E-06	1,274278419	0,784757856	4,133928571
PSMC2	8,56761E-09	0,349374633	0,329	0,074	0,000180785	1,274008261	0,784924267	4,445945946
TXNDC15	8,53187E-08	0,349083208	0,463	0,151	0,001800311	1,273750937	0,785082837	3,066225166
RNF8	0,005817772	0,348913117	0,134	0,046	1	1,273600774	0,785175402	2,913043478
PTPRZ1	1,06389E-05	0,348730915	0,829	0,446	0,224491855	1,273439937	0,785274571	1,858744395
HACD3	3,04026E-08	0,348685782	0,329	0,077	0,000641526	1,2734001	0,785299137	4,272727273
RPS25	8,93741E-05	0,348682267	0,976	0,912	1	1,273396997	0,785301051	1,070175439
SLC2A4RG	4,64222E-08	0,347886906	0,439	0,133	0,000979555	1,272695164	0,785734108	3,30075188
EMC4	8,63294E-10	0,347884855	0,561	0,168	1,82164E-05	1,272693355	0,785735225	3,339285714
BTF3L4	8,68784E-10	0,347705338	0,463	0,123	1,83322E-05	1,272535001	0,785833002	3,764227642
LPGAT1	9,9121E-08	0,347582893	0,5	0,175	0,002091552	1,272427002	0,7858997	2,857142857
G6PC3	5,12172E-11	0,347580208	0,354	0,067	1,08073E-06	1,272424634	0,785901163	5,28358209
SURF2	3,61383E-13	0,347552823	0,354	0,049	7,62555E-09	1,272400481	0,785916081	7,224489796
COPA	4,28447E-08	0,347539339	0,451	0,137	0,000904066	1,272388589	0,785923427	3,291970803
RSRC1	0,00164994	0,346865549	0,293	0,126	1	1,271794477	0,786290567	2,325396825
NDUFB1	6,01926E-06	0,346843056	0,695	0,319	0,127012314	1,271774649	0,786302825	2,178683386
MRPS34	2,66702E-07	0,346524592	0,439	0,144	0,005627687	1,271493945	0,786476415	3,048611111
RPS24	8,69121E-05	0,345934594	0,988	0,926	1	1,270974068	0,786798115	1,066954644
TXNDC12	1,25316E-07	0,345864997	0,39	0,116	0,002644288	1,270912756	0,786836072	3,362068966
CTDSP1	3,30747E-11	0,345793013	0,476	0,112	6,97909E-07	1,270849344	0,786875332	4,25
IFNGR2	1,22842E-05	0,345510714	0,585	0,26	0,259209833	1,270600696	0,787029319	2,25
PLTP	6,64447E-07	0,345225247	0,415	0,14	0,014020496	1,270349306	0,787185064	2,964285714
AGTRAP	1,88551E-10	0,345215827	0,329	0,06	3,97862E-06	1,270341011	0,787190204	5,483333333
GPX7	1,38675E-13	0,344980721	0,256	0,018	2,92619E-09	1,270134009	0,787318498	14,22222222

Supplementary Table 2

Differentially expressed genes_keloid-XL_ comparing SC-Keloid vs. (SC-Myel, SC-Nonmyel, SC-Nonmyel2)								
**gene**	**p_val**	**avg_log2FC**	**pct.1**	**pct.2**	**p_val_adj**	**Foldchange_UP**	**Foldchange_DOWN**	**Ratio_pct1_pct2**
SF3B6	2,70482E-06	0,34456436	0,646	0,305	0,057074369	1,269767502	0,78754575	2,118032787
YBX1	0,012922442	0,344554142	0,866	0,66	1	1,269758509	0,787551328	1,312121212
SNW1	1,83542E-07	0,344501554	0,439	0,144	0,003872911	1,269712225	0,787580036	3,048611111
ZCCHC17	5,70089E-10	0,344076741	0,451	0,116	1,20295E-05	1,269338404	0,787811979	3,887931034
TNS3	2,63506E-05	0,344011354	0,646	0,316	0,556024936	1,269280876	0,787847685	2,044303797
C20orf27	3,57903E-11	0,343893681	0,305	0,046	7,55211E-07	1,269177351	0,787911949	6,630434783
SMARCD1	6,27562E-09	0,343882453	0,415	0,109	0,000132422	1,269167474	0,787918081	3,80733945
MT1F	2,11449E-10	0,343799655	0,341	0,063	4,46179E-06	1,269094636	0,787963302	5,412698413
PTOV1	1,96304E-07	0,343679354	0,561	0,211	0,004142206	1,268988816	0,78802901	2,658767773
WDR83OS	3,18087E-06	0,343432617	0,744	0,368	0,067119594	1,268771806	0,788163794	2,02173913
MARS1	2,37167E-08	0,343378108	0,244	0,042	0,000500446	1,268723868	0,788193574	5,80952381
GGCT	1,33067E-09	0,343050063	0,402	0,102	2,80785E-05	1,268435415	0,788372816	3,941176471
TMEM179B	8,53737E-11	0,342997184	0,537	0,144	1,80147E-06	1,268388923	0,788401713	3,729166667
SETD4	8,15897E-07	0,342683476	0,171	0,025	0,017216233	1,268113148	0,788573166	6,84
RNASEH2B	4,20731E-13	0,342665799	0,415	0,074	8,87784E-09	1,26809761	0,788582829	5,608108108
CDC5L	4,30994E-10	0,342651598	0,476	0,123	9,09441E-06	1,268085128	0,788590591	3,869918699
PLEKHO1	8,82356E-10	0,342584574	0,61	0,2	1,86186E-05	1,268026217	0,788627228	3,05
P2RX4	2,13829E-13	0,342536689	0,293	0,028	4,512E-09	1,26798413	0,788653404	10,46428571
HDLBP	1,89319E-05	0,342435917	0,646	0,298	0,399482045	1,267895564	0,788708493	2,167785235
LAMTOR3	1,24737E-05	0,342429043	0,244	0,067	0,263207229	1,267889524	0,788712251	3,641791045
TIMM13	3,10733E-07	0,342156505	0,549	0,211	0,006556776	1,26765003	0,78886126	2,601895735
KCNC2	3,30148E-08	0,342089245	0,122	0,004	0,000696645	1,267590933	0,788898038	30,5
NDUFC1	3,29936E-05	0,34205641	0,768	0,407	0,696197413	1,267562084	0,788915993	1,886977887
DCAF11	6,00386E-07	0,341770987	0,22	0,042	0,012668742	1,267311333	0,789072088	5,238095238
USP22	5,98669E-07	0,341767864	0,622	0,256	0,012632513	1,26730859	0,789073796	2,4296875
OXA1L	1,55701E-10	0,341763481	0,439	0,105	3,28544E-06	1,26730474	0,789076193	4,180952381
SMIM15	2,95982E-15	0,341519038	0,476	0,077	6,24551E-11	1,267090032	0,789209902	6,181818182
ARL14EP	1,14768E-07	0,34141999	0,22	0,039	0,002421718	1,267003043	0,789264087	5,641025641
ASAH1	3,75959E-05	0,341395084	0,598	0,267	0,79331062	1,266981171	0,789277712	2,239700375
ROMO1	3,22129E-06	0,341303993	0,622	0,267	0,067972476	1,266901176	0,789327549	2,329588015

Supplementary Table 2

Differentially expressed genes_keloid-XL_ comparing SC-Keloid vs. (SC-Myel, SC-Nonmyel, SC-Nonmyel2)								
**gene**	**p_val**	**avg_log2FC**	**pct.1**	**pct.2**	**p_val_adj**	**Foldchange_UP**	**Foldchange_DOWN**	**Ratio_pct1_pct2**
PRR16	3,10908E-17	0,341102469	0,256	0,004	6,56046E-13	1,266724221	0,789437814	64
DPCD	2,65887E-14	0,340776697	0,268	0,018	5,61048E-10	1,266438217	0,789616096	14,88888889
SMG9	1,52838E-06	0,340704588	0,256	0,063	0,032250266	1,266374919	0,789655563	4,063492063
GPX8	2,44473E-11	0,340076996	0,439	0,095	5,15862E-07	1,265824149	0,789999149	4,621052632
HDGFL3	3,35472E-06	0,33946123	0,256	0,067	0,070788009	1,265283989	0,790336406	3,820895522
MAGOH	7,67515E-11	0,339392298	0,402	0,088	1,61953E-06	1,265223536	0,790374168	4,568181818
FAM234A	3,46272E-10	0,339194481	0,549	0,161	7,30668E-06	1,265050065	0,790482549	3,409937888
AP2B1	1,312E-08	0,338687174	0,488	0,147	0,000276844	1,264605303	0,790760562	3,319727891
PSMA3	4,82285E-06	0,338305409	0,561	0,232	0,101766859	1,264270708	0,79096984	2,418103448
CLTC	6,71575E-07	0,338028354	0,524	0,2	0,014170896	1,264027941	0,791121753	2,62
PSMA4	4,68324E-09	0,337914124	0,585	0,2	9,88211E-05	1,263927861	0,791184395	2,925
B4GALT7	8,18246E-15	0,337661569	0,305	0,025	1,72658E-10	1,26370662	0,79132291	12,2
POLDIP2	7,73736E-12	0,337606963	0,268	0,028	1,63266E-07	1,263658789	0,791352862	9,571428571
SLC35G1	8,07006E-10	0,337166121	0,146	0,004	1,70286E-05	1,263272714	0,791594712	36,5
LGMN	4,04008E-10	0,336979366	0,354	0,07	8,52497E-06	1,263109196	0,791697189	5,057142857
MYG1	9,04102E-09	0,336972498	0,366	0,088	0,000190775	1,263103183	0,791700958	4,159090909
NDUFAF3	8,40927E-08	0,336965557	0,707	0,284	0,00177444	1,263097106	0,791704767	2,48943662
ANAPC16	2,09508E-08	0,336765746	0,598	0,207	0,000442082	1,262922182	0,791814424	2,888888889
UBXN6	3,41235E-07	0,336342199	0,341	0,095	0,00720041	1,262551467	0,79204692	3,589473684
OSTM1	2,01659E-07	0,336301718	0,366	0,105	0,004255213	1,262516041	0,792069144	3,485714286
FLNC	1,76167E-13	0,33624664	0,232	0,011	3,7173E-09	1,262467843	0,792099384	21,09090909
DIRAS1	1,03738E-15	0,335878643	0,28	0,014	2,18897E-11	1,262145858	0,792301455	20
TAF15	2,10502E-10	0,335561668	0,5	0,13	4,4418E-06	1,261868582	0,792475551	3,846153846
ST3GAL5	1,42955E-10	0,335407947	0,329	0,056	3,01649E-06	1,261734136	0,792559995	5,875
LNPEP	2,6882E-07	0,335177203	0,354	0,098	0,005672372	1,261532351	0,792686767	3,612244898
MRPL37	1,84289E-11	0,334236828	0,341	0,056	3,88867E-07	1,260710329	0,793203623	6,089285714
UCK2	3,04255E-12	0,333986776	0,317	0,042	6,4201E-08	1,260491838	0,793341115	7,547619048
ENO1	0,00016186	0,333905064	0,756	0,411	1	1,260420447	0,79338605	1,839416058
TUFM	2,08268E-07	0,333760597	0,585	0,225	0,00439467	1,260294239	0,793465501	2,6
AXL	4,79221E-08	0,333632285	0,476	0,147	0,001011204	1,260182155	0,793536074	3,238095238

Supplementary Table 2

Differentially expressed genes_keloid-XL_ comparing SC-Keloid vs. (SC-Myel, SC-Nonmyel, SC-Nonmyel2)								
**gene**	**p_val**	**avg_log2FC**	**pct.1**	**pct.2**	**p_val_adj**	**Foldchange_UP**	**Foldchange_DOWN**	**Ratio_pct1_pct2**
LIMS1	1,21835E-06	0,333532017	0,768	0,365	0,025708501	1,260094574	0,793591228	2,104109589
RNF181	6,48082E-09	0,333353179	0,585	0,196	0,000136752	1,259938381	0,793689608	2,984693878
UBE2D4	1,45089E-10	0,33332011	0,268	0,035	3,06152E-06	1,259909502	0,793707801	7,657142857
GRINA	5,22494E-09	0,333082158	0,39	0,098	0,000110251	1,259701715	0,793838722	3,979591837
RNPEPL1	2,50396E-11	0,333054065	0,451	0,102	5,2836E-07	1,259677186	0,79385418	4,421568627
COPZ2	1,07195E-07	0,332756089	0,524	0,172	0,002261917	1,259417037	0,794018161	3,046511628
MAP3K13	2,92492E-07	0,332655003	0,329	0,088	0,006171877	1,259328796	0,794073798	3,738636364
MSRB3	0,000153059	0,332484061	0,159	0,039	1	1,25917959	0,794167892	4,076923077
ZDHHC1	3,86345E-11	0,332034306	0,268	0,032	8,15226E-07	1,258787106	0,794415509	8,375
TTLL7	1,033E-08	0,331841012	0,427	0,116	0,000217973	1,258618463	0,794521953	3,681034483
MTLN	4,19262E-12	0,331534121	0,439	0,091	8,84686E-08	1,258350758	0,794690982	4,824175824
TRIM16	7,01137E-09	0,331452941	0,171	0,014	0,000147947	1,258279953	0,794735701	12,21428571
SHKBP1	1,18356E-09	0,330875831	0,28	0,046	2,49742E-05	1,257776714	0,795053676	6,086956522
COA6	7,56045E-13	0,330575493	0,293	0,032	1,59533E-08	1,257514899	0,795219206	9,15625
NHP2	5,42179E-10	0,330420369	0,463	0,123	1,14405E-05	1,257379694	0,795304716	3,764227642
IDS	3,43545E-06	0,330103095	0,585	0,242	0,072491469	1,257103204	0,795479637	2,417355372
MAGOHB	6,88854E-08	0,330083386	0,268	0,056	0,00145355	1,25708603	0,795490504	4,785714286
EEF1D	0,001127966	0,329958508	0,902	0,646	1	1,256977223	0,795559364	1,39628483
MESD	7,9925E-10	0,329951962	0,476	0,126	1,6865E-05	1,25697152	0,795562973	3,777777778
CFAP410	1,12546E-09	0,329807242	0,415	0,102	2,37483E-05	1,256845437	0,795642782	4,068627451
CERS2	9,71046E-06	0,329368167	0,488	0,193	0,204900375	1,256462982	0,795884968	2,528497409
SNAPIN	1,90747E-09	0,329279876	0,293	0,053	4,02494E-05	1,256386091	0,795933676	5,528301887
C9orf78	2,91172E-13	0,329261861	0,549	0,123	6,14402E-09	1,256370402	0,795943615	4,463414634
CIB1	1,1727E-07	0,328740817	0,537	0,193	0,002474519	1,255916733	0,796231131	2,78238342
MACROD1	2,41166E-08	0,328575207	0,207	0,028	0,000508885	1,255772572	0,796322537	7,392857143
TMEM9B	1,89715E-10	0,328541726	0,488	0,126	4,00318E-06	1,25574343	0,796341017	3,873015873
CDK4	3,93477E-10	0,328440879	0,402	0,091	8,30275E-06	1,255655653	0,796396685	4,417582418
FLOT1	1,00104E-08	0,328320198	0,61	0,207	0,000211229	1,255550623	0,796463306	2,946859903
GLT8D1	1,68611E-08	0,328236755	0,378	0,098	0,000355785	1,255478006	0,796509373	3,857142857
PSMD4	1,05803E-08	0,328232326	0,476	0,144	0,000223255	1,255474152	0,796511819	3,305555556

Supplementary Table 2

Differentially expressed genes_keloid-XL_ comparing SC-Keloid vs. (SC-Myel, SC-Nonmyel, SC-Nonmyel2)								
**gene**	**p_val**	**avg_log2FC**	**pct.1**	**pct.2**	**p_val_adj**	**Foldchange_UP**	**Foldchange_DOWN**	**Ratio_pct1_pct2**
ZCRB1	2,62658E-10	0,328143016	0,415	0,095	5,54235E-06	1,255396434	0,796561128	4,368421053
SMARCA1	5,39766E-06	0,32801307	0,28	0,081	0,113895985	1,255283363	0,796632879	3,456790123
ZNF593	5,78906E-10	0,327858286	0,293	0,049	1,22155E-05	1,255148693	0,796718353	5,979591837
RPL37	0,00309164	0,327553031	0,988	0,958	1	1,254883149	0,796886946	1,03131524
SMS	4,84298E-07	0,327482294	0,451	0,154	0,010219176	1,254821622	0,796926019	2,928571429
SLC39A1	8,13211E-07	0,326864288	0,439	0,151	0,017159564	1,25428421	0,797267471	2,907284768
NUCB1	2,30657E-05	0,326835734	0,598	0,281	0,486708321	1,254259386	0,79728325	2,128113879
IQSEC1	2,94435E-07	0,326805413	0,305	0,077	0,006212871	1,254233025	0,797300007	3,961038961
ABHD17A	5,01626E-07	0,326553794	0,537	0,207	0,010584813	1,254014295	0,797439076	2,594202899
BOLA2B	1,48591E-12	0,326055905	0,28	0,028	3,13541E-08	1,253581596	0,797714327	10
GOLGA2	2,16194E-07	0,326010364	0,524	0,186	0,004561916	1,253542025	0,797739509	2,817204301
POLE4	8,23737E-05	0,325794416	0,317	0,116	1	1,253354405	0,797858927	2,732758621
RALB	6,48827E-12	0,325582942	0,341	0,053	1,36909E-07	1,253170699	0,797975887	6,433962264
UBE2V1	7,64765E-05	0,325438643	0,524	0,253	1	1,253045362	0,798055705	2,071146245
KLHL12	0,170152369	0,325411185	0,049	0,021	1	1,253021514	0,798070894	2,333333333
CD320	3,43946E-11	0,325320865	0,354	0,063	7,2576E-07	1,252943071	0,798120859	5,619047619
SUCLG1	5,51237E-09	0,325206927	0,329	0,07	0,000116317	1,252844122	0,798183894	4,7
SKP1	0,002054185	0,32509735	0,89	0,646	1	1,252748969	0,798244521	1,377708978
PKP1	1,46644E-12	0,324612504	0,256	0,021	3,09434E-08	1,252328028	0,798512832	12,19047619
MIEN1	5,03402E-10	0,324553436	0,537	0,154	1,06223E-05	1,252276756	0,798545526	3,487012987
MEGF8	1,84502E-07	0,323401632	0,366	0,105	0,003893167	1,251277375	0,799183315	3,485714286
SHROOM2	6,01275E-14	0,323250443	0,207	0,004	1,26875E-09	1,251146253	0,79926707	51,75
ANKRD54	1,2439E-09	0,323246981	0,268	0,042	2,62476E-05	1,251143251	0,799268988	6,380952381
CRELD1	2,902E-11	0,32295416	0,329	0,053	6,12352E-07	1,250889334	0,799431231	6,20754717
CCT6A	5,54824E-07	0,322517787	0,537	0,196	0,011707349	1,250511034	0,799673072	2,739795918
RASSF4	3,18559E-07	0,322385811	0,598	0,239	0,006721905	1,250396644	0,799746228	2,50209205
HGSNAT	2,28084E-09	0,322077978	0,415	0,105	4,8128E-05	1,250129871	0,799916891	3,952380952
HOXB6	1,32627E-12	0,321784508	0,232	0,014	2,79856E-08	1,249875598	0,800079625	16,57142857
MRPL3	3,49803E-10	0,32175111	0,293	0,046	7,3812E-06	1,249846664	0,800098147	6,369565217
DYNLT3	1,2777E-06	0,321712657	0,476	0,168	0,026960836	1,249813351	0,800119473	2,833333333

Supplementary Table 2

Differentially expressed genes_keloid-XL_ comparing SC-Keloid vs. (SC-Myel, SC-Nonmyel, SC-Nonmyel2)								
**gene**	**p_val**	**avg_log2FC**	**pct.1**	**pct.2**	**p_val_adj**	**Foldchange_UP**	**Foldchange_DOWN**	**Ratio_pct1_pct2**
B3GALT6	1,00057E-14	0,321433009	0,22	0,004	2,11131E-10	1,249571114	0,800274581	55
SEPTIN7	0,000329591	0,321377484	0,878	0,551	1	1,249523023	0,800305382	1,593466425
SDF2	1,89747E-13	0,32130244	0,463	0,084	4,00384E-09	1,249458028	0,800347012	5,511904762
MRPL4	4,19221E-06	0,321214974	0,341	0,112	0,088459877	1,249382281	0,800395536	3,044642857
SLC35E2B	1,59293E-05	0,321143901	0,22	0,056	0,336125017	1,249320732	0,800434968	3,928571429
CHCHD5	1,18681E-09	0,320918997	0,366	0,081	2,50428E-05	1,249125989	0,800559758	4,518518519
FSTL3	8,58766E-09	0,320746024	0,72	0,288	0,000181208	1,248976233	0,800655748	2,5
NPEPL1	2,27654E-07	0,320640584	0,341	0,095	0,004803722	1,248884955	0,800714266	3,589473684
ZNRD2	1,81511E-11	0,3203608	0,28	0,035	3,83007E-07	1,24864278	0,800869565	8
RAD23A	9,74322E-07	0,320188239	0,598	0,232	0,020559168	1,248493438	0,800965363	2,577586207
UXT	4,23879E-07	0,31999639	0,537	0,2	0,008944265	1,248327426	0,801071882	2,685
GDE1	4,81925E-09	0,319951799	0,329	0,07	0,000101691	1,248288843	0,801096642	4,7
PRPF31	1,54785E-08	0,319589308	0,39	0,102	0,000326611	1,247975237	0,801297951	3,823529412
MCTS1	5,1942E-08	0,319188087	0,366	0,098	0,001096028	1,247628217	0,801520827	3,734693878
TMOD3	2,5471E-07	0,318924151	0,39	0,116	0,005374625	1,247399989	0,801667476	3,362068966
NPTXR	1,0745E-11	0,318150872	0,232	0,018	2,2673E-07	1,246731566	0,802097282	12,88888889
BAD	3,31435E-07	0,318147547	0,427	0,137	0,006993611	1,246728693	0,80209913	3,116788321
SLC15A4	5,39465E-10	0,317739383	0,268	0,039	1,13833E-05	1,246376021	0,80232609	6,871794872
VCL	0,000381883	0,317725627	0,707	0,407	1	1,246364137	0,80233374	1,737100737
SMDT1	7,28726E-07	0,317562964	0,5	0,189	0,015376853	1,246223618	0,802424208	2,645502646
SVBP	3,4886E-10	0,317233007	0,317	0,056	7,36129E-06	1,245938629	0,80260775	5,660714286
SNRPD1	1,03963E-08	0,317215363	0,549	0,186	0,000219372	1,245923391	0,802617566	2,951612903
CRLS1	1,86955E-11	0,317184266	0,341	0,056	3,94493E-07	1,245896536	0,802634867	6,089285714
CANT1	3,43575E-11	0,316879356	0,183	0,007	7,24977E-07	1,245633247	0,802804519	26,14285714
DOK1	1,36269E-09	0,316388713	0,232	0,028	2,87541E-05	1,245209694	0,80307759	8,285714286
CZIB	7,65047E-10	0,316377788	0,488	0,133	1,61432E-05	1,245200264	0,803083672	3,669172932
TIMM17B	7,94638E-10	0,31632693	0,341	0,07	1,67677E-05	1,245156369	0,803111982	4,871428571
PCDHGC3	1,45398E-09	0,316279882	0,232	0,028	3,06804E-05	1,245115763	0,803138174	8,285714286
MAD2L2	7,67373E-08	0,316077676	0,439	0,137	0,001619234	1,244941263	0,803250748	3,204379562
HK1	2,494E-11	0,31593493	0,427	0,091	5,26259E-07	1,244818089	0,803330229	4,692307692

Supplementary Table 2

Differentially expressed genes_keloid-XL_ comparing SC-Keloid vs. (SC-Myel, SC-Nonmyel, SC-Nonmyel2)								
gene	p_val	avg_log2FC	pct.1	pct.2	p_val_adj	Foldchange_UP	Foldchange_DOWN	Ratio_pct1_pct2
STX12	7,72211E-10	0,315419353	0,439	0,112	1,62944E-05	1,244373307	0,803617366	3,919642857
AKR1B1	1,1557E-09	0,315400692	0,415	0,098	2,43864E-05	1,244357211	0,803627761	4,234693878
UFD1	1,15129E-12	0,315246689	0,439	0,084	2,42933E-08	1,244224387	0,803713551	5,226190476
UBLCP1	9,54135E-18	0,315245378	0,402	0,039	2,01332E-13	1,244223257	0,803714281	10,30769231
NCK2	2,41934E-11	0,314969676	0,341	0,056	5,10506E-07	1,243985506	0,803867887	6,089285714
NDUFB6	7,16941E-08	0,31488651	0,585	0,211	0,001512818	1,243913796	0,803914229	2,772511848
MAN1B1	4,58338E-09	0,314855035	0,439	0,116	9,67139E-05	1,243886659	0,803931767	3,784482759
TMCO3	5,92941E-08	0,314794119	0,329	0,081	0,001251166	1,243834139	0,803965713	4,061728395
SARAF	0,000180637	0,314671013	0,805	0,449	1	1,243728005	0,804034319	1,792873051
TIMM10	8,56761E-09	0,314665091	0,329	0,074	0,000180785	1,2437229	0,804037619	4,445945946
ARAP3	2,19475E-07	0,314559241	0,244	0,049	0,004631148	1,243631652	0,804096614	4,979591837
KPNA3	1,31109E-05	0,31453579	0,268	0,077	0,276653936	1,243611437	0,804109684	3,480519481
RFLNB	2,12562E-10	0,314431009	0,171	0,007	4,48528E-06	1,243521118	0,804168088	24,42857143
SPATS2L	1,21625E-07	0,314116745	0,646	0,253	0,002566403	1,24325027	0,80434328	2,553359684
IFT22	6,97121E-18	0,313845066	0,39	0,035	1,47099E-13	1,243016172	0,804494763	11,14285714
TMEM178B	9,83782E-08	0,313843506	0,195	0,028	0,002075879	1,243014827	0,804495633	6,964285714
LRRC59	9,76123E-07	0,313671986	0,39	0,13	0,020597179	1,242867056	0,804591284	3
CCNI	0,003572266	0,313490013	0,78	0,516	1	1,242710298	0,804692776	1,511627907
NDUFA8	1,39338E-08	0,313101559	0,366	0,091	0,000294018	1,242375736	0,804909474	4,021978022
MRPS33	6,73125E-14	0,312945395	0,402	0,06	1,42036E-09	1,242241263	0,804996606	6,7
MTCO2P12	0,425842823	0,312859144	0,805	0,677	1	1,242166998	0,805044733	1,189069424
ABCD4	5,74357E-09	0,312437635	0,378	0,095	0,000121195	1,24180413	0,805279976	3,978947368
C18orf32	0,000102387	0,312067856	0,622	0,326	1	1,241485882	0,805486405	1,90797546
PSMC4	1,34814E-09	0,311988396	0,402	0,098	2,84472E-05	1,241417506	0,80553077	4,102040816
MRPS16	1,19163E-15	0,31131519	0,378	0,042	2,51446E-11	1,240838358	0,805906743	9
CAMK1D	1,87865E-10	0,311230384	0,256	0,032	3,96413E-06	1,24076542	0,805954119	8
FHOD1	5,84024E-12	0,311186781	0,354	0,056	1,23235E-07	1,240727921	0,805978477	6,321428571
PHB	4,90476E-08	0,310918567	0,5	0,161	0,001034954	1,240497276	0,806128332	3,105590062
XRCC6	1,70997E-05	0,310225926	0,537	0,228	0,360820705	1,239901854	0,806515449	2,355263158
RAB23	6,16607E-11	0,309962509	0,232	0,021	1,3011E-06	1,239675485	0,806662721	11,04761905

Supplementary Table 2

Differentially expressed genes_keloid-XL_ comparing SC-Keloid vs. (SC-Myel, SC-Nonmyel, SC-Nonmyel2)								
gene	p_val	avg_log2FC	pct.1	pct.2	p_val_adj	Foldchange_UP	Foldchange_DOWN	Ratio_pct1_pct2
CMAS	8,24922E-07	0,309187133	0,256	0,06	0,017406682	1,2390094	0,807096379	4,266666667
ATG13	1,1603E-08	0,309146004	0,244	0,039	0,000244834	1,238974079	0,807119388	6,256410256
EMC6	1,1633E-10	0,308790796	0,39	0,081	2,45468E-06	1,238669066	0,807318135	4,814814815
CTNNBIP1	1,70489E-12	0,308480112	0,366	0,06	3,5975E-08	1,238402348	0,807492009	6,1
ZNF480	8,2597E-10	0,308302714	0,146	0,004	1,74288E-05	1,23825008	0,807591307	36,5
IMMP1L	3,67004E-12	0,308022519	0,329	0,046	7,74416E-08	1,238009615	0,807748169	7,152173913
METTL23	6,2874E-12	0,307690812	0,293	0,035	1,3267E-07	1,237725002	0,80793391	8,371428571
TNFRSF6B	8,26544E-06	0,307338398	0,122	0,014	0,174409102	1,237422694	0,808131291	8,714285714
PEPD	2,53429E-05	0,307251756	0,488	0,2	0,534761166	1,237348382	0,808179826	2,44
TUBGCP2	1,18895E-07	0,307226457	0,305	0,074	0,002508802	1,237326684	0,808193998	4,121621622
PDZD11	3,94721E-08	0,3068534	0,293	0,063	0,000832901	1,237006773	0,808403011	4,650793651
TMED10	0,012557722	0,306653592	0,829	0,537	1	1,236835464	0,80851498	1,543761639
DNAJC19	2,35197E-09	0,306623961	0,512	0,151	4,96288E-05	1,236810062	0,808531586	3,390728477
VIRMA	0,000197025	0,306594596	0,171	0,046	1	1,236784887	0,808548043	3,717391304
ERG28	9,73562E-10	0,306363097	0,22	0,025	2,05431E-05	1,236586445	0,808677795	8,8
ATXN10	3,07543E-08	0,306130001	0,451	0,137	0,000648947	1,236386666	0,808808464	3,291970803
PSMC1	5,80406E-07	0,306097719	0,451	0,158	0,012247149	1,236359001	0,808826562	2,85443038
GSTT2B	3,58104E-07	0,306038249	0,232	0,046	0,007556354	1,236308038	0,808859903	5,043478261
JKAMP	8,37966E-08	0,305997575	0,39	0,109	0,001768191	1,236273183	0,808882708	3,577981651
PDCD6	3,66692E-07	0,305854475	0,659	0,263	0,007737576	1,236150564	0,808962945	2,505703422
CFAP298	9,29465E-08	0,305851721	0,341	0,088	0,001961263	1,236148204	0,808964489	3,875
POMGNT1	5,47919E-10	0,305753828	0,305	0,053	1,15616E-05	1,236064329	0,809019382	5,754716981
LDLRAP1	3,63748E-08	0,305229281	0,244	0,042	0,000767544	1,235614992	0,809313586	5,80952381
GPX4	0,000437663	0,305119793	0,841	0,526	1	1,235521223	0,809375008	1,598859316
RAPH1	8,35573E-09	0,304631647	0,366	0,088	0,000176314	1,235103247	0,809648912	4,159090909
EHD2	1,7404E-13	0,30435659	0,354	0,046	3,67243E-09	1,234867791	0,80980329	7,695652174
ORMDL2	4,81548E-07	0,304347426	0,244	0,053	0,010161135	1,234859947	0,809808434	4,603773585
SPSB3	1,94322E-08	0,303949977	0,512	0,161	0,000410039	1,234519801	0,81003156	3,180124224
ATP2C1	1,33137E-05	0,303808823	0,366	0,13	0,280932307	1,234399022	0,810110817	2,815384615
ACTR3	2,10268E-09	0,303802723	0,549	0,168	4,43687E-05	1,234393802	0,810114243	3,267857143

Supplementary Table 2

Differentially expressed genes_keloid-XL_ comparing SC-Keloid vs. (SC-Myel, SC-Nonmyel, SC-Nonmyel2)								
**gene**	**p_val**	**avg_log2FC**	**pct.1**	**pct.2**	**p_val_adj**	**Foldchange_UP**	**Foldchange_DOWN**	**Ratio_pct1_pct2**
RNF187	1,70078E-08	0,303779316	0,524	0,168	0,000358882	1,234373774	0,810127387	3,119047619
FAM177A1	3,39525E-07	0,303633149	0,573	0,211	0,007164324	1,23424872	0,810209469	2,71563981
TRPV2	5,73029E-16	0,303407043	0,22	0	1,20915E-11	1,234055298	0,810336459	#DIV/0!
SRPX2	1,8561E-11	0,302984084	0,305	0,042	3,91657E-07	1,233693559	0,810574062	7,261904762
MRPS7	8,17685E-11	0,302918396	0,329	0,056	1,7254E-06	1,233637389	0,810610969	5,875
CCDC28B	1,2997E-09	0,30284312	0,268	0,042	2,74249E-05	1,233573023	0,810653266	6,380952381
GRSF1	5,16897E-09	0,302493971	0,317	0,067	0,00010907	1,233274519	0,810849478	4,731343284
FEZ2	2,71224E-06	0,302328771	0,524	0,2	0,057231036	1,233133308	0,810942331	2,62
TMX1	3,87537E-08	0,302208738	0,463	0,14	0,000817741	1,233030715	0,811009805	3,307142857
BCHE	1,69521E-05	0,30218974	0,195	0,046	0,357706445	1,233014478	0,811020485	4,239130435
LSM4	3,54349E-08	0,301894607	0,415	0,116	0,000747712	1,232762265	0,811186413	3,577586207
FBXL6	4,4767E-08	0,301665011	0,232	0,039	0,000944628	1,232566094	0,811315519	5,948717949
NSMCE4A	9,91294E-10	0,301610767	0,256	0,035	2,09173E-05	1,232519752	0,811346024	7,314285714
PLGRKT	6,26286E-09	0,301607898	0,28	0,053	0,000132153	1,232517301	0,811347637	5,283018868
MRPL47	4,30624E-10	0,301535115	0,366	0,077	9,0866E-06	1,232455123	0,81138857	4,753246753
CEP89	4,11479E-08	0,301194521	0,232	0,039	0,000868262	1,232164197	0,811580147	5,948717949
MRPS14	5,21434E-11	0,301073794	0,305	0,046	1,10028E-06	1,232061092	0,811648064	6,630434783
SH2B3	2,77734E-07	0,30075775	0,329	0,088	0,005860474	1,23179122	0,811825887	3,738636364
ANKRD44	9,84372E-07	0,300493689	0,293	0,077	0,02077124	1,231565782	0,811974492	3,805194805
TPRKB	8,0679E-10	0,300356009	0,305	0,053	1,70241E-05	1,231448256	0,812051985	5,754716981
PAWR	1,22894E-08	0,299906718	0,244	0,039	0,000259318	1,231064812	0,812304917	6,256410256
CCL14	0,0103374	0,299449512	0,159	0,063	1	1,230674736	0,812562386	2,523809524
MFN1	4,22033E-11	0,29901085	0,244	0,025	8,90531E-07	1,230300597	0,812809489	9,76
IFITM10	1,64161E-12	0,298926212	0,256	0,021	3,46396E-08	1,230228422	0,812857175	12,19047619
SUMF2	6,09665E-05	0,298803499	0,378	0,151	1	1,230123786	0,812926318	2,503311258
RAB14	5,29106E-06	0,298783311	0,622	0,274	0,111646684	1,230106572	0,812937694	2,270072993
PYCR1	1,45951E-09	0,298639118	0,22	0,025	3,07971E-05	1,229983633	0,813018949	8,8
DECR1	2,60628E-07	0,297885803	0,451	0,147	0,005499514	1,229341555	0,813443584	3,068027211
BEX1	5,73002E-06	0,297707166	0,207	0,046	0,120909104	1,229189345	0,813544312	4,5
DALRD3	1,00176E-10	0,297699555	0,28	0,039	2,11382E-06	1,22918286	0,813548604	7,179487179

Supplementary Table 2

Differentially expressed genes_keloid-XL_ comparing SC-Keloid vs. (SC-Myel, SC-Nonmyel, SC-Nonmyel2)								
**gene**	**p_val**	**avg_log2FC**	**pct.1**	**pct.2**	**p_val_adj**	**Foldchange_UP**	**Foldchange_DOWN**	**Ratio_pct1_pct2**
PTRHD1	7,37514E-10	0,297560059	0,329	0,063	1,55623E-05	1,229064014	0,813627271	5,222222222
EEF2	0,005169813	0,297500053	0,939	0,772	1	1,229012895	0,813661113	1,216321244
CIAPIN1	4,9772E-15	0,297468142	0,305	0,025	1,05024E-10	1,228985711	0,813679111	12,2
HMGB1	0,005698602	0,297357959	0,951	0,849	1	1,228891853	0,813741256	1,120141343
ATAD1	3,86389E-09	0,297354205	0,39	0,095	8,1532E-05	1,228888655	0,813743374	4,105263158
FIS1	4,07396E-07	0,297184712	0,732	0,309	0,008596459	1,22874429	0,81383898	2,368932039
RPS4X	0,004252664	0,296849708	0,976	0,902	1	1,228459	0,814027981	1,082039911
RALA	5,94843E-10	0,29661811	0,427	0,105	1,25518E-05	1,228261809	0,814158669	4,066666667
CAMLG	7,79464E-06	0,296617486	0,622	0,256	0,164474797	1,228261278	0,814159021	2,4296875
GNB2	0,000102149	0,296073769	0,744	0,379	1	1,227798463	0,814465916	1,963060686
REX1BD	1,3949E-07	0,295987624	0,598	0,228	0,002943375	1,227725152	0,81451455	2,622807018
SSBP4	6,96116E-09	0,29590277	0,439	0,119	0,000146887	1,227652944	0,814562459	3,68907563
PFDN6	1,30225E-11	0,295894092	0,402	0,077	2,74788E-07	1,227645559	0,814567358	5,220779221
KRR1	1,61217E-08	0,295201715	0,378	0,095	0,000340184	1,22705653	0,814958378	3,978947368
STK11	1,92674E-05	0,294682401	0,366	0,133	0,406561383	1,226614917	0,815251785	2,751879699
TMEM258	0,000125006	0,293943539	0,89	0,561	1	1,225986879	0,815669414	1,586452763
SOCS2	2,69111E-05	0,293525193	0,634	0,291	0,567850574	1,225631424	0,815905973	2,178694158
SURF1	1,01713E-09	0,293491556	0,415	0,102	2,14624E-05	1,225602849	0,815924997	4,068627451
CRTAP	1,42152E-05	0,292875143	0,488	0,204	0,29995459	1,225079304	0,816273687	2,392156863
GSPT1	1,71513E-06	0,292267949	0,415	0,14	0,036191023	1,224563807	0,816617308	2,964285714
GNS	0,000198924	0,292171568	0,207	0,063	1	1,224482002	0,816671865	3,285714286
PTPRE	8,61929E-08	0,29207589	0,512	0,175	0,001818755	1,224400798	0,816726028	2,925714286
BCL2L1	6,81442E-11	0,291887908	0,354	0,063	1,43791E-06	1,22424127	0,816832454	5,619047619
RARS1	5,66777E-09	0,291874419	0,268	0,046	0,000119596	1,224229823	0,816840091	5,826086957
AGPAT1	1,87975E-08	0,291283979	0,402	0,109	0,000396647	1,223728895	0,817174461	3,688073394
EIF2AK1	2,93233E-08	0,291223834	0,305	0,067	0,00061875	1,22367788	0,817208529	4,552238806
PXYLP1	6,01716E-05	0,291188983	0,085	0,007	1	1,22364832	0,81722827	12,14285714
UQCC3	2,279E-10	0,29109728	0,402	0,088	4,80892E-06	1,223570543	0,817280218	4,568181818
AAR2	8,65211E-10	0,290279683	0,146	0,004	1,82568E-05	1,222877324	0,817743514	36,5
IL11	9,98574E-09	0,290263887	0,159	0,011	0,000210709	1,222863935	0,817752468	14,45454545

Supplementary Table 2

Differentially expressed genes_keloid-XL_ comparing SC-Keloid vs. (SC-Myel, SC-Nonmyel, SC-Nonmyel2)								
gene	p_val	avg_log2FC	pct.1	pct.2	p_val_adj	Foldchange_UP	Foldchange_DOWN	Ratio_pct1_pct2
FKBP9	4,32263E-08	0,29002264	0,439	0,133	0,000912118	1,222659465	0,817889223	3,30075188
IFT57	1,16197E-07	0,289789572	0,39	0,112	0,00245188	1,222461959	0,818021364	3,482142857
PMPCA	5,68688E-07	0,289754491	0,268	0,063	0,01199989	1,222432234	0,818041256	4,253968254
CHST11	7,12258E-09	0,289623519	0,183	0,018	0,000150293	1,222321263	0,818115523	10,16666667
VPS45	7,26135E-07	0,289003704	0,207	0,039	0,015322168	1,221796239	0,81846708	5,307692308
LRP1	0,001259814	0,288685788	0,585	0,337	1	1,22152703	0,818647459	1,735905045
TMEM51	5,15386E-08	0,288682172	0,159	0,014	0,001087517	1,221523968	0,818649511	11,35714286
TMX2	3,63456E-07	0,288612627	0,28	0,067	0,007669294	1,221465086	0,818688975	4,179104478
SLC41A3	1,56249E-06	0,288427396	0,256	0,063	0,032970135	1,22130827	0,818794095	4,063492063
NCOR2	3,01136E-06	0,288219849	0,585	0,242	0,063542715	1,221132584	0,818911896	2,417355372
TSPAN9	6,42925E-12	0,287891552	0,305	0,039	1,35664E-07	1,220854737	0,819098268	7,820512821
C19orf25	8,04034E-08	0,287865893	0,28	0,06	0,001696593	1,220833023	0,819112836	4,666666667
LENG1	9,5155E-05	0,287827238	0,171	0,042	1	1,220800313	0,819134783	4,071428571
TWF1	2,92147E-09	0,287377051	0,354	0,077	6,1646E-05	1,220419428	0,81939043	4,597402597
DIAPH1	2,60756E-09	0,286941585	0,39	0,095	5,50221E-05	1,220051109	0,819637794	4,105263158
TASP1	5,55324E-11	0,286736247	0,329	0,053	1,17179E-06	1,219877472	0,819754461	6,20754717
HAGH	6,76335E-07	0,28670315	0,427	0,14	0,01427134	1,219849487	0,819773267	3,05
PINK1	2,40937E-08	0,286620238	0,354	0,088	0,000508401	1,219779384	0,819820381	4,022727273
ATP13A1	1,41131E-09	0,286551659	0,22	0,025	2,978E-05	1,219721403	0,819859353	8,8
RAC1	0,000685981	0,286504953	0,89	0,726	1	1,219681916	0,819885896	1,225895317
PTDSS2	1,16562E-12	0,286035639	0,293	0,032	2,45957E-08	1,219285214	0,820152651	9,15625
SSR1	1,73394E-08	0,285965548	0,585	0,2	0,000365878	1,219225978	0,820192498	2,925
RPL29	0,007320851	0,285961005	0,976	0,888	1	1,219222139	0,82019508	1,099099099
SNHG9	4,05114E-07	0,285909988	0,5	0,179	0,008548309	1,219179025	0,820224085	2,793296089
ATP1B1	4,86399E-05	0,285707035	0,695	0,351	1	1,219007528	0,820339479	1,98005698
NADK	1,2467E-08	0,285694715	0,244	0,039	0,000263067	1,218997118	0,820346484	6,256410256
RWDD1	5,5758E-06	0,285561627	0,598	0,26	0,117655055	1,218884671	0,820422165	2,3
MED24	0,000637069	0,285489477	0,171	0,053	1	1,218823716	0,820463195	3,226415094
IFT52	4,83465E-11	0,285275887	0,317	0,049	1,02016E-06	1,218643283	0,820584673	6,469387755
BTC	3,35288E-07	0,285241719	0,134	0,011	0,00707491	1,218614422	0,820604108	12,18181818

Supplementary Table 2

Differentially expressed genes_keloid-XL_ comparing SC-Keloid vs. (SC-Myel, SC-Nonmyel, SC-Nonmyel2)								
gene	p_val	avg_log2FC	pct.1	pct.2	p_val_adj	Foldchange_UP	Foldchange_DOWN	Ratio_pct1_pct2
MMADHC	7,78968E-10	0,285204186	0,293	0,049	1,6437E-05	1,218582719	0,820625457	5,979591837
TSG101	2,47394E-09	0,285052337	0,402	0,098	5,22026E-05	1,218454465	0,820711835	4,102040816
AZI2	1,80608E-07	0,284913725	0,341	0,088	0,003811004	1,218337403	0,820790692	3,875
ABHD14A	1,47881E-08	0,284574481	0,232	0,035	0,000312045	1,21805095	0,82098372	6,628571429
GOLT1B	1,55549E-07	0,28454097	0,402	0,123	0,003282246	1,218022657	0,82100279	3,268292683
MMP28	3,42792E-05	0,284512363	0,232	0,067	0,723324825	1,217998505	0,82101907	3,462686567
NFIX	3,47856E-05	0,284348026	0,732	0,368	0,734010428	1,217859771	0,821112597	1,989130435
ZNF22	3,79794E-07	0,284304981	0,463	0,151	0,008014026	1,217823436	0,821137097	3,066225166
AFAP1L1	1,0923E-11	0,284101498	0,305	0,042	2,30485E-07	1,217651681	0,821252921	7,261904762
EIF2S2	3,96782E-05	0,283974273	0,622	0,312	0,837249287	1,217544307	0,821325347	1,993589744
CADM1	0,00496992	0,283442885	0,878	0,656	1	1,217095931	0,821627922	1,338414634
GAP43	1,33009E-07	0,283297106	0,305	0,074	0,002806627	1,216972954	0,821710948	4,121621622
LARP6	7,40022E-05	0,283277719	0,22	0,063	1	1,2169566	0,82172199	3,492063492
PMPCB	5,07535E-08	0,283271991	0,329	0,081	0,001070949	1,216951769	0,821725253	4,061728395
RAB11B	3,70878E-07	0,283095481	0,585	0,228	0,007825896	1,216802887	0,821825795	2,565789474
SLC22A18	1,69222E-09	0,283013322	0,171	0,011	3,57076E-05	1,216733594	0,821872598	15,54545455
MFSD1	3,98217E-09	0,28249107	0,28	0,049	8,40279E-05	1,216293219	0,822170168	5,714285714
LONP1	4,5734E-10	0,281573164	0,28	0,042	9,65032E-06	1,215519606	0,822693435	6,666666667
SART1	3,37602E-10	0,281411307	0,354	0,07	7,12373E-06	1,215383244	0,822785738	5,057142857
NOL7	3,17706E-07	0,281257467	0,646	0,256	0,006703911	1,21525365	0,82287348	2,5234375
FAM210B	4,35363E-07	0,28106763	0,537	0,193	0,009186595	1,215093752	0,822981765	2,78238342
SPOCK1	3,71719E-10	0,280529279	0,195	0,014	7,84365E-06	1,214640415	0,823288924	13,92857143
RAB18	7,49454E-07	0,280504998	0,463	0,158	0,015814224	1,214619973	0,823302779	2,930379747
HDDC2	1,56882E-09	0,280363368	0,341	0,07	3,31037E-05	1,214500739	0,823383608	4,871428571
ZBTB8OS	2,05217E-09	0,28036154	0,305	0,056	4,33028E-05	1,2144992	0,823384651	5,446428571
ATG4B	3,97798E-07	0,28022555	0,305	0,077	0,008393938	1,214384726	0,823462268	3,961038961
MRPL2	1,01953E-08	0,28015077	0,305	0,063	0,000215131	1,214321781	0,823504952	4,841269841
POLR2C	1,11771E-06	0,279977891	0,305	0,084	0,023584828	1,214176277	0,823603639	3,630952381
CLEC11A	1,32817E-07	0,279912391	0,39	0,116	0,002802565	1,214121154	0,823641032	3,362068966
PBDC1	2,98878E-11	0,279761359	0,366	0,067	6,30663E-07	1,213994057	0,823727262	5,462686567

Supplementary Table 2

Differentially expressed genes_keloid-XL_ comparing SC-Keloid vs. (SC-Myel, SC-Nonmyel, SC-Nonmyel2)								
gene	p_val	avg_log2FC	pct.1	pct.2	p_val_adj	Foldchange_UP	Foldchange_DOWN	Ratio_pct1_pct2
TSHZ3	4,86028E-08	0,279310698	0,171	0,018	0,001025568	1,213614895	0,823984613	9,5
RPS5	0,02774199	0,279180291	0,854	0,709	1	1,2135052	0,824059098	1,204513399
GTF2H2	5,5067E-05	0,278733834	0,098	0,011	1	1,213129727	0,82431415	8,909090909
COX7C	0,003361054	0,278447224	0,854	0,572	1	1,212888747	0,824477927	1,493006993
HMGN4	2,38503E-11	0,278413848	0,341	0,056	5,03265E-07	1,212860688	0,824497001	6,089285714
P4HTM	7,44811E-06	0,278302939	0,354	0,119	0,157162491	1,212767451	0,824560388	2,974789916
PPHLN1	5,27282E-08	0,278246042	0,341	0,084	0,001112618	1,212719623	0,824592908	4,05952381
CSK	2,8562E-11	0,278185051	0,293	0,039	6,02687E-07	1,212668355	0,824627769	7,512820513
KCTD5	2,22375E-07	0,277982764	0,268	0,06	0,004692332	1,212498333	0,824743402	4,466666667
SLC25A11	5,96944E-08	0,277749576	0,305	0,07	0,001259611	1,212302368	0,824876719	4,357142857
UBALD1	5,56778E-08	0,277553657	0,305	0,07	0,001174857	1,212137748	0,824988745	4,357142857
PAK1IP1	6,39267E-10	0,277500461	0,293	0,049	1,34892E-05	1,212093054	0,825019165	5,979591837
PPP1R7	2,00108E-08	0,277260312	0,378	0,098	0,000422248	1,211891308	0,825156508	3,857142857
PMS1	7,85877E-07	0,277231602	0,207	0,039	0,01658279	1,211867191	0,825172929	5,307692308
ATP6V0B	1,12934E-05	0,277131136	0,634	0,291	0,238301132	1,211782803	0,825230394	2,178694158
THUMPD3	1,10294E-09	0,277083548	0,232	0,028	2,32731E-05	1,211742831	0,825257616	8,285714286
RDH11	1,84116E-06	0,27707633	0,39	0,133	0,038850284	1,211736769	0,825261745	2,932330827
RPS15A	0,002945226	0,277007134	0,976	0,881	1	1,211678652	0,825301328	1,107832009
HS2ST1	3,2095E-08	0,276932646	0,28	0,056	0,000677237	1,211616093	0,82534394	5
USE1	3,09073E-07	0,276922947	0,317	0,084	0,006521747	1,211607948	0,825349488	3,773809524
KRT10-AS1	1,21581E-07	0,276645054	0,195	0,028	0,002565474	1,21137459	0,825508483	6,964285714
COLGALT1	1,58854E-07	0,276379353	0,28	0,063	0,003351972	1,211151511	0,825660531	4,444444444
PCYOX1	1,21174E-06	0,276189513	0,305	0,084	0,025568919	1,21099215	0,825769184	3,630952381
DNAJB11	5,30193E-09	0,276127848	0,5	0,147	0,000111876	1,21094039	0,825804481	3,401360544
SSR3	0,000766378	0,276013976	0,561	0,298	1	1,210844814	0,825869664	1,882550336
XRCC5	2,09367E-06	0,275953453	0,61	0,26	0,044178433	1,210794018	0,825904311	2,346153846
UCHL5	1,95169E-07	0,275951885	0,268	0,06	0,004118266	1,210792703	0,825905209	4,466666667
ITGB1BP1	4,85426E-06	0,275935705	0,549	0,225	0,102429787	1,210779123	0,825914472	2,44
CLASP1	0,000348582	0,275758744	0,207	0,067	1	1,210630618	0,826015785	3,089552239
ITGA5	6,53581E-10	0,275648219	0,256	0,035	1,37912E-05	1,210537875	0,826079068	7,314285714

Supplementary Table 2

Differentially expressed genes_keloid-XL_ comparing SC-Keloid vs. (SC-Myel, SC-Nonmyel, SC-Nonmyel2)								
**gene**	**p_val**	**avg_log2FC**	**pct.1**	**pct.2**	**p_val_adj**	**Foldchange_UP**	**Foldchange_DOWN**	**Ratio_pct1_pct2**
PDCD6IP	8,5331E-08	0,275443114	0,524	0,182	0,001800569	1,210365788	0,826196518	2,879120879
NUTF2	7,7476E-12	0,275203861	0,463	0,102	1,63482E-07	1,21016508	0,826333544	4,539215686
DAGLA	0,00174268	0,274848315	0,134	0,039	1	1,209866877	0,826537216	3,435897436
OTUB1	1,86918E-07	0,274778652	0,39	0,116	0,003944153	1,209808458	0,826577128	3,362068966
CCDC43	6,56905E-07	0,27462587	0,22	0,042	0,013861358	1,209680345	0,826664667	5,238095238
ARNTL	0,000105356	0,274403186	0,268	0,091	1	1,209493642	0,826792275	2,945054945
C1QBP	4,47854E-07	0,273990468	0,451	0,151	0,009450175	1,209147687	0,827028832	2,986754967
FAM168A	6,7829E-08	0,273925642	0,354	0,091	0,00143126	1,209093356	0,827065995	3,89010989
PEX16	6,6848E-05	0,273873728	0,159	0,035	1	1,209049849	0,827095757	4,542857143
NTAN1	1,52639E-06	0,273627731	0,305	0,084	0,032208425	1,208843709	0,827236799	3,630952381
NFE2L3	0,002795762	0,273510213	0,122	0,035	1	1,208745244	0,827304186	3,485714286
ADAM12	8,45368E-10	0,273281903	0,146	0,004	1,78381E-05	1,208553972	0,827435119	36,5
B3GAT3	2,00309E-07	0,27294831	0,366	0,102	0,004226728	1,208274552	0,827626468	3,588235294
RPS29	0,029862512	0,272929351	0,976	0,933	1	1,208258673	0,827637345	1,046087889
PSMG3	1,24543E-08	0,272835555	0,329	0,074	0,000262798	1,208180122	0,827691154	4,445945946
TOMM7	0,001045251	0,272460172	0,915	0,649	1	1,207865799	0,827906545	1,409861325
GOLIM4	6,10303E-07	0,272427855	0,488	0,172	0,012878006	1,207838743	0,82792509	2,837209302
CWC15	2,77981E-07	0,272349903	0,402	0,123	0,005865672	1,207773482	0,827969826	3,268292683
ZDHHC12	5,94697E-11	0,272279027	0,232	0,021	1,25487E-06	1,207714149	0,828010503	11,04761905
SPAG7	2,2262E-08	0,271959461	0,378	0,098	0,00046975	1,207446663	0,828193933	3,857142857
MPC2	4,9959E-09	0,271900997	0,524	0,158	0,000105419	1,207397733	0,828227495	3,316455696
PSMA2	3,68663E-06	0,271805464	0,634	0,267	0,077791682	1,207317783	0,828282341	2,374531835
COPB1	1,454E-07	0,271649321	0,39	0,112	0,003068085	1,207187122	0,828371991	3,482142857
PITPNM3	5,5905E-11	0,271522248	0,146	0	1,17965E-06	1,207080797	0,828444958	#DIV/0!
HTRA2	1,18209E-06	0,271487386	0,268	0,067	0,024943384	1,207051629	0,828464977	4
XPO6	1,84816E-05	0,271330488	0,268	0,081	0,389981025	1,206920366	0,82855508	3,308641975
PCMTD2	5,9403E-06	0,27123039	0,341	0,109	0,125346349	1,206836629	0,82861257	3,128440367
CETN2	9,93147E-13	0,27088177	0,427	0,077	2,09564E-08	1,206545038	0,828812824	5,545454545
SGPL1	0,003210374	0,270829431	0,134	0,042	1	1,206501268	0,828842892	3,19047619
SPCS3	1,40989E-05	0,270820271	0,5	0,204	0,297500221	1,206493607	0,828848155	2,450980392

Supplementary Table 2

Differentially expressed genes_keloid-XL_ comparing SC-Keloid vs. (SC-Myel, SC-Nonmyel, SC-Nonmyel2)								
**gene**	**p_val**	**avg_log2FC**	**pct.1**	**pct.2**	**p_val_adj**	**Foldchange_UP**	**Foldchange_DOWN**	**Ratio_pct1_pct2**
LDHB	0,000202357	0,270671537	0,561	0,281	1	1,206369231	0,828933609	1,996441281
PRRC2B	1,05217E-05	0,270644365	0,488	0,196	0,222018888	1,206346509	0,828949222	2,489795918
TMEM141	1,7624E-07	0,270587302	0,354	0,098	0,003718836	1,206298796	0,82898201	3,612244898
LAMA2	0,000219066	0,270489539	0,402	0,175	1	1,206217055	0,829038187	2,297142857
ABHD11	9,6391E-09	0,270470622	0,183	0,018	0,000203395	1,206201239	0,829049057	10,16666667
MRPL19	4,14669E-07	0,270248307	0,378	0,112	0,008749923	1,206015381	0,829176821	3,375
ERAP2	1,71287E-08	0,270127929	0,232	0,035	0,000361434	1,205914756	0,82924601	6,628571429
LIX1L	6,70227E-08	0,270097623	0,28	0,06	0,001414246	1,205889424	0,82926343	4,666666667
AP1M1	2,36321E-11	0,269913806	0,293	0,039	4,98662E-07	1,205735789	0,829369095	7,512820513
PRR14	2,0035E-05	0,269645386	0,366	0,133	0,422759089	1,205511477	0,829523417	2,751879699
IFRD2	1,33166E-11	0,269311104	0,317	0,046	2,80994E-07	1,205232184	0,829715646	6,891304348
TRIAP1	1,16175E-10	0,269271645	0,268	0,035	2,4514E-06	1,205199221	0,829738339	7,657142857
WDR61	5,3475E-08	0,269204062	0,415	0,119	0,001128375	1,205142764	0,829777209	3,487394958
DYNC2I2	4,90851E-08	0,269123337	0,329	0,081	0,001035744	1,205075333	0,829823641	4,061728395
DHX30	1,59245E-07	0,269109011	0,366	0,102	0,003360224	1,205063367	0,829831881	3,588235294
USB1	1,3444E-10	0,269066769	0,28	0,039	2,83682E-06	1,205028083	0,829856178	7,179487179
LRRC28	4,41502E-09	0,268773396	0,232	0,032	9,31613E-05	1,204783065	0,830024948	7,25
SNX7	1,09012E-09	0,268755677	0,329	0,063	2,30026E-05	1,204768268	0,830035141	5,222222222
ALDH18A1	1,76146E-12	0,268645124	0,244	0,018	3,71687E-08	1,204675951	0,830098749	13,55555556
TELO2	1,54556E-08	0,268537339	0,232	0,035	0,000326128	1,204585952	0,830160769	6,628571429
GAS8	1,02456E-11	0,268181369	0,22	0,014	2,16192E-07	1,204288769	0,830365628	15,71428571
UBE2C	0,043000486	0,268109399	0,037	0,007	1	1,204228694	0,830407052	5,285714286
RGS19	5,25198E-12	0,268108253	0,195	0,007	1,10822E-07	1,204227737	0,830407712	27,85714286
SEC14L2	9,67084E-10	0,268032508	0,244	0,032	2,04064E-05	1,204164514	0,830451311	7,625
LIG4	1,30526E-10	0,267775377	0,159	0,004	2,75423E-06	1,203949916	0,830599336	39,75
VTI1B	4,14827E-07	0,267717226	0,341	0,095	0,00875327	1,203901388	0,830632816	3,589473684
GTF3C3	1,82755E-05	0,266820085	0,171	0,035	0,385631777	1,203152974	0,831149506	4,885714286
PPP2R3C	6,29382E-10	0,266612138	0,305	0,053	1,32806E-05	1,202979567	0,831269315	5,754716981
ERP44	9,26112E-05	0,266606705	0,39	0,158	1	1,202975037	0,831272445	2,46835443
FURIN	4,50205E-10	0,266333754	0,415	0,095	9,49978E-06	1,202747461	0,831429732	4,368421053

Supplementary Table 2

Differentially expressed genes_keloid-XL_ comparing SC-Keloid vs. (SC-Myel, SC-Nonmyel, SC-Nonmyel2)								
**gene**	**p_val**	**avg_log2FC**	**pct.1**	**pct.2**	**p_val_adj**	**Foldchange_UP**	**Foldchange_DOWN**	**Ratio_pct1_pct2**
UQCRC2	3,14186E-06	0,265752554	0,329	0,102	0,066296306	1,202263023	0,831764748	3,225490196
RPF1	1,1539E-09	0,265554087	0,354	0,074	2,43484E-05	1,202097644	0,831879178	4,783783784
FOXRED2	2,80682E-05	0,265543219	0,061	0	0,592266086	1,202088587	0,831885446	#DIV/0!
B4GALNT3	2,0388E-08	0,265520187	0,268	0,049	0,000430207	1,202069397	0,831898726	5,469387755
MLPH	1,58349E-08	0,265467545	0,11	0	0,000334132	1,202025536	0,831929082	#DIV/0!
AKT1	2,12321E-07	0,265441792	0,476	0,158	0,004480194	1,20200408	0,831943932	3,012658228
STK16	1,31086E-07	0,265399158	0,195	0,028	0,002766052	1,201968558	0,831968518	6,964285714
LSM2	1,04563E-08	0,265332076	0,524	0,161	0,000220639	1,201912671	0,832007203	3,254658385
EIF3D	1,48308E-06	0,26517444	0,512	0,204	0,031294491	1,201781351	0,832098117	2,509803922
TMED1	3,61895E-08	0,265153498	0,268	0,053	0,000763635	1,201763907	0,832110196	5,056603774
MDFIC	1,1603E-08	0,265142041	0,244	0,039	0,000244834	1,201754362	0,832116805	6,256410256
GOPC	2,54803E-09	0,265104376	0,366	0,081	5,37659E-05	1,201722988	0,832138529	4,518518519
TMEM250	2,09267E-07	0,2649446	0,159	0,018	0,004415741	1,201589907	0,832230692	8,833333333
SCG5	1,99881E-07	0,26491202	0,171	0,021	0,004217683	1,201562772	0,832249486	8,142857143
ZMAT3	3,46453E-05	0,264896289	0,293	0,098	0,7310502	1,20154967	0,832258561	2,989795918
PRCP	9,57539E-08	0,264700115	0,378	0,105	0,002020502	1,201386298	0,832371737	3,6
TUSC2	4,60404E-08	0,264516489	0,232	0,039	0,000971499	1,201233396	0,832477688	5,948717949
GLG1	0,000255757	0,264393661	0,671	0,361	1	1,20113113	0,832548566	1,858725762
PTS	4,10921E-08	0,264212819	0,366	0,095	0,000867085	1,200980577	0,832652933	3,852631579
HAUS3	1,6798E-05	0,264211834	0,22	0,056	0,354453774	1,200979757	0,832653502	3,928571429
SMIM19	3,5297E-06	0,264124352	0,5	0,196	0,074480095	1,200906935	0,832703993	2,551020408
APMAP	1,37151E-09	0,264063059	0,354	0,074	2,89402E-05	1,200855915	0,832739372	4,783783784
FAM174C	4,48201E-09	0,26384589	0,366	0,084	9,4575E-05	1,200675164	0,832864733	4,357142857
PTPMT1	2,54344E-10	0,263768274	0,329	0,06	5,36691E-06	1,200610569	0,832909542	5,483333333
IARS2	4,2636E-09	0,263626641	0,28	0,049	8,99661E-05	1,200492708	0,832991315	5,714285714
ISOC2	5,52108E-08	0,263440922	0,439	0,13	0,001165003	1,200338178	0,833098554	3,376923077
SNRNP25	4,91815E-08	0,263241271	0,268	0,053	0,001037778	1,200172078	0,833213852	5,056603774
NDUFV2	1,70923E-07	0,262991113	0,598	0,228	0,003606655	1,199963991	0,833358341	2,622807018
NNT	5,31341E-09	0,262629148	0,232	0,032	0,000112118	1,199662963	0,833567453	7,25
THAP11	2,22401E-09	0,262423148	0,268	0,042	4,69289E-05	1,199491677	0,833686485	6,380952381

Supplementary Table 2

Differentially expressed genes_keloid-XL_ comparing SC-Keloid vs. (SC-Myel, SC-Nonmyel, SC-Nonmyel2)								
**gene**	**p_val**	**avg_log2FC**	**pct.1**	**pct.2**	**p_val_adj**	**Foldchange_UP**	**Foldchange_DOWN**	**Ratio_pct1_pct2**
FASTK	5,47672E-09	0,262147604	0,402	0,102	0,000115564	1,199262606	0,833845727	3,941176471
PRKRA	3,90973E-07	0,261975349	0,402	0,123	0,00824993	1,199119425	0,833945293	3,268292683
EIF3G	9,8552E-06	0,261840479	0,634	0,288	0,207954591	1,19900733	0,834023258	2,201388889
ITM2C	5,14375E-05	0,261790312	0,646	0,312	1	1,198965638	0,83405226	2,070512821
ELOVL1	1,39668E-13	0,261667091	0,366	0,053	2,94714E-09	1,198863238	0,8341235	6,905660377
WDR46	1,15731E-07	0,261639606	0,232	0,042	0,002442033	1,198840399	0,834139391	5,523809524
MSRA	2,39174E-09	0,261081208	0,256	0,039	5,0468E-05	1,198376475	0,834462309	6,564102564
NDUFA12	2,90595E-09	0,261022463	0,659	0,235	6,13184E-05	1,198327679	0,834496288	2,804255319
UCHL1	1,14068E-05	0,260889753	0,183	0,039	0,240695295	1,198217454	0,834573054	4,692307692
MGAT2	6,47862E-13	0,260610287	0,207	0,007	1,36705E-08	1,197985367	0,834734737	29,57142857
OLA1	3,83964E-06	0,260535538	0,366	0,123	0,081020327	1,197923299	0,834777987	2,975609756
MAD1L1	6,39229E-15	0,260516311	0,268	0,014	1,34884E-10	1,197907334	0,834789112	19,14285714
LSM5	4,80345E-09	0,260504832	0,366	0,084	0,000101358	1,197897803	0,834795754	4,357142857
NSMF	8,23642E-06	0,260393769	0,195	0,042	0,173796802	1,197805589	0,834860022	4,642857143
CBY1	1,45455E-07	0,259930546	0,305	0,074	0,003069244	1,197421057	0,835128123	4,121621622
RNF20	2,45763E-07	0,259893574	0,244	0,049	0,005185847	1,197390371	0,835149525	4,979591837
PHB2	1,5997E-06	0,259803326	0,585	0,235	0,033755317	1,197315471	0,83520177	2,489361702
HGS	1,29115E-07	0,259694344	0,341	0,088	0,002724452	1,197225028	0,835264864	3,875
KATNAL1	1,77302E-05	0,259557418	0,305	0,098	0,374125844	1,197111405	0,835344142	3,112244898
TRAP1	3,7569E-06	0,259047581	0,232	0,056	0,079274306	1,196688431	0,835639398	4,142857143
PAM16	5,32974E-08	0,259039947	0,378	0,102	0,001124629	1,196682098	0,835643821	3,705882353
MRPL15	1,07496E-11	0,258898679	0,268	0,028	2,26827E-07	1,196564926	0,83572565	9,571428571
FAM207A	7,51442E-09	0,258806607	0,207	0,025	0,000158562	1,196488564	0,835778987	8,28
ANTKMT	2,31989E-09	0,258728964	0,5	0,144	4,8952E-05	1,196424173	0,835823969	3,472222222
USP11	2,66205E-05	0,258413902	0,476	0,2	0,561718121	1,196162921	0,836006519	2,38
MTCH2	2,89322E-08	0,258208659	0,317	0,07	0,000610499	1,195992762	0,836125461	4,528571429
NELFB	5,43568E-06	0,257997077	0,305	0,091	0,11469838	1,195817374	0,836248094	3,351648352
MRPS10	2,52304E-07	0,257940301	0,293	0,07	0,005323857	1,195770314	0,836281005	4,185714286
WDR11	8,34326E-09	0,257882052	0,268	0,046	0,000176051	1,195722036	0,83631477	5,826086957
TNIP1	2,39981E-08	0,257865364	0,463	0,137	0,000506384	1,195708205	0,836324444	3,379562044

Supplementary Table 2

Differentially expressed genes_keloid-XL_ comparing SC-Keloid vs. (SC-Myel, SC-Nonmyel, SC-Nonmyel2)								
**gene**	**p_val**	**avg_log2FC**	**pct.1**	**pct.2**	**p_val_adj**	**Foldchange_UP**	**Foldchange_DOWN**	**Ratio_pct1_pct2**
CHD8	0,000396649	0,257814053	0,244	0,088	1	1,195665679	0,836354189	2,772727273
ZNF205	8,69795E-08	0,257767633	0,22	0,035	0,001835353	1,195627208	0,8363811	6,285714286
COX14	1,98687E-06	0,257759912	0,524	0,2	0,041925018	1,195620809	0,836385577	2,62
LY6D	0,034389558	0,257729777	0,073	0,025	1	1,195595836	0,836403047	2,92
RPS9	0,01482614	0,257712467	0,976	0,891	1	1,195581491	0,836413082	1,095398429
SPCS2	6,68613E-05	0,257239817	0,768	0,396	1	1,195189863	0,83668715	1,939393939
NKIRAS2	3,25629E-09	0,256265053	0,317	0,063	6,87111E-05	1,1943826	0,837252652	5,031746032
NDUFB5	8,30963E-07	0,25603467	0,476	0,168	0,017534159	1,194191885	0,837386363	2,833333333
MTHFD1L	7,60717E-11	0,255983862	0,293	0,042	1,60519E-06	1,194149829	0,837415855	6,976190476
PPT1	5,58195E-06	0,255939866	0,378	0,126	0,117784784	1,194113413	0,837441393	3
TM9SF1	9,01084E-10	0,255800109	0,402	0,095	1,90138E-05	1,193997743	0,837522521	4,231578947
DPY30	4,03438E-10	0,255649014	0,463	0,116	8,51294E-06	1,1938727	0,837610241	3,99137931
KCTD15	1,86304E-06	0,255584306	0,256	0,063	0,039312064	1,193819154	0,83764781	4,063492063
ZNF32	2,20768E-07	0,255544481	0,341	0,091	0,004658418	1,193786199	0,837670933	3,747252747
HKDC1	2,8576E-10	0,255517331	0,207	0,018	6,02982E-06	1,193763734	0,837686698	11,5
UBE2J2	1,81484E-07	0,255435159	0,341	0,091	0,0038295	1,193695743	0,837734411	3,747252747
ST6GAL1	1,35285E-05	0,255320214	0,366	0,13	0,285464448	1,19360064	0,837801159	2,815384615
CBX3	0,000194481	0,254953263	0,634	0,344	1	1,193297085	0,838014282	1,843023256
ADI1	6,18412E-06	0,254814264	0,451	0,175	0,1304911	1,19318212	0,838095026	2,577142857
ABCA3	0,000177614	0,253979989	0,171	0,046	1	1,192492332	0,838579816	3,717391304
GALNT11	2,55606E-07	0,253946721	0,329	0,088	0,005393547	1,192464833	0,838599154	3,738636364
HSPBP1	1,68863E-09	0,253914296	0,28	0,046	3,56318E-05	1,192438033	0,838618001	6,086956522
F8A1	4,91932E-09	0,253802009	0,232	0,032	0,000103803	1,192345228	0,838683275	7,25
TMEM50A	6,78376E-05	0,253731102	0,756	0,389	1	1,192286626	0,838724496	1,94344473
NUDT9	1,32939E-08	0,253615287	0,28	0,053	0,000280515	1,192190917	0,838791829	5,283018868
SPATC1L	1,18545E-11	0,25360906	0,329	0,049	2,50142E-07	1,192185771	0,838795449	6,714285714
RAB2A	8,0578E-06	0,253493085	0,756	0,372	0,170027667	1,192089938	0,838862881	2,032258065
EIF5A	0,000928199	0,253199401	0,695	0,411	1	1,191847293	0,839033663	1,690997567
UBXN1	0,00032027	0,253100817	0,598	0,312	1	1,191765853	0,839090999	1,916666667
SLC27A4	1,30012E-12	0,252951334	0,22	0,011	2,74338E-08	1,191642377	0,839177944	20

Supplementary Table 2

Differentially expressed genes_keloid-XL_ comparing SC-Keloid vs. (SC-Myel, SC-Nonmyel, SC-Nonmyel2)								
**gene**	**p_val**	**avg_log2FC**	**pct.1**	**pct.2**	**p_val_adj**	**Foldchange_UP**	**Foldchange_DOWN**	**Ratio_pct1_pct2**
ZFAND3	4,55581E-07	0,252910766	0,451	0,151	0,00961321	1,191608868	0,839201542	2,986754967
HES6	1,79015E-06	0,252801142	0,305	0,084	0,03777386	1,191518327	0,839265311	3,630952381
FAM174B	1,00204E-07	0,252723238	0,22	0,035	0,002114405	1,191453988	0,839310632	6,285714286
ACOT7	1,09934E-12	0,252477849	0,415	0,074	2,31971E-08	1,19125135	0,839453403	5,608108108
ECI1	4,13192E-08	0,252377065	0,463	0,144	0,000871877	1,191168134	0,839512048	3,215277778
FIBP	1,93996E-08	0,252332087	0,366	0,095	0,000409351	1,191130998	0,839538222	3,852631579
CHRNB1	0,002631406	0,252122781	0,085	0,018	1	1,190958202	0,83966003	4,722222222
DDX54	3,37847E-06	0,252089066	0,28	0,077	0,071289034	1,190930371	0,839679653	3,636363636
CYC1	2,68356E-05	0,251826581	0,488	0,2	0,566258295	1,190713711	0,839832439	2,44
RIC8A	4,65274E-10	0,251638517	0,329	0,063	9,81774E-06	1,190558505	0,839941923	5,222222222
HIVEP3	6,60534E-06	0,251413406	0,171	0,032	0,139379264	1,19037275	0,840072994	5,34375
RPL23	0,005692361	0,251327931	0,915	0,667	1	1,190302226	0,840122767	1,371814093
MRPS11	6,79963E-10	0,251095913	0,341	0,067	1,43479E-05	1,190110815	0,840257888	5,089552239
TOR1A	4,45779E-09	0,250847356	0,244	0,035	9,40638E-05	1,189905792	0,840402666	6,971428571
YIPF3	3,96615E-08	0,250818627	0,5	0,165	0,000836897	1,189882097	0,840419402	3,03030303
TRIM27	0,001146878	0,250789347	0,183	0,063	1	1,189857948	0,840436458	2,904761905
VPS16	6,80875E-07	0,25063896	0,171	0,025	0,01436714	1,189733924	0,84052407	6,84
HSD17B10	2,90327E-07	0,250607927	0,476	0,158	0,006126187	1,189708332	0,840542151	3,012658228
NME3	2,11061E-05	0,250550191	0,646	0,295	0,445360491	1,189660721	0,84057579	2,189830508
MRPL9	1,47593E-06	0,250518427	0,244	0,056	0,031143703	1,189634529	0,840594297	4,357142857
IFT43	9,08244E-09	0,250491178	0,28	0,053	0,000191649	1,18961206	0,840610174	5,283018868
PPP3CB	8,64303E-09	0,250443931	0,366	0,088	0,000182377	1,189573102	0,840637703	4,159090909
COMMD10	1,68794E-08	0,250435876	0,232	0,035	0,000356172	1,18956646	0,840642397	6,628571429
PDCD2	3,81283E-11	0,250298818	0,341	0,056	8,04545E-07	1,189453455	0,840722263	6,089285714
FEZ1	3,5617E-05	0,250018648	0,524	0,228	0,751554721	1,189222486	0,840885546	2,298245614
S100B	0,02040716	0,250001068	1	0,87	1	1,189207995	0,840895793	1,149425287
DDA1	6,11497E-09	0,249855956	0,366	0,084	0,000129032	1,189088386	0,840980378	4,357142857
RPAIN	2,35718E-06	0,249489366	0,524	0,2	0,049738895	1,188786276	0,841194099	2,62
REXO2	1,10359E-08	0,24904416	0,463	0,137	0,000232868	1,188419481	0,841453725	3,379562044
NNT-AS1	0,00010162	0,24901587	0,171	0,042	1	1,188396177	0,841470226	4,071428571

Supplementary Table 2

Differentially expressed genes_keloid-XL_ comparing SC-Keloid vs. (SC-Myel, SC-Nonmyel, SC-Nonmyel2)								
**gene**	**p_val**	**avg_log2FC**	**pct.1**	**pct.2**	**p_val_adj**	**Foldchange_UP**	**Foldchange_DOWN**	**Ratio_pct1_pct2**
RPL19	0,009043395	0,24883965	0,976	0,916	1	1,188251028	0,841573015	1,065502183
TAX1BP1	1,74044E-06	0,248715059	0,634	0,263	0,036725011	1,188148416	0,841645696	2,410646388
GOSR2	3,03746E-05	0,248314313	0,195	0,049	0,640934995	1,187818423	0,841879517	3,979591837
BCL7B	5,89042E-07	0,24829507	0,415	0,133	0,012429366	1,187802579	0,841890747	3,120300752
ANKRD13D	6,67722E-07	0,24810963	0,317	0,088	0,014089604	1,187649912	0,841998968	3,602272727
CNPY4	7,2721E-09	0,247950665	0,207	0,025	0,000153449	1,187519056	0,84209175	8,28
POP7	7,70775E-08	0,247749466	0,317	0,077	0,001626412	1,187353456	0,842209196	4,116883117
NDFIP1	0,000343944	0,247620323	0,61	0,323	1	1,187247175	0,84228459	1,888544892
RTCA	2,16672E-07	0,247474999	0,171	0,021	0,004571988	1,187127588	0,842369438	8,142857143
ZNF280D	0,0001721	0,24733175	0,28	0,102	1	1,187009721	0,842453084	2,745098039
IRF9	1,42215E-06	0,246907486	0,39	0,13	0,030008763	1,1866607	0,842700867	3
CILK1	1,26827E-08	0,246862688	0,317	0,067	0,000267618	1,186623852	0,842727034	4,731343284
PCYT1A	9,74502E-11	0,246698707	0,305	0,046	2,0563E-06	1,186488985	0,842822826	6,630434783
PREB	4,14983E-09	0,246620437	0,232	0,032	8,75655E-05	1,186424616	0,842868553	7,25
ALYREF	6,58939E-09	0,24651196	0,366	0,088	0,000139043	1,186335412	0,842931931	4,159090909
IKBIP	5,35595E-06	0,246323019	0,22	0,053	0,11301595	1,186180055	0,843042332	4,150943396
PGGT1B	2,53936E-05	0,246061343	0,207	0,053	0,535831396	1,185964925	0,843195257	3,905660377
SPATA7	9,75345E-06	0,245934426	0,11	0,011	0,205807544	1,185860598	0,843269438	10
KATNB1	7,64681E-12	0,245731789	0,207	0,011	1,61355E-07	1,185694047	0,84338789	18,81818182
AGK	5,3647E-11	0,245730714	0,256	0,028	1,13201E-06	1,185693164	0,843388518	9,142857143
LAPTM4B	7,53627E-07	0,245578604	0,28	0,07	0,015902288	1,185568157	0,843477445	4
DHX57	0,037317289	0,245296358	0,073	0,025	1	1,185336237	0,843642477	2,92
OGT	0,000121938	0,245097364	0,329	0,126	1	1,185172753	0,843758851	2,611111111
GPATCH8	4,74119E-05	0,244902449	0,39	0,147	1	1,185012641	0,843872855	2,653061224
EI24	1,705E-07	0,244722375	0,463	0,147	0,003597712	1,18486474	0,843978191	3,149659864
CNOT3	1,31579E-06	0,244501878	0,244	0,056	0,027764392	1,184683662	0,844107192	4,357142857
FARSB	4,12755E-11	0,244069536	0,268	0,032	8,70954E-07	1,184328694	0,844360189	8,375
SNRNP40	6,07476E-09	0,243922218	0,268	0,046	0,000128183	1,184207765	0,844446414	5,826086957
C5orf51	9,41876E-06	0,243859838	0,171	0,032	0,198745195	1,184156562	0,844482927	5,34375
MON2	6,33638E-08	0,243794103	0,256	0,049	0,00133704	1,184102608	0,844521407	5,224489796

Supplementary Table 2

Differentially expressed genes_keloid-XL_ comparing SC-Keloid vs. (SC-Myel, SC-Nonmyel, SC-Nonmyel2)								
gene	p_val	avg_log2FC	pct.1	pct.2	p_val_adj	Foldchange_UP	Foldchange_DOWN	Ratio_pct1_pct2
RAI14	8,69881E-05	0,243609234	0,268	0,091	1	1,183950886	0,844629631	2,945054945
PRAG1	4,279E-08	0,243416451	0,195	0,025	0,000902911	1,183792688	0,844742504	7,8
ACADVL	4,52618E-06	0,243333365	0,756	0,354	0,09550683	1,183724514	0,844791155	2,13559322
AGPS	1,4818E-10	0,243331718	0,317	0,053	3,12675E-06	1,183723163	0,844792119	5,981132075
PSMD2	1,22103E-06	0,243315135	0,463	0,168	0,025765029	1,183709557	0,84480183	2,755952381
AIMP1	3,346E-07	0,24318266	0,476	0,161	0,007060403	1,183600869	0,844879407	2,956521739
EEF1E1	2,49987E-12	0,24275793	0,415	0,077	5,27498E-08	1,183252467	0,845128177	5,38961039
GPS1	1,07345E-08	0,24262779	0,415	0,112	0,000226508	1,183145735	0,845204416	3,705357143
GCSH	1,5444E-05	0,242421268	0,549	0,232	0,325884619	1,18297638	0,845325416	2,36637931
DESI1	2,37451E-09	0,242393449	0,305	0,056	5,01045E-05	1,182953569	0,845341716	5,446428571
OGFOD3	4,86934E-08	0,242269276	0,232	0,039	0,00102748	1,182851757	0,845414478	5,948717949
FAF1	2,71759E-07	0,242264238	0,268	0,06	0,005734397	1,182847626	0,84541743	4,466666667
KXD1	1,48565E-08	0,242166011	0,427	0,119	0,000313487	1,182767094	0,845474993	3,588235294
NDN	1,38887E-07	0,24148909	0,268	0,056	0,002930647	1,182212262	0,845871788	4,785714286
CYP27A1	1,87107E-05	0,241481273	0,232	0,063	0,39481551	1,182205857	0,845876371	3,682539683
HACD2	3,15221E-05	0,241299946	0,232	0,063	0,665147428	1,182057279	0,845982693	3,682539683
SIPA1L3	3,36918E-06	0,241264319	0,159	0,025	0,071092969	1,182028089	0,846003584	6,36
CTBP1	1,68966E-06	0,241131899	0,439	0,154	0,035653436	1,1819196	0,846081239	2,850649351
ERLIN1	3,50888E-05	0,241078156	0,207	0,053	0,740408865	1,181875572	0,846112758	3,905660377
WSB2	1,39021E-08	0,240924851	0,415	0,112	0,000293348	1,181749989	0,846202673	3,705357143
MAN2B2	7,55582E-06	0,240856189	0,256	0,07	0,159435276	1,181693747	0,846242948	3,657142857
TACO1	6,18103E-08	0,24085447	0,159	0,014	0,00130426	1,18169234	0,846243956	11,35714286
SRA1	8,78189E-09	0,240756164	0,293	0,056	0,000185307	1,181611821	0,846301622	5,232142857
EFHD2	6,51225E-09	0,240687297	0,402	0,102	0,000137415	1,181555418	0,84634202	3,941176471
TFG	0,000658476	0,240484136	0,488	0,242	1	1,181389042	0,846461211	2,016528926
C1QTNF1	0,000129099	0,240447575	0,11	0,018	1	1,181359104	0,846482663	6,111111111
SDC1	5,30439E-09	0,240166099	0,134	0,004	0,000111928	1,181128638	0,846647831	33,5
OGDH	5,25076E-05	0,240155735	0,256	0,081	1	1,181120153	0,846653913	3,160493827
ESS2	7,4007E-05	0,240130886	0,134	0,025	1	1,18109981	0,846668496	5,36
KCTD10	2,77201E-06	0,239937232	0,171	0,028	0,058492215	1,180941281	0,846782153	6,107142857

Supplementary Table 2

Differentially expressed genes_keloid-XL_ comparing SC-Keloid vs. (SC-Myel, SC-Nonmyel, SC-Nonmyel2)								
**gene**	**p_val**	**avg_log2FC**	**pct.1**	**pct.2**	**p_val_adj**	**Foldchange_UP**	**Foldchange_DOWN**	**Ratio_pct1_pct2**
PAIP1	4,67089E-07	0,239810707	0,439	0,144	0,009856035	1,180837716	0,84685642	3,048611111
ATP5MC1	8,00609E-05	0,239467992	0,537	0,246	1	1,180557239	0,847057615	2,182926829
RTCB	1,07084E-09	0,239296888	0,28	0,046	2,25957E-05	1,180417233	0,847158083	6,086956522
KLHDC2	1,05184E-06	0,239247116	0,305	0,084	0,022194871	1,18037651	0,84718731	3,630952381
CAPN5	1,01265E-06	0,239125124	0,256	0,06	0,021367881	1,180276704	0,847258949	4,266666667
ARL8A	2,0662E-06	0,238979394	0,61	0,242	0,04359881	1,180157487	0,847344537	2,520661157
STIM1	1,77642E-08	0,238855208	0,341	0,081	0,000374842	1,180055904	0,847417479	4,209876543
COPS4	6,03701E-12	0,238788168	0,28	0,032	1,27387E-07	1,18000107	0,847456859	8,75
DGAT1	2,5942E-05	0,23877113	0,207	0,053	0,547402383	1,179987135	0,847466867	3,905660377
MSI2	1,38251E-08	0,238597926	0,28	0,053	0,000291722	1,179845479	0,847568616	5,283018868
NSDHL	2,92786E-07	0,238472996	0,122	0,007	0,006178086	1,179743315	0,847642015	17,42857143
CDC26	1,16283E-07	0,238315082	0,317	0,077	0,00245368	1,17961419	0,847734801	4,116883117
USP18	2,18116E-07	0,238196639	0,11	0,004	0,004602468	1,17951735	0,847804401	27,5
EFCAB11	5,47929E-05	0,237936349	0,085	0,007	1	1,179304561	0,847957375	12,14285714
TRIM26	2,65511E-07	0,237769362	0,207	0,035	0,005602541	1,179168068	0,848055529	5,914285714
TRADD	7,1242E-11	0,237737286	0,232	0,021	1,50328E-06	1,179141852	0,848074384	11,04761905
BTBD1	1,50549E-06	0,23766171	0,28	0,074	0,031767255	1,179080083	0,848118813	3,783783784
JAGN1	6,40582E-09	0,237649142	0,28	0,049	0,000135169	1,179069812	0,8481262	5,714285714
HDHD2	4,24684E-10	0,236964399	0,28	0,042	8,96126E-06	1,178510326	0,84852884	6,666666667
MMGT1	2,72034E-07	0,236920916	0,329	0,088	0,005740184	1,178474806	0,848554416	3,738636364
GOLGA5	2,85515E-09	0,236888042	0,256	0,039	6,02466E-05	1,178447953	0,848573751	6,564102564
RHOQ	1,93718E-06	0,236785055	0,646	0,27	0,040876361	1,178363832	0,848634329	2,392592593
SCRIB	2,43784E-05	0,236726216	0,305	0,102	0,514407964	1,178315775	0,84866894	2,990196078
MTHFD2	2,82336E-06	0,236633913	0,268	0,07	0,059575721	1,178240389	0,84872324	3,828571429
FLOT2	1,42781E-05	0,236515233	0,195	0,046	0,30128174	1,178143468	0,848793061	4,239130435
SLC39A10	2,02994E-11	0,236256593	0,427	0,088	4,28338E-07	1,177932274	0,848945243	4,852272727
TCHP	1,01816E-07	0,236100915	0,207	0,032	0,002148429	1,177805173	0,849036855	6,46875
CYP1B1	0,043000486	0,236001253	0,037	0,007	1	1,177723812	0,849095509	5,285714286
EXOSC8	2,13127E-06	0,23593156	0,317	0,091	0,044971967	1,177666921	0,849136528	3,483516484
ABCC3	2,26856E-10	0,235867904	0,171	0,007	4,78689E-06	1,17761496	0,849173995	24,42857143

Supplementary Table 2

Differentially expressed genes_keloid-XL_ comparing SC-Keloid vs. (SC-Myel, SC-Nonmyel, SC-Nonmyel2)								
gene	p_val	avg_log2FC	pct.1	pct.2	p_val_adj	Foldchange_UP	Foldchange_DOWN	Ratio_pct1_pct2
DGCR2	2,45791E-05	0,235628835	0,28	0,088	0,518643421	1,177419833	0,849314723	3,181818182
CYBC1	3,4708E-07	0,235620391	0,256	0,056	0,007323729	1,177412942	0,849319694	4,571428571
LIG3	2,1468E-05	0,235553426	0,134	0,021	0,452995664	1,177358292	0,849359118	6,380952381
MTDH	0,000109705	0,235508097	0,732	0,382	1	1,1773213	0,849385805	1,916230366
SSNA1	2,46646E-08	0,235422811	0,427	0,123	0,000520448	1,177251704	0,849436019	3,471544715
RARS2	1,27595E-08	0,235183287	0,232	0,035	0,000269237	1,177056267	0,849577058	6,628571429
IFNAR2	2,57425E-07	0,235099253	0,329	0,084	0,005431932	1,176987708	0,849626545	3,916666667
PMVK	1,60401E-07	0,234898496	0,378	0,109	0,003384618	1,176823936	0,849744783	3,467889908
NIFK-AS1	3,22222E-07	0,234568143	0,146	0,014	0,006799216	1,176554494	0,849939382	10,42857143
MSC	4,23876E-13	0,234544791	0,195	0,004	8,9442E-09	1,17653545	0,84995314	48,75
PCDHGA3	0,000183568	0,234481354	0,049	0	1	1,176483718	0,849990514	#DIV/0!
C14orf119	2,07731E-10	0,234469556	0,305	0,049	4,38333E-06	1,176474097	0,849997465	6,224489796
STOML2	3,92798E-08	0,234457064	0,378	0,102	0,000828843	1,17646391	0,850004825	3,705882353
YWHAZ	0,015878117	0,23425189	0,829	0,582	1	1,17629661	0,850125718	1,424398625
ANAPC13	1,46236E-08	0,234155128	0,378	0,095	0,000308573	1,176217718	0,850182738	3,978947368
METTL25B	1,02952E-07	0,233840865	0,098	0	0,002172399	1,17596153	0,850367954	#DIV/0!
EBP	1,06374E-07	0,233796068	0,207	0,032	0,002244596	1,175925016	0,850394359	6,46875
PDHA1	1,84634E-08	0,233777833	0,317	0,07	0,000389597	1,175910153	0,850405108	4,528571429
OTUD5	2,14719E-06	0,233556805	0,329	0,095	0,045307852	1,175730011	0,850535404	3,463157895
POLR2F	2,11437E-06	0,233538127	0,72	0,309	0,044615222	1,17571479	0,850546415	2,330097087
PHTF2	3,51943E-06	0,233429809	0,159	0,025	0,074263512	1,17562652	0,850610277	6,36
C1orf131	2,8975E-09	0,233267282	0,256	0,039	6,11401E-05	1,175494087	0,850706108	6,564102564
PPP1R8	1,61686E-07	0,233251702	0,195	0,028	0,003411727	1,175481392	0,850715295	6,964285714
RPL31	0,018651435	0,233223933	0,866	0,681	1	1,175458768	0,85073167	1,271659325
MRPL12	4,15604E-09	0,233171938	0,305	0,06	8,76965E-05	1,175416404	0,850762331	5,083333333
TRAPPC6A	0,000145935	0,232970259	0,183	0,049	1	1,175252101	0,85088127	3,734693878
CUL4A	1,83332E-06	0,232866251	0,244	0,056	0,038684881	1,175167376	0,850942615	4,357142857
PON2	7,53218E-05	0,232783731	0,598	0,27	1	1,17510016	0,850991289	2,214814815
C11orf58	0,000677693	0,2327322	0,671	0,365	1	1,175058188	0,851021686	1,838356164
ITPR3	2,70578E-06	0,232710928	0,524	0,207	0,057094629	1,175040863	0,851034233	2,531400966

Supplementary Table 2

Differentially expressed genes_keloid-XL_ comparing SC-Keloid vs. (SC-Myel, SC-Nonmyel, SC-Nonmyel2)								
**gene**	**p_val**	**avg_log2FC**	**pct.1**	**pct.2**	**p_val_adj**	**Foldchange_UP**	**Foldchange_DOWN**	**Ratio_pct1_pct2**
STX5	1,11067E-07	0,232598371	0,293	0,067	0,002343632	1,174949191	0,851100633	4,373134328
LINC00667	3,62981E-06	0,232327204	0,341	0,105	0,076592544	1,17472837	0,85126062	3,247619048
HP1BP3	0,00345878	0,232321698	0,72	0,414	1	1,174723887	0,851263868	1,739130435
ITPK1	0,000104007	0,232245583	0,707	0,354	1	1,174661911	0,851308781	1,997175141
WDR27	0,000915822	0,231968301	0,085	0,014	1	1,174436166	0,851472416	6,071428571
LSM1	1,55679E-08	0,231909974	0,415	0,112	0,000328499	1,174388686	0,851506841	3,705357143
ARL6IP6	2,59526E-06	0,231431217	0,427	0,151	0,05476258	1,17399903	0,85178946	2,82781457
STUB1	2,23119E-06	0,231319765	0,463	0,165	0,047080442	1,173908339	0,851855265	2,806060606
CIT	0,001187461	0,231310787	0,463	0,228	1	1,173901034	0,851860566	2,030701754
APH1A	5,24385E-08	0,231271742	0,415	0,119	0,001106505	1,173869264	0,851883622	3,487394958
SRPRB	1,44041E-10	0,231241633	0,317	0,053	3,03941E-06	1,173844766	0,8519014	5,981132075
NANS	4,17549E-07	0,231033769	0,329	0,091	0,008810694	1,17367565	0,852024152	3,615384615
POLR2K	8,10671E-06	0,230948255	0,61	0,263	0,171059792	1,173606084	0,852074656	2,319391635
RPL18A	0,048687854	0,230785146	0,988	0,874	1	1,173473405	0,852170996	1,130434783
ENDOD1	1,59252E-07	0,230782608	0,61	0,221	0,003360382	1,173471341	0,852172495	2,760180995
DUSP10	1,66557E-13	0,230729147	0,305	0,032	3,51453E-09	1,173427857	0,852204074	9,53125
GLRX3	8,81576E-07	0,23067076	0,329	0,095	0,018602126	1,173380368	0,852238564	3,463157895
POLR1A	3,22222E-07	0,230616231	0,146	0,014	0,006799216	1,17333602	0,852270776	10,42857143
PRPF19	4,23218E-08	0,230598831	0,232	0,039	0,000893032	1,173321868	0,852281055	5,948717949
PPP1R11	3,44039E-06	0,230487948	0,244	0,06	0,072595679	1,173231693	0,852346562	4,066666667
SLITRK6	2,7748E-06	0,230428059	0,573	0,228	0,058551153	1,17318299	0,852381946	2,513157895
TCF4	2,05906E-05	0,23024687	0,72	0,337	0,434481595	1,173035659	0,852489004	2,136498516
TBC1D19	1,6416E-05	0,230185954	0,146	0,025	0,346394762	1,172986129	0,852525	5,84
KIFAP3	6,16527E-08	0,230152903	0,39	0,105	0,001300933	1,172959258	0,852544531	3,714285714
MORN4	1,33474E-06	0,229635389	0,146	0,018	0,028164367	1,172538578	0,852850404	8,111111111
GNL1	5,86151E-07	0,229392615	0,366	0,109	0,012368368	1,172341281	0,852993933	3,357798165
DCTN1	1,49822E-06	0,2292438	0,402	0,13	0,031614038	1,17222036	0,853081924	3,092307692
ZNF226	5,21699E-11	0,228994584	0,207	0,014	1,10084E-06	1,172017884	0,853229301	14,78571429
EAPP	6,069E-06	0,228942479	0,39	0,137	0,128061934	1,171975555	0,853260117	2,846715328
ACYP2	8,1914E-06	0,228904034	0,256	0,07	0,17284666	1,171944325	0,853282855	3,657142857

Supplementary Table 2

Differentially expressed genes_keloid-XL_ comparing SC-Keloid vs. (SC-Myel, SC-Nonmyel, SC-Nonmyel2)								
**gene**	**p_val**	**avg_log2FC**	**pct.1**	**pct.2**	**p_val_adj**	**Foldchange_UP**	**Foldchange_DOWN**	**Ratio_pct1_pct2**
RPF2	4,41936E-08	0,228886634	0,28	0,06	0,00093253	1,17193019	0,853293147	4,666666667
IQGAP1	6,51352E-05	0,228886588	0,634	0,302	1	1,171930153	0,853293174	2,099337748
COA4	5,01244E-08	0,228804909	0,427	0,126	0,001057676	1,171863806	0,853341485	3,388888889
NRP1	6,23082E-08	0,228453759	0,354	0,091	0,001314765	1,17157861	0,853549212	3,89010989
NT5C3B	1,02014E-05	0,228414945	0,28	0,084	0,215259219	1,171547091	0,853572176	3,333333333
TRMT112	0,003021431	0,228327805	0,732	0,467	1	1,17147633	0,853623735	1,56745182
GAR1	4,45121E-11	0,22799367	0,256	0,028	9,39249E-07	1,171205043	0,85382146	9,142857143
SDHAF3	1,09597E-08	0,227945296	0,317	0,067	0,000231262	1,171165772	0,85385009	4,731343284
NAV1	4,77528E-07	0,22793059	0,378	0,112	0,010076326	1,171153834	0,853858794	3,375
MYO19	1,08035E-06	0,227910111	0,159	0,021	0,022796397	1,17113721	0,853870914	7,571428571
GAA	8,67253E-05	0,227901481	0,427	0,175	1	1,171130204	0,853876022	2,44
MFSD12	7,05026E-06	0,227646202	0,463	0,175	0,148767611	1,170922996	0,854027125	2,645714286
ARF3	1,69606E-07	0,227486516	0,402	0,119	0,003578854	1,170793398	0,854121659	3,378151261
ANXA4	3,12549E-05	0,227339944	0,415	0,165	0,659509823	1,170674456	0,854208439	2,515151515
RTL8C	1,05257E-05	0,227252784	0,549	0,228	0,222102989	1,170603733	0,854260047	2,407894737
DDB1	3,03493E-06	0,227110604	0,427	0,147	0,064040084	1,170488373	0,85434424	2,904761905
HSPB3	0,000183568	0,226858092	0,049	0	1	1,170283523	0,854493787	#DIV/0!
SCN1B	0,000150458	0,226789564	0,183	0,049	1	1,170227936	0,854534377	3,734693878
DMAC1	1,60803E-07	0,22655544	0,305	0,074	0,003393111	1,170038044	0,854673064	4,121621622
PARL	6,69648E-09	0,226516823	0,329	0,07	0,000141302	1,170006726	0,854695941	4,7
SNAI2	4,66435E-13	0,226507134	0,195	0,004	9,84225E-09	1,169998868	0,854701681	48,75
KCTD17	3,82684E-10	0,226468355	0,232	0,025	8,07502E-06	1,169967419	0,854724656	9,28
NDUFB9	2,30981E-10	0,22639909	0,561	0,161	4,87392E-06	1,16991125	0,854765693	3,48447205
ZFPM1	2,83891E-08	0,226221398	0,329	0,077	0,000599039	1,169767165	0,854870978	4,272727273
BAX	9,96265E-08	0,226189277	0,537	0,189	0,002102219	1,16974112	0,854890012	2,841269841
SLC9A7	8,40386E-12	0,226160731	0,159	0	1,7733E-07	1,169717975	0,854906927	#DIV/0!
GCFC2	1,44381E-06	0,226141344	0,195	0,035	0,030465921	1,169702257	0,854918415	5,571428571
HADHB	1,90575E-05	0,226043047	0,476	0,193	0,40213175	1,169622562	0,854976667	2,466321244
KCNG1	1,17656E-05	0,226027309	0,232	0,06	0,248265924	1,169609803	0,854985994	3,866666667
ARNT2	5,03918E-07	0,225999878	0,183	0,028	0,01063317	1,169587566	0,85500225	6,535714286

Supplementary Table 2

Differentially expressed genes_keloid-XL_ comparing SC-Keloid vs. (SC-Myel, SC-Nonmyel, SC-Nonmyel2)								
**gene**	**p_val**	**avg_log2FC**	**pct.1**	**pct.2**	**p_val_adj**	**Foldchange_UP**	**Foldchange_DOWN**	**Ratio_pct1_pct2**
ATF6	3,09191E-07	0,225922128	0,268	0,06	0,00652424	1,169524535	0,85504833	4,466666667
COMMD4	9,64312E-11	0,225868231	0,463	0,112	2,03479E-06	1,169480844	0,855080273	4,133928571
HAX1	5,53789E-07	0,225822245	0,329	0,091	0,011685504	1,169443568	0,855107529	3,615384615
CDK5RAP2	2,47738E-06	0,225348154	0,232	0,053	0,052275269	1,169059334	0,855388577	4,377358491
DGKI	5,5905E-11	0,225327266	0,146	0	1,17965E-06	1,169042408	0,855400961	#DIV/0!
NR2C2AP	4,23186E-07	0,22519752	0,22	0,042	0,008929644	1,168937277	0,855477894	5,238095238
RPL10	0,009883443	0,225126923	1	0,954	1	1,168880078	0,855519757	1,048218029
EPHA3	1,02952E-07	0,225102176	0,098	0	0,002172399	1,168860028	0,855534432	#DIV/0!
IAH1	0,000383552	0,225046224	0,585	0,298	1	1,168814697	0,855567613	1,963087248
GPR153	0,001789861	0,224786213	0,195	0,07	1	1,168604065	0,855721822	2,785714286
RO60	5,52939E-07	0,224719964	0,573	0,214	0,011667575	1,168550404	0,855761118	2,677570093
EMCN	0,00032041	0,224718299	0,073	0,007	1	1,168549055	0,855762106	10,42857143
PRKAB2	0,002984434	0,224657821	0,134	0,042	1	1,168500071	0,85579798	3,19047619
ABHD12	1,62836E-06	0,224458137	0,415	0,137	0,034360006	1,168338349	0,85591644	3,02919708
RPL6	0,015686261	0,224413711	0,951	0,87	1	1,168302372	0,855942797	1,093103448
DR1	4,09471E-05	0,224345985	0,329	0,119	0,864024304	1,168247528	0,855982979	2,764705882
HSD17B11	2,68582E-07	0,22428466	0,268	0,06	0,005667358	1,168197871	0,856019366	4,466666667
ITPA	1,32141E-06	0,224228133	0,427	0,144	0,027883002	1,1681521	0,856052906	2,965277778
ADAMTS6	6,67422E-07	0,224217685	0,085	0	0,014083281	1,16814364	0,856059106	#DIV/0!
RAB5B	0,01756438	0,224129122	0,183	0,088	1	1,168071933	0,856111659	2,079545455
ZNF783	0,000640402	0,223945747	0,098	0,018	1	1,167923473	0,856220483	5,444444444
XRCC4	3,3975E-07	0,223919723	0,146	0,014	0,007169057	1,167902406	0,856235928	10,42857143
SARS1	5,7489E-05	0,223804621	0,549	0,239	1	1,167809231	0,856304243	2,29707113
CAV2	7,59676E-05	0,223794649	0,439	0,179	1	1,16780116	0,856310162	2,452513966
NRAS	1,36458E-08	0,223733803	0,28	0,053	0,000287939	1,167751908	0,856346278	5,283018868
CYRIB	2,41327E-06	0,223732768	0,268	0,07	0,050922305	1,167751071	0,856346892	3,828571429
LBX2-AS1	1,86382E-06	0,223087671	0,122	0,011	0,039328534	1,167229031	0,85672989	11,09090909
TMEM14A	5,64889E-07	0,222918946	0,439	0,144	0,011919723	1,16709253	0,856830092	3,048611111
NDUFV3	2,70723E-05	0,222818846	0,317	0,109	0,571252879	1,167011555	0,856889545	2,908256881
BOP1	2,14886E-10	0,222727966	0,244	0,028	4,53431E-06	1,166938044	0,856943525	8,714285714

Supplementary Table 2

Differentially expressed genes_keloid-XL_ comparing SC-Keloid vs. (SC-Myel, SC-Nonmyel, SC-Nonmyel2)								
gene	p_val	avg_log2FC	pct.1	pct.2	p_val_adj	Foldchange_UP	Foldchange_DOWN	Ratio_pct1_pct2
CKLF	3,92935E-08	0,222717173	0,439	0,126	0,000829133	1,166929313	0,856949936	3,484126984
TATDN1	2,26578E-07	0,222405284	0,256	0,053	0,004781017	1,166677068	0,857135215	4,830188679
SPOCD1	6,10551E-11	0,222399642	0,207	0,014	1,28832E-06	1,166672505	0,857138567	14,78571429
C3orf38	0,000325846	0,222274567	0,183	0,053	1	1,166571365	0,857212881	3,452830189
NAA60	7,7401E-07	0,222059709	0,293	0,074	0,016332389	1,166397642	0,857340553	3,959459459
UBA1	4,08995E-06	0,221915647	0,537	0,207	0,086302106	1,166281177	0,857426168	2,594202899
PIGS	1,03424E-05	0,221830333	0,22	0,053	0,218233962	1,16621221	0,857476874	4,150943396
LAMTOR5	0,000163737	0,221825942	0,732	0,407	1	1,16620866	0,857479484	1,798525799
GPSM3	9,76427E-12	0,221551779	0,232	0,018	2,06036E-07	1,165987061	0,85764245	12,88888889
TP53I3	1,85115E-08	0,221503589	0,293	0,06	0,000390611	1,165948115	0,857671098	4,883333333
STN1	4,91932E-09	0,221398757	0,232	0,032	0,000103803	1,165863395	0,857733423	7,25
RNF14	8,10174E-08	0,22139646	0,22	0,035	0,001709548	1,165861539	0,857734788	6,285714286
BAG6	9,41071E-08	0,221298763	0,5	0,165	0,001985753	1,165782591	0,857792875	3,03030303
CPSF2	9,83374E-05	0,220853667	0,171	0,042	1	1,165422983	0,858057559	4,071428571
VAMP7	3,32918E-06	0,220811401	0,244	0,06	0,070248972	1,16538884	0,858082698	4,066666667
BIN3	3,1358E-11	0,220685342	0,305	0,042	6,61686E-07	1,165287016	0,858157678	7,261904762
PTTG1IP	1,90618E-05	0,22063493	0,671	0,316	0,40222353	1,165246298	0,858187665	2,123417722
TMEM134	3,92734E-07	0,220593074	0,354	0,102	0,008287076	1,165212492	0,858212563	3,470588235
MAPKAPK5-AS1	2,09583E-07	0,220533955	0,268	0,06	0,004422404	1,165164745	0,858247732	4,466666667
TOMM40	8,70092E-11	0,220470561	0,378	0,074	1,83598E-06	1,165113547	0,858285446	5,108108108
SNRPB2	2,05636E-06	0,220436115	0,622	0,253	0,043391198	1,16508573	0,858305938	2,458498024
CDK5	3,61835E-10	0,220314631	0,22	0,021	7,63508E-06	1,164987626	0,858378216	10,47619048
FPGS	1,35362E-08	0,220314116	0,232	0,035	0,000285628	1,16498721	0,858378523	6,628571429
TIMM29	0,002239651	0,220272768	0,159	0,053	1	1,164953822	0,858403124	3
ZMAT2	8,92651E-09	0,220033177	0,39	0,098	0,000188358	1,164760372	0,858545693	3,979591837
TXNL1	1,76866E-06	0,219955148	0,537	0,207	0,037320419	1,164697376	0,858592129	2,594202899
IFT74	0,001085403	0,219864447	0,159	0,049	1	1,164624155	0,858646109	3,244897959
ACBD6	2,86433E-05	0,219725206	0,329	0,112	0,604401632	1,164511758	0,858728985	2,9375
MPV17L2	3,36921E-06	0,219693754	0,159	0,025	0,071093597	1,164486371	0,858747706	6,36
CAPZA1	6,50826E-08	0,219398125	0,512	0,165	0,001373308	1,164247775	0,858923694	3,103030303

Supplementary Table 2

Differentially expressed genes_keloid-XL_ comparing SC-Keloid vs. (SC-Myel, SC-Nonmyel, SC-Nonmyel2)								
**gene**	**p_val**	**avg_log2FC**	**pct.1**	**pct.2**	**p_val_adj**	**Foldchange_UP**	**Foldchange_DOWN**	**Ratio_pct1_pct2**
C6orf47	2,31636E-07	0,219379875	0,159	0,018	0,004887745	1,164233047	0,85893456	8,833333333
CRAT	2,97058E-10	0,2192031	0,28	0,042	6,26822E-06	1,164090401	0,859039812	6,666666667
TIMM22	4,68278E-07	0,219080257	0,183	0,028	0,009881124	1,163991286	0,859112961	6,535714286
DAPK3	2,1731E-08	0,218936313	0,329	0,074	0,000458546	1,163875154	0,859198683	4,445945946
NIFK	1,37162E-07	0,21871903	0,39	0,112	0,002894247	1,163699878	0,859328096	3,482142857
DNAJC5	2,74168E-06	0,218696102	0,207	0,042	0,057852276	1,163681384	0,859341753	4,928571429
PIH1D1	1,65299E-09	0,218641262	0,268	0,042	3,48798E-05	1,16363715	0,859374419	6,380952381
PMF1	2,19361E-06	0,218622796	0,317	0,091	0,046287366	1,163622256	0,859385419	3,483516484
TUT7	0,011292245	0,218490172	0,159	0,067	1	1,163515292	0,859464424	2,373134328
RPAP3	0,000203227	0,21844169	0,171	0,046	1	1,163476192	0,859493307	3,717391304
TES	2,32149E-08	0,218399402	0,22	0,032	0,000489857	1,163442089	0,859518501	6,875
CCNT2	3,15003E-05	0,218236525	0,256	0,077	0,664688396	1,163310747	0,859615544	3,324675325
HEATR3	5,60121E-05	0,21821122	0,098	0,011	1	1,163290342	0,859630622	8,909090909
AGAP5	0,001876622	0,21816731	0,049	0,004	1	1,163254937	0,859656786	12,25
ATG7	7,34252E-09	0,218058217	0,22	0,028	0,000154935	1,163166978	0,859721793	7,857142857
TMEM185B	3,77042E-10	0,217856725	0,207	0,018	7,95596E-06	1,163004537	0,859841874	11,5
ZNF771	2,12559E-07	0,217702946	0,22	0,039	0,004485207	1,162880577	0,85993353	5,641025641
GNB1	0,000385961	0,217622543	0,707	0,432	1	1,162815771	0,859981457	1,636574074
ANXA7	5,34295E-07	0,217496685	0,585	0,211	0,011274163	1,162714333	0,860056483	2,772511848
FKBP11	3,24801E-06	0,217383159	0,195	0,039	0,068536275	1,162622843	0,860124164	5
AFAP1	8,92419E-08	0,217045929	0,354	0,091	0,001883093	1,162351111	0,860325242	3,89010989
INKA2	6,11901E-07	0,216963993	0,293	0,074	0,012911733	1,162285098	0,860374104	3,959459459
SV2A	1,8347E-06	0,216928299	0,11	0,007	0,038713925	1,162256343	0,860395391	15,71428571
RPS23	0,039800247	0,216839312	0,915	0,895	1	1,162184656	0,860448462	1,022346369
TBC1D9B	4,50613E-06	0,216658659	0,427	0,151	0,095083938	1,162039137	0,860556214	2,82781457
ANKRD10	0,011744583	0,216645236	0,878	0,667	1	1,162028326	0,86056422	1,316341829
VPS29	7,74748E-07	0,216508098	0,5	0,172	0,016347966	1,161917872	0,860646027	2,906976744
DGKZ	4,20027E-06	0,216497322	0,232	0,056	0,088629973	1,161909193	0,860652455	4,142857143
COMMD9	7,99158E-10	0,21633925	0,256	0,035	1,6863E-05	1,161781893	0,86074676	7,314285714
QDPR	3,53319E-06	0,216266819	0,341	0,105	0,074553904	1,161723567	0,860789975	3,247619048

Supplementary Table 2

Differentially expressed genes_keloid-XL_ comparing SC-Keloid vs. (SC-Myel, SC-Nonmyel, SC-Nonmyel2)								
gene	p_val	avg_log2FC	pct.1	pct.2	p_val_adj	Foldchange_UP	Foldchange_DOWN	Ratio_pct1_pct2
CCDC112	7,98662E-08	0,216212702	0,329	0,081	0,001685256	1,16167999	0,860822265	4,061728395
ALG13	3,10407E-09	0,216207285	0,293	0,053	6,54989E-05	1,161675629	0,860825497	5,528301887
CCDC66	0,000208146	0,2160298	0,256	0,091	1	1,161532724	0,860931405	2,813186813
POLR3K	1,31916E-09	0,21587368	0,232	0,028	2,78356E-05	1,161407036	0,861024575	8,285714286
SH3GLB1	2,1743E-07	0,215735732	0,646	0,256	0,004587986	1,161295991	0,861106908	2,5234375
NECAB3	9,92806E-06	0,215730434	0,11	0,011	0,209492051	1,161291726	0,861110071	10
CHMP1A	1,15223E-06	0,215589281	0,329	0,091	0,024313194	1,16117811	0,861194326	3,615384615
GDI2	1,05392E-06	0,2154851	0,61	0,249	0,022238787	1,161094262	0,861256517	2,449799197
SLC35A3	0,00039981	0,215391123	0,171	0,049	1	1,161018631	0,861312621	3,489795918
CREM	7,49796E-06	0,215333914	0,293	0,088	0,158214551	1,160972592	0,861346777	3,329545455
BPNT2	2,87861E-07	0,215154526	0,476	0,158	0,006074163	1,160828243	0,861453885	3,012658228
UGGT2	4,03486E-06	0,215032405	0,317	0,095	0,085139556	1,160729986	0,861526808	3,336842105
SH3KBP1	4,78358E-08	0,214645332	0,329	0,077	0,001009383	1,160418605	0,861757986	4,272727273
GTF3C1	5,08847E-08	0,214622049	0,354	0,091	0,001073718	1,160399878	0,861771894	3,89010989
DNAJC4	1,46971E-07	0,214490958	0,366	0,102	0,00310123	1,160294443	0,861850202	3,588235294
RMDN3	3,47825E-06	0,214383948	0,244	0,06	0,073394486	1,160208382	0,861914131	4,066666667
PDIA5	1,61236E-06	0,214209599	0,134	0,014	0,034022396	1,160068181	0,862018299	9,571428571
NEK3	4,35326E-08	0,214192469	0,183	0,021	0,000918581	1,160054406	0,862028535	8,714285714
L3HYPDH	5,33878E-06	0,214037733	0,171	0,032	0,112653644	1,159929992	0,862120996	5,34375
CPTP	1,06622E-11	0,214034019	0,22	0,014	2,24984E-07	1,159927005	0,862123216	15,71428571
ABR	8,89414E-13	0,213752949	0,354	0,049	1,87675E-08	1,159701047	0,862291193	7,224489796
VAC14	5,20852E-08	0,21365429	0,171	0,018	0,00109905	1,159621743	0,862350164	9,5
TMEM115	1,05024E-09	0,213476269	0,232	0,028	2,2161E-05	1,159478661	0,862456579	8,285714286
CELSR1	3,08328E-08	0,21315021	0,122	0,004	0,000650604	1,15921664	0,862651523	30,5
TMED4	1,4775E-06	0,213137207	0,573	0,221	0,03117672	1,159206192	0,862659298	2,592760181
MBD3	1,80137E-06	0,212967535	0,329	0,095	0,038010805	1,159069869	0,862760759	3,463157895
DNAJC11	8,63999E-06	0,212662258	0,195	0,042	0,182312455	1,158824633	0,86294334	4,642857143
POGLUT2	3,05139E-07	0,212611111	0,134	0,011	0,006438732	1,158783551	0,862973934	12,18181818
IK	5,59693E-06	0,212489509	0,463	0,175	0,118100874	1,158685884	0,863046675	2,645714286
AZIN2	1,69291E-06	0,212443724	0,22	0,046	0,035722153	1,158649113	0,863074065	4,782608696

Supplementary Table 2

Differentially expressed genes_keloid-XL_ comparing SC-Keloid vs. (SC-Myel, SC-Nonmyel, SC-Nonmyel2)								
**gene**	**p_val**	**avg_log2FC**	**pct.1**	**pct.2**	**p_val_adj**	**Foldchange_UP**	**Foldchange_DOWN**	**Ratio_pct1_pct2**
CHP1	0,001496408	0,212269446	0,378	0,175	1	1,158509156	0,863178331	2,16
CDR2L	2,03597E-09	0,21203193	0,207	0,021	4,2961E-05	1,158318442	0,863320451	9,857142857
MLX	3,23409E-10	0,212028509	0,354	0,067	6,82426E-06	1,158315696	0,863322498	5,28358209
EFR3A	7,70873E-07	0,212026184	0,268	0,063	0,016266189	1,158313829	0,863323889	4,253968254
KIF9	1,38557E-05	0,212017573	0,183	0,039	0,292368974	1,158306915	0,863329043	4,692307692
ENOPH1	5,0539E-07	0,211973477	0,415	0,13	0,01066424	1,158271512	0,86335543	3,192307692
GAS7	0,054637783	0,211903623	0,854	0,635	1	1,158215431	0,863397235	1,34488189
NAGK	9,01088E-06	0,211902488	0,366	0,126	0,190138555	1,158214519	0,863397914	2,904761905
SLC35B1	3,2832E-08	0,211774755	0,341	0,084	0,000692788	1,158111979	0,86347436	4,05952381
MEA1	2,63114E-08	0,211721858	0,354	0,088	0,000555196	1,158069516	0,863506021	4,022727273
ISG15	8,45557E-08	0,211504029	0,61	0,228	0,001784209	1,157894676	0,863636409	2,675438596
L3MBTL2	5,05208E-06	0,211463866	0,183	0,035	0,106604025	1,157862442	0,863660452	5,228571429
MAST4	0,002714415	0,211442896	0,122	0,035	1	1,157845612	0,863673006	3,485714286
PIK3C3	1,48049E-07	0,211319569	0,244	0,046	0,003123987	1,157746639	0,863746839	5,304347826
RPS3	0,010742368	0,211303987	0,976	0,825	1	1,157734135	0,863756168	1,183030303
NSRP1	9,54854E-07	0,211183324	0,354	0,105	0,020148373	1,157637309	0,863828413	3,371428571
SLC16A3	5,5905E-11	0,211024983	0,146	0	1,17965E-06	1,157510261	0,863923227	#DIV/0!
DPH3	1,60049E-07	0,210970287	0,329	0,084	0,003377185	1,157466378	0,863955981	3,916666667
BET1L	4,1982E-09	0,210947329	0,244	0,035	8,85863E-05	1,157447959	0,863969729	6,971428571
ODF2L	2,46803E-08	0,210796504	0,341	0,081	0,00052078	1,157326961	0,864060057	4,209876543
TMEM126A	1,22402E-06	0,210669007	0,305	0,084	0,025827941	1,157224688	0,86413642	3,630952381
HOXD-AS2	2,96175E-10	0,210583202	0,232	0,025	6,24958E-06	1,157155864	0,864187817	9,28
GID8	2,21466E-06	0,210498838	0,354	0,109	0,046731624	1,157088199	0,864238354	3,247706422
OCIAD1	0,000128162	0,210437444	0,598	0,288	1	1,15703896	0,864275132	2,076388889
FARSA	7,96284E-07	0,210402124	0,207	0,039	0,01680238	1,157010634	0,864296291	5,307692308
MRPL40	1,1465E-10	0,210258746	0,439	0,098	2,41922E-06	1,156895653	0,864382191	4,479591837
VPS9D1	5,13587E-08	0,210186889	0,183	0,021	0,00108372	1,156838033	0,864425245	8,714285714
FUCA2	1,28965E-06	0,21018004	0,293	0,077	0,027212834	1,156832541	0,864429349	3,805194805
CCDC34	5,00259E-05	0,210059658	0,171	0,039	1	1,156736016	0,864501482	4,384615385
ITGA7	0,00216227	0,210009061	0,329	0,144	1	1,156695449	0,864531801	2,284722222

Supplementary Table 2

Differentially expressed genes_keloid-XL_ comparing SC-Keloid vs. (SC-Myel, SC-Nonmyel, SC-Nonmyel2)								
**gene**	**p_val**	**avg_log2FC**	**pct.1**	**pct.2**	**p_val_adj**	**Foldchange_UP**	**Foldchange_DOWN**	**Ratio_pct1_pct2**
GGA2	4,61553E-06	0,209990146	0,183	0,035	0,097392261	1,156680283	0,864543136	5,228571429
GNPAT	6,04746E-06	0,209961284	0,232	0,056	0,127607439	1,156657144	0,864560432	4,142857143
NDUFA9	1,58854E-07	0,20995171	0,28	0,063	0,003351972	1,156649467	0,86456617	4,444444444
TMEM60	2,23108E-08	0,209823209	0,207	0,028	0,000470781	1,156546449	0,86464318	7,392857143
PPP1R9B	6,0984E-06	0,209666142	0,268	0,074	0,128682266	1,156420543	0,864737319	3,621621622
BBS12	5,47929E-05	0,20966609	0,085	0,007	1	1,156420501	0,864737351	12,14285714
C11orf68	2,4143E-06	0,209535249	0,317	0,091	0,050944168	1,156315628	0,864815779	3,483516484
ZER1	9,81975E-07	0,209507925	0,207	0,039	0,020720645	1,156293727	0,864832158	5,307692308
PLCB3	1,13299E-05	0,2094815	0,159	0,028	0,239071246	1,156272548	0,864847999	5,678571429
MTFR1L	3,51938E-05	0,209434417	0,256	0,077	0,742624535	1,156234813	0,864876224	3,324675325
MRPL38	5,3308E-08	0,209379055	0,341	0,084	0,001124852	1,156190445	0,864909414	4,05952381
NFKBIB	7,24379E-07	0,209169093	0,244	0,053	0,015285114	1,156022192	0,865035297	4,603773585
GOLM2	4,64152E-05	0,209147101	0,415	0,165	0,979406552	1,15600457	0,865048484	2,515151515
MKRN2	5,23929E-05	0,209022013	0,171	0,039	1	1,155904343	0,86512349	4,384615385
GMFG	5,21474E-05	0,208763942	0,073	0,004	1	1,155697592	0,865278258	18,25
AKT2	2,83974E-07	0,208745935	0,354	0,098	0,005992126	1,155683168	0,865289058	3,612244898
MAP3K10	0,000180605	0,208552726	0,159	0,039	1	1,155528406	0,865404948	4,076923077
CTBS	7,81632E-09	0,208458587	0,293	0,056	0,000164932	1,155453008	0,865461419	5,232142857
MACO1	4,27945E-05	0,208250741	0,305	0,105	0,903006819	1,155286556	0,865586113	2,904761905
TNFRSF19	1,39205E-05	0,208206962	0,524	0,211	0,293735503	1,1552515	0,86561238	2,483412322
SLC39A13	1,2755E-06	0,20801473	0,329	0,095	0,026914284	1,155097578	0,865727726	3,463157895
PEX26	2,49998E-07	0,207849529	0,22	0,039	0,005275213	1,154965317	0,865826865	5,641025641
FYCO1	0,000648131	0,207846349	0,122	0,028	1	1,154962771	0,865828774	4,357142857
HPS1	0,001467056	0,207755476	0,171	0,056	1	1,154890024	0,865883313	3,053571429
FOCAD	0,000204598	0,207740233	0,098	0,014	1	1,154877822	0,865892461	7
MTX1	3,09191E-07	0,207700032	0,268	0,06	0,00652424	1,154845642	0,86591659	4,466666667
HOXD8	1,23009E-06	0,207646821	0,146	0,018	0,02595621	1,154803048	0,865948528	8,111111111
EIF2D	9,82182E-06	0,207621508	0,268	0,077	0,207250257	1,154782787	0,865963722	3,480519481
LINC00926	1,58349E-08	0,207544329	0,11	0	0,000334132	1,154721012	0,866010049	#DIV/0!
KMT5A	1,08254E-07	0,207254179	0,415	0,123	0,00228426	1,154488801	0,866184236	3,37398374

Supplementary Table 2

Differentially expressed genes_keloid-XL_ comparing SC-Keloid vs. (SC-Myel, SC-Nonmyel, SC-Nonmyel2)								
**gene**	**p_val**	**avg_log2FC**	**pct.1**	**pct.2**	**p_val_adj**	**Foldchange_UP**	**Foldchange_DOWN**	**Ratio_pct1_pct2**
RPA1	1,10663E-08	0,207219518	0,28	0,053	0,00023351	1,154461065	0,866205046	5,283018868
RAB4B	5,53955E-05	0,207112363	0,256	0,081	1	1,154375322	0,866269385	3,160493827
NFE2L1	3,39312E-05	0,207112069	0,354	0,133	0,715983286	1,154375086	0,866269562	2,661654135
BBS7	0,000507286	0,207068194	0,146	0,039	1	1,15433998	0,866295907	3,743589744
CLPTM1L	8,55595E-07	0,207029456	0,305	0,081	0,018053905	1,154308985	0,866319168	3,765432099
MRPS18C	1,20723E-05	0,206918991	0,305	0,095	0,254738633	1,154220605	0,866385503	3,210526316
KLHL29	1,95495E-05	0,206649292	0,293	0,091	0,412514008	1,154004854	0,866547482	3,21978022
AARSD1	1,3747E-06	0,206626503	0,22	0,046	0,029007469	1,153986625	0,86656117	4,782608696
RGS20	3,54737E-07	0,206491398	0,134	0,011	0,007485302	1,153878562	0,866642325	12,18181818
HSD11B1L	5,23216E-05	0,206369551	0,098	0,011	1	1,153781112	0,866715523	8,909090909
PCCB	1,27699E-09	0,206228013	0,232	0,028	2,69458E-05	1,153667924	0,866800558	8,285714286
GSTZ1	2,77227E-09	0,206156077	0,256	0,039	5,84976E-05	1,153610401	0,866843779	6,564102564
FUBP1	7,37791E-05	0,205987057	0,366	0,14	1	1,153475257	0,866945341	2,614285714
SPRY4	0,006606071	0,205562928	0,098	0,028	1	1,153136204	0,867200246	3,5
DPP7	3,58629E-05	0,205545835	0,439	0,179	0,756742776	1,153122541	0,867210521	2,452513966
ST6GAL2	3,69345E-10	0,205528056	0,134	0	7,79355E-06	1,153108331	0,867221208	#DIV/0!
MRPS36	3,73965E-06	0,205459477	0,463	0,172	0,078910337	1,153053519	0,867262433	2,691860465
TINF2	1,042E-07	0,20544714	0,244	0,046	0,002198723	1,153043658	0,86726985	5,304347826
SLC49A3	0,000189633	0,205439864	0,098	0,014	1	1,153037844	0,867274223	7
ATP13A2	6,01581E-08	0,205404995	0,146	0,011	0,001269396	1,153009975	0,867295185	13,27272727
KLHL4	7,70344E-10	0,205305539	0,146	0,004	1,6255E-05	1,152930493	0,867354976	36,5
EIF2AK4	3,22E-06	0,205301568	0,305	0,088	0,067945183	1,152927319	0,867357364	3,465909091
ZNF706	0,003218054	0,205001881	0,793	0,446	1	1,15268785	0,867537556	1,778026906
VPS26A	2,68547E-05	0,20497741	0,463	0,186	0,566661979	1,152668298	0,867552272	2,489247312
CEP162	0,000214102	0,204936025	0,098	0,014	1	1,152635233	0,867577158	7
ZNF362	3,67813E-08	0,204889286	0,305	0,067	0,000776123	1,152597892	0,867605266	4,552238806
ELP5	7,0129E-09	0,204834837	0,378	0,091	0,000147979	1,152554393	0,86763801	4,153846154
VIM	0,018650911	0,204783045	1	0,993	1	1,152513017	0,867669159	1,007049345
TTLL1	0,000105335	0,204779658	0,122	0,021	1	1,152510311	0,867671196	5,80952381
SERBP1	0,002056492	0,204705615	0,744	0,432	1	1,152451162	0,867715729	1,722222222

Supplementary Table 2

Differentially expressed genes_keloid-XL_ comparing SC-Keloid vs. (SC-Myel, SC-Nonmyel, SC-Nonmyel2)								
gene	p_val	avg_log2FC	pct.1	pct.2	p_val_adj	Foldchange_UP	Foldchange_DOWN	Ratio_pct1_pct2
TTC1	9,93213E-08	0,204579496	0,39	0,112	0,002095778	1,152350421	0,867791587	3,482142857
IFNAR1	1,97851E-05	0,204569914	0,463	0,182	0,417484455	1,152342768	0,86779735	2,543956044
CDC42BPB	1,68283E-09	0,20454549	0,415	0,102	3,55095E-05	1,152323259	0,867812042	4,068627451
LAMP1	0,007559161	0,204361229	0,756	0,474	1	1,152176094	0,867922886	1,594936709
TAF9	1,04123E-06	0,204031021	0,305	0,084	0,021971013	1,151912411	0,868121561	3,630952381
RASGEF1C	1,21191E-05	0,203650975	0,39	0,14	0,255724915	1,151609005	0,868350279	2,785714286
ZMPSTE24	3,01009E-06	0,203617783	0,305	0,088	0,063515875	1,15158251	0,868370257	3,465909091
ANKRD52	1,5202E-06	0,203601659	0,146	0,018	0,0320778	1,15156964	0,868379962	8,111111111
SAR1B	1,11852E-06	0,203469931	0,341	0,102	0,023601851	1,151464498	0,868459255	3,343137255
WBP1	0,000493127	0,203402358	0,305	0,123	1	1,151410568	0,868499932	2,479674797
POLD4	3,32561E-05	0,20324575	0,317	0,109	0,701736723	1,151285586	0,868594215	2,908256881
COX20	6,34055E-07	0,203002567	0,61	0,239	0,013379203	1,15109154	0,868740639	2,552301255
ZNF720	0,000181872	0,202928082	0,195	0,056	1	1,151032111	0,868785493	3,482142857
PRR13	9,11153E-08	0,202735002	0,293	0,063	0,001922624	1,150878075	0,868901773	4,650793651
PDZRN4	0,000183568	0,202726164	0,049	0	1	1,150871026	0,868907095	#DIV/0!
UQCR10	1,01644E-05	0,202622111	0,598	0,27	0,21447991	1,150788023	0,868969767	2,214814815
SLC35E1	0,000261614	0,202449608	0,122	0,025	1	1,150650431	0,869073676	4,88
ACTL6A	1,89091E-08	0,202429394	0,305	0,063	0,000399001	1,15063431	0,869085852	4,841269841
OXLD1	3,84049E-10	0,202340576	0,207	0,018	8,10383E-06	1,150563474	0,869139359	11,5
IDH3B	2,95473E-10	0,202316098	0,415	0,095	6,23478E-06	1,150543953	0,869154105	4,368421053
PAM	1,02845E-07	0,202131818	0,244	0,046	0,002170139	1,150397	0,869265132	5,304347826
RPL23A	0,035594651	0,201733128	0,963	0,898	1	1,15007913	0,869505388	1,072383073
UNC119	1,02173E-05	0,201646471	0,098	0,007	0,215595665	1,150010051	0,869557617	14
EIF2S1	7,08191E-06	0,201458822	0,305	0,095	0,149435441	1,149860481	0,869670726	3,210526316
LIME1	0,001278637	0,201411732	0,073	0,011	1	1,14982295	0,869699113	6,636363636
ILVBL	5,31902E-06	0,201357922	0,183	0,035	0,112236587	1,149780065	0,869731552	5,228571429
BRCC3	1,08035E-06	0,201306629	0,159	0,021	0,022796397	1,149739186	0,869762475	7,571428571
NCAPH2	0,000187187	0,201275876	0,195	0,056	1	1,149714679	0,869781015	3,482142857
LTBP3	0,040689728	0,201091914	0,598	0,393	1	1,149568085	0,86989193	1,521628499
TOM1	8,40872E-07	0,200680392	0,159	0,021	0,017743232	1,149240222	0,870140098	7,571428571

Supplementary Table 2

Differentially expressed genes_keloid-XL_ comparing SC-Keloid vs. (SC-Myel, SC-Nonmyel, SC-Nonmyel2)								
gene	p_val	avg_log2FC	pct.1	pct.2	p_val_adj	Foldchange_UP	Foldchange_DOWN	Ratio_pct1_pct2
UNC93B1	6,44539E-06	0,199903871	0,183	0,035	0,136004184	1,148621818	0,870608571	5,228571429
EFS	6,63905E-06	0,19966269	0,463	0,172	0,140090583	1,148429815	0,870754126	2,691860465
MYEF2	2,61613E-05	0,199585491	0,244	0,07	0,55202967	1,148368363	0,870800722	3,485714286
ZNF260	0,000420675	0,19953811	0,159	0,042	1	1,148330649	0,870829321	3,785714286
MRPL11	2,13808E-10	0,199484106	0,317	0,053	4,51156E-06	1,148287665	0,87086192	5,981132075
AQP7	1,047E-05	0,199378478	0,11	0,011	0,220927383	1,148203595	0,870925683	10
POLR3B	1,02952E-07	0,199264872	0,098	0	0,002172399	1,148113183	0,870994267	#DIV/0!
TFDP1	4,13102E-08	0,199150769	0,341	0,081	0,000871686	1,148022382	0,871063157	4,209876543
PHAX	4,53829E-09	0,199138	0,354	0,077	9,57625E-05	1,148012221	0,871070866	4,597402597
ADIPOR1	1,32567E-06	0,199111745	0,329	0,095	0,027973041	1,147991329	0,871086719	3,463157895
ATIC	1,80693E-07	0,199107603	0,183	0,025	0,003812806	1,147988033	0,87108922	7,32
TSEN34	3,33003E-08	0,199047437	0,317	0,07	0,00070267	1,147940159	0,871125548	4,528571429
ESCO1	9,47787E-07	0,198902958	0,171	0,025	0,019999248	1,147825203	0,871212792	6,84
STING1	0,000119672	0,19874891	0,22	0,067	1	1,147702648	0,871305823	3,28358209
RHBDF2	2,9671E-05	0,198621957	0,159	0,032	0,626087906	1,147601658	0,871382499	4,96875
FLYWCH2	2,38462E-09	0,198473444	0,39	0,091	5,03179E-05	1,147483528	0,871472205	4,285714286
TXNDC9	5,32006E-10	0,198471996	0,293	0,046	1,12259E-05	1,147482376	0,87147308	6,369565217
LAMP2	0,016993087	0,198455043	0,817	0,54	1	1,147468892	0,87148332	1,512962963
CHURC1	1,15906E-05	0,198434634	0,659	0,302	0,244573686	1,14745266	0,871495649	2,182119205
MVB12A	4,85608E-09	0,198402902	0,402	0,098	0,000102468	1,147427422	0,871514817	4,102040816
COA3	2,34093E-11	0,19839499	0,305	0,042	4,9396E-07	1,147421129	0,871519597	7,261904762
LINC00920	5,29929E-08	0,198377034	0,171	0,018	0,001118203	1,147406849	0,871530444	9,5
DPM3	3,39548E-07	0,198295302	0,439	0,14	0,007164809	1,147341847	0,87157982	3,135714286
STK40	5,46195E-06	0,198094467	0,268	0,074	0,115252604	1,147182139	0,871701159	3,621621622
GRB10	1,41456E-06	0,197790602	0,402	0,133	0,029848528	1,146940541	0,871884779	3,022556391
CDK16	5,01014E-08	0,19767576	0,378	0,102	0,00105719	1,146849246	0,871954186	3,705882353
SRR	1,44656E-09	0,197575044	0,159	0,007	3,05239E-05	1,146769186	0,87201506	22,71428571
CRK	4,05398E-07	0,197286843	0,488	0,168	0,008554308	1,146540123	0,872189276	2,904761905
FAM219A	0,000230198	0,1972785	0,183	0,053	1	1,146533493	0,87219432	3,452830189
CD58	5,22271E-10	0,197219699	0,39	0,084	1,10204E-05	1,146486764	0,872229869	4,642857143

Supplementary Table 2

Differentially expressed genes_keloid-XL_ comparing SC-Keloid vs. (SC-Myel, SC-Nonmyel, SC-Nonmyel2)								
**gene**	**p_val**	**avg_log2FC**	**pct.1**	**pct.2**	**p_val_adj**	**Foldchange_UP**	**Foldchange_DOWN**	**Ratio_pct1_pct2**
DSC3	0,059627559	0,197189476	0,061	0,021	1	1,146462746	0,872248142	2,904761905
MACROH2A1	3,05228E-08	0,197149943	0,537	0,175	0,000644061	1,146431331	0,872272044	3,068571429
ZNHIT2	5,79627E-05	0,197099523	0,085	0,007	1	1,146391266	0,872302529	12,14285714
FKBP15	3,95199E-06	0,196963339	0,159	0,025	0,083391026	1,146283057	0,872384874	6,36
LRRC45	1,80693E-07	0,196956531	0,183	0,025	0,003812806	1,146277648	0,872388991	7,32
CDC37	0,000202826	0,196865502	0,659	0,337	1	1,146205323	0,872444038	1,955489614
CDIN1	8,1138E-06	0,196850241	0,085	0,004	0,171209209	1,146193199	0,872453266	21,25
PCNX2	3,95284E-10	0,196795348	0,22	0,021	8,34089E-06	1,146149588	0,872486463	10,47619048
CNOT7	0,006569046	0,196791855	0,183	0,074	1	1,146146814	0,872488575	2,472972973
HOXB8	1,35664E-09	0,196757961	0,159	0,007	2,86265E-05	1,146119887	0,872509073	22,71428571
B4GALT6	8,93582E-09	0,19672582	0,268	0,046	0,000188555	1,146094353	0,872528511	5,826086957
OSR1	5,30286E-08	0,196637898	0,134	0,007	0,001118956	1,146024509	0,872581687	19,14285714
RAD50	7,20148E-09	0,196594433	0,329	0,07	0,000151958	1,145989983	0,872607977	4,7
NUBP2	5,07562E-06	0,196507479	0,39	0,133	0,107100642	1,145920913	0,872660572	2,932330827
PSMD7	4,44657E-05	0,196486006	0,427	0,168	0,938269864	1,145903858	0,872673561	2,541666667
IFI6	1,28153E-07	0,196407951	0,573	0,225	0,002704153	1,145841862	0,872720777	2,546666667
SGSH	5,69725E-05	0,196354139	0,098	0,011	1	1,145799123	0,87275333	8,909090909
ANKRD6	1,95401E-08	0,196226181	0,232	0,035	0,000412315	1,145697503	0,872830741	6,628571429
IMMT	2,60919E-09	0,19617645	0,341	0,07	5,50565E-05	1,14565801	0,872860829	4,871428571
DPM1	3,52125E-07	0,196172536	0,293	0,07	0,007430198	1,145654902	0,872863197	4,185714286
DDX10	1,89218E-06	0,195988209	0,183	0,032	0,039926833	1,145508536	0,872974726	5,71875
CISD1	3,66571E-08	0,195809004	0,268	0,053	0,000773502	1,145366255	0,87308317	5,056603774
TMEM201	0,013668311	0,195798212	0,134	0,053	1	1,145357687	0,873089701	2,528301887
FBLIM1	4,16452E-05	0,195784841	0,11	0,014	0,878756316	1,145347071	0,873097793	7,857142857
DNAJC10	2,01923E-06	0,195642254	0,341	0,102	0,042607711	1,145233878	0,873184088	3,343137255
TMEM63B	4,66646E-05	0,19557091	0,171	0,039	0,984669334	1,145177246	0,87322727	4,384615385
DYRK1B	3,04946E-07	0,195441499	0,122	0,007	0,006434672	1,145074527	0,873305603	17,42857143
OSBPL8	0,000457116	0,195358693	0,402	0,182	1	1,145008806	0,873355729	2,208791209
SLC1A5	4,23165E-06	0,195209858	0,293	0,084	0,089292063	1,144890687	0,873445833	3,488095238
ARHGAP22	8,29253E-06	0,195197764	0,085	0,004	0,174980583	1,14488109	0,873453155	21,25

Supplementary Table 2

Differentially expressed genes_keloid-XL_ comparing SC-Keloid vs. (SC-Myel, SC-Nonmyel, SC-Nonmyel2)								
**gene**	**p_val**	**avg_log2FC**	**pct.1**	**pct.2**	**p_val_adj**	**Foldchange_UP**	**Foldchange_DOWN**	**Ratio_pct1_pct2**
EFCAB7	3,32276E-05	0,195170178	0,159	0,032	0,701135887	1,144859199	0,873469856	4,96875
SETDB1	0,000285723	0,195161436	0,122	0,025	1	1,144852261	0,87347515	4,88
DHRS1	1,17194E-07	0,195157725	0,244	0,046	0,002472921	1,144849316	0,873477396	5,304347826
RALYL	2,26856E-10	0,195061086	0,171	0,007	4,78689E-06	1,144772631	0,873535908	24,42857143
BBS4	4,88779E-08	0,195038844	0,183	0,021	0,001031372	1,144754982	0,873549376	8,714285714
RABIF	1,96537E-06	0,194974874	0,232	0,053	0,041471269	1,144704224	0,87358811	4,377358491
ERLEC1	3,0295E-07	0,194940491	0,354	0,098	0,006392545	1,144676944	0,87360893	3,612244898
ENTPD6	6,36417E-07	0,194830016	0,183	0,028	0,013429045	1,144589293	0,87367583	6,535714286
UBE2I	3,08436E-05	0,194662668	0,646	0,295	0,650831393	1,144456531	0,873777179	2,189830508
NKX6-1	1,89824E-06	0,194553316	0,122	0,011	0,040054753	1,144369788	0,873843412	11,09090909
NAE1	1,42969E-09	0,194348384	0,317	0,06	3,01678E-05	1,144207245	0,873967548	5,283333333
CPSF1	0,013396864	0,194283139	0,122	0,046	1	1,1441555	0,874007074	2,652173913
PRDX2	5,0436E-05	0,194178512	0,451	0,179	1	1,144072526	0,87407046	2,519553073
TMEM259	0,00018375	0,194141208	0,659	0,323	1	1,144042944	0,874093062	2,040247678
SH3BP1	3,35288E-07	0,194099877	0,134	0,011	0,00707491	1,14401017	0,874118103	12,18181818
PIEZO1	9,09958E-08	0,194048526	0,439	0,13	0,001920103	1,143969451	0,874149217	3,376923077
TENT2	9,66716E-06	0,193880779	0,244	0,063	0,203986677	1,143836445	0,874250863	3,873015873
MRPS23	7,53784E-07	0,19384105	0,268	0,063	0,0159056	1,143804946	0,874274939	4,253968254
DAG1	0,001758994	0,193756124	0,817	0,463	1	1,143737617	0,874326406	1,764578834
UROD	7,64015E-08	0,193578059	0,256	0,049	0,001612148	1,14359646	0,874434327	5,224489796
TBX3	0,011324043	0,193321204	0,085	0,025	1	1,143392874	0,874590023	3,4
NXPH3	2,42495E-09	0,193120886	0,122	0	5,11688E-05	1,143234125	0,874711468	#DIV/0!
PEX11B	7,27037E-06	0,193116523	0,134	0,018	0,153412017	1,143230667	0,874714114	7,444444444
FNTB	1,20154E-05	0,193108641	0,232	0,06	0,253537089	1,143224422	0,874718892	3,866666667
ACAD9	3,62311E-06	0,19276626	0,159	0,025	0,076451158	1,142953144	0,874926506	6,36
GGA1	3,09185E-05	0,1927365	0,22	0,06	0,652412253	1,142929567	0,874944554	3,666666667
NACC1	1,57118E-07	0,192675251	0,183	0,025	0,003315355	1,142881045	0,8749817	7,32
IL27RA	3,35857E-05	0,192516454	0,122	0,018	0,708691935	1,142755256	0,875078014	6,777777778
SUMF1	6,59742E-06	0,192474647	0,207	0,046	0,13921213	1,14272214	0,875103373	4,5
ZCCHC9	5,10936E-08	0,192471029	0,268	0,053	0,001078126	1,142719275	0,875105568	5,056603774

Supplementary Table 2

Differentially expressed genes_keloid-XL_ comparing SC-Keloid vs. (SC-Myel, SC-Nonmyel, SC-Nonmyel2)								
**gene**	**p_val**	**avg_log2FC**	**pct.1**	**pct.2**	**p_val_adj**	**Foldchange_UP**	**Foldchange_DOWN**	**Ratio_pct1_pct2**
RHOBTB2	1,06202E-05	0,192310162	0,098	0,007	0,224096034	1,142591864	0,875203151	14
ADK	0,000662137	0,192263229	0,488	0,228	1	1,142554694	0,875231624	2,140350877
SLC25A3	0,007505446	0,192203049	0,793	0,572	1	1,142507035	0,875268133	1,386363636
OSBPL10	5,23929E-05	0,19200114	0,171	0,039	1	1,142347149	0,875390638	4,384615385
ACLY	2,08426E-07	0,191878864	0,366	0,102	0,004397995	1,142250334	0,875464835	3,588235294
RDX	0,01453352	0,191744442	0,707	0,477	1	1,142143911	0,875546409	1,482180294
ETFRF1	0,039502926	0,191438042	0,305	0,168	1	1,141901368	0,875732378	1,81547619
MRPL33	0,000547206	0,191371461	0,646	0,361	1	1,141848669	0,875772795	1,789473684
TBRG1	0,001040606	0,19122383	0,305	0,13	1	1,14173183	0,875862417	2,346153846
TSPAN31	2,93846E-08	0,191137496	0,28	0,056	0,000620044	1,141663508	0,875914832	5
ACSL4	3,77007E-07	0,19108781	0,268	0,06	0,007955225	1,14162419	0,875944999	4,466666667
EXOSC1	8,51851E-10	0,19100957	0,244	0,032	1,79749E-05	1,14156228	0,875992504	7,625
TCOF1	2,70805E-06	0,190979186	0,207	0,042	0,057142629	1,141538238	0,876010954	4,928571429
MFSD5	8,93956E-06	0,190833714	0,171	0,032	0,188633563	1,141423138	0,876099289	5,34375
SDHB	4,63908E-06	0,190771892	0,439	0,161	0,097889147	1,141374227	0,876136832	2,726708075
HEBP2	2,32358E-06	0,190487553	0,317	0,091	0,04902987	1,141149298	0,876309526	3,483516484
AK2	3,59359E-09	0,190462039	0,463	0,126	7,58283E-05	1,141129117	0,876325023	3,674603175
KRCC1	6,61008E-08	0,190457408	0,439	0,13	0,001394793	1,141125454	0,876327836	3,376923077
PSMD6	5,16215E-06	0,190398392	0,415	0,144	0,108926617	1,141078774	0,876363685	2,881944444
USP5	6,27593E-07	0,190380273	0,244	0,053	0,01324283	1,141064444	0,876374691	4,603773585
LIMD1	2,49785E-05	0,190343342	0,134	0,021	0,527071154	1,141035235	0,876397126	6,380952381
AC147651.4	4,20685E-09	0,190291097	0,22	0,028	8,87686E-05	1,140993915	0,876428864	7,857142857
APTX	2,27685E-06	0,19006452	0,207	0,042	0,048043722	1,140814734	0,876566519	4,928571429
DUS1L	6,95698E-07	0,18999998	0,305	0,081	0,014679919	1,1407637	0,876605734	3,765432099
PSMA5	3,60687E-06	0,189961611	0,439	0,165	0,076108533	1,140733361	0,876629048	2,660606061
GBP3	1,33284E-06	0,189856333	0,195	0,035	0,028124213	1,140650121	0,87669302	5,571428571
MORC2	0,001602565	0,189851902	0,098	0,021	1	1,140646619	0,876695713	4,666666667
SLC50A1	0,00123424	0,189803635	0,171	0,056	1	1,140608457	0,876725044	3,053571429
MGMT	1,15167E-06	0,189721647	0,354	0,105	0,024301363	1,140543638	0,87677487	3,371428571
ZDHHC4	1,41434E-07	0,189689871	0,232	0,042	0,002984409	1,140518518	0,876794181	5,523809524

Supplementary Table 2

Differentially expressed genes_keloid-XL_ comparing SC-Keloid vs. (SC-Myel, SC-Nonmyel, SC-Nonmyel2)								
**gene**	**p_val**	**avg_log2FC**	**pct.1**	**pct.2**	**p_val_adj**	**Foldchange_UP**	**Foldchange_DOWN**	**Ratio_pct1_pct2**
CRABP1	1,39592E-06	0,189655271	0,098	0,004	0,029455268	1,140491165	0,87681521	24,5
SUMO3	1,01769E-08	0,189630207	0,5	0,147	0,000214742	1,140471352	0,876830442	3,401360544
DDX23	8,0798E-05	0,189626189	0,195	0,053	1	1,140468175	0,876832885	3,679245283
RUSC1	0,001185897	0,189620609	0,183	0,063	1	1,140463764	0,876836276	2,904761905
DHCR7	1,02952E-07	0,189599648	0,098	0	0,002172399	1,140447195	0,876849016	#DIV/0!
SEMA4F	0,000317802	0,189498134	0,061	0,004	1	1,140366951	0,876910717	15,25
RASA1	8,04146E-06	0,189480677	0,195	0,042	0,169682876	1,140353152	0,876921327	4,642857143
IGFBP2	0,074868323	0,18936746	0,22	0,133	1	1,140263665	0,876990148	1,654135338
RWDD4	9,14205E-08	0,189208925	0,305	0,07	0,001929064	1,140138371	0,877086523	4,357142857
PIF1	6,01716E-05	0,189110718	0,085	0,007	1	1,140060762	0,87714623	12,14285714
MZT1	1,83774E-05	0,18901611	0,329	0,112	0,387782228	1,139986002	0,877203753	2,9375
MRPS15	6,41683E-10	0,188954899	0,341	0,067	1,35402E-05	1,139937636	0,877240973	5,089552239
NIPSNAP3A	1,09747E-09	0,188770436	0,244	0,032	2,31577E-05	1,139791892	0,877353144	7,625
RTKN2	1,01057E-05	0,188155147	0,11	0,011	0,21323951	1,139305891	0,877727402	10
PCLAF	0,303137231	0,188117633	0,037	0,018	1	1,139276267	0,877750226	2,055555556
EPRS1	4,49256E-07	0,188024494	0,341	0,095	0,00947976	1,139202718	0,877806894	3,589473684
NF1	6,65788E-06	0,187770265	0,439	0,161	0,140488022	1,139001988	0,877961593	2,726708075
DCBLD1	2,1468E-05	0,187716025	0,134	0,021	0,452995664	1,138959167	0,877994602	6,380952381
ADPGK	0,000115143	0,187676898	0,244	0,077	1	1,138928278	0,878018414	3,168831169
SAMM50	5,65719E-06	0,187633586	0,268	0,074	0,119372465	1,138894086	0,878044774	3,621621622
MZT2A	2,18447E-06	0,187540198	0,402	0,133	0,046094477	1,138820365	0,878101613	3,022556391
PTDSS1	9,5155E-05	0,187536258	0,171	0,042	1	1,138817255	0,878104011	4,071428571
RABL6	4,41673E-07	0,187154739	0,39	0,119	0,00931974	1,138516136	0,878336256	3,277310924
ARHGEF11	2,09545E-06	0,186941603	0,341	0,102	0,044215994	1,13834795	0,878466026	3,343137255
CCDC74A	8,29253E-06	0,186936592	0,085	0,004	0,174980583	1,138343997	0,878469077	21,25
RPL26L1	3,67678E-07	0,186741496	0,39	0,116	0,007758375	1,138190069	0,87858788	3,362068966
GGH	3,3376E-08	0,186741142	0,207	0,028	0,000704268	1,138189789	0,878588096	7,392857143
GSTK1	7,21159E-05	0,186707597	0,561	0,253	1	1,138163325	0,878608525	2,217391304
NEDD4	1,93327E-06	0,18666972	0,122	0,011	0,040793846	1,138133444	0,878631593	11,09090909
CPSF6	4,33869E-07	0,186602057	0,341	0,095	0,009155073	1,138080066	0,878672802	3,589473684

Supplementary Table 2

Differentially expressed genes_keloid-XL_ comparing SC-Keloid vs. (SC-Myel, SC-Nonmyel, SC-Nonmyel2)								
gene	p_val	avg_log2FC	pct.1	pct.2	p_val_adj	Foldchange_UP	Foldchange_DOWN	Ratio_pct1_pct2
TTF1	6,20327E-06	0,186371112	0,183	0,035	0,13089519	1,137897897	0,87881347	5,228571429
C12orf73	5,13587E-08	0,186370726	0,183	0,021	0,00108372	1,137897593	0,878813705	8,714285714
TRIT1	0,000198484	0,186370446	0,098	0,014	1	1,137897373	0,878813876	7
LRWD1	5,97624E-09	0,186365736	0,22	0,028	0,000126105	1,137893658	0,878816745	7,857142857
UGGT1	1,44381E-06	0,18634078	0,195	0,035	0,030465921	1,137873974	0,878831947	5,571428571
TPRN	0,001602574	0,186325047	0,098	0,021	1	1,137861566	0,878841531	4,666666667
GTF2H5	9,9754E-09	0,18623289	0,573	0,189	0,000210491	1,137788883	0,878897672	3,031746032
SATB1	7,86018E-05	0,18615457	0,305	0,109	1	1,137727117	0,878945386	2,798165138
PDHX	3,24738E-10	0,186150723	0,195	0,014	6,85231E-06	1,137724084	0,878947729	13,92857143
UBE2M	0,000146365	0,185930978	0,439	0,189	1	1,137550803	0,879081618	2,322751323
DPP3	1,66973E-09	0,185853565	0,183	0,014	3,52329E-05	1,137489766	0,879128789	13,07142857
HMGN2	0,001113718	0,185778081	0,671	0,372	1	1,137430252	0,879174787	1,803763441
MAPRE3	1,56288E-06	0,185581271	0,244	0,056	0,032978351	1,137275096	0,879294731	4,357142857
KLHL2	0,001526092	0,185528473	0,098	0,021	1	1,137233476	0,879326911	4,666666667
DYNLRB1	0,007253273	0,185521472	0,805	0,509	1	1,137227958	0,879331178	1,581532417
CRYZ	2,17226E-10	0,185514639	0,171	0,007	4,58369E-06	1,137222571	0,879335343	24,42857143
PTRH1	6,8349E-05	0,185451697	0,134	0,025	1	1,137172958	0,879373707	5,36
TSPAN17	4,58906E-08	0,185440732	0,146	0,011	0,000968339	1,137164315	0,879380391	13,27272727
CDK18	1,04809E-07	0,185438673	0,28	0,06	0,002211585	1,137162692	0,879381646	4,666666667
TSR3	1,96302E-09	0,185360775	0,341	0,07	4,14217E-05	1,137101293	0,87942913	4,871428571
TOP3B	0,000109756	0,185316628	0,122	0,021	1	1,137066498	0,87945604	5,80952381
ERO1B	0,00017668	0,185300392	0,159	0,039	1	1,137053701	0,879465938	4,076923077
YEATS4	2,47065E-09	0,185099412	0,305	0,056	5,21333E-05	1,136895311	0,879588464	5,446428571
NCLN	7,79976E-09	0,184938087	0,256	0,042	0,000164583	1,136768188	0,879686827	6,095238095
RPL11	0,013163286	0,184858663	0,976	0,912	1	1,136705607	0,879735257	1,070175439
TMBIM4	0,00015338	0,184350253	0,683	0,34	1	1,1363051	0,880045333	2,008823529
STXBP6	1,44077E-09	0,184313873	0,293	0,049	3,04016E-05	1,136276446	0,880067525	5,979591837
BAK1	1,90824E-09	0,184279172	0,171	0,011	4,02658E-05	1,136249116	0,880088694	15,54545455
SHISAL1	4,32324E-06	0,184257888	0,073	0	0,09122464	1,136232353	0,880101678	#DIV/0!
EIF3J	1,66729E-05	0,18404421	0,512	0,218	0,35181423	1,136064078	0,880232039	2,348623853

Supplementary Table 2

Differentially expressed genes_keloid-XL_ comparing SC-Keloid vs. (SC-Myel, SC-Nonmyel, SC-Nonmyel2)								
gene	p_val	avg_log2FC	pct.1	pct.2	p_val_adj	Foldchange_UP	Foldchange_DOWN	Ratio_pct1_pct2
ARIH2	6,79308E-05	0,184002828	0,305	0,105	1	1,136031492	0,880257288	2,904761905
DPY19L3	1,17398E-05	0,183884512	0,256	0,07	0,247722026	1,135938329	0,880329481	3,657142857
SYNGR1	2,33208E-06	0,183797206	0,366	0,116	0,049209204	1,135869589	0,880382757	3,155172414
IRF7	2,00956E-05	0,183768096	0,268	0,081	0,424036398	1,13584667	0,880400521	3,308641975
MTREX	0,000883159	0,183755184	0,183	0,06	1	1,135836504	0,880408401	3,05
RALY	1,01974E-08	0,183717515	0,524	0,158	0,000215176	1,135806848	0,880431389	3,316455696
ABCF1	4,50019E-05	0,183548833	0,293	0,098	0,949584281	1,135674055	0,880534336	2,989795918
ALKBH7	0,00011244	0,183311667	0,463	0,2	1	1,135487376	0,8806791	2,315
PAPSS1	0,000730684	0,183302002	0,366	0,161	1	1,135479769	0,880684999	2,273291925
MORF4L2	0,005090405	0,183251116	0,707	0,439	1	1,13543972	0,880716063	1,61047836
ENTR1	0,002273841	0,183093339	0,183	0,067	1	1,135315552	0,880812386	2,731343284
SMPD1	4,23152E-06	0,183027949	0,195	0,039	0,089289314	1,135264095	0,880852309	5
AP3M2	0,037517008	0,183017033	0,11	0,046	1	1,135255505	0,880858974	2,391304348
RTL8A	4,36499E-07	0,182990777	0,451	0,151	0,009210571	1,135234845	0,880875005	2,986754967
PHLDA3	0,000125988	0,182715794	0,415	0,175	1	1,135018485	0,88104292	2,371428571
SMARCD2	8,96112E-07	0,18248936	0,207	0,039	0,018908861	1,134840355	0,881181212	5,307692308
FMNL3	1,94017E-06	0,182484097	0,329	0,098	0,040939605	1,134836216	0,881184426	3,357142857
GNAI3	2,55251E-07	0,182378246	0,512	0,172	0,005386043	1,134752955	0,881249082	2,976744186
COPG1	1,56288E-06	0,182362381	0,244	0,056	0,032978351	1,134740477	0,881258773	4,357142857
PML	6,2277E-05	0,182329505	0,207	0,056	1	1,134714619	0,881278855	3,696428571
SRRD	5,90231E-05	0,182320027	0,134	0,025	1	1,134707164	0,881284645	5,36
AHNAK2	6,37324E-08	0,18230617	0,146	0,011	0,001344817	1,134696266	0,881293109	13,27272727
C9orf64	0,000614877	0,182222966	0,098	0,018	1	1,134630827	0,881343937	5,444444444
PRMT9	0,001428181	0,181790442	0,122	0,032	1	1,134290712	0,881608206	3,8125
RNH1	4,05527E-05	0,181744993	0,695	0,34	0,855702222	1,134254979	0,88163598	2,044117647
MTRNR2L1	0,008167365	0,18163895	0,134	0,049	1	1,134171611	0,881700786	2,734693878
PEF1	2,49456E-07	0,181599131	0,329	0,084	0,005263767	1,134140307	0,881725121	3,916666667
IFT27	4,72583E-07	0,181578578	0,256	0,056	0,009971983	1,134124151	0,881737682	4,571428571
SRP14-DT	0,513508152	0,181537085	0,024	0,014	1	1,134091533	0,881763042	1,714285714
POLD2	1,79149E-06	0,181522729	0,317	0,091	0,037802275	1,134080248	0,881771816	3,483516484

Supplementary Table 2

Differentially expressed genes_keloid-XL_ comparing SC-Keloid vs. (SC-Myel, SC-Nonmyel, SC-Nonmyel2)								
**gene**	**p_val**	**avg_log2FC**	**pct.1**	**pct.2**	**p_val_adj**	**Foldchange_UP**	**Foldchange_DOWN**	**Ratio_pct1_pct2**
GNPDA2	4,00328E-08	0,181512663	0,244	0,042	0,000844731	1,134072335	0,881777969	5,80952381
IRAK1	1,29829E-06	0,18135719	0,256	0,06	0,027395138	1,133950128	0,881872999	4,266666667
NDUFA10	4,07505E-05	0,181321279	0,476	0,193	0,859876183	1,133921902	0,881894951	2,466321244
C8orf33	7,6302E-08	0,181311532	0,293	0,063	0,001610048	1,133914241	0,881900909	4,650793651
SMIM30	2,87705E-06	0,181290853	0,256	0,063	0,060708706	1,133897988	0,88191355	4,063492063
B3GALT4	2,34822E-07	0,181228414	0,171	0,021	0,004954974	1,133848915	0,88195172	8,142857143
EVL	3,73156E-08	0,181205807	0,28	0,056	0,000787397	1,133831148	0,88196554	5
WDR41	3,47772E-07	0,181189499	0,207	0,035	0,007338339	1,133818331	0,881975509	5,914285714
SNRNP200	1,52554E-05	0,181160837	0,366	0,126	0,321903617	1,133795806	0,881993032	2,904761905
CRCP	0,000380805	0,180845057	0,171	0,049	1	1,133547665	0,882186106	3,489795918
PPP1R35	2,77004E-07	0,180772842	0,317	0,081	0,005845069	1,133490926	0,882230265	3,913580247
CACNA1D	0,000210889	0,180662128	0,098	0,014	1	1,133403944	0,882297971	7
LMNA	0,024908882	0,180583124	0,951	0,821	1	1,133341879	0,882346288	1,158343484
DLGAP4	0,000149751	0,180576247	0,683	0,354	1	1,133336476	0,882350494	1,929378531
FUT8	6,87072E-05	0,180408768	0,305	0,109	1	1,133204918	0,88245293	2,798165138
CREB3	4,77161E-08	0,180401589	0,317	0,074	0,001006857	1,133199279	0,882457321	4,283783784
TRIQK	1,55242E-07	0,180195951	0,232	0,042	0,003275771	1,133037767	0,882583113	5,523809524
SDHC	7,38549E-10	0,180178465	0,488	0,126	1,55841E-05	1,133024034	0,882593811	3,873015873
LTF	0,011128779	0,180173378	0,037	0,004	1	1,13302004	0,882596922	9,25
TFPT	2,77308E-08	0,18011305	0,207	0,028	0,000585148	1,132972662	0,88263383	7,392857143
RBM4B	2,01765E-05	0,180055283	0,171	0,035	0,425744527	1,132927297	0,882669172	4,885714286
EIF3H	0,001321454	0,180047991	0,622	0,333	1	1,132921571	0,882673634	1,867867868
NMI	7,28917E-09	0,17997415	0,329	0,07	0,000153809	1,132863587	0,882718812	4,7
CASD1	0,000174788	0,179936803	0,268	0,091	1	1,132834261	0,882741664	2,945054945
DLG4	1,16779E-06	0,17989347	0,159	0,021	0,02464156	1,132800235	0,882768178	7,571428571
SMAP2	2,71249E-10	0,179875262	0,244	0,028	5,72363E-06	1,132785938	0,88277932	8,714285714
FAM229A	1,25037E-06	0,179608747	0,146	0,018	0,026384074	1,132576694	0,882942414	8,111111111
TMED2	4,58849E-06	0,179381673	0,524	0,207	0,096821662	1,132398445	0,883081397	2,531400966
CNIH4	2,93871E-07	0,179343989	0,402	0,123	0,006200974	1,132368866	0,883104463	3,268292683
KRT80	3,41654E-07	0,179114449	0,134	0,011	0,007209233	1,132188715	0,883244981	12,18181818

Supplementary Table 2

Differentially expressed genes_keloid-XL_ comparing SC-Keloid vs. (SC-Myel, SC-Nonmyel, SC-Nonmyel2)								
gene	p_val	avg_log2FC	pct.1	pct.2	p_val_adj	Foldchange_UP	Foldchange_DOWN	Ratio_pct1_pct2
TRAPPC3	1,84426E-06	0,179112417	0,317	0,091	0,038915657	1,13218712	0,883246225	3,483516484
RHBDF1	0,000386187	0,179072183	0,585	0,288	1	1,132155546	0,883270858	2,03125
S1PR2	3,15996E-05	0,179015407	0,159	0,032	0,666782936	1,132110992	0,883305619	4,96875
PMM1	3,76659E-08	0,17900629	0,305	0,067	0,000794787	1,132103838	0,8833112	4,552238806
MAP3K7	2,2314E-08	0,179001545	0,256	0,046	0,000470848	1,132100115	0,883314105	5,565217391
OSGEP	3,79244E-09	0,178933112	0,317	0,063	8,00244E-05	1,132046415	0,883356006	5,031746032
EFTUD2	3,34992E-08	0,17886556	0,244	0,042	0,000706866	1,131993411	0,883397368	5,80952381
CCS	9,72975E-07	0,178610622	0,354	0,105	0,02053075	1,131793394	0,883553487	3,371428571
NDUFV1	1,45148E-05	0,178499133	0,402	0,147	0,306275918	1,131705934	0,883621769	2,734693878
DTWD1	3,47822E-05	0,178492068	0,329	0,112	0,733938611	1,131700393	0,883626096	2,9375
PFDN2	0,007580823	0,178281603	0,671	0,389	1	1,131535308	0,883755012	1,724935733
OCEL1	6,44461E-06	0,178215189	0,207	0,046	0,135987778	1,131483219	0,883795697	4,5
NDFIP2	7,31031E-06	0,178035624	0,329	0,105	0,154254858	1,131342399	0,883905705	3,133333333
GFM2	1,36536E-06	0,177990353	0,098	0,004	0,028810488	1,131306898	0,883933441	24,5
SUPT4H1	2,39168E-05	0,177957556	0,39	0,147	0,504667401	1,13128118	0,883953537	2,653061224
MARCHF7	0,000102144	0,177931821	0,256	0,084	1	1,131261	0,883969305	3,047619048
RAP1B	8,34361E-05	0,177822654	0,695	0,344	1	1,131175402	0,884036196	2,020348837
PSMD11	0,000205624	0,177671052	0,427	0,182	1	1,131056542	0,884129098	2,346153846
CARM1	0,000406709	0,177525103	0,232	0,081	1	1,130942125	0,884218545	2,864197531
BECN1	0,000104049	0,177513357	0,207	0,06	1	1,130932918	0,884225743	3,45
RGL2	1,60803E-07	0,177396966	0,305	0,074	0,003393111	1,130841682	0,884297082	4,121621622
SAAL1	5,4137E-05	0,177390362	0,098	0,011	1	1,130836505	0,884301131	8,909090909
METTL3	2,97214E-05	0,177359615	0,183	0,042	0,627150464	1,130812405	0,884319977	4,357142857
PRKAR1A	0,000254871	0,177266536	0,659	0,326	1	1,130739451	0,884377033	2,021472393
UBE2A	2,51891E-06	0,177207778	0,524	0,189	0,053151473	1,130693399	0,884413052	2,772486772
CASP6	6,93636E-06	0,177164546	0,134	0,018	0,146364163	1,130659516	0,884439555	7,444444444
TMEM161B	1,37895E-06	0,177101748	0,146	0,018	0,029097295	1,130610302	0,884478054	8,111111111
MAP2	1,047E-05	0,177094022	0,11	0,011	0,220927383	1,130604247	0,884482791	10
SLC10A3	1,17809E-06	0,17708029	0,207	0,039	0,024858914	1,130593486	0,88449121	5,307692308
LASP1	1,02768E-05	0,176948474	0,427	0,158	0,21685103	1,13049019	0,884572028	2,702531646

Supplementary Table 2

Differentially expressed genes_keloid-XL_ comparing SC-Keloid vs. (SC-Myel, SC-Nonmyel, SC-Nonmyel2)								
**gene**	**p_val**	**avg_log2FC**	**pct.1**	**pct.2**	**p_val_adj**	**Foldchange_UP**	**Foldchange_DOWN**	**Ratio_pct1_pct2**
MRPS25	3,86364E-07	0,17694784	0,256	0,056	0,008152673	1,130489694	0,884572416	4,571428571
KCTD20	1,32748E-06	0,176709097	0,402	0,13	0,028011242	1,130302631	0,884718811	3,092307692
VASP	5,10767E-06	0,176677129	0,329	0,102	0,107776959	1,130277586	0,884738415	3,225490196
HEXA	1,13354E-06	0,176620245	0,39	0,123	0,023918817	1,130233021	0,8847733	3,170731707
ACO2	0,000689538	0,176587795	0,28	0,112	1	1,130207599	0,884793201	2,5
POLR2D	0,000153426	0,176535882	0,11	0,018	1	1,130166932	0,88482504	6,111111111
SUGT1	1,84178E-07	0,176473224	0,5	0,172	0,003886336	1,130117848	0,884863469	2,906976744
C6orf89	9,74531E-05	0,176462733	0,244	0,077	1	1,13010963	0,884869904	3,168831169
HOXB5	1,02952E-07	0,176396119	0,098	0	0,002172399	1,130057451	0,884910762	#DIV/0!
DDX39B	0,000973817	0,176356598	0,659	0,354	1	1,130026494	0,884935004	1,861581921
AAMDC	2,39131E-06	0,176291921	0,317	0,091	0,050458975	1,129975836	0,884974677	3,483516484
EIF3F	5,98473E-05	0,176201116	0,646	0,295	1	1,129904716	0,88503038	2,189830508
GLO1	2,00813E-07	0,176186826	0,439	0,137	0,004237346	1,129893524	0,885039147	3,204379562
SYNJ2	0,000275125	0,175940307	0,085	0,011	1	1,129700471	0,885190389	7,727272727
PRH1	0,000289263	0,175728761	0,146	0,035	1	1,129534832	0,885320197	4,171428571
MRPL27	1,0336E-08	0,175675283	0,329	0,07	0,000218099	1,129492963	0,885353015	4,7
IL7R	3,53478E-08	0,175550328	0,122	0,004	0,000745874	1,12939514	0,8854297	30,5
SNRNP35	8,28943E-09	0,175532559	0,207	0,025	0,000174915	1,129381229	0,885440606	8,28
TMEM30A	1,65227E-06	0,175507571	0,646	0,26	0,0348645	1,129361668	0,885455942	2,484615385
CSMD2	2,00519E-06	0,175414031	0,122	0,011	0,042311532	1,129288447	0,885513354	11,09090909
ZNF146	5,66206E-06	0,175347365	0,232	0,056	0,119475056	1,129236264	0,885554274	4,142857143
RIOK2	1,42472E-06	0,175333893	0,195	0,035	0,030063027	1,129225719	0,885562544	5,571428571
UFSP2	1,43452E-10	0,175272738	0,415	0,091	3,02699E-06	1,129177853	0,885600083	4,56043956
NDUFS1	4,56806E-08	0,174894297	0,366	0,095	0,000963906	1,128881691	0,88583242	3,852631579
MRPL22	1,33265E-05	0,174889464	0,439	0,165	0,281203077	1,128877909	0,885835387	2,660606061
GNPTG	0,003010501	0,174823811	0,28	0,126	1	1,128826539	0,8858757	2,222222222
MCRS1	3,36919E-08	0,174787621	0,317	0,07	0,000710933	1,128798222	0,885897923	4,528571429
SND1	5,45188E-06	0,174785552	0,439	0,158	0,115040123	1,128796604	0,885899193	2,778481013
PTPN14	4,0895E-06	0,174721771	0,354	0,116	0,086292601	1,128746701	0,885938359	3,051724138
GINM1	0,000258071	0,174697788	0,39	0,168	1	1,128727937	0,885953087	2,321428571

Supplementary Table 2

Differentially expressed genes_keloid-XL_ comparing SC-Keloid vs. (SC-Myel, SC-Nonmyel, SC-Nonmyel2)								
**gene**	**p_val**	**avg_log2FC**	**pct.1**	**pct.2**	**p_val_adj**	**Foldchange_UP**	**Foldchange_DOWN**	**Ratio_pct1_pct2**
PIP5K1C	3,90887E-07	0,174616174	0,207	0,035	0,008248112	1,128664086	0,886003207	5,914285714
ILF3	1,98236E-05	0,174531659	0,646	0,281	0,418297139	1,128597969	0,886055112	2,298932384
MRPL13	1,75178E-06	0,174507714	0,244	0,056	0,036964228	1,128579238	0,886069818	4,357142857
RPL34	0,055476334	0,174271223	0,988	0,912	1	1,128394253	0,886215077	1,083333333
CRELD2	5,54229E-08	0,17425207	0,354	0,091	0,001169479	1,128379273	0,886226842	3,89010989
SAR1A	3,88948E-06	0,174149835	0,634	0,267	0,082072011	1,128299314	0,886289646	2,374531835
SLC25A1	1,82206E-06	0,17393981	0,268	0,067	0,038447379	1,12813507	0,88641868	4
TRAPPC6B	2,84621E-06	0,173888156	0,256	0,063	0,060057957	1,128094679	0,886450418	4,063492063
DDX1	1,64054E-07	0,173768022	0,415	0,123	0,003461697	1,128000746	0,886524237	3,37398374
GMPPB	3,06022E-10	0,173751557	0,183	0,011	6,45737E-06	1,127987873	0,886534354	16,63636364
SREBF2	6,69901E-06	0,173718531	0,305	0,091	0,141355793	1,127962051	0,886554649	3,351648352
DOCK6	4,17761E-06	0,173517979	0,22	0,049	0,08815171	1,127805262	0,886677899	4,489795918
SLC17A9	8,92738E-07	0,173496396	0,171	0,025	0,018837654	1,12778839	0,886691164	6,84
RMDN1	3,2095E-08	0,173479382	0,28	0,056	0,000677237	1,12777509	0,886701621	5
DCAF13	1,13955E-05	0,173380829	0,256	0,07	0,240455983	1,127698052	0,886762195	3,657142857
RNF167	0,0004083	0,173335317	0,268	0,102	1	1,127662477	0,88679017	2,62745098
C19orf48	8,25615E-07	0,173240338	0,244	0,053	0,017421299	1,127588241	0,886848553	4,603773585
PGD	2,69541E-08	0,173150831	0,256	0,046	0,000568759	1,127518286	0,886903576	5,565217391
HIKESHI	1,22172E-07	0,173077894	0,451	0,137	0,002577949	1,127461284	0,886948416	3,291970803
ANKRA2	6,61706E-06	0,173016761	0,134	0,018	0,139626629	1,12741351	0,886986	7,444444444
PNKP	3,50403E-06	0,172992996	0,195	0,039	0,073938602	1,127394939	0,887000611	5
COG8	5,60121E-05	0,172879146	0,098	0,011	1	1,127305974	0,887070612	8,909090909
LAS1L	0,020196758	0,172816444	0,134	0,056	1	1,127256981	0,887109166	2,392857143
DDT	5,20716E-06	0,172796789	0,537	0,214	0,109876208	1,127241624	0,887121252	2,509345794
DBNL	5,17893E-06	0,17277703	0,317	0,095	0,109280678	1,127226185	0,887133402	3,336842105
FDPS	1,52027E-05	0,172537053	0,451	0,179	0,320791962	1,127038698	0,88728098	2,519553073
FUT11	0,000216184	0,172528556	0,171	0,046	1	1,127032061	0,887286205	3,717391304
ZDHHC16	1,11454E-06	0,172508316	0,159	0,021	0,023517921	1,127016249	0,887298653	7,571428571
POMGNT2	1,7018E-09	0,17246279	0,183	0,014	3,59096E-05	1,126980685	0,887326654	13,07142857
MAP7D3	3,52827E-07	0,172446076	0,244	0,049	0,007444996	1,126967629	0,887336933	4,979591837

Supplementary Table 2

Differentially expressed genes_keloid-XL_ comparing SC-Keloid vs. (SC-Myel, SC-Nonmyel, SC-Nonmyel2)								
**gene**	**p_val**	**avg_log2FC**	**pct.1**	**pct.2**	**p_val_adj**	**Foldchange_UP**	**Foldchange_DOWN**	**Ratio_pct1_pct2**
FADD	4,7289E-08	0,172403089	0,183	0,021	0,000997844	1,12693405	0,887363373	8,714285714
FDX2	2,06593E-08	0,172350919	0,268	0,049	0,000435932	1,126893299	0,887395462	5,469387755
RPP21	1,72558E-05	0,172348093	0,402	0,151	0,36411532	1,126891091	0,887397201	2,662251656
RUVBL1	9,60448E-08	0,172217779	0,22	0,035	0,002026641	1,126789307	0,88747736	6,285714286
DNAJC13	2,11962E-05	0,172193991	0,171	0,035	0,44726133	1,126770729	0,887491993	4,885714286
SCAP	0,000160436	0,172110282	0,195	0,056	1	1,126705352	0,887543489	3,482142857
TIMM17A	3,92746E-06	0,172101484	0,366	0,119	0,08287335	1,126698482	0,887548902	3,075630252
RWDD2A	8,68705E-06	0,172037344	0,122	0,014	0,183305486	1,126648391	0,887588362	8,714285714
MIOS	0,001049213	0,171678162	0,11	0,025	1	1,126367929	0,887809369	4,4
APPL2	2,28182E-05	0,171652307	0,171	0,035	0,481487455	1,126347743	0,88782528	4,885714286
SZRD1	8,87214E-05	0,171630047	0,415	0,168	1	1,126330365	0,887838978	2,470238095
EIF2B1	2,68515E-11	0,171602599	0,354	0,06	5,66594E-07	1,126308936	0,88785587	5,9
GDPD5	0,001876622	0,171577587	0,049	0,004	1	1,126289409	0,887871263	12,25
NRDE2	0,000174671	0,171545962	0,244	0,081	1	1,12626472	0,887890726	3,012345679
CTNNBL1	6,56905E-07	0,171498755	0,22	0,042	0,013861358	1,126227868	0,887919779	5,238095238
ARSA	0,000141395	0,171356682	0,195	0,056	1	1,126116965	0,888007224	3,482142857
HS3ST3B1	0,001952737	0,171296221	0,049	0,004	1	1,126069772	0,88804444	12,25
MRPS18A	1,82755E-05	0,171290647	0,171	0,035	0,385631777	1,126065422	0,888047871	4,885714286
MYEOV	5,5905E-11	0,171180616	0,146	0	1,17965E-06	1,125979543	0,888115602	#DIV/0!
IKBKG	7,97812E-05	0,171123942	0,159	0,035	1	1,125935311	0,888150492	4,542857143
TCIRG1	6,36417E-07	0,171088933	0,183	0,028	0,013429045	1,125907989	0,888172044	6,535714286
PLXND1	0,000106922	0,170838932	0,463	0,2	1	1,125712901	0,888325966	2,315
MTG2	0,000223551	0,170666483	0,183	0,053	1	1,125578349	0,888432157	3,452830189
UBFD1	2,82044E-07	0,170611772	0,244	0,049	0,005951411	1,125535664	0,888465849	4,979591837
CMTR2	5,3728E-09	0,170490052	0,28	0,049	0,000113372	1,125440708	0,888540812	5,714285714
NAT10	0,002321451	0,170463093	0,11	0,028	1	1,125419677	0,888557416	3,928571429
WAC-AS1	7,14685E-07	0,170434125	0,183	0,028	0,015080561	1,12539708	0,888575258	6,535714286
HPRT1	8,62361E-05	0,170425604	0,232	0,07	1	1,125390433	0,888580506	3,314285714
MSL3	1,23359E-05	0,17029836	0,256	0,07	0,260299981	1,125291179	0,888658881	3,657142857
CAMK2N2	1,58349E-08	0,170161385	0,11	0	0,000334132	1,125184345	0,888743258	#DIV/0!

Supplementary Table 2

Differentially expressed genes_keloid-XL_ comparing SC-Keloid vs. (SC-Myel, SC-Nonmyel, SC-Nonmyel2)								
**gene**	**p_val**	**avg_log2FC**	**pct.1**	**pct.2**	**p_val_adj**	**Foldchange_UP**	**Foldchange_DOWN**	**Ratio_pct1_pct2**
RALBP1	0,000398295	0,17014664	0,561	0,26	1	1,125172845	0,888752341	2,157692308
SGCB	1,18547E-05	0,169991182	0,671	0,305	0,250145686	1,125051608	0,888848114	2,2
ZNF3	2,82921E-07	0,169947257	0,134	0,011	0,005969912	1,125017354	0,888875177	12,18181818
RGS8	6,67422E-07	0,169931495	0,085	0	0,014083281	1,125005064	0,888884888	#DIV/0!
TTC8	7,86343E-06	0,169872685	0,122	0,014	0,165926312	1,124959205	0,888921123	8,714285714
GLTP	9,10158E-05	0,16969389	0,317	0,116	1	1,124819796	0,889031295	2,732758621
SIRT7	1,54086E-05	0,169609704	0,244	0,067	0,325135945	1,124754161	0,889083175	3,641791045
UNC5B	0,000276795	0,16959874	0,183	0,053	1	1,124745613	0,889089931	3,452830189
PACC1	3,62311E-06	0,169433207	0,159	0,025	0,076451158	1,124616569	0,88919195	6,36
BBIP1	1,80554E-06	0,169429759	0,354	0,109	0,038098798	1,124613881	0,889194076	3,247706422
TRAM2-AS1	0,000685342	0,16937977	0,159	0,046	1	1,124574914	0,889224886	3,456521739
DNMT3A	1,74547E-06	0,169376948	0,268	0,067	0,036831262	1,124572714	0,889226626	4
PGP	1,64229E-07	0,169342577	0,427	0,126	0,003465402	1,124545922	0,889247811	3,388888889
INSYN2A	1,33545E-06	0,169268125	0,098	0,004	0,028179294	1,12448789	0,889293703	24,5
ZNRF1	0,00097948	0,16913588	0,183	0,06	1	1,124384819	0,889375224	3,05
VIPAS39	0,00109994	0,169113168	0,11	0,025	1	1,124367118	0,889389225	4,4
NGDN	3,39554E-05	0,169111451	0,268	0,084	0,716492954	1,12436578	0,889390284	3,19047619
F2R	0,0016151	0,169110456	0,22	0,084	1	1,124365005	0,889390897	2,619047619
C1QTNF6	0,000685342	0,168896844	0,159	0,046	1	1,124198538	0,889522594	3,456521739
CBFB	5,54468E-06	0,168781999	0,512	0,196	0,116998267	1,12410905	0,889593407	2,612244898
SPG21	2,01615E-06	0,168700515	0,305	0,084	0,042542862	1,124045562	0,889643653	3,630952381
HS3ST3A1	1,5822E-05	0,168617795	0,183	0,039	0,333859568	1,123981114	0,889694664	4,692307692
NSD2	0,001366307	0,168539003	0,146	0,042	1	1,12391973	0,889743256	3,476190476
P3H4	5,38783E-06	0,168475064	0,183	0,035	0,113688628	1,12386992	0,889782689	5,228571429
TPD52L2	2,51654E-07	0,168460872	0,39	0,112	0,00531015	1,123858865	0,889791442	3,482142857
CERS5	9,87009E-08	0,168104547	0,28	0,06	0,002082687	1,123581322	0,890011235	4,666666667
LCTL	5,08758E-08	0,168030007	0,134	0,007	0,001073531	1,123523271	0,890057221	19,14285714
RNASEH1-AS1	3,16576E-07	0,167879383	0,146	0,014	0,006680081	1,123405976	0,890150152	10,42857143
POLR3F	0,004199164	0,167828288	0,073	0,014	1	1,12336619	0,890181678	5,214285714
TMED7	4,81185E-05	0,167776951	0,476	0,2	1	1,123326217	0,890213355	2,38

Supplementary Table 2

Differentially expressed genes_keloid-XL_ comparing SC-Keloid vs. (SC-Myel, SC-Nonmyel, SC-Nonmyel2)								
**gene**	**p_val**	**avg_log2FC**	**pct.1**	**pct.2**	**p_val_adj**	**Foldchange_UP**	**Foldchange_DOWN**	**Ratio_pct1_pct2**
AK1	1,89511E-06	0,167720021	0,415	0,137	0,039988737	1,12328189	0,890248484	3,02919708
HTATSF1	6,6303E-07	0,167680518	0,329	0,091	0,013990596	1,123251134	0,890272861	3,615384615
SMARCA4	6,67316E-05	0,167670817	0,268	0,088	1	1,123243581	0,890278847	3,045454545
CLPP	1,9583E-08	0,167639645	0,317	0,067	0,000413222	1,123219311	0,890298083	4,731343284
PARP1	1,56982E-08	0,167538508	0,427	0,116	0,000331248	1,123140573	0,890360498	3,681034483
CENPW	4,95036E-09	0,167500748	0,134	0,004	0,000104458	1,123111177	0,890383802	33,5
TAOK2	0,000314739	0,16747038	0,22	0,074	1	1,123087537	0,890402544	2,972972973
FARP1	1,78419E-05	0,167468024	0,146	0,025	0,376480972	1,123085703	0,890403998	5,84
SEZ6L2	9,20529E-08	0,167352276	0,22	0,035	0,001942408	1,122995601	0,890475438	6,285714286
ZNF251	1,02863E-05	0,167305686	0,11	0,011	0,217050943	1,122959336	0,890504196	10
MRPS2	1,30385E-07	0,167272542	0,207	0,032	0,002751254	1,122933538	0,890524654	6,46875
RNF139	7,61401E-05	0,167138931	0,256	0,081	1	1,122829545	0,890607131	3,160493827
RNF40	1,60931E-05	0,167001524	0,28	0,084	0,339580086	1,122722608	0,89069196	3,333333333
CCNY	6,40156E-09	0,166976157	0,366	0,084	0,000135079	1,122702867	0,890707621	4,357142857
UBE2L6	8,92496E-08	0,166877262	0,207	0,032	0,001883256	1,12262591	0,89076868	6,46875
SNX6	0,00020312	0,166817975	0,512	0,239	1	1,122579777	0,890805286	2,142259414
STAMBP	9,94033E-08	0,166810023	0,329	0,081	0,002097509	1,12257359	0,890810197	4,061728395
YPEL3	0,00252273	0,166762379	0,415	0,211	1	1,122536518	0,890839616	1,966824645
RRP15	9,21927E-06	0,16672122	0,305	0,095	0,19453582	1,122504493	0,890865031	3,210526316
GIT2	0,002296533	0,166666848	0,11	0,028	1	1,122462189	0,890898606	3,928571429
SEC31A	5,57713E-07	0,166625202	0,598	0,225	0,011768307	1,122429788	0,890924324	2,657777778
NUDT19	0,006076786	0,166569506	0,061	0,011	1	1,122386457	0,890958719	5,545454545
VBP1	1,5347E-06	0,166540566	0,415	0,137	0,032383681	1,122363942	0,890976592	3,02919708
BEND6	3,89526E-06	0,1665109	0,159	0,025	0,08219389	1,122340863	0,890994913	6,36
RNF157	3,16224E-05	0,166467498	0,122	0,018	0,667263526	1,1223071	0,891021718	6,777777778
TAF11	4,27361E-08	0,166377379	0,329	0,077	0,000901775	1,122236996	0,891077378	4,272727273
CLSTN1	2,57351E-05	0,16637594	0,659	0,305	0,543035814	1,122235877	0,891078267	2,160655738
ZNF655	2,47738E-06	0,166329821	0,232	0,053	0,052275269	1,122200003	0,891106753	4,377358491
LRRC23	7,35655E-07	0,16607509	0,183	0,028	0,015523062	1,122001877	0,891264106	6,535714286
NOC2L	6,00108E-05	0,166052346	0,256	0,081	1	1,12198419	0,891278156	3,160493827

Supplementary Table 2

Differentially expressed genes_keloid-XL_ comparing SC-Keloid vs. (SC-Myel, SC-Nonmyel, SC-Nonmyel2)								
**gene**	**p_val**	**avg_log2FC**	**pct.1**	**pct.2**	**p_val_adj**	**Foldchange_UP**	**Foldchange_DOWN**	**Ratio_pct1_pct2**
SCFD1	1,27407E-05	0,16603644	0,329	0,105	0,268840694	1,121971819	0,891287983	3,133333333
ROGDI	0,000590302	0,165930214	0,098	0,018	1	1,121889211	0,891353611	5,444444444
TRIM14	2,04948E-09	0,165886384	0,195	0,018	4,3246E-05	1,121855128	0,891380691	10,83333333
ZNF524	2,47712E-06	0,165550751	0,28	0,074	0,052269763	1,121594166	0,89158809	3,783783784
F8	0,008614542	0,165433119	0,049	0,007	1	1,121502719	0,891660789	7
PCBD1	9,46653E-07	0,165426068	0,293	0,074	0,019975331	1,121497238	0,891665147	3,959459459
PMAIP1	4,69377E-05	0,165371203	0,146	0,028	0,990433334	1,121454589	0,891699057	5,214285714
HDAC1	6,73816E-07	0,165338724	0,293	0,074	0,014218182	1,121429342	0,891719132	3,959459459
RIDA	0,00037988	0,165297044	0,11	0,021	1	1,121396944	0,891744895	5,238095238
PRDX6	6,43711E-05	0,165292985	0,671	0,312	1	1,12139379	0,891747403	2,150641026
DPYSL3	4,31746E-10	0,165276732	0,22	0,021	9,11026E-06	1,121381156	0,89175745	10,47619048
ENSA	6,57335E-05	0,165211101	0,476	0,2	1	1,121330143	0,891798019	2,38
NUBP1	3,90887E-07	0,165192203	0,207	0,035	0,008248112	1,121315455	0,8918097	5,914285714
GPATCH2	1,54134E-05	0,165092303	0,329	0,109	0,325239048	1,121237812	0,891871456	3,018348624
PTGR1	4,24453E-10	0,164996375	0,329	0,06	8,95639E-06	1,121163261	0,891930761	5,483333333
BLOC1S4	7,65346E-08	0,164963465	0,22	0,035	0,001614956	1,121137686	0,891951107	6,285714286
TTC17	0,000613079	0,164899501	0,305	0,123	1	1,121087979	0,891990654	2,479674797
FKBP1B	0,000444147	0,164651923	0,11	0,021	1	1,120895608	0,89214374	5,238095238
TRPV4	5,55398E-09	0,164537207	0,134	0,004	0,000117194	1,120806484	0,892214682	33,5
SLC25A45	0,00380062	0,164512106	0,098	0,025	1	1,120786984	0,892230205	3,92
MAP2K5	0,009081904	0,164394662	0,11	0,035	1	1,120695749	0,89230284	3,142857143
YIPF5	3,85E-07	0,164330105	0,317	0,084	0,00812389	1,120645602	0,89234277	3,773809524
CYB561D2	1,7192E-06	0,164312984	0,183	0,032	0,036276865	1,120632302	0,89235336	5,71875
APOBEC3C	7,55226E-06	0,16423543	0,305	0,091	0,159360183	1,120572063	0,892401331	3,351648352
MYCBP	1,37895E-06	0,164081086	0,146	0,018	0,029097295	1,120452187	0,892496808	8,111111111
ACY1	1,24261E-06	0,164068454	0,159	0,021	0,02622034	1,120442376	0,892504622	7,571428571
CEP63	3,853E-06	0,164007679	0,268	0,07	0,081302136	1,120395178	0,892542221	3,828571429
MAN2B1	1,18479E-05	0,163949106	0,329	0,109	0,250001937	1,120349691	0,892578458	3,018348624
TMEM199	7,36862E-09	0,163911272	0,146	0,007	0,000155485	1,12032031	0,892601866	20,85714286
EIF3B	8,89321E-05	0,163821846	0,366	0,14	1	1,120250869	0,892657196	2,614285714

Supplementary Table 2

Differentially expressed genes_keloid-XL_ comparing SC-Keloid vs. (SC-Myel, SC-Nonmyel, SC-Nonmyel2)								
gene	p_val	avg_log2FC	pct.1	pct.2	p_val_adj	Foldchange_UP	Foldchange_DOWN	Ratio_pct1_pct2
COA5	7,99092E-07	0,163671398	0,293	0,074	0,016861648	1,120134052	0,89275029	3,959459459
TMEM8B	0,001428181	0,163604326	0,122	0,032	1	1,120081978	0,892791795	3,8125
ANAPC5	1,92922E-07	0,163559772	0,512	0,168	0,004070853	1,120047388	0,892819367	3,047619048
C2orf74	0,001024669	0,163489049	0,11	0,025	1	1,119992483	0,892863136	4,4
DUT	0,004183708	0,163244477	0,488	0,253	1	1,119802633	0,89301451	1,928853755
UQCRFS1	1,88117E-07	0,162758552	0,378	0,105	0,003969465	1,119425527	0,893315344	3,6
TMEM42	1,45421E-05	0,162703115	0,183	0,039	0,306852515	1,119382513	0,893349672	4,692307692
SIKE1	5,83999E-07	0,162624052	0,244	0,053	0,012322958	1,11932117	0,89339863	4,603773585
RPP38	0,000194727	0,162520298	0,134	0,028	1	1,119240675	0,893462883	4,785714286
PLOD2	7,09401E-07	0,162496773	0,22	0,042	0,014969078	1,119222424	0,893477452	5,238095238
RNPS1	4,85095E-05	0,16243191	0,524	0,225	1	1,119172106	0,893517623	2,328888889
RPL22	0,054032787	0,162339417	0,951	0,877	1	1,119100356	0,89357491	1,084378563
MAD2L1BP	1,47187E-05	0,162252996	0,183	0,039	0,310578899	1,119033321	0,893628439	4,692307692
MICALL1	8,65211E-10	0,162181154	0,146	0,004	1,82568E-05	1,118977598	0,89367294	36,5
RBMS2	1,8284E-06	0,162144776	0,293	0,077	0,038581109	1,118949383	0,893695474	3,805194805
UBALD2	0,003541359	0,162098302	0,585	0,326	1	1,118913339	0,893724264	1,794478528
PDHB	6,26271E-08	0,162072236	0,366	0,095	0,001321495	1,118893123	0,893740412	3,852631579
ANGEL2	0,000164834	0,162053956	0,183	0,049	1	1,118878946	0,893751736	3,734693878
BAP1	4,1788E-06	0,162049615	0,195	0,039	0,088176872	1,118875579	0,893754425	5
ZNF775	2,11962E-05	0,162010378	0,171	0,035	0,44726133	1,118845149	0,893778733	4,885714286
SH3YL1	4,63516E-05	0,161970453	0,268	0,084	0,978065431	1,118814187	0,893803468	3,19047619
COPS5	2,20468E-06	0,161790347	0,354	0,109	0,046520996	1,118674523	0,893915057	3,247706422
ZDHHC5	4,28183E-08	0,161633426	0,28	0,056	0,000903509	1,118552852	0,894012293	5
CEP78	5,60121E-05	0,161548759	0,098	0,011	1	1,11848721	0,894064761	8,909090909
CCDC24	8,1138E-06	0,161541999	0,085	0,004	0,171209209	1,118481969	0,894068951	21,25
TLN1	0,000353318	0,16151746	0,695	0,354	1	1,118462945	0,894084158	1,963276836
UBAC2	5,62892E-06	0,161331661	0,268	0,074	0,118775827	1,118318912	0,894199311	3,621621622
GLMP	7,44051E-06	0,16127959	0,232	0,056	0,157002258	1,118278549	0,894231585	4,142857143
TMEM45A	3,52728E-05	0,161059134	0,183	0,042	0,744292094	1,11810768	0,894368242	4,357142857
DYNC2H1	0,000839097	0,161043444	0,171	0,053	1	1,11809552	0,894377969	3,226415094

Supplementary Table 2

Differentially expressed genes_keloid-XL_ comparing SC-Keloid vs. (SC-Myel, SC-Nonmyel, SC-Nonmyel2)								
**gene**	**p_val**	**avg_log2FC**	**pct.1**	**pct.2**	**p_val_adj**	**Foldchange_UP**	**Foldchange_DOWN**	**Ratio_pct1_pct2**
KLHL36	0,006415308	0,160965159	0,207	0,088	1	1,11803485	0,894426502	2,352272727
CLDN5	0,003759144	0,160936126	0,098	0,025	1	1,118012351	0,894444502	3,92
COASY	0,00020114	0,160844287	0,171	0,046	1	1,117941183	0,894501442	3,717391304
POLR3G	4,42145E-07	0,160835013	0,207	0,035	0,009329705	1,117933996	0,894507192	5,914285714
SLC25A39	6,7035E-07	0,160717832	0,341	0,095	0,014145062	1,117843198	0,89457985	3,589473684
TIA1	5,81883E-06	0,160665921	0,378	0,126	0,122783037	1,117802976	0,894612039	3
ROPN1	2,31826E-10	0,160398162	0,171	0,007	4,89176E-06	1,117595535	0,894778092	24,42857143
TNK2	4,21083E-05	0,160340755	0,317	0,109	0,888528264	1,117551065	0,894813697	2,908256881
C6orf226	1,22548E-06	0,160327351	0,232	0,049	0,025858797	1,117540682	0,894822011	4,734693878
COMMD1	1,75605E-07	0,160112792	0,378	0,105	0,003705448	1,117374493	0,894955099	3,6
MPV17	1,01178E-07	0,160094998	0,39	0,109	0,00213495	1,117360711	0,894966137	3,577981651
C1orf56	0,000332001	0,160094862	0,366	0,158	1	1,117360606	0,894966222	2,316455696
SHARPIN	2,69483E-05	0,160046242	0,317	0,105	0,568635509	1,117322951	0,894996384	3,019047619
CCT5	4,45417E-05	0,160035122	0,463	0,189	0,939873909	1,117314338	0,895003282	2,44973545
MVK	2,2307E-07	0,15999355	0,11	0,004	0,004706999	1,117282143	0,895029073	27,5
TTL	2,47712E-06	0,159986464	0,28	0,074	0,052269763	1,117276655	0,895033468	3,783783784
XPNPEP1	4,05203E-06	0,159922194	0,244	0,06	0,085501984	1,117226884	0,895073342	4,066666667
CCT7	1,02028E-05	0,159782764	0,488	0,186	0,215290172	1,117118914	0,895159851	2,623655914
RPA2	3,9635E-05	0,159657986	0,171	0,039	0,836337258	1,117022298	0,895237276	4,384615385
NBPF1	8,26781E-07	0,159234711	0,22	0,042	0,017445897	1,116694621	0,89549997	5,238095238
ZNF84	1,84929E-06	0,159233283	0,134	0,014	0,039021837	1,116693516	0,895500856	9,571428571
ADAM10	0,000264134	0,159218736	0,598	0,284	1	1,116682256	0,895509886	2,105633803
TMEM121	2,90594E-08	0,159176521	0,22	0,032	0,000613182	1,116649582	0,89553609	6,875
METTL5	6,88037E-08	0,159089219	0,366	0,095	0,001451826	1,116582011	0,895590283	3,852631579
ZNF133	0,037995958	0,158882646	0,073	0,025	1	1,116422145	0,895718528	2,92
FBXO22	1,65209E-06	0,158868601	0,22	0,046	0,034860754	1,116411276	0,895727248	4,782608696
RECQL	0,000312434	0,158790149	0,28	0,105	1	1,116350568	0,895775958	2,666666667
PKIA	3,44186E-07	0,158742426	0,244	0,049	0,007262675	1,116313642	0,89580559	4,979591837
NDUFAF5	6,18181E-07	0,158715844	0,183	0,028	0,013044241	1,116293073	0,895822096	6,535714286
GLCE	2,30998E-05	0,158545439	0,171	0,035	0,487428804	1,116161229	0,895927912	4,885714286

Supplementary Table 2

Differentially expressed genes_keloid-XL_ comparing SC-Keloid vs. (SC-Myel, SC-Nonmyel, SC-Nonmyel2)								
gene	p_val	avg_log2FC	pct.1	pct.2	p_val_adj	Foldchange_UP	Foldchange_DOWN	Ratio_pct1_pct2
ZADH2	3,71682E-08	0,158453665	0,207	0,028	0,000784286	1,116090229	0,895984907	7,392857143
NFS1	0,000210889	0,158405399	0,098	0,014	1	1,11605289	0,896014883	7
RPL30	0,261451897	0,158375036	0,963	0,919	1	1,116029402	0,896033741	1,047878128
PRPF38A	3,13385E-06	0,158358015	0,28	0,074	0,066127404	1,116016235	0,896044312	3,783783784
CARF	0,00174496	0,158348685	0,098	0,021	1	1,116009018	0,896050107	4,666666667
YBEY	5,7507E-09	0,158312817	0,341	0,074	0,000121346	1,115981272	0,896072385	4,608108108
TMEM248	1,19347E-05	0,158246299	0,366	0,126	0,251834589	1,115929819	0,896113701	2,904761905
DPH2	0,001703135	0,158214896	0,098	0,021	1	1,115905529	0,896133207	4,666666667
CDC42SE2	1,90706E-05	0,158193965	0,22	0,056	0,402407918	1,115889339	0,896146208	3,928571429
DDX49	6,87095E-08	0,158162992	0,317	0,074	0,001449839	1,115865383	0,896165447	4,283783784
DRG1	3,3093E-06	0,158003602	0,22	0,049	0,069829535	1,115742108	0,896264462	4,489795918
CDC16	4,72583E-07	0,157943486	0,256	0,056	0,009971983	1,115695617	0,896301809	4,571428571
MED7	3,64556E-07	0,15787622	0,146	0,014	0,007692497	1,115643599	0,8963436	10,42857143
SLC35F6	6,55188E-11	0,157849239	0,22	0,018	1,38251E-06	1,115622734	0,896360364	12,22222222
ALDH16A1	1,27887E-05	0,157772899	0,159	0,028	0,26985512	1,115563703	0,896407796	5,678571429
API5	7,86753E-06	0,157257121	0,256	0,067	0,166012805	1,115164949	0,896728328	3,820895522
BCL10	3,18917E-06	0,157149869	0,305	0,088	0,067294762	1,115082049	0,896794994	3,465909091
PAFAH1B3	2,29032E-05	0,157138136	0,232	0,063	0,483279746	1,11507298	0,896802288	3,682539683
COX7B	0,013370272	0,157137745	0,659	0,428	1	1,115072678	0,896802531	1,539719626
LZTR1	2,12429E-06	0,157127435	0,244	0,056	0,044824715	1,11506471	0,89680894	4,357142857
TRAF7	0,00012023	0,157106802	0,268	0,091	1	1,115048762	0,896821766	2,945054945
GRHPR	2,60469E-06	0,157068128	0,451	0,161	0,054961627	1,115018872	0,896845807	2,801242236
DCTN6	1,68627E-05	0,156929891	0,561	0,232	0,355819959	1,114912037	0,896931746	2,418103448
PTBP1	0,000228288	0,156898773	0,598	0,284	1	1,11488799	0,896951092	2,105633803
MTIF3	1,17297E-06	0,156864355	0,366	0,112	0,024750878	1,114861392	0,896972491	3,267857143
ILRUN	0,002077581	0,156682764	0,232	0,091	1	1,114721074	0,897085399	2,549450549
ADAMTS12	5,25571E-08	0,156639922	0,146	0,011	0,001109008	1,114687972	0,897112039	13,27272727
PSMD9	8,14288E-07	0,156592204	0,476	0,168	0,017182298	1,114651104	0,897141712	2,833333333
SNAI3-AS1	0,008751649	0,156506303	0,049	0,007	1	1,114584737	0,897195132	7
ADA2	1,33545E-06	0,156492944	0,098	0,004	0,028179294	1,114574417	0,897203439	24,5

Supplementary Table 2

Differentially expressed genes_keloid-XL_ comparing SC-Keloid vs. (SC-Myel, SC-Nonmyel, SC-Nonmyel2)								
**gene**	**p_val**	**avg_log2FC**	**pct.1**	**pct.2**	**p_val_adj**	**Foldchange_UP**	**Foldchange_DOWN**	**Ratio_pct1_pct2**
AMPH	3,53478E-08	0,156414853	0,122	0,004	0,000745874	1,114514088	0,897252005	30,5
CBX5	0,000636146	0,156392372	0,561	0,291	1	1,114496721	0,897265987	1,927835052
CALHM2	0,00017875	0,156318683	0,134	0,028	1	1,114439797	0,897311818	4,785714286
ZNF766	0,00010679	0,156309081	0,122	0,021	1	1,114432379	0,89731779	5,80952381
SANBR	0,001474456	0,156187671	0,122	0,032	1	1,114338598	0,897393307	3,8125
YWHAH	0,00026985	0,156112283	0,659	0,326	1	1,114280371	0,897440201	2,021472393
SRSF9	2,01206E-05	0,156056562	0,537	0,221	0,424565537	1,114237335	0,897474864	2,429864253
CYP20A1	6,40582E-09	0,156043397	0,28	0,049	0,000135169	1,114227167	0,897483054	5,714285714
ATP2B2	0,001003155	0,155960639	0,134	0,035	1	1,114163253	0,897534538	3,828571429
LEF1	2,3778E-06	0,155850443	0,244	0,056	0,050173887	1,114078154	0,897603096	4,357142857
CNOT8	1,03076E-06	0,155821415	0,28	0,07	0,021750085	1,114055738	0,897621156	4
EXOC4	1,25713E-08	0,155682536	0,256	0,042	0,000265267	1,1139485	0,897707569	6,095238095
UCK1	3,10799E-05	0,155677239	0,207	0,053	0,655817737	1,11394441	0,897710865	3,905660377
MAP1LC3A	0,001122356	0,15559704	0,622	0,337	1	1,113882488	0,89776077	1,845697329
LCMT1	4,01294E-08	0,155577368	0,207	0,028	0,00084677	1,1138673	0,897773012	7,392857143
CTNNA1	0,005856707	0,155549299	0,854	0,649	1	1,113845629	0,897790479	1,31587057
CUEDC1	6,2031E-07	0,155455897	0,39	0,119	0,013089169	1,113773519	0,897848605	3,277310924
CLDN7	0,001839595	0,155442353	0,049	0,004	1	1,113763063	0,897857034	12,25
UBA6	7,37793E-05	0,155293421	0,366	0,14	1	1,113648093	0,897949726	2,614285714
SYCE1L	4,78823E-05	0,155219305	0,073	0,004	1	1,113590883	0,897995858	18,25
H2AZ2	0,002248882	0,155218005	0,634	0,34	1	1,11358988	0,897996667	1,864705882
CEP55	0,009031726	0,155182325	0,049	0,007	1	1,113562339	0,898018876	7
ANAPC10	9,03745E-05	0,155100923	0,195	0,053	1	1,113499509	0,898069547	3,679245283
FBXO38	0,02175178	0,155091666	0,073	0,021	1	1,113492365	0,898075309	3,476190476
UBL4A	2,92313E-09	0,155031517	0,244	0,035	6,1681E-05	1,113445942	0,898112752	6,971428571
CBARP	0,009329829	0,154742167	0,11	0,035	1	1,11322265	0,898292898	3,142857143
EXOC7	0,000446164	0,154715133	0,28	0,109	1	1,113201789	0,898309731	2,568807339
DNAL4	2,98523E-07	0,154502735	0,171	0,021	0,006299136	1,113037913	0,898441992	8,142857143
PPIL1	3,29037E-07	0,154484324	0,134	0,011	0,006943005	1,113023709	0,898453458	12,18181818
APLNR	9,45499E-06	0,154469586	0,098	0,007	0,199509675	1,113012338	0,898462637	14

Supplementary Table 2

Differentially expressed genes_keloid-XL_ comparing SC-Keloid vs. (SC-Myel, SC-Nonmyel, SC-Nonmyel2)								
gene	p_val	avg_log2FC	pct.1	pct.2	p_val_adj	Foldchange_UP	Foldchange_DOWN	Ratio_pct1_pct2
PHF11	0,003846395	0,154458327	0,22	0,091	1	1,113003653	0,898469648	2,417582418
INO80E	0,000336578	0,154455975	0,207	0,067	1	1,113001838	0,898471113	3,089552239
SGO2	0,001803241	0,154346171	0,049	0,004	1	1,11291713	0,898539498	12,25
MMS19	1,65923E-08	0,154193082	0,244	0,039	0,000350114	1,112799041	0,89863485	6,256410256
SEC22A	1,90824E-09	0,154165206	0,171	0,011	4,02658E-05	1,112777539	0,898652214	15,54545455
ATP6V0E2-AS1	0,008401736	0,154088921	0,024	0	1	1,112718701	0,898699733	#DIV/0!
HIRA	0,001474456	0,154081802	0,122	0,032	1	1,112713211	0,898704168	3,8125
SPACA9	1,6403E-06	0,153972629	0,134	0,014	0,034611876	1,112629011	0,898772178	9,571428571
ZNF211	0,001126146	0,153819296	0,11	0,025	1	1,112510765	0,898867707	4,4
BMERB1	1,06086E-05	0,153798227	0,244	0,063	0,223852694	1,112494518	0,898880834	3,873015873
ZNF585B	0,000332109	0,153740684	0,073	0,007	1	1,112450146	0,898916687	10,42857143
RFNG	0,000421287	0,153526475	0,305	0,119	1	1,112284984	0,899050167	2,56302521
CCNB1IP1	4,99173E-06	0,153402119	0,317	0,095	0,105330494	1,112189112	0,899127666	3,336842105
RPS6KB2	0,011312565	0,153374927	0,171	0,07	1	1,112168149	0,899144613	2,442857143
DUSP23	6,51755E-08	0,153118665	0,39	0,105	0,001375268	1,111970616	0,899304339	3,714285714
SLC35C2	6,4873E-09	0,153057075	0,232	0,032	0,000136889	1,111923146	0,899342732	7,25
NUDT2	4,97361E-07	0,152876475	0,28	0,067	0,010494812	1,111783961	0,899455321	4,179104478
FBXL17	2,68861E-05	0,152869374	0,232	0,063	0,567322603	1,11177849	0,899459748	3,682539683
GPR108	0,00390695	0,152709392	0,207	0,084	1	1,111655209	0,899559496	2,464285714
SF3B5	6,48146E-06	0,152622331	0,561	0,232	0,136765335	1,111588128	0,899613782	2,418103448
MICU1	1,32108E-05	0,152593733	0,378	0,133	0,278761391	1,111566093	0,899631615	2,842105263
TM2D1	0,009487909	0,152522592	0,171	0,07	1	1,111511282	0,899675978	2,442857143
MAN1A2	1,02768E-05	0,152477202	0,451	0,165	0,216851261	1,111476312	0,899704284	2,733333333
ATG3	9,93562E-05	0,152457527	0,354	0,14	1	1,111461155	0,899716554	2,528571429
TMEM33	3,28733E-05	0,152194599	0,329	0,112	0,693659897	1,111258612	0,89988054	2,9375
CAMTA2	3,36584E-06	0,152084562	0,244	0,06	0,07102265	1,111173857	0,899949179	4,066666667
TNFAIP1	6,3637E-06	0,15183991	0,183	0,035	0,13428035	1,11098544	0,900101805	5,228571429
UMPS	5,08758E-08	0,151740767	0,134	0,007	0,001073531	1,110909095	0,900163662	19,14285714
ZBTB17	0,001659385	0,151709866	0,134	0,039	1	1,110885301	0,900182943	3,435897436
NDRG4	6,67422E-07	0,151657049	0,085	0	0,014083281	1,110844632	0,9002159	#DIV/0!

Supplementary Table 2

Differentially expressed genes_keloid-XL_ comparing SC-Keloid vs. (SC-Myel, SC-Nonmyel, SC-Nonmyel2)								
**gene**	**p_val**	**avg_log2FC**	**pct.1**	**pct.2**	**p_val_adj**	**Foldchange_UP**	**Foldchange_DOWN**	**Ratio_pct1_pct2**
NLRX1	0,026685693	0,151622185	0,049	0,011	1	1,110817788	0,900237654	4,454545455
AIMP2	8,40774E-07	0,151591028	0,171	0,025	0,017741166	1,110793798	0,900257097	6,84
BRD3OS	2,47998E-05	0,151471804	0,134	0,021	0,523300999	1,110702006	0,900331497	6,380952381
CDKN2B	0,000412162	0,151402768	0,159	0,042	1	1,110648859	0,90037458	3,785714286
WDR13	7,23726E-05	0,151306914	0,451	0,182	1	1,110575069	0,900434404	2,478021978
CUL4B	6,83314E-06	0,15124506	0,207	0,046	0,144186145	1,110527454	0,90047301	4,5
TMEM267	8,07227E-05	0,151061669	0,159	0,035	1	1,110386297	0,900587483	4,542857143
GBA	9,41154E-05	0,151035475	0,195	0,053	1	1,110366136	0,900603834	3,679245283
GTF2E1	6,67422E-07	0,150930807	0,085	0	0,014083281	1,110285582	0,900669175	#DIV/0!
BEND5	1,83428E-05	0,150791314	0,146	0,025	0,387051502	1,110178235	0,900756265	5,84
MUL1	1,90655E-09	0,150561256	0,195	0,018	4,02302E-05	1,110001215	0,900899915	10,83333333
ARFRP1	7,26295E-05	0,150552404	0,305	0,109	1	1,109994404	0,900905442	2,798165138
NT5C3A	2,43477E-06	0,150364242	0,354	0,109	0,051376124	1,109849644	0,90102295	3,247706422
RPS12	0,026100466	0,150126059	0,976	0,912	1	1,109666428	0,901171717	1,070175439
ZNF576	0,000702674	0,15004971	0,122	0,028	1	1,109607704	0,901219409	4,357142857
C4orf3	0,002130089	0,150038138	0,683	0,404	1	1,109598804	0,901226638	1,690594059
ARL4D	8,25251E-08	0,14998468	0,256	0,049	0,001741362	1,10955769	0,901260033	5,224489796
IPO4	1,81794E-06	0,149907736	0,134	0,014	0,038360322	1,109498514	0,901308102	9,571428571
LINC00665	3,12046E-05	0,149804473	0,159	0,032	0,658448012	1,109419103	0,901372617	4,96875
LTA4H	0,000705559	0,149745177	0,159	0,046	1	1,109373506	0,901409664	3,456521739
DRG2	0,000290452	0,149567018	0,183	0,053	1	1,109236518	0,901520987	3,452830189
CFAP20	2,2086E-05	0,149396633	0,573	0,249	0,466036878	1,109105523	0,901627464	2,301204819
CHMP6	1,0926E-05	0,149389822	0,22	0,053	0,230548859	1,109100286	0,901631721	4,150943396
FMNL1	0,006076786	0,149368023	0,061	0,011	1	1,109083528	0,901645345	5,545454545
MED8	2,63838E-07	0,149304996	0,22	0,039	0,00556724	1,109035077	0,901684736	5,641025641
BMP8B	1,72676E-05	0,149249429	0,415	0,154	0,364363962	1,108992362	0,901719466	2,694805195
TIMM23	9,11343E-09	0,149233707	0,195	0,021	0,000192303	1,108980277	0,901729292	9,285714286
SLC25A24	1,912E-06	0,149194211	0,22	0,046	0,040345126	1,108949917	0,901753979	4,782608696
FAT1	1,46962E-07	0,149179019	0,415	0,119	0,003101049	1,108938239	0,901763475	3,487394958
IL17RA	7,4007E-05	0,149070531	0,134	0,025	1	1,108854852	0,901831288	5,36

Supplementary Table 2

Differentially expressed genes_keloid-XL_ comparing SC-Keloid vs. (SC-Myel, SC-Nonmyel, SC-Nonmyel2)								
**gene**	**p_val**	**avg_log2FC**	**pct.1**	**pct.2**	**p_val_adj**	**Foldchange_UP**	**Foldchange_DOWN**	**Ratio_pct1_pct2**
PCDHGA10	0,000284208	0,149030537	0,085	0,011	1	1,108824113	0,901856289	7,727272727
DNAJC17	7,14685E-07	0,148963369	0,183	0,028	0,015080561	1,108772491	0,901898278	6,535714286
SCAMP3	5,85622E-06	0,148819669	0,305	0,091	0,123572035	1,108662057	0,901988116	3,351648352
TMEM184B	0,000359592	0,148777439	0,329	0,133	1	1,108629605	0,902014519	2,473684211
PABIR2	4,9418E-05	0,148679777	0,146	0,028	1	1,10855456	0,902075582	5,214285714
MKNK1	1,55729E-05	0,14858204	0,207	0,049	0,328603271	1,108479462	0,902136696	4,224489796
VCP	1,22724E-05	0,148475522	0,524	0,211	0,258959273	1,108397623	0,902203306	2,483412322
RETREG2	4,49189E-05	0,148452138	0,317	0,109	0,947833764	1,108379658	0,90221793	2,908256881
RBMX2	1,66436E-05	0,14827628	0,207	0,049	0,351196556	1,108244559	0,902327913	4,224489796
MTFMT	3,53748E-05	0,148271977	0,159	0,032	0,746444206	1,108241254	0,902330604	4,96875
SLC2A8	1,22606E-05	0,148037383	0,268	0,077	0,25871057	1,108061059	0,902477343	3,480519481
DNAJC1	4,95818E-06	0,147915954	0,427	0,154	0,104622475	1,1079678	0,902553306	2,772727273
TPCN2	0,00046173	0,147657673	0,11	0,021	1	1,107769462	0,902714901	5,238095238
MRGBP	5,79791E-06	0,147635494	0,207	0,046	0,122341755	1,107752432	0,902728779	4,5
TBC1D16	0,001545113	0,147436968	0,183	0,063	1	1,107600007	0,90285301	2,904761905
RILPL1	5,94946E-06	0,147411362	0,28	0,077	0,125539597	1,107580348	0,902869035	3,636363636
REXO4	1,86648E-06	0,147372297	0,183	0,032	0,039384609	1,107550359	0,902893482	5,71875
ITGA11	0,000344207	0,147341932	0,073	0,007	1	1,107527048	0,902912486	10,42857143
RPL39	0,176132979	0,147182579	0,963	0,919	1	1,107404722	0,903012223	1,047878128
ATP6V0A1	0,000167444	0,147116085	0,244	0,081	1	1,107353683	0,903053844	3,012345679
CNPPD1	6,99759E-08	0,147109558	0,329	0,077	0,001476561	1,107348673	0,90305793	4,272727273
PPM1M	9,76523E-07	0,146911776	0,171	0,025	0,020605606	1,107196875	0,90318174	6,84
PABPC1L	0,000316068	0,146658191	0,146	0,035	1	1,107002278	0,903340508	4,171428571
ARID3B	4,18695E-06	0,146628843	0,159	0,025	0,088348819	1,106979759	0,903358884	6,36
LYPLA1	1,0229E-06	0,146608217	0,232	0,049	0,021584237	1,106963933	0,9033718	4,734693878
LNPK	3,15192E-06	0,146539233	0,232	0,053	0,06650873	1,106911004	0,903414996	4,377358491
ZNF346	0,000204598	0,14651093	0,098	0,014	1	1,106889289	0,903432719	7
FBXW4	0,046537539	0,146411118	0,122	0,056	1	1,106812711	0,903495226	2,178571429
MRPS35	3,53006E-11	0,146352207	0,28	0,035	7,44877E-07	1,106767517	0,903532119	8
PLAGL1	2,13269E-07	0,14629833	0,11	0,004	0,004500182	1,106726186	0,903565862	27,5

Supplementary Table 2

Differentially expressed genes_keloid-XL_ comparing SC-Keloid vs. (SC-Myel, SC-Nonmyel, SC-Nonmyel2)								
**gene**	**p_val**	**avg_log2FC**	**pct.1**	**pct.2**	**p_val_adj**	**Foldchange_UP**	**Foldchange_DOWN**	**Ratio_pct1_pct2**
DCUN1D5	2,66021E-06	0,146292223	0,244	0,056	0,056133048	1,106721501	0,903569687	4,357142857
NUDT5	1,39884E-06	0,146265585	0,317	0,088	0,029516935	1,106701066	0,903586371	3,602272727
C1orf159	0,097164846	0,146131412	0,085	0,039	1	1,106598146	0,903670409	2,179487179
DIRAS3	5,55398E-09	0,146053854	0,134	0,004	0,000117194	1,106538658	0,903718992	33,5
CTSO	8,72937E-07	0,146026708	0,207	0,039	0,018419843	1,106517837	0,903735996	5,307692308
ETV5	1,02292E-05	0,145989817	0,22	0,053	0,215846465	1,106489543	0,903759106	4,150943396
NCBP2	6,07784E-07	0,145951408	0,354	0,102	0,012824844	1,106460085	0,903783167	3,470588235
RAD51C	1,69089E-07	0,145907095	0,195	0,028	0,003567937	1,1064261	0,903810927	6,964285714
PLBD2	6,04672E-06	0,145724092	0,183	0,035	0,127591781	1,106285762	0,903925581	5,228571429
ADSL	2,16855E-06	0,145693171	0,183	0,032	0,045758632	1,10626205	0,903944956	5,71875
TEDC1	2,21914E-08	0,145640944	0,22	0,032	0,00046826	1,106222004	0,90397768	6,875
ARRB2	0,012780645	0,145567961	0,085	0,025	1	1,106166043	0,904023411	3,4
GALK1	3,21999E-05	0,145499944	0,183	0,042	0,679450306	1,106113894	0,904066033	4,357142857
HAUS1	0,000132233	0,145495287	0,146	0,032	1	1,106110323	0,904068951	4,5625
BASP1	0,304816249	0,145421001	0,061	0,035	1	1,10605337	0,904115504	1,742857143
SERP2	1,93327E-06	0,145393646	0,122	0,011	0,040793846	1,106032398	0,904132647	11,09090909
RBM23	0,000172682	0,145286847	0,317	0,119	1	1,105950524	0,904199581	2,663865546
BCL2L13	2,08677E-06	0,145177045	0,207	0,042	0,044033036	1,105866355	0,904268401	4,928571429
DERL1	3,7347E-06	0,14511337	0,28	0,077	0,078805884	1,105817548	0,904308312	3,636363636
SNX16	0,001777061	0,145056008	0,134	0,039	1	1,105773581	0,904344269	3,435897436
MED6	5,40733E-06	0,145017209	0,268	0,074	0,114100115	1,105743843	0,90436859	3,621621622
SIVA1	0,000158074	0,144945615	0,549	0,253	1	1,105688972	0,90441347	2,169960474
HTRA1	9,54858E-06	0,144829612	0,585	0,246	0,201484516	1,10560007	0,904486195	2,37804878
DXO	4,06927E-05	0,144530614	0,195	0,049	0,858655708	1,105370958	0,904673669	3,979591837
MRPL14	1,70731E-07	0,144419794	0,476	0,154	0,003602598	1,105286053	0,904743163	3,090909091
ERFE	4,32324E-06	0,144418899	0,073	0	0,09122464	1,105285367	0,904743725	#DIV/0!
PRPS2	1,01061E-06	0,14431953	0,232	0,049	0,021324863	1,105209241	0,904806043	4,734693878
TUBGCP3	3,3707E-06	0,144280128	0,171	0,028	0,071125139	1,105179057	0,904830755	6,107142857
CCDC22	4,30939E-06	0,144278831	0,159	0,025	0,09093244	1,105178063	0,904831569	6,36
PRKAG1	1,11655E-08	0,144140023	0,195	0,021	0,000235604	1,105071734	0,90491863	9,285714286

Supplementary Table 2

Differentially expressed genes_keloid-XL_ comparing SC-Keloid vs. (SC-Myel, SC-Nonmyel, SC-Nonmyel2)								
**gene**	**p_val**	**avg_log2FC**	**pct.1**	**pct.2**	**p_val_adj**	**Foldchange_UP**	**Foldchange_DOWN**	**Ratio_pct1_pct2**
BABAM1	3,72895E-06	0,144099408	0,341	0,105	0,07868457	1,105040624	0,904944106	3,247619048
ISYNA1	1,75178E-06	0,143979194	0,244	0,056	0,036964228	1,10494855	0,905019514	4,357142857
ANKIB1	2,2405E-06	0,143938179	0,329	0,095	0,047276696	1,104917137	0,905045244	3,463157895
CASP3	7,19892E-09	0,143820404	0,317	0,063	0,000151904	1,10482694	0,905119131	5,031746032
AASDHPPT	5,96711E-05	0,143814001	0,244	0,074	1	1,104822037	0,905123148	3,297297297
RBCK1	4,72109E-07	0,143756032	0,463	0,154	0,009961982	1,104777645	0,905159518	3,006493506
RUFY3	0,02014484	0,143704776	0,207	0,102	1	1,104738395	0,905191676	2,029411765
ARHGAP23	6,64427E-05	0,143692587	0,28	0,095	1	1,104729061	0,905199324	2,947368421
GOLGA8B	0,009645779	0,143680927	0,134	0,049	1	1,104720133	0,90520664	2,734693878
TRAM2	0,000130745	0,143560258	0,293	0,102	1	1,104627736	0,905282357	2,87254902
FAF2	0,000267571	0,143516052	0,146	0,035	1	1,10459389	0,905310096	4,171428571
GSS	4,01294E-08	0,143425408	0,207	0,028	0,00084677	1,104524491	0,905366978	7,392857143
COA8	1,08166E-07	0,143376062	0,366	0,098	0,002282419	1,104486712	0,905397945	3,734693878
ZNF263	0,004566226	0,143327637	0,11	0,032	1	1,10444964	0,905428336	3,4375
SUSD4	0,000983548	0,14319995	0,085	0,014	1	1,104351894	0,905508475	6,071428571
METTL27	6,67422E-07	0,143131114	0,085	0	0,014083281	1,104299203	0,905551681	#DIV/0!
CCM2	4,85913E-05	0,143089227	0,329	0,116	1	1,104267141	0,905577974	2,836206897
RPL18	0,043644107	0,14306101	0,976	0,874	1	1,104245544	0,905595685	1,116704805
BSDC1	0,000380385	0,1430551	0,28	0,109	1	1,10424102	0,905599395	2,568807339
POFUT1	0,001182686	0,142853201	0,146	0,042	1	1,104086497	0,905726139	3,476190476
SSU72	2,73649E-06	0,14279025	0,512	0,189	0,057742693	1,104038322	0,90576566	2,708994709
BRMS1	3,60614E-08	0,142747328	0,329	0,077	0,000760932	1,104005476	0,905792609	4,272727273
LRRC61	1,59595E-06	0,142746617	0,146	0,018	0,033676041	1,104004931	0,905793055	8,111111111
CYHR1	0,000605906	0,142738468	0,354	0,151	1	1,103998696	0,905798171	2,344370861
AARD	4,32324E-06	0,142703171	0,073	0	0,09122464	1,103971686	0,905820333	#DIV/0!
MAPKAPK5	7,3023E-05	0,142593494	0,183	0,046	1	1,103887763	0,905889198	3,97826087
LRRC75A	1,18512E-05	0,142475604	0,28	0,084	0,250071219	1,103797562	0,905963226	3,333333333
DDRGK1	2,52402E-06	0,142452312	0,354	0,109	0,053259401	1,103779741	0,905977853	3,247706422
WWC1	1,02952E-07	0,142420208	0,098	0	0,002172399	1,103755179	0,905998014	#DIV/0!
RAMP1	1,80697E-08	0,142334234	0,244	0,039	0,00038129	1,103689406	0,906052006	6,256410256

Supplementary Table 2

Differentially expressed genes_keloid-XL_ comparing SC-Keloid vs. (SC-Myel, SC-Nonmyel, SC-Nonmyel2)								
**gene**	**p_val**	**avg_log2FC**	**pct.1**	**pct.2**	**p_val_adj**	**Foldchange_UP**	**Foldchange_DOWN**	**Ratio_pct1_pct2**
PUS7L	1,09579E-05	0,14232457	0,195	0,042	0,231222427	1,103682013	0,906058075	4,642857143
RAE1	4,34375E-05	0,142264002	0,146	0,028	0,916575713	1,103635679	0,906096114	5,214285714
SMIM29	4,0422E-06	0,141986822	0,341	0,105	0,085294427	1,103423661	0,906270216	3,247619048
MCUR1	2,13049E-05	0,141984755	0,354	0,123	0,449554349	1,10342208	0,906271515	2,87804878
SCO1	1,02863E-05	0,141947657	0,11	0,011	0,217050943	1,103393706	0,90629482	10
EIF2B3	3,73206E-06	0,141931219	0,195	0,039	0,078750233	1,103381135	0,906305145	5
PEX2	5,50109E-07	0,141834765	0,305	0,077	0,011607854	1,103307368	0,90636574	3,961038961
DHRSX	7,61186E-11	0,141794248	0,22	0,018	1,60618E-06	1,103276383	0,906391196	12,22222222
ESYT1	4,91433E-07	0,14174702	0,232	0,046	0,010369724	1,103240267	0,906420867	5,043478261
PPID	6,1337E-06	0,141651862	0,146	0,021	0,129427162	1,103167501	0,906480656	6,952380952
MDK	2,19759E-05	0,141597183	0,463	0,186	0,463713113	1,103125692	0,906515012	2,489247312
FAM104B	0,000491694	0,14159376	0,183	0,056	1	1,103123075	0,906517163	3,267857143
PHKG1	5,61434E-05	0,141511226	0,171	0,039	1	1,103059968	0,906569025	4,384615385
FAM50A	3,81697E-06	0,141475865	0,402	0,14	0,080541829	1,103032932	0,906591246	2,871428571
OAZ1	0,080810863	0,141411007	0,951	0,786	1	1,102983345	0,906632003	1,209923664
HOXB4	7,59943E-06	0,141369536	0,085	0,004	0,160355666	1,10295164	0,906658065	21,25
PSKH1	2,1468E-05	0,141258651	0,134	0,021	0,452995664	1,102866871	0,906727753	6,380952381
RAB8A	1,2913E-08	0,141227321	0,293	0,056	0,000272478	1,102842921	0,906747444	5,232142857
HNRNPUL2	0,000114968	0,14122507	0,354	0,137	1	1,1028412	0,906748859	2,583941606
ICAM2	0,016071749	0,141063367	0,061	0,014	1	1,102717597	0,906850497	4,357142857
CAPZA2	8,45131E-06	0,141032861	0,561	0,235	0,178331074	1,10269428	0,906869672	2,387234043
C15orf61	2,5872E-07	0,140996388	0,28	0,063	0,005459252	1,102666403	0,906892599	4,444444444
MRPL28	5,47239E-06	0,140975394	0,317	0,095	0,115473007	1,102650357	0,906905797	3,336842105
ZFYVE27	0,000110887	0,14091582	0,171	0,042	1	1,102604825	0,906943247	4,071428571
TMEM101	7,90708E-07	0,140783435	0,183	0,028	0,016684724	1,102503652	0,907026474	6,535714286
ECHDC1	0,000336448	0,140763239	0,305	0,119	1	1,102488219	0,907039171	2,56302521
BCAT2	3,3465E-05	0,140612449	0,207	0,053	0,706144696	1,102372993	0,907133979	3,905660377
RPS27	0,125047335	0,140480163	0,988	0,94	1	1,102271917	0,907217162	1,05106383
STX18	0,000745181	0,140377502	0,171	0,053	1	1,102193483	0,907281721	3,226415094
RPS28	0,063518306	0,140349235	0,976	0,909	1	1,102171888	0,907299497	1,073707371

Supplementary Table 2

Differentially expressed genes_keloid-XL_ comparing SC-Keloid vs. (SC-Myel, SC-Nonmyel, SC-Nonmyel2)								
**gene**	**p_val**	**avg_log2FC**	**pct.1**	**pct.2**	**p_val_adj**	**Foldchange_UP**	**Foldchange_DOWN**	**Ratio_pct1_pct2**
KIAA0753	8,29253E-06	0,140288753	0,085	0,004	0,174980583	1,102125683	0,907337535	21,25
TMEM41B	3,57942E-05	0,140242264	0,317	0,109	0,755292466	1,102090168	0,907366773	2,908256881
IMPACT	7,28732E-05	0,140160667	0,256	0,081	1	1,102027837	0,907418094	3,160493827
PPM1G	1,5112E-06	0,140029329	0,402	0,13	0,031887762	1,101927517	0,907500706	3,092307692
NARF	7,31286E-06	0,139987544	0,305	0,091	0,15430866	1,101895602	0,907526991	3,351648352
ANPEP	1,10381E-05	0,139862061	0,098	0,007	0,232915863	1,101799766	0,907605929	14
GAMT	1,86156E-06	0,139843451	0,268	0,067	0,039280779	1,101785553	0,907617637	4
C22orf39	3,19473E-07	0,139814343	0,244	0,049	0,006741204	1,101763323	0,90763595	4,979591837
PJA1	0,000505741	0,139730132	0,268	0,102	1	1,101699015	0,90768893	2,62745098
SMC6	2,17282E-06	0,139589669	0,244	0,056	0,04584871	1,101591756	0,907777309	4,357142857
TFAM	1,05306E-05	0,139414272	0,28	0,081	0,22220625	1,101457838	0,907887679	3,456790123
TMEM127	4,14509E-08	0,139407313	0,195	0,025	0,000874655	1,101452525	0,907892059	7,8
ATMIN	2,62381E-08	0,139405784	0,256	0,046	0,00055365	1,101451357	0,907893021	5,565217391
SCP2	2,87412E-06	0,139223899	0,585	0,232	0,060646839	1,101312503	0,908007489	2,521551724
METTL26	1,83242E-05	0,13919871	0,537	0,228	0,38665924	1,101293275	0,908023342	2,355263158
PRPF4	0,000271704	0,13914175	0,122	0,025	1	1,101249794	0,908059194	4,88
CCNC	2,81634E-08	0,139054028	0,207	0,028	0,000594277	1,101182836	0,908114409	7,392857143
MRPS26	1,35748E-05	0,139047757	0,415	0,151	0,286440893	1,10117805	0,908118356	2,748344371
STOML1	1,19159E-08	0,139039831	0,183	0,018	0,000251436	1,101171999	0,908123345	10,16666667
ARMC9	2,04211E-06	0,138949945	0,122	0,011	0,043090571	1,101103394	0,908179927	11,09090909
PPP1R16A	0,034527937	0,138854796	0,207	0,109	1	1,101030776	0,908239825	1,899082569
ZNF384	1,02116E-06	0,138828284	0,207	0,039	0,021547494	1,101010543	0,908256516	5,307692308
CPSF4	0,001338917	0,13880066	0,073	0,011	1	1,100989462	0,908273907	6,636363636
CHMP3	0,000225716	0,138500184	0,585	0,281	1	1,100760178	0,908463097	2,081850534
NAF1	7,68252E-07	0,138464026	0,171	0,025	0,016210885	1,10073259	0,908485865	6,84
PRNP	0,232700802	0,138435514	0,89	0,818	1	1,100710836	0,90850382	1,08801956
NAGPA	3,04946E-07	0,13839089	0,122	0,007	0,006434672	1,100676791	0,908531921	17,42857143
PPP2R1B	0,001052504	0,138248389	0,146	0,042	1	1,100568078	0,908621665	3,476190476
ZNF579	6,09253E-07	0,138240334	0,183	0,028	0,012855847	1,100561933	0,908626739	6,535714286
SIT1	0,063203483	0,138207475	0,012	0	1	1,100536867	0,908647434	#DIV/0!

Supplementary Table 2

Differentially expressed genes_keloid-XL_ comparing SC-Keloid vs. (SC-Myel, SC-Nonmyel, SC-Nonmyel2)								
**gene**	**p_val**	**avg_log2FC**	**pct.1**	**pct.2**	**p_val_adj**	**Foldchange_UP**	**Foldchange_DOWN**	**Ratio_pct1_pct2**
TRAPPC13	3,37031E-05	0,138173049	0,183	0,042	0,711169579	1,100510606	0,908669117	4,357142857
TTC4	0,000569058	0,138169842	0,146	0,039	1	1,100508159	0,908671137	3,743589744
ATAT1	0,001074299	0,138139534	0,11	0,025	1	1,10048504	0,908690226	4,4
CCNQ	2,88302E-07	0,138122452	0,159	0,018	0,006083471	1,100472011	0,908700985	8,833333333
PHETA2	0,000201519	0,138118123	0,098	0,014	1	1,100468708	0,908703712	7
COQ9	1,07752E-08	0,137885312	0,171	0,014	0,000227368	1,100291137	0,908850363	12,21428571
ASPSCR1	2,70805E-06	0,137719694	0,207	0,042	0,057142629	1,100164834	0,908954703	4,928571429
HOMER1	0,001268404	0,137696374	0,159	0,049	1	1,100147051	0,908969396	3,244897959
ADRM1	1,37545E-05	0,137688598	0,463	0,179	0,290232906	1,100141121	0,908974295	2,586592179
MTERF3	8,93956E-06	0,137668028	0,171	0,032	0,188633563	1,100125436	0,908987255	5,34375
C1GALT1C1	6,83314E-06	0,137654534	0,207	0,046	0,144186145	1,100115145	0,908995758	4,5
TIPRL	2,26301E-06	0,137643912	0,293	0,077	0,047751839	1,100107046	0,90900245	3,805194805
DCTPP1	1,3407E-06	0,137628717	0,207	0,039	0,028290029	1,100095459	0,909012024	5,307692308
PXDC1	0,00243811	0,137592574	0,451	0,232	1	1,1000679	0,909034797	1,943965517
AC093323.1	1,21195E-05	0,137529778	0,159	0,028	0,255733306	1,100020018	0,909074366	5,678571429
SLX4IP	0,008751649	0,137421022	0,049	0,007	1	1,099937097	0,909142898	7
CDC37L1	0,003902084	0,137399765	0,171	0,063	1	1,099920891	0,909156293	2,714285714
GALNT2	0,000131491	0,137332918	0,634	0,295	1	1,099869927	0,90919842	2,149152542
TRMT5	0,000151245	0,137152721	0,11	0,018	1	1,099732559	0,909311989	6,111111111
B3GNT7	6,40456E-08	0,137058239	0,171	0,018	0,001351425	1,09966054	0,909371541	9,5
MRPL43	7,91168E-10	0,137017849	0,573	0,165	1,66944E-05	1,099629754	0,909397	3,472727273
NSA2	7,8591E-06	0,136952331	0,622	0,267	0,165834882	1,099579817	0,909438301	2,329588015
NCSTN	2,92707E-06	0,136893789	0,39	0,126	0,061764055	1,099535199	0,909475205	3,095238095
RUNX2	1,20154E-05	0,136875829	0,232	0,06	0,253537089	1,09952151	0,909486527	3,866666667
NMRAL1	2,7334E-06	0,136873896	0,171	0,028	0,057677459	1,099520037	0,909487746	6,107142857
GOLM1	2,57745E-06	0,136834368	0,207	0,042	0,054386685	1,099489913	0,909512664	4,928571429
HLTF	1,72088E-05	0,136766215	0,183	0,039	0,363123284	1,099437974	0,909555631	4,692307692
APOBEC3G	2,68848E-08	0,136482518	0,207	0,028	0,000567297	1,099221797	0,909734507	7,392857143
AGPAT4	3,00544E-06	0,136406133	0,268	0,07	0,063417838	1,0991636	0,909782675	3,828571429
ATG5	1,31687E-06	0,136100017	0,232	0,049	0,027787357	1,0989304	0,909975737	4,734693878

Supplementary Table 2

Differentially expressed genes_keloid-XL_ comparing SC-Keloid vs. (SC-Myel, SC-Nonmyel, SC-Nonmyel2)								
gene	p_val	avg_log2FC	pct.1	pct.2	p_val_adj	Foldchange_UP	Foldchange_DOWN	Ratio_pct1_pct2
MSH5	0,10804138	0,136010177	0,049	0,018	1	1,098861969	0,910032405	2,722222222
RPS6KA4	3,29598E-07	0,135940561	0,159	0,018	0,006954841	1,098808946	0,910076318	8,833333333
GOT2	0,000190779	0,135825085	0,159	0,039	1	1,098720998	0,910149166	4,076923077
ARSB	0,001013666	0,135686582	0,134	0,035	1	1,098615523	0,910236547	3,828571429
MVD	1,25745E-05	0,135652223	0,183	0,039	0,265334346	1,098589359	0,910258225	4,692307692
VTA1	0,000306151	0,135615078	0,195	0,06	1	1,098561074	0,910281662	3,25
ESYT2	5,63527E-06	0,135603247	0,488	0,182	0,118909792	1,098552065	0,910289127	2,681318681
TAB1	2,7563E-07	0,135416947	0,171	0,021	0,005816066	1,098410215	0,910406683	8,142857143
CLN6	0,001662234	0,135296085	0,098	0,021	1	1,098318199	0,910482956	4,666666667
METTL21A	6,84305E-07	0,135180772	0,183	0,028	0,01443952	1,098230415	0,910555733	6,535714286
NDOR1	0,025678378	0,135178163	0,049	0,011	1	1,098228429	0,910557379	4,454545455
BLMH	6,91886E-05	0,134981422	0,22	0,063	1	1,098078674	0,91068156	3,492063492
AARS1	1,90351E-07	0,134952466	0,317	0,077	0,004016601	1,098056634	0,910699839	4,116883117
XPOT	0,000233466	0,134847187	0,268	0,095	1	1,097976508	0,910766298	2,821052632
TRIM66	0,000344207	0,134837937	0,073	0,007	1	1,097969468	0,910772138	10,42857143
MAGED1	1,15167E-06	0,134821375	0,354	0,105	0,024301363	1,097956863	0,910782594	3,371428571
MED16	4,65134E-08	0,134791825	0,183	0,021	0,000981479	1,097934375	0,910801249	8,714285714
SIGIRR	3,71162E-05	0,134759063	0,537	0,232	0,78318991	1,097909442	0,910821933	2,314655172
RILP	3,0502E-07	0,134617222	0,207	0,035	0,006436236	1,097801505	0,910911486	5,914285714
H1-0	0,000194108	0,134565194	0,598	0,288	1	1,097761915	0,910944337	2,076388889
PHGDH	3,13577E-07	0,134498388	0,207	0,035	0,006616784	1,097711084	0,91098652	5,914285714
LFNG	7,50152E-06	0,13433582	0,134	0,018	0,158289655	1,097587396	0,911089179	7,444444444
MARVELD2	0,000183568	0,134276659	0,049	0	1	1,097542388	0,911126541	#DIV/0!
NDUFA5	5,4526E-06	0,134251001	0,476	0,179	0,115055224	1,097522868	0,911142746	2,659217877
LPCAT2	4,75231E-05	0,134179737	0,317	0,109	1	1,097468657	0,911187754	2,908256881
NTMT1	0,001774933	0,134170898	0,293	0,13	1	1,097461932	0,911193337	2,253846154
FDFT1	1,15209E-05	0,134167161	0,232	0,06	0,243101689	1,09745909	0,911195697	3,866666667
SLC36A4	0,001826896	0,134131198	0,146	0,046	1	1,097431733	0,911218411	3,173913043
ERCC8	3,16224E-05	0,134051065	0,122	0,018	0,667263526	1,097370779	0,911269025	6,777777778
CILP2	0,063203483	0,133891976	0,012	0	1	1,097249776	0,911369518	#DIV/0!

Supplementary Table 2

Differentially expressed genes_keloid-XL_ comparing SC-Keloid vs. (SC-Myel, SC-Nonmyel, SC-Nonmyel2)								
gene	p_val	avg_log2FC	pct.1	pct.2	p_val_adj	Foldchange_UP	Foldchange_DOWN	Ratio_pct1_pct2
GLB1L2	0,063203483	0,133891976	0,012	0	1	1,097249776	0,911369518	#DIV/0!
LYZ	0,063203483	0,133891976	0,012	0	1	1,097249776	0,911369518	#DIV/0!
PLCXD3	0,063203483	0,133891976	0,012	0	1	1,097249776	0,911369518	#DIV/0!
TAP2	0,000406709	0,133878576	0,232	0,081	1	1,097239585	0,911377983	2,864197531
PIGP	7,62776E-06	0,133842608	0,402	0,14	0,160953443	1,09721223	0,911400705	2,871428571
MAN2C1	0,002132794	0,133756721	0,256	0,109	1	1,097146912	0,911454965	2,348623853
HMGB3	0,013862815	0,133713735	0,146	0,06	1	1,097114223	0,911482122	2,433333333
PLCG1	0,000593207	0,133648888	0,146	0,039	1	1,09706491	0,911523093	3,743589744
SMAP1	2,02559E-05	0,13363906	0,293	0,091	0,427420187	1,097057437	0,911529302	3,21978022
ZNF853	3,61459E-07	0,133612873	0,134	0,011	0,007627136	1,097037523	0,911545848	12,18181818
SCAMP4	5,33977E-05	0,133610518	0,232	0,067	1	1,097035733	0,911547336	3,462686567
DHX29	7,67868E-05	0,133499582	0,341	0,126	1	1,096951379	0,911617433	2,706349206
DNAJC8	0,000843558	0,133323371	0,524	0,26	1	1,096817406	0,911728784	2,015384615
ASMTL	0,000166509	0,133079073	0,183	0,049	1	1,096631693	0,911883184	3,734693878
GHITM	3,68861E-05	0,133011023	0,451	0,179	0,778332658	1,096579967	0,911926198	2,519553073
RHOT2	1,35563E-05	0,132905139	0,39	0,137	0,286050813	1,096499489	0,911993129	2,846715328
COX18	0,000151245	0,132665491	0,11	0,018	1	1,096317363	0,912144635	6,111111111
ALG3	1,30411E-05	0,132584872	0,183	0,039	0,275180202	1,096256101	0,912195607	4,692307692
HSDL1	0,000983548	0,132578399	0,085	0,014	1	1,096251183	0,9121997	6,071428571
NOC4L	9,17106E-06	0,132578039	0,195	0,042	0,193518607	1,096250909	0,912199927	4,642857143
SDAD1	4,37693E-05	0,132555876	0,244	0,07	0,923575029	1,096234068	0,912213942	3,485714286
HSDL2	0,000133291	0,132393648	0,171	0,042	1	1,096110806	0,912316524	4,071428571
ATP2B1-AS1	0,000200163	0,132271834	0,195	0,056	1	1,09601826	0,912393558	3,482142857
RNF123	0,040095269	0,132191683	0,073	0,025	1	1,095957371	0,912444249	2,92
EDEM3	0,000433156	0,131989658	0,207	0,067	1	1,095803911	0,91257203	3,089552239
CXADR	1,39592E-06	0,131929379	0,098	0,004	0,029455268	1,095758127	0,91261016	24,5
GMPR2	4,88116E-05	0,131854357	0,195	0,049	1	1,095701148	0,912657618	3,979591837
NAGLU	3,28023E-06	0,131683301	0,232	0,053	0,069216199	1,095571241	0,912765836	4,377358491
ACAD10	1,87147E-06	0,131582059	0,11	0,007	0,039489991	1,095494362	0,912829892	15,71428571
APIP	1,28715E-05	0,131548713	0,22	0,053	0,271600545	1,095469041	0,912850992	4,150943396

Supplementary Table 2

Differentially expressed genes_keloid-XL_ comparing SC-Keloid vs. (SC-Myel, SC-Nonmyel, SC-Nonmyel2)								
**gene**	**p_val**	**avg_log2FC**	**pct.1**	**pct.2**	**p_val_adj**	**Foldchange_UP**	**Foldchange_DOWN**	**Ratio_pct1_pct2**
LARP1B	0,001280533	0,131520096	0,293	0,126	1	1,095447312	0,912869098	2,325396825
EMC1	9,74291E-07	0,131484715	0,244	0,053	0,02055852	1,095420447	0,912891486	4,603773585
MALSU1	1,11633E-06	0,131418753	0,341	0,098	0,023555745	1,095370364	0,912933226	3,479591837
RB1	7,54588E-05	0,131416982	0,293	0,102	1	1,095369019	0,912934347	2,87254902
DNALI1	4,43815E-05	0,13135687	0,11	0,014	0,936495011	1,09532338	0,912972386	7,857142857
NRSN2	0,001182686	0,131340431	0,146	0,042	1	1,0953109	0,912982789	3,476190476
EIF4G2	0,024824017	0,131276473	0,793	0,589	1	1,095262343	0,913023265	1,346349745
SCRN2	1,02065E-05	0,13081346	0,195	0,042	0,215368013	1,09491089	0,913316334	4,642857143
SPATA20	0,00036744	0,13080301	0,195	0,06	1	1,094902959	0,913322949	3,25
DDX52	1,25521E-06	0,130792394	0,256	0,06	0,026486091	1,094894902	0,91332967	4,266666667
SYAP1	4,69788E-05	0,130714373	0,39	0,151	0,99130034	1,094835692	0,913379064	2,582781457
LYPLAL1	1,02116E-06	0,130669338	0,207	0,039	0,021547494	1,094801517	0,913407576	5,307692308
FBXO32	0,014152931	0,130662781	0,122	0,046	1	1,09479654	0,913411728	2,652173913
COG6	8,70878E-06	0,130491617	0,171	0,032	0,183763963	1,09466666	0,913520103	5,34375
LARP1	5,26E-05	0,130440742	0,5	0,207	1	1,094628058	0,913552318	2,415458937
PIGQ	5,16791E-07	0,130440368	0,232	0,046	0,010904805	1,094627775	0,913552555	5,043478261
MRPS27	0,000444781	0,130404367	0,171	0,049	1	1,094600459	0,913575352	3,489795918
AP1G2	0,008401736	0,130386372	0,024	0	1	1,094586806	0,913586748	#DIV/0!
RABEPK	3,18768E-07	0,130374011	0,159	0,018	0,006726334	1,094577428	0,913594575	8,833333333
CARD8	4,37189E-06	0,130350663	0,159	0,025	0,092251246	1,094559713	0,913609361	6,36
CHST1	2,98807E-07	0,130142228	0,122	0,007	0,006305119	1,094401588	0,913741365	17,42857143
SPRED2	0,003835983	0,130117845	0,159	0,056	1	1,094383091	0,913756808	2,839285714
JAM3	1,43301E-05	0,129999297	0,61	0,256	0,30237855	1,094293168	0,913831896	2,3828125
WASHC2A	0,014512383	0,129953573	0,171	0,074	1	1,094258487	0,913860858	2,310810811
KLHL42	0,000100509	0,129949701	0,317	0,116	1	1,094255549	0,913863311	2,732758621
OGG1	1,06184E-06	0,129891623	0,207	0,039	0,022405895	1,0942115	0,913900101	5,307692308
APBB3	1,94649E-06	0,129723555	0,134	0,014	0,041072798	1,094084036	0,914006573	9,571428571
U2SURP	4,81362E-06	0,129691929	0,5	0,193	0,10157209	1,094060052	0,914026609	2,590673575
MGAT5	0,131674021	0,129650363	0,085	0,042	1	1,094028531	0,914052944	2,023809524
OAF	0,001001916	0,129543095	0,39	0,182	1	1,093947191	0,914120909	2,142857143

Supplementary Table 2

Differentially expressed genes_keloid-XL_ comparing SC-Keloid vs. (SC-Myel, SC-Nonmyel, SC-Nonmyel2)								
gene	p_val	avg_log2FC	pct.1	pct.2	p_val_adj	Foldchange_UP	Foldchange_DOWN	Ratio_pct1_pct2
CPXM1	0,005768901	0,129434381	0,134	0,046	1	1,093864759	0,914189795	2,913043478
RBM27	0,019027132	0,129432991	0,28	0,147	1	1,093863706	0,914190675	1,904761905
ATP6V0D1	2,72241E-07	0,129307381	0,439	0,133	0,005744549	1,093768471	0,914270274	3,30075188
MED31	0,000558164	0,129227251	0,195	0,063	1	1,093707723	0,914321056	3,095238095
POLR3E	1,51036E-05	0,129219642	0,22	0,056	0,318701911	1,093701954	0,914325878	3,928571429
USP25	0,082545931	0,129137749	0,122	0,063	1	1,093639874	0,91437778	1,936507937
SNAP29	1,40587E-06	0,129101761	0,195	0,035	0,02966526	1,093612593	0,91440059	5,571428571
AP4M1	2,51728E-06	0,129097114	0,183	0,032	0,053117118	1,093609071	0,914403535	5,71875
MBD4	0,000220788	0,129013179	0,22	0,07	1	1,093545447	0,914456736	3,142857143
C1QTNF3	5,23216E-05	0,1290029	0,098	0,011	1	1,093537655	0,914463251	8,909090909
RETREG3	5,68695E-05	0,128953066	0,207	0,056	1	1,093499883	0,914494839	3,696428571
PSMG1	7,2721E-09	0,128800246	0,207	0,025	0,000153449	1,093384058	0,914591714	8,28
TMEM120B	1,88116E-06	0,128774713	0,134	0,014	0,039694305	1,093364708	0,914607901	9,571428571
LEO1	2,28962E-06	0,128694893	0,183	0,032	0,048313355	1,093304217	0,914658505	5,71875
ARHGAP18	1,11851E-06	0,128650906	0,207	0,039	0,023601757	1,093270883	0,914686392	5,307692308
NLRP1	0,000167584	0,128644294	0,232	0,074	1	1,093265872	0,914690585	3,135135135
RNF215	0,000719024	0,128600555	0,122	0,028	1	1,093232728	0,914718316	4,357142857
CRIPT	0,001650073	0,128572638	0,268	0,112	1	1,093211573	0,914736017	2,392857143
NAPG	4,22631E-06	0,12852886	0,22	0,049	0,089179439	1,093178401	0,914763775	4,489795918
DTX3L	0,003470627	0,128520986	0,146	0,049	1	1,093172434	0,914768767	2,979591837
SLC35C1	8,2597E-10	0,128227875	0,146	0,004	1,74288E-05	1,092950358	0,914954639	36,5
GOLGA7B	1,93327E-06	0,12817178	0,122	0,011	0,040793846	1,092907863	0,914990215	11,09090909
LAGE3	1,44627E-06	0,128140635	0,354	0,105	0,030517746	1,092884269	0,915009968	3,371428571
EXD2	2,2307E-07	0,128126216	0,11	0,004	0,004706999	1,092873347	0,915019113	27,5
FRG1	4,89835E-07	0,128033374	0,415	0,13	0,010336005	1,092803019	0,915077999	3,192307692
STYXL1	3,51943E-06	0,127997511	0,159	0,025	0,074263512	1,092775854	0,915100747	6,36
NGRN	0,00014153	0,127966456	0,622	0,291	1	1,092752332	0,915120445	2,137457045
ARSK	0,000119644	0,127885222	0,171	0,042	1	1,092690803	0,915171975	4,071428571
TBL2	1,42819E-06	0,127726629	0,159	0,021	0,030136228	1,092570692	0,915272583	7,571428571
EXTL1	4,9862E-05	0,127605395	0,195	0,049	1	1,092478884	0,9153495	3,979591837

Supplementary Table 2

Differentially expressed genes_keloid-XL_ comparing SC-Keloid vs. (SC-Myel, SC-Nonmyel, SC-Nonmyel2)								
gene	p_val	avg_log2FC	pct.1	pct.2	p_val_adj	Foldchange_UP	Foldchange_DOWN	Ratio_pct1_pct2
HAUS4	0,00100048	0,127549125	0,195	0,067	1	1,092436275	0,915385202	2,910447761
CRKL	6,0016E-06	0,127545856	0,329	0,102	0,126639684	1,092433799	0,915387276	3,225490196
CCDC6	0,000719084	0,127533653	0,207	0,07	1	1,092424559	0,915395019	2,957142857
NACA	0,06414866	0,127523013	0,976	0,888	1	1,092416502	0,91540177	1,099099099
RNF208	0,015603254	0,127495678	0,098	0,032	1	1,092395804	0,915419115	3,0625
PLEKHO2	1,01121E-05	0,127434211	0,256	0,07	0,213375452	1,092349263	0,915458117	3,657142857
RAB20	0,000220667	0,127418677	0,098	0,014	1	1,092337502	0,915467974	7
RGMB	8,1517E-05	0,12720836	0,549	0,242	1	1,092178271	0,915601442	2,268595041
BCKDK	4,00328E-08	0,127105635	0,244	0,042	0,000844731	1,092100507	0,915666638	5,80952381
PNKD	3,69522E-05	0,127102274	0,402	0,154	0,779729001	1,092097963	0,915668772	2,61038961
ALG5	6,43106E-05	0,127005733	0,293	0,098	1	1,092024885	0,915730048	2,989795918
DHRS4	1,56328E-05	0,126931897	0,183	0,039	0,329867241	1,091968998	0,915776915	4,692307692
FANCI	0,065143806	0,126874475	0,024	0,004	1	1,091925536	0,915813366	6
SEPHS1	0,111382367	0,126778035	0,098	0,049	1	1,091852546	0,915874587	2
S100A3	3,24105E-07	0,126734753	0,122	0,007	0,00683894	1,09181979	0,915902065	17,42857143
SHQ1	0,00590611	0,126701855	0,061	0,011	1	1,091794894	0,91592295	5,545454545
TPM1	8,21019E-07	0,126406435	0,305	0,081	0,017324322	1,09157135	0,916110523	3,765432099
SYNCRIP	0,001174271	0,12635034	0,5	0,249	1	1,091528909	0,916146143	2,008032129
FGFR4	0,011128779	0,126304061	0,037	0,004	1	1,091493895	0,916175532	9,25
TECPR2	0,060753558	0,126280155	0,098	0,042	1	1,091475809	0,916190713	2,333333333
ITGA6	0,003028132	0,126212109	0,744	0,46	1	1,091424329	0,916233928	1,617391304
FIP1L1	0,000298895	0,126212107	0,268	0,098	1	1,091424328	0,916233929	2,734693878
PPP1R37	1,30173E-06	0,126158616	0,159	0,021	0,027467822	1,091383862	0,916267901	7,571428571
TMEM128	1,01173E-05	0,126155751	0,22	0,053	0,213486071	1,091381695	0,91626972	4,150943396
TTC33	0,002780459	0,126155443	0,159	0,053	1	1,091381462	0,916269916	3
FBXO17	2,41758E-05	0,126073186	0,268	0,081	0,510133599	1,091319237	0,91632216	3,308641975
PPP5C	5,67761E-08	0,125953174	0,232	0,039	0,001198033	1,091228458	0,916398388	5,948717949
DPY19L1	1,22144E-05	0,125896721	0,256	0,07	0,257736615	1,091185759	0,916434247	3,657142857
RPS6KC1	0,000174748	0,125771318	0,159	0,039	1	1,091090914	0,91651391	4,076923077
PWP2	0,001220269	0,125692926	0,037	0	1	1,091031629	0,916563712	#DIV/0!

Supplementary Table 2

Differentially expressed genes_keloid-XL_ comparing SC-Keloid vs. (SC-Myel, SC-Nonmyel, SC-Nonmyel2)								
gene	p_val	avg_log2FC	pct.1	pct.2	p_val_adj	Foldchange_UP	Foldchange_DOWN	Ratio_pct1_pct2
CAAP1	5,48545E-08	0,125571446	0,171	0,018	0,001157484	1,090939764	0,916640894	9,5
DHRS12	6,36887E-05	0,125562128	0,171	0,039	1	1,090932718	0,916646814	4,384615385
DNAJC21	0,002391845	0,125457872	0,39	0,189	1	1,090853885	0,916713057	2,063492063
RBFA	0,002952442	0,125360744	0,22	0,088	1	1,090780447	0,916774776	2,5
ISY1	2,27685E-06	0,125197222	0,207	0,042	0,048043722	1,090656819	0,916878694	4,928571429
RPL8	0,05578835	0,125162157	0,915	0,811	1	1,090630311	0,916900979	1,128236745
KIN	0,001300227	0,125030969	0,171	0,056	1	1,090531142	0,916984359	3,053571429
TMEM41A	0,00237203	0,125026819	0,11	0,028	1	1,090528005	0,916986997	3,928571429
RPL14	0,282383378	0,125011722	0,939	0,881	1	1,090516593	0,916996592	1,065834279
TMEM53	1,58349E-08	0,124931698	0,11	0	0,000334132	1,090456105	0,917047459	#DIV/0!
PRLR	0,00621443	0,124852215	0,183	0,074	1	1,09039603	0,917097983	2,472972973
THOC5	4,72873E-05	0,124747414	0,11	0,014	0,997809544	1,090316824	0,917164606	7,857142857
ECD	6,52415E-08	0,124744893	0,232	0,039	0,001376661	1,090314919	0,917166209	5,948717949
CMC4	1,63503E-05	0,124617236	0,317	0,102	0,345007624	1,090218447	0,917247367	3,107843137
RAPGEF3	0,00590611	0,124516305	0,061	0,011	1	1,090142177	0,91731154	5,545454545
VSTM4	1,02952E-07	0,124367173	0,098	0	0,002172399	1,090029495	0,917406368	#DIV/0!
CYFIP1	0,001460731	0,124243846	0,268	0,112	1	1,089936319	0,917484795	2,392857143
SECISBP2	7,71811E-06	0,124208622	0,354	0,116	0,162859933	1,089909708	0,917507196	3,051724138
UCHL3	5,00285E-08	0,124174256	0,305	0,067	0,001055652	1,089883746	0,917529052	4,552238806
AP4B1	0,038685095	0,124152228	0,073	0,025	1	1,089867105	0,917543061	2,92
UTP4	5,83288E-06	0,12406372	0,134	0,018	0,123079618	1,089800245	0,917599354	7,444444444
PRMT1	0,001172696	0,124031655	0,561	0,288	1	1,089776023	0,917619748	1,947916667
RNF126	0,005984451	0,124026095	0,341	0,175	1	1,089771823	0,917623285	1,948571429
BRI3	0,000113745	0,123890583	0,646	0,305	1	1,089669467	0,91770948	2,118032787
IFI44L	2,62336E-07	0,123675252	0,268	0,06	0,005535545	1,089506838	0,917846465	4,466666667
TRIM24	0,000266511	0,123614451	0,256	0,091	1	1,089460923	0,917885147	2,813186813
FANCG	0,001602574	0,123610233	0,098	0,021	1	1,089457738	0,917887831	4,666666667
BRD3	0,00041513	0,123529682	0,402	0,182	1	1,089396911	0,917939081	2,208791209
AL441883.1	0,001772311	0,12350292	0,061	0,007	1	1,089376703	0,917956109	8,714285714
MAP4K4	0,000958553	0,123466632	0,622	0,33	1	1,089349303	0,917979199	1,884848485

Supplementary Table 2

Differentially expressed genes_keloid-XL_ comparing SC-Keloid vs. (SC-Myel, SC-Nonmyel, SC-Nonmyel2)								
gene	p_val	avg_log2FC	pct.1	pct.2	p_val_adj	Foldchange_UP	Foldchange_DOWN	Ratio_pct1_pct2
ALG1	8,91037E-09	0,123432183	0,146	0,007	0,000188018	1,089323292	0,918001118	20,85714286
RPL17	0,170560756	0,123395173	0,902	0,867	1	1,089295347	0,918024669	1,040369089
OXTR	4,32324E-06	0,123306755	0,073	0	0,09122464	1,089228589	0,918080933	#DIV/0!
DNAJC14	3,30843E-05	0,123306743	0,122	0,018	0,698111591	1,08922858	0,918080941	6,777777778
DPAGT1	7,53788E-06	0,123276928	0,171	0,032	0,159056749	1,08920607	0,918099914	5,34375
NUDT22	1,59263E-06	0,12318764	0,22	0,046	0,033605986	1,089138662	0,918156737	4,782608696
ITGB3	2,80682E-05	0,123146525	0,061	0	0,592266086	1,089107623	0,918182904	#DIV/0!
LEPROTL1	2,56035E-07	0,122918697	0,427	0,13	0,005402594	1,088935646	0,918327913	3,284615385
LMLN	3,67491E-05	0,122698209	0,122	0,018	0,77544318	1,088769237	0,918468272	6,777777778
TRIP4	6,74467E-05	0,122659479	0,134	0,025	1	1,088740009	0,918492929	5,36
LRRC40	3,0557E-07	0,122550406	0,146	0,014	0,006447828	1,088657699	0,918562374	10,42857143
RCC2	1,741E-05	0,12251975	0,5	0,2	0,367367805	1,088634566	0,918581893	2,5
PTGES2	6,11409E-06	0,12236037	0,232	0,056	0,129013334	1,088514307	0,918683378	4,142857143
MOXD1	3,58198E-05	0,122338612	0,159	0,032	0,755833467	1,088497891	0,918697232	4,96875
YES1	0,000138689	0,122268532	0,207	0,06	1	1,088445017	0,91874186	3,45
SEC13	7,54963E-08	0,122260558	0,378	0,102	0,001593048	1,088439001	0,918746938	3,705882353
ANO8	0,002094809	0,122259326	0,146	0,046	1	1,088438072	0,918747723	3,173913043
UTP11	9,42612E-06	0,122231935	0,366	0,123	0,198900636	1,088417407	0,918765166	2,975609756
ZPR1	6,84492E-05	0,122224625	0,183	0,046	1	1,088411892	0,918769822	3,97826087
DPP9	0,002535781	0,122126759	0,183	0,067	1	1,088338061	0,918832149	2,731343284
RNASEH2C	1,7212E-05	0,122107719	0,39	0,144	0,363190693	1,088323699	0,918844275	2,708333333
NKAPD1	6,73808E-06	0,12206928	0,268	0,074	0,142180123	1,088294702	0,918868757	3,621621622
NTAQ1	0,000338108	0,122010174	0,073	0,007	1	1,088250116	0,918906403	10,42857143
POMT2	2,16342E-05	0,121834409	0,22	0,056	0,456502346	1,088117541	0,919018361	3,928571429
IBTK	3,41158E-05	0,121807021	0,232	0,063	0,719876608	1,088096885	0,919035808	3,682539683
DCSTAMP	1,02952E-07	0,121664086	0,098	0	0,002172399	1,087989087	0,919126866	#DIV/0!
TPP1	0,000941213	0,121492932	0,317	0,133	1	1,087860021	0,919235913	2,383458647
CSTF1	4,80414E-05	0,121484006	0,11	0,014	1	1,08785329	0,919241601	7,857142857
RCE1	2,1468E-05	0,121435471	0,134	0,021	0,452995664	1,087816694	0,919272526	6,380952381
VPS26C	0,001245481	0,121422241	0,159	0,049	1	1,087806718	0,919280956	3,244897959

Supplementary Table 2

Differentially expressed genes_keloid-XL_ comparing SC-Keloid vs. (SC-Myel, SC-Nonmyel, SC-Nonmyel2)								
gene	p_val	avg_log2FC	pct.1	pct.2	p_val_adj	Foldchange_UP	Foldchange_DOWN	Ratio_pct1_pct2
ORAI2	3,64556E-07	0,121229018	0,146	0,014	0,007692497	1,087661036	0,919404086	10,42857143
PLEKHJ1	1,42388E-07	0,121190659	0,305	0,07	0,003004531	1,087632117	0,919428531	4,357142857
FUCA1	2,80682E-05	0,121162941	0,061	0	0,592266086	1,087611221	0,919446196	#DIV/0!
TMEM222	1,37664E-05	0,121128572	0,232	0,06	0,290485377	1,087585312	0,9194681	3,866666667
PPIL3	0,001201942	0,121069719	0,183	0,06	1	1,087540946	0,91950561	3,05
EPB41L3	2,33385E-05	0,121054015	0,402	0,147	0,492466511	1,087529107	0,919515619	2,734693878
SNX4	0,00045166	0,121053788	0,22	0,074	1	1,087528936	0,919515763	2,972972973
TSTD3	0,009726554	0,121004428	0,122	0,042	1	1,087491729	0,919547224	2,904761905
WDR81	0,00140181	0,120999922	0,073	0,011	1	1,087488332	0,919550096	6,636363636
POC1A	2,80682E-05	0,120975517	0,061	0	0,592266086	1,087469936	0,919565651	#DIV/0!
SAMD5	0,000199303	0,12097257	0,159	0,039	1	1,087467715	0,919567529	4,076923077
SLC7A5	0,003970716	0,120953061	0,098	0,025	1	1,087453009	0,919579965	3,92
IL4R	7,01879E-05	0,120901627	0,134	0,025	1	1,087414241	0,919612749	5,36
ZBTB14	6,13057E-05	0,120811614	0,085	0,007	1	1,087346397	0,919670128	12,14285714
MIEF1	1,8347E-06	0,120754372	0,11	0,007	0,038713925	1,087303255	0,919706619	15,71428571
FNDC4	0,00019003	0,12063536	0,134	0,028	1	1,087213564	0,919782491	4,785714286
MRS2	0,000105335	0,120601095	0,122	0,021	1	1,087187742	0,919804337	5,80952381
TIMM9	1,19155E-05	0,120545879	0,293	0,088	0,251429383	1,087146133	0,919839541	3,329545455
PRPF6	4,10982E-05	0,120544886	0,317	0,109	0,867213402	1,087145385	0,919840174	2,908256881
SLC30A5	6,21382E-06	0,120508801	0,256	0,067	0,131117733	1,087118193	0,919863182	3,820895522
TBC1D2B	0,003546515	0,120484225	0,134	0,042	1	1,087099675	0,919878851	3,19047619
PTK7	4,65444E-05	0,120415443	0,11	0,014	0,9821336	1,087047847	0,919922709	7,857142857
TMEM209	5,20222E-05	0,120405439	0,146	0,028	1	1,087040309	0,919929088	5,214285714
ARHGEF18	0,040202307	0,120398834	0,085	0,032	1	1,087035332	0,9199333	2,65625
CIB2	3,33807E-07	0,120291231	0,146	0,014	0,00704367	1,086954259	0,920001915	10,42857143
SLC3A1	0,008401736	0,12014902	0,024	0	1	1,08684712	0,920092607	#DIV/0!
OPN3	0,006222372	0,120140515	0,134	0,046	1	1,086840713	0,920098031	2,913043478
GEMIN7	3,17593E-07	0,120007748	0,122	0,007	0,006701534	1,086740699	0,920182709	17,42857143
THOC6	3,90076E-05	0,11998291	0,22	0,06	0,82309976	1,086721989	0,920198551	3,666666667
MSRB1	2,27392E-07	0,119972624	0,171	0,021	0,004798199	1,086714241	0,920205112	8,142857143

Supplementary Table 2

Differentially expressed genes_keloid-XL_ comparing SC-Keloid vs. (SC-Myel, SC-Nonmyel, SC-Nonmyel2)								
**gene**	**p_val**	**avg_log2FC**	**pct.1**	**pct.2**	**p_val_adj**	**Foldchange_UP**	**Foldchange_DOWN**	**Ratio_pct1_pct2**
KLHL9	2,32089E-06	0,119894613	0,183	0,032	0,048973121	1,086655481	0,920254872	5,71875
SLC6A6	2,11962E-05	0,11985184	0,171	0,035	0,44726133	1,086623264	0,920282155	4,885714286
ARHGAP35	0,000554509	0,119842727	0,317	0,13	1	1,086616401	0,920287969	2,438461538
MIR4458HG	0,002552281	0,119826421	0,195	0,074	1	1,086604119	0,92029837	2,635135135
ZNF286A	0,002115747	0,119694904	0,134	0,039	1	1,086505068	0,920382269	3,435897436
ADCY1	6,67422E-07	0,119690988	0,085	0	0,014083281	1,086502119	0,920384768	#DIV/0!
SRP72	5,6999E-05	0,119664265	0,439	0,179	1	1,086481994	0,920401816	2,452513966
PCDH1	0,000411811	0,119661013	0,341	0,14	1	1,086479545	0,92040389	2,435714286
ZSWIM1	0,063203483	0,119553145	0,012	0	1	1,086398314	0,92047271	#DIV/0!
ARHGEF33	0,063203483	0,119512516	0,012	0	1	1,086367719	0,920498633	#DIV/0!
SNX2	7,58018E-07	0,119503327	0,268	0,063	0,015994934	1,0863608	0,920504496	4,253968254
EEF1AKMT1	0,005916634	0,119481622	0,134	0,046	1	1,086344456	0,920518345	2,913043478
PENK	0,000183568	0,119449402	0,049	0	1	1,086320194	0,920538903	#DIV/0!
PCGF1	0,000190779	0,119424325	0,159	0,039	1	1,086301312	0,920554904	4,076923077
PRDX3	1,15564E-05	0,11941109	0,427	0,154	0,243850634	1,086291346	0,920563349	2,772727273
HARS2	0,004014306	0,119403264	0,098	0,025	1	1,086285454	0,920568343	3,92
CDC42EP3	1,20119E-05	0,119384624	0,622	0,263	0,253463883	1,086271419	0,920580237	2,365019011
CCDC191	9,64031E-06	0,119352065	0,098	0,007	0,20342014	1,086246904	0,920601013	14
SMIM12	5,85172E-06	0,119294254	0,232	0,056	0,123477184	1,086203377	0,920637904	4,142857143
AKR1A1	1,09701E-05	0,119292703	0,293	0,088	0,231479565	1,08620221	0,920638893	3,329545455
ARPC5L	2,69182E-05	0,119248163	0,378	0,137	0,568000084	1,086168676	0,920667317	2,759124088
PDE6D	1,14837E-05	0,119124625	0,159	0,028	0,242318466	1,086075671	0,920746156	5,678571429
DYNC1LI2	0,022439685	0,118749472	0,659	0,432	1	1,085793289	0,920985615	1,525462963
LETMD1	7,54346E-05	0,118746592	0,22	0,063	1	1,085791121	0,920987454	3,492063492
ANAPC4	0,035965312	0,118725483	0,11	0,046	1	1,085775235	0,921000929	2,391304348
GCNT1	5,23216E-05	0,118625708	0,098	0,011	1	1,085700146	0,921064627	8,909090909
PGAP3	0,002664794	0,118621072	0,085	0,018	1	1,085696658	0,921067586	4,722222222
EGLN1	1,77675E-05	0,118615508	0,268	0,077	0,374912466	1,085692471	0,921071139	3,480519481
TRIM23	4,07113E-05	0,118610543	0,146	0,028	0,859049315	1,085688734	0,921074309	5,214285714
CSNK2A2	9,19588E-05	0,118563188	0,39	0,151	1	1,085653098	0,921104543	2,582781457

Supplementary Table 2

Differentially expressed genes_keloid-XL_ comparing SC-Keloid vs. (SC-Myel, SC-Nonmyel, SC-Nonmyel2)								
**gene**	**p_val**	**avg_log2FC**	**pct.1**	**pct.2**	**p_val_adj**	**Foldchange_UP**	**Foldchange_DOWN**	**Ratio_pct1_pct2**
SHLD1	4,23142E-05	0,11838989	0,11	0,014	0,892872791	1,085522696	0,921215193	7,857142857
HCFC1R1	0,002875634	0,118170483	0,646	0,361	1	1,085357621	0,921355303	1,789473684
FAM3A	2,49928E-07	0,118000817	0,28	0,063	0,00527372	1,085229986	0,921463665	4,444444444
DCXR	0,000103853	0,117993104	0,341	0,13	1	1,085224185	0,92146859	2,623076923
KCNA2	0,000103074	0,117979473	0,195	0,053	1	1,085213931	0,921477297	3,679245283
PEX13	7,52278E-05	0,11797573	0,159	0,035	1	1,085211116	0,921479688	4,542857143
TSSC4	5,09271E-09	0,117752257	0,378	0,088	0,000107461	1,08504303	0,921622436	4,295454545
SMURF2	3,11595E-06	0,117716414	0,415	0,14	0,065749715	1,085016073	0,921645333	2,964285714
ZNF558	0,002767295	0,117704687	0,085	0,018	1	1,085007253	0,921652825	4,722222222
FBXW5	1,38207E-06	0,117625259	0,366	0,112	0,029162994	1,084947519	0,921703568	3,267857143
SHROOM4	2,05285E-05	0,117616051	0,22	0,056	0,433171245	1,084940595	0,921709451	3,928571429
FKBP7	1,71213E-08	0,117580116	0,415	0,109	0,000361277	1,084913572	0,921732409	3,80733945
SCFD2	8,66134E-06	0,117574876	0,085	0,004	0,1827629	1,084909631	0,921735757	21,25
ZNF677	1,64028E-05	0,117481427	0,183	0,039	0,346114767	1,084839359	0,921795463	4,692307692
SPSB1	0,003961102	0,117182051	0,159	0,056	1	1,084614266	0,921986767	2,839285714
CCDC77	0,001600233	0,117140136	0,061	0,007	1	1,084582755	0,922013554	8,714285714
C2orf49	4,61419E-07	0,117096966	0,232	0,046	0,009736407	1,084550301	0,922041144	5,043478261
BMPER	4,32324E-06	0,117050114	0,073	0	0,09122464	1,08451508	0,922071088	#DIV/0!
TXNDC16	0,002397697	0,116770231	0,11	0,028	1	1,084304705	0,922249987	3,928571429
YJU2	0,000444147	0,116712881	0,11	0,021	1	1,084261602	0,922286649	5,238095238
RPS21	0,077152328	0,116501619	0,915	0,814	1	1,084102839	0,922421715	1,124078624
LAMC1	0,018821254	0,116428784	0,573	0,358	1	1,084048109	0,922468285	1,600558659
AAGAB	2,66449E-05	0,11639077	0,134	0,021	0,562233009	1,084019546	0,922492592	6,380952381
PTPRA	2,03205E-05	0,116363513	0,463	0,179	0,428783269	1,083999066	0,92251002	2,586592179
LINC01063	0,347088417	0,1163223	0,012	0,004	1	1,0839681	0,922536374	3
PCSK7	0,000224892	0,116262659	0,256	0,088	1	1,083923289	0,922574512	2,909090909
TCEAL3	4,26468E-05	0,116240515	0,463	0,189	0,899889921	1,083906652	0,922588673	2,44973545
MRPL58	2,08681E-09	0,116207939	0,195	0,018	4,40337E-05	1,083882178	0,922609505	10,83333333
DPYSL2	0,003527906	0,116177719	0,427	0,225	1	1,083859474	0,922628831	1,897777778
TMEM39A	0,003026608	0,116174853	0,183	0,067	1	1,083857321	0,922630664	2,731343284

Supplementary Table 2

Differentially expressed genes_keloid-XL_ comparing SC-Keloid vs. (SC-Myel, SC-Nonmyel, SC-Nonmyel2)								
**gene**	**p_val**	**avg_log2FC**	**pct.1**	**pct.2**	**p_val_adj**	**Foldchange_UP**	**Foldchange_DOWN**	**Ratio_pct1_pct2**
AP5M1	1,98272E-07	0,116151167	0,183	0,025	0,004183744	1,083839527	0,922645812	7,32
ARNTL2	5,55398E-09	0,116027167	0,134	0,004	0,000117194	1,083746374	0,922725117	33,5
SUPT16H	0,000166809	0,115998386	0,366	0,147	1	1,083724755	0,922743525	2,489795918
MRPS17	0,000127665	0,115990268	0,171	0,042	1	1,083718657	0,922748717	4,071428571
DNM1L	3,82735E-06	0,115972812	0,366	0,119	0,080761017	1,083705544	0,922759882	3,075630252
NECAP2	8,06384E-06	0,115938617	0,28	0,081	0,170155105	1,083679858	0,922781754	3,456790123
ATPAF2	0,000194727	0,115856092	0,134	0,028	1	1,083617871	0,922834541	4,785714286
GNA12	1,648E-08	0,115851301	0,39	0,098	0,000347744	1,083614273	0,922837605	3,979591837
ZNF599	0,00059839	0,115845901	0,098	0,018	1	1,083610217	0,922841059	5,444444444
TMUB1	6,21769E-05	0,115782362	0,256	0,081	1	1,083562493	0,922881704	3,160493827
PRRC2A	0,03492659	0,115775674	0,415	0,246	1	1,083557471	0,922885982	1,68699187
TRMT6	2,51588E-05	0,115769328	0,134	0,021	0,530876209	1,083552704	0,922890041	6,380952381
EDC3	0,001011915	0,115620815	0,085	0,014	1	1,083441168	0,92298505	6,071428571
DYNC1I1	0,513508152	0,115599886	0,024	0,014	1	1,08342545	0,922998439	1,714285714
TUBG1	0,000557329	0,115567698	0,146	0,039	1	1,083401278	0,923019033	3,743589744
ANAPC2	0,013301771	0,115508283	0,085	0,025	1	1,083356661	0,923057046	3,4
ERI1	9,30589E-05	0,11546882	0,122	0,021	1	1,083327027	0,923082296	5,80952381
TRIB3	0,010425992	0,115457856	0,073	0,018	1	1,083318794	0,923089311	4,055555556
HELZ2	0,010031179	0,11538871	0,134	0,049	1	1,083266874	0,923133555	2,734693878
PIGO	0,000210889	0,115264752	0,098	0,014	1	1,083173803	0,923212874	7
CDKAL1	5,20222E-05	0,115263649	0,146	0,028	1	1,083172975	0,92321358	5,214285714
TEAD3	3,14105E-05	0,115207098	0,207	0,053	0,66279258	1,083130517	0,923249769	3,905660377
ADGRE5	2,24366E-05	0,1151996	0,366	0,13	0,473434236	1,083124888	0,923254568	2,815384615
WDR18	4,15926E-07	0,115177591	0,28	0,067	0,00877646	1,083108364	0,923268652	4,179104478
MAP3K6	0,001474456	0,115142145	0,122	0,032	1	1,083081753	0,923291337	3,8125
MDH1	0,000107854	0,115044317	0,5	0,214	1	1,083008313	0,923353946	2,336448598
SOX18	0,065834577	0,115022425	0,061	0,021	1	1,082991879	0,923367958	2,904761905
RACK1	0,035150541	0,114961016	0,927	0,811	1	1,082945782	0,923407262	1,143033292
MRI1	0,000164012	0,114911445	0,134	0,028	1	1,082908572	0,923438991	4,785714286
KNL1	0,001220269	0,114892056	0,037	0	1	1,082894019	0,923451402	#DIV/0!

Supplementary Table 2

Differentially expressed genes_keloid-XL_ comparing SC-Keloid vs. (SC-Myel, SC-Nonmyel, SC-Nonmyel2)								
gene	p_val	avg_log2FC	pct.1	pct.2	p_val_adj	Foldchange_UP	Foldchange_DOWN	Ratio_pct1_pct2
ELAVL4	6,67422E-07	0,114881508	0,085	0	0,014083281	1,082886102	0,923458153	#DIV/0!
BET1	1,01629E-07	0,114880475	0,22	0,035	0,002144469	1,082885327	0,923458815	6,285714286
NUDT8	0,000942376	0,114829751	0,085	0,014	1	1,082847253	0,923491284	6,071428571
ORMDL3	1,07355E-05	0,114682109	0,256	0,07	0,226529633	1,082736444	0,923585796	3,657142857
EYA3	0,000685144	0,114588463	0,098	0,018	1	1,082666165	0,923645748	5,444444444
RNASET2	0,016071749	0,114564177	0,061	0,014	1	1,082647939	0,923661297	4,357142857
PKD1	0,000236006	0,114546765	0,378	0,154	1	1,082634873	0,923672445	2,454545455
CAMK1	4,33882E-06	0,114475995	0,195	0,039	0,091553477	1,082581767	0,923717756	5
TMEM181	0,000184612	0,114403703	0,159	0,039	1	1,082527521	0,923764044	4,076923077
TRMT61B	0,00067597	0,114388507	0,098	0,018	1	1,082516119	0,923773773	5,444444444
SCPEP1	9,32723E-05	0,114346068	0,28	0,095	1	1,082484275	0,923800948	2,947368421
PRDM1	0,510048238	0,114343433	0,024	0,014	1	1,082482298	0,923802635	1,714285714
TMUB2	5,7449E-05	0,114337216	0,171	0,039	1	1,082477634	0,923806616	4,384615385
APEX2	0,000186767	0,114298639	0,098	0,014	1	1,082448689	0,923831319	7
SEPTIN6	0,008751649	0,114288192	0,049	0,007	1	1,082440851	0,923838008	7
MRPL46	4,63364E-05	0,114236289	0,146	0,028	0,977743891	1,082401909	0,923871245	5,214285714
ARMC5	0,002631406	0,11409975	0,085	0,018	1	1,082299474	0,923958686	4,722222222
PPA2	6,99475E-06	0,113996838	0,207	0,046	0,147596116	1,082222273	0,924024598	4,5
TMEM185A	0,000129749	0,113936792	0,207	0,06	1	1,082177231	0,924063057	3,45
ZNF469	8,66134E-06	0,113907744	0,085	0,004	0,1827629	1,082155442	0,924081663	21,25
CIAO2A	2,02929E-05	0,113825404	0,207	0,049	0,42819948	1,082093681	0,924134405	4,224489796
CSDE1	0,025663028	0,113769898	0,72	0,442	1	1,082052049	0,924169961	1,628959276
CACNA1C	5,32686E-05	0,113712413	0,073	0,004	1	1,082008935	0,924206785	18,25
PSMD3	2,2595E-06	0,113667315	0,402	0,133	0,047677729	1,081975113	0,924235676	3,022556391
ZBTB8A	3,19993E-05	0,113620394	0,159	0,032	0,675218046	1,081939924	0,924265736	4,96875
SLC30A9	6,3387E-07	0,113536529	0,256	0,056	0,013375287	1,081877032	0,924319466	4,571428571
SCOC	4,15086E-07	0,113473129	0,537	0,189	0,008758723	1,081829489	0,924360086	2,841269841
TMEM203	2,95204E-06	0,113314457	0,207	0,042	0,062291075	1,081710513	0,924461756	4,928571429
SACM1L	6,2277E-05	0,113203944	0,207	0,056	1	1,081627655	0,924532574	3,696428571
NACC2	6,88723E-06	0,113184999	0,256	0,067	0,145327362	1,081613451	0,924544715	3,820895522

Supplementary Table 2

Differentially expressed genes_keloid-XL_ comparing SC-Keloid vs. (SC-Myel, SC-Nonmyel, SC-Nonmyel2)								
**gene**	**p_val**	**avg_log2FC**	**pct.1**	**pct.2**	**p_val_adj**	**Foldchange_UP**	**Foldchange_DOWN**	**Ratio_pct1_pct2**
EXT2	1,70226E-06	0,113171719	0,293	0,077	0,035919458	1,081603495	0,924553226	3,805194805
RMDN2	1,49573E-06	0,113151239	0,146	0,018	0,03156145	1,081588141	0,92456635	8,111111111
ZNF525	0,011553628	0,113148962	0,037	0,004	1	1,081586434	0,924567809	9,25
ZNF880	0,006170448	0,113134788	0,134	0,046	1	1,081575808	0,924576893	2,913043478
PPP6C	3,07307E-06	0,113074467	0,305	0,084	0,064844951	1,081530587	0,924615551	3,630952381
AMACR	9,56641E-05	0,113073335	0,122	0,021	1	1,081529739	0,924616277	5,80952381
DYRK4	9,64033E-06	0,112937735	0,293	0,088	0,203420635	1,08142809	0,924703186	3,329545455
AP5Z1	9,62051E-07	0,11281244	0,171	0,025	0,020300248	1,081334173	0,924783499	6,84
RPS3A	0,08781488	0,112773781	0,976	0,884	1	1,081305199	0,924808279	1,104072398
ORC4	1,23461E-07	0,112748603	0,244	0,046	0,002605148	1,081286327	0,924824419	5,304347826
MNAT1	1,65927E-06	0,112739949	0,293	0,077	0,035012201	1,081279841	0,924829967	3,805194805
ZFPL1	6,78231E-06	0,112686345	0,183	0,035	0,143113616	1,081239667	0,92486433	5,228571429
CPD	0,002886858	0,112667259	0,207	0,081	1	1,081225363	0,924876565	2,555555556
EEF1B2	0,02317115	0,112651522	0,78	0,561	1	1,081213568	0,924886655	1,390374332
MOB3A	3,64556E-07	0,112602326	0,146	0,014	0,007692497	1,0811767	0,924918194	10,42857143
CFDP1	0,001912292	0,112472864	0,39	0,189	1	1,081079684	0,925001196	2,063492063
SLF1	2,05364E-06	0,112459087	0,183	0,032	0,043333918	1,081069359	0,925010029	5,71875
WIZ	1,54009E-05	0,112431959	0,207	0,049	0,324975096	1,081049031	0,925027423	4,224489796
ENPP4	0,011128779	0,112393058	0,037	0,004	1	1,081019882	0,925052366	9,25
IVD	7,78762E-06	0,11237548	0,256	0,067	0,164326581	1,081006711	0,925063637	3,820895522
CD2BP2	1,55636E-05	0,112273276	0,244	0,067	0,328407081	1,080930133	0,925129173	3,641791045
LRIF1	2,51588E-05	0,112221063	0,134	0,021	0,530876209	1,080891013	0,925162656	6,380952381
C12orf43	3,17593E-07	0,112168019	0,122	0,007	0,006701534	1,080851273	0,925196672	17,42857143
SCO2	7,05689E-06	0,112149252	0,171	0,032	0,148907436	1,080837212	0,925208707	5,34375
FBXO4	0,000120189	0,112143178	0,146	0,032	1	1,080832662	0,925212602	4,5625
MPND	7,7397E-06	0,112132784	0,134	0,018	0,163315378	1,080824875	0,925219268	7,444444444
TSFM	8,37326E-06	0,112042662	0,171	0,032	0,176684187	1,080757361	0,925277066	5,34375
PARD6A	0,000214102	0,111987539	0,098	0,014	1	1,080716067	0,92531242	7
TBC1D7	7,65928E-07	0,111915132	0,22	0,042	0,016161843	1,080661829	0,925358861	5,238095238
PDE4B	0,000198484	0,111845289	0,098	0,014	1	1,080609514	0,925403661	7

Supplementary Table 2

Differentially expressed genes_keloid-XL_ comparing SC-Keloid vs. (SC-Myel, SC-Nonmyel, SC-Nonmyel2)								
**gene**	**p_val**	**avg_log2FC**	**pct.1**	**pct.2**	**p_val_adj**	**Foldchange_UP**	**Foldchange_DOWN**	**Ratio_pct1_pct2**
BCAP29	0,00112445	0,111805393	0,463	0,225	1	1,080579632	0,925429252	2,057777778
ELOA	0,003560464	0,11178269	0,146	0,049	1	1,080562627	0,925443815	2,979591837
NLE1	2,18116E-07	0,111651268	0,11	0,004	0,004602468	1,080464198	0,925528122	27,5
VPS37D	0,10804138	0,111638237	0,049	0,018	1	1,080454439	0,925536482	2,722222222
CBR1	0,000130744	0,11163075	0,512	0,228	1	1,080448831	0,925541285	2,245614035
YIPF2	9,87009E-08	0,111465311	0,28	0,06	0,002082687	1,08032494	0,925647426	4,666666667
B4GALT1	6,04502E-06	0,111445004	0,341	0,109	0,127555985	1,080309733	0,925660456	3,128440367
ALG14	0,00018342	0,111408844	0,268	0,091	1	1,080282656	0,925683657	2,945054945
ELK3	1,35049E-05	0,111376828	0,463	0,175	0,284966246	1,080258684	0,925704199	2,645714286
NADSYN1	0,009407308	0,111259615	0,122	0,042	1	1,08017092	0,925779412	2,904761905
GMDS	5,77801E-06	0,111240486	0,146	0,021	0,121921893	1,080156599	0,925791687	6,952380952
INTS12	3,45006E-05	0,111172744	0,159	0,032	0,727996941	1,080105881	0,925835159	4,96875
GIN1	0,000432775	0,111091262	0,11	0,021	1	1,080044879	0,925887451	5,238095238
ZNF557	5,90574E-05	0,111084465	0,085	0,007	1	1,080039791	0,925891813	12,14285714
ALKBH6	0,000421675	0,111069215	0,11	0,021	1	1,080028374	0,9259016	5,238095238
MCM6	0,000332109	0,111031036	0,073	0,007	1	1,079999793	0,925926103	10,42857143
KRI1	0,014817086	0,110914591	0,11	0,039	1	1,079912626	0,926000841	2,820512821
ARFIP2	2,45218E-08	0,110883762	0,268	0,049	0,000517435	1,07988955	0,926020629	5,469387755
SLC39A4	0,000425319	0,11087101	0,134	0,032	1	1,079880005	0,926028814	4,1875
ZNF662	0,001914332	0,110668924	0,049	0,004	1	1,07972875	0,926158537	12,25
RHNO1	1,08469E-05	0,110651932	0,11	0,011	0,228879507	1,079716034	0,926169445	10
YARS1	0,000113219	0,11056737	0,22	0,067	1	1,079652749	0,926223734	3,28358209
CHST14	3,49528E-07	0,110553801	0,22	0,039	0,007375398	1,079642595	0,926232445	5,641025641
IQCK	1,61892E-05	0,11055214	0,146	0,025	0,341608689	1,079641352	0,926233511	5,84
POLR3GL	2,75993E-05	0,110423508	0,28	0,088	0,582372342	1,079545094	0,926316099	3,181818182
CLPTM1	0,000292146	0,110401317	0,415	0,179	1	1,079528489	0,926330347	2,318435754
ABAT	0,186442875	0,110375661	0,037	0,014	1	1,079509291	0,926346821	2,642857143
FAIM	0,005867015	0,110213294	0,134	0,046	1	1,079387806	0,926451081	2,913043478
ECH1	3,40124E-06	0,110110365	0,463	0,165	0,071769648	1,0793108	0,926517181	2,806060606
PTGER1	0,000298645	0,110073403	0,061	0,004	1	1,079283148	0,926540919	15,25

Supplementary Table 2

Differentially expressed genes_keloid-XL_ comparing SC-Keloid vs. (SC-Myel, SC-Nonmyel, SC-Nonmyel2)								
gene	p_val	avg_log2FC	pct.1	pct.2	p_val_adj	Foldchange_UP	Foldchange_DOWN	Ratio_pct1_pct2
SULF1	9,28126E-06	0,110040333	0,122	0,014	0,195843832	1,079258408	0,926562158	8,714285714
RAD1	0,009021414	0,110035559	0,122	0,042	1	1,079254837	0,926565224	2,904761905
WASHC5	0,001139464	0,110010052	0,11	0,025	1	1,079235756	0,926581606	4,4
LRRC27	1,08469E-05	0,109985959	0,11	0,011	0,228879507	1,079217733	0,92659708	10
SDK1	0,004564654	0,109931612	0,122	0,039	1	1,079177079	0,926631986	3,128205128
ADSS2	0,000787841	0,109852583	0,256	0,098	1	1,079117965	0,926682747	2,612244898
INPP5K	7,9173E-05	0,109851348	0,317	0,112	1	1,079117041	0,92668354	2,830357143
AKTIP	2,27964E-05	0,109838627	0,22	0,056	0,481026575	1,079107526	0,926691711	3,928571429
CXXC1	0,000188991	0,109827362	0,195	0,056	1	1,0790991	0,926698947	3,482142857
LSM6	6,26294E-07	0,109794002	0,354	0,098	0,013215423	1,079074148	0,926720376	3,612244898
TFAP2A-AS1	0,001713147	0,10977414	0,061	0,007	1	1,079059292	0,926733134	8,714285714
MRPL32	0,000434918	0,109735807	0,402	0,179	1	1,079030622	0,926757758	2,245810056
ZBED5	0,000448556	0,109719146	0,183	0,056	1	1,07901816	0,926768461	3,267857143
UBA5	0,000801747	0,109669229	0,171	0,053	1	1,078980827	0,926800528	3,226415094
GLRX5	0,000346043	0,109520766	0,39	0,165	1	1,078869799	0,926895906	2,363636364
EPSTI1	6,67422E-07	0,10940105	0,085	0	0,014083281	1,078780277	0,926972824	#DIV/0!
MTHFD1	0,000148936	0,10936457	0,183	0,049	1	1,078752999	0,926996265	3,734693878
POLD1	0,057650786	0,109352764	0,049	0,014	1	1,078744171	0,92700385	3,5
JMJD4	3,73031E-05	0,109229318	0,122	0,018	0,787133093	1,078651871	0,927083174	6,777777778
FIG4	3,24105E-07	0,109193183	0,122	0,007	0,00683894	1,078624854	0,927106395	17,42857143
TIMM21	0,000458752	0,109007606	0,11	0,021	1	1,078486118	0,927225658	5,238095238
LYRM2	7,60802E-09	0,108948129	0,354	0,081	0,000160537	1,078441656	0,927263885	4,37037037
OXER1	0,063203483	0,108926143	0,012	0	1	1,078425222	0,927278016	#DIV/0!
MRPL30	8,4837E-06	0,108889721	0,171	0,032	0,179014635	1,078397996	0,927301427	5,34375
ADPRM	0,001239853	0,108867053	0,073	0,011	1	1,078381052	0,927315997	6,636363636
KANK2	0,002711112	0,108864763	0,488	0,253	1	1,07837934	0,927317469	1,928853755
SH3BP4	9,91694E-09	0,10864503	0,207	0,025	0,000209257	1,078215108	0,927458716	8,28
ULBP2	2,2307E-07	0,108633974	0,11	0,004	0,004706999	1,078206845	0,927465824	27,5
OSBPL6	0,001952737	0,108622768	0,049	0,004	1	1,078198471	0,927473028	12,25
SH3BGRL2	0,510048238	0,108611192	0,024	0,014	1	1,078189819	0,92748047	1,714285714

Supplementary Table 2

Differentially expressed genes_keloid-XL_ comparing SC-Keloid vs. (SC-Myel, SC-Nonmyel, SC-Nonmyel2)								
**gene**	**p_val**	**avg_log2FC**	**pct.1**	**pct.2**	**p_val_adj**	**Foldchange_UP**	**Foldchange_DOWN**	**Ratio_pct1_pct2**
AP001816.1	0,001126735	0,108519814	0,146	0,042	1	1,07812153	0,927539217	3,476190476
MRFAP1	0,004565065	0,108509046	0,768	0,477	1	1,078113483	0,92754614	1,610062893
ZNF195	5,13592E-05	0,108410338	0,146	0,028	1	1,078039722	0,927609604	5,214285714
FAM219B	0,000952436	0,108408885	0,171	0,053	1	1,078038637	0,927610538	3,226415094
HNRNPH2	0,000212919	0,108290194	0,402	0,165	1	1,077949949	0,927686856	2,436363636
CHMP4A	3,80998E-07	0,108270034	0,402	0,119	0,00803944	1,077934887	0,92769982	3,378151261
BDH2	0,035247036	0,108255846	0,28	0,161	1	1,077924286	0,927708943	1,739130435
NPHP1	5,69725E-05	0,108253433	0,098	0,011	1	1,077922483	0,927710495	8,909090909
RAB35	4,48757E-05	0,108203544	0,22	0,06	0,946921188	1,077885209	0,927742576	3,666666667
CYREN	3,68303E-07	0,108174411	0,134	0,011	0,007771562	1,077863442	0,927761311	12,18181818
CENPN	0,292304842	0,108111823	0,037	0,018	1	1,077816683	0,92780156	2,055555556
DACT1	5,40601E-05	0,108105985	0,146	0,028	1	1,077812322	0,927805314	5,214285714
NARS1	6,34443E-06	0,108055482	0,415	0,147	0,133873818	1,077774592	0,927837794	2,823129252
RTTN	2,93176E-06	0,108054893	0,171	0,028	0,061863053	1,077774153	0,927838172	6,107142857
XRCC1	0,00475002	0,108043441	0,11	0,032	1	1,077765597	0,927845538	3,4375
ABCC10	0,006556321	0,107898233	0,085	0,021	1	1,077657125	0,927938931	4,047619048
HID1	0,05889129	0,107734102	0,049	0,014	1	1,077534531	0,928044505	3,5
SEMA6D	1,71092E-05	0,107711467	0,268	0,077	0,361021405	1,077517624	0,928059066	3,480519481
STK36	0,179467859	0,107693801	0,049	0,021	1	1,07750443	0,92807043	2,333333333
CAMKK2	1,46543E-05	0,107609525	0,232	0,06	0,309220926	1,077441489	0,928124646	3,866666667
PAICS	6,60988E-06	0,107573143	0,256	0,067	0,139475168	1,077414318	0,928148051	3,820895522
TCTA	1,7849E-06	0,107457585	0,195	0,035	0,037663249	1,077328022	0,928222398	5,571428571
ZBTB22	8,76126E-05	0,107432986	0,159	0,035	1	1,077309653	0,928238225	4,542857143
MRPS12	5,15784E-12	0,107400334	0,293	0,035	1,08836E-07	1,077285271	0,928259233	8,371428571
ECSCR	0,020887559	0,107395376	0,073	0,021	1	1,077281569	0,928262424	3,476190476
ZFAND6	0,000292546	0,107321333	0,5	0,228	1	1,077226281	0,928310066	2,192982456
POLM	9,30589E-05	0,107310031	0,122	0,021	1	1,077217842	0,928317338	5,80952381
CISD3	4,87689E-10	0,107190009	0,305	0,049	1,02907E-05	1,07712823	0,92839457	6,224489796
UVRAG	0,002505089	0,107103327	0,195	0,074	1	1,077063514	0,928450353	2,635135135
YIPF1	1,42457E-06	0,107084929	0,146	0,018	0,030059903	1,077049778	0,928462194	8,111111111

Supplementary Table 2

Differentially expressed genes_keloid-XL_ comparing SC-Keloid vs. (SC-Myel, SC-Nonmyel, SC-Nonmyel2)								
**gene**	**p_val**	**avg_log2FC**	**pct.1**	**pct.2**	**p_val_adj**	**Foldchange_UP**	**Foldchange_DOWN**	**Ratio_pct1_pct2**
DCAF17	0,000467734	0,107049345	0,11	0,021	1	1,077023214	0,928485094	5,238095238
MICAL2	1,44656E-09	0,106968451	0,159	0,007	3,05239E-05	1,076962825	0,928537158	22,71428571
COMMD2	0,001799836	0,106965433	0,232	0,091	1	1,076960572	0,9285391	2,549450549
CHFR	1,43675E-05	0,106913475	0,183	0,039	0,303168791	1,076921787	0,928572541	4,692307692
SDR39U1	0,000517403	0,106897815	0,232	0,081	1	1,076910097	0,928582621	2,864197531
POP5	0,000114365	0,106821458	0,293	0,102	1	1,076853101	0,928631769	2,87254902
YKT6	8,02218E-06	0,106786811	0,293	0,084	0,169276077	1,07682724	0,928654071	3,488095238
MAPK3	4,1086E-07	0,106724979	0,293	0,07	0,008669559	1,07678109	0,928693873	4,185714286
FKBP2	0,000170208	0,106717808	0,28	0,098	1	1,076775737	0,928698489	2,857142857
PYROXD1	3,40712E-05	0,106715325	0,159	0,032	0,718936484	1,076773885	0,928700087	4,96875
CCDC71	1,06202E-05	0,106601929	0,098	0,007	0,224096034	1,076689253	0,928773086	14
ACAD8	1,52268E-06	0,106545817	0,195	0,035	0,032130029	1,076647378	0,92880921	5,571428571
CYP27C1	5,93202E-07	0,106407312	0,232	0,046	0,012517148	1,07654402	0,928898384	5,043478261
UTP20	0,000972222	0,106263789	0,134	0,035	1	1,076436928	0,928990798	3,828571429
AIP	0,000321509	0,10622722	0,463	0,207	1	1,076409642	0,929014346	2,236714976
C2orf92	0,183494953	0,106184855	0,024	0,007	1	1,076378034	0,929041627	3,428571429
HELQ	0,006340275	0,106164367	0,085	0,021	1	1,076362748	0,929054821	4,047619048
CD52	0,063203483	0,10610024	0,012	0	1	1,076314906	0,929096117	#DIV/0!
FAM110D	0,063203483	0,10610024	0,012	0	1	1,076314906	0,929096117	#DIV/0!
TMEM241	0,004035611	0,106091706	0,073	0,014	1	1,076308539	0,929101613	5,214285714
SFT2D1	2,21056E-05	0,106065095	0,463	0,179	0,46644981	1,076288687	0,929118751	2,586592179
MTMR6	0,003026098	0,105972854	0,195	0,074	1	1,076219875	0,929178157	2,635135135
EIF3L	0,001686373	0,105897773	0,549	0,288	1	1,076163867	0,929226516	1,90625
POLR3H	3,14724E-05	0,105887448	0,183	0,042	0,66409926	1,076156165	0,929233166	4,357142857
NUDCD2	5,36697E-05	0,105869616	0,451	0,182	1	1,076142864	0,929244651	2,478021978
RRP7A	2,05375E-07	0,105808259	0,256	0,053	0,004333616	1,076097097	0,929284172	4,830188679
FADS2	0,001041902	0,105808229	0,171	0,053	1	1,076097074	0,929284192	3,226415094
WDR5B	5,69725E-05	0,105801171	0,098	0,011	1	1,07609181	0,929288738	8,909090909
BLVRA	7,32957E-05	0,105710022	0,22	0,063	1	1,076023825	0,929347452	3,492063492
SFI1	0,001380549	0,105680681	0,073	0,011	1	1,076001941	0,929366353	6,636363636

Supplementary Table 2

Differentially expressed genes_keloid-XL_ comparing SC-Keloid vs. (SC-Myel, SC-Nonmyel, SC-Nonmyel2)								
gene	p_val	avg_log2FC	pct.1	pct.2	p_val_adj	Foldchange_UP	Foldchange_DOWN	Ratio_pct1_pct2
GXYLT2	7,01531E-08	0,105603761	0,159	0,014	0,0014803	1,075944574	0,929415905	11,35714286
FAM50B	6,04286E-06	0,105586921	0,146	0,021	0,127510438	1,075932015	0,929426754	6,952380952
CCDC32	0,0001121	0,105573457	0,171	0,042	1	1,075921974	0,929435428	4,071428571
IL31RA	0,001220269	0,105548763	0,037	0	1	1,075903558	0,929451337	#DIV/0!
ZNF446	8,47501E-06	0,105523433	0,085	0,004	0,178831285	1,075884668	0,929467656	21,25
CALML5	0,347088417	0,105466828	0,012	0,004	1	1,075842456	0,929504125	3
CHKB	1,07595E-06	0,105466464	0,28	0,07	0,02270366	1,075842185	0,929504359	4
PHC3	0,002866349	0,105412289	0,207	0,081	1	1,075801786	0,929539264	2,555555556
BTBD6	3,80729E-05	0,105349689	0,305	0,102	0,803375267	1,075755107	0,929579598	2,990196078
FAM120A	0,001411781	0,105277316	0,366	0,165	1	1,075701143	0,929626232	2,218181818
CDK1	0,510048238	0,105169714	0,024	0,014	1	1,075620916	0,92969557	1,714285714
SLC35A2	0,009572322	0,105090808	0,183	0,077	1	1,075562087	0,92974642	2,376623377
EIF2B2	1,75178E-06	0,104955213	0,244	0,056	0,036964228	1,075461003	0,929833808	4,357142857
PTCD3	0,001847472	0,104798707	0,207	0,077	1	1,075344342	0,929934683	2,688311688
ASF1A	0,00055155	0,104722031	0,146	0,039	1	1,075287191	0,929984109	3,743589744
MRPL10	3,95199E-06	0,104589316	0,159	0,025	0,083391026	1,075188279	0,930069662	6,36
MINPP1	5,60121E-05	0,104414153	0,098	0,011	1	1,075057744	0,930182593	8,909090909
TYMS	0,000314706	0,104270953	0,073	0,007	1	1,074951041	0,930274926	10,42857143
RNF182	0,065143806	0,104246125	0,024	0,004	1	1,074932542	0,930290935	6
SUCO	0,002635829	0,104234487	0,183	0,067	1	1,07492387	0,930298441	2,731343284
CBX1	1,44332E-05	0,104149205	0,293	0,088	0,304555555	1,07486033	0,930353435	3,329545455
COIL	9,66747E-06	0,104034624	0,171	0,032	0,203993199	1,074774967	0,930427327	5,34375
AMBRA1	0,014921301	0,10390492	0,098	0,032	1	1,074678345	0,93051098	3,0625
GPATCH3	0,000275125	0,103859186	0,085	0,011	1	1,074644277	0,930540479	7,727272727
PPP2R5C	0,000900049	0,103848365	0,317	0,133	1	1,074636217	0,930547458	2,383458647
PHAF1	0,000114351	0,103757469	0,122	0,021	1	1,074568512	0,930606088	5,80952381
NOL12	4,72753E-05	0,103667266	0,195	0,049	0,997556556	1,074501328	0,930664276	3,979591837
FANCE	0,001359586	0,103504478	0,073	0,011	1	1,074380093	0,930769294	6,636363636
ATF6B	5,94173E-05	0,103355512	0,268	0,088	1	1,074269163	0,930865406	3,045454545
CMC1	2,21943E-06	0,103341307	0,329	0,095	0,046832239	1,074258585	0,930874571	3,463157895

Supplementary Table 2

Differentially expressed genes_keloid-XL_ comparing SC-Keloid vs. (SC-Myel, SC-Nonmyel, SC-Nonmyel2)								
gene	p_val	avg_log2FC	pct.1	pct.2	p_val_adj	Foldchange_UP	Foldchange_DOWN	Ratio_pct1_pct2
TOE1	5,32686E-05	0,103207023	0,073	0,004	1	1,0741586	0,93096122	18,25
PCED1A	0,002231776	0,103183837	0,146	0,046	1	1,074141337	0,930976182	3,173913043
MRPL45	2,8218E-07	0,103076163	0,22	0,039	0,005954283	1,074061172	0,931045667	5,641025641
RPS27A	0,150848743	0,103070784	0,988	0,926	1	1,074057167	0,931049138	1,066954644
MYD88	1,12366E-05	0,102993361	0,11	0,011	0,23710448	1,073999529	0,931099105	10
GMPPA	1,1086E-08	0,102936697	0,256	0,042	0,000233926	1,073957347	0,931135676	6,095238095
IDH2	4,23152E-06	0,102933882	0,195	0,039	0,089289314	1,073955252	0,931137492	5
CCNH	1,86156E-06	0,102913494	0,268	0,067	0,039280779	1,073940074	0,931150652	4
MRPL36	3,06976E-05	0,102876384	0,28	0,088	0,64775032	1,07391245	0,931174603	3,181818182
TRPM8	4,32324E-06	0,102860598	0,073	0	0,09122464	1,0739007	0,931184792	#DIV/0!
KCTD14	2,80682E-05	0,102840102	0,061	0	0,592266086	1,073885443	0,931198022	#DIV/0!
LRRC47	8,76126E-05	0,102742318	0,159	0,035	1	1,073812659	0,931261139	4,542857143
LINC00592	2,80682E-05	0,102644022	0,061	0	0,592266086	1,073739499	0,931324591	#DIV/0!
PSMD12	2,8323E-05	0,102619739	0,366	0,13	0,597643657	1,073721426	0,931340267	2,815384615
OGFR	0,000422705	0,102612705	0,329	0,133	1	1,073716191	0,931344808	2,473684211
TMLHE	3,46103E-05	0,102518945	0,122	0,018	0,730311277	1,073646414	0,931405337	6,777777778
MED25	2,57378E-05	0,102484879	0,195	0,046	0,543093082	1,073621062	0,931427331	4,239130435
EHD3	0,026685693	0,10227232	0,049	0,011	1	1,073462892	0,931564573	4,454545455
LONP2	0,012157541	0,102184475	0,22	0,102	1	1,073397532	0,931621297	2,156862745
PIGL	0,02110076	0,102165306	0,073	0,021	1	1,073383269	0,931633675	3,476190476
MPDU1	1,82813E-08	0,102158956	0,293	0,06	0,000385753	1,073378545	0,931637776	4,883333333
CCDC86	2,3412E-05	0,102108677	0,134	0,021	0,494016028	1,073341137	0,931670245	6,380952381
HUWE1	0,000294847	0,102078697	0,366	0,147	1	1,073318833	0,931689605	2,489795918
RABGGTB	4,47684E-05	0,1020578	0,439	0,168	0,944657555	1,073303286	0,931703101	2,613095238
PDS5A	1,54166E-05	0,101963654	0,268	0,077	0,325305094	1,073233248	0,931763903	3,480519481
ZNF540	0,011553628	0,101939729	0,037	0,004	1	1,07321545	0,931779355	9,25
IFT122	0,000719024	0,101865251	0,122	0,028	1	1,073160047	0,931827459	4,357142857
UBASH3B	0,000344207	0,101821882	0,073	0,007	1	1,073127787	0,931855471	10,42857143
NFYB	0,007429969	0,101782479	0,293	0,14	1	1,073098478	0,931880923	2,092857143
CTU2	5,54605E-05	0,101782461	0,146	0,028	1	1,073098465	0,931880934	5,214285714

Supplementary Table 2

Differentially expressed genes_keloid-XL_ comparing SC-Keloid vs. (SC-Myel, SC-Nonmyel, SC-Nonmyel2)								
**gene**	**p_val**	**avg_log2FC**	**pct.1**	**pct.2**	**p_val_adj**	**Foldchange_UP**	**Foldchange_DOWN**	**Ratio_pct1_pct2**
FCRLA	0,000253594	0,10174606	0,085	0,011	1	1,07307139	0,931904447	7,727272727
HACD1	0,000309097	0,10170431	0,073	0,007	1	1,073040337	0,931931415	10,42857143
CHSY1	0,001557806	0,101670457	0,183	0,063	1	1,073015158	0,931953283	2,904761905
HDHD3	0,02110076	0,101555966	0,073	0,021	1	1,072930008	0,932027245	3,476190476
TXN2	4,92227E-07	0,101487409	0,305	0,077	0,010386484	1,072879023	0,932071537	3,961038961
IDH3G	7,11085E-05	0,101431189	0,341	0,123	1	1,072837216	0,932107858	2,772357724
PIGM	0,00072733	0,101319291	0,122	0,028	1	1,072754008	0,932180158	4,357142857
NOL6	1,10401E-05	0,101208281	0,11	0,011	0,23295734	1,072671467	0,932251888	10
CLCN7	0,002755295	0,101201047	0,22	0,088	1	1,072666088	0,932256563	2,5
ABHD8	5,5067E-05	0,101059554	0,098	0,011	1	1,072560891	0,932347999	8,909090909
RHBDD2	1,90987E-06	0,10105782	0,341	0,102	0,040300207	1,072559602	0,932349119	3,343137255
NUP43	0,006251893	0,101031159	0,061	0,011	1	1,072539781	0,932366349	5,545454545
RAB11FIP3	8,33671E-07	0,100957221	0,268	0,063	0,017591299	1,072484815	0,932414134	4,253968254
PLCXD1	0,011339437	0,100922881	0,037	0,004	1	1,072459287	0,932436328	9,25
NRM	0,000322117	0,100782486	0,207	0,067	1	1,072354926	0,932527072	3,089552239
PIGB	0,001772311	0,100712099	0,061	0,007	1	1,072302609	0,93257257	8,714285714
REEP5	0,00021556	0,100406978	0,524	0,242	1	1,072075848	0,932769824	2,165289256
SMIM20	4,40464E-08	0,100136335	0,244	0,042	0,000929423	1,07187475	0,932944824	5,80952381
FPGT	0,000115923	0,100116854	0,122	0,021	1	1,071860276	0,932957422	5,80952381
MAP3K1	4,20589E-05	0,100104121	0,305	0,102	0,887485753	1,071850816	0,932965656	2,990196078
MAP9	2,60923E-05	0,100030475	0,232	0,063	0,55057388	1,071796103	0,933013283	3,682539683
FBLN7	0,009031726	0,100016544	0,049	0,007	1	1,071785753	0,933022292	7
ITGB3BP	7,76016E-06	0,099936488	0,28	0,081	0,163747111	1,071726281	0,933074067	3,456790123
BAALC	0,000915822	0,099915045	0,085	0,014	1	1,071710352	0,933087936	6,071428571
GTPBP2	0,00296515	0,099896614	0,122	0,035	1	1,07169666	0,933099857	3,485714286
PDZD8	0,039805415	0,09989588	0,244	0,13	1	1,071696115	0,933100331	1,876923077
WNT16	8,66134E-06	0,099840903	0,085	0,004	0,1827629	1,071655276	0,93313589	21,25
TTC37	7,09608E-07	0,099761227	0,378	0,112	0,014973446	1,071596093	0,933187426	3,375
ZBTB42	2,04211E-06	0,099730234	0,122	0,011	0,043090571	1,071573073	0,933207473	11,09090909
ENKD1	0,001531269	0,099596147	0,171	0,056	1	1,071473484	0,933294211	3,053571429

Supplementary Table 2

Differentially expressed genes_keloid-XL_ comparing SC-Keloid vs. (SC-Myel, SC-Nonmyel, SC-Nonmyel2)								
**gene**	**p_val**	**avg_log2FC**	**pct.1**	**pct.2**	**p_val_adj**	**Foldchange_UP**	**Foldchange_DOWN**	**Ratio_pct1_pct2**
ATP6V1H	3,09036E-05	0,099578093	0,232	0,063	0,652096183	1,071460075	0,933305891	3,682539683
PTP4A3	0,008751649	0,099566479	0,049	0,007	1	1,07145145	0,933313404	7
NCBP2AS2	0,000128817	0,099462468	0,22	0,067	1	1,071374206	0,933380694	3,28358209
EHMT1	0,002790291	0,099365238	0,195	0,074	1	1,071302004	0,933443601	2,635135135
HRAS	0,002380183	0,099310095	0,341	0,161	1	1,071261057	0,93347928	2,118012422
PCDHB2	0,347088417	0,099292373	0,012	0,004	1	1,071247898	0,933490747	3
SLC27A5	0,039238167	0,099207921	0,085	0,032	1	1,071185191	0,933545393	2,65625
CCDC74B	4,80414E-05	0,099156148	0,11	0,014	1	1,071146751	0,933578895	7,857142857
C17orf58	4,80414E-05	0,099137759	0,11	0,014	1	1,071133098	0,933590794	7,857142857
HSD17B1	0,008890703	0,099124202	0,049	0,007	1	1,071123033	0,933599568	7
TPR	0,000406003	0,099069985	0,427	0,186	1	1,07108278	0,933634653	2,295698925
CLN3	2,57871E-05	0,098980952	0,171	0,035	0,544132901	1,071016682	0,933692273	4,885714286
DCAF12	0,000579236	0,098947202	0,183	0,056	1	1,070991627	0,933714115	3,267857143
STEAP3	4,32324E-06	0,098759258	0,073	0	0,09122464	1,070852115	0,933835761	#DIV/0!
TMEM243	6,41967E-05	0,098751684	0,244	0,074	1	1,070846493	0,933840664	3,297297297
PRADC1	7,91729E-07	0,098745603	0,171	0,025	0,016706276	1,07084198	0,9338446	6,84
RGS1	0,347088417	0,09874312	0,012	0,004	1	1,070840137	0,933846207	3
IFTAP	1,62791E-05	0,098734181	0,207	0,049	0,343505831	1,070833502	0,933851993	4,224489796
MRM2	0,009163884	0,098680534	0,11	0,035	1	1,070793684	0,933886719	3,142857143
LAP3	1,70575E-05	0,098655466	0,256	0,07	0,359930495	1,070775078	0,933902946	3,657142857
CHD9	0,001155777	0,098638222	0,671	0,361	1	1,07076228	0,933914109	1,858725762
IL32	0,01375973	0,098613452	0,098	0,032	1	1,070743895	0,933930144	3,0625
ARNT	0,773305148	0,098580512	0,073	0,063	1	1,070719448	0,933951468	1,158730159
RPL21	0,093891472	0,098532733	0,976	0,835	1	1,070683989	0,933982398	1,168862275
AIFM1	4,18695E-06	0,098484419	0,159	0,025	0,088348819	1,070648134	0,934013677	6,36
SIDT2	5,61434E-05	0,098294276	0,171	0,039	1	1,070507035	0,934136785	4,384615385
SEMA4D	1,45516E-05	0,098289639	0,305	0,095	0,307053931	1,070503593	0,934139788	3,210526316
SERPINB8	0,000894103	0,098255004	0,171	0,053	1	1,070477894	0,934162214	3,226415094
METRN	0,01669849	0,098178354	0,744	0,449	1	1,070421021	0,934211847	1,65701559
VASH1	4,08921E-07	0,098178093	0,268	0,06	0,008628642	1,070420828	0,934212016	4,466666667

Supplementary Table 2

Differentially expressed genes_keloid-XL_ comparing SC-Keloid vs. (SC-Myel, SC-Nonmyel, SC-Nonmyel2)								
gene	p_val	avg_log2FC	pct.1	pct.2	p_val_adj	Foldchange_UP	Foldchange_DOWN	Ratio_pct1_pct2
VPS51	0,002375385	0,098165752	0,341	0,154	1	1,070411671	0,934220008	2,214285714
TMEM205	0,005827726	0,098160392	0,341	0,165	1	1,070407695	0,934223478	2,066666667
SLC5A6	0,006341138	0,098075998	0,061	0,011	1	1,07034508	0,93427813	5,545454545
IDE	0,000162451	0,098040482	0,11	0,018	1	1,070318731	0,93430113	6,111111111
DMAC2	1,51927E-07	0,098038521	0,244	0,046	0,00320582	1,070317276	0,9343024	5,304347826
PAGR1	5,58393E-08	0,097974986	0,268	0,053	0,001178264	1,070270141	0,934343547	5,056603774
TMEM18	0,000162836	0,097959632	0,317	0,119	1	1,070258751	0,934353491	2,663865546
DNAAF10	0,023331282	0,097958115	0,098	0,035	1	1,070257626	0,934354473	2,8
MAPKAPK3	1,36536E-06	0,097798912	0,098	0,004	0,028810488	1,070139528	0,934457586	24,5
CDK5R1	1,10381E-05	0,097755833	0,098	0,007	0,232915863	1,070107574	0,934485489	14
NIPSNAP2	8,77377E-06	0,09773508	0,341	0,109	0,185135409	1,070092181	0,934498932	3,128440367
SMU1	0,000183673	0,097699452	0,305	0,112	1	1,070065755	0,93452201	2,723214286
MRPL35	4,67659E-06	0,097684073	0,195	0,039	0,098680786	1,070054348	0,934531972	5
EPDR1	0,000963552	0,097673867	0,268	0,105	1	1,070046779	0,934538583	2,552380952
SIRT6	0,003174574	0,097654926	0,122	0,035	1	1,07003273	0,934550853	3,485714286
ADTRP	0,065143806	0,097622098	0,024	0,004	1	1,070008382	0,934572119	6
ZNF287	8,47501E-06	0,097604583	0,085	0,004	0,178831285	1,069995391	0,934583465	21,25
UBE2D2	0,001099284	0,097566652	0,659	0,354	1	1,06996726	0,934608037	1,861581921
HOGA1	0,000183568	0,097534135	0,049	0	1	1,069943144	0,934629102	#DIV/0!
MMAB	3,12046E-05	0,097527023	0,159	0,032	0,658448012	1,069937869	0,93463371	4,96875
VCPKMT	0,005577099	0,09752378	0,134	0,046	1	1,069935465	0,93463581	2,913043478
CARD10	0,006062183	0,097489184	0,085	0,021	1	1,069909808	0,934658223	4,047619048
CPEB1	0,060153875	0,097468153	0,049	0,014	1	1,069894211	0,934671849	3,5
GPALPP1	2,74168E-06	0,097439549	0,207	0,042	0,057852276	1,069872999	0,93469038	4,928571429
XYLT2	0,000752789	0,097367841	0,122	0,028	1	1,069819823	0,934736839	4,357142857
EDC4	4,0245E-06	0,097339347	0,195	0,039	0,084920902	1,069798694	0,934755301	5
MOSPD1	3,62029E-05	0,097316653	0,122	0,018	0,763918221	1,069781866	0,934770005	6,777777778
ZNF605	6,62286E-08	0,097303566	0,146	0,011	0,001397491	1,069772161	0,934778485	13,27272727
PLPP6	0,000311292	0,097214845	0,061	0,004	1	1,069706376	0,934835973	15,25
UBXN2B	0,009031726	0,097152766	0,049	0,007	1	1,069660348	0,934876199	7

Supplementary Table 2

Differentially expressed genes_keloid-XL_ comparing SC-Keloid vs. (SC-Myel, SC-Nonmyel, SC-Nonmyel2)								
**gene**	**p_val**	**avg_log2FC**	**pct.1**	**pct.2**	**p_val_adj**	**Foldchange_UP**	**Foldchange_DOWN**	**Ratio_pct1_pct2**
C19orf54	0,001423372	0,097122719	0,073	0,011	1	1,06963807	0,93489567	6,636363636
BTN3A1	5,69725E-05	0,097107645	0,098	0,011	1	1,069626894	0,934905438	8,909090909
RITA1	6,13057E-05	0,097074128	0,085	0,007	1	1,069602045	0,934927158	12,14285714
ZNF451	2,06984E-05	0,097041205	0,28	0,084	0,436756518	1,069577636	0,934948494	3,333333333
C1orf216	0,010188387	0,096899061	0,073	0,018	1	1,069472259	0,935040616	4,055555556
CCDC9B	8,02431E-05	0,096893909	0,256	0,081	1	1,06946844	0,935043955	3,160493827
VANGL1	1,39592E-06	0,096845627	0,098	0,004	0,029455268	1,069432649	0,935075248	24,5
SETMAR	0,00140181	0,096717332	0,073	0,011	1	1,069337552	0,935158406	6,636363636
MOSPD3	8,71822E-08	0,09663519	0,232	0,039	0,001839631	1,069276669	0,935211652	5,948717949
C3orf18	0,000230869	0,096469829	0,098	0,014	1	1,069154116	0,935318851	7
NAB1	8,62361E-05	0,096350843	0,232	0,07	1	1,069065941	0,935395995	3,314285714
STK31	0,347088417	0,096313548	0,012	0,004	1	1,069038306	0,935420176	3
GEMIN8	6,54879E-05	0,096287539	0,207	0,056	1	1,069019033	0,93543704	3,696428571
TFPI2	0,006269696	0,096219439	0,085	0,021	1	1,068968573	0,935481197	4,047619048
GSTA4	0,000121331	0,096108042	0,244	0,077	1	1,068886036	0,935553433	3,168831169
EFNA4	0,000649127	0,096052843	0,098	0,018	1	1,06884514	0,935589229	5,444444444
TEDC2	0,008401736	0,095910805	0,024	0	1	1,068739914	0,935681345	#DIV/0!
SPTLC1	6,96677E-05	0,095897375	0,354	0,13	1	1,068729965	0,935690055	2,723076923
TEX261	6,0173E-08	0,095779779	0,244	0,042	0,00126971	1,068642856	0,935766327	5,80952381
VPS35L	0,000153426	0,095740859	0,11	0,018	1	1,068614027	0,935791572	6,111111111
SEC24D	3,96744E-06	0,09571381	0,329	0,098	0,083716951	1,068593991	0,935809118	3,357142857
ZCCHC7	7,05328E-05	0,095658127	0,22	0,063	1	1,068552748	0,935845237	3,492063492
STK3	0,014373075	0,095557993	0,171	0,074	1	1,068478585	0,935910195	2,310810811
MRPS28	0,000395663	0,095445918	0,268	0,098	1	1,068395584	0,935982903	2,734693878
TRIM37	0,009930926	0,095434153	0,122	0,042	1	1,068386871	0,935990536	2,904761905
MNS1	0,002449793	0,095400676	0,11	0,028	1	1,06836208	0,936012255	3,928571429
CEBPZOS	4,33053E-06	0,095386747	0,305	0,088	0,091378607	1,068351766	0,936021292	3,465909091
CD164	0,000110095	0,095324272	0,646	0,302	1	1,068305502	0,936061827	2,139072848
PLRG1	3,41358E-06	0,095299602	0,232	0,053	0,072029977	1,068287234	0,936077833	4,377358491
CREB3L1	3,17593E-07	0,095293315	0,122	0,007	0,006701534	1,068282579	0,936081913	17,42857143

Supplementary Table 2

Differentially expressed genes_keloid-XL_ comparing SC-Keloid vs. (SC-Myel, SC-Nonmyel, SC-Nonmyel2)								
**gene**	**p_val**	**avg_log2FC**	**pct.1**	**pct.2**	**p_val_adj**	**Foldchange_UP**	**Foldchange_DOWN**	**Ratio_pct1_pct2**
PUSL1	3,53478E-08	0,09527563	0,122	0,004	0,000745874	1,068269484	0,936093387	30,5
ZNF136	6,246E-05	0,095246414	0,085	0,007	1	1,068247851	0,936112345	12,14285714
DOK5	0,001217477	0,095159387	0,146	0,042	1	1,068183413	0,936168815	3,476190476
ACAA2	2,35206E-06	0,094976415	0,232	0,053	0,049630756	1,068047948	0,936287553	4,377358491
MEIS3	0,163217468	0,094956426	0,061	0,028	1	1,06803315	0,936300526	2,178571429
EIF2B4	8,53692E-07	0,09493231	0,256	0,06	0,018013746	1,068015297	0,936316177	4,266666667
LAMA5	1,63503E-05	0,09490994	0,317	0,102	0,345007624	1,067998737	0,936330696	3,107843137
APCDD1L	4,32324E-06	0,094822749	0,073	0	0,09122464	1,067934193	0,936387286	#DIV/0!
PIDD1	0,000288855	0,094811112	0,085	0,011	1	1,067925579	0,936394839	7,727272727
THUMPD2	4,99665E-06	0,094759249	0,22	0,049	0,105434212	1,067887189	0,936428502	4,489795918
TMEM63A	0,009972289	0,094729128	0,122	0,042	1	1,067864893	0,936448053	2,904761905
ADAMTS10	0,644071107	0,09472358	0,012	0,007	1	1,067860787	0,936451654	1,714285714
ENOSF1	0,000470825	0,094691161	0,134	0,032	1	1,067836791	0,936472697	4,1875
ZNF138	0,001703135	0,094517076	0,098	0,021	1	1,067707946	0,936585705	4,666666667
SLC25A20	0,333178656	0,094507932	0,024	0,011	1	1,06770118	0,936591641	2,181818182
DHX34	0,011339437	0,094366121	0,037	0,004	1	1,067596234	0,936683709	9,25
WDPCP	0,01030658	0,094356043	0,073	0,018	1	1,067588776	0,936690253	4,055555556
GINS2	2,80682E-05	0,094320512	0,061	0	0,592266086	1,067562484	0,936713322	#DIV/0!
FOXM1	0,001772311	0,09431152	0,061	0,007	1	1,06755583	0,93671916	8,714285714
ZNF268	0,000969643	0,094212345	0,085	0,014	1	1,067482445	0,936783555	6,071428571
NTRK3	0,169837703	0,094205831	0,11	0,063	1	1,067477626	0,936787784	1,746031746
INPPL1	3,36272E-06	0,094078591	0,329	0,098	0,070956689	1,067383483	0,936870409	3,357142857
GALNS	0,000432069	0,094072609	0,171	0,049	1	1,067379056	0,936874294	3,489795918
RTN3	0,000147144	0,094004261	0,476	0,211	1	1,06732849	0,93691868	2,255924171
URB1	0,000169117	0,093983046	0,207	0,06	1	1,067312795	0,936932457	3,45
C11orf49	2,18601E-08	0,093889325	0,22	0,032	0,000461269	1,067243462	0,936993325	6,875
NXN	0,016071749	0,093785165	0,061	0,014	1	1,067166412	0,937060976	4,357142857
EBAG9	0,000266143	0,093764332	0,207	0,063	1	1,067151002	0,937074508	3,285714286
NME6	3,68303E-07	0,093748872	0,134	0,011	0,007771562	1,067139567	0,937084549	12,18181818
LMAN2L	0,000877287	0,093710205	0,085	0,014	1	1,067110965	0,937109666	6,071428571

Supplementary Table 2

Differentially expressed genes_keloid-XL_ comparing SC-Keloid vs. (SC-Myel, SC-Nonmyel, SC-Nonmyel2)								
**gene**	**p_val**	**avg_log2FC**	**pct.1**	**pct.2**	**p_val_adj**	**Foldchange_UP**	**Foldchange_DOWN**	**Ratio_pct1_pct2**
AP1B1	6,44461E-06	0,093701016	0,207	0,046	0,135987778	1,067104169	0,937115635	4,5
TBRG4	0,001353292	0,093675583	0,146	0,042	1	1,067085357	0,937132155	3,476190476
SPOP	1,95723E-05	0,093674264	0,317	0,102	0,412994159	1,067084382	0,937133011	3,107843137
ARF1	0,048464513	0,093658351	0,805	0,572	1	1,067072611	0,937143348	1,407342657
HLCS	0,000109756	0,093656972	0,122	0,021	1	1,067071592	0,937144244	5,80952381
AAAS	0,000335451	0,093611582	0,159	0,042	1	1,06703802	0,937173729	3,785714286
EFCAB13	0,001952737	0,093594471	0,049	0,004	1	1,067025364	0,937184844	12,25
NFKBIL1	0,000752789	0,093590232	0,122	0,028	1	1,067022229	0,937187598	4,357142857
CHST8	4,32324E-06	0,093586191	0,073	0	0,09122464	1,06701924	0,937190223	#DIV/0!
ARMC8	0,000896751	0,093581019	0,256	0,098	1	1,067015415	0,937193583	2,612244898
CRYBG1	0,004255004	0,093565786	0,073	0,014	1	1,067004149	0,937203478	5,214285714
GTPBP3	5,69725E-05	0,093545829	0,098	0,011	1	1,066989389	0,937216443	8,909090909
GABRB1	5,32686E-05	0,093538976	0,073	0,004	1	1,066984321	0,937220895	18,25
NDP	0,001703135	0,093513453	0,098	0,021	1	1,066965445	0,937237476	4,666666667
MANEA	1,38141E-06	0,093454421	0,232	0,049	0,029149134	1,066921787	0,937275826	4,734693878
USP14	7,43082E-06	0,093415442	0,415	0,144	0,156797671	1,066892961	0,93730115	2,881944444
SMAD1	3,90623E-05	0,093315774	0,183	0,042	0,824254035	1,066819258	0,937365905	4,357142857
BLCAP	0,00038684	0,093287417	0,268	0,098	1	1,06679829	0,93738433	2,734693878
ZNF154	0,011339437	0,093251975	0,037	0,004	1	1,066772082	0,937407359	9,25
EGFL8	0,08742224	0,093032588	0,902	0,688	1	1,066609873	0,937549919	1,311046512
PPIG	5,53349E-05	0,093010008	0,549	0,235	1	1,06659318	0,937564593	2,336170213
DONSON	0,002016171	0,093001428	0,134	0,039	1	1,066586837	0,937570168	3,435897436
SLC35G2	7,99531E-06	0,092999123	0,122	0,014	0,1687091	1,066585133	0,937571666	8,714285714
TRIM5	0,008346075	0,092969925	0,049	0,007	1	1,066563547	0,937590642	7
MYT1	0,000183568	0,092755209	0,049	0	1	1,066404822	0,937730193	#DIV/0!
TOLLIP	4,61669E-05	0,092717694	0,378	0,14	0,9741677	1,066377092	0,937754578	2,7
DTNA	0,00102428	0,092702743	0,134	0,035	1	1,066366041	0,937764296	3,828571429
ATP6AP1L	0,00032444	0,092696703	0,061	0,004	1	1,066361577	0,937768222	15,25
INTS4	0,009972289	0,092670161	0,122	0,042	1	1,066341959	0,937785475	2,904761905
PTPRM	0,000918685	0,092637236	0,171	0,053	1	1,066317623	0,937806877	3,226415094

Supplementary Table 2

Differentially expressed genes_keloid-XL_ comparing SC-Keloid vs. (SC-Myel, SC-Nonmyel, SC-Nonmyel2)								
**gene**	**p_val**	**avg_log2FC**	**pct.1**	**pct.2**	**p_val_adj**	**Foldchange_UP**	**Foldchange_DOWN**	**Ratio_pct1_pct2**
TVP23B	0,015183551	0,092532892	0,146	0,06	1	1,066240503	0,937874707	2,433333333
VHL	3,75113E-07	0,092515173	0,207	0,035	0,007915261	1,066227408	0,937886226	5,914285714
DNAJC9	3,03887E-08	0,092475045	0,22	0,032	0,000641233	1,066197752	0,937912313	6,875
AC004637.1	4,32324E-06	0,092443476	0,073	0	0,09122464	1,066174422	0,937932837	#DIV/0!
CDCA4	2,80682E-05	0,092427267	0,061	0	0,592266086	1,066162443	0,937943375	#DIV/0!
SLC10A7	0,016266828	0,092362991	0,061	0,014	1	1,066114943	0,937985164	4,357142857
ABHD18	0,001772311	0,092258976	0,061	0,007	1	1,066038082	0,938052793	8,714285714
TRAPPC9	2,07968E-06	0,09222943	0,122	0,011	0,043883378	1,06601625	0,938072005	11,09090909
ABCE1	0,000489294	0,092008356	0,293	0,116	1	1,065852909	0,938215762	2,525862069
PRELID3B	0,000397613	0,091937774	0,244	0,084	1	1,065800766	0,938261664	2,904761905
PILRB	0,282277357	0,091925148	0,085	0,053	1	1,065791437	0,938269876	1,603773585
MOGS	4,38829E-07	0,0919021	0,293	0,07	0,009259729	1,065774411	0,938284865	4,185714286
TRIM41	0,005234552	0,091874433	0,122	0,039	1	1,065753973	0,938302859	3,128205128
RPS8	0,239145891	0,091840365	1	0,958	1	1,065728806	0,938325017	1,043841336
ZNF781	0,000631786	0,091810707	0,098	0,018	1	1,065706898	0,938344306	5,444444444
PTRH2	7,97812E-05	0,091735729	0,159	0,035	1	1,065651513	0,938393075	4,542857143
GTF2H2C	0,000217361	0,091629372	0,098	0,014	1	1,065572956	0,938462256	7
CEP290	0,009040853	0,09154953	0,195	0,084	1	1,065513986	0,938514195	2,321428571
PHETA1	0,00032621	0,091504127	0,073	0,007	1	1,065480454	0,93854373	10,42857143
H2BC9	0,000703841	0,091475489	0,098	0,018	1	1,065459304	0,938562361	5,444444444
LINC01554	0,342070304	0,091472502	0,024	0,011	1	1,065457098	0,938564304	2,181818182
P2RY1	0,000173945	0,091396076	0,207	0,06	1	1,065400657	0,938614026	3,45
LCLAT1	4,8807E-05	0,091355768	0,11	0,014	1	1,065370891	0,938640251	7,857142857
OIP5	0,008401736	0,091203629	0,024	0	1	1,065258549	0,93873924	#DIV/0!
NIPA2	0,000203192	0,091194722	0,244	0,081	1	1,065251972	0,938745036	3,012345679
COQ10B	0,000133905	0,091145633	0,317	0,116	1	1,065215726	0,938776978	2,732758621
SGSM3	1,2511E-06	0,091102675	0,329	0,095	0,026399467	1,065184008	0,938804931	3,463157895
KIAA0895L	1,08469E-05	0,090990327	0,11	0,011	0,228879507	1,065101062	0,938878042	10
TICAM2	0,04287207	0,090937565	0,085	0,032	1	1,06506211	0,938912379	2,65625
TAPBPL	0,001538307	0,090857568	0,122	0,032	1	1,065003054	0,938964444	3,8125

Supplementary Table 2

Differentially expressed genes_keloid-XL_ comparing SC-Keloid vs. (SC-Myel, SC-Nonmyel, SC-Nonmyel2)								
**gene**	**p_val**	**avg_log2FC**	**pct.1**	**pct.2**	**p_val_adj**	**Foldchange_UP**	**Foldchange_DOWN**	**Ratio_pct1_pct2**
SLC33A1	0,00011679	0,09077887	0,207	0,06	1	1,064944961	0,939015664	3,45
BCAS2	4,50067E-06	0,090759662	0,293	0,081	0,094968624	1,064930782	0,939028166	3,617283951
VAPB	0,000121815	0,090737605	0,329	0,119	1	1,064914501	0,939042523	2,764705882
PPP4R3B	0,031343802	0,090680076	0,256	0,137	1	1,064872037	0,939079969	1,868613139
COMMD5	0,000127665	0,090656868	0,171	0,042	1	1,064854907	0,939095076	4,071428571
ALG2	0,008760492	0,090540717	0,11	0,035	1	1,06476918	0,939170685	3,142857143
RAB12	0,000238764	0,090484499	0,207	0,063	1	1,064727689	0,939207283	3,285714286
TSN	2,80628E-07	0,090417146	0,378	0,105	0,005921541	1,064677982	0,939251132	3,6
PITPNA	1,34032E-05	0,090406665	0,317	0,102	0,282821059	1,064670248	0,939257955	3,107843137
SNRK	0,037995958	0,09024946	0,073	0,025	1	1,064554241	0,939360308	2,92
CCAR2	0,000470825	0,090166246	0,134	0,032	1	1,06449284	0,939414491	4,1875
KEAP1	9,19469E-05	0,09014618	0,232	0,07	1	1,064478034	0,939427558	3,314285714
GEMIN5	1,7871E-06	0,090112762	0,134	0,014	0,03770959	1,064453378	0,939449318	9,571428571
TMEM106C	5,90121E-06	0,090046916	0,256	0,067	0,124521343	1,064404796	0,939492197	3,820895522
CROT	2,55228E-05	0,090045198	0,134	0,021	0,538555655	1,064403528	0,939493316	6,380952381
TRIP11	0,000171728	0,089974491	0,305	0,112	1	1,064351363	0,939539362	2,723214286
STAM2	0,00683044	0,089930856	0,146	0,053	1	1,064319172	0,939567779	2,754716981
ZNF468	5,32686E-05	0,089910538	0,073	0,004	1	1,064304182	0,939581011	18,25
MCAT	4,95841E-05	0,089895776	0,11	0,014	1	1,064293292	0,939590625	7,857142857
MIB2	0,04045173	0,089858676	0,159	0,077	1	1,064265924	0,939614788	2,064935065
THADA	0,001241194	0,089793105	0,146	0,042	1	1,064217553	0,939657495	3,476190476
IGFLR1	0,001220269	0,08976894	0,037	0	1	1,064199728	0,939673234	#DIV/0!
ARAF	2,3522E-05	0,089756555	0,22	0,056	0,496337612	1,064190592	0,939681301	3,928571429
CHUK	0,001571179	0,089735169	0,122	0,032	1	1,064174817	0,93969523	3,8125
RSPRY1	0,000276176	0,089642472	0,171	0,046	1	1,064106443	0,93975561	3,717391304
RUFY2	0,000107105	0,089602165	0,207	0,06	1	1,064076714	0,939781866	3,45
CCZ1	4,29376E-05	0,089560209	0,305	0,102	0,906027166	1,064045769	0,939809197	2,990196078
UBE2V2	4,10545E-05	0,089517663	0,427	0,161	0,866291261	1,064014391	0,939836913	2,652173913
NUP93	0,001861343	0,08951708	0,171	0,056	1	1,064013961	0,939837293	3,053571429
BCAT1	1,02952E-07	0,089503631	0,098	0	0,002172399	1,064004042	0,939846054	#DIV/0!

Supplementary Table 2

Differentially expressed genes_keloid-XL_ comparing SC-Keloid vs. (SC-Myel, SC-Nonmyel, SC-Nonmyel2)								
gene	p_val	avg_log2FC	pct.1	pct.2	p_val_adj	Foldchange_UP	Foldchange_DOWN	Ratio_pct1_pct2
ARCN1	2,09384E-05	0,089397643	0,415	0,154	0,441821824	1,063925877	0,939915102	2,694805195
MFSD9	0,000183568	0,089354333	0,049	0	1	1,063893938	0,93994332	#DIV/0!
EXOSC4	1,28715E-05	0,089337172	0,22	0,053	0,271600545	1,063881283	0,9399545	4,150943396
SMO	0,060153875	0,089325474	0,049	0,014	1	1,063872657	0,939962122	3,5
CYFIP2	0,000315825	0,089297935	0,122	0,025	1	1,063852349	0,939980065	4,88
GTF2H1	0,002016171	0,089211878	0,134	0,039	1	1,063788893	0,940036136	3,435897436
AIF1L	0,000576852	0,0892046	0,305	0,123	1	1,063783526	0,940040878	2,479674797
MTMR14	7,4992E-05	0,089036397	0,134	0,025	1	1,063659507	0,940150484	5,36
FKRP	0,025343006	0,089011371	0,11	0,042	1	1,063641056	0,940166792	2,619047619
NEK7	0,036903994	0,088977166	0,232	0,126	1	1,063615839	0,940189083	1,841269841
SNX9	0,000384038	0,088954076	0,232	0,077	1	1,063598816	0,94020413	3,012987013
LRRC8E	0,008401736	0,088949122	0,024	0	1	1,063595163	0,94020736	#DIV/0!
CEP41	0,004102792	0,088892066	0,098	0,025	1	1,063553101	0,940244544	3,92
LRRC41	0,000651262	0,08859858	0,183	0,056	1	1,063336766	0,940435836	3,267857143
NUDT16L1	0,000127297	0,088584422	0,207	0,06	1	1,06332633	0,940445065	3,45
ARF4	0,05206881	0,088583011	0,634	0,446	1	1,06332529	0,940445985	1,421524664
WIPF2	0,00096313	0,088450752	0,256	0,098	1	1,063227815	0,940532204	2,612244898
GSPT2	4,88624E-05	0,088440522	0,098	0,011	1	1,063220275	0,940538874	8,909090909
ARMC6	0,000651072	0,088404776	0,146	0,039	1	1,063193932	0,940562177	3,743589744
APOM	0,00031979	0,088285413	0,122	0,025	1	1,063105972	0,940639999	4,88
FBXO16	0,008401736	0,088250447	0,024	0	1	1,063080206	0,940662797	#DIV/0!
EEF2KMT	5,58308E-05	0,088137468	0,085	0,007	1	1,062996958	0,940736465	12,14285714
EIF3K	0,000584763	0,088122796	0,671	0,347	1	1,062986147	0,940746032	1,933717579
ARL8B	3,99546E-05	0,08803661	0,354	0,126	0,843080964	1,062922647	0,940802233	2,80952381
GAL3ST4	0,000298361	0,087961514	0,085	0,011	1	1,062867321	0,940851206	7,727272727
GPAT4	0,000262066	0,087946861	0,232	0,077	1	1,062856525	0,940860762	3,012987013
ZNF33A	0,014137725	0,087930785	0,098	0,032	1	1,062844682	0,940871246	3,0625
LBR	1,96144E-06	0,087910916	0,256	0,06	0,041388341	1,062830044	0,940884204	4,266666667
ANO1	0,00032444	0,087872925	0,061	0,004	1	1,062802057	0,940908981	15,25
LPAR4	0,000332109	0,087848842	0,073	0,007	1	1,062784316	0,940924687	10,42857143

Supplementary Table 2

Differentially expressed genes_keloid-XL_ comparing SC-Keloid vs. (SC-Myel, SC-Nonmyel, SC-Nonmyel2)								
**gene**	**p_val**	**avg_log2FC**	**pct.1**	**pct.2**	**p_val_adj**	**Foldchange_UP**	**Foldchange_DOWN**	**Ratio_pct1_pct2**
BAG4	0,01352207	0,087650617	0,183	0,081	1	1,0626383	0,941053979	2,259259259
DHX16	0,003053309	0,087583875	0,122	0,035	1	1,062589141	0,941097515	3,485714286
TTLL12	0,010188387	0,087536958	0,073	0,018	1	1,062554586	0,94112812	4,055555556
TAFAZZIN	0,033702567	0,087519228	0,134	0,06	1	1,062541528	0,941139686	2,233333333
SAP30L	0,00079604	0,087499274	0,305	0,126	1	1,062526832	0,941152703	2,420634921
TIMELESS	0,011339437	0,087427468	0,037	0,004	1	1,062473949	0,941199547	9,25
CNTRL	0,015153743	0,087422601	0,134	0,053	1	1,062470365	0,941202723	2,528301887
FAM174A	0,00017554	0,087349199	0,22	0,067	1	1,062416309	0,941250611	3,28358209
JADE3	0,001802592	0,087265128	0,061	0,007	1	1,0623544	0,941305462	8,714285714
SLFN5	1,98732E-05	0,087078619	0,195	0,046	0,419345238	1,06221707	0,94142716	4,239130435
SNCG	0,060153875	0,087053432	0,049	0,014	1	1,062198525	0,941443597	3,5
NATD1	5,32686E-05	0,087044118	0,073	0,004	1	1,062191668	0,941449675	18,25
UBXN11	0,002631406	0,087024709	0,085	0,018	1	1,062177378	0,94146234	4,722222222
GRK5	0,058268295	0,087009218	0,049	0,014	1	1,062165973	0,941472449	3,5
ANKRD49	0,014394785	0,086934126	0,098	0,032	1	1,062110689	0,941521454	3,0625
TPI1	0,004808897	0,086929107	0,805	0,484	1	1,062106994	0,941524729	1,66322314
RFX5	0,006340275	0,086864029	0,085	0,021	1	1,062059085	0,941567201	4,047619048
CHMP7	0,00050647	0,086821497	0,134	0,032	1	1,062027775	0,94159496	4,1875
CASTOR1	8,66134E-06	0,08680606	0,085	0,004	0,1827629	1,062016411	0,941605035	21,25
RANGRF	6,36949E-06	0,086788176	0,207	0,046	0,134402518	1,062003246	0,941616707	4,5
RALGAPA2	0,00296515	0,08674472	0,122	0,035	1	1,061971257	0,941645071	3,485714286
VRK1	0,002767295	0,086690636	0,085	0,018	1	1,061931447	0,941680372	4,722222222
AHCY	1,37509E-08	0,086654578	0,341	0,077	0,000290159	1,061904906	0,941703908	4,428571429
UBE2F	0,000372541	0,086626724	0,268	0,098	1	1,061884404	0,94172209	2,734693878
YJU2B	6,03301E-05	0,086494955	0,305	0,102	1	1,061787421	0,941808106	2,990196078
OGFRL1	0,002698569	0,086494379	0,085	0,018	1	1,061786997	0,941808482	4,722222222
RRP8	0,000212764	0,086464057	0,159	0,039	1	1,061764681	0,941828277	4,076923077
SLC22A18AS	0,001876622	0,086438627	0,049	0,004	1	1,061745965	0,941844879	12,25
TMEM129	0,00024535	0,086407429	0,207	0,063	1	1,061723006	0,941865246	3,285714286
CNFN	1,02173E-05	0,086396663	0,098	0,007	0,215595665	1,061715083	0,941872274	14

Supplementary Table 2

Differentially expressed genes_keloid-XL_ comparing SC-Keloid vs. (SC-Myel, SC-Nonmyel, SC-Nonmyel2)								
gene	p_val	avg_log2FC	pct.1	pct.2	p_val_adj	Foldchange_UP	Foldchange_DOWN	Ratio_pct1_pct2
VPS52	0,002095478	0,086375987	0,134	0,039	1	1,061699867	0,941885773	3,435897436
WRAP53	5,89403E-05	0,086177551	0,098	0,011	1	1,061553846	0,942015334	8,909090909
DTX2	3,64556E-07	0,086136105	0,146	0,014	0,007692497	1,061523349	0,942042397	10,42857143
UTP14A	0,00405833	0,086128825	0,098	0,025	1	1,061517993	0,94204715	3,92
DMTF1	0,001547529	0,086112147	0,159	0,049	1	1,061505721	0,942058041	3,244897959
ZNF391	0,011128779	0,086004666	0,037	0,004	1	1,061426642	0,942128227	9,25
OSBP2	0,043000486	0,085971375	0,037	0,007	1	1,061402149	0,942149967	5,285714286
DYSF	5,90574E-05	0,085965946	0,085	0,007	1	1,061398155	0,942153513	12,14285714
PHF5A	0,000635621	0,085796188	0,195	0,063	1	1,06127327	0,94226438	3,095238095
PCDHGB7	0,099301202	0,085786411	0,037	0,011	1	1,061266078	0,942270766	3,363636364
KCNMB3	0,00032444	0,085769146	0,061	0,004	1	1,061253378	0,942282042	15,25
PXN-AS1	2,62657E-05	0,085739126	0,134	0,021	0,554232678	1,061231295	0,94230165	6,380952381
CFAP97	1,86553E-05	0,085572637	0,622	0,263	0,393644434	1,061108835	0,942410399	2,365019011
LRPPRC	0,000641617	0,085541291	0,28	0,109	1	1,06108578	0,942430875	2,568807339
PPIL6	0,000284208	0,085515218	0,085	0,011	1	1,061066604	0,942447907	7,727272727
TMEM87B	4,90412E-06	0,08548928	0,159	0,025	0,103481741	1,061047527	0,942464852	6,36
PSMG4	0,000228156	0,08539568	0,207	0,063	1	1,060978691	0,942525999	3,285714286
ZNF2	0,065143806	0,08534444	0,024	0,004	1	1,060941008	0,942559476	6
HDDC3	4,3682E-05	0,085319624	0,11	0,014	0,921734445	1,060922759	0,942575689	7,857142857
TMX4	5,2807E-07	0,085315025	0,439	0,14	0,011142803	1,060919377	0,942578693	3,135714286
NUP88	2,74188E-05	0,085308737	0,134	0,021	0,578564323	1,060914753	0,942582802	6,380952381
ITPRIPL2	2,58919E-07	0,085298141	0,415	0,123	0,005463454	1,060906961	0,942589725	3,37398374
THY1	0,049682602	0,085243088	0,11	0,049	1	1,060866478	0,942625694	2,244897959
C5orf34	2,80682E-05	0,085218497	0,061	0	0,592266086	1,060848395	0,942641762	#DIV/0!
SLC29A1	6,246E-05	0,085196532	0,085	0,007	1	1,060832244	0,942656114	12,14285714
MAPKAP1	0,000410787	0,085154968	0,268	0,098	1	1,060801682	0,942683271	2,734693878
PLA2G4A	0,000103093	0,08509707	0,159	0,035	1	1,060759111	0,942721104	4,542857143
EPN2	0,001757527	0,085052616	0,305	0,133	1	1,060726426	0,942750152	2,293233083
IQCE	1,42345E-05	0,085050452	0,159	0,028	0,300362154	1,060724835	0,942751567	5,678571429
OR7C1	5,21474E-05	0,085030594	0,073	0,004	1	1,060710235	0,942764543	18,25

Supplementary Table 2

Differentially expressed genes_keloid-XL_ comparing SC-Keloid vs. (SC-Myel, SC-Nonmyel, SC-Nonmyel2)								
**gene**	**p_val**	**avg_log2FC**	**pct.1**	**pct.2**	**p_val_adj**	**Foldchange_UP**	**Foldchange_DOWN**	**Ratio_pct1_pct2**
DDX56	0,023818859	0,084976838	0,134	0,056	1	1,060670713	0,942799672	2,392857143
YDJC	1,08272E-05	0,084966759	0,098	0,007	0,228465315	1,060663303	0,942806259	14
MAST2	0,05064237	0,084764232	0,134	0,063	1	1,060514416	0,94293862	2,126984127
ARFGAP2	4,81779E-06	0,084660279	0,293	0,081	0,101660119	1,060438004	0,943006566	3,617283951
NOL8	0,00244171	0,084572848	0,146	0,046	1	1,06037374	0,943063716	3,173913043
TBC1D22A	0,003759144	0,084565427	0,098	0,025	1	1,060368286	0,943068567	3,92
MAEA	0,000496265	0,084483945	0,256	0,095	1	1,060308399	0,943121832	2,694736842
ARFGAP1	0,000901737	0,084452595	0,28	0,112	1	1,060285358	0,943142327	2,5
ELP6	0,000148395	0,084363856	0,171	0,042	1	1,060220143	0,943200341	4,071428571
PLEKHH3	0,02311933	0,0843572	0,232	0,119	1	1,060215252	0,943204692	1,949579832
SNX21	1,33137E-05	0,084336228	0,159	0,028	0,280931553	1,06019984	0,943218403	5,678571429
KNSTRN	0,000332109	0,084311223	0,073	0,007	1	1,060181465	0,943234751	10,42857143
MAGEF1	1,01637E-05	0,084307247	0,305	0,091	0,214465091	1,060178543	0,943237351	3,351648352
FLRT1	0,052459136	0,084292139	0,134	0,063	1	1,060167441	0,943247228	2,126984127
NOP9	0,016864604	0,084247881	0,061	0,014	1	1,060134918	0,943276165	4,357142857
PEX6	0,000304206	0,08423239	0,122	0,025	1	1,060123535	0,943286294	4,88
BORCS8	2,42595E-05	0,084115476	0,171	0,035	0,511899209	1,060037627	0,94336274	4,885714286
DEF8	5,03394E-08	0,084084957	0,28	0,056	0,001062213	1,060015203	0,943382696	5
UBL7	7,30342E-05	0,084082436	0,134	0,025	1	1,060013351	0,943384344	5,36
HYKK	1,06202E-05	0,084024289	0,098	0,007	0,224096034	1,059970629	0,943422367	14
MUC12	0,000304206	0,083997818	0,122	0,025	1	1,059951181	0,943439678	4,88
NSMCE3	7,12794E-09	0,083995576	0,341	0,074	0,000150407	1,059949533	0,943441144	4,608108108
EXTL2	3,62294E-05	0,083981513	0,244	0,07	0,764475716	1,059939201	0,943450341	3,485714286
ETV4	8,66134E-06	0,08393237	0,085	0,004	0,1827629	1,059903097	0,943482478	21,25
GSK3A	1,60713E-05	0,083917492	0,317	0,102	0,339120865	1,059892167	0,943492208	3,107843137
MORN3	0,00032444	0,083901013	0,061	0,004	1	1,05988006	0,943502985	15,25
POLA2	0,003982392	0,083879785	0,073	0,014	1	1,059864465	0,943516868	5,214285714
PFAS	1,12366E-05	0,083879362	0,11	0,011	0,23710448	1,059864154	0,943517144	10
HEATR1	5,55011E-05	0,08385753	0,171	0,039	1	1,059848115	0,943531423	4,384615385
DDX60L	6,13057E-05	0,083851795	0,085	0,007	1	1,059843902	0,943535173	12,14285714

Supplementary Table 2

Differentially expressed genes_keloid-XL_ comparing SC-Keloid vs. (SC-Myel, SC-Nonmyel, SC-Nonmyel2)								
**gene**	**p_val**	**avg_log2FC**	**pct.1**	**pct.2**	**p_val_adj**	**Foldchange_UP**	**Foldchange_DOWN**	**Ratio_pct1_pct2**
PIGW	8,66134E-06	0,083794073	0,085	0,004	0,1827629	1,059801499	0,943572925	21,25
BCAS4	0,00012579	0,083751767	0,122	0,021	1	1,059770422	0,943600595	5,80952381
CERK	0,001673714	0,083738606	0,122	0,032	1	1,059760754	0,943609203	3,8125
ADAMTS13	0,008401736	0,083717816	0,024	0	1	1,059745482	0,943622801	#DIV/0!
GON7	0,000111268	0,08368296	0,122	0,021	1	1,059719879	0,943645599	5,80952381
DOP1A	0,006411568	0,083672549	0,085	0,021	1	1,059712231	0,943652409	4,047619048
CA11	0,001492907	0,083671066	0,159	0,049	1	1,059711142	0,943653379	3,244897959
CINP	4,4995E-06	0,083650044	0,159	0,025	0,094944043	1,0596957	0,94366713	6,36
ARMC7	5,32686E-05	0,083589497	0,073	0,004	1	1,059651228	0,943706734	18,25
SERPINB5	0,016463985	0,083552598	0,061	0,014	1	1,059624127	0,943730871	4,357142857
CASP4	0,000938221	0,083548087	0,183	0,06	1	1,059620813	0,943733822	3,05
RASL11B	4,3682E-05	0,083540276	0,11	0,014	0,921734445	1,059615076	0,943738932	7,857142857
PHF6	0,009816935	0,08345436	0,171	0,07	1	1,059551976	0,943795135	2,442857143
ICA1	0,006163779	0,083414271	0,061	0,011	1	1,059522534	0,943821361	5,545454545
ZNF711	0,041525285	0,083393737	0,098	0,039	1	1,059507453	0,943834795	2,512820513
ZNF277	0,010863732	0,083304118	0,146	0,056	1	1,05944164	0,943893427	2,607142857
FAM222B	0,516980245	0,08321746	0,024	0,014	1	1,059378005	0,943950125	1,714285714
VPS37C	1,17619E-05	0,083158235	0,195	0,042	0,24818749	1,059334517	0,943988876	4,642857143
MAN2A1	1,5947E-05	0,082977469	0,293	0,088	0,336497046	1,059201793	0,944107163	3,329545455
CREBL2	0,000641125	0,082862466	0,195	0,063	1	1,059117363	0,944182425	3,095238095
SLC30A7	8,57134E-05	0,082841057	0,207	0,056	1	1,059101646	0,944196436	3,696428571
ERI2	0,004311516	0,082837246	0,073	0,014	1	1,059098848	0,944198931	5,214285714
PCDHB14	0,0059273	0,082795032	0,085	0,021	1	1,059067859	0,944226559	4,047619048
C14orf28	0,001798559	0,082784912	0,098	0,021	1	1,05906043	0,944233182	4,666666667
PCOLCE2	1,39592E-06	0,082752421	0,098	0,004	0,029455268	1,059036579	0,944254448	24,5
TMEM150A	0,000649127	0,082670422	0,098	0,018	1	1,058976388	0,944308118	5,444444444
COQ2	1,36536E-06	0,082597894	0,098	0,004	0,028810488	1,058923152	0,944355592	24,5
TBL3	0,00018544	0,082561739	0,134	0,028	1	1,058896615	0,944379258	4,785714286
HAUS2	0,058461383	0,0825279	0,122	0,056	1	1,058871779	0,944401409	2,178571429
AKIP1	0,000465553	0,082506695	0,134	0,032	1	1,058856215	0,944415291	4,1875

Supplementary Table 2

Differentially expressed genes_keloid-XL_ comparing SC-Keloid vs. (SC-Myel, SC-Nonmyel, SC-Nonmyel2)								
gene	p_val	avg_log2FC	pct.1	pct.2	p_val_adj	Foldchange_UP	Foldchange_DOWN	Ratio_pct1_pct2
PDCD2L	5,32686E-05	0,082461195	0,073	0,004	1	1,058822821	0,944445076	18,25
FAHD1	0,000264599	0,082449858	0,146	0,035	1	1,0588145	0,944452498	4,171428571
ZC3H13	4,24249E-07	0,082406403	0,439	0,137	0,008952082	1,058782609	0,944480946	3,204379562
CCDC113	6,01716E-05	0,082361674	0,085	0,007	1	1,058749783	0,944510229	12,14285714
RIOK1	0,000815224	0,082268547	0,159	0,046	1	1,058681442	0,9445712	3,456521739
RPL13A	0,427836809	0,082207958	0,988	0,888	1	1,058636982	0,94461087	1,112612613
ARMT1	0,000220667	0,082180633	0,098	0,014	1	1,058616931	0,944628761	7
REP15	0,000332109	0,082152225	0,073	0,007	1	1,058596086	0,944647362	10,42857143
CDK10	0,001694829	0,082148016	0,195	0,07	1	1,058592998	0,944650118	2,785714286
SPDL1	0,008145887	0,082012618	0,098	0,028	1	1,058493653	0,944738778	3,5
GPR39	0,000183568	0,081965974	0,049	0	1	1,058459431	0,944769323	#DIV/0!
ACSL3	0,004152316	0,08187636	0,329	0,154	1	1,058393686	0,94482801	2,136363636
PDPK1	0,002668862	0,0817809	0,195	0,074	1	1,058323657	0,944890529	2,635135135
AP4S1	0,040095269	0,081776165	0,073	0,025	1	1,058320183	0,94489363	2,92
EML1	6,01472E-05	0,081737379	0,171	0,039	1	1,058291731	0,944919034	4,384615385
DZIP1L	0,010071401	0,081714763	0,073	0,018	1	1,058275141	0,944933847	4,055555556
GATD3A	9,64031E-06	0,081671093	0,098	0,007	0,20342014	1,058243108	0,94496245	14
PUF60	0,001723599	0,081594219	0,585	0,295	1	1,058186721	0,945012803	1,983050847
ARSI	4,72873E-05	0,081585291	0,11	0,014	0,997809544	1,058180173	0,945018651	7,857142857
ZKSCAN4	2,80682E-05	0,081547507	0,061	0	0,592266086	1,058152459	0,945043402	#DIV/0!
NSUN5	9,07324E-05	0,081538191	0,159	0,035	1	1,058145627	0,945049504	4,542857143
GM2A	0,001194179	0,081476558	0,146	0,042	1	1,058100422	0,945089879	3,476190476
GATC	2,51588E-05	0,0814403	0,134	0,021	0,530876209	1,05807383	0,945113631	6,380952381
KLHDC10	0,000749037	0,081436033	0,244	0,091	1	1,058070701	0,945116426	2,681318681
CIRBP	0,107517407	0,081433256	0,902	0,656	1	1,058068664	0,945118246	1,375
UMAD1	0,000336119	0,08141429	0,122	0,025	1	1,058054755	0,94513067	4,88
MICAL3	0,003951254	0,081408337	0,329	0,158	1	1,05805039	0,94513457	2,082278481
NDUFAF1	5,68952E-05	0,081372518	0,146	0,028	1	1,058024121	0,945158036	5,214285714
TAOK3	0,000179872	0,0813141	0,354	0,14	1	1,057981279	0,945196309	2,528571429
SEMA6A	0,000124579	0,081287248	0,146	0,032	1	1,057961588	0,945213901	4,5625

Supplementary Table 2

Differentially expressed genes_keloid-XL_ comparing SC-Keloid vs. (SC-Myel, SC-Nonmyel, SC-Nonmyel2)								
gene	p_val	avg_log2FC	pct.1	pct.2	p_val_adj	Foldchange_UP	Foldchange_DOWN	Ratio_pct1_pct2
RAD18	0,346572919	0,081273407	0,049	0,028	1	1,057951438	0,945222969	1,75
RRM2B	0,015078158	0,081240693	0,146	0,06	1	1,057927449	0,945244403	2,433333333
PEX7	0,000742666	0,081234895	0,098	0,018	1	1,057923197	0,945248202	5,444444444
AP1AR	0,009423296	0,081184854	0,171	0,07	1	1,057886503	0,945280989	2,442857143
DNAAF3	0,000183568	0,081181805	0,049	0	1	1,057884267	0,945282987	#DIV/0!
NXT2	0,000157878	0,081043717	0,11	0,018	1	1,057783016	0,945373469	6,111111111
GPR180	0,000839209	0,081030087	0,134	0,035	1	1,057773023	0,945382401	3,828571429
INTS7	4,65444E-05	0,080962033	0,11	0,014	0,9821336	1,057723127	0,945426997	7,857142857
ABCB6	0,001713147	0,080891827	0,061	0,007	1	1,057671657	0,945473005	8,714285714
DYNC2LI1	1,48158E-05	0,080726923	0,159	0,028	0,312628803	1,057550769	0,945581082	5,678571429
BMS1	3,1659E-06	0,080649862	0,28	0,074	0,06680361	1,057494282	0,945631591	3,783783784
EIF2AK2	1,97578E-07	0,080600395	0,378	0,105	0,004169088	1,057458023	0,945664015	3,6
GUF1	1,91355E-06	0,080578177	0,134	0,014	0,0403779	1,057441738	0,945678579	9,571428571
JPT1	6,47486E-06	0,080570543	0,268	0,074	0,136625959	1,057436142	0,945683583	3,621621622
NVL	8,26419E-06	0,080556011	0,171	0,032	0,174382754	1,057425491	0,945693108	5,34375
RET	0,063203483	0,080469133	0,012	0	1	1,057361815	0,94575006	#DIV/0!
AMOT	0,107332601	0,080399549	0,061	0,025	1	1,057310818	0,945795676	2,44
LYPLA2	5,72295E-05	0,080269915	0,195	0,049	1	1,057215817	0,945880665	3,979591837
ZNF511	1,26621E-05	0,080251184	0,232	0,06	0,267183289	1,057202091	0,945892946	3,866666667
CD6	0,000183568	0,080226535	0,049	0	1	1,057184029	0,945909107	#DIV/0!
TPBG	0,009031726	0,080218909	0,049	0,007	1	1,05717844	0,945914107	7
LIPA	7,35158E-05	0,080216533	0,256	0,081	1	1,057176699	0,945915664	3,160493827
DCUN1D1	0,001047006	0,080198281	0,268	0,105	1	1,057163325	0,945927631	2,552380952
MPI	0,000160149	0,080191123	0,11	0,018	1	1,05715808	0,945932325	6,111111111
SNUPN	0,002401544	0,080167668	0,183	0,067	1	1,057140893	0,945947704	2,731343284
DCUN1D2	0,006431528	0,080149737	0,061	0,011	1	1,057127754	0,94595946	5,545454545
XRN2	0,004264256	0,080139718	0,341	0,165	1	1,057120412	0,94596603	2,066666667
NEIL2	0,001772311	0,080129911	0,061	0,007	1	1,057113227	0,94597246	8,714285714
PPFIA1	2,70394E-05	0,080127997	0,366	0,13	0,570557601	1,057111824	0,945973716	2,815384615
CCDC57	0,005992082	0,080107756	0,122	0,039	1	1,057096993	0,945986988	3,128205128

Supplementary Table 2

Differentially expressed genes_keloid-XL_ comparing SC-Keloid vs. (SC-Myel, SC-Nonmyel, SC-Nonmyel2)								
**gene**	**p_val**	**avg_log2FC**	**pct.1**	**pct.2**	**p_val_adj**	**Foldchange_UP**	**Foldchange_DOWN**	**Ratio_pct1_pct2**
HERPUD2	1,95737E-06	0,080078179	0,195	0,035	0,041302386	1,057075321	0,946006382	5,571428571
ECRG4	4,32324E-06	0,080074149	0,073	0	0,09122464	1,057072368	0,946009025	#DIV/0!
TPX2	0,059519563	0,080036468	0,049	0,014	1	1,05704476	0,946033733	3,5
TNFRSF1A	6,70333E-06	0,079999279	0,415	0,147	0,141446985	1,057017512	0,94605812	2,823129252
TNFAIP8	0,000308034	0,079965102	0,122	0,025	1	1,056992472	0,946080532	4,88
ENTPD5	0,005342271	0,079964639	0,11	0,032	1	1,056992133	0,946080835	3,4375
TRIP6	3,33782E-06	0,079910368	0,39	0,126	0,070431359	1,056952372	0,946116425	3,095238095
KCNRG	0,001220269	0,079908489	0,037	0	1	1,056950995	0,946117658	#DIV/0!
DDX39A	0,000711065	0,079894695	0,341	0,144	1	1,05694089	0,946126703	2,368055556
RNF4	0,000189302	0,079892084	0,232	0,074	1	1,056938977	0,946128416	3,135135135
SCCPDH	0,000955702	0,079870331	0,659	0,326	1	1,05692304	0,946142682	2,021472393
BEAN1	0,000317802	0,079843234	0,061	0,004	1	1,056903189	0,946160453	15,25
MINDY3	7,13605E-06	0,079839811	0,183	0,035	0,150577744	1,056900681	0,946162698	5,228571429
STK25	2,31592E-07	0,079795549	0,354	0,095	0,004886813	1,056868256	0,946191726	3,726315789
FAM118B	1,36355E-06	0,079783638	0,159	0,021	0,028772289	1,05685953	0,946199539	7,571428571
TOR4A	2,80682E-05	0,079753708	0,061	0	0,592266086	1,056837605	0,946219168	#DIV/0!
NAP1L5	0,00140181	0,079747227	0,073	0,011	1	1,056832858	0,946223419	6,636363636
PRRC1	0,000190951	0,079668961	0,232	0,074	1	1,056775526	0,946274753	3,135135135
USP35	0,060793535	0,079661361	0,049	0,014	1	1,056769959	0,946279738	3,5
NCKAP5L	8,8541E-05	0,079642824	0,183	0,046	1	1,056756381	0,946291896	3,97826087
DIP2A	0,004375273	0,079639466	0,28	0,13	1	1,056753921	0,946294099	2,153846154
POU5F2	0,009102897	0,079494947	0,049	0,007	1	1,056648068	0,946388897	7
FAM229B	0,00035282	0,079453714	0,244	0,084	1	1,056617869	0,946415946	2,904761905
RNFT1	5,69725E-05	0,079371335	0,098	0,011	1	1,056557537	0,946469988	8,909090909
STX2	0,000367621	0,079316928	0,354	0,144	1	1,056517693	0,946505682	2,458333333
SLC66A1	0,002983177	0,079310979	0,085	0,018	1	1,056513337	0,946509585	4,722222222
CLCN6	0,009413802	0,079285616	0,11	0,035	1	1,056494763	0,946526225	3,142857143
FAHD2B	0,00140181	0,079244406	0,073	0,011	1	1,056464585	0,946553263	6,636363636
HRCT1	8,66134E-06	0,079213517	0,085	0,004	0,1827629	1,056441966	0,946573529	21,25
DRAM2	1,77191E-05	0,079181982	0,244	0,067	0,373891544	1,056418874	0,94659422	3,641791045

Supplementary Table 2

Differentially expressed genes_keloid-XL_ comparing SC-Keloid vs. (SC-Myel, SC-Nonmyel, SC-Nonmyel2)								
**gene**	**p_val**	**avg_log2FC**	**pct.1**	**pct.2**	**p_val_adj**	**Foldchange_UP**	**Foldchange_DOWN**	**Ratio_pct1_pct2**
NELFCD	1,45285E-05	0,079167962	0,329	0,109	0,3065655	1,056408608	0,946603419	3,018348624
ZNF627	0,00032444	0,07914894	0,061	0,004	1	1,056394679	0,9466159	15,25
PPP2CB	0,014076621	0,07911044	0,683	0,414	1	1,056366488	0,946641162	1,649758454
COMMD8	7,83921E-08	0,078991013	0,256	0,049	0,001654152	1,056279045	0,946719529	5,224489796
LEKR1	0,008401736	0,078942628	0,024	0	1	1,05624362	0,94675128	#DIV/0!
TBC1D13	0,004147695	0,078846596	0,098	0,025	1	1,056173315	0,946814302	3,92
SLX1A	0,001952737	0,078832034	0,049	0,004	1	1,056162654	0,946823859	12,25
RAPGEF6	0,004750396	0,078805159	0,22	0,091	1	1,05614298	0,946841497	2,417582418
PRKD2	7,24402E-06	0,078737872	0,207	0,046	0,152856065	1,056093723	0,946885658	4,5
PHACTR2	0,018791522	0,078737845	0,183	0,084	1	1,056093703	0,946885676	2,178571429
UNC13B	0,001003155	0,078736296	0,134	0,035	1	1,056092569	0,946886693	3,828571429
RPS13	0,16109071	0,078657872	0,963	0,898	1	1,056035162	0,946938166	1,072383073
RCBTB2	0,065834577	0,07862391	0,061	0,021	1	1,056010303	0,946960458	2,904761905
TARBP2	5,79485E-05	0,078535985	0,098	0,011	1	1,055945946	0,947018173	8,909090909
AGBL4	0,001220269	0,078512859	0,037	0	1	1,05592902	0,947033353	#DIV/0!
FANCC	0,001833348	0,078443312	0,061	0,007	1	1,055878119	0,947079007	8,714285714
ST7L	0,023657379	0,078416202	0,134	0,056	1	1,055858278	0,947096804	2,392857143
NAPEPLD	0,000276176	0,078308253	0,171	0,046	1	1,055779276	0,947167673	3,717391304
EDEM2	0,000182598	0,078270261	0,159	0,039	1	1,055751473	0,947192616	4,076923077
LIAS	0,006067769	0,078175387	0,134	0,046	1	1,055682048	0,947254906	2,913043478
KIF4A	0,064667995	0,078166336	0,061	0,021	1	1,055675425	0,947260849	2,904761905
IFIT5	0,100834226	0,078124689	0,073	0,032	1	1,055644951	0,947288195	2,28125
ZNF611	0,001914332	0,078124641	0,049	0,004	1	1,055644915	0,947288227	12,25
GINS3	4,17072E-05	0,078076542	0,207	0,053	0,880063939	1,055609721	0,947319809	3,905660377
SPATA2L	9,91418E-06	0,078057957	0,122	0,014	0,209199094	1,055596123	0,947332013	8,714285714
INTS9	0,008401736	0,078050991	0,024	0	1	1,055591026	0,947336587	#DIV/0!
BIRC5	0,001914332	0,078035576	0,049	0,004	1	1,055579748	0,947346709	12,25
AC007952.4	0,002474156	0,077990644	0,207	0,081	1	1,055546873	0,947376214	2,555555556
PLEKHS1	0,000338108	0,077972706	0,073	0,007	1	1,055533748	0,947387994	10,42857143
AC002454.1	2,80682E-05	0,077917977	0,061	0	0,592266086	1,055493707	0,947423934	#DIV/0!

Supplementary Table 2

Differentially expressed genes_keloid-XL_ comparing SC-Keloid vs. (SC-Myel, SC-Nonmyel, SC-Nonmyel2)								
**gene**	**p_val**	**avg_log2FC**	**pct.1**	**pct.2**	**p_val_adj**	**Foldchange_UP**	**Foldchange_DOWN**	**Ratio_pct1_pct2**
TXNRD2	0,007277368	0,077895415	0,159	0,06	1	1,0554772	0,947438751	2,65
ACTR8	0,000201519	0,077884953	0,098	0,014	1	1,055469546	0,947445621	7
CFAP20DC	4,32324E-06	0,077795993	0,073	0	0,09122464	1,055404466	0,947504044	#DIV/0!
PGM2	0,000988848	0,077782707	0,11	0,025	1	1,055394746	0,94751277	4,4
PYCR2	0,000143162	0,077765196	0,329	0,126	1	1,055381937	0,947524271	2,611111111
CAMKMT	0,024538047	0,077755896	0,085	0,028	1	1,055375133	0,947530379	3,035714286
EFHB	0,063203483	0,077730952	0,012	0	1	1,055356886	0,947546762	#DIV/0!
KEL	0,063203483	0,077730952	0,012	0	1	1,055356886	0,947546762	#DIV/0!
LGALS2	0,063203483	0,077730952	0,012	0	1	1,055356886	0,947546762	#DIV/0!
STAB1	0,063203483	0,077730952	0,012	0	1	1,055356886	0,947546762	#DIV/0!
UBQLNL	0,063203483	0,077730952	0,012	0	1	1,055356886	0,947546762	#DIV/0!
ZFP82	0,063203483	0,077730952	0,012	0	1	1,055356886	0,947546762	#DIV/0!
GBF1	1,18313E-05	0,077726898	0,195	0,042	0,249652213	1,055353921	0,947549424	4,642857143
UBB	3,62718E-10	0,077690997	0,854	0,4	7,65371E-06	1,055327658	0,947573005	2,135
CBX2	0,05889129	0,077667811	0,049	0,014	1	1,055310698	0,947588233	3,5
DHX33	0,002095478	0,077508163	0,134	0,039	1	1,055193924	0,947693099	3,435897436
PDCD10	0,000753737	0,077466405	0,415	0,182	1	1,055163383	0,947720529	2,28021978
AMH	0,010921605	0,077337814	0,037	0,004	1	1,055069338	0,947805006	9,25
STT3A	0,000118625	0,077295316	0,195	0,053	1	1,055038259	0,947832926	3,679245283
GLI3	0,000790232	0,07728549	0,439	0,2	1	1,055031073	0,947839382	2,195
FOXC2	0,00140181	0,077214616	0,073	0,011	1	1,054979244	0,947885947	6,636363636
NFU1	2,2882E-05	0,077209258	0,341	0,112	0,482833656	1,054975327	0,947889467	3,044642857
SLC25A35	0,059519805	0,077177486	0,049	0,014	1	1,054952094	0,947910342	3,5
SMG6	0,02622152	0,077134693	0,183	0,088	1	1,054920802	0,94793846	2,079545455
FZR1	0,001557671	0,077132351	0,171	0,056	1	1,054919089	0,947939999	3,053571429
SLC31A2	1,00787E-05	0,077052926	0,122	0,014	0,212671388	1,054861014	0,947992187	8,714285714
ULK4	0,000332109	0,077045337	0,073	0,007	1	1,054855465	0,947997174	10,42857143
MCM2	0,183494953	0,077025727	0,024	0,007	1	1,054841128	0,948010059	3,428571429
MCM4	0,006556321	0,077000502	0,085	0,021	1	1,054822684	0,948026635	4,047619048
DCAF7	0,005194404	0,076980579	0,28	0,13	1	1,054808117	0,948039727	2,153846154

Supplementary Table 2

Differentially expressed genes_keloid-XL_ comparing SC-Keloid vs. (SC-Myel, SC-Nonmyel, SC-Nonmyel2)								
**gene**	**p_val**	**avg_log2FC**	**pct.1**	**pct.2**	**p_val_adj**	**Foldchange_UP**	**Foldchange_DOWN**	**Ratio_pct1_pct2**
LSS	0,003269243	0,076878078	0,134	0,042	1	1,054733178	0,948107086	3,19047619
CCNB2	0,065143806	0,076873227	0,024	0,004	1	1,054729632	0,948110274	6
ELMOD1	0,001220269	0,076790454	0,037	0	1	1,054669119	0,948164672	#DIV/0!
SRRM3	0,008401736	0,076771039	0,024	0	1	1,054654926	0,948177433	#DIV/0!
NKAP	0,000777309	0,076691501	0,232	0,084	1	1,054596783	0,948229708	2,761904762
LZTFL1	0,000445012	0,076688079	0,134	0,032	1	1,054594281	0,948231958	4,1875
GNG11	3,69832E-08	0,076627377	0,598	0,2	0,000780383	1,05454991	0,948271855	2,99
TEX9	0,00140181	0,076545573	0,073	0,011	1	1,054490116	0,948325627	6,636363636
BBOF1	0,00047996	0,07653513	0,11	0,021	1	1,054482484	0,948332491	5,238095238
BPNT1	0,001236845	0,07653109	0,11	0,025	1	1,05447953	0,948335147	4,4
ZFP62	0,002640395	0,07650774	0,11	0,028	1	1,054462464	0,948350495	3,928571429
INTS3	0,004480116	0,076492281	0,232	0,098	1	1,054451165	0,948360657	2,367346939
TIGAR	0,000292988	0,076447285	0,122	0,025	1	1,054418278	0,948390236	4,88
ARHGEF40	0,000928531	0,076445335	0,5	0,235	1	1,054416853	0,948391518	2,127659574
PRPF8	0,000433914	0,076443813	0,366	0,154	1	1,054415741	0,948392518	2,376623377
CAPN1	3,75508E-05	0,076433686	0,415	0,158	0,792359671	1,05440834	0,948399175	2,626582278
BRMS1L	0,00031979	0,076401105	0,122	0,025	1	1,054384528	0,948420594	4,88
TMEM184C	0,041519021	0,07637243	0,085	0,032	1	1,054363571	0,948439445	2,65625
FSD1	6,13057E-05	0,07633643	0,085	0,007	1	1,054337262	0,948463112	12,14285714
CCDC186	0,020615501	0,07630044	0,22	0,109	1	1,05431096	0,948486773	2,018348624
DEGS1	0,003397095	0,076298206	0,537	0,274	1	1,054309327	0,948488242	1,959854015
DHRS7B	4,13314E-05	0,076180579	0,183	0,042	0,872133784	1,05422337	0,948565578	4,357142857
RHBDD3	0,000685139	0,076172099	0,098	0,018	1	1,054217173	0,948571154	5,444444444
RAB30-DT	0,000504006	0,076135103	0,171	0,049	1	1,054190139	0,948595479	3,489795918
SMIM10	0,000354997	0,076129502	0,183	0,053	1	1,054186047	0,948599161	3,452830189
NCAPD2	0,000197116	0,076075469	0,134	0,028	1	1,054146566	0,948634689	4,785714286
FGF5	0,000183568	0,075956209	0,049	0	1	1,054059428	0,948713112	#DIV/0!
EDRF1	0,036803449	0,075759821	0,061	0,018	1	1,053915953	0,948842265	3,388888889
MTRF1	0,065249136	0,075747673	0,061	0,021	1	1,053907079	0,948850254	2,904761905
GLB1	8,58922E-07	0,075697844	0,22	0,042	0,018124104	1,053870679	0,948883027	5,238095238

Supplementary Table 2

Differentially expressed genes_keloid-XL_ comparing SC-Keloid vs. (SC-Myel, SC-Nonmyel, SC-Nonmyel2)								
**gene**	**p_val**	**avg_log2FC**	**pct.1**	**pct.2**	**p_val_adj**	**Foldchange_UP**	**Foldchange_DOWN**	**Ratio_pct1_pct2**
NSUN2	0,000770218	0,075684483	0,122	0,028	1	1,053860919	0,948891815	4,357142857
SLC25A23	9,91418E-06	0,075648424	0,122	0,014	0,209199094	1,053834579	0,948915532	8,714285714
ALDH3B1	0,000744212	0,075645478	0,122	0,028	1	1,053832427	0,948917469	4,357142857
IFI35	0,000635621	0,075627955	0,195	0,063	1	1,053819627	0,948928995	3,095238095
CLPB	0,065834577	0,07560988	0,061	0,021	1	1,053806425	0,948940884	2,904761905
FZD6	0,000204598	0,075557919	0,098	0,014	1	1,053768471	0,948975062	7
ZNF407-AS1	0,003268385	0,075547813	0,122	0,035	1	1,053761089	0,94898171	3,485714286
POLR1E	7,68227E-07	0,075444679	0,183	0,028	0,016210348	1,053685761	0,949049552	6,535714286
ZNF721	0,16838416	0,075415669	0,134	0,081	1	1,053664574	0,949068636	1,654320988
HAT1	0,001091792	0,075366474	0,305	0,13	1	1,053628646	0,949100999	2,346153846
SELENOO	0,000932352	0,075323878	0,134	0,035	1	1,053597537	0,949129022	3,828571429
ZNF69	0,000183568	0,075310036	0,049	0	1	1,053587429	0,949138128	#DIV/0!
TRPM4	0,004255004	0,075297292	0,073	0,014	1	1,053578121	0,949146513	5,214285714
SH2B1	0,005482257	0,075265256	0,22	0,095	1	1,053554727	0,949167589	2,315789474
ATF1	0,002949192	0,075209992	0,207	0,081	1	1,05351437	0,949203949	2,555555556
ADGRE2	2,80682E-05	0,075207706	0,061	0	0,592266086	1,0535127	0,949205453	#DIV/0!
DOCK7	0,001315659	0,07511636	0,354	0,161	1	1,053445998	0,949265555	2,198757764
WDR3	0,000217361	0,075048092	0,098	0,014	1	1,05339615	0,949310476	7
BBS1	0,111964825	0,075015922	0,049	0,018	1	1,053372661	0,949331644	2,722222222
ZNF548	0,016463985	0,074987408	0,061	0,014	1	1,053351842	0,949350407	4,357142857
SEC23IP	0,002296533	0,074974517	0,11	0,028	1	1,05334243	0,94935889	3,928571429
PI4K2B	0,00095592	0,074947643	0,085	0,014	1	1,053322809	0,949376574	6,071428571
CBR4	0,003102112	0,074927289	0,171	0,06	1	1,053307949	0,949389968	2,85
ATR	0,000713367	0,074894318	0,098	0,018	1	1,053283877	0,949411666	5,444444444
AP2A2	2,16516E-06	0,0748195	0,354	0,109	0,045686941	1,053229255	0,949460903	3,247706422
OXCT1	0,016651717	0,074793006	0,122	0,046	1	1,053209913	0,94947834	2,652173913
ATP6V1D	2,63989E-05	0,074739981	0,39	0,14	0,557043007	1,053171204	0,949513238	2,785714286
ST8SIA4	0,000183568	0,074707357	0,049	0	1	1,053147389	0,949534709	#DIV/0!
BCS1L	0,000199533	0,074682037	0,134	0,028	1	1,053128906	0,949551374	4,785714286
HSD17B4	6,64597E-05	0,074665246	0,293	0,098	1	1,053116649	0,949562426	2,989795918

Supplementary Table 2

Differentially expressed genes_keloid-XL_ comparing SC-Keloid vs. (SC-Myel, SC-Nonmyel, SC-Nonmyel2)								
**gene**	**p_val**	**avg_log2FC**	**pct.1**	**pct.2**	**p_val_adj**	**Foldchange_UP**	**Foldchange_DOWN**	**Ratio_pct1_pct2**
HS1BP3	9,44252E-07	0,074548265	0,207	0,039	0,019924668	1,05303126	0,949639425	5,307692308
HOXC5	0,001898897	0,074522259	0,098	0,021	1	1,053012278	0,949656543	4,666666667
SFT2D3	2,04211E-06	0,074508895	0,122	0,011	0,043090571	1,053002524	0,94966534	11,09090909
ZNF81	0,000997638	0,074506894	0,085	0,014	1	1,053001064	0,949666657	6,071428571
NF2	0,000905476	0,074495626	0,159	0,046	1	1,05299284	0,949674074	3,456521739
PIM1	0,035683499	0,074491653	0,134	0,06	1	1,05298994	0,949676689	2,233333333
SRBD1	0,00405833	0,074441489	0,098	0,025	1	1,052953327	0,949709711	3,92
YTHDF3-DT	2,07968E-06	0,07439743	0,122	0,011	0,043883378	1,052921171	0,949738715	11,09090909
PEDS1	4,12502E-05	0,074355609	0,293	0,095	0,870421401	1,052890649	0,949766247	3,084210526
AKR7A2	2,55312E-06	0,074296716	0,305	0,084	0,05387347	1,052847669	0,949805019	3,630952381
TRAM1L1	0,000338108	0,074274604	0,073	0,007	1	1,052831533	0,949819576	10,42857143
RBM42	0,000351959	0,074263106	0,415	0,182	1	1,052823142	0,949827146	2,28021978
ZNF414	3,21999E-05	0,074189613	0,183	0,042	0,679450306	1,052769511	0,949875533	4,357142857
SLC43A1	0,008401736	0,074140943	0,024	0	1	1,052733996	0,949907578	#DIV/0!
KLHL17	0,000167149	0,074070497	0,11	0,018	1	1,052682593	0,949953962	6,111111111
GCDH	0,006115259	0,074069136	0,146	0,053	1	1,0526816	0,949954858	2,754716981
MEN1	0,026163616	0,074062207	0,098	0,035	1	1,052676544	0,949959421	2,8
BRPF1	0,003205568	0,074012735	0,122	0,035	1	1,052640446	0,949991997	3,485714286
DRD2	5,21474E-05	0,073937733	0,073	0,004	1	1,052585724	0,950041386	18,25
NFYA	0,485575622	0,073889612	0,061	0,042	1	1,052550616	0,950073075	1,452380952
CFAP251	0,001772311	0,073888168	0,061	0,007	1	1,052549562	0,950074026	8,714285714
KIAA2013	2,8305E-07	0,073853373	0,366	0,102	0,005972641	1,052524177	0,95009694	3,588235294
ZNF175	0,103792275	0,073770869	0,037	0,011	1	1,052463988	0,950151275	3,363636364
C11orf1	0,000624735	0,073756121	0,146	0,039	1	1,052453229	0,950160988	3,743589744
FAM162A	5,43281E-07	0,073694982	0,378	0,109	0,011463774	1,052408628	0,950201256	3,467889908
BAG2	0,000214102	0,073690639	0,098	0,014	1	1,052405461	0,950204116	7
DSCC1	0,006251893	0,073571313	0,061	0,011	1	1,052318419	0,950282711	5,545454545
RBM18	0,000133816	0,073568672	0,146	0,032	1	1,052316492	0,950284451	4,5625
TF	0,066254792	0,073554499	0,024	0,004	1	1,052306154	0,950293786	6
TMEM223	8,68688E-08	0,073553405	0,256	0,049	0,001833018	1,052305357	0,950294507	5,224489796

Supplementary Table 2

Differentially expressed genes_keloid-XL_ comparing SC-Keloid vs. (SC-Myel, SC-Nonmyel, SC-Nonmyel2)								
gene	p_val	avg_log2FC	pct.1	pct.2	p_val_adj	Foldchange_UP	Foldchange_DOWN	Ratio_pct1_pct2
ZKSCAN1	0,0001082	0,073544923	0,293	0,102	1	1,05229917	0,950300094	2,87254902
POU3F2	0,016274784	0,073516318	0,183	0,081	1	1,052278305	0,950318936	2,259259259
SUPT3H	1,72088E-05	0,07349788	0,183	0,039	0,363123284	1,052264858	0,950331081	4,692307692
DNAJC18	0,001194205	0,073410497	0,11	0,025	1	1,052201124	0,950388644	4,4
PSORS1C1	0,000338108	0,073369196	0,073	0,007	1	1,052171003	0,950415852	10,42857143
CC2D2A	0,014921301	0,07335729	0,098	0,032	1	1,05216232	0,950423695	3,0625
DROSHA	2,10551E-05	0,073349221	0,146	0,025	0,44428446	1,052156435	0,950429011	5,84
BNIP3	0,005276004	0,073205143	0,22	0,095	1	1,052051364	0,950523933	2,315789474
ZNF660	0,067018449	0,073202026	0,061	0,021	1	1,052049091	0,950525986	2,904761905
DLD	1,06888E-05	0,073149014	0,22	0,053	0,225545313	1,052010435	0,950560914	4,150943396
JAK2	0,000183568	0,073142581	0,049	0	1	1,052005743	0,950565153	#DIV/0!
GPS2	3,69029E-05	0,073131363	0,476	0,189	0,778688535	1,051997563	0,950572544	2,518518519
SEL1L	1,1546E-05	0,073124286	0,354	0,116	0,243632596	1,051992403	0,950577207	3,051724138
CBWD1	9,06518E-06	0,073079214	0,268	0,074	0,191284269	1,051959538	0,950606905	3,621621622
HSPA4	1,83347E-05	0,073005209	0,341	0,116	0,386880192	1,051905577	0,950655669	2,939655172
RPUSD1	0,000710805	0,072862484	0,122	0,028	1	1,051801518	0,950749722	4,357142857
CNEP1R1	0,013704854	0,072848636	0,085	0,025	1	1,051791422	0,950758847	3,4
CYYR1	0,183494953	0,072814515	0,024	0,007	1	1,051766547	0,950781334	3,428571429
C5orf58	0,008401736	0,072778581	0,024	0	1	1,05174035	0,950805016	#DIV/0!
DHDDS	0,001101503	0,072692882	0,134	0,035	1	1,051677877	0,950861497	3,828571429
NEMP2	8,66134E-06	0,072622762	0,085	0,004	0,1827629	1,051626762	0,950907714	21,25
MTHFD2L	5,55011E-05	0,072573131	0,171	0,039	1	1,051590585	0,950940427	4,384615385
TIRAP	0,00032444	0,072510994	0,061	0,004	1	1,051545295	0,950981384	15,25
CDK7	0,001831458	0,07245849	0,098	0,021	1	1,051507026	0,951015995	4,666666667
SPRING1	0,000166278	0,07239646	0,22	0,067	1	1,051461817	0,951056885	3,28358209
MCUB	0,00371808	0,072369614	0,098	0,025	1	1,051442251	0,951074583	3,92
PRCC	0,000436709	0,072367667	0,28	0,105	1	1,051440832	0,951075866	2,666666667
ENAH	6,2249E-05	0,072334376	0,171	0,039	1	1,051416569	0,951097813	4,384615385
SF3B2	0,004845864	0,072286496	0,463	0,242	1	1,051381675	0,951129379	1,91322314
C1orf50	5,81126E-05	0,072238356	0,171	0,039	1	1,051346594	0,951161117	4,384615385

Supplementary Table 2

Differentially expressed genes_keloid-XL_ comparing SC-Keloid vs. (SC-Myel, SC-Nonmyel, SC-Nonmyel2)								
gene	p_val	avg_log2FC	pct.1	pct.2	p_val_adj	Foldchange_UP	Foldchange_DOWN	Ratio_pct1_pct2
ENO4	0,001220269	0,072180807	0,037	0	1	1,051304657	0,951199059	#DIV/0!
ZNF319	0,006431528	0,072146432	0,061	0,011	1	1,051279607	0,951221724	5,545454545
RSPH9	0,00095592	0,072143147	0,085	0,014	1	1,051277214	0,951223889	6,071428571
HDAC6	0,069914007	0,072120469	0,122	0,06	1	1,051260689	0,951238842	2,033333333
CEP68	0,003205568	0,07208467	0,122	0,035	1	1,051234603	0,951262446	3,485714286
TMEM9	5,95845E-05	0,072069878	0,28	0,091	1	1,051223825	0,9512722	3,076923077
TCEAL7	2,19535E-06	0,072047178	0,293	0,077	0,046324103	1,051207285	0,951287167	3,805194805
GEMIN6	3,24105E-07	0,072028388	0,122	0,007	0,00683894	1,051193593	0,951299557	17,42857143
SPIN2A	0,008401736	0,07201636	0,024	0	1	1,051184829	0,951307489	#DIV/0!
PFKP	0,000360636	0,071860025	0,159	0,042	1	1,051070926	0,951410581	3,785714286
SPHK2	0,016147858	0,071756342	0,122	0,046	1	1,05099539	0,951478959	2,652173913
MRPL48	5,30011E-05	0,071723186	0,171	0,039	1	1,050971237	0,951500826	4,384615385
PLIN3	0,000192241	0,07171709	0,366	0,144	1	1,050966796	0,951504847	2,541666667
TBCK	0,000713367	0,071690093	0,098	0,018	1	1,05094713	0,951522652	5,444444444
ZCWPW2	0,00414399	0,071610946	0,073	0,014	1	1,050889475	0,951574855	5,214285714
CMTR1	0,002508126	0,071539956	0,146	0,046	1	1,050837767	0,951621679	3,173913043
EPS15L1	1,08291E-05	0,071437593	0,195	0,042	0,228504817	1,050763209	0,951689202	4,642857143
SPIRE2	0,347088417	0,071418194	0,012	0,004	1	1,05074908	0,951701999	3
HOXC-AS1	0,013704854	0,071408351	0,085	0,025	1	1,050741912	0,951708492	3,4
RPA3	0,000115675	0,071367659	0,207	0,06	1	1,050712275	0,951735336	3,45
MEDAG	0,006341138	0,071366267	0,061	0,011	1	1,050711261	0,951736254	5,545454545
FAM98A	0,000762572	0,071316646	0,22	0,077	1	1,050675123	0,951768989	2,857142857
CIAO3	0,000311907	0,0712919	0,122	0,025	1	1,050657102	0,951785315	4,88
UBR1	0,000642543	0,071258327	0,183	0,056	1	1,050632652	0,951807464	3,267857143
PPARD	0,040310506	0,071223077	0,098	0,039	1	1,050606982	0,95183072	2,512820513
DUSP4	0,001229283	0,071221296	0,146	0,042	1	1,050605685	0,951831895	3,476190476
PRKAR2B	4,32324E-06	0,071192187	0,073	0	0,09122464	1,050584487	0,9518511	#DIV/0!
DNPEP	0,000761642	0,07115754	0,207	0,07	1	1,050559257	0,95187396	2,957142857
RAP1GDS1	6,25578E-05	0,071139838	0,488	0,204	1	1,050546367	0,951885639	2,392156863
STARD3NL	0,002817764	0,071137663	0,232	0,095	1	1,050544783	0,951887074	2,442105263

Supplementary Table 2

Differentially expressed genes_keloid-XL_ comparing SC-Keloid vs. (SC-Myel, SC-Nonmyel, SC-Nonmyel2)								
**gene**	**p_val**	**avg_log2FC**	**pct.1**	**pct.2**	**p_val_adj**	**Foldchange_UP**	**Foldchange_DOWN**	**Ratio_pct1_pct2**
ZWILCH	0,000685144	0,071105051	0,098	0,018	1	1,050521036	0,951908592	5,444444444
MIER2	0,041181302	0,071100601	0,073	0,025	1	1,050517795	0,951911528	2,92
ABCG1	0,260282533	0,071098352	0,049	0,025	1	1,050516158	0,951913012	1,96
PES1	6,20327E-06	0,071098089	0,183	0,035	0,13089519	1,050515966	0,951913186	5,228571429
RUVBL2	0,000138689	0,071055572	0,207	0,06	1	1,050485007	0,95194124	3,45
ZNF598	0,009423296	0,071014384	0,171	0,07	1	1,050455017	0,951968417	2,442857143
ZNF578	0,008401736	0,070991474	0,024	0	1	1,050438336	0,951983534	#DIV/0!
FMN1	0,028081741	0,070946897	0,049	0,011	1	1,05040588	0,95201295	4,454545455
PABIR3	0,016864604	0,070928964	0,061	0,014	1	1,050392823	0,952024783	4,357142857
EPB41L4A	0,040202307	0,070858343	0,085	0,032	1	1,050341407	0,952071387	2,65625
ROPN1B	0,004199164	0,070854227	0,073	0,014	1	1,05033841	0,952074103	5,214285714
TRAPPC12	6,25295E-06	0,07082339	0,268	0,07	0,131943405	1,05031596	0,952094453	3,828571429
RNF180	5,32686E-05	0,070812039	0,073	0,004	1	1,050307696	0,952101945	18,25
WDR12	0,00095592	0,070801828	0,085	0,014	1	1,050300262	0,952108684	6,071428571
MMP16	5,32686E-05	0,070779384	0,073	0,004	1	1,050283923	0,952123496	18,25
LZTS1	0,000124092	0,07070669	0,122	0,021	1	1,050231003	0,952171472	5,80952381
DNAI4	0,347088417	0,070649652	0,012	0,004	1	1,050189482	0,952209117	3
FADS1	0,002570239	0,070639732	0,22	0,088	1	1,050182261	0,952215665	2,5
TMEM254	0,000279632	0,070400342	0,085	0,011	1	1,050008016	0,952373682	7,727272727
RAMAC	4,4719E-05	0,070222867	0,183	0,042	0,943616019	1,049878856	0,952490846	4,357142857
CHDH	0,911002271	0,070201288	0,012	0,014	1	1,049863153	0,952505093	0,857142857
POGLUT3	6,27412E-05	0,070188312	0,232	0,067	1	1,04985371	0,95251366	3,462686567
HDAC8	3,90623E-05	0,070181214	0,183	0,042	0,824254035	1,049848545	0,952518347	4,357142857
YY2	0,001742498	0,070174826	0,061	0,007	1	1,049843896	0,952522564	8,714285714
ZNF584	0,004368707	0,070134047	0,073	0,014	1	1,049814222	0,952549489	5,214285714
ZNF439	0,001003155	0,070070696	0,134	0,035	1	1,049768124	0,952591317	3,828571429
CUL2	1,13313E-06	0,07005325	0,207	0,039	0,023910185	1,049755429	0,952602837	5,307692308
AL162431.1	0,649511996	0,070036385	0,012	0,007	1	1,049743158	0,952613972	1,714285714
PRDM10	0,191478184	0,070013132	0,037	0,014	1	1,049726239	0,952629326	2,642857143
RINL	0,103792275	0,06999066	0,037	0,011	1	1,049709888	0,952644165	3,363636364

Supplementary Table 2

Differentially expressed genes_keloid-XL_ comparing SC-Keloid vs. (SC-Myel, SC-Nonmyel, SC-Nonmyel2)								
**gene**	**p_val**	**avg_log2FC**	**pct.1**	**pct.2**	**p_val_adj**	**Foldchange_UP**	**Foldchange_DOWN**	**Ratio_pct1_pct2**
NPIPA5	0,188110324	0,069956847	0,037	0,014	1	1,049685286	0,952666493	2,642857143
FAM98C	0,000247417	0,069912232	0,22	0,07	1	1,049652825	0,952695954	3,142857143
CYB5R4	1,11779E-05	0,069887028	0,159	0,028	0,235865591	1,049634487	0,952712599	5,678571429
TMEM154	0,000997638	0,069881537	0,085	0,014	1	1,049630492	0,952716225	6,071428571
SNAPC2	6,37734E-07	0,069869939	0,354	0,102	0,013456825	1,049622054	0,952723884	3,470588235
ATP6V1G1	0,000186641	0,069846734	0,695	0,344	1	1,049605172	0,952739208	2,020348837
KLHDC3	0,000161996	0,069835838	0,195	0,056	1	1,049597245	0,952746403	3,482142857
ZNF555	0,065143806	0,069834632	0,024	0,004	1	1,049596367	0,9527472	6
ACOT9	9,89053E-06	0,069824319	0,305	0,091	0,208699979	1,049588864	0,952754011	3,351648352
FAM110B	0,513508152	0,069823408	0,024	0,014	1	1,049588202	0,952754612	1,714285714
BVES	0,003268385	0,069786802	0,122	0,035	1	1,04956157	0,952778787	3,485714286
ZNF510	0,040816555	0,069766328	0,073	0,025	1	1,049546676	0,952792308	2,92
PREP	0,009955614	0,069765896	0,073	0,018	1	1,049546361	0,952792594	4,055555556
BIVM	0,01356931	0,069757804	0,085	0,025	1	1,049540475	0,952797938	3,4
DOLK	6,13057E-05	0,069736512	0,085	0,007	1	1,049524985	0,952812	12,14285714
HEPH	0,015316495	0,069685485	0,11	0,039	1	1,049487865	0,952845701	2,820512821
AL592183.1	0,178114335	0,069677629	0,049	0,021	1	1,04948215	0,952850889	2,333333333
PDCL3	3,40892E-05	0,069637904	0,183	0,042	0,719316414	1,049453253	0,952877127	4,357142857
NAPA	5,81918E-05	0,069627124	0,476	0,193	1	1,049445411	0,952884247	2,466321244
SEMA3E	0,001220269	0,069594061	0,037	0	1	1,049421361	0,952906084	#DIV/0!
SCNM1	7,22352E-06	0,069584929	0,268	0,074	0,152423491	1,049414718	0,952912117	3,621621622
ZNF100	0,001055888	0,06952009	0,085	0,014	1	1,049367555	0,952954944	6,071428571
LINC00526	6,246E-05	0,069517065	0,085	0,007	1	1,049365355	0,952956942	12,14285714
INTS14	9,91418E-06	0,069509678	0,122	0,014	0,209199094	1,049359982	0,952961822	8,714285714
CASTOR3	0,000234367	0,0695016	0,098	0,014	1	1,049354106	0,952967158	7
TPMT	0,00032897	0,069443075	0,183	0,053	1	1,049311539	0,953005817	3,452830189
MYPOP	0,060793535	0,069427994	0,049	0,014	1	1,04930057	0,953015779	3,5
ZNF668	0,002802255	0,069364155	0,085	0,018	1	1,049254139	0,953057951	4,722222222
HHAT	0,000332109	0,069362794	0,073	0,007	1	1,04925315	0,95305885	10,42857143
H2BC3	0,063203483	0,069349355	0,012	0	1	1,049243376	0,953067728	#DIV/0!

Supplementary Table 2

Differentially expressed genes_keloid-XL_ comparing SC-Keloid vs. (SC-Myel, SC-Nonmyel, SC-Nonmyel2)								
**gene**	**p_val**	**avg_log2FC**	**pct.1**	**pct.2**	**p_val_adj**	**Foldchange_UP**	**Foldchange_DOWN**	**Ratio_pct1_pct2**
TMEM109	1,56722E-05	0,069287448	0,524	0,211	0,330699952	1,049198353	0,953108625	2,483412322
GPR176	0,009329829	0,069231064	0,11	0,035	1	1,049157349	0,953145876	3,142857143
FANCA	0,006163779	0,069227734	0,061	0,011	1	1,049154927	0,953148076	5,545454545
FANCL	0,001112971	0,069185424	0,11	0,025	1	1,049124159	0,953176029	4,4
CASP7	5,81663E-05	0,069182094	0,232	0,067	1	1,049121738	0,953178229	3,462686567
CACNB4	0,064048232	0,069057788	0,024	0,004	1	1,049031346	0,953260361	6
CDKL5	0,015883623	0,069043504	0,098	0,032	1	1,04902096	0,9532698	3,0625
KIAA1614	0,193178656	0,069017333	0,037	0,014	1	1,049001931	0,953287092	2,642857143
STK19	0,000165969	0,069015712	0,207	0,06	1	1,049000752	0,953288163	3,45
TP53	0,000926625	0,069005677	0,28	0,112	1	1,048993455	0,953294794	2,5
THAP7	0,000176699	0,068952778	0,195	0,056	1	1,048954993	0,953329749	3,482142857
UBE2G2	0,000432529	0,068926095	0,402	0,172	1	1,048935592	0,953347381	2,337209302
DELE1	3,72758E-05	0,068844632	0,244	0,07	0,786557383	1,048876365	0,953401214	3,485714286
MEIG1	0,008401736	0,068737237	0,024	0	1	1,048798289	0,953472188	#DIV/0!
PROCR	0,000287585	0,068703326	0,073	0,007	1	1,048773637	0,9534946	10,42857143
HSD17B8	4,58126E-05	0,068689455	0,11	0,014	0,966691375	1,048763553	0,953503768	7,857142857
GPRASP2	2,80682E-05	0,068686294	0,061	0	0,592266086	1,048761256	0,953505857	#DIV/0!
IRF3	0,00022786	0,068616128	0,354	0,14	1	1,04871025	0,953552232	2,528571429
C18orf21	0,101551261	0,068561357	0,073	0,032	1	1,048670437	0,953588434	2,28125
TRAPPC8	0,003974717	0,068505013	0,146	0,049	1	1,048629482	0,953625677	2,979591837
PRC1	0,110292725	0,068493968	0,061	0,025	1	1,048621454	0,953632978	2,44
OSBPL3	0,000194727	0,068485623	0,134	0,028	1	1,048615389	0,953638494	4,785714286
ZNF219	0,210417192	0,068450043	0,244	0,168	1	1,048589528	0,953662013	1,452380952
ALG12	2,51654E-05	0,068405424	0,171	0,035	0,531014339	1,048557098	0,953691508	4,885714286
ZNF580	6,81628E-06	0,068387303	0,476	0,175	0,143830269	1,048543928	0,953703487	2,72
CENPQ	1,87659E-07	0,068326442	0,195	0,028	0,003959786	1,048499696	0,95374372	6,964285714
COQ5	1,62791E-05	0,068220155	0,207	0,049	0,343505831	1,048422453	0,953813987	4,224489796
HFE	0,036427227	0,068210071	0,061	0,018	1	1,048415125	0,953820654	3,388888889
ALDH9A1	0,000914102	0,06817569	0,28	0,112	1	1,04839014	0,953843385	2,5
CRMP1	0,063203483	0,06817349	0,012	0	1	1,048388541	0,95384484	#DIV/0!

Supplementary Table 2

Differentially expressed genes_keloid-XL_ comparing SC-Keloid vs. (SC-Myel, SC-Nonmyel, SC-Nonmyel2)								
**gene**	**p_val**	**avg_log2FC**	**pct.1**	**pct.2**	**p_val_adj**	**Foldchange_UP**	**Foldchange_DOWN**	**Ratio_pct1_pct2**
IWS1	0,005294521	0,068172452	0,183	0,07	1	1,048387786	0,953845526	2,614285714
CDPF1	0,000288855	0,068171756	0,085	0,011	1	1,048387281	0,953845986	7,727272727
GEMIN2	0,004102792	0,068167429	0,098	0,025	1	1,048384136	0,953848847	3,92
FAM200A	0,000338108	0,068059508	0,073	0,007	1	1,048305715	0,953920203	10,42857143
PDCL	0,000710721	0,068010334	0,195	0,063	1	1,048269984	0,953952718	3,095238095
EMC8	1,88294E-07	0,067968044	0,317	0,077	0,003973196	1,048239257	0,953980681	4,116883117
AC040977.1	0,347088417	0,06794392	0,012	0,004	1	1,048221728	0,953996633	3
DPH6	0,010139169	0,067912742	0,122	0,042	1	1,048199076	0,95401725	2,904761905
STMN3	4,31504E-09	0,067905829	0,439	0,112	9,10516E-05	1,048194053	0,954021822	3,919642857
ELOVL4	0,004147695	0,06784227	0,098	0,025	1	1,048147875	0,954063853	3,92
TWNK	8,66134E-06	0,067800155	0,085	0,004	0,1827629	1,048117278	0,954091704	21,25
RNF135	0,00021046	0,067742753	0,159	0,039	1	1,048075577	0,954129666	4,076923077
PAPPA2	0,008401736	0,067619211	0,024	0	1	1,047985831	0,954211374	#DIV/0!
WRAP73	9,23892E-05	0,067576274	0,183	0,046	1	1,047954641	0,954239774	3,97826087
KLHL23	0,006523076	0,067567051	0,061	0,011	1	1,047947942	0,954245875	5,545454545
CEP250	0,010188387	0,067483328	0,073	0,018	1	1,047887129	0,954301253	4,055555556
ARMCX6	0,002539974	0,067437525	0,28	0,123	1	1,047853861	0,954331551	2,276422764
PDRG1	5,33977E-05	0,067414339	0,232	0,067	1	1,047837021	0,954346888	3,462686567
RSL24D1	8,27571E-05	0,067353194	0,61	0,281	1	1,047792612	0,954387337	2,170818505
RANBP3	0,001171783	0,067311877	0,183	0,06	1	1,047762605	0,954414669	3,05
AFF2	0,001220269	0,067288505	0,037	0	1	1,04774563	0,954430132	#DIV/0!
CCDC121	0,043000486	0,067222413	0,037	0,007	1	1,047697633	0,954473856	5,285714286
ASB13	2,80682E-05	0,067198387	0,061	0	0,592266086	1,047680185	0,954489752	#DIV/0!
NUDT15	0,005038283	0,067186609	0,11	0,032	1	1,047671632	0,954497544	3,4375
PHOSPHO2	5,32686E-05	0,067162534	0,073	0,004	1	1,047654149	0,954513473	18,25
ABT1	5,5243E-06	0,067162295	0,146	0,021	0,11656823	1,047653976	0,954513631	6,952380952
ST3GAL3	0,002498171	0,067135842	0,171	0,06	1	1,047634766	0,954531133	2,85
GPRIN1	0,000183568	0,067105498	0,049	0	1	1,047612732	0,954551209	#DIV/0!
MED19	0,01915118	0,067103315	0,171	0,077	1	1,047611146	0,954552654	2,220779221
C19orf44	1,25621E-08	0,067082837	0,183	0,018	0,000265072	1,047596277	0,954566203	10,16666667

Supplementary Table 2

Differentially expressed genes_keloid-XL_ comparing SC-Keloid vs. (SC-Myel, SC-Nonmyel, SC-Nonmyel2)								
**gene**	**p_val**	**avg_log2FC**	**pct.1**	**pct.2**	**p_val_adj**	**Foldchange_UP**	**Foldchange_DOWN**	**Ratio_pct1_pct2**
NGEF	0,000183568	0,067082223	0,049	0	1	1,047595831	0,954566609	#DIV/0!
NFATC2IP	1,46979E-05	0,067054267	0,293	0,088	0,310139365	1,047575531	0,954585107	3,329545455
BOLA3-AS1	0,001445239	0,067017453	0,073	0,011	1	1,0475488	0,954609466	6,636363636
MFHAS1	0,002565774	0,066927024	0,085	0,018	1	1,047483141	0,954669303	4,722222222
RCOR1	0,006941731	0,066926455	0,183	0,074	1	1,047482727	0,95466968	2,472972973
FICD	9,43577E-06	0,066863893	0,122	0,014	0,199104144	1,047437305	0,954711079	8,714285714
NDC80	0,000183568	0,066862169	0,049	0	1	1,047436053	0,95471222	#DIV/0!
INTS13	0,000120762	0,066860504	0,122	0,021	1	1,047434845	0,954713322	5,80952381
MECR	2,40964E-05	0,066823598	0,134	0,021	0,508457956	1,04740805	0,954737745	6,380952381
S100A9	0,347088417	0,066794536	0,012	0,004	1	1,047386951	0,954756978	3
RMI1	0,001742498	0,06671608	0,061	0,007	1	1,047329994	0,9548089	8,714285714
TONSL	0,008401736	0,066715801	0,024	0	1	1,047329791	0,954809085	#DIV/0!
CNOT10	0,02702556	0,066698144	0,098	0,035	1	1,047316973	0,954820771	2,8
RAB3GAP2	0,020471455	0,066650813	0,195	0,091	1	1,047282614	0,954852096	2,142857143
HDAC10	0,000187722	0,066625157	0,134	0,028	1	1,04726399	0,954869077	4,785714286
HTRA3	0,347088417	0,066590953	0,012	0,004	1	1,047239161	0,954891716	3
PRPSAP2	0,001725089	0,066577649	0,171	0,056	1	1,047229504	0,954900522	3,053571429
CCR1	0,011128779	0,066456583	0,037	0,004	1	1,047141628	0,954980657	9,25
BNIP1	0,000575008	0,066453752	0,146	0,039	1	1,047139573	0,954982531	3,743589744
TRMU	0,000140328	0,066453101	0,146	0,032	1	1,047139101	0,954982962	4,5625
RIN3	0,001298447	0,066392697	0,073	0,011	1	1,04709526	0,955022946	6,636363636
ZNHIT3	2,24762E-06	0,066344896	0,244	0,056	0,047426935	1,047060566	0,95505459	4,357142857
SCAMP1	0,000797971	0,066323633	0,293	0,116	1	1,047045134	0,955068666	2,525862069
NT5C	0,000213484	0,06629673	0,439	0,189	1	1,047025609	0,955086477	2,322751323
ZDHHC3	0,001059744	0,066270575	0,195	0,067	1	1,047006628	0,955103791	2,910447761
ZNF865	0,087380487	0,066252528	0,098	0,046	1	1,046993531	0,955115739	2,130434783
STARD4	0,008751649	0,066168329	0,049	0,007	1	1,046932428	0,955171483	7
BCDIN3D	0,001772311	0,066131191	0,061	0,007	1	1,046905478	0,955196072	8,714285714
HYPK	0,260460861	0,066082262	0,073	0,042	1	1,046869972	0,955228468	1,738095238
WDR77	0,000265199	0,066062141	0,171	0,046	1	1,046855372	0,955241791	3,717391304

Supplementary Table 2

Differentially expressed genes_keloid-XL_ comparing SC-Keloid vs. (SC-Myel, SC-Nonmyel, SC-Nonmyel2)								
**gene**	**p_val**	**avg_log2FC**	**pct.1**	**pct.2**	**p_val_adj**	**Foldchange_UP**	**Foldchange_DOWN**	**Ratio_pct1_pct2**
RWDD3	1,43623E-06	0,066007311	0,256	0,06	0,030305912	1,046815587	0,955278096	4,266666667
SEC16A	6,36887E-05	0,065960544	0,171	0,039	1	1,046781653	0,955309063	4,384615385
CABLES1	0,147332039	0,065908435	0,073	0,035	1	1,046743845	0,955343569	2,085714286
METTL14	0,063551963	0,065814648	0,085	0,035	1	1,0466758	0,955405675	2,428571429
PLAAT1	0,001220269	0,065771599	0,037	0	1	1,046644569	0,955434184	#DIV/0!
CIAO1	0,000126344	0,065758659	0,341	0,13	1	1,046635181	0,955442754	2,623076923
KIF27	0,001772311	0,06557642	0,061	0,007	1	1,04650298	0,955563452	8,714285714
ATE1	0,000587352	0,065568252	0,232	0,081	1	1,046497055	0,955568862	2,864197531
KIF9-AS1	0,001713147	0,065529207	0,061	0,007	1	1,046468733	0,955594723	8,714285714
NPLOC4	0,001564553	0,065520466	0,256	0,105	1	1,046462393	0,955600513	2,438095238
GRK6	0,000486977	0,065469849	0,134	0,032	1	1,046425679	0,955634041	4,1875
MEOX1	0,02175178	0,065382872	0,073	0,021	1	1,046362593	0,955691656	3,476190476
POP4	3,76534E-05	0,065335286	0,159	0,032	0,794523918	1,046328081	0,955723179	4,96875
PDIK1L	0,036427227	0,065256253	0,061	0,018	1	1,046270763	0,955775537	3,388888889
DTWD2	0,016864604	0,065235206	0,061	0,014	1	1,046255499	0,95578948	4,357142857
DHX37	0,111964825	0,065115329	0,049	0,018	1	1,046168567	0,955868902	2,722222222
TPRA1	0,006884292	0,065102372	0,146	0,053	1	1,046159171	0,955877487	2,754716981
ZNF487	0,004485159	0,065078814	0,073	0,014	1	1,046142089	0,955893096	5,214285714
KIF3B	2,75193E-05	0,065050019	0,195	0,046	0,580684188	1,046121209	0,955912175	4,239130435
TSPAN14	0,016613854	0,065021256	0,146	0,06	1	1,046100353	0,955931233	2,433333333
MALT1	0,014394785	0,064993981	0,098	0,032	1	1,046080575	0,955949306	3,0625
LRRCC1	5,02314E-05	0,064894397	0,39	0,147	1	1,046008371	0,956015294	2,653061224
NOP58	0,001281628	0,06488209	0,256	0,102	1	1,045999448	0,956023449	2,509803922
CRACR2B	0,008401736	0,06485022	0,024	0	1	1,045976342	0,956044568	#DIV/0!
IKZF2	0,001802592	0,064843396	0,061	0,007	1	1,045971394	0,956049091	8,714285714
LPCAT1	0,000214626	0,064842512	0,134	0,028	1	1,045970753	0,956049677	4,785714286
NIT2	9,29125E-06	0,064667959	0,354	0,116	0,196054616	1,045844208	0,956165357	3,051724138
MTIF2	0,001194205	0,064587566	0,11	0,025	1	1,045785931	0,95621864	4,4
ZNF12	0,040011563	0,06456268	0,098	0,039	1	1,045767891	0,956235134	2,512820513
C2orf68	0,002376906	0,064557279	0,146	0,046	1	1,045763976	0,956238714	3,173913043

Supplementary Table 2

Differentially expressed genes_keloid-XL_ comparing SC-Keloid vs. (SC-Myel, SC-Nonmyel, SC-Nonmyel2)								
**gene**	**p_val**	**avg_log2FC**	**pct.1**	**pct.2**	**p_val_adj**	**Foldchange_UP**	**Foldchange_DOWN**	**Ratio_pct1_pct2**
ACOX3	1,10381E-05	0,064507142	0,098	0,007	0,232915863	1,045727634	0,956271946	14
YTHDC2	0,000190951	0,064471572	0,232	0,074	1	1,045701851	0,956295524	3,135135135
EVI5L	0,183574298	0,06445767	0,049	0,021	1	1,045691775	0,956304739	2,333333333
MCCC1	0,040856169	0,064411433	0,085	0,032	1	1,045658262	0,956335388	2,65625
TTC7A	0,006341138	0,064411335	0,061	0,011	1	1,045658192	0,956335453	5,545454545
CCDC97	0,00019003	0,064408832	0,134	0,028	1	1,045656377	0,956337112	4,785714286
SLC43A3	0,005685201	0,064397492	0,256	0,116	1	1,045648158	0,956344629	2,206896552
CCAR1	0,000165246	0,064391865	0,317	0,119	1	1,04564408	0,956348359	2,663865546
ANKRD17	0,018313621	0,064391	0,305	0,161	1	1,045643453	0,956348933	1,894409938
GTF3C2	0,001149257	0,064364399	0,171	0,053	1	1,045624173	0,956366566	3,226415094
KLK6	0,18492249	0,064286207	0,11	0,063	1	1,045567503	0,956418402	1,746031746
CMPK1	0,017117784	0,064273224	0,451	0,256	1	1,045558094	0,956427009	1,76171875
ZXDC	0,010223539	0,064243995	0,122	0,042	1	1,045536911	0,956446386	2,904761905
CETN3	0,002470351	0,064238481	0,159	0,053	1	1,045532915	0,956450042	3
WNT10A	0,001220269	0,064230591	0,037	0	1	1,045527197	0,956455272	#DIV/0!
TRMT2A	0,000126076	0,06421894	0,146	0,032	1	1,045518754	0,956462997	4,5625
CALM2	0,33202586	0,064042231	0,854	0,835	1	1,045390701	0,956580156	1,022754491
CATSPER2	0,183494953	0,064013145	0,024	0,007	1	1,045369625	0,956599442	3,428571429
TBC1D8B	3,90131E-05	0,063897539	0,122	0,018	0,823214902	1,045285861	0,956676099	6,777777778
PLXNA1	0,005342271	0,063858053	0,11	0,032	1	1,045257252	0,956702283	3,4375
DHX36	0,000287151	0,063832946	0,488	0,214	1	1,045239062	0,956718933	2,280373832
HUS1	0,004892231	0,063682989	0,11	0,032	1	1,045130423	0,956818382	3,4375
OXSM	0,000300423	0,063548785	0,122	0,025	1	1,045033206	0,956907392	4,88
TTC26	0,004311516	0,063455471	0,073	0,014	1	1,044965615	0,956969287	5,214285714
PSPH	0,067616925	0,063379501	0,061	0,021	1	1,044910591	0,957019681	2,904761905
DENND2C	0,024758179	0,063364212	0,085	0,028	1	1,044899517	0,957029823	3,035714286
TEPSIN	3,8435E-05	0,063343724	0,122	0,018	0,811016692	1,044884679	0,957043414	6,777777778
TACC2	0,00479282	0,063271658	0,159	0,056	1	1,044832485	0,957091222	2,839285714
RPS6KL1	0,181340493	0,063242441	0,024	0,007	1	1,044811326	0,957110605	3,428571429
PROSER3	0,016864604	0,063191207	0,061	0,014	1	1,044774223	0,957144595	4,357142857

Supplementary Table 2

Differentially expressed genes_keloid-XL_ comparing SC-Keloid vs. (SC-Myel, SC-Nonmyel, SC-Nonmyel2)								
**gene**	**p_val**	**avg_log2FC**	**pct.1**	**pct.2**	**p_val_adj**	**Foldchange_UP**	**Foldchange_DOWN**	**Ratio_pct1_pct2**
DMPK	0,003291251	0,063027668	0,207	0,081	1	1,044655797	0,9572531	2,555555556
TOP3A	0,041186466	0,0630238	0,085	0,032	1	1,044652996	0,957255666	2,65625
SAYSD1	0,001265337	0,063018859	0,146	0,042	1	1,044649419	0,957258945	3,476190476
TDG	5,08305E-05	0,062994677	0,305	0,102	1	1,044631909	0,95727499	2,990196078
GID4	0,18978875	0,062974843	0,037	0,014	1	1,044617547	0,957288151	2,642857143
MAML3	0,003073142	0,062949298	0,183	0,067	1	1,044599051	0,957305101	2,731343284
DDX19B	2,30545E-07	0,062915883	0,22	0,039	0,004864739	1,044574857	0,957327274	5,641025641
TMEM237	2,41717E-06	0,062912679	0,183	0,032	0,05100473	1,044572537	0,9573294	5,71875
NEK8	0,001952737	0,062877638	0,049	0,004	1	1,044547166	0,957352652	12,25
SEPTIN1	0,014257957	0,062803424	0,146	0,06	1	1,044493435	0,957401901	2,433333333
TMEM233	0,00032621	0,062784076	0,073	0,007	1	1,044479427	0,957414741	10,42857143
ICAM3	3,35145E-07	0,06277737	0,159	0,018	0,007071888	1,044474572	0,957419192	8,833333333
ZFYVE19	0,001078907	0,06271986	0,134	0,035	1	1,044432937	0,957457358	3,828571429
ST6GALNAC4	1,32203E-06	0,062686002	0,159	0,021	0,027896192	1,044408426	0,957479828	7,571428571
TRAF3IP2-AS1	0,00380062	0,062659785	0,098	0,025	1	1,044389447	0,957497228	3,92
ZSCAN21	0,010071401	0,062568208	0,073	0,018	1	1,044323155	0,957558008	4,055555556
FXYD3	0,02349685	0,062525699	0,134	0,056	1	1,044292385	0,957586223	2,392857143
ZMAT5	2,83299E-08	0,062413359	0,268	0,049	0,000597789	1,04421107	0,957660791	5,469387755
TRIB1	0,065238659	0,06238735	0,159	0,081	1	1,044192245	0,957678056	1,962962963
AC023590.1	0,036803449	0,062238669	0,061	0,018	1	1,044084639	0,957776757	3,388888889
LANCL1	5,16791E-07	0,062209738	0,232	0,046	0,010904805	1,044063702	0,957795964	5,043478261
NOVA1	0,000240966	0,062193021	0,402	0,172	1	1,044051604	0,957807063	2,337209302
TRPM7	0,011511464	0,062158658	0,305	0,158	1	1,044026736	0,957829876	1,930379747
HECTD2	0,015178235	0,062105137	0,171	0,074	1	1,043988005	0,957865411	2,310810811
SYNC	0,000149616	0,062102192	0,207	0,06	1	1,043985874	0,957867366	3,45
CSTF2	0,001952737	0,062075871	0,049	0,004	1	1,043966828	0,957884842	12,25
NBEAL2	0,109440534	0,061976565	0,061	0,025	1	1,04389497	0,957950779	2,44
IRAK3	0,017481575	0,061941608	0,061	0,014	1	1,043869677	0,957973991	4,357142857
SMCO4	0,000171975	0,061908276	0,11	0,018	1	1,043845559	0,957996124	6,111111111
RNF144B	0,111964825	0,061823685	0,049	0,018	1	1,043784356	0,958052297	2,722222222

Supplementary Table 2

Differentially expressed genes_keloid-XL_ comparing SC-Keloid vs. (SC-Myel, SC-Nonmyel, SC-Nonmyel2)								
**gene**	**p_val**	**avg_log2FC**	**pct.1**	**pct.2**	**p_val_adj**	**Foldchange_UP**	**Foldchange_DOWN**	**Ratio_pct1_pct2**
PIK3CD	0,103792275	0,06182201	0,037	0,011	1	1,043783145	0,958053409	3,363636364
IFI27	0,000765513	0,06182065	0,415	0,2	1	1,04378216	0,958054312	2,075
REM1	0,006523076	0,061818053	0,061	0,011	1	1,043780282	0,958056037	5,545454545
ADAMTS15	0,001208265	0,06178108	0,11	0,025	1	1,043753533	0,95808059	4,4
ASB3	0,001745559	0,061737915	0,293	0,126	1	1,043722304	0,958109256	2,325396825
NOL11	0,030285543	0,061703634	0,146	0,067	1	1,043697504	0,958132022	2,179104478
HOXB3	0,042398422	0,061688856	0,037	0,007	1	1,043686812	0,958141837	5,285714286
ABHD16A	0,000770252	0,061677755	0,28	0,112	1	1,043678782	0,958149209	2,5
DUSP14	0,000347218	0,061659461	0,244	0,084	1	1,043665548	0,958161359	2,904761905
RTL10	0,026010433	0,061618102	0,049	0,011	1	1,043635628	0,958188828	4,454545455
RANBP6	0,015190866	0,061611653	0,098	0,032	1	1,043630963	0,958193112	3,0625
VEPH1	0,063203483	0,061548305	0,012	0	1	1,043585139	0,958235186	#DIV/0!
PYGL	0,078768491	0,061510696	0,146	0,077	1	1,043557935	0,958260166	1,896103896
CERS1	0,016343264	0,061503071	0,244	0,119	1	1,043552419	0,958265231	2,050420168
TTC13	0,001423372	0,061419858	0,073	0,011	1	1,04349223	0,958320504	6,636363636
SMPDL3A	0,004089475	0,061338503	0,073	0,014	1	1,043433388	0,958374546	5,214285714
CDRT4	0,00032444	0,061334774	0,061	0,004	1	1,043430691	0,958377024	15,25
AGAP1	0,048744367	0,061317478	0,354	0,211	1	1,043418181	0,958388514	1,677725118
FBXO8	0,000188702	0,061256633	0,159	0,039	1	1,043374177	0,958428934	4,076923077
ZNF639	0,000398607	0,061254132	0,195	0,06	1	1,043372368	0,958430595	3,25
SMYD2	2,80801E-05	0,061125915	0,171	0,035	0,592517408	1,043279644	0,958515778	4,885714286
MMP21	0,063203483	0,061014609	0,012	0	1	1,043199157	0,958589732	#DIV/0!
MAP3K7CL	0,001041037	0,061008982	0,085	0,014	1	1,043195088	0,958593471	6,071428571
THUMPD3-AS1	3,6929E-05	0,06100452	0,293	0,095	0,779239858	1,043191862	0,958596435	3,084210526
HARS1	1,57119E-05	0,060979226	0,268	0,077	0,331537855	1,043173572	0,958613242	3,480519481
TANGO2	6,13057E-05	0,060972372	0,085	0,007	1	1,043168616	0,958617796	12,14285714
TAF6L	6,13057E-05	0,060892938	0,085	0,007	1	1,043111182	0,958670578	12,14285714
TRIM32	0,017068103	0,060854152	0,061	0,014	1	1,043083139	0,958696352	4,357142857
PPIP5K2	0,000116217	0,060852885	0,28	0,095	1	1,043082222	0,958697194	2,947368421
SLC30A6	0,004311516	0,060850182	0,073	0,014	1	1,043080268	0,95869899	5,214285714

Supplementary Table 2

Differentially expressed genes_keloid-XL_ comparing SC-Keloid vs. (SC-Myel, SC-Nonmyel, SC-Nonmyel2)								
gene	p_val	avg_log2FC	pct.1	pct.2	p_val_adj	Foldchange_UP	Foldchange_DOWN	Ratio_pct1_pct2
USP3	0,077011555	0,060827565	0,11	0,053	1	1,043063916	0,95871402	2,075471698
DHRS4-AS1	3,75273E-07	0,060815128	0,134	0,011	0,007918625	1,043054925	0,958722284	12,18181818
TRMT1L	0,001055888	0,06080287	0,085	0,014	1	1,043046062	0,95873043	6,071428571
RTP4	0,009031726	0,06073788	0,049	0,007	1	1,042999076	0,95877362	7
TMEM102	4,89164E-05	0,060686576	0,073	0,004	1	1,042961986	0,958807716	18,25
PLAA	0,005447223	0,060679851	0,11	0,032	1	1,042957125	0,958812185	3,4375
NDUFAF6	0,001692235	0,06067329	0,159	0,049	1	1,042952382	0,958816545	3,244897959
PLGLB2	0,001952737	0,060607005	0,049	0,004	1	1,042904464	0,9588606	12,25
TMEM182	0,065143806	0,060507337	0,024	0,004	1	1,042832418	0,958926845	6
RASSF1	0,00486739	0,060501855	0,341	0,161	1	1,042828455	0,958930489	2,118012422
SMIM26	0,001592539	0,060485709	0,439	0,214	1	1,042816785	0,95894122	2,051401869
ADCY4	0,011128779	0,060466295	0,037	0,004	1	1,042802752	0,958954124	9,25
FOLR1	0,001220269	0,060445913	0,037	0	1	1,04278802	0,958967672	#DIV/0!
TMEM17	0,008145887	0,060410136	0,098	0,028	1	1,04276216	0,958991454	3,5
MT-ND6	0,043000486	0,0603199	0,037	0,007	1	1,04269694	0,959051438	5,285714286
PITPNB	0,001354578	0,060318554	0,5	0,242	1	1,042695968	0,959052332	2,066115702
IFT140	0,009174744	0,060226897	0,049	0,007	1	1,042629726	0,959113265	7
VARS1	0,000291801	0,060203273	0,22	0,07	1	1,042612653	0,95912897	3,142857143
MLH3	0,002075387	0,060198982	0,134	0,039	1	1,042609551	0,959131823	3,435897436
GORAB	8,9681E-05	0,060176025	0,159	0,035	1	1,042592961	0,959147085	4,542857143
SYT10	6,246E-05	0,060121441	0,085	0,007	1	1,042553516	0,959183375	12,14285714
ZNF19	0,001220269	0,060108052	0,037	0	1	1,04254384	0,959192277	#DIV/0!
CDK6	0,002062384	0,060096019	0,5	0,246	1	1,042535145	0,959200277	2,032520325
TBC1D2	0,065143806	0,059994795	0,024	0,004	1	1,042462	0,95926758	6
CDKN3	0,001802592	0,059988734	0,061	0,007	1	1,04245762	0,95927161	8,714285714
MRPL44	0,002284574	0,059970622	0,171	0,06	1	1,042444533	0,959283653	2,85
STAU2	0,015064968	0,059890875	0,11	0,039	1	1,042386912	0,95933668	2,820512821
TGS1	0,000381936	0,059871397	0,256	0,091	1	1,042372838	0,959349633	2,813186813
ENGASE	0,202985042	0,059852552	0,073	0,039	1	1,042359223	0,959362164	1,871794872
COP1	0,02471232	0,059826794	0,098	0,035	1	1,042340613	0,959379293	2,8

Supplementary Table 2

Differentially expressed genes_keloid-XL_ comparing SC-Keloid vs. (SC-Myel, SC-Nonmyel, SC-Nonmyel2)								
gene	p_val	avg_log2FC	pct.1	pct.2	p_val_adj	Foldchange_UP	Foldchange_DOWN	Ratio_pct1_pct2
EMP1	0,00052017	0,059816088	0,829	0,467	1	1,042332878	0,959386412	1,7751606
MYO5A	1,08253E-06	0,059750365	0,378	0,112	0,022842468	1,042285395	0,959430119	3,375
PCNA	0,000298566	0,059643166	0,28	0,102	1	1,042207951	0,959501411	2,745098039
FZD2	0,010546634	0,05961817	0,073	0,018	1	1,042189894	0,959518036	4,055555556
DPF1	2,80682E-05	0,059562567	0,061	0	0,592266086	1,042149727	0,959555018	#DIV/0!
TGFBRAP1	0,000124579	0,059545279	0,146	0,032	1	1,042137239	0,959566516	4,5625
SUV39H2	0,001742498	0,059525151	0,061	0,007	1	1,0421227	0,959579904	8,714285714
FRMPD4	0,000183568	0,059488243	0,049	0	1	1,04209604	0,959604453	#DIV/0!
CYB5D1	0,02737604	0,059482172	0,049	0,011	1	1,042091655	0,959608491	4,454545455
UBR4	2,78974E-08	0,059468618	0,39	0,102	0,000588662	1,042081864	0,959617506	3,823529412
LYSMD4	0,00032444	0,059430864	0,061	0,004	1	1,042054595	0,959642619	15,25
FZD1	0,008226643	0,059389554	0,098	0,028	1	1,042024757	0,959670098	3,5
SNAPC5	6,48528E-07	0,059247405	0,22	0,042	0,013684591	1,041922091	0,959764659	5,238095238
MRPL39	3,4874E-05	0,059198391	0,183	0,042	0,73587676	1,041886694	0,959797266	4,357142857
ODF3B	0,008890703	0,059192781	0,049	0,007	1	1,041882642	0,959800999	7
MSANTD2	0,036993563	0,059144572	0,11	0,046	1	1,041847827	0,959833072	2,391304348
C1QTNF4	0,02595188	0,059140655	0,098	0,035	1	1,041844998	0,959835678	2,8
PINK1-AS	0,001220269	0,059099052	0,037	0	1	1,041814955	0,959863357	#DIV/0!
ARL6	0,00032444	0,059076152	0,061	0,004	1	1,041798418	0,959878593	15,25
PAXIP1	0,567510207	0,059054449	0,037	0,025	1	1,041782746	0,959893033	1,48
LIN7B	0,008308113	0,059027412	0,098	0,028	1	1,041763223	0,959911022	3,5
DDX41	0,000807603	0,059012367	0,232	0,084	1	1,041752359	0,959921033	2,761904762
TCERG1	0,002606467	0,058996141	0,256	0,109	1	1,041740643	0,959931829	2,348623853
HMGCL	2,67471E-05	0,058992998	0,171	0,035	0,564390132	1,041738373	0,95993392	4,885714286
GAS2L1	2,30359E-05	0,058957982	0,22	0,056	0,486079608	1,041713089	0,959957219	3,928571429
FNDC10	0,011339437	0,05889478	0,037	0,004	1	1,041667455	0,959999274	9,25
DBR1	0,193178656	0,058880876	0,037	0,014	1	1,041657415	0,960008526	2,642857143
SCT	0,008401736	0,058782538	0,024	0	1	1,041586415	0,960073965	#DIV/0!
REEP4	0,001003155	0,058700629	0,134	0,035	1	1,041527281	0,960128475	3,828571429
PGPEP1	0,040856169	0,058664887	0,085	0,032	1	1,041501478	0,960152262	2,65625

Supplementary Table 2

Differentially expressed genes_keloid-XL_ comparing SC-Keloid vs. (SC-Myel, SC-Nonmyel, SC-Nonmyel2)								
gene	p_val	avg_log2FC	pct.1	pct.2	p_val_adj	Foldchange_UP	Foldchange_DOWN	Ratio_pct1_pct2
DSG2	5,32686E-05	0,058652775	0,073	0,004	1	1,041492734	0,960160323	18,25
TSPAN5	2,42558E-05	0,058647965	0,415	0,154	0,511822217	1,041489262	0,960163524	2,694805195
GDF15	0,065143806	0,058497213	0,024	0,004	1	1,041380439	0,96026386	6
RPUSD3	0,001638871	0,058465627	0,122	0,032	1	1,041357639	0,960284884	3,8125
ANKMY1	0,892889287	0,058457675	0,012	0,011	1	1,041351899	0,960290177	1,090909091
GTF2IRD1	0,208267874	0,058424353	0,098	0,056	1	1,041327848	0,960312357	1,75
TMEM68	0,000432775	0,058409823	0,11	0,021	1	1,04131736	0,960322029	5,238095238
ZFHX2	0,065143806	0,058295018	0,024	0,004	1	1,041234499	0,960398451	6
NOL9	0,005729257	0,058274341	0,122	0,039	1	1,041219576	0,960412216	3,128205128
DMWD	0,002048849	0,058272004	0,232	0,091	1	1,041217889	0,960413772	2,549450549
RNASEH2B-AS1	0,000183568	0,05820823	0,049	0	1	1,041171864	0,960456227	#DIV/0!
RAB15	0,000183568	0,058190731	0,049	0	1	1,041159234	0,960467877	#DIV/0!
LRRC14	0,016663237	0,058145356	0,061	0,014	1	1,041126489	0,960498086	4,357142857
GSKIP	0,001013666	0,058125293	0,134	0,035	1	1,041112011	0,960511443	3,828571429
MANEAL	0,001220269	0,05811781	0,037	0	1	1,041106611	0,960516425	#DIV/0!
GOLGA1	0,003743072	0,058078094	0,134	0,042	1	1,04107795	0,960542868	3,19047619
CDH24	0,025349996	0,058057116	0,049	0,011	1	1,041062813	0,960556835	4,454545455
SH3TC2	0,113968254	0,058048557	0,049	0,018	1	1,041056636	0,960562533	2,722222222
PRPF40B	0,00140181	0,058026163	0,073	0,011	1	1,041040476	0,960577444	6,636363636
MED15	0,000998569	0,058017638	0,524	0,249	1	1,041034325	0,96058312	2,104417671
SPA17	0,00022402	0,05795228	0,098	0,014	1	1,040987165	0,960626638	7
GATA3	0,065143806	0,057924446	0,024	0,004	1	1,040967081	0,960645172	6
LDAF1	0,003205568	0,057911506	0,122	0,035	1	1,040957744	0,960653788	3,485714286
NUDT21	0,000655292	0,05790767	0,28	0,109	1	1,040954976	0,960656342	2,568807339
ALK	0,00032444	0,057865787	0,061	0,004	1	1,040924756	0,960684232	15,25
CASC3	0,003065232	0,057821082	0,39	0,189	1	1,040892502	0,960714001	2,063492063
LRFN3	0,067584209	0,057815812	0,073	0,028	1	1,0408887	0,96071751	2,607142857
THOC3	2,33847E-05	0,057728705	0,171	0,035	0,493439878	1,040825855	0,960775518	4,885714286
WDR54	1,40243E-05	0,057715767	0,183	0,039	0,295927476	1,040816521	0,960784134	4,692307692
ZNF449	0,05889129	0,057694637	0,049	0,014	1	1,040801277	0,960798206	3,5

Supplementary Table 2

Differentially expressed genes_keloid-XL_ comparing SC-Keloid vs. (SC-Myel, SC-Nonmyel, SC-Nonmyel2)								
gene	p_val	avg_log2FC	pct.1	pct.2	p_val_adj	Foldchange_UP	Foldchange_DOWN	Ratio_pct1_pct2
PAX8	0,000183568	0,057679644	0,049	0	1	1,04079046	0,960808191	#DIV/0!
RAB28	0,000146815	0,057566913	0,171	0,042	1	1,040709137	0,960883271	4,071428571
AKR1C3	0,422305786	0,05753448	0,037	0,021	1	1,040685742	0,960904873	1,761904762
SDSL	8,66134E-06	0,057456121	0,085	0,004	0,1827629	1,040629219	0,960957065	21,25
TMEM104	0,000234367	0,0574417	0,098	0,014	1	1,040618817	0,960966671	7
DUSP12	1,08068E-05	0,057440302	0,22	0,053	0,228033983	1,040617809	0,960967602	4,150943396
IHO1	0,001220269	0,057435753	0,037	0	1	1,040614527	0,960970632	#DIV/0!
SMARCAL1	0,001041037	0,05741864	0,085	0,014	1	1,040602184	0,960982031	6,071428571
CSNK2A1	0,00042088	0,057407951	0,402	0,175	1	1,040594474	0,960989151	2,297142857
NME5	0,183494953	0,05734545	0,024	0,007	1	1,040549394	0,961030784	3,428571429
CLBA1	0,001802592	0,05728319	0,061	0,007	1	1,040504489	0,961072259	8,714285714
TMEM218	0,000175126	0,057250115	0,183	0,049	1	1,040480635	0,961094293	3,734693878
LCA5L	0,009031726	0,057150128	0,049	0,007	1	1,040408526	0,961160904	7
ZSCAN9	0,006163779	0,057033434	0,061	0,011	1	1,040324375	0,961238652	5,545454545
LAMC3	0,008401736	0,057030501	0,024	0	1	1,04032226	0,961240606	#DIV/0!
PPP1R26	0,041853847	0,056892743	0,085	0,032	1	1,040222929	0,961332396	2,65625
LINC01561	0,001220269	0,056868076	0,037	0	1	1,040205143	0,961348833	#DIV/0!
HMBS	0,001853689	0,056801571	0,098	0,021	1	1,040157193	0,961393149	4,666666667
LACTB2	0,002584599	0,056782467	0,11	0,028	1	1,040143419	0,96140588	3,928571429
PROCA1	0,066254792	0,056771908	0,024	0,004	1	1,040135807	0,961412917	6
LRRC20	0,00032444	0,056762824	0,061	0,004	1	1,040129257	0,961418971	15,25
PFN4	0,066254792	0,056731786	0,024	0,004	1	1,040106881	0,961439655	6
HDAC2	0,001919235	0,056629083	0,439	0,214	1	1,04003284	0,9615081	2,051401869
TBCCD1	0,000234367	0,056551886	0,098	0,014	1	1,03997719	0,961559551	7
RPL9	0,247038789	0,056519107	0,927	0,86	1	1,039953561	0,961581399	1,077906977
PPP2R5B	1,03455E-06	0,056441517	0,207	0,039	0,021830049	1,039897633	0,961633115	5,307692308
MYO1D	0,042398422	0,056437092	0,037	0,007	1	1,039894443	0,961636065	5,285714286
LMAN1	0,000453761	0,056419154	0,598	0,288	1	1,039881514	0,961648021	2,076388889
CCDC125	0,008401736	0,056389206	0,024	0	1	1,039859928	0,961667984	#DIV/0!
PPFIBP2	0,072365958	0,056291726	0,122	0,06	1	1,039789669	0,961732964	2,033333333

Supplementary Table 2

Differentially expressed genes_keloid-XL_ comparing SC-Keloid vs. (SC-Myel, SC-Nonmyel, SC-Nonmyel2)								
**gene**	**p_val**	**avg_log2FC**	**pct.1**	**pct.2**	**p_val_adj**	**Foldchange_UP**	**Foldchange_DOWN**	**Ratio_pct1_pct2**
PAIP2	0,000820553	0,05622242	0,585	0,284	1	1,03973972	0,961779166	2,059859155
FAM210A	0,025532849	0,056201013	0,098	0,035	1	1,039724291	0,961793437	2,8
USP7	0,000232722	0,056114717	0,354	0,137	1	1,039662101	0,961850969	2,583941606
MINDY4	0,001952737	0,056011847	0,049	0,004	1	1,039587972	0,961919555	12,25
DENR	0,000214813	0,055962984	0,293	0,109	1	1,039552762	0,961952136	2,688073394
B3GALT2	4,95841E-05	0,055868289	0,11	0,014	1	1,039484531	0,962015278	7,857142857
TSPAN6	0,000961052	0,055859442	0,171	0,053	1	1,039478157	0,962021177	3,226415094
FAM47E	0,001833348	0,055772953	0,061	0,007	1	1,039415842	0,962078852	8,714285714
SNAP47	0,000302387	0,055730396	0,146	0,035	1	1,039385181	0,962107232	4,171428571
KIRREL3-AS3	0,001220269	0,055726279	0,037	0	1	1,039382215	0,962109978	#DIV/0!
DHRS11	0,000174436	0,05572134	0,11	0,018	1	1,039378657	0,962113271	6,111111111
DIMT1	0,000614042	0,055668113	0,195	0,063	1	1,039340311	0,962148768	3,095238095
OSTF1	0,002758267	0,055591452	0,341	0,158	1	1,039285085	0,962199895	2,158227848
NCBP1	0,036803449	0,055536038	0,061	0,018	1	1,039245166	0,962236855	3,388888889
SPOUT1	0,001621701	0,055486221	0,122	0,032	1	1,039209281	0,962270082	3,8125
DENND6B	3,8435E-05	0,055473022	0,122	0,018	0,811016692	1,039199774	0,962278885	6,777777778
RSPH14	0,011553628	0,055323655	0,037	0,004	1	1,039092187	0,962378519	9,25
PPP1R13L	0,112963039	0,055295287	0,049	0,018	1	1,039071756	0,962397442	2,722222222
SACS	0,000863362	0,055275275	0,159	0,046	1	1,039057343	0,962410792	3,456521739
SNX19	0,000225277	0,055197323	0,134	0,028	1	1,039001202	0,962462794	4,785714286
TRPT1	3,1659E-06	0,055192546	0,28	0,074	0,06680361	1,038997761	0,962465981	3,783783784
LRP1B	0,043000486	0,055192094	0,037	0,007	1	1,038997436	0,962466282	5,285714286
KSR2	0,001952737	0,055169369	0,049	0,004	1	1,03898107	0,962481443	12,25
ZCWPW1	0,103792275	0,055165818	0,037	0,011	1	1,038978513	0,962483812	3,363636364
EGFL7	0,001026381	0,055112645	0,085	0,014	1	1,03894022	0,962519287	6,071428571
CUL5	0,001168951	0,055086664	0,195	0,067	1	1,03892151	0,962536621	2,910447761
STRN4	0,040113022	0,055072297	0,232	0,126	1	1,038911165	0,962546206	1,841269841
PRPF18	2,24871E-06	0,055069906	0,207	0,042	0,047450017	1,038909443	0,962547801	4,928571429
FGF10-AS1	0,008401736	0,054999492	0,024	0	1	1,038858737	0,962594782	#DIV/0!
P2RX6	0,065143806	0,054987829	0,024	0,004	1	1,038850339	0,962602564	6

Supplementary Table 2

Differentially expressed genes_keloid-XL_ comparing SC-Keloid vs. (SC-Myel, SC-Nonmyel, SC-Nonmyel2)								
**gene**	**p_val**	**avg_log2FC**	**pct.1**	**pct.2**	**p_val_adj**	**Foldchange_UP**	**Foldchange_DOWN**	**Ratio_pct1_pct2**
DCPS	4,27E-06	0,054926131	0,268	0,07	0,090101259	1,038805913	0,962643731	3,828571429
GLT8D2	0,063203483	0,054919165	0,012	0	1	1,038800897	0,962648379	#DIV/0!
VARS2	0,002755303	0,054854262	0,11	0,028	1	1,038754165	0,962691687	3,928571429
AAMP	9,03059E-05	0,054841052	0,317	0,112	1	1,038744654	0,962700502	2,830357143
CHCHD7	0,000606013	0,054830535	0,183	0,056	1	1,038737082	0,96270752	3,267857143
ZFP37	5,32686E-05	0,054819067	0,073	0,004	1	1,038728825	0,962715173	18,25
PHC1	0,003853665	0,054788358	0,207	0,084	1	1,038706715	0,962735665	2,464285714
PIK3R4	0,036427227	0,054765172	0,061	0,018	1	1,038690022	0,962751137	3,388888889
UROS	7,72204E-10	0,05474109	0,329	0,06	1,62943E-05	1,038672684	0,962767208	5,483333333
TIMM44	0,000715732	0,054714328	0,293	0,116	1	1,038653416	0,962785068	2,525862069
ZMYND15	0,347088417	0,054713515	0,012	0,004	1	1,038652831	0,96278561	3
TMEM169	4,32324E-06	0,054652371	0,073	0	0,09122464	1,038608812	0,962826416	#DIV/0!
AVEN	0,008065843	0,054623427	0,098	0,028	1	1,038587975	0,962845733	3,5
IPO13	2,74919E-05	0,054564657	0,22	0,056	0,580107244	1,038545668	0,962884956	3,928571429
SH2D3C	0,347088417	0,054485014	0,012	0,004	1	1,038488337	0,962938113	3
PKD1L1	0,008401736	0,054446437	0,024	0	1	1,038460569	0,962963862	#DIV/0!
IFT80	0,000302452	0,054392861	0,171	0,046	1	1,038422005	0,962999623	3,717391304
DNAJA3	0,000631225	0,054370083	0,146	0,039	1	1,03840561	0,963014828	3,743589744
PHKB	0,009669783	0,054358803	0,11	0,035	1	1,038397491	0,963022358	3,142857143
PPCDC	1,10381E-05	0,054272379	0,098	0,007	0,232915863	1,038335288	0,963080049	14
PRUNE1	0,035684609	0,054244659	0,061	0,018	1	1,038315338	0,963098553	3,388888889
ZC2HC1A	0,000998742	0,054097502	0,244	0,091	1	1,038209434	0,963196796	2,681318681
MORF4L1	0,027723624	0,054065296	0,841	0,677	1	1,038186257	0,963218298	1,242245199
SIN3B	0,010007568	0,05406241	0,195	0,084	1	1,038184181	0,963220225	2,321428571
SLC41A2	0,001041037	0,054049992	0,085	0,014	1	1,038175245	0,963228516	6,071428571
CCDC40	0,001220269	0,054034368	0,037	0	1	1,038164002	0,963238947	#DIV/0!
AGPAT3	0,000138689	0,054020901	0,207	0,06	1	1,038154311	0,963247939	3,45
CCDC47	0,00081557	0,054005234	0,561	0,263	1	1,038143037	0,963258399	2,133079848
SCAMP2	0,000575761	0,053833416	0,244	0,088	1	1,038019406	0,963373126	2,772727273
PITPNC1	0,261979564	0,053814521	0,049	0,025	1	1,038005812	0,963385743	1,96

Supplementary Table 2

Differentially expressed genes_keloid-XL_ comparing SC-Keloid vs. (SC-Myel, SC-Nonmyel, SC-Nonmyel2)								
**gene**	**p_val**	**avg_log2FC**	**pct.1**	**pct.2**	**p_val_adj**	**Foldchange_UP**	**Foldchange_DOWN**	**Ratio_pct1_pct2**
ZNF471	0,911002271	0,053742739	0,012	0,014	1	1,037954167	0,963433678	0,857142857
ZNF292	0,00315705	0,053710698	0,329	0,154	1	1,037931114	0,963455075	2,136363636
PTCD2	2,80682E-05	0,053691173	0,061	0	0,592266086	1,037917067	0,963468115	#DIV/0!
DDR2	4,05831E-05	0,053671223	0,366	0,133	0,856344048	1,037902715	0,963481438	2,751879699
TMEM50B	0,000150077	0,05361887	0,402	0,161	1	1,037865052	0,963516402	2,49689441
FBXL5	0,000853855	0,053600412	0,207	0,07	1	1,037851773	0,963528729	2,957142857
H2BC13	0,063203483	0,053589144	0,012	0	1	1,037843668	0,963536254	#DIV/0!
GALC	0,000472068	0,053558804	0,207	0,067	1	1,037821842	0,963556518	3,089552239
PCDH18	0,066254792	0,053553495	0,024	0,004	1	1,037818023	0,963560063	6
ZNF770	3,64035E-05	0,053533855	0,207	0,053	0,768150495	1,037803895	0,963573181	3,905660377
GPN3	0,000271022	0,05352675	0,232	0,077	1	1,037798784	0,963577926	3,012987013
ZNF30	0,342070304	0,053456863	0,024	0,011	1	1,037748512	0,963624605	2,181818182
MRTFB	0,000197116	0,053448845	0,134	0,028	1	1,037742745	0,963629961	4,785714286
SIRT5	0,016864604	0,053353694	0,061	0,014	1	1,037674304	0,963693518	4,357142857
TRNP1	1,25823E-05	0,053289272	0,256	0,07	0,265499884	1,037627969	0,963736551	3,657142857
RPE	0,000453449	0,053274656	0,171	0,049	1	1,037617456	0,963746315	3,489795918
ARHGDIG	0,911002271	0,053254281	0,012	0,014	1	1,037602802	0,963759926	0,857142857
OGFOD1	0,025535806	0,053243743	0,11	0,042	1	1,037595223	0,963766966	2,619047619
ANAPC1	0,042518051	0,053103544	0,11	0,046	1	1,037494396	0,963860628	2,391304348
PJA2	0,174519833	0,053081687	0,732	0,491	1	1,037478678	0,963875231	1,490835031
METTL6	0,112963039	0,053028431	0,049	0,018	1	1,037440381	0,963910812	2,722222222
TMEM254-AS1	0,011553628	0,052967804	0,037	0,004	1	1,037396785	0,963951319	9,25
ZNF782	0,025678378	0,052816539	0,049	0,011	1	1,037288021	0,964052394	4,454545455
TPRG1L	0,00381076	0,052813933	0,134	0,042	1	1,037286148	0,964054135	3,19047619
FAM153CP	0,0059909	0,052793614	0,061	0,011	1	1,037271538	0,964067713	5,545454545
MIGA1	0,054199044	0,052792363	0,195	0,105	1	1,037270638	0,96406855	1,857142857
ABHD4	0,025343006	0,052785739	0,11	0,042	1	1,037265876	0,964072976	2,619047619
FRA10AC1	0,004409658	0,052764147	0,195	0,077	1	1,037250352	0,964087404	2,532467532
ARHGAP29	0,50438121	0,052656304	0,073	0,053	1	1,03717282	0,964159474	1,377358491
FBXL18	0,000344207	0,052628385	0,073	0,007	1	1,037152748	0,964178132	10,42857143

Supplementary Table 2

Differentially expressed genes_keloid-XL_ comparing SC-Keloid vs. (SC-Myel, SC-Nonmyel, SC-Nonmyel2)								
**gene**	**p_val**	**avg_log2FC**	**pct.1**	**pct.2**	**p_val_adj**	**Foldchange_UP**	**Foldchange_DOWN**	**Ratio_pct1_pct2**
EIPR1	0,001222478	0,052625911	0,11	0,025	1	1,03715097	0,964179786	4,4
EHHADH	0,001220269	0,052589281	0,037	0	1	1,037124637	0,964204267	#DIV/0!
IFT81	0,000678439	0,052587673	0,146	0,039	1	1,037123481	0,964205342	3,743589744
IQCJ-SCHIP1	0,000770218	0,052582201	0,122	0,028	1	1,037119547	0,964208999	4,357142857
HOXC6	0,00620106	0,052581301	0,268	0,123	1	1,0371189	0,9642096	2,178861789
COLQ	0,644071107	0,052563638	0,012	0,007	1	1,037106203	0,964221405	1,714285714
B9D1	5,60121E-05	0,05255628	0,098	0,011	1	1,037100913	0,964226323	8,909090909
DUSP18	0,00032786	0,05251096	0,122	0,025	1	1,037068335	0,964256613	4,88
GK5	0,036365456	0,05247597	0,134	0,06	1	1,037043183	0,96428	2,233333333
PIGX	0,002075387	0,052460629	0,134	0,039	1	1,037032156	0,964290253	3,435897436
PBK	0,001220269	0,05239805	0,037	0	1	1,036987174	0,964332082	#DIV/0!
PLD2	0,000685144	0,052390664	0,098	0,018	1	1,036981865	0,964337019	5,444444444
MCRIP2	0,002502924	0,052349547	0,11	0,028	1	1,036952311	0,964364503	3,928571429
AMZ2	7,29081E-05	0,05231211	0,244	0,074	1	1,036925403	0,964389528	3,297297297
GDPD1	0,104939896	0,052309182	0,037	0,011	1	1,036923299	0,964391485	3,363636364
TMEM80	0,054965454	0,052165788	0,159	0,081	1	1,036820241	0,964487344	1,962962963
MTHFS	0,000140652	0,051970192	0,171	0,042	1	1,036679681	0,964618115	4,071428571
AFG1L	0,065143806	0,051926872	0,024	0,004	1	1,036648554	0,96464708	6
WDR83	0,006704008	0,051907699	0,085	0,021	1	1,036634777	0,9646599	4,047619048
LYPD6	0,001833348	0,05190249	0,061	0,007	1	1,036631034	0,964663383	8,714285714
NOB1	0,000967641	0,051883913	0,159	0,046	1	1,036617686	0,964675805	3,456521739
MOB2	0,005161375	0,051800376	0,354	0,175	1	1,036557664	0,964731664	2,022857143
KRT17	0,003643588	0,051756714	0,134	0,042	1	1,036526294	0,964760861	3,19047619
MRPS30	2,27964E-05	0,051663812	0,22	0,056	0,481026575	1,036459549	0,964822989	3,928571429
SYT1	0,000344207	0,051650073	0,073	0,007	1	1,036449679	0,964832177	10,42857143
MAD2L1	0,000344207	0,051631317	0,073	0,007	1	1,036436204	0,964844721	10,42857143
RAD17	0,00049803	0,05152928	0,134	0,032	1	1,036362903	0,964912964	4,1875
CARD6	0,001220269	0,051506133	0,037	0	1	1,036346276	0,964928445	#DIV/0!
VSTM2L	0,063203483	0,051429746	0,012	0	1	1,036291405	0,964979536	#DIV/0!
TM2D3	0,000508002	0,05130902	0,305	0,119	1	1,036204691	0,965060291	2,56302521

Supplementary Table 2

Differentially expressed genes_keloid-XL_ comparing SC-Keloid vs. (SC-Myel, SC-Nonmyel, SC-Nonmyel2)								
**gene**	**p_val**	**avg_log2FC**	**pct.1**	**pct.2**	**p_val_adj**	**Foldchange_UP**	**Foldchange_DOWN**	**Ratio_pct1_pct2**
PIP5KL1	0,001220269	0,051290788	0,037	0	1	1,036191596	0,965072487	#DIV/0!
ZNF317	0,000298361	0,051240971	0,085	0,011	1	1,036155817	0,965105811	7,727272727
ZNF302	0,000617042	0,051175551	0,183	0,056	1	1,036108832	0,965149576	3,267857143
RAC2	0,001220269	0,051175192	0,037	0	1	1,036108575	0,965149816	#DIV/0!
ZNF438	0,194890196	0,051169251	0,037	0,014	1	1,036104308	0,965153791	2,642857143
STX10	2,66046E-05	0,05114729	0,354	0,123	0,561384403	1,036088536	0,965168482	2,87804878
TOMM6	0,000499064	0,051056113	0,634	0,319	1	1,036023058	0,965229482	1,987460815
POLR1D	0,023921081	0,050988138	0,549	0,323	1	1,035974245	0,965274962	1,699690402
NOP2	0,000420675	0,050976099	0,159	0,042	1	1,0359656	0,965283017	3,785714286
MITD1	0,009992675	0,05094139	0,207	0,091	1	1,035940677	0,96530624	2,274725275
ZNF181	0,001338917	0,050897652	0,073	0,011	1	1,035909271	0,965335505	6,636363636
ANKRD13C	0,001351989	0,050867109	0,207	0,074	1	1,03588734	0,965355943	2,797297297
STX6	0,001085451	0,050839802	0,183	0,06	1	1,035867734	0,965374215	3,05
BDKRB2	0,008401736	0,050789637	0,024	0	1	1,035831716	0,965407783	#DIV/0!
LPAR6	0,008526144	0,050769665	0,11	0,035	1	1,035817376	0,965421148	3,142857143
USP28	0,017068103	0,050767927	0,061	0,014	1	1,035816128	0,965422311	4,357142857
FCF1	0,001647567	0,050728752	0,159	0,049	1	1,035788002	0,965448526	3,244897959
ZNF101	0,104364397	0,050603569	0,037	0,011	1	1,03569813	0,965532302	3,363636364
TNNI2	0,347088417	0,050574358	0,012	0,004	1	1,03567716	0,965551852	3
NT5M	0,183494953	0,050456817	0,024	0,007	1	1,035592784	0,965630522	3,428571429
PTPN9	0,010505641	0,050448441	0,171	0,07	1	1,035586771	0,965636128	2,442857143
RPS15	0,237450556	0,050443289	0,976	0,926	1	1,035583073	0,965639576	1,05399568
TOP2A	0,041803504	0,050430589	0,037	0,007	1	1,035573957	0,965648077	5,285714286
HOXD9	0,347088417	0,050389257	0,012	0,004	1	1,035544289	0,965675742	3
KBTBD3	0,24086264	0,050386238	0,061	0,032	1	1,035542121	0,965677764	1,90625
FAM41C	0,001220269	0,050364398	0,037	0	1	1,035526445	0,965692382	#DIV/0!
EMB	0,00032444	0,050317169	0,061	0,004	1	1,035492546	0,965723996	15,25
CLUAP1	6,01472E-05	0,050302238	0,171	0,039	1	1,03548183	0,965733991	4,384615385
CCDC102A	0,001876168	0,050200941	0,098	0,021	1	1,035409128	0,965801801	4,666666667
RWDD2B	0,000340322	0,050138526	0,122	0,025	1	1,035364333	0,965843585	4,88

Supplementary Table 2

Differentially expressed genes_keloid-XL_ comparing SC-Keloid vs. (SC-Myel, SC-Nonmyel, SC-Nonmyel2)								
**gene**	**p_val**	**avg_log2FC**	**pct.1**	**pct.2**	**p_val_adj**	**Foldchange_UP**	**Foldchange_DOWN**	**Ratio_pct1_pct2**
CCDC142	0,184958462	0,050130119	0,049	0,021	1	1,0353583	0,965849214	2,333333333
CDKN2AIPNL	0,015190866	0,050128812	0,098	0,032	1	1,035357362	0,965850089	3,0625
QSOX2	0,000288855	0,05010457	0,085	0,011	1	1,035339965	0,965866318	7,727272727
BOLA1	0,002177637	0,050087831	0,134	0,039	1	1,035327953	0,965877525	3,435897436
MKI67	0,043000486	0,050060463	0,037	0,007	1	1,035308313	0,965895847	5,285714286
LLPH	3,35418E-06	0,04994458	0,293	0,081	0,070776525	1,035225156	0,965973435	3,617283951
GABPB1	0,039738699	0,049821203	0,073	0,025	1	1,035136629	0,966056047	2,92
ERVMER34-1	4,32324E-06	0,049753402	0,073	0	0,09122464	1,035087982	0,966101449	#DIV/0!
VPS41	9,09477E-05	0,049710297	0,207	0,056	1	1,035057057	0,966130315	3,696428571
SELENBP1	0,001220269	0,04969998	0,037	0	1	1,035049655	0,966137224	#DIV/0!
CDYL2	0,181340493	0,049698925	0,024	0,007	1	1,035048898	0,96613793	3,428571429
SMYD5	0,017068103	0,049691856	0,061	0,014	1	1,035043826	0,966142665	4,357142857
PIGC	1,31736E-08	0,049654207	0,378	0,091	0,000277975	1,035016815	0,966167878	4,153846154
ABCC1	0,564675598	0,049625617	0,037	0,025	1	1,034996305	0,966187025	1,48
SRD5A3	0,010916043	0,049598528	0,073	0,018	1	1,034976871	0,966205166	4,055555556
SEC31B	0,00931978	0,049594478	0,049	0,007	1	1,034973966	0,966207879	7
TFAP2B	0,010268947	0,049577058	0,134	0,049	1	1,034961469	0,966219545	2,734693878
TRIM25	0,003497178	0,049544877	0,122	0,035	1	1,034938383	0,966241098	3,485714286
GBA2	0,014910424	0,049543681	0,159	0,067	1	1,034937525	0,966241899	2,373134328
ZNF45	0,001914332	0,049393164	0,049	0,004	1	1,034829556	0,966342713	12,25
METTL22	0,00032444	0,049366867	0,061	0,004	1	1,034810693	0,966360327	15,25
ZNF382	0,043000486	0,049363381	0,037	0,007	1	1,034808192	0,966362663	5,285714286
MYNN	0,097309365	0,049268793	0,073	0,032	1	1,034740349	0,966426022	2,28125
HIF1AN	0,000723012	0,049240048	0,098	0,018	1	1,034719733	0,966445278	5,444444444
HPS6	0,011553628	0,049226023	0,037	0,004	1	1,034709674	0,966454674	9,25
NUBPL	0,027028964	0,049222518	0,049	0,011	1	1,03470716	0,966457021	4,454545455
SLCO2A1	0,064048232	0,049172583	0,024	0,004	1	1,034671347	0,966490473	6
CENPM	0,065143806	0,049141847	0,024	0,004	1	1,034649304	0,966511064	6
GOLGA8R	0,008401736	0,049127892	0,024	0	1	1,034639296	0,966520413	#DIV/0!
NTF3	0,028081741	0,049092536	0,049	0,011	1	1,034613941	0,9665441	4,454545455

Supplementary Table 2

Differentially expressed genes_keloid-XL_ comparing SC-Keloid vs. (SC-Myel, SC-Nonmyel, SC-Nonmyel2)								
gene	p_val	avg_log2FC	pct.1	pct.2	p_val_adj	Foldchange_UP	Foldchange_DOWN	Ratio_pct1_pct2
LBX2	0,00931978	0,049087082	0,049	0,007	1	1,034610029	0,966547754	7
ACSF3	0,001180297	0,049085391	0,11	0,025	1	1,034608817	0,966548886	4,4
SPRYD7	0,000230242	0,049085231	0,207	0,063	1	1,034608702	0,966548994	3,285714286
SEC61A2	0,010425992	0,049071967	0,073	0,018	1	1,03459919	0,96655788	4,055555556
ARMC1	0,000496956	0,049063717	0,207	0,067	1	1,034593273	0,966563408	3,089552239
APBA3	2,51588E-05	0,049026964	0,134	0,021	0,530876209	1,034566917	0,966588031	6,380952381
RAB29	0,06642434	0,049016623	0,061	0,021	1	1,034559502	0,966594959	2,904761905
LINC01137	0,000183568	0,049014199	0,049	0	1	1,034557764	0,966596584	#DIV/0!
TRIOBP	0,002173845	0,0489616	0,293	0,13	1	1,034520045	0,966631826	2,253846154
PPT2	3,29775E-05	0,048957337	0,268	0,081	0,695859044	1,034516988	0,966634682	3,308641975
ALG9	0,003927556	0,048938222	0,098	0,025	1	1,034503282	0,966647489	3,92
TNFSF13B	0,008401736	0,048928981	0,024	0	1	1,034496655	0,966653681	#DIV/0!
BVES-AS1	0,001220269	0,048917019	0,037	0	1	1,034488078	0,966661696	#DIV/0!
NPRL3	0,000322749	0,048914904	0,183	0,053	1	1,034486561	0,966663113	3,452830189
BRD9	5,31671E-05	0,048852701	0,317	0,109	1	1,03444196	0,966704792	2,908256881
TAFA2	0,347088417	0,04885063	0,012	0,004	1	1,034440475	0,96670618	3
RCC1L	4,22736E-05	0,048809199	0,183	0,042	0,892014976	1,034410768	0,966733942	4,357142857
C1RL	0,013704854	0,048803365	0,085	0,025	1	1,034406585	0,966737852	3,4
RASSF8	0,000282912	0,048730541	0,524	0,239	1	1,034354372	0,966786651	2,192468619
ZNF790	0,006615795	0,048720183	0,061	0,011	1	1,034346946	0,966793593	5,545454545
PELP1	0,003026608	0,048610976	0,183	0,067	1	1,034268653	0,966866778	2,731343284
PRICKLE3	0,012780645	0,048561516	0,085	0,025	1	1,034233195	0,966899926	3,4
RRP12	0,008145887	0,048517384	0,098	0,028	1	1,034201558	0,966929504	3,5
USP31	0,686285647	0,048511524	0,037	0,028	1	1,034197358	0,966933432	1,321428571
SLC39A11	0,179204865	0,048482892	0,024	0,007	1	1,034176833	0,966952622	3,428571429
LPP	2,8894E-05	0,048395387	0,524	0,214	0,609691488	1,034114109	0,967011273	2,448598131
CAPN10	0,00020149	0,048369528	0,159	0,039	1	1,034095573	0,967028606	4,076923077
LINC00839	0,000169546	0,048358592	0,11	0,018	1	1,034087734	0,967035936	6,111111111
C2CD2L	0,004368707	0,04834472	0,073	0,014	1	1,034077791	0,967045235	5,214285714
DTD1	3,32776E-06	0,048299182	0,39	0,123	0,070219133	1,034045151	0,967075759	3,170731707

Supplementary Table 2

Differentially expressed genes_keloid-XL_ comparing SC-Keloid vs. (SC-Myel, SC-Nonmyel, SC-Nonmyel2)								
**gene**	**p_val**	**avg_log2FC**	**pct.1**	**pct.2**	**p_val_adj**	**Foldchange_UP**	**Foldchange_DOWN**	**Ratio_pct1_pct2**
CDC42SE1	0,001448225	0,048294124	0,476	0,235	1	1,034041526	0,96707915	2,025531915
USP8	0,004876141	0,048266042	0,293	0,133	1	1,034021399	0,967097974	2,203007519
GREB1	0,106097567	0,048249884	0,037	0,011	1	1,034009818	0,967108806	3,363636364
ZNF23	0,000230869	0,048209095	0,098	0,014	1	1,033980584	0,967136149	7
FAAP100	0,001099251	0,048202706	0,171	0,053	1	1,033976005	0,967140432	3,226415094
NDUFS7	0,000139668	0,048138658	0,488	0,204	1	1,033930103	0,967183369	2,392156863
KIF13B	0,000143959	0,048114925	0,427	0,172	1	1,033913094	0,967199279	2,48255814
ABCF3	0,000713367	0,047861432	0,098	0,018	1	1,033731444	0,967369239	5,444444444
RPP25L	0,00380062	0,04783273	0,098	0,025	1	1,033710878	0,967388485	3,92
ZNF823	0,011553628	0,047826593	0,037	0,004	1	1,033706481	0,9673926	9,25
GNB1L	0,006062183	0,047818434	0,085	0,021	1	1,033700635	0,967398071	4,047619048
ZNF174	0,001318539	0,047790494	0,073	0,011	1	1,033680615	0,967416807	6,636363636
SURF6	1,79815E-05	0,047765458	0,207	0,049	0,379426863	1,033662678	0,967433594	4,224489796
ZNF749	4,65444E-05	0,047517477	0,11	0,014	0,9821336	1,03348502	0,967599898	7,857142857
RPL36AL	0,052472184	0,047486986	0,841	0,596	1	1,033463178	0,967620348	1,411073826
TOPBP1	9,07324E-05	0,047411045	0,159	0,035	1	1,033408779	0,967671284	4,542857143
ST7	0,026685693	0,047352866	0,049	0,011	1	1,033367106	0,967710307	4,454545455
NHLRC2	0,002512624	0,04733999	0,159	0,053	1	1,033357883	0,967718944	3
HRH1	0,001802592	0,047300001	0,061	0,007	1	1,033329241	0,967745768	8,714285714
GRPEL2	0,43516567	0,047294906	0,037	0,021	1	1,033325591	0,967749186	1,761904762
DLGAP5	0,347088417	0,047290972	0,012	0,004	1	1,033322774	0,967751825	3
TMEM217	2,80682E-05	0,047284951	0,061	0	0,592266086	1,033318461	0,967755864	#DIV/0!
C2orf81	0,000983548	0,047205057	0,085	0,014	1	1,03326124	0,967809458	6,071428571
ALKBH2	9,95727E-05	0,047179735	0,159	0,035	1	1,033243104	0,967826445	4,542857143
SMAD4	0,043184756	0,047153523	0,378	0,225	1	1,033224332	0,967844029	1,68
OGN	0,691844906	0,047140927	0,024	0,018	1	1,03321531	0,967852479	1,333333333
DDX42	0,001047991	0,047120097	0,354	0,151	1	1,033200393	0,967866454	2,344370861
LRFN4	1,7171E-06	0,047078339	0,28	0,07	0,036232561	1,033170488	0,967894468	4
ZNF85	5,32686E-05	0,047044991	0,073	0,004	1	1,033146606	0,967916841	18,25
PLA2G15	0,002726155	0,047033113	0,11	0,028	1	1,0331381	0,967924811	3,928571429

Supplementary Table 2

Differentially expressed genes_keloid-XL_ comparing SC-Keloid vs. (SC-Myel, SC-Nonmyel, SC-Nonmyel2)								
**gene**	**p_val**	**avg_log2FC**	**pct.1**	**pct.2**	**p_val_adj**	**Foldchange_UP**	**Foldchange_DOWN**	**Ratio_pct1_pct2**
PDCD7	0,004945257	0,046923474	0,256	0,112	1	1,033059589	0,967998372	2,285714286
ZBED5-AS1	0,024758179	0,04691703	0,085	0,028	1	1,033054975	0,968002695	3,035714286
C2orf72	0,066254792	0,046807232	0,024	0,004	1	1,032976355	0,96807637	6
CYP2R1	0,001423372	0,046787839	0,073	0,011	1	1,032962471	0,968089382	6,636363636
CENPP	0,001952737	0,046751059	0,049	0,004	1	1,032936136	0,968114064	12,25
ACOT4	0,043000486	0,046748325	0,037	0,007	1	1,032934179	0,968115898	5,285714286
IKBKE	5,32686E-05	0,046723659	0,073	0,004	1	1,032916519	0,96813245	18,25
C20orf96	0,001723931	0,046704752	0,098	0,021	1	1,032902982	0,968145138	4,666666667
FERMT3	0,010916043	0,046682944	0,073	0,018	1	1,032887368	0,968159773	4,055555556
GPR137	0,002585356	0,046682521	0,183	0,067	1	1,032887066	0,968160057	2,731343284
CYB5B	0,000232459	0,046667977	0,305	0,116	1	1,032876654	0,968169816	2,629310345
B3GLCT	0,001898897	0,046618692	0,098	0,021	1	1,032841369	0,968202892	4,666666667
ZNF48	0,017068103	0,046590289	0,061	0,014	1	1,032821035	0,968221953	4,357142857
NOL3	0,000723012	0,046587558	0,098	0,018	1	1,03281908	0,968223786	5,444444444
BRF2	0,000338108	0,046574241	0,073	0,007	1	1,032809547	0,968232723	10,42857143
GBE1	0,000871637	0,046554372	0,159	0,046	1	1,032795323	0,968246058	3,456521739
SLC25A15	0,00032444	0,046541808	0,061	0,004	1	1,032786328	0,968254491	15,25
MIEF2	0,043609761	0,04649121	0,037	0,007	1	1,032750107	0,968288449	5,285714286
MYLPF	0,001952737	0,046479014	0,049	0,004	1	1,032741377	0,968296635	12,25
PRMT6	2,80682E-05	0,046415238	0,061	0	0,592266086	1,032695724	0,968339441	#DIV/0!
KCNH1	0,001220269	0,046391602	0,037	0	1	1,032678806	0,968355305	#DIV/0!
EIF1AD	0,000234367	0,046375017	0,098	0,014	1	1,032666934	0,968366437	7
WBP11	0,001717164	0,046232738	0,451	0,218	1	1,032565097	0,968461942	2,068807339
NOSIP	4,34026E-05	0,046202504	0,293	0,095	0,915839002	1,032543458	0,968482239	3,084210526
CDC27	7,13199E-06	0,046194992	0,341	0,109	0,150492048	1,032538082	0,968487282	3,128440367
ZNF160	0,138914913	0,046158892	0,085	0,042	1	1,032512246	0,968511516	2,023809524
LRBA	0,007608639	0,046082606	0,159	0,06	1	1,032457651	0,968562729	2,65
KCNK6	0,008401736	0,046070715	0,024	0	1	1,032449141	0,968570713	#DIV/0!
SAMD13	0,008401736	0,046070715	0,024	0	1	1,032449141	0,968570713	#DIV/0!
RRS1	0,000713367	0,046063995	0,098	0,018	1	1,032444332	0,968575224	5,444444444

Supplementary Table 2

Differentially expressed genes_keloid-XL_ comparing SC-Keloid vs. (SC-Myel, SC-Nonmyel, SC-Nonmyel2)								
**gene**	**p_val**	**avg_log2FC**	**pct.1**	**pct.2**	**p_val_adj**	**Foldchange_UP**	**Foldchange_DOWN**	**Ratio_pct1_pct2**
LINC01060	0,00032444	0,046047838	0,061	0,004	1	1,032432769	0,968586072	15,25
ELAC1	0,010668516	0,045957302	0,073	0,018	1	1,032367981	0,968646857	4,055555556
NFYC-AS1	0,008401736	0,045925197	0,024	0	1	1,032345008	0,968668413	#DIV/0!
TMCO4	0,002502924	0,045924921	0,11	0,028	1	1,03234481	0,968668598	3,928571429
COG4	3,9586E-05	0,045865885	0,207	0,053	0,835303213	1,032302567	0,968708237	3,905660377
GGT7	0,025838473	0,045825533	0,122	0,049	1	1,032273694	0,968735332	2,489795918
AP000462.3	0,342070304	0,045779201	0,024	0,011	1	1,032240543	0,968766444	2,181818182
DMAP1	0,000914715	0,045727363	0,244	0,091	1	1,032203454	0,968801253	2,681318681
SNX25	0,00931978	0,045691147	0,049	0,007	1	1,032177543	0,968825574	7
GTF2H3	3,2263E-07	0,045685894	0,22	0,039	0,00680781	1,032173785	0,968829101	5,641025641
MPST	0,003048205	0,045667382	0,268	0,116	1	1,03216054	0,968841533	2,310344828
UNG	0,000694434	0,04563873	0,098	0,018	1	1,032140042	0,968860774	5,444444444
CHKB-DT	0,001220269	0,045636393	0,037	0	1	1,03213837	0,968862344	#DIV/0!
TMTC3	0,023592192	0,045632721	0,146	0,063	1	1,032135743	0,96886481	2,317460317
IPO9	0,001919241	0,045595967	0,195	0,07	1	1,032109449	0,968889493	2,785714286
PRR7	0,001739762	0,045591544	0,171	0,056	1	1,032106284	0,968892463	3,053571429
ARHGAP31	0,054343153	0,045589224	0,122	0,056	1	1,032104624	0,968894021	2,178571429
RPS20	0,432409985	0,045576327	0,927	0,765	1	1,032095398	0,968902683	1,211764706
ALDH3B2	0,063203483	0,045560371	0,012	0	1	1,032083983	0,968913399	#DIV/0!
DPYS	0,063203483	0,045560371	0,012	0	1	1,032083983	0,968913399	#DIV/0!
DSC2	0,063203483	0,045560371	0,012	0	1	1,032083983	0,968913399	#DIV/0!
GPR21	0,063203483	0,045560371	0,012	0	1	1,032083983	0,968913399	#DIV/0!
RTN4RL1	0,063203483	0,045560371	0,012	0	1	1,032083983	0,968913399	#DIV/0!
WNK4	0,063203483	0,045560371	0,012	0	1	1,032083983	0,968913399	#DIV/0!
CYP3A5	0,065143806	0,045549533	0,024	0,004	1	1,03207623	0,968920678	6
CCDC30	0,010668516	0,0455225	0,073	0,018	1	1,032056891	0,968938833	4,055555556
LRR1	0,000969643	0,045497182	0,085	0,014	1	1,03203878	0,968955838	6,071428571
B9D2	0,006431528	0,04549432	0,061	0,011	1	1,032036732	0,96895776	5,545454545
ZNF573	0,036427227	0,045437181	0,061	0,018	1	1,031995858	0,968996137	3,388888889
BTBD2	0,003073868	0,045389561	0,232	0,095	1	1,031961795	0,969028122	2,442105263

Supplementary Table 2

Differentially expressed genes_keloid-XL_ comparing SC-Keloid vs. (SC-Myel, SC-Nonmyel, SC-Nonmyel2)								
gene	p_val	avg_log2FC	pct.1	pct.2	p_val_adj	Foldchange_UP	Foldchange_DOWN	Ratio_pct1_pct2
CHTF18	0,068827069	0,045367855	0,061	0,021	1	1,031946269	0,969042701	2,904761905
TMEM25	0,644071107	0,045357852	0,012	0,007	1	1,031939114	0,96904942	1,714285714
MED14	0,011360658	0,045333977	0,207	0,095	1	1,031922037	0,969065457	2,178947368
CNPY3	1,09513E-05	0,04529415	0,256	0,07	0,231083882	1,03189355	0,969092209	3,657142857
PTPN1	0,008280952	0,045267838	0,256	0,119	1	1,03187473	0,969109884	2,151260504
GOLPH3	0,037649564	0,045252617	0,28	0,158	1	1,031863844	0,969120108	1,772151899
FAM124A	0,347088417	0,045124732	0,012	0,004	1	1,03177238	0,969206018	3
C11orf80	0,004426608	0,045113715	0,098	0,025	1	1,031764501	0,969213419	3,92
UBIAD1	0,003949482	0,045096369	0,134	0,042	1	1,031752096	0,969225072	3,19047619
RRAS2	1,10008E-06	0,045028486	0,171	0,025	0,023212815	1,03170355	0,969270679	6,84
PLCL1	0,649511996	0,044989646	0,012	0,007	1	1,031675775	0,969296773	1,714285714
PRSS36	0,339089703	0,044953613	0,024	0,011	1	1,031650008	0,969320983	2,181818182
CLN5	0,001118483	0,04481277	0,378	0,165	1	1,031549298	0,969415618	2,290909091
ZMYND19	1,38478E-06	0,04477582	0,159	0,021	0,029220202	1,031522879	0,969440446	7,571428571
ESF1	4,60262E-08	0,044725596	0,451	0,133	0,000971199	1,031486969	0,969474196	3,390977444
GRB7	0,179204865	0,044694465	0,024	0,007	1	1,031464712	0,969495115	3,428571429
RMI2	0,001220269	0,044693377	0,037	0	1	1,031463934	0,969495847	#DIV/0!
FAM102B	0,002485806	0,044687926	0,146	0,046	1	1,031460037	0,96949951	3,173913043
XYLB	0,008401736	0,04465032	0,024	0	1	1,031433151	0,969524782	#DIV/0!
DYNC1I2	0,001361616	0,04464829	0,646	0,358	1	1,0314317	0,969526146	1,804469274
UBE2Q1	0,000136423	0,044632493	0,28	0,098	1	1,031420406	0,969536762	2,857142857
DDI2	0,014940574	0,044618356	0,11	0,039	1	1,031410299	0,969546262	2,820512821
KRBOX4	0,023887799	0,044615158	0,085	0,028	1	1,031408012	0,969548412	3,035714286
DNMBP	0,106083716	0,044569505	0,061	0,025	1	1,031375375	0,969579093	2,44
ASB7	0,113968254	0,044543638	0,049	0,018	1	1,031356883	0,969596477	2,722222222
ABHD17C	0,001713147	0,044518573	0,061	0,007	1	1,031338964	0,969613323	8,714285714
UBE3B	0,00020948	0,044514091	0,134	0,028	1	1,03133576	0,969616335	4,785714286
TBC1D10A	3,09476E-05	0,044508586	0,268	0,081	0,653024352	1,031331825	0,969620035	3,308641975
PDCD1LG2	0,000183568	0,044383871	0,049	0	1	1,031242675	0,969703858	#DIV/0!
LRRC49	0,009031726	0,044376653	0,049	0,007	1	1,031237515	0,969708709	7

Supplementary Table 2

Differentially expressed genes_keloid-XL_ comparing SC-Keloid vs. (SC-Myel, SC-Nonmyel, SC-Nonmyel2)								
**gene**	**p_val**	**avg_log2FC**	**pct.1**	**pct.2**	**p_val_adj**	**Foldchange_UP**	**Foldchange_DOWN**	**Ratio_pct1_pct2**
CASC2	0,063203483	0,044343083	0,012	0	1	1,031213519	0,969731274	#DIV/0!
CCDC181	0,011553628	0,044300354	0,037	0,004	1	1,031182978	0,969759995	9,25
MTRES1	0,001041037	0,0442986	0,085	0,014	1	1,031181725	0,969761174	6,071428571
IRAK4	0,003974717	0,044205796	0,146	0,049	1	1,031115394	0,969823558	2,979591837
ZNF565	0,689228839	0,044205733	0,037	0,028	1	1,031115349	0,969823601	1,321428571
TTC5	0,025428865	0,044187331	0,085	0,028	1	1,031102197	0,969835971	3,035714286
SNURF	0,000183568	0,044177346	0,049	0	1	1,03109506	0,969842683	#DIV/0!
THAP7-AS1	0,006629795	0,044173769	0,085	0,021	1	1,031092504	0,969845088	4,047619048
NIF3L1	5,89403E-05	0,04414444	0,098	0,011	1	1,031071543	0,969864804	8,909090909
RPRD1B	4,08678E-05	0,044131349	0,183	0,042	0,862351563	1,031062187	0,969873605	4,357142857
IRX3	0,183494953	0,044121658	0,024	0,007	1	1,031055261	0,96988012	3,428571429
ANO5	0,063203483	0,044070927	0,012	0	1	1,031019005	0,969914225	#DIV/0!
PAQR4	0,001949134	0,044047765	0,195	0,07	1	1,031002453	0,969929797	2,785714286
THEMIS2	0,000183568	0,044042316	0,049	0	1	1,030998559	0,969933461	#DIV/0!
CPPED1	0,001314487	0,044034683	0,22	0,081	1	1,030993104	0,969938592	2,716049383
MFSD3	3,56336E-06	0,044028206	0,171	0,028	0,075190555	1,030988475	0,969942947	6,107142857
NIP7	0,000227074	0,044025321	0,159	0,039	1	1,030986414	0,969944886	4,076923077
CDON	0,180829015	0,043971415	0,049	0,021	1	1,030947892	0,969981129	2,333333333
DDX60	0,001921879	0,043831246	0,098	0,021	1	1,030847732	0,970075375	4,666666667
SLC37A4	0,001055888	0,043829073	0,085	0,014	1	1,030846179	0,970076836	6,071428571
EBPL	0,000819022	0,043783099	0,28	0,109	1	1,03081333	0,970107749	2,568807339
GNB4	0,000122962	0,043760124	0,268	0,088	1	1,030796915	0,970123198	3,045454545
KIF2A	0,033649462	0,043685314	0,256	0,137	1	1,030743465	0,970173505	1,868613139
RCBTB1	0,026685693	0,043679555	0,049	0,011	1	1,03073935	0,970177378	4,454545455
FAM89A	0,00685582	0,043644773	0,159	0,06	1	1,030714501	0,970200768	2,65
PRKAG2	0,998241088	0,043644284	0,024	0,025	1	1,030714151	0,970201097	0,96
CROCC	0,001256896	0,043600364	0,159	0,049	1	1,030682774	0,970230633	3,244897959
OGFOD2	0,001833348	0,04355317	0,061	0,007	1	1,030649058	0,970262373	8,714285714
LSM11	0,065143806	0,043528857	0,024	0,004	1	1,030631689	0,970278724	6
ZNHIT6	0,145204976	0,043452259	0,11	0,06	1	1,03057697	0,970330241	1,833333333

Supplementary Table 2

Differentially expressed genes_keloid-XL_ comparing SC-Keloid vs. (SC-Myel, SC-Nonmyel, SC-Nonmyel2)								
**gene**	**p_val**	**avg_log2FC**	**pct.1**	**pct.2**	**p_val_adj**	**Foldchange_UP**	**Foldchange_DOWN**	**Ratio_pct1_pct2**
EXOC3-AS1	1,10381E-05	0,04343251	0,098	0,007	0,232915863	1,030562863	0,970343524	14
RRP1B	0,001124536	0,043412573	0,134	0,035	1	1,030548621	0,970356934	3,828571429
ALPK3	0,00931978	0,043381275	0,049	0,007	1	1,030526264	0,970377985	7
XXYLT1	0,000212038	0,043362689	0,134	0,028	1	1,030512989	0,970390486	4,785714286
KRBA1	0,024758179	0,043349048	0,085	0,028	1	1,030503245	0,970399661	3,035714286
PGS1	0,402953631	0,043317012	0,061	0,039	1	1,030480363	0,97042121	1,564102564
SAP30BP	0,000260089	0,043281404	0,476	0,214	1	1,030454929	0,970445162	2,224299065
DLGAP1-AS2	0,022420212	0,043184062	0,073	0,021	1	1,030385404	0,970510642	3,476190476
OLFML3	0,004426586	0,043180153	0,073	0,014	1	1,030382612	0,970513272	5,214285714
PPIF	0,001853678	0,043155185	0,098	0,021	1	1,03036478	0,970530068	4,666666667
SUSD1	0,00931978	0,043090047	0,049	0,007	1	1,030318259	0,970573889	7
TTC38	0,007676086	0,043038022	0,098	0,028	1	1,030281106	0,970608889	3,5
PLPBP	0,055866845	0,043036878	0,11	0,049	1	1,030280289	0,970609659	2,244897959
RIPK1	0,001045816	0,04301766	0,134	0,035	1	1,030266565	0,970622589	3,828571429
NBPF20	0,011553628	0,042999874	0,037	0,004	1	1,030253864	0,970634554	9,25
AGL	0,064483381	0,042996357	0,085	0,035	1	1,030251352	0,970636921	2,428571429
TMEM167B	1,87827E-05	0,042957008	0,232	0,06	0,396334767	1,030223252	0,970663395	3,866666667
ADGRG6	0,000453615	0,042939833	0,366	0,154	1	1,030210988	0,970674951	2,376623377
NQO1	0,166583455	0,04291708	0,061	0,028	1	1,030194741	0,970690259	2,178571429
THNSL2	0,208798809	0,042886516	0,073	0,039	1	1,030172916	0,970710824	1,871794872
ITPRIPL1	0,043609761	0,042874875	0,037	0,007	1	1,030164604	0,970718657	5,285714286
ZFP69	0,011553628	0,042855853	0,037	0,004	1	1,030151021	0,970731456	9,25
SLC2A6	0,001220269	0,042849233	0,037	0	1	1,030146294	0,97073591	#DIV/0!
ARL5A	0,002174808	0,042829999	0,427	0,207	1	1,03013256	0,970748852	2,062801932
SRD5A1	5,89403E-05	0,042826259	0,098	0,011	1	1,03012989	0,970751368	8,909090909
ABITRAM	0,000162451	0,042793996	0,11	0,018	1	1,030106853	0,970773077	6,111111111
USP40	0,000513745	0,042766187	0,171	0,049	1	1,030086997	0,97079179	3,489795918
MECOM	0,347088417	0,042749949	0,012	0,004	1	1,030075404	0,970802717	3
CREG1	0,001013666	0,042684627	0,134	0,035	1	1,030028765	0,970846674	3,828571429
ZNF75D	0,017068103	0,042676329	0,061	0,014	1	1,030022841	0,970852257	4,357142857

Supplementary Table 2

Differentially expressed genes_keloid-XL_ comparing SC-Keloid vs. (SC-Myel, SC-Nonmyel, SC-Nonmyel2)								
**gene**	**p_val**	**avg_log2FC**	**pct.1**	**pct.2**	**p_val_adj**	**Foldchange_UP**	**Foldchange_DOWN**	**Ratio_pct1_pct2**
MTAP	5,5269E-05	0,042628319	0,22	0,06	1	1,029988564	0,970884566	3,666666667
ANKRD27	0,168856267	0,042622856	0,061	0,028	1	1,029984664	0,970888243	2,178571429
ZSCAN31	0,193178656	0,04262085	0,037	0,014	1	1,029983232	0,970889592	2,642857143
PARP2	0,023439606	0,042611058	0,146	0,063	1	1,029976241	0,970896182	2,317460317
NPFF	0,02737604	0,042608001	0,049	0,011	1	1,029974059	0,970898239	4,454545455
ZNF107	0,001380549	0,042586761	0,073	0,011	1	1,029958894	0,970912534	6,636363636
CACNA2D4	3,24139E-07	0,042570644	0,159	0,018	0,006839666	1,029947389	0,97092338	8,833333333
MT-CYB	0,3565742	0,042504699	0,841	0,846	1	1,029900311	0,970967762	0,994089835
LINC00345	0,008401736	0,042504685	0,024	0	1	1,029900301	0,970967771	#DIV/0!
INAVA	0,008401736	0,042504003	0,024	0	1	1,029899815	0,97096823	#DIV/0!
BACE1	0,000252448	0,04249817	0,268	0,095	1	1,02989565	0,970972156	2,821052632
KPTN	0,016864604	0,042454004	0,061	0,014	1	1,029864122	0,971001881	4,357142857
NUP133	0,068219791	0,042429808	0,061	0,021	1	1,02984685	0,971018167	2,904761905
ABCD1	0,002909566	0,042395843	0,085	0,018	1	1,029822605	0,971041027	4,722222222
TEX264	0,000602812	0,042365431	0,256	0,095	1	1,029800896	0,971061497	2,694736842
C5orf63	5,32686E-05	0,042326006	0,073	0,004	1	1,029772755	0,971088034	18,25
USO1	6,48505E-05	0,042308833	0,329	0,116	1	1,029760497	0,971099593	2,836206897
STK38	0,017038619	0,042292435	0,122	0,046	1	1,029748793	0,971110631	2,652173913
AC003102.1	0,825514901	0,0422256	0,037	0,032	1	1,029701089	0,97115562	1,15625
CHD1L	0,000288855	0,042202897	0,085	0,011	1	1,029684885	0,971170903	7,727272727
TIMM50	8,33671E-07	0,042181332	0,268	0,063	0,017591299	1,029669494	0,97118542	4,253968254
FBXW8	0,001445239	0,042087408	0,073	0,011	1	1,029602462	0,971248649	6,636363636
ASPHD2	0,000183568	0,042077391	0,049	0	1	1,029595313	0,971255393	#DIV/0!
CCDC138	0,011553628	0,041977107	0,037	0,004	1	1,029523747	0,971322909	9,25
NAB2	0,000322775	0,041963885	0,293	0,109	1	1,029514311	0,971331811	2,688073394
HECTD3	0,185668317	0,041928701	0,024	0,007	1	1,029489204	0,971355499	3,428571429
C6orf47-AS1	0,063203483	0,041923236	0,012	0	1	1,029485305	0,971359179	#DIV/0!
CDK15	0,063203483	0,041923236	0,012	0	1	1,029485305	0,971359179	#DIV/0!
CFAP52	0,063203483	0,041923236	0,012	0	1	1,029485305	0,971359179	#DIV/0!
KCTD8	0,063203483	0,041923236	0,012	0	1	1,029485305	0,971359179	#DIV/0!

Supplementary Table 2

Differentially expressed genes_keloid-XL_ comparing SC-Keloid vs. (SC-Myel, SC-Nonmyel, SC-Nonmyel2)								
**gene**	**p_val**	**avg_log2FC**	**pct.1**	**pct.2**	**p_val_adj**	**Foldchange_UP**	**Foldchange_DOWN**	**Ratio_pct1_pct2**
WNT3	0,063203483	0,041923236	0,012	0	1	1,029485305	0,971359179	#DIV/0!
SIAE	0,060153875	0,04191092	0,049	0,014	1	1,029476516	0,971367471	3,5
UBE2T	0,009174744	0,041900237	0,049	0,007	1	1,029468892	0,971374664	7
ZNF747	0,00931978	0,04187359	0,049	0,007	1	1,029449878	0,971392606	7
DHRS13	0,066254792	0,041832728	0,024	0,004	1	1,029420721	0,971420119	6
OAZ3	0,001220269	0,0418239	0,037	0	1	1,029414422	0,971426064	#DIV/0!
COPB2	1,75588E-07	0,041800165	0,378	0,102	0,00370509	1,029397487	0,971442045	3,705882353
DAAM1	0,000764781	0,041770957	0,329	0,137	1	1,029376646	0,971461713	2,401459854
PRELID2	0,013301771	0,041730953	0,085	0,025	1	1,029348103	0,971488651	3,4
PPP6R1	1,89992E-05	0,041661186	0,207	0,049	0,400901198	1,029298327	0,971535632	4,224489796
RBMS3-AS3	0,00032444	0,041646081	0,061	0,004	1	1,02928755	0,971545804	15,25
RGP1	0,003927556	0,041628773	0,098	0,025	1	1,029275201	0,97155746	3,92
CGREF1	0,001220269	0,041616332	0,037	0	1	1,029266325	0,971565838	#DIV/0!
ZNF341	0,183494953	0,041609256	0,024	0,007	1	1,029261277	0,971570603	3,428571429
SAC3D1	0,002055474	0,041565665	0,134	0,039	1	1,029230179	0,97159996	3,435897436
CRYBB1	0,008401736	0,041553481	0,024	0	1	1,029221487	0,971608165	#DIV/0!
H4-16	0,105939109	0,041463587	0,073	0,032	1	1,029157358	0,971668708	2,28125
SMYD4	0,461793215	0,041440395	0,049	0,032	1	1,029140814	0,971684328	1,53125
KICS2	0,027028964	0,041432836	0,049	0,011	1	1,029135422	0,971689419	4,454545455
TMEM67	0,006707687	0,041389673	0,134	0,046	1	1,029104632	0,971718491	2,913043478
MRPL53	0,024102868	0,041377094	0,085	0,028	1	1,029095659	0,971726963	3,035714286
HOXD1	0,011553628	0,041338695	0,037	0,004	1	1,029068269	0,971752827	9,25
MT-CO3	0,807222086	0,041324009	0,841	0,765	1	1,029057794	0,971762719	1,099346405
NPW	0,831491253	0,041320438	0,037	0,032	1	1,029055246	0,971765125	1,15625
FXN	0,010071401	0,041310548	0,073	0,018	1	1,029048193	0,971771786	4,055555556
AREG	0,02737604	0,04130612	0,049	0,011	1	1,029045034	0,971774769	4,454545455
BICRAL	0,112012827	0,041302471	0,061	0,025	1	1,029042431	0,971777227	2,44
TNFRSF4	0,347088417	0,041298701	0,012	0,004	1	1,029039742	0,971779766	3
ICA1L	0,113968254	0,041252561	0,049	0,018	1	1,029006832	0,971810846	2,722222222
HNRNPUL1	0,000739049	0,041207432	0,549	0,27	1	1,028974644	0,971841246	2,033333333

Supplementary Table 2

Differentially expressed genes_keloid-XL_ comparing SC-Keloid vs. (SC-Myel, SC-Nonmyel, SC-Nonmyel2)								
**gene**	**p_val**	**avg_log2FC**	**pct.1**	**pct.2**	**p_val_adj**	**Foldchange_UP**	**Foldchange_DOWN**	**Ratio_pct1_pct2**
LOXL4	0,001952737	0,041183852	0,049	0,004	1	1,028957826	0,97185713	12,25
ZSCAN25	0,017481575	0,041151044	0,061	0,014	1	1,028934427	0,971879231	4,357142857
FAM167B	0,000183568	0,041141525	0,049	0	1	1,028927638	0,971885643	#DIV/0!
TYW5	0,010546634	0,041113296	0,073	0,018	1	1,028907506	0,971904661	4,055555556
CFI	0,103792275	0,041106273	0,037	0,011	1	1,028902497	0,971909392	3,363636364
CC2D1A	0,002018358	0,041094067	0,183	0,063	1	1,028893792	0,971917615	2,904761905
SLC26A2	0,109353288	0,0410867	0,11	0,056	1	1,028888538	0,971922578	1,964285714
MRPS9	8,36103E-05	0,041078013	0,159	0,035	1	1,028882343	0,97192843	4,542857143
LLGL1	0,001833348	0,041077113	0,061	0,007	1	1,028881701	0,971929036	8,714285714
CCHCR1	0,000470501	0,041067242	0,159	0,042	1	1,028874661	0,971935686	3,785714286
MCM8	0,191478184	0,041052868	0,037	0,014	1	1,02886441	0,97194537	2,642857143
PCAT6	0,00032621	0,04097313	0,073	0,007	1	1,028807546	0,971999091	10,42857143
ARHGDIB	0,010425992	0,040972099	0,073	0,018	1	1,028806811	0,971999786	4,055555556
XPNPEP3	0,002612361	0,040950711	0,11	0,028	1	1,028791559	0,972014196	3,928571429
GGPS1	0,014934204	0,040928194	0,183	0,081	1	1,028775503	0,972029367	2,259259259
APOC1	0,011553628	0,040853036	0,037	0,004	1	1,028721909	0,972080007	9,25
MAP1S	8,6678E-05	0,040846095	0,134	0,025	1	1,02871696	0,972084683	5,36
DOCK8	0,010916043	0,040644446	0,073	0,018	1	1,028573184	0,972220563	4,055555556
RNF6	2,29157E-05	0,040635174	0,22	0,056	0,483544625	1,028566573	0,972226812	3,928571429
INSIG2	0,000599391	0,040599614	0,146	0,039	1	1,028541221	0,972250776	3,743589744
UPRT	0,00414399	0,040536833	0,073	0,014	1	1,028496464	0,972293086	5,214285714
YARS2	7,15736E-06	0,040503578	0,134	0,018	0,151027427	1,028472756	0,972315498	7,444444444
BCAS3	0,000735726	0,040470716	0,122	0,028	1	1,028449329	0,972337646	4,357142857
SBF2-AS1	0,185668317	0,040427465	0,024	0,007	1	1,028418498	0,972366796	3,428571429
EHD4	6,24531E-06	0,040397965	0,415	0,147	0,13178233	1,028397469	0,972386679	2,823129252
TMEM198	0,011553628	0,04035737	0,037	0,004	1	1,028368533	0,972414041	9,25
MACROH2A2	0,001220269	0,040291135	0,037	0	1	1,02832132	0,972458686	#DIV/0!
ASAP1	0,055255667	0,040252286	0,159	0,081	1	1,02829363	0,972484873	1,962962963
C14orf93	0,181340493	0,040208538	0,024	0,007	1	1,028262449	0,972514362	3,428571429
LZTS3	0,001787728	0,040200767	0,098	0,021	1	1,02825691	0,972519601	4,666666667

Supplementary Table 2

Differentially expressed genes_keloid-XL_ comparing SC-Keloid vs. (SC-Myel, SC-Nonmyel, SC-Nonmyel2)								
gene	p_val	avg_log2FC	pct.1	pct.2	p_val_adj	Foldchange_UP	Foldchange_DOWN	Ratio_pct1_pct2
GPN2	0,017068103	0,040187822	0,061	0,014	1	1,028247683	0,972528328	4,357142857
MFAP3	0,001876168	0,040169194	0,098	0,021	1	1,028234407	0,972540885	4,666666667
ABCB8	0,000109686	0,04013976	0,171	0,042	1	1,028213429	0,972560727	4,071428571
GNPDA1	0,001440066	0,040054594	0,159	0,049	1	1,028152733	0,972618141	3,244897959
SDE2	0,017271324	0,040029946	0,134	0,053	1	1,028135167	0,972634758	2,528301887
BHMT2	0,008401736	0,039927388	0,024	0	1	1,028062082	0,972703903	#DIV/0!
TSNAX	8,80969E-05	0,039918237	0,244	0,074	1	1,028055561	0,972710073	3,297297297
GIT1	0,12027408	0,039916059	0,378	0,246	1	1,028054009	0,972711542	1,536585366
ACOT8	0,000214626	0,039764718	0,134	0,028	1	1,02794617	0,972813586	4,785714286
NAIF1	0,001220269	0,039728772	0,037	0	1	1,027920559	0,972837824	#DIV/0!
STAT2	0,000407186	0,03967239	0,244	0,084	1	1,027880387	0,972875845	2,904761905
PTGS1	0,008401736	0,039661023	0,024	0	1	1,027872288	0,97288351	#DIV/0!
UXS1	0,005641411	0,03958929	0,293	0,137	1	1,027821182	0,972931885	2,138686131
KDM8	0,347088417	0,039547779	0,012	0,004	1	1,027791609	0,972959879	3
TRPC4AP	2,44026E-05	0,039541532	0,317	0,102	0,514918259	1,027787158	0,972964093	3,107843137
ZNF70	0,043609761	0,039483084	0,037	0,007	1	1,027745521	0,973003511	5,285714286
ASB6	0,003430365	0,039391331	0,122	0,035	1	1,02768016	0,973065395	3,485714286
AMMECR1	0,198346594	0,039372708	0,037	0,014	1	1,027666894	0,973077955	2,642857143
AATF	2,61027E-05	0,039361667	0,22	0,056	0,550793183	1,027659029	0,973085402	3,928571429
OARD1	2,91165E-05	0,039354732	0,232	0,063	0,614386888	1,027654089	0,97309008	3,682539683
RNF166	0,025884674	0,039348224	0,085	0,028	1	1,027649453	0,97309447	3,035714286
KCNA3	0,063203483	0,039344876	0,012	0	1	1,027647069	0,973096728	#DIV/0!
LSM10	5,26643E-08	0,039341319	0,415	0,116	0,001111269	1,027644535	0,973099127	3,577586207
MSANTD4	1,52159E-05	0,03933704	0,159	0,028	0,321070382	1,027641487	0,973102013	5,678571429
ADGRG2	0,008401736	0,039288114	0,024	0	1	1,027606638	0,973135014	#DIV/0!
FAM131A	0,000295757	0,039251377	0,146	0,035	1	1,027580471	0,973159795	4,171428571
OR7A5	0,065143806	0,039241571	0,024	0,004	1	1,027573486	0,973166409	6
CAVIN1	0,148262174	0,039238277	0,744	0,523	1	1,02757114	0,973168631	1,422562141
ZNF750	0,008401736	0,039164055	0,024	0	1	1,027518276	0,973218699	#DIV/0!
PRR19	0,001952737	0,03912495	0,049	0,004	1	1,027490425	0,973245079	12,25

Supplementary Table 2

Differentially expressed genes_keloid-XL_ comparing SC-Keloid vs. (SC-Myel, SC-Nonmyel, SC-Nonmyel2)								
**gene**	**p_val**	**avg_log2FC**	**pct.1**	**pct.2**	**p_val_adj**	**Foldchange_UP**	**Foldchange_DOWN**	**Ratio_pct1_pct2**
RINT1	0,561847818	0,039057942	0,037	0,025	1	1,027442703	0,973290284	1,48
MFNG	0,065143806	0,039041986	0,024	0,004	1	1,02743134	0,973301048	6
MYL5	0,001809471	0,038970264	0,098	0,021	1	1,027380263	0,973349436	4,666666667
ZCCHC24	0,000665196	0,038945422	0,573	0,277	1	1,027362573	0,973366196	2,068592058
KRBOX1	0,006615795	0,038784079	0,061	0,011	1	1,027247685	0,973475058	5,545454545
GALE	0,001604696	0,038762694	0,122	0,032	1	1,027232458	0,973489488	3,8125
INHBB	0,179204865	0,038757057	0,024	0,007	1	1,027228444	0,973493292	3,428571429
ZCCHC10	3,47519E-06	0,038706555	0,268	0,07	0,07333007	1,027192487	0,97352737	3,828571429
CACNG4	0,008401736	0,038653922	0,024	0	1	1,027155013	0,973562887	#DIV/0!
BUD31	0,001024593	0,038593903	0,537	0,253	1	1,027112282	0,97360339	2,122529644
KPNA5	0,017043378	0,038544118	0,11	0,039	1	1,027076838	0,973636989	2,820512821
RUBCNL	0,001952737	0,038483901	0,049	0,004	1	1,02703397	0,973677628	12,25
ZNF784	0,001220269	0,03847057	0,037	0	1	1,02702448	0,973686625	#DIV/0!
OPRL1	0,001220269	0,038458751	0,037	0	1	1,027016066	0,973694602	#DIV/0!
MAP3K12	0,001099251	0,038449809	0,171	0,053	1	1,027009701	0,973700637	3,226415094
ACAT2	0,005476874	0,038444641	0,122	0,039	1	1,027006022	0,973704125	3,128205128
OLFM2	0,005447223	0,038418526	0,11	0,032	1	1,026987432	0,973721751	3,4375
AMN1	0,003970716	0,038399967	0,098	0,025	1	1,02697422	0,973734277	3,92
TUBE1	0,025838473	0,038389112	0,122	0,049	1	1,026966494	0,973741603	2,489795918
TIGD1	0,027726954	0,038388471	0,049	0,011	1	1,026966037	0,973742036	4,454545455
CALML4	0,006431528	0,038298556	0,061	0,011	1	1,026902034	0,973802726	5,545454545
TJAP1	0,021765621	0,038290332	0,183	0,084	1	1,02689618	0,973808277	2,178571429
DDX50	0,002342756	0,038271129	0,195	0,07	1	1,026882512	0,973821239	2,785714286
ZNF746	0,006778966	0,038222022	0,085	0,021	1	1,026847559	0,973854387	4,047619048
TBC1D31	0,066254792	0,038180385	0,024	0,004	1	1,026817924	0,973882493	6
SLC7A6OS	0,000444147	0,038165432	0,11	0,021	1	1,026807282	0,973892587	5,238095238
BEX5	0,009174744	0,038147295	0,049	0,007	1	1,026794373	0,973904831	7
TOP2B	0,000258631	0,038127731	0,39	0,161	1	1,026780449	0,973918037	2,422360248
THAP5	0,000606723	0,038084208	0,268	0,102	1	1,026749474	0,973947419	2,62745098
BEST4	0,008401736	0,038077768	0,024	0	1	1,02674489	0,973951767	#DIV/0!

Supplementary Table 2

Differentially expressed genes_keloid-XL_ comparing SC-Keloid vs. (SC-Myel, SC-Nonmyel, SC-Nonmyel2)								
**gene**	**p_val**	**avg_log2FC**	**pct.1**	**pct.2**	**p_val_adj**	**Foldchange_UP**	**Foldchange_DOWN**	**Ratio_pct1_pct2**
HECW2	0,044226311	0,038063786	0,037	0,007	1	1,02673494	0,973961206	5,285714286
ZNF547	0,065143806	0,038062303	0,024	0,004	1	1,026733885	0,973962207	6
METTL13	0,01030658	0,038049638	0,073	0,018	1	1,026724871	0,973970757	4,055555556
NR1H2	1,07319E-05	0,038030448	0,305	0,091	0,226454834	1,026711214	0,973983713	3,351648352
ASS1	0,058268295	0,038027374	0,049	0,014	1	1,026709026	0,973985788	3,5
GYG1	1,68287E-05	0,038018485	0,207	0,049	0,355103187	1,026702701	0,973991789	4,224489796
OSBPL7	0,02175178	0,038005593	0,073	0,021	1	1,026693526	0,974000493	3,476190476
GLIPR1L2	0,520464457	0,037990512	0,024	0,014	1	1,026682794	0,974010674	1,714285714
CALCOCO1	0,004395453	0,037985783	0,146	0,049	1	1,026679429	0,974013867	2,979591837
NSUN3	0,004426608	0,037980727	0,098	0,025	1	1,02667583	0,97401728	3,92
RPS11	0,946283832	0,037960306	0,939	0,877	1	1,026661298	0,974031068	1,070695553
MTERF1	0,00032444	0,037957407	0,061	0,004	1	1,026659235	0,974033025	15,25
MEAF6	1,62781E-05	0,037940472	0,5	0,193	0,34348333	1,026647184	0,974044458	2,590673575
CWF19L1	0,017481575	0,037767519	0,061	0,014	1	1,026524115	0,974161236	4,357142857
AGBL1	0,063203483	0,037716905	0,012	0	1	1,026488102	0,974195413	#DIV/0!
P4HA3	0,00140181	0,037703002	0,073	0,011	1	1,02647821	0,974204801	6,636363636
SELENOT	0,00813047	0,037674636	0,488	0,263	1	1,026458028	0,974223956	1,855513308
MYLK-AS1	0,036427227	0,037658732	0,061	0,018	1	1,026446712	0,974234696	3,388888889
TFAP4	0,060793535	0,037618835	0,049	0,014	1	1,026418326	0,974261638	3,5
LTC4S	0,010071401	0,037614165	0,073	0,018	1	1,026415004	0,974264792	4,055555556
ZNF830	0,003546515	0,037605795	0,134	0,042	1	1,02640905	0,974270444	3,19047619
DHRS4L2	0,000657816	0,037605005	0,146	0,039	1	1,026408487	0,974270978	3,743589744
SUPT20H	0,001504284	0,037594159	0,268	0,109	1	1,026400771	0,974278302	2,458715596
ZC3HAV1L	0,011553628	0,037581571	0,037	0,004	1	1,026391815	0,974286803	9,25
C6orf52	0,008401736	0,037519703	0,024	0	1	1,026347801	0,974328585	#DIV/0!
FOXS1	0,00032444	0,037517499	0,061	0,004	1	1,026346232	0,974330073	15,25
TRIM58	0,000503644	0,037509194	0,134	0,032	1	1,026340324	0,974335682	4,1875
PACSIN2	0,002472188	0,03750589	0,39	0,182	1	1,026337974	0,974337913	2,142857143
KCTD7	0,001831458	0,037468535	0,098	0,021	1	1,0263114	0,974363142	4,666666667
SLC35B3	0,000108174	0,037462067	0,22	0,063	1	1,026306798	0,97436751	3,492063492

Supplementary Table 2

Differentially expressed genes_keloid-XL_ comparing SC-Keloid vs. (SC-Myel, SC-Nonmyel, SC-Nonmyel2)								
**gene**	**p_val**	**avg_log2FC**	**pct.1**	**pct.2**	**p_val_adj**	**Foldchange_UP**	**Foldchange_DOWN**	**Ratio_pct1_pct2**
CHST10	0,007986503	0,037414845	0,098	0,028	1	1,026273207	0,974399403	3,5
NKAIN4	0,000174436	0,037369453	0,11	0,018	1	1,026240917	0,974430062	6,111111111
ABL1	0,002631923	0,037326102	0,28	0,119	1	1,02621008	0,974459342	2,352941176
LSM12	4,24587E-05	0,03730063	0,366	0,133	0,895921905	1,026191962	0,974476547	2,751879699
SELENOK	0,028614003	0,037289525	0,768	0,502	1	1,026184063	0,974484049	1,529880478
PYCR3	0,000983548	0,037279512	0,085	0,014	1	1,026176941	0,974490812	6,071428571
RHOT1	0,000279292	0,037230157	0,268	0,095	1	1,026141835	0,97452415	2,821052632
DUS4L	0,001220269	0,037199765	0,037	0	1	1,026120219	0,97454468	#DIV/0!
CA3	0,063203483	0,037123965	0,012	0	1	1,026066307	0,974595884	#DIV/0!
FOXB1	0,063203483	0,037123965	0,012	0	1	1,026066307	0,974595884	#DIV/0!
C2orf69	0,103792275	0,037115656	0,037	0,011	1	1,026060398	0,974601497	3,363636364
CD34	0,307543976	0,037099328	0,037	0,018	1	1,026048785	0,974612528	2,055555556
ICE1	0,000360591	0,037039174	0,305	0,116	1	1,026006005	0,974653165	2,629310345
PPAN	0,003268385	0,037035204	0,122	0,035	1	1,026003181	0,974655847	3,485714286
RNF220	0,000379338	0,037009083	0,183	0,053	1	1,025984605	0,974673494	3,452830189
ENTPD3-AS1	0,000111268	0,03700768	0,122	0,021	1	1,025983607	0,974674442	5,80952381
IQSEC2	0,00032444	0,036977146	0,061	0,004	1	1,025961893	0,974695071	15,25
ERMAP	0,061438821	0,036922292	0,049	0,014	1	1,025922884	0,974732132	3,5
MEAK7	0,004485159	0,036872878	0,073	0,014	1	1,025887746	0,974765518	5,214285714
WDR73	0,001379438	0,036872484	0,146	0,042	1	1,025887466	0,974765784	3,476190476
UHRF1BP1	0,00931978	0,036843666	0,049	0,007	1	1,025866974	0,974785255	7
TMEM163	0,000183568	0,036823047	0,049	0	1	1,025852312	0,974799187	#DIV/0!
GLRB	0,00032444	0,036801217	0,061	0,004	1	1,025836789	0,974813938	15,25
GATD1	1,14878E-05	0,03675555	0,195	0,042	0,242404618	1,025804319	0,974844794	4,642857143
ZNF227	0,961674519	0,036735337	0,037	0,039	1	1,025789947	0,974858452	0,948717949
EXOSC3	1,45037E-05	0,036714985	0,22	0,053	0,306043147	1,025775476	0,974872205	4,150943396
POLR3C	0,001055888	0,036621824	0,085	0,014	1	1,025709239	0,974935159	6,071428571
BTN2A2	0,001222478	0,036600423	0,11	0,025	1	1,025694024	0,974949621	4,4
MTERF2	0,002387752	0,036552553	0,159	0,053	1	1,025659991	0,974981971	3
CABCOCO1	0,183494953	0,036550391	0,024	0,007	1	1,025658454	0,974983433	3,428571429

Supplementary Table 2

Differentially expressed genes_keloid-XL_ comparing SC-Keloid vs. (SC-Myel, SC-Nonmyel, SC-Nonmyel2)								
**gene**	**p_val**	**avg_log2FC**	**pct.1**	**pct.2**	**p_val_adj**	**Foldchange_UP**	**Foldchange_DOWN**	**Ratio_pct1_pct2**
EFCAB2	0,069914007	0,036506484	0,122	0,06	1	1,025627239	0,975013106	2,033333333
PUS10	0,044226311	0,036504659	0,037	0,007	1	1,025625942	0,975014339	5,285714286
RIN1	5,32686E-05	0,036456156	0,073	0,004	1	1,025591462	0,975047119	18,25
CNIH2	0,066254792	0,036415257	0,024	0,004	1	1,025562387	0,975074761	6
ZNF586	0,35185965	0,036343888	0,012	0,004	1	1,025511655	0,975122999	3
HNRNPA1L2	0,194890196	0,036332971	0,037	0,014	1	1,025503895	0,975130377	2,642857143
ZNF780B	0,004368707	0,036246136	0,073	0,014	1	1,025442172	0,975189072	5,214285714
VAX2	0,001952737	0,036239668	0,049	0,004	1	1,025437575	0,975193444	12,25
NUP37	0,002909566	0,036192211	0,085	0,018	1	1,025403844	0,975225523	4,722222222
SLC25A6	0,109098021	0,036081162	0,756	0,477	1	1,025324918	0,975300592	1,58490566
ANKS3	0,005188266	0,036067187	0,11	0,032	1	1,025314986	0,97531004	3,4375
ERICH3	0,063203483	0,036003362	0,012	0	1	1,025269627	0,975353189	#DIV/0!
SNRK-AS1	0,063203483	0,036003362	0,012	0	1	1,025269627	0,975353189	#DIV/0!
SHB	0,146677521	0,035969907	0,11	0,06	1	1,025245852	0,975375806	1,833333333
RFXANK	5,25695E-05	0,035928007	0,256	0,077	1	1,025216076	0,975404135	3,324675325
ASB2	0,001220269	0,035920483	0,037	0	1	1,02521073	0,975409222	#DIV/0!
VPS72	2,40727E-06	0,035894411	0,305	0,084	0,050795843	1,025192202	0,975426849	3,630952381
MED14OS	0,004485159	0,035871883	0,073	0,014	1	1,025176194	0,975442081	5,214285714
MTSS1	0,008401736	0,035861645	0,024	0	1	1,025168919	0,975449003	#DIV/0!
PHF7	0,00931978	0,035854973	0,049	0,007	1	1,025164178	0,975453514	7
COL22A1	0,000344207	0,035849756	0,073	0,007	1	1,025160471	0,975457042	10,42857143
TYK2	2,18131E-05	0,035809994	0,244	0,067	0,460278759	1,025132217	0,975483926	3,641791045
ZNF589	0,183494953	0,035807576	0,024	0,007	1	1,025130498	0,975485562	3,428571429
PDE1C	0,001419533	0,035796974	0,146	0,042	1	1,025122965	0,97549273	3,476190476
ZC3H8	0,006483581	0,035774897	0,085	0,021	1	1,025107278	0,975507658	4,047619048
GLB1L	0,187860656	0,035755664	0,024	0,007	1	1,025093612	0,975520663	3,428571429
KLHL22	6,3637E-06	0,03570113	0,183	0,035	0,13428035	1,025054865	0,975557538	5,228571429
LINC00893	0,008401736	0,03569867	0,024	0	1	1,025053117	0,975559201	#DIV/0!
GPC2	0,027726954	0,035626637	0,049	0,011	1	1,025001938	0,975607912	4,454545455
BGLAP	0,001055888	0,035589085	0,085	0,014	1	1,024975258	0,975633306	6,071428571

Supplementary Table 2

Differentially expressed genes_keloid-XL_ comparing SC-Keloid vs. (SC-Myel, SC-Nonmyel, SC-Nonmyel2)								
gene	p_val	avg_log2FC	pct.1	pct.2	p_val_adj	Foldchange_UP	Foldchange_DOWN	Ratio_pct1_pct2
WWC3	0,336979217	0,03556159	0,171	0,123	1	1,024955725	0,9756519	1,390243902
LMBR1	0,025203577	0,035440548	0,085	0,028	1	1,024869734	0,975733761	3,035714286
RASSF7	0,001802592	0,035372114	0,061	0,007	1	1,024821121	0,975780046	8,714285714
ASPRV1	0,181340493	0,035363426	0,024	0,007	1	1,024814949	0,975785922	3,428571429
LINC00460	0,008401736	0,035356417	0,024	0	1	1,024809971	0,975790662	#DIV/0!
LRATD1	0,066254792	0,035336543	0,024	0,004	1	1,024795853	0,975804105	6
ZNF688	4,30313E-05	0,035305162	0,207	0,053	0,908002961	1,024773562	0,97582533	3,905660377
CYP4V2	0,001876168	0,035265712	0,098	0,021	1	1,024745541	0,975852014	4,666666667
TLCD1	0,000183568	0,035249059	0,049	0	1	1,024733712	0,975863278	#DIV/0!
GBGT1	0,008401736	0,035214695	0,024	0	1	1,024709304	0,975886523	#DIV/0!
FGF12	0,063203483	0,035091334	0,012	0	1	1,024621688	0,975969972	#DIV/0!
H3C7	0,063203483	0,035091334	0,012	0	1	1,024621688	0,975969972	#DIV/0!
HPCAL4	0,063203483	0,035091334	0,012	0	1	1,024621688	0,975969972	#DIV/0!
IL7	0,063203483	0,035091334	0,012	0	1	1,024621688	0,975969972	#DIV/0!
OR7D2	0,063203483	0,035091334	0,012	0	1	1,024621688	0,975969972	#DIV/0!
PFKFB1	0,063203483	0,035091334	0,012	0	1	1,024621688	0,975969972	#DIV/0!
TAFA3	0,063203483	0,035091334	0,012	0	1	1,024621688	0,975969972	#DIV/0!
TNPO2	0,00013292	0,03506139	0,244	0,077	1	1,024600421	0,975990229	3,168831169
SERF1A	0,043609761	0,034981343	0,037	0,007	1	1,024543574	0,976044383	5,285714286
CKMT1A	0,063203483	0,03493802	0,012	0	1	1,024512808	0,976073693	#DIV/0!
KRT79	0,063203483	0,03493802	0,012	0	1	1,024512808	0,976073693	#DIV/0!
MGAT5B	0,063203483	0,03493802	0,012	0	1	1,024512808	0,976073693	#DIV/0!
NPM2	0,063203483	0,03493802	0,012	0	1	1,024512808	0,976073693	#DIV/0!
RPL24	0,481746482	0,034889716	0,939	0,821	1	1,024478506	0,976106374	1,143727162
BRME1	0,748990771	0,03485526	0,012	0,018	1	1,024454039	0,976129687	0,666666667
TDRKH	0,347088417	0,034841937	0,012	0,004	1	1,024444578	0,976138702	3
ETNK2	0,037565828	0,034826168	0,061	0,018	1	1,02443338	0,976149371	3,388888889
NCKAP1	1,28469E-06	0,034789918	0,463	0,154	0,027108267	1,02440764	0,976173899	3,006493506
ZNF839	0,427422808	0,034752988	0,037	0,021	1	1,024381418	0,976198887	1,761904762
SKAP1	2,80682E-05	0,034577632	0,061	0	0,592266086	1,024256914	0,976317549	#DIV/0!

Supplementary Table 2

Differentially expressed genes_keloid-XL_ comparing SC-Keloid vs. (SC-Myel, SC-Nonmyel, SC-Nonmyel2)								
**gene**	**p_val**	**avg_log2FC**	**pct.1**	**pct.2**	**p_val_adj**	**Foldchange_UP**	**Foldchange_DOWN**	**Ratio_pct1_pct2**
XKR8	0,001833348	0,034569253	0,061	0,007	1	1,024250966	0,976323219	8,714285714
PRR5L	0,183494953	0,034567754	0,024	0,007	1	1,024249902	0,976324233	3,428571429
SCD	0,032306429	0,034482611	0,573	0,34	1	1,024189456	0,976381854	1,685294118
KCNJ2	0,339089703	0,034448406	0,024	0,011	1	1,024165173	0,976405004	2,181818182
ACACA	0,000114351	0,034426258	0,122	0,021	1	1,024149451	0,976419994	5,80952381
ISG20L2	3,13909E-06	0,034423669	0,207	0,042	0,066237957	1,024147613	0,976421746	4,928571429
CES2	0,182936607	0,034401188	0,085	0,046	1	1,024131653	0,976436962	1,847826087
FAM151B	0,008401736	0,034398347	0,024	0	1	1,024129637	0,976438884	#DIV/0!
SLC4A5	0,107265343	0,034383425	0,037	0,011	1	1,024119045	0,976448983	3,363636364
TCN2	0,186442875	0,034340663	0,037	0,014	1	1,02408869	0,976477926	2,642857143
DNAH14	0,001220269	0,034338487	0,037	0	1	1,024087145	0,9764794	#DIV/0!
CDCA7L	0,108593558	0,034291395	0,061	0,025	1	1,024053718	0,976511274	2,44
NSFL1C	8,68606E-05	0,034285733	0,378	0,147	1	1,024049699	0,976515106	2,571428571
KIAA0319L	0,000868198	0,034273776	0,232	0,084	1	1,024041211	0,976523199	2,761904762
SLC6A9	0,001220269	0,034273113	0,037	0	1	1,024040741	0,976523648	#DIV/0!
CBLB	0,024309048	0,034260764	0,134	0,056	1	1,024031975	0,976532007	2,392857143
ARL16	1,8075E-05	0,03424968	0,244	0,067	0,381401007	1,024024108	0,976539509	3,641791045
RAD54L2	0,000594027	0,034226952	0,207	0,067	1	1,024007975	0,976554894	3,089552239
BAIAP2L2	0,065143806	0,034150788	0,024	0,004	1	1,023953917	0,976606451	6
ZNF544	0,010791649	0,034137659	0,073	0,018	1	1,023944599	0,976615338	4,055555556
EXO5	0,001952737	0,034137069	0,049	0,004	1	1,02394418	0,976615737	12,25
GCAT	0,00414399	0,034132757	0,073	0,014	1	1,023941119	0,976618656	5,214285714
ATAD3A	0,000259864	0,034119638	0,171	0,046	1	1,023931808	0,976627537	3,717391304
SLC9A5	0,00931978	0,034106408	0,049	0,007	1	1,023922419	0,976636493	7
CUL7	4,31662E-05	0,034083164	0,159	0,032	0,910850685	1,023905922	0,976652228	4,96875
ZNF626	0,10265465	0,034071234	0,037	0,011	1	1,023897455	0,976660305	3,363636364
GABRA2	6,05627E-05	0,034041547	0,561	0,235	1	1,023876386	0,976680402	2,387234043
GNB5	0,003143853	0,034031411	0,122	0,035	1	1,023869192	0,976687264	3,485714286
LKAAEAR1	0,008401736	0,034025905	0,024	0	1	1,023865284	0,976690992	#DIV/0!
NUP50	0,011065297	0,034008114	0,195	0,084	1	1,023852659	0,976703036	2,321428571

Supplementary Table 2

Differentially expressed genes_keloid-XL_ comparing SC-Keloid vs. (SC-Myel, SC-Nonmyel, SC-Nonmyel2)								
**gene**	**p_val**	**avg_log2FC**	**pct.1**	**pct.2**	**p_val_adj**	**Foldchange_UP**	**Foldchange_DOWN**	**Ratio_pct1_pct2**
BTBD16	0,008401736	0,033997374	0,024	0	1	1,023845037	0,976710307	#DIV/0!
TRIM38	0,009578684	0,033964026	0,146	0,056	1	1,023821371	0,976732884	2,607142857
LOXL1-AS1	0,000183568	0,033867629	0,049	0	1	1,023752964	0,976798148	#DIV/0!
LACC1	0,042398422	0,033816279	0,037	0,007	1	1,023716526	0,976832916	5,285714286
LAPTM4A	0,153658175	0,033780769	0,878	0,646	1	1,023691329	0,976856961	1,359133127
ZNF582-DT	0,347088417	0,033713784	0,012	0,004	1	1,0236438	0,976902317	3
RPAP1	0,000511843	0,033693784	0,11	0,021	1	1,023629609	0,97691586	5,238095238
BFAR	6,7885E-05	0,033684335	0,268	0,088	1	1,023622905	0,976922258	3,045454545
B3GNTL1	5,32686E-05	0,033632945	0,073	0,004	1	1,023586443	0,976957058	18,25
AKNA	0,061438821	0,033628348	0,049	0,014	1	1,023583182	0,976960171	3,5
BCL2L15	0,063203483	0,033520128	0,012	0	1	1,023506403	0,977033458	#DIV/0!
CYSRT1	0,063203483	0,033520128	0,012	0	1	1,023506403	0,977033458	#DIV/0!
SLC16A8	0,063203483	0,033520128	0,012	0	1	1,023506403	0,977033458	#DIV/0!
RAD23B	0,006735904	0,033496919	0,329	0,161	1	1,023489938	0,977049175	2,043478261
SFN	0,069455719	0,0334513	0,159	0,084	1	1,023457575	0,977080071	1,892857143
GPAT2	0,059519805	0,033433745	0,049	0,014	1	1,023445121	0,977091961	3,5
VPS4A	0,001609618	0,033397696	0,293	0,123	1	1,023419549	0,977116375	2,382113821
AANAT	0,063203483	0,033392881	0,012	0	1	1,023416133	0,977119637	#DIV/0!
C1QL4	0,063203483	0,033392881	0,012	0	1	1,023416133	0,977119637	#DIV/0!
PCDH15	0,063203483	0,033392881	0,012	0	1	1,023416133	0,977119637	#DIV/0!
ZNF132	0,063203483	0,033392881	0,012	0	1	1,023416133	0,977119637	#DIV/0!
SPR	0,042398422	0,033364419	0,037	0,007	1	1,023395942	0,977138914	5,285714286
RTN2	0,006431528	0,033287672	0,061	0,011	1	1,023341503	0,977190896	5,545454545
VAMP3	0,017562356	0,033275342	0,427	0,232	1	1,023332757	0,977199247	1,840517241
CTPS2	0,106097567	0,033228518	0,037	0,011	1	1,023299544	0,977230964	3,363636364
ZNF564	0,011553628	0,033225577	0,037	0,004	1	1,023297458	0,977232956	9,25
ZNF250	0,022195444	0,033192427	0,073	0,021	1	1,023273945	0,977255411	3,476190476
MTARC2	0,001898897	0,033148312	0,098	0,021	1	1,023242656	0,977285294	4,666666667
CNOT9	0,000329164	0,033139896	0,22	0,07	1	1,023236687	0,977290995	3,142857143
MR1	0,004368707	0,033107141	0,073	0,014	1	1,023213455	0,977313184	5,214285714

Supplementary Table 2

Differentially expressed genes_keloid-XL_ comparing SC-Keloid vs. (SC-Myel, SC-Nonmyel, SC-Nonmyel2)								
**gene**	**p_val**	**avg_log2FC**	**pct.1**	**pct.2**	**p_val_adj**	**Foldchange_UP**	**Foldchange_DOWN**	**Ratio_pct1_pct2**
CCNJL	0,001423372	0,032987717	0,073	0,011	1	1,023128759	0,977394088	6,636363636
PGBD1	0,060153875	0,032987432	0,049	0,014	1	1,023128557	0,977394281	3,5
AL590714.1	0,063203483	0,032976644	0,012	0	1	1,023120906	0,97740159	#DIV/0!
FAM149B1	0,000124092	0,032973026	0,122	0,021	1	1,023118341	0,977404041	5,80952381
JMJD7	0,043609761	0,032968032	0,037	0,007	1	1,023114798	0,977407424	5,285714286
ACOT2	2,46117E-05	0,032940837	0,195	0,046	0,519330851	1,023095513	0,977425849	4,239130435
TUSC1	5,17434E-06	0,032903417	0,354	0,109	0,109183652	1,023068977	0,977451201	3,247706422
HMGXB4	0,004823017	0,032894958	0,207	0,084	1	1,023062978	0,977456932	2,464285714
PCK2	0,000234367	0,03286654	0,098	0,014	1	1,023042826	0,977476186	7
TKFC	0,001853689	0,032866417	0,098	0,021	1	1,023042739	0,97747627	4,666666667
NUF2	0,103792275	0,032847013	0,037	0,011	1	1,023028979	0,977489417	3,363636364
ZNF595	0,027726954	0,03279711	0,049	0,011	1	1,022993593	0,977523229	4,454545455
PXMP2	0,003364716	0,032767341	0,122	0,035	1	1,022972485	0,977543399	3,485714286
WWP2	9,39569E-05	0,032748275	0,159	0,035	1	1,022958965	0,977556318	4,542857143
NCDN	0,037565828	0,032748032	0,061	0,018	1	1,022958793	0,977556483	3,388888889
LDOC1	0,242293287	0,032704802	0,061	0,032	1	1,022928141	0,977585775	1,90625
CDC42EP5	0,026068228	0,032701855	0,634	0,393	1	1,022926051	0,977587773	1,613231552
EPHB2	0,644071107	0,03267285	0,012	0,007	1	1,022905486	0,977607427	1,714285714
GTSE1	0,044226311	0,032650733	0,037	0,007	1	1,022889805	0,977622413	5,285714286
DCLRE1B	0,00931978	0,032613425	0,049	0,007	1	1,022863353	0,977647695	7
IMP4	0,000319433	0,032612592	0,28	0,102	1	1,022862763	0,97764826	2,745098039
SPAAR	0,008401736	0,032603552	0,024	0	1	1,022856354	0,977654386	#DIV/0!
CHCHD6	0,000685144	0,032536439	0,098	0,018	1	1,022808772	0,977699866	5,444444444
BAIAP2-DT	0,005885676	0,032527617	0,122	0,039	1	1,022802518	0,977705845	3,128205128
LPCAT4	0,005137829	0,032512628	0,11	0,032	1	1,022791891	0,977716003	3,4375
CORO2A	0,006341138	0,032488165	0,061	0,011	1	1,022774549	0,977732582	5,545454545
ENTPD1-AS1	0,00031979	0,032416982	0,122	0,025	1	1,022724086	0,977780825	4,88
AC092687.3	0,342070304	0,032409848	0,024	0,011	1	1,022719029	0,97778566	2,181818182
FH	0,001639094	0,032359793	0,207	0,074	1	1,022683545	0,977819585	2,797297297
ZBTB5	0,015603254	0,03235902	0,098	0,032	1	1,022682997	0,977820109	3,0625

Supplementary Table 2

Differentially expressed genes_keloid-XL_ comparing SC-Keloid vs. (SC-Myel, SC-Nonmyel, SC-Nonmyel2)								
**gene**	**p_val**	**avg_log2FC**	**pct.1**	**pct.2**	**p_val_adj**	**Foldchange_UP**	**Foldchange_DOWN**	**Ratio_pct1_pct2**
DDN	0,008401736	0,032340073	0,024	0	1	1,022669567	0,977832951	#DIV/0!
ZNF765	0,104939896	0,032329086	0,037	0,011	1	1,022661778	0,977840398	3,363636364
GPN1	6,21985E-05	0,032257303	0,146	0,028	1	1,022610896	0,977889052	5,214285714
FASTKD2	0,008390305	0,032239668	0,098	0,028	1	1,022598396	0,977901006	3,5
ANGEL1	0,516980245	0,032193918	0,024	0,014	1	1,022565968	0,977932017	1,714285714
CCDC96	0,103792275	0,032179072	0,037	0,011	1	1,022555446	0,977942081	3,363636364
LANCL2	0,067018449	0,032154487	0,061	0,021	1	1,02253802	0,977958746	2,904761905
THBS3	0,006876865	0,032120856	0,134	0,046	1	1,022514184	0,977981544	2,913043478
TDP1	0,111964825	0,032092094	0,049	0,018	1	1,022493799	0,978001041	2,722222222
DUSP7	0,018467596	0,032091796	0,244	0,119	1	1,022493588	0,978001243	2,050420168
RNF19B	0,000261384	0,032090017	0,207	0,063	1	1,022492327	0,978002449	3,285714286
YWHAQ	0,044966845	0,032086969	0,744	0,512	1	1,022490167	0,978004516	1,453125
RIMS2	0,008401736	0,032079133	0,024	0	1	1,022484613	0,978009827	#DIV/0!
KIFBP	3,20938E-05	0,032063045	0,22	0,056	0,677210702	1,022473211	0,978020734	3,928571429
ZNF135	0,066254792	0,032003785	0,024	0,004	1	1,022431213	0,978060908	6
TRIM39	0,011553628	0,031994382	0,037	0,004	1	1,022424549	0,978067282	9,25
PBLD	0,027726954	0,03197589	0,049	0,011	1	1,022411444	0,978079819	4,454545455
SNRPB	0,03364801	0,031943494	0,659	0,393	1	1,022388486	0,978101782	1,676844784
RNF44	0,13485524	0,031882431	0,085	0,042	1	1,022345213	0,978143182	2,023809524
MAK16	0,006854678	0,031880996	0,085	0,021	1	1,022344197	0,978144154	4,047619048
PPP2R5D	0,062847269	0,031828658	0,098	0,042	1	1,022307109	0,97817964	2,333333333
ZNF71	0,004102792	0,031778874	0,098	0,025	1	1,022271832	0,978213396	3,92
NEDD1	0,004286645	0,031777399	0,146	0,049	1	1,022270787	0,978214395	2,979591837
ALS2	0,459043308	0,031768496	0,122	0,091	1	1,022264478	0,978220432	1,340659341
CCZ1B	0,00124327	0,031731763	0,183	0,06	1	1,022238451	0,978245339	3,05
CFAP53	0,008401736	0,031722184	0,024	0	1	1,022231663	0,978251835	#DIV/0!
CIP2A	0,008401736	0,031719362	0,024	0	1	1,022229663	0,978253748	#DIV/0!
PLCD4	0,064048232	0,03169915	0,024	0,004	1	1,022215343	0,978267453	6
ARHGEF10L	0,015517806	0,031667322	0,159	0,067	1	1,022192791	0,978289036	2,373134328
RABGGTA	0,000931651	0,031655584	0,159	0,046	1	1,022184474	0,978296996	3,456521739

Supplementary Table 2

Differentially expressed genes_keloid-XL_ comparing SC-Keloid vs. (SC-Myel, SC-Nonmyel, SC-Nonmyel2)								
gene	p_val	avg_log2FC	pct.1	pct.2	p_val_adj	Foldchange_UP	Foldchange_DOWN	Ratio_pct1_pct2
GRHL3	0,063203483	0,031645008	0,012	0	1	1,022176981	0,978304167	#DIV/0!
PRKCI	0,002166012	0,031628218	0,232	0,091	1	1,022165085	0,978315552	2,549450549
ZFP92	0,008401736	0,031606611	0,024	0	1	1,022149777	0,978330204	#DIV/0!
MUTYH	0,004102792	0,031597558	0,098	0,025	1	1,022143362	0,978336344	3,92
CCN4	0,008401736	0,031583891	0,024	0	1	1,022133679	0,978345612	#DIV/0!
AKAP11	0,001574491	0,031532672	0,232	0,088	1	1,022097392	0,978380346	2,636363636
TEP1	0,112963039	0,031494966	0,049	0,018	1	1,022070679	0,978405917	2,722222222
CDC42BPG	0,193178656	0,03148236	0,037	0,014	1	1,022061749	0,978414466	2,642857143
ACADS	0,007159314	0,031466488	0,146	0,053	1	1,022050504	0,978425231	2,754716981
EEF1AKMT3	0,011553628	0,031447894	0,037	0,004	1	1,022037331	0,978437841	9,25
CENPS	0,000298361	0,031409156	0,085	0,011	1	1,022009889	0,978464114	7,727272727
CST6	0,001220269	0,031399704	0,037	0	1	1,022003193	0,978470524	#DIV/0!
REPS1	0,008308113	0,031329203	0,098	0,028	1	1,021953252	0,97851834	3,5
CAST	0,001336086	0,0313289	0,561	0,274	1	1,021953037	0,978518546	2,047445255
VPS33A	0,00931978	0,031269868	0,049	0,007	1	1,021911222	0,978558586	7
VILL	0,011553628	0,031239514	0,037	0,004	1	1,021889721	0,978579175	9,25
TMEM44	0,00032444	0,031176619	0,061	0,004	1	1,021845173	0,978621837	15,25
TADA2B	0,006615795	0,031129223	0,061	0,011	1	1,021811603	0,978653988	5,545454545
IQGAP3	0,347088417	0,031117492	0,012	0,004	1	1,021803294	0,978661946	3
AQP10	0,347088417	0,031105166	0,012	0,004	1	1,021794564	0,978670307	3
TMEM132A	0,000956293	0,031089673	0,256	0,098	1	1,021783591	0,978680817	2,612244898
FBN3	0,008401736	0,031067037	0,024	0	1	1,021767559	0,978696173	#DIV/0!
FBXO46	0,042668024	0,031014537	0,073	0,025	1	1,021730378	0,978731789	2,92
ATRNL1	0,008401736	0,031005519	0,024	0	1	1,021723991	0,978737907	#DIV/0!
TRIM62	0,000293572	0,030932059	0,085	0,011	1	1,021671968	0,978787743	7,727272727
ZDHHC2	0,695998291	0,030924571	0,061	0,049	1	1,021666665	0,978792824	1,244897959
ANO9	0,008401736	0,030912599	0,024	0	1	1,021658187	0,978800946	#DIV/0!
MOG	0,066254792	0,030906688	0,024	0,004	1	1,021654001	0,978804957	6
RHBDL1	0,001220269	0,030903223	0,037	0	1	1,021651547	0,978807307	#DIV/0!
ZNF304	0,027726954	0,030872289	0,049	0,011	1	1,021629641	0,978828295	4,454545455

Supplementary Table 2

Differentially expressed genes_keloid-XL_ comparing SC-Keloid vs. (SC-Myel, SC-Nonmyel, SC-Nonmyel2)								
**gene**	**p_val**	**avg_log2FC**	**pct.1**	**pct.2**	**p_val_adj**	**Foldchange_UP**	**Foldchange_DOWN**	**Ratio_pct1_pct2**
ZNF543	0,044226311	0,030864233	0,037	0,007	1	1,021623936	0,978833761	5,285714286
ZNF214	0,001220269	0,030842773	0,037	0	1	1,02160874	0,978848321	#DIV/0!
GPATCH11	0,001589697	0,030825629	0,159	0,049	1	1,0215966	0,978859953	3,244897959
PTBP3	0,001743633	0,03081531	0,232	0,091	1	1,021589293	0,978866955	2,549450549
ANKRD16	0,000344207	0,030773288	0,073	0,007	1	1,021559537	0,978895467	10,42857143
ITPRIP	0,068052562	0,030725597	0,146	0,074	1	1,021525768	0,978927826	1,972972973
TOR2A	0,011553628	0,030722786	0,037	0,004	1	1,021523778	0,978929734	9,25
SLC2A13	0,302095643	0,030669783	0,122	0,081	1	1,021486249	0,978965699	1,50617284
SNRPA	0,00060856	0,030663435	0,317	0,13	1	1,021481755	0,978970006	2,438461538
KRIT1	0,00212947	0,030632724	0,195	0,07	1	1,02146001	0,978990847	2,785714286
ZNF93	0,00931978	0,03062312	0,049	0,007	1	1,02145321	0,978997364	7
CATSPER1	0,000183568	0,030590875	0,049	0	1	1,021430381	0,979019245	#DIV/0!
TATDN2	0,14913678	0,030525866	0,134	0,077	1	1,021384355	0,979063361	1,74025974
TOMM34	8,57548E-05	0,030509526	0,183	0,046	1	1,021372787	0,97907445	3,97826087
NIPA1	0,000732778	0,030445555	0,098	0,018	1	1,021327499	0,979117865	5,444444444
CYGB	0,043609761	0,030422992	0,037	0,007	1	1,021311526	0,979133178	5,285714286
TRMT61A	7,99666E-05	0,030415174	0,207	0,056	1	1,021305991	0,979138484	3,696428571
RUFY1	0,002595385	0,030414779	0,183	0,067	1	1,021305712	0,979138752	2,731343284
SH3D21	0,10265465	0,030400127	0,037	0,011	1	1,021295339	0,979148696	3,363636364
SOS1	0,014889645	0,030362297	0,171	0,074	1	1,02126856	0,979174371	2,310810811
PNPLA4	0,000142807	0,030350265	0,11	0,018	1	1,021260042	0,979182538	6,111111111
ORC5	0,00196861	0,030251951	0,098	0,021	1	1,02119045	0,979249267	4,666666667
MZF1	0,003842512	0,030221766	0,098	0,025	1	1,021169084	0,979269756	3,92
WDR55	6,41411E-06	0,030210245	0,146	0,021	0,135344167	1,021160929	0,979277576	6,952380952
ZNF813	0,008401736	0,030198611	0,024	0	1	1,021152695	0,979285473	#DIV/0!
UPK3BL1	0,000240941	0,030197205	0,207	0,063	1	1,0211517	0,979286428	3,285714286
LINC02584	0,001220269	0,030044468	0,037	0	1	1,021043597	0,97939011	#DIV/0!
AR	0,194890196	0,030037797	0,037	0,014	1	1,021038875	0,979394638	2,642857143
SLC35F2	0,058268295	0,029998538	0,049	0,014	1	1,021011091	0,97942129	3,5
IRAK1BP1	0,004199164	0,029952466	0,073	0,014	1	1,020978486	0,979452568	5,214285714

Supplementary Table 2

Differentially expressed genes_keloid-XL_ comparing SC-Keloid vs. (SC-Myel, SC-Nonmyel, SC-Nonmyel2)								
**gene**	**p_val**	**avg_log2FC**	**pct.1**	**pct.2**	**p_val_adj**	**Foldchange_UP**	**Foldchange_DOWN**	**Ratio_pct1_pct2**
IZUMO4	0,008401736	0,029904443	0,024	0	1	1,020944501	0,979485172	#DIV/0!
MICAL1	0,00716736	0,029895859	0,134	0,046	1	1,020938427	0,979490999	2,913043478
SAMD10	0,185668317	0,029883894	0,024	0,007	1	1,020929959	0,979499123	3,428571429
ST8SIA1	0,345067611	0,029882299	0,024	0,011	1	1,020928831	0,979500206	2,181818182
WDR37	0,027726954	0,029881989	0,049	0,011	1	1,020928611	0,979500416	4,454545455
DNAJC28	0,066254792	0,029852238	0,024	0,004	1	1,020907558	0,979520616	6
METTL1	0,000344207	0,029796807	0,073	0,007	1	1,020868334	0,979558252	10,42857143
MAFG-DT	0,004485159	0,029795866	0,073	0,014	1	1,020867668	0,979558891	5,214285714
CDCP1	0,008401736	0,029762772	0,024	0	1	1,020844251	0,979581361	#DIV/0!
MN1	0,564675598	0,029726786	0,037	0,025	1	1,020818788	0,979605795	1,48
EML2	0,043216097	0,029674108	0,085	0,032	1	1,020781514	0,979641565	2,65625
TMEM255A	0,270579294	0,029665209	0,049	0,025	1	1,020775218	0,979647608	1,96
DSG3	0,183494953	0,029659291	0,024	0,007	1	1,02077103	0,979651626	3,428571429
ABHD6	0,001112971	0,029647731	0,11	0,025	1	1,020762852	0,979659476	4,4
CCDC180	0,345067611	0,029613241	0,024	0,011	1	1,020738448	0,979682897	2,181818182
CLIC5	0,347088417	0,029610942	0,012	0,004	1	1,020736822	0,979684458	3
LRRC73	0,347088417	0,029605803	0,012	0,004	1	1,020733186	0,979687947	3
RFC5	4,95841E-05	0,029591327	0,11	0,014	1	1,020722944	0,979697778	7,857142857
ZFYVE9	0,053680889	0,029553662	0,122	0,056	1	1,020696296	0,979723356	2,178571429
RNASEH1	0,004684683	0,029528772	0,183	0,07	1	1,020678687	0,979740258	2,614285714
TNFSF4	0,011553628	0,02952416	0,037	0,004	1	1,020675424	0,97974339	9,25
CNNM2	0,748990771	0,029497635	0,012	0,018	1	1,020656658	0,979761404	0,666666667
PHYHIP	0,001952737	0,029489983	0,049	0,004	1	1,020651244	0,979766601	12,25
TRMT12	0,001952737	0,029468322	0,049	0,004	1	1,02063592	0,979781311	12,25
CABIN1	0,09581901	0,029455232	0,134	0,07	1	1,02062666	0,979790201	1,914285714
KLHL13	0,196612832	0,029405545	0,037	0,014	1	1,02059151	0,979823946	2,642857143
CAMKK1	0,00931978	0,029385913	0,049	0,007	1	1,020577622	0,979837279	7
TMSB15B	0,044226311	0,029384925	0,037	0,007	1	1,020576923	0,97983795	5,285714286
RBM15B	0,002734083	0,02932145	0,159	0,053	1	1,020532021	0,979881062	3
UBAC2-AS1	0,008401736	0,029305663	0,024	0	1	1,020520853	0,979891784	#DIV/0!

Supplementary Table 2

Differentially expressed genes_keloid-XL_ comparing SC-Keloid vs. (SC-Myel, SC-Nonmyel, SC-Nonmyel2)								
**gene**	**p_val**	**avg_log2FC**	**pct.1**	**pct.2**	**p_val_adj**	**Foldchange_UP**	**Foldchange_DOWN**	**Ratio_pct1_pct2**
TESK1	0,006969362	0,029283555	0,171	0,067	1	1,020505215	0,9799068	2,552238806
TTI2	0,010546634	0,029279126	0,073	0,018	1	1,020502082	0,979909809	4,055555556
FKTN	0,268844049	0,029266317	0,049	0,025	1	1,020493022	0,979918509	1,96
CDK20	0,112963039	0,029263613	0,049	0,018	1	1,020491109	0,979920346	2,722222222
LINC00393	0,008401736	0,029252475	0,024	0	1	1,02048323	0,979927911	#DIV/0!
DBT	0,138095537	0,029251038	0,085	0,042	1	1,020482214	0,979928887	2,023809524
SLC12A9	0,041519021	0,02920182	0,085	0,032	1	1,020447401	0,979962318	2,65625
TMEM145	0,066254792	0,029201387	0,024	0,004	1	1,020447095	0,979962612	6
LRCH2	0,008401736	0,029200742	0,024	0	1	1,020446638	0,97996305	#DIV/0!
ZNF433	0,000344207	0,029102164	0,073	0,007	1	1,020376914	0,980030013	10,42857143
TOM1L1	0,000713367	0,029074793	0,098	0,018	1	1,020357556	0,980048606	5,444444444
TEF	0,016767057	0,029049073	0,11	0,039	1	1,020339366	0,980066078	2,820512821
CERS3	0,063203483	0,029048221	0,012	0	1	1,020338762	0,980066657	#DIV/0!
CLCA4	0,063203483	0,029048221	0,012	0	1	1,020338762	0,980066657	#DIV/0!
DQX1	0,063203483	0,029048221	0,012	0	1	1,020338762	0,980066657	#DIV/0!
EPN3	0,063203483	0,029048221	0,012	0	1	1,020338762	0,980066657	#DIV/0!
HCAR2	0,063203483	0,029048221	0,012	0	1	1,020338762	0,980066657	#DIV/0!
PPARGC1B	0,063203483	0,029048221	0,012	0	1	1,020338762	0,980066657	#DIV/0!
SGPP2	0,063203483	0,029048221	0,012	0	1	1,020338762	0,980066657	#DIV/0!
ZNF257	0,063203483	0,029048221	0,012	0	1	1,020338762	0,980066657	#DIV/0!
NPAS3	0,066254792	0,029042259	0,024	0,004	1	1,020334546	0,980070707	6
RMND1	0,000147137	0,028998654	0,146	0,032	1	1,020303707	0,98010033	4,5625
TGDS	0,000742666	0,028968762	0,098	0,018	1	1,020282568	0,980120637	5,444444444
RASIP1	0,101526965	0,028963956	0,037	0,011	1	1,020279168	0,980123902	3,363636364
RNASEL	0,000162451	0,02895091	0,11	0,018	1	1,020269942	0,980132766	6,111111111
RPP40	0,004426586	0,02889057	0,073	0,014	1	1,020227271	0,98017376	5,214285714
SPATA22	0,106097567	0,028878375	0,037	0,011	1	1,020218647	0,980182045	3,363636364
URGCP	0,039702859	0,02884048	0,122	0,053	1	1,02019185	0,980207792	2,301886792
CPNE8	0,005780975	0,028765056	0,122	0,039	1	1,020138515	0,980259038	3,128205128
ALG6	0,003268385	0,028722013	0,122	0,035	1	1,02010808	0,980288285	3,485714286

Supplementary Table 2

Differentially expressed genes_keloid-XL_ comparing SC-Keloid vs. (SC-Myel, SC-Nonmyel, SC-Nonmyel2)								
**gene**	**p_val**	**avg_log2FC**	**pct.1**	**pct.2**	**p_val_adj**	**Foldchange_UP**	**Foldchange_DOWN**	**Ratio_pct1_pct2**
LINC00685	0,005677957	0,028704596	0,122	0,039	1	1,020095764	0,98030012	3,128205128
H3C10	0,065143806	0,028676058	0,024	0,004	1	1,020075586	0,980319511	6
AFP	0,063203483	0,028636864	0,012	0	1	1,020047874	0,980346144	#DIV/0!
IRX1	0,063203483	0,028636864	0,012	0	1	1,020047874	0,980346144	#DIV/0!
BUB1B	0,063203483	0,028633369	0,012	0	1	1,020045403	0,980348519	#DIV/0!
DIAPH3	0,063203483	0,028633369	0,012	0	1	1,020045403	0,980348519	#DIV/0!
STAP2	0,011553628	0,028627578	0,037	0,004	1	1,020041308	0,980352454	9,25
PCDHGB6	0,00931978	0,028592703	0,049	0,007	1	1,020016651	0,980376153	7
MRM1	0,006523076	0,028554316	0,061	0,011	1	1,019989511	0,980402239	5,545454545
B3GNT9	0,017068103	0,028498111	0,061	0,014	1	1,019949774	0,980440435	4,357142857
C16orf95	0,00931978	0,028446328	0,049	0,007	1	1,019913166	0,980475627	7
CEMIP	0,065143806	0,02842968	0,024	0,004	1	1,019901397	0,980486941	6
ADAMTS14	0,063203483	0,028327928	0,012	0	1	1,019829467	0,980556096	#DIV/0!
COL4A6	0,063203483	0,028327928	0,012	0	1	1,019829467	0,980556096	#DIV/0!
RN7SL832P	0,063203483	0,028327928	0,012	0	1	1,019829467	0,980556096	#DIV/0!
WAS	0,063203483	0,028327928	0,012	0	1	1,019829467	0,980556096	#DIV/0!
XIRP1	0,063203483	0,028327928	0,012	0	1	1,019829467	0,980556096	#DIV/0!
SPC24	0,008401736	0,028308392	0,024	0	1	1,019815657	0,980569374	#DIV/0!
CMTM1	0,066254792	0,028289244	0,024	0,004	1	1,019802121	0,980582389	6
KRTAP2-2	0,347088417	0,028246026	0,012	0,004	1	1,019771572	0,980611764	3
GPATCH4	7,57218E-07	0,028201351	0,183	0,028	0,015978058	1,019739994	0,98064213	6,535714286
MFSD14B	0,001574353	0,028184997	0,244	0,095	1	1,019728434	0,980653247	2,568421053
PEX19	0,138914913	0,028174309	0,085	0,042	1	1,01972088	0,980660512	2,023809524
C1orf112	0,017481575	0,028172162	0,061	0,014	1	1,019719362	0,980661971	4,357142857
BMP8A	0,347088417	0,028158238	0,012	0,004	1	1,019709521	0,980671436	3
ME1	0,001952737	0,028150401	0,049	0,004	1	1,019703982	0,980676763	12,25
TPM3	0,027602734	0,028142021	0,683	0,404	1	1,019698058	0,98068246	1,690594059
MAPK13	0,065143806	0,028136456	0,024	0,004	1	1,019694125	0,980686242	6
THAP4	0,00014217	0,028128942	0,171	0,042	1	1,019688814	0,98069135	4,071428571
MRPS22	0,000280656	0,028111655	0,244	0,081	1	1,019676596	0,980703101	3,012345679

Supplementary Table 2

Differentially expressed genes_keloid-XL_ comparing SC-Keloid vs. (SC-Myel, SC-Nonmyel, SC-Nonmyel2)								
**gene**	**p_val**	**avg_log2FC**	**pct.1**	**pct.2**	**p_val_adj**	**Foldchange_UP**	**Foldchange_DOWN**	**Ratio_pct1_pct2**
DAB2	0,010546634	0,028088805	0,073	0,018	1	1,019660446	0,980718634	4,055555556
ZSCAN2	0,011339437	0,028030737	0,037	0,004	1	1,019619406	0,980758109	9,25
MAST3	0,739733711	0,027952899	0,037	0,046	1	1,019564396	0,980811025	0,804347826
TCTN2	0,017691584	0,027909794	0,061	0,014	1	1,019533934	0,98084033	4,357142857
USP39	0,000449575	0,027885186	0,244	0,088	1	1,019516543	0,980857061	2,772727273
TICAM1	0,038046743	0,027823434	0,11	0,046	1	1,019472906	0,980899045	2,391304348
ST7-AS1	0,044226311	0,02779716	0,037	0,007	1	1,01945434	0,980916909	5,285714286
KRT86	0,008401736	0,02777981	0,024	0	1	1,01944208	0,980928706	#DIV/0!
TXNDC11	0,105099631	0,027767473	0,122	0,063	1	1,019433362	0,980937094	1,936507937
RPGRIP1L	0,006615795	0,027740827	0,061	0,011	1	1,019414534	0,980955212	5,545454545
ARPP19	0,006012214	0,027726583	0,659	0,354	1	1,019404469	0,980964897	1,861581921
NXT1	0,002167609	0,027656035	0,39	0,179	1	1,019354621	0,981012868	2,17877095
ATP6V0C	0,223927175	0,027650725	0,841	0,6	1	1,019350869	0,981016478	1,401666667
IGFL4	0,008401736	0,027568461	0,024	0	1	1,019292746	0,981072419	#DIV/0!
SUSD5	0,008401736	0,027568461	0,024	0	1	1,019292746	0,981072419	#DIV/0!
FXYD2	0,008401736	0,02756167	0,024	0	1	1,019287948	0,981077037	#DIV/0!
POLR2B	0,000131858	0,0275507	0,378	0,147	1	1,019280198	0,981084497	2,571428571
CCDC88A	0,000194502	0,027544769	0,195	0,056	1	1,019276008	0,98108853	3,482142857
SENP8	0,001220269	0,027541144	0,037	0	1	1,019273446	0,981090995	#DIV/0!
GMIP	0,008401736	0,027518198	0,024	0	1	1,019257235	0,981106599	#DIV/0!
LRRN3	0,644071107	0,027503277	0,012	0,007	1	1,019246693	0,981116747	1,714285714
RFT1	0,011168661	0,027499537	0,073	0,018	1	1,019244051	0,98111929	4,055555556
ZNF862	0,025203577	0,027475941	0,085	0,028	1	1,019227381	0,981135337	3,035714286
UBE3C	5,91048E-06	0,027454281	0,244	0,06	0,124717129	1,019212079	0,981150067	4,066666667
NDST3	0,011553628	0,027453357	0,037	0,004	1	1,019211426	0,981150696	9,25
ARMCX5	0,002909566	0,027450829	0,085	0,018	1	1,019209641	0,981152415	4,722222222
PEX12	0,043609761	0,027432528	0,037	0,007	1	1,019196712	0,981164861	5,285714286
FAM161B	0,026685693	0,02741823	0,049	0,011	1	1,019186611	0,981174585	4,454545455
FDX1	0,000102459	0,02737596	0,415	0,161	1	1,01915675	0,981203333	2,577639752
KLF12	0,036803449	0,027347445	0,061	0,018	1	1,019136606	0,981222727	3,388888889

Supplementary Table 2

Differentially expressed genes_keloid-XL_ comparing SC-Keloid vs. (SC-Myel, SC-Nonmyel, SC-Nonmyel2)								
gene	p_val	avg_log2FC	pct.1	pct.2	p_val_adj	Foldchange_UP	Foldchange_DOWN	Ratio_pct1_pct2
RAD51AP1	0,183494953	0,027339638	0,024	0,007	1	1,019131091	0,981228037	3,428571429
SEC24B	0,129105122	0,027328584	0,122	0,067	1	1,019123283	0,981235555	1,820895522
COL2A1	0,063203483	0,027326916	0,012	0	1	1,019122104	0,98123669	#DIV/0!
ARPP21	0,063203483	0,027307828	0,012	0	1	1,019108621	0,981249672	#DIV/0!
TMEM89	0,063203483	0,027307828	0,012	0	1	1,019108621	0,981249672	#DIV/0!
ZFP69B	0,065143806	0,027300075	0,024	0,004	1	1,019103144	0,981254946	6
CCNB1	0,027726954	0,027274284	0,049	0,011	1	1,019084926	0,981272488	4,454545455
GNPNAT1	0,011803785	0,027240185	0,134	0,049	1	1,01906084	0,98129568	2,734693878
SH3PXD2A	0,005151743	0,027239497	0,756	0,446	1	1,019060353	0,981296149	1,695067265
PRMT3	0,03795203	0,027215108	0,061	0,018	1	1,019043126	0,981312738	3,388888889
TIAM1	0,026685693	0,027112222	0,049	0,011	1	1,018970456	0,981382723	4,454545455
ZNF74	0,067616925	0,027082204	0,061	0,021	1	1,018949254	0,981403142	2,904761905
REEP6	0,000486185	0,027070943	0,11	0,021	1	1,0189413	0,981410803	5,238095238
ANKRD20A3P	0,063203483	0,02704338	0,012	0	1	1,018921834	0,981429553	#DIV/0!
ARMC12	0,063203483	0,02704338	0,012	0	1	1,018921834	0,981429553	#DIV/0!
CRCT1	0,063203483	0,02704338	0,012	0	1	1,018921834	0,981429553	#DIV/0!
DERL3	0,063203483	0,02704338	0,012	0	1	1,018921834	0,981429553	#DIV/0!
NTS	0,063203483	0,02704338	0,012	0	1	1,018921834	0,981429553	#DIV/0!
TDRD3	0,004165279	0,027020556	0,22	0,091	1	1,018905714	0,98144508	2,417582418
ISM1	0,347088417	0,027002603	0,012	0,004	1	1,018893035	0,981457293	3
RANBP17	0,001833348	0,027000821	0,061	0,007	1	1,018891777	0,981458505	8,714285714
GRIPAP1	0,077011555	0,026901393	0,11	0,053	1	1,018821559	0,981526148	2,075471698
LRIT3	0,063203483	0,026894554	0,012	0	1	1,018816729	0,981530801	#DIV/0!
TMEM97	0,105197534	0,026890543	0,073	0,032	1	1,018813897	0,98153353	2,28125
GMEB2	0,009031726	0,026888266	0,049	0,007	1	1,018812288	0,981535079	7
TTC30B	0,185668317	0,026864614	0,024	0,007	1	1,018795586	0,981551171	3,428571429
SHC2	0,649511996	0,026862587	0,012	0,007	1	1,018794155	0,981552549	1,714285714
ARL17A	0,180829015	0,026854223	0,049	0,021	1	1,018788248	0,98155824	2,333333333
RSL1D1	0,024899155	0,026828792	0,463	0,263	1	1,01877029	0,981575543	1,760456274
ZNF674-AS1	0,00931978	0,026791377	0,049	0,007	1	1,018743869	0,981600999	7

Supplementary Table 2

Differentially expressed genes_keloid-XL_ comparing SC-Keloid vs. (SC-Myel, SC-Nonmyel, SC-Nonmyel2)								
**gene**	**p_val**	**avg_log2FC**	**pct.1**	**pct.2**	**p_val_adj**	**Foldchange_UP**	**Foldchange_DOWN**	**Ratio_pct1_pct2**
PAOX	0,044226311	0,026786515	0,037	0,007	1	1,018740436	0,981604307	5,285714286
NDUFA4L2	0,345067611	0,026754505	0,024	0,011	1	1,018717832	0,981626087	2,181818182
CCSER1	0,008401736	0,026731939	0,024	0	1	1,018701898	0,981641442	#DIV/0!
CCDC92	0,000181982	0,026713498	0,22	0,067	1	1,018688877	0,98165399	3,28358209
NECAB1	0,348081613	0,026677695	0,024	0,011	1	1,018663597	0,981678351	2,181818182
EFHC1	0,001013979	0,026676625	0,341	0,147	1	1,018662842	0,981679079	2,319727891
MEPE	0,001952737	0,026633653	0,049	0,004	1	1,0186325	0,98170832	12,25
ZNF583	0,02737604	0,026621083	0,049	0,011	1	1,018623625	0,981716873	4,454545455
DOCK8-AS1	0,008401736	0,02661115	0,024	0	1	1,018616612	0,981723632	#DIV/0!
HOTAIRM1	0,695525935	0,026595485	0,024	0,018	1	1,018605551	0,981734292	1,333333333
FAP	0,103792275	0,026580417	0,037	0,011	1	1,018594913	0,981744546	3,363636364
MAN1C1	0,002612361	0,026535486	0,11	0,028	1	1,01856319	0,981775122	3,928571429
ZNF430	0,008726393	0,026504031	0,098	0,028	1	1,018540983	0,981796527	3,5
MDFI	0,006993141	0,026436051	0,146	0,053	1	1,01849299	0,981842791	2,754716981
CPOX	0,001055888	0,026421182	0,085	0,014	1	1,018482494	0,98185291	6,071428571
FKBP14	0,011604277	0,026413577	0,146	0,056	1	1,018477125	0,981858086	2,607142857
RAB11B-AS1	0,06274641	0,026390561	0,049	0,014	1	1,018460876	0,98187375	3,5
ADAMTS5	0,196612832	0,026383983	0,037	0,014	1	1,018456232	0,981878227	2,642857143
PIFO	0,000183568	0,026335743	0,049	0	1	1,018422178	0,981911059	#DIV/0!
SLC38A7	0,179467859	0,026259976	0,049	0,021	1	1,018368695	0,981962628	2,333333333
KYAT1	0,066254792	0,026213944	0,024	0,004	1	1,018336202	0,98199396	6
FZD3	0,178775462	0,026186129	0,134	0,081	1	1,018316569	0,982012893	1,654320988
MAPK1IP1L	1,36192E-05	0,026133702	0,378	0,13	0,287378965	1,018279564	0,98204858	2,907692308
PKP3	0,008401736	0,026129797	0,024	0	1	1,018276808	0,982051238	#DIV/0!
FAM85B	0,347088417	0,026111951	0,012	0,004	1	1,018264212	0,982063386	3
CYCS	0,00093246	0,026102731	0,634	0,33	1	1,018257705	0,982069662	1,921212121
COX15	0,015327249	0,026098815	0,098	0,032	1	1,018254941	0,982072327	3,0625
SUPT6H	0,004954407	0,026083814	0,207	0,084	1	1,018244353	0,982082539	2,464285714
TUB	0,011553628	0,026062703	0,037	0,004	1	1,018229454	0,98209691	9,25
SP2	0,018247048	0,026030879	0,122	0,046	1	1,018206993	0,982118574	2,652173913

Supplementary Table 2

Differentially expressed genes_keloid-XL_ comparing SC-Keloid vs. (SC-Myel, SC-Nonmyel, SC-Nonmyel2)								
gene	p_val	avg_log2FC	pct.1	pct.2	p_val_adj	Foldchange_UP	Foldchange_DOWN	Ratio_pct1_pct2
TSPYL5	0,005290483	0,025976648	0,11	0,032	1	1,018168719	0,982155493	3,4375
FAM228A	0,008401736	0,025958225	0,024	0	1	1,018155718	0,982168034	#DIV/0!
AC026202.3	0,066254792	0,025941132	0,024	0,004	1	1,018143654	0,982179671	6
TNNT2	0,063203483	0,025895029	0,012	0	1	1,018111119	0,982211059	#DIV/0!
HAPLN2	0,066254792	0,025845776	0,024	0,004	1	1,018076362	0,982244592	6
CKB	0,088467486	0,02583006	0,451	0,309	1	1,018065271	0,982255292	1,459546926
RTKN	0,000148287	0,025825613	0,402	0,158	1	1,018062133	0,98225832	2,544303797
RGMB-AS1	0,104939896	0,025811966	0,037	0,011	1	1,018052503	0,982267611	3,363636364
SLCO2B1	0,065143806	0,025748477	0,024	0,004	1	1,018007702	0,982310839	6
EVC2	0,348081613	0,025747329	0,024	0,011	1	1,018006892	0,982311621	2,181818182
TMEM216	0,002449793	0,025745222	0,11	0,028	1	1,018005406	0,982313055	3,928571429
TLR6	0,044226311	0,025740033	0,037	0,007	1	1,018001744	0,982316588	5,285714286
SNX14	0,003256641	0,025732566	0,207	0,081	1	1,017996475	0,982321672	2,555555556
SETX	0,00297168	0,025578406	0,293	0,13	1	1,017887702	0,982426645	2,253846154
ME3	0,000608755	0,025577135	0,195	0,063	1	1,017886805	0,98242751	3,095238095
RNF170	0,000230869	0,025494703	0,098	0,014	1	1,017828648	0,982483645	7
SLC8A2	0,347088417	0,025484484	0,012	0,004	1	1,017821438	0,982490604	3
DNAJC3	0,004853657	0,025441937	0,476	0,253	1	1,017791421	0,98251958	1,881422925
TCF19	0,339089703	0,025385099	0,024	0,011	1	1,017751325	0,982558289	2,181818182
RDH8	0,008401736	0,0253683	0,024	0	1	1,017739473	0,98256973	#DIV/0!
C4orf47	0,011553628	0,025300307	0,037	0,004	1	1,01769151	0,982616039	9,25
ZNF180	0,066254792	0,025290566	0,024	0,004	1	1,017684638	0,982622673	6
NBPF9	0,001445239	0,025233914	0,073	0,011	1	1,017644676	0,98266126	6,636363636
MAPK11	0,065143806	0,02511084	0,024	0,004	1	1,017557866	0,982745093	6
BAG1	0,014569513	0,02506365	0,378	0,204	1	1,017524583	0,982777239	1,852941176
SMYD3	8,11676E-05	0,025056673	0,134	0,025	1	1,017519662	0,982781991	5,36
FAM104A	0,001696084	0,025054749	0,171	0,056	1	1,017518305	0,982783302	3,053571429
FUNDC1	1,40255E-05	0,025054677	0,195	0,042	0,29595285	1,017518254	0,982783351	4,642857143
RAB22A	0,00035081	0,025029653	0,341	0,137	1	1,017500606	0,982800398	2,489051095
DENND1A	0,03795203	0,024998333	0,061	0,018	1	1,017478517	0,982821734	3,388888889

Supplementary Table 2

Differentially expressed genes_keloid-XL_ comparing SC-Keloid vs. (SC-Myel, SC-Nonmyel, SC-Nonmyel2)								
**gene**	**p_val**	**avg_log2FC**	**pct.1**	**pct.2**	**p_val_adj**	**Foldchange_UP**	**Foldchange_DOWN**	**Ratio_pct1_pct2**
SLC36A1	0,06274641	0,024995648	0,049	0,014	1	1,017476623	0,982823563	3,5
FDXACB1	0,044226311	0,024963065	0,037	0,007	1	1,017453644	0,98284576	5,285714286
ETAA1	0,001833348	0,024920521	0,061	0,007	1	1,01742364	0,982874744	8,714285714
RELL2	0,011553628	0,024918505	0,037	0,004	1	1,017422218	0,982876118	9,25
SLC25A10	0,184958462	0,024898113	0,049	0,021	1	1,017407837	0,982890011	2,333333333
NCAPG2	0,063408785	0,024864428	0,049	0,014	1	1,017384083	0,98291296	3,5
PITHD1	2,9328E-05	0,024849685	0,476	0,189	0,618850375	1,017373686	0,982923004	2,518518519
SNAPC1	0,000207953	0,024831011	0,195	0,056	1	1,017360517	0,982935727	3,482142857
LINC00654	0,011553628	0,024762228	0,037	0,004	1	1,017312014	0,982982592	9,25
FIBCD1	0,063203483	0,02475112	0,012	0	1	1,017304182	0,98299016	#DIV/0!
FOXD2-AS1	0,107265343	0,02472348	0,037	0,011	1	1,017284692	0,983008993	3,363636364
SIRT4	0,008401736	0,024723027	0,024	0	1	1,017284372	0,983009302	#DIV/0!
ANKRD22	0,063203483	0,024676481	0,012	0	1	1,017251552	0,983041017	#DIV/0!
VTN	0,063203483	0,024676481	0,012	0	1	1,017251552	0,983041017	#DIV/0!
ZNF222	0,183494953	0,024670583	0,024	0,007	1	1,017247393	0,983045036	3,428571429
SLC25A14	0,002983177	0,024616485	0,085	0,018	1	1,017209249	0,983081899	4,722222222
ZNF805	0,000225277	0,02461221	0,134	0,028	1	1,017206235	0,983084812	4,785714286
PRSS12	0,008401736	0,024595058	0,024	0	1	1,017194142	0,9830965	#DIV/0!
PCDHGA4	0,066254792	0,024577716	0,024	0,004	1	1,017181915	0,983108317	6
CCDC88B	0,001952737	0,024558485	0,049	0,004	1	1,017168355	0,983121422	12,25
TRDMT1	0,004092775	0,024555792	0,134	0,042	1	1,017166457	0,983123257	3,19047619
CYB5R1	5,96828E-06	0,024548998	0,22	0,049	0,125936664	1,017161667	0,983127887	4,489795918
NCOA5	0,067616925	0,024523203	0,061	0,021	1	1,01714348	0,983145465	2,904761905
ENPP1	0,028081741	0,02449238	0,049	0,011	1	1,017121749	0,98316647	4,454545455
ASB9	0,008401736	0,024473567	0,024	0	1	1,017108486	0,983179291	#DIV/0!
APOL2	0,010480408	0,024465557	0,122	0,042	1	1,017102839	0,983184749	2,904761905
PAN3-AS1	0,347088417	0,024443438	0,012	0,004	1	1,017087245	0,983199824	3
ETV2	0,001952737	0,024422971	0,049	0,004	1	1,017072816	0,983213772	12,25
GGACT	0,011553628	0,024418925	0,037	0,004	1	1,017069964	0,983216529	9,25
FIZ1	0,001723931	0,024390689	0,098	0,021	1	1,017050058	0,983235773	4,666666667

Supplementary Table 2

Differentially expressed genes_keloid-XL_ comparing SC-Keloid vs. (SC-Myel, SC-Nonmyel, SC-Nonmyel2)								
**gene**	**p_val**	**avg_log2FC**	**pct.1**	**pct.2**	**p_val_adj**	**Foldchange_UP**	**Foldchange_DOWN**	**Ratio_pct1_pct2**
LGALS4	0,066254792	0,024337744	0,024	0,004	1	1,017012735	0,983271857	6
BTBD9	0,069438782	0,024335933	0,061	0,021	1	1,017011458	0,983273091	2,904761905
DNAAF5	0,006596991	0,024325066	0,134	0,046	1	1,017003797	0,983280498	2,913043478
KIAA1522	0,117026191	0,024318209	0,049	0,018	1	1,016998963	0,983285171	2,722222222
SNAP23	7,10704E-05	0,024303258	0,268	0,084	1	1,016988424	0,983295361	3,19047619
CLINT1	0,004010237	0,024280289	0,244	0,105	1	1,016972233	0,983311016	2,323809524
DUSP19	0,011553628	0,024277616	0,037	0,004	1	1,016970349	0,983312838	9,25
DLL3	0,35185965	0,0242157	0,012	0,004	1	1,016926704	0,98335504	3
SLC25A38	0,027124406	0,024178004	0,11	0,042	1	1,016900133	0,983380734	2,619047619
MLLT11	2,31391E-05	0,02414686	0,244	0,067	0,488258256	1,016878181	0,983401963	3,641791045
FANK1	0,008401736	0,024132663	0,024	0	1	1,016868175	0,98341164	#DIV/0!
ALKAL2	0,063203483	0,024111704	0,012	0	1	1,016853402	0,983425927	#DIV/0!
ZNF431	0,001492437	0,024099302	0,171	0,056	1	1,016844661	0,983434381	3,053571429
ARHGAP20	0,066254792	0,024098433	0,024	0,004	1	1,016844049	0,983434973	6
CAPN15	0,010349311	0,024073552	0,134	0,049	1	1,016826512	0,983451934	2,734693878
FAM184B	0,001026381	0,024068951	0,085	0,014	1	1,01682327	0,98345507	6,071428571
ATL1	0,002751905	0,024058765	0,171	0,06	1	1,01681609	0,983462014	2,85
RARRES1	0,044226311	0,024053089	0,037	0,007	1	1,01681209	0,983465883	5,285714286
DKC1	0,002957421	0,02398755	0,171	0,06	1	1,016765899	0,983510562	2,85
IDNK	0,112963039	0,023949266	0,049	0,018	1	1,016738918	0,98353666	2,722222222
ZBTB45	0,060793535	0,023948054	0,049	0,014	1	1,016738064	0,983537487	3,5
DAPL1	0,006523076	0,023920596	0,061	0,011	1	1,016718713	0,983556206	5,545454545
PABIR1	0,011168661	0,023912455	0,073	0,018	1	1,016712976	0,983561756	4,055555556
ZNF90	0,106097567	0,023911741	0,037	0,011	1	1,016712472	0,983562243	3,363636364
CHCHD4	0,006483581	0,023909242	0,085	0,021	1	1,016710712	0,983563946	4,047619048
ZNF614	0,187860656	0,02390213	0,024	0,007	1	1,016705699	0,983568796	3,428571429
HDAC5	0,036746792	0,02387422	0,195	0,098	1	1,01668603	0,983587824	1,989795918
RAP2C-AS1	0,006615795	0,023830544	0,061	0,011	1	1,016655252	0,983617601	5,545454545
ALG8	5,96828E-06	0,023730302	0,22	0,049	0,125936664	1,016584615	0,983685947	4,489795918
STX1A	0,026685693	0,023702773	0,049	0,011	1	1,016565217	0,983704718	4,454545455

Supplementary Table 2

Differentially expressed genes_keloid-XL_ comparing SC-Keloid vs. (SC-Myel, SC-Nonmyel, SC-Nonmyel2)								
**gene**	**p_val**	**avg_log2FC**	**pct.1**	**pct.2**	**p_val_adj**	**Foldchange_UP**	**Foldchange_DOWN**	**Ratio_pct1_pct2**
PTGR2	0,105197534	0,023690033	0,073	0,032	1	1,01655624	0,983713405	2,28125
PSTK	0,006704008	0,023682635	0,085	0,021	1	1,016551027	0,983718449	4,047619048
TNKS2	9,88338E-05	0,023604482	0,341	0,123	1	1,01649596	0,98377174	2,772357724
KLC2	0,001236845	0,023598343	0,11	0,025	1	1,016491635	0,983775926	4,4
AFAP1-AS1	0,063203483	0,023597494	0,012	0	1	1,016491037	0,983776505	#DIV/0!
C8G	0,063203483	0,023597494	0,012	0	1	1,016491037	0,983776505	#DIV/0!
CD200R1	0,063203483	0,023597494	0,012	0	1	1,016491037	0,983776505	#DIV/0!
CFP	0,063203483	0,023597494	0,012	0	1	1,016491037	0,983776505	#DIV/0!
F5	0,063203483	0,023597494	0,012	0	1	1,016491037	0,983776505	#DIV/0!
IL15RA	0,063203483	0,023597494	0,012	0	1	1,016491037	0,983776505	#DIV/0!
LCN10	0,063203483	0,023597494	0,012	0	1	1,016491037	0,983776505	#DIV/0!
OSM	0,063203483	0,023597494	0,012	0	1	1,016491037	0,983776505	#DIV/0!
PCDH19	0,063203483	0,023597494	0,012	0	1	1,016491037	0,983776505	#DIV/0!
PLSCR2	0,063203483	0,023597494	0,012	0	1	1,016491037	0,983776505	#DIV/0!
RAB3B	0,063203483	0,023597494	0,012	0	1	1,016491037	0,983776505	#DIV/0!
RIPOR2	0,063203483	0,023597494	0,012	0	1	1,016491037	0,983776505	#DIV/0!
SEC24B-AS1	0,063203483	0,023597494	0,012	0	1	1,016491037	0,983776505	#DIV/0!
SLC1A1	0,063203483	0,023597494	0,012	0	1	1,016491037	0,983776505	#DIV/0!
LINC00303	0,008401736	0,023564339	0,024	0	1	1,016467677	0,983799114	#DIV/0!
SRXN1	0,021972638	0,023541609	0,073	0,021	1	1,016451662	0,983814614	3,476190476
PARG	0,06642434	0,023540044	0,061	0,021	1	1,01645056	0,983815681	2,904761905
CCDC122	0,113968254	0,023535626	0,049	0,018	1	1,016447447	0,983818694	2,722222222
ACP2	7,20735E-05	0,0234654	0,134	0,025	1	1,016397971	0,983866585	5,36
RNF121	0,015742889	0,02344621	0,098	0,032	1	1,016384451	0,983879672	3,0625
PHEX	0,001220269	0,023436116	0,037	0	1	1,01637734	0,983886555	#DIV/0!
FAM131B	0,063203483	0,023435612	0,012	0	1	1,016376985	0,983886899	#DIV/0!
FBXO24	0,063203483	0,023435612	0,012	0	1	1,016376985	0,983886899	#DIV/0!
RIPOR3	0,106097567	0,023417132	0,037	0,011	1	1,016363966	0,983899502	3,363636364
SKA3	0,008401736	0,023361174	0,024	0	1	1,016324545	0,983937666	#DIV/0!
ZNF613	0,008401736	0,023361174	0,024	0	1	1,016324545	0,983937666	#DIV/0!

Supplementary Table 2

Differentially expressed genes_keloid-XL_ comparing SC-Keloid vs. (SC-Myel, SC-Nonmyel, SC-Nonmyel2)								
gene	p_val	avg_log2FC	pct.1	pct.2	p_val_adj	Foldchange_UP	Foldchange_DOWN	Ratio_pct1_pct2
CCDC189	0,009174744	0,023323924	0,049	0,007	1	1,016298304	0,983963071	7
CHTOP	7,02824E-05	0,023314518	0,415	0,165	1	1,016291678	0,983969486	2,515151515
CNDP2	0,000838973	0,02330031	0,159	0,046	1	1,016281669	0,983979176	3,456521739
TNPO3	0,008146582	0,023245542	0,171	0,067	1	1,016243089	0,984016531	2,552238806
PPL	0,008401736	0,023234162	0,024	0	1	1,016235073	0,984024294	#DIV/0!
AC011043.1	0,067051517	0,023226678	0,073	0,028	1	1,016229802	0,984029398	2,607142857
TFR2	0,066254792	0,023184823	0,024	0,004	1	1,016200319	0,984057947	6
TMEM161A	0,001474664	0,023141308	0,146	0,042	1	1,016169669	0,984087629	3,476190476
GUCD1	0,000195581	0,023128265	0,183	0,049	1	1,016160482	0,984096526	3,734693878
FAM66B	0,008401736	0,023105716	0,024	0	1	1,0161446	0,984111907	#DIV/0!
MFF	5,13464E-05	0,023094746	0,402	0,154	1	1,016136874	0,98411939	2,61038961
C1RL-AS1	0,044226311	0,023001959	0,037	0,007	1	1,016071523	0,984182686	5,285714286
ALKBH3	0,000323801	0,022996106	0,122	0,025	1	1,0160674	0,984186679	4,88
SLC16A5	0,011553628	0,022907365	0,037	0,004	1	1,016004903	0,984247218	9,25
RADX	0,027465648	0,022904982	0,098	0,035	1	1,016003225	0,984248844	2,8
PEX11G	0,004311516	0,022844933	0,073	0,014	1	1,015960937	0,984289812	5,214285714
VGF	0,066254792	0,022836531	0,024	0,004	1	1,015955021	0,984295544	6
ZNF785	0,044226311	0,02283318	0,037	0,007	1	1,015952661	0,98429783	5,285714286
PSMB8-AS1	0,011553628	0,022819881	0,037	0,004	1	1,015943296	0,984306904	9,25
BORCS7	5,14053E-06	0,022792542	0,341	0,105	0,108470277	1,015924043	0,984325557	3,247619048
ASCC2	0,000313622	0,022753593	0,183	0,053	1	1,015896617	0,984352132	3,452830189
GPR137B	0,002551837	0,022753038	0,402	0,193	1	1,015896226	0,98435251	2,082901554
NAGS	0,065143806	0,022743378	0,024	0,004	1	1,015889424	0,984359101	6
PHYHD1	0,066380027	0,022628843	0,085	0,035	1	1,015808776	0,984437252	2,428571429
ERCC3	0,004475867	0,022592389	0,171	0,063	1	1,015783109	0,984462127	2,714285714
FAM71E1	0,065143806	0,022558884	0,024	0,004	1	1,015759519	0,98448499	6
ALKBH4	0,021972638	0,022553894	0,073	0,021	1	1,015756005	0,984488396	3,476190476
PPP1R3B	0,183574298	0,022526525	0,049	0,021	1	1,015736736	0,984507072	2,333333333
ZFP90	0,003236838	0,022522918	0,122	0,035	1	1,015734196	0,984509534	3,485714286
PCDH12	0,065143806	0,022509272	0,024	0,004	1	1,015724589	0,984518846	6

Supplementary Table 2

Differentially expressed genes_keloid-XL_ comparing SC-Keloid vs. (SC-Myel, SC-Nonmyel, SC-Nonmyel2)								
**gene**	**p_val**	**avg_log2FC**	**pct.1**	**pct.2**	**p_val_adj**	**Foldchange_UP**	**Foldchange_DOWN**	**Ratio_pct1_pct2**
SFXN1	0,003332322	0,022497023	0,122	0,035	1	1,015715965	0,984527205	3,485714286
LRRC17	0,001220269	0,02249574	0,037	0	1	1,015715061	0,984528081	#DIV/0!
AURKC	0,187860656	0,022492344	0,024	0,007	1	1,015712671	0,984530398	3,428571429
IBA57	0,567510207	0,022487232	0,037	0,025	1	1,015709072	0,984533887	1,48
TUBA4A	0,68014566	0,022471293	0,061	0,049	1	1,01569785	0,984544764	1,244897959
SLC25A13	0,000133512	0,022459125	0,207	0,06	1	1,015689283	0,984553068	3,45
DMGDH	0,347088417	0,022451147	0,012	0,004	1	1,015683667	0,984558512	3
RAVER1	0,174061207	0,022417967	0,134	0,077	1	1,015660308	0,984581156	1,74025974
ZDHHC13	0,006615795	0,02238784	0,061	0,011	1	1,015639099	0,984601716	5,545454545
KIF22	0,015687817	0,022384258	0,183	0,081	1	1,015636577	0,984604161	2,259259259
MAPK12	0,183494953	0,022358118	0,024	0,007	1	1,015618175	0,984622002	3,428571429
FAM220A	0,063203483	0,022341902	0,012	0	1	1,01560676	0,984633068	#DIV/0!
LRRC63	0,063203483	0,022341902	0,012	0	1	1,01560676	0,984633068	#DIV/0!
SORL1	0,063203483	0,022341902	0,012	0	1	1,01560676	0,984633068	#DIV/0!
WWTR1-AS1	0,063203483	0,022341902	0,012	0	1	1,01560676	0,984633068	#DIV/0!
NEK11	0,000298361	0,022320905	0,085	0,011	1	1,015591978	0,984647399	7,727272727
C15orf65	0,906339215	0,022318433	0,012	0,014	1	1,015590238	0,984649086	0,857142857
AP2A1	8,91065E-05	0,022311206	0,293	0,098	1	1,01558515	0,984654019	2,989795918
WIPI2	0,00044542	0,022303242	0,305	0,119	1	1,015579544	0,984659454	2,56302521
CLOCK	0,000201128	0,022295382	0,232	0,074	1	1,015574012	0,984664819	3,135135135
ZNF117	0,013434952	0,02229479	0,085	0,025	1	1,015573595	0,984665223	3,4
ATG10	0,00269729	0,022257465	0,11	0,028	1	1,015547321	0,984690698	3,928571429
GDPGP1	0,066254792	0,022236406	0,024	0,004	1	1,015532496	0,984705072	6
KCNJ2-AS1	0,063203483	0,02220374	0,012	0	1	1,015509503	0,984727368	#DIV/0!
LUCAT1	0,063203483	0,02220374	0,012	0	1	1,015509503	0,984727368	#DIV/0!
NEK5	0,063203483	0,02220374	0,012	0	1	1,015509503	0,984727368	#DIV/0!
POT1-AS1	0,063203483	0,02220374	0,012	0	1	1,015509503	0,984727368	#DIV/0!
TMEM38A	0,063203483	0,02220374	0,012	0	1	1,015509503	0,984727368	#DIV/0!
CEP131	0,13246382	0,022184897	0,085	0,042	1	1,01549624	0,984740229	2,023809524
CMYA5	8,94415E-06	0,022147078	0,427	0,151	0,188730414	1,015469619	0,984766044	2,82781457

Supplementary Table 2

Differentially expressed genes_keloid-XL_ comparing SC-Keloid vs. (SC-Myel, SC-Nonmyel, SC-Nonmyel2)								
**gene**	**p_val**	**avg_log2FC**	**pct.1**	**pct.2**	**p_val_adj**	**Foldchange_UP**	**Foldchange_DOWN**	**Ratio_pct1_pct2**
EXTL3	0,001691389	0,022116866	0,122	0,032	1	1,015448354	0,984786667	3,8125
UTRN	0,010777728	0,022100724	0,671	0,375	1	1,015436993	0,984797685	1,789333333
ZNF696	0,008401736	0,022073443	0,024	0	1	1,015417791	0,984816308	#DIV/0!
GRAMD1C	0,35185965	0,022059888	0,012	0,004	1	1,015408251	0,98482556	3
DVL1	0,002020829	0,022039762	0,366	0,168	1	1,015394085	0,9848393	2,178571429
PCGF6	0,007226764	0,022020067	0,134	0,046	1	1,015380224	0,984852744	2,913043478
USP13	0,011168661	0,021983083	0,073	0,018	1	1,015354195	0,984877992	4,055555556
LENG8-AS1	0,695525935	0,021977264	0,024	0,018	1	1,0153501	0,984881964	1,333333333
FAXC	0,106097567	0,021885674	0,037	0,011	1	1,015285642	0,984944491	3,363636364
DLX3	0,063203483	0,021867055	0,012	0	1	1,015272538	0,984957203	#DIV/0!
COLGALT2	0,063203483	0,021829071	0,012	0	1	1,015245808	0,984983136	#DIV/0!
FAM180A	0,063203483	0,021829071	0,012	0	1	1,015245808	0,984983136	#DIV/0!
MYB	0,063203483	0,021829071	0,012	0	1	1,015245808	0,984983136	#DIV/0!
OSCP1	0,063203483	0,021829071	0,012	0	1	1,015245808	0,984983136	#DIV/0!
PLEKHN1	0,063203483	0,021829071	0,012	0	1	1,015245808	0,984983136	#DIV/0!
GPER1	0,00931978	0,021814016	0,049	0,007	1	1,015235214	0,984993414	7
UBE4A	0,034701433	0,021810161	0,207	0,105	1	1,015232501	0,984996047	1,971428571
LSR	0,008473224	0,021759408	0,098	0,028	1	1,015196787	0,985030698	3,5
RASA4	0,345067611	0,021702186	0,024	0,011	1	1,015156521	0,985069769	2,181818182
DNLZ	0,002645942	0,02168586	0,146	0,046	1	1,015145034	0,985080916	3,173913043
SH3RF2	0,011553628	0,021635992	0,037	0,004	1	1,015109945	0,985114967	9,25
NUDT18	0,066254792	0,021623778	0,024	0,004	1	1,015101351	0,985123307	6
ABCB9	0,004485159	0,02161251	0,073	0,014	1	1,015093423	0,985131001	5,214285714
NDUFAF7	0,064483381	0,021494532	0,085	0,035	1	1,015010416	0,985211565	2,428571429
RNGTT	0,000225277	0,021484722	0,134	0,028	1	1,015003514	0,985218264	4,785714286
ORAI1	0,000369356	0,021459859	0,341	0,133	1	1,014986022	0,985235243	2,563909774
ZSWIM4	0,000657963	0,021440008	0,098	0,018	1	1,014972056	0,9852488	5,444444444
TMEM107	4,41829E-05	0,02143104	0,244	0,07	0,932302481	1,014965747	0,985254924	3,485714286
SLC49A4	0,01104171	0,02142412	0,073	0,018	1	1,014960878	0,98525965	4,055555556
TMEM225B	0,063203483	0,021414578	0,012	0	1	1,014954165	0,985266167	#DIV/0!

Supplementary Table 2

Differentially expressed genes_keloid-XL_ comparing SC-Keloid vs. (SC-Myel, SC-Nonmyel, SC-Nonmyel2)								
gene	p_val	avg_log2FC	pct.1	pct.2	p_val_adj	Foldchange_UP	Foldchange_DOWN	Ratio_pct1_pct2
DOCK3	0,649511996	0,021232494	0,012	0,007	1	1,014826075	0,985390526	1,714285714
TSTD2	0,13405442	0,021213522	0,085	0,042	1	1,01481273	0,985403484	2,023809524
SPAG16	0,035868595	0,021203716	0,159	0,074	1	1,014805832	0,985410182	2,148648649
CCDC136	0,066254792	0,021119749	0,024	0,004	1	1,014746771	0,985467536	6
BRWD3	0,085377891	0,021117623	0,146	0,077	1	1,014745275	0,985468988	1,896103896
ABCB4	0,004426586	0,021110252	0,073	0,014	1	1,014740091	0,985474023	5,214285714
CYB561	0,06642434	0,021105307	0,061	0,021	1	1,014736613	0,985477401	2,904761905
H4C11	0,044226311	0,021101964	0,037	0,007	1	1,014734262	0,985479684	5,285714286
TMEM214	0,002803916	0,021080115	0,159	0,053	1	1,014718894	0,985494609	3
PIGH	5,66281E-05	0,021063738	0,195	0,049	1	1,014707375	0,985505797	3,979591837
DOCK2	0,00931978	0,021054475	0,049	0,007	1	1,01470086	0,985512124	7
BCL2L12	6,86914E-06	0,021051614	0,183	0,035	0,144945752	1,014698848	0,985514078	5,228571429
BLZF1	1,04518E-05	0,020977978	0,171	0,032	0,220543226	1,014647058	0,985564381	5,34375
ADORA1	0,008401736	0,020871824	0,024	0	1	1,014572403	0,985636902	#DIV/0!
GSTM4	0,005447223	0,02086631	0,11	0,032	1	1,014568526	0,985640669	3,4375
TMEM11	0,000151129	0,020855644	0,268	0,091	1	1,014561025	0,985647956	2,945054945
ZNF239	0,00032444	0,02082548	0,061	0,004	1	1,014539813	0,985668564	15,25
CACNA1C-AS2	0,063203483	0,020809223	0,012	0	1	1,01452838	0,985679671	#DIV/0!
DEGS2	0,063203483	0,020809223	0,012	0	1	1,01452838	0,985679671	#DIV/0!
MBL2	0,063203483	0,020809223	0,012	0	1	1,01452838	0,985679671	#DIV/0!
SIGLEC10	0,063203483	0,020809223	0,012	0	1	1,01452838	0,985679671	#DIV/0!
ETV1	0,125592074	0,020799295	0,098	0,049	1	1,014521398	0,985686454	2
FASTKD3	0,061438821	0,020791073	0,049	0,014	1	1,014515617	0,985692071	3,5
MICU3	0,00370964	0,020785767	0,134	0,042	1	1,014511886	0,985695697	3,19047619
DYNLT5	0,065143806	0,020785027	0,024	0,004	1	1,014511365	0,985696202	6
ZSCAN32	0,042398422	0,020780622	0,037	0,007	1	1,014508267	0,985699212	5,285714286
MED9	0,036803449	0,020767762	0,061	0,018	1	1,014499224	0,985707999	3,388888889
KIAA1549	0,906339215	0,020759076	0,012	0,014	1	1,014493116	0,985713934	0,857142857
ZNF441	0,114980497	0,020751184	0,049	0,018	1	1,014487567	0,985719326	2,722222222
HINT3	0,001662334	0,020738937	0,159	0,049	1	1,014478955	0,985727693	3,244897959

Supplementary Table 2

Differentially expressed genes_keloid-XL_ comparing SC-Keloid vs. (SC-Myel, SC-Nonmyel, SC-Nonmyel2)								
**gene**	**p_val**	**avg_log2FC**	**pct.1**	**pct.2**	**p_val_adj**	**Foldchange_UP**	**Foldchange_DOWN**	**Ratio_pct1_pct2**
MSS51	0,649511996	0,020730782	0,012	0,007	1	1,01447322	0,985733265	1,714285714
CLIP3	0,025659188	0,020723477	0,134	0,056	1	1,014468084	0,985738256	2,392857143
INAFM1	0,010288979	0,020644227	0,22	0,098	1	1,014412358	0,985792407	2,244897959
FGGY	0,014118687	0,020629396	0,085	0,025	1	1,01440193	0,98580254	3,4
RTEL1	0,066254792	0,020574472	0,024	0,004	1	1,014363312	0,985840071	6
STK33	0,063203483	0,020568378	0,012	0	1	1,014359027	0,985844236	#DIV/0!
LGI1	0,000894757	0,020546348	0,244	0,091	1	1,014343539	0,985859289	2,681318681
BTRC	0,017481575	0,020519282	0,061	0,014	1	1,014324509	0,985877785	4,357142857
ADAL	0,18978875	0,020513693	0,037	0,014	1	1,01432058	0,985881604	2,642857143
ITGB2	0,008401736	0,02051255	0,024	0	1	1,014319776	0,985882385	#DIV/0!
PRXL2B	0,001289912	0,020464937	0,146	0,042	1	1,014286301	0,985914923	3,476190476
DNAJC22	0,008401736	0,020461905	0,024	0	1	1,014284169	0,985916995	#DIV/0!
FAAP24	0,008401736	0,020447444	0,024	0	1	1,014274003	0,985926877	#DIV/0!
SNHG10	0,104067166	0,020336983	0,11	0,056	1	1,014196347	0,986002368	1,964285714
ARHGEF35	0,187860656	0,020297478	0,024	0,007	1	1,014168576	0,986029368	3,428571429
SUFU	0,069203207	0,020270869	0,073	0,028	1	1,014149871	0,986047554	2,607142857
ASTE1	0,113968254	0,020240535	0,049	0,018	1	1,014128548	0,986068287	2,722222222
NUP85	0,003879558	0,02023511	0,134	0,042	1	1,014124734	0,986071995	3,19047619
AL592528.1	0,063203483	0,020188386	0,012	0	1	1,01409189	0,986103931	#DIV/0!
ALG1L	0,063203483	0,020188386	0,012	0	1	1,01409189	0,986103931	#DIV/0!
LAT2	0,063203483	0,020188386	0,012	0	1	1,01409189	0,986103931	#DIV/0!
NLGN1-AS1	0,063203483	0,020188386	0,012	0	1	1,01409189	0,986103931	#DIV/0!
SFXN2	0,063203483	0,020188386	0,012	0	1	1,01409189	0,986103931	#DIV/0!
SPATA21	0,063203483	0,020188386	0,012	0	1	1,01409189	0,986103931	#DIV/0!
BEX2	0,008401736	0,020132191	0,024	0	1	1,014052391	0,986142343	#DIV/0!
UBA6-DT	0,000761457	0,020099409	0,122	0,028	1	1,014029349	0,98616475	4,357142857
AC019171.1	0,066254792	0,020083251	0,024	0,004	1	1,014017992	0,986175796	6
TMEM229B	0,04253036	0,020055221	0,085	0,032	1	1,013998291	0,986194956	2,65625
KHDC1	0,649511996	0,020044489	0,012	0,007	1	1,013990748	0,986202292	1,714285714
RNF11	0,000794756	0,02003924	0,463	0,218	1	1,013987059	0,98620588	2,123853211

Supplementary Table 2

Differentially expressed genes_keloid-XL_ comparing SC-Keloid vs. (SC-Myel, SC-Nonmyel, SC-Nonmyel2)								
**gene**	**p_val**	**avg_log2FC**	**pct.1**	**pct.2**	**p_val_adj**	**Foldchange_UP**	**Foldchange_DOWN**	**Ratio_pct1_pct2**
CS	0,003501912	0,020002529	0,195	0,074	1	1,013961257	0,986230976	2,635135135
FAM172A	0,003344358	0,019985203	0,305	0,137	1	1,01394908	0,98624282	2,226277372
MTRF1L	0,03501243	0,019943127	0,134	0,06	1	1,013919509	0,986271584	2,233333333
ATP5F1B	0,041866742	0,019846829	0,756	0,491	1	1,013851833	0,986337419	1,539714868
ZNF773	0,066254792	0,019821043	0,024	0,004	1	1,013833712	0,986355048	6
HES2	0,35185965	0,019810033	0,012	0,004	1	1,013825976	0,986362575	3
RPP30	7,65035E-08	0,019796392	0,317	0,074	0,0016143	1,01381639	0,986371901	4,283783784
ABCA7	0,011553628	0,019752349	0,037	0,004	1	1,01378544	0,986402015	9,25
LTO1	0,001236845	0,019741098	0,11	0,025	1	1,013777534	0,986409707	4,4
LIN7A	0,06274641	0,019727092	0,049	0,014	1	1,013767692	0,986419283	3,5
ANAPC7	0,002018358	0,019713254	0,183	0,063	1	1,013757968	0,986428745	2,904761905
ADAM22	0,008401736	0,019698339	0,024	0	1	1,013747487	0,986438943	#DIV/0!
LINC01551	0,347088417	0,019613624	0,012	0,004	1	1,013687962	0,986496868	3
GMNN	0,001302365	0,0195976	0,146	0,042	1	1,013676703	0,986507825	3,476190476
LDLRAD4	0,342070304	0,019516709	0,024	0,011	1	1,013619869	0,98656314	2,181818182
ARHGAP12	0,002270124	0,019482775	0,39	0,182	1	1,013596027	0,986586345	2,142857143
DNAJB5	0,009669783	0,019452049	0,11	0,035	1	1,01357444	0,986607357	3,142857143
TNFRSF10D	0,022646956	0,01941812	0,073	0,021	1	1,013550604	0,98663056	3,476190476
BMF	0,585233179	0,019416688	0,085	0,067	1	1,013549598	0,98663154	1,268656716
CTU1	0,001673714	0,019342352	0,122	0,032	1	1,013497375	0,986682378	3,8125
PCMTD1	0,005338226	0,019318479	0,329	0,161	1	1,013480604	0,986698706	2,043478261
LINC01556	0,008401736	0,019315898	0,024	0	1	1,013478791	0,986700471	#DIV/0!
GCNT2	0,898084959	0,019302441	0,012	0,011	1	1,013469338	0,986709674	1,090909091
PYROXD2	0,006523076	0,019291336	0,061	0,011	1	1,013461537	0,986717269	5,545454545
MT-ND5	0,478097036	0,019280901	0,659	0,509	1	1,013454206	0,986724406	1,294695481
LIG1	0,008751649	0,019255991	0,049	0,007	1	1,013436708	0,986741443	7
POC1B	0,039951184	0,019213207	0,11	0,046	1	1,013406655	0,986770706	2,391304348
EXOSC9	0,000197139	0,019162529	0,159	0,039	1	1,013371056	0,98680537	4,076923077
PLGLB1	0,35185965	0,019138222	0,012	0,004	1	1,013353983	0,986821995	3
MATN4	0,011553628	0,019133226	0,037	0,004	1	1,013350474	0,986825413	9,25

Supplementary Table 2

Differentially expressed genes_keloid-XL_ comparing SC-Keloid vs. (SC-Myel, SC-Nonmyel, SC-Nonmyel2)								
**gene**	**p_val**	**avg_log2FC**	**pct.1**	**pct.2**	**p_val_adj**	**Foldchange_UP**	**Foldchange_DOWN**	**Ratio_pct1_pct2**
DNMT3B	0,044226311	0,019130326	0,037	0,007	1	1,013348437	0,986827397	5,285714286
AGBL5	0,006523076	0,019102672	0,061	0,011	1	1,013329013	0,986846313	5,545454545
SDHAF1	0,000397671	0,019095035	0,183	0,053	1	1,013323649	0,986851537	3,452830189
KLHL7	0,002096749	0,019049436	0,207	0,077	1	1,013291621	0,986882729	2,688311688
SHLD3	0,066254792	0,018984877	0,024	0,004	1	1,013246279	0,986926891	6
EVI2A	0,063203483	0,018981705	0,012	0	1	1,013244051	0,986929061	#DIV/0!
FOXL1	0,063203483	0,018981705	0,012	0	1	1,013244051	0,986929061	#DIV/0!
FPR1	0,063203483	0,018981705	0,012	0	1	1,013244051	0,986929061	#DIV/0!
JARID2-AS1	0,063203483	0,018981705	0,012	0	1	1,013244051	0,986929061	#DIV/0!
KCNJ6	0,063203483	0,018981705	0,012	0	1	1,013244051	0,986929061	#DIV/0!
MED18	0,063203483	0,018981705	0,012	0	1	1,013244051	0,986929061	#DIV/0!
RNF125	0,063203483	0,018981705	0,012	0	1	1,013244051	0,986929061	#DIV/0!
TNFRSF10A	0,063203483	0,018981705	0,012	0	1	1,013244051	0,986929061	#DIV/0!
DDX19A	4,46557E-05	0,0188787	0,256	0,077	0,942279781	1,01317171	0,986999528	3,324675325
SMG5	0,059191799	0,018844459	0,11	0,049	1	1,013147664	0,987022954	2,244897959
ATG4D	0,000732778	0,018825871	0,098	0,018	1	1,01313461	0,987035671	5,444444444
HCN3	0,35185965	0,018798789	0,012	0,004	1	1,013115592	0,9870542	3
ZNF419	0,185668317	0,018797652	0,024	0,007	1	1,013114794	0,987054977	3,428571429
CCDC51	0,001423372	0,018773501	0,073	0,011	1	1,013097834	0,987071501	6,636363636
ZNF282	0,000234367	0,018739644	0,098	0,014	1	1,013074059	0,987094666	7
FBXW9	0,010916043	0,018710487	0,073	0,018	1	1,013053585	0,987114615	4,055555556
CORT	0,066254792	0,018700896	0,024	0,004	1	1,01304685	0,987121178	6
AC005597.1	0,063203483	0,01868139	0,012	0	1	1,013033154	0,987134524	#DIV/0!
ACOT11	0,063203483	0,01868139	0,012	0	1	1,013033154	0,987134524	#DIV/0!
GPC6	0,063203483	0,01868139	0,012	0	1	1,013033154	0,987134524	#DIV/0!
PCDHGB1	0,063203483	0,01868139	0,012	0	1	1,013033154	0,987134524	#DIV/0!
SFTPD	0,063203483	0,01868139	0,012	0	1	1,013033154	0,987134524	#DIV/0!
TNNI3	0,063203483	0,01868139	0,012	0	1	1,013033154	0,987134524	#DIV/0!
ZNF658	0,063203483	0,01868139	0,012	0	1	1,013033154	0,987134524	#DIV/0!
CCDC91	6,88939E-06	0,018660196	0,28	0,077	0,145373117	1,013018271	0,987149026	3,636363636

Supplementary Table 2

Differentially expressed genes_keloid-XL_ comparing SC-Keloid vs. (SC-Myel, SC-Nonmyel, SC-Nonmyel2)								
**gene**	**p_val**	**avg_log2FC**	**pct.1**	**pct.2**	**p_val_adj**	**Foldchange_UP**	**Foldchange_DOWN**	**Ratio_pct1_pct2**
TTLL3	0,017600145	0,018597044	0,22	0,105	1	1,012973929	0,987192238	2,095238095
NAV3	0,12773253	0,018582077	0,098	0,049	1	1,01296342	0,98720248	2
PNPLA6	0,007103533	0,018576404	0,146	0,053	1	1,012959437	0,987206361	2,754716981
PRKAA1	0,000961052	0,01850896	0,171	0,053	1	1,012912084	0,987252513	3,226415094
AC009506.1	0,027028964	0,018429649	0,049	0,011	1	1,012856401	0,987306788	4,454545455
TRIM61	0,011553628	0,018388474	0,037	0,004	1	1,012827495	0,987334966	9,25
PRDM4	0,095840528	0,018382917	0,098	0,046	1	1,012823593	0,98733877	2,130434783
PLEKHH1	0,006556321	0,018377362	0,085	0,021	1	1,012819693	0,987342571	4,047619048
C2	0,654971645	0,018371161	0,012	0,007	1	1,01281534	0,987346815	1,714285714
CFAP45	0,348081613	0,018368805	0,024	0,011	1	1,012813686	0,987348427	2,181818182
RPS14	0,539771885	0,018361967	0,927	0,853	1	1,012808886	0,987353107	1,086752638
TNFAIP8L3	0,008401736	0,018353999	0,024	0	1	1,012803292	0,98735856	#DIV/0!
CUBN	0,148735876	0,018319506	0,073	0,035	1	1,012779077	0,987382167	2,085714286
CD68	0,008556874	0,018275198	0,098	0,028	1	1,012747973	0,987412492	3,5
CCDC174	0,000128556	0,018248475	0,232	0,07	1	1,012729214	0,987430782	3,314285714
SIRT2	0,001176662	0,018234885	0,427	0,2	1	1,012719675	0,987440083	2,135
[truncated: 428,901 more chars]
